# Reliability and validity of the NeuroCognitive Performance Test, a web-based neuropsychological assessment

**DOI:** 10.3389/fpsyg.2015.01652

**Published:** 2015-11-03

**Authors:** Glenn E. Morrison, Christa M. Simone, Nicole F. Ng, Joseph L. Hardy

**Affiliations:** ^1^Department of Research and Development, Lumos LabsSan Francisco, CA, USA; ^2^Formerly of Lumos LabsSan Francisco, CA, USA

**Keywords:** neuropsychological assessment, web-based, reliability, concurrent validity, normative data, memory, fluid reasoning, psychomotor speed

## Abstract

The NeuroCognitive Performance Test (NCPT) is a brief, repeatable, web-based cognitive assessment platform that measures performance across several cognitive domains. The NCPT platform is modular and includes 18 subtests that can be arranged into customized batteries. Here we present normative data from a sample of 130,140 healthy volunteers for an NCPT battery consisting of 8 subtests. Participants took the NCPT remotely and without supervision. Factor structure and effects of age, education, and gender were evaluated with this normative dataset. Test-retest reliability was evaluated in a subset of participants who took the battery again an average of 78.8 days later. The eight NCPT subtests group into 4 putative cognitive domains, have adequate to good test-retest reliability, and are sensitive to expected age- and education-related cognitive effects. Concurrent validity to standard neuropsychological tests was demonstrated in 73 healthy volunteers. In an exploratory analysis the NCPT battery could differentiate those who self-reported Mild Cognitive Impairment or Alzheimer's disease from matched healthy controls. Overall these results demonstrate the reliability and validity of the NCPT battery as a measure of cognitive performance and support the feasibility of web-based, unsupervised testing, with potential utility in clinical and research settings.

## Introduction

Neuropsychological assessments are designed to measure cognitive functions in both healthy and clinical populations and remain important tools for research studies, clinical diagnoses, patient outcomes, and intervention monitoring (Wild et al., 2008; Bauer et al., 2012; Kueider et al., 2012; Lampit et al., 2014; Zygouris and Tsolaki, 2014). The current standard for testing an individual's cognitive functioning is through conventional validated pencil-paper neuropsychological tests that are administered one-on-one in a clinic or lab setting by a trained psychometrician. The high cost and time commitment associated with this type of testing may serve as a barrier to optimized patient care and more efficient research.

Computerized administration of clinical instruments is not an entirely new phenomenon and the application of computers to the evaluation of cognition has been studied previously (Wild et al., 2008; Bauer et al., 2012; Kueider et al., 2012; Zygouris and Tsolaki, 2014). With advances in technology, computerized neuropsychological tests (cNPTs) have been able to address several shortcomings of conventional testing methods (Wild et al., 2008; Lampit et al., 2014; Zygouris and Tsolaki, 2014). Key advantages of cNPTs over pencil-paper assessments include: (1) consistency in administration and scoring, (2) ability to generate numerous alternate forms for repeated testing, (3) precise stimulus control, (4) ability to record multiple components of a participant's response, (5) adaptability of difficulty levels, (6) decreased cost of administration, (7) increased access for a broader population which can lead to greater diversity among subjects, patients, and normative databases, and (8) ability to run larger validation and reliability studies leading to larger, more accurate normative databases (Kane and Kay, 1992; Gualtieri and Johnson, 2006; Kueider et al., 2012; Nakayama et al., 2014). In their relatively short history, cNPTs have proven advantageous compared to pencil-paper neuropsychological tests, lowering the cost of testing, and expanding their utility and potential applications.

During recent years many cNPTs have been developed (for a recent review see Zygouris and Tsolaki, 2014). At a minimum, cNPTs should provide a set of tests with a range of assessment capabilities that are consistent with a well-defined purpose (Schlegel and Gilliland, 2007). In addition, cNPTs must have supporting data to demonstrate response characteristics, reliability, and validity similar to traditional pencil-paper assessments or other validated cNPTs (Schlegel and Gilliland, 2007). Data from several peer-reviewed, published reports have supported the validity, reliability, and feasibility of the use of cNPTs in both clinical and research settings (e.g., Robbins et al., 1998; Maruff et al., 2004; Gualtieri and Johnson, 2006; Gur et al., 2010).

The NeuroCognitive Performance Test (NCPT; Lumos Labs, Inc.) is a brief, repeatable, web-based platform of cognitive assessments intended to measure functioning across several cognitive domains including working memory, visuospatial memory, psychomotor speed, fluid and logical reasoning, response inhibition, numerical calculation, and selective and divided attention. To date, the platform includes 18 subtests that are online adaptations of widely used conventional neuropsychological tests. The NCPT is being developed as an assessment tool applicable to a broad population. In clinical research, the NCPT could have specific utility as a screening tool for entering participants into trials, or as an outcome measure to support efficacy; in clinical settings it could aid in the diagnosis of cognitive impairment and monitor cognitive change over time.

The NCPT platform is modular and the subtests can be arranged into customized batteries. Here we present normative data for more than 130,000 individuals aged 13–89 years from a NCPT battery that includes 8 subtests and several analyses to demonstrate reliability and validity of the NCPT battery as a measure of cognitive performance. In addition, data from participants who self-reported cognitive impairment was used to test the ability of the NCPT battery to differentiate clinical populations from healthy ones, demonstrating potential utility in research and clinical settings.

## Methods and results

### Ethical statement

Since these studies evaluated performance on cognitive assessments and did not include an intervention they were exempt from IRB review. Participants were informed prior to starting the assessment (see Section Participants and Normative Data below) that their data would be used for research purposes, and opted in by choosing to take the assessment or not. Data included in all analyses were de-identified and analyzed in aggregate in accordance with Lumos Labs' Privacy Policy (www.lumosity.com/legal/privacy_policy).

### Participants and normative data

Data used to generate the normative database were derived in aggregate from Lumosity subscribers who took the NCPT as part of their user experience. The majority of these users had paid for a premium Lumosity subscription. Within 1 week of their initial sign-up, Lumosity users were invited via email and an in-app prompt to take the NCPT battery (time 1). Taking the NCPT was optional and not required for continued use of Lumosity. Normative data were derived from a sample of 130,140 individuals aged 13–89 years who were generally healthy (as assessed via self-report survey) and had taken the NCPT battery at least once (Normative Sample). Participants in the Normative Sample represented 187 countries, with the majority from the United States (68.0%), Canada (9.1%), and Australia (8.6%) (representation from all other countries was < 5%, or 14.3% combined). Following completion of the NCPT battery at time 1, participants were asked to report if they had ever been diagnosed with a variety of clinical conditions. Anyone reporting a clinical diagnosis was excluded from the Normative Sample. Assessment scores were grouped into 5-year age bins (except for 13–19 and 80–89) and scaled as described below (see Section Scoring). Analyses to evaluate inter-assessment correlations, factor structure, and effects of age, education, and gender were performed using the Normative Sample. Lumosity users who took the NCPT battery at time 1 were then invited 70 days later via a follow-up email and an in-app prompt to take the NCPT battery a second time (time 2). Throughout this period, participants could freely play a variety of cognitive training games as part of their Lumosity subscription. The cognitive training games are distinct from the NCPT battery, and none of the NCPT subtests were presented during this period. A total of 35,779 users (Pre-Post Sample) took the NCPT battery at both time points and these data were used to evaluate test-retest reliability. In order to generate a complete normative database and conduct the appropriate analyses, only data for participants who completed all subtests of the NCPT in a single session were included in the Normative and Pre-Post Samples. Finally, in a separate study that included 73 young healthy adults, concurrent validity for five of the eight NCPT subtests to their corresponding pencil-paper neuropsychological tests was evaluated. All analyses were conducted in R (R Core Team, 2015). Demographic characteristics for all participants are summarized in Table 1.

**Table 1:** **Demographic characteristics of the groups studied**.

	**Normative sample**	**Pre-post sample**	**Concurrent validity study**
Number	130,140	35,779	73
Mean age (range)	46.3 (13.01–89.78)	50.7 (13.01–89.78)	29.0 (21–43)
% Female/Male/ND	53.3/40.1/6.6	58.3/34.4/7.6	38.3/61.7
Years of education (%)			
0–12 years	14.4	12.8	0
13–16 years	45.4	44.6	54.8
17+ years	30.2	31.5	23.3
ND	9.9	11.1	21.9

*The Normative Sample includes Lumosity users who were generally healthy (as assessed via self-report survey) and took the NCPT battery at least once; the Pre-Post Sample includes a subset of the Normative Sample who took the NCPT at time 2; The Concurrent Validity Study includes individuals from a separate study who took the NCPT and corresponding pencil-paper assessments*.

*ND = No Data*.

### The neurocognitive performance test

#### Development

In general, NCPT subtests are based on existing pencil-paper assessments where shifting to computerized administration would not negatively impact the test mechanic. The process for developing NCPT subtests consists of six stages, as follows:

1. ***A specific cognitive function or domain is highlighted as an area of focus for a new subtest***. Based on review of the neuroscience and neuropsycology literature, a team of scientists at Lumos Labs evaluates existing neuropsychological assessments in the designated area to identify a currently existing assessment, or the fundamental components required for testing in the designated area. The resulting NCPT subtest may be a direct computerized, web-based replication of an existing paper-pencil test (assuming it is open-access), like the Trail Making Test; or it may based on an existing test but not an exact replication, like Progressive Matrices.
2. ***A software engineer develops a beta version of the subtest***. A Lumos Labs scientist will write the subtest specification for an engineer to follow as he/she develops the beta version. The specification includes background information, objective, design mockups, including for a tutorial, copy for instructions, test mechanics (e.g., 12 objects are presented, subject responds via arrow buttons), data and metadata to be stored, and scoring mechanism. The development process includes several rounds of back and forth between the developer and the scientist in an iterative process of development and quality assurance (QA) testing.
3. ***The beta version undergoes quality assurance testing***. When the final beta version of the subtest is approved by the scientist, QA testing proceeds, which may result in suggested changes. Suggested changes are reviewed by the scientist and implemented, if applicable. QA testing is deemed complete when no further suggestions are received.
4. ***The beta version then undergoes user testing***. When the beta version of the subtest is ready, it undergoes user testing in one of two ways:

4.1. The subtest is included in an online test-retest reliability study: A subset of Lumosity users are invited via email to take the new subtest twice, approximately 2 weeks apart. These experiments run for a few months, with hundreds of users completing the subtest at both timepoints.
4.2. The subtest is included in the default NCPT battery for all Lumosity subscribers to take as part of an ongoing research study of the NCPT. Beta versions of subtests are not included in the calculation of the Grand Index score. This testing method generates vast amounts of performance data quickly, as all Lumosity subscribers are invited to take the NCPT within the first week of their subscription and then again 70 days later. Typically this default NCPT is taken about 400–500 times per day by Lumosity subscribers.

5. ***User test data is analyzed***. After enough data is gathered, Lumos Labs scientists analyze score distributions and psychometric properties such as test-retest reliability. Finally, the subtest's correlation with other NCPT subtests and its position within the factor structure of the full NCPT battery are examined, as evidence that it is assessing the target cognitive function.
6. ***The subtest is released***. If the subtest shows good score distribution, test-retest reliability, and correlation with other subtests, then it is considered complete and made available for inclusion in NCPT batteries. If any of these metrics are unsatisfactory, scientists and developers will work to make improvements; it will then be tested again in Step 4. If the improvements do not address the shortcomings, the assessment is dropped.

Content for the NCPT is algorithmically generated. The algorithm for each subtest is based on individual subtest specifications resulting in numerous, randomly generated alternate forms and offering the possibility of retesting any number of times without repeating the exact content. The NCPT subtests are currently published with Adobe Flash 10.1 compatibility. The target frame rate is 30 frames per second. The Flash file for each subtest loads individually before beginning the subtest and data is sent at the conclusion of each subtest. The NCPT is optimized for administration in an unsupervised environment on desktop or laptop computers with Internet connectivity. Internet connectivity is required for subtest loading and data transmission, but not for the active test taking. For each subtest, users must successfully complete a tutorial and practice session before they are able to move on to the assessment to ensure they understand the task requirements.

#### Scoring

**Figure 1:**
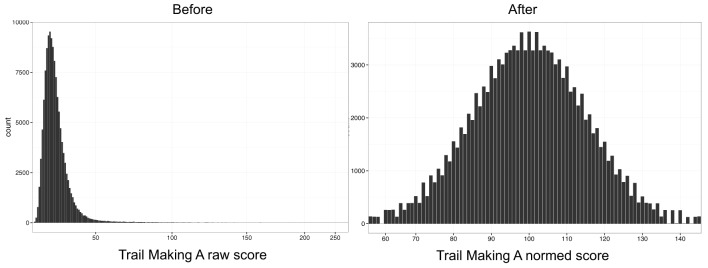
**NCPT normalization**. Score distributions for Trail Making A before **(left)** and after **(right)** the normalization procedure. Each NCPT subtest is scaled following a percentile rank-based inverse normal transformation. The position of that percentile on a normal distribution is used to convert the raw score to a scaled score where the distribution has a mean of 100 and a standard deviation of 15.

Each NCPT subtest is scaled following a percentile rank-based inverse normal transformation, a protocol used in well-accepted measures of cognitive ability such as the Wechsler Adult Intelligence Scale (Wechsler, 1955). Normative tables are created for each NCPT subtest. Each of these tables provides the corresponding scaled score for each observed raw score by 5-year age bin. To create these age-binned normative tables, the raw score from each subtest (e.g., the number of correct responses, completion time, etc.) is ranked within each age bin to obtain a percentile for each raw score. The position of that percentile on a normal distribution is used to convert the raw score to a scaled score where the distribution has a mean of 100 and a standard deviation of 15. An example of normalization using the Trail Making A subtest is shown in Figure 1.

After normative tables are created for each subtest, they are used to transform an individual's raw scores to scaled scores for each subtest included in the battery. These scaled subtest scores are then summed and the same rank-based inverse normal transformation is applied to the sum of the scaled subtest scores for all individuals to calculate an aggregate measure called the “Grand Index,” which can be interpreted as an overall measure of cognitive performance for the domains tested. These scaling procedures provide the benefit of having all scaled scores derived from an NCPT battery (each subtest scaled score and the Grand Index) on the same normal distribution that has a mean of 100 and a standard deviation of 15.

#### Subtests

The NCPT battery used in these analyses is relatively brief, taking between 20 and 30 min to complete, and includes 8 subtests, as follows:

(1) Arithmetic Reasoning is designed to assess numerical problem solving ability and requires the participant to respond as quickly and accurately as possible to arithmetic problems written in words (e.g., “Four plus two = ”) (Deloche et al., 1994). The primary measure is number of correct responses minus number of incorrect responses in 45 s.
(2) Digit Symbol Coding is based on the Digit Symbol Substitution Task (Royer, 1971) and assesses processing speed. The subtest lasts 90 s and participants are required to match a series of numbers that correspond to randomly generated symbols. The primary measure is number of correct responses minus number of incorrect responses.
(3) Forward and (4) Reverse Memory Span assess visual short-term and working memory, respectively, and are based on the Corsi Blocks tasks (Milner, 1971). These subtests require participants to recall a sequence of randomized spatial locations in either forward or reverse order. The subtest concludes when three consecutive errors on one sequence length are made. The primary measure is number correct.
(5) Grammatical Reasoning is based on Baddeley's Grammatical Reasoning Test (Baddeley, 1968) and is designed to assess cognitive flexibility and reasoning. The subtest lasts 45 s and requires participants to rapidly and accurately evaluate potentially confusing grammatical statements. The primary measure is number of correct responses minus number of incorrect responses.
(6) Progressive Matrices is based on established matrix reasoning assessments (Raven, 2000) and is designed to assess problem solving and fluid reasoning. The subtest lasts up to 17 trials, or concludes when three consecutive errors are made. The primary measure is number of correct responses.
(7) Trail Making A and (8) Trail Making B assess attention and processing speed and are based on the Army Individual Test Battery (Army Individual Test Battery, 1944) and the Halstead-Reitan Battery (Reitan and Wolfson, 1995). In Trail Making A participants are required to click on a sequential series of encircled numbers from 1 to 24. Task requirements are similar for Trail Making B except the circles include both numbers and letters and the participant must alternate sequentially between numbers and letters. For both Trail Making A and B, the primary measure is completion time (there is no time limit).

A more detailed description of the NCPT subtests used in these analyses is provided in Supplementary Data Sheet 2.

### Normative sample

Supplementary Table 1 describes the gender, race, education level, household income level, and handedness for each 5-year age bin in the Normative Sample. Participants who took the NCPT battery at time 1 had a mean age of 46.3 years (range 13–89; 53.3% female). Raw scores for each NCPT subtest in the battery were normed as described above to calculate the NCPT Grand Index. The overall mean (SD) for the Grand Index was 100.73 (15.39). Raw scores for each subtest by 5-year age bins (except 13–19 and 80–89) are provided in Supplementary Table 2. Normative tables by age, education, and gender are provided in Supplementary Data Sheet 1.

### Performance on the NCPT is sensitive to age, education, and gender

To examine the effects of demographic variables on baseline NCPT scores, we performed a multiple linear regression analysis to predict the non-age-normed sum score from age, education, and gender. Non-age-normed overall scores were used as the predicted variable in the model in order to measure the effects of age as a continuous variable. Non-age-normed scores for each subtest were generated by the same scaling procedure on the entire Normative Sample (i.e., without age-binning the scores first). The non-age-normed subtest scores were then summed to generate an overall score. Age and education (years) were continuous numeric variables and gender was a factor with males as the reference group. The model also included interaction terms for age X gender, age X education, and gender X education. Included in the model were 110,551 participants for whom complete demographic data were available.

**Figure 2:**
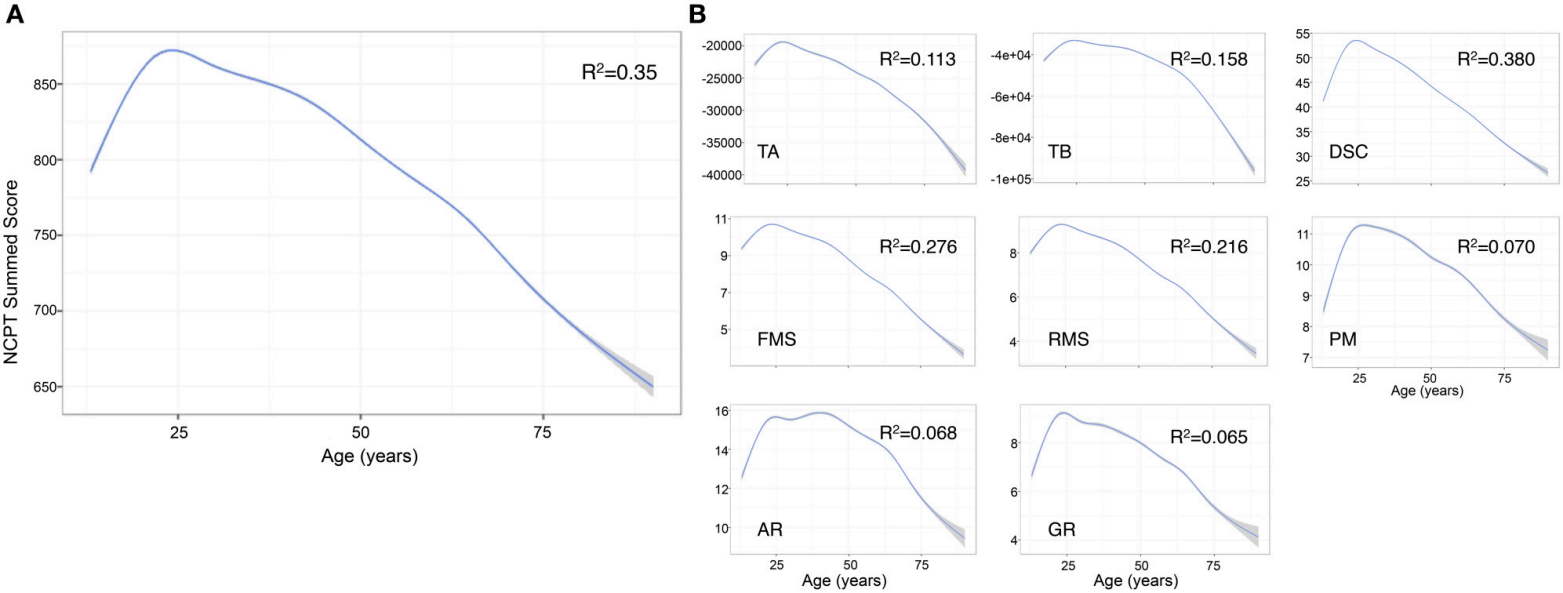
**Effect of age on individual NCPT subtest scores and overall sum score**. **(A)** The effect of age on the Grand Index was significant with peak performance occurring around age 25 and then declining in a linear fashion. **(B)** The effect of age on the individual subtests followed the same general trend. The decline in performance with age after 25 years appears linear for most subtests with the exception of Arithmetic Reasoning, Grammatical Reasoning, and Trail Making B. The curves were smoothed with a General Additive Model (GAM), a type of general linear model in which the linear predictor depends on linear smoothed functions (Wood, 2011). GAM was selected over a simple linear smoother because we observed that the effect of age on NCPT scores was non-linear. TA, Trail Making A; TB, Trail Making B; DSC, Digit Symbol Coding; FMS, Forward Memory Span; RMS, Reverse Memory Span; PM, Progressive Matrices; AR, Arithmetic Reasoning; GR, Grammatical Reasoning.

The predictors in the model explained 36.1% of the variance in the NCPT score [*R*^2^ = 0.361, *F*_(6, 110544)_ = 104100, *p* < 0.001] (Table 2). The effect of age was significant (β = −0.797, *p* < 0.001) with peak performance occurring at approximately age 25 and then declining linearly (Figure 2A). The effect of age on the individual subtests was further explored and the same general trend observed: performance peaks in the 20 s and then steadily declines with increasing age (Salthouse, 1996). The decline in performance with age after 25 years appears linear for most subtests with the exception of Arithmetic Reasoning, Grammatical Reasoning, and Trail Making B (Figure 2B).

**Table 2:** **Linear regression model for age, education, gender**.

	**Estimate**	**Std. Error**	***t*-value**	**Pr(>|t|)**
Intercept	748.073001	2.925407	255.716	<2e-16^**^
Gender (female)	−11.766653	2.465341	−4.773	1.82e-06^**^
Education	13.290844	0.193952	68.526	<2e-16^**^
Age	−0.797219	0.062988	−12.657	<2e-16^**^
Education X Age	−0.139730	0.003975	−35.151	<2e-16^**^
Gender X Education	−0.342012	0.150970	−2.265	0.0235^*^
Gender X Age	0.201607	0.024660	8.176	2.98e-16^**^

*Modeling the effects demographic of demographic variables on baseline NCPT score. Age and education are continuous variables, and gender is a factor with males as the reference. The overall model had an R^2^ of 0.36 [F_(6, 110544)_ = 1.041e^4^, p < 2.2e^−16^]*.

Significance codes:

** *p < 0.001*,

* *p < 0.01*.

**Figure 3:**
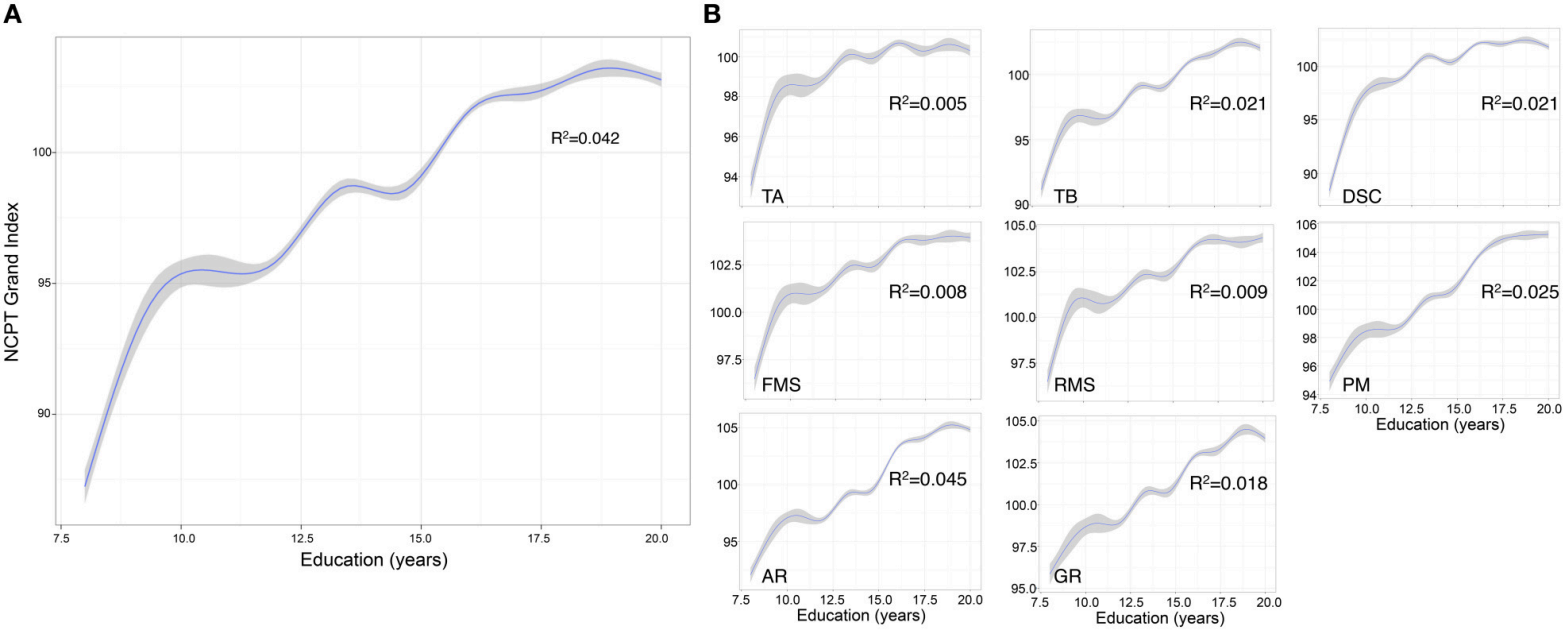
**Effect of education on NCPT Grand Index score and individual NCPT subtest scores**. **(A)** The effect of education on the Grand Index was significant with scores increasing with number of years education. **(B)** The same trend was observed for each subtest, with increasing years of education correlated to higher subtest scores. The curves were smoothed with a GAM function. GAM was selected over a simple linear smoother because we observed that the effect of education on NCPT scores was non-linear. TA, Trail Making A; TB, Trail Making B; DSC, Digit Symbol Coding; FMS, Forward Memory Span; RMS, Reverse Memory Span; PM, Progressive Matrices; AR, Arithmetic Reasoning; GR, Grammatical Reasoning.

The effect of education was also significant (β = 13.291, *p* < 0.001) with Grand Index scores increasing with number of years of education (Figure 3A). The same trend was observed for each subtest, with increasing years of education correlated to higher subtest scores (Figure 3B). The interaction between age and education was also significant (β = −0.139, *p* < 0.001) suggesting the positive main effect of education diminishes with age.

**Figure 4:**
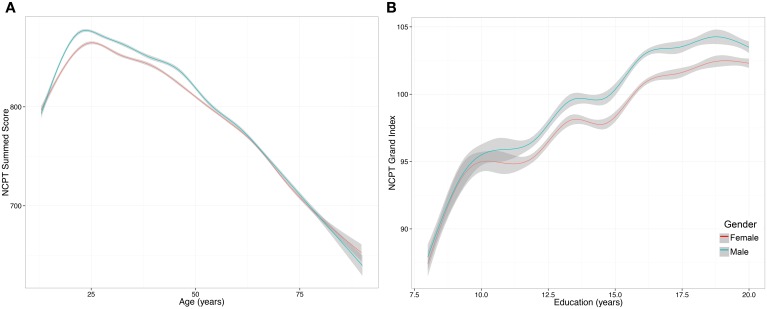
**Effect of gender on NCPT Grand Index score**. **(A)** The interaction between age and gender was significant and suggests that the negative main effect of gender diminishes with age. The y axis is the non-age-normed NCPT sum score. **(B)** The interaction between education and gender was significant and suggests that education has a greater positive impact on males compared to females. The y axis is the non-age-normed NCPT sum score. The curves were smoothed with a GAM function. GAM was selected over a simple linear model because we observed that the effect of gender on NCPT scores was non-linear.

The mean (SD) Grand Index scores for males was 100.99 (14.62) and for females was 99.54 (14.62). The model showed that the effect of gender was significant (β = −11.767, *p* < 0.001), as were the interaction between age and gender (β = 0.202, *p* < 0.001) (Figure 4A) and education and gender (β = −0.342, *p* = 0.024) (Figure 4B). The positive interaction between age and gender suggests that the negative main effect of gender diminishes with age, whereas the negative interaction between education and gender suggests that education has a greater positive impact on males compared to females.

### NCPT subtests are positively correlated yet capture distinct cognitive abilities

Pearson correlations were calculated for each pair of subtests in the battery using the scaled subtest scores of the *N* = 130,140 participants who took the NCPT at time 1. Subtest scores were scaled based on the age-normed tables from time 1 as described above.

**Figure 5:**
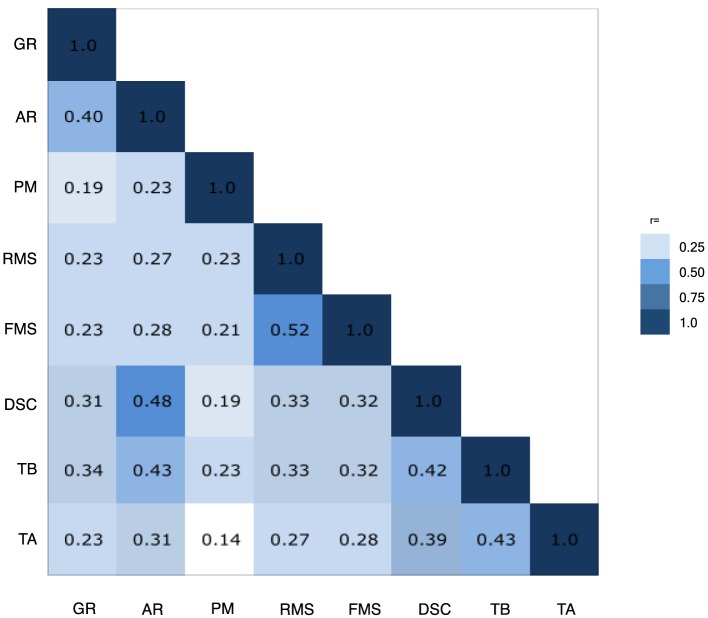
**Inter-assessment correlations**. Heat map showing that each NCPT subtest was significantly correlated with performance on more than one other subtest. The heat map suggests three groupings that share higher correlations, as follows: (1) Arithmetic Reasoning (AR), Grammatical Reasoning (GR), and Digit Symbol Coding (DSC); (2) Forward (FMS) and Reverse Memory Span (RMS); and (3) Trail Making A (TA) and Trail Making B (TB). Progressive Matrices (PM) does not appear to correlate strongly with any other subtest.

Pearson correlations comparing each of the NCPT subtests to the other subtests in the battery showed that each NCPT subtest was significantly correlated with performance on more than one other subtest (*p* < 0.0001 for all correlations). Seven of the 8 subtests correlated at least *r* = 0.3 with at least one other subtest, suggesting reasonable factorability. Correlations ranged from *r* = 0.14 (Trail Making A:Progressive Matrices) to *r* = 0.52 (Forward Memory Span:Reverse Memory Span). The correlations are depicted in a heat map in Figure 5. The heat map suggests three groupings that share higher correlations, as follows: (1) Arithmetic Reasoning, Grammatical Reasoning, and Digit Symbol Coding; (2) Forward and Reverse Memory Span; and (3) Trail Making A and Trail Making B. Progressive Matrices does not appear to correlate strongly with any other subtest. This was further supported using a Euclidian distance matrix to create a dendrogram of the NCPT subtests (Supplementary Image 1). Initially, each subtest was assigned to its own cluster and then the algorithm proceeded iteratively, at each stage joining the two most similar clusters, continuing until there was just a single cluster. Using this methodology, the right-most position of the dendrogram nodes appears to support the three groupings derived from the subtest correlation matrix.

To further understand construct validity, factor analysis was performed in order to maximize the ability to explain the variance of the observed subtest scores (Ford et al., 1986). To determine the number of factors, we first performed parallel analysis with 10,000 iterations using the psych package for R (Revelle, 2015), which resulted in a four-factor structure. In parallel analysis, the eigenvalues of factors from the observed data are compared with those of a random data matrix of the same size and factors with eigenvalues greater than zero are retained. Factor analysis was performed on the normalized subtest scores from the Normative Sample using the factanal function from the stats package in R (R Core Team, 2015) for a four-factor solution with varimax rotation (Table 3). The four-factor solution was significant [$\chi_{\left(2\right)}^{2}=19.01$, *p* < 0.001] indicating the model is not satisfactory; however, because the sample size is large it does not automatically result in rejecting the model. The root mean square error of approximation (RMSEA) = 0.0081 (95% CI = 0.004, 0.012) indicative of acceptable model fit (Hu and Bentler, 1999).

**Table 3:** **Factor analysis**.

	**Factor 1**	**Factor 2**	**Factor 3**	**Factor 4**
Trail Making A	0.186	0.178	**0.671**	
Trail Making B	0.376	0.228	**0.455**	0.300
Forward memory span	0.179	**0.621**	0.196	
Reverse memory span	0.158	**0.731**	0.158	0.112
Digit symbol coding	**0.450**	0.260	0.388	
Progressive matrices	0.219	0.217		0.211
Arithmetic reasoning	**0.754**	0.154	0.211	
Grammatical reasoning	**0.442**	0.160	0.160	0.218
Proportion of total variance	0.156	0.146	0.118	0.026
Cumulative variance		0.302	0.420	0.447

*Factor loadings for the 4-factor model of scaled NCPT subtest scores. Loadings greater than 0.4 are bolded*.

The four factors explained a total of 44.7% of the variance in the subtest scores. Based on the cognitive domains captured by the subtests, the factors can be grouped with the following labels: (1) Mental Flexibility: Digit Symbol Coding, Arithmetic Reasoning, and Grammatical Reasoning (Baddeley, 1968; Prabhakaran et al., 2001), (2) Memory: Forward Memory Span and Reverse Memory Span (Richardson, 2007), and (3) Attention and Speed of Processing: Trail Making A and Trail Making B (Army Individual Test Battery, 1944). Progressive Matrices, which did not group under these labels, is a measure of fluid intelligence (Prabhakaran et al., 1997). Progressive Matrices did not contribute to a simple factor structure, loading equally to Factors 1, 2, and 4, and failed to meet a minimum criteria of having a primary factor loading of 0.4 or above, and no cross-loading of 0.3 or above (Ford et al., 1986).

### The NCPT grand index is repeatable and reliable

Participants included in the Pre-Post Sample (*N* = 35,779) took the NCPT battery at time 1 and time 2 and were included in the analysis of test-retest reliability. The mean age of the Pre-Post Sample was 50.7 years (range 13–89; 50.7% female). Participants were invited to take the NCPT 70 days after the first administration, yet they had the ability to navigate directly to the NCPT and take the second test at any time. Due to this variability, the mean Inter-test Interval (ITI) was 78.8 days, ranging from 29 to 235 days. Scores at time 2 were scaled using the original normative tables from the Normative Sample (*N* = 130,140). Test-retest reliability of the NCPT battery was calculated with Pearson correlations and 95% CIs for each subtest and for the Grand Index at time 1 and time 2. The correlation between NCPT Grand Index scores at time 1 and time 2 demonstrated strong test-retest reliability for the Grand Index (*r* = 0.831; 95% CI [0.829, 0.832]; *p* < 0.001). Reliability of the Grand Index was good for all ages and ranged from 0.756 in participants age 80–89 to 0.848 in participants aged 13–19 (Supplementary Table 3).

Test-retest reliabilities for each of the 8 subtests were statistically significant (*p* < 0.001) and ranged from 0.388 for Progressive Matrices (95% CI 0.383, 0.393) to 0.738 for Digit Symbol Coding (95% CI 0.735, 0.740). A non-parametric bootstrapping procedure with 10,000 iterations was performed to calculate the median, inter-quartile range (IQR), and non-parametric 95% CI (i.e., based on the 2.5 and 97.5% of the distribution) test-retest reliabilities for each subtest and the Grand Index. Pearson correlations, IQR, and 95% CIs for each NCPT subtest in the battery are listed in Table 4. Published reliabilities for the traditional pencil-paper correlates are provided in Supplementary Table 5.

**Table 4:** **NCPT test-retest correlations**.

**NCPT subtest**	**Pearson's r**	**95% CI**	**Bootstrap statistics**		
			**Median**	**95% CI**	**IQR**
Grand index	0.831	[0.829, 0.832]	0.831	[0.827, 0.834]	0.0024
Trail Making A	0.570	[0.566, 0.574]	0.570	[0.561, 0.579]	0.0058
Trail Making B	0.529	[0.525, 0.533]	0.529	[0.521, 0.538]	00057
Digit symbol coding	0.738	[0.735, 0.740]	0.738	[0.732, 0.743]	0.0039
Forward memory span	0.530	[0.526, 0.533]	0.529	[0.522, 0.537]	0.0054
Reverse memory span	0.510	[0.506, 0.514]	0.510	[0.502, 0.518]	0.0056
Progressive matrices	0.388	[0.383, 0.393]	0.388	[0.379, 0.397]	0.0062
Arithmetic reasoning	0.734	[0.732, 0.737]	0.735	[0.729, 0.740]	0.0036
Grammatical reasoning	0.533	[0.529, 0.537]	0.533	[0.526, 0.541]	0.0055

*Pearson's correlations and 95%CIs for test-retest reliability for each of the subtests and the Grand Index as measured from the Pre-Post Sample. Correlations between NCPT Grand Index scores at time 1 and time 2 (mean ITI = 78.8 days, range 29–235 days) demonstrated strong test-retest reliability for the Grand Index. Test-retest reliabilities for scaled scores for each subtest were all statistically significant (p < 0.001). A non-parametric bootstrapping procedure with 10,000 iterations was performed to calculate the median, 95%CI, and IQR*.

To explore whether the low reliability for Progressive Matrices had an effect on the overall reliability of the Grand Index, Pearson correlations for the Grand Index scores at time 1 and time 2 were repeated without including Progressive Matrices. The reliability for the Grand Index without Progressive Matrices was nearly identical (*r* = 0.833; 95% CI [0.830, 0.836]), and a Fisher r-to-z transformation comparing correlation coefficients showed that they were not statistically significantly different (*z* = 0.95, *p* = 0.342).

Because of the wide range in ITI, the interaction of ITI duration and test-retest reliability was evaluated. Based on a mean (SD) ITI of 78.8 (19.86) days, ITI range was divided into four bins based on 0.5 standard deviations of the mean ITI: < 0.5 SD (29–68 days), mean ITI ± 0.5 SD (69–88 days), 0.5–1.5 SD (89–108 days), and >1.5 SD (109–235 days). Because the sample size in the two extreme bins was small with large standard deviations for reliability of the mean Grand Index (0.55 and 0.28, respectively) Pearson correlations and 95% CIs for the median Grand Index were calculated for each of the bins and found to be highest for an ITI of 29–68 days (*r* = 0.87 [0.50, 0.78]) and relatively indistinguishable for the other ITI groups (0.83 [0.83, 0.84], 0.84 [0.81, 0.84], and 0.85, [0.78, 0.81] respectively; Supplementary Table 4).

Participants were also free to play Lumosity between testing at time 1 and time 2. The number of unique days a user played at least one Lumosity game was used as the measure of engagement and the effect of unique days played on test-retest reliability was evaluated. Participants played at least one game a mean (SD) of 36.7 (19.2) days, ranging from 0 to 185 days. Because the number of unique days played was skewed to the left, Pearson correlations and 95% CIs for each quartile of unique days playing Lumosity were calculated for the Grand Index score and were found to be highest for the shortest number of unique days: 0–22 days: *r* = 0.906 (0.902–0.910); 23–37 days: *r* = 0.830 (0.824–0.837); 38–51 days: *r* = 0.831 (0.824–0.837); 51–185 days: *r* = 0.835 (0.829–0.842).

To evaluate the effect of days played on test-retest reliability, we first constructed a linear model predicting Grand Index score at time 2 from the number of days playing Lumosity, controlling for Grand Index at time 1 (Grand Index 2 ~ Grand Index 1 + unique days played). The R^2^ for this model was 0.6947 [*F*_(2, 35776)_ = 4.071E^4^, *p* < 0.001]. The R^2^ for this model is a combination of the variance explained by score at time 1, which can be interpreted as a measure of test-retest reliability, and the variance explained by the linear effect of the number of days the participant played. The proportion of variance remaining (1–R^2^), is equal to the sum of the squared residuals from this model divided by the total sum of squares for the scores at time 2 and to the mean of the squared residuals from the model divided by the variance of the scores at time 2.

The squared residuals, whose mean based on the equalities noted above, is equal to (1–R^2^) ^*^ s^2^, where s^2^ is the variance in scores at time 2 and R^2^ is the proportion of that variance accounted for by the model. In order to address whether the remaining variance might be due to differences in test-retest reliability at different amounts of days playing Lumosity, the squared residuals from the first model were used as the outcome measure for a second model using days played as the predictor (square residuals from model 1 ~ unique days playing). If reliability were lower for participants who played more often, the average value of the squared residuals from the first model would be greater at higher values of number of days played, resulting in a positive coefficient for days played in the second model, whereas if reliability were higher for participants who played more often, the average value of the squared residuals would be smaller at these values, resulting in a negative coefficient for days played in the second model. In the second model, the effect of number of days played was not significant (β = 0.042, *p* = 0.163) nor was the overall model [*R*^2^ = 5.427E^−5^, *F*_(1, 35777)_ = 1.942, *p* = 0.1635], suggesting that the residuals don't grow or shrink linearly with increasing number of days playing Lumosity, though there is a slight trend toward increasing variance, and thus decreasing reliability, with more days played.

Since the prescribed ITI was 70 days, test-retest reliability for those who did not play Lumosity (0 unique days; *N* = 161) were compared to those who played 70 days (*N* = 177). Pearson correlations and 95% CIs for those who did not play Lumosity between testing was *r* = 0.846 (0.796–0.885) and for those who played 70 days was *r* = 0.836 (0.785–0.875). A Fisher r-to-z transformation was used to compare correlation coefficients and found not to be significant (*z* = 0.32, *p* = 0.749) confirming no effect of unique days played on reliability.

The number of games played during the ITI was also evaluated for its effect on test-retest reliability. Participants played a mean (SD) 353.7 (432.2) Lumosty games during the ITI (range 0–8997). Pearson correlations and 95% CIs for each quartile of number of Lumosity games played were calculated for the Grand Index score and were found to be similar for each quartile: 0–135 games: *r* = 0.830 (0.823–0.836); 136–240 games: *r* = 0.834 (0.827–0.840); 241–395 games: *r* = 0.834 (0.828–0.840); ≥ 396 games: *r* = 0.830 (0.823–0.837). Repeating the model above using number of games played instead of unique days played revealed a small, yet significant effect on the squared residuals (β = 0.0064, *p* < 0.0001) and the model was significant [*R*^2^ = 0.00062, *F*_(1, 35777)_ = 22.1, *p* < 0.0001], indicating that the number of games played has a greater effect on test-retest reliability than does number of days at least one game was played.

### The NCPT demonstrates good concordance to comparator pencil-paper neuropsychological tests

Concurrent validity of NCPT subtests to corresponding pencil-paper assessments was determined in a study in which participants were equally randomized (i.e., 1:1) to receive the NCPT followed by a pencil-paper neuropsychological test battery of corresponding assessments (or vice versa) in a single session. Assessments were administered in a lab setting by a trained rater. Both assessment batteries were performed once within a session that took approximately 1 h to complete.

A shortened NCPT battery with 5 subtests was used in this study; Arithmetic Reasoning, Grammatical Reasoning, and Progressive Matrices were excluded in order to focus on subtests with well-known, widely used pencil-paper correlates that could be administered in the allotted time for each participant. The corresponding pencil-paper assessments were HRB Trail Making Test, Parts A and B, BACS Digit Symbol Coding, and Wechsler Memory Scale III Forward and Reverse Spatial Span (see Table 5).

**Table 5:** **NCPT subtests and corresponding pencil-paper assessments for concurrent validity**.

**NCPT subtests**	**Pencil-paper assessments**	**Pearson's r**
Trail Making A	HRB Army Trail Making Test Part A (Reitan and Wolfson, 1995)	0.47^**^
Trail Making B	HRB Army Trail Making Test Part B (Reitan and Wolfson, 1995)	0.58^**^
Digit symbol coding	BACS Symbol Coding (MATRICS) (Keefe et al., 2004; Nuechterlein et al., 2008)	0.71^**^
Forward memory span	Wechsler Memory Scale (WMS-III) Forward Spatial Span Board (MATRICS) (Wechsler, 1945; Nuechterlein et al., 2008)	0.48^*^
Reverse memory span	Wechsler Memory Scale (WMS-III) Reverse Spatial Span Board (MATRICS) (Wechsler, 1945; Nuechterlein et al., 2008)	0.55^**^

*Concordance between NCPT subtests and corresponding pencil-paper tests were significant*.

Significance codes:

** p < 0.01;

* *p < 0.05*.

The study enrolled 73 adults with a mean age of 29 years (range 21–43; 38.3% female) who reported being in good general health with no known cognitive impairment or visual or hearing impairment that could affect testing. Participants had a mean (SD) 16.95 (2.0) years education at the time of participation. All participants reviewed and signed informed consent prior to participating in the study.

Pearson correlations between raw scores on NCPT subtests and comparator pencil-paper assessments were calculated to determine the extent to which performance on each NCPT subtest correlated with performance on the comparator assessment. Correlations between NCPT subtests and corresponding pencil-paper assessments were moderate to large. Individual correlations (Pearson's r) are provided in Table 5.

### Ability to differentiate individuals with cognitive impairment

NCPT scores for 1493 individuals 50 years of age and older who self-reported Mild Cognitive Impairment (MCI) and for 105 individuals who self-reported Alzheimer's Disease (AD) were compared to age, education, and gender matched healthy controls (HC) from the Normative Sample (i.e., those who reported no clinical diagnoses). Matching was performed using the Matching package in R (Sekhon, 2011). For each individual in the MCI or AD groups, an individual who exactly matched on all three factors—age, gender, and education—was identified from the Normative Sample without replacement until a matched group of equal size was generated (i.e., *N* = 1493 for MCI-matched healthy control group and *N* = 105 for AD-matched healthy control group). The mean (SD) age for those who self-reported MCI (and the MCI-matched HC) was 65.2 (8.53) years and those who self-reported AD (and the AD- matched HC) was 68.8 (8.31) years (Table 6).

**Table 6:** **Demographic characteristics for self-report MCI and AD diagnoses**.

	**MCI**	**AD**	**All HC ≥50**
Number	1493	105	60,191
Mean (SD) age	65.2 (8.53)	68.8 (8.31)	62.1 (7.76)
% Female/Male	58.9/41.1	51.4/48.6	71.8/28.2
Years of education (%)			
0–12 years	10.2	13.3	13.9
13–16 years	48.3	43.8	44.2
17+ years	34.1	36.2	32.1
ND	7.6	6.7	9.7

*Summary data for individuals 50 years of age and older who self-reported Mild Cognitive Impairment (MCI) or Alzheimer's Disease (AD) are presented along with summary data for healthy controls (HC) 50 years of age and older from the Normative Sample*.

*ND = No Data*.

As this procedure can be sensitive to the order in which MCI or AD participants are selected for the matching, it was repeated 1000 times in order to obtain a range of estimates. After each run, a paired Student's *t*-test was performed to determine if the MCI and AD groups' mean Grand Index scores were significantly different from those of the matched healthy controls. For the MCI group, the mean difference (HC—MCI) in Grand Index scores ranged from 10.00 to 12.41 (mean 11.00). The *t*-test showed a significant difference between MCI and HCs in all 1000 datasets (T-statistic range [−22.42, −17.57], all *p* < 10^−63^). For the AD group, the mean difference (HC—AD) in Grand Index scores ranged from 12.19 to 19.92 (mean 16.18). The *t*-test showed a significant difference between AD and HCs in all 1000 datasets (T-statistic range [−10.12, −5.44], all *p* < 10^−6^).

**Figure 6:**
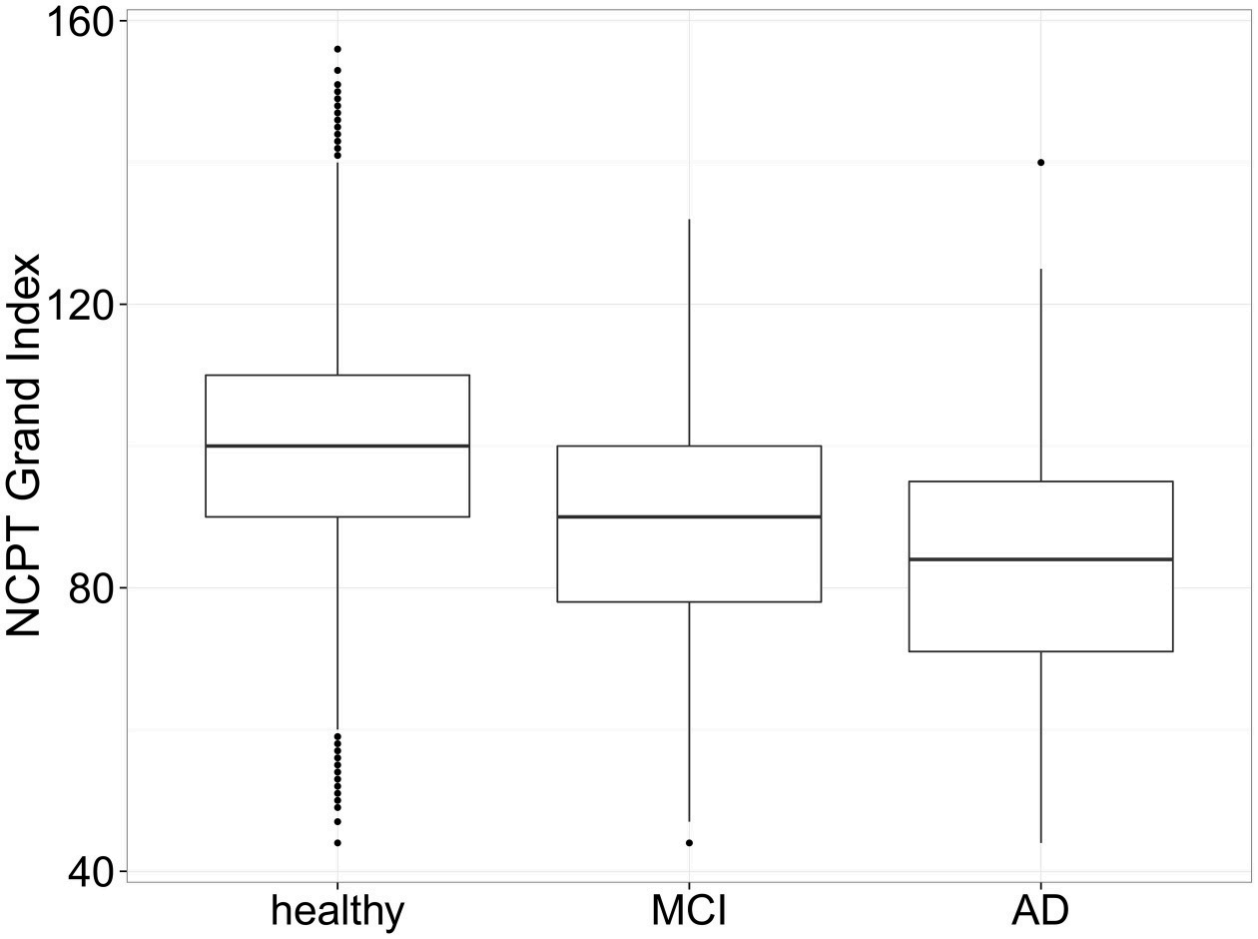
**Box plots of NCPT Grand Index scores for self-report MCI, AD, and healthy controls**. Compared to all healthy controls in the Normative Sample, the Grand Index score was 0.78 SD (*p* < 0.05) lower for those who self-reported MCI and 1.17 SD (*p* < 0.05) lower for those who self-reported AD suggesting the NCPT is able to differentiate those who self-report MCI or AD from those who don't.

As verification for the comparisons, a One-Way analysis of variance (ANOVA) was calculated comparing Grand Index scores for all HC participants 50 years of age or older from the Normative Sample (*N* = 60, 191) to those who self-reported MCI or AD and was found to be significant, *F*_(2,61,967)_ = 567.76, *p* < 0.001. Compared to HC participants, the Grand Index score was 0.78 SD (*p* < 0.05) lower for those who self-reported MCI and 1.17 SD (*p* < 0.05) lower for those who self-reported AD (Figure 6). These results suggest that in this group of older adults, those indicating a diagnosis of MCI or AD performed worse on the NCPT battery compared to age, gender, and education matched controls (Table 7) as well as the entire sample of healthy adults over 50.

**Table 7:** **NCPT grand index and subtest scores for self-report MCI and AD and matched controls**.

	**Healthy Controls (HC)**	**MCI**	**AD**
N	60,191	1493	105
Grand index	100.2 (14.49)	89.2 (15.82)	84.1 (17.12)
Trail Making A	100.0 (14.97)	92.2 (14.79)	90.3 (15.06)
Trail Making B	100.0 (14.97)	91.9 (15.46)	88.0 (16.39)
Forward memory span	103.0 (15.04)	96.5 (15.24)	95.5 (16.12)
Reverse memory span	103.2 (14.97)	96.5 (15.65)	93.8 (16.19)
Digit symbol coding	101.2 (14.94)	90.7 (16.12)	83.9 (18.27)
Progressive matrices	102.5 (15.49)	98.9 (14.80)	98.5 (14.47)
Arithmetic reasoning	101.8 (14.97)	94.3 (15.88)	89.8 (16.18)
Grammatical reasoning	102.2 (14.37)	97.7 (13.58)	93.3 (12.47)

*Mean (SD) Grand Index and subtest scores for individuals 50 years of age and older who self-reported Mild Cognitive Impairment (MCI) or Alzheimer's Disease (AD). Compared to healthy controls age 50 and older, the Grand Index score was 0.78 SD (p < 0.05) lower for those who self-reported MCI and 1.17 SD (p < 0.05) lower for those who self-reported AD*.

Further, a Welch unpaired, two-sample *t*-test comparing Grand Index scores for those who self-reported MCI to those who self-reported AD was performed. The Welch *t*-test is an adaptation of the Student's *t*-test and considered more reliable when the two samples have unequal variance and unequal sample size. The results of the Welch *t*-test were significant; *t*_(116.83)_ = 2.98, *p* = 0.0035, suggesting that mean Grand Index scores can be differentiated between these two self-report diagnoses.

## Discussion

The NCPT is being developed as an online, repeatable, and customizable cognitive assessment tool that has a range of potential utilities in clinical and research settings. In order to serve these purposes it is important to demonstrate psychometric properties relevant for the intended use cases, including normative properties, reliability, concordance to well-accepted tests, and ability to discriminate. The data presented are derived from a normative sample of 130,140 generally healthy individuals aged 13–89 years, representing one of the largest published normative datasets for a computerized cognitive battery. By comparison, Gualtieri et al. report normative data for CNS Vital Signs for 1069 individuals (Gualtieri and Johnson, 2006) and CANTAB includes an internal normative database of 3000 healthy volunteers (Zygouris and Tsolaki, 2014).

The data demonstrate the validity and reliability of the tested NCPT battery as a measure of cognitive performance and support the feasibility of web-based, unsupervised testing of large cohorts within the general population.

NCPT subtests were designed to be replications of standard pencil-paper assessments used in neuropsychological evaluations; however, it cannot be assumed that the NCPT subtests have the same response characteristics, reliability, and validity as traditional pencil-paper assessments simply because the tests were designed as “look-alikes” (Bauer et al., 2012). In a study in young healthy adults the NCPT subtests demonstrated good concordance with corresponding pencil-paper assessments, supporting concurrent validity. The observed concordance in this study is likely to be a conservative estimate of true concordance because the study was restricted to a sample of young, highly educated adults. Reliability coefficients have been shown to be lower in more homogeneous populations (Gulliksen, 1950) so it is reasonable to assume that in a more heterogeneous sample spanning wider age and education brackets, concordance between the NCPT subtests and pencil-paper correlates would be higher. Nevertheless, the moderate to high correlation coefficients observed supports the feasibility of implementing the NCPT as an alternative to traditional pencil-paper testing. Overall, these data supply several “different lines of validity evidence” which are “all in service of providing information relevant to a specific intended interpretation of test scores” (American Educational Research Association, 1999).

All of the subtests in the NCPT battery were found to positively correlate with one another and factor analyses indicated that four distinct factors explained the correlational structure. We observed the following groupings of more strongly correlated subtests and applied factor labels accordingly: Arithmetic Reasoning, Grammatical Reasoning, and Digit Symbol Coding, which assess cognitive flexibility (Baddeley, 1968; Prabhakaran et al., 2001) and loaded most on Factor 1; Forward Memory Span and Reverse Memory Span, which assess short-term and working memory (Richardson, 2007) and loaded most strongly on Factor 2; and Trail Making A and Trail Making B, which are measures of attention and speed of processing (Army Individual Test Battery, 1944), and load most strongly on Factor 3 These groupings are in line with the specific cognitive functions targeted by the standard pencil-paper neuropsychological assessments on which the NCPT subtests are based. Progressive Matrices, a measure of fluid reasoning and problem solving (Prabhakaran et al., 1997), loaded equally on Factors 1, 3, and 4 and did correlate strongly with other subtests in this battery. This may not be unexpected since Progressive Matrices is the only measure of fluid intelligence included in the tested battery.

A battery with good test-retest reliability increases the likelihood of obtaining the same scores under multiple administrations and supports its use as a longitudinal measure of cognitive performance. Good test-retest reliability indicates that the battery has minimal measurement error related to random variance (Anastasi and Urbina, 1997). Neuropsychological tests have been shown to have good to high test-retest reliability in the range of *r* = 0.70–0.90 (Bird et al., 2003; Williams et al., 2005), with the exception of memory tests, where lower reliability coefficients have been consistently observed (Dikmen et al., 1999). The NCPT battery used in these analyses has good test-retest reliability of *r* = 0.83 for the Grand Index score with a mean inter-trial interval of approximately 78.8 days. In general, reliability of the NCPT battery was highest with the shortest inter-trial interval and age had no effect until age 50, where reliability then decreased with increasing age (see Supplementary Tables 3, 4). The age effects may be due to the interaction of age-related decline in performance on the NCPT and inter-trial interval (Lemay et al., 2004).

Overall, the reliability values for each of the NCPT subtests were within the range reported for other computerized neurocognitive tests (see Appendix E of Gualtieri and Johnson, 2006). With the exception of Progressive Matrices (*r* = 0.388), correlations for the individual subtests at time 1 and time 2 were moderate ranging from *r* = 0.510 for Reverse Memory Span to *r* = 0.738 for Digit Symbol Coding. These values do trend lower compared to the original published data (see Supplementary Table 5). In particular, the relatively low correlation coefficient for Progressive Matrices observed in the NCPT battery was in contrast to the high test-retest correlation for pencil-paper versions reported by Burke (*r* = 0.96) (Burke, 2010) and a computerized version of Raven's Progressive Matrices reported by Williams (*r* = 0.95) (Williams and McCord, 2006). However, the low reliability for Progressive Matrices had little effect on the overall reliability of the NCPT Grand Index score; reliability of the Grand Index without Progressive Matrices was near identical to the Grand Index reliability including Progressive Matrices. Digit Symbol Coding, which had the highest correlation coefficient of the NCPT subtests, was also lower than its pencil paper correlate, the WAIS Digit Symbol Substitution subtest (*r* = 0.82) (Wechsler, 1981).

One hypothesis for the lower reliability is that participants were free to engage with Lumosity during the inter-trial interval; the range in number of unique days engaging with Lumosity between testing was broad (0–185 days), as was the total number of game played (0–8997). Lumosity cognitive training has been demonstrated to increase NCPT scores (Hardy et al., 2015). Consistent with this finding, there was a small, but significant effect on test-retest reliability when number of games played was the measure of engagement; however, when test-retest reliability is limited to those participants with no days playing Lumosity between time 1 and time 2, the correlation coefficients are nearly identical to those who played 70 unique days (the prescribed ITI). The low correlation coefficients for the NCPT subtests compared to their pencil-paper correlates demonstrates that there is room for improvement in the reliability of some of the subtests currently in use and highlights one of the challenges for remote computerized cognitive testing compared to testing in a controlled, and supervised environment. It is perhaps, not unexpected that remote, unsupervised, cognitive testing produces lower test-retest reliability than testing in a controlled, supervised environment. Despite the many advantages for remote computerized cognitive testing, administering neuropsychological tests in the absence of a trained test administrator is likely to produce more measurement error. In this particular case, not only was there the opportunity to play Lumosity between testing, but due to the nature of remote testing there is also uncertainty around consistent administration. A simple lack of consistent administration, for example taking the test in two different rooms or on two different computers, may lead to lack of consistent measurement results; thus, negatively impacting reliability.

Practice effects are distinct from day-to-day fluctuations in performance (i.e., reliability) and refer to a bias that is introduced at subsequent test sessions, due to familiarity with the test procedure and also specific test items. It is theoretically possible for a test to be very reliable and yet show large effects of practice (Bird et al., 2003). Practice effects have the ability to alter the interpretation of change in cognitive performance (Collie et al., 2003). Individual practice effects vary according to age, ability, and the complexity of the task (Rabbitt et al., 2001). An important variable influencing practice effects is whether or not alternative versions or forms of the test are available (Bird et al., 2003). The ability to customize the NCPT with dynamically generated content results in a battery with a virtually unlimited number of versions available making them more repeatable, which may serve to reduce practice effects related to content. Practice effects for the tested NCPT battery were minimal; the mean (SD) change from baseline for those who did not play between testing was 1.1 (8.47) points. Due to the limited alternate forms or versions for standard pencil-paper assessments, the same cannot be stated as these standard assessments are prone to practice effects with repeated applications of the same forms over brief inter-trial intervals (Wild et al., 2008).

Internal consistency reflects the coherence (or redundancy) of the components of an assessment and is conceptually independent of test-retest reliability (McCrae et al., 2011). The components of the NCPT (i.e., subtests) loaded onto three distinct factor groupings (in addition to Progressive Matrices) and while they each contribute to the aggregated score, they are measuring different cognitive domains. Given the intended use of the NCPT (measuring longitudinal stability or change in cognition) test-retest reliability is more relevant than internal consistency (McCrae et al., 2011). To this end, we report Pearson's correlation coefficients to measure test-retest reliability of each subtest, capture consistency across items within a subtest, and capture the effects of time and practice on each subtest.

Observed performance on the NCPT subtests demonstrated expected changes with age, replicating well-known trajectories of cognitive decline in healthy aging that show lower performance with increasing age (Salthouse, 1996). Notably for Arithmetic Reasoning, Grammatical Reasoning, and Trail Making B, performance peaked in the 20 s and then plateaued (or declined slowly) until about age 50 before declining in a linear fashion. In general the pattern of decline as an interaction of age may be reflective of fluid and crystallized intelligence (Cattell, 1987). Fluid intelligence is independent of acquired knowledge and reflects the capacity to think logically and solve problems in novel situations, whereas crystallized intelligence is the ability to use skills, knowledge, and experience. Progressive Matrices is an example of a non-verbal measure of fluid intelligence that does not rely on crystallized knowledge (Prabhakaran et al., 1997) and shows the expected approximately linear decline in performance with increased age (Salthouse, 1996). This is also evident for subtests such as Trail Making A and Digit Symbol Coding that include a speeded component, which is known to be highly susceptible to age (Salthouse, 1996). Arithmetic Reasoning, on the other hand, is a complex task that requires numerical calculation (Prabhakaran et al., 2001) and may be more protected from age-related decline because it relies on components of crystallized intelligence, including accessing information from long-term memory, such as general mathematical knowledge and reading ability (Prabhakaran et al., 1997, 2001).

Participants with more years of education obtained higher scores on each of the NCPT subtests and on the Grand Index. Further investigation on the interaction of age and education revealed that the decline with age appears less steep for those with ≤ 12 years of education (see Supplementary Image 2). Capitani et al. (1996) proposed three different patterns of association that could be expected between age-related decline and education: (a) Parallelism: The age-related decline runs the same course in different educational groups, that is, no interaction is observed; (b) Protection: The age-related decline is attenuated in well-educated participants; and (c) Confluence: The initial advantage of well-educated groups in middle age is reduced in later life. Using a set of five neuropsychological tests in a group of Italian subjects aged 40–85 they report differential effects depending on the test: parallelism was observed for some tests (verbal fluency, spatial memory, progressive matrices) and protection for others (visual attention and verbal memory). They did not observe confluence. In contrast to these findings, visual inspection of the interaction between age and education for each of the NCPT subtests (see Supplementary Image 2) suggests that parallelism is observed for Grammatical Reasoning and Progressive Matrices, whereas confluence is observed for all other subtests and the Grand Index score; we do not see any protection.

Differences in Grand Index scores by ethnic groups were not compared since variability cannot be attributed to ethnicity *per se*; rather, they are likely the result of other factors (Rossetti et al., 2011). Acculturation, quality of education, literacy, and racial socialization play a more meaningful role in many of the differences in cognitive functioning that were previously interpreted as race-related than race/ethnicity in adjusting expectations for cognitive test scores and improving specificity of cognitive tests (Manly, 2008).

A validated, unsupervised, web-based cognitive assessment could have several wide-reaching benefits for measuring cognitive performance. First, in the medical space, it could facilitate longitudinal patient monitoring, leading to increased preventative care and decreased cost of screening and care for a vast number of medical conditions in which cognition is affected. Second, for clinical trials, it could serve as a fast and easy screening tool to evaluate potential clinical trial participants, creating “trial-ready cohorts” with specific cognitive profiles, or as an outcome measure, detecting cognitive change due to intervention. Finally, in research, the ease of deployment of unsupervised, web-based neurocognitive assessments provides the opportunity for more large-scale studies that would yield a better understanding of intervention efficacy and better characterization of cognitive profiles of healthy and clinical populations. The initial data reported here may support the use of the NCPT for all of these applications.

Additionally, as an initial test of the clinical utility of the NCPT for differentiating those with cognitive impairment from those who are cognitively normal, an exploratory analysis found that NCPT Grand Index scores for the tested battery were found to be 0.78 standard deviations (*p* < 0.05) lower than those of age, gender, and education matched healthy adults 50 years and older for those who self-reported a diagnosis of MCI. Similarly, those who self-reported a diagnosis of AD were 1.17 standard deviations (*p* < 0.05) lower than older individuals from the Normative Sample. These data are encouraging given that the diagnoses were self-reported online and not clinically confirmed. However, given the basic functional skills needed to navigate the NCPT subtest tutorials before proceeding to take the assessment without assistance, this group of self-report individuals is probably not fully representative of a clinically confirmed cohort. Follow-up, well-controlled studies in patients with clinically confirmed diagnoses are needed to establish the sensitivity and specificity for discriminating those with cognitive impairment from those who are cognitively normal.

The studies presented do have limitations. First, the data for the Normative Sample are derived from Lumosity users, the majority of which have paid for a premium account. These individuals present a sample that is skewed toward more highly educated and computer-competent individuals, which is not fully representative of the general population. It is possible that normative data from a computer-competent population may be different compared to a computer-naïve population (Feldstein et al., 1999). Presumably individuals signed-up for Lumosity with the intention of participating in computerized cognitive training. This could introduce bias as the study population is based largely on computer-literate individuals who may have been motivated to improve their NCPT scores between testing at time 1 and time 2. The motivation to improve on their scores may have resulted in the long inter-trial interval between testing and impacted the test-retest reliability. However, the effect on unique days playing Lumosity between testing had no statistically significant impact on reliability. Second, data for the Normative Sample were collected using unsupervised remote administration in which users typically took the assessment at home on their personal computers. This method of administration presents challenges that are both common to computerized testing in general, and specific to the NCPT. Invariably, the method of test administration for an unsupervised computerized assessment removes observation by a trained examiner and therefore control over the test environment. Testing in the absence of an examiner may result in important information not being collected, for example level of task engagement, display of emotion, frustration, or tendency to give up easily when confronted with more challenging test items (Bauer et al., 2012). Significant changes in test environment may include testing at different times of day, or increased distraction during one test compared to the other, that are likely to impact reliability. Unsupervised, remote testing also introduces the opportunity to “cheat” the system, for example, by having someone else take the test. However, with aggregated analyses as presented, the numbers of users who would have had to cheat would be extremely large to have any impact on the quality of the data. Lastly, simply translating or adapting existing standardized tests to computerized administration does not assume that these “new” computerized tests have the same response characteristics, reliability, and validity as traditional paper-and-pencil tests (or even other computerized assessment tests) (Schlegel and Gilliland, 2007; Wild et al., 2008). While we report data to support the reliability and validity of the current NCPT battery of eight subtests, additional NCPT subtests have been developed and now total 18. Future plans include building on the current data to further support validity (face, content, construct, concurrent, and discriminative), reliability and stability, and specificity and sensitivity of the NCPT by running large-scale online and in-clinic studies to generate data for all NCPT subtests.

## Author contributions

GM was responsible for drafting the manuscript and analysis and interpretation of the data. NN and CS were responsible for designing and conducting the concordance study, data acquisition, analysis and interpretation, and drafting and revising the manuscript. JH was responsible for developing the NCPT and drafting and revising the manuscript.

### Conflict of interest statement

Glenn E. Morrison, Christa M. Simone, Nicole F. Ng are employed at Lumos Labs, the company that produces the computerized cognitive assessment that is used in this study. These authors hold stock options in the company. Joseph L. Hardy is a former employee of Lumos Labs.

## References

[B1] American Educational Research Association (1999). Standards for Educational and Psychological Testing. Washington, DC: AERA.

[B2] Anastasi A. Urbina S. (1997). Psychological Testing, 7th Edn. Upper Saddle River, NJ: Prentice Hall.

[B3] Army Individual Test Battery (1944). Army Individual Test Battery: Manual of directions and scoring. Washington, DC: War Department, Adjutant General's Office.

[B4] Baddeley A. D. (1968). A 3 min reasoning test based on grammatical transformation. Psychon. Sci. 10, 341–342. 10.3758/BF03331551

[B5] Bauer R. M. Iverson G. L. Cernich A. N. Binder L. M. Ruff R. M. Naugle R. I. (2012). Computerized neuropsychological assessment devices: joint position paper of the american academy of clinical neuropsychology and the national academy of neuropsychology. Arch. Clin. Neuropsychol. 27, 362–373. 10.1093/arclin/acs027 22382386 PMC3499090

[B6] Bird C. M. Papadopoulou K. Ricciardelli P. Rossor M. N. Cipolotti L. (2003). Test-retest reliability, practice effects and reliable change indices for the recognition memory test. Br. J. Clin. Psychol. 42(Pt 4), 407–425. 10.1348/014466503322528946 14633416

[B7] Burke H. R. (2010). Raven's progressive matrices: validity, reliability, and norms. J. Psychol. 82, 253–257. 10.1080/00223980.1972.9923815

[B8] Capitani E. Barbarotto R. Laicana M. (1996). Does education influence age-related cognitive decline? A further inquiry. Dev. Neuropsychol. 12, 231–240. 10.1080/87565649609540648

[B9] Cattell R. B. (1987). Intelligence: Its Structure, Growth and Action: Its Structure, Growth and Action. Amsterdam: Elsevier Science Publishers B.V.

[B10] Collie A. Maruff P. Darby D. G. McStephen M. (2003). The effects of practice on the cognitive test performance of neurologically normal individuals assessed at brief test-retest intervals. J. Int. Neuropsychol. Soc. 9, 419–428. 10.1017/S1355617703930074 12666766

[B11] Deloche G. Seron X. Larroque C. Magnien C. Metz-Lutz M. N. Noel M. N.. (1994). Calculation and number processing: assessment battery; role of demographic factors. J. Clin. Exp. Neuropsychol. 16, 195–208. 10.1080/01688639408402631 8021307

[B12] Dikmen S. S. Heaton R. K. GRANT I. Temkin N. R. (1999). Test-retest reliability and practice effects of expanded Halstead-Reitan Neuropsychological Test Battery. J. Int. Neuropsychol. Soc. 5, 346–356. 10.1017/S1355617799544056 10349297

[B13] Feldstein S. N. Keller F. R. Portman R. E. Durham R. L. Klebe K. J. Davis H. P. (1999). A comparison of computerized and standard versions of the Wisconsin Card Sorting Test. Clin. Neuropsychol. 13, 303–313. 10.1076/clin.13.3.303.1744 10726602

[B14] Ford J. K. MacCallum R. C. Tail M. (1986). The application of exploratory factor analysis in applied psychology: a critical review and analysis. Pers. Psychol. 39, 291–314. 10.1111/j.1744-6570.1986.tb00583.x

[B15] Gualtieri C. Johnson L. (2006). Reliability and validity of a computerized neurocognitive test battery, CNS Vital Signs. Arch. Clin. Neuropsychol. 21, 623–643. 10.1016/j.acn.2006.05.007 17014981

[B16] Gulliksen H. (1950). Effect of Group Heterogeneity on Test Reliability. Theory of Mental Tests. Hoboken: John Wiley & Sons Inc 108–127.

[B17] Gur R. C. Richard J. Hughett P. Calkins M. E. Macy L. Bilker W. B.. (2010). A cognitive neuroscience-based computerized battery for efficient measurement of individual differences: standardization and initial construct validation. J. Neurosci. Methods 187, 254–262. 10.1016/j.jneumeth.2009.11.017 19945485 PMC2832711

[B18] Hardy J. L. Nelson R. A. Thomason M. E. Sternberg D. A. Katovich K. Farzin F.. (2015). Enhancing cognitive abilities with comprehensive training: a large, online, randomized, active-controlled trial. PLoS ONE 10:e0134467. 10.1371/journal.pone.0134467 26333022 PMC4557999

[B19] Hu L. Bentler P. (1999). Cutoff criteria for fit indexes in covariance structure analysis: conventional criteria versus new alternatives. Struct. Equat. Model. Multidis. J. 6, 1–55. 10.1080/10705519909540118

[B20] Kane R. L. Kay G. G. (1992). Computerized assessment in neuropsychology: a review of tests and test batteries. Neuropsychol. Rev. 3, 1–117. 10.1007/BF01108787 1300218

[B21] Keefe R. S. Goldberg T. E. Harvey P. D. Gold J. M. Poe M. P. Coughenour L. (2004). The Brief Assessment of Cognition in Schizophrenia: reliability, sensitivity, and comparison with a standard neurocognitive battery. Schizophr. Res. 68, 283–297. 10.1016/j.schres.2003.09.011 15099610

[B22] Kueider A. M. Parisi J. M. Gross A. L. Rebok G. W. (2012). Computerized cognitive training with older adults: a systematic review. PLoS ONE 7:e40588. 10.1371/journal.pone.0040588 22792378 PMC3394709

[B23] Lampit A. Hallock H. Valenzuela M. (2014). Computerized cognitive training in cognitively healthy older adults: a systematic review and meta-analysis of effect modifiers. PLoS Med. 11:e1001756. 10.1371/journal.pmed.1001756 25405755 PMC4236015

[B24] Lemay S. Bédard M. A. Rouleau I. (2004). Practice effect and test-retest reliability of attentional and executive tests in middle-aged to elderly subjects. Clin. Neuropsychol. 18, 1–19. 10.1080/13854040490501718 15587675

[B25] Manly J. (2008). Race, culture, education, and cognitive test performance among older adults, in Handbook of Cognitive Aging: Interdisciplinary Perspectives, eds Hofer S. Alwin D. (Thousand Oaks, CA: SAGE Publications, Inc), 398–418.

[B26] Maruff P. Collie A. Darby D. Weaver-Cargin J. Masters C. Currie J. (2004). Subtle memory decline over 12 months in mild cognitive impairment. Dement. Geriatr. Cogn. Disord. 18, 342–348. 10.1159/000080229 15316183

[B27] McCrae R. R. Kurtz J. E. Yamagata S. Terracciano A. (2011). Internal consistency, retest reliability, and their implications for personality scale validity. Pers. Soc. Psychol. Rev. 15, 28–50. 10.1177/1088868310366253 20435807 PMC2927808

[B28] Milner B. (1971). Interhemispheric differences in the localization of psychological processes in man. Br. Med. Bull. 27, 272–277. 4937273 10.1093/oxfordjournals.bmb.a070866

[B29] Nakayama Y. Covassin T. Schatz P. Nogle S. Kovan J. (2014). Examination of the Test-Retest Reliability of a Computerized Neurocognitive Test Battery. Am. J. Sports Med. 42, 2000–2005. 10.1177/0363546514535901 24907286

[B30] Nuechterlein K. H. Green M. F. Kern R. S. Baade L. E. Barch D. M. Cohen J. D.. (2008). The MATRICS Consensus Cognitive Battery, part 1: test selection, reliability, and validity. Am. J. Psychiatry 165, 203–213. 10.1176/appi.ajp.2007.07010042 18172019

[B31] Prabhakaran V. Rypma B. Gabrieli J. D. E. (2001). Neural substrates of mathematical reasoning: a functional magnetic resonance imaging study of neocortical activation during performance of the necessary arithmetic operations test. Neuropsychology 15, 115–127. 10.1037/0894-4105.15.1.115 11216882

[B32] Prabhakaran V. Smith J. A. Desmond J. E. Glover G. H. Gabrieli J. D. (1997). Neural substrates of fluid reasoning: an fMRI study of neocortical activation during performance of the Raven's Progressive Matrices Test. Cogn. Psychol. 33, 43–63. 9212721 10.1006/cogp.1997.0659

[B33] Rabbitt P. Diggle P. Smith D. Holland F. Mc Innes L. (2001). Identifying and separating the effects of practice and of cognitive ageing during a large longitudinal study of elderly community residents. Neuropsychologia 39, 532–543. 10.1016/S0028-3932(00)00099-3 11254936

[B34] Raven J. (2000). The Raven's progressive matrices: change and stability over culture and time. Cogn Psychol. 41, 1–48. 10.1006/cogp.1999.0735 10945921

[B35] R Core Team (2015). R: A Language and Environment for Statistical Computing. Vienna: R Foundation for Statistical Computing Available online at: http://www.R-project.org/

[B36] Reitan R. M. Wolfson D. (1995). The Halstead–Reitan Neuropsycholgical Test Battery: Therapy and Clinical Interpretation. Tucson, AZ: Neuropsychological Press.

[B37] Revelle W. (2015). Psych: Procedures for Personality and Psychological Research. Evanston, IL: Northwestern University Available online at: http://CRAN.R-project.org/package=psych Version = 1.5.8.

[B38] Richardson J. T. E. (2007). Measures of short-term memory: a historical review. Cortex 43, 635–650. 10.1016/S0010-9452(08)70493-3 17715798

[B39] Robbins T. W. James L. F. Owen A. M. Sahakian B. J. Lawrence A. D. McInnes L.. (1998). A study of performance on tests from the CANTAB battery sensitive to frontal lobe dysfunction in a large sample of normal volunteers: implications for theories of executive functioning and cognitive aging. Cambridge Neuropsychological Test Automated Battery. J. Int. Neuropsychol. Soc. 4, 474–490. 10.1017/s1355617798455073 9745237

[B40] Rossetti H. C. Lacritz L. H. Cullum C. M. Weiner M. F. (2011). Normative data for the Montreal Cognitive Assessment (MoCA) in a population-based sample. Neurology 77, 1272–1275. 10.1212/WNL.0b013e318230208a 21917776

[B41] Royer F. L. (1971). Spatial orientational and figural information in free recall of visual figures. J. Exp. Psychol. 91, 326–332. 10.1037/h0031846 5134676

[B42] Salthouse T. A. (1996). The processing-speed theory of adult age differences in cognition. Psychol. Rev. 103, 403–428. 10.1037/0033-295X.103.3.403 8759042

[B43] Schlegel R. Gilliland K. (2007). Development and quality assurance of computer-based assessment batteries. Arch. Clin. Neuropsychol. 22, 49–61. 10.1016/j.acn.2006.10.005 17085010

[B44] Sekhon J. S. (2011). Multivariate and propensity score matching software with automated balance optimization: the matching package for R. J. Statist. Softw. 42, 1–52.

[B45] Wechsler D. (1945). Wechsler Memory Scale. San Antonio, TX: Psychological Corporation.

[B46] Wechsler D. (1955). Manual for the Wechsler Adult Intelligence Scale. Oxford, England: Psychological Corp 110.

[B47] Wechsler D. (1981). Wechsler Adult Intelligence Scale-Revised. San Antonio, TX: Psychological Corporation.

[B48] Wild K. Howieson D. Webbe F. Seelye A. Kaye J. (2008). Status of computerized cognitive testing in aging: a systematic review. Alzheim. Demen. 4, 428–437. 10.1016/j.jalz.2008.07.003 19012868 PMC2645803

[B49] Williams J. E. McCord D. M. (2006). Equivalence of standard and computerized versions of the Raven Progressive Matrices Test. Comput. Hum. Behav. 22, 791–800. 10.1016/j.chb.2004.03.005

[B50] Williams L. M. Simms E. Clark C. R. Paul R. H. Rowe D. Gordon E. (2005). The test-retest reliability of a standardized neurocognitive and neurophysiological test battery: "neuromarker". Int. J. Neurosci. 115, 1605–1630. 10.1080/00207450590958475 16287629

[B51] Wood S. N. (2011). Fast stable restricted maximum likelihood and marginal likelihood estimation of semiparametric generalized linear models. J. R. Statist. Soc. B 73, 3–36. 10.1111/j.1467-9868.2010.00749.x

[B52] Zygouris S. Tsolaki M. (2014). Computerized cognitive testing for older adults: a review. Am. J. Alzheimers. Dis. Other Demen. 30, 13–28. 10.1177/1533317514522852 24526761 PMC10852880

