# Supplementary material for: Reliability and validity of the NeuroCognitive Performance Test, a web-based neuropsychological assessment
Source: Front Psychol. 2015 Nov 3;6:1652. doi: 10.3389/fpsyg.2015.01652 (PMC4630791; doi:10.3389/fpsyg.2015.01652)
Supplement: Supplementary file 1 [file Table1.PDF]

Supplementary Table 1. Normative sample demographics. Demographic summaries by 5-year age bin (age 13-89).

	Overall	13-19	20-24	25-29	30-34	35-39	40-44	45-49	50-54
N (%)	130,140	6,128 (4.7)	10,678 (8.2)	13,590 (10.4)	11,415 (8.8)	8,865 (6.8)	9,005 (6.9)	10,268 (7.9)	12,493 (9.9)
% Female	53.3	39.1	36.9	40.8	41.7	44.0	49.0	56.1	62.5
Race, n (%)									
White	100,065 (76.9)	3,693 (60.2)	6,863 (64.3)	9,077 (66.8)	7,654 (67.1)	6,069 (68.5)	6,534 (72.6)	8,036 (78.3)	10,629 (82.1)
Black	3,032 (2.3)	165 (2.7)	361 (3.4)	373 (2.7)	322 (2.8)	267 (3.0)	255 (2.8)	267 (2.6)	275 (2.1)
Hispanic	5,735 (4.4)	360 (5.9)	720 (6.7)	928 (6.8)	780 (6.8)	606 (6.8)	480 (5.3)	468 (4.6)	447 (3.5)
Asian	8,105 (6.2)	658 (10.7)	1,206 (11.3)	1,457 (10.7)	1,191 (10.4)	862 (9.7)	728 (8.1)	554 (5.4)	446 (3.4)
Other	13,203 (10.1)	1,252 (20.4)	1.528 (14.3)	1,755 (12.9)	1,468 (12.9)	1,061 (12.0)	1,008 (11.2)	943 (9.2)	1,146 (8.9)
Handedness (%R)	76.6	74.2	77.0	77.5	77.6	76.8	76.0	75.3	76.5
Education, years (%)									
0-12 years	14.4	61.4	14.2	8.4	8.6	9.2	10.1	12.2	14.1
13-16 years	45.4	18.2	64.2	50.7	45.3	43.8	44.4	44.8	47.2
17+ years	30.2	1.9	12.0	32.3	37.0	37.5	35.9	33.5	29.4
ND	9.9	18.5	9.7	8.5	9.2	9.5	9.6	9.5	9.3
Household Income (%)									
$0-24,999	4.7	8.8	17.3	9.6	4.4	2.5	1.7	1.6	1.7
$25,000-49,999	9.9	2.6	14.9	18.3	12.1	7.9	6.6	6.1	6.2
$50,000-99,999	21.0	4.9	11.3	23.7	25.4	22.4	19.9	18.9	20.2
$100,000-199,999	19.1	4.5	4.4	10.3	19.8	25.1	25.7	26.4	25.6
≥$200,000	9.0	2.9	2.2	1.9	5.7	9.9	14.7	16.3	15.1
ND	36.3	76.3	49.9	36.1	32.5	32.2	31.7	30.7	31.2

	**55-59**	**60-64**	**65-69**	**70-74**	**75-79**	**80-89**
N (%)	13,687 (10.5)	13,012 (10.0)	10,804 (8.3)	5,941 (4.6)	2,540 (2.0)	1,264 (1.0)
% Female	65.6	66.1	64.0	62.9	63.6	55.5
Race, n (%)						
White	11,676 (85.3)	11,369 (87.4)	9,620 (89.0)	5,372 (90.4)	2,324 (91.5)	1,148 (90.8)
Black	249 (1.8)	233 (1.8)	161 (1.5)	71 (1.2)	20 (0.8)	13 (1.0)
Hispanic	402 (2.9)	257 (2.0)	176 (1.6)	79 (1.3)	26 (1.0)	6 (0.4)
Asian	343 (2.5)	319 (2.5)	186 (1.7)	82 (1.4)	47 (1.9)	26 (2.1)
Other	1,017 (7.4)	834 (6.4)	661 (6.1)	337 (5.7)	123 (4.8)	71 (5.6)
Handedness (%R)	76.2	76.7	76.4	76.9	77.7	77.2
Education, years (%)						
0-12 years	14.0	12.6	12.7	15.6	18.1	17.6
13-16 years	45.4	44.0	42.5	41.3	39.6	41.9
17+ years	30.5	33.9	34.9	33.5	32.2	29.0
ND	10.1	9.5	9.9	9.6	10.1	11.4
Household Income (%)						
$0-24,999	1.9	2.4	2.8	3.3	4.1	6.3
$25,000-49,999	7.0	8.5	10.9	12.3	14.5	15.1
$50,000-99,999	21.8	24.1	25.9	24.7	24.7	25.4
$100,000-199,999	24.7	22.6	19.1	16.3	13.6	10.4
≥$200,000	13.3	10.6	7.7	6.6	4.4	4.3
ND	31.3	31.8	33.7	36.9	38.8	38.4
