# Supplementary material for: Reliability and validity of the NeuroCognitive Performance Test, a web-based neuropsychological assessment
Source: Front Psychol. 2015 Nov 3;6:1652. doi: 10.3389/fpsyg.2015.01652 (PMC4630791; doi:10.3389/fpsyg.2015.01652)
Supplement: Supplementary file 2 [file Table2.PDF]

Supplementary Table 2. Normative sample baseline NCPT raw scores. Mean (SD) scores for each NCPT subtest by age, education, and gender. TA = Trail Making A, TB = Trail Making B, FMS = Forward Memory Span, RMS = Reverse Memory Span, DSC = Digit Symbol Coding, PM = Progressive Matrices, AR = Arithmetic Reasoning, GR = Grammatical Reasoning.

Age	Education	Gender	N	TA (s) Mean	TA SD	TB (s) Mean	TB SD	FMS (correct) Mean	FMS SD	RMS (correct) Mean	RMS SD	DSC (correct – incorrect) Mean	DSC SD	PM (correct) Mean	PM SD	AR (correct – incorrect) Mean	AR SD	GR (correct – incorrect) Mean	GR SD
13-19			6,128	21.1	21.1	7.5	37.9	17.9	10.11	2.65	8.68	2.67	47.36	10.94	9.70	3.54	14.12	5.19	7.97
	0-12,years	Female	898	20.3	5.1	34.2	11.9	10.09	2.44	8.67	2.54	49.48	9.16	9.74	3.30	14.59	4.78	8.32	4.68
		Male	1,411	19.6	5.7	35.5	14.8	10.71	2.50	9.23	2.49	49.93	10.07	10.13	3.63	14.99	5.03	8.43	4.88
	13-16,years	Female	415	20.5	5.7	34.4	15.1	10.21	2.49	9.07	2.54	51.33	9.71	10.57	3.29	14.80	4.74	8.66	4.82
		Male	638	19.3	5.8	34.3	12.7	10.96	2.58	9.30	2.59	52.57	10.85	10.80	3.60	15.93	5.09	9.18	5.02
	17+,years	Female	853	23.1	8.7	42.7	22.7	9.27	2.54	8.00	2.65	42.31	9.87	8.98	3.42	12.35	4.94	6.85	4.53
		Male	954	22.8	9.9	43.5	21.8	9.56	2.78	8.02	2.71	41.94	10.12	8.70	3.47	13.00	5.16	7.01	4.79
20-24			10,678	19.6	5.8	33.7	13.3	10.62	2.56	9.20	2.53	52.84	10.28	11.04	3.39	15.43	5.01	9.09	4.68
	0-12,years	Female	365	21.2	5.9	36.7	14.9	9.67	2.35	8.42	2.35	50.01	9.12	9.87	3.42	12.34	4.60	7.78	4.38
		Male	967	20.2	6.0	37.8	15.6	10.46	2.43	8.90	2.46	50.00	9.86	10.22	3.58	13.41	4.56	8.17	4.52
	13-16,years	Female	2,632	20.0	6.2	32.2	11.3	10.28	2.44	9.03	2.47	53.03	9.20	10.94	3.19	15.23	4.74	9.30	4.57
		Male	3,905	18.9	5.5	33.3	13.2	11.00	2.59	9.46	2.53	53.63	10.76	11.39	3.37	16.15	4.99	9.28	4.75
	17+,years	Female	589	20.1	5.9	32.5	12.4	10.34	2.65	9.01	2.64	53.59	10.24	11.06	3.41	15.76	4.89	9.11	4.54
		Male	784	19.4	5.5	34.6	14.3	10.93	2.63	9.40	2.68	52.89	11.18	11.30	3.51	15.97	5.42	8.85	4.84
25-29			13,590	19.9	6.6	33.6	13.1	10.53	2.55	9.13	2.52	52.77	10.04	11.26	3.29	15.60	4.95	9.02	4.59
	0-12,years	Female	300	22.0	8.7	40.0	22.6	9.72	2.51	8.48	2.44	50.00	9.61	9.58	3.49	12.58	4.36	7.33	4.54
		Male	712	21.0	6.5	37.9	14.9	10.40	2.60	8.89	2.54	49.62	10.27	10.42	3.61	13.39	4.67	7.96	4.46
	13-16,years	Female	2,695	20.3	6.2	33.6	12.6	10.06	2.47	8.84	2.48	52.53	9.26	10.80	3.15	15.05	4.73	9.12	4.58
		Male	3,875	19.4	6.3	33.4	12.4	10.90	2.51	9.36	2.48	52.96	10.00	11.41	3.27	15.93	4.86	9.03	4.53
	17+,years	Female	2,094	20.1	6.1	31.8	11.3	10.18	2.46	9.12	2.51	53.30	9.49	11.47	3.07	15.92	4.86	9.20	4.67
		Male	2,190	19.1	6.7	32.9	13.7	11.01	2.59	9.39	2.61	53.72	10.88	11.95	3.30	16.41	5.14	9.37	4.60
30-34			11,415	20.9	7.4	35.0	14.8	10.29	2.55	8.89	2.48	51.41	9.88	11.22	3.30	15.64	4.94	8.85	4.49

Age	Education	Gender	N	TA (s) Mean	TA SD	TB (s) Mean	TB SD	FMS (correct) Mean	FMS SD	RMS (correct) Mean	RMS SD	DSC (correct – incorrect) Mean	DSC SD	PM (correct) Mean	PM SD	AR (correct – incorrect) Mean	AR SD	GR (correct – incorrect) Mean	GR SD
	0-12,years	Female	269	23.3	10.6	38.1	14.7	9.31	2.40	8.06	2.20	49.46	9.43	9.80	3.19	13.06	4.63	7.42	4.21
		Male	591	21.9	7.5	39.0	15.4	10.20	2.62	8.63	2.60	48.27	9.25	10.14	3.46	13.79	4.53	7.92	4.40
	13-16,years	Female	2,026	21.2	6.4	34.6	13.7	9.93	2.46	8.67	2.42	51.96	9.79	10.78	3.24	14.95	4.74	8.74	4.34
		Male	2,880	20.4	7.8	35.6	15.6	10.61	2.53	9.05	2.42	51.54	10.15	11.43	3.32	15.74	4.89	8.77	4.44
	17+,years	Female	2,023	21.1	7.4	33.8	14.4	9.94	2.49	8.72	2.47	51.91	9.27	11.40	3.12	16.05	4.88	9.34	4.48
		Male	2,084	20.2	7.0	34.1	14.3	10.73	2.58	9.29	2.53	51.57	10.20	11.76	3.28	16.56	4.97	9.01	4.70
35-39			8,865	21.5	7.6	35.8	15.8	9.97	2.53	8.66	2.47	49.53	9.16	11.02	3.26	15.79	4.85	8.67	4.40
	0-12,years	Female	250	22.3	7.4	39.4	18.2	9.07	2.30	8.06	2.42	48.98	8.40	9.89	3.22	13.89	4.77	7.78	4.31
		Male	441	23.1	13.2	41.7	21.8	9.92	2.56	8.41	2.51	46.10	8.57	9.95	3.47	13.53	4.46	7.58	4.05
	13-16,years	Female	1,706	21.6	6.8	35.3	13.3	9.54	2.51	8.29	2.45	50.24	9.11	10.68	3.14	15.28	4.68	8.57	4.46
		Male	1,948	20.9	6.8	35.4	13.9	10.39	2.53	8.93	2.45	49.37	9.36	11.09	3.28	15.97	4.76	8.84	4.35
	17+,years	Female	1,589	21.9	8.1	34.5	15.1	9.65	2.42	8.48	2.45	49.84	8.47	11.22	3.17	16.21	4.83	8.98	4.38
		Male	1,651	21.0	7.0	35.3	16.4	10.39	2.54	9.01	2.50	49.58	9.40	11.58	3.26	16.62	4.88	8.86	4.54
40-44			9,005	22.4	8.7	37.0	16.6	9.69	2.53	8.40	2.48	47.66	8.40	10.80	3.27	15.77	4.78	8.48	4.33
	0-12,years	Female	347	23.0	7.0	39.8	18.1	8.77	2.44	7.66	2.29	47.49	8.88	9.73	3.36	13.53	4.75	7.13	4.29
		Male	417	24.0	12.1	41.7	18.9	9.53	2.50	8.19	2.52	44.12	8.52	9.95	3.44	13.67	4.54	7.67	4.12
	13-16,years	Female	1,914	22.6	8.6	37.3	16.1	9.23	2.43	8.02	2.41	48.48	8.37	10.48	3.17	15.25	4.63	8.22	4.13
		Male	1,828	22.1	10.3	36.9	18.3	10.19	2.52	8.81	2.49	47.27	8.14	10.93	3.23	16.07	4.65	8.56	4.16
	17+,years	Female	1,744	22.3	7.2	35.6	15.2	9.42	2.45	8.24	2.39	48.56	8.26	11.03	3.15	16.29	4.84	8.90	4.57
		Male	1,412	21.9	7.3	35.8	14.5	10.23	2.52	8.75	2.55	47.17	8.42	11.38	3.28	16.55	4.75	8.61	4.30
45-49			10,268	23.6	10.0	38.7	18.0	9.13	2.51	7.96	2.44	45.36	7.80	10.39	3.33	15.51	4.61	8.18	4.18
	0-12,years	Female	618	24.8	12.7	43.0	20.5	8.34	2.55	7.19	2.36	44.32	7.97	9.40	3.19	13.42	4.36	6.74	3.99
		Male	467	24.7	9.0	42.7	19.5	9.12	2.44	8.04	2.39	42.00	6.84	9.57	3.46	13.76	4.40	7.20	3.99
	13-16,years	Female	2,671	23.7	9.0	38.8	17.2	8.81	2.44	7.64	2.41	45.97	8.08	10.20	3.27	15.21	4.51	8.01	4.11
		Male	1,653	22.9	8.6	38.5	18.9	9.71	2.52	8.42	2.44	44.86	7.30	10.56	3.41	15.60	4.48	8.31	4.03
	17+,years	Female	1,947	24.0	10.5	37.8	17.3	8.87	2.42	7.80	2.36	45.78	7.72	10.55	3.23	16.24	4.61	8.65	4.25

Age	Education	Gender	N	TA (s) Mean	TA SD	TB (s) Mean	TB SD	FMS (correct) Mean	FMS SD	RMS (correct) Mean	RMS SD	DSC (correct – incorrect) Mean	DSC SD	PM (correct) Mean	PM SD	AR (correct – incorrect) Mean	AR SD	GR (correct – incorrect) Mean	GR SD
		Male	1,368	22.4	6.6	36.5	17.0	9.79	2.56	8.59	2.47	45.72	7.59	11.15	3.29	16.44	4.62	8.72	4.25
50-54			12,943	24.6	9.5	41.9	21.1	8.47	2.47	7.45	2.36	42.99	7.04	10.12	3.29	14.90	4.42	7.73	4.06
	0-12,years	Female	1,074	25.2	11.7	45.7	25.0	7.95	2.41	6.83	2.24	42.63	7.23	9.28	3.24	13.50	4.18	6.27	3.93
		Male	557	26.1	9.8	47.4	23.0	8.37	2.65	7.50	2.45	39.43	6.07	9.15	3.53	12.90	4.16	6.72	3.79
	13-16,years	Female	3,853	24.6	9.8	41.3	20.3	8.27	2.40	7.25	2.31	43.89	7.31	10.12	3.24	14.69	4.39	7.66	4.01
		Male	1,782	24.2	7.8	41.8	19.9	8.87	2.48	7.80	2.38	41.96	6.58	10.04	3.31	15.12	4.21	7.90	3.99
	17+,years	Female	2,436	24.6	9.2	40.4	20.2	8.36	2.43	7.44	2.33	43.46	6.97	10.50	3.20	15.51	4.38	8.24	4.08
		Male	1,228	24.1	10.1	40.1	20.5	9.15	2.44	8.02	2.42	42.68	6.32	10.66	3.35	16.06	4.37	8.40	4.05
55-59			13,687	25.8	10.8	44.8	23.4	7.86	2.50	6.98	2.34	41.04	6.69	9.87	3.33	14.56	4.22	7.37	3.91
	0-12,years	Female	1,229	26.6	12.2	50.0	28.1	7.25	2.55	6.41	2.39	40.75	7.10	9.00	3.31	12.97	4.20	6.30	3.76
		Male	493	27.5	11.5	52.9	28.5	7.69	2.62	6.77	2.38	37.49	6.30	8.84	3.58	12.61	4.03	6.38	3.61
	13-16,years	Female	4,147	25.5	10.8	44.4	22.7	7.72	2.50	6.82	2.30	41.83	6.84	9.84	3.23	14.41	4.13	7.29	3.89
		Male	1,612	25.5	8.8	44.8	22.5	8.30	2.46	7.33	2.35	39.84	6.16	9.78	3.43	14.81	4.02	7.46	3.86
	17+,years	Female	2,748	25.4	10.7	42.0	21.0	7.88	2.38	7.03	2.28	41.74	6.51	10.46	3.12	15.32	4.23	7.86	3.94
		Male	1,249	26.0	12.3	43.1	19.8	8.40	2.44	7.58	2.38	40.57	6.17	10.16	3.45	15.45	4.21	7.88	4.04
60-64			13,012	27.2	12.4	48.3	25.7	7.39	2.54	6.59	2.28	38.92	6.23	9.49	3.36	14.05	4.01	7.00	3.84
	0-12,years	Female	1,050	27.2	11.1	53.7	30.2	6.82	2.61	6.10	2.27	38.89	6.48	8.50	3.30	12.59	3.82	5.80	3.63
		Male	420	28.7	17.8	55.5	33.3	7.52	2.58	6.54	2.22	36.25	5.95	8.28	3.60	12.74	4.03	6.34	3.61
	13-16,years	Female	3,794	26.8	12.0	48.0	25.3	7.19	2.55	6.43	2.23	39.57	6.45	9.46	3.26	13.76	3.98	6.81	3.83
		Male	1,499	27.4	10.2	49.4	24.8	7.80	2.57	6.93	2.32	37.80	5.89	9.18	3.44	14.04	3.97	7.02	3.81
	17+,years	Female	2,991	27.1	12.9	45.7	24.0	7.41	2.47	6.61	2.23	39.46	5.98	10.04	3.25	14.67	3.97	7.54	3.86
		Male	1,166	26.5	9.9	44.5	20.6	7.98	2.51	7.18	2.31	38.42	5.82	10.08	3.43	15.18	3.80	7.60	3.81
65-69			10,804	29.0	14.9	54.4	31.3	6.73	2.62	6.07	2.32	36.50	6.04	9.02	3.41	13.19	3.84	6.38	3.74
	0-12,years	Female	931	29.5	16.1	60.6	35.9	5.94	2.57	5.43	2.29	35.98	6.21	7.96	3.41	11.66	3.74	5.20	3.52
		Male	282	31.0	15.9	63.9	36.7	6.63	2.65	5.97	2.34	33.46	5.82	7.76	3.61	11.59	3.82	5.07	3.71
	13-16,years	Female	2,991	28.3	13.7	54.8	32.2	6.60	2.53	5.94	2.25	36.94	6.10	9.01	3.30	12.89	3.72	6.16	3.65

Age	Education	Gender	N	TA (s) Mean	TA SD	TB (s) Mean	TB SD	FMS (correct) Mean	FMS SD	RMS (correct) Mean	RMS SD	DSC (correct – incorrect) Mean	DSC SD	PM (correct) Mean	PM SD	AR (correct – incorrect) Mean	AR SD	GR (correct – incorrect) Mean	GR SD
		Male	1,214	29.7	15.5	55.8	32.5	7.03	2.75	6.32	2.38	35.32	6.23	8.69	3.49	13.15	3.80	6.41	3.71
	17+,years	Female	2,340	28.9	15.8	50.2	26.6	6.85	2.52	6.18	2.28	37.19	5.89	9.72	3.24	13.96	3.82	6.99	3.78
		Male	1,235	29.5	15.2	51.5	29.4	7.24	2.66	6.57	2.31	36.34	5.71	9.28	3.44	14.24	3.79	7.08	3.82
70-74			5,941	30.4	15.3	62.0	36.5	6.06	2.73	5.46	2.35	34.11	5.93	8.53	3.42	12.09	3.82	5.73	3.62
	0-12,years	Female	608	30.5	15.2	67.6	37.4	5.54	2.69	4.93	2.41	34.20	6.49	7.55	3.28	10.82	3.84	4.76	3.35
		Male	204	32.6	17.1	73.0	42.8	5.46	2.88	5.00	2.39	31.65	5.63	7.07	3.37	10.88	3.52	4.50	3.37
	13-16,years	Female	1,599	29.5	13.9	63.3	37.5	5.96	2.71	5.41	2.37	34.42	6.09	8.59	3.25	11.73	3.75	5.52	3.46
		Male	631	30.7	12.7	59.1	33.7	6.47	2.74	5.82	2.36	33.62	5.62	8.50	3.49	12.40	3.70	5.89	3.59
	17+,years	Female	1,189	30.0	16.8	56.9	34.2	6.18	2.63	5.51	2.19	34.86	5.58	9.29	3.26	12.78	3.78	6.41	3.68
		Male	720	31.6	16.6	59.6	34.1	6.40	2.79	5.90	2.39	33.76	5.40	8.90	3.60	13.20	3.62	6.36	3.70
75-79			2,540	32.6	16.6	70.5	42.3	5.13	2.76	4.76	2.35	31.74	5.94	8.05	3.45	11.23	3.58	5.14	3.43
	0-12,years	Female	322	32.0	18.7	74.5	44.4	4.90	2.89	4.38	2.26	32.08	5.87	7.01	3.25	10.26	3.34	4.57	3.27
		Male	81	32.9	16.3	75.5	46.8	5.28	2.80	4.74	2.57	29.41	5.52	7.15	3.34	9.88	3.45	4.21	3.02
	13-16,years	Female	657	31.4	15.6	70.4	44.0	5.13	2.72	4.66	2.33	32.23	6.05	8.19	3.46	11.20	3.59	5.14	3.42
		Male	274	34.0	19.9	72.4	40.1	5.39	2.82	5.08	2.50	30.87	6.06	7.95	3.32	11.57	3.60	5.31	3.33
	17+,years	Female	484	32.6	15.9	66.5	38.4	5.11	2.64	4.82	2.28	32.53	6.07	8.50	3.43	11.60	3.56	5.36	3.37
		Male	309	33.4	13.4	67.9	40.2	5.40	2.73	5.09	2.36	31.02	5.71	8.50	3.66	12.34	3.55	5.72	3.62
80-89			1,264	35.5	19.1	82.8	49.2	4.60	2.90	4.22	2.50	29.23	6.20	7.67	3.41	10.21	3.58	4.57	3.31
	0-12,years	Female	129	33.8	16.9	90.5	52.0	4.63	2.79	4.05	2.49	29.06	6.47	6.86	3.61	9.54	3.20	4.19	3.24
		Male	58	41.5	34.8	94.0	61.9	4.10	2.77	3.72	2.34	27.86	6.83	6.45	3.25	9.19	3.59	3.48	3.22
	13-16,years	Female	326	33.9	18.0	80.0	43.7	4.51	2.79	4.05	2.41	30.37	5.87	7.73	3.22	10.03	3.39	4.49	3.20
		Male	157	37.3	19.3	90.7	51.8	4.52	2.77	4.29	2.62	27.84	5.75	7.67	3.23	10.24	3.55	4.43	2.99
	17+,years	Female	177	34.5	14.7	77.8	47.2	4.55	3.00	4.15	2.51	30.02	6.33	8.20	3.35	10.72	3.58	5.08	3.70
		Male	184	37.0	16.8	75.9	45.3	4.74	2.96	4.68	2.65	28.80	5.93	8.51	3.65	10.79	3.70	5.10	3.57
