# Supplementary material for: Reliability and validity of the NeuroCognitive Performance Test, a web-based neuropsychological assessment
Source: Front Psychol. 2015 Nov 3;6:1652. doi: 10.3389/fpsyg.2015.01652 (PMC4630791; doi:10.3389/fpsyg.2015.01652)
Supplement: Supplementary file 3 [file Table3.PDF]

Supplementary Table 3. Test-retest reliability by 5-year age bin. Test-retest reliability is reported as the Pearson correlation coefficient between baseline and follow-up assessment (mean 78.8 days between assessments). Test-retest correlations for conventional tests are provided in Supplementary Table 5 and for computerized tests in Gualtieri and Johnson, 2006. TA = Trail Making A, TB = Trail Making B, FMS = Forward Memory Span, RMS = Reverse Memory Span, DSC = Digit Symbol Coding, PM = Progressive Matrices, AR = Arithmetic Reasoning, GR = Grammatical Reasoning.

Age Bin	N	Grand Index	TA	TB	FMS	RMS	DSC	PM	AR	GR
13-19	914	0.8710	0.6010	0.6529	0.5392	0.4948	0.8042	0.4964	0.8153	0.5083
20-24	1,843	0.8517	0.6188	0.6067	0.5458	0.4983	0.7653	0.4855	0.7600	0.6069
25-29	2,688	0.8614	0.5899	0.6267	0.5814	0.5140	0.7666	0.4706	0.7744	0.6150
30-34	2,456	0.8693	0.6391	0.6337	0.5638	0.5364	0.7902	0.4879	0.7841	0.5885
35-39	1,985	0.8479	0.6129	0.6173	0.5607	0.5405	0.7780	0.4011	0.7751	0.5585
40-44	2,322	0.8484	0.6185	0.5321	0.5472	0.5264	0.7834	0.4135	0.7763	0.5739
45-49	2,864	0.8440	0.6143	0.5628	0.5466	0.5235	0.7557	0.4040	0.7653	0.5583
50-54	4,067	0.8326	0.5620	0.5504	0.5549	0.5141	0.7450	0.3740	0.7498	0.5431
55-59	4,537	0.8194	0.5734	0.5044	0.5119	0.5175	0.7274	0.3355	0.7242	0.5149
60-64	4,675	0.8024	0.5381	0.4673	0.4966	0.4891	0.6979	0.3370	0.7010	0.4888
65-69	4,013	0.8048	0.5218	0.4470	0.4961	0.4991	0.7172	0.3439	0.6957	0.4848
70-74	2,104	0.7920	0.4710	0.4083	0.4894	0.4896	0.6625	0.3152	0.6499	0.4793
75-79	898	0.7872	0.4972	0.3958	0.4547	0.4956	0.6942	0.2983	0.6347	0.4340
80-89	413	0.7800	0.5123	0.4069	0.5082	0.4258	0.6555	0.3290	0.6232	0.3535
