# Supplementary material for: Reliability and validity of the NeuroCognitive Performance Test, a web-based neuropsychological assessment
Source: Front Psychol. 2015 Nov 3;6:1652. doi: 10.3389/fpsyg.2015.01652 (PMC4630791; doi:10.3389/fpsyg.2015.01652)
Supplement: Supplementary file 4 [file Table4.PDF]

Supplementary Table 4. Test-retest reliability (r) by inter-trial interval. Inter-trial interval (ITI) is the number of days between assessments, and test-retest reliability (TRT) is the Pearson correlation coefficient between scores at baseline and follow-up. Most participants took their second assessment approximately 10 weeks later (median ITI = 78.8 days)

ITI (days)	N	Median TRT (Pearson's r)	Mean TRT (SD) (Pearson's r)	95% Confidence Interval
29-68	63	0.87	0.66 (0.550)	[0.50, 0.78]
69-88	30,873	0.83	0.83 (0.013)	[0.83, 0.84]
89-108	2,328	0.84	0.83 (0.038)	[0.81, 0.84]
109-225	2,515	0.85	0.79 (0.279)	[0.78, 0.81]
