# Supplementary material for: Reliability and validity of the NeuroCognitive Performance Test, a web-based neuropsychological assessment
Source: Front Psychol. 2015 Nov 3;6:1652. doi: 10.3389/fpsyg.2015.01652 (PMC4630791; doi:10.3389/fpsyg.2015.01652)
Supplement: Supplementary file 5 [file Table5.PDF]

Supplementary Table 5. Test-retest reliability references. Test-retest reliability (Pearson coefficients) for conventional tests.

**Conventional Test**	**r**	**References**
Trail Making	0.79 – 0.89	Dikmen et al. 1999
Memory Span/Corsi Blocks	0.74	Nuechterlein, K. H. et al. 2008
Digit Symbol Coding	0.84 - 0.85	Parrott 1991<br>Nuechterlein, K. H. et al. 2008
Grammatical Reasoning	0.80	Baddeley 1968
Arithmetic Reasoning		NA
Progressive Matrices	0.74 – 0.96	Wechsler 1955<br>Burke 2010

Burke, H. R. (2010). Raven's Progressive Matrices: Validity, Reliability, and Norms. *The Journal of Psychology* 82, 253–257. doi:10.1080/00223980.1972.9923815.

Dikmen, S. S., Heaton, R. K., GRANT, I., & Temkin, N. R. (1999). Test-retest reliability and practice effects of expanded Halstead-Reitan Neuropsychological Test Battery. Journal of the International Neuropsychological Society : JINS, 5(4), 346–356.

Nuechterlein, K. H. et al. (2008) The MATRICS Consensus Cognitive Battery, Part 1: Test selection, reliability, and validity. *Am J Psychiatry* 165:203-213.

Parrott, A. C. (1991). Performance tests in human psychopharmacology (1): test reliability and standardization. Human Psychopharmacology: Clinical and ….

Wechsler, D. (1955). Manual for the Wechsler Adult Intelligence Scale. Oxford, England: Psychological Corp. (1955). vi 110 pp.
