# Supplementary material for: Reliability and validity of the NeuroCognitive Performance Test, a web-based neuropsychological assessment
Source: Front Psychol. 2015 Nov 3;6:1652. doi: 10.3389/fpsyg.2015.01652 (PMC4630791; doi:10.3389/fpsyg.2015.01652)

Supplementary Figure 1: Inter-assessment Cluster Analysis Dendrogram. A Euclidian distance matrix was used to create a dendrogram of the NCPT subtests. Initially, each subtest was assigned to its own cluster and then the algorithm proceeded iteratively, at each stage joining the two most similar clusters, continuing until there was just a single cluster. Using this methodology, the most right position of the dendrogram nodes appears to support three groupings.

AR (Arithmetic Reasoning), DS (Digit Symbol Coding), GR (Grammatical Reasoning), FMS (Forward Memory Span), PM (Progressive Matrices), RMS (Reverse Memory Span), TA (Trail (Making A), TB (Trail Making B)

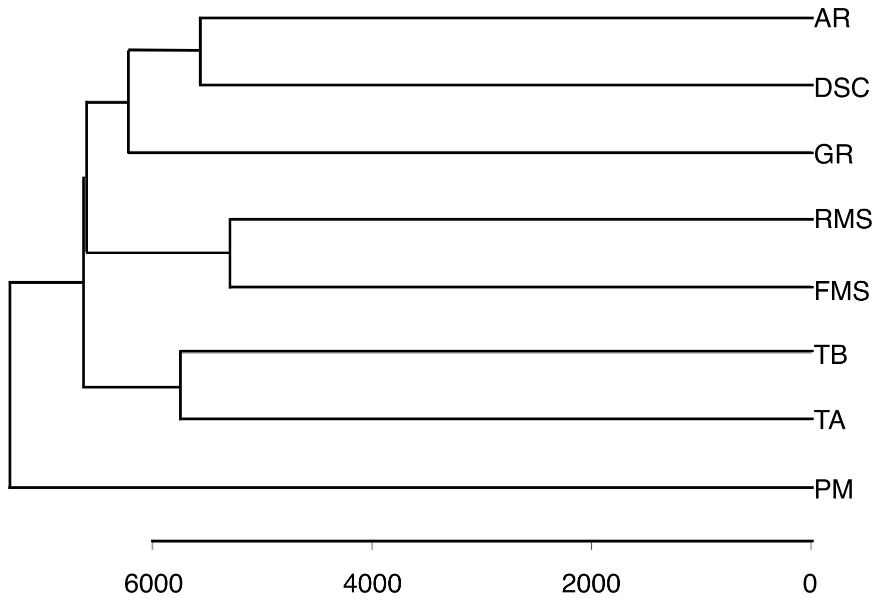

Supplement: Supplementary file 6 [file Image1.PDF]
