# Supplementary material for: Reliability and validity of the NeuroCognitive Performance Test, a web-based neuropsychological assessment
Source: Front Psychol. 2015 Nov 3;6:1652. doi: 10.3389/fpsyg.2015.01652 (PMC4630791; doi:10.3389/fpsyg.2015.01652)

Supplementary Figure 2: Interaction between age and education. Education was grouped by years that correspond to levels of education: High school or less (0 - 12 years); Bachelor's degree (13 - 16 years); Post-graduate (17+ years). The interaction of age and education revealed that the decline in NCPT Grand Index with age was less steep for those with ≤ 12 years of education. A similar pattern of confluence is seen for all other subtests, with the exception of Grammatical Reasoning and Progressive Matrices, which show a pattern of parallelism. The curves were smoothed with a General Additive Model (GAM).

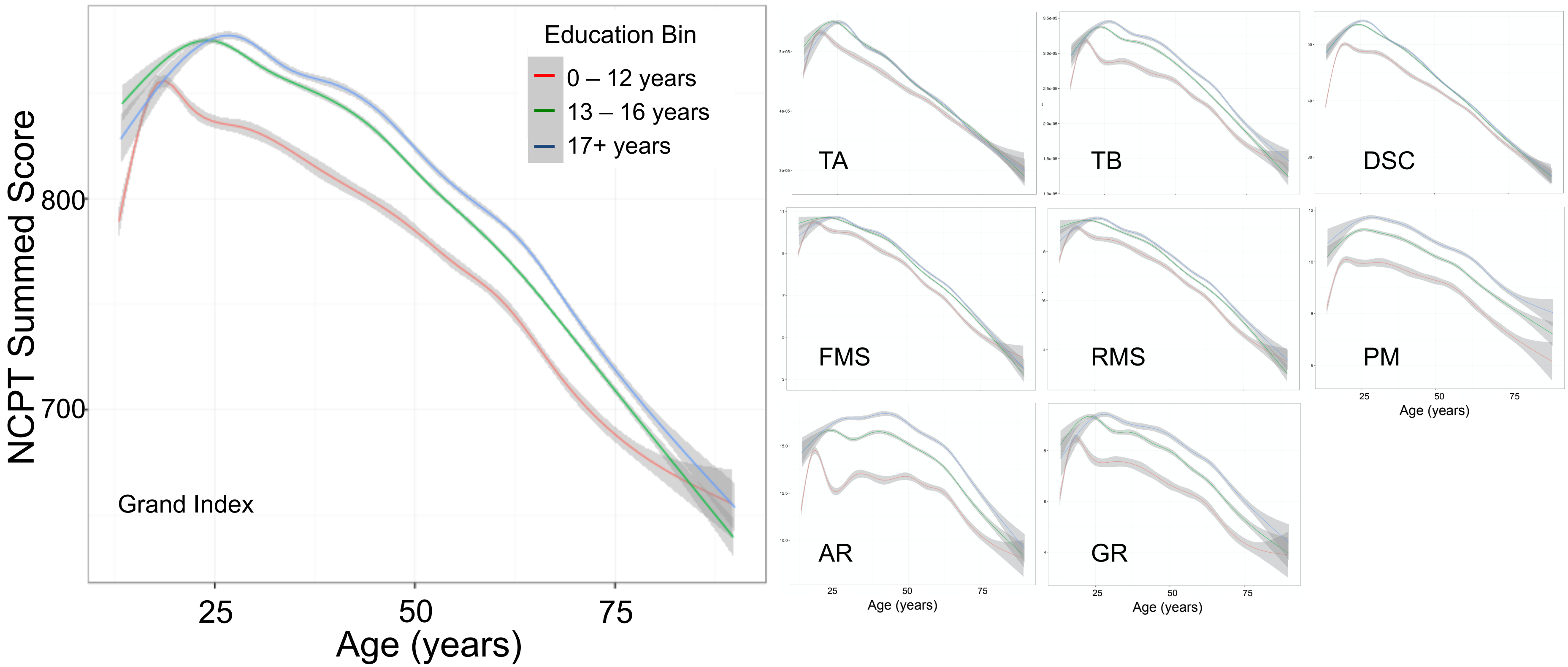

Supplement: Supplementary file 7 [file Image2.PDF]
