# Supplementary material for: Reliability and validity of the NeuroCognitive Performance Test, a web-based neuropsychological assessment
Source: Front Psychol. 2015 Nov 3;6:1652. doi: 10.3389/fpsyg.2015.01652 (PMC4630791; doi:10.3389/fpsyg.2015.01652)

## 1. NCPT Conceptual Framework

The subtests of the NCPT are based on or inspired by existing neuropsychological assessments. Each subtest has an interactive, clear tutorial prior to assessment administration to ensure a minimum level of proficiency with the task. Each assessment also has dynamically-generated content, which allows for increased number of trials and repeatability of the subtest. These assessments feature a white background with black text, and fill a window on the computer screen 640 pixels in width by 480 pixels in height. The NCPT subtests used in the study are described below.

### 1.1. Trail Making A and Trail Making B

The Trail Making Test (TMT) is one of the most popular neuropsychological tests and is included in most test batteries (Camara et al., 2000; Rabin et al., 2005). The TMT provides information on attention and speed of processing. Originally, it was part of the Army Individual Test Battery (Army Individual Test Battery, 1944) and subsequently was incorporated into the Halstead–Reitan Battery (Reitan and Wolfson, 1995).

In Trail Making A, blue circles numbered from 1 to 24 are arranged in 6 possible layouts with non-overlapping spatial locations. Layouts are normalized for completion time with total path length equal to 5 1/3 times the screen height, as well as equivalent circle-to-circle distance and angle distributions and circle density distributions. The circles have radii equal to 1/25 of the screen height and the number height is equal to 1/24 of the screen height. The participant is required to click on the circles sequentially connecting the encircled numbers. When clicked the blue circles change color to orange and a straight line appears connecting the circles. Task requirements are similar for Trail Making B except the circles now include numbers (1 to 12) and capital letters (A to L) and the participant must alternate between numbers and letters (e.g., 1, A, 2, B, 3, C, etc.). The timer begins when the participant clicks number 1. If the participant clicks on an incorrect circle, an X appears and they must go back to the previous circle. The amount of time required to complete the task is the dependent measure.

Trail Making A Trail Making B


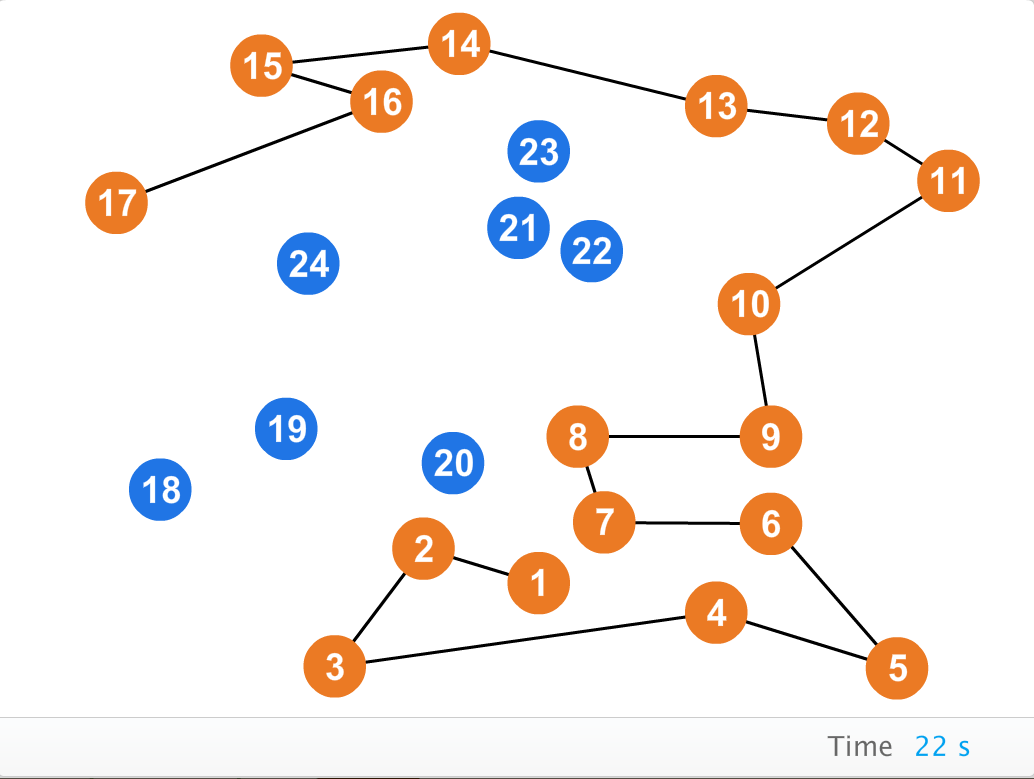

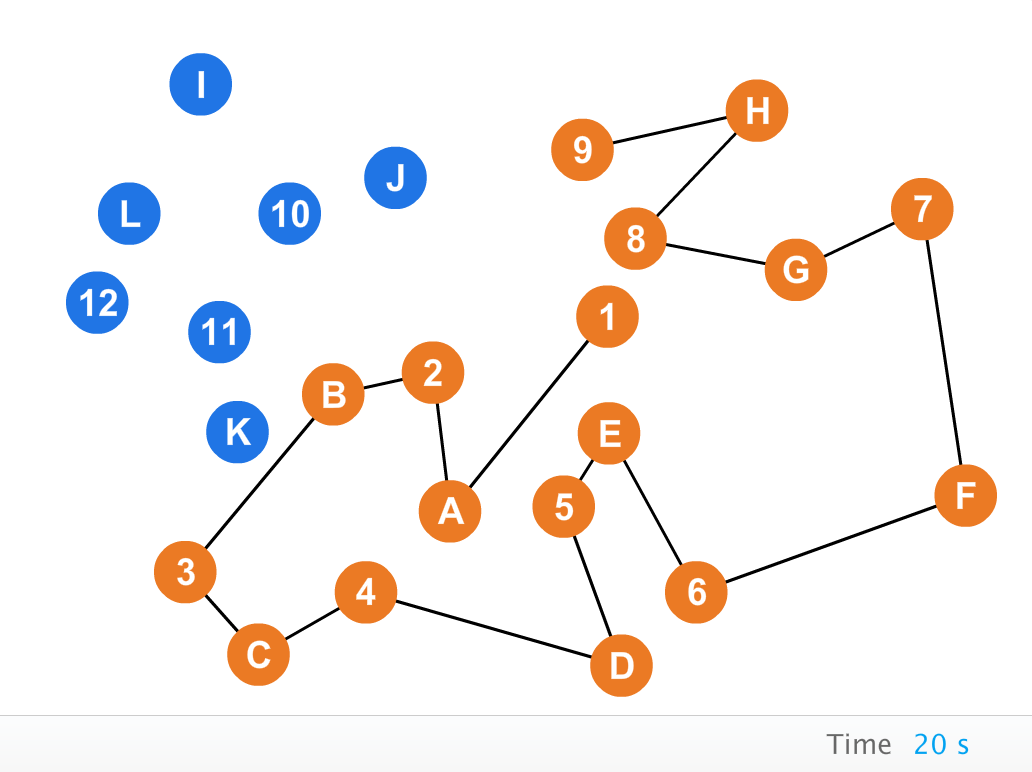


### 1.2. Visual Memory Span – Forward and Visual Memory Span – Reverse

Memory span is a common measure of visual [short-term memory](http://en.wikipedia.org/wiki/Short-term_memory) and functionally appears to measure the number of discrete units over which an individual can successively distribute attention and still organize into a working unit. To generalize, forward memory span refers to the ability of an individual to reproduce immediately, after one presentation, a series of discrete stimuli in their original order whereas reverse memory span requires reproducing the order in reverse.

The Visual Memory Span tasks are derived from the Corsi block-tapping test, which is a [psychological test](http://en.wikipedia.org/wiki/Psychological_testing) that assesses [visuospatial](http://en.wikipedia.org/wiki/Visual-spatial_ability) [short term working memory](http://en.wikipedia.org/wiki/Short_term_memory). The Corsi block-tapping task involves mimicking a researcher as he/she taps a sequence of up to nine identical spatially separated blocks. The backward Corsi block-tapping task is a slightly altered version of the original Corsi block-tapping task, and is considered a measure of working memory. In the backward task, the subjects are asked to watch the sequence and instead of mimicking the researcher's pattern, they are asked to repeat the sequence in reverse order. In computerized versions of the Visual Memory Span tasks, instead of cubes to be tapped on a board, the tests consist of circles that flash on a computer screen and participants reproduce the sequences by using a mouse to click on the circles (Schoenberg and Scott,, 2011; Pearson and Sahraie, 2003).

Blue circles with radii equal to 1/20 of the window height are placed at randomized, non-overlapping spatial locations and individually highlighted in orange following a particular sequence. Circles are highlighted for 500 msec with a 100 msec inter-stimulus interval. The participant is asked to recall the sequence by clicking on each circle in same order as originally presented. The length of the sequence increases by one every three trials. The session ends when the participant gives three incorrect answers at the same span level. The total number of correct responses is the dependent measure.

Forward Memory Span Reverse Memory Span


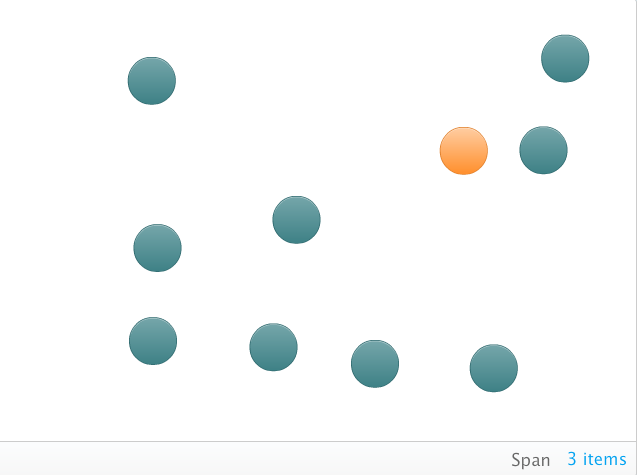

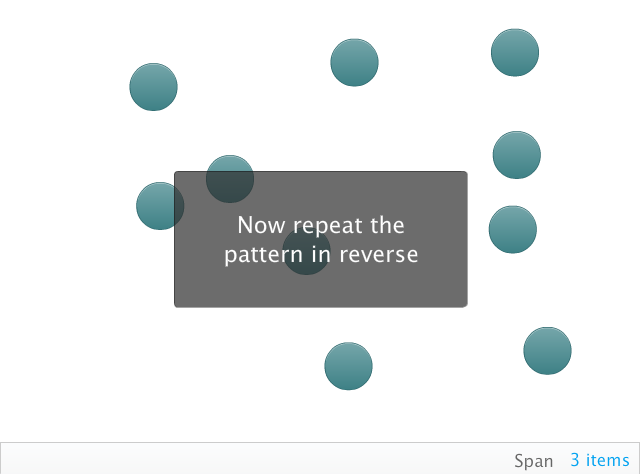


### 1.3. Arithmetic Reasoning

Arithmetic Reasoning is a cognitive task in which individuals solve basic arithmetic questions, written in words. Arithmetic Reasoning is similar to sections of assessments of mathematical processing that present problems in modalities other than Arabic numerals (Deloche et al., 1994).

This assessment requires the participant to rapidly and accurately solve simple arithmetic problems that are written in words – for example, “Four plus two =”. The answer is input using the number keys on the keyboard (“6” in the example above). For addition and multiplication problems, operands are uniformly sampled from the integers in the range 1-9, and for subtraction and division problems, the second operand and solution are uniformly sampled from the integers in the range 1-9. The addition operator is used in the first five trials. Subsequent trials use addition 50% of the time. In non-addition trials, operators are chosen uniformly from subtraction, multiplication, and division. The assessment lasts 90 seconds, and the total number of correct responses in that time period is the dependent measure. It is considered a measure of problem solving ability.

Arithmetic Reasoning


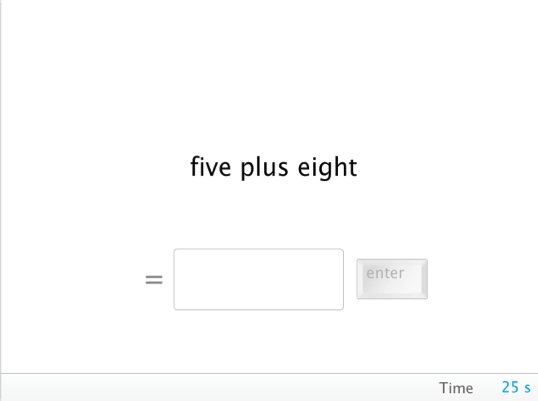


### 1.4. Grammatical Reasoning

The grammatical reasoning test is a test of the ability to carry out mental operations involving chains of logical reasoning. The test is derived from Baddeley’s Grammatical Reasoning Test (Baddeley, 1968) and measures the participant’s facility to rapidly and accurately evaluate a potentially confusing grammatical statement.

A blue square and a blue equilateral triangle, each with height equal to 1/5 of the window height are shown side by side, with a logical statement written below. For example, the square may be positioned to the left of the triangle on a particular trial. The participant could be prompted with a statement of the form, “The square is not to the left of the triangle.” In this case, the answer would be “false.” The participant responds whether the statement is true or false by pressing a key on the keyboard that is indicated as corresponding to true or false. The probability that the statement includes a negative (“not”) is 50%. The net number of correct responses (number correct – number incorrect) in 45 seconds is the dependent measure, with a floor of zero. This test is a measure of cognitive flexibility and logical reasoning.

Grammatical Reasoning


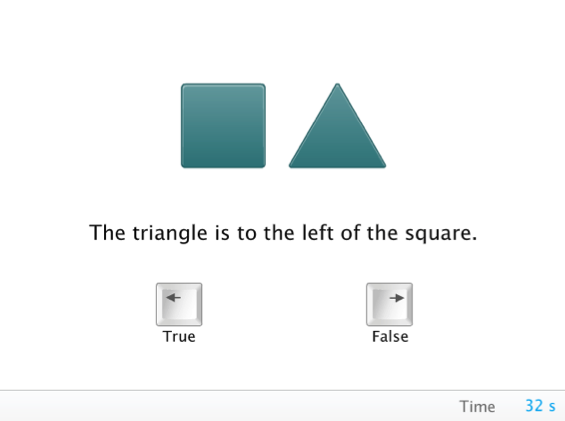


### 1.5. Progressive Matrices

The Progressive Matrices test was inspired by Raven’s Progressive Matrices (Penrose and Raven, 2011), with the exception that the NCPT version has dynamically generated problems. Matrix reasoning assessments require the participant to determine which stimulus most logically completes a multi-dimensional pattern. In this version of matrix reasoning, the participant is shown a 3x3 grid (each grid slot has width and height equal to 1/5 the window height) with abstract stimuli in the 8 upper-left slots. The task is to choose which of six possible answer choices best completes the pattern in the grid. The assessment is made up of 17 problems of increasing difficulty that are algorithmically generated from a set of parameters. The 17 problems are divided into three broad problem types: progression matrix, orbital/lateral movement, and Boolean logic. The first 12 trials involve progression matrix rules of increasing complexity. Characteristics of the stimuli that may change include shape, number, color, rotation angle, and size. These patterns may progress across the matrix horizontally, vertically, from upper-left to lower-right diagonal, or from lower-left to upper-right diagonal. Trials 13-15 involve orbital or lateral movement in which square grids or circular orbits are partially filled with elements that progress according to a lateral or rotational movement rule. Trials 16-17 involve Boolean logic in which spatial patterns are combined using Boolean operators such as AND, OR, and XOR. For each problem type, the correct answer is indicated regardless of whether the participant answers correctly. The assessment ends once the participant completes 17 trials or answers three consecutive trials incorrectly. The total number correct is the dependent measure. Matrix reasoning is considered a measure of problem solving and fluid reasoning (often referred to as fluid intelligence).

Progressive Matrices


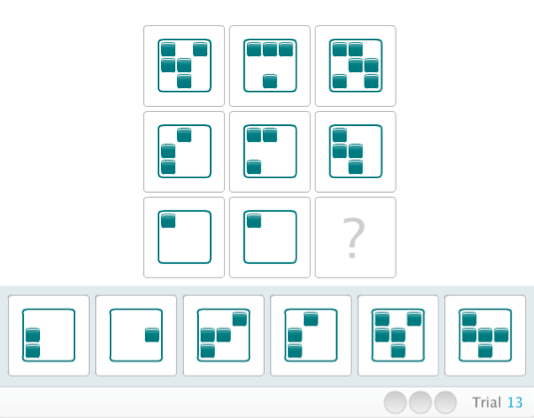


### 1.6. Digit Symbol Coding

Digit Symbol Coding is based on the Digit Symbol Substitution Task in the Brief Assessment of Cognition in Schizophrenia (BACS) and is used to measure information processing speed, the principal components of which are visual search and memory (Royer, 1971).

A legend with numbers 1 to 9 and corresponding abstract symbols is presented across the top of the screen. The height of the numbers is equal to 1/24 the height of the screen, and symbols fill a space with height and width equal to 1/12 the height of the screen. The symbols are randomly selected from a pool of 18 possible symbols. In the middle of the screen a large, two-section box (top and bottom sections each having a height equal to ¼ of the screen height) is presented with the symbol in the top section. The participant is required to use the number keyboard to type the number that corresponds to the symbol. Once a number is pressed, it moves on to the next trial. Participants can see the next symbol grayed out, but they cannot go back to a previous trial. The assessment is timed for 90 seconds and the dependent measure is the total number of correct trials minus incorrect trials.

Digit Symbol Coding


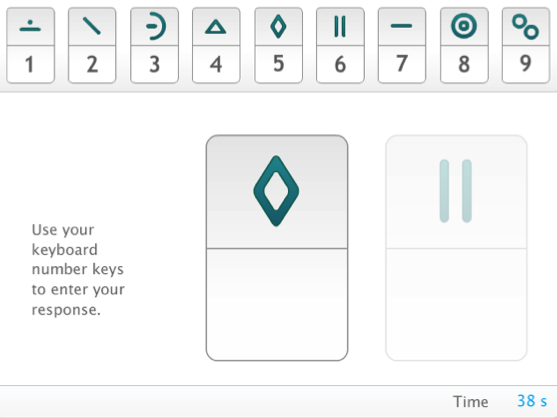

Supplement: Supplementary file 9 [file DataSheet2.DOCX]
