# Supplementary material for: Reliability and validity of the NeuroCognitive Performance Test, a web-based neuropsychological assessment
Source: Front Psychol. 2015 Nov 3;6:1652. doi: 10.3389/fpsyg.2015.01652 (PMC4630791; doi:10.3389/fpsyg.2015.01652)
Supplement: Supplementary file 8 [file DataSheet1.PDF]

Age	Gender	Education	Percentile	Raw Score	Norm Score	Assessment
13	f	0-12 years	0.001	553.7151359	54	Grand Index
13	f	0-12 years	0.002	573.0291849	57	Grand Index
13	f	0-12 years	0.003	587.369463	59	Grand Index
13	f	0-12 years	0.004	595.8553794	60	Grand Index
13	f	0-12 years	0.005	601.657875	61	Grand Index
13	f	0-12 years	0.006	605.4277272	62	Grand Index
13	f	0-12 years	0.007	608.5253878	63	Grand Index
13	f	0-12 years	0.008	609.5043627	64	Grand Index
13	f	0-12 years	0.009	610.7323775	65	Grand Index
13	f	0-12 years	0.01	619.27	65	Grand Index
13	f	0-12 years	0.011	622.399	66	Grand Index
13	f	0-12 years	0.012	623.2681792	66	Grand Index
13	f	0-12 years	0.013	628.0519181	67	Grand Index
13	f	0-12 years	0.014	632.0143985	67	Grand Index
13	f	0-12 years	0.015	634.3364639	67	Grand Index
13	f	0-12 years	0.016	637.9965938	68	Grand Index
13	f	0-12 years	0.017	646.0578717	68	Grand Index
13	f	0-12 years	0.018	649.6286006	69	Grand Index
13	f	0-12 years	0.019	650.871	69	Grand Index
13	f	0-12 years	0.02	653.1173643	69	Grand Index
13	f	0-12 years	0.021	653.8090228	69	Grand Index
13	f	0-12 years	0.022	657.9845631	70	Grand Index
13	f	0-12 years	0.023	659.107	70	Grand Index
13	f	0-12 years	0.024	660	70	Grand Index
13	f	0-12 years	0.025	660.725	71	Grand Index
13	f	0-12 years	0.026	662.068	71	Grand Index
13	f	0-12 years	0.027	664.0264985	71	Grand Index
13	f	0-12 years	0.028	665.3308847	71	Grand Index
13	f	0-12 years	0.029	666.961	72	Grand Index
13	f	0-12 years	0.03	667	72	Grand Index
13	f	0-12 years	0.031	667.579	72	Grand Index
13	f	0-12 years	0.032	668.888	72	Grand Index
13	f	0-12 years	0.033	670.197	72	Grand Index
13	f	0-12 years	0.034	671	73	Grand Index
13	f	0-12 years	0.035	671.815	73	Grand Index
13	f	0-12 years	0.036	675	73	Grand Index
13	f	0-12 years	0.037	675.1355437	73	Grand Index
13	f	0-12 years	0.038	675.8227628	73	Grand Index
13	f	0-12 years	0.039	676.8820756	74	Grand Index
13	f	0-12 years	0.04	677.36	74	Grand Index
13	f	0-12 years	0.041	678	74	Grand Index
13	f	0-12 years	0.042	678.978	74	Grand Index
13	f	0-12 years	0.043	680.5279327	74	Grand Index
13	f	0-12 years	0.044	681.596	74	Grand Index
13	f	0-12 years	0.045	683.81	75	Grand Index
13	f	0-12 years	0.046	684.214	75	Grand Index
13	f	0-12 years	0.048	685	75	Grand Index
13	f	0-12 years	0.049	685.0333107	75	Grand Index
13	f	0-12 years	0.05	685.5799353	75	Grand Index
13	f	0-12 years	0.051	686	75	Grand Index
13	f	0-12 years	0.052	686.067512	76	Grand Index
13	f	0-12 years	0.053	686.9955286	76	Grand Index
13	f	0-12 years	0.054	687	76	Grand Index
13	f	0-12 years	0.055	687.3395195	76	Grand Index
13	f	0-12 years	0.056	690.6605578	76	Grand Index
13	f	0-12 years	0.057	693.019038	76	Grand Index

Age	Gender	Education	Percentile	Raw Score	Norm Score	Assessment
13	f	0-12 years	0.058	693.9244225	76	Grand Index
13	f	0-12 years	0.059	695	77	Grand Index
13	f	0-12 years	0.06	695.54	77	Grand Index
13	f	0-12 years	0.061	696.849	77	Grand Index
13	f	0-12 years	0.062	697.0244583	77	Grand Index
13	f	0-12 years	0.063	697.5495082	77	Grand Index
13	f	0-12 years	0.064	698.0211471	77	Grand Index
13	f	0-12 years	0.065	698.3101151	77	Grand Index
13	f	0-12 years	0.066	699.0593091	77	Grand Index
13	f	0-12 years	0.067	699.7438023	78	Grand Index
13	f	0-12 years	0.07	700	78	Grand Index
13	f	0-12 years	0.071	700.7376527	78	Grand Index
13	f	0-12 years	0.072	701	78	Grand Index
13	f	0-12 years	0.073	701.0885706	78	Grand Index
13	f	0-12 years	0.074	701.4530742	78	Grand Index
13	f	0-12 years	0.075	703	78	Grand Index
13	f	0-12 years	0.076	704.452	79	Grand Index
13	f	0-12 years	0.077	706.187343	79	Grand Index
13	f	0-12 years	0.078	707	79	Grand Index
13	f	0-12 years	0.079	707.411	79	Grand Index
13	f	0-12 years	0.081	708	79	Grand Index
13	f	0-12 years	0.082	708.338	79	Grand Index
13	f	0-12 years	0.084	709	79	Grand Index
13	f	0-12 years	0.085	709.5234561	79	Grand Index
13	f	0-12 years	0.086	710	80	Grand Index
13	f	0-12 years	0.087	710.883	80	Grand Index
13	f	0-12 years	0.088	711.0258214	80	Grand Index
13	f	0-12 years	0.089	711.1459298	80	Grand Index
13	f	0-12 years	0.09	711.7524822	80	Grand Index
13	f	0-12 years	0.091	712	80	Grand Index
13	f	0-12 years	0.092	712.4140522	80	Grand Index
13	f	0-12 years	0.093	712.9914293	80	Grand Index
13	f	0-12 years	0.094	713.046	80	Grand Index
13	f	0-12 years	0.096	714	80	Grand Index
13	f	0-12 years	0.097	714.973	81	Grand Index
13	f	0-12 years	0.098	715	81	Grand Index
13	f	0-12 years	0.099	715.591	81	Grand Index
13	f	0-12 years	0.1	716	81	Grand Index
13	f	0-12 years	0.101	716.004727	81	Grand Index
13	f	0-12 years	0.102	716.4036412	81	Grand Index
13	f	0-12 years	0.103	716.9358056	81	Grand Index
13	f	0-12 years	0.104	717.0059861	81	Grand Index
13	f	0-12 years	0.105	717.4036308	81	Grand Index
13	f	0-12 years	0.106	717.9636265	81	Grand Index
13	f	0-12 years	0.107	718	81	Grand Index
13	f	0-12 years	0.108	718.3619722	81	Grand Index
13	f	0-12 years	0.109	719.6724009	82	Grand Index
13	f	0-12 years	0.11	720	82	Grand Index
13	f	0-12 years	0.111	720.299	82	Grand Index
13	f	0-12 years	0.112	721	82	Grand Index
13	f	0-12 years	0.113	721.917	82	Grand Index
13	f	0-12 years	0.114	722	82	Grand Index
13	f	0-12 years	0.115	722.0024052	82	Grand Index
13	f	0-12 years	0.116	722.0848633	82	Grand Index
13	f	0-12 years	0.117	722.6777174	82	Grand Index
13	f	0-12 years	0.118	723	82	Grand Index

Age	Gender	Education	Percentile	Raw Score	Norm Score	Assessment
13	f	0-12 years	0.119	723.771	82	Grand Index
13	f	0-12 years	0.12	725.0751434	82	Grand Index
13	f	0-12 years	0.121	726.1518685	82	Grand Index
13	f	0-12 years	0.122	726.8159031	83	Grand Index
13	f	0-12 years	0.123	727	83	Grand Index
13	f	0-12 years	0.124	727.316	83	Grand Index
13	f	0-12 years	0.127	728	83	Grand Index
13	f	0-12 years	0.128	728.552	83	Grand Index
13	f	0-12 years	0.132	729	83	Grand Index
13	f	0-12 years	0.133	729.097	83	Grand Index
13	f	0-12 years	0.136	730	84	Grand Index
13	f	0-12 years	0.137	730.333	84	Grand Index
13	f	0-12 years	0.138	731	84	Grand Index
13	f	0-12 years	0.139	731.951	84	Grand Index
13	f	0-12 years	0.14	732	84	Grand Index
13	f	0-12 years	0.141	732.0069298	84	Grand Index
13	f	0-12 years	0.142	733.7574858	84	Grand Index
13	f	0-12 years	0.144	734	84	Grand Index
13	f	0-12 years	0.145	734.805	84	Grand Index
13	f	0-12 years	0.147	735	84	Grand Index
13	f	0-12 years	0.148	735.732	84	Grand Index
13	f	0-12 years	0.149	736	84	Grand Index
13	f	0-12 years	0.15	736.0113499	84	Grand Index
13	f	0-12 years	0.151	736.1186561	85	Grand Index
13	f	0-12 years	0.152	736.9600914	85	Grand Index
13	f	0-12 years	0.154	737	85	Grand Index
13	f	0-12 years	0.155	737.895	85	Grand Index
13	f	0-12 years	0.156	738	85	Grand Index
13	f	0-12 years	0.157	738.1553959	85	Grand Index
13	f	0-12 years	0.158	739.697919	85	Grand Index
13	f	0-12 years	0.159	740.0429379	85	Grand Index
13	f	0-12 years	0.16	740.6235511	85	Grand Index
13	f	0-12 years	0.161	741	85	Grand Index
13	f	0-12 years	0.162	741.004816	85	Grand Index
13	f	0-12 years	0.163	741.4195612	85	Grand Index
13	f	0-12 years	0.165	742	85	Grand Index
13	f	0-12 years	0.166	742.0756629	85	Grand Index
13	f	0-12 years	0.167	742.6687263	86	Grand Index
13	f	0-12 years	0.168	742.9946814	86	Grand Index
13	f	0-12 years	0.171	743	86	Grand Index
13	f	0-12 years	0.172	743.148	86	Grand Index
13	f	0-12 years	0.177	744	86	Grand Index
13	f	0-12 years	0.178	744.3698516	86	Grand Index
13	f	0-12 years	0.181	745	86	Grand Index
13	f	0-12 years	0.182	745.238	86	Grand Index
13	f	0-12 years	0.185	746	87	Grand Index
13	f	0-12 years	0.186	746.474	87	Grand Index
13	f	0-12 years	0.187	747.783	87	Grand Index
13	f	0-12 years	0.192	748	87	Grand Index
13	f	0-12 years	0.193	748.1504887	87	Grand Index
13	f	0-12 years	0.194	748.2971769	87	Grand Index
13	f	0-12 years	0.197	749	87	Grand Index
13	f	0-12 years	0.2	750	87	Grand Index
13	f	0-12 years	0.201	750.0233576	87	Grand Index
13	f	0-12 years	0.202	750.4773079	87	Grand Index
13	f	0-12 years	0.203	750.9572808	88	Grand Index

Age	Gender	Education	Percentile	Raw Score	Norm Score	Assessment
13	f	0-12 years	0.204	752	88	Grand Index
13	f	0-12 years	0.205	752.345	88	Grand Index
13	f	0-12 years	0.207	753	88	Grand Index
13	f	0-12 years	0.208	753.4457187	88	Grand Index
13	f	0-12 years	0.209	753.9883987	88	Grand Index
13	f	0-12 years	0.213	754	88	Grand Index
13	f	0-12 years	0.216	755	88	Grand Index
13	f	0-12 years	0.217	755.3617513	88	Grand Index
13	f	0-12 years	0.22	756	88	Grand Index
13	f	0-12 years	0.223	757	89	Grand Index
13	f	0-12 years	0.224	757.6419984	89	Grand Index
13	f	0-12 years	0.225	757.9965868	89	Grand Index
13	f	0-12 years	0.226	758	89	Grand Index
13	f	0-12 years	0.227	758.143	89	Grand Index
13	f	0-12 years	0.233	759	89	Grand Index
13	f	0-12 years	0.236	760	89	Grand Index
13	f	0-12 years	0.237	760.1362608	89	Grand Index
13	f	0-12 years	0.238	760.7289533	89	Grand Index
13	f	0-12 years	0.239	760.8552629	89	Grand Index
13	f	0-12 years	0.242	761	90	Grand Index
13	f	0-12 years	0.243	761.1545688	90	Grand Index
13	f	0-12 years	0.244	762.2165078	90	Grand Index
13	f	0-12 years	0.245	762.9635737	90	Grand Index
13	f	0-12 years	0.247	763	90	Grand Index
13	f	0-12 years	0.248	763.3197129	90	Grand Index
13	f	0-12 years	0.249	763.8488651	90	Grand Index
13	f	0-12 years	0.252	764	90	Grand Index
13	f	0-12 years	0.253	764.4860035	90	Grand Index
13	f	0-12 years	0.254	765.486	90	Grand Index
13	f	0-12 years	0.255	766	90	Grand Index
13	f	0-12 years	0.256	766.104	90	Grand Index
13	f	0-12 years	0.257	767.413	90	Grand Index
13	f	0-12 years	0.261	768	90	Grand Index
13	f	0-12 years	0.262	768.958	90	Grand Index
13	f	0-12 years	0.264	769	91	Grand Index
13	f	0-12 years	0.265	769.885	91	Grand Index
13	f	0-12 years	0.266	770.0844012	91	Grand Index
13	f	0-12 years	0.267	770.7192238	91	Grand Index
13	f	0-12 years	0.268	771.812	91	Grand Index
13	f	0-12 years	0.27	772	91	Grand Index
13	f	0-12 years	0.271	772.739	91	Grand Index
13	f	0-12 years	0.273	773	91	Grand Index
13	f	0-12 years	0.274	773.666	91	Grand Index
13	f	0-12 years	0.278	774	91	Grand Index
13	f	0-12 years	0.279	774.0861957	91	Grand Index
13	f	0-12 years	0.28	774.7160851	91	Grand Index
13	f	0-12 years	0.284	775	91	Grand Index
13	f	0-12 years	0.285	775.065	91	Grand Index
13	f	0-12 years	0.292	776	92	Grand Index
13	f	0-12 years	0.293	776.537	92	Grand Index
13	f	0-12 years	0.295	777	92	Grand Index
13	f	0-12 years	0.296	777.4079505	92	Grand Index
13	f	0-12 years	0.297	777.9725792	92	Grand Index
13	f	0-12 years	0.298	778	92	Grand Index
13	f	0-12 years	0.299	778.0163205	92	Grand Index
13	f	0-12 years	0.3	778.4255512	92	Grand Index

Age	Gender	Education	Percentile	Raw Score	Norm Score	Assessment
13	f	0-12 years	0.307	779	92	Grand Index
13	f	0-12 years	0.308	779.8457402	92	Grand Index
13	f	0-12 years	0.313	780	93	Grand Index
13	f	0-12 years	0.314	780.4407541	93	Grand Index
13	f	0-12 years	0.317	781	93	Grand Index
13	f	0-12 years	0.318	781.262	93	Grand Index
13	f	0-12 years	0.322	782	93	Grand Index
13	f	0-12 years	0.323	782.7413741	93	Grand Index
13	f	0-12 years	0.33	783	93	Grand Index
13	f	0-12 years	0.331	783.2476162	93	Grand Index
13	f	0-12 years	0.332	783.9536554	93	Grand Index
13	f	0-12 years	0.337	784	94	Grand Index
13	f	0-12 years	0.338	784.442	94	Grand Index
13	f	0-12 years	0.341	785	94	Grand Index
13	f	0-12 years	0.342	785.678	94	Grand Index
13	f	0-12 years	0.347	786	94	Grand Index
13	f	0-12 years	0.348	786.532	94	Grand Index
13	f	0-12 years	0.357	787	95	Grand Index
13	f	0-12 years	0.358	787.2045306	95	Grand Index
13	f	0-12 years	0.359	787.9536891	95	Grand Index
13	f	0-12 years	0.363	788	95	Grand Index
13	f	0-12 years	0.364	788.476	95	Grand Index
13	f	0-12 years	0.368	789	95	Grand Index
13	f	0-12 years	0.374	790	95	Grand Index
13	f	0-12 years	0.375	790.875	95	Grand Index
13	f	0-12 years	0.378	791	95	Grand Index
13	f	0-12 years	0.379	791.6469635	95	Grand Index
13	f	0-12 years	0.388	792	96	Grand Index
13	f	0-12 years	0.393	793	96	Grand Index
13	f	0-12 years	0.394	793.746	96	Grand Index
13	f	0-12 years	0.399	794	96	Grand Index
13	f	0-12 years	0.4	794.1471078	96	Grand Index
13	f	0-12 years	0.401	794.9313113	96	Grand Index
13	f	0-12 years	0.407	795	96	Grand Index
13	f	0-12 years	0.408	795.2386795	97	Grand Index
13	f	0-12 years	0.411	796	97	Grand Index
13	f	0-12 years	0.412	796.0689119	97	Grand Index
13	f	0-12 years	0.413	796.6597681	97	Grand Index
13	f	0-12 years	0.42	797	97	Grand Index
13	f	0-12 years	0.421	797.8041722	97	Grand Index
13	f	0-12 years	0.422	797.9271572	97	Grand Index
13	f	0-12 years	0.426	798	97	Grand Index
13	f	0-12 years	0.427	798.943	97	Grand Index
13	f	0-12 years	0.428	799.0295816	97	Grand Index
13	f	0-12 years	0.429	799.279781	97	Grand Index
13	f	0-12 years	0.43	799.9228917	97	Grand Index
13	f	0-12 years	0.436	800	98	Grand Index
13	f	0-12 years	0.437	800.033	98	Grand Index
13	f	0-12 years	0.441	801	98	Grand Index
13	f	0-12 years	0.442	801.3799122	98	Grand Index
13	f	0-12 years	0.443	801.9612735	98	Grand Index
13	f	0-12 years	0.445	802	98	Grand Index
13	f	0-12 years	0.446	802.814	98	Grand Index
13	f	0-12 years	0.449	803	98	Grand Index
13	f	0-12 years	0.45	803.0193938	98	Grand Index
13	f	0-12 years	0.451	803.4195886	98	Grand Index

Age	Gender	Education	Percentile	Raw Score	Norm Score	Assessment
13	f	0-12 years	0.452	803.8261028	98	Grand Index
13	f	0-12 years	0.455	804	98	Grand Index
13	f	0-12 years	0.456	804.7893615	98	Grand Index
13	f	0-12 years	0.457	804.9802641	98	Grand Index
13	f	0-12 years	0.461	805	99	Grand Index
13	f	0-12 years	0.462	805.1006432	99	Grand Index
13	f	0-12 years	0.463	805.70646	99	Grand Index
13	f	0-12 years	0.464	806	99	Grand Index
13	f	0-12 years	0.465	806.685	99	Grand Index
13	f	0-12 years	0.475	807	99	Grand Index
13	f	0-12 years	0.476	807.084	99	Grand Index
13	f	0-12 years	0.482	808	99	Grand Index
13	f	0-12 years	0.483	808.0102626	99	Grand Index
13	f	0-12 years	0.484	808.5744478	99	Grand Index
13	f	0-12 years	0.487	809	100	Grand Index
13	f	0-12 years	0.488	809.0388115	100	Grand Index
13	f	0-12 years	0.494	810	100	Grand Index
13	f	0-12 years	0.495	810.0370197	100	Grand Index
13	f	0-12 years	0.496	810.8402672	100	Grand Index
13	f	0-12 years	0.499	811	100	Grand Index
13	f	0-12 years	0.5	811.5	100	Grand Index
13	f	0-12 years	0.508	812	100	Grand Index
13	f	0-12 years	0.509	812.2572936	100	Grand Index
13	f	0-12 years	0.51	812.9654106	100	Grand Index
13	f	0-12 years	0.515	813	101	Grand Index
13	f	0-12 years	0.516	813.3805714	101	Grand Index
13	f	0-12 years	0.517	813.9647143	101	Grand Index
13	f	0-12 years	0.518	814.062	101	Grand Index
13	f	0-12 years	0.519	815.371	101	Grand Index
13	f	0-12 years	0.522	816	101	Grand Index
13	f	0-12 years	0.523	816.607	101	Grand Index
13	f	0-12 years	0.526	817	101	Grand Index
13	f	0-12 years	0.527	817.3278085	101	Grand Index
13	f	0-12 years	0.53	818	101	Grand Index
13	f	0-12 years	0.536	819	101	Grand Index
13	f	0-12 years	0.537	819.1791108	101	Grand Index
13	f	0-12 years	0.541	820	102	Grand Index
13	f	0-12 years	0.542	820.478	102	Grand Index
13	f	0-12 years	0.553	821	102	Grand Index
13	f	0-12 years	0.554	821.9314719	102	Grand Index
13	f	0-12 years	0.559	822	102	Grand Index
13	f	0-12 years	0.56	822.04	102	Grand Index
13	f	0-12 years	0.565	823	102	Grand Index
13	f	0-12 years	0.566	823.0168123	102	Grand Index
13	f	0-12 years	0.567	823.4417433	103	Grand Index
13	f	0-12 years	0.568	823.8885959	103	Grand Index
13	f	0-12 years	0.57	824	103	Grand Index
13	f	0-12 years	0.571	824.439	103	Grand Index
13	f	0-12 years	0.576	825	103	Grand Index
13	f	0-12 years	0.577	825.2638708	103	Grand Index
13	f	0-12 years	0.578	825.9604321	103	Grand Index
13	f	0-12 years	0.581	826	103	Grand Index
13	f	0-12 years	0.582	826.838	103	Grand Index
13	f	0-12 years	0.585	827	103	Grand Index
13	f	0-12 years	0.59	828	103	Grand Index
13	f	0-12 years	0.591	828.4247531	103	Grand Index

Age	Gender	Education	Percentile	Raw Score	Norm Score	Assessment
13	f	0-12 years	0.592	828.9774059	103	Grand Index
13	f	0-12 years	0.597	829	104	Grand Index
13	f	0-12 years	0.598	829.0495089	104	Grand Index
13	f	0-12 years	0.599	829.8393356	104	Grand Index
13	f	0-12 years	0.604	830	104	Grand Index
13	f	0-12 years	0.605	830.4925421	104	Grand Index
13	f	0-12 years	0.606	830.6939476	104	Grand Index
13	f	0-12 years	0.61	831	104	Grand Index
13	f	0-12 years	0.611	831.799	104	Grand Index
13	f	0-12 years	0.614	832	104	Grand Index
13	f	0-12 years	0.618	833	105	Grand Index
13	f	0-12 years	0.619	833.8374766	105	Grand Index
13	f	0-12 years	0.623	834	105	Grand Index
13	f	0-12 years	0.624	834.816	105	Grand Index
13	f	0-12 years	0.626	835	105	Grand Index
13	f	0-12 years	0.627	835.743	105	Grand Index
13	f	0-12 years	0.629	836	105	Grand Index
13	f	0-12 years	0.63	836.0332231	105	Grand Index
13	f	0-12 years	0.631	836.9800413	105	Grand Index
13	f	0-12 years	0.633	837	105	Grand Index
13	f	0-12 years	0.634	837.7601983	105	Grand Index
13	f	0-12 years	0.637	838	105	Grand Index
13	f	0-12 years	0.638	838.2285801	105	Grand Index
13	f	0-12 years	0.648	839	106	Grand Index
13	f	0-12 years	0.649	839.541	106	Grand Index
13	f	0-12 years	0.655	840	106	Grand Index
13	f	0-12 years	0.656	840.704	106	Grand Index
13	f	0-12 years	0.66	841	106	Grand Index
13	f	0-12 years	0.661	841.888152	106	Grand Index
13	f	0-12 years	0.662	841.9784606	106	Grand Index
13	f	0-12 years	0.666	842	106	Grand Index
13	f	0-12 years	0.667	842.0399634	106	Grand Index
13	f	0-12 years	0.668	842.580586	107	Grand Index
13	f	0-12 years	0.669	842.9596708	107	Grand Index
13	f	0-12 years	0.67	843	107	Grand Index
13	f	0-12 years	0.671	843.2696349	107	Grand Index
13	f	0-12 years	0.672	843.9279749	107	Grand Index
13	f	0-12 years	0.676	844	107	Grand Index
13	f	0-12 years	0.679	845	107	Grand Index
13	f	0-12 years	0.68	846.12	107	Grand Index
13	f	0-12 years	0.684	847	107	Grand Index
13	f	0-12 years	0.685	847.6552545	107	Grand Index
13	f	0-12 years	0.686	847.999619	107	Grand Index
13	f	0-12 years	0.687	848	107	Grand Index
13	f	0-12 years	0.688	848.592	107	Grand Index
13	f	0-12 years	0.692	849	108	Grand Index
13	f	0-12 years	0.693	849.3245078	108	Grand Index
13	f	0-12 years	0.699	850	108	Grand Index
13	f	0-12 years	0.7	850.3	108	Grand Index
13	f	0-12 years	0.705	851	108	Grand Index
13	f	0-12 years	0.706	851.2535294	108	Grand Index
13	f	0-12 years	0.708	852	108	Grand Index
13	f	0-12 years	0.712	853	108	Grand Index
13	f	0-12 years	0.713	853.317	108	Grand Index
13	f	0-12 years	0.714	854	108	Grand Index
13	f	0-12 years	0.715	854.935	109	Grand Index

Age	Gender	Education	Percentile	Raw Score	Norm Score	Assessment
13	f	0-12 years	0.721	855	109	Grand Index
13	f	0-12 years	0.722	855.0908601	109	Grand Index
13	f	0-12 years	0.723	855.9567964	109	Grand Index
13	f	0-12 years	0.726	856	109	Grand Index
13	f	0-12 years	0.727	856.643	109	Grand Index
13	f	0-12 years	0.732	857	109	Grand Index
13	f	0-12 years	0.733	857.497	109	Grand Index
13	f	0-12 years	0.734	858	109	Grand Index
13	f	0-12 years	0.735	858.115	109	Grand Index
13	f	0-12 years	0.737	859	110	Grand Index
13	f	0-12 years	0.739	860	110	Grand Index
13	f	0-12 years	0.74	860.0561895	110	Grand Index
13	f	0-12 years	0.741	860.330963	110	Grand Index
13	f	0-12 years	0.742	861.278	110	Grand Index
13	f	0-12 years	0.748	862	110	Grand Index
13	f	0-12 years	0.749	862.441	110	Grand Index
13	f	0-12 years	0.75	863	110	Grand Index
13	f	0-12 years	0.751	863.059	110	Grand Index
13	f	0-12 years	0.755	864	110	Grand Index
13	f	0-12 years	0.756	864.604	110	Grand Index
13	f	0-12 years	0.757	865	110	Grand Index
13	f	0-12 years	0.758	865.222	110	Grand Index
13	f	0-12 years	0.76	866	111	Grand Index
13	f	0-12 years	0.761	866.1157039	111	Grand Index
13	f	0-12 years	0.762	866.8788827	111	Grand Index
13	f	0-12 years	0.768	867	111	Grand Index
13	f	0-12 years	0.769	867.621	111	Grand Index
13	f	0-12 years	0.771	868	111	Grand Index
13	f	0-12 years	0.772	868.3457377	111	Grand Index
13	f	0-12 years	0.773	868.9472199	111	Grand Index
13	f	0-12 years	0.779	869	112	Grand Index
13	f	0-12 years	0.783	870	112	Grand Index
13	f	0-12 years	0.784	870.0813975	112	Grand Index
13	f	0-12 years	0.785	870.4003289	112	Grand Index
13	f	0-12 years	0.786	870.9324321	112	Grand Index
13	f	0-12 years	0.787	871.1718764	112	Grand Index
13	f	0-12 years	0.788	871.9691213	112	Grand Index
13	f	0-12 years	0.792	872	112	Grand Index
13	f	0-12 years	0.793	872.037	112	Grand Index
13	f	0-12 years	0.797	873	112	Grand Index
13	f	0-12 years	0.798	873.582	113	Grand Index
13	f	0-12 years	0.802	874	113	Grand Index
13	f	0-12 years	0.803	875.127	113	Grand Index
13	f	0-12 years	0.804	876	113	Grand Index
13	f	0-12 years	0.805	876.5657379	113	Grand Index
13	f	0-12 years	0.806	877.054	113	Grand Index
13	f	0-12 years	0.807	878.2472404	113	Grand Index
13	f	0-12 years	0.808	878.8231948	113	Grand Index
13	f	0-12 years	0.809	878.9257125	113	Grand Index
13	f	0-12 years	0.812	879	113	Grand Index
13	f	0-12 years	0.813	879.5360234	113	Grand Index
13	f	0-12 years	0.815	880	113	Grand Index
13	f	0-12 years	0.818	881	114	Grand Index
13	f	0-12 years	0.819	881.0838485	114	Grand Index
13	f	0-12 years	0.822	882	114	Grand Index
13	f	0-12 years	0.823	882.0133185	114	Grand Index

Age	Gender	Education	Percentile	Raw Score	Norm Score	Assessment
13	f	0-12 years	0.824	882.0700509	114	Grand Index
13	f	0-12 years	0.825	882.9315006	114	Grand Index
13	f	0-12 years	0.828	883	114	Grand Index
13	f	0-12 years	0.829	883.1509943	114	Grand Index
13	f	0-12 years	0.83	883.9184359	114	Grand Index
13	f	0-12 years	0.831	884	114	Grand Index
13	f	0-12 years	0.832	884.088	114	Grand Index
13	f	0-12 years	0.833	885	114	Grand Index
13	f	0-12 years	0.834	885.706	115	Grand Index
13	f	0-12 years	0.835	886	115	Grand Index
13	f	0-12 years	0.836	886.324	115	Grand Index
13	f	0-12 years	0.841	887	115	Grand Index
13	f	0-12 years	0.842	887.9241062	115	Grand Index
13	f	0-12 years	0.844	888	115	Grand Index
13	f	0-12 years	0.845	888.9563821	115	Grand Index
13	f	0-12 years	0.846	889	115	Grand Index
13	f	0-12 years	0.847	889.723	115	Grand Index
13	f	0-12 years	0.848	890.0248754	115	Grand Index
13	f	0-12 years	0.849	890.8309643	115	Grand Index
13	f	0-12 years	0.85	890.9770967	116	Grand Index
13	f	0-12 years	0.851	891	116	Grand Index
13	f	0-12 years	0.852	891.1153776	116	Grand Index
13	f	0-12 years	0.853	891.5577246	116	Grand Index
13	f	0-12 years	0.854	892.8462121	116	Grand Index
13	f	0-12 years	0.855	893	116	Grand Index
13	f	0-12 years	0.856	893.504	116	Grand Index
13	f	0-12 years	0.857	894	116	Grand Index
13	f	0-12 years	0.858	895	116	Grand Index
13	f	0-12 years	0.859	895.431	116	Grand Index
13	f	0-12 years	0.861	896	116	Grand Index
13	f	0-12 years	0.862	896.1072183	116	Grand Index
13	f	0-12 years	0.863	896.766731	116	Grand Index
13	f	0-12 years	0.864	897	116	Grand Index
13	f	0-12 years	0.865	897.3724166	117	Grand Index
13	f	0-12 years	0.868	898	117	Grand Index
13	f	0-12 years	0.869	898.521	117	Grand Index
13	f	0-12 years	0.872	899	117	Grand Index
13	f	0-12 years	0.873	900.514	117	Grand Index
13	f	0-12 years	0.874	901	117	Grand Index
13	f	0-12 years	0.875	901.375	117	Grand Index
13	f	0-12 years	0.876	902	117	Grand Index
13	f	0-12 years	0.877	902.5810408	117	Grand Index
13	f	0-12 years	0.88	903	118	Grand Index
13	f	0-12 years	0.882	904	118	Grand Index
13	f	0-12 years	0.883	904.010507	118	Grand Index
13	f	0-12 years	0.886	905	118	Grand Index
13	f	0-12 years	0.887	905.083	118	Grand Index
13	f	0-12 years	0.888	906	118	Grand Index
13	f	0-12 years	0.889	906.0160542	118	Grand Index
13	f	0-12 years	0.89	906.5378992	118	Grand Index
13	f	0-12 years	0.892	907	119	Grand Index
13	f	0-12 years	0.893	907.937	119	Grand Index
13	f	0-12 years	0.894	908.246	119	Grand Index
13	f	0-12 years	0.898	909	119	Grand Index
13	f	0-12 years	0.899	909.791	119	Grand Index
13	f	0-12 years	0.9	910	119	Grand Index

Age	Gender	Education	Percentile	Raw Score	Norm Score	Assessment
13	f	0-12 years	0.901	910.409	119	Grand Index
13	f	0-12 years	0.902	911	119	Grand Index
13	f	0-12 years	0.903	911.0212368	119	Grand Index
13	f	0-12 years	0.904	911.8582685	120	Grand Index
13	f	0-12 years	0.905	912	120	Grand Index
13	f	0-12 years	0.906	912.954	120	Grand Index
13	f	0-12 years	0.909	913	120	Grand Index
13	f	0-12 years	0.91	915.19	120	Grand Index
13	f	0-12 years	0.912	916	120	Grand Index
13	f	0-12 years	0.913	916.1071912	120	Grand Index
13	f	0-12 years	0.914	916.9518781	120	Grand Index
13	f	0-12 years	0.915	917	121	Grand Index
13	f	0-12 years	0.916	918.0285092	121	Grand Index
13	f	0-12 years	0.917	918.7722152	121	Grand Index
13	f	0-12 years	0.918	919.0023752	121	Grand Index
13	f	0-12 years	0.919	919.6425702	121	Grand Index
13	f	0-12 years	0.921	920	121	Grand Index
13	f	0-12 years	0.922	920.898	121	Grand Index
13	f	0-12 years	0.923	921.0174385	121	Grand Index
13	f	0-12 years	0.924	921.3862293	121	Grand Index
13	f	0-12 years	0.925	921.9421602	122	Grand Index
13	f	0-12 years	0.926	922.134	122	Grand Index
13	f	0-12 years	0.928	923	122	Grand Index
13	f	0-12 years	0.929	923.7906022	122	Grand Index
13	f	0-12 years	0.931	924	122	Grand Index
13	f	0-12 years	0.932	925.4234794	122	Grand Index
13	f	0-12 years	0.933	926	122	Grand Index
13	f	0-12 years	0.934	926.606	123	Grand Index
13	f	0-12 years	0.935	927.915	123	Grand Index
13	f	0-12 years	0.936	928	123	Grand Index
13	f	0-12 years	0.937	928.0220618	123	Grand Index
13	f	0-12 years	0.938	928.8485399	123	Grand Index
13	f	0-12 years	0.939	929.3009504	123	Grand Index
13	f	0-12 years	0.94	930.46	123	Grand Index
13	f	0-12 years	0.941	931	123	Grand Index
13	f	0-12 years	0.942	932.078	124	Grand Index
13	f	0-12 years	0.943	933	124	Grand Index
13	f	0-12 years	0.944	933.0718273	124	Grand Index
13	f	0-12 years	0.945	933.9776	124	Grand Index
13	f	0-12 years	0.946	934.314	124	Grand Index
13	f	0-12 years	0.947	935	124	Grand Index
13	f	0-12 years	0.948	935.6983029	124	Grand Index
13	f	0-12 years	0.949	936.1725964	125	Grand Index
13	f	0-12 years	0.95	936.8722754	125	Grand Index
13	f	0-12 years	0.951	937	125	Grand Index
13	f	0-12 years	0.952	938	125	Grand Index
13	f	0-12 years	0.953	938.477	125	Grand Index
13	f	0-12 years	0.954	939	125	Grand Index
13	f	0-12 years	0.955	940.0433395	125	Grand Index
13	f	0-12 years	0.956	941	126	Grand Index
13	f	0-12 years	0.957	943.139	126	Grand Index
13	f	0-12 years	0.958	944	126	Grand Index
13	f	0-12 years	0.959	944.331	126	Grand Index
13	f	0-12 years	0.96	945.0032779	126	Grand Index
13	f	0-12 years	0.961	946.8982612	126	Grand Index
13	f	0-12 years	0.962	948.258	127	Grand Index

Age	Gender	Education	Percentile	Raw Score	Norm Score	Assessment
13	f	0-12 years	0.963	949.0725983	127	Grand Index
13	f	0-12 years	0.964	949.8918769	127	Grand Index
13	f	0-12 years	0.965	950.8472823	127	Grand Index
13	f	0-12 years	0.966	952.4981542	127	Grand Index
13	f	0-12 years	0.967	953.3837766	128	Grand Index
13	f	0-12 years	0.968	954.0466422	128	Grand Index
13	f	0-12 years	0.969	955.9251234	128	Grand Index
13	f	0-12 years	0.97	958	128	Grand Index
13	f	0-12 years	0.971	960.7150011	128	Grand Index
13	f	0-12 years	0.972	960.97704	129	Grand Index
13	f	0-12 years	0.973	961	129	Grand Index
13	f	0-12 years	0.974	963.2728179	129	Grand Index
13	f	0-12 years	0.975	964.275	129	Grand Index
13	f	0-12 years	0.976	965	130	Grand Index
13	f	0-12 years	0.977	965.3447633	130	Grand Index
13	f	0-12 years	0.978	966.3451917	130	Grand Index
13	f	0-12 years	0.979	967.8576374	131	Grand Index
13	f	0-12 years	0.98	968	131	Grand Index
13	f	0-12 years	0.981	968.129	131	Grand Index
13	f	0-12 years	0.982	969.438	131	Grand Index
13	f	0-12 years	0.983	970.1421816	132	Grand Index
13	f	0-12 years	0.984	972.056	132	Grand Index
13	f	0-12 years	0.985	973.365	133	Grand Index
13	f	0-12 years	0.986	976.696	133	Grand Index
13	f	0-12 years	0.987	978.983	133	Grand Index
13	f	0-12 years	0.988	982.6359014	134	Grand Index
13	f	0-12 years	0.989	984.67192	134	Grand Index
13	f	0-12 years	0.99	987.73	135	Grand Index
13	f	0-12 years	0.991	995.3314727	135	Grand Index
13	f	0-12 years	0.992	997.2141403	136	Grand Index
13	f	0-12 years	0.993	999.6027868	137	Grand Index
13	f	0-12 years	0.994	1001.333853	138	Grand Index
13	f	0-12 years	0.995	1009.086741	139	Grand Index
13	f	0-12 years	0.996	1009.260339	140	Grand Index
13	f	0-12 years	0.997	1011.369676	141	Grand Index
13	f	0-12 years	0.998	1016.551616	143	Grand Index
13	f	0-12 years	0.999	1020.698827	146	Grand Index
13	m	0-12 years	0.001	553.8547653	54	Grand Index
13	m	0-12 years	0.002	572.9847152	57	Grand Index
13	m	0-12 years	0.003	584.331877	59	Grand Index
13	m	0-12 years	0.004	590.7420262	60	Grand Index
13	m	0-12 years	0.005	597.9620254	61	Grand Index
13	m	0-12 years	0.006	606.0809515	62	Grand Index
13	m	0-12 years	0.007	613.0940402	63	Grand Index
13	m	0-12 years	0.008	617	64	Grand Index
13	m	0-12 years	0.009	619.9996427	65	Grand Index
13	m	0-12 years	0.01	621.0608634	65	Grand Index
13	m	0-12 years	0.011	625.7505698	66	Grand Index
13	m	0-12 years	0.012	626.8869271	66	Grand Index
13	m	0-12 years	0.013	629	67	Grand Index
13	m	0-12 years	0.014	632.884	67	Grand Index
13	m	0-12 years	0.015	639	67	Grand Index
13	m	0-12 years	0.016	641	68	Grand Index
13	m	0-12 years	0.017	644.085652	68	Grand Index
13	m	0-12 years	0.018	645.2229236	69	Grand Index
13	m	0-12 years	0.019	646.0646738	69	Grand Index

Age	Gender	Education	Percentile	Raw Score	Norm Score	Assessment
13	m	0-12 years	0.02	649.0400553	69	Grand Index
13	m	0-12 years	0.021	650	69	Grand Index
13	m	0-12 years	0.022	650.844	70	Grand Index
13	m	0-12 years	0.023	652	70	Grand Index
13	m	0-12 years	0.024	655.9874131	70	Grand Index
13	m	0-12 years	0.025	657	71	Grand Index
13	m	0-12 years	0.026	657.9237895	71	Grand Index
13	m	0-12 years	0.027	658.285336	71	Grand Index
13	m	0-12 years	0.028	659.011042	71	Grand Index
13	m	0-12 years	0.029	661	72	Grand Index
13	m	0-12 years	0.03	662.12	72	Grand Index
13	m	0-12 years	0.031	664	72	Grand Index
13	m	0-12 years	0.033	665	72	Grand Index
13	m	0-12 years	0.034	666.5080355	73	Grand Index
13	m	0-12 years	0.035	667	73	Grand Index
13	m	0-12 years	0.036	669.1215458	73	Grand Index
13	m	0-12 years	0.037	670.2255718	73	Grand Index
13	m	0-12 years	0.038	671	73	Grand Index
13	m	0-12 years	0.039	671.178	74	Grand Index
13	m	0-12 years	0.04	673.0688088	74	Grand Index
13	m	0-12 years	0.041	673.997482	74	Grand Index
13	m	0-12 years	0.042	674.0105135	74	Grand Index
13	m	0-12 years	0.043	674.4875032	74	Grand Index
13	m	0-12 years	0.044	675.6811685	74	Grand Index
13	m	0-12 years	0.045	676.59	75	Grand Index
13	m	0-12 years	0.046	677	75	Grand Index
13	m	0-12 years	0.047	677.178376	75	Grand Index
13	m	0-12 years	0.048	678	75	Grand Index
13	m	0-12 years	0.049	678.198	75	Grand Index
13	m	0-12 years	0.05	680	75	Grand Index
13	m	0-12 years	0.051	680.002	75	Grand Index
13	m	0-12 years	0.052	681.904	76	Grand Index
13	m	0-12 years	0.053	682.237129	76	Grand Index
13	m	0-12 years	0.054	683	76	Grand Index
13	m	0-12 years	0.055	684.5682224	76	Grand Index
13	m	0-12 years	0.056	685	76	Grand Index
13	m	0-12 years	0.057	686	76	Grand Index
13	m	0-12 years	0.058	686.316	76	Grand Index
13	m	0-12 years	0.059	687.436	77	Grand Index
13	m	0-12 years	0.061	689	77	Grand Index
13	m	0-12 years	0.062	689.924	77	Grand Index
13	m	0-12 years	0.063	690.766662	77	Grand Index
13	m	0-12 years	0.064	691	77	Grand Index
13	m	0-12 years	0.065	692.26	77	Grand Index
13	m	0-12 years	0.067	693	78	Grand Index
13	m	0-12 years	0.069	694	78	Grand Index
13	m	0-12 years	0.07	694.4038612	78	Grand Index
13	m	0-12 years	0.071	695.0403235	78	Grand Index
13	m	0-12 years	0.072	695.9977647	78	Grand Index
13	m	0-12 years	0.073	696.8531656	78	Grand Index
13	m	0-12 years	0.074	697	78	Grand Index
13	m	0-12 years	0.075	697.65	78	Grand Index
13	m	0-12 years	0.077	699	79	Grand Index
13	m	0-12 years	0.078	699.0956475	79	Grand Index
13	m	0-12 years	0.079	699.9892827	79	Grand Index
13	m	0-12 years	0.08	700.193745	79	Grand Index

Age	Gender	Education	Percentile	Raw Score	Norm Score	Assessment
13	m	0-12 years	0.081	700.7838824	79	Grand Index
13	m	0-12 years	0.082	700.9941754	79	Grand Index
13	m	0-12 years	0.084	701	79	Grand Index
13	m	0-12 years	0.085	701.7628845	79	Grand Index
13	m	0-12 years	0.088	703	80	Grand Index
13	m	0-12 years	0.089	703.534437	80	Grand Index
13	m	0-12 years	0.09	704	80	Grand Index
13	m	0-12 years	0.091	704.1019417	80	Grand Index
13	m	0-12 years	0.093	705	80	Grand Index
13	m	0-12 years	0.094	705.788	80	Grand Index
13	m	0-12 years	0.095	706.69	80	Grand Index
13	m	0-12 years	0.096	707	80	Grand Index
13	m	0-12 years	0.1	708	81	Grand Index
13	m	0-12 years	0.101	708.1421559	81	Grand Index
13	m	0-12 years	0.102	709.004	81	Grand Index
13	m	0-12 years	0.103	710	81	Grand Index
13	m	0-12 years	0.104	710.808	81	Grand Index
13	m	0-12 years	0.106	711	81	Grand Index
13	m	0-12 years	0.107	712	81	Grand Index
13	m	0-12 years	0.108	712.3764735	81	Grand Index
13	m	0-12 years	0.11	713	82	Grand Index
13	m	0-12 years	0.111	713.909752	82	Grand Index
13	m	0-12 years	0.113	714	82	Grand Index
13	m	0-12 years	0.114	714.828	82	Grand Index
13	m	0-12 years	0.115	715.7926292	82	Grand Index
13	m	0-12 years	0.116	715.9652532	82	Grand Index
13	m	0-12 years	0.118	716	82	Grand Index
13	m	0-12 years	0.119	717	82	Grand Index
13	m	0-12 years	0.12	717.967803	82	Grand Index
13	m	0-12 years	0.121	718	82	Grand Index
13	m	0-12 years	0.122	718.0106442	83	Grand Index
13	m	0-12 years	0.123	718.9590633	83	Grand Index
13	m	0-12 years	0.124	719.0130406	83	Grand Index
13	m	0-12 years	0.125	719.8580886	83	Grand Index
13	m	0-12 years	0.126	720	83	Grand Index
13	m	0-12 years	0.127	720.554	83	Grand Index
13	m	0-12 years	0.128	721	83	Grand Index
13	m	0-12 years	0.129	721.3362722	83	Grand Index
13	m	0-12 years	0.13	722.0272154	83	Grand Index
13	m	0-12 years	0.132	723	83	Grand Index
13	m	0-12 years	0.133	723.099463	83	Grand Index
13	m	0-12 years	0.135	724	83	Grand Index
13	m	0-12 years	0.136	724.672	84	Grand Index
13	m	0-12 years	0.138	725	84	Grand Index
13	m	0-12 years	0.139	725.4181577	84	Grand Index
13	m	0-12 years	0.141	726	84	Grand Index
13	m	0-12 years	0.142	726.5468787	84	Grand Index
13	m	0-12 years	0.143	726.9998857	84	Grand Index
13	m	0-12 years	0.144	727.888	84	Grand Index
13	m	0-12 years	0.146	728	84	Grand Index
13	m	0-12 years	0.147	728.7736736	84	Grand Index
13	m	0-12 years	0.148	729	84	Grand Index
13	m	0-12 years	0.149	729.0710865	84	Grand Index
13	m	0-12 years	0.15	730.4827976	84	Grand Index
13	m	0-12 years	0.153	731	85	Grand Index
13	m	0-12 years	0.154	731.7889849	85	Grand Index

Age	Gender	Education	Percentile	Raw Score	Norm Score	Assessment
13	m	0-12 years	0.156	732	85	Grand Index
13	m	0-12 years	0.157	732.5165774	85	Grand Index
13	m	0-12 years	0.158	732.9972488	85	Grand Index
13	m	0-12 years	0.161	733	85	Grand Index
13	m	0-12 years	0.162	733.3720099	85	Grand Index
13	m	0-12 years	0.165	734	85	Grand Index
13	m	0-12 years	0.166	734.5313903	85	Grand Index
13	m	0-12 years	0.167	734.8760033	86	Grand Index
13	m	0-12 years	0.168	734.9553747	86	Grand Index
13	m	0-12 years	0.169	734.9973517	86	Grand Index
13	m	0-12 years	0.171	735	86	Grand Index
13	m	0-12 years	0.172	735.7051237	86	Grand Index
13	m	0-12 years	0.175	736	86	Grand Index
13	m	0-12 years	0.176	736.9989856	86	Grand Index
13	m	0-12 years	0.179	737	86	Grand Index
13	m	0-12 years	0.18	737.3837614	86	Grand Index
13	m	0-12 years	0.182	738	86	Grand Index
13	m	0-12 years	0.186	739	87	Grand Index
13	m	0-12 years	0.187	739.674	87	Grand Index
13	m	0-12 years	0.188	740.576	87	Grand Index
13	m	0-12 years	0.189	741	87	Grand Index
13	m	0-12 years	0.19	741.1829211	87	Grand Index
13	m	0-12 years	0.194	742	87	Grand Index
13	m	0-12 years	0.195	742.9128558	87	Grand Index
13	m	0-12 years	0.196	743.792	87	Grand Index
13	m	0-12 years	0.197	744	87	Grand Index
13	m	0-12 years	0.198	744.4262627	87	Grand Index
13	m	0-12 years	0.2	745	87	Grand Index
13	m	0-12 years	0.202	746	87	Grand Index
13	m	0-12 years	0.203	746.3895595	88	Grand Index
13	m	0-12 years	0.205	747	88	Grand Index
13	m	0-12 years	0.206	747.9936233	88	Grand Index
13	m	0-12 years	0.209	748	88	Grand Index
13	m	0-12 years	0.21	748.4414736	88	Grand Index
13	m	0-12 years	0.213	749	88	Grand Index
13	m	0-12 years	0.214	749.028	88	Grand Index
13	m	0-12 years	0.216	750	88	Grand Index
13	m	0-12 years	0.217	750.3675173	88	Grand Index
13	m	0-12 years	0.218	751.0085645	88	Grand Index
13	m	0-12 years	0.221	752	88	Grand Index
13	m	0-12 years	0.225	753	89	Grand Index
13	m	0-12 years	0.227	754	89	Grand Index
13	m	0-12 years	0.228	755	89	Grand Index
13	m	0-12 years	0.229	755.0120117	89	Grand Index
13	m	0-12 years	0.232	756	89	Grand Index
13	m	0-12 years	0.233	756.8772409	89	Grand Index
13	m	0-12 years	0.238	757	89	Grand Index
13	m	0-12 years	0.239	757.578	89	Grand Index
13	m	0-12 years	0.242	758	90	Grand Index
13	m	0-12 years	0.243	758.186	90	Grand Index
13	m	0-12 years	0.246	759	90	Grand Index
13	m	0-12 years	0.247	759.0580606	90	Grand Index
13	m	0-12 years	0.248	759.7644037	90	Grand Index
13	m	0-12 years	0.25	760	90	Grand Index
13	m	0-12 years	0.251	760.3156549	90	Grand Index
13	m	0-12 years	0.256	761	90	Grand Index

Age	Gender	Education	Percentile	Raw Score	Norm Score	Assessment
13	m	0-12 years	0.257	762	90	Grand Index
13	m	0-12 years	0.26	763	90	Grand Index
13	m	0-12 years	0.261	763.0100841	90	Grand Index
13	m	0-12 years	0.266	764	91	Grand Index
13	m	0-12 years	0.267	764.7700072	91	Grand Index
13	m	0-12 years	0.269	765	91	Grand Index
13	m	0-12 years	0.27	765.0044888	91	Grand Index
13	m	0-12 years	0.271	765.5821274	91	Grand Index
13	m	0-12 years	0.273	766	91	Grand Index
13	m	0-12 years	0.274	766.148	91	Grand Index
13	m	0-12 years	0.276	767	91	Grand Index
13	m	0-12 years	0.277	767.5397481	91	Grand Index
13	m	0-12 years	0.278	767.9079791	91	Grand Index
13	m	0-12 years	0.28	768	91	Grand Index
13	m	0-12 years	0.281	768.462	91	Grand Index
13	m	0-12 years	0.287	769	92	Grand Index
13	m	0-12 years	0.288	769.920535	92	Grand Index
13	m	0-12 years	0.292	770	92	Grand Index
13	m	0-12 years	0.295	771	92	Grand Index
13	m	0-12 years	0.296	771.9295173	92	Grand Index
13	m	0-12 years	0.299	772	92	Grand Index
13	m	0-12 years	0.3	772.3336545	92	Grand Index
13	m	0-12 years	0.301	772.8159481	92	Grand Index
13	m	0-12 years	0.304	773	92	Grand Index
13	m	0-12 years	0.305	773.5311469	92	Grand Index
13	m	0-12 years	0.309	774	93	Grand Index
13	m	0-12 years	0.31	774.2870667	93	Grand Index
13	m	0-12 years	0.311	774.9968438	93	Grand Index
13	m	0-12 years	0.313	775	93	Grand Index
13	m	0-12 years	0.314	775.228	93	Grand Index
13	m	0-12 years	0.318	776	93	Grand Index
13	m	0-12 years	0.319	776.738	93	Grand Index
13	m	0-12 years	0.32	777.1298449	93	Grand Index
13	m	0-12 years	0.324	778	93	Grand Index
13	m	0-12 years	0.329	779	93	Grand Index
13	m	0-12 years	0.33	779.0424716	93	Grand Index
13	m	0-12 years	0.331	779.6621066	93	Grand Index
13	m	0-12 years	0.335	780	94	Grand Index
13	m	0-12 years	0.336	780.0417282	94	Grand Index
13	m	0-12 years	0.337	780.9890685	94	Grand Index
13	m	0-12 years	0.339	781	94	Grand Index
13	m	0-12 years	0.34	781.7102798	94	Grand Index
13	m	0-12 years	0.343	782	94	Grand Index
13	m	0-12 years	0.349	783	94	Grand Index
13	m	0-12 years	0.35	783.815879	94	Grand Index
13	m	0-12 years	0.351	784.064612	94	Grand Index
13	m	0-12 years	0.353	785	94	Grand Index
13	m	0-12 years	0.36	786	95	Grand Index
13	m	0-12 years	0.361	786.2628282	95	Grand Index
13	m	0-12 years	0.362	786.5751481	95	Grand Index
13	m	0-12 years	0.364	787	95	Grand Index
13	m	0-12 years	0.365	787.0072659	95	Grand Index
13	m	0-12 years	0.366	787.2174801	95	Grand Index
13	m	0-12 years	0.371	788	95	Grand Index
13	m	0-12 years	0.372	788.4634917	95	Grand Index
13	m	0-12 years	0.377	789	95	Grand Index

Age	Gender	Education	Percentile	Raw Score	Norm Score	Assessment
13	m	0-12 years	0.378	789.0915458	95	Grand Index
13	m	0-12 years	0.38	790	95	Grand Index
13	m	0-12 years	0.381	790.5664441	95	Grand Index
13	m	0-12 years	0.385	791	96	Grand Index
13	m	0-12 years	0.386	791.1077	96	Grand Index
13	m	0-12 years	0.387	791.8281911	96	Grand Index
13	m	0-12 years	0.392	792	96	Grand Index
13	m	0-12 years	0.396	793	96	Grand Index
13	m	0-12 years	0.397	793.094	96	Grand Index
13	m	0-12 years	0.401	794	96	Grand Index
13	m	0-12 years	0.402	794.6935498	96	Grand Index
13	m	0-12 years	0.409	795	97	Grand Index
13	m	0-12 years	0.41	795.4224446	97	Grand Index
13	m	0-12 years	0.413	796	97	Grand Index
13	m	0-12 years	0.414	796.6299634	97	Grand Index
13	m	0-12 years	0.42	797	97	Grand Index
13	m	0-12 years	0.421	797.4475485	97	Grand Index
13	m	0-12 years	0.422	797.9486135	97	Grand Index
13	m	0-12 years	0.427	798	97	Grand Index
13	m	0-12 years	0.428	798.05483	97	Grand Index
13	m	0-12 years	0.429	798.9991225	97	Grand Index
13	m	0-12 years	0.43	799	97	Grand Index
13	m	0-12 years	0.431	799.2389528	97	Grand Index
13	m	0-12 years	0.437	800	98	Grand Index
13	m	0-12 years	0.438	800.1043146	98	Grand Index
13	m	0-12 years	0.439	800.7895328	98	Grand Index
13	m	0-12 years	0.443	801	98	Grand Index
13	m	0-12 years	0.448	802	98	Grand Index
13	m	0-12 years	0.449	802.115626	98	Grand Index
13	m	0-12 years	0.455	803	98	Grand Index
13	m	0-12 years	0.458	804	98	Grand Index
13	m	0-12 years	0.461	805	99	Grand Index
13	m	0-12 years	0.462	805.0043237	99	Grand Index
13	m	0-12 years	0.465	806	99	Grand Index
13	m	0-12 years	0.466	806.0117141	99	Grand Index
13	m	0-12 years	0.471	807	99	Grand Index
13	m	0-12 years	0.472	807.2465567	99	Grand Index
13	m	0-12 years	0.473	807.8597975	99	Grand Index
13	m	0-12 years	0.476	808	99	Grand Index
13	m	0-12 years	0.477	808.7400866	99	Grand Index
13	m	0-12 years	0.479	809	99	Grand Index
13	m	0-12 years	0.48	809.8480251	99	Grand Index
13	m	0-12 years	0.485	810	99	Grand Index
13	m	0-12 years	0.488	811	100	Grand Index
13	m	0-12 years	0.489	811.8490661	100	Grand Index
13	m	0-12 years	0.494	812	100	Grand Index
13	m	0-12 years	0.495	812.5081475	100	Grand Index
13	m	0-12 years	0.499	813	100	Grand Index
13	m	0-12 years	0.5	813.888	100	Grand Index
13	m	0-12 years	0.503	814	100	Grand Index
13	m	0-12 years	0.508	815	100	Grand Index
13	m	0-12 years	0.513	816	100	Grand Index
13	m	0-12 years	0.514	816.8193034	101	Grand Index
13	m	0-12 years	0.518	817	101	Grand Index
13	m	0-12 years	0.519	817.0147759	101	Grand Index
13	m	0-12 years	0.52	817.9470345	101	Grand Index

Age	Gender	Education	Percentile	Raw Score	Norm Score	Assessment
13	m	0-12 years	0.525	818	101	Grand Index
13	m	0-12 years	0.529	819	101	Grand Index
13	m	0-12 years	0.53	819.06	101	Grand Index
13	m	0-12 years	0.536	820	101	Grand Index
13	m	0-12 years	0.537	820.010349	101	Grand Index
13	m	0-12 years	0.544	821	102	Grand Index
13	m	0-12 years	0.545	821.950097	102	Grand Index
13	m	0-12 years	0.547	822	102	Grand Index
13	m	0-12 years	0.554	823	102	Grand Index
13	m	0-12 years	0.559	824	102	Grand Index
13	m	0-12 years	0.563	825	102	Grand Index
13	m	0-12 years	0.564	825.8635623	102	Grand Index
13	m	0-12 years	0.565	826	102	Grand Index
13	m	0-12 years	0.566	826.532	102	Grand Index
13	m	0-12 years	0.571	827	103	Grand Index
13	m	0-12 years	0.572	827.8790847	103	Grand Index
13	m	0-12 years	0.576	828	103	Grand Index
13	m	0-12 years	0.577	828.7428247	103	Grand Index
13	m	0-12 years	0.58	829	103	Grand Index
13	m	0-12 years	0.581	829.2816076	103	Grand Index
13	m	0-12 years	0.583	830	103	Grand Index
13	m	0-12 years	0.586	831	103	Grand Index
13	m	0-12 years	0.587	831.1022477	103	Grand Index
13	m	0-12 years	0.588	831.9259279	103	Grand Index
13	m	0-12 years	0.593	832	104	Grand Index
13	m	0-12 years	0.594	832.7417756	104	Grand Index
13	m	0-12 years	0.596	833	104	Grand Index
13	m	0-12 years	0.597	833.7704966	104	Grand Index
13	m	0-12 years	0.6	834	104	Grand Index
13	m	0-12 years	0.601	834.0000846	104	Grand Index
13	m	0-12 years	0.606	835	104	Grand Index
13	m	0-12 years	0.607	835.6361755	104	Grand Index
13	m	0-12 years	0.611	836	104	Grand Index
13	m	0-12 years	0.612	836.0067424	104	Grand Index
13	m	0-12 years	0.613	836.9096826	104	Grand Index
13	m	0-12 years	0.615	837	104	Grand Index
13	m	0-12 years	0.616	837.7911806	104	Grand Index
13	m	0-12 years	0.621	838	105	Grand Index
13	m	0-12 years	0.624	839	105	Grand Index
13	m	0-12 years	0.625	839.6201149	105	Grand Index
13	m	0-12 years	0.629	840	105	Grand Index
13	m	0-12 years	0.63	840.004998	105	Grand Index
13	m	0-12 years	0.631	840.2717517	105	Grand Index
13	m	0-12 years	0.635	841	105	Grand Index
13	m	0-12 years	0.636	841.672	105	Grand Index
13	m	0-12 years	0.638	842	105	Grand Index
13	m	0-12 years	0.639	842.378	105	Grand Index
13	m	0-12 years	0.642	843	105	Grand Index
13	m	0-12 years	0.643	843.986	105	Grand Index
13	m	0-12 years	0.648	844	106	Grand Index
13	m	0-12 years	0.649	844.398	106	Grand Index
13	m	0-12 years	0.654	845	106	Grand Index
13	m	0-12 years	0.657	846	106	Grand Index
13	m	0-12 years	0.66	847	106	Grand Index
13	m	0-12 years	0.661	847.222	106	Grand Index
13	m	0-12 years	0.666	848	106	Grand Index

Age	Gender	Education	Percentile	Raw Score	Norm Score	Assessment
13	m	0-12 years	0.667	848.385355	106	Grand Index
13	m	0-12 years	0.672	849	107	Grand Index
13	m	0-12 years	0.673	849.046	107	Grand Index
13	m	0-12 years	0.675	850	107	Grand Index
13	m	0-12 years	0.676	850.9054972	107	Grand Index
13	m	0-12 years	0.681	851	107	Grand Index
13	m	0-12 years	0.682	851.0510312	107	Grand Index
13	m	0-12 years	0.683	851.4082546	107	Grand Index
13	m	0-12 years	0.684	851.9968008	107	Grand Index
13	m	0-12 years	0.689	852	107	Grand Index
13	m	0-12 years	0.691	853	107	Grand Index
13	m	0-12 years	0.692	853.0942765	108	Grand Index
13	m	0-12 years	0.693	853.9572227	108	Grand Index
13	m	0-12 years	0.697	854	108	Grand Index
13	m	0-12 years	0.698	854.6234785	108	Grand Index
13	m	0-12 years	0.705	855	108	Grand Index
13	m	0-12 years	0.706	855.3743795	108	Grand Index
13	m	0-12 years	0.709	856	108	Grand Index
13	m	0-12 years	0.71	856.0473806	108	Grand Index
13	m	0-12 years	0.713	857	108	Grand Index
13	m	0-12 years	0.714	857.0132169	108	Grand Index
13	m	0-12 years	0.715	857.7697316	109	Grand Index
13	m	0-12 years	0.716	857.9739269	109	Grand Index
13	m	0-12 years	0.72	858	109	Grand Index
13	m	0-12 years	0.725	859	109	Grand Index
13	m	0-12 years	0.726	859.1992595	109	Grand Index
13	m	0-12 years	0.732	860	109	Grand Index
13	m	0-12 years	0.733	860.9967274	109	Grand Index
13	m	0-12 years	0.736	861	109	Grand Index
13	m	0-12 years	0.737	861.0712204	110	Grand Index
13	m	0-12 years	0.738	861.9639147	110	Grand Index
13	m	0-12 years	0.741	862	110	Grand Index
13	m	0-12 years	0.745	863	110	Grand Index
13	m	0-12 years	0.746	863.3414028	110	Grand Index
13	m	0-12 years	0.747	863.7916274	110	Grand Index
13	m	0-12 years	0.748	863.993978	110	Grand Index
13	m	0-12 years	0.75	864	110	Grand Index
13	m	0-12 years	0.751	864.402	110	Grand Index
13	m	0-12 years	0.754	865	110	Grand Index
13	m	0-12 years	0.755	865.0001259	110	Grand Index
13	m	0-12 years	0.756	865.435181	110	Grand Index
13	m	0-12 years	0.757	865.6171908	110	Grand Index
13	m	0-12 years	0.76	866	111	Grand Index
13	m	0-12 years	0.761	866.6441521	111	Grand Index
13	m	0-12 years	0.762	866.918574	111	Grand Index
13	m	0-12 years	0.764	867	111	Grand Index
13	m	0-12 years	0.765	867.03	111	Grand Index
13	m	0-12 years	0.767	868	111	Grand Index
13	m	0-12 years	0.768	868.8952367	111	Grand Index
13	m	0-12 years	0.769	869	111	Grand Index
13	m	0-12 years	0.773	870	111	Grand Index
13	m	0-12 years	0.774	870.9828328	111	Grand Index
13	m	0-12 years	0.778	871	111	Grand Index
13	m	0-12 years	0.782	872	112	Grand Index
13	m	0-12 years	0.783	872.113973	112	Grand Index
13	m	0-12 years	0.784	872.9873939	112	Grand Index

Age	Gender	Education	Percentile	Raw Score	Norm Score	Assessment
13	m	0-12 years	0.787	873	112	Grand Index
13	m	0-12 years	0.788	873.0096769	112	Grand Index
13	m	0-12 years	0.789	873.8716961	112	Grand Index
13	m	0-12 years	0.792	874	112	Grand Index
13	m	0-12 years	0.794	875	112	Grand Index
13	m	0-12 years	0.795	875.041356	112	Grand Index
13	m	0-12 years	0.796	875.790896	112	Grand Index
13	m	0-12 years	0.798	876	113	Grand Index
13	m	0-12 years	0.803	877	113	Grand Index
13	m	0-12 years	0.804	877.208	113	Grand Index
13	m	0-12 years	0.805	878	113	Grand Index
13	m	0-12 years	0.806	878.9742016	113	Grand Index
13	m	0-12 years	0.81	879	113	Grand Index
13	m	0-12 years	0.811	879.5860255	113	Grand Index
13	m	0-12 years	0.814	880	113	Grand Index
13	m	0-12 years	0.818	881	114	Grand Index
13	m	0-12 years	0.819	881.738	114	Grand Index
13	m	0-12 years	0.82	882	114	Grand Index
13	m	0-12 years	0.823	883	114	Grand Index
13	m	0-12 years	0.824	883.2397515	114	Grand Index
13	m	0-12 years	0.827	884	114	Grand Index
13	m	0-12 years	0.828	884.2209024	114	Grand Index
13	m	0-12 years	0.832	885	114	Grand Index
13	m	0-12 years	0.837	886	115	Grand Index
13	m	0-12 years	0.838	886.8820326	115	Grand Index
13	m	0-12 years	0.84	887	115	Grand Index
13	m	0-12 years	0.841	887.8091425	115	Grand Index
13	m	0-12 years	0.842	888.309756	115	Grand Index
13	m	0-12 years	0.844	889	115	Grand Index
13	m	0-12 years	0.845	889.0831737	115	Grand Index
13	m	0-12 years	0.846	889.8431089	115	Grand Index
13	m	0-12 years	0.847	889.9999037	115	Grand Index
13	m	0-12 years	0.849	890	115	Grand Index
13	m	0-12 years	0.85	890.7547368	116	Grand Index
13	m	0-12 years	0.851	891	116	Grand Index
13	m	0-12 years	0.852	891.504	116	Grand Index
13	m	0-12 years	0.853	892	116	Grand Index
13	m	0-12 years	0.855	893	116	Grand Index
13	m	0-12 years	0.856	893.4563617	116	Grand Index
13	m	0-12 years	0.857	894	116	Grand Index
13	m	0-12 years	0.858	894.2536102	116	Grand Index
13	m	0-12 years	0.859	894.9939613	116	Grand Index
13	m	0-12 years	0.861	895	116	Grand Index
13	m	0-12 years	0.862	895.524	116	Grand Index
13	m	0-12 years	0.864	896	116	Grand Index
13	m	0-12 years	0.865	896.021799	117	Grand Index
13	m	0-12 years	0.866	897	117	Grand Index
13	m	0-12 years	0.867	897.4216081	117	Grand Index
13	m	0-12 years	0.868	897.9629834	117	Grand Index
13	m	0-12 years	0.87	898	117	Grand Index
13	m	0-12 years	0.871	899	117	Grand Index
13	m	0-12 years	0.872	899.544	117	Grand Index
13	m	0-12 years	0.876	900	117	Grand Index
13	m	0-12 years	0.877	900.0255255	117	Grand Index
13	m	0-12 years	0.878	900.7342123	117	Grand Index
13	m	0-12 years	0.879	901	118	Grand Index

Age	Gender	Education	Percentile	Raw Score	Norm Score	Assessment
13	m	0-12 years	0.88	901.0452961	118	Grand Index
13	m	0-12 years	0.881	901.6907267	118	Grand Index
13	m	0-12 years	0.882	902	118	Grand Index
13	m	0-12 years	0.883	902.466	118	Grand Index
13	m	0-12 years	0.884	903	118	Grand Index
13	m	0-12 years	0.885	904.27	118	Grand Index
13	m	0-12 years	0.886	905	118	Grand Index
13	m	0-12 years	0.887	905.0477061	118	Grand Index
13	m	0-12 years	0.888	905.9914722	118	Grand Index
13	m	0-12 years	0.889	906	118	Grand Index
13	m	0-12 years	0.89	907	118	Grand Index
13	m	0-12 years	0.891	908.360244	118	Grand Index
13	m	0-12 years	0.893	909	119	Grand Index
13	m	0-12 years	0.894	910	119	Grand Index
13	m	0-12 years	0.895	910.1073896	119	Grand Index
13	m	0-12 years	0.896	911	119	Grand Index
13	m	0-12 years	0.897	911.0516868	119	Grand Index
13	m	0-12 years	0.898	911.9784694	119	Grand Index
13	m	0-12 years	0.899	912	119	Grand Index
13	m	0-12 years	0.9	912.819412	119	Grand Index
13	m	0-12 years	0.903	913	119	Grand Index
13	m	0-12 years	0.904	913.0005858	120	Grand Index
13	m	0-12 years	0.905	913.3144094	120	Grand Index
13	m	0-12 years	0.906	914	120	Grand Index
13	m	0-12 years	0.907	914.6018439	120	Grand Index
13	m	0-12 years	0.909	915	120	Grand Index
13	m	0-12 years	0.91	915.5141763	120	Grand Index
13	m	0-12 years	0.911	916	120	Grand Index
13	m	0-12 years	0.912	917.624	120	Grand Index
13	m	0-12 years	0.914	918	120	Grand Index
13	m	0-12 years	0.916	919	121	Grand Index
13	m	0-12 years	0.917	920	121	Grand Index
13	m	0-12 years	0.918	920.2661695	121	Grand Index
13	m	0-12 years	0.919	921	121	Grand Index
13	m	0-12 years	0.92	921.3393723	121	Grand Index
13	m	0-12 years	0.921	921.8749244	121	Grand Index
13	m	0-12 years	0.923	922	121	Grand Index
13	m	0-12 years	0.924	922.8521967	121	Grand Index
13	m	0-12 years	0.926	923	122	Grand Index
13	m	0-12 years	0.927	924	122	Grand Index
13	m	0-12 years	0.928	924.056	122	Grand Index
13	m	0-12 years	0.929	925	122	Grand Index
13	m	0-12 years	0.932	926	122	Grand Index
13	m	0-12 years	0.933	927	122	Grand Index
13	m	0-12 years	0.934	928.3426885	123	Grand Index
13	m	0-12 years	0.935	928.9854582	123	Grand Index
13	m	0-12 years	0.936	929.0711761	123	Grand Index
13	m	0-12 years	0.937	931.1686619	123	Grand Index
13	m	0-12 years	0.938	932.003375	123	Grand Index
13	m	0-12 years	0.939	932.978977	123	Grand Index
13	m	0-12 years	0.94	933.8911519	123	Grand Index
13	m	0-12 years	0.941	934	123	Grand Index
13	m	0-12 years	0.942	934.8043883	124	Grand Index
13	m	0-12 years	0.943	936	124	Grand Index
13	m	0-12 years	0.944	936.2493192	124	Grand Index
13	m	0-12 years	0.945	937	124	Grand Index

Age	Gender	Education	Percentile	Raw Score	Norm Score	Assessment
13	m	0-12 years	0.946	937.292	124	Grand Index
13	m	0-12 years	0.947	938	124	Grand Index
13	m	0-12 years	0.948	938.096	124	Grand Index
13	m	0-12 years	0.949	939	125	Grand Index
13	m	0-12 years	0.95	939.9072112	125	Grand Index
13	m	0-12 years	0.951	941.802	125	Grand Index
13	m	0-12 years	0.952	943	125	Grand Index
13	m	0-12 years	0.953	943.9723785	125	Grand Index
13	m	0-12 years	0.954	945	125	Grand Index
13	m	0-12 years	0.955	947.3708767	125	Grand Index
13	m	0-12 years	0.958	948	126	Grand Index
13	m	0-12 years	0.96	949	126	Grand Index
13	m	0-12 years	0.961	951.644	126	Grand Index
13	m	0-12 years	0.962	952.8086689	127	Grand Index
13	m	0-12 years	0.963	953.4173647	127	Grand Index
13	m	0-12 years	0.964	954	127	Grand Index
13	m	0-12 years	0.965	955	127	Grand Index
13	m	0-12 years	0.966	957.2974706	127	Grand Index
13	m	0-12 years	0.967	958.1289922	128	Grand Index
13	m	0-12 years	0.969	959	128	Grand Index
13	m	0-12 years	0.97	960.88	128	Grand Index
13	m	0-12 years	0.971	961	128	Grand Index
13	m	0-12 years	0.972	962	129	Grand Index
13	m	0-12 years	0.973	962.9123464	129	Grand Index
13	m	0-12 years	0.974	965.0446455	129	Grand Index
13	m	0-12 years	0.975	968.4102357	129	Grand Index
13	m	0-12 years	0.976	969.3186394	130	Grand Index
13	m	0-12 years	0.977	972.254	130	Grand Index
13	m	0-12 years	0.978	974.0300826	130	Grand Index
13	m	0-12 years	0.979	975.8600635	131	Grand Index
13	m	0-12 years	0.98	978	131	Grand Index
13	m	0-12 years	0.981	979	131	Grand Index
13	m	0-12 years	0.982	979.8680967	131	Grand Index
13	m	0-12 years	0.983	980.0133736	132	Grand Index
13	m	0-12 years	0.984	980.8422213	132	Grand Index
13	m	0-12 years	0.985	981.9438156	133	Grand Index
13	m	0-12 years	0.986	983.3504009	133	Grand Index
13	m	0-12 years	0.987	984.983666	133	Grand Index
13	m	0-12 years	0.988	988.528	134	Grand Index
13	m	0-12 years	0.989	993.548639	134	Grand Index
13	m	0-12 years	0.99	997.8711316	135	Grand Index
13	m	0-12 years	0.991	999.7158334	135	Grand Index
13	m	0-12 years	0.992	1004.829012	136	Grand Index
13	m	0-12 years	0.993	1012.294638	137	Grand Index
13	m	0-12 years	0.994	1016.336495	138	Grand Index
13	m	0-12 years	0.995	1021.47	139	Grand Index
13	m	0-12 years	0.996	1026.646229	140	Grand Index
13	m	0-12 years	0.997	1033.708476	141	Grand Index
13	m	0-12 years	0.998	1039.222313	143	Grand Index
13	m	0-12 years	0.999	1058.664624	146	Grand Index
13	f	13-16 years	0.001	553.75	54	Grand Index
13	f	13-16 years	0.002	564.5	57	Grand Index
13	f	13-16 years	0.003	571.3737495	59	Grand Index
13	f	13-16 years	0.004	576.3762056	60	Grand Index
13	f	13-16 years	0.005	584.5620688	61	Grand Index
13	f	13-16 years	0.006	598.6902917	62	Grand Index

Age	Gender	Education	Percentile	Raw Score	Norm Score	Assessment
13	f	13-16 years	0.007	612.7550518	63	Grand Index
13	f	13-16 years	0.008	624.1543727	64	Grand Index
13	f	13-16 years	0.009	635.5536937	65	Grand Index
13	f	13-16 years	0.01	641.3673019	65	Grand Index
13	f	13-16 years	0.011	644.7604347	66	Grand Index
13	f	13-16 years	0.012	647.2284454	66	Grand Index
13	f	13-16 years	0.013	648.1353126	67	Grand Index
13	f	13-16 years	0.014	649.02	67	Grand Index
13	f	13-16 years	0.015	649.45	67	Grand Index
13	f	13-16 years	0.016	649.88	68	Grand Index
13	f	13-16 years	0.017	650.31	68	Grand Index
13	f	13-16 years	0.018	650.74	69	Grand Index
13	f	13-16 years	0.019	651.85	69	Grand Index
13	f	13-16 years	0.02	654	69	Grand Index
13	f	13-16 years	0.023	656	70	Grand Index
13	f	13-16 years	0.024	657.772103	70	Grand Index
13	f	13-16 years	0.025	660.1533664	71	Grand Index
13	f	13-16 years	0.026	663.061014	71	Grand Index
13	f	13-16 years	0.027	666.6997505	71	Grand Index
13	f	13-16 years	0.028	670.16	71	Grand Index
13	f	13-16 years	0.029	671.88	72	Grand Index
13	f	13-16 years	0.03	673.6	72	Grand Index
13	f	13-16 years	0.031	674.66	72	Grand Index
13	f	13-16 years	0.032	675.52	72	Grand Index
13	f	13-16 years	0.033	676.2225299	72	Grand Index
13	f	13-16 years	0.034	676.7261502	73	Grand Index
13	f	13-16 years	0.035	677.2115767	73	Grand Index
13	f	13-16 years	0.036	677.5587297	73	Grand Index
13	f	13-16 years	0.037	677.9058827	73	Grand Index
13	f	13-16 years	0.038	679.3458381	73	Grand Index
13	f	13-16 years	0.039	681.0750648	74	Grand Index
13	f	13-16 years	0.04	682.2	74	Grand Index
13	f	13-16 years	0.041	682.63	74	Grand Index
13	f	13-16 years	0.042	683.06	74	Grand Index
13	f	13-16 years	0.043	683.49	74	Grand Index
13	f	13-16 years	0.044	683.92	74	Grand Index
13	f	13-16 years	0.045	685.4	75	Grand Index
13	f	13-16 years	0.046	687.12	75	Grand Index
13	f	13-16 years	0.048	688	75	Grand Index
13	f	13-16 years	0.049	688.07	75	Grand Index
13	f	13-16 years	0.05	688.5	75	Grand Index
13	f	13-16 years	0.051	688.93	75	Grand Index
13	f	13-16 years	0.052	689.72	76	Grand Index
13	f	13-16 years	0.053	690.58	76	Grand Index
13	f	13-16 years	0.054	691.22	76	Grand Index
13	f	13-16 years	0.055	691.65	76	Grand Index
13	f	13-16 years	0.056	692.08	76	Grand Index
13	f	13-16 years	0.057	692.51	76	Grand Index
13	f	13-16 years	0.058	692.94	76	Grand Index
13	f	13-16 years	0.059	693.37	77	Grand Index
13	f	13-16 years	0.06	693.8	77	Grand Index
13	f	13-16 years	0.061	694.23	77	Grand Index
13	f	13-16 years	0.062	694.66	77	Grand Index
13	f	13-16 years	0.065	695	77	Grand Index
13	f	13-16 years	0.066	695.76	77	Grand Index
13	f	13-16 years	0.067	696.62	78	Grand Index

Age	Gender	Education	Percentile	Raw Score	Norm Score	Assessment
13	f	13-16 years	0.068	697.24	78	Grand Index
13	f	13-16 years	0.069	697.67	78	Grand Index
13	f	13-16 years	0.07	698.2	78	Grand Index
13	f	13-16 years	0.071	699.06	78	Grand Index
13	f	13-16 years	0.072	699.92	78	Grand Index
13	f	13-16 years	0.073	700.39	78	Grand Index
13	f	13-16 years	0.074	700.82	78	Grand Index
13	f	13-16 years	0.075	701.25	78	Grand Index
13	f	13-16 years	0.076	701.68	79	Grand Index
13	f	13-16 years	0.079	702	79	Grand Index
13	f	13-16 years	0.08	702.4	79	Grand Index
13	f	13-16 years	0.081	702.83	79	Grand Index
13	f	13-16 years	0.082	703.26	79	Grand Index
13	f	13-16 years	0.083	703.69	79	Grand Index
13	f	13-16 years	0.084	704.12	79	Grand Index
13	f	13-16 years	0.085	704.55	79	Grand Index
13	f	13-16 years	0.086	704.98	80	Grand Index
13	f	13-16 years	0.087	705.855032	80	Grand Index
13	f	13-16 years	0.088	706.751773	80	Grand Index
13	f	13-16 years	0.089	707.3323741	80	Grand Index
13	f	13-16 years	0.09	707.7256332	80	Grand Index
13	f	13-16 years	0.093	708	80	Grand Index
13	f	13-16 years	0.094	708.8502934	80	Grand Index
13	f	13-16 years	0.095	709.7208318	80	Grand Index
13	f	13-16 years	0.096	710.2976457	80	Grand Index
13	f	13-16 years	0.097	710.7171073	81	Grand Index
13	f	13-16 years	0.098	711.42	81	Grand Index
13	f	13-16 years	0.099	712.71	81	Grand Index
13	f	13-16 years	0.1	714	81	Grand Index
13	f	13-16 years	0.101	714.43	81	Grand Index
13	f	13-16 years	0.102	714.86	81	Grand Index
13	f	13-16 years	0.103	715.87	81	Grand Index
13	f	13-16 years	0.104	717.16	81	Grand Index
13	f	13-16 years	0.105	718.15	81	Grand Index
13	f	13-16 years	0.106	718.58	81	Grand Index
13	f	13-16 years	0.107	719.01	81	Grand Index
13	f	13-16 years	0.108	719.44	81	Grand Index
13	f	13-16 years	0.109	719.87	82	Grand Index
13	f	13-16 years	0.111	720	82	Grand Index
13	f	13-16 years	0.112	720.32	82	Grand Index
13	f	13-16 years	0.113	721.18	82	Grand Index
13	f	13-16 years	0.114	722.02	82	Grand Index
13	f	13-16 years	0.115	722.45	82	Grand Index
13	f	13-16 years	0.116	722.88	82	Grand Index
13	f	13-16 years	0.117	723.31	82	Grand Index
13	f	13-16 years	0.118	723.74	82	Grand Index
13	f	13-16 years	0.119	724.0404637	82	Grand Index
13	f	13-16 years	0.12	724.142813	82	Grand Index
13	f	13-16 years	0.121	724.320881	82	Grand Index
13	f	13-16 years	0.122	725.5085317	83	Grand Index
13	f	13-16 years	0.123	726.6961824	83	Grand Index
13	f	13-16 years	0.124	727.32	83	Grand Index
13	f	13-16 years	0.125	727.75	83	Grand Index
13	f	13-16 years	0.126	728.18	83	Grand Index
13	f	13-16 years	0.127	728.61	83	Grand Index
13	f	13-16 years	0.13	729	83	Grand Index

Age	Gender	Education	Percentile	Raw Score	Norm Score	Assessment
13	f	13-16 years	0.131	729.33	83	Grand Index
13	f	13-16 years	0.132	729.76	83	Grand Index
13	f	13-16 years	0.134	730	83	Grand Index
13	f	13-16 years	0.135	730.05	83	Grand Index
13	f	13-16 years	0.136	730.48	84	Grand Index
13	f	13-16 years	0.137	730.91	84	Grand Index
13	f	13-16 years	0.138	731.68	84	Grand Index
13	f	13-16 years	0.139	732.54	84	Grand Index
13	f	13-16 years	0.141	733	84	Grand Index
13	f	13-16 years	0.142	733.06	84	Grand Index
13	f	13-16 years	0.143	733.49	84	Grand Index
13	f	13-16 years	0.144	733.92	84	Grand Index
13	f	13-16 years	0.146	734	84	Grand Index
13	f	13-16 years	0.147	734.21	84	Grand Index
13	f	13-16 years	0.148	734.64	84	Grand Index
13	f	13-16 years	0.151	735	85	Grand Index
13	f	13-16 years	0.152	735.36	85	Grand Index
13	f	13-16 years	0.153	735.79	85	Grand Index
13	f	13-16 years	0.155	736	85	Grand Index
13	f	13-16 years	0.156	736.08	85	Grand Index
13	f	13-16 years	0.157	736.51	85	Grand Index
13	f	13-16 years	0.158	736.94	85	Grand Index
13	f	13-16 years	0.159	737.37	85	Grand Index
13	f	13-16 years	0.16	737.8	85	Grand Index
13	f	13-16 years	0.162	738	85	Grand Index
13	f	13-16 years	0.163	738.09	85	Grand Index
13	f	13-16 years	0.164	738.52	85	Grand Index
13	f	13-16 years	0.165	738.95	85	Grand Index
13	f	13-16 years	0.167	739	86	Grand Index
13	f	13-16 years	0.168	739.72	86	Grand Index
13	f	13-16 years	0.169	741.01	86	Grand Index
13	f	13-16 years	0.174	742	86	Grand Index
13	f	13-16 years	0.175	742.2261905	86	Grand Index
13	f	13-16 years	0.176	742.6152381	86	Grand Index
13	f	13-16 years	0.177	742.9152381	86	Grand Index
13	f	13-16 years	0.178	742.9561905	86	Grand Index
13	f	13-16 years	0.179	742.9971429	86	Grand Index
13	f	13-16 years	0.181	743	86	Grand Index
13	f	13-16 years	0.182	743.26	86	Grand Index
13	f	13-16 years	0.183	743.69	86	Grand Index
13	f	13-16 years	0.19	744	87	Grand Index
13	f	13-16 years	0.191	744.13	87	Grand Index
13	f	13-16 years	0.192	744.56	87	Grand Index
13	f	13-16 years	0.193	744.99	87	Grand Index
13	f	13-16 years	0.194	745.42	87	Grand Index
13	f	13-16 years	0.195	745.85	87	Grand Index
13	f	13-16 years	0.196	746.56	87	Grand Index
13	f	13-16 years	0.197	747.42	87	Grand Index
13	f	13-16 years	0.204	748	88	Grand Index
13	f	13-16 years	0.205	748.15	88	Grand Index
13	f	13-16 years	0.206	748.58	88	Grand Index
13	f	13-16 years	0.207	749.01	88	Grand Index
13	f	13-16 years	0.208	749.44	88	Grand Index
13	f	13-16 years	0.209	749.87	88	Grand Index
13	f	13-16 years	0.21	750.3	88	Grand Index
13	f	13-16 years	0.211	750.73	88	Grand Index

Age	Gender	Education	Percentile	Raw Score	Norm Score	Assessment
13	f	13-16 years	0.212	751.16	88	Grand Index
13	f	13-16 years	0.213	751.59	88	Grand Index
13	f	13-16 years	0.218	752	88	Grand Index
13	f	13-16 years	0.219	752.17	88	Grand Index
13	f	13-16 years	0.22	752.6	88	Grand Index
13	f	13-16 years	0.223	753	89	Grand Index
13	f	13-16 years	0.224	753.32	89	Grand Index
13	f	13-16 years	0.225	753.75	89	Grand Index
13	f	13-16 years	0.226	754.1759042	89	Grand Index
13	f	13-16 years	0.227	754.5961198	89	Grand Index
13	f	13-16 years	0.228	754.9781557	89	Grand Index
13	f	13-16 years	0.229	754.9879401	89	Grand Index
13	f	13-16 years	0.23	754.9977246	89	Grand Index
13	f	13-16 years	0.232	755	89	Grand Index
13	f	13-16 years	0.233	755.19	89	Grand Index
13	f	13-16 years	0.234	755.62	89	Grand Index
13	f	13-16 years	0.241	756	89	Grand Index
13	f	13-16 years	0.242	756.06	90	Grand Index
13	f	13-16 years	0.243	756.49	90	Grand Index
13	f	13-16 years	0.244	756.92	90	Grand Index
13	f	13-16 years	0.245	757.2467425	90	Grand Index
13	f	13-16 years	0.246	757.5498832	90	Grand Index
13	f	13-16 years	0.247	757.766933	90	Grand Index
13	f	13-16 years	0.248	757.8937923	90	Grand Index
13	f	13-16 years	0.249	758.07	90	Grand Index
13	f	13-16 years	0.25	758.5	90	Grand Index
13	f	13-16 years	0.251	758.93	90	Grand Index
13	f	13-16 years	0.252	759.1346565	90	Grand Index
13	f	13-16 years	0.253	759.2954962	90	Grand Index
13	f	13-16 years	0.254	759.5117557	90	Grand Index
13	f	13-16 years	0.255	759.780916	90	Grand Index
13	f	13-16 years	0.262	760	90	Grand Index
13	f	13-16 years	0.263	760.36	90	Grand Index
13	f	13-16 years	0.264	762.08	91	Grand Index
13	f	13-16 years	0.265	763.8	91	Grand Index
13	f	13-16 years	0.274	764	91	Grand Index
13	f	13-16 years	0.275	764.25	91	Grand Index
13	f	13-16 years	0.276	764.68	91	Grand Index
13	f	13-16 years	0.277	765.11	91	Grand Index
13	f	13-16 years	0.278	765.54	91	Grand Index
13	f	13-16 years	0.279	765.97	91	Grand Index
13	f	13-16 years	0.281	766	91	Grand Index
13	f	13-16 years	0.282	766.1908861	91	Grand Index
13	f	13-16 years	0.283	766.5065823	91	Grand Index
13	f	13-16 years	0.284	766.7660759	91	Grand Index
13	f	13-16 years	0.285	766.8803797	91	Grand Index
13	f	13-16 years	0.286	766.9946835	92	Grand Index
13	f	13-16 years	0.288	767	92	Grand Index
13	f	13-16 years	0.289	767.1317276	92	Grand Index
13	f	13-16 years	0.29	767.341516	92	Grand Index
13	f	13-16 years	0.291	767.5544556	92	Grand Index
13	f	13-16 years	0.292	767.7746672	92	Grand Index
13	f	13-16 years	0.293	767.9948788	92	Grand Index
13	f	13-16 years	0.294	769.26	92	Grand Index
13	f	13-16 years	0.295	770.55	92	Grand Index
13	f	13-16 years	0.3	771	92	Grand Index

Age	Gender	Education	Percentile	Raw Score	Norm Score	Assessment
13	f	13-16 years	0.301	771.43	92	Grand Index
13	f	13-16 years	0.302	771.86	92	Grand Index
13	f	13-16 years	0.304	772	92	Grand Index
13	f	13-16 years	0.305	772.15	92	Grand Index
13	f	13-16 years	0.306	772.58	92	Grand Index
13	f	13-16 years	0.307	773.01	92	Grand Index
13	f	13-16 years	0.308	773.44	92	Grand Index
13	f	13-16 years	0.309	773.87	93	Grand Index
13	f	13-16 years	0.311	774	93	Grand Index
13	f	13-16 years	0.312	774.16	93	Grand Index
13	f	13-16 years	0.313	774.59	93	Grand Index
13	f	13-16 years	0.314	775.04	93	Grand Index
13	f	13-16 years	0.315	775.9	93	Grand Index
13	f	13-16 years	0.316	776.76	93	Grand Index
13	f	13-16 years	0.317	777.31	93	Grand Index
13	f	13-16 years	0.318	777.74	93	Grand Index
13	f	13-16 years	0.319	778.17	93	Grand Index
13	f	13-16 years	0.32	778.6	93	Grand Index
13	f	13-16 years	0.323	779	93	Grand Index
13	f	13-16 years	0.324	779.1654807	93	Grand Index
13	f	13-16 years	0.325	779.3878453	93	Grand Index
13	f	13-16 years	0.326	779.6040442	93	Grand Index
13	f	13-16 years	0.327	779.8116796	93	Grand Index
13	f	13-16 years	0.33	780	93	Grand Index
13	f	13-16 years	0.331	780.33	93	Grand Index
13	f	13-16 years	0.332	780.76	93	Grand Index
13	f	13-16 years	0.334	781	94	Grand Index
13	f	13-16 years	0.335	781.05	94	Grand Index
13	f	13-16 years	0.336	781.48	94	Grand Index
13	f	13-16 years	0.337	781.91	94	Grand Index
13	f	13-16 years	0.338	782.3382631	94	Grand Index
13	f	13-16 years	0.339	782.7660664	94	Grand Index
13	f	13-16 years	0.34	783.3959132	94	Grand Index
13	f	13-16 years	0.341	784.2581098	94	Grand Index
13	f	13-16 years	0.346	785	94	Grand Index
13	f	13-16 years	0.347	785.42	94	Grand Index
13	f	13-16 years	0.348	786.28	94	Grand Index
13	f	13-16 years	0.353	787	94	Grand Index
13	f	13-16 years	0.354	787.44	94	Grand Index
13	f	13-16 years	0.355	788.3	94	Grand Index
13	f	13-16 years	0.358	789	95	Grand Index
13	f	13-16 years	0.359	789.37	95	Grand Index
13	f	13-16 years	0.36	789.8	95	Grand Index
13	f	13-16 years	0.361	790.0445091	95	Grand Index
13	f	13-16 years	0.362	790.1277216	95	Grand Index
13	f	13-16 years	0.363	790.266101	95	Grand Index
13	f	13-16 years	0.364	790.6128885	95	Grand Index
13	f	13-16 years	0.365	790.9596759	95	Grand Index
13	f	13-16 years	0.367	791	95	Grand Index
13	f	13-16 years	0.368	791.48	95	Grand Index
13	f	13-16 years	0.369	792.34	95	Grand Index
13	f	13-16 years	0.379	793	95	Grand Index
13	f	13-16 years	0.38	793.4	95	Grand Index
13	f	13-16 years	0.381	793.83	95	Grand Index
13	f	13-16 years	0.388	794	96	Grand Index
13	f	13-16 years	0.389	794.27	96	Grand Index

Age	Gender	Education	Percentile	Raw Score	Norm Score	Assessment
13	f	13-16 years	0.39	794.7	96	Grand Index
13	f	13-16 years	0.393	795	96	Grand Index
13	f	13-16 years	0.394	795.375	96	Grand Index
13	f	13-16 years	0.395	795.7589286	96	Grand Index
13	f	13-16 years	0.396	795.9228571	96	Grand Index
13	f	13-16 years	0.397	795.9689286	96	Grand Index
13	f	13-16 years	0.404	796	96	Grand Index
13	f	13-16 years	0.405	796.15	96	Grand Index
13	f	13-16 years	0.406	796.58	96	Grand Index
13	f	13-16 years	0.413	797	97	Grand Index
13	f	13-16 years	0.414	797.02	97	Grand Index
13	f	13-16 years	0.415	797.45	97	Grand Index
13	f	13-16 years	0.416	797.88	97	Grand Index
13	f	13-16 years	0.417	798.0697353	97	Grand Index
13	f	13-16 years	0.418	798.166465	97	Grand Index
13	f	13-16 years	0.419	798.3567108	97	Grand Index
13	f	13-16 years	0.42	798.6899811	97	Grand Index
13	f	13-16 years	0.421	799.03	97	Grand Index
13	f	13-16 years	0.422	799.46	97	Grand Index
13	f	13-16 years	0.423	799.89	97	Grand Index
13	f	13-16 years	0.43	800	97	Grand Index
13	f	13-16 years	0.431	800.33	97	Grand Index
13	f	13-16 years	0.432	800.76	97	Grand Index
13	f	13-16 years	0.434	801	98	Grand Index
13	f	13-16 years	0.435	801.05	98	Grand Index
13	f	13-16 years	0.436	801.48	98	Grand Index
13	f	13-16 years	0.437	801.91	98	Grand Index
13	f	13-16 years	0.438	802.34	98	Grand Index
13	f	13-16 years	0.439	802.77	98	Grand Index
13	f	13-16 years	0.441	803	98	Grand Index
13	f	13-16 years	0.442	803.06	98	Grand Index
13	f	13-16 years	0.443	803.49	98	Grand Index
13	f	13-16 years	0.444	803.92	98	Grand Index
13	f	13-16 years	0.446	804	98	Grand Index
13	f	13-16 years	0.447	804.21	98	Grand Index
13	f	13-16 years	0.448	804.64	98	Grand Index
13	f	13-16 years	0.455	805	98	Grand Index
13	f	13-16 years	0.456	805.08	98	Grand Index
13	f	13-16 years	0.457	805.51	98	Grand Index
13	f	13-16 years	0.458	805.94	98	Grand Index
13	f	13-16 years	0.462	806	99	Grand Index
13	f	13-16 years	0.463	806.18	99	Grand Index
13	f	13-16 years	0.464	807.04	99	Grand Index
13	f	13-16 years	0.465	807.9	99	Grand Index
13	f	13-16 years	0.466	808.1560066	99	Grand Index
13	f	13-16 years	0.467	808.3325405	99	Grand Index
13	f	13-16 years	0.468	808.5114856	99	Grand Index
13	f	13-16 years	0.469	808.6923396	99	Grand Index
13	f	13-16 years	0.47	808.8480211	99	Grand Index
13	f	13-16 years	0.471	808.9206332	99	Grand Index
13	f	13-16 years	0.472	808.9932454	99	Grand Index
13	f	13-16 years	0.476	809	99	Grand Index
13	f	13-16 years	0.477	809.11	99	Grand Index
13	f	13-16 years	0.478	809.54	99	Grand Index
13	f	13-16 years	0.479	809.97	99	Grand Index
13	f	13-16 years	0.48	810.4	99	Grand Index

Age	Gender	Education	Percentile	Raw Score	Norm Score	Assessment
13	f	13-16 years	0.481	810.83	99	Grand Index
13	f	13-16 years	0.482	811.2389883	99	Grand Index
13	f	13-16 years	0.483	811.6342383	99	Grand Index
13	f	13-16 years	0.484	811.9288836	99	Grand Index
13	f	13-16 years	0.485	811.9636336	99	Grand Index
13	f	13-16 years	0.486	811.9983837	99	Grand Index
13	f	13-16 years	0.49	812	100	Grand Index
13	f	13-16 years	0.491	812.26	100	Grand Index
13	f	13-16 years	0.492	813.12	100	Grand Index
13	f	13-16 years	0.493	813.98	100	Grand Index
13	f	13-16 years	0.495	814	100	Grand Index
13	f	13-16 years	0.496	814.28	100	Grand Index
13	f	13-16 years	0.497	814.71	100	Grand Index
13	f	13-16 years	0.5	815	100	Grand Index
13	f	13-16 years	0.501	815.4265227	100	Grand Index
13	f	13-16 years	0.502	815.8530453	100	Grand Index
13	f	13-16 years	0.503	815.9942583	100	Grand Index
13	f	13-16 years	0.504	815.9977357	100	Grand Index
13	f	13-16 years	0.506	816	100	Grand Index
13	f	13-16 years	0.507	816.01	100	Grand Index
13	f	13-16 years	0.508	816.44	100	Grand Index
13	f	13-16 years	0.509	816.87	100	Grand Index
13	f	13-16 years	0.511	817	100	Grand Index
13	f	13-16 years	0.512	817.0673171	100	Grand Index
13	f	13-16 years	0.513	817.2482317	100	Grand Index
13	f	13-16 years	0.514	817.4523171	101	Grand Index
13	f	13-16 years	0.515	818.1314024	101	Grand Index
13	f	13-16 years	0.516	818.8104878	101	Grand Index
13	f	13-16 years	0.525	819	101	Grand Index
13	f	13-16 years	0.526	819.18	101	Grand Index
13	f	13-16 years	0.527	819.61	101	Grand Index
13	f	13-16 years	0.53	820	101	Grand Index
13	f	13-16 years	0.531	820.66	101	Grand Index
13	f	13-16 years	0.532	821.52	101	Grand Index
13	f	13-16 years	0.544	822	102	Grand Index
13	f	13-16 years	0.545	822.35	102	Grand Index
13	f	13-16 years	0.546	822.78	102	Grand Index
13	f	13-16 years	0.551	823	102	Grand Index
13	f	13-16 years	0.552	823.36	102	Grand Index
13	f	13-16 years	0.553	823.79	102	Grand Index
13	f	13-16 years	0.56	824	102	Grand Index
13	f	13-16 years	0.561	824.1303241	102	Grand Index
13	f	13-16 years	0.562	824.3739736	102	Grand Index
13	f	13-16 years	0.563	824.6056303	102	Grand Index
13	f	13-16 years	0.564	824.7919808	102	Grand Index
13	f	13-16 years	0.565	824.9783313	102	Grand Index
13	f	13-16 years	0.567	825	103	Grand Index
13	f	13-16 years	0.568	825.24	103	Grand Index
13	f	13-16 years	0.569	825.67	103	Grand Index
13	f	13-16 years	0.572	826	103	Grand Index
13	f	13-16 years	0.573	826.39	103	Grand Index
13	f	13-16 years	0.574	826.82	103	Grand Index
13	f	13-16 years	0.575	827.25	103	Grand Index
13	f	13-16 years	0.576	827.68	103	Grand Index
13	f	13-16 years	0.583	828	103	Grand Index
13	f	13-16 years	0.584	828.0056576	103	Grand Index

Age	Gender	Education	Percentile	Raw Score	Norm Score	Assessment
13	f	13-16 years	0.585	828.0259305	103	Grand Index
13	f	13-16 years	0.586	828.0462035	103	Grand Index
13	f	13-16 years	0.587	828.4378164	103	Grand Index
13	f	13-16 years	0.588	828.8475434	103	Grand Index
13	f	13-16 years	0.589	829.54	103	Grand Index
13	f	13-16 years	0.59	830.4	103	Grand Index
13	f	13-16 years	0.595	831	104	Grand Index
13	f	13-16 years	0.596	831.28	104	Grand Index
13	f	13-16 years	0.597	831.71	104	Grand Index
13	f	13-16 years	0.598	832.0188785	104	Grand Index
13	f	13-16 years	0.599	832.0768627	104	Grand Index
13	f	13-16 years	0.6	832.1348468	104	Grand Index
13	f	13-16 years	0.601	832.5068627	104	Grand Index
13	f	13-16 years	0.602	832.8788785	104	Grand Index
13	f	13-16 years	0.603	833.29	104	Grand Index
13	f	13-16 years	0.604	833.72	104	Grand Index
13	f	13-16 years	0.611	834	104	Grand Index
13	f	13-16 years	0.612	834.16	104	Grand Index
13	f	13-16 years	0.613	834.59	104	Grand Index
13	f	13-16 years	0.618	835	105	Grand Index
13	f	13-16 years	0.619	835.1694642	105	Grand Index
13	f	13-16 years	0.62	835.598109	105	Grand Index
13	f	13-16 years	0.621	835.9969428	105	Grand Index
13	f	13-16 years	0.622	835.9982981	105	Grand Index
13	f	13-16 years	0.623	835.9996533	105	Grand Index
13	f	13-16 years	0.627	836	105	Grand Index
13	f	13-16 years	0.628	836.04	105	Grand Index
13	f	13-16 years	0.629	836.47	105	Grand Index
13	f	13-16 years	0.63	836.9	105	Grand Index
13	f	13-16 years	0.637	837	105	Grand Index
13	f	13-16 years	0.638	837.34	105	Grand Index
13	f	13-16 years	0.639	837.77	105	Grand Index
13	f	13-16 years	0.64	838.0164118	105	Grand Index
13	f	13-16 years	0.641	838.0516971	105	Grand Index
13	f	13-16 years	0.642	838.1371354	105	Grand Index
13	f	13-16 years	0.643	838.5318501	105	Grand Index
13	f	13-16 years	0.644	838.9265647	106	Grand Index
13	f	13-16 years	0.651	839	106	Grand Index
13	f	13-16 years	0.652	839.36	106	Grand Index
13	f	13-16 years	0.653	839.79	106	Grand Index
13	f	13-16 years	0.655	840	106	Grand Index
13	f	13-16 years	0.656	840.08	106	Grand Index
13	f	13-16 years	0.657	840.51	106	Grand Index
13	f	13-16 years	0.658	840.94	106	Grand Index
13	f	13-16 years	0.66	841	106	Grand Index
13	f	13-16 years	0.661	841.23	106	Grand Index
13	f	13-16 years	0.662	841.66	106	Grand Index
13	f	13-16 years	0.663	842.09	106	Grand Index
13	f	13-16 years	0.664	842.52	106	Grand Index
13	f	13-16 years	0.665	842.95	106	Grand Index
13	f	13-16 years	0.667	843	106	Grand Index
13	f	13-16 years	0.668	843.24	107	Grand Index
13	f	13-16 years	0.669	843.67	107	Grand Index
13	f	13-16 years	0.676	844	107	Grand Index
13	f	13-16 years	0.677	844.44	107	Grand Index
13	f	13-16 years	0.678	846.16	107	Grand Index

Age	Gender	Education	Percentile	Raw Score	Norm Score	Assessment
13	f	13-16 years	0.679	847.88	107	Grand Index
13	f	13-16 years	0.683	848	107	Grand Index
13	f	13-16 years	0.684	848.12	107	Grand Index
13	f	13-16 years	0.685	848.55	107	Grand Index
13	f	13-16 years	0.686	848.98	107	Grand Index
13	f	13-16 years	0.695	849	108	Grand Index
13	f	13-16 years	0.696	849.56	108	Grand Index
13	f	13-16 years	0.697	850.42	108	Grand Index
13	f	13-16 years	0.7	851	108	Grand Index
13	f	13-16 years	0.701	851.43	108	Grand Index
13	f	13-16 years	0.702	851.86	108	Grand Index
13	f	13-16 years	0.703	852.29	108	Grand Index
13	f	13-16 years	0.704	852.72	108	Grand Index
13	f	13-16 years	0.706	853	108	Grand Index
13	f	13-16 years	0.707	853.0016409	108	Grand Index
13	f	13-16 years	0.708	853.0721998	108	Grand Index
13	f	13-16 years	0.709	853.1427586	108	Grand Index
13	f	13-16 years	0.71	853.4148633	108	Grand Index
13	f	13-16 years	0.711	853.7743044	108	Grand Index
13	f	13-16 years	0.713	854	108	Grand Index
13	f	13-16 years	0.714	854.04	108	Grand Index
13	f	13-16 years	0.715	854.9	109	Grand Index
13	f	13-16 years	0.716	855.76	109	Grand Index
13	f	13-16 years	0.72	856	109	Grand Index
13	f	13-16 years	0.721	856.03	109	Grand Index
13	f	13-16 years	0.722	856.46	109	Grand Index
13	f	13-16 years	0.723	856.89	109	Grand Index
13	f	13-16 years	0.725	857	109	Grand Index
13	f	13-16 years	0.726	857.0875472	109	Grand Index
13	f	13-16 years	0.727	857.2966876	109	Grand Index
13	f	13-16 years	0.728	857.5069182	109	Grand Index
13	f	13-16 years	0.729	857.7277778	109	Grand Index
13	f	13-16 years	0.73	857.9486373	109	Grand Index
13	f	13-16 years	0.731	858.66	109	Grand Index
13	f	13-16 years	0.732	859.52	109	Grand Index
13	f	13-16 years	0.733	860.19	109	Grand Index
13	f	13-16 years	0.734	860.62	109	Grand Index
13	f	13-16 years	0.737	861	110	Grand Index
13	f	13-16 years	0.738	861.34	110	Grand Index
13	f	13-16 years	0.739	861.77	110	Grand Index
13	f	13-16 years	0.74	862.2	110	Grand Index
13	f	13-16 years	0.741	862.63	110	Grand Index
13	f	13-16 years	0.742	863.12	110	Grand Index
13	f	13-16 years	0.743	863.98	110	Grand Index
13	f	13-16 years	0.744	864.84	110	Grand Index
13	f	13-16 years	0.745	865.7	110	Grand Index
13	f	13-16 years	0.746	866.56	110	Grand Index
13	f	13-16 years	0.748	867	110	Grand Index
13	f	13-16 years	0.749	867.0031818	110	Grand Index
13	f	13-16 years	0.75	867.0227273	110	Grand Index
13	f	13-16 years	0.751	867.0422727	110	Grand Index
13	f	13-16 years	0.752	867.2804117	110	Grand Index
13	f	13-16 years	0.753	867.5610549	110	Grand Index
13	f	13-16 years	0.754	867.7645283	110	Grand Index
13	f	13-16 years	0.755	867.8943396	110	Grand Index
13	f	13-16 years	0.76	868	111	Grand Index

Age	Gender	Education	Percentile	Raw Score	Norm Score	Assessment
13	f	13-16 years	0.761	868.69	111	Grand Index
13	f	13-16 years	0.762	869.98	111	Grand Index
13	f	13-16 years	0.765	871	111	Grand Index
13	f	13-16 years	0.766	871.76	111	Grand Index
13	f	13-16 years	0.767	872.62	111	Grand Index
13	f	13-16 years	0.768	873.24	111	Grand Index
13	f	13-16 years	0.769	873.67	111	Grand Index
13	f	13-16 years	0.772	874	111	Grand Index
13	f	13-16 years	0.773	874.39	111	Grand Index
13	f	13-16 years	0.774	874.82	111	Grand Index
13	f	13-16 years	0.779	875	112	Grand Index
13	f	13-16 years	0.78	875.4	112	Grand Index
13	f	13-16 years	0.781	875.83	112	Grand Index
13	f	13-16 years	0.783	876	112	Grand Index
13	f	13-16 years	0.784	876.12	112	Grand Index
13	f	13-16 years	0.785	876.55	112	Grand Index
13	f	13-16 years	0.786	876.98	112	Grand Index
13	f	13-16 years	0.79	877	112	Grand Index
13	f	13-16 years	0.791	877.13	112	Grand Index
13	f	13-16 years	0.792	877.56	112	Grand Index
13	f	13-16 years	0.793	877.99	112	Grand Index
13	f	13-16 years	0.795	878	112	Grand Index
13	f	13-16 years	0.796	878.28	112	Grand Index
13	f	13-16 years	0.797	878.71	112	Grand Index
13	f	13-16 years	0.798	879.14	113	Grand Index
13	f	13-16 years	0.799	879.57	113	Grand Index
13	f	13-16 years	0.8	880	113	Grand Index
13	f	13-16 years	0.801	880.43	113	Grand Index
13	f	13-16 years	0.802	880.86	113	Grand Index
13	f	13-16 years	0.803	881.29	113	Grand Index
13	f	13-16 years	0.804	881.72	113	Grand Index
13	f	13-16 years	0.811	882	113	Grand Index
13	f	13-16 years	0.812	882.32	113	Grand Index
13	f	13-16 years	0.813	883.18	113	Grand Index
13	f	13-16 years	0.816	884	114	Grand Index
13	f	13-16 years	0.817	884.31	114	Grand Index
13	f	13-16 years	0.818	884.74	114	Grand Index
13	f	13-16 years	0.823	885	114	Grand Index
13	f	13-16 years	0.824	885.32	114	Grand Index
13	f	13-16 years	0.825	885.75	114	Grand Index
13	f	13-16 years	0.827	886	114	Grand Index
13	f	13-16 years	0.828	886.060316	114	Grand Index
13	f	13-16 years	0.829	886.7087128	114	Grand Index
13	f	13-16 years	0.83	887.3571097	114	Grand Index
13	f	13-16 years	0.831	887.6702928	114	Grand Index
13	f	13-16 years	0.832	887.8818959	114	Grand Index
13	f	13-16 years	0.834	888	115	Grand Index
13	f	13-16 years	0.835	888.15	115	Grand Index
13	f	13-16 years	0.836	889.44	115	Grand Index
13	f	13-16 years	0.837	890.73	115	Grand Index
13	f	13-16 years	0.838	891.68	115	Grand Index
13	f	13-16 years	0.839	892.54	115	Grand Index
13	f	13-16 years	0.84	893.0061572	115	Grand Index
13	f	13-16 years	0.841	893.0193951	115	Grand Index
13	f	13-16 years	0.842	893.0889387	115	Grand Index
13	f	13-16 years	0.843	893.5057008	115	Grand Index

Age	Gender	Education	Percentile	Raw Score	Norm Score	Assessment
13	f	13-16 years	0.844	893.9224629	115	Grand Index
13	f	13-16 years	0.846	894	115	Grand Index
13	f	13-16 years	0.847	894.21	115	Grand Index
13	f	13-16 years	0.848	894.64	115	Grand Index
13	f	13-16 years	0.849	895.07	115	Grand Index
13	f	13-16 years	0.85	895.5	116	Grand Index
13	f	13-16 years	0.851	895.93	116	Grand Index
13	f	13-16 years	0.853	896	116	Grand Index
13	f	13-16 years	0.854	896.22	116	Grand Index
13	f	13-16 years	0.855	896.65	116	Grand Index
13	f	13-16 years	0.856	897.08	116	Grand Index
13	f	13-16 years	0.857	897.51	116	Grand Index
13	f	13-16 years	0.858	897.94	116	Grand Index
13	f	13-16 years	0.86	898	116	Grand Index
13	f	13-16 years	0.861	898.23	116	Grand Index
13	f	13-16 years	0.862	898.66	116	Grand Index
13	f	13-16 years	0.863	899.09	116	Grand Index
13	f	13-16 years	0.864	899.52	116	Grand Index
13	f	13-16 years	0.865	899.95	117	Grand Index
13	f	13-16 years	0.866	900.0021271	117	Grand Index
13	f	13-16 years	0.867	900.0045341	117	Grand Index
13	f	13-16 years	0.868	900.2421411	117	Grand Index
13	f	13-16 years	0.869	900.6659479	117	Grand Index
13	f	13-16 years	0.87	900.9920755	117	Grand Index
13	f	13-16 years	0.871	900.9958616	117	Grand Index
13	f	13-16 years	0.872	900.9996478	117	Grand Index
13	f	13-16 years	0.874	901	117	Grand Index
13	f	13-16 years	0.875	901.5	117	Grand Index
13	f	13-16 years	0.876	902.36	117	Grand Index
13	f	13-16 years	0.881	903	118	Grand Index
13	f	13-16 years	0.882	903.52	118	Grand Index
13	f	13-16 years	0.883	904.38	118	Grand Index
13	f	13-16 years	0.884	905.12	118	Grand Index
13	f	13-16 years	0.885	905.55	118	Grand Index
13	f	13-16 years	0.886	905.98	118	Grand Index
13	f	13-16 years	0.887	906.41	118	Grand Index
13	f	13-16 years	0.888	906.84	118	Grand Index
13	f	13-16 years	0.89	907	118	Grand Index
13	f	13-16 years	0.891	907.0453488	118	Grand Index
13	f	13-16 years	0.892	907.1953488	119	Grand Index
13	f	13-16 years	0.893	907.3453488	119	Grand Index
13	f	13-16 years	0.894	908.0423256	119	Grand Index
13	f	13-16 years	0.895	908.7523256	119	Grand Index
13	f	13-16 years	0.896	909.28	119	Grand Index
13	f	13-16 years	0.897	909.71	119	Grand Index
13	f	13-16 years	0.898	910.14	119	Grand Index
13	f	13-16 years	0.899	910.57	119	Grand Index
13	f	13-16 years	0.902	911	119	Grand Index
13	f	13-16 years	0.903	911.29	119	Grand Index
13	f	13-16 years	0.904	911.72	120	Grand Index
13	f	13-16 years	0.905	912.3	120	Grand Index
13	f	13-16 years	0.906	913.16	120	Grand Index
13	f	13-16 years	0.911	914	120	Grand Index
13	f	13-16 years	0.912	914.16	120	Grand Index
13	f	13-16 years	0.913	914.59	120	Grand Index
13	f	13-16 years	0.916	915	121	Grand Index

Age	Gender	Education	Percentile	Raw Score	Norm Score	Assessment
13	f	13-16 years	0.917	916.55	121	Grand Index
13	f	13-16 years	0.918	918.7	121	Grand Index
13	f	13-16 years	0.919	920.34	121	Grand Index
13	f	13-16 years	0.92	921.2	121	Grand Index
13	f	13-16 years	0.923	922	121	Grand Index
13	f	13-16 years	0.924	922.32	121	Grand Index
13	f	13-16 years	0.925	922.75	122	Grand Index
13	f	13-16 years	0.926	923.2956075	122	Grand Index
13	f	13-16 years	0.927	924.0017811	122	Grand Index
13	f	13-16 years	0.928	924.6565736	122	Grand Index
13	f	13-16 years	0.929	924.8104	122	Grand Index
13	f	13-16 years	0.93	924.9642264	122	Grand Index
13	f	13-16 years	0.931	925.33	122	Grand Index
13	f	13-16 years	0.932	925.76	122	Grand Index
13	f	13-16 years	0.933	926.205	122	Grand Index
13	f	13-16 years	0.934	926.6689474	123	Grand Index
13	f	13-16 years	0.935	927.0924419	123	Grand Index
13	f	13-16 years	0.936	927.2084945	123	Grand Index
13	f	13-16 years	0.937	927.3245471	123	Grand Index
13	f	13-16 years	0.938	927.6220984	123	Grand Index
13	f	13-16 years	0.939	927.9676934	123	Grand Index
13	f	13-16 years	0.94	928.1650865	123	Grand Index
13	f	13-16 years	0.941	928.1920476	123	Grand Index
13	f	13-16 years	0.942	928.2623318	124	Grand Index
13	f	13-16 years	0.943	928.5997758	124	Grand Index
13	f	13-16 years	0.944	928.9372197	124	Grand Index
13	f	13-16 years	0.945	929.7	124	Grand Index
13	f	13-16 years	0.946	930.56	124	Grand Index
13	f	13-16 years	0.947	931.21	124	Grand Index
13	f	13-16 years	0.948	931.64	124	Grand Index
13	f	13-16 years	0.949	932.07	125	Grand Index
13	f	13-16 years	0.95	932.5	125	Grand Index
13	f	13-16 years	0.951	932.93	125	Grand Index
13	f	13-16 years	0.952	933.72	125	Grand Index
13	f	13-16 years	0.953	934.58	125	Grand Index
13	f	13-16 years	0.954	935.22	125	Grand Index
13	f	13-16 years	0.955	935.65	125	Grand Index
13	f	13-16 years	0.956	936.24	126	Grand Index
13	f	13-16 years	0.957	937.53	126	Grand Index
13	f	13-16 years	0.958	938.82	126	Grand Index
13	f	13-16 years	0.959	939.171617	126	Grand Index
13	f	13-16 years	0.96	939.3710638	126	Grand Index
13	f	13-16 years	0.961	939.8237809	126	Grand Index
13	f	13-16 years	0.962	940.496733	127	Grand Index
13	f	13-16 years	0.963	941.1162395	127	Grand Index
13	f	13-16 years	0.964	941.5338406	127	Grand Index
13	f	13-16 years	0.965	941.9514417	127	Grand Index
13	f	13-16 years	0.966	943.9066667	127	Grand Index
13	f	13-16 years	0.967	946.0642105	128	Grand Index
13	f	13-16 years	0.968	947.2533333	128	Grand Index
13	f	13-16 years	0.969	947.6757895	128	Grand Index
13	f	13-16 years	0.97	948.3	128	Grand Index
13	f	13-16 years	0.971	949.59	128	Grand Index
13	f	13-16 years	0.972	950.88	129	Grand Index
13	f	13-16 years	0.973	955.29	129	Grand Index
13	f	13-16 years	0.974	960.02	129	Grand Index

Age	Gender	Education	Percentile	Raw Score	Norm Score	Assessment
13	f	13-16 years	0.975	963.2878788	129	Grand Index
13	f	13-16 years	0.976	965.5030303	130	Grand Index
13	f	13-16 years	0.977	967.3548485	130	Grand Index
13	f	13-16 years	0.978	968.149697	130	Grand Index
13	f	13-16 years	0.979	968.9445455	131	Grand Index
13	f	13-16 years	0.98	969.0093023	131	Grand Index
13	f	13-16 years	0.981	969.0193023	131	Grand Index
13	f	13-16 years	0.982	969.5372093	131	Grand Index
13	f	13-16 years	0.983	970.3872093	132	Grand Index
13	f	13-16 years	0.984	971.5671677	132	Grand Index
13	f	13-16 years	0.985	973.5995188	133	Grand Index
13	f	13-16 years	0.986	975.6318699	133	Grand Index
13	f	13-16 years	0.987	976.2485747	133	Grand Index
13	f	13-16 years	0.988	976.7962237	134	Grand Index
13	f	13-16 years	0.989	980.78	134	Grand Index
13	f	13-16 years	0.99	986.8	135	Grand Index
13	f	13-16 years	0.991	992.04	135	Grand Index
13	f	13-16 years	0.992	995.48	136	Grand Index
13	f	13-16 years	0.993	998.92	137	Grand Index
13	f	13-16 years	0.994	999.42	138	Grand Index
13	f	13-16 years	0.995	999.85	139	Grand Index
13	f	13-16 years	0.996	1000.424828	140	Grand Index
13	f	13-16 years	0.997	1001.077241	141	Grand Index
13	f	13-16 years	0.998	1002.004828	143	Grand Index
13	f	13-16 years	0.999	1003.502414	146	Grand Index
13	m	13-16 years	0.001	544.9186774	54	Grand Index
13	m	13-16 years	0.002	558.3844795	57	Grand Index
13	m	13-16 years	0.003	576.6298382	59	Grand Index
13	m	13-16 years	0.004	586.614351	60	Grand Index
13	m	13-16 years	0.005	594.6100427	61	Grand Index
13	m	13-16 years	0.006	602.1971278	62	Grand Index
13	m	13-16 years	0.007	607.3519426	63	Grand Index
13	m	13-16 years	0.008	611.0901444	64	Grand Index
13	m	13-16 years	0.009	615.0385774	65	Grand Index
13	m	13-16 years	0.01	617.5761033	65	Grand Index
13	m	13-16 years	0.011	618.3600735	66	Grand Index
13	m	13-16 years	0.012	626.7963641	66	Grand Index
13	m	13-16 years	0.013	635.312	67	Grand Index
13	m	13-16 years	0.014	640.336	67	Grand Index
13	m	13-16 years	0.015	643.68	67	Grand Index
13	m	13-16 years	0.016	646.096	68	Grand Index
13	m	13-16 years	0.017	647.352	68	Grand Index
13	m	13-16 years	0.018	648.0212391	69	Grand Index
13	m	13-16 years	0.019	648.0651146	69	Grand Index
13	m	13-16 years	0.02	650.8307408	69	Grand Index
13	m	13-16 years	0.021	653.188	69	Grand Index
13	m	13-16 years	0.022	653.816	70	Grand Index
13	m	13-16 years	0.023	654.444	70	Grand Index
13	m	13-16 years	0.024	655.3580524	70	Grand Index
13	m	13-16 years	0.025	658.4810647	71	Grand Index
13	m	13-16 years	0.026	660.9493093	71	Grand Index
13	m	13-16 years	0.027	662.8186811	71	Grand Index
13	m	13-16 years	0.028	664.147057	71	Grand Index
13	m	13-16 years	0.029	666.272	72	Grand Index
13	m	13-16 years	0.03	670.04	72	Grand Index
13	m	13-16 years	0.031	671.468	72	Grand Index

Age	Gender	Education	Percentile	Raw Score	Norm Score	Assessment
13	m	13-16 years	0.032	672.096	72	Grand Index
13	m	13-16 years	0.033	672.724	72	Grand Index
13	m	13-16 years	0.035	673	73	Grand Index
13	m	13-16 years	0.036	676.04	73	Grand Index
13	m	13-16 years	0.038	678	73	Grand Index
13	m	13-16 years	0.039	678.492	74	Grand Index
13	m	13-16 years	0.04	679.2085963	74	Grand Index
13	m	13-16 years	0.041	680.3002504	74	Grand Index
13	m	13-16 years	0.042	681.2173726	74	Grand Index
13	m	13-16 years	0.043	682.0323752	74	Grand Index
13	m	13-16 years	0.044	685.1645723	74	Grand Index
13	m	13-16 years	0.045	687.78	75	Grand Index
13	m	13-16 years	0.046	689.664	75	Grand Index
13	m	13-16 years	0.047	690	75	Grand Index
13	m	13-16 years	0.048	690.288	75	Grand Index
13	m	13-16 years	0.049	691.544	75	Grand Index
13	m	13-16 years	0.05	692.8	75	Grand Index
13	m	13-16 years	0.052	694	76	Grand Index
13	m	13-16 years	0.053	694.568	76	Grand Index
13	m	13-16 years	0.054	695.824	76	Grand Index
13	m	13-16 years	0.055	696.4438054	76	Grand Index
13	m	13-16 years	0.056	696.851789	76	Grand Index
13	m	13-16 years	0.057	696.9636598	76	Grand Index
13	m	13-16 years	0.058	697.424	76	Grand Index
13	m	13-16 years	0.059	698.1266452	77	Grand Index
13	m	13-16 years	0.06	699.656129	77	Grand Index
13	m	13-16 years	0.061	701.2253548	77	Grand Index
13	m	13-16 years	0.062	702.835871	77	Grand Index
13	m	13-16 years	0.065	703	77	Grand Index
13	m	13-16 years	0.066	703.896	77	Grand Index
13	m	13-16 years	0.068	705	78	Grand Index
13	m	13-16 years	0.069	705.332	78	Grand Index
13	m	13-16 years	0.07	705.96	78	Grand Index
13	m	13-16 years	0.071	706.588	78	Grand Index
13	m	13-16 years	0.072	707.0383499	78	Grand Index
13	m	13-16 years	0.073	707.1498486	78	Grand Index
13	m	13-16 years	0.074	707.5430374	78	Grand Index
13	m	13-16 years	0.075	708.0567033	78	Grand Index
13	m	13-16 years	0.076	708.7149148	79	Grand Index
13	m	13-16 years	0.077	709.3791288	79	Grand Index
13	m	13-16 years	0.078	710.0479291	79	Grand Index
13	m	13-16 years	0.079	710.1059755	79	Grand Index
13	m	13-16 years	0.08	711.3701085	79	Grand Index
13	m	13-16 years	0.081	714.6098954	79	Grand Index
13	m	13-16 years	0.082	716.1385983	79	Grand Index
13	m	13-16 years	0.083	717.248	79	Grand Index
13	m	13-16 years	0.084	718.504	79	Grand Index
13	m	13-16 years	0.087	719	80	Grand Index
13	m	13-16 years	0.088	719.3715919	80	Grand Index
13	m	13-16 years	0.089	720.2555302	80	Grand Index
13	m	13-16 years	0.09	720.7156217	80	Grand Index
13	m	13-16 years	0.092	721	80	Grand Index
13	m	13-16 years	0.093	721.404	80	Grand Index
13	m	13-16 years	0.095	722	80	Grand Index
13	m	13-16 years	0.096	722.288	80	Grand Index
13	m	13-16 years	0.097	722.916	81	Grand Index

Age	Gender	Education	Percentile	Raw Score	Norm Score	Assessment
13	m	13-16 years	0.098	723	81	Grand Index
13	m	13-16 years	0.099	723.172	81	Grand Index
13	m	13-16 years	0.1	723.8	81	Grand Index
13	m	13-16 years	0.101	724.3804872	81	Grand Index
13	m	13-16 years	0.102	724.8952054	81	Grand Index
13	m	13-16 years	0.103	724.9649204	81	Grand Index
13	m	13-16 years	0.105	725	81	Grand Index
13	m	13-16 years	0.106	725.568	81	Grand Index
13	m	13-16 years	0.107	726.0037679	81	Grand Index
13	m	13-16 years	0.108	726.0158404	81	Grand Index
13	m	13-16 years	0.109	726.0201054	82	Grand Index
13	m	13-16 years	0.11	726.0994803	82	Grand Index
13	m	13-16 years	0.111	726.7141829	82	Grand Index
13	m	13-16 years	0.114	727	82	Grand Index
13	m	13-16 years	0.115	727.22	82	Grand Index
13	m	13-16 years	0.116	727.848	82	Grand Index
13	m	13-16 years	0.121	728	82	Grand Index
13	m	13-16 years	0.122	729.232	83	Grand Index
13	m	13-16 years	0.123	730.244	83	Grand Index
13	m	13-16 years	0.124	730.872	83	Grand Index
13	m	13-16 years	0.125	731.1904177	83	Grand Index
13	m	13-16 years	0.126	731.4600885	83	Grand Index
13	m	13-16 years	0.127	731.8489238	83	Grand Index
13	m	13-16 years	0.128	732.384	83	Grand Index
13	m	13-16 years	0.129	733.012	83	Grand Index
13	m	13-16 years	0.13	733.64	83	Grand Index
13	m	13-16 years	0.135	734	83	Grand Index
13	m	13-16 years	0.136	734.3399807	84	Grand Index
13	m	13-16 years	0.137	734.8392876	84	Grand Index
13	m	13-16 years	0.138	734.9439841	84	Grand Index
13	m	13-16 years	0.14	735	84	Grand Index
13	m	13-16 years	0.141	735.548	84	Grand Index
13	m	13-16 years	0.144	736	84	Grand Index
13	m	13-16 years	0.145	736.06	84	Grand Index
13	m	13-16 years	0.146	736.688	84	Grand Index
13	m	13-16 years	0.147	737.0631379	84	Grand Index
13	m	13-16 years	0.148	737.1886144	84	Grand Index
13	m	13-16 years	0.149	737.6575159	84	Grand Index
13	m	13-16 years	0.152	738	85	Grand Index
13	m	13-16 years	0.153	738.084	85	Grand Index
13	m	13-16 years	0.154	738.712	85	Grand Index
13	m	13-16 years	0.155	739.34	85	Grand Index
13	m	13-16 years	0.156	739.968	85	Grand Index
13	m	13-16 years	0.157	740	85	Grand Index
13	m	13-16 years	0.158	740.224	85	Grand Index
13	m	13-16 years	0.159	740.852	85	Grand Index
13	m	13-16 years	0.16	741	85	Grand Index
13	m	13-16 years	0.161	741.108	85	Grand Index
13	m	13-16 years	0.162	741.736	85	Grand Index
13	m	13-16 years	0.164	742	85	Grand Index
13	m	13-16 years	0.165	742.6000416	85	Grand Index
13	m	13-16 years	0.166	742.9757924	85	Grand Index
13	m	13-16 years	0.167	742.9960083	86	Grand Index
13	m	13-16 years	0.168	743	86	Grand Index
13	m	13-16 years	0.169	743.0334599	86	Grand Index
13	m	13-16 years	0.17	743.192648	86	Grand Index

Age	Gender	Education	Percentile	Raw Score	Norm Score	Assessment
13	m	13-16 years	0.171	743.5431323	86	Grand Index
13	m	13-16 years	0.175	744	86	Grand Index
13	m	13-16 years	0.176	744.528	86	Grand Index
13	m	13-16 years	0.177	745.156	86	Grand Index
13	m	13-16 years	0.178	745.784	86	Grand Index
13	m	13-16 years	0.184	746	86	Grand Index
13	m	13-16 years	0.185	746.18	87	Grand Index
13	m	13-16 years	0.186	746.808	87	Grand Index
13	m	13-16 years	0.191	747	87	Grand Index
13	m	13-16 years	0.192	747.576	87	Grand Index
13	m	13-16 years	0.195	748	87	Grand Index
13	m	13-16 years	0.196	748.088	87	Grand Index
13	m	13-16 years	0.197	748.716	87	Grand Index
13	m	13-16 years	0.198	749.344	87	Grand Index
13	m	13-16 years	0.199	749.972	87	Grand Index
13	m	13-16 years	0.203	750	88	Grand Index
13	m	13-16 years	0.204	750.112	88	Grand Index
13	m	13-16 years	0.205	750.74	88	Grand Index
13	m	13-16 years	0.21	751	88	Grand Index
13	m	13-16 years	0.211	751.2668773	88	Grand Index
13	m	13-16 years	0.212	751.5899015	88	Grand Index
13	m	13-16 years	0.213	751.8879824	88	Grand Index
13	m	13-16 years	0.216	752	88	Grand Index
13	m	13-16 years	0.217	752.242731	88	Grand Index
13	m	13-16 years	0.218	752.7950318	88	Grand Index
13	m	13-16 years	0.219	753.4755873	88	Grand Index
13	m	13-16 years	0.222	754	89	Grand Index
13	m	13-16 years	0.223	754.0438532	89	Grand Index
13	m	13-16 years	0.224	754.6697576	89	Grand Index
13	m	13-16 years	0.225	754.9976641	89	Grand Index
13	m	13-16 years	0.226	754.9997597	89	Grand Index
13	m	13-16 years	0.227	755	89	Grand Index
13	m	13-16 years	0.228	755.368	89	Grand Index
13	m	13-16 years	0.229	756.624	89	Grand Index
13	m	13-16 years	0.23	757.4209967	89	Grand Index
13	m	13-16 years	0.231	757.9597475	89	Grand Index
13	m	13-16 years	0.232	757.9868704	89	Grand Index
13	m	13-16 years	0.234	758	89	Grand Index
13	m	13-16 years	0.235	758.5712037	89	Grand Index
13	m	13-16 years	0.236	758.9879885	89	Grand Index
13	m	13-16 years	0.237	758.9975128	89	Grand Index
13	m	13-16 years	0.24	759	89	Grand Index
13	m	13-16 years	0.241	759.348	89	Grand Index
13	m	13-16 years	0.242	759.976	90	Grand Index
13	m	13-16 years	0.243	760	90	Grand Index
13	m	13-16 years	0.244	760.232	90	Grand Index
13	m	13-16 years	0.245	760.86	90	Grand Index
13	m	13-16 years	0.246	761	90	Grand Index
13	m	13-16 years	0.247	761.116	90	Grand Index
13	m	13-16 years	0.248	761.744	90	Grand Index
13	m	13-16 years	0.251	762	90	Grand Index
13	m	13-16 years	0.252	762.256	90	Grand Index
13	m	13-16 years	0.253	762.884	90	Grand Index
13	m	13-16 years	0.259	763	90	Grand Index
13	m	13-16 years	0.26	763.0238185	90	Grand Index
13	m	13-16 years	0.261	763.0772401	90	Grand Index

Age	Gender	Education	Percentile	Raw Score	Norm Score	Assessment
13	m	13-16 years	0.262	763.5754707	90	Grand Index
13	m	13-16 years	0.263	764.164	90	Grand Index
13	m	13-16 years	0.264	764.792	91	Grand Index
13	m	13-16 years	0.265	765.42	91	Grand Index
13	m	13-16 years	0.27	766	91	Grand Index
13	m	13-16 years	0.271	766.3504218	91	Grand Index
13	m	13-16 years	0.272	767.5209796	91	Grand Index
13	m	13-16 years	0.273	767.8699359	91	Grand Index
13	m	13-16 years	0.274	767.8862618	91	Grand Index
13	m	13-16 years	0.275	767.9632312	91	Grand Index
13	m	13-16 years	0.276	768.328	91	Grand Index
13	m	13-16 years	0.277	768.956	91	Grand Index
13	m	13-16 years	0.285	769	91	Grand Index
13	m	13-16 years	0.286	769.0067807	92	Grand Index
13	m	13-16 years	0.287	769.0170967	92	Grand Index
13	m	13-16 years	0.288	769.0329145	92	Grand Index
13	m	13-16 years	0.289	769.5104607	92	Grand Index
13	m	13-16 years	0.292	770	92	Grand Index
13	m	13-16 years	0.293	770.004	92	Grand Index
13	m	13-16 years	0.294	770.632	92	Grand Index
13	m	13-16 years	0.296	771	92	Grand Index
13	m	13-16 years	0.297	772.032	92	Grand Index
13	m	13-16 years	0.298	773.1433007	92	Grand Index
13	m	13-16 years	0.299	773.768251	92	Grand Index
13	m	13-16 years	0.3	773.9970863	92	Grand Index
13	m	13-16 years	0.304	774	92	Grand Index
13	m	13-16 years	0.305	774.4433149	92	Grand Index
13	m	13-16 years	0.306	774.8510333	92	Grand Index
13	m	13-16 years	0.307	774.9634745	92	Grand Index
13	m	13-16 years	0.31	775	93	Grand Index
13	m	13-16 years	0.311	775.308	93	Grand Index
13	m	13-16 years	0.312	775.936	93	Grand Index
13	m	13-16 years	0.316	776	93	Grand Index
13	m	13-16 years	0.317	776.076	93	Grand Index
13	m	13-16 years	0.318	776.704	93	Grand Index
13	m	13-16 years	0.321	777	93	Grand Index
13	m	13-16 years	0.322	777.2141441	93	Grand Index
13	m	13-16 years	0.323	777.8367482	93	Grand Index
13	m	13-16 years	0.324	777.9954633	93	Grand Index
13	m	13-16 years	0.326	778	93	Grand Index
13	m	13-16 years	0.327	778.356	93	Grand Index
13	m	13-16 years	0.328	778.984	93	Grand Index
13	m	13-16 years	0.334	779	94	Grand Index
13	m	13-16 years	0.335	779.3063161	94	Grand Index
13	m	13-16 years	0.336	779.8076462	94	Grand Index
13	m	13-16 years	0.337	779.9294186	94	Grand Index
13	m	13-16 years	0.343	780	94	Grand Index
13	m	13-16 years	0.344	780.0319027	94	Grand Index
13	m	13-16 years	0.345	780.6579932	94	Grand Index
13	m	13-16 years	0.346	780.9978351	94	Grand Index
13	m	13-16 years	0.347	780.9997446	94	Grand Index
13	m	13-16 years	0.351	781	94	Grand Index
13	m	13-16 years	0.352	781.056	94	Grand Index
13	m	13-16 years	0.353	781.684	94	Grand Index
13	m	13-16 years	0.354	782.3081661	94	Grand Index
13	m	13-16 years	0.355	782.9284491	94	Grand Index

Age	Gender	Education	Percentile	Raw Score	Norm Score	Assessment
13	m	13-16 years	0.356	782.9946915	94	Grand Index
13	m	13-16 years	0.359	783	95	Grand Index
13	m	13-16 years	0.36	783.08	95	Grand Index
13	m	13-16 years	0.361	783.708	95	Grand Index
13	m	13-16 years	0.362	784.336	95	Grand Index
13	m	13-16 years	0.363	784.964	95	Grand Index
13	m	13-16 years	0.367	785	95	Grand Index
13	m	13-16 years	0.368	785.0441349	95	Grand Index
13	m	13-16 years	0.369	785.310642	95	Grand Index
13	m	13-16 years	0.37	785.6315996	95	Grand Index
13	m	13-16 years	0.371	785.9930925	95	Grand Index
13	m	13-16 years	0.374	786	95	Grand Index
13	m	13-16 years	0.375	786.5	95	Grand Index
13	m	13-16 years	0.378	787	95	Grand Index
13	m	13-16 years	0.379	787.024	95	Grand Index
13	m	13-16 years	0.38	788.28	95	Grand Index
13	m	13-16 years	0.386	789	96	Grand Index
13	m	13-16 years	0.387	789.036	96	Grand Index
13	m	13-16 years	0.388	789.664	96	Grand Index
13	m	13-16 years	0.393	790	96	Grand Index
13	m	13-16 years	0.394	790.432	96	Grand Index
13	m	13-16 years	0.396	791	96	Grand Index
13	m	13-16 years	0.397	791.316	96	Grand Index
13	m	13-16 years	0.398	791.944	96	Grand Index
13	m	13-16 years	0.404	792	96	Grand Index
13	m	13-16 years	0.405	792.34	96	Grand Index
13	m	13-16 years	0.406	792.968	96	Grand Index
13	m	13-16 years	0.407	793	96	Grand Index
13	m	13-16 years	0.408	793.224	97	Grand Index
13	m	13-16 years	0.409	793.852	97	Grand Index
13	m	13-16 years	0.41	794	97	Grand Index
13	m	13-16 years	0.411	794.108	97	Grand Index
13	m	13-16 years	0.412	794.736	97	Grand Index
13	m	13-16 years	0.415	795	97	Grand Index
13	m	13-16 years	0.416	795.248	97	Grand Index
13	m	13-16 years	0.417	795.876	97	Grand Index
13	m	13-16 years	0.418	796	97	Grand Index
13	m	13-16 years	0.419	796.132	97	Grand Index
13	m	13-16 years	0.42	796.76	97	Grand Index
13	m	13-16 years	0.425	797	97	Grand Index
13	m	13-16 years	0.426	797.528	97	Grand Index
13	m	13-16 years	0.429	798	97	Grand Index
13	m	13-16 years	0.43	798.04	97	Grand Index
13	m	13-16 years	0.431	798.668	97	Grand Index
13	m	13-16 years	0.434	799	98	Grand Index
13	m	13-16 years	0.435	799.0405	98	Grand Index
13	m	13-16 years	0.436	799.1818	98	Grand Index
13	m	13-16 years	0.437	799.5629	98	Grand Index
13	m	13-16 years	0.439	800	98	Grand Index
13	m	13-16 years	0.44	800.32	98	Grand Index
13	m	13-16 years	0.441	800.948	98	Grand Index
13	m	13-16 years	0.442	801	98	Grand Index
13	m	13-16 years	0.443	801.204	98	Grand Index
13	m	13-16 years	0.444	801.832	98	Grand Index
13	m	13-16 years	0.445	802.92	98	Grand Index
13	m	13-16 years	0.447	804	98	Grand Index

Age	Gender	Education	Percentile	Raw Score	Norm Score	Assessment
13	m	13-16 years	0.448	804.001902	98	Grand Index
13	m	13-16 years	0.449	804.0053741	98	Grand Index
13	m	13-16 years	0.45	804.6022116	98	Grand Index
13	m	13-16 years	0.455	805	98	Grand Index
13	m	13-16 years	0.456	805.368	98	Grand Index
13	m	13-16 years	0.457	805.996	98	Grand Index
13	m	13-16 years	0.458	806	98	Grand Index
13	m	13-16 years	0.459	806.252	98	Grand Index
13	m	13-16 years	0.46	806.88	98	Grand Index
13	m	13-16 years	0.466	807	99	Grand Index
13	m	13-16 years	0.467	807.276	99	Grand Index
13	m	13-16 years	0.468	807.904	99	Grand Index
13	m	13-16 years	0.469	808	99	Grand Index
13	m	13-16 years	0.47	808.16	99	Grand Index
13	m	13-16 years	0.471	808.788	99	Grand Index
13	m	13-16 years	0.472	809	99	Grand Index
13	m	13-16 years	0.473	809.044	99	Grand Index
13	m	13-16 years	0.474	809.672	99	Grand Index
13	m	13-16 years	0.48	810	99	Grand Index
13	m	13-16 years	0.481	810.0206519	99	Grand Index
13	m	13-16 years	0.482	810.2113778	99	Grand Index
13	m	13-16 years	0.483	810.486081	99	Grand Index
13	m	13-16 years	0.484	810.8395778	99	Grand Index
13	m	13-16 years	0.485	810.9439706	99	Grand Index
13	m	13-16 years	0.493	811	100	Grand Index
13	m	13-16 years	0.494	811.232	100	Grand Index
13	m	13-16 years	0.495	811.86	100	Grand Index
13	m	13-16 years	0.5	812	100	Grand Index
13	m	13-16 years	0.501	812.628	100	Grand Index
13	m	13-16 years	0.504	813	100	Grand Index
13	m	13-16 years	0.505	813.1037608	100	Grand Index
13	m	13-16 years	0.506	813.5692019	100	Grand Index
13	m	13-16 years	0.507	813.8436535	100	Grand Index
13	m	13-16 years	0.511	814	100	Grand Index
13	m	13-16 years	0.512	814.2571689	100	Grand Index
13	m	13-16 years	0.513	814.5651067	100	Grand Index
13	m	13-16 years	0.514	814.8917969	101	Grand Index
13	m	13-16 years	0.515	815	101	Grand Index
13	m	13-16 years	0.516	815.0006136	101	Grand Index
13	m	13-16 years	0.517	815.0086416	101	Grand Index
13	m	13-16 years	0.518	815.3128973	101	Grand Index
13	m	13-16 years	0.519	815.9328693	101	Grand Index
13	m	13-16 years	0.52	816	101	Grand Index
13	m	13-16 years	0.521	816.188	101	Grand Index
13	m	13-16 years	0.522	816.816	101	Grand Index
13	m	13-16 years	0.525	817	101	Grand Index
13	m	13-16 years	0.526	817.328	101	Grand Index
13	m	13-16 years	0.527	817.956	101	Grand Index
13	m	13-16 years	0.53	818	101	Grand Index
13	m	13-16 years	0.531	818.0258097	101	Grand Index
13	m	13-16 years	0.532	818.126635	101	Grand Index
13	m	13-16 years	0.533	818.5942732	101	Grand Index
13	m	13-16 years	0.534	818.8702676	101	Grand Index
13	m	13-16 years	0.535	818.9959959	101	Grand Index
13	m	13-16 years	0.539	819	101	Grand Index
13	m	13-16 years	0.54	819.12	102	Grand Index

Age	Gender	Education	Percentile	Raw Score	Norm Score	Assessment
13	m	13-16 years	0.541	819.748	102	Grand Index
13	m	13-16 years	0.547	820	102	Grand Index
13	m	13-16 years	0.548	820.144	102	Grand Index
13	m	13-16 years	0.549	820.772	102	Grand Index
13	m	13-16 years	0.55	821.4	102	Grand Index
13	m	13-16 years	0.555	822	102	Grand Index
13	m	13-16 years	0.556	822.0553455	102	Grand Index
13	m	13-16 years	0.557	822.2622323	102	Grand Index
13	m	13-16 years	0.558	822.3740956	102	Grand Index
13	m	13-16 years	0.559	822.4641556	102	Grand Index
13	m	13-16 years	0.56	822.8191242	102	Grand Index
13	m	13-16 years	0.562	823	102	Grand Index
13	m	13-16 years	0.563	823.0122486	102	Grand Index
13	m	13-16 years	0.564	823.2095476	102	Grand Index
13	m	13-16 years	0.565	823.8239091	102	Grand Index
13	m	13-16 years	0.568	824	103	Grand Index
13	m	13-16 years	0.569	824.332	103	Grand Index
13	m	13-16 years	0.57	824.96	103	Grand Index
13	m	13-16 years	0.573	825	103	Grand Index
13	m	13-16 years	0.574	825.4658913	103	Grand Index
13	m	13-16 years	0.575	825.988352	103	Grand Index
13	m	13-16 years	0.576	825.9964797	103	Grand Index
13	m	13-16 years	0.582	826	103	Grand Index
13	m	13-16 years	0.583	826.124	103	Grand Index
13	m	13-16 years	0.584	826.752	103	Grand Index
13	m	13-16 years	0.59	827	103	Grand Index
13	m	13-16 years	0.591	827.0045275	103	Grand Index
13	m	13-16 years	0.592	827.0237387	103	Grand Index
13	m	13-16 years	0.593	827.4222323	104	Grand Index
13	m	13-16 years	0.595	828	104	Grand Index
13	m	13-16 years	0.596	828.0827566	104	Grand Index
13	m	13-16 years	0.597	828.263212	104	Grand Index
13	m	13-16 years	0.598	828.6750313	104	Grand Index
13	m	13-16 years	0.601	829	104	Grand Index
13	m	13-16 years	0.602	829.056	104	Grand Index
13	m	13-16 years	0.603	829.684	104	Grand Index
13	m	13-16 years	0.613	830	104	Grand Index
13	m	13-16 years	0.614	830.592	104	Grand Index
13	m	13-16 years	0.621	831	105	Grand Index
13	m	13-16 years	0.622	831.4767035	105	Grand Index
13	m	13-16 years	0.623	831.8290452	105	Grand Index
13	m	13-16 years	0.624	831.9710553	105	Grand Index
13	m	13-16 years	0.625	832.5	105	Grand Index
13	m	13-16 years	0.627	833	105	Grand Index
13	m	13-16 years	0.628	833.384	105	Grand Index
13	m	13-16 years	0.632	834	105	Grand Index
13	m	13-16 years	0.633	834.524	105	Grand Index
13	m	13-16 years	0.638	835	105	Grand Index
13	m	13-16 years	0.639	835.292	105	Grand Index
13	m	13-16 years	0.64	835.92	105	Grand Index
13	m	13-16 years	0.641	836	105	Grand Index
13	m	13-16 years	0.642	836.176	105	Grand Index
13	m	13-16 years	0.643	836.804	105	Grand Index
13	m	13-16 years	0.644	837	106	Grand Index
13	m	13-16 years	0.645	837.0265548	106	Grand Index
13	m	13-16 years	0.646	837.3044955	106	Grand Index

Age	Gender	Education	Percentile	Raw Score	Norm Score	Assessment
13	m	13-16 years	0.647	837.6187252	106	Grand Index
13	m	13-16 years	0.648	837.9687845	106	Grand Index
13	m	13-16 years	0.651	838	106	Grand Index
13	m	13-16 years	0.652	838.0836114	106	Grand Index
13	m	13-16 years	0.653	838.2519563	106	Grand Index
13	m	13-16 years	0.654	838.7648072	106	Grand Index
13	m	13-16 years	0.655	839.34	106	Grand Index
13	m	13-16 years	0.656	839.968	106	Grand Index
13	m	13-16 years	0.659	840	106	Grand Index
13	m	13-16 years	0.66	840.4710037	106	Grand Index
13	m	13-16 years	0.661	840.983282	106	Grand Index
13	m	13-16 years	0.662	840.9950521	106	Grand Index
13	m	13-16 years	0.663	841.364	106	Grand Index
13	m	13-16 years	0.664	841.992	106	Grand Index
13	m	13-16 years	0.665	842	106	Grand Index
13	m	13-16 years	0.666	842.248	106	Grand Index
13	m	13-16 years	0.667	842.876	106	Grand Index
13	m	13-16 years	0.668	843	107	Grand Index
13	m	13-16 years	0.669	843.132	107	Grand Index
13	m	13-16 years	0.67	843.76	107	Grand Index
13	m	13-16 years	0.673	844	107	Grand Index
13	m	13-16 years	0.674	844.2132727	107	Grand Index
13	m	13-16 years	0.675	844.7056818	107	Grand Index
13	m	13-16 years	0.676	844.8980909	107	Grand Index
13	m	13-16 years	0.679	845	107	Grand Index
13	m	13-16 years	0.68	845.04	107	Grand Index
13	m	13-16 years	0.681	845.668	107	Grand Index
13	m	13-16 years	0.683	846	107	Grand Index
13	m	13-16 years	0.684	846.552	107	Grand Index
13	m	13-16 years	0.687	847	107	Grand Index
13	m	13-16 years	0.688	847.1161942	107	Grand Index
13	m	13-16 years	0.689	848.2563495	107	Grand Index
13	m	13-16 years	0.69	848.8745631	107	Grand Index
13	m	13-16 years	0.691	848.9904078	107	Grand Index
13	m	13-16 years	0.692	849	108	Grand Index
13	m	13-16 years	0.693	849.0378505	108	Grand Index
13	m	13-16 years	0.694	849.1543708	108	Grand Index
13	m	13-16 years	0.695	849.5601926	108	Grand Index
13	m	13-16 years	0.699	850	108	Grand Index
13	m	13-16 years	0.7	850.6	108	Grand Index
13	m	13-16 years	0.701	851.0536922	108	Grand Index
13	m	13-16 years	0.702	851.2015812	108	Grand Index
13	m	13-16 years	0.703	851.6055139	108	Grand Index
13	m	13-16 years	0.708	852	108	Grand Index
13	m	13-16 years	0.709	852.252	108	Grand Index
13	m	13-16 years	0.71	852.88	108	Grand Index
13	m	13-16 years	0.713	853	108	Grand Index
13	m	13-16 years	0.714	853.0043745	108	Grand Index
13	m	13-16 years	0.715	853.0509361	109	Grand Index
13	m	13-16 years	0.716	854.2999281	109	Grand Index
13	m	13-16 years	0.721	855	109	Grand Index
13	m	13-16 years	0.722	855.416	109	Grand Index
13	m	13-16 years	0.726	856	109	Grand Index
13	m	13-16 years	0.727	856.201596	109	Grand Index
13	m	13-16 years	0.728	856.3992456	109	Grand Index
13	m	13-16 years	0.729	856.5243776	109	Grand Index

Age	Gender	Education	Percentile	Raw Score	Norm Score	Assessment
13	m	13-16 years	0.73	856.754629	109	Grand Index
13	m	13-16 years	0.737	857	110	Grand Index
13	m	13-16 years	0.738	857.3710528	110	Grand Index
13	m	13-16 years	0.739	857.8181119	110	Grand Index
13	m	13-16 years	0.74	857.9439111	110	Grand Index
13	m	13-16 years	0.741	858.348	110	Grand Index
13	m	13-16 years	0.742	858.976	110	Grand Index
13	m	13-16 years	0.743	859.604	110	Grand Index
13	m	13-16 years	0.745	860	110	Grand Index
13	m	13-16 years	0.746	860.488	110	Grand Index
13	m	13-16 years	0.748	861	110	Grand Index
13	m	13-16 years	0.749	861.0473146	110	Grand Index
13	m	13-16 years	0.75	861.1271898	110	Grand Index
13	m	13-16 years	0.751	861.6753146	110	Grand Index
13	m	13-16 years	0.757	862	110	Grand Index
13	m	13-16 years	0.758	862.0118857	110	Grand Index
13	m	13-16 years	0.759	862.3228952	111	Grand Index
13	m	13-16 years	0.76	862.6365714	111	Grand Index
13	m	13-16 years	0.761	862.9535619	111	Grand Index
13	m	13-16 years	0.765	863	111	Grand Index
13	m	13-16 years	0.766	863.048	111	Grand Index
13	m	13-16 years	0.767	863.676	111	Grand Index
13	m	13-16 years	0.768	864.2135979	111	Grand Index
13	m	13-16 years	0.769	864.6548461	111	Grand Index
13	m	13-16 years	0.77	864.8691548	111	Grand Index
13	m	13-16 years	0.773	865	111	Grand Index
13	m	13-16 years	0.774	865.072	111	Grand Index
13	m	13-16 years	0.775	865.7	111	Grand Index
13	m	13-16 years	0.777	866	111	Grand Index
13	m	13-16 years	0.778	866.4910322	111	Grand Index
13	m	13-16 years	0.779	866.8745572	112	Grand Index
13	m	13-16 years	0.78	866.9745294	112	Grand Index
13	m	13-16 years	0.783	867	112	Grand Index
13	m	13-16 years	0.784	867.0182397	112	Grand Index
13	m	13-16 years	0.785	867.0507809	112	Grand Index
13	m	13-16 years	0.786	867.6283123	112	Grand Index
13	m	13-16 years	0.788	868	112	Grand Index
13	m	13-16 years	0.789	868.0331399	112	Grand Index
13	m	13-16 years	0.79	868.1792746	112	Grand Index
13	m	13-16 years	0.791	868.7649741	112	Grand Index
13	m	13-16 years	0.792	869	112	Grand Index
13	m	13-16 years	0.793	869.004	112	Grand Index
13	m	13-16 years	0.794	869.632	112	Grand Index
13	m	13-16 years	0.799	870	113	Grand Index
13	m	13-16 years	0.8	870.4	113	Grand Index
13	m	13-16 years	0.804	871	113	Grand Index
13	m	13-16 years	0.805	871.8986821	113	Grand Index
13	m	13-16 years	0.806	872.7206361	113	Grand Index
13	m	13-16 years	0.807	872.9315021	113	Grand Index
13	m	13-16 years	0.81	873	113	Grand Index
13	m	13-16 years	0.811	873.308	113	Grand Index
13	m	13-16 years	0.812	873.936	113	Grand Index
13	m	13-16 years	0.813	874	113	Grand Index
13	m	13-16 years	0.814	874.192	113	Grand Index
13	m	13-16 years	0.815	874.82	113	Grand Index
13	m	13-16 years	0.816	875.448	114	Grand Index

Age	Gender	Education	Percentile	Raw Score	Norm Score	Assessment
13	m	13-16 years	0.817	876.0757556	114	Grand Index
13	m	13-16 years	0.818	876.7017361	114	Grand Index
13	m	13-16 years	0.819	876.9978519	114	Grand Index
13	m	13-16 years	0.82	876.9998714	114	Grand Index
13	m	13-16 years	0.823	877	114	Grand Index
13	m	13-16 years	0.824	877.0056197	114	Grand Index
13	m	13-16 years	0.825	877.0780885	114	Grand Index
13	m	13-16 years	0.826	877.4937137	114	Grand Index
13	m	13-16 years	0.827	877.7898819	114	Grand Index
13	m	13-16 years	0.828	877.9947797	114	Grand Index
13	m	13-16 years	0.831	878	114	Grand Index
13	m	13-16 years	0.832	878.496	114	Grand Index
13	m	13-16 years	0.833	879.124	114	Grand Index
13	m	13-16 years	0.834	879.752	115	Grand Index
13	m	13-16 years	0.835	880.38	115	Grand Index
13	m	13-16 years	0.837	881	115	Grand Index
13	m	13-16 years	0.838	881.0077161	115	Grand Index
13	m	13-16 years	0.839	881.026071	115	Grand Index
13	m	13-16 years	0.84	881.5340292	115	Grand Index
13	m	13-16 years	0.841	882.148	115	Grand Index
13	m	13-16 years	0.842	882.776	115	Grand Index
13	m	13-16 years	0.845	883	115	Grand Index
13	m	13-16 years	0.846	883.576	115	Grand Index
13	m	13-16 years	0.847	884.832	115	Grand Index
13	m	13-16 years	0.848	885.544	115	Grand Index
13	m	13-16 years	0.853	886	116	Grand Index
13	m	13-16 years	0.854	886.312	116	Grand Index
13	m	13-16 years	0.855	886.94	116	Grand Index
13	m	13-16 years	0.856	887.1588602	116	Grand Index
13	m	13-16 years	0.857	887.4208654	116	Grand Index
13	m	13-16 years	0.858	887.8732243	116	Grand Index
13	m	13-16 years	0.859	888.904	116	Grand Index
13	m	13-16 years	0.861	890	116	Grand Index
13	m	13-16 years	0.862	890.336	116	Grand Index
13	m	13-16 years	0.863	890.964	116	Grand Index
13	m	13-16 years	0.864	891	116	Grand Index
13	m	13-16 years	0.865	891.1332298	117	Grand Index
13	m	13-16 years	0.866	891.5135404	117	Grand Index
13	m	13-16 years	0.867	891.7933292	117	Grand Index
13	m	13-16 years	0.871	892	117	Grand Index
13	m	13-16 years	0.872	892.616	117	Grand Index
13	m	13-16 years	0.874	893	117	Grand Index
13	m	13-16 years	0.875	893.5	117	Grand Index
13	m	13-16 years	0.876	894.128	117	Grand Index
13	m	13-16 years	0.877	894.756	117	Grand Index
13	m	13-16 years	0.878	895.384	117	Grand Index
13	m	13-16 years	0.88	896	118	Grand Index
13	m	13-16 years	0.881	896.268	118	Grand Index
13	m	13-16 years	0.882	896.896	118	Grand Index
13	m	13-16 years	0.885	897	118	Grand Index
13	m	13-16 years	0.886	897.0080957	118	Grand Index
13	m	13-16 years	0.887	897.0677403	118	Grand Index
13	m	13-16 years	0.888	897.9032926	118	Grand Index
13	m	13-16 years	0.889	898.4056645	118	Grand Index
13	m	13-16 years	0.89	898.5246508	118	Grand Index
13	m	13-16 years	0.891	899.349115	118	Grand Index

Age	Gender	Education	Percentile	Raw Score	Norm Score	Assessment
13	m	13-16 years	0.892	900.3657157	119	Grand Index
13	m	13-16 years	0.893	901.6112625	119	Grand Index
13	m	13-16 years	0.894	902	119	Grand Index
13	m	13-16 years	0.895	902.06	119	Grand Index
13	m	13-16 years	0.896	902.688	119	Grand Index
13	m	13-16 years	0.898	903	119	Grand Index
13	m	13-16 years	0.899	903.572	119	Grand Index
13	m	13-16 years	0.901	904	119	Grand Index
13	m	13-16 years	0.902	904.3274311	119	Grand Index
13	m	13-16 years	0.903	904.7200801	119	Grand Index
13	m	13-16 years	0.904	904.7352519	120	Grand Index
13	m	13-16 years	0.905	906.1818136	120	Grand Index
13	m	13-16 years	0.906	908.8408469	120	Grand Index
13	m	13-16 years	0.907	910.182441	120	Grand Index
13	m	13-16 years	0.908	911.224	120	Grand Index
13	m	13-16 years	0.909	911.852	120	Grand Index
13	m	13-16 years	0.91	912.3696422	120	Grand Index
13	m	13-16 years	0.911	912.9076575	120	Grand Index
13	m	13-16 years	0.912	913.7075988	120	Grand Index
13	m	13-16 years	0.913	914.3919076	120	Grand Index
13	m	13-16 years	0.914	914.992351	120	Grand Index
13	m	13-16 years	0.917	915	121	Grand Index
13	m	13-16 years	0.918	915.1123567	121	Grand Index
13	m	13-16 years	0.92	915.2229299	121	Grand Index
13	m	13-16 years	0.921	915.5244331	121	Grand Index
13	m	13-16 years	0.923	916	121	Grand Index
13	m	13-16 years	0.924	916.1414968	121	Grand Index
13	m	13-16 years	0.925	916.4681881	122	Grand Index
13	m	13-16 years	0.926	917.3015386	122	Grand Index
13	m	13-16 years	0.927	918.936	122	Grand Index
13	m	13-16 years	0.928	922.704	122	Grand Index
13	m	13-16 years	0.929	924.119305	122	Grand Index
13	m	13-16 years	0.93	924.3979923	122	Grand Index
13	m	13-16 years	0.931	926.100139	122	Grand Index
13	m	13-16 years	0.932	927.2538784	122	Grand Index
13	m	13-16 years	0.933	927.7925123	122	Grand Index
13	m	13-16 years	0.934	927.9362484	123	Grand Index
13	m	13-16 years	0.935	928.36	123	Grand Index
13	m	13-16 years	0.936	929.616	123	Grand Index
13	m	13-16 years	0.937	930.436	123	Grand Index
13	m	13-16 years	0.942	931	124	Grand Index
13	m	13-16 years	0.943	931.612	124	Grand Index
13	m	13-16 years	0.944	933.496	124	Grand Index
13	m	13-16 years	0.945	935.2964138	124	Grand Index
13	m	13-16 years	0.946	936.8837827	124	Grand Index
13	m	13-16 years	0.947	937.3511558	124	Grand Index
13	m	13-16 years	0.948	937.7130102	124	Grand Index
13	m	13-16 years	0.949	937.9877504	125	Grand Index
13	m	13-16 years	0.95	938.6	125	Grand Index
13	m	13-16 years	0.951	939.228	125	Grand Index
13	m	13-16 years	0.952	939.856	125	Grand Index
13	m	13-16 years	0.953	941.452	125	Grand Index
13	m	13-16 years	0.954	943.112	125	Grand Index
13	m	13-16 years	0.955	943.74	125	Grand Index
13	m	13-16 years	0.956	946.208	126	Grand Index
13	m	13-16 years	0.957	949.976	126	Grand Index

Age	Gender	Education	Percentile	Raw Score	Norm Score	Assessment
13	m	13-16 years	0.958	953.12	126	Grand Index
13	m	13-16 years	0.96	955	126	Grand Index
13	m	13-16 years	0.961	956.8916595	126	Grand Index
13	m	13-16 years	0.962	958.7916292	127	Grand Index
13	m	13-16 years	0.963	959.1051209	127	Grand Index
13	m	13-16 years	0.964	959.5275414	127	Grand Index
13	m	13-16 years	0.965	960.04	127	Grand Index
13	m	13-16 years	0.966	961.296	127	Grand Index
13	m	13-16 years	0.967	962.276	128	Grand Index
13	m	13-16 years	0.968	962.904	128	Grand Index
13	m	13-16 years	0.969	963.532	128	Grand Index
13	m	13-16 years	0.97	964.32	128	Grand Index
13	m	13-16 years	0.971	965.576	128	Grand Index
13	m	13-16 years	0.972	967.664	129	Grand Index
13	m	13-16 years	0.973	970.264	129	Grand Index
13	m	13-16 years	0.974	974.032	129	Grand Index
13	m	13-16 years	0.975	976.3	129	Grand Index
13	m	13-16 years	0.976	976.928	130	Grand Index
13	m	13-16 years	0.977	979.224	130	Grand Index
13	m	13-16 years	0.978	981.92	130	Grand Index
13	m	13-16 years	0.979	985.06	131	Grand Index
13	m	13-16 years	0.98	986.3950445	131	Grand Index
13	m	13-16 years	0.981	986.9727761	131	Grand Index
13	m	13-16 years	0.982	987.6649399	131	Grand Index
13	m	13-16 years	0.983	988.9454673	132	Grand Index
13	m	13-16 years	0.984	990.7780396	132	Grand Index
13	m	13-16 years	0.985	991.8222506	133	Grand Index
13	m	13-16 years	0.986	994.3809226	133	Grand Index
13	m	13-16 years	0.987	1000.129383	133	Grand Index
13	m	13-16 years	0.988	1002.757865	134	Grand Index
13	m	13-16 years	0.989	1004.211236	134	Grand Index
13	m	13-16 years	0.99	1005.243032	135	Grand Index
13	m	13-16 years	0.991	1007.218584	135	Grand Index
13	m	13-16 years	0.992	1009.95348	136	Grand Index
13	m	13-16 years	0.993	1018.567667	137	Grand Index
13	m	13-16 years	0.994	1027.180503	138	Grand Index
13	m	13-16 years	0.995	1035.392279	139	Grand Index
13	m	13-16 years	0.996	1053.714867	140	Grand Index
13	m	13-16 years	0.997	1072.234105	141	Grand Index
13	m	13-16 years	0.998	1078.81848	143	Grand Index
13	m	13-16 years	0.999	1083.938762	146	Grand Index
13	f	17+ years	0.001	606.1945468	NA	Grand Index
13	f	17+ years	0.002	611.7425195	NA	Grand Index
13	f	17+ years	0.003	617.2904921	NA	Grand Index
13	f	17+ years	0.004	622.8384648	NA	Grand Index
13	f	17+ years	0.005	628.3864375	NA	Grand Index
13	f	17+ years	0.006	633.9344102	NA	Grand Index
13	f	17+ years	0.007	639.4823829	NA	Grand Index
13	f	17+ years	0.008	645.0303556	NA	Grand Index
13	f	17+ years	0.009	650.5783282	NA	Grand Index
13	f	17+ years	0.01	656.1263009	NA	Grand Index
13	f	17+ years	0.011	661.6742736	NA	Grand Index
13	f	17+ years	0.012	667.2222463	NA	Grand Index
13	f	17+ years	0.013	672.770219	NA	Grand Index
13	f	17+ years	0.014	678.3181917	NA	Grand Index
13	f	17+ years	0.015	683.8661644	NA	Grand Index

Age	Gender	Education	Percentile	Raw Score	Norm Score	Assessment
13	f	17+ years	0.016	686.64	NA	Grand Index
13	f	17+ years	0.017	687.68	NA	Grand Index
13	f	17+ years	0.018	688.72	NA	Grand Index
13	f	17+ years	0.019	689.76	NA	Grand Index
13	f	17+ years	0.02	690.8	NA	Grand Index
13	f	17+ years	0.021	691.84	NA	Grand Index
13	f	17+ years	0.022	692.88	NA	Grand Index
13	f	17+ years	0.023	693.92	NA	Grand Index
13	f	17+ years	0.024	694.96	NA	Grand Index
13	f	17+ years	0.025	696	NA	Grand Index
13	f	17+ years	0.026	697.04	NA	Grand Index
13	f	17+ years	0.027	698.08	NA	Grand Index
13	f	17+ years	0.028	699.12	NA	Grand Index
13	f	17+ years	0.029	700.16	NA	Grand Index
13	f	17+ years	0.03	701.2	NA	Grand Index
13	f	17+ years	0.031	702.255	NA	Grand Index
13	f	17+ years	0.032	703.36	NA	Grand Index
13	f	17+ years	0.033	704.465	NA	Grand Index
13	f	17+ years	0.034	705.57	NA	Grand Index
13	f	17+ years	0.035	706.675	NA	Grand Index
13	f	17+ years	0.036	707.78	NA	Grand Index
13	f	17+ years	0.037	708.885	NA	Grand Index
13	f	17+ years	0.038	709.99	NA	Grand Index
13	f	17+ years	0.039	711.095	NA	Grand Index
13	f	17+ years	0.04	712.2	NA	Grand Index
13	f	17+ years	0.041	713.305	NA	Grand Index
13	f	17+ years	0.042	714.41	NA	Grand Index
13	f	17+ years	0.043	715.515	NA	Grand Index
13	f	17+ years	0.044	716.62	NA	Grand Index
13	f	17+ years	0.045	717.725	NA	Grand Index
13	f	17+ years	0.046	718.83	NA	Grand Index
13	f	17+ years	0.047	719.165	NA	Grand Index
13	f	17+ years	0.048	719.36	NA	Grand Index
13	f	17+ years	0.049	719.555	NA	Grand Index
13	f	17+ years	0.05	719.75	NA	Grand Index
13	f	17+ years	0.051	719.945	NA	Grand Index
13	f	17+ years	0.052	720.14	NA	Grand Index
13	f	17+ years	0.053	720.335	NA	Grand Index
13	f	17+ years	0.054	720.53	NA	Grand Index
13	f	17+ years	0.055	720.725	NA	Grand Index
13	f	17+ years	0.056	720.92	NA	Grand Index
13	f	17+ years	0.057	721.115	NA	Grand Index
13	f	17+ years	0.058	721.31	NA	Grand Index
13	f	17+ years	0.059	721.505	NA	Grand Index
13	f	17+ years	0.06	721.7	NA	Grand Index
13	f	17+ years	0.061	721.895	NA	Grand Index
13	f	17+ years	0.076	722	NA	Grand Index
13	f	17+ years	0.077	722.01	NA	Grand Index
13	f	17+ years	0.078	722.14	NA	Grand Index
13	f	17+ years	0.079	722.27	NA	Grand Index
13	f	17+ years	0.08	722.4	NA	Grand Index
13	f	17+ years	0.081	722.53	NA	Grand Index
13	f	17+ years	0.082	722.66	NA	Grand Index
13	f	17+ years	0.083	722.79	NA	Grand Index
13	f	17+ years	0.084	722.92	NA	Grand Index
13	f	17+ years	0.085	723.05	NA	Grand Index

Age	Gender	Education	Percentile	Raw Score	Norm Score	Assessment
13	f	17+ years	0.086	723.18	NA	Grand Index
13	f	17+ years	0.087	723.31	NA	Grand Index
13	f	17+ years	0.088	723.44	NA	Grand Index
13	f	17+ years	0.089	723.57	NA	Grand Index
13	f	17+ years	0.09	723.7	NA	Grand Index
13	f	17+ years	0.091	723.83	NA	Grand Index
13	f	17+ years	0.092	723.96	NA	Grand Index
13	f	17+ years	0.093	724.135	NA	Grand Index
13	f	17+ years	0.094	724.33	NA	Grand Index
13	f	17+ years	0.095	724.525	NA	Grand Index
13	f	17+ years	0.096	724.72	NA	Grand Index
13	f	17+ years	0.097	724.915	NA	Grand Index
13	f	17+ years	0.098	725.11	NA	Grand Index
13	f	17+ years	0.099	725.305	NA	Grand Index
13	f	17+ years	0.1	725.5	NA	Grand Index
13	f	17+ years	0.101	725.695	NA	Grand Index
13	f	17+ years	0.102	725.89	NA	Grand Index
13	f	17+ years	0.103	726.085	NA	Grand Index
13	f	17+ years	0.104	726.28	NA	Grand Index
13	f	17+ years	0.105	726.475	NA	Grand Index
13	f	17+ years	0.106	726.67	NA	Grand Index
13	f	17+ years	0.107	726.865	NA	Grand Index
13	f	17+ years	0.108	727.08	NA	Grand Index
13	f	17+ years	0.109	727.34	NA	Grand Index
13	f	17+ years	0.11	727.6	NA	Grand Index
13	f	17+ years	0.111	727.86	NA	Grand Index
13	f	17+ years	0.112	728.12	NA	Grand Index
13	f	17+ years	0.113	728.38	NA	Grand Index
13	f	17+ years	0.114	728.64	NA	Grand Index
13	f	17+ years	0.115	728.9	NA	Grand Index
13	f	17+ years	0.116	729.16	NA	Grand Index
13	f	17+ years	0.117	729.42	NA	Grand Index
13	f	17+ years	0.118	729.68	NA	Grand Index
13	f	17+ years	0.119	729.94	NA	Grand Index
13	f	17+ years	0.12	730.2	NA	Grand Index
13	f	17+ years	0.121	730.46	NA	Grand Index
13	f	17+ years	0.122	730.72	NA	Grand Index
13	f	17+ years	0.123	730.98	NA	Grand Index
13	f	17+ years	0.124	731.48	NA	Grand Index
13	f	17+ years	0.125	732	NA	Grand Index
13	f	17+ years	0.126	732.52	NA	Grand Index
13	f	17+ years	0.127	733.04	NA	Grand Index
13	f	17+ years	0.128	733.56	NA	Grand Index
13	f	17+ years	0.129	734.08	NA	Grand Index
13	f	17+ years	0.13	734.6	NA	Grand Index
13	f	17+ years	0.131	735.12	NA	Grand Index
13	f	17+ years	0.132	735.64	NA	Grand Index
13	f	17+ years	0.133	736.16	NA	Grand Index
13	f	17+ years	0.134	736.68	NA	Grand Index
13	f	17+ years	0.135	737.2	NA	Grand Index
13	f	17+ years	0.136	737.72	NA	Grand Index
13	f	17+ years	0.137	738.24	NA	Grand Index
13	f	17+ years	0.138	738.76	NA	Grand Index
13	f	17+ years	0.139	739.21	NA	Grand Index
13	f	17+ years	0.14	739.6	NA	Grand Index
13	f	17+ years	0.141	739.99	NA	Grand Index

Age	Gender	Education	Percentile	Raw Score	Norm Score	Assessment
13	f	17+ years	0.142	740.38	NA	Grand Index
13	f	17+ years	0.143	740.77	NA	Grand Index
13	f	17+ years	0.144	741.16	NA	Grand Index
13	f	17+ years	0.145	741.55	NA	Grand Index
13	f	17+ years	0.146	741.94	NA	Grand Index
13	f	17+ years	0.147	742.33	NA	Grand Index
13	f	17+ years	0.148	742.72	NA	Grand Index
13	f	17+ years	0.149	743.11	NA	Grand Index
13	f	17+ years	0.15	743.5	NA	Grand Index
13	f	17+ years	0.151	743.89	NA	Grand Index
13	f	17+ years	0.152	744.28	NA	Grand Index
13	f	17+ years	0.153	744.67	NA	Grand Index
13	f	17+ years	0.154	745.02	NA	Grand Index
13	f	17+ years	0.155	745.15	NA	Grand Index
13	f	17+ years	0.156	745.28	NA	Grand Index
13	f	17+ years	0.157	745.41	NA	Grand Index
13	f	17+ years	0.158	745.54	NA	Grand Index
13	f	17+ years	0.159	745.67	NA	Grand Index
13	f	17+ years	0.16	745.8	NA	Grand Index
13	f	17+ years	0.161	745.93	NA	Grand Index
13	f	17+ years	0.162	746.06	NA	Grand Index
13	f	17+ years	0.163	746.19	NA	Grand Index
13	f	17+ years	0.164	746.32	NA	Grand Index
13	f	17+ years	0.165	746.45	NA	Grand Index
13	f	17+ years	0.166	746.58	NA	Grand Index
13	f	17+ years	0.167	746.71	NA	Grand Index
13	f	17+ years	0.168	746.84	NA	Grand Index
13	f	17+ years	0.169	746.97	NA	Grand Index
13	f	17+ years	0.17	747.05	NA	Grand Index
13	f	17+ years	0.171	747.115	NA	Grand Index
13	f	17+ years	0.172	747.18	NA	Grand Index
13	f	17+ years	0.173	747.245	NA	Grand Index
13	f	17+ years	0.174	747.31	NA	Grand Index
13	f	17+ years	0.175	747.375	NA	Grand Index
13	f	17+ years	0.176	747.44	NA	Grand Index
13	f	17+ years	0.177	747.505	NA	Grand Index
13	f	17+ years	0.178	747.57	NA	Grand Index
13	f	17+ years	0.179	747.635	NA	Grand Index
13	f	17+ years	0.18	747.7	NA	Grand Index
13	f	17+ years	0.181	747.765	NA	Grand Index
13	f	17+ years	0.182	747.83	NA	Grand Index
13	f	17+ years	0.183	747.895	NA	Grand Index
13	f	17+ years	0.184	747.96	NA	Grand Index
13	f	17+ years	0.185	748.075	NA	Grand Index
13	f	17+ years	0.186	748.27	NA	Grand Index
13	f	17+ years	0.187	748.465	NA	Grand Index
13	f	17+ years	0.188	748.66	NA	Grand Index
13	f	17+ years	0.189	748.855	NA	Grand Index
13	f	17+ years	0.19	749.05	NA	Grand Index
13	f	17+ years	0.191	749.245	NA	Grand Index
13	f	17+ years	0.192	749.44	NA	Grand Index
13	f	17+ years	0.193	749.635	NA	Grand Index
13	f	17+ years	0.194	749.83	NA	Grand Index
13	f	17+ years	0.195	750.025	NA	Grand Index
13	f	17+ years	0.196	750.22	NA	Grand Index
13	f	17+ years	0.197	750.415	NA	Grand Index

Age	Gender	Education	Percentile	Raw Score	Norm Score	Assessment
13	f	17+ years	0.198	750.61	NA	Grand Index
13	f	17+ years	0.199	750.805	NA	Grand Index
13	f	17+ years	0.215	751	NA	Grand Index
13	f	17+ years	0.216	751.16	NA	Grand Index
13	f	17+ years	0.217	751.42	NA	Grand Index
13	f	17+ years	0.218	751.68	NA	Grand Index
13	f	17+ years	0.219	751.94	NA	Grand Index
13	f	17+ years	0.22	752.2	NA	Grand Index
13	f	17+ years	0.221	752.46	NA	Grand Index
13	f	17+ years	0.222	752.72	NA	Grand Index
13	f	17+ years	0.223	752.98	NA	Grand Index
13	f	17+ years	0.224	753.24	NA	Grand Index
13	f	17+ years	0.225	753.5	NA	Grand Index
13	f	17+ years	0.226	753.76	NA	Grand Index
13	f	17+ years	0.227	754.02	NA	Grand Index
13	f	17+ years	0.228	754.28	NA	Grand Index
13	f	17+ years	0.229	754.54	NA	Grand Index
13	f	17+ years	0.23	754.8	NA	Grand Index
13	f	17+ years	0.231	755.1616947	NA	Grand Index
13	f	17+ years	0.232	755.8623716	NA	Grand Index
13	f	17+ years	0.233	756.5630486	NA	Grand Index
13	f	17+ years	0.234	757.2637255	NA	Grand Index
13	f	17+ years	0.235	757.9644024	NA	Grand Index
13	f	17+ years	0.236	758.6650794	NA	Grand Index
13	f	17+ years	0.237	759.3657563	NA	Grand Index
13	f	17+ years	0.238	760.0664332	NA	Grand Index
13	f	17+ years	0.239	760.7671102	NA	Grand Index
13	f	17+ years	0.24	761.4677871	NA	Grand Index
13	f	17+ years	0.241	762.1684641	NA	Grand Index
13	f	17+ years	0.242	762.869141	NA	Grand Index
13	f	17+ years	0.243	763.5698179	NA	Grand Index
13	f	17+ years	0.244	764.2704949	NA	Grand Index
13	f	17+ years	0.245	764.9711718	NA	Grand Index
13	f	17+ years	0.246	765.6718487	NA	Grand Index
13	f	17+ years	0.247	765.8467647	NA	Grand Index
13	f	17+ years	0.248	765.9260878	NA	Grand Index
13	f	17+ years	0.249	766.0054108	NA	Grand Index
13	f	17+ years	0.25	766.0847339	NA	Grand Index
13	f	17+ years	0.251	766.164057	NA	Grand Index
13	f	17+ years	0.252	766.24338	NA	Grand Index
13	f	17+ years	0.253	766.3227031	NA	Grand Index
13	f	17+ years	0.254	766.4020261	NA	Grand Index
13	f	17+ years	0.255	766.4813492	NA	Grand Index
13	f	17+ years	0.256	766.5606723	NA	Grand Index
13	f	17+ years	0.257	766.6399953	NA	Grand Index
13	f	17+ years	0.258	766.7193184	NA	Grand Index
13	f	17+ years	0.259	766.7986415	NA	Grand Index
13	f	17+ years	0.26	766.8779645	NA	Grand Index
13	f	17+ years	0.261	766.9572876	NA	Grand Index
13	f	17+ years	0.262	767.1427273	NA	Grand Index
13	f	17+ years	0.263	767.4519697	NA	Grand Index
13	f	17+ years	0.264	767.7612121	NA	Grand Index
13	f	17+ years	0.265	768.0704545	NA	Grand Index
13	f	17+ years	0.266	768.379697	NA	Grand Index
13	f	17+ years	0.267	768.6889394	NA	Grand Index
13	f	17+ years	0.268	768.9981818	NA	Grand Index

Age	Gender	Education	Percentile	Raw Score	Norm Score	Assessment
13	f	17+ years	0.269	769.3074242	NA	Grand Index
13	f	17+ years	0.27	769.6166667	NA	Grand Index
13	f	17+ years	0.271	769.9259091	NA	Grand Index
13	f	17+ years	0.272	770.2351515	NA	Grand Index
13	f	17+ years	0.273	770.5443939	NA	Grand Index
13	f	17+ years	0.274	770.8536364	NA	Grand Index
13	f	17+ years	0.275	771.1628788	NA	Grand Index
13	f	17+ years	0.276	771.4721212	NA	Grand Index
13	f	17+ years	0.277	771.7637879	NA	Grand Index
13	f	17+ years	0.278	771.8445455	NA	Grand Index
13	f	17+ years	0.279	771.925303	NA	Grand Index
13	f	17+ years	0.28	772.0060606	NA	Grand Index
13	f	17+ years	0.281	772.0868182	NA	Grand Index
13	f	17+ years	0.282	772.1675758	NA	Grand Index
13	f	17+ years	0.283	772.2483333	NA	Grand Index
13	f	17+ years	0.284	772.3290909	NA	Grand Index
13	f	17+ years	0.285	772.4098485	NA	Grand Index
13	f	17+ years	0.286	772.4906061	NA	Grand Index
13	f	17+ years	0.287	772.5713636	NA	Grand Index
13	f	17+ years	0.288	772.6521212	NA	Grand Index
13	f	17+ years	0.289	772.7328788	NA	Grand Index
13	f	17+ years	0.29	772.8136364	NA	Grand Index
13	f	17+ years	0.291	772.8943939	NA	Grand Index
13	f	17+ years	0.292	772.9751515	NA	Grand Index
13	f	17+ years	0.293	773.18	NA	Grand Index
13	f	17+ years	0.294	773.44	NA	Grand Index
13	f	17+ years	0.295	773.7	NA	Grand Index
13	f	17+ years	0.296	773.96	NA	Grand Index
13	f	17+ years	0.297	774.22	NA	Grand Index
13	f	17+ years	0.298	774.48	NA	Grand Index
13	f	17+ years	0.299	774.74	NA	Grand Index
13	f	17+ years	0.3	775	NA	Grand Index
13	f	17+ years	0.301	775.26	NA	Grand Index
13	f	17+ years	0.302	775.52	NA	Grand Index
13	f	17+ years	0.303	775.78	NA	Grand Index
13	f	17+ years	0.304	776.04	NA	Grand Index
13	f	17+ years	0.305	776.3	NA	Grand Index
13	f	17+ years	0.306	776.56	NA	Grand Index
13	f	17+ years	0.307	776.82	NA	Grand Index
13	f	17+ years	0.308	777.04	NA	Grand Index
13	f	17+ years	0.309	777.17	NA	Grand Index
13	f	17+ years	0.31	777.3	NA	Grand Index
13	f	17+ years	0.311	777.43	NA	Grand Index
13	f	17+ years	0.312	777.56	NA	Grand Index
13	f	17+ years	0.313	777.69	NA	Grand Index
13	f	17+ years	0.314	777.82	NA	Grand Index
13	f	17+ years	0.315	777.95	NA	Grand Index
13	f	17+ years	0.316	778.08	NA	Grand Index
13	f	17+ years	0.317	778.21	NA	Grand Index
13	f	17+ years	0.318	778.34	NA	Grand Index
13	f	17+ years	0.319	778.47	NA	Grand Index
13	f	17+ years	0.32	778.6	NA	Grand Index
13	f	17+ years	0.321	778.73	NA	Grand Index
13	f	17+ years	0.322	778.86	NA	Grand Index
13	f	17+ years	0.323	778.99	NA	Grand Index
13	f	17+ years	0.324	779.06	NA	Grand Index

Age	Gender	Education	Percentile	Raw Score	Norm Score	Assessment
13	f	17+ years	0.325	779.125	NA	Grand Index
13	f	17+ years	0.326	779.19	NA	Grand Index
13	f	17+ years	0.327	779.255	NA	Grand Index
13	f	17+ years	0.328	779.32	NA	Grand Index
13	f	17+ years	0.329	779.385	NA	Grand Index
13	f	17+ years	0.33	779.45	NA	Grand Index
13	f	17+ years	0.331	779.515	NA	Grand Index
13	f	17+ years	0.332	779.58	NA	Grand Index
13	f	17+ years	0.333	779.645	NA	Grand Index
13	f	17+ years	0.334	779.71	NA	Grand Index
13	f	17+ years	0.335	779.775	NA	Grand Index
13	f	17+ years	0.336	779.84	NA	Grand Index
13	f	17+ years	0.337	779.905	NA	Grand Index
13	f	17+ years	0.338	779.97	NA	Grand Index
13	f	17+ years	0.339	780.1057825	NA	Grand Index
13	f	17+ years	0.34	780.3022357	NA	Grand Index
13	f	17+ years	0.341	780.4986889	NA	Grand Index
13	f	17+ years	0.342	780.6951421	NA	Grand Index
13	f	17+ years	0.343	780.8915952	NA	Grand Index
13	f	17+ years	0.344	781.0880484	NA	Grand Index
13	f	17+ years	0.345	781.2845016	NA	Grand Index
13	f	17+ years	0.346	781.4809548	NA	Grand Index
13	f	17+ years	0.347	781.677408	NA	Grand Index
13	f	17+ years	0.348	781.8738612	NA	Grand Index
13	f	17+ years	0.349	782.0703144	NA	Grand Index
13	f	17+ years	0.35	782.2667676	NA	Grand Index
13	f	17+ years	0.351	782.4632208	NA	Grand Index
13	f	17+ years	0.352	782.659674	NA	Grand Index
13	f	17+ years	0.353	782.8561272	NA	Grand Index
13	f	17+ years	0.354	783.0621332	NA	Grand Index
13	f	17+ years	0.355	783.32068	NA	Grand Index
13	f	17+ years	0.356	783.5792268	NA	Grand Index
13	f	17+ years	0.357	783.8377736	NA	Grand Index
13	f	17+ years	0.358	784.0963204	NA	Grand Index
13	f	17+ years	0.359	784.3548673	NA	Grand Index
13	f	17+ years	0.36	784.6134141	NA	Grand Index
13	f	17+ years	0.361	784.8719609	NA	Grand Index
13	f	17+ years	0.362	785.1305077	NA	Grand Index
13	f	17+ years	0.363	785.3890545	NA	Grand Index
13	f	17+ years	0.364	785.6476013	NA	Grand Index
13	f	17+ years	0.365	785.9061481	NA	Grand Index
13	f	17+ years	0.366	786.1646949	NA	Grand Index
13	f	17+ years	0.367	786.4232417	NA	Grand Index
13	f	17+ years	0.368	786.6817885	NA	Grand Index
13	f	17+ years	0.369	786.9403354	NA	Grand Index
13	f	17+ years	0.37	787.2176139	NA	Grand Index
13	f	17+ years	0.371	787.500512	NA	Grand Index
13	f	17+ years	0.372	787.7834101	NA	Grand Index
13	f	17+ years	0.373	788.0663082	NA	Grand Index
13	f	17+ years	0.374	788.3492063	NA	Grand Index
13	f	17+ years	0.375	788.6321045	NA	Grand Index
13	f	17+ years	0.376	788.9150026	NA	Grand Index
13	f	17+ years	0.377	789.1979007	NA	Grand Index
13	f	17+ years	0.378	789.4807988	NA	Grand Index
13	f	17+ years	0.379	789.7636969	NA	Grand Index
13	f	17+ years	0.38	790.046595	NA	Grand Index

Age	Gender	Education	Percentile	Raw Score	Norm Score	Assessment
13	f	17+ years	0.381	790.3294931	NA	Grand Index
13	f	17+ years	0.382	790.6123912	NA	Grand Index
13	f	17+ years	0.383	790.8952893	NA	Grand Index
13	f	17+ years	0.384	791.1781874	NA	Grand Index
13	f	17+ years	0.385	791.4434716	NA	Grand Index
13	f	17+ years	0.386	791.6805735	NA	Grand Index
13	f	17+ years	0.387	791.9176754	NA	Grand Index
13	f	17+ years	0.388	792.1547773	NA	Grand Index
13	f	17+ years	0.389	792.3918792	NA	Grand Index
13	f	17+ years	0.39	792.6289811	NA	Grand Index
13	f	17+ years	0.391	792.8660829	NA	Grand Index
13	f	17+ years	0.392	793.1031848	NA	Grand Index
13	f	17+ years	0.393	793.3402867	NA	Grand Index
13	f	17+ years	0.394	793.5773886	NA	Grand Index
13	f	17+ years	0.395	793.8144905	NA	Grand Index
13	f	17+ years	0.396	794.0515924	NA	Grand Index
13	f	17+ years	0.397	794.2886943	NA	Grand Index
13	f	17+ years	0.398	794.5257962	NA	Grand Index
13	f	17+ years	0.399	794.7628981	NA	Grand Index
13	f	17+ years	0.4	795	NA	Grand Index
13	f	17+ years	0.401	795.13	NA	Grand Index
13	f	17+ years	0.402	795.26	NA	Grand Index
13	f	17+ years	0.403	795.39	NA	Grand Index
13	f	17+ years	0.404	795.52	NA	Grand Index
13	f	17+ years	0.405	795.65	NA	Grand Index
13	f	17+ years	0.406	795.78	NA	Grand Index
13	f	17+ years	0.407	795.91	NA	Grand Index
13	f	17+ years	0.408	796.04	NA	Grand Index
13	f	17+ years	0.409	796.17	NA	Grand Index
13	f	17+ years	0.41	796.3	NA	Grand Index
13	f	17+ years	0.411	796.43	NA	Grand Index
13	f	17+ years	0.412	796.56	NA	Grand Index
13	f	17+ years	0.413	796.69	NA	Grand Index
13	f	17+ years	0.414	796.82	NA	Grand Index
13	f	17+ years	0.415	796.95	NA	Grand Index
13	f	17+ years	0.416	797.04	NA	Grand Index
13	f	17+ years	0.417	797.105	NA	Grand Index
13	f	17+ years	0.418	797.17	NA	Grand Index
13	f	17+ years	0.419	797.235	NA	Grand Index
13	f	17+ years	0.42	797.3	NA	Grand Index
13	f	17+ years	0.421	797.365	NA	Grand Index
13	f	17+ years	0.422	797.43	NA	Grand Index
13	f	17+ years	0.423	797.495	NA	Grand Index
13	f	17+ years	0.424	797.56	NA	Grand Index
13	f	17+ years	0.425	797.625	NA	Grand Index
13	f	17+ years	0.426	797.69	NA	Grand Index
13	f	17+ years	0.427	797.755	NA	Grand Index
13	f	17+ years	0.428	797.82	NA	Grand Index
13	f	17+ years	0.429	797.885	NA	Grand Index
13	f	17+ years	0.43	797.95	NA	Grand Index
13	f	17+ years	0.431	798.045	NA	Grand Index
13	f	17+ years	0.432	798.24	NA	Grand Index
13	f	17+ years	0.433	798.435	NA	Grand Index
13	f	17+ years	0.434	798.63	NA	Grand Index
13	f	17+ years	0.435	798.825	NA	Grand Index
13	f	17+ years	0.436	799.02	NA	Grand Index

Age	Gender	Education	Percentile	Raw Score	Norm Score	Assessment
13	f	17+ years	0.437	799.215	NA	Grand Index
13	f	17+ years	0.438	799.41	NA	Grand Index
13	f	17+ years	0.439	799.605	NA	Grand Index
13	f	17+ years	0.44	799.8	NA	Grand Index
13	f	17+ years	0.441	799.995	NA	Grand Index
13	f	17+ years	0.442	800.19	NA	Grand Index
13	f	17+ years	0.443	800.385	NA	Grand Index
13	f	17+ years	0.444	800.58	NA	Grand Index
13	f	17+ years	0.445	800.775	NA	Grand Index
13	f	17+ years	0.446	800.97	NA	Grand Index
13	f	17+ years	0.447	801.055	NA	Grand Index
13	f	17+ years	0.448	801.12	NA	Grand Index
13	f	17+ years	0.449	801.185	NA	Grand Index
13	f	17+ years	0.45	801.25	NA	Grand Index
13	f	17+ years	0.451	801.315	NA	Grand Index
13	f	17+ years	0.452	801.38	NA	Grand Index
13	f	17+ years	0.453	801.445	NA	Grand Index
13	f	17+ years	0.454	801.51	NA	Grand Index
13	f	17+ years	0.455	801.575	NA	Grand Index
13	f	17+ years	0.456	801.64	NA	Grand Index
13	f	17+ years	0.457	801.705	NA	Grand Index
13	f	17+ years	0.458	801.77	NA	Grand Index
13	f	17+ years	0.459	801.835	NA	Grand Index
13	f	17+ years	0.46	801.9	NA	Grand Index
13	f	17+ years	0.461	801.965	NA	Grand Index
13	f	17+ years	0.462	802.09	NA	Grand Index
13	f	17+ years	0.463	802.285	NA	Grand Index
13	f	17+ years	0.464	802.48	NA	Grand Index
13	f	17+ years	0.465	802.675	NA	Grand Index
13	f	17+ years	0.466	802.87	NA	Grand Index
13	f	17+ years	0.467	803.065	NA	Grand Index
13	f	17+ years	0.468	803.26	NA	Grand Index
13	f	17+ years	0.469	803.455	NA	Grand Index
13	f	17+ years	0.47	803.65	NA	Grand Index
13	f	17+ years	0.471	803.845	NA	Grand Index
13	f	17+ years	0.472	804.04	NA	Grand Index
13	f	17+ years	0.473	804.235	NA	Grand Index
13	f	17+ years	0.474	804.43	NA	Grand Index
13	f	17+ years	0.475	804.625	NA	Grand Index
13	f	17+ years	0.476	804.82	NA	Grand Index
13	f	17+ years	0.477	805.005	NA	Grand Index
13	f	17+ years	0.478	805.07	NA	Grand Index
13	f	17+ years	0.479	805.135	NA	Grand Index
13	f	17+ years	0.48	805.2	NA	Grand Index
13	f	17+ years	0.481	805.265	NA	Grand Index
13	f	17+ years	0.482	805.33	NA	Grand Index
13	f	17+ years	0.483	805.395	NA	Grand Index
13	f	17+ years	0.484	805.46	NA	Grand Index
13	f	17+ years	0.485	805.525	NA	Grand Index
13	f	17+ years	0.486	805.59	NA	Grand Index
13	f	17+ years	0.487	805.655	NA	Grand Index
13	f	17+ years	0.488	805.72	NA	Grand Index
13	f	17+ years	0.489	805.785	NA	Grand Index
13	f	17+ years	0.49	805.85	NA	Grand Index
13	f	17+ years	0.491	805.915	NA	Grand Index
13	f	17+ years	0.492	805.98	NA	Grand Index

Age	Gender	Education	Percentile	Raw Score	Norm Score	Assessment
13	f	17+ years	0.493	806.225	NA	Grand Index
13	f	17+ years	0.494	806.55	NA	Grand Index
13	f	17+ years	0.495	806.875	NA	Grand Index
13	f	17+ years	0.496	807.2	NA	Grand Index
13	f	17+ years	0.497	807.525	NA	Grand Index
13	f	17+ years	0.498	807.85	NA	Grand Index
13	f	17+ years	0.499	808.175	NA	Grand Index
13	f	17+ years	0.5	808.5	NA	Grand Index
13	f	17+ years	0.501	808.825	NA	Grand Index
13	f	17+ years	0.502	809.15	NA	Grand Index
13	f	17+ years	0.503	809.475	NA	Grand Index
13	f	17+ years	0.504	809.8	NA	Grand Index
13	f	17+ years	0.505	810.125	NA	Grand Index
13	f	17+ years	0.506	810.45	NA	Grand Index
13	f	17+ years	0.507	810.775	NA	Grand Index
13	f	17+ years	0.508	811.12	NA	Grand Index
13	f	17+ years	0.509	811.51	NA	Grand Index
13	f	17+ years	0.51	811.9	NA	Grand Index
13	f	17+ years	0.511	812.29	NA	Grand Index
13	f	17+ years	0.512	812.68	NA	Grand Index
13	f	17+ years	0.513	813.07	NA	Grand Index
13	f	17+ years	0.514	813.46	NA	Grand Index
13	f	17+ years	0.515	813.85	NA	Grand Index
13	f	17+ years	0.516	814.24	NA	Grand Index
13	f	17+ years	0.517	814.63	NA	Grand Index
13	f	17+ years	0.518	815.02	NA	Grand Index
13	f	17+ years	0.519	815.41	NA	Grand Index
13	f	17+ years	0.52	815.8	NA	Grand Index
13	f	17+ years	0.521	816.19	NA	Grand Index
13	f	17+ years	0.522	816.58	NA	Grand Index
13	f	17+ years	0.523	816.97	NA	Grand Index
13	f	17+ years	0.524	817.12	NA	Grand Index
13	f	17+ years	0.525	817.25	NA	Grand Index
13	f	17+ years	0.526	817.38	NA	Grand Index
13	f	17+ years	0.527	817.51	NA	Grand Index
13	f	17+ years	0.528	817.64	NA	Grand Index
13	f	17+ years	0.529	817.77	NA	Grand Index
13	f	17+ years	0.53	817.9	NA	Grand Index
13	f	17+ years	0.531	818.03	NA	Grand Index
13	f	17+ years	0.532	818.16	NA	Grand Index
13	f	17+ years	0.533	818.29	NA	Grand Index
13	f	17+ years	0.534	818.42	NA	Grand Index
13	f	17+ years	0.535	818.55	NA	Grand Index
13	f	17+ years	0.536	818.68	NA	Grand Index
13	f	17+ years	0.537	818.81	NA	Grand Index
13	f	17+ years	0.538	818.94	NA	Grand Index
13	f	17+ years	0.539	819.0215385	NA	Grand Index
13	f	17+ years	0.54	819.0615385	NA	Grand Index
13	f	17+ years	0.541	819.1015385	NA	Grand Index
13	f	17+ years	0.542	819.1415385	NA	Grand Index
13	f	17+ years	0.543	819.1815385	NA	Grand Index
13	f	17+ years	0.544	819.2215385	NA	Grand Index
13	f	17+ years	0.545	819.2615385	NA	Grand Index
13	f	17+ years	0.546	819.3015385	NA	Grand Index
13	f	17+ years	0.547	819.3415385	NA	Grand Index
13	f	17+ years	0.548	819.3815385	NA	Grand Index

Age	Gender	Education	Percentile	Raw Score	Norm Score	Assessment
13	f	17+ years	0.549	819.4215385	NA	Grand Index
13	f	17+ years	0.55	819.4615385	NA	Grand Index
13	f	17+ years	0.551	819.5015385	NA	Grand Index
13	f	17+ years	0.552	819.5415385	NA	Grand Index
13	f	17+ years	0.553	819.5815385	NA	Grand Index
13	f	17+ years	0.554	819.6292308	NA	Grand Index
13	f	17+ years	0.555	819.7192308	NA	Grand Index
13	f	17+ years	0.556	819.8092308	NA	Grand Index
13	f	17+ years	0.557	819.8992308	NA	Grand Index
13	f	17+ years	0.558	819.9892308	NA	Grand Index
13	f	17+ years	0.559	820.0792308	NA	Grand Index
13	f	17+ years	0.56	820.1692308	NA	Grand Index
13	f	17+ years	0.561	820.2592308	NA	Grand Index
13	f	17+ years	0.562	820.3492308	NA	Grand Index
13	f	17+ years	0.563	820.4392308	NA	Grand Index
13	f	17+ years	0.564	820.5292308	NA	Grand Index
13	f	17+ years	0.565	820.6192308	NA	Grand Index
13	f	17+ years	0.566	820.7092308	NA	Grand Index
13	f	17+ years	0.567	820.7992308	NA	Grand Index
13	f	17+ years	0.568	820.8892308	NA	Grand Index
13	f	17+ years	0.569	820.9792308	NA	Grand Index
13	f	17+ years	0.57	821.1095549	NA	Grand Index
13	f	17+ years	0.571	821.2519763	NA	Grand Index
13	f	17+ years	0.572	821.3943977	NA	Grand Index
13	f	17+ years	0.573	821.5368191	NA	Grand Index
13	f	17+ years	0.574	821.6792404	NA	Grand Index
13	f	17+ years	0.575	821.8216618	NA	Grand Index
13	f	17+ years	0.576	821.9640832	NA	Grand Index
13	f	17+ years	0.577	822.1065046	NA	Grand Index
13	f	17+ years	0.578	822.248926	NA	Grand Index
13	f	17+ years	0.579	822.3913474	NA	Grand Index
13	f	17+ years	0.58	822.5337687	NA	Grand Index
13	f	17+ years	0.581	822.6761901	NA	Grand Index
13	f	17+ years	0.582	822.8186115	NA	Grand Index
13	f	17+ years	0.583	822.9610329	NA	Grand Index
13	f	17+ years	0.584	823.1034543	NA	Grand Index
13	f	17+ years	0.585	823.2113208	NA	Grand Index
13	f	17+ years	0.586	823.2638994	NA	Grand Index
13	f	17+ years	0.587	823.316478	NA	Grand Index
13	f	17+ years	0.588	823.3690566	NA	Grand Index
13	f	17+ years	0.589	823.4216352	NA	Grand Index
13	f	17+ years	0.59	823.4742138	NA	Grand Index
13	f	17+ years	0.591	823.5267925	NA	Grand Index
13	f	17+ years	0.592	823.5793711	NA	Grand Index
13	f	17+ years	0.593	823.6319497	NA	Grand Index
13	f	17+ years	0.594	823.6845283	NA	Grand Index
13	f	17+ years	0.595	823.7371069	NA	Grand Index
13	f	17+ years	0.596	823.7896855	NA	Grand Index
13	f	17+ years	0.597	823.8422642	NA	Grand Index
13	f	17+ years	0.598	823.8948428	NA	Grand Index
13	f	17+ years	0.599	823.9474214	NA	Grand Index
13	f	17+ years	0.6	824	NA	Grand Index
13	f	17+ years	0.601	824.325	NA	Grand Index
13	f	17+ years	0.602	824.65	NA	Grand Index
13	f	17+ years	0.603	824.975	NA	Grand Index
13	f	17+ years	0.604	825.3	NA	Grand Index

Age	Gender	Education	Percentile	Raw Score	Norm Score	Assessment
13	f	17+ years	0.605	825.625	NA	Grand Index
13	f	17+ years	0.606	825.95	NA	Grand Index
13	f	17+ years	0.607	826.275	NA	Grand Index
13	f	17+ years	0.608	826.6	NA	Grand Index
13	f	17+ years	0.609	826.925	NA	Grand Index
13	f	17+ years	0.61	827.25	NA	Grand Index
13	f	17+ years	0.611	827.575	NA	Grand Index
13	f	17+ years	0.612	827.9	NA	Grand Index
13	f	17+ years	0.613	828.225	NA	Grand Index
13	f	17+ years	0.614	828.55	NA	Grand Index
13	f	17+ years	0.615	828.875	NA	Grand Index
13	f	17+ years	0.616	829.12	NA	Grand Index
13	f	17+ years	0.617	829.315	NA	Grand Index
13	f	17+ years	0.618	829.51	NA	Grand Index
13	f	17+ years	0.619	829.705	NA	Grand Index
13	f	17+ years	0.62	829.9	NA	Grand Index
13	f	17+ years	0.621	830.095	NA	Grand Index
13	f	17+ years	0.622	830.29	NA	Grand Index
13	f	17+ years	0.623	830.485	NA	Grand Index
13	f	17+ years	0.624	830.68	NA	Grand Index
13	f	17+ years	0.625	830.875	NA	Grand Index
13	f	17+ years	0.626	831.07	NA	Grand Index
13	f	17+ years	0.627	831.265	NA	Grand Index
13	f	17+ years	0.628	831.46	NA	Grand Index
13	f	17+ years	0.629	831.655	NA	Grand Index
13	f	17+ years	0.63	831.85	NA	Grand Index
13	f	17+ years	0.631	832.015	NA	Grand Index
13	f	17+ years	0.632	832.08	NA	Grand Index
13	f	17+ years	0.633	832.145	NA	Grand Index
13	f	17+ years	0.634	832.21	NA	Grand Index
13	f	17+ years	0.635	832.275	NA	Grand Index
13	f	17+ years	0.636	832.34	NA	Grand Index
13	f	17+ years	0.637	832.405	NA	Grand Index
13	f	17+ years	0.638	832.47	NA	Grand Index
13	f	17+ years	0.639	832.535	NA	Grand Index
13	f	17+ years	0.64	832.6	NA	Grand Index
13	f	17+ years	0.641	832.665	NA	Grand Index
13	f	17+ years	0.642	832.73	NA	Grand Index
13	f	17+ years	0.643	832.795	NA	Grand Index
13	f	17+ years	0.644	832.86	NA	Grand Index
13	f	17+ years	0.645	832.925	NA	Grand Index
13	f	17+ years	0.646	832.99	NA	Grand Index
13	f	17+ years	0.647	833.165	NA	Grand Index
13	f	17+ years	0.648	833.36	NA	Grand Index
13	f	17+ years	0.649	833.555	NA	Grand Index
13	f	17+ years	0.65	833.75	NA	Grand Index
13	f	17+ years	0.651	833.945	NA	Grand Index
13	f	17+ years	0.652	834.14	NA	Grand Index
13	f	17+ years	0.653	834.335	NA	Grand Index
13	f	17+ years	0.654	834.53	NA	Grand Index
13	f	17+ years	0.655	834.725	NA	Grand Index
13	f	17+ years	0.656	834.92	NA	Grand Index
13	f	17+ years	0.657	835.115	NA	Grand Index
13	f	17+ years	0.658	835.31	NA	Grand Index
13	f	17+ years	0.659	835.505	NA	Grand Index
13	f	17+ years	0.66	835.7	NA	Grand Index

Age	Gender	Education	Percentile	Raw Score	Norm Score	Assessment
13	f	17+ years	0.661	835.895	NA	Grand Index
13	f	17+ years	0.662	836.03	NA	Grand Index
13	f	17+ years	0.663	836.095	NA	Grand Index
13	f	17+ years	0.664	836.16	NA	Grand Index
13	f	17+ years	0.665	836.225	NA	Grand Index
13	f	17+ years	0.666	836.29	NA	Grand Index
13	f	17+ years	0.667	836.355	NA	Grand Index
13	f	17+ years	0.668	836.42	NA	Grand Index
13	f	17+ years	0.669	836.485	NA	Grand Index
13	f	17+ years	0.67	836.55	NA	Grand Index
13	f	17+ years	0.671	836.615	NA	Grand Index
13	f	17+ years	0.672	836.68	NA	Grand Index
13	f	17+ years	0.673	836.745	NA	Grand Index
13	f	17+ years	0.674	836.81	NA	Grand Index
13	f	17+ years	0.675	836.875	NA	Grand Index
13	f	17+ years	0.676	836.94	NA	Grand Index
13	f	17+ years	0.677	837.01	NA	Grand Index
13	f	17+ years	0.678	837.14	NA	Grand Index
13	f	17+ years	0.679	837.27	NA	Grand Index
13	f	17+ years	0.68	837.4	NA	Grand Index
13	f	17+ years	0.681	837.53	NA	Grand Index
13	f	17+ years	0.682	837.66	NA	Grand Index
13	f	17+ years	0.683	837.79	NA	Grand Index
13	f	17+ years	0.684	837.92	NA	Grand Index
13	f	17+ years	0.685	838.05	NA	Grand Index
13	f	17+ years	0.686	838.18	NA	Grand Index
13	f	17+ years	0.687	838.31	NA	Grand Index
13	f	17+ years	0.688	838.44	NA	Grand Index
13	f	17+ years	0.689	838.57	NA	Grand Index
13	f	17+ years	0.69	838.7	NA	Grand Index
13	f	17+ years	0.691	838.83	NA	Grand Index
13	f	17+ years	0.692	838.96	NA	Grand Index
13	f	17+ years	0.693	839.045	NA	Grand Index
13	f	17+ years	0.694	839.11	NA	Grand Index
13	f	17+ years	0.695	839.175	NA	Grand Index
13	f	17+ years	0.696	839.24	NA	Grand Index
13	f	17+ years	0.697	839.305	NA	Grand Index
13	f	17+ years	0.698	839.37	NA	Grand Index
13	f	17+ years	0.699	839.435	NA	Grand Index
13	f	17+ years	0.7	839.5	NA	Grand Index
13	f	17+ years	0.701	839.565	NA	Grand Index
13	f	17+ years	0.702	839.63	NA	Grand Index
13	f	17+ years	0.703	839.695	NA	Grand Index
13	f	17+ years	0.704	839.76	NA	Grand Index
13	f	17+ years	0.705	839.825	NA	Grand Index
13	f	17+ years	0.706	839.89	NA	Grand Index
13	f	17+ years	0.707	839.955	NA	Grand Index
13	f	17+ years	0.708	840.02	NA	Grand Index
13	f	17+ years	0.709	840.085	NA	Grand Index
13	f	17+ years	0.71	840.15	NA	Grand Index
13	f	17+ years	0.711	840.215	NA	Grand Index
13	f	17+ years	0.712	840.28	NA	Grand Index
13	f	17+ years	0.713	840.345	NA	Grand Index
13	f	17+ years	0.714	840.41	NA	Grand Index
13	f	17+ years	0.715	840.475	NA	Grand Index
13	f	17+ years	0.716	840.54	NA	Grand Index

Age	Gender	Education	Percentile	Raw Score	Norm Score	Assessment
13	f	17+ years	0.717	840.605	NA	Grand Index
13	f	17+ years	0.718	840.67	NA	Grand Index
13	f	17+ years	0.719	840.735	NA	Grand Index
13	f	17+ years	0.72	840.8	NA	Grand Index
13	f	17+ years	0.721	840.865	NA	Grand Index
13	f	17+ years	0.722	840.93	NA	Grand Index
13	f	17+ years	0.723	840.995	NA	Grand Index
13	f	17+ years	0.724	841.3	NA	Grand Index
13	f	17+ years	0.725	841.625	NA	Grand Index
13	f	17+ years	0.726	841.95	NA	Grand Index
13	f	17+ years	0.727	842.275	NA	Grand Index
13	f	17+ years	0.728	842.6	NA	Grand Index
13	f	17+ years	0.729	842.925	NA	Grand Index
13	f	17+ years	0.73	843.25	NA	Grand Index
13	f	17+ years	0.731	843.575	NA	Grand Index
13	f	17+ years	0.732	843.9	NA	Grand Index
13	f	17+ years	0.733	844.225	NA	Grand Index
13	f	17+ years	0.734	844.55	NA	Grand Index
13	f	17+ years	0.735	844.875	NA	Grand Index
13	f	17+ years	0.736	845.2	NA	Grand Index
13	f	17+ years	0.737	845.525	NA	Grand Index
13	f	17+ years	0.738	845.85	NA	Grand Index
13	f	17+ years	0.739	846.07	NA	Grand Index
13	f	17+ years	0.74	846.2	NA	Grand Index
13	f	17+ years	0.741	846.33	NA	Grand Index
13	f	17+ years	0.742	846.46	NA	Grand Index
13	f	17+ years	0.743	846.59	NA	Grand Index
13	f	17+ years	0.744	846.72	NA	Grand Index
13	f	17+ years	0.745	846.85	NA	Grand Index
13	f	17+ years	0.746	846.98	NA	Grand Index
13	f	17+ years	0.747	847.11	NA	Grand Index
13	f	17+ years	0.748	847.24	NA	Grand Index
13	f	17+ years	0.749	847.37	NA	Grand Index
13	f	17+ years	0.75	847.5	NA	Grand Index
13	f	17+ years	0.751	847.63	NA	Grand Index
13	f	17+ years	0.752	847.76	NA	Grand Index
13	f	17+ years	0.753	847.89	NA	Grand Index
13	f	17+ years	0.754	848.04	NA	Grand Index
13	f	17+ years	0.755	848.3	NA	Grand Index
13	f	17+ years	0.756	848.56	NA	Grand Index
13	f	17+ years	0.757	848.82	NA	Grand Index
13	f	17+ years	0.758	849.08	NA	Grand Index
13	f	17+ years	0.759	849.34	NA	Grand Index
13	f	17+ years	0.76	849.6	NA	Grand Index
13	f	17+ years	0.761	849.86	NA	Grand Index
13	f	17+ years	0.762	850.12	NA	Grand Index
13	f	17+ years	0.763	850.38	NA	Grand Index
13	f	17+ years	0.764	850.64	NA	Grand Index
13	f	17+ years	0.765	850.9	NA	Grand Index
13	f	17+ years	0.766	851.16	NA	Grand Index
13	f	17+ years	0.767	851.42	NA	Grand Index
13	f	17+ years	0.768	851.68	NA	Grand Index
13	f	17+ years	0.769	851.94	NA	Grand Index
13	f	17+ years	0.77	852.1	NA	Grand Index
13	f	17+ years	0.771	852.23	NA	Grand Index
13	f	17+ years	0.772	852.36	NA	Grand Index

Age	Gender	Education	Percentile	Raw Score	Norm Score	Assessment
13	f	17+ years	0.773	852.49	NA	Grand Index
13	f	17+ years	0.774	852.62	NA	Grand Index
13	f	17+ years	0.775	852.75	NA	Grand Index
13	f	17+ years	0.776	852.88	NA	Grand Index
13	f	17+ years	0.777	853.01	NA	Grand Index
13	f	17+ years	0.778	853.14	NA	Grand Index
13	f	17+ years	0.779	853.27	NA	Grand Index
13	f	17+ years	0.78	853.4	NA	Grand Index
13	f	17+ years	0.781	853.53	NA	Grand Index
13	f	17+ years	0.782	853.66	NA	Grand Index
13	f	17+ years	0.783	853.79	NA	Grand Index
13	f	17+ years	0.784	853.92	NA	Grand Index
13	f	17+ years	0.785	854.2456897	NA	Grand Index
13	f	17+ years	0.786	854.8844828	NA	Grand Index
13	f	17+ years	0.787	855.5232759	NA	Grand Index
13	f	17+ years	0.788	856.162069	NA	Grand Index
13	f	17+ years	0.789	856.8008621	NA	Grand Index
13	f	17+ years	0.79	857.4396552	NA	Grand Index
13	f	17+ years	0.791	858.0784483	NA	Grand Index
13	f	17+ years	0.792	858.7172414	NA	Grand Index
13	f	17+ years	0.793	859.3560345	NA	Grand Index
13	f	17+ years	0.794	859.9948276	NA	Grand Index
13	f	17+ years	0.795	860.6336207	NA	Grand Index
13	f	17+ years	0.796	861.2724138	NA	Grand Index
13	f	17+ years	0.797	861.9112069	NA	Grand Index
13	f	17+ years	0.798	862.55	NA	Grand Index
13	f	17+ years	0.799	863.1887931	NA	Grand Index
13	f	17+ years	0.8	863.8275862	NA	Grand Index
13	f	17+ years	0.801	864.0987931	NA	Grand Index
13	f	17+ years	0.802	864.37	NA	Grand Index
13	f	17+ years	0.803	864.6412069	NA	Grand Index
13	f	17+ years	0.804	864.9124138	NA	Grand Index
13	f	17+ years	0.805	865.1836207	NA	Grand Index
13	f	17+ years	0.806	865.4548276	NA	Grand Index
13	f	17+ years	0.807	865.7260345	NA	Grand Index
13	f	17+ years	0.808	865.9972414	NA	Grand Index
13	f	17+ years	0.809	866.2684483	NA	Grand Index
13	f	17+ years	0.81	866.5396552	NA	Grand Index
13	f	17+ years	0.811	866.8108621	NA	Grand Index
13	f	17+ years	0.812	867.082069	NA	Grand Index
13	f	17+ years	0.813	867.3532759	NA	Grand Index
13	f	17+ years	0.814	867.6244828	NA	Grand Index
13	f	17+ years	0.815	867.8956897	NA	Grand Index
13	f	17+ years	0.816	868.0646154	NA	Grand Index
13	f	17+ years	0.817	868.1696154	NA	Grand Index
13	f	17+ years	0.818	868.2746154	NA	Grand Index
13	f	17+ years	0.819	868.3796154	NA	Grand Index
13	f	17+ years	0.82	868.4846154	NA	Grand Index
13	f	17+ years	0.821	868.5896154	NA	Grand Index
13	f	17+ years	0.822	868.6946154	NA	Grand Index
13	f	17+ years	0.823	868.7996154	NA	Grand Index
13	f	17+ years	0.824	868.9046154	NA	Grand Index
13	f	17+ years	0.825	869.0096154	NA	Grand Index
13	f	17+ years	0.826	869.1146154	NA	Grand Index
13	f	17+ years	0.827	869.2196154	NA	Grand Index
13	f	17+ years	0.828	869.3246154	NA	Grand Index

Age	Gender	Education	Percentile	Raw Score	Norm Score	Assessment
13	f	17+ years	0.829	869.4296154	NA	Grand Index
13	f	17+ years	0.83	869.5346154	NA	Grand Index
13	f	17+ years	0.831	869.7411538	NA	Grand Index
13	f	17+ years	0.832	870.2861538	NA	Grand Index
13	f	17+ years	0.833	870.8311538	NA	Grand Index
13	f	17+ years	0.834	871.3761538	NA	Grand Index
13	f	17+ years	0.835	871.9211538	NA	Grand Index
13	f	17+ years	0.836	872.4661538	NA	Grand Index
13	f	17+ years	0.837	873.0111538	NA	Grand Index
13	f	17+ years	0.838	873.5561538	NA	Grand Index
13	f	17+ years	0.839	874.1011538	NA	Grand Index
13	f	17+ years	0.84	874.6461538	NA	Grand Index
13	f	17+ years	0.841	875.1911538	NA	Grand Index
13	f	17+ years	0.842	875.7361538	NA	Grand Index
13	f	17+ years	0.843	876.2811538	NA	Grand Index
13	f	17+ years	0.844	876.8261538	NA	Grand Index
13	f	17+ years	0.845	877.3711538	NA	Grand Index
13	f	17+ years	0.846	877.9161538	NA	Grand Index
13	f	17+ years	0.847	879.54	NA	Grand Index
13	f	17+ years	0.848	881.36	NA	Grand Index
13	f	17+ years	0.849	883.18	NA	Grand Index
13	f	17+ years	0.85	885	NA	Grand Index
13	f	17+ years	0.851	886.82	NA	Grand Index
13	f	17+ years	0.852	888.64	NA	Grand Index
13	f	17+ years	0.853	890.46	NA	Grand Index
13	f	17+ years	0.854	892.28	NA	Grand Index
13	f	17+ years	0.855	894.1	NA	Grand Index
13	f	17+ years	0.856	895.92	NA	Grand Index
13	f	17+ years	0.857	897.74	NA	Grand Index
13	f	17+ years	0.858	899.56	NA	Grand Index
13	f	17+ years	0.859	901.38	NA	Grand Index
13	f	17+ years	0.86	903.2	NA	Grand Index
13	f	17+ years	0.861	905.02	NA	Grand Index
13	f	17+ years	0.862	906.75	NA	Grand Index
13	f	17+ years	0.863	908.375	NA	Grand Index
13	f	17+ years	0.864	910	NA	Grand Index
13	f	17+ years	0.865	911.625	NA	Grand Index
13	f	17+ years	0.866	913.25	NA	Grand Index
13	f	17+ years	0.867	914.875	NA	Grand Index
13	f	17+ years	0.868	916.5	NA	Grand Index
13	f	17+ years	0.869	918.125	NA	Grand Index
13	f	17+ years	0.87	919.75	NA	Grand Index
13	f	17+ years	0.871	921.375	NA	Grand Index
13	f	17+ years	0.872	923	NA	Grand Index
13	f	17+ years	0.873	924.625	NA	Grand Index
13	f	17+ years	0.874	926.25	NA	Grand Index
13	f	17+ years	0.875	927.875	NA	Grand Index
13	f	17+ years	0.876	929.5	NA	Grand Index
13	f	17+ years	0.877	931.035	NA	Grand Index
13	f	17+ years	0.878	931.49	NA	Grand Index
13	f	17+ years	0.879	931.945	NA	Grand Index
13	f	17+ years	0.88	932.4	NA	Grand Index
13	f	17+ years	0.881	932.855	NA	Grand Index
13	f	17+ years	0.882	933.31	NA	Grand Index
13	f	17+ years	0.883	933.765	NA	Grand Index
13	f	17+ years	0.884	934.22	NA	Grand Index

Age	Gender	Education	Percentile	Raw Score	Norm Score	Assessment
13	f	17+ years	0.885	934.675	NA	Grand Index
13	f	17+ years	0.886	935.13	NA	Grand Index
13	f	17+ years	0.887	935.585	NA	Grand Index
13	f	17+ years	0.888	936.04	NA	Grand Index
13	f	17+ years	0.889	936.495	NA	Grand Index
13	f	17+ years	0.89	936.95	NA	Grand Index
13	f	17+ years	0.891	937.405	NA	Grand Index
13	f	17+ years	0.892	937.86	NA	Grand Index
13	f	17+ years	0.893	938.240339	NA	Grand Index
13	f	17+ years	0.894	938.5874954	NA	Grand Index
13	f	17+ years	0.895	938.9346518	NA	Grand Index
13	f	17+ years	0.896	939.2818081	NA	Grand Index
13	f	17+ years	0.897	939.6289645	NA	Grand Index
13	f	17+ years	0.898	939.9761209	NA	Grand Index
13	f	17+ years	0.899	940.3232772	NA	Grand Index
13	f	17+ years	0.9	940.6704336	NA	Grand Index
13	f	17+ years	0.901	941.01759	NA	Grand Index
13	f	17+ years	0.902	941.3647463	NA	Grand Index
13	f	17+ years	0.903	941.7119027	NA	Grand Index
13	f	17+ years	0.904	942.0590591	NA	Grand Index
13	f	17+ years	0.905	942.4062154	NA	Grand Index
13	f	17+ years	0.906	942.7533718	NA	Grand Index
13	f	17+ years	0.907	943.1005282	NA	Grand Index
13	f	17+ years	0.908	943.3740499	NA	Grand Index
13	f	17+ years	0.909	943.4818935	NA	Grand Index
13	f	17+ years	0.91	943.5897371	NA	Grand Index
13	f	17+ years	0.911	943.6975808	NA	Grand Index
13	f	17+ years	0.912	943.8054244	NA	Grand Index
13	f	17+ years	0.913	943.913268	NA	Grand Index
13	f	17+ years	0.914	944.0211116	NA	Grand Index
13	f	17+ years	0.915	944.1289553	NA	Grand Index
13	f	17+ years	0.916	944.2367989	NA	Grand Index
13	f	17+ years	0.917	944.3446425	NA	Grand Index
13	f	17+ years	0.918	944.4524862	NA	Grand Index
13	f	17+ years	0.919	944.5603298	NA	Grand Index
13	f	17+ years	0.92	944.6681734	NA	Grand Index
13	f	17+ years	0.921	944.7760171	NA	Grand Index
13	f	17+ years	0.922	944.8838607	NA	Grand Index
13	f	17+ years	0.923	944.9917043	NA	Grand Index
13	f	17+ years	0.924	945.3	NA	Grand Index
13	f	17+ years	0.925	945.625	NA	Grand Index
13	f	17+ years	0.926	945.95	NA	Grand Index
13	f	17+ years	0.927	946.275	NA	Grand Index
13	f	17+ years	0.928	946.6	NA	Grand Index
13	f	17+ years	0.929	946.925	NA	Grand Index
13	f	17+ years	0.93	947.25	NA	Grand Index
13	f	17+ years	0.931	947.575	NA	Grand Index
13	f	17+ years	0.932	947.9	NA	Grand Index
13	f	17+ years	0.933	948.225	NA	Grand Index
13	f	17+ years	0.934	948.55	NA	Grand Index
13	f	17+ years	0.935	948.875	NA	Grand Index
13	f	17+ years	0.936	949.2	NA	Grand Index
13	f	17+ years	0.937	949.525	NA	Grand Index
13	f	17+ years	0.938	949.85	NA	Grand Index
13	f	17+ years	0.939	950.035	NA	Grand Index
13	f	17+ years	0.94	950.1	NA	Grand Index

Age	Gender	Education	Percentile	Raw Score	Norm Score	Assessment
13	f	17+ years	0.941	950.165	NA	Grand Index
13	f	17+ years	0.942	950.23	NA	Grand Index
13	f	17+ years	0.943	950.295	NA	Grand Index
13	f	17+ years	0.944	950.36	NA	Grand Index
13	f	17+ years	0.945	950.425	NA	Grand Index
13	f	17+ years	0.946	950.49	NA	Grand Index
13	f	17+ years	0.947	950.555	NA	Grand Index
13	f	17+ years	0.948	950.62	NA	Grand Index
13	f	17+ years	0.949	950.685	NA	Grand Index
13	f	17+ years	0.95	950.75	NA	Grand Index
13	f	17+ years	0.951	950.815	NA	Grand Index
13	f	17+ years	0.952	950.88	NA	Grand Index
13	f	17+ years	0.953	950.945	NA	Grand Index
13	f	17+ years	0.954	951.02	NA	Grand Index
13	f	17+ years	0.955	951.15	NA	Grand Index
13	f	17+ years	0.956	951.28	NA	Grand Index
13	f	17+ years	0.957	951.41	NA	Grand Index
13	f	17+ years	0.958	951.54	NA	Grand Index
13	f	17+ years	0.959	951.67	NA	Grand Index
13	f	17+ years	0.96	951.8	NA	Grand Index
13	f	17+ years	0.961	951.93	NA	Grand Index
13	f	17+ years	0.962	952.06	NA	Grand Index
13	f	17+ years	0.963	952.19	NA	Grand Index
13	f	17+ years	0.964	952.32	NA	Grand Index
13	f	17+ years	0.965	952.45	NA	Grand Index
13	f	17+ years	0.966	952.58	NA	Grand Index
13	f	17+ years	0.967	952.71	NA	Grand Index
13	f	17+ years	0.968	952.84	NA	Grand Index
13	f	17+ years	0.969	952.97	NA	Grand Index
13	f	17+ years	0.97	954.25	NA	Grand Index
13	f	17+ years	0.971	955.875	NA	Grand Index
13	f	17+ years	0.972	957.5	NA	Grand Index
13	f	17+ years	0.973	959.125	NA	Grand Index
13	f	17+ years	0.974	960.75	NA	Grand Index
13	f	17+ years	0.975	962.375	NA	Grand Index
13	f	17+ years	0.976	964	NA	Grand Index
13	f	17+ years	0.977	965.625	NA	Grand Index
13	f	17+ years	0.978	967.25	NA	Grand Index
13	f	17+ years	0.979	968.875	NA	Grand Index
13	f	17+ years	0.98	970.5	NA	Grand Index
13	f	17+ years	0.981	972.125	NA	Grand Index
13	f	17+ years	0.982	973.75	NA	Grand Index
13	f	17+ years	0.983	975.375	NA	Grand Index
13	f	17+ years	0.984	977	NA	Grand Index
13	f	17+ years	0.985	978.825	NA	Grand Index
13	f	17+ years	0.986	980.97	NA	Grand Index
13	f	17+ years	0.987	983.115	NA	Grand Index
13	f	17+ years	0.988	985.26	NA	Grand Index
13	f	17+ years	0.989	987.405	NA	Grand Index
13	f	17+ years	0.99	989.55	NA	Grand Index
13	f	17+ years	0.991	991.695	NA	Grand Index
13	f	17+ years	0.992	993.84	NA	Grand Index
13	f	17+ years	0.993	995.985	NA	Grand Index
13	f	17+ years	0.994	998.13	NA	Grand Index
13	f	17+ years	0.995	1000.275	NA	Grand Index
13	f	17+ years	0.996	1002.42	NA	Grand Index

Age	Gender	Education	Percentile	Raw Score	Norm Score	Assessment
13	f	17+ years	0.997	1004.565	NA	Grand Index
13	f	17+ years	0.998	1006.71	NA	Grand Index
13	f	17+ years	0.999	1008.855	NA	Grand Index
13	m	17+ years	0.001	571.68	NA	Grand Index
13	m	17+ years	0.002	576.36	NA	Grand Index
13	m	17+ years	0.003	581.04	NA	Grand Index
13	m	17+ years	0.004	585.72	NA	Grand Index
13	m	17+ years	0.005	590.4	NA	Grand Index
13	m	17+ years	0.006	595.08	NA	Grand Index
13	m	17+ years	0.007	599.76	NA	Grand Index
13	m	17+ years	0.008	604.44	NA	Grand Index
13	m	17+ years	0.009	609.12	NA	Grand Index
13	m	17+ years	0.01	613.8	NA	Grand Index
13	m	17+ years	0.011	618.48	NA	Grand Index
13	m	17+ years	0.012	623.16	NA	Grand Index
13	m	17+ years	0.013	627.84	NA	Grand Index
13	m	17+ years	0.014	632.0481604	NA	Grand Index
13	m	17+ years	0.015	632.4816036	NA	Grand Index
13	m	17+ years	0.016	632.9150468	NA	Grand Index
13	m	17+ years	0.017	633.34849	NA	Grand Index
13	m	17+ years	0.018	633.7819332	NA	Grand Index
13	m	17+ years	0.019	634.2153764	NA	Grand Index
13	m	17+ years	0.02	634.6488196	NA	Grand Index
13	m	17+ years	0.021	635.0822628	NA	Grand Index
13	m	17+ years	0.022	635.515706	NA	Grand Index
13	m	17+ years	0.023	635.9491492	NA	Grand Index
13	m	17+ years	0.024	636.3825924	NA	Grand Index
13	m	17+ years	0.025	636.8160356	NA	Grand Index
13	m	17+ years	0.026	637.2494788	NA	Grand Index
13	m	17+ years	0.027	637.682922	NA	Grand Index
13	m	17+ years	0.028	638.4825982	NA	Grand Index
13	m	17+ years	0.029	640.5640897	NA	Grand Index
13	m	17+ years	0.03	642.6455811	NA	Grand Index
13	m	17+ years	0.031	644.7270726	NA	Grand Index
13	m	17+ years	0.032	646.8085641	NA	Grand Index
13	m	17+ years	0.033	648.8900555	NA	Grand Index
13	m	17+ years	0.034	650.971547	NA	Grand Index
13	m	17+ years	0.035	653.0530385	NA	Grand Index
13	m	17+ years	0.036	655.1345299	NA	Grand Index
13	m	17+ years	0.037	657.2160214	NA	Grand Index
13	m	17+ years	0.038	659.2975129	NA	Grand Index
13	m	17+ years	0.039	661.3790043	NA	Grand Index
13	m	17+ years	0.04	663.4604958	NA	Grand Index
13	m	17+ years	0.041	665.5419873	NA	Grand Index
13	m	17+ years	0.042	667.1953367	NA	Grand Index
13	m	17+ years	0.043	667.992402	NA	Grand Index
13	m	17+ years	0.044	668.7894673	NA	Grand Index
13	m	17+ years	0.045	669.5865327	NA	Grand Index
13	m	17+ years	0.046	670.383598	NA	Grand Index
13	m	17+ years	0.047	671.1806633	NA	Grand Index
13	m	17+ years	0.048	671.9777286	NA	Grand Index
13	m	17+ years	0.049	672.774794	NA	Grand Index
13	m	17+ years	0.05	673.5718593	NA	Grand Index
13	m	17+ years	0.051	674.3689246	NA	Grand Index
13	m	17+ years	0.052	675.1659899	NA	Grand Index
13	m	17+ years	0.053	675.9630553	NA	Grand Index

Age	Gender	Education	Percentile	Raw Score	Norm Score	Assessment
13	m	17+ years	0.054	676.7601206	NA	Grand Index
13	m	17+ years	0.055	677.5571859	NA	Grand Index
13	m	17+ years	0.056	678.224	NA	Grand Index
13	m	17+ years	0.057	678.728	NA	Grand Index
13	m	17+ years	0.058	679.232	NA	Grand Index
13	m	17+ years	0.059	679.736	NA	Grand Index
13	m	17+ years	0.06	680.24	NA	Grand Index
13	m	17+ years	0.061	680.744	NA	Grand Index
13	m	17+ years	0.062	681.248	NA	Grand Index
13	m	17+ years	0.063	681.752	NA	Grand Index
13	m	17+ years	0.064	682.256	NA	Grand Index
13	m	17+ years	0.065	682.76	NA	Grand Index
13	m	17+ years	0.066	683.264	NA	Grand Index
13	m	17+ years	0.067	683.768	NA	Grand Index
13	m	17+ years	0.068	684.272	NA	Grand Index
13	m	17+ years	0.069	684.776	NA	Grand Index
13	m	17+ years	0.07	685.08	NA	Grand Index
13	m	17+ years	0.071	685.224	NA	Grand Index
13	m	17+ years	0.072	685.368	NA	Grand Index
13	m	17+ years	0.073	685.512	NA	Grand Index
13	m	17+ years	0.074	685.656	NA	Grand Index
13	m	17+ years	0.075	685.8	NA	Grand Index
13	m	17+ years	0.076	685.944	NA	Grand Index
13	m	17+ years	0.077	686.088	NA	Grand Index
13	m	17+ years	0.078	686.232	NA	Grand Index
13	m	17+ years	0.079	686.376	NA	Grand Index
13	m	17+ years	0.08	686.52	NA	Grand Index
13	m	17+ years	0.081	686.664	NA	Grand Index
13	m	17+ years	0.082	686.808	NA	Grand Index
13	m	17+ years	0.083	686.952	NA	Grand Index
13	m	17+ years	0.084	687.048	NA	Grand Index
13	m	17+ years	0.085	687.12	NA	Grand Index
13	m	17+ years	0.086	687.192	NA	Grand Index
13	m	17+ years	0.087	687.264	NA	Grand Index
13	m	17+ years	0.088	687.336	NA	Grand Index
13	m	17+ years	0.089	687.408	NA	Grand Index
13	m	17+ years	0.09	687.48	NA	Grand Index
13	m	17+ years	0.091	687.552	NA	Grand Index
13	m	17+ years	0.092	687.624	NA	Grand Index
13	m	17+ years	0.093	687.696	NA	Grand Index
13	m	17+ years	0.094	687.768	NA	Grand Index
13	m	17+ years	0.095	687.84	NA	Grand Index
13	m	17+ years	0.096	687.912	NA	Grand Index
13	m	17+ years	0.097	687.984	NA	Grand Index
13	m	17+ years	0.098	688.448	NA	Grand Index
13	m	17+ years	0.099	689.024	NA	Grand Index
13	m	17+ years	0.1	689.6	NA	Grand Index
13	m	17+ years	0.101	690.176	NA	Grand Index
13	m	17+ years	0.102	690.752	NA	Grand Index
13	m	17+ years	0.103	691.328	NA	Grand Index
13	m	17+ years	0.104	691.904	NA	Grand Index
13	m	17+ years	0.105	692.48	NA	Grand Index
13	m	17+ years	0.106	693.056	NA	Grand Index
13	m	17+ years	0.107	693.632	NA	Grand Index
13	m	17+ years	0.108	694.208	NA	Grand Index
13	m	17+ years	0.109	694.784	NA	Grand Index

Age	Gender	Education	Percentile	Raw Score	Norm Score	Assessment
13	m	17+ years	0.11	695.36	NA	Grand Index
13	m	17+ years	0.111	695.936	NA	Grand Index
13	m	17+ years	0.112	697.024	NA	Grand Index
13	m	17+ years	0.113	698.176	NA	Grand Index
13	m	17+ years	0.114	699.328	NA	Grand Index
13	m	17+ years	0.115	700.48	NA	Grand Index
13	m	17+ years	0.116	701.632	NA	Grand Index
13	m	17+ years	0.117	702.784	NA	Grand Index
13	m	17+ years	0.118	703.936	NA	Grand Index
13	m	17+ years	0.119	705.088	NA	Grand Index
13	m	17+ years	0.12	706.24	NA	Grand Index
13	m	17+ years	0.121	707.392	NA	Grand Index
13	m	17+ years	0.122	708.544	NA	Grand Index
13	m	17+ years	0.123	709.696	NA	Grand Index
13	m	17+ years	0.124	710.848	NA	Grand Index
13	m	17+ years	0.125	712	NA	Grand Index
13	m	17+ years	0.126	712.144	NA	Grand Index
13	m	17+ years	0.127	712.288	NA	Grand Index
13	m	17+ years	0.128	712.432	NA	Grand Index
13	m	17+ years	0.129	712.576	NA	Grand Index
13	m	17+ years	0.13	712.72	NA	Grand Index
13	m	17+ years	0.131	712.864	NA	Grand Index
13	m	17+ years	0.132	713.008	NA	Grand Index
13	m	17+ years	0.133	713.152	NA	Grand Index
13	m	17+ years	0.134	713.296	NA	Grand Index
13	m	17+ years	0.135	713.44	NA	Grand Index
13	m	17+ years	0.136	713.584	NA	Grand Index
13	m	17+ years	0.137	713.728	NA	Grand Index
13	m	17+ years	0.138	713.872	NA	Grand Index
13	m	17+ years	0.139	714.136	NA	Grand Index
13	m	17+ years	0.14	715.36	NA	Grand Index
13	m	17+ years	0.141	716.584	NA	Grand Index
13	m	17+ years	0.142	717.808	NA	Grand Index
13	m	17+ years	0.143	719.032	NA	Grand Index
13	m	17+ years	0.144	720.256	NA	Grand Index
13	m	17+ years	0.145	721.48	NA	Grand Index
13	m	17+ years	0.146	722.704	NA	Grand Index
13	m	17+ years	0.147	723.928	NA	Grand Index
13	m	17+ years	0.148	725.152	NA	Grand Index
13	m	17+ years	0.149	726.376	NA	Grand Index
13	m	17+ years	0.15	727.6	NA	Grand Index
13	m	17+ years	0.151	728.824	NA	Grand Index
13	m	17+ years	0.152	730.048	NA	Grand Index
13	m	17+ years	0.153	731.048	NA	Grand Index
13	m	17+ years	0.154	731.264	NA	Grand Index
13	m	17+ years	0.155	731.48	NA	Grand Index
13	m	17+ years	0.156	731.696	NA	Grand Index
13	m	17+ years	0.157	731.912	NA	Grand Index
13	m	17+ years	0.158	732.128	NA	Grand Index
13	m	17+ years	0.159	732.344	NA	Grand Index
13	m	17+ years	0.16	732.56	NA	Grand Index
13	m	17+ years	0.161	732.776	NA	Grand Index
13	m	17+ years	0.162	732.992	NA	Grand Index
13	m	17+ years	0.163	733.208	NA	Grand Index
13	m	17+ years	0.164	733.424	NA	Grand Index
13	m	17+ years	0.165	733.64	NA	Grand Index

Age	Gender	Education	Percentile	Raw Score	Norm Score	Assessment
13	m	17+ years	0.166	733.856	NA	Grand Index
13	m	17+ years	0.167	734.144	NA	Grand Index
13	m	17+ years	0.168	734.576	NA	Grand Index
13	m	17+ years	0.169	735.008	NA	Grand Index
13	m	17+ years	0.17	735.44	NA	Grand Index
13	m	17+ years	0.171	735.872	NA	Grand Index
13	m	17+ years	0.172	736.304	NA	Grand Index
13	m	17+ years	0.173	736.736	NA	Grand Index
13	m	17+ years	0.174	737.168	NA	Grand Index
13	m	17+ years	0.175	737.6	NA	Grand Index
13	m	17+ years	0.176	738.032	NA	Grand Index
13	m	17+ years	0.177	738.464	NA	Grand Index
13	m	17+ years	0.178	738.896	NA	Grand Index
13	m	17+ years	0.179	739.328	NA	Grand Index
13	m	17+ years	0.18	739.76	NA	Grand Index
13	m	17+ years	0.181	740.64	NA	Grand Index
13	m	17+ years	0.182	742.08	NA	Grand Index
13	m	17+ years	0.183	743.52	NA	Grand Index
13	m	17+ years	0.184	744.96	NA	Grand Index
13	m	17+ years	0.185	746.4	NA	Grand Index
13	m	17+ years	0.186	747.84	NA	Grand Index
13	m	17+ years	0.187	749.28	NA	Grand Index
13	m	17+ years	0.188	750.72	NA	Grand Index
13	m	17+ years	0.189	752.16	NA	Grand Index
13	m	17+ years	0.19	753.6	NA	Grand Index
13	m	17+ years	0.191	755.04	NA	Grand Index
13	m	17+ years	0.192	756.48	NA	Grand Index
13	m	17+ years	0.193	757.92	NA	Grand Index
13	m	17+ years	0.194	759.36	NA	Grand Index
13	m	17+ years	0.195	760.08	NA	Grand Index
13	m	17+ years	0.196	760.224	NA	Grand Index
13	m	17+ years	0.197	760.368	NA	Grand Index
13	m	17+ years	0.198	760.512	NA	Grand Index
13	m	17+ years	0.199	760.656	NA	Grand Index
13	m	17+ years	0.2	760.8	NA	Grand Index
13	m	17+ years	0.201	760.944	NA	Grand Index
13	m	17+ years	0.202	761.088	NA	Grand Index
13	m	17+ years	0.203	761.232	NA	Grand Index
13	m	17+ years	0.204	761.376	NA	Grand Index
13	m	17+ years	0.205	761.52	NA	Grand Index
13	m	17+ years	0.206	761.664	NA	Grand Index
13	m	17+ years	0.207	761.808	NA	Grand Index
13	m	17+ years	0.208	761.952	NA	Grand Index
13	m	17+ years	0.209	762.2327258	NA	Grand Index
13	m	17+ years	0.21	762.5818146	NA	Grand Index
13	m	17+ years	0.211	762.9309033	NA	Grand Index
13	m	17+ years	0.212	763.2799921	NA	Grand Index
13	m	17+ years	0.213	763.6290808	NA	Grand Index
13	m	17+ years	0.214	763.9781696	NA	Grand Index
13	m	17+ years	0.215	764.3272583	NA	Grand Index
13	m	17+ years	0.216	764.6763471	NA	Grand Index
13	m	17+ years	0.217	765.0254358	NA	Grand Index
13	m	17+ years	0.218	765.3745246	NA	Grand Index
13	m	17+ years	0.219	765.7236133	NA	Grand Index
13	m	17+ years	0.22	766.0727021	NA	Grand Index
13	m	17+ years	0.221	766.4217909	NA	Grand Index

Age	Gender	Education	Percentile	Raw Score	Norm Score	Assessment
13	m	17+ years	0.222	766.7708796	NA	Grand Index
13	m	17+ years	0.223	767.1902351	NA	Grand Index
13	m	17+ years	0.224	767.6296668	NA	Grand Index
13	m	17+ years	0.225	768.0690985	NA	Grand Index
13	m	17+ years	0.226	768.5085302	NA	Grand Index
13	m	17+ years	0.227	768.9479619	NA	Grand Index
13	m	17+ years	0.228	769.3873936	NA	Grand Index
13	m	17+ years	0.229	769.8268253	NA	Grand Index
13	m	17+ years	0.23	770.266257	NA	Grand Index
13	m	17+ years	0.231	770.7056886	NA	Grand Index
13	m	17+ years	0.232	771.1451203	NA	Grand Index
13	m	17+ years	0.233	771.584552	NA	Grand Index
13	m	17+ years	0.234	772.0239837	NA	Grand Index
13	m	17+ years	0.235	772.4634154	NA	Grand Index
13	m	17+ years	0.236	772.9028471	NA	Grand Index
13	m	17+ years	0.237	773.4027658	NA	Grand Index
13	m	17+ years	0.238	773.9102454	NA	Grand Index
13	m	17+ years	0.239	774.4177249	NA	Grand Index
13	m	17+ years	0.24	774.9252045	NA	Grand Index
13	m	17+ years	0.241	775.432684	NA	Grand Index
13	m	17+ years	0.242	775.9401636	NA	Grand Index
13	m	17+ years	0.243	776.4476431	NA	Grand Index
13	m	17+ years	0.244	776.9551227	NA	Grand Index
13	m	17+ years	0.245	777.4626022	NA	Grand Index
13	m	17+ years	0.246	777.9700818	NA	Grand Index
13	m	17+ years	0.247	778.4775613	NA	Grand Index
13	m	17+ years	0.248	778.9850409	NA	Grand Index
13	m	17+ years	0.249	779.4925204	NA	Grand Index
13	m	17+ years	0.25	780	NA	Grand Index
13	m	17+ years	0.251	780.216	NA	Grand Index
13	m	17+ years	0.252	780.432	NA	Grand Index
13	m	17+ years	0.253	780.648	NA	Grand Index
13	m	17+ years	0.254	780.864	NA	Grand Index
13	m	17+ years	0.255	781.08	NA	Grand Index
13	m	17+ years	0.256	781.296	NA	Grand Index
13	m	17+ years	0.257	781.512	NA	Grand Index
13	m	17+ years	0.258	781.728	NA	Grand Index
13	m	17+ years	0.259	781.944	NA	Grand Index
13	m	17+ years	0.26	782.16	NA	Grand Index
13	m	17+ years	0.261	782.376	NA	Grand Index
13	m	17+ years	0.262	782.592	NA	Grand Index
13	m	17+ years	0.263	782.808	NA	Grand Index
13	m	17+ years	0.264	783.0000232	NA	Grand Index
13	m	17+ years	0.265	783.0002319	NA	Grand Index
13	m	17+ years	0.266	783.0004406	NA	Grand Index
13	m	17+ years	0.267	783.0006493	NA	Grand Index
13	m	17+ years	0.268	783.000858	NA	Grand Index
13	m	17+ years	0.269	783.0010667	NA	Grand Index
13	m	17+ years	0.27	783.0012754	NA	Grand Index
13	m	17+ years	0.271	783.0014841	NA	Grand Index
13	m	17+ years	0.272	783.0016928	NA	Grand Index
13	m	17+ years	0.273	783.0019014	NA	Grand Index
13	m	17+ years	0.274	783.0021101	NA	Grand Index
13	m	17+ years	0.275	783.0023188	NA	Grand Index
13	m	17+ years	0.276	783.0025275	NA	Grand Index
13	m	17+ years	0.277	783.0027362	NA	Grand Index

Age	Gender	Education	Percentile	Raw Score	Norm Score	Assessment
13	m	17+ years	0.278	783.0188522	NA	Grand Index
13	m	17+ years	0.279	783.0906435	NA	Grand Index
13	m	17+ years	0.28	783.1624348	NA	Grand Index
13	m	17+ years	0.281	783.2342261	NA	Grand Index
13	m	17+ years	0.282	783.3060174	NA	Grand Index
13	m	17+ years	0.283	783.3778087	NA	Grand Index
13	m	17+ years	0.284	783.4496	NA	Grand Index
13	m	17+ years	0.285	783.5213913	NA	Grand Index
13	m	17+ years	0.286	783.5931826	NA	Grand Index
13	m	17+ years	0.287	783.6649739	NA	Grand Index
13	m	17+ years	0.288	783.7367652	NA	Grand Index
13	m	17+ years	0.289	783.8085565	NA	Grand Index
13	m	17+ years	0.29	783.8803478	NA	Grand Index
13	m	17+ years	0.291	783.9521391	NA	Grand Index
13	m	17+ years	0.292	784.072	NA	Grand Index
13	m	17+ years	0.293	784.288	NA	Grand Index
13	m	17+ years	0.294	784.504	NA	Grand Index
13	m	17+ years	0.295	784.72	NA	Grand Index
13	m	17+ years	0.296	784.936	NA	Grand Index
13	m	17+ years	0.297	785.152	NA	Grand Index
13	m	17+ years	0.298	785.368	NA	Grand Index
13	m	17+ years	0.299	785.584	NA	Grand Index
13	m	17+ years	0.3	785.8	NA	Grand Index
13	m	17+ years	0.301	786.016	NA	Grand Index
13	m	17+ years	0.302	786.232	NA	Grand Index
13	m	17+ years	0.303	786.448	NA	Grand Index
13	m	17+ years	0.304	786.664	NA	Grand Index
13	m	17+ years	0.305	786.88	NA	Grand Index
13	m	17+ years	0.319	787	NA	Grand Index
13	m	17+ years	0.32	787.04	NA	Grand Index
13	m	17+ years	0.321	787.112	NA	Grand Index
13	m	17+ years	0.322	787.184	NA	Grand Index
13	m	17+ years	0.323	787.256	NA	Grand Index
13	m	17+ years	0.324	787.328	NA	Grand Index
13	m	17+ years	0.325	787.4	NA	Grand Index
13	m	17+ years	0.326	787.472	NA	Grand Index
13	m	17+ years	0.327	787.544	NA	Grand Index
13	m	17+ years	0.328	787.616	NA	Grand Index
13	m	17+ years	0.329	787.688	NA	Grand Index
13	m	17+ years	0.33	787.76	NA	Grand Index
13	m	17+ years	0.331	787.832	NA	Grand Index
13	m	17+ years	0.332	787.904	NA	Grand Index
13	m	17+ years	0.333	787.976	NA	Grand Index
13	m	17+ years	0.334	788.24	NA	Grand Index
13	m	17+ years	0.335	788.6	NA	Grand Index
13	m	17+ years	0.336	788.96	NA	Grand Index
13	m	17+ years	0.337	789.32	NA	Grand Index
13	m	17+ years	0.338	789.68	NA	Grand Index
13	m	17+ years	0.339	790.04	NA	Grand Index
13	m	17+ years	0.34	790.4	NA	Grand Index
13	m	17+ years	0.341	790.76	NA	Grand Index
13	m	17+ years	0.342	791.12	NA	Grand Index
13	m	17+ years	0.343	791.48	NA	Grand Index
13	m	17+ years	0.344	791.84	NA	Grand Index
13	m	17+ years	0.345	792.2	NA	Grand Index
13	m	17+ years	0.346	792.56	NA	Grand Index

Age	Gender	Education	Percentile	Raw Score	Norm Score	Assessment
13	m	17+ years	0.347	792.92	NA	Grand Index
13	m	17+ years	0.348	793.056	NA	Grand Index
13	m	17+ years	0.349	793.128	NA	Grand Index
13	m	17+ years	0.35	793.2	NA	Grand Index
13	m	17+ years	0.351	793.272	NA	Grand Index
13	m	17+ years	0.352	793.344	NA	Grand Index
13	m	17+ years	0.353	793.416	NA	Grand Index
13	m	17+ years	0.354	793.488	NA	Grand Index
13	m	17+ years	0.355	793.56	NA	Grand Index
13	m	17+ years	0.356	793.632	NA	Grand Index
13	m	17+ years	0.357	793.704	NA	Grand Index
13	m	17+ years	0.358	793.776	NA	Grand Index
13	m	17+ years	0.359	793.848	NA	Grand Index
13	m	17+ years	0.36	793.92	NA	Grand Index
13	m	17+ years	0.361	793.992	NA	Grand Index
13	m	17+ years	0.388	794	NA	Grand Index
13	m	17+ years	0.389	794.008	NA	Grand Index
13	m	17+ years	0.39	794.08	NA	Grand Index
13	m	17+ years	0.391	794.152	NA	Grand Index
13	m	17+ years	0.392	794.224	NA	Grand Index
13	m	17+ years	0.393	794.296	NA	Grand Index
13	m	17+ years	0.394	794.368	NA	Grand Index
13	m	17+ years	0.395	794.44	NA	Grand Index
13	m	17+ years	0.396	794.512	NA	Grand Index
13	m	17+ years	0.397	794.584	NA	Grand Index
13	m	17+ years	0.398	794.656	NA	Grand Index
13	m	17+ years	0.399	794.728	NA	Grand Index
13	m	17+ years	0.4	794.8	NA	Grand Index
13	m	17+ years	0.401	794.872	NA	Grand Index
13	m	17+ years	0.402	794.944	NA	Grand Index
13	m	17+ years	0.403	795.032	NA	Grand Index
13	m	17+ years	0.404	795.176	NA	Grand Index
13	m	17+ years	0.405	795.32	NA	Grand Index
13	m	17+ years	0.406	795.464	NA	Grand Index
13	m	17+ years	0.407	795.608	NA	Grand Index
13	m	17+ years	0.408	795.752	NA	Grand Index
13	m	17+ years	0.409	795.896	NA	Grand Index
13	m	17+ years	0.41	796.04	NA	Grand Index
13	m	17+ years	0.411	796.184	NA	Grand Index
13	m	17+ years	0.412	796.328	NA	Grand Index
13	m	17+ years	0.413	796.472	NA	Grand Index
13	m	17+ years	0.414	796.616	NA	Grand Index
13	m	17+ years	0.415	796.76	NA	Grand Index
13	m	17+ years	0.416	796.904	NA	Grand Index
13	m	17+ years	0.417	797.096	NA	Grand Index
13	m	17+ years	0.418	797.384	NA	Grand Index
13	m	17+ years	0.419	797.672	NA	Grand Index
13	m	17+ years	0.42	797.96	NA	Grand Index
13	m	17+ years	0.421	798.248	NA	Grand Index
13	m	17+ years	0.422	798.536	NA	Grand Index
13	m	17+ years	0.423	798.824	NA	Grand Index
13	m	17+ years	0.424	799.112	NA	Grand Index
13	m	17+ years	0.425	799.4	NA	Grand Index
13	m	17+ years	0.426	799.688	NA	Grand Index
13	m	17+ years	0.427	799.976	NA	Grand Index
13	m	17+ years	0.428	800.264	NA	Grand Index

Age	Gender	Education	Percentile	Raw Score	Norm Score	Assessment
13	m	17+ years	0.429	800.552	NA	Grand Index
13	m	17+ years	0.43	800.84	NA	Grand Index
13	m	17+ years	0.431	801.0393846	NA	Grand Index
13	m	17+ years	0.432	801.128	NA	Grand Index
13	m	17+ years	0.433	801.2166154	NA	Grand Index
13	m	17+ years	0.434	801.3052308	NA	Grand Index
13	m	17+ years	0.435	801.3938462	NA	Grand Index
13	m	17+ years	0.436	801.4824615	NA	Grand Index
13	m	17+ years	0.437	801.5710769	NA	Grand Index
13	m	17+ years	0.438	801.6596923	NA	Grand Index
13	m	17+ years	0.439	801.7483077	NA	Grand Index
13	m	17+ years	0.44	801.8369231	NA	Grand Index
13	m	17+ years	0.441	801.9255385	NA	Grand Index
13	m	17+ years	0.442	802.0141538	NA	Grand Index
13	m	17+ years	0.443	802.1027692	NA	Grand Index
13	m	17+ years	0.444	802.1913846	NA	Grand Index
13	m	17+ years	0.445	802.3387469	NA	Grand Index
13	m	17+ years	0.446	802.5331068	NA	Grand Index
13	m	17+ years	0.447	802.7274667	NA	Grand Index
13	m	17+ years	0.448	802.9218266	NA	Grand Index
13	m	17+ years	0.449	803.1161865	NA	Grand Index
13	m	17+ years	0.45	803.3105463	NA	Grand Index
13	m	17+ years	0.451	803.5049062	NA	Grand Index
13	m	17+ years	0.452	803.6992661	NA	Grand Index
13	m	17+ years	0.453	803.893626	NA	Grand Index
13	m	17+ years	0.454	804.0879859	NA	Grand Index
13	m	17+ years	0.455	804.2823457	NA	Grand Index
13	m	17+ years	0.456	804.4767056	NA	Grand Index
13	m	17+ years	0.457	804.6710655	NA	Grand Index
13	m	17+ years	0.458	804.8654254	NA	Grand Index
13	m	17+ years	0.459	804.9815618	NA	Grand Index
13	m	17+ years	0.46	805.0585866	NA	Grand Index
13	m	17+ years	0.461	805.1356113	NA	Grand Index
13	m	17+ years	0.462	805.212636	NA	Grand Index
13	m	17+ years	0.463	805.2896608	NA	Grand Index
13	m	17+ years	0.464	805.3666855	NA	Grand Index
13	m	17+ years	0.465	805.4437102	NA	Grand Index
13	m	17+ years	0.466	805.520735	NA	Grand Index
13	m	17+ years	0.467	805.5977597	NA	Grand Index
13	m	17+ years	0.468	805.6747845	NA	Grand Index
13	m	17+ years	0.469	805.7518092	NA	Grand Index
13	m	17+ years	0.47	805.8288339	NA	Grand Index
13	m	17+ years	0.471	805.9058587	NA	Grand Index
13	m	17+ years	0.472	805.9828834	NA	Grand Index
13	m	17+ years	0.473	806.112	NA	Grand Index
13	m	17+ years	0.474	806.256	NA	Grand Index
13	m	17+ years	0.475	806.4	NA	Grand Index
13	m	17+ years	0.476	806.544	NA	Grand Index
13	m	17+ years	0.477	806.688	NA	Grand Index
13	m	17+ years	0.478	806.832	NA	Grand Index
13	m	17+ years	0.479	806.976	NA	Grand Index
13	m	17+ years	0.48	807.12	NA	Grand Index
13	m	17+ years	0.481	807.264	NA	Grand Index
13	m	17+ years	0.482	807.408	NA	Grand Index
13	m	17+ years	0.483	807.552	NA	Grand Index
13	m	17+ years	0.484	807.696	NA	Grand Index

Age	Gender	Education	Percentile	Raw Score	Norm Score	Assessment
13	m	17+ years	0.485	807.84	NA	Grand Index
13	m	17+ years	0.486	807.984	NA	Grand Index
13	m	17+ years	0.487	808.064	NA	Grand Index
13	m	17+ years	0.488	808.136	NA	Grand Index
13	m	17+ years	0.489	808.208	NA	Grand Index
13	m	17+ years	0.49	808.28	NA	Grand Index
13	m	17+ years	0.491	808.352	NA	Grand Index
13	m	17+ years	0.492	808.424	NA	Grand Index
13	m	17+ years	0.493	808.496	NA	Grand Index
13	m	17+ years	0.494	808.568	NA	Grand Index
13	m	17+ years	0.495	808.64	NA	Grand Index
13	m	17+ years	0.496	808.712	NA	Grand Index
13	m	17+ years	0.497	808.784	NA	Grand Index
13	m	17+ years	0.498	808.856	NA	Grand Index
13	m	17+ years	0.499	808.928	NA	Grand Index
13	m	17+ years	0.5	809	NA	Grand Index
13	m	17+ years	0.501	809.216	NA	Grand Index
13	m	17+ years	0.502	809.432	NA	Grand Index
13	m	17+ years	0.503	809.648	NA	Grand Index
13	m	17+ years	0.504	809.864	NA	Grand Index
13	m	17+ years	0.505	810.08	NA	Grand Index
13	m	17+ years	0.506	810.296	NA	Grand Index
13	m	17+ years	0.507	810.512	NA	Grand Index
13	m	17+ years	0.508	810.728	NA	Grand Index
13	m	17+ years	0.509	810.944	NA	Grand Index
13	m	17+ years	0.51	811.16	NA	Grand Index
13	m	17+ years	0.511	811.376	NA	Grand Index
13	m	17+ years	0.512	811.592	NA	Grand Index
13	m	17+ years	0.513	811.808	NA	Grand Index
13	m	17+ years	0.514	812.048	NA	Grand Index
13	m	17+ years	0.515	812.48	NA	Grand Index
13	m	17+ years	0.516	812.912	NA	Grand Index
13	m	17+ years	0.517	813.344	NA	Grand Index
13	m	17+ years	0.518	813.776	NA	Grand Index
13	m	17+ years	0.519	814.208	NA	Grand Index
13	m	17+ years	0.52	814.64	NA	Grand Index
13	m	17+ years	0.521	815.072	NA	Grand Index
13	m	17+ years	0.522	815.504	NA	Grand Index
13	m	17+ years	0.523	815.936	NA	Grand Index
13	m	17+ years	0.524	816.368	NA	Grand Index
13	m	17+ years	0.525	816.8	NA	Grand Index
13	m	17+ years	0.526	817.232	NA	Grand Index
13	m	17+ years	0.527	817.664	NA	Grand Index
13	m	17+ years	0.528	818.096	NA	Grand Index
13	m	17+ years	0.529	818.528	NA	Grand Index
13	m	17+ years	0.53	818.96	NA	Grand Index
13	m	17+ years	0.531	819.392	NA	Grand Index
13	m	17+ years	0.532	819.824	NA	Grand Index
13	m	17+ years	0.533	820.256	NA	Grand Index
13	m	17+ years	0.534	820.688	NA	Grand Index
13	m	17+ years	0.535	821.12	NA	Grand Index
13	m	17+ years	0.536	821.552	NA	Grand Index
13	m	17+ years	0.537	821.984	NA	Grand Index
13	m	17+ years	0.538	822.416	NA	Grand Index
13	m	17+ years	0.539	822.848	NA	Grand Index
13	m	17+ years	0.54	823.28	NA	Grand Index

Age	Gender	Education	Percentile	Raw Score	Norm Score	Assessment
13	m	17+ years	0.541	823.712	NA	Grand Index
13	m	17+ years	0.542	824.072	NA	Grand Index
13	m	17+ years	0.543	824.288	NA	Grand Index
13	m	17+ years	0.544	824.504	NA	Grand Index
13	m	17+ years	0.545	824.72	NA	Grand Index
13	m	17+ years	0.546	824.936	NA	Grand Index
13	m	17+ years	0.547	825.152	NA	Grand Index
13	m	17+ years	0.548	825.368	NA	Grand Index
13	m	17+ years	0.549	825.584	NA	Grand Index
13	m	17+ years	0.55	825.8	NA	Grand Index
13	m	17+ years	0.551	826.016	NA	Grand Index
13	m	17+ years	0.552	826.232	NA	Grand Index
13	m	17+ years	0.553	826.448	NA	Grand Index
13	m	17+ years	0.554	826.664	NA	Grand Index
13	m	17+ years	0.555	826.88	NA	Grand Index
13	m	17+ years	0.556	827.0955404	NA	Grand Index
13	m	17+ years	0.557	827.3105064	NA	Grand Index
13	m	17+ years	0.558	827.5254724	NA	Grand Index
13	m	17+ years	0.559	827.7404384	NA	Grand Index
13	m	17+ years	0.56	827.9554044	NA	Grand Index
13	m	17+ years	0.561	828.1703704	NA	Grand Index
13	m	17+ years	0.562	828.3853364	NA	Grand Index
13	m	17+ years	0.563	828.6003023	NA	Grand Index
13	m	17+ years	0.564	828.8152683	NA	Grand Index
13	m	17+ years	0.565	829.0302343	NA	Grand Index
13	m	17+ years	0.566	829.2452003	NA	Grand Index
13	m	17+ years	0.567	829.4601663	NA	Grand Index
13	m	17+ years	0.568	829.6751323	NA	Grand Index
13	m	17+ years	0.569	829.8900983	NA	Grand Index
13	m	17+ years	0.57	829.9862132	NA	Grand Index
13	m	17+ years	0.571	829.9872472	NA	Grand Index
13	m	17+ years	0.572	829.9882812	NA	Grand Index
13	m	17+ years	0.573	829.9893152	NA	Grand Index
13	m	17+ years	0.574	829.9903492	NA	Grand Index
13	m	17+ years	0.575	829.9913832	NA	Grand Index
13	m	17+ years	0.576	829.9924172	NA	Grand Index
13	m	17+ years	0.577	829.9934512	NA	Grand Index
13	m	17+ years	0.578	829.9944853	NA	Grand Index
13	m	17+ years	0.579	829.9955193	NA	Grand Index
13	m	17+ years	0.58	829.9965533	NA	Grand Index
13	m	17+ years	0.581	829.9975873	NA	Grand Index
13	m	17+ years	0.582	829.9986213	NA	Grand Index
13	m	17+ years	0.583	829.9996553	NA	Grand Index
13	m	17+ years	0.584	830.048	NA	Grand Index
13	m	17+ years	0.585	830.12	NA	Grand Index
13	m	17+ years	0.586	830.192	NA	Grand Index
13	m	17+ years	0.587	830.264	NA	Grand Index
13	m	17+ years	0.588	830.336	NA	Grand Index
13	m	17+ years	0.589	830.408	NA	Grand Index
13	m	17+ years	0.59	830.48	NA	Grand Index
13	m	17+ years	0.591	830.552	NA	Grand Index
13	m	17+ years	0.592	830.624	NA	Grand Index
13	m	17+ years	0.593	830.696	NA	Grand Index
13	m	17+ years	0.594	830.768	NA	Grand Index
13	m	17+ years	0.595	830.84	NA	Grand Index
13	m	17+ years	0.596	830.912	NA	Grand Index

Age	Gender	Education	Percentile	Raw Score	Norm Score	Assessment
13	m	17+ years	0.597	830.984	NA	Grand Index
13	m	17+ years	0.611	831	NA	Grand Index
13	m	17+ years	0.612	831.064	NA	Grand Index
13	m	17+ years	0.613	831.136	NA	Grand Index
13	m	17+ years	0.614	831.208	NA	Grand Index
13	m	17+ years	0.615	831.28	NA	Grand Index
13	m	17+ years	0.616	831.352	NA	Grand Index
13	m	17+ years	0.617	831.424	NA	Grand Index
13	m	17+ years	0.618	831.496	NA	Grand Index
13	m	17+ years	0.619	831.568	NA	Grand Index
13	m	17+ years	0.62	831.64	NA	Grand Index
13	m	17+ years	0.621	831.712	NA	Grand Index
13	m	17+ years	0.622	831.784	NA	Grand Index
13	m	17+ years	0.623	831.856	NA	Grand Index
13	m	17+ years	0.624	831.928	NA	Grand Index
13	m	17+ years	0.625	832	NA	Grand Index
13	m	17+ years	0.626	832.072	NA	Grand Index
13	m	17+ years	0.627	832.144	NA	Grand Index
13	m	17+ years	0.628	832.216	NA	Grand Index
13	m	17+ years	0.629	832.288	NA	Grand Index
13	m	17+ years	0.63	832.36	NA	Grand Index
13	m	17+ years	0.631	832.432	NA	Grand Index
13	m	17+ years	0.632	832.504	NA	Grand Index
13	m	17+ years	0.633	832.576	NA	Grand Index
13	m	17+ years	0.634	832.648	NA	Grand Index
13	m	17+ years	0.635	832.72	NA	Grand Index
13	m	17+ years	0.636	832.792	NA	Grand Index
13	m	17+ years	0.637	832.864	NA	Grand Index
13	m	17+ years	0.638	832.936	NA	Grand Index
13	m	17+ years	0.639	833.0056604	NA	Grand Index
13	m	17+ years	0.64	833.0566044	NA	Grand Index
13	m	17+ years	0.641	833.1075484	NA	Grand Index
13	m	17+ years	0.642	833.1584924	NA	Grand Index
13	m	17+ years	0.643	833.2094364	NA	Grand Index
13	m	17+ years	0.644	833.2603804	NA	Grand Index
13	m	17+ years	0.645	833.3113244	NA	Grand Index
13	m	17+ years	0.646	833.3622684	NA	Grand Index
13	m	17+ years	0.647	833.4132124	NA	Grand Index
13	m	17+ years	0.648	833.4641564	NA	Grand Index
13	m	17+ years	0.649	833.5151004	NA	Grand Index
13	m	17+ years	0.65	833.5660444	NA	Grand Index
13	m	17+ years	0.651	833.6169884	NA	Grand Index
13	m	17+ years	0.652	833.6679324	NA	Grand Index
13	m	17+ years	0.653	833.7282347	NA	Grand Index
13	m	17+ years	0.654	833.8212907	NA	Grand Index
13	m	17+ years	0.655	833.9143467	NA	Grand Index
13	m	17+ years	0.656	834.0074027	NA	Grand Index
13	m	17+ years	0.657	834.1004587	NA	Grand Index
13	m	17+ years	0.658	834.1935147	NA	Grand Index
13	m	17+ years	0.659	834.2865707	NA	Grand Index
13	m	17+ years	0.66	834.3796267	NA	Grand Index
13	m	17+ years	0.661	834.4726827	NA	Grand Index
13	m	17+ years	0.662	834.5657387	NA	Grand Index
13	m	17+ years	0.663	834.6587947	NA	Grand Index
13	m	17+ years	0.664	834.7518507	NA	Grand Index
13	m	17+ years	0.665	834.8449067	NA	Grand Index

Age	Gender	Education	Percentile	Raw Score	Norm Score	Assessment
13	m	17+ years	0.666	834.9379627	NA	Grand Index
13	m	17+ years	0.667	835.192	NA	Grand Index
13	m	17+ years	0.668	835.768	NA	Grand Index
13	m	17+ years	0.669	836.344	NA	Grand Index
13	m	17+ years	0.67	836.92	NA	Grand Index
13	m	17+ years	0.671	837.496	NA	Grand Index
13	m	17+ years	0.672	838.072	NA	Grand Index
13	m	17+ years	0.673	838.648	NA	Grand Index
13	m	17+ years	0.674	839.224	NA	Grand Index
13	m	17+ years	0.675	839.8	NA	Grand Index
13	m	17+ years	0.676	840.376	NA	Grand Index
13	m	17+ years	0.677	840.952	NA	Grand Index
13	m	17+ years	0.678	841.528	NA	Grand Index
13	m	17+ years	0.679	842.104	NA	Grand Index
13	m	17+ years	0.68	842.68	NA	Grand Index
13	m	17+ years	0.681	843.256	NA	Grand Index
13	m	17+ years	0.682	843.832	NA	Grand Index
13	m	17+ years	0.683	844.408	NA	Grand Index
13	m	17+ years	0.684	844.984	NA	Grand Index
13	m	17+ years	0.685	845.56	NA	Grand Index
13	m	17+ years	0.686	846.136	NA	Grand Index
13	m	17+ years	0.687	846.712	NA	Grand Index
13	m	17+ years	0.688	847.288	NA	Grand Index
13	m	17+ years	0.689	847.864	NA	Grand Index
13	m	17+ years	0.69	848.44	NA	Grand Index
13	m	17+ years	0.691	849.016	NA	Grand Index
13	m	17+ years	0.692	849.592	NA	Grand Index
13	m	17+ years	0.693	850.168	NA	Grand Index
13	m	17+ years	0.694	850.744	NA	Grand Index
13	m	17+ years	0.695	851.04	NA	Grand Index
13	m	17+ years	0.696	851.112	NA	Grand Index
13	m	17+ years	0.697	851.184	NA	Grand Index
13	m	17+ years	0.698	851.256	NA	Grand Index
13	m	17+ years	0.699	851.328	NA	Grand Index
13	m	17+ years	0.7	851.4	NA	Grand Index
13	m	17+ years	0.701	851.472	NA	Grand Index
13	m	17+ years	0.702	851.544	NA	Grand Index
13	m	17+ years	0.703	851.616	NA	Grand Index
13	m	17+ years	0.704	851.688	NA	Grand Index
13	m	17+ years	0.705	851.76	NA	Grand Index
13	m	17+ years	0.706	851.832	NA	Grand Index
13	m	17+ years	0.707	851.904	NA	Grand Index
13	m	17+ years	0.708	851.976	NA	Grand Index
13	m	17+ years	0.709	852.048	NA	Grand Index
13	m	17+ years	0.71	852.12	NA	Grand Index
13	m	17+ years	0.711	852.192	NA	Grand Index
13	m	17+ years	0.712	852.264	NA	Grand Index
13	m	17+ years	0.713	852.336	NA	Grand Index
13	m	17+ years	0.714	852.408	NA	Grand Index
13	m	17+ years	0.715	852.48	NA	Grand Index
13	m	17+ years	0.716	852.552	NA	Grand Index
13	m	17+ years	0.717	852.624	NA	Grand Index
13	m	17+ years	0.718	852.696	NA	Grand Index
13	m	17+ years	0.719	852.768	NA	Grand Index
13	m	17+ years	0.72	852.84	NA	Grand Index
13	m	17+ years	0.721	852.912	NA	Grand Index

Age	Gender	Education	Percentile	Raw Score	Norm Score	Assessment
13	m	17+ years	0.722	852.984	NA	Grand Index
13	m	17+ years	0.723	853.056	NA	Grand Index
13	m	17+ years	0.724	853.128	NA	Grand Index
13	m	17+ years	0.725	853.2	NA	Grand Index
13	m	17+ years	0.726	853.272	NA	Grand Index
13	m	17+ years	0.727	853.344	NA	Grand Index
13	m	17+ years	0.728	853.416	NA	Grand Index
13	m	17+ years	0.729	853.488	NA	Grand Index
13	m	17+ years	0.73	853.56	NA	Grand Index
13	m	17+ years	0.731	853.632	NA	Grand Index
13	m	17+ years	0.732	853.704	NA	Grand Index
13	m	17+ years	0.733	853.776	NA	Grand Index
13	m	17+ years	0.734	853.848	NA	Grand Index
13	m	17+ years	0.735	853.92	NA	Grand Index
13	m	17+ years	0.736	853.992	NA	Grand Index
13	m	17+ years	0.737	854.576	NA	Grand Index
13	m	17+ years	0.738	855.224	NA	Grand Index
13	m	17+ years	0.739	855.872	NA	Grand Index
13	m	17+ years	0.74	856.52	NA	Grand Index
13	m	17+ years	0.741	857.168	NA	Grand Index
13	m	17+ years	0.742	857.816	NA	Grand Index
13	m	17+ years	0.743	858.464	NA	Grand Index
13	m	17+ years	0.744	859.112	NA	Grand Index
13	m	17+ years	0.745	859.76	NA	Grand Index
13	m	17+ years	0.746	860.408	NA	Grand Index
13	m	17+ years	0.747	861.056	NA	Grand Index
13	m	17+ years	0.748	861.704	NA	Grand Index
13	m	17+ years	0.749	862.352	NA	Grand Index
13	m	17+ years	0.75	863	NA	Grand Index
13	m	17+ years	0.751	863.1455223	NA	Grand Index
13	m	17+ years	0.752	863.2910447	NA	Grand Index
13	m	17+ years	0.753	863.436567	NA	Grand Index
13	m	17+ years	0.754	863.5820893	NA	Grand Index
13	m	17+ years	0.755	863.7276117	NA	Grand Index
13	m	17+ years	0.756	863.873134	NA	Grand Index
13	m	17+ years	0.757	864.0186564	NA	Grand Index
13	m	17+ years	0.758	864.1641787	NA	Grand Index
13	m	17+ years	0.759	864.309701	NA	Grand Index
13	m	17+ years	0.76	864.4552234	NA	Grand Index
13	m	17+ years	0.761	864.6007457	NA	Grand Index
13	m	17+ years	0.762	864.746268	NA	Grand Index
13	m	17+ years	0.763	864.8917904	NA	Grand Index
13	m	17+ years	0.764	865.0369744	NA	Grand Index
13	m	17+ years	0.765	865.1794521	NA	Grand Index
13	m	17+ years	0.766	865.3219297	NA	Grand Index
13	m	17+ years	0.767	865.4644074	NA	Grand Index
13	m	17+ years	0.768	865.6068851	NA	Grand Index
13	m	17+ years	0.769	865.7493627	NA	Grand Index
13	m	17+ years	0.77	865.8918404	NA	Grand Index
13	m	17+ years	0.771	866.0343181	NA	Grand Index
13	m	17+ years	0.772	866.1767957	NA	Grand Index
13	m	17+ years	0.773	866.3192734	NA	Grand Index
13	m	17+ years	0.774	866.461751	NA	Grand Index
13	m	17+ years	0.775	866.6042287	NA	Grand Index
13	m	17+ years	0.776	866.7467064	NA	Grand Index
13	m	17+ years	0.777	866.889184	NA	Grand Index

Age	Gender	Education	Percentile	Raw Score	Norm Score	Assessment
13	m	17+ years	0.778	867.048	NA	Grand Index
13	m	17+ years	0.779	867.264	NA	Grand Index
13	m	17+ years	0.78	867.48	NA	Grand Index
13	m	17+ years	0.781	867.696	NA	Grand Index
13	m	17+ years	0.782	867.912	NA	Grand Index
13	m	17+ years	0.783	868.128	NA	Grand Index
13	m	17+ years	0.784	868.344	NA	Grand Index
13	m	17+ years	0.785	868.56	NA	Grand Index
13	m	17+ years	0.786	868.776	NA	Grand Index
13	m	17+ years	0.787	868.992	NA	Grand Index
13	m	17+ years	0.788	869.208	NA	Grand Index
13	m	17+ years	0.789	869.424	NA	Grand Index
13	m	17+ years	0.79	869.64	NA	Grand Index
13	m	17+ years	0.791	869.856	NA	Grand Index
13	m	17+ years	0.792	870.3276686	NA	Grand Index
13	m	17+ years	0.793	871.3106746	NA	Grand Index
13	m	17+ years	0.794	872.2936805	NA	Grand Index
13	m	17+ years	0.795	873.2766864	NA	Grand Index
13	m	17+ years	0.796	874.2596923	NA	Grand Index
13	m	17+ years	0.797	875.2426982	NA	Grand Index
13	m	17+ years	0.798	876.2257041	NA	Grand Index
13	m	17+ years	0.799	877.2087101	NA	Grand Index
13	m	17+ years	0.8	878.191716	NA	Grand Index
13	m	17+ years	0.801	879.1747219	NA	Grand Index
13	m	17+ years	0.802	880.1577278	NA	Grand Index
13	m	17+ years	0.803	881.1407337	NA	Grand Index
13	m	17+ years	0.804	882.1237396	NA	Grand Index
13	m	17+ years	0.805	883.1067456	NA	Grand Index
13	m	17+ years	0.806	883.6959684	NA	Grand Index
13	m	17+ years	0.807	883.7929625	NA	Grand Index
13	m	17+ years	0.808	883.8899566	NA	Grand Index
13	m	17+ years	0.809	883.9869507	NA	Grand Index
13	m	17+ years	0.81	884.0839448	NA	Grand Index
13	m	17+ years	0.811	884.1809389	NA	Grand Index
13	m	17+ years	0.812	884.2779329	NA	Grand Index
13	m	17+ years	0.813	884.374927	NA	Grand Index
13	m	17+ years	0.814	884.4719211	NA	Grand Index
13	m	17+ years	0.815	884.5689152	NA	Grand Index
13	m	17+ years	0.816	884.6659093	NA	Grand Index
13	m	17+ years	0.817	884.7629034	NA	Grand Index
13	m	17+ years	0.818	884.8598974	NA	Grand Index
13	m	17+ years	0.819	884.9568915	NA	Grand Index
13	m	17+ years	0.82	885.2573633	NA	Grand Index
13	m	17+ years	0.821	885.7206174	NA	Grand Index
13	m	17+ years	0.822	886.1838714	NA	Grand Index
13	m	17+ years	0.823	886.6471254	NA	Grand Index
13	m	17+ years	0.824	887.1103794	NA	Grand Index
13	m	17+ years	0.825	887.5736334	NA	Grand Index
13	m	17+ years	0.826	888.0368875	NA	Grand Index
13	m	17+ years	0.827	888.5001415	NA	Grand Index
13	m	17+ years	0.828	888.9633955	NA	Grand Index
13	m	17+ years	0.829	889.4266495	NA	Grand Index
13	m	17+ years	0.83	889.8899035	NA	Grand Index
13	m	17+ years	0.831	890.3531576	NA	Grand Index
13	m	17+ years	0.832	890.8164116	NA	Grand Index
13	m	17+ years	0.833	891.2796656	NA	Grand Index

Age	Gender	Education	Percentile	Raw Score	Norm Score	Assessment
13	m	17+ years	0.834	891.5999143	NA	Grand Index
13	m	17+ years	0.835	891.8486602	NA	Grand Index
13	m	17+ years	0.836	892.0974062	NA	Grand Index
13	m	17+ years	0.837	892.3461522	NA	Grand Index
13	m	17+ years	0.838	892.5948982	NA	Grand Index
13	m	17+ years	0.839	892.8436442	NA	Grand Index
13	m	17+ years	0.84	893.0923901	NA	Grand Index
13	m	17+ years	0.841	893.3411361	NA	Grand Index
13	m	17+ years	0.842	893.5898821	NA	Grand Index
13	m	17+ years	0.843	893.8386281	NA	Grand Index
13	m	17+ years	0.844	894.0873741	NA	Grand Index
13	m	17+ years	0.845	894.33612	NA	Grand Index
13	m	17+ years	0.846	894.584866	NA	Grand Index
13	m	17+ years	0.847	894.833612	NA	Grand Index
13	m	17+ years	0.848	895.2311111	NA	Grand Index
13	m	17+ years	0.849	895.6711111	NA	Grand Index
13	m	17+ years	0.85	896.1111111	NA	Grand Index
13	m	17+ years	0.851	896.5511111	NA	Grand Index
13	m	17+ years	0.852	896.9911111	NA	Grand Index
13	m	17+ years	0.853	897.4311111	NA	Grand Index
13	m	17+ years	0.854	897.8711111	NA	Grand Index
13	m	17+ years	0.855	898.3111111	NA	Grand Index
13	m	17+ years	0.856	898.7511111	NA	Grand Index
13	m	17+ years	0.857	899.1911111	NA	Grand Index
13	m	17+ years	0.858	899.6311111	NA	Grand Index
13	m	17+ years	0.859	900.0711111	NA	Grand Index
13	m	17+ years	0.86	900.5111111	NA	Grand Index
13	m	17+ years	0.861	900.9511111	NA	Grand Index
13	m	17+ years	0.862	901.128	NA	Grand Index
13	m	17+ years	0.863	901.272	NA	Grand Index
13	m	17+ years	0.864	901.416	NA	Grand Index
13	m	17+ years	0.865	901.56	NA	Grand Index
13	m	17+ years	0.866	901.704	NA	Grand Index
13	m	17+ years	0.867	901.848	NA	Grand Index
13	m	17+ years	0.868	901.992	NA	Grand Index
13	m	17+ years	0.869	902.136	NA	Grand Index
13	m	17+ years	0.87	902.28	NA	Grand Index
13	m	17+ years	0.871	902.424	NA	Grand Index
13	m	17+ years	0.872	902.568	NA	Grand Index
13	m	17+ years	0.873	902.712	NA	Grand Index
13	m	17+ years	0.874	902.856	NA	Grand Index
13	m	17+ years	0.875	903	NA	Grand Index
13	m	17+ years	0.876	903.216	NA	Grand Index
13	m	17+ years	0.877	903.432	NA	Grand Index
13	m	17+ years	0.878	903.648	NA	Grand Index
13	m	17+ years	0.879	903.864	NA	Grand Index
13	m	17+ years	0.88	904.08	NA	Grand Index
13	m	17+ years	0.881	904.296	NA	Grand Index
13	m	17+ years	0.882	904.512	NA	Grand Index
13	m	17+ years	0.883	904.728	NA	Grand Index
13	m	17+ years	0.884	904.944	NA	Grand Index
13	m	17+ years	0.885	905.16	NA	Grand Index
13	m	17+ years	0.886	905.376	NA	Grand Index
13	m	17+ years	0.887	905.592	NA	Grand Index
13	m	17+ years	0.888	905.808	NA	Grand Index
13	m	17+ years	0.889	906.12	NA	Grand Index

Age	Gender	Education	Percentile	Raw Score	Norm Score	Assessment
13	m	17+ years	0.89	907.2	NA	Grand Index
13	m	17+ years	0.891	908.28	NA	Grand Index
13	m	17+ years	0.892	909.36	NA	Grand Index
13	m	17+ years	0.893	910.44	NA	Grand Index
13	m	17+ years	0.894	911.52	NA	Grand Index
13	m	17+ years	0.895	912.6	NA	Grand Index
13	m	17+ years	0.896	913.68	NA	Grand Index
13	m	17+ years	0.897	914.76	NA	Grand Index
13	m	17+ years	0.898	915.84	NA	Grand Index
13	m	17+ years	0.899	916.92	NA	Grand Index
13	m	17+ years	0.9	918	NA	Grand Index
13	m	17+ years	0.901	919.08	NA	Grand Index
13	m	17+ years	0.902	920.16	NA	Grand Index
13	m	17+ years	0.903	921.112	NA	Grand Index
13	m	17+ years	0.904	921.616	NA	Grand Index
13	m	17+ years	0.905	922.12	NA	Grand Index
13	m	17+ years	0.906	922.624	NA	Grand Index
13	m	17+ years	0.907	923.128	NA	Grand Index
13	m	17+ years	0.908	923.632	NA	Grand Index
13	m	17+ years	0.909	924.136	NA	Grand Index
13	m	17+ years	0.91	924.64	NA	Grand Index
13	m	17+ years	0.911	925.144	NA	Grand Index
13	m	17+ years	0.912	925.648	NA	Grand Index
13	m	17+ years	0.913	926.152	NA	Grand Index
13	m	17+ years	0.914	926.656	NA	Grand Index
13	m	17+ years	0.915	927.16	NA	Grand Index
13	m	17+ years	0.916	927.664	NA	Grand Index
13	m	17+ years	0.93	928	NA	Grand Index
13	m	17+ years	0.931	928.096	NA	Grand Index
13	m	17+ years	0.932	928.312	NA	Grand Index
13	m	17+ years	0.933	928.528	NA	Grand Index
13	m	17+ years	0.934	928.744	NA	Grand Index
13	m	17+ years	0.935	928.96	NA	Grand Index
13	m	17+ years	0.936	929.176	NA	Grand Index
13	m	17+ years	0.937	929.392	NA	Grand Index
13	m	17+ years	0.938	929.608	NA	Grand Index
13	m	17+ years	0.939	929.824	NA	Grand Index
13	m	17+ years	0.94	930.04	NA	Grand Index
13	m	17+ years	0.941	930.256	NA	Grand Index
13	m	17+ years	0.942	930.472	NA	Grand Index
13	m	17+ years	0.943	930.688	NA	Grand Index
13	m	17+ years	0.944	930.904	NA	Grand Index
13	m	17+ years	0.945	931.68	NA	Grand Index
13	m	17+ years	0.946	932.904	NA	Grand Index
13	m	17+ years	0.947	934.128	NA	Grand Index
13	m	17+ years	0.948	935.352	NA	Grand Index
13	m	17+ years	0.949	936.576	NA	Grand Index
13	m	17+ years	0.95	937.8	NA	Grand Index
13	m	17+ years	0.951	939.024	NA	Grand Index
13	m	17+ years	0.952	940.248	NA	Grand Index
13	m	17+ years	0.953	941.472	NA	Grand Index
13	m	17+ years	0.954	942.696	NA	Grand Index
13	m	17+ years	0.955	943.92	NA	Grand Index
13	m	17+ years	0.956	945.144	NA	Grand Index
13	m	17+ years	0.957	946.368	NA	Grand Index
13	m	17+ years	0.958	947.592	NA	Grand Index

Age	Gender	Education	Percentile	Raw Score	Norm Score	Assessment
13	m	17+ years	0.959	949.44	NA	Grand Index
13	m	17+ years	0.96	951.6	NA	Grand Index
13	m	17+ years	0.961	953.76	NA	Grand Index
13	m	17+ years	0.962	955.92	NA	Grand Index
13	m	17+ years	0.963	958.08	NA	Grand Index
13	m	17+ years	0.964	960.24	NA	Grand Index
13	m	17+ years	0.965	962.4	NA	Grand Index
13	m	17+ years	0.966	964.56	NA	Grand Index
13	m	17+ years	0.967	966.72	NA	Grand Index
13	m	17+ years	0.968	968.88	NA	Grand Index
13	m	17+ years	0.969	971.04	NA	Grand Index
13	m	17+ years	0.97	973.2	NA	Grand Index
13	m	17+ years	0.971	975.36	NA	Grand Index
13	m	17+ years	0.972	977.52	NA	Grand Index
13	m	17+ years	0.973	979.064	NA	Grand Index
13	m	17+ years	0.974	980.432	NA	Grand Index
13	m	17+ years	0.975	981.8	NA	Grand Index
13	m	17+ years	0.976	983.168	NA	Grand Index
13	m	17+ years	0.977	984.536	NA	Grand Index
13	m	17+ years	0.978	985.904	NA	Grand Index
13	m	17+ years	0.979	987.272	NA	Grand Index
13	m	17+ years	0.98	988.64	NA	Grand Index
13	m	17+ years	0.981	990.008	NA	Grand Index
13	m	17+ years	0.982	991.376	NA	Grand Index
13	m	17+ years	0.983	992.744	NA	Grand Index
13	m	17+ years	0.984	994.112	NA	Grand Index
13	m	17+ years	0.985	995.48	NA	Grand Index
13	m	17+ years	0.986	996.848	NA	Grand Index
13	m	17+ years	0.987	999.112	NA	Grand Index
13	m	17+ years	0.988	1001.488	NA	Grand Index
13	m	17+ years	0.989	1003.864	NA	Grand Index
13	m	17+ years	0.99	1006.24	NA	Grand Index
13	m	17+ years	0.991	1008.616	NA	Grand Index
13	m	17+ years	0.992	1010.992	NA	Grand Index
13	m	17+ years	0.993	1013.368	NA	Grand Index
13	m	17+ years	0.994	1015.744	NA	Grand Index
13	m	17+ years	0.995	1018.12	NA	Grand Index
13	m	17+ years	0.996	1020.496	NA	Grand Index
13	m	17+ years	0.997	1022.872	NA	Grand Index
13	m	17+ years	0.998	1025.248	NA	Grand Index
13	m	17+ years	0.999	1027.624	NA	Grand Index
20	f	0-12 years	0.001	537.7504898	54	Grand Index
20	f	0-12 years	0.002	540.3639011	57	Grand Index
20	f	0-12 years	0.003	545.4915409	59	Grand Index
20	f	0-12 years	0.004	551.5138142	60	Grand Index
20	f	0-12 years	0.005	556.6769013	61	Grand Index
20	f	0-12 years	0.006	560.8907921	62	Grand Index
20	f	0-12 years	0.007	567.3236337	63	Grand Index
20	f	0-12 years	0.008	581.9713143	64	Grand Index
20	f	0-12 years	0.009	596.618995	65	Grand Index
20	f	0-12 years	0.01	600.5520308	65	Grand Index
20	f	0-12 years	0.011	603.9238232	66	Grand Index
20	f	0-12 years	0.012	612.5836822	66	Grand Index
20	f	0-12 years	0.013	623.6440449	67	Grand Index
20	f	0-12 years	0.014	630.376	67	Grand Index
20	f	0-12 years	0.015	631.26	67	Grand Index

Age	Gender	Education	Percentile	Raw Score	Norm Score	Assessment
20	f	0-12 years	0.016	632.6871714	68	Grand Index
20	f	0-12 years	0.017	636.9056401	68	Grand Index
20	f	0-12 years	0.018	641.1241088	69	Grand Index
20	f	0-12 years	0.019	645.3016762	69	Grand Index
20	f	0-12 years	0.02	649.4747219	69	Grand Index
20	f	0-12 years	0.021	652.1174646	69	Grand Index
20	f	0-12 years	0.022	653.8919502	70	Grand Index
20	f	0-12 years	0.023	655.664	70	Grand Index
20	f	0-12 years	0.024	657.432	70	Grand Index
20	f	0-12 years	0.025	659.05	71	Grand Index
20	f	0-12 years	0.026	659.492	71	Grand Index
20	f	0-12 years	0.027	659.934	71	Grand Index
20	f	0-12 years	0.028	663.384	71	Grand Index
20	f	0-12 years	0.029	667.362	72	Grand Index
20	f	0-12 years	0.03	670.82	72	Grand Index
20	f	0-12 years	0.031	673.914	72	Grand Index
20	f	0-12 years	0.032	676.288	72	Grand Index
20	f	0-12 years	0.033	677.172	72	Grand Index
20	f	0-12 years	0.034	678.084	73	Grand Index
20	f	0-12 years	0.035	679.41	73	Grand Index
20	f	0-12 years	0.036	680.736	73	Grand Index
20	f	0-12 years	0.037	681.6999071	73	Grand Index
20	f	0-12 years	0.038	682.5738024	73	Grand Index
20	f	0-12 years	0.039	683.6965796	74	Grand Index
20	f	0-12 years	0.04	685.0326844	74	Grand Index
20	f	0-12 years	0.041	686.1211663	74	Grand Index
20	f	0-12 years	0.042	686.5601458	74	Grand Index
20	f	0-12 years	0.043	687.0059287	74	Grand Index
20	f	0-12 years	0.044	687.9460959	74	Grand Index
20	f	0-12 years	0.045	688.8862631	75	Grand Index
20	f	0-12 years	0.046	690.4083212	75	Grand Index
20	f	0-12 years	0.047	692.1231745	75	Grand Index
20	f	0-12 years	0.048	693.703305	75	Grand Index
20	f	0-12 years	0.049	695.1424754	75	Grand Index
20	f	0-12 years	0.05	696.3304374	75	Grand Index
20	f	0-12 years	0.051	696.6592671	75	Grand Index
20	f	0-12 years	0.052	696.9880967	76	Grand Index
20	f	0-12 years	0.053	697.852	76	Grand Index
20	f	0-12 years	0.054	698.736	76	Grand Index
20	f	0-12 years	0.055	699.31	76	Grand Index
20	f	0-12 years	0.056	699.752	76	Grand Index
20	f	0-12 years	0.057	700.388	76	Grand Index
20	f	0-12 years	0.058	701.272	76	Grand Index
20	f	0-12 years	0.059	702.624	77	Grand Index
20	f	0-12 years	0.06	706.16	77	Grand Index
20	f	0-12 years	0.061	709.696	77	Grand Index
20	f	0-12 years	0.063	710	77	Grand Index
20	f	0-12 years	0.064	710.576	77	Grand Index
20	f	0-12 years	0.065	711.46	77	Grand Index
20	f	0-12 years	0.067	712	78	Grand Index
20	f	0-12 years	0.068	712.0100637	78	Grand Index
20	f	0-12 years	0.069	712.0894955	78	Grand Index
20	f	0-12 years	0.07	712.1689272	78	Grand Index
20	f	0-12 years	0.071	712.4386065	78	Grand Index
20	f	0-12 years	0.072	712.7381676	78	Grand Index
20	f	0-12 years	0.073	713.1613682	78	Grand Index

Age	Gender	Education	Percentile	Raw Score	Norm Score	Assessment
20	f	0-12 years	0.074	713.6663754	78	Grand Index
20	f	0-12 years	0.075	714.2866525	78	Grand Index
20	f	0-12 years	0.076	715.131322	79	Grand Index
20	f	0-12 years	0.077	715.9140424	79	Grand Index
20	f	0-12 years	0.078	715.9533729	79	Grand Index
20	f	0-12 years	0.079	715.9927034	79	Grand Index
20	f	0-12 years	0.08	716.36	79	Grand Index
20	f	0-12 years	0.081	716.802	79	Grand Index
20	f	0-12 years	0.082	717.244	79	Grand Index
20	f	0-12 years	0.083	717.686	79	Grand Index
20	f	0-12 years	0.085	718	79	Grand Index
20	f	0-12 years	0.086	718.012	80	Grand Index
20	f	0-12 years	0.087	718.454	80	Grand Index
20	f	0-12 years	0.088	718.896	80	Grand Index
20	f	0-12 years	0.089	719.338	80	Grand Index
20	f	0-12 years	0.09	719.78	80	Grand Index
20	f	0-12 years	0.092	720	80	Grand Index
20	f	0-12 years	0.093	720.212	80	Grand Index
20	f	0-12 years	0.094	721.096	80	Grand Index
20	f	0-12 years	0.095	721.98	80	Grand Index
20	f	0-12 years	0.096	722.864	80	Grand Index
20	f	0-12 years	0.097	723.748	81	Grand Index
20	f	0-12 years	0.099	724	81	Grand Index
20	f	0-12 years	0.1	724.1246802	81	Grand Index
20	f	0-12 years	0.101	724.4002235	81	Grand Index
20	f	0-12 years	0.102	724.6323149	81	Grand Index
20	f	0-12 years	0.103	724.679219	81	Grand Index
20	f	0-12 years	0.104	724.7261231	81	Grand Index
20	f	0-12 years	0.105	724.8331439	81	Grand Index
20	f	0-12 years	0.106	724.9448568	81	Grand Index
20	f	0-12 years	0.107	724.9874776	81	Grand Index
20	f	0-12 years	0.108	724.9953174	81	Grand Index
20	f	0-12 years	0.11	725	82	Grand Index
20	f	0-12 years	0.111	725.124	82	Grand Index
20	f	0-12 years	0.112	726.008	82	Grand Index
20	f	0-12 years	0.113	726.892	82	Grand Index
20	f	0-12 years	0.114	727.388	82	Grand Index
20	f	0-12 years	0.115	727.83	82	Grand Index
20	f	0-12 years	0.116	728.272	82	Grand Index
20	f	0-12 years	0.117	728.714	82	Grand Index
20	f	0-12 years	0.122	729	83	Grand Index
20	f	0-12 years	0.123	729.366	83	Grand Index
20	f	0-12 years	0.124	729.808	83	Grand Index
20	f	0-12 years	0.125	730.75	83	Grand Index
20	f	0-12 years	0.126	732.076	83	Grand Index
20	f	0-12 years	0.131	733	83	Grand Index
20	f	0-12 years	0.132	734.032	83	Grand Index
20	f	0-12 years	0.133	735.358	83	Grand Index
20	f	0-12 years	0.134	736.0129365	83	Grand Index
20	f	0-12 years	0.135	736.0380151	83	Grand Index
20	f	0-12 years	0.136	736.1623842	84	Grand Index
20	f	0-12 years	0.137	736.5793056	84	Grand Index
20	f	0-12 years	0.138	736.996227	84	Grand Index
20	f	0-12 years	0.139	737.438	84	Grand Index
20	f	0-12 years	0.14	737.88	84	Grand Index
20	f	0-12 years	0.144	738	84	Grand Index

Age	Gender	Education	Percentile	Raw Score	Norm Score	Assessment
20	f	0-12 years	0.145	738.18	84	Grand Index
20	f	0-12 years	0.146	739.064	84	Grand Index
20	f	0-12 years	0.147	739.948	84	Grand Index
20	f	0-12 years	0.149	740	84	Grand Index
20	f	0-12 years	0.15	740.1326851	84	Grand Index
20	f	0-12 years	0.151	740.3281744	85	Grand Index
20	f	0-12 years	0.152	740.5449034	85	Grand Index
20	f	0-12 years	0.153	740.7914141	85	Grand Index
20	f	0-12 years	0.154	741.136	85	Grand Index
20	f	0-12 years	0.155	742.02	85	Grand Index
20	f	0-12 years	0.156	742.904	85	Grand Index
20	f	0-12 years	0.157	743.3915528	85	Grand Index
20	f	0-12 years	0.158	743.8308075	85	Grand Index
20	f	0-12 years	0.159	744.5515155	85	Grand Index
20	f	0-12 years	0.16	745.4382609	85	Grand Index
20	f	0-12 years	0.165	746	85	Grand Index
20	f	0-12 years	0.166	746.744	85	Grand Index
20	f	0-12 years	0.167	747.628	86	Grand Index
20	f	0-12 years	0.169	748	86	Grand Index
20	f	0-12 years	0.17	748.1075574	86	Grand Index
20	f	0-12 years	0.171	748.4471315	86	Grand Index
20	f	0-12 years	0.172	748.7738288	86	Grand Index
20	f	0-12 years	0.173	748.8762547	86	Grand Index
20	f	0-12 years	0.174	748.9786806	86	Grand Index
20	f	0-12 years	0.175	749.35	86	Grand Index
20	f	0-12 years	0.176	749.792	86	Grand Index
20	f	0-12 years	0.178	750	86	Grand Index
20	f	0-12 years	0.179	750.118	86	Grand Index
20	f	0-12 years	0.18	750.56	86	Grand Index
20	f	0-12 years	0.181	751.002	86	Grand Index
20	f	0-12 years	0.182	751.444	86	Grand Index
20	f	0-12 years	0.183	751.886	86	Grand Index
20	f	0-12 years	0.184	752.328	86	Grand Index
20	f	0-12 years	0.185	752.77	87	Grand Index
20	f	0-12 years	0.19	753	87	Grand Index
20	f	0-12 years	0.191	753.844	87	Grand Index
20	f	0-12 years	0.192	754.728	87	Grand Index
20	f	0-12 years	0.196	755	87	Grand Index
20	f	0-12 years	0.197	755.148	87	Grand Index
20	f	0-12 years	0.198	756.032	87	Grand Index
20	f	0-12 years	0.199	756.916	87	Grand Index
20	f	0-12 years	0.2	757.4	87	Grand Index
20	f	0-12 years	0.201	757.842	87	Grand Index
20	f	0-12 years	0.203	758	88	Grand Index
20	f	0-12 years	0.204	758.168	88	Grand Index
20	f	0-12 years	0.205	758.61	88	Grand Index
20	f	0-12 years	0.21	759	88	Grand Index
20	f	0-12 years	0.211	759.025839	88	Grand Index
20	f	0-12 years	0.212	759.0694299	88	Grand Index
20	f	0-12 years	0.213	759.2302231	88	Grand Index
20	f	0-12 years	0.214	759.6286322	88	Grand Index
20	f	0-12 years	0.219	760	88	Grand Index
20	f	0-12 years	0.22	760.24	88	Grand Index
20	f	0-12 years	0.221	760.682	88	Grand Index
20	f	0-12 years	0.23	761	89	Grand Index
20	f	0-12 years	0.231	761.102	89	Grand Index

Age	Gender	Education	Percentile	Raw Score	Norm Score	Assessment
20	f	0-12 years	0.232	761.544	89	Grand Index
20	f	0-12 years	0.233	761.986	89	Grand Index
20	f	0-12 years	0.242	762	90	Grand Index
20	f	0-12 years	0.243	762.406	90	Grand Index
20	f	0-12 years	0.244	762.848	90	Grand Index
20	f	0-12 years	0.248	763	90	Grand Index
20	f	0-12 years	0.249	763.058	90	Grand Index
20	f	0-12 years	0.25	763.5	90	Grand Index
20	f	0-12 years	0.251	763.942	90	Grand Index
20	f	0-12 years	0.253	764	90	Grand Index
20	f	0-12 years	0.254	764.268	90	Grand Index
20	f	0-12 years	0.255	764.71	90	Grand Index
20	f	0-12 years	0.256	765.304	90	Grand Index
20	f	0-12 years	0.257	766.188	90	Grand Index
20	f	0-12 years	0.264	767	91	Grand Index
20	f	0-12 years	0.265	767.13	91	Grand Index
20	f	0-12 years	0.266	767.572	91	Grand Index
20	f	0-12 years	0.273	768	91	Grand Index
20	f	0-12 years	0.274	768.108	91	Grand Index
20	f	0-12 years	0.275	768.55	91	Grand Index
20	f	0-12 years	0.276	768.992	91	Grand Index
20	f	0-12 years	0.277	769.434	91	Grand Index
20	f	0-12 years	0.278	769.876	91	Grand Index
20	f	0-12 years	0.28	770	91	Grand Index
20	f	0-12 years	0.281	770.202	91	Grand Index
20	f	0-12 years	0.282	770.644	91	Grand Index
20	f	0-12 years	0.287	771	92	Grand Index
20	f	0-12 years	0.288	771.296	92	Grand Index
20	f	0-12 years	0.289	771.738	92	Grand Index
20	f	0-12 years	0.291	772	92	Grand Index
20	f	0-12 years	0.292	772.064	92	Grand Index
20	f	0-12 years	0.293	772.506	92	Grand Index
20	f	0-12 years	0.294	772.948	92	Grand Index
20	f	0-12 years	0.295	773.78	92	Grand Index
20	f	0-12 years	0.296	774.664	92	Grand Index
20	f	0-12 years	0.303	775	92	Grand Index
20	f	0-12 years	0.304	775.368	92	Grand Index
20	f	0-12 years	0.305	775.81	92	Grand Index
20	f	0-12 years	0.306	776.252	92	Grand Index
20	f	0-12 years	0.307	776.694	92	Grand Index
20	f	0-12 years	0.312	777	93	Grand Index
20	f	0-12 years	0.313	777.3403794	93	Grand Index
20	f	0-12 years	0.314	777.7751993	93	Grand Index
20	f	0-12 years	0.315	777.9874917	93	Grand Index
20	f	0-12 years	0.316	777.9946718	93	Grand Index
20	f	0-12 years	0.319	778	93	Grand Index
20	f	0-12 years	0.32	778.44	93	Grand Index
20	f	0-12 years	0.321	778.882	93	Grand Index
20	f	0-12 years	0.322	779.324	93	Grand Index
20	f	0-12 years	0.323	779.766	93	Grand Index
20	f	0-12 years	0.328	780	93	Grand Index
20	f	0-12 years	0.329	780.8457384	93	Grand Index
20	f	0-12 years	0.33	781.740036	93	Grand Index
20	f	0-12 years	0.331	782.3182618	93	Grand Index
20	f	0-12 years	0.332	782.7499642	93	Grand Index
20	f	0-12 years	0.337	783	94	Grand Index

Age	Gender	Education	Percentile	Raw Score	Norm Score	Assessment
20	f	0-12 years	0.338	783.396	94	Grand Index
20	f	0-12 years	0.339	783.838	94	Grand Index
20	f	0-12 years	0.346	784	94	Grand Index
20	f	0-12 years	0.347	784.1196581	94	Grand Index
20	f	0-12 years	0.348	784.2610723	94	Grand Index
20	f	0-12 years	0.349	784.4953966	94	Grand Index
20	f	0-12 years	0.35	784.7959825	94	Grand Index
20	f	0-12 years	0.351	785.142	94	Grand Index
20	f	0-12 years	0.352	785.584	94	Grand Index
20	f	0-12 years	0.359	786	95	Grand Index
20	f	0-12 years	0.36	786.1021047	95	Grand Index
20	f	0-12 years	0.361	786.4781902	95	Grand Index
20	f	0-12 years	0.362	786.8514688	95	Grand Index
20	f	0-12 years	0.363	786.9173832	95	Grand Index
20	f	0-12 years	0.364	786.9832977	95	Grand Index
20	f	0-12 years	0.366	787	95	Grand Index
20	f	0-12 years	0.367	787.214	95	Grand Index
20	f	0-12 years	0.368	787.656	95	Grand Index
20	f	0-12 years	0.377	788	95	Grand Index
20	f	0-12 years	0.378	788.076	95	Grand Index
20	f	0-12 years	0.379	788.518	95	Grand Index
20	f	0-12 years	0.38	788.96	95	Grand Index
20	f	0-12 years	0.384	789	96	Grand Index
20	f	0-12 years	0.385	789.17	96	Grand Index
20	f	0-12 years	0.386	789.612	96	Grand Index
20	f	0-12 years	0.393	790	96	Grand Index
20	f	0-12 years	0.394	790.148	96	Grand Index
20	f	0-12 years	0.395	790.59	96	Grand Index
20	f	0-12 years	0.398	791	96	Grand Index
20	f	0-12 years	0.399	791.358	96	Grand Index
20	f	0-12 years	0.4	791.8	96	Grand Index
20	f	0-12 years	0.402	792	96	Grand Index
20	f	0-12 years	0.403	792.126	96	Grand Index
20	f	0-12 years	0.404	792.568	96	Grand Index
20	f	0-12 years	0.418	793	97	Grand Index
20	f	0-12 years	0.419	793.198	97	Grand Index
20	f	0-12 years	0.42	793.64	97	Grand Index
20	f	0-12 years	0.421	794.164	97	Grand Index
20	f	0-12 years	0.422	795.048	97	Grand Index
20	f	0-12 years	0.423	795.932	97	Grand Index
20	f	0-12 years	0.427	796	97	Grand Index
20	f	0-12 years	0.428	796.0535901	97	Grand Index
20	f	0-12 years	0.429	796.1881744	97	Grand Index
20	f	0-12 years	0.43	796.3376376	97	Grand Index
20	f	0-12 years	0.431	796.5818298	97	Grand Index
20	f	0-12 years	0.432	796.826022	97	Grand Index
20	f	0-12 years	0.433	796.9121737	97	Grand Index
20	f	0-12 years	0.434	796.9753972	98	Grand Index
20	f	0-12 years	0.438	797	98	Grand Index
20	f	0-12 years	0.439	797.038	98	Grand Index
20	f	0-12 years	0.44	797.48	98	Grand Index
20	f	0-12 years	0.441	797.922	98	Grand Index
20	f	0-12 years	0.445	798	98	Grand Index
20	f	0-12 years	0.446	798.132	98	Grand Index
20	f	0-12 years	0.447	798.574	98	Grand Index
20	f	0-12 years	0.45	799	98	Grand Index

Age	Gender	Education	Percentile	Raw Score	Norm Score	Assessment
20	f	0-12 years	0.451	799.684	98	Grand Index
20	f	0-12 years	0.452	800.568	98	Grand Index
20	f	0-12 years	0.457	801	98	Grand Index
20	f	0-12 years	0.458	801.1742978	98	Grand Index
20	f	0-12 years	0.459	801.3509941	98	Grand Index
20	f	0-12 years	0.46	801.5918406	98	Grand Index
20	f	0-12 years	0.461	801.8571442	99	Grand Index
20	f	0-12 years	0.463	802	99	Grand Index
20	f	0-12 years	0.464	802.088	99	Grand Index
20	f	0-12 years	0.465	802.53	99	Grand Index
20	f	0-12 years	0.466	802.972	99	Grand Index
20	f	0-12 years	0.468	803	99	Grand Index
20	f	0-12 years	0.469	803.298	99	Grand Index
20	f	0-12 years	0.47	803.74	99	Grand Index
20	f	0-12 years	0.475	804	99	Grand Index
20	f	0-12 years	0.476	804.392	99	Grand Index
20	f	0-12 years	0.477	804.834	99	Grand Index
20	f	0-12 years	0.478	805.276	99	Grand Index
20	f	0-12 years	0.479	805.718	99	Grand Index
20	f	0-12 years	0.48	806.16	99	Grand Index
20	f	0-12 years	0.481	806.602	99	Grand Index
20	f	0-12 years	0.486	807	99	Grand Index
20	f	0-12 years	0.487	807.254	100	Grand Index
20	f	0-12 years	0.488	807.696	100	Grand Index
20	f	0-12 years	0.489	808.1067737	100	Grand Index
20	f	0-12 years	0.49	808.4487591	100	Grand Index
20	f	0-12 years	0.491	808.7787007	100	Grand Index
20	f	0-12 years	0.492	808.8787153	100	Grand Index
20	f	0-12 years	0.493	808.9787299	100	Grand Index
20	f	0-12 years	0.497	809	100	Grand Index
20	f	0-12 years	0.498	809.0976586	100	Grand Index
20	f	0-12 years	0.499	809.4697716	100	Grand Index
20	f	0-12 years	0.5	809.8418845	100	Grand Index
20	f	0-12 years	0.501	810.3537716	100	Grand Index
20	f	0-12 years	0.502	810.8656586	100	Grand Index
20	f	0-12 years	0.503	811.326	100	Grand Index
20	f	0-12 years	0.504	811.768	100	Grand Index
20	f	0-12 years	0.506	812	100	Grand Index
20	f	0-12 years	0.507	812.0747148	100	Grand Index
20	f	0-12 years	0.508	812.4260335	100	Grand Index
20	f	0-12 years	0.509	812.7773523	100	Grand Index
20	f	0-12 years	0.51	812.8810065	100	Grand Index
20	f	0-12 years	0.511	812.9716877	100	Grand Index
20	f	0-12 years	0.52	813	101	Grand Index
20	f	0-12 years	0.521	813.282	101	Grand Index
20	f	0-12 years	0.522	813.724	101	Grand Index
20	f	0-12 years	0.523	814.166	101	Grand Index
20	f	0-12 years	0.524	814.608	101	Grand Index
20	f	0-12 years	0.527	815	101	Grand Index
20	f	0-12 years	0.528	815.752	101	Grand Index
20	f	0-12 years	0.529	816.636	101	Grand Index
20	f	0-12 years	0.53	817.26	101	Grand Index
20	f	0-12 years	0.531	817.702	101	Grand Index
20	f	0-12 years	0.532	818.012	101	Grand Index
20	f	0-12 years	0.533	818.0488333	101	Grand Index
20	f	0-12 years	0.534	818.109	101	Grand Index

Age	Gender	Education	Percentile	Raw Score	Norm Score	Assessment
20	f	0-12 years	0.535	818.5141667	101	Grand Index
20	f	0-12 years	0.536	818.9193333	101	Grand Index
20	f	0-12 years	0.54	819	102	Grand Index
20	f	0-12 years	0.541	819.122	102	Grand Index
20	f	0-12 years	0.542	819.564	102	Grand Index
20	f	0-12 years	0.545	820	102	Grand Index
20	f	0-12 years	0.546	820.332	102	Grand Index
20	f	0-12 years	0.547	820.774	102	Grand Index
20	f	0-12 years	0.548	821.6333693	102	Grand Index
20	f	0-12 years	0.549	822.9294304	102	Grand Index
20	f	0-12 years	0.55	824.0390386	102	Grand Index
20	f	0-12 years	0.551	824.5109774	102	Grand Index
20	f	0-12 years	0.552	824.9829162	102	Grand Index
20	f	0-12 years	0.553	825.4080438	102	Grand Index
20	f	0-12 years	0.554	825.8314132	102	Grand Index
20	f	0-12 years	0.555	825.9709161	102	Grand Index
20	f	0-12 years	0.556	825.9895466	102	Grand Index
20	f	0-12 years	0.561	826	102	Grand Index
20	f	0-12 years	0.562	826.404	102	Grand Index
20	f	0-12 years	0.563	826.846	102	Grand Index
20	f	0-12 years	0.567	827	103	Grand Index
20	f	0-12 years	0.568	827.0409848	103	Grand Index
20	f	0-12 years	0.569	827.3644722	103	Grand Index
20	f	0-12 years	0.57	827.6879595	103	Grand Index
20	f	0-12 years	0.571	827.8342968	103	Grand Index
20	f	0-12 years	0.572	827.9528094	103	Grand Index
20	f	0-12 years	0.574	828	103	Grand Index
20	f	0-12 years	0.575	828.1379847	103	Grand Index
20	f	0-12 years	0.576	828.5445795	103	Grand Index
20	f	0-12 years	0.577	828.9226212	103	Grand Index
20	f	0-12 years	0.578	828.9580264	103	Grand Index
20	f	0-12 years	0.579	828.9934316	103	Grand Index
20	f	0-12 years	0.581	829	103	Grand Index
20	f	0-12 years	0.582	829.732	103	Grand Index
20	f	0-12 years	0.583	831.058	103	Grand Index
20	f	0-12 years	0.588	832	103	Grand Index
20	f	0-12 years	0.589	832.338	103	Grand Index
20	f	0-12 years	0.59	832.78	103	Grand Index
20	f	0-12 years	0.591	833.222	103	Grand Index
20	f	0-12 years	0.592	833.664	103	Grand Index
20	f	0-12 years	0.593	834.106	104	Grand Index
20	f	0-12 years	0.594	834.548	104	Grand Index
20	f	0-12 years	0.595	834.99	104	Grand Index
20	f	0-12 years	0.597	835	104	Grand Index
20	f	0-12 years	0.598	835.316	104	Grand Index
20	f	0-12 years	0.599	835.758	104	Grand Index
20	f	0-12 years	0.601	836	104	Grand Index
20	f	0-12 years	0.602	836.084	104	Grand Index
20	f	0-12 years	0.603	836.526	104	Grand Index
20	f	0-12 years	0.604	836.968	104	Grand Index
20	f	0-12 years	0.606	837	104	Grand Index
20	f	0-12 years	0.607	837.1696755	104	Grand Index
20	f	0-12 years	0.608	837.424766	104	Grand Index
20	f	0-12 years	0.609	837.6523989	104	Grand Index
20	f	0-12 years	0.61	837.8393085	104	Grand Index
20	f	0-12 years	0.615	838	104	Grand Index

Age	Gender	Education	Percentile	Raw Score	Norm Score	Assessment
20	f	0-12 years	0.616	838.272	104	Grand Index
20	f	0-12 years	0.617	838.714	104	Grand Index
20	f	0-12 years	0.618	839.156	105	Grand Index
20	f	0-12 years	0.619	839.598	105	Grand Index
20	f	0-12 years	0.626	840	105	Grand Index
20	f	0-12 years	0.627	840.134	105	Grand Index
20	f	0-12 years	0.628	840.576	105	Grand Index
20	f	0-12 years	0.631	841	105	Grand Index
20	f	0-12 years	0.632	841.344	105	Grand Index
20	f	0-12 years	0.633	841.786	105	Grand Index
20	f	0-12 years	0.635	842	105	Grand Index
20	f	0-12 years	0.636	842.112	105	Grand Index
20	f	0-12 years	0.637	842.554	105	Grand Index
20	f	0-12 years	0.638	842.996	105	Grand Index
20	f	0-12 years	0.64	843	105	Grand Index
20	f	0-12 years	0.641	843.322	105	Grand Index
20	f	0-12 years	0.642	843.764	105	Grand Index
20	f	0-12 years	0.649	844	106	Grand Index
20	f	0-12 years	0.65	844.1642735	106	Grand Index
20	f	0-12 years	0.651	844.4063032	106	Grand Index
20	f	0-12 years	0.652	844.630824	106	Grand Index
20	f	0-12 years	0.653	844.8307943	106	Grand Index
20	f	0-12 years	0.656	845	106	Grand Index
20	f	0-12 years	0.657	845.394	106	Grand Index
20	f	0-12 years	0.658	845.836	106	Grand Index
20	f	0-12 years	0.662	846	106	Grand Index
20	f	0-12 years	0.663	846.046	106	Grand Index
20	f	0-12 years	0.664	846.488	106	Grand Index
20	f	0-12 years	0.665	846.93	106	Grand Index
20	f	0-12 years	0.671	847	107	Grand Index
20	f	0-12 years	0.672	847.024	107	Grand Index
20	f	0-12 years	0.673	847.466	107	Grand Index
20	f	0-12 years	0.674	847.908	107	Grand Index
20	f	0-12 years	0.678	848	107	Grand Index
20	f	0-12 years	0.679	848.118	107	Grand Index
20	f	0-12 years	0.68	848.56	107	Grand Index
20	f	0-12 years	0.681	849.002	107	Grand Index
20	f	0-12 years	0.682	849.444	107	Grand Index
20	f	0-12 years	0.683	849.886	107	Grand Index
20	f	0-12 years	0.684	850.328	107	Grand Index
20	f	0-12 years	0.685	850.77	107	Grand Index
20	f	0-12 years	0.687	851	107	Grand Index
20	f	0-12 years	0.688	851.096	107	Grand Index
20	f	0-12 years	0.689	851.538	107	Grand Index
20	f	0-12 years	0.69	851.98	107	Grand Index
20	f	0-12 years	0.696	852	108	Grand Index
20	f	0-12 years	0.697	852.0767683	108	Grand Index
20	f	0-12 years	0.698	852.5353031	108	Grand Index
20	f	0-12 years	0.699	852.993838	108	Grand Index
20	f	0-12 years	0.7	853.4224455	108	Grand Index
20	f	0-12 years	0.701	853.8479106	108	Grand Index
20	f	0-12 years	0.703	854	108	Grand Index
20	f	0-12 years	0.704	854.168	108	Grand Index
20	f	0-12 years	0.705	854.61	108	Grand Index
20	f	0-12 years	0.708	855	108	Grand Index
20	f	0-12 years	0.709	855.4583557	108	Grand Index

Age	Gender	Education	Percentile	Raw Score	Norm Score	Assessment
20	f	0-12 years	0.71	855.9943167	108	Grand Index
20	f	0-12 years	0.711	856.3428205	108	Grand Index
20	f	0-12 years	0.712	856.562537	108	Grand Index
20	f	0-12 years	0.713	856.7520645	108	Grand Index
20	f	0-12 years	0.714	856.8803871	108	Grand Index
20	f	0-12 years	0.715	857.03	109	Grand Index
20	f	0-12 years	0.716	857.472	109	Grand Index
20	f	0-12 years	0.717	857.914	109	Grand Index
20	f	0-12 years	0.718	858.356	109	Grand Index
20	f	0-12 years	0.719	858.798	109	Grand Index
20	f	0-12 years	0.721	859	109	Grand Index
20	f	0-12 years	0.722	859.124	109	Grand Index
20	f	0-12 years	0.723	859.566	109	Grand Index
20	f	0-12 years	0.73	860	109	Grand Index
20	f	0-12 years	0.731	860.0021008	109	Grand Index
20	f	0-12 years	0.732	860.011204	109	Grand Index
20	f	0-12 years	0.733	860.0203073	109	Grand Index
20	f	0-12 years	0.734	860.4397807	109	Grand Index
20	f	0-12 years	0.735	860.8726774	109	Grand Index
20	f	0-12 years	0.736	861.312	109	Grand Index
20	f	0-12 years	0.737	861.754	110	Grand Index
20	f	0-12 years	0.739	862	110	Grand Index
20	f	0-12 years	0.74	862.08	110	Grand Index
20	f	0-12 years	0.741	862.522	110	Grand Index
20	f	0-12 years	0.742	862.964	110	Grand Index
20	f	0-12 years	0.743	863.406	110	Grand Index
20	f	0-12 years	0.744	863.848	110	Grand Index
20	f	0-12 years	0.745	864.29	110	Grand Index
20	f	0-12 years	0.746	864.732	110	Grand Index
20	f	0-12 years	0.748	865	110	Grand Index
20	f	0-12 years	0.749	865.058	110	Grand Index
20	f	0-12 years	0.75	865.5	110	Grand Index
20	f	0-12 years	0.751	865.942	110	Grand Index
20	f	0-12 years	0.755	866	110	Grand Index
20	f	0-12 years	0.756	866.152	110	Grand Index
20	f	0-12 years	0.757	866.594	110	Grand Index
20	f	0-12 years	0.762	867	111	Grand Index
20	f	0-12 years	0.763	867.0073985	111	Grand Index
20	f	0-12 years	0.764	867.0206917	111	Grand Index
20	f	0-12 years	0.765	867.1361354	111	Grand Index
20	f	0-12 years	0.766	867.4967401	111	Grand Index
20	f	0-12 years	0.767	867.8479084	111	Grand Index
20	f	0-12 years	0.768	867.9105917	111	Grand Index
20	f	0-12 years	0.769	867.973275	111	Grand Index
20	f	0-12 years	0.77	867.9919087	111	Grand Index
20	f	0-12 years	0.771	867.9973274	111	Grand Index
20	f	0-12 years	0.772	868.224	111	Grand Index
20	f	0-12 years	0.773	868.666	111	Grand Index
20	f	0-12 years	0.774	869.0068203	111	Grand Index
20	f	0-12 years	0.775	869.0347328	111	Grand Index
20	f	0-12 years	0.776	869.0626454	111	Grand Index
20	f	0-12 years	0.777	869.9037432	111	Grand Index
20	f	0-12 years	0.778	870.7598307	111	Grand Index
20	f	0-12 years	0.779	871.318	112	Grand Index
20	f	0-12 years	0.78	871.76	112	Grand Index
20	f	0-12 years	0.785	872	112	Grand Index

Age	Gender	Education	Percentile	Raw Score	Norm Score	Assessment
20	f	0-12 years	0.786	872.412	112	Grand Index
20	f	0-12 years	0.787	872.854	112	Grand Index
20	f	0-12 years	0.788	873.592	112	Grand Index
20	f	0-12 years	0.789	874.476	112	Grand Index
20	f	0-12 years	0.79	875.0326118	112	Grand Index
20	f	0-12 years	0.791	875.1126918	112	Grand Index
20	f	0-12 years	0.792	875.2335812	112	Grand Index
20	f	0-12 years	0.793	875.5955012	112	Grand Index
20	f	0-12 years	0.794	875.9574212	112	Grand Index
20	f	0-12 years	0.796	876	112	Grand Index
20	f	0-12 years	0.797	876.274	112	Grand Index
20	f	0-12 years	0.798	876.716	113	Grand Index
20	f	0-12 years	0.803	877	113	Grand Index
20	f	0-12 years	0.804	877.368	113	Grand Index
20	f	0-12 years	0.805	877.81	113	Grand Index
20	f	0-12 years	0.809	878	113	Grand Index
20	f	0-12 years	0.81	878.02	113	Grand Index
20	f	0-12 years	0.811	878.462	113	Grand Index
20	f	0-12 years	0.812	878.904	113	Grand Index
20	f	0-12 years	0.813	879.2122716	113	Grand Index
20	f	0-12 years	0.814	879.4834394	113	Grand Index
20	f	0-12 years	0.815	879.7023964	113	Grand Index
20	f	0-12 years	0.816	879.8732286	114	Grand Index
20	f	0-12 years	0.817	880.0004752	114	Grand Index
20	f	0-12 years	0.818	880.0023177	114	Grand Index
20	f	0-12 years	0.819	880.0041603	114	Grand Index
20	f	0-12 years	0.82	880.8823344	114	Grand Index
20	f	0-12 years	0.821	881.7644919	114	Grand Index
20	f	0-12 years	0.823	882	114	Grand Index
20	f	0-12 years	0.824	882.208	114	Grand Index
20	f	0-12 years	0.825	882.65	114	Grand Index
20	f	0-12 years	0.826	883.092	114	Grand Index
20	f	0-12 years	0.827	883.534	114	Grand Index
20	f	0-12 years	0.828	883.976	114	Grand Index
20	f	0-12 years	0.83	884	114	Grand Index
20	f	0-12 years	0.831	884.302	114	Grand Index
20	f	0-12 years	0.832	884.744	114	Grand Index
20	f	0-12 years	0.833	885.0023261	114	Grand Index
20	f	0-12 years	0.834	885.0078536	115	Grand Index
20	f	0-12 years	0.835	885.0665271	115	Grand Index
20	f	0-12 years	0.836	885.4076333	115	Grand Index
20	f	0-12 years	0.837	885.7487395	115	Grand Index
20	f	0-12 years	0.838	885.8696805	115	Grand Index
20	f	0-12 years	0.839	885.9650468	115	Grand Index
20	f	0-12 years	0.84	886.28	115	Grand Index
20	f	0-12 years	0.841	886.722	115	Grand Index
20	f	0-12 years	0.842	887.492	115	Grand Index
20	f	0-12 years	0.843	888.818	115	Grand Index
20	f	0-12 years	0.85	890	116	Grand Index
20	f	0-12 years	0.851	890.142	116	Grand Index
20	f	0-12 years	0.852	890.584	116	Grand Index
20	f	0-12 years	0.853	891.026	116	Grand Index
20	f	0-12 years	0.854	891.468	116	Grand Index
20	f	0-12 years	0.855	891.91	116	Grand Index
20	f	0-12 years	0.856	893.056	116	Grand Index
20	f	0-12 years	0.857	894.382	116	Grand Index

Age	Gender	Education	Percentile	Raw Score	Norm Score	Assessment
20	f	0-12 years	0.859	895	116	Grand Index
20	f	0-12 years	0.86	895.12	116	Grand Index
20	f	0-12 years	0.861	895.562	116	Grand Index
20	f	0-12 years	0.862	896.008	116	Grand Index
20	f	0-12 years	0.863	896.892	116	Grand Index
20	f	0-12 years	0.864	897.776	116	Grand Index
20	f	0-12 years	0.866	898	117	Grand Index
20	f	0-12 years	0.867	898.428	117	Grand Index
20	f	0-12 years	0.868	899.312	117	Grand Index
20	f	0-12 years	0.877	900	117	Grand Index
20	f	0-12 years	0.878	900.076	117	Grand Index
20	f	0-12 years	0.879	900.518	118	Grand Index
20	f	0-12 years	0.88	900.96	118	Grand Index
20	f	0-12 years	0.884	901	118	Grand Index
20	f	0-12 years	0.885	901.17	118	Grand Index
20	f	0-12 years	0.886	901.612	118	Grand Index
20	f	0-12 years	0.887	902.054	118	Grand Index
20	f	0-12 years	0.888	902.496	118	Grand Index
20	f	0-12 years	0.889	902.938	118	Grand Index
20	f	0-12 years	0.89	903.76	118	Grand Index
20	f	0-12 years	0.891	904.644	118	Grand Index
20	f	0-12 years	0.895	905	119	Grand Index
20	f	0-12 years	0.896	905.064	119	Grand Index
20	f	0-12 years	0.897	905.948	119	Grand Index
20	f	0-12 years	0.898	906.832	119	Grand Index
20	f	0-12 years	0.9	907	119	Grand Index
20	f	0-12 years	0.901	907.484	119	Grand Index
20	f	0-12 years	0.902	908.368	119	Grand Index
20	f	0-12 years	0.903	909.2109547	119	Grand Index
20	f	0-12 years	0.904	909.9509704	120	Grand Index
20	f	0-12 years	0.905	910.6900116	120	Grand Index
20	f	0-12 years	0.906	911.3869575	120	Grand Index
20	f	0-12 years	0.907	912.0839033	120	Grand Index
20	f	0-12 years	0.908	912.5026933	120	Grand Index
20	f	0-12 years	0.909	912.8337318	120	Grand Index
20	f	0-12 years	0.911	913	120	Grand Index
20	f	0-12 years	0.912	913.104	120	Grand Index
20	f	0-12 years	0.913	913.546	120	Grand Index
20	f	0-12 years	0.914	913.988	120	Grand Index
20	f	0-12 years	0.915	914.0277339	121	Grand Index
20	f	0-12 years	0.916	914.0562417	121	Grand Index
20	f	0-12 years	0.917	914.3461749	121	Grand Index
20	f	0-12 years	0.918	914.7426764	121	Grand Index
20	f	0-12 years	0.919	914.9691708	121	Grand Index
20	f	0-12 years	0.92	914.9861614	121	Grand Index
20	f	0-12 years	0.923	915	121	Grand Index
20	f	0-12 years	0.924	915.408	121	Grand Index
20	f	0-12 years	0.925	915.85	122	Grand Index
20	f	0-12 years	0.926	916.292	122	Grand Index
20	f	0-12 years	0.927	916.734	122	Grand Index
20	f	0-12 years	0.928	917.528	122	Grand Index
20	f	0-12 years	0.929	918.854	122	Grand Index
20	f	0-12 years	0.932	920	122	Grand Index
20	f	0-12 years	0.933	921.5277434	122	Grand Index
20	f	0-12 years	0.934	923.2771283	123	Grand Index
20	f	0-12 years	0.935	923.9692556	123	Grand Index

Age	Gender	Education	Percentile	Raw Score	Norm Score	Assessment
20	f	0-12 years	0.936	923.9878707	123	Grand Index
20	f	0-12 years	0.938	924	123	Grand Index
20	f	0-12 years	0.939	924.0214067	123	Grand Index
20	f	0-12 years	0.94	924.2704005	123	Grand Index
20	f	0-12 years	0.941	924.5193944	123	Grand Index
20	f	0-12 years	0.942	924.7222807	124	Grand Index
20	f	0-12 years	0.943	924.9152869	124	Grand Index
20	f	0-12 years	0.944	925.7626166	124	Grand Index
20	f	0-12 years	0.945	927.1217962	124	Grand Index
20	f	0-12 years	0.946	928.3291582	124	Grand Index
20	f	0-12 years	0.947	929.1799786	124	Grand Index
20	f	0-12 years	0.948	930.032	124	Grand Index
20	f	0-12 years	0.949	930.916	125	Grand Index
20	f	0-12 years	0.95	931.8	125	Grand Index
20	f	0-12 years	0.951	932.5583424	125	Grand Index
20	f	0-12 years	0.952	933.2799429	125	Grand Index
20	f	0-12 years	0.953	933.7156171	125	Grand Index
20	f	0-12 years	0.954	933.8780166	125	Grand Index
20	f	0-12 years	0.957	934	126	Grand Index
20	f	0-12 years	0.958	934.872	126	Grand Index
20	f	0-12 years	0.959	935.756	126	Grand Index
20	f	0-12 years	0.96	937.28	126	Grand Index
20	f	0-12 years	0.961	939.048	126	Grand Index
20	f	0-12 years	0.962	940.816	127	Grand Index
20	f	0-12 years	0.963	942.584	127	Grand Index
20	f	0-12 years	0.964	944.352	127	Grand Index
20	f	0-12 years	0.965	946.12	127	Grand Index
20	f	0-12 years	0.966	947.888	127	Grand Index
20	f	0-12 years	0.967	949.242	128	Grand Index
20	f	0-12 years	0.968	950.568	128	Grand Index
20	f	0-12 years	0.97	951	128	Grand Index
20	f	0-12 years	0.971	951.381514	128	Grand Index
20	f	0-12 years	0.972	952.3080481	129	Grand Index
20	f	0-12 years	0.973	953.6838797	129	Grand Index
20	f	0-12 years	0.974	957.6193456	129	Grand Index
20	f	0-12 years	0.975	961.5548115	129	Grand Index
20	f	0-12 years	0.976	962.392	130	Grand Index
20	f	0-12 years	0.977	962.834	130	Grand Index
20	f	0-12 years	0.978	963.828	130	Grand Index
20	f	0-12 years	0.979	965.154	131	Grand Index
20	f	0-12 years	0.98	966.16	131	Grand Index
20	f	0-12 years	0.981	966.602	131	Grand Index
20	f	0-12 years	0.982	967.044	131	Grand Index
20	f	0-12 years	0.983	967.486	132	Grand Index
20	f	0-12 years	0.984	967.928	132	Grand Index
20	f	0-12 years	0.985	971.1250737	133	Grand Index
20	f	0-12 years	0.986	974.8582698	133	Grand Index
20	f	0-12 years	0.987	977.1015047	133	Grand Index
20	f	0-12 years	0.988	978.2419336	134	Grand Index
20	f	0-12 years	0.989	979.0583487	134	Grand Index
20	f	0-12 years	0.99	979.1609942	135	Grand Index
20	f	0-12 years	0.991	979.3149999	135	Grand Index
20	f	0-12 years	0.992	980.4495192	136	Grand Index
20	f	0-12 years	0.993	981.5840386	137	Grand Index
20	f	0-12 years	0.994	989.4637406	138	Grand Index
20	f	0-12 years	0.995	999.1654173	139	Grand Index

Age	Gender	Education	Percentile	Raw Score	Norm Score	Assessment
20	f	0-12 years	0.996	1006.379055	140	Grand Index
20	f	0-12 years	0.997	1011.340588	141	Grand Index
20	f	0-12 years	0.998	1018.574186	143	Grand Index
20	f	0-12 years	0.999	1032.193069	146	Grand Index
20	m	0-12 years	0.001	600.5119777	54	Grand Index
20	m	0-12 years	0.002	616.0597998	57	Grand Index
20	m	0-12 years	0.003	633.9996995	59	Grand Index
20	m	0-12 years	0.004	644.32	60	Grand Index
20	m	0-12 years	0.005	647.7416175	61	Grand Index
20	m	0-12 years	0.006	653.0119043	62	Grand Index
20	m	0-12 years	0.007	653.6051228	63	Grand Index
20	m	0-12 years	0.008	655.7932539	64	Grand Index
20	m	0-12 years	0.009	658.8315722	65	Grand Index
20	m	0-12 years	0.01	660	65	Grand Index
20	m	0-12 years	0.011	660.98	66	Grand Index
20	m	0-12 years	0.012	661.4875345	66	Grand Index
20	m	0-12 years	0.013	662.34	67	Grand Index
20	m	0-12 years	0.014	663	67	Grand Index
20	m	0-12 years	0.015	663.4307692	67	Grand Index
20	m	0-12 years	0.016	665.2186693	68	Grand Index
20	m	0-12 years	0.017	665.5015152	68	Grand Index
20	m	0-12 years	0.018	666.1953994	69	Grand Index
20	m	0-12 years	0.019	667.3122152	69	Grand Index
20	m	0-12 years	0.02	668	69	Grand Index
20	m	0-12 years	0.021	668.78	69	Grand Index
20	m	0-12 years	0.022	669.96	70	Grand Index
20	m	0-12 years	0.023	672.14	70	Grand Index
20	m	0-12 years	0.024	673.64	70	Grand Index
20	m	0-12 years	0.025	675.5	71	Grand Index
20	m	0-12 years	0.026	676.5205212	71	Grand Index
20	m	0-12 years	0.027	677.8271661	71	Grand Index
20	m	0-12 years	0.028	678	71	Grand Index
20	m	0-12 years	0.029	678.6242604	72	Grand Index
20	m	0-12 years	0.03	681.3025284	72	Grand Index
20	m	0-12 years	0.031	682	72	Grand Index
20	m	0-12 years	0.032	683.52	72	Grand Index
20	m	0-12 years	0.033	684.2633675	72	Grand Index
20	m	0-12 years	0.034	685.12	73	Grand Index
20	m	0-12 years	0.035	686.6	73	Grand Index
20	m	0-12 years	0.038	688	73	Grand Index
20	m	0-12 years	0.04	689	74	Grand Index
20	m	0-12 years	0.041	689.3656716	74	Grand Index
20	m	0-12 years	0.042	689.9834093	74	Grand Index
20	m	0-12 years	0.043	690	74	Grand Index
20	m	0-12 years	0.044	690.1329729	74	Grand Index
20	m	0-12 years	0.045	694.1	75	Grand Index
20	m	0-12 years	0.047	695	75	Grand Index
20	m	0-12 years	0.048	695.64	75	Grand Index
20	m	0-12 years	0.049	696	75	Grand Index
20	m	0-12 years	0.05	697	75	Grand Index
20	m	0-12 years	0.051	697.0049471	75	Grand Index
20	m	0-12 years	0.052	697.6069635	76	Grand Index
20	m	0-12 years	0.053	699.2167137	76	Grand Index
20	m	0-12 years	0.054	699.9189168	76	Grand Index
20	m	0-12 years	0.055	700	76	Grand Index
20	m	0-12 years	0.056	700.16	76	Grand Index

Age	Gender	Education	Percentile	Raw Score	Norm Score	Assessment
20	m	0-12 years	0.059	702	77	Grand Index
20	m	0-12 years	0.06	702.8	77	Grand Index
20	m	0-12 years	0.061	703.0180141	77	Grand Index
20	m	0-12 years	0.062	704	77	Grand Index
20	m	0-12 years	0.063	704.0227706	77	Grand Index
20	m	0-12 years	0.064	704.5521467	77	Grand Index
20	m	0-12 years	0.066	705	77	Grand Index
20	m	0-12 years	0.067	705.06	78	Grand Index
20	m	0-12 years	0.068	706	78	Grand Index
20	m	0-12 years	0.069	706.42	78	Grand Index
20	m	0-12 years	0.07	707	78	Grand Index
20	m	0-12 years	0.071	707.3065727	78	Grand Index
20	m	0-12 years	0.072	707.9757217	78	Grand Index
20	m	0-12 years	0.074	708	78	Grand Index
20	m	0-12 years	0.075	708.5	78	Grand Index
20	m	0-12 years	0.076	709	79	Grand Index
20	m	0-12 years	0.077	709.86	79	Grand Index
20	m	0-12 years	0.078	710	79	Grand Index
20	m	0-12 years	0.079	710.2165485	79	Grand Index
20	m	0-12 years	0.08	710.9905869	79	Grand Index
20	m	0-12 years	0.081	711	79	Grand Index
20	m	0-12 years	0.082	711.76	79	Grand Index
20	m	0-12 years	0.086	712	80	Grand Index
20	m	0-12 years	0.087	712.66	80	Grand Index
20	m	0-12 years	0.088	713.0384975	80	Grand Index
20	m	0-12 years	0.089	714	80	Grand Index
20	m	0-12 years	0.09	714.2	80	Grand Index
20	m	0-12 years	0.091	715.38	80	Grand Index
20	m	0-12 years	0.093	716	80	Grand Index
20	m	0-12 years	0.094	716.1331932	80	Grand Index
20	m	0-12 years	0.095	716.218328	80	Grand Index
20	m	0-12 years	0.096	716.8281317	80	Grand Index
20	m	0-12 years	0.097	716.9637478	81	Grand Index
20	m	0-12 years	0.1	717	81	Grand Index
20	m	0-12 years	0.101	717.18	81	Grand Index
20	m	0-12 years	0.104	718	81	Grand Index
20	m	0-12 years	0.105	719.1829833	81	Grand Index
20	m	0-12 years	0.106	719.7371429	81	Grand Index
20	m	0-12 years	0.107	720	81	Grand Index
20	m	0-12 years	0.108	720.44	81	Grand Index
20	m	0-12 years	0.109	721	82	Grand Index
20	m	0-12 years	0.11	721.8	82	Grand Index
20	m	0-12 years	0.113	722	82	Grand Index
20	m	0-12 years	0.114	722.109726	82	Grand Index
20	m	0-12 years	0.115	722.7633035	82	Grand Index
20	m	0-12 years	0.117	723	82	Grand Index
20	m	0-12 years	0.118	723.24	82	Grand Index
20	m	0-12 years	0.12	724	82	Grand Index
20	m	0-12 years	0.121	724.78	82	Grand Index
20	m	0-12 years	0.123	725	83	Grand Index
20	m	0-12 years	0.124	725.0164497	83	Grand Index
20	m	0-12 years	0.125	726.0257027	83	Grand Index
20	m	0-12 years	0.127	727	83	Grand Index
20	m	0-12 years	0.128	727.1603266	83	Grand Index
20	m	0-12 years	0.129	727.4220663	83	Grand Index
20	m	0-12 years	0.132	728	83	Grand Index

Age	Gender	Education	Percentile	Raw Score	Norm Score	Assessment
20	m	0-12 years	0.133	728.94	83	Grand Index
20	m	0-12 years	0.135	729	83	Grand Index
20	m	0-12 years	0.136	729.2981213	84	Grand Index
20	m	0-12 years	0.137	729.8711693	84	Grand Index
20	m	0-12 years	0.138	730	84	Grand Index
20	m	0-12 years	0.139	730.1874143	84	Grand Index
20	m	0-12 years	0.141	731	84	Grand Index
20	m	0-12 years	0.142	731.1280307	84	Grand Index
20	m	0-12 years	0.143	731.7994428	84	Grand Index
20	m	0-12 years	0.144	732.92	84	Grand Index
20	m	0-12 years	0.15	733	84	Grand Index
20	m	0-12 years	0.151	734	85	Grand Index
20	m	0-12 years	0.152	734.3655385	85	Grand Index
20	m	0-12 years	0.153	735.0526039	85	Grand Index
20	m	0-12 years	0.154	735.7436066	85	Grand Index
20	m	0-12 years	0.156	736	85	Grand Index
20	m	0-12 years	0.157	736.0093882	85	Grand Index
20	m	0-12 years	0.158	736.4602208	85	Grand Index
20	m	0-12 years	0.161	737	85	Grand Index
20	m	0-12 years	0.162	737.4235294	85	Grand Index
20	m	0-12 years	0.165	738	85	Grand Index
20	m	0-12 years	0.166	738.88	85	Grand Index
20	m	0-12 years	0.168	739	86	Grand Index
20	m	0-12 years	0.169	739.1196781	86	Grand Index
20	m	0-12 years	0.17	739.3930489	86	Grand Index
20	m	0-12 years	0.171	739.5049512	86	Grand Index
20	m	0-12 years	0.172	739.9806475	86	Grand Index
20	m	0-12 years	0.173	740	86	Grand Index
20	m	0-12 years	0.174	740.32	86	Grand Index
20	m	0-12 years	0.177	741	86	Grand Index
20	m	0-12 years	0.178	741.0040501	86	Grand Index
20	m	0-12 years	0.179	741.2989771	86	Grand Index
20	m	0-12 years	0.182	742	86	Grand Index
20	m	0-12 years	0.183	742.3718681	86	Grand Index
20	m	0-12 years	0.188	743	87	Grand Index
20	m	0-12 years	0.189	743.0282108	87	Grand Index
20	m	0-12 years	0.192	744	87	Grand Index
20	m	0-12 years	0.193	744.6370435	87	Grand Index
20	m	0-12 years	0.194	744.9697863	87	Grand Index
20	m	0-12 years	0.2	745	87	Grand Index
20	m	0-12 years	0.203	746	88	Grand Index
20	m	0-12 years	0.204	746.4310815	88	Grand Index
20	m	0-12 years	0.205	746.9598724	88	Grand Index
20	m	0-12 years	0.211	748	88	Grand Index
20	m	0-12 years	0.212	748.0990183	88	Grand Index
20	m	0-12 years	0.213	748.7484507	88	Grand Index
20	m	0-12 years	0.215	749	88	Grand Index
20	m	0-12 years	0.216	749.0397291	88	Grand Index
20	m	0-12 years	0.217	750	88	Grand Index
20	m	0-12 years	0.218	750.24	88	Grand Index
20	m	0-12 years	0.22	751	88	Grand Index
20	m	0-12 years	0.221	751.78	88	Grand Index
20	m	0-12 years	0.224	752	89	Grand Index
20	m	0-12 years	0.225	752.1360013	89	Grand Index
20	m	0-12 years	0.226	752.7670408	89	Grand Index
20	m	0-12 years	0.228	753	89	Grand Index

Age	Gender	Education	Percentile	Raw Score	Norm Score	Assessment
20	m	0-12 years	0.229	753.22	89	Grand Index
20	m	0-12 years	0.236	754	89	Grand Index
20	m	0-12 years	0.237	754.3538012	89	Grand Index
20	m	0-12 years	0.238	754.92577	89	Grand Index
20	m	0-12 years	0.239	755	89	Grand Index
20	m	0-12 years	0.24	755.2	89	Grand Index
20	m	0-12 years	0.243	756	90	Grand Index
20	m	0-12 years	0.244	756.92	90	Grand Index
20	m	0-12 years	0.247	757	90	Grand Index
20	m	0-12 years	0.248	757.0428571	90	Grand Index
20	m	0-12 years	0.249	757.3814446	90	Grand Index
20	m	0-12 years	0.251	758	90	Grand Index
20	m	0-12 years	0.252	758.3566832	90	Grand Index
20	m	0-12 years	0.253	758.9957619	90	Grand Index
20	m	0-12 years	0.255	759	90	Grand Index
20	m	0-12 years	0.259	760	90	Grand Index
20	m	0-12 years	0.26	760.6059843	90	Grand Index
20	m	0-12 years	0.261	760.892812	90	Grand Index
20	m	0-12 years	0.263	761	90	Grand Index
20	m	0-12 years	0.264	761.52	91	Grand Index
20	m	0-12 years	0.266	762	91	Grand Index
20	m	0-12 years	0.269	763	91	Grand Index
20	m	0-12 years	0.27	763.6	91	Grand Index
20	m	0-12 years	0.281	764	91	Grand Index
20	m	0-12 years	0.282	764.76	91	Grand Index
20	m	0-12 years	0.284	765	91	Grand Index
20	m	0-12 years	0.285	765.2143395	91	Grand Index
20	m	0-12 years	0.286	765.8515218	92	Grand Index
20	m	0-12 years	0.289	766	92	Grand Index
20	m	0-12 years	0.29	766.2	92	Grand Index
20	m	0-12 years	0.294	767	92	Grand Index
20	m	0-12 years	0.297	768	92	Grand Index
20	m	0-12 years	0.298	768.4361062	92	Grand Index
20	m	0-12 years	0.299	768.9394943	92	Grand Index
20	m	0-12 years	0.302	769	92	Grand Index
20	m	0-12 years	0.303	769.54	92	Grand Index
20	m	0-12 years	0.312	770	93	Grand Index
20	m	0-12 years	0.313	770.34	93	Grand Index
20	m	0-12 years	0.314	771	93	Grand Index
20	m	0-12 years	0.315	771.4190361	93	Grand Index
20	m	0-12 years	0.316	771.9518348	93	Grand Index
20	m	0-12 years	0.317	772.0165	93	Grand Index
20	m	0-12 years	0.318	772.449	93	Grand Index
20	m	0-12 years	0.319	773	93	Grand Index
20	m	0-12 years	0.32	773.6	93	Grand Index
20	m	0-12 years	0.322	774	93	Grand Index
20	m	0-12 years	0.323	774.14	93	Grand Index
20	m	0-12 years	0.327	775	93	Grand Index
20	m	0-12 years	0.328	775.0391543	93	Grand Index
20	m	0-12 years	0.329	775.9835095	93	Grand Index
20	m	0-12 years	0.33	776	93	Grand Index
20	m	0-12 years	0.331	776.3440678	93	Grand Index
20	m	0-12 years	0.332	776.9023729	93	Grand Index
20	m	0-12 years	0.334	777	94	Grand Index
20	m	0-12 years	0.335	777.0930396	94	Grand Index
20	m	0-12 years	0.336	777.6412687	94	Grand Index

Age	Gender	Education	Percentile	Raw Score	Norm Score	Assessment
20	m	0-12 years	0.339	778	94	Grand Index
20	m	0-12 years	0.34	778.0600812	94	Grand Index
20	m	0-12 years	0.341	778.5662517	94	Grand Index
20	m	0-12 years	0.347	779	94	Grand Index
20	m	0-12 years	0.348	779.632611	94	Grand Index
20	m	0-12 years	0.349	779.9979218	94	Grand Index
20	m	0-12 years	0.351	780	94	Grand Index
20	m	0-12 years	0.352	780.36	94	Grand Index
20	m	0-12 years	0.356	781	94	Grand Index
20	m	0-12 years	0.357	781.0348877	95	Grand Index
20	m	0-12 years	0.358	781.5151427	95	Grand Index
20	m	0-12 years	0.361	782	95	Grand Index
20	m	0-12 years	0.362	782.1580932	95	Grand Index
20	m	0-12 years	0.363	782.9921343	95	Grand Index
20	m	0-12 years	0.366	783	95	Grand Index
20	m	0-12 years	0.367	783.0094376	95	Grand Index
20	m	0-12 years	0.368	783.3595424	95	Grand Index
20	m	0-12 years	0.372	784	95	Grand Index
20	m	0-12 years	0.373	784.8279601	95	Grand Index
20	m	0-12 years	0.374	784.9676741	95	Grand Index
20	m	0-12 years	0.377	785	95	Grand Index
20	m	0-12 years	0.378	785.0141718	95	Grand Index
20	m	0-12 years	0.379	785.4623307	95	Grand Index
20	m	0-12 years	0.38	785.907218	95	Grand Index
20	m	0-12 years	0.385	786	96	Grand Index
20	m	0-12 years	0.386	786.345	96	Grand Index
20	m	0-12 years	0.387	787.564375	96	Grand Index
20	m	0-12 years	0.389	788	96	Grand Index
20	m	0-12 years	0.39	788.2	96	Grand Index
20	m	0-12 years	0.392	789	96	Grand Index
20	m	0-12 years	0.393	789.74	96	Grand Index
20	m	0-12 years	0.398	790	96	Grand Index
20	m	0-12 years	0.399	790.82	96	Grand Index
20	m	0-12 years	0.4	791	96	Grand Index
20	m	0-12 years	0.405	792	96	Grand Index
20	m	0-12 years	0.406	792.0955493	96	Grand Index
20	m	0-12 years	0.411	793	97	Grand Index
20	m	0-12 years	0.412	793.0759061	97	Grand Index
20	m	0-12 years	0.413	793.6531128	97	Grand Index
20	m	0-12 years	0.415	794	97	Grand Index
20	m	0-12 years	0.416	794.0309677	97	Grand Index
20	m	0-12 years	0.421	795	97	Grand Index
20	m	0-12 years	0.422	795.0257143	97	Grand Index
20	m	0-12 years	0.423	796.0233999	97	Grand Index
20	m	0-12 years	0.424	796.2326535	97	Grand Index
20	m	0-12 years	0.425	796.6859324	97	Grand Index
20	m	0-12 years	0.427	797	97	Grand Index
20	m	0-12 years	0.428	798.04	97	Grand Index
20	m	0-12 years	0.437	799	98	Grand Index
20	m	0-12 years	0.438	799.2922465	98	Grand Index
20	m	0-12 years	0.439	800	98	Grand Index
20	m	0-12 years	0.44	800.1232993	98	Grand Index
20	m	0-12 years	0.441	800.7622279	98	Grand Index
20	m	0-12 years	0.444	801	98	Grand Index
20	m	0-12 years	0.445	801.1	98	Grand Index
20	m	0-12 years	0.448	802	98	Grand Index

Age	Gender	Education	Percentile	Raw Score	Norm Score	Assessment
20	m	0-12 years	0.449	802.2393789	98	Grand Index
20	m	0-12 years	0.45	803	98	Grand Index
20	m	0-12 years	0.451	803.18	98	Grand Index
20	m	0-12 years	0.454	804	98	Grand Index
20	m	0-12 years	0.455	804.9	98	Grand Index
20	m	0-12 years	0.46	805	98	Grand Index
20	m	0-12 years	0.461	805.781134	99	Grand Index
20	m	0-12 years	0.465	806	99	Grand Index
20	m	0-12 years	0.466	806.88	99	Grand Index
20	m	0-12 years	0.471	807	99	Grand Index
20	m	0-12 years	0.472	807.168927	99	Grand Index
20	m	0-12 years	0.477	808	99	Grand Index
20	m	0-12 years	0.478	808.04	99	Grand Index
20	m	0-12 years	0.48	809	99	Grand Index
20	m	0-12 years	0.481	809.58	99	Grand Index
20	m	0-12 years	0.486	810	99	Grand Index
20	m	0-12 years	0.487	810.66	100	Grand Index
20	m	0-12 years	0.492	811	100	Grand Index
20	m	0-12 years	0.493	811.74	100	Grand Index
20	m	0-12 years	0.494	812.92	100	Grand Index
20	m	0-12 years	0.497	813	100	Grand Index
20	m	0-12 years	0.498	813.5721832	100	Grand Index
20	m	0-12 years	0.499	813.9809265	100	Grand Index
20	m	0-12 years	0.504	814	100	Grand Index
20	m	0-12 years	0.505	814.3297414	100	Grand Index
20	m	0-12 years	0.514	815	101	Grand Index
20	m	0-12 years	0.515	815.7	101	Grand Index
20	m	0-12 years	0.521	816	101	Grand Index
20	m	0-12 years	0.522	816.9542023	101	Grand Index
20	m	0-12 years	0.528	817	101	Grand Index
20	m	0-12 years	0.529	817.1686732	101	Grand Index
20	m	0-12 years	0.53	817.8600178	101	Grand Index
20	m	0-12 years	0.533	818	101	Grand Index
20	m	0-12 years	0.534	818.8697674	101	Grand Index
20	m	0-12 years	0.543	819	102	Grand Index
20	m	0-12 years	0.544	819.92	102	Grand Index
20	m	0-12 years	0.545	820.0104803	102	Grand Index
20	m	0-12 years	0.546	820.2279049	102	Grand Index
20	m	0-12 years	0.547	820.7540038	102	Grand Index
20	m	0-12 years	0.549	821	102	Grand Index
20	m	0-12 years	0.55	821.0895522	102	Grand Index
20	m	0-12 years	0.555	822	102	Grand Index
20	m	0-12 years	0.556	822.08	102	Grand Index
20	m	0-12 years	0.56	823	102	Grand Index
20	m	0-12 years	0.561	823.98	102	Grand Index
20	m	0-12 years	0.565	824	102	Grand Index
20	m	0-12 years	0.566	824.7355224	102	Grand Index
20	m	0-12 years	0.567	825.06	103	Grand Index
20	m	0-12 years	0.57	826	103	Grand Index
20	m	0-12 years	0.571	826.4362712	103	Grand Index
20	m	0-12 years	0.572	826.9823729	103	Grand Index
20	m	0-12 years	0.579	827	103	Grand Index
20	m	0-12 years	0.58	827.4	103	Grand Index
20	m	0-12 years	0.583	828	103	Grand Index
20	m	0-12 years	0.584	828.9144073	103	Grand Index
20	m	0-12 years	0.586	829	103	Grand Index

Age	Gender	Education	Percentile	Raw Score	Norm Score	Assessment
20	m	0-12 years	0.587	829.218612	103	Grand Index
20	m	0-12 years	0.588	829.8929968	103	Grand Index
20	m	0-12 years	0.591	830	103	Grand Index
20	m	0-12 years	0.592	830.56	103	Grand Index
20	m	0-12 years	0.594	831	104	Grand Index
20	m	0-12 years	0.597	832	104	Grand Index
20	m	0-12 years	0.598	832.048172	104	Grand Index
20	m	0-12 years	0.599	832.8335484	104	Grand Index
20	m	0-12 years	0.601	833	104	Grand Index
20	m	0-12 years	0.602	833.3543844	104	Grand Index
20	m	0-12 years	0.603	833.9859891	104	Grand Index
20	m	0-12 years	0.604	833.9964557	104	Grand Index
20	m	0-12 years	0.61	834	104	Grand Index
20	m	0-12 years	0.611	834.9304615	104	Grand Index
20	m	0-12 years	0.612	834.9729703	104	Grand Index
20	m	0-12 years	0.615	835	104	Grand Index
20	m	0-12 years	0.616	835.8692025	104	Grand Index
20	m	0-12 years	0.619	836	105	Grand Index
20	m	0-12 years	0.62	836.2292683	105	Grand Index
20	m	0-12 years	0.621	836.6635675	105	Grand Index
20	m	0-12 years	0.622	836.9897181	105	Grand Index
20	m	0-12 years	0.624	837	105	Grand Index
20	m	0-12 years	0.625	837.5	105	Grand Index
20	m	0-12 years	0.63	838	105	Grand Index
20	m	0-12 years	0.631	838.58	105	Grand Index
20	m	0-12 years	0.633	839	105	Grand Index
20	m	0-12 years	0.634	839.12	105	Grand Index
20	m	0-12 years	0.637	840	105	Grand Index
20	m	0-12 years	0.638	840.84	105	Grand Index
20	m	0-12 years	0.64	841	105	Grand Index
20	m	0-12 years	0.641	841.38	105	Grand Index
20	m	0-12 years	0.646	842	106	Grand Index
20	m	0-12 years	0.647	842.46	106	Grand Index
20	m	0-12 years	0.649	843	106	Grand Index
20	m	0-12 years	0.655	844	106	Grand Index
20	m	0-12 years	0.656	844.9347097	106	Grand Index
20	m	0-12 years	0.657	845.26	106	Grand Index
20	m	0-12 years	0.661	846	106	Grand Index
20	m	0-12 years	0.662	846.16	106	Grand Index
20	m	0-12 years	0.665	847	106	Grand Index
20	m	0-12 years	0.666	847.0474861	106	Grand Index
20	m	0-12 years	0.669	848	107	Grand Index
20	m	0-12 years	0.67	848.0342741	107	Grand Index
20	m	0-12 years	0.671	848.1038963	107	Grand Index
20	m	0-12 years	0.672	848.2445321	107	Grand Index
20	m	0-12 years	0.673	848.8724949	107	Grand Index
20	m	0-12 years	0.675	849	107	Grand Index
20	m	0-12 years	0.676	849.0112211	107	Grand Index
20	m	0-12 years	0.677	849.6438975	107	Grand Index
20	m	0-12 years	0.682	850	107	Grand Index
20	m	0-12 years	0.683	850.94	107	Grand Index
20	m	0-12 years	0.685	851	107	Grand Index
20	m	0-12 years	0.686	851.0838095	107	Grand Index
20	m	0-12 years	0.687	851.5983	107	Grand Index
20	m	0-12 years	0.688	851.9706509	107	Grand Index
20	m	0-12 years	0.695	852	108	Grand Index

Age	Gender	Education	Percentile	Raw Score	Norm Score	Assessment
20	m	0-12 years	0.696	852.28	108	Grand Index
20	m	0-12 years	0.699	853	108	Grand Index
20	m	0-12 years	0.7	853.0283286	108	Grand Index
20	m	0-12 years	0.701	853.5023121	108	Grand Index
20	m	0-12 years	0.705	854	108	Grand Index
20	m	0-12 years	0.711	855	108	Grand Index
20	m	0-12 years	0.712	855.2433528	108	Grand Index
20	m	0-12 years	0.713	855.9964396	108	Grand Index
20	m	0-12 years	0.716	856	109	Grand Index
20	m	0-12 years	0.723	857	109	Grand Index
20	m	0-12 years	0.724	857.0093047	109	Grand Index
20	m	0-12 years	0.725	857.0549721	109	Grand Index
20	m	0-12 years	0.726	857.6482504	109	Grand Index
20	m	0-12 years	0.727	857.9881356	109	Grand Index
20	m	0-12 years	0.728	858.04	109	Grand Index
20	m	0-12 years	0.731	859	109	Grand Index
20	m	0-12 years	0.732	859.137192	109	Grand Index
20	m	0-12 years	0.733	859.9508309	109	Grand Index
20	m	0-12 years	0.734	860	109	Grand Index
20	m	0-12 years	0.735	860.3	109	Grand Index
20	m	0-12 years	0.736	861.1945783	109	Grand Index
20	m	0-12 years	0.737	861.7978263	110	Grand Index
20	m	0-12 years	0.74	862	110	Grand Index
20	m	0-12 years	0.741	862.0219769	110	Grand Index
20	m	0-12 years	0.742	862.5854469	110	Grand Index
20	m	0-12 years	0.744	863	110	Grand Index
20	m	0-12 years	0.745	863.1	110	Grand Index
20	m	0-12 years	0.746	864.28	110	Grand Index
20	m	0-12 years	0.75	865	110	Grand Index
20	m	0-12 years	0.755	866	110	Grand Index
20	m	0-12 years	0.756	866.08	110	Grand Index
20	m	0-12 years	0.761	867	111	Grand Index
20	m	0-12 years	0.762	867.0026287	111	Grand Index
20	m	0-12 years	0.763	867.3508434	111	Grand Index
20	m	0-12 years	0.766	868	111	Grand Index
20	m	0-12 years	0.767	868.012212	111	Grand Index
20	m	0-12 years	0.768	868.3946855	111	Grand Index
20	m	0-12 years	0.77	869	111	Grand Index
20	m	0-12 years	0.771	869.78	111	Grand Index
20	m	0-12 years	0.772	870	111	Grand Index
20	m	0-12 years	0.773	870.4490227	111	Grand Index
20	m	0-12 years	0.775	871	111	Grand Index
20	m	0-12 years	0.776	871.6366166	111	Grand Index
20	m	0-12 years	0.777	871.9784041	111	Grand Index
20	m	0-12 years	0.782	872	112	Grand Index
20	m	0-12 years	0.783	872.7135508	112	Grand Index
20	m	0-12 years	0.784	873	112	Grand Index
20	m	0-12 years	0.785	873.3	112	Grand Index
20	m	0-12 years	0.794	874	112	Grand Index
20	m	0-12 years	0.798	875	113	Grand Index
20	m	0-12 years	0.799	875.4680308	113	Grand Index
20	m	0-12 years	0.8	876	113	Grand Index
20	m	0-12 years	0.801	876.0928462	113	Grand Index
20	m	0-12 years	0.802	876.69012	113	Grand Index
20	m	0-12 years	0.803	877	113	Grand Index
20	m	0-12 years	0.804	877.72	113	Grand Index

Age	Gender	Education	Percentile	Raw Score	Norm Score	Assessment
20	m	0-12 years	0.807	878	113	Grand Index
20	m	0-12 years	0.808	878.44	113	Grand Index
20	m	0-12 years	0.81	879	113	Grand Index
20	m	0-12 years	0.811	879.4028512	113	Grand Index
20	m	0-12 years	0.812	879.9079908	113	Grand Index
20	m	0-12 years	0.815	880	113	Grand Index
20	m	0-12 years	0.816	880.88	114	Grand Index
20	m	0-12 years	0.819	881	114	Grand Index
20	m	0-12 years	0.82	881.6	114	Grand Index
20	m	0-12 years	0.822	882	114	Grand Index
20	m	0-12 years	0.823	883	114	Grand Index
20	m	0-12 years	0.824	883.32	114	Grand Index
20	m	0-12 years	0.827	884	114	Grand Index
20	m	0-12 years	0.828	884.04	114	Grand Index
20	m	0-12 years	0.83	885	114	Grand Index
20	m	0-12 years	0.831	885.0391411	114	Grand Index
20	m	0-12 years	0.832	885.7761963	114	Grand Index
20	m	0-12 years	0.837	886	115	Grand Index
20	m	0-12 years	0.838	886.0991234	115	Grand Index
20	m	0-12 years	0.839	886.1398693	115	Grand Index
20	m	0-12 years	0.84	886.8605059	115	Grand Index
20	m	0-12 years	0.841	887	115	Grand Index
20	m	0-12 years	0.842	887.0437675	115	Grand Index
20	m	0-12 years	0.843	887.7603206	115	Grand Index
20	m	0-12 years	0.844	888.92	115	Grand Index
20	m	0-12 years	0.845	889.0101167	115	Grand Index
20	m	0-12 years	0.846	889.3528405	115	Grand Index
20	m	0-12 years	0.849	890	115	Grand Index
20	m	0-12 years	0.85	890.0669975	116	Grand Index
20	m	0-12 years	0.851	890.9521739	116	Grand Index
20	m	0-12 years	0.854	891	116	Grand Index
20	m	0-12 years	0.855	891.9	116	Grand Index
20	m	0-12 years	0.856	892.2868639	116	Grand Index
20	m	0-12 years	0.861	893	116	Grand Index
20	m	0-12 years	0.862	894	116	Grand Index
20	m	0-12 years	0.863	894.0887524	116	Grand Index
20	m	0-12 years	0.864	894.6377467	116	Grand Index
20	m	0-12 years	0.865	894.9956438	117	Grand Index
20	m	0-12 years	0.867	895	117	Grand Index
20	m	0-12 years	0.868	895.24	117	Grand Index
20	m	0-12 years	0.87	896	117	Grand Index
20	m	0-12 years	0.871	896.536129	117	Grand Index
20	m	0-12 years	0.872	896.9874938	117	Grand Index
20	m	0-12 years	0.873	897.0137244	117	Grand Index
20	m	0-12 years	0.874	897.3866614	117	Grand Index
20	m	0-12 years	0.877	898	117	Grand Index
20	m	0-12 years	0.878	899	117	Grand Index
20	m	0-12 years	0.879	899.0125389	118	Grand Index
20	m	0-12 years	0.88	899.1909092	118	Grand Index
20	m	0-12 years	0.881	899.7445479	118	Grand Index
20	m	0-12 years	0.882	900.4985321	118	Grand Index
20	m	0-12 years	0.883	900.9793578	118	Grand Index
20	m	0-12 years	0.884	901.12	118	Grand Index
20	m	0-12 years	0.885	902.257505	118	Grand Index
20	m	0-12 years	0.886	902.9263421	118	Grand Index
20	m	0-12 years	0.89	903	118	Grand Index

Age	Gender	Education	Percentile	Raw Score	Norm Score	Assessment
20	m	0-12 years	0.891	903.3312453	118	Grand Index
20	m	0-12 years	0.892	903.9435472	119	Grand Index
20	m	0-12 years	0.894	904	119	Grand Index
20	m	0-12 years	0.895	904.4067798	119	Grand Index
20	m	0-12 years	0.896	905	119	Grand Index
20	m	0-12 years	0.897	905.46	119	Grand Index
20	m	0-12 years	0.898	906.64	119	Grand Index
20	m	0-12 years	0.901	907	119	Grand Index
20	m	0-12 years	0.902	907.36	119	Grand Index
20	m	0-12 years	0.903	908.54	119	Grand Index
20	m	0-12 years	0.905	909	120	Grand Index
20	m	0-12 years	0.906	909.1209044	120	Grand Index
20	m	0-12 years	0.907	910	120	Grand Index
20	m	0-12 years	0.908	910.0338973	120	Grand Index
20	m	0-12 years	0.909	910.6492749	120	Grand Index
20	m	0-12 years	0.911	911	120	Grand Index
20	m	0-12 years	0.912	911.16	120	Grand Index
20	m	0-12 years	0.913	912.3583926	120	Grand Index
20	m	0-12 years	0.914	913.4657898	120	Grand Index
20	m	0-12 years	0.915	913.9537445	121	Grand Index
20	m	0-12 years	0.916	914	121	Grand Index
20	m	0-12 years	0.917	915	121	Grand Index
20	m	0-12 years	0.918	915.24	121	Grand Index
20	m	0-12 years	0.921	916	121	Grand Index
20	m	0-12 years	0.922	916.5604071	121	Grand Index
20	m	0-12 years	0.923	917.0784	121	Grand Index
20	m	0-12 years	0.924	917.7008	121	Grand Index
20	m	0-12 years	0.925	918.5	122	Grand Index
20	m	0-12 years	0.926	919.68	122	Grand Index
20	m	0-12 years	0.927	920.86	122	Grand Index
20	m	0-12 years	0.928	921.0375376	122	Grand Index
20	m	0-12 years	0.929	921.9519836	122	Grand Index
20	m	0-12 years	0.93	922	122	Grand Index
20	m	0-12 years	0.931	922.056129	122	Grand Index
20	m	0-12 years	0.932	922.6944785	122	Grand Index
20	m	0-12 years	0.933	922.9929936	122	Grand Index
20	m	0-12 years	0.934	923.0979853	123	Grand Index
20	m	0-12 years	0.935	924.1715811	123	Grand Index
20	m	0-12 years	0.936	925	123	Grand Index
20	m	0-12 years	0.937	925.66	123	Grand Index
20	m	0-12 years	0.938	926	123	Grand Index
20	m	0-12 years	0.939	926.0770765	123	Grand Index
20	m	0-12 years	0.941	929	123	Grand Index
20	m	0-12 years	0.942	929.0238121	124	Grand Index
20	m	0-12 years	0.943	930.4910556	124	Grand Index
20	m	0-12 years	0.944	932.6404819	124	Grand Index
20	m	0-12 years	0.945	933	124	Grand Index
20	m	0-12 years	0.946	933.28	124	Grand Index
20	m	0-12 years	0.947	934.420802	124	Grand Index
20	m	0-12 years	0.948	934.9693233	124	Grand Index
20	m	0-12 years	0.949	935.780308	125	Grand Index
20	m	0-12 years	0.95	937	125	Grand Index
20	m	0-12 years	0.951	937.4660828	125	Grand Index
20	m	0-12 years	0.952	939.4845231	125	Grand Index
20	m	0-12 years	0.953	941.08	125	Grand Index
20	m	0-12 years	0.954	942	125	Grand Index

Age	Gender	Education	Percentile	Raw Score	Norm Score	Assessment
20	m	0-12 years	0.955	942.9	125	Grand Index
20	m	0-12 years	0.956	943.0058877	126	Grand Index
20	m	0-12 years	0.957	943.2060038	126	Grand Index
20	m	0-12 years	0.958	943.7664	126	Grand Index
20	m	0-12 years	0.959	944.62	126	Grand Index
20	m	0-12 years	0.96	945.7698925	126	Grand Index
20	m	0-12 years	0.961	945.9992473	126	Grand Index
20	m	0-12 years	0.962	949.5429384	127	Grand Index
20	m	0-12 years	0.963	951	127	Grand Index
20	m	0-12 years	0.964	951.52	127	Grand Index
20	m	0-12 years	0.965	952.0491321	127	Grand Index
20	m	0-12 years	0.966	952.8884226	127	Grand Index
20	m	0-12 years	0.967	954.0545455	128	Grand Index
20	m	0-12 years	0.968	954.9309091	128	Grand Index
20	m	0-12 years	0.969	955.0304348	128	Grand Index
20	m	0-12 years	0.97	956.8289855	128	Grand Index
20	m	0-12 years	0.971	958	128	Grand Index
20	m	0-12 years	0.972	958.508497	129	Grand Index
20	m	0-12 years	0.973	960.1892683	129	Grand Index
20	m	0-12 years	0.974	963.32	129	Grand Index
20	m	0-12 years	0.975	964.5188727	129	Grand Index
20	m	0-12 years	0.976	965.6472917	130	Grand Index
20	m	0-12 years	0.977	965.9344909	130	Grand Index
20	m	0-12 years	0.979	966	131	Grand Index
20	m	0-12 years	0.98	966.7050847	131	Grand Index
20	m	0-12 years	0.981	968.480339	131	Grand Index
20	m	0-12 years	0.982	969.76	131	Grand Index
20	m	0-12 years	0.983	971.6526797	132	Grand Index
20	m	0-12 years	0.984	972	132	Grand Index
20	m	0-12 years	0.985	972.6	133	Grand Index
20	m	0-12 years	0.986	974.3745875	133	Grand Index
20	m	0-12 years	0.987	974.9253328	133	Grand Index
20	m	0-12 years	0.988	977.5060549	134	Grand Index
20	m	0-12 years	0.989	979.0714491	134	Grand Index
20	m	0-12 years	0.99	982.6579637	135	Grand Index
20	m	0-12 years	0.992	983	136	Grand Index
20	m	0-12 years	0.993	988.92	137	Grand Index
20	m	0-12 years	0.994	995.6	138	Grand Index
20	m	0-12 years	0.995	1001.7	139	Grand Index
20	m	0-12 years	0.996	1008.049574	140	Grand Index
20	m	0-12 years	0.997	1013.68841	141	Grand Index
20	m	0-12 years	0.998	1034.136976	143	Grand Index
20	m	0-12 years	0.999	1048.56	146	Grand Index
20	f	13-16 years	0.001	590.828509	54	Grand Index
20	f	13-16 years	0.002	607.7718503	57	Grand Index
20	f	13-16 years	0.003	613.6047824	59	Grand Index
20	f	13-16 years	0.004	618.146541	60	Grand Index
20	f	13-16 years	0.005	620	61	Grand Index
20	f	13-16 years	0.006	622.3814319	62	Grand Index
20	f	13-16 years	0.007	623.983525	63	Grand Index
20	f	13-16 years	0.008	626.4262446	64	Grand Index
20	f	13-16 years	0.009	631.0602144	65	Grand Index
20	f	13-16 years	0.01	634.7585924	65	Grand Index
20	f	13-16 years	0.011	639.7137491	66	Grand Index
20	f	13-16 years	0.012	643.744608	66	Grand Index
20	f	13-16 years	0.013	648.8940059	67	Grand Index

Age	Gender	Education	Percentile	Raw Score	Norm Score	Assessment
20	f	13-16 years	0.014	651.4021913	67	Grand Index
20	f	13-16 years	0.015	652.3712814	67	Grand Index
20	f	13-16 years	0.016	653	68	Grand Index
20	f	13-16 years	0.017	655.16	68	Grand Index
20	f	13-16 years	0.018	657	69	Grand Index
20	f	13-16 years	0.019	657.9614211	69	Grand Index
20	f	13-16 years	0.02	660.0482318	69	Grand Index
20	f	13-16 years	0.021	661.9730639	69	Grand Index
20	f	13-16 years	0.022	663	70	Grand Index
20	f	13-16 years	0.023	664.0353546	70	Grand Index
20	f	13-16 years	0.024	666.16	70	Grand Index
20	f	13-16 years	0.025	667.6780132	71	Grand Index
20	f	13-16 years	0.026	669.0456598	71	Grand Index
20	f	13-16 years	0.027	669.8902594	71	Grand Index
20	f	13-16 years	0.028	670.718895	71	Grand Index
20	f	13-16 years	0.029	671.36	72	Grand Index
20	f	13-16 years	0.03	672.493196	72	Grand Index
20	f	13-16 years	0.031	675.04	72	Grand Index
20	f	13-16 years	0.032	677	72	Grand Index
20	f	13-16 years	0.033	678.3433329	72	Grand Index
20	f	13-16 years	0.034	679	73	Grand Index
20	f	13-16 years	0.035	680.4	73	Grand Index
20	f	13-16 years	0.036	682	73	Grand Index
20	f	13-16 years	0.037	683.08	73	Grand Index
20	f	13-16 years	0.038	684.4480079	73	Grand Index
20	f	13-16 years	0.039	685	74	Grand Index
20	f	13-16 years	0.04	685.9721844	74	Grand Index
20	f	13-16 years	0.041	686.1396825	74	Grand Index
20	f	13-16 years	0.042	687	74	Grand Index
20	f	13-16 years	0.043	687.2343039	74	Grand Index
20	f	13-16 years	0.044	688.4700286	74	Grand Index
20	f	13-16 years	0.045	688.9910946	75	Grand Index
20	f	13-16 years	0.046	689	75	Grand Index
20	f	13-16 years	0.047	689.2775143	75	Grand Index
20	f	13-16 years	0.048	690	75	Grand Index
20	f	13-16 years	0.049	691.16	75	Grand Index
20	f	13-16 years	0.05	692.5241801	75	Grand Index
20	f	13-16 years	0.051	694	75	Grand Index
20	f	13-16 years	0.053	695	76	Grand Index
20	f	13-16 years	0.054	695.4534239	76	Grand Index
20	f	13-16 years	0.055	696.2	76	Grand Index
20	f	13-16 years	0.056	697	76	Grand Index
20	f	13-16 years	0.057	697.5070687	76	Grand Index
20	f	13-16 years	0.058	698	76	Grand Index
20	f	13-16 years	0.059	699.0440666	77	Grand Index
20	f	13-16 years	0.06	700	77	Grand Index
20	f	13-16 years	0.061	700.8745738	77	Grand Index
20	f	13-16 years	0.062	701	77	Grand Index
20	f	13-16 years	0.063	702	77	Grand Index
20	f	13-16 years	0.065	703	77	Grand Index
20	f	13-16 years	0.066	704	77	Grand Index
20	f	13-16 years	0.067	704.8285714	78	Grand Index
20	f	13-16 years	0.07	706	78	Grand Index
20	f	13-16 years	0.071	707.64	78	Grand Index
20	f	13-16 years	0.072	708	78	Grand Index
20	f	13-16 years	0.073	708.3166942	78	Grand Index

Age	Gender	Education	Percentile	Raw Score	Norm Score	Assessment
20	f	13-16 years	0.074	709	78	Grand Index
20	f	13-16 years	0.077	710	79	Grand Index
20	f	13-16 years	0.079	711	79	Grand Index
20	f	13-16 years	0.08	711.1950604	79	Grand Index
20	f	13-16 years	0.083	712	79	Grand Index
20	f	13-16 years	0.084	712.165527	79	Grand Index
20	f	13-16 years	0.085	713	79	Grand Index
20	f	13-16 years	0.086	713.6024784	80	Grand Index
20	f	13-16 years	0.087	714	80	Grand Index
20	f	13-16 years	0.088	714.1428802	80	Grand Index
20	f	13-16 years	0.09	715	80	Grand Index
20	f	13-16 years	0.091	715.1758764	80	Grand Index
20	f	13-16 years	0.092	715.8067368	80	Grand Index
20	f	13-16 years	0.093	716.000663	80	Grand Index
20	f	13-16 years	0.094	717	80	Grand Index
20	f	13-16 years	0.095	717.2600318	80	Grand Index
20	f	13-16 years	0.097	718	81	Grand Index
20	f	13-16 years	0.098	718.014382	81	Grand Index
20	f	13-16 years	0.1	719	81	Grand Index
20	f	13-16 years	0.101	720	81	Grand Index
20	f	13-16 years	0.102	720.2029851	81	Grand Index
20	f	13-16 years	0.104	721	81	Grand Index
20	f	13-16 years	0.105	722	81	Grand Index
20	f	13-16 years	0.106	722.04	81	Grand Index
20	f	13-16 years	0.108	723	81	Grand Index
20	f	13-16 years	0.109	723.4006145	82	Grand Index
20	f	13-16 years	0.112	724	82	Grand Index
20	f	13-16 years	0.116	725	82	Grand Index
20	f	13-16 years	0.117	726.1340551	82	Grand Index
20	f	13-16 years	0.119	727	82	Grand Index
20	f	13-16 years	0.12	727.3729758	82	Grand Index
20	f	13-16 years	0.123	728	83	Grand Index
20	f	13-16 years	0.124	729	83	Grand Index
20	f	13-16 years	0.125	729.3552769	83	Grand Index
20	f	13-16 years	0.127	730	83	Grand Index
20	f	13-16 years	0.13	731	83	Grand Index
20	f	13-16 years	0.133	732	83	Grand Index
20	f	13-16 years	0.134	732.0596867	83	Grand Index
20	f	13-16 years	0.135	732.7988338	83	Grand Index
20	f	13-16 years	0.136	733.5817025	84	Grand Index
20	f	13-16 years	0.139	734	84	Grand Index
20	f	13-16 years	0.141	735	84	Grand Index
20	f	13-16 years	0.146	736	84	Grand Index
20	f	13-16 years	0.147	736.52	84	Grand Index
20	f	13-16 years	0.15	737	84	Grand Index
20	f	13-16 years	0.153	738	85	Grand Index
20	f	13-16 years	0.154	738.0851577	85	Grand Index
20	f	13-16 years	0.156	739	85	Grand Index
20	f	13-16 years	0.159	740	85	Grand Index
20	f	13-16 years	0.16	740.202791	85	Grand Index
20	f	13-16 years	0.163	741	85	Grand Index
20	f	13-16 years	0.166	742	85	Grand Index
20	f	13-16 years	0.167	742.28	86	Grand Index
20	f	13-16 years	0.17	743	86	Grand Index
20	f	13-16 years	0.171	743.0817723	86	Grand Index
20	f	13-16 years	0.174	744	86	Grand Index

Age	Gender	Education	Percentile	Raw Score	Norm Score	Assessment
20	f	13-16 years	0.177	745	86	Grand Index
20	f	13-16 years	0.181	746	86	Grand Index
20	f	13-16 years	0.185	747	87	Grand Index
20	f	13-16 years	0.186	747.4309704	87	Grand Index
20	f	13-16 years	0.189	748	87	Grand Index
20	f	13-16 years	0.194	749	87	Grand Index
20	f	13-16 years	0.195	749.7620234	87	Grand Index
20	f	13-16 years	0.198	750	87	Grand Index
20	f	13-16 years	0.199	750.7275676	87	Grand Index
20	f	13-16 years	0.202	751	87	Grand Index
20	f	13-16 years	0.203	751.0200735	88	Grand Index
20	f	13-16 years	0.204	751.1660228	88	Grand Index
20	f	13-16 years	0.205	751.5956583	88	Grand Index
20	f	13-16 years	0.209	752	88	Grand Index
20	f	13-16 years	0.212	753	88	Grand Index
20	f	13-16 years	0.213	753.0129613	88	Grand Index
20	f	13-16 years	0.214	753.9807143	88	Grand Index
20	f	13-16 years	0.217	754	88	Grand Index
20	f	13-16 years	0.221	755	88	Grand Index
20	f	13-16 years	0.222	755.3770596	89	Grand Index
20	f	13-16 years	0.225	756	89	Grand Index
20	f	13-16 years	0.229	757	89	Grand Index
20	f	13-16 years	0.23	757.6635843	89	Grand Index
20	f	13-16 years	0.233	758	89	Grand Index
20	f	13-16 years	0.234	758.614433	89	Grand Index
20	f	13-16 years	0.235	759	89	Grand Index
20	f	13-16 years	0.236	759.0249039	89	Grand Index
20	f	13-16 years	0.24	760	89	Grand Index
20	f	13-16 years	0.244	761	90	Grand Index
20	f	13-16 years	0.245	761.9888039	90	Grand Index
20	f	13-16 years	0.247	762	90	Grand Index
20	f	13-16 years	0.248	762.32	90	Grand Index
20	f	13-16 years	0.251	763	90	Grand Index
20	f	13-16 years	0.252	763.8725091	90	Grand Index
20	f	13-16 years	0.254	764	90	Grand Index
20	f	13-16 years	0.255	764.0247788	90	Grand Index
20	f	13-16 years	0.256	764.937131	90	Grand Index
20	f	13-16 years	0.26	765	90	Grand Index
20	f	13-16 years	0.265	766	91	Grand Index
20	f	13-16 years	0.266	766.4763116	91	Grand Index
20	f	13-16 years	0.268	767	91	Grand Index
20	f	13-16 years	0.269	767.96	91	Grand Index
20	f	13-16 years	0.271	768	91	Grand Index
20	f	13-16 years	0.272	768.3226554	91	Grand Index
20	f	13-16 years	0.273	768.9537415	91	Grand Index
20	f	13-16 years	0.276	769	91	Grand Index
20	f	13-16 years	0.277	769.6501369	91	Grand Index
20	f	13-16 years	0.278	769.9717746	91	Grand Index
20	f	13-16 years	0.283	770	91	Grand Index
20	f	13-16 years	0.284	770.3255385	91	Grand Index
20	f	13-16 years	0.286	771	92	Grand Index
20	f	13-16 years	0.287	771.08	92	Grand Index
20	f	13-16 years	0.291	772	92	Grand Index
20	f	13-16 years	0.292	772.107451	92	Grand Index
20	f	13-16 years	0.296	773	92	Grand Index
20	f	13-16 years	0.297	773.0863309	92	Grand Index

Age	Gender	Education	Percentile	Raw Score	Norm Score	Assessment
20	f	13-16 years	0.298	773.8545829	92	Grand Index
20	f	13-16 years	0.302	774	92	Grand Index
20	f	13-16 years	0.308	775	92	Grand Index
20	f	13-16 years	0.309	775.1675528	93	Grand Index
20	f	13-16 years	0.311	776	93	Grand Index
20	f	13-16 years	0.312	776.0184615	93	Grand Index
20	f	13-16 years	0.318	777	93	Grand Index
20	f	13-16 years	0.323	778	93	Grand Index
20	f	13-16 years	0.324	778.8239052	93	Grand Index
20	f	13-16 years	0.329	779	93	Grand Index
20	f	13-16 years	0.33	779.3994125	93	Grand Index
20	f	13-16 years	0.334	780	94	Grand Index
20	f	13-16 years	0.335	780.0984421	94	Grand Index
20	f	13-16 years	0.34	781	94	Grand Index
20	f	13-16 years	0.345	782	94	Grand Index
20	f	13-16 years	0.346	782.64	94	Grand Index
20	f	13-16 years	0.35	783	94	Grand Index
20	f	13-16 years	0.351	783.3754967	94	Grand Index
20	f	13-16 years	0.355	784	94	Grand Index
20	f	13-16 years	0.356	784.0100465	94	Grand Index
20	f	13-16 years	0.357	784.9629236	95	Grand Index
20	f	13-16 years	0.361	785	95	Grand Index
20	f	13-16 years	0.366	786	95	Grand Index
20	f	13-16 years	0.367	786.8894232	95	Grand Index
20	f	13-16 years	0.372	787	95	Grand Index
20	f	13-16 years	0.377	788	95	Grand Index
20	f	13-16 years	0.378	788.0479785	95	Grand Index
20	f	13-16 years	0.379	788.9945299	95	Grand Index
20	f	13-16 years	0.384	789	96	Grand Index
20	f	13-16 years	0.385	789.029106	96	Grand Index
20	f	13-16 years	0.387	790	96	Grand Index
20	f	13-16 years	0.388	790.3237537	96	Grand Index
20	f	13-16 years	0.392	791	96	Grand Index
20	f	13-16 years	0.393	791.0072414	96	Grand Index
20	f	13-16 years	0.394	791.8221185	96	Grand Index
20	f	13-16 years	0.398	792	96	Grand Index
20	f	13-16 years	0.402	793	96	Grand Index
20	f	13-16 years	0.403	793.1054197	96	Grand Index
20	f	13-16 years	0.405	794	96	Grand Index
20	f	13-16 years	0.406	794.0237714	96	Grand Index
20	f	13-16 years	0.411	795	97	Grand Index
20	f	13-16 years	0.412	795.7475812	97	Grand Index
20	f	13-16 years	0.415	796	97	Grand Index
20	f	13-16 years	0.416	796.3798229	97	Grand Index
20	f	13-16 years	0.419	797	97	Grand Index
20	f	13-16 years	0.42	797.800722	97	Grand Index
20	f	13-16 years	0.422	798	97	Grand Index
20	f	13-16 years	0.423	798.32	97	Grand Index
20	f	13-16 years	0.426	799	97	Grand Index
20	f	13-16 years	0.428	800	97	Grand Index
20	f	13-16 years	0.429	800.1770841	97	Grand Index
20	f	13-16 years	0.43	800.7191372	97	Grand Index
20	f	13-16 years	0.435	801	98	Grand Index
20	f	13-16 years	0.436	801.136063	98	Grand Index
20	f	13-16 years	0.441	802	98	Grand Index
20	f	13-16 years	0.442	802.9868197	98	Grand Index

Age	Gender	Education	Percentile	Raw Score	Norm Score	Assessment
20	f	13-16 years	0.447	803	98	Grand Index
20	f	13-16 years	0.453	804	98	Grand Index
20	f	13-16 years	0.454	804.01662	98	Grand Index
20	f	13-16 years	0.455	804.9343885	98	Grand Index
20	f	13-16 years	0.457	805	98	Grand Index
20	f	13-16 years	0.458	805.9357087	98	Grand Index
20	f	13-16 years	0.464	806	99	Grand Index
20	f	13-16 years	0.465	806.9111111	99	Grand Index
20	f	13-16 years	0.471	807	99	Grand Index
20	f	13-16 years	0.475	808	99	Grand Index
20	f	13-16 years	0.476	808.4532374	99	Grand Index
20	f	13-16 years	0.48	809	99	Grand Index
20	f	13-16 years	0.486	810	99	Grand Index
20	f	13-16 years	0.487	810.7627876	100	Grand Index
20	f	13-16 years	0.491	811	100	Grand Index
20	f	13-16 years	0.492	811.5855916	100	Grand Index
20	f	13-16 years	0.497	812	100	Grand Index
20	f	13-16 years	0.498	812.5577695	100	Grand Index
20	f	13-16 years	0.502	813	100	Grand Index
20	f	13-16 years	0.503	813.0027936	100	Grand Index
20	f	13-16 years	0.504	813.1740819	100	Grand Index
20	f	13-16 years	0.508	814	100	Grand Index
20	f	13-16 years	0.514	815	101	Grand Index
20	f	13-16 years	0.515	815.8165605	101	Grand Index
20	f	13-16 years	0.518	816	101	Grand Index
20	f	13-16 years	0.519	816.9089791	101	Grand Index
20	f	13-16 years	0.526	817	101	Grand Index
20	f	13-16 years	0.527	817.88	101	Grand Index
20	f	13-16 years	0.533	818	101	Grand Index
20	f	13-16 years	0.534	818.4590484	101	Grand Index
20	f	13-16 years	0.539	819	101	Grand Index
20	f	13-16 years	0.54	819.8455266	102	Grand Index
20	f	13-16 years	0.544	820	102	Grand Index
20	f	13-16 years	0.545	820.372093	102	Grand Index
20	f	13-16 years	0.546	820.7993273	102	Grand Index
20	f	13-16 years	0.55	821	102	Grand Index
20	f	13-16 years	0.557	822	102	Grand Index
20	f	13-16 years	0.56	823	102	Grand Index
20	f	13-16 years	0.561	823.2848636	102	Grand Index
20	f	13-16 years	0.569	824	103	Grand Index
20	f	13-16 years	0.576	825	103	Grand Index
20	f	13-16 years	0.577	825.0499182	103	Grand Index
20	f	13-16 years	0.578	825.8698305	103	Grand Index
20	f	13-16 years	0.583	826	103	Grand Index
20	f	13-16 years	0.589	827	103	Grand Index
20	f	13-16 years	0.59	827.6394	103	Grand Index
20	f	13-16 years	0.593	828	104	Grand Index
20	f	13-16 years	0.594	828.1556757	104	Grand Index
20	f	13-16 years	0.599	829	104	Grand Index
20	f	13-16 years	0.6	829.2466611	104	Grand Index
20	f	13-16 years	0.603	830	104	Grand Index
20	f	13-16 years	0.604	830.7851748	104	Grand Index
20	f	13-16 years	0.608	831	104	Grand Index
20	f	13-16 years	0.611	832	104	Grand Index
20	f	13-16 years	0.612	832.0585471	104	Grand Index
20	f	13-16 years	0.615	833	104	Grand Index

Age	Gender	Education	Percentile	Raw Score	Norm Score	Assessment
20	f	13-16 years	0.616	833.7181675	104	Grand Index
20	f	13-16 years	0.622	834	105	Grand Index
20	f	13-16 years	0.623	834.128	105	Grand Index
20	f	13-16 years	0.627	835	105	Grand Index
20	f	13-16 years	0.628	835.1210811	105	Grand Index
20	f	13-16 years	0.633	836	105	Grand Index
20	f	13-16 years	0.634	836.5521039	105	Grand Index
20	f	13-16 years	0.637	837	105	Grand Index
20	f	13-16 years	0.638	837.92	105	Grand Index
20	f	13-16 years	0.64	838	105	Grand Index
20	f	13-16 years	0.641	838.2812519	105	Grand Index
20	f	13-16 years	0.642	838.9327299	105	Grand Index
20	f	13-16 years	0.646	839	106	Grand Index
20	f	13-16 years	0.647	839.5358192	106	Grand Index
20	f	13-16 years	0.651	840	106	Grand Index
20	f	13-16 years	0.655	841	106	Grand Index
20	f	13-16 years	0.656	841.0305394	106	Grand Index
20	f	13-16 years	0.657	841.9778462	106	Grand Index
20	f	13-16 years	0.661	842	106	Grand Index
20	f	13-16 years	0.662	842.067027	106	Grand Index
20	f	13-16 years	0.663	842.8933203	106	Grand Index
20	f	13-16 years	0.669	843	107	Grand Index
20	f	13-16 years	0.67	843.9014409	107	Grand Index
20	f	13-16 years	0.674	844	107	Grand Index
20	f	13-16 years	0.675	844.2753304	107	Grand Index
20	f	13-16 years	0.676	844.917404	107	Grand Index
20	f	13-16 years	0.68	845	107	Grand Index
20	f	13-16 years	0.681	845.4144	107	Grand Index
20	f	13-16 years	0.682	845.9913708	107	Grand Index
20	f	13-16 years	0.686	846	107	Grand Index
20	f	13-16 years	0.687	846.5603922	107	Grand Index
20	f	13-16 years	0.69	847	107	Grand Index
20	f	13-16 years	0.691	847.9631579	107	Grand Index
20	f	13-16 years	0.692	848	108	Grand Index
20	f	13-16 years	0.693	848.0887879	108	Grand Index
20	f	13-16 years	0.696	849	108	Grand Index
20	f	13-16 years	0.697	849.0063717	108	Grand Index
20	f	13-16 years	0.698	849.8713568	108	Grand Index
20	f	13-16 years	0.701	850	108	Grand Index
20	f	13-16 years	0.702	850.9076923	108	Grand Index
20	f	13-16 years	0.704	851	108	Grand Index
20	f	13-16 years	0.705	851.5453744	108	Grand Index
20	f	13-16 years	0.707	852	108	Grand Index
20	f	13-16 years	0.71	853	108	Grand Index
20	f	13-16 years	0.711	853.2444393	108	Grand Index
20	f	13-16 years	0.713	854	108	Grand Index
20	f	13-16 years	0.714	854.7151927	108	Grand Index
20	f	13-16 years	0.716	855	109	Grand Index
20	f	13-16 years	0.717	855.28	109	Grand Index
20	f	13-16 years	0.72	856	109	Grand Index
20	f	13-16 years	0.721	856.64	109	Grand Index
20	f	13-16 years	0.724	857	109	Grand Index
20	f	13-16 years	0.725	857.1354167	109	Grand Index
20	f	13-16 years	0.728	858	109	Grand Index
20	f	13-16 years	0.732	859	109	Grand Index
20	f	13-16 years	0.733	859.2801053	109	Grand Index

Age	Gender	Education	Percentile	Raw Score	Norm Score	Assessment
20	f	13-16 years	0.736	860	109	Grand Index
20	f	13-16 years	0.737	860.0287179	110	Grand Index
20	f	13-16 years	0.738	860.6982999	110	Grand Index
20	f	13-16 years	0.74	861	110	Grand Index
20	f	13-16 years	0.741	861.8364964	110	Grand Index
20	f	13-16 years	0.743	862	110	Grand Index
20	f	13-16 years	0.744	862.4094323	110	Grand Index
20	f	13-16 years	0.745	862.9554099	110	Grand Index
20	f	13-16 years	0.748	863	110	Grand Index
20	f	13-16 years	0.749	863.2829268	110	Grand Index
20	f	13-16 years	0.751	864	110	Grand Index
20	f	13-16 years	0.752	864.1530684	110	Grand Index
20	f	13-16 years	0.756	865	110	Grand Index
20	f	13-16 years	0.757	865.9348259	110	Grand Index
20	f	13-16 years	0.76	866	111	Grand Index
20	f	13-16 years	0.761	866.4498965	111	Grand Index
20	f	13-16 years	0.765	867	111	Grand Index
20	f	13-16 years	0.766	867.0874172	111	Grand Index
20	f	13-16 years	0.769	868	111	Grand Index
20	f	13-16 years	0.77	868.1806452	111	Grand Index
20	f	13-16 years	0.774	869	111	Grand Index
20	f	13-16 years	0.775	869.9542484	111	Grand Index
20	f	13-16 years	0.78	870	112	Grand Index
20	f	13-16 years	0.784	871	112	Grand Index
20	f	13-16 years	0.785	872	112	Grand Index
20	f	13-16 years	0.786	872.321626	112	Grand Index
20	f	13-16 years	0.787	872.9687237	112	Grand Index
20	f	13-16 years	0.791	873	112	Grand Index
20	f	13-16 years	0.792	873.2951595	112	Grand Index
20	f	13-16 years	0.795	874	112	Grand Index
20	f	13-16 years	0.796	874.8360279	112	Grand Index
20	f	13-16 years	0.8	875	113	Grand Index
20	f	13-16 years	0.801	875.9893333	113	Grand Index
20	f	13-16 years	0.804	876	113	Grand Index
20	f	13-16 years	0.807	877	113	Grand Index
20	f	13-16 years	0.808	877.4270827	113	Grand Index
20	f	13-16 years	0.809	877.9308571	113	Grand Index
20	f	13-16 years	0.813	878	113	Grand Index
20	f	13-16 years	0.814	878.6142319	113	Grand Index
20	f	13-16 years	0.815	879	113	Grand Index
20	f	13-16 years	0.816	879.3484082	114	Grand Index
20	f	13-16 years	0.818	880	114	Grand Index
20	f	13-16 years	0.819	880.0635019	114	Grand Index
20	f	13-16 years	0.821	881	114	Grand Index
20	f	13-16 years	0.824	882	114	Grand Index
20	f	13-16 years	0.825	882.0889831	114	Grand Index
20	f	13-16 years	0.828	883	114	Grand Index
20	f	13-16 years	0.829	883.43887	114	Grand Index
20	f	13-16 years	0.833	884	114	Grand Index
20	f	13-16 years	0.836	885	115	Grand Index
20	f	13-16 years	0.837	885.2926991	115	Grand Index
20	f	13-16 years	0.84	886	115	Grand Index
20	f	13-16 years	0.844	887	115	Grand Index
20	f	13-16 years	0.845	888	115	Grand Index
20	f	13-16 years	0.846	888.7556202	115	Grand Index
20	f	13-16 years	0.848	889	115	Grand Index

Age	Gender	Education	Percentile	Raw Score	Norm Score	Assessment
20	f	13-16 years	0.85	890	116	Grand Index
20	f	13-16 years	0.851	890.9920879	116	Grand Index
20	f	13-16 years	0.854	891	116	Grand Index
20	f	13-16 years	0.857	892	116	Grand Index
20	f	13-16 years	0.858	892.7653984	116	Grand Index
20	f	13-16 years	0.86	893	116	Grand Index
20	f	13-16 years	0.862	894	116	Grand Index
20	f	13-16 years	0.863	894.9523511	116	Grand Index
20	f	13-16 years	0.865	895	117	Grand Index
20	f	13-16 years	0.866	895.1733783	117	Grand Index
20	f	13-16 years	0.868	896	117	Grand Index
20	f	13-16 years	0.869	896.5197452	117	Grand Index
20	f	13-16 years	0.872	897	117	Grand Index
20	f	13-16 years	0.873	897.0087579	117	Grand Index
20	f	13-16 years	0.876	898	117	Grand Index
20	f	13-16 years	0.877	898.032381	117	Grand Index
20	f	13-16 years	0.88	899	118	Grand Index
20	f	13-16 years	0.883	900	118	Grand Index
20	f	13-16 years	0.884	900.974802	118	Grand Index
20	f	13-16 years	0.886	901	118	Grand Index
20	f	13-16 years	0.887	901.2770966	118	Grand Index
20	f	13-16 years	0.889	902	118	Grand Index
20	f	13-16 years	0.89	902.5498958	118	Grand Index
20	f	13-16 years	0.892	903	119	Grand Index
20	f	13-16 years	0.893	903.0589178	119	Grand Index
20	f	13-16 years	0.894	903.9975742	119	Grand Index
20	f	13-16 years	0.895	904	119	Grand Index
20	f	13-16 years	0.896	904.7489231	119	Grand Index
20	f	13-16 years	0.898	905	119	Grand Index
20	f	13-16 years	0.901	906	119	Grand Index
20	f	13-16 years	0.902	906.796196	119	Grand Index
20	f	13-16 years	0.903	907	119	Grand Index
20	f	13-16 years	0.904	907.3930787	120	Grand Index
20	f	13-16 years	0.905	908	120	Grand Index
20	f	13-16 years	0.907	909	120	Grand Index
20	f	13-16 years	0.908	910.9994411	120	Grand Index
20	f	13-16 years	0.91	911	120	Grand Index
20	f	13-16 years	0.911	912.9648	120	Grand Index
20	f	13-16 years	0.912	913.08	120	Grand Index
20	f	13-16 years	0.914	914	120	Grand Index
20	f	13-16 years	0.915	914.7286661	121	Grand Index
20	f	13-16 years	0.918	915	121	Grand Index
20	f	13-16 years	0.919	915.9824212	121	Grand Index
20	f	13-16 years	0.921	916	121	Grand Index
20	f	13-16 years	0.922	917	121	Grand Index
20	f	13-16 years	0.923	917.32	121	Grand Index
20	f	13-16 years	0.924	918.133181	121	Grand Index
20	f	13-16 years	0.925	919	122	Grand Index
20	f	13-16 years	0.926	919.661502	122	Grand Index
20	f	13-16 years	0.927	920	122	Grand Index
20	f	13-16 years	0.928	920.0259318	122	Grand Index
20	f	13-16 years	0.93	921	122	Grand Index
20	f	13-16 years	0.931	921.0069421	122	Grand Index
20	f	13-16 years	0.932	922	122	Grand Index
20	f	13-16 years	0.933	922.5738978	122	Grand Index
20	f	13-16 years	0.934	923	123	Grand Index

Age	Gender	Education	Percentile	Raw Score	Norm Score	Assessment
20	f	13-16 years	0.935	924	123	Grand Index
20	f	13-16 years	0.936	925	123	Grand Index
20	f	13-16 years	0.937	925.0623246	123	Grand Index
20	f	13-16 years	0.938	925.9946614	123	Grand Index
20	f	13-16 years	0.94	926	123	Grand Index
20	f	13-16 years	0.941	927.3331792	123	Grand Index
20	f	13-16 years	0.942	928	124	Grand Index
20	f	13-16 years	0.943	928.0656371	124	Grand Index
20	f	13-16 years	0.944	929	124	Grand Index
20	f	13-16 years	0.945	929.7940138	124	Grand Index
20	f	13-16 years	0.946	930	124	Grand Index
20	f	13-16 years	0.947	932.0531488	124	Grand Index
20	f	13-16 years	0.948	933	124	Grand Index
20	f	13-16 years	0.949	934.2482261	125	Grand Index
20	f	13-16 years	0.95	935	125	Grand Index
20	f	13-16 years	0.951	936	125	Grand Index
20	f	13-16 years	0.952	937	125	Grand Index
20	f	13-16 years	0.953	938	125	Grand Index
20	f	13-16 years	0.954	939.1517355	125	Grand Index
20	f	13-16 years	0.955	940.1020408	125	Grand Index
20	f	13-16 years	0.956	941.0667865	126	Grand Index
20	f	13-16 years	0.957	943	126	Grand Index
20	f	13-16 years	0.958	944	126	Grand Index
20	f	13-16 years	0.959	945.5113826	126	Grand Index
20	f	13-16 years	0.96	946	126	Grand Index
20	f	13-16 years	0.961	946.8556496	126	Grand Index
20	f	13-16 years	0.962	947	127	Grand Index
20	f	13-16 years	0.963	948.9283582	127	Grand Index
20	f	13-16 years	0.964	950.17134	127	Grand Index
20	f	13-16 years	0.965	951	127	Grand Index
20	f	13-16 years	0.966	951.44	127	Grand Index
20	f	13-16 years	0.967	952.0298012	128	Grand Index
20	f	13-16 years	0.969	953	128	Grand Index
20	f	13-16 years	0.97	954.0862921	128	Grand Index
20	f	13-16 years	0.971	955	128	Grand Index
20	f	13-16 years	0.972	956	129	Grand Index
20	f	13-16 years	0.973	957.4772123	129	Grand Index
20	f	13-16 years	0.974	959	129	Grand Index
20	f	13-16 years	0.975	960	129	Grand Index
20	f	13-16 years	0.976	961.84	130	Grand Index
20	f	13-16 years	0.977	962.5712471	130	Grand Index
20	f	13-16 years	0.978	963.0713473	130	Grand Index
20	f	13-16 years	0.979	963.9552726	131	Grand Index
20	f	13-16 years	0.98	964.1152716	131	Grand Index
20	f	13-16 years	0.981	966.0019048	131	Grand Index
20	f	13-16 years	0.982	967.7927273	131	Grand Index
20	f	13-16 years	0.983	968.72	132	Grand Index
20	f	13-16 years	0.984	970.56	132	Grand Index
20	f	13-16 years	0.985	971.3773585	133	Grand Index
20	f	13-16 years	0.986	973.00178	133	Grand Index
20	f	13-16 years	0.987	976.0132	133	Grand Index
20	f	13-16 years	0.988	977.2529231	134	Grand Index
20	f	13-16 years	0.989	978.836169	134	Grand Index
20	f	13-16 years	0.99	981.6	135	Grand Index
20	f	13-16 years	0.991	984.0727148	135	Grand Index
20	f	13-16 years	0.992	986.3072826	136	Grand Index

Age	Gender	Education	Percentile	Raw Score	Norm Score	Assessment
20	f	13-16 years	0.993	992	137	Grand Index
20	f	13-16 years	0.994	993.7950183	138	Grand Index
20	f	13-16 years	0.995	998.9522138	139	Grand Index
20	f	13-16 years	0.996	1002.218141	140	Grand Index
20	f	13-16 years	0.997	1011.47767	141	Grand Index
20	f	13-16 years	0.998	1013.131765	143	Grand Index
20	f	13-16 years	0.999	1024.326866	146	Grand Index
20	m	13-16 years	0.001	571.805	54	Grand Index
20	m	13-16 years	0.002	596.7017275	57	Grand Index
20	m	13-16 years	0.003	603.5191261	59	Grand Index
20	m	13-16 years	0.004	610.7888648	60	Grand Index
20	m	13-16 years	0.005	617.3533659	61	Grand Index
20	m	13-16 years	0.006	620	62	Grand Index
20	m	13-16 years	0.007	621.581591	63	Grand Index
20	m	13-16 years	0.008	630.76	64	Grand Index
20	m	13-16 years	0.009	634.035	65	Grand Index
20	m	13-16 years	0.01	636.7442164	65	Grand Index
20	m	13-16 years	0.011	640.265	66	Grand Index
20	m	13-16 years	0.012	642	66	Grand Index
20	m	13-16 years	0.013	643.3919483	67	Grand Index
20	m	13-16 years	0.014	645.7814259	67	Grand Index
20	m	13-16 years	0.015	647.7486247	67	Grand Index
20	m	13-16 years	0.016	650.68	68	Grand Index
20	m	13-16 years	0.017	652.1274731	68	Grand Index
20	m	13-16 years	0.018	654.009199	69	Grand Index
20	m	13-16 years	0.019	656	69	Grand Index
20	m	13-16 years	0.02	658.2297021	69	Grand Index
20	m	13-16 years	0.021	659.415	69	Grand Index
20	m	13-16 years	0.022	661	70	Grand Index
20	m	13-16 years	0.023	663.6187835	70	Grand Index
20	m	13-16 years	0.024	664.2732793	70	Grand Index
20	m	13-16 years	0.025	665	71	Grand Index
20	m	13-16 years	0.026	665.99	71	Grand Index
20	m	13-16 years	0.027	667	71	Grand Index
20	m	13-16 years	0.028	668.1214408	71	Grand Index
20	m	13-16 years	0.029	669	72	Grand Index
20	m	13-16 years	0.03	670.1006755	72	Grand Index
20	m	13-16 years	0.031	672.565	72	Grand Index
20	m	13-16 years	0.032	674	72	Grand Index
20	m	13-16 years	0.034	675	73	Grand Index
20	m	13-16 years	0.035	676.1267104	73	Grand Index
20	m	13-16 years	0.036	677	73	Grand Index
20	m	13-16 years	0.037	677.255	73	Grand Index
20	m	13-16 years	0.038	679	73	Grand Index
20	m	13-16 years	0.039	680	74	Grand Index
20	m	13-16 years	0.04	681	74	Grand Index
20	m	13-16 years	0.041	682.6831263	74	Grand Index
20	m	13-16 years	0.042	684	74	Grand Index
20	m	13-16 years	0.043	684.945	74	Grand Index
20	m	13-16 years	0.045	686	75	Grand Index
20	m	13-16 years	0.046	686.645	75	Grand Index
20	m	13-16 years	0.047	687.405	75	Grand Index
20	m	13-16 years	0.048	688	75	Grand Index
20	m	13-16 years	0.05	689	75	Grand Index
20	m	13-16 years	0.051	690	75	Grand Index
20	m	13-16 years	0.052	690.7011681	76	Grand Index

Age	Gender	Education	Percentile	Raw Score	Norm Score	Assessment
20	m	13-16 years	0.053	691	76	Grand Index
20	m	13-16 years	0.054	691.7954251	76	Grand Index
20	m	13-16 years	0.056	692	76	Grand Index
20	m	13-16 years	0.057	693	76	Grand Index
20	m	13-16 years	0.058	693.2375666	76	Grand Index
20	m	13-16 years	0.059	694	77	Grand Index
20	m	13-16 years	0.061	695	77	Grand Index
20	m	13-16 years	0.062	696	77	Grand Index
20	m	13-16 years	0.063	696.7827266	77	Grand Index
20	m	13-16 years	0.064	697	77	Grand Index
20	m	13-16 years	0.065	697.1821471	77	Grand Index
20	m	13-16 years	0.066	697.9857824	77	Grand Index
20	m	13-16 years	0.067	698	78	Grand Index
20	m	13-16 years	0.068	699	78	Grand Index
20	m	13-16 years	0.069	699.1299323	78	Grand Index
20	m	13-16 years	0.07	700	78	Grand Index
20	m	13-16 years	0.072	701	78	Grand Index
20	m	13-16 years	0.073	701.6144339	78	Grand Index
20	m	13-16 years	0.074	702	78	Grand Index
20	m	13-16 years	0.075	703	78	Grand Index
20	m	13-16 years	0.076	703.74	79	Grand Index
20	m	13-16 years	0.077	704.5399054	79	Grand Index
20	m	13-16 years	0.078	705.56874	79	Grand Index
20	m	13-16 years	0.08	706	79	Grand Index
20	m	13-16 years	0.081	706.9947383	79	Grand Index
20	m	13-16 years	0.082	707	79	Grand Index
20	m	13-16 years	0.083	708	79	Grand Index
20	m	13-16 years	0.084	708.7342971	79	Grand Index
20	m	13-16 years	0.086	709	80	Grand Index
20	m	13-16 years	0.087	710	80	Grand Index
20	m	13-16 years	0.088	710.0010745	80	Grand Index
20	m	13-16 years	0.089	710.5015037	80	Grand Index
20	m	13-16 years	0.091	711	80	Grand Index
20	m	13-16 years	0.092	712	80	Grand Index
20	m	13-16 years	0.093	712.6613558	80	Grand Index
20	m	13-16 years	0.094	713.4569704	80	Grand Index
20	m	13-16 years	0.096	714	80	Grand Index
20	m	13-16 years	0.098	715	81	Grand Index
20	m	13-16 years	0.099	716	81	Grand Index
20	m	13-16 years	0.1	716.9422665	81	Grand Index
20	m	13-16 years	0.104	717	81	Grand Index
20	m	13-16 years	0.105	717.075	81	Grand Index
20	m	13-16 years	0.106	718.0194574	81	Grand Index
20	m	13-16 years	0.108	719	81	Grand Index
20	m	13-16 years	0.109	719.3821182	82	Grand Index
20	m	13-16 years	0.111	720	82	Grand Index
20	m	13-16 years	0.112	720.8555161	82	Grand Index
20	m	13-16 years	0.113	721	82	Grand Index
20	m	13-16 years	0.116	722	82	Grand Index
20	m	13-16 years	0.117	722.4511084	82	Grand Index
20	m	13-16 years	0.119	723	82	Grand Index
20	m	13-16 years	0.12	723.9669089	82	Grand Index
20	m	13-16 years	0.122	724	83	Grand Index
20	m	13-16 years	0.123	724.0840952	83	Grand Index
20	m	13-16 years	0.125	725	83	Grand Index
20	m	13-16 years	0.126	725.0439213	83	Grand Index

Age	Gender	Education	Percentile	Raw Score	Norm Score	Assessment
20	m	13-16 years	0.127	725.9740759	83	Grand Index
20	m	13-16 years	0.13	726	83	Grand Index
20	m	13-16 years	0.133	727	83	Grand Index
20	m	13-16 years	0.137	728	84	Grand Index
20	m	13-16 years	0.139	729	84	Grand Index
20	m	13-16 years	0.14	729.1386419	84	Grand Index
20	m	13-16 years	0.143	730	84	Grand Index
20	m	13-16 years	0.145	731	84	Grand Index
20	m	13-16 years	0.146	731.8327744	84	Grand Index
20	m	13-16 years	0.147	732	84	Grand Index
20	m	13-16 years	0.148	732.7346439	84	Grand Index
20	m	13-16 years	0.15	733	84	Grand Index
20	m	13-16 years	0.151	733.8529718	85	Grand Index
20	m	13-16 years	0.152	734	85	Grand Index
20	m	13-16 years	0.153	734.251455	85	Grand Index
20	m	13-16 years	0.155	735	85	Grand Index
20	m	13-16 years	0.156	735.6181736	85	Grand Index
20	m	13-16 years	0.159	736	85	Grand Index
20	m	13-16 years	0.16	736.050495	85	Grand Index
20	m	13-16 years	0.162	737	85	Grand Index
20	m	13-16 years	0.163	737.8444028	85	Grand Index
20	m	13-16 years	0.166	738	85	Grand Index
20	m	13-16 years	0.169	739	86	Grand Index
20	m	13-16 years	0.17	739.4348087	86	Grand Index
20	m	13-16 years	0.175	740	86	Grand Index
20	m	13-16 years	0.178	741	86	Grand Index
20	m	13-16 years	0.179	741.148871	86	Grand Index
20	m	13-16 years	0.181	742	86	Grand Index
20	m	13-16 years	0.182	742.4770842	86	Grand Index
20	m	13-16 years	0.185	743	87	Grand Index
20	m	13-16 years	0.186	743.387104	87	Grand Index
20	m	13-16 years	0.19	744	87	Grand Index
20	m	13-16 years	0.191	744.7440862	87	Grand Index
20	m	13-16 years	0.194	745	87	Grand Index
20	m	13-16 years	0.195	745.8040049	87	Grand Index
20	m	13-16 years	0.198	746	87	Grand Index
20	m	13-16 years	0.2	747	87	Grand Index
20	m	13-16 years	0.201	747.1321101	87	Grand Index
20	m	13-16 years	0.205	748	88	Grand Index
20	m	13-16 years	0.206	748.3939421	88	Grand Index
20	m	13-16 years	0.21	749	88	Grand Index
20	m	13-16 years	0.211	749.5574855	88	Grand Index
20	m	13-16 years	0.213	750	88	Grand Index
20	m	13-16 years	0.214	750.0919691	88	Grand Index
20	m	13-16 years	0.215	750.9369105	88	Grand Index
20	m	13-16 years	0.216	751	88	Grand Index
20	m	13-16 years	0.217	751.3894532	88	Grand Index
20	m	13-16 years	0.221	752	88	Grand Index
20	m	13-16 years	0.222	752.9513807	89	Grand Index
20	m	13-16 years	0.225	753	89	Grand Index
20	m	13-16 years	0.226	753.4641418	89	Grand Index
20	m	13-16 years	0.23	754	89	Grand Index
20	m	13-16 years	0.231	754.9984801	89	Grand Index
20	m	13-16 years	0.233	755	89	Grand Index
20	m	13-16 years	0.234	755.9222167	89	Grand Index
20	m	13-16 years	0.235	756	89	Grand Index

Age	Gender	Education	Percentile	Raw Score	Norm Score	Assessment
20	m	13-16 years	0.236	756.0539187	89	Grand Index
20	m	13-16 years	0.239	757	89	Grand Index
20	m	13-16 years	0.243	758	90	Grand Index
20	m	13-16 years	0.247	759	90	Grand Index
20	m	13-16 years	0.248	759.2662116	90	Grand Index
20	m	13-16 years	0.253	760	90	Grand Index
20	m	13-16 years	0.254	760.3988427	90	Grand Index
20	m	13-16 years	0.256	761	90	Grand Index
20	m	13-16 years	0.257	761.425674	90	Grand Index
20	m	13-16 years	0.26	762	90	Grand Index
20	m	13-16 years	0.261	762.1750702	90	Grand Index
20	m	13-16 years	0.265	763	91	Grand Index
20	m	13-16 years	0.266	763.7641736	91	Grand Index
20	m	13-16 years	0.269	764	91	Grand Index
20	m	13-16 years	0.272	765	91	Grand Index
20	m	13-16 years	0.273	765.4533874	91	Grand Index
20	m	13-16 years	0.277	766	91	Grand Index
20	m	13-16 years	0.28	767	91	Grand Index
20	m	13-16 years	0.281	767.8339697	91	Grand Index
20	m	13-16 years	0.283	768	91	Grand Index
20	m	13-16 years	0.284	768.1317285	91	Grand Index
20	m	13-16 years	0.289	769	92	Grand Index
20	m	13-16 years	0.29	769.3736323	92	Grand Index
20	m	13-16 years	0.295	770	92	Grand Index
20	m	13-16 years	0.296	770.7083779	92	Grand Index
20	m	13-16 years	0.3	771	92	Grand Index
20	m	13-16 years	0.301	771.1009287	92	Grand Index
20	m	13-16 years	0.302	771.9293887	92	Grand Index
20	m	13-16 years	0.305	772	92	Grand Index
20	m	13-16 years	0.306	772.1953595	92	Grand Index
20	m	13-16 years	0.311	773	93	Grand Index
20	m	13-16 years	0.314	774	93	Grand Index
20	m	13-16 years	0.315	774.1422414	93	Grand Index
20	m	13-16 years	0.319	775	93	Grand Index
20	m	13-16 years	0.32	775.9736695	93	Grand Index
20	m	13-16 years	0.324	776	93	Grand Index
20	m	13-16 years	0.327	777	93	Grand Index
20	m	13-16 years	0.328	777.0749619	93	Grand Index
20	m	13-16 years	0.33	778	93	Grand Index
20	m	13-16 years	0.331	778.2347941	93	Grand Index
20	m	13-16 years	0.336	779	94	Grand Index
20	m	13-16 years	0.337	779.0465599	94	Grand Index
20	m	13-16 years	0.34	780	94	Grand Index
20	m	13-16 years	0.341	780.2430831	94	Grand Index
20	m	13-16 years	0.346	781	94	Grand Index
20	m	13-16 years	0.347	781.9601498	94	Grand Index
20	m	13-16 years	0.35	782	94	Grand Index
20	m	13-16 years	0.351	782.8698148	94	Grand Index
20	m	13-16 years	0.354	783	94	Grand Index
20	m	13-16 years	0.355	783.8631342	94	Grand Index
20	m	13-16 years	0.36	784	95	Grand Index
20	m	13-16 years	0.361	784.289821	95	Grand Index
20	m	13-16 years	0.367	785	95	Grand Index
20	m	13-16 years	0.371	786	95	Grand Index
20	m	13-16 years	0.375	787	95	Grand Index
20	m	13-16 years	0.38	788	95	Grand Index

Age	Gender	Education	Percentile	Raw Score	Norm Score	Assessment
20	m	13-16 years	0.383	789	96	Grand Index
20	m	13-16 years	0.389	790	96	Grand Index
20	m	13-16 years	0.39	790.8599324	96	Grand Index
20	m	13-16 years	0.394	791	96	Grand Index
20	m	13-16 years	0.4	792	96	Grand Index
20	m	13-16 years	0.406	793	96	Grand Index
20	m	13-16 years	0.409	794	97	Grand Index
20	m	13-16 years	0.414	795	97	Grand Index
20	m	13-16 years	0.415	795.7242156	97	Grand Index
20	m	13-16 years	0.419	796	97	Grand Index
20	m	13-16 years	0.42	796.9917151	97	Grand Index
20	m	13-16 years	0.424	797	97	Grand Index
20	m	13-16 years	0.425	797.1112077	97	Grand Index
20	m	13-16 years	0.426	797.8302098	97	Grand Index
20	m	13-16 years	0.429	798	97	Grand Index
20	m	13-16 years	0.43	798.8680765	97	Grand Index
20	m	13-16 years	0.435	799	98	Grand Index
20	m	13-16 years	0.436	799.9814197	98	Grand Index
20	m	13-16 years	0.441	800	98	Grand Index
20	m	13-16 years	0.445	801	98	Grand Index
20	m	13-16 years	0.446	801.5126418	98	Grand Index
20	m	13-16 years	0.45	802	98	Grand Index
20	m	13-16 years	0.454	803	98	Grand Index
20	m	13-16 years	0.46	804	98	Grand Index
20	m	13-16 years	0.465	805	99	Grand Index
20	m	13-16 years	0.47	806	99	Grand Index
20	m	13-16 years	0.471	806.1802434	99	Grand Index
20	m	13-16 years	0.475	807	99	Grand Index
20	m	13-16 years	0.476	807.1318859	99	Grand Index
20	m	13-16 years	0.479	808	99	Grand Index
20	m	13-16 years	0.484	809	99	Grand Index
20	m	13-16 years	0.485	809.5396446	99	Grand Index
20	m	13-16 years	0.488	810	100	Grand Index
20	m	13-16 years	0.489	810.135112	100	Grand Index
20	m	13-16 years	0.49	810.7352342	100	Grand Index
20	m	13-16 years	0.495	811	100	Grand Index
20	m	13-16 years	0.496	811.847571	100	Grand Index
20	m	13-16 years	0.501	812	100	Grand Index
20	m	13-16 years	0.502	812.2428806	100	Grand Index
20	m	13-16 years	0.509	813	100	Grand Index
20	m	13-16 years	0.51	813.5989441	100	Grand Index
20	m	13-16 years	0.511	813.9961401	100	Grand Index
20	m	13-16 years	0.515	814	101	Grand Index
20	m	13-16 years	0.516	814.2841806	101	Grand Index
20	m	13-16 years	0.52	815	101	Grand Index
20	m	13-16 years	0.521	815.4516094	101	Grand Index
20	m	13-16 years	0.526	816	101	Grand Index
20	m	13-16 years	0.527	816.0386769	101	Grand Index
20	m	13-16 years	0.531	817	101	Grand Index
20	m	13-16 years	0.536	818	101	Grand Index
20	m	13-16 years	0.537	818.9708373	101	Grand Index
20	m	13-16 years	0.543	819	102	Grand Index
20	m	13-16 years	0.544	819.5111269	102	Grand Index
20	m	13-16 years	0.547	820	102	Grand Index
20	m	13-16 years	0.548	820.0052704	102	Grand Index
20	m	13-16 years	0.549	820.9947848	102	Grand Index

Age	Gender	Education	Percentile	Raw Score	Norm Score	Assessment
20	m	13-16 years	0.553	821	102	Grand Index
20	m	13-16 years	0.559	822	102	Grand Index
20	m	13-16 years	0.566	823	102	Grand Index
20	m	13-16 years	0.567	823.0482732	103	Grand Index
20	m	13-16 years	0.571	824	103	Grand Index
20	m	13-16 years	0.575	825	103	Grand Index
20	m	13-16 years	0.576	825.6881328	103	Grand Index
20	m	13-16 years	0.579	826	103	Grand Index
20	m	13-16 years	0.582	827	103	Grand Index
20	m	13-16 years	0.583	827.0025641	103	Grand Index
20	m	13-16 years	0.584	827.957503	103	Grand Index
20	m	13-16 years	0.588	828	103	Grand Index
20	m	13-16 years	0.589	828.3703837	103	Grand Index
20	m	13-16 years	0.59	828.9786318	103	Grand Index
20	m	13-16 years	0.594	829	104	Grand Index
20	m	13-16 years	0.595	829.2364896	104	Grand Index
20	m	13-16 years	0.599	830	104	Grand Index
20	m	13-16 years	0.602	831	104	Grand Index
20	m	13-16 years	0.603	831.0020223	104	Grand Index
20	m	13-16 years	0.604	831.4966681	104	Grand Index
20	m	13-16 years	0.609	832	104	Grand Index
20	m	13-16 years	0.61	832.0623586	104	Grand Index
20	m	13-16 years	0.612	833	104	Grand Index
20	m	13-16 years	0.613	833.003859	104	Grand Index
20	m	13-16 years	0.614	833.9667139	104	Grand Index
20	m	13-16 years	0.617	834	104	Grand Index
20	m	13-16 years	0.62	835	105	Grand Index
20	m	13-16 years	0.621	835.2880072	105	Grand Index
20	m	13-16 years	0.626	836	105	Grand Index
20	m	13-16 years	0.631	837	105	Grand Index
20	m	13-16 years	0.632	837.1614815	105	Grand Index
20	m	13-16 years	0.636	838	105	Grand Index
20	m	13-16 years	0.637	838.6196462	105	Grand Index
20	m	13-16 years	0.641	839	105	Grand Index
20	m	13-16 years	0.642	839.4010356	105	Grand Index
20	m	13-16 years	0.646	840	106	Grand Index
20	m	13-16 years	0.647	840.6807527	106	Grand Index
20	m	13-16 years	0.652	841	106	Grand Index
20	m	13-16 years	0.653	841.0141285	106	Grand Index
20	m	13-16 years	0.657	842	106	Grand Index
20	m	13-16 years	0.658	842.67	106	Grand Index
20	m	13-16 years	0.663	843	106	Grand Index
20	m	13-16 years	0.664	843.997457	106	Grand Index
20	m	13-16 years	0.668	844	107	Grand Index
20	m	13-16 years	0.671	845	107	Grand Index
20	m	13-16 years	0.672	845.0212731	107	Grand Index
20	m	13-16 years	0.674	846	107	Grand Index
20	m	13-16 years	0.675	846.0274099	107	Grand Index
20	m	13-16 years	0.679	847	107	Grand Index
20	m	13-16 years	0.68	847.9980231	107	Grand Index
20	m	13-16 years	0.683	848	107	Grand Index
20	m	13-16 years	0.684	848.6716054	107	Grand Index
20	m	13-16 years	0.69	849	107	Grand Index
20	m	13-16 years	0.691	849.8343954	107	Grand Index
20	m	13-16 years	0.697	850	108	Grand Index
20	m	13-16 years	0.698	850.6258124	108	Grand Index

Age	Gender	Education	Percentile	Raw Score	Norm Score	Assessment
20	m	13-16 years	0.699	851	108	Grand Index
20	m	13-16 years	0.7	851.3226213	108	Grand Index
20	m	13-16 years	0.704	852	108	Grand Index
20	m	13-16 years	0.705	852.9215027	108	Grand Index
20	m	13-16 years	0.709	853	108	Grand Index
20	m	13-16 years	0.71	853.3057241	108	Grand Index
20	m	13-16 years	0.713	854	108	Grand Index
20	m	13-16 years	0.714	854.000872	108	Grand Index
20	m	13-16 years	0.718	855	109	Grand Index
20	m	13-16 years	0.719	855.9956638	109	Grand Index
20	m	13-16 years	0.725	856	109	Grand Index
20	m	13-16 years	0.73	857	109	Grand Index
20	m	13-16 years	0.733	858	109	Grand Index
20	m	13-16 years	0.734	858.1970432	109	Grand Index
20	m	13-16 years	0.735	858.8890741	109	Grand Index
20	m	13-16 years	0.738	859	110	Grand Index
20	m	13-16 years	0.739	859.8473398	110	Grand Index
20	m	13-16 years	0.74	860.0281314	110	Grand Index
20	m	13-16 years	0.744	861	110	Grand Index
20	m	13-16 years	0.745	861.9954669	110	Grand Index
20	m	13-16 years	0.748	862	110	Grand Index
20	m	13-16 years	0.75	863	110	Grand Index
20	m	13-16 years	0.751	863.0194062	110	Grand Index
20	m	13-16 years	0.752	863.4544579	110	Grand Index
20	m	13-16 years	0.756	864	110	Grand Index
20	m	13-16 years	0.759	865	111	Grand Index
20	m	13-16 years	0.76	865.3705903	111	Grand Index
20	m	13-16 years	0.762	866	111	Grand Index
20	m	13-16 years	0.763	866.0720147	111	Grand Index
20	m	13-16 years	0.765	867	111	Grand Index
20	m	13-16 years	0.766	867.7728252	111	Grand Index
20	m	13-16 years	0.77	868	111	Grand Index
20	m	13-16 years	0.774	869	111	Grand Index
20	m	13-16 years	0.778	870	111	Grand Index
20	m	13-16 years	0.782	871	112	Grand Index
20	m	13-16 years	0.786	872	112	Grand Index
20	m	13-16 years	0.79	873	112	Grand Index
20	m	13-16 years	0.791	873.9707607	112	Grand Index
20	m	13-16 years	0.793	874	112	Grand Index
20	m	13-16 years	0.797	875	112	Grand Index
20	m	13-16 years	0.798	875.9316832	113	Grand Index
20	m	13-16 years	0.801	876	113	Grand Index
20	m	13-16 years	0.802	876.630887	113	Grand Index
20	m	13-16 years	0.804	877	113	Grand Index
20	m	13-16 years	0.805	877.7404931	113	Grand Index
20	m	13-16 years	0.808	878	113	Grand Index
20	m	13-16 years	0.811	879	113	Grand Index
20	m	13-16 years	0.812	879.5043674	113	Grand Index
20	m	13-16 years	0.816	880	114	Grand Index
20	m	13-16 years	0.819	881	114	Grand Index
20	m	13-16 years	0.822	882	114	Grand Index
20	m	13-16 years	0.823	882.3166993	114	Grand Index
20	m	13-16 years	0.825	883	114	Grand Index
20	m	13-16 years	0.826	883.0173415	114	Grand Index
20	m	13-16 years	0.829	884	114	Grand Index
20	m	13-16 years	0.83	884.0419079	114	Grand Index

Age	Gender	Education	Percentile	Raw Score	Norm Score	Assessment
20	m	13-16 years	0.833	885	114	Grand Index
20	m	13-16 years	0.834	885.8182226	115	Grand Index
20	m	13-16 years	0.836	886	115	Grand Index
20	m	13-16 years	0.837	886.9838122	115	Grand Index
20	m	13-16 years	0.84	887	115	Grand Index
20	m	13-16 years	0.842	888	115	Grand Index
20	m	13-16 years	0.844	889	115	Grand Index
20	m	13-16 years	0.845	889.0961538	115	Grand Index
20	m	13-16 years	0.847	890	115	Grand Index
20	m	13-16 years	0.848	890.6086035	115	Grand Index
20	m	13-16 years	0.85	891	116	Grand Index
20	m	13-16 years	0.851	891.9981416	116	Grand Index
20	m	13-16 years	0.854	892	116	Grand Index
20	m	13-16 years	0.856	893	116	Grand Index
20	m	13-16 years	0.857	893.7053696	116	Grand Index
20	m	13-16 years	0.859	894	116	Grand Index
20	m	13-16 years	0.862	895	116	Grand Index
20	m	13-16 years	0.863	895.0624424	116	Grand Index
20	m	13-16 years	0.864	895.9817529	116	Grand Index
20	m	13-16 years	0.866	896	117	Grand Index
20	m	13-16 years	0.867	896.6500221	117	Grand Index
20	m	13-16 years	0.869	897	117	Grand Index
20	m	13-16 years	0.871	898	117	Grand Index
20	m	13-16 years	0.872	898.286956	117	Grand Index
20	m	13-16 years	0.873	899.2818732	117	Grand Index
20	m	13-16 years	0.876	900	117	Grand Index
20	m	13-16 years	0.878	901	117	Grand Index
20	m	13-16 years	0.88	902	118	Grand Index
20	m	13-16 years	0.881	902.4817619	118	Grand Index
20	m	13-16 years	0.882	903.0417916	118	Grand Index
20	m	13-16 years	0.884	904	118	Grand Index
20	m	13-16 years	0.885	904.7328269	118	Grand Index
20	m	13-16 years	0.886	905	118	Grand Index
20	m	13-16 years	0.887	906	118	Grand Index
20	m	13-16 years	0.888	906.8899653	118	Grand Index
20	m	13-16 years	0.89	907	118	Grand Index
20	m	13-16 years	0.892	908	119	Grand Index
20	m	13-16 years	0.894	909	119	Grand Index
20	m	13-16 years	0.895	910	119	Grand Index
20	m	13-16 years	0.896	910.7009591	119	Grand Index
20	m	13-16 years	0.898	911	119	Grand Index
20	m	13-16 years	0.899	911.0265108	119	Grand Index
20	m	13-16 years	0.901	912	119	Grand Index
20	m	13-16 years	0.902	913	119	Grand Index
20	m	13-16 years	0.904	914	120	Grand Index
20	m	13-16 years	0.905	914.075	120	Grand Index
20	m	13-16 years	0.906	915	120	Grand Index
20	m	13-16 years	0.907	915.9700548	120	Grand Index
20	m	13-16 years	0.908	916	120	Grand Index
20	m	13-16 years	0.909	917	120	Grand Index
20	m	13-16 years	0.91	917.4295015	120	Grand Index
20	m	13-16 years	0.911	918	120	Grand Index
20	m	13-16 years	0.912	918.0630464	120	Grand Index
20	m	13-16 years	0.913	919	120	Grand Index
20	m	13-16 years	0.915	920	121	Grand Index
20	m	13-16 years	0.916	921	121	Grand Index

Age	Gender	Education	Percentile	Raw Score	Norm Score	Assessment
20	m	13-16 years	0.918	922	121	Grand Index
20	m	13-16 years	0.919	922.6309315	121	Grand Index
20	m	13-16 years	0.92	923	121	Grand Index
20	m	13-16 years	0.921	923.5318317	121	Grand Index
20	m	13-16 years	0.922	924	121	Grand Index
20	m	13-16 years	0.923	925	121	Grand Index
20	m	13-16 years	0.924	926	121	Grand Index
20	m	13-16 years	0.925	926.3751056	122	Grand Index
20	m	13-16 years	0.927	927	122	Grand Index
20	m	13-16 years	0.928	928	122	Grand Index
20	m	13-16 years	0.929	928.3372271	122	Grand Index
20	m	13-16 years	0.93	929	122	Grand Index
20	m	13-16 years	0.931	929.8458562	122	Grand Index
20	m	13-16 years	0.932	930	122	Grand Index
20	m	13-16 years	0.933	930.1245863	122	Grand Index
20	m	13-16 years	0.934	931	123	Grand Index
20	m	13-16 years	0.935	931.0902558	123	Grand Index
20	m	13-16 years	0.936	932	123	Grand Index
20	m	13-16 years	0.937	932.9895574	123	Grand Index
20	m	13-16 years	0.938	933	123	Grand Index
20	m	13-16 years	0.939	933.496882	123	Grand Index
20	m	13-16 years	0.94	934.0114777	123	Grand Index
20	m	13-16 years	0.941	935	123	Grand Index
20	m	13-16 years	0.942	935.1405577	124	Grand Index
20	m	13-16 years	0.943	936	124	Grand Index
20	m	13-16 years	0.944	936.9680839	124	Grand Index
20	m	13-16 years	0.945	937.9106958	124	Grand Index
20	m	13-16 years	0.946	938	124	Grand Index
20	m	13-16 years	0.947	938.8082788	124	Grand Index
20	m	13-16 years	0.948	940	124	Grand Index
20	m	13-16 years	0.949	940.117612	125	Grand Index
20	m	13-16 years	0.95	941	125	Grand Index
20	m	13-16 years	0.951	941.6791424	125	Grand Index
20	m	13-16 years	0.953	942	125	Grand Index
20	m	13-16 years	0.954	943.71	125	Grand Index
20	m	13-16 years	0.955	945	125	Grand Index
20	m	13-16 years	0.956	945.992713	126	Grand Index
20	m	13-16 years	0.957	946.0546979	126	Grand Index
20	m	13-16 years	0.958	947.0233239	126	Grand Index
20	m	13-16 years	0.959	948	126	Grand Index
20	m	13-16 years	0.96	948.0572961	126	Grand Index
20	m	13-16 years	0.961	949.5515315	126	Grand Index
20	m	13-16 years	0.962	950.2448617	127	Grand Index
20	m	13-16 years	0.963	951	127	Grand Index
20	m	13-16 years	0.964	952	127	Grand Index
20	m	13-16 years	0.965	954	127	Grand Index
20	m	13-16 years	0.966	954.2961534	127	Grand Index
20	m	13-16 years	0.967	955.0151944	128	Grand Index
20	m	13-16 years	0.968	956	128	Grand Index
20	m	13-16 years	0.969	957	128	Grand Index
20	m	13-16 years	0.97	957.4043198	128	Grand Index
20	m	13-16 years	0.971	958.8664255	128	Grand Index
20	m	13-16 years	0.972	959	129	Grand Index
20	m	13-16 years	0.973	959.9657355	129	Grand Index
20	m	13-16 years	0.974	960	129	Grand Index
20	m	13-16 years	0.975	961	129	Grand Index

Age	Gender	Education	Percentile	Raw Score	Norm Score	Assessment
20	m	13-16 years	0.976	961.1871785	130	Grand Index
20	m	13-16 years	0.977	962.1624961	130	Grand Index
20	m	13-16 years	0.978	963.4748594	130	Grand Index
20	m	13-16 years	0.979	965.181148	131	Grand Index
20	m	13-16 years	0.98	967	131	Grand Index
20	m	13-16 years	0.981	968.4055472	131	Grand Index
20	m	13-16 years	0.982	969.9662039	131	Grand Index
20	m	13-16 years	0.983	971.3046862	132	Grand Index
20	m	13-16 years	0.984	973.0054446	132	Grand Index
20	m	13-16 years	0.985	976	133	Grand Index
20	m	13-16 years	0.986	977	133	Grand Index
20	m	13-16 years	0.987	978.9486301	133	Grand Index
20	m	13-16 years	0.988	979.8499898	134	Grand Index
20	m	13-16 years	0.989	981.793127	134	Grand Index
20	m	13-16 years	0.99	983	135	Grand Index
20	m	13-16 years	0.991	986.895	135	Grand Index
20	m	13-16 years	0.992	992	136	Grand Index
20	m	13-16 years	0.993	998.3993003	137	Grand Index
20	m	13-16 years	0.994	1003.2906	138	Grand Index
20	m	13-16 years	0.995	1007.038012	139	Grand Index
20	m	13-16 years	0.996	1012.025402	140	Grand Index
20	m	13-16 years	0.997	1017.959887	141	Grand Index
20	m	13-16 years	0.998	1035.520747	143	Grand Index
20	m	13-16 years	0.999	1047.235673	146	Grand Index
20	f	17+ years	0.001	589.4112968	54	Grand Index
20	f	17+ years	0.002	595.4817199	57	Grand Index
20	f	17+ years	0.003	603.5685671	59	Grand Index
20	f	17+ years	0.004	611.9385925	60	Grand Index
20	f	17+ years	0.005	616.84	61	Grand Index
20	f	17+ years	0.006	620.608	62	Grand Index
20	f	17+ years	0.007	627.5100819	63	Grand Index
20	f	17+ years	0.008	636.2482925	64	Grand Index
20	f	17+ years	0.009	641.4530277	65	Grand Index
20	f	17+ years	0.01	643.2277783	65	Grand Index
20	f	17+ years	0.011	644.6244995	66	Grand Index
20	f	17+ years	0.012	645.4155383	66	Grand Index
20	f	17+ years	0.013	646.984	67	Grand Index
20	f	17+ years	0.014	650.752	67	Grand Index
20	f	17+ years	0.015	654.2770441	67	Grand Index
20	f	17+ years	0.016	656.2845482	68	Grand Index
20	f	17+ years	0.017	658.2883813	68	Grand Index
20	f	17+ years	0.018	660.0488772	69	Grand Index
20	f	17+ years	0.019	661.8093731	69	Grand Index
20	f	17+ years	0.02	663.2348187	69	Grand Index
20	f	17+ years	0.021	664.6195797	69	Grand Index
20	f	17+ years	0.022	665.3237484	70	Grand Index
20	f	17+ years	0.023	665.8229874	70	Grand Index
20	f	17+ years	0.024	666.304	70	Grand Index
20	f	17+ years	0.025	666.775	71	Grand Index
20	f	17+ years	0.026	667.5796735	71	Grand Index
20	f	17+ years	0.027	668.6895362	71	Grand Index
20	f	17+ years	0.028	669.7344354	71	Grand Index
20	f	17+ years	0.029	670.6815438	72	Grand Index
20	f	17+ years	0.03	671.4495007	72	Grand Index
20	f	17+ years	0.031	671.7475296	72	Grand Index
20	f	17+ years	0.032	672.432	72	Grand Index

Age	Gender	Education	Percentile	Raw Score	Norm Score	Assessment
20	f	17+ years	0.033	675.258	72	Grand Index
20	f	17+ years	0.034	678.028	73	Grand Index
20	f	17+ years	0.035	678.97	73	Grand Index
20	f	17+ years	0.036	679.912	73	Grand Index
20	f	17+ years	0.037	680.854	73	Grand Index
20	f	17+ years	0.038	681.796	73	Grand Index
20	f	17+ years	0.039	683.2259695	74	Grand Index
20	f	17+ years	0.04	684.790825	74	Grand Index
20	f	17+ years	0.041	685.3832918	74	Grand Index
20	f	17+ years	0.042	685.4754944	74	Grand Index
20	f	17+ years	0.043	685.6400729	74	Grand Index
20	f	17+ years	0.044	685.8670149	74	Grand Index
20	f	17+ years	0.045	686.195	75	Grand Index
20	f	17+ years	0.046	686.666	75	Grand Index
20	f	17+ years	0.048	687	75	Grand Index
20	f	17+ years	0.049	687.1457295	75	Grand Index
20	f	17+ years	0.05	688.0145727	75	Grand Index
20	f	17+ years	0.051	688.8479394	75	Grand Index
20	f	17+ years	0.052	688.9210963	76	Grand Index
20	f	17+ years	0.053	688.9942531	76	Grand Index
20	f	17+ years	0.054	690.302	76	Grand Index
20	f	17+ years	0.055	691.715	76	Grand Index
20	f	17+ years	0.057	692	76	Grand Index
20	f	17+ years	0.058	692.2164467	76	Grand Index
20	f	17+ years	0.059	692.5370329	77	Grand Index
20	f	17+ years	0.06	692.7636811	77	Grand Index
20	f	17+ years	0.061	692.9140949	77	Grand Index
20	f	17+ years	0.062	694.01	77	Grand Index
20	f	17+ years	0.063	696.365	77	Grand Index
20	f	17+ years	0.064	698.0420813	77	Grand Index
20	f	17+ years	0.065	698.1797222	77	Grand Index
20	f	17+ years	0.066	698.4390993	77	Grand Index
20	f	17+ years	0.067	699.2434584	78	Grand Index
20	f	17+ years	0.068	700.056	78	Grand Index
20	f	17+ years	0.069	700.998	78	Grand Index
20	f	17+ years	0.07	701.94	78	Grand Index
20	f	17+ years	0.071	704.205	78	Grand Index
20	f	17+ years	0.072	706.56	78	Grand Index
20	f	17+ years	0.074	707	78	Grand Index
20	f	17+ years	0.075	708.3685212	78	Grand Index
20	f	17+ years	0.076	710.3518243	79	Grand Index
20	f	17+ years	0.077	711.3108896	79	Grand Index
20	f	17+ years	0.078	711.4873911	79	Grand Index
20	f	17+ years	0.079	711.8811878	79	Grand Index
20	f	17+ years	0.08	712.5473832	79	Grand Index
20	f	17+ years	0.082	713	79	Grand Index
20	f	17+ years	0.083	713.093	79	Grand Index
20	f	17+ years	0.084	713.564	79	Grand Index
20	f	17+ years	0.087	714	80	Grand Index
20	f	17+ years	0.088	714.448	80	Grand Index
20	f	17+ years	0.089	714.919	80	Grand Index
20	f	17+ years	0.091	715	80	Grand Index
20	f	17+ years	0.092	715.2829802	80	Grand Index
20	f	17+ years	0.093	715.6844369	80	Grand Index
20	f	17+ years	0.094	715.892806	80	Grand Index
20	f	17+ years	0.095	715.9623492	80	Grand Index

Age	Gender	Education	Percentile	Raw Score	Norm Score	Assessment
20	f	17+ years	0.096	716.216	80	Grand Index
20	f	17+ years	0.097	716.687	81	Grand Index
20	f	17+ years	0.099	717	81	Grand Index
20	f	17+ years	0.1	717.1	81	Grand Index
20	f	17+ years	0.101	717.571	81	Grand Index
20	f	17+ years	0.102	718.084	81	Grand Index
20	f	17+ years	0.103	719.026	81	Grand Index
20	f	17+ years	0.104	719.968	81	Grand Index
20	f	17+ years	0.105	720.455	81	Grand Index
20	f	17+ years	0.106	720.926	81	Grand Index
20	f	17+ years	0.108	721	81	Grand Index
20	f	17+ years	0.109	721.339	82	Grand Index
20	f	17+ years	0.11	721.81	82	Grand Index
20	f	17+ years	0.112	722	82	Grand Index
20	f	17+ years	0.113	722.223	82	Grand Index
20	f	17+ years	0.114	722.694	82	Grand Index
20	f	17+ years	0.115	723.165	82	Grand Index
20	f	17+ years	0.116	723.636	82	Grand Index
20	f	17+ years	0.117	724.214	82	Grand Index
20	f	17+ years	0.118	725.156	82	Grand Index
20	f	17+ years	0.123	726	83	Grand Index
20	f	17+ years	0.124	726.404	83	Grand Index
20	f	17+ years	0.125	726.875	83	Grand Index
20	f	17+ years	0.126	727.0485908	83	Grand Index
20	f	17+ years	0.127	727.1147361	83	Grand Index
20	f	17+ years	0.128	727.6730114	83	Grand Index
20	f	17+ years	0.129	728.5439943	83	Grand Index
20	f	17+ years	0.13	728.9920355	83	Grand Index
20	f	17+ years	0.131	728.9969073	83	Grand Index
20	f	17+ years	0.133	729	83	Grand Index
20	f	17+ years	0.134	729.114	83	Grand Index
20	f	17+ years	0.135	729.585	83	Grand Index
20	f	17+ years	0.14	730	84	Grand Index
20	f	17+ years	0.141	730.1287806	84	Grand Index
20	f	17+ years	0.142	730.2763613	84	Grand Index
20	f	17+ years	0.143	730.9087276	84	Grand Index
20	f	17+ years	0.144	731.7031469	84	Grand Index
20	f	17+ years	0.145	732.295	84	Grand Index
20	f	17+ years	0.146	732.766	84	Grand Index
20	f	17+ years	0.15	733	84	Grand Index
20	f	17+ years	0.151	733.0540721	85	Grand Index
20	f	17+ years	0.152	733.2645509	85	Grand Index
20	f	17+ years	0.153	733.4817233	85	Grand Index
20	f	17+ years	0.154	733.7422444	85	Grand Index
20	f	17+ years	0.155	734.005	85	Grand Index
20	f	17+ years	0.156	734.476	85	Grand Index
20	f	17+ years	0.157	734.947	85	Grand Index
20	f	17+ years	0.158	735.418	85	Grand Index
20	f	17+ years	0.159	735.889	85	Grand Index
20	f	17+ years	0.16	736.36	85	Grand Index
20	f	17+ years	0.161	736.831	85	Grand Index
20	f	17+ years	0.165	737	85	Grand Index
20	f	17+ years	0.166	737.186	85	Grand Index
20	f	17+ years	0.167	737.657	86	Grand Index
20	f	17+ years	0.174	738	86	Grand Index
20	f	17+ years	0.175	738.425	86	Grand Index

Age	Gender	Education	Percentile	Raw Score	Norm Score	Assessment
20	f	17+ years	0.176	738.896	86	Grand Index
20	f	17+ years	0.178	739	86	Grand Index
20	f	17+ years	0.179	739.618	86	Grand Index
20	f	17+ years	0.18	740.56	86	Grand Index
20	f	17+ years	0.181	741.251	86	Grand Index
20	f	17+ years	0.182	741.722	86	Grand Index
20	f	17+ years	0.183	742.0846427	86	Grand Index
20	f	17+ years	0.184	742.291206	86	Grand Index
20	f	17+ years	0.185	742.5143573	87	Grand Index
20	f	17+ years	0.186	742.778794	87	Grand Index
20	f	17+ years	0.188	743	87	Grand Index
20	f	17+ years	0.189	743.0186117	87	Grand Index
20	f	17+ years	0.19	743.4799871	87	Grand Index
20	f	17+ years	0.191	743.9413624	87	Grand Index
20	f	17+ years	0.192	744.4203932	87	Grand Index
20	f	17+ years	0.193	744.9010179	87	Grand Index
20	f	17+ years	0.195	745	87	Grand Index
20	f	17+ years	0.196	745.316	87	Grand Index
20	f	17+ years	0.197	745.787	87	Grand Index
20	f	17+ years	0.199	746	87	Grand Index
20	f	17+ years	0.2	746.0051185	87	Grand Index
20	f	17+ years	0.201	746.0171726	87	Grand Index
20	f	17+ years	0.202	746.1639585	87	Grand Index
20	f	17+ years	0.203	746.6229043	88	Grand Index
20	f	17+ years	0.204	747.084	88	Grand Index
20	f	17+ years	0.205	747.555	88	Grand Index
20	f	17+ years	0.206	748.026	88	Grand Index
20	f	17+ years	0.207	748.497	88	Grand Index
20	f	17+ years	0.208	748.968	88	Grand Index
20	f	17+ years	0.209	749.439	88	Grand Index
20	f	17+ years	0.21	749.91	88	Grand Index
20	f	17+ years	0.212	750	88	Grand Index
20	f	17+ years	0.213	750.323	88	Grand Index
20	f	17+ years	0.214	750.794	88	Grand Index
20	f	17+ years	0.215	751.265	88	Grand Index
20	f	17+ years	0.216	751.736	88	Grand Index
20	f	17+ years	0.22	752	88	Grand Index
20	f	17+ years	0.221	752.091	88	Grand Index
20	f	17+ years	0.222	752.562	89	Grand Index
20	f	17+ years	0.223	753.033	89	Grand Index
20	f	17+ years	0.224	753.504	89	Grand Index
20	f	17+ years	0.225	753.975	89	Grand Index
20	f	17+ years	0.226	754.5357864	89	Grand Index
20	f	17+ years	0.227	755.1016056	89	Grand Index
20	f	17+ years	0.228	755.5112046	89	Grand Index
20	f	17+ years	0.229	755.8873854	89	Grand Index
20	f	17+ years	0.233	756	89	Grand Index
20	f	17+ years	0.234	756.5087163	89	Grand Index
20	f	17+ years	0.235	757.6283677	89	Grand Index
20	f	17+ years	0.236	758.4743391	89	Grand Index
20	f	17+ years	0.237	758.7676878	89	Grand Index
20	f	17+ years	0.239	759	89	Grand Index
20	f	17+ years	0.24	759.026008	89	Grand Index
20	f	17+ years	0.241	759.3322524	89	Grand Index
20	f	17+ years	0.242	759.6384967	90	Grand Index
20	f	17+ years	0.243	759.8086596	90	Grand Index

Age	Gender	Education	Percentile	Raw Score	Norm Score	Assessment
20	f	17+ years	0.244	759.9734152	90	Grand Index
20	f	17+ years	0.245	760.395	90	Grand Index
20	f	17+ years	0.246	760.866	90	Grand Index
20	f	17+ years	0.247	761.337	90	Grand Index
20	f	17+ years	0.248	761.808	90	Grand Index
20	f	17+ years	0.249	762.279	90	Grand Index
20	f	17+ years	0.25	762.75	90	Grand Index
20	f	17+ years	0.254	763	90	Grand Index
20	f	17+ years	0.255	763.105	90	Grand Index
20	f	17+ years	0.256	763.576	90	Grand Index
20	f	17+ years	0.261	764	90	Grand Index
20	f	17+ years	0.262	764.402	90	Grand Index
20	f	17+ years	0.263	764.873	90	Grand Index
20	f	17+ years	0.269	765	91	Grand Index
20	f	17+ years	0.27	765.119561	91	Grand Index
20	f	17+ years	0.271	765.4508153	91	Grand Index
20	f	17+ years	0.272	765.7365304	91	Grand Index
20	f	17+ years	0.273	765.8762761	91	Grand Index
20	f	17+ years	0.28	766	91	Grand Index
20	f	17+ years	0.281	766.351	91	Grand Index
20	f	17+ years	0.282	766.822	91	Grand Index
20	f	17+ years	0.283	767.586	91	Grand Index
20	f	17+ years	0.284	768.528	91	Grand Index
20	f	17+ years	0.285	769.235	91	Grand Index
20	f	17+ years	0.286	769.706	92	Grand Index
20	f	17+ years	0.287	770.354	92	Grand Index
20	f	17+ years	0.288	771.296	92	Grand Index
20	f	17+ years	0.29	772	92	Grand Index
20	f	17+ years	0.291	772.122	92	Grand Index
20	f	17+ years	0.292	773.064	92	Grand Index
20	f	17+ years	0.295	774	92	Grand Index
20	f	17+ years	0.296	774.0589972	92	Grand Index
20	f	17+ years	0.297	774.1257946	92	Grand Index
20	f	17+ years	0.298	774.4490486	92	Grand Index
20	f	17+ years	0.299	774.8532513	92	Grand Index
20	f	17+ years	0.3	775.0292874	92	Grand Index
20	f	17+ years	0.301	775.0752687	92	Grand Index
20	f	17+ years	0.302	775.3159996	92	Grand Index
20	f	17+ years	0.303	775.7410183	92	Grand Index
20	f	17+ years	0.305	776	92	Grand Index
20	f	17+ years	0.306	776.252	92	Grand Index
20	f	17+ years	0.307	777.194	92	Grand Index
20	f	17+ years	0.308	778.0519563	92	Grand Index
20	f	17+ years	0.309	778.4118297	93	Grand Index
20	f	17+ years	0.31	778.7664219	93	Grand Index
20	f	17+ years	0.311	778.8775485	93	Grand Index
20	f	17+ years	0.312	778.988675	93	Grand Index
20	f	17+ years	0.313	779.2446772	93	Grand Index
20	f	17+ years	0.314	779.5171192	93	Grand Index
20	f	17+ years	0.315	779.732305	93	Grand Index
20	f	17+ years	0.316	779.930863	93	Grand Index
20	f	17+ years	0.32	780	93	Grand Index
20	f	17+ years	0.321	780.191	93	Grand Index
20	f	17+ years	0.322	780.662	93	Grand Index
20	f	17+ years	0.326	781	93	Grand Index
20	f	17+ years	0.327	781.0139152	93	Grand Index

Age	Gender	Education	Percentile	Raw Score	Norm Score	Assessment
20	f	17+ years	0.328	781.3994477	93	Grand Index
20	f	17+ years	0.329	781.7849803	93	Grand Index
20	f	17+ years	0.33	781.896568	93	Grand Index
20	f	17+ years	0.331	781.9820355	93	Grand Index
20	f	17+ years	0.332	782.0712977	93	Grand Index
20	f	17+ years	0.333	782.1615699	94	Grand Index
20	f	17+ years	0.334	782.4454792	94	Grand Index
20	f	17+ years	0.335	782.826207	94	Grand Index
20	f	17+ years	0.339	783	94	Grand Index
20	f	17+ years	0.34	783.14	94	Grand Index
20	f	17+ years	0.341	783.611	94	Grand Index
20	f	17+ years	0.343	784	94	Grand Index
20	f	17+ years	0.344	784.024	94	Grand Index
20	f	17+ years	0.345	784.495	94	Grand Index
20	f	17+ years	0.346	784.966	94	Grand Index
20	f	17+ years	0.348	785	94	Grand Index
20	f	17+ years	0.349	785.379	94	Grand Index
20	f	17+ years	0.35	785.85	94	Grand Index
20	f	17+ years	0.354	786	94	Grand Index
20	f	17+ years	0.355	786.0112601	94	Grand Index
20	f	17+ years	0.356	786.0371309	94	Grand Index
20	f	17+ years	0.357	786.193853	95	Grand Index
20	f	17+ years	0.358	786.6389822	95	Grand Index
20	f	17+ years	0.36	787	95	Grand Index
20	f	17+ years	0.361	787.0284462	95	Grand Index
20	f	17+ years	0.362	787.460645	95	Grand Index
20	f	17+ years	0.363	787.8928438	95	Grand Index
20	f	17+ years	0.364	787.9541965	95	Grand Index
20	f	17+ years	0.365	787.9929977	95	Grand Index
20	f	17+ years	0.367	788	95	Grand Index
20	f	17+ years	0.368	788.984	95	Grand Index
20	f	17+ years	0.369	790.397	95	Grand Index
20	f	17+ years	0.377	791	95	Grand Index
20	f	17+ years	0.378	791.038	95	Grand Index
20	f	17+ years	0.379	791.509	95	Grand Index
20	f	17+ years	0.38	791.98	95	Grand Index
20	f	17+ years	0.386	792	96	Grand Index
20	f	17+ years	0.387	792.277	96	Grand Index
20	f	17+ years	0.388	792.748	96	Grand Index
20	f	17+ years	0.392	793	96	Grand Index
20	f	17+ years	0.393	793.0060153	96	Grand Index
20	f	17+ years	0.394	793.033522	96	Grand Index
20	f	17+ years	0.395	793.1007727	96	Grand Index
20	f	17+ years	0.396	793.5442659	96	Grand Index
20	f	17+ years	0.397	793.9877592	96	Grand Index
20	f	17+ years	0.405	794	96	Grand Index
20	f	17+ years	0.406	794.226	96	Grand Index
20	f	17+ years	0.407	794.697	96	Grand Index
20	f	17+ years	0.409	795	97	Grand Index
20	f	17+ years	0.41	795.11	97	Grand Index
20	f	17+ years	0.411	795.581	97	Grand Index
20	f	17+ years	0.418	796	97	Grand Index
20	f	17+ years	0.419	796.349	97	Grand Index
20	f	17+ years	0.42	796.82	97	Grand Index
20	f	17+ years	0.422	797	97	Grand Index
20	f	17+ years	0.423	797.233	97	Grand Index

Age	Gender	Education	Percentile	Raw Score	Norm Score	Assessment
20	f	17+ years	0.424	797.704	97	Grand Index
20	f	17+ years	0.425	798.175	97	Grand Index
20	f	17+ years	0.426	798.646	97	Grand Index
20	f	17+ years	0.428	799	97	Grand Index
20	f	17+ years	0.429	799.059	97	Grand Index
20	f	17+ years	0.43	799.53	97	Grand Index
20	f	17+ years	0.435	800	98	Grand Index
20	f	17+ years	0.436	800.0123653	98	Grand Index
20	f	17+ years	0.437	800.0287251	98	Grand Index
20	f	17+ years	0.438	800.6203833	98	Grand Index
20	f	17+ years	0.439	801.5460236	98	Grand Index
20	f	17+ years	0.441	802	98	Grand Index
20	f	17+ years	0.442	802.182	98	Grand Index
20	f	17+ years	0.443	802.653	98	Grand Index
20	f	17+ years	0.45	803	98	Grand Index
20	f	17+ years	0.451	803.023641	98	Grand Index
20	f	17+ years	0.452	803.0500897	98	Grand Index
20	f	17+ years	0.453	803.3987704	98	Grand Index
20	f	17+ years	0.454	803.8433216	98	Grand Index
20	f	17+ years	0.458	804	98	Grand Index
20	f	17+ years	0.459	804.189	98	Grand Index
20	f	17+ years	0.46	804.66	98	Grand Index
20	f	17+ years	0.462	805	99	Grand Index
20	f	17+ years	0.463	805.073	99	Grand Index
20	f	17+ years	0.464	805.544	99	Grand Index
20	f	17+ years	0.473	806	99	Grand Index
20	f	17+ years	0.474	806.254	99	Grand Index
20	f	17+ years	0.475	806.725	99	Grand Index
20	f	17+ years	0.481	807	99	Grand Index
20	f	17+ years	0.482	807.022	99	Grand Index
20	f	17+ years	0.483	807.493	99	Grand Index
20	f	17+ years	0.484	807.964	99	Grand Index
20	f	17+ years	0.485	808.4297824	99	Grand Index
20	f	17+ years	0.486	808.8951331	99	Grand Index
20	f	17+ years	0.487	808.9925275	100	Grand Index
20	f	17+ years	0.488	808.9981768	100	Grand Index
20	f	17+ years	0.492	809	100	Grand Index
20	f	17+ years	0.493	809.203	100	Grand Index
20	f	17+ years	0.494	809.674	100	Grand Index
20	f	17+ years	0.495	810.145	100	Grand Index
20	f	17+ years	0.496	810.616	100	Grand Index
20	f	17+ years	0.497	811.0299505	100	Grand Index
20	f	17+ years	0.498	811.1920964	100	Grand Index
20	f	17+ years	0.499	811.3632752	100	Grand Index
20	f	17+ years	0.5	811.6721294	100	Grand Index
20	f	17+ years	0.501	811.9809835	100	Grand Index
20	f	17+ years	0.505	812	100	Grand Index
20	f	17+ years	0.506	812.326	100	Grand Index
20	f	17+ years	0.507	812.797	100	Grand Index
20	f	17+ years	0.511	813	100	Grand Index
20	f	17+ years	0.512	813.152	100	Grand Index
20	f	17+ years	0.513	813.623	100	Grand Index
20	f	17+ years	0.515	814	101	Grand Index
20	f	17+ years	0.516	814.036	101	Grand Index
20	f	17+ years	0.517	814.507	101	Grand Index
20	f	17+ years	0.518	814.978	101	Grand Index

Age	Gender	Education	Percentile	Raw Score	Norm Score	Assessment
20	f	17+ years	0.522	815	101	Grand Index
20	f	17+ years	0.523	815.333	101	Grand Index
20	f	17+ years	0.524	815.804	101	Grand Index
20	f	17+ years	0.53	816	101	Grand Index
20	f	17+ years	0.531	816.0763429	101	Grand Index
20	f	17+ years	0.532	816.4323578	101	Grand Index
20	f	17+ years	0.533	816.7663679	101	Grand Index
20	f	17+ years	0.534	816.881353	101	Grand Index
20	f	17+ years	0.535	816.9963381	101	Grand Index
20	f	17+ years	0.537	817	101	Grand Index
20	f	17+ years	0.538	817.367865	101	Grand Index
20	f	17+ years	0.539	817.8032028	101	Grand Index
20	f	17+ years	0.54	817.9500275	102	Grand Index
20	f	17+ years	0.541	817.9856897	102	Grand Index
20	f	17+ years	0.543	818	102	Grand Index
20	f	17+ years	0.544	818.224	102	Grand Index
20	f	17+ years	0.545	818.695	102	Grand Index
20	f	17+ years	0.547	819	102	Grand Index
20	f	17+ years	0.548	819.216	102	Grand Index
20	f	17+ years	0.549	820.158	102	Grand Index
20	f	17+ years	0.55	821.05	102	Grand Index
20	f	17+ years	0.551	821.521	102	Grand Index
20	f	17+ years	0.552	821.992	102	Grand Index
20	f	17+ years	0.554	822	102	Grand Index
20	f	17+ years	0.555	822.0853833	102	Grand Index
20	f	17+ years	0.556	822.1846809	102	Grand Index
20	f	17+ years	0.557	822.4846674	102	Grand Index
20	f	17+ years	0.558	822.8563698	102	Grand Index
20	f	17+ years	0.559	823.289	102	Grand Index
20	f	17+ years	0.56	823.76	102	Grand Index
20	f	17+ years	0.562	824	102	Grand Index
20	f	17+ years	0.563	824.062359	102	Grand Index
20	f	17+ years	0.564	824.2321342	102	Grand Index
20	f	17+ years	0.565	824.5490043	102	Grand Index
20	f	17+ years	0.566	825.3212291	102	Grand Index
20	f	17+ years	0.573	826	103	Grand Index
20	f	17+ years	0.574	826.354	103	Grand Index
20	f	17+ years	0.575	826.825	103	Grand Index
20	f	17+ years	0.581	827	103	Grand Index
20	f	17+ years	0.582	827.0450199	103	Grand Index
20	f	17+ years	0.583	827.2188261	103	Grand Index
20	f	17+ years	0.584	827.4093984	103	Grand Index
20	f	17+ years	0.585	827.7065921	103	Grand Index
20	f	17+ years	0.592	828	103	Grand Index
20	f	17+ years	0.593	828.303	104	Grand Index
20	f	17+ years	0.594	828.774	104	Grand Index
20	f	17+ years	0.595	829.1184639	104	Grand Index
20	f	17+ years	0.596	829.3462048	104	Grand Index
20	f	17+ years	0.597	829.5801069	104	Grand Index
20	f	17+ years	0.598	829.823366	104	Grand Index
20	f	17+ years	0.599	830.0167427	104	Grand Index
20	f	17+ years	0.6	830.0778732	104	Grand Index
20	f	17+ years	0.601	830.1915736	104	Grand Index
20	f	17+ years	0.602	830.6014432	104	Grand Index
20	f	17+ years	0.605	831	104	Grand Index
20	f	17+ years	0.606	831.426	104	Grand Index

Age	Gender	Education	Percentile	Raw Score	Norm Score	Assessment
20	f	17+ years	0.607	831.897	104	Grand Index
20	f	17+ years	0.609	832	104	Grand Index
20	f	17+ years	0.61	832.31	104	Grand Index
20	f	17+ years	0.611	832.781	104	Grand Index
20	f	17+ years	0.622	833	105	Grand Index
20	f	17+ years	0.623	833.0573244	105	Grand Index
20	f	17+ years	0.624	833.1196797	105	Grand Index
20	f	17+ years	0.625	833.3949948	105	Grand Index
20	f	17+ years	0.626	833.7248278	105	Grand Index
20	f	17+ years	0.627	833.8857144	105	Grand Index
20	f	17+ years	0.628	833.9645263	105	Grand Index
20	f	17+ years	0.629	834.3071509	105	Grand Index
20	f	17+ years	0.63	834.8657148	105	Grand Index
20	f	17+ years	0.631	835.5505426	105	Grand Index
20	f	17+ years	0.632	836.4049787	105	Grand Index
20	f	17+ years	0.633	837.143	105	Grand Index
20	f	17+ years	0.634	837.614	105	Grand Index
20	f	17+ years	0.636	838	105	Grand Index
20	f	17+ years	0.637	838.0077598	105	Grand Index
20	f	17+ years	0.638	838.143125	105	Grand Index
20	f	17+ years	0.639	838.2784903	105	Grand Index
20	f	17+ years	0.64	838.6009438	105	Grand Index
20	f	17+ years	0.641	838.9365786	105	Grand Index
20	f	17+ years	0.642	839.382	105	Grand Index
20	f	17+ years	0.643	839.853	105	Grand Index
20	f	17+ years	0.644	840.324	106	Grand Index
20	f	17+ years	0.645	840.795	106	Grand Index
20	f	17+ years	0.651	841	106	Grand Index
20	f	17+ years	0.652	841.092	106	Grand Index
20	f	17+ years	0.653	841.563	106	Grand Index
20	f	17+ years	0.66	842	106	Grand Index
20	f	17+ years	0.661	842.1012691	106	Grand Index
20	f	17+ years	0.662	842.2453711	106	Grand Index
20	f	17+ years	0.663	842.4954249	106	Grand Index
20	f	17+ years	0.664	842.8223229	106	Grand Index
20	f	17+ years	0.665	843.215	106	Grand Index
20	f	17+ years	0.666	843.686	106	Grand Index
20	f	17+ years	0.675	844	107	Grand Index
20	f	17+ years	0.676	844.2556608	107	Grand Index
20	f	17+ years	0.677	844.5597422	107	Grand Index
20	f	17+ years	0.678	844.7653926	107	Grand Index
20	f	17+ years	0.679	844.9323112	107	Grand Index
20	f	17+ years	0.685	845	107	Grand Index
20	f	17+ years	0.686	845.0952792	107	Grand Index
20	f	17+ years	0.687	845.5186426	107	Grand Index
20	f	17+ years	0.688	845.9037153	107	Grand Index
20	f	17+ years	0.689	845.951352	107	Grand Index
20	f	17+ years	0.69	845.9989886	107	Grand Index
20	f	17+ years	0.691	846.461	107	Grand Index
20	f	17+ years	0.692	846.932	108	Grand Index
20	f	17+ years	0.693	847.403	108	Grand Index
20	f	17+ years	0.694	847.874	108	Grand Index
20	f	17+ years	0.696	848	108	Grand Index
20	f	17+ years	0.697	848.574	108	Grand Index
20	f	17+ years	0.698	849.516	108	Grand Index
20	f	17+ years	0.702	850	108	Grand Index

Age	Gender	Education	Percentile	Raw Score	Norm Score	Assessment
20	f	17+ years	0.703	850.113	108	Grand Index
20	f	17+ years	0.704	850.584	108	Grand Index
20	f	17+ years	0.707	851	108	Grand Index
20	f	17+ years	0.708	851.1171329	108	Grand Index
20	f	17+ years	0.709	851.2350167	108	Grand Index
20	f	17+ years	0.71	851.5576676	108	Grand Index
20	f	17+ years	0.711	851.9107838	108	Grand Index
20	f	17+ years	0.712	852.1941964	108	Grand Index
20	f	17+ years	0.713	852.4540443	108	Grand Index
20	f	17+ years	0.714	852.6834961	108	Grand Index
20	f	17+ years	0.715	852.8946481	109	Grand Index
20	f	17+ years	0.721	853	109	Grand Index
20	f	17+ years	0.722	853.062	109	Grand Index
20	f	17+ years	0.723	853.533	109	Grand Index
20	f	17+ years	0.726	854	109	Grand Index
20	f	17+ years	0.727	854.417	109	Grand Index
20	f	17+ years	0.728	854.888	109	Grand Index
20	f	17+ years	0.73	855	109	Grand Index
20	f	17+ years	0.731	855.301	109	Grand Index
20	f	17+ years	0.732	855.772	109	Grand Index
20	f	17+ years	0.734	856	109	Grand Index
20	f	17+ years	0.735	856.185	109	Grand Index
20	f	17+ years	0.736	856.656	109	Grand Index
20	f	17+ years	0.738	857	110	Grand Index
20	f	17+ years	0.739	857.069	110	Grand Index
20	f	17+ years	0.74	857.54	110	Grand Index
20	f	17+ years	0.741	858.011	110	Grand Index
20	f	17+ years	0.742	858.482	110	Grand Index
20	f	17+ years	0.743	858.953	110	Grand Index
20	f	17+ years	0.747	859	110	Grand Index
20	f	17+ years	0.748	859.308	110	Grand Index
20	f	17+ years	0.749	859.779	110	Grand Index
20	f	17+ years	0.753	860	110	Grand Index
20	f	17+ years	0.754	860.0820692	110	Grand Index
20	f	17+ years	0.755	860.3705363	110	Grand Index
20	f	17+ years	0.756	860.64191	110	Grand Index
20	f	17+ years	0.757	860.8244429	110	Grand Index
20	f	17+ years	0.758	861.036	110	Grand Index
20	f	17+ years	0.759	861.978	111	Grand Index
20	f	17+ years	0.76	862.92	111	Grand Index
20	f	17+ years	0.761	863.168824	111	Grand Index
20	f	17+ years	0.762	863.353316	111	Grand Index
20	f	17+ years	0.763	863.8370212	111	Grand Index
20	f	17+ years	0.764	864.3993399	111	Grand Index
20	f	17+ years	0.765	864.7161261	111	Grand Index
20	f	17+ years	0.766	864.9113153	111	Grand Index
20	f	17+ years	0.77	865	111	Grand Index
20	f	17+ years	0.771	865.141	111	Grand Index
20	f	17+ years	0.772	865.612	111	Grand Index
20	f	17+ years	0.774	866	111	Grand Index
20	f	17+ years	0.775	866.025	111	Grand Index
20	f	17+ years	0.776	866.496	111	Grand Index
20	f	17+ years	0.777	866.967	111	Grand Index
20	f	17+ years	0.778	867.438	111	Grand Index
20	f	17+ years	0.779	867.909	112	Grand Index
20	f	17+ years	0.78	868.38	112	Grand Index

Age	Gender	Education	Percentile	Raw Score	Norm Score	Assessment
20	f	17+ years	0.781	868.851	112	Grand Index
20	f	17+ years	0.787	869	112	Grand Index
20	f	17+ years	0.788	869.148	112	Grand Index
20	f	17+ years	0.789	869.619	112	Grand Index
20	f	17+ years	0.79	870.0700413	112	Grand Index
20	f	17+ years	0.791	870.4365907	112	Grand Index
20	f	17+ years	0.792	870.8262608	112	Grand Index
20	f	17+ years	0.793	871.5331177	112	Grand Index
20	f	17+ years	0.794	872.2399746	112	Grand Index
20	f	17+ years	0.795	872.5998418	112	Grand Index
20	f	17+ years	0.796	872.9394355	112	Grand Index
20	f	17+ years	0.8	873	113	Grand Index
20	f	17+ years	0.801	873.271	113	Grand Index
20	f	17+ years	0.802	873.742	113	Grand Index
20	f	17+ years	0.803	874.213	113	Grand Index
20	f	17+ years	0.804	874.684	113	Grand Index
20	f	17+ years	0.811	875	113	Grand Index
20	f	17+ years	0.812	875.452	113	Grand Index
20	f	17+ years	0.813	875.923	113	Grand Index
20	f	17+ years	0.814	876.2875976	113	Grand Index
20	f	17+ years	0.815	876.6314009	113	Grand Index
20	f	17+ years	0.816	876.8206823	114	Grand Index
20	f	17+ years	0.817	876.947879	114	Grand Index
20	f	17+ years	0.819	877	114	Grand Index
20	f	17+ years	0.82	877.1155507	114	Grand Index
20	f	17+ years	0.821	877.3629343	114	Grand Index
20	f	17+ years	0.822	877.6021432	114	Grand Index
20	f	17+ years	0.823	877.8257596	114	Grand Index
20	f	17+ years	0.824	878.104	114	Grand Index
20	f	17+ years	0.825	878.575	114	Grand Index
20	f	17+ years	0.828	879	114	Grand Index
20	f	17+ years	0.829	879.459	114	Grand Index
20	f	17+ years	0.83	879.93	114	Grand Index
20	f	17+ years	0.832	880	114	Grand Index
20	f	17+ years	0.833	880.343	114	Grand Index
20	f	17+ years	0.834	880.814	115	Grand Index
20	f	17+ years	0.836	881	115	Grand Index
20	f	17+ years	0.837	881.227	115	Grand Index
20	f	17+ years	0.838	881.698	115	Grand Index
20	f	17+ years	0.839	882.1288021	115	Grand Index
20	f	17+ years	0.84	882.4877713	115	Grand Index
20	f	17+ years	0.841	882.8995449	115	Grand Index
20	f	17+ years	0.842	883.4825757	115	Grand Index
20	f	17+ years	0.843	884.106	115	Grand Index
20	f	17+ years	0.844	885.048	115	Grand Index
20	f	17+ years	0.845	885.99	115	Grand Index
20	f	17+ years	0.853	886	116	Grand Index
20	f	17+ years	0.854	886.234	116	Grand Index
20	f	17+ years	0.855	886.705	116	Grand Index
20	f	17+ years	0.856	887.352	116	Grand Index
20	f	17+ years	0.857	888.294	116	Grand Index
20	f	17+ years	0.858	889.118	116	Grand Index
20	f	17+ years	0.859	889.589	116	Grand Index
20	f	17+ years	0.86	890.06	116	Grand Index
20	f	17+ years	0.861	890.531	116	Grand Index
20	f	17+ years	0.862	891.002	116	Grand Index

Age	Gender	Education	Percentile	Raw Score	Norm Score	Assessment
20	f	17+ years	0.863	891.473	116	Grand Index
20	f	17+ years	0.864	891.944	116	Grand Index
20	f	17+ years	0.865	892.415	117	Grand Index
20	f	17+ years	0.866	892.886	117	Grand Index
20	f	17+ years	0.867	893.2834262	117	Grand Index
20	f	17+ years	0.868	893.6573583	117	Grand Index
20	f	17+ years	0.869	893.8555316	117	Grand Index
20	f	17+ years	0.87	893.9525995	117	Grand Index
20	f	17+ years	0.871	894.241	117	Grand Index
20	f	17+ years	0.872	894.712	117	Grand Index
20	f	17+ years	0.873	895.243661	117	Grand Index
20	f	17+ years	0.874	895.8707883	117	Grand Index
20	f	17+ years	0.875	896.5400455	117	Grand Index
20	f	17+ years	0.876	897.3259182	117	Grand Index
20	f	17+ years	0.877	898.1405469	117	Grand Index
20	f	17+ years	0.878	899.1285705	117	Grand Index
20	f	17+ years	0.879	900.1058352	118	Grand Index
20	f	17+ years	0.88	900.5308116	118	Grand Index
20	f	17+ years	0.881	900.955788	118	Grand Index
20	f	17+ years	0.882	901.422	118	Grand Index
20	f	17+ years	0.883	901.893	118	Grand Index
20	f	17+ years	0.887	902	118	Grand Index
20	f	17+ years	0.888	902.496	118	Grand Index
20	f	17+ years	0.889	903.438	118	Grand Index
20	f	17+ years	0.89	904.38	118	Grand Index
20	f	17+ years	0.891	905.322	118	Grand Index
20	f	17+ years	0.892	906.132	119	Grand Index
20	f	17+ years	0.893	906.603	119	Grand Index
20	f	17+ years	0.894	907.0021996	119	Grand Index
20	f	17+ years	0.895	907.0161996	119	Grand Index
20	f	17+ years	0.896	907.0452484	119	Grand Index
20	f	17+ years	0.897	907.5022484	119	Grand Index
20	f	17+ years	0.898	907.9592484	119	Grand Index
20	f	17+ years	0.899	909.287	119	Grand Index
20	f	17+ years	0.9	910.7	119	Grand Index
20	f	17+ years	0.901	911.371	119	Grand Index
20	f	17+ years	0.902	911.842	119	Grand Index
20	f	17+ years	0.903	912.626	119	Grand Index
20	f	17+ years	0.904	913.568	120	Grand Index
20	f	17+ years	0.906	914	120	Grand Index
20	f	17+ years	0.907	914.394	120	Grand Index
20	f	17+ years	0.908	915.336	120	Grand Index
20	f	17+ years	0.909	916.139	120	Grand Index
20	f	17+ years	0.91	916.61	120	Grand Index
20	f	17+ years	0.912	917	120	Grand Index
20	f	17+ years	0.913	917.0101523	120	Grand Index
20	f	17+ years	0.914	917.2180547	120	Grand Index
20	f	17+ years	0.915	917.425957	121	Grand Index
20	f	17+ years	0.916	917.6849531	121	Grand Index
20	f	17+ years	0.917	917.9480508	121	Grand Index
20	f	17+ years	0.918	918.378	121	Grand Index
20	f	17+ years	0.919	918.849	121	Grand Index
20	f	17+ years	0.92	919.32	121	Grand Index
20	f	17+ years	0.921	919.791	121	Grand Index
20	f	17+ years	0.922	921.7379901	121	Grand Index
20	f	17+ years	0.923	924.8623921	121	Grand Index

Age	Gender	Education	Percentile	Raw Score	Norm Score	Assessment
20	f	17+ years	0.924	926.7083057	121	Grand Index
20	f	17+ years	0.925	926.8809037	122	Grand Index
20	f	17+ years	0.926	927.2117295	122	Grand Index
20	f	17+ years	0.927	927.8947745	122	Grand Index
20	f	17+ years	0.928	928.498584	122	Grand Index
20	f	17+ years	0.929	928.757539	122	Grand Index
20	f	17+ years	0.93	929.06	122	Grand Index
20	f	17+ years	0.931	930.002	122	Grand Index
20	f	17+ years	0.932	930.944	122	Grand Index
20	f	17+ years	0.933	931.1193672	122	Grand Index
20	f	17+ years	0.934	931.2462791	123	Grand Index
20	f	17+ years	0.935	931.9357129	123	Grand Index
20	f	17+ years	0.936	932.7508011	123	Grand Index
20	f	17+ years	0.937	933.654	123	Grand Index
20	f	17+ years	0.938	934.596	123	Grand Index
20	f	17+ years	0.939	935.269	123	Grand Index
20	f	17+ years	0.94	935.74	123	Grand Index
20	f	17+ years	0.942	936	124	Grand Index
20	f	17+ years	0.943	936.306	124	Grand Index
20	f	17+ years	0.944	937.248	124	Grand Index
20	f	17+ years	0.945	938.0030551	124	Grand Index
20	f	17+ years	0.946	938.0182022	124	Grand Index
20	f	17+ years	0.947	938.1789695	124	Grand Index
20	f	17+ years	0.948	940.0478224	124	Grand Index
20	f	17+ years	0.949	941.9166753	125	Grand Index
20	f	17+ years	0.95	942.9	125	Grand Index
20	f	17+ years	0.951	943.842	125	Grand Index
20	f	17+ years	0.953	944	125	Grand Index
20	f	17+ years	0.954	944.334	125	Grand Index
20	f	17+ years	0.955	944.805	125	Grand Index
20	f	17+ years	0.956	945.0076828	126	Grand Index
20	f	17+ years	0.957	945.0207937	126	Grand Index
20	f	17+ years	0.958	945.5465278	126	Grand Index
20	f	17+ years	0.959	946.6671869	126	Grand Index
20	f	17+ years	0.96	947.8220102	126	Grand Index
20	f	17+ years	0.961	949.0432402	126	Grand Index
20	f	17+ years	0.962	950.306	127	Grand Index
20	f	17+ years	0.963	951.719	127	Grand Index
20	f	17+ years	0.964	953.0247032	127	Grand Index
20	f	17+ years	0.965	953.2891399	127	Grand Index
20	f	17+ years	0.966	953.5535766	127	Grand Index
20	f	17+ years	0.967	953.7618601	128	Grand Index
20	f	17+ years	0.968	953.9684234	128	Grand Index
20	f	17+ years	0.969	955.596	128	Grand Index
20	f	17+ years	0.97	957.48	128	Grand Index
20	f	17+ years	0.972	958	129	Grand Index
20	f	17+ years	0.973	958.8285422	129	Grand Index
20	f	17+ years	0.974	960.207494	129	Grand Index
20	f	17+ years	0.975	962.9689759	129	Grand Index
20	f	17+ years	0.976	967.2420241	130	Grand Index
20	f	17+ years	0.977	970.3402935	130	Grand Index
20	f	17+ years	0.978	971.3000436	130	Grand Index
20	f	17+ years	0.979	972.4695781	131	Grand Index
20	f	17+ years	0.98	974.3358281	131	Grand Index
20	f	17+ years	0.981	976.204	131	Grand Index
20	f	17+ years	0.982	978.088	131	Grand Index

Age	Gender	Education	Percentile	Raw Score	Norm Score	Assessment
20	f	17+ years	0.983	979.972	132	Grand Index
20	f	17+ years	0.984	981.392	132	Grand Index
20	f	17+ years	0.985	982.805	133	Grand Index
20	f	17+ years	0.986	987.2879433	133	Grand Index
20	f	17+ years	0.987	992.26238	133	Grand Index
20	f	17+ years	0.988	994.0646275	134	Grand Index
20	f	17+ years	0.989	994.7456702	134	Grand Index
20	f	17+ years	0.99	995.7385692	135	Grand Index
20	f	17+ years	0.991	996.9261096	135	Grand Index
20	f	17+ years	0.992	998.4745222	136	Grand Index
20	f	17+ years	0.993	1000.394695	137	Grand Index
20	f	17+ years	0.994	1003.238454	138	Grand Index
20	f	17+ years	0.995	1007.658676	139	Grand Index
20	f	17+ years	0.996	1017.121885	140	Grand Index
20	f	17+ years	0.997	1042.018372	141	Grand Index
20	f	17+ years	0.998	1063.907633	143	Grand Index
20	f	17+ years	0.999	1064.383373	146	Grand Index
20	m	17+ years	0.001	602.7813939	54	Grand Index
20	m	17+ years	0.002	603.4815354	57	Grand Index
20	m	17+ years	0.003	604.1220823	59	Grand Index
20	m	17+ years	0.004	606.1402046	60	Grand Index
20	m	17+ years	0.005	608.9088033	61	Grand Index
20	m	17+ years	0.006	612.8520378	62	Grand Index
20	m	17+ years	0.007	620.7905658	63	Grand Index
20	m	17+ years	0.008	628.9440671	64	Grand Index
20	m	17+ years	0.009	633.4439617	65	Grand Index
20	m	17+ years	0.01	639.9767103	65	Grand Index
20	m	17+ years	0.011	647.1495714	66	Grand Index
20	m	17+ years	0.012	650.2587269	66	Grand Index
20	m	17+ years	0.013	652.1447195	67	Grand Index
20	m	17+ years	0.014	652.9843068	67	Grand Index
20	m	17+ years	0.015	654.89	67	Grand Index
20	m	17+ years	0.016	656.544	68	Grand Index
20	m	17+ years	0.017	657.828	68	Grand Index
20	m	17+ years	0.018	658	69	Grand Index
20	m	17+ years	0.019	659.188	69	Grand Index
20	m	17+ years	0.02	663.04	69	Grand Index
20	m	17+ years	0.021	664.9149685	69	Grand Index
20	m	17+ years	0.022	666.1588888	70	Grand Index
20	m	17+ years	0.023	667.5081963	70	Grand Index
20	m	17+ years	0.024	668.408	70	Grand Index
20	m	17+ years	0.025	669.0306753	71	Grand Index
20	m	17+ years	0.026	669.4245466	71	Grand Index
20	m	17+ years	0.027	669.6768588	71	Grand Index
20	m	17+ years	0.028	669.7986315	71	Grand Index
20	m	17+ years	0.029	673.6328162	72	Grand Index
20	m	17+ years	0.031	676	72	Grand Index
20	m	17+ years	0.032	677.3108249	72	Grand Index
20	m	17+ years	0.033	678.4836543	72	Grand Index
20	m	17+ years	0.034	678.7392456	73	Grand Index
20	m	17+ years	0.035	681.7180925	73	Grand Index
20	m	17+ years	0.037	685	73	Grand Index
20	m	17+ years	0.038	685.396	73	Grand Index
20	m	17+ years	0.042	686	74	Grand Index
20	m	17+ years	0.043	686.606	74	Grand Index
20	m	17+ years	0.045	687	75	Grand Index

Age	Gender	Education	Percentile	Raw Score	Norm Score	Assessment
20	m	17+ years	0.046	687.532	75	Grand Index
20	m	17+ years	0.048	688	75	Grand Index
20	m	17+ years	0.049	688.8132526	75	Grand Index
20	m	17+ years	0.05	689.859433	75	Grand Index
20	m	17+ years	0.051	690.3972506	75	Grand Index
20	m	17+ years	0.052	690.7618439	76	Grand Index
20	m	17+ years	0.054	691	76	Grand Index
20	m	17+ years	0.055	691.2663347	76	Grand Index
20	m	17+ years	0.056	691.8179052	76	Grand Index
20	m	17+ years	0.057	691.9428125	76	Grand Index
20	m	17+ years	0.058	692.0366118	76	Grand Index
20	m	17+ years	0.059	692.1362085	77	Grand Index
20	m	17+ years	0.06	692.5944648	77	Grand Index
20	m	17+ years	0.061	693.324	77	Grand Index
20	m	17+ years	0.062	694.608	77	Grand Index
20	m	17+ years	0.063	696.338	77	Grand Index
20	m	17+ years	0.064	698.088	77	Grand Index
20	m	17+ years	0.065	698.73	77	Grand Index
20	m	17+ years	0.066	699.013481	77	Grand Index
20	m	17+ years	0.067	699.0391107	78	Grand Index
20	m	17+ years	0.068	699.1707894	78	Grand Index
20	m	17+ years	0.069	699.4490284	78	Grand Index
20	m	17+ years	0.07	699.8964511	78	Grand Index
20	m	17+ years	0.071	699.9741953	78	Grand Index
20	m	17+ years	0.074	700	78	Grand Index
20	m	17+ years	0.075	700.1300388	78	Grand Index
20	m	17+ years	0.076	700.686605	79	Grand Index
20	m	17+ years	0.077	701.3586799	79	Grand Index
20	m	17+ years	0.079	702	79	Grand Index
20	m	17+ years	0.08	702.36	79	Grand Index
20	m	17+ years	0.082	703	79	Grand Index
20	m	17+ years	0.083	703.572	79	Grand Index
20	m	17+ years	0.084	704.856	79	Grand Index
20	m	17+ years	0.085	706.0609396	79	Grand Index
20	m	17+ years	0.086	706.8907025	80	Grand Index
20	m	17+ years	0.087	706.9797494	80	Grand Index
20	m	17+ years	0.088	707.992	80	Grand Index
20	m	17+ years	0.089	709.138	80	Grand Index
20	m	17+ years	0.09	709.78	80	Grand Index
20	m	17+ years	0.091	710.8119407	80	Grand Index
20	m	17+ years	0.092	711.9288922	80	Grand Index
20	m	17+ years	0.093	711.9776648	80	Grand Index
20	m	17+ years	0.094	712.348	80	Grand Index
20	m	17+ years	0.095	712.99	80	Grand Index
20	m	17+ years	0.098	713	81	Grand Index
20	m	17+ years	0.099	713.558	81	Grand Index
20	m	17+ years	0.101	714	81	Grand Index
20	m	17+ years	0.102	714.484	81	Grand Index
20	m	17+ years	0.109	715	82	Grand Index
20	m	17+ years	0.11	715.62	82	Grand Index
20	m	17+ years	0.112	716	82	Grand Index
20	m	17+ years	0.113	716.0077143	82	Grand Index
20	m	17+ years	0.114	716.1994725	82	Grand Index
20	m	17+ years	0.115	716.8324019	82	Grand Index
20	m	17+ years	0.116	717.472	82	Grand Index
20	m	17+ years	0.117	718.114	82	Grand Index

Age	Gender	Education	Percentile	Raw Score	Norm Score	Assessment
20	m	17+ years	0.118	718.756	82	Grand Index
20	m	17+ years	0.119	719.3628334	82	Grand Index
20	m	17+ years	0.12	719.9151761	82	Grand Index
20	m	17+ years	0.121	719.9719021	82	Grand Index
20	m	17+ years	0.123	720	83	Grand Index
20	m	17+ years	0.124	720.608	83	Grand Index
20	m	17+ years	0.125	721.25	83	Grand Index
20	m	17+ years	0.126	721.892	83	Grand Index
20	m	17+ years	0.127	722.534	83	Grand Index
20	m	17+ years	0.128	723.176	83	Grand Index
20	m	17+ years	0.129	723.818	83	Grand Index
20	m	17+ years	0.13	724.92	83	Grand Index
20	m	17+ years	0.133	726	83	Grand Index
20	m	17+ years	0.134	726.028	83	Grand Index
20	m	17+ years	0.135	726.67	83	Grand Index
20	m	17+ years	0.136	727.312	84	Grand Index
20	m	17+ years	0.137	727.954	84	Grand Index
20	m	17+ years	0.138	728	84	Grand Index
20	m	17+ years	0.139	728.238	84	Grand Index
20	m	17+ years	0.14	728.88	84	Grand Index
20	m	17+ years	0.141	730.044	84	Grand Index
20	m	17+ years	0.143	731	84	Grand Index
20	m	17+ years	0.144	731.2657389	84	Grand Index
20	m	17+ years	0.145	731.6297822	84	Grand Index
20	m	17+ years	0.146	731.8909688	84	Grand Index
20	m	17+ years	0.147	732.01676	84	Grand Index
20	m	17+ years	0.148	732.0760957	84	Grand Index
20	m	17+ years	0.149	733.331326	84	Grand Index
20	m	17+ years	0.15	734.3	84	Grand Index
20	m	17+ years	0.151	734.942	85	Grand Index
20	m	17+ years	0.152	735.584	85	Grand Index
20	m	17+ years	0.153	736.226	85	Grand Index
20	m	17+ years	0.154	736.868	85	Grand Index
20	m	17+ years	0.158	737	85	Grand Index
20	m	17+ years	0.159	737.078	85	Grand Index
20	m	17+ years	0.16	737.72	85	Grand Index
20	m	17+ years	0.163	738	85	Grand Index
20	m	17+ years	0.164	738.288	85	Grand Index
20	m	17+ years	0.165	738.93	85	Grand Index
20	m	17+ years	0.168	739	86	Grand Index
20	m	17+ years	0.169	739.498	86	Grand Index
20	m	17+ years	0.171	740	86	Grand Index
20	m	17+ years	0.172	740.424	86	Grand Index
20	m	17+ years	0.173	741.0777458	86	Grand Index
20	m	17+ years	0.174	741.834	86	Grand Index
20	m	17+ years	0.175	742.465678	86	Grand Index
20	m	17+ years	0.176	742.9934237	86	Grand Index
20	m	17+ years	0.177	743.6064662	86	Grand Index
20	m	17+ years	0.178	743.9685577	86	Grand Index
20	m	17+ years	0.179	743.9964389	86	Grand Index
20	m	17+ years	0.18	744.56	86	Grand Index
20	m	17+ years	0.182	745	86	Grand Index
20	m	17+ years	0.183	745.0089933	86	Grand Index
20	m	17+ years	0.184	745.1441362	86	Grand Index
20	m	17+ years	0.185	745.7742561	87	Grand Index
20	m	17+ years	0.188	746	87	Grand Index

Age	Gender	Education	Percentile	Raw Score	Norm Score	Assessment
20	m	17+ years	0.189	746.3283044	87	Grand Index
20	m	17+ years	0.19	746.9518884	87	Grand Index
20	m	17+ years	0.191	746.989157	87	Grand Index
20	m	17+ years	0.192	747.2123199	87	Grand Index
20	m	17+ years	0.193	747.7286434	87	Grand Index
20	m	17+ years	0.194	747.9115175	87	Grand Index
20	m	17+ years	0.195	748.19	87	Grand Index
20	m	17+ years	0.196	748.832	87	Grand Index
20	m	17+ years	0.202	749	87	Grand Index
20	m	17+ years	0.203	749.326	88	Grand Index
20	m	17+ years	0.204	749.968	88	Grand Index
20	m	17+ years	0.205	750.61	88	Grand Index
20	m	17+ years	0.207	751	88	Grand Index
20	m	17+ years	0.208	751.536	88	Grand Index
20	m	17+ years	0.21	752	88	Grand Index
20	m	17+ years	0.211	752.462	88	Grand Index
20	m	17+ years	0.216	753	88	Grand Index
20	m	17+ years	0.217	753.314	88	Grand Index
20	m	17+ years	0.218	753.956	88	Grand Index
20	m	17+ years	0.219	754.598	88	Grand Index
20	m	17+ years	0.221	755	88	Grand Index
20	m	17+ years	0.222	755.524	89	Grand Index
20	m	17+ years	0.224	756	89	Grand Index
20	m	17+ years	0.225	756.45	89	Grand Index
20	m	17+ years	0.233	757	89	Grand Index
20	m	17+ years	0.234	757.228	89	Grand Index
20	m	17+ years	0.235	757.87	89	Grand Index
20	m	17+ years	0.239	758	89	Grand Index
20	m	17+ years	0.24	758.0774114	89	Grand Index
20	m	17+ years	0.241	758.6986379	89	Grand Index
20	m	17+ years	0.242	758.9794206	90	Grand Index
20	m	17+ years	0.246	759	90	Grand Index
20	m	17+ years	0.247	759.574	90	Grand Index
20	m	17+ years	0.249	760	90	Grand Index
20	m	17+ years	0.25	760.5	90	Grand Index
20	m	17+ years	0.251	761.0011241	90	Grand Index
20	m	17+ years	0.252	761.0062064	90	Grand Index
20	m	17+ years	0.253	761.430544	90	Grand Index
20	m	17+ years	0.255	762	90	Grand Index
20	m	17+ years	0.256	762.068531	90	Grand Index
20	m	17+ years	0.257	762.1935221	90	Grand Index
20	m	17+ years	0.258	763.3428673	90	Grand Index
20	m	17+ years	0.26	764	90	Grand Index
20	m	17+ years	0.261	764.562	90	Grand Index
20	m	17+ years	0.266	765	91	Grand Index
20	m	17+ years	0.267	765.414	91	Grand Index
20	m	17+ years	0.269	766	91	Grand Index
20	m	17+ years	0.27	766.34	91	Grand Index
20	m	17+ years	0.271	766.982	91	Grand Index
20	m	17+ years	0.274	767	91	Grand Index
20	m	17+ years	0.275	767.55	91	Grand Index
20	m	17+ years	0.281	768	91	Grand Index
20	m	17+ years	0.282	768.0812308	91	Grand Index
20	m	17+ years	0.283	769.2664615	91	Grand Index
20	m	17+ years	0.284	769.8966154	91	Grand Index
20	m	17+ years	0.285	769.9953846	91	Grand Index

Age	Gender	Education	Percentile	Raw Score	Norm Score	Assessment
20	m	17+ years	0.286	770	92	Grand Index
20	m	17+ years	0.287	770.254	92	Grand Index
20	m	17+ years	0.288	770.896	92	Grand Index
20	m	17+ years	0.289	771	92	Grand Index
20	m	17+ years	0.29	771.18	92	Grand Index
20	m	17+ years	0.291	771.822	92	Grand Index
20	m	17+ years	0.294	772	92	Grand Index
20	m	17+ years	0.295	772.076957	92	Grand Index
20	m	17+ years	0.296	772.2230112	92	Grand Index
20	m	17+ years	0.297	772.7383282	92	Grand Index
20	m	17+ years	0.298	773.316	92	Grand Index
20	m	17+ years	0.299	773.958	92	Grand Index
20	m	17+ years	0.302	774	92	Grand Index
20	m	17+ years	0.303	774.526	92	Grand Index
20	m	17+ years	0.304	775.168	92	Grand Index
20	m	17+ years	0.305	775.81	92	Grand Index
20	m	17+ years	0.309	776	93	Grand Index
20	m	17+ years	0.31	776.02	93	Grand Index
20	m	17+ years	0.311	776.662	93	Grand Index
20	m	17+ years	0.316	777	93	Grand Index
20	m	17+ years	0.317	777.514	93	Grand Index
20	m	17+ years	0.319	778	93	Grand Index
20	m	17+ years	0.32	778.288142	93	Grand Index
20	m	17+ years	0.321	778.6831689	93	Grand Index
20	m	17+ years	0.322	778.9047436	93	Grand Index
20	m	17+ years	0.327	779	93	Grand Index
20	m	17+ years	0.328	779.576	93	Grand Index
20	m	17+ years	0.333	780	94	Grand Index
20	m	17+ years	0.334	780.428	94	Grand Index
20	m	17+ years	0.335	781.07	94	Grand Index
20	m	17+ years	0.336	781.712	94	Grand Index
20	m	17+ years	0.341	782	94	Grand Index
20	m	17+ years	0.342	782.564	94	Grand Index
20	m	17+ years	0.343	783.206	94	Grand Index
20	m	17+ years	0.344	783.848	94	Grand Index
20	m	17+ years	0.345	784	94	Grand Index
20	m	17+ years	0.346	784.0474375	94	Grand Index
20	m	17+ years	0.347	784.2781563	94	Grand Index
20	m	17+ years	0.348	784.625875	94	Grand Index
20	m	17+ years	0.349	785.058	94	Grand Index
20	m	17+ years	0.35	785.7	94	Grand Index
20	m	17+ years	0.353	786	94	Grand Index
20	m	17+ years	0.354	786.268	94	Grand Index
20	m	17+ years	0.355	786.91	94	Grand Index
20	m	17+ years	0.358	787	95	Grand Index
20	m	17+ years	0.359	787.478	95	Grand Index
20	m	17+ years	0.361	788	95	Grand Index
20	m	17+ years	0.362	788.1094312	95	Grand Index
20	m	17+ years	0.363	788.3024355	95	Grand Index
20	m	17+ years	0.364	788.7429911	95	Grand Index
20	m	17+ years	0.365	788.9712522	95	Grand Index
20	m	17+ years	0.366	788.9987986	95	Grand Index
20	m	17+ years	0.37	789	95	Grand Index
20	m	17+ years	0.371	789.546	95	Grand Index
20	m	17+ years	0.372	791.472	95	Grand Index
20	m	17+ years	0.38	792	95	Grand Index

Age	Gender	Education	Percentile	Raw Score	Norm Score	Assessment
20	m	17+ years	0.381	792.602	95	Grand Index
20	m	17+ years	0.386	793	96	Grand Index
20	m	17+ years	0.387	793.454	96	Grand Index
20	m	17+ years	0.395	794	96	Grand Index
20	m	17+ years	0.396	794.232	96	Grand Index
20	m	17+ years	0.397	794.874	96	Grand Index
20	m	17+ years	0.4	795	96	Grand Index
20	m	17+ years	0.401	795.2767855	96	Grand Index
20	m	17+ years	0.402	795.6576097	96	Grand Index
20	m	17+ years	0.403	795.8975819	96	Grand Index
20	m	17+ years	0.408	796	97	Grand Index
20	m	17+ years	0.409	796.578	97	Grand Index
20	m	17+ years	0.412	797	97	Grand Index
20	m	17+ years	0.413	797.146	97	Grand Index
20	m	17+ years	0.414	797.788	97	Grand Index
20	m	17+ years	0.415	798.43	97	Grand Index
20	m	17+ years	0.417	799	97	Grand Index
20	m	17+ years	0.418	799.356	97	Grand Index
20	m	17+ years	0.419	799.998	97	Grand Index
20	m	17+ years	0.426	800	97	Grand Index
20	m	17+ years	0.427	800.0246723	97	Grand Index
20	m	17+ years	0.428	800.1428782	97	Grand Index
20	m	17+ years	0.429	800.5251586	97	Grand Index
20	m	17+ years	0.431	801	97	Grand Index
20	m	17+ years	0.432	801.344	97	Grand Index
20	m	17+ years	0.433	801.986	97	Grand Index
20	m	17+ years	0.439	802	98	Grand Index
20	m	17+ years	0.44	802.48	98	Grand Index
20	m	17+ years	0.443	803	98	Grand Index
20	m	17+ years	0.444	803.048	98	Grand Index
20	m	17+ years	0.445	803.69	98	Grand Index
20	m	17+ years	0.447	804	98	Grand Index
20	m	17+ years	0.448	804.616	98	Grand Index
20	m	17+ years	0.453	805	98	Grand Index
20	m	17+ years	0.454	805.468	98	Grand Index
20	m	17+ years	0.457	806	98	Grand Index
20	m	17+ years	0.458	806.036	98	Grand Index
20	m	17+ years	0.459	806.678	98	Grand Index
20	m	17+ years	0.462	807	99	Grand Index
20	m	17+ years	0.463	807.2372415	99	Grand Index
20	m	17+ years	0.464	807.8563838	99	Grand Index
20	m	17+ years	0.465	807.9689137	99	Grand Index
20	m	17+ years	0.466	807.9775775	99	Grand Index
20	m	17+ years	0.467	807.9949631	99	Grand Index
20	m	17+ years	0.473	808	99	Grand Index
20	m	17+ years	0.474	808.308	99	Grand Index
20	m	17+ years	0.475	808.95	99	Grand Index
20	m	17+ years	0.478	809	99	Grand Index
20	m	17+ years	0.479	809.518	99	Grand Index
20	m	17+ years	0.485	810	99	Grand Index
20	m	17+ years	0.486	810.0018262	99	Grand Index
20	m	17+ years	0.487	810.09953	100	Grand Index
20	m	17+ years	0.488	810.4031393	100	Grand Index
20	m	17+ years	0.489	810.9474356	100	Grand Index
20	m	17+ years	0.49	811.58	100	Grand Index
20	m	17+ years	0.501	812	100	Grand Index

Age	Gender	Education	Percentile	Raw Score	Norm Score	Assessment
20	m	17+ years	0.502	812.284	100	Grand Index
20	m	17+ years	0.503	812.926	100	Grand Index
20	m	17+ years	0.504	813.204247	100	Grand Index
20	m	17+ years	0.505	813.3628106	100	Grand Index
20	m	17+ years	0.506	813.3726569	100	Grand Index
20	m	17+ years	0.507	813.6837129	100	Grand Index
20	m	17+ years	0.509	814	100	Grand Index
20	m	17+ years	0.51	814.42	100	Grand Index
20	m	17+ years	0.514	815	101	Grand Index
20	m	17+ years	0.515	815.0681887	101	Grand Index
20	m	17+ years	0.516	815.3507958	101	Grand Index
20	m	17+ years	0.517	815.9233083	101	Grand Index
20	m	17+ years	0.521	816	101	Grand Index
20	m	17+ years	0.522	816.04108	101	Grand Index
20	m	17+ years	0.523	816.2537683	101	Grand Index
20	m	17+ years	0.524	816.6041238	101	Grand Index
20	m	17+ years	0.528	817	101	Grand Index
20	m	17+ years	0.529	817.618	101	Grand Index
20	m	17+ years	0.53	818.0830649	101	Grand Index
20	m	17+ years	0.531	818.2881714	101	Grand Index
20	m	17+ years	0.532	818.6896831	101	Grand Index
20	m	17+ years	0.537	819	101	Grand Index
20	m	17+ years	0.538	819.0315927	101	Grand Index
20	m	17+ years	0.539	819.1527479	101	Grand Index
20	m	17+ years	0.54	820.3855294	102	Grand Index
20	m	17+ years	0.549	821	102	Grand Index
20	m	17+ years	0.55	821.1	102	Grand Index
20	m	17+ years	0.551	821.742	102	Grand Index
20	m	17+ years	0.552	822	102	Grand Index
20	m	17+ years	0.553	822.026	102	Grand Index
20	m	17+ years	0.554	822.668	102	Grand Index
20	m	17+ years	0.559	823	102	Grand Index
20	m	17+ years	0.56	823.52	102	Grand Index
20	m	17+ years	0.565	824	102	Grand Index
20	m	17+ years	0.566	824.372	102	Grand Index
20	m	17+ years	0.568	825	103	Grand Index
20	m	17+ years	0.569	825.298	103	Grand Index
20	m	17+ years	0.57	825.94	103	Grand Index
20	m	17+ years	0.574	826	103	Grand Index
20	m	17+ years	0.575	826.0320475	103	Grand Index
20	m	17+ years	0.576	826.1692107	103	Grand Index
20	m	17+ years	0.577	826.2584937	103	Grand Index
20	m	17+ years	0.578	826.3688865	103	Grand Index
20	m	17+ years	0.579	826.8073875	103	Grand Index
20	m	17+ years	0.58	827.36	103	Grand Index
20	m	17+ years	0.587	828	103	Grand Index
20	m	17+ years	0.588	828.4788569	103	Grand Index
20	m	17+ years	0.589	828.9702069	103	Grand Index
20	m	17+ years	0.59	828.9923962	103	Grand Index
20	m	17+ years	0.591	829.2636108	103	Grand Index
20	m	17+ years	0.592	829.7126911	103	Grand Index
20	m	17+ years	0.593	830.595653	104	Grand Index
20	m	17+ years	0.598	831	104	Grand Index
20	m	17+ years	0.599	831.4998199	104	Grand Index
20	m	17+ years	0.6	831.9165877	104	Grand Index
20	m	17+ years	0.601	831.9835261	104	Grand Index

Age	Gender	Education	Percentile	Raw Score	Norm Score	Assessment
20	m	17+ years	0.605	832	104	Grand Index
20	m	17+ years	0.606	832.052	104	Grand Index
20	m	17+ years	0.607	832.694	104	Grand Index
20	m	17+ years	0.615	833	104	Grand Index
20	m	17+ years	0.616	833.472	104	Grand Index
20	m	17+ years	0.617	834.114	104	Grand Index
20	m	17+ years	0.618	834.756	105	Grand Index
20	m	17+ years	0.619	835	105	Grand Index
20	m	17+ years	0.62	835.04	105	Grand Index
20	m	17+ years	0.621	835.682	105	Grand Index
20	m	17+ years	0.622	836.0062125	105	Grand Index
20	m	17+ years	0.623	836.0185224	105	Grand Index
20	m	17+ years	0.624	836.6155163	105	Grand Index
20	m	17+ years	0.625	837.2344256	105	Grand Index
20	m	17+ years	0.626	837.8364304	105	Grand Index
20	m	17+ years	0.627	837.9709693	105	Grand Index
20	m	17+ years	0.635	838	105	Grand Index
20	m	17+ years	0.636	838.1570351	105	Grand Index
20	m	17+ years	0.637	838.4801649	105	Grand Index
20	m	17+ years	0.638	838.7993403	105	Grand Index
20	m	17+ years	0.641	839	105	Grand Index
20	m	17+ years	0.642	839.164	105	Grand Index
20	m	17+ years	0.643	839.806	105	Grand Index
20	m	17+ years	0.644	840	106	Grand Index
20	m	17+ years	0.645	840.09	106	Grand Index
20	m	17+ years	0.646	840.732	106	Grand Index
20	m	17+ years	0.649	841	106	Grand Index
20	m	17+ years	0.65	841.3	106	Grand Index
20	m	17+ years	0.651	841.942	106	Grand Index
20	m	17+ years	0.657	842	106	Grand Index
20	m	17+ years	0.658	842.1130773	106	Grand Index
20	m	17+ years	0.659	842.3171222	106	Grand Index
20	m	17+ years	0.66	842.7926185	106	Grand Index
20	m	17+ years	0.668	843	107	Grand Index
20	m	17+ years	0.669	843.1902373	107	Grand Index
20	m	17+ years	0.67	843.4685222	107	Grand Index
20	m	17+ years	0.671	843.8652766	107	Grand Index
20	m	17+ years	0.672	844.4231914	107	Grand Index
20	m	17+ years	0.673	844.9982187	107	Grand Index
20	m	17+ years	0.674	844.9994431	107	Grand Index
20	m	17+ years	0.68	845	107	Grand Index
20	m	17+ years	0.681	845.202	107	Grand Index
20	m	17+ years	0.682	845.844	107	Grand Index
20	m	17+ years	0.683	846	107	Grand Index
20	m	17+ years	0.684	846.128	107	Grand Index
20	m	17+ years	0.685	846.77	107	Grand Index
20	m	17+ years	0.686	847	107	Grand Index
20	m	17+ years	0.687	847.0518472	107	Grand Index
20	m	17+ years	0.688	847.6682523	107	Grand Index
20	m	17+ years	0.689	847.9736077	107	Grand Index
20	m	17+ years	0.69	847.9992027	107	Grand Index
20	m	17+ years	0.691	848.622	107	Grand Index
20	m	17+ years	0.694	849	108	Grand Index
20	m	17+ years	0.695	849.19	108	Grand Index
20	m	17+ years	0.696	849.832	108	Grand Index
20	m	17+ years	0.697	850.1610771	108	Grand Index

Age	Gender	Education	Percentile	Raw Score	Norm Score	Assessment
20	m	17+ years	0.698	850.3777387	108	Grand Index
20	m	17+ years	0.699	850.587571	108	Grand Index
20	m	17+ years	0.7	850.7856704	108	Grand Index
20	m	17+ years	0.701	850.9656487	108	Grand Index
20	m	17+ years	0.702	850.9881592	108	Grand Index
20	m	17+ years	0.703	850.9994872	108	Grand Index
20	m	17+ years	0.704	850.9999757	108	Grand Index
20	m	17+ years	0.705	852.209479	108	Grand Index
20	m	17+ years	0.706	852.9870988	108	Grand Index
20	m	17+ years	0.707	852.9981718	108	Grand Index
20	m	17+ years	0.708	853	108	Grand Index
20	m	17+ years	0.709	853.0223782	108	Grand Index
20	m	17+ years	0.71	853.1030905	108	Grand Index
20	m	17+ years	0.711	853.5296374	108	Grand Index
20	m	17+ years	0.712	854.104	108	Grand Index
20	m	17+ years	0.713	854.746	108	Grand Index
20	m	17+ years	0.714	855.388	108	Grand Index
20	m	17+ years	0.716	856	109	Grand Index
20	m	17+ years	0.717	856.2174218	109	Grand Index
20	m	17+ years	0.718	856.6619594	109	Grand Index
20	m	17+ years	0.719	856.8763553	109	Grand Index
20	m	17+ years	0.72	857.24	109	Grand Index
20	m	17+ years	0.721	857.882	109	Grand Index
20	m	17+ years	0.724	858	109	Grand Index
20	m	17+ years	0.725	858.45	109	Grand Index
20	m	17+ years	0.728	859	109	Grand Index
20	m	17+ years	0.729	859.018	109	Grand Index
20	m	17+ years	0.73	859.66	109	Grand Index
20	m	17+ years	0.732	860	109	Grand Index
20	m	17+ years	0.733	860.586	109	Grand Index
20	m	17+ years	0.735	861	109	Grand Index
20	m	17+ years	0.736	861.512	109	Grand Index
20	m	17+ years	0.741	862	110	Grand Index
20	m	17+ years	0.742	862.33475	110	Grand Index
20	m	17+ years	0.743	862.920125	110	Grand Index
20	m	17+ years	0.744	862.9717143	110	Grand Index
20	m	17+ years	0.752	863	110	Grand Index
20	m	17+ years	0.753	863.426	110	Grand Index
20	m	17+ years	0.754	864.068	110	Grand Index
20	m	17+ years	0.755	864.71	110	Grand Index
20	m	17+ years	0.758	865	110	Grand Index
20	m	17+ years	0.759	865.0594017	111	Grand Index
20	m	17+ years	0.76	865.1965812	111	Grand Index
20	m	17+ years	0.761	865.6555897	111	Grand Index
20	m	17+ years	0.763	866	111	Grand Index
20	m	17+ years	0.764	866.488	111	Grand Index
20	m	17+ years	0.766	867	111	Grand Index
20	m	17+ years	0.767	867.414	111	Grand Index
20	m	17+ years	0.768	868.056	111	Grand Index
20	m	17+ years	0.769	868.698	111	Grand Index
20	m	17+ years	0.772	869	111	Grand Index
20	m	17+ years	0.773	869.266	111	Grand Index
20	m	17+ years	0.774	869.908	111	Grand Index
20	m	17+ years	0.775	870	111	Grand Index
20	m	17+ years	0.776	870.192	111	Grand Index
20	m	17+ years	0.777	870.834	111	Grand Index

Age	Gender	Education	Percentile	Raw Score	Norm Score	Assessment
20	m	17+ years	0.781	871	112	Grand Index
20	m	17+ years	0.782	871.088	112	Grand Index
20	m	17+ years	0.783	872.372	112	Grand Index
20	m	17+ years	0.784	873.328	112	Grand Index
20	m	17+ years	0.785	873.97	112	Grand Index
20	m	17+ years	0.789	874	112	Grand Index
20	m	17+ years	0.79	874.18	112	Grand Index
20	m	17+ years	0.791	874.822	112	Grand Index
20	m	17+ years	0.792	875	112	Grand Index
20	m	17+ years	0.793	875.0588415	112	Grand Index
20	m	17+ years	0.794	875.4152214	112	Grand Index
20	m	17+ years	0.795	875.7286164	112	Grand Index
20	m	17+ years	0.797	876	112	Grand Index
20	m	17+ years	0.798	876.316	113	Grand Index
20	m	17+ years	0.799	876.958	113	Grand Index
20	m	17+ years	0.806	877	113	Grand Index
20	m	17+ years	0.807	877.094	113	Grand Index
20	m	17+ years	0.808	877.736	113	Grand Index
20	m	17+ years	0.809	878	113	Grand Index
20	m	17+ years	0.81	878.0111353	113	Grand Index
20	m	17+ years	0.811	878.3685792	113	Grand Index
20	m	17+ years	0.812	878.6915092	113	Grand Index
20	m	17+ years	0.813	878.9760654	113	Grand Index
20	m	17+ years	0.814	880.1606875	113	Grand Index
20	m	17+ years	0.815	880.9799479	113	Grand Index
20	m	17+ years	0.816	880.9966667	114	Grand Index
20	m	17+ years	0.819	881	114	Grand Index
20	m	17+ years	0.82	881.44	114	Grand Index
20	m	17+ years	0.822	882	114	Grand Index
20	m	17+ years	0.823	882.366	114	Grand Index
20	m	17+ years	0.825	883	114	Grand Index
20	m	17+ years	0.826	883.0882368	114	Grand Index
20	m	17+ years	0.827	883.2822368	114	Grand Index
20	m	17+ years	0.828	883.7041246	114	Grand Index
20	m	17+ years	0.829	884.218	114	Grand Index
20	m	17+ years	0.83	884.86	114	Grand Index
20	m	17+ years	0.831	885.1795474	114	Grand Index
20	m	17+ years	0.832	885.4501606	114	Grand Index
20	m	17+ years	0.833	885.8625401	114	Grand Index
20	m	17+ years	0.834	886	115	Grand Index
20	m	17+ years	0.835	886.07	115	Grand Index
20	m	17+ years	0.836	886.712	115	Grand Index
20	m	17+ years	0.837	887.354	115	Grand Index
20	m	17+ years	0.838	887.996	115	Grand Index
20	m	17+ years	0.839	888.638	115	Grand Index
20	m	17+ years	0.841	889	115	Grand Index
20	m	17+ years	0.842	889.564	115	Grand Index
20	m	17+ years	0.845	890	115	Grand Index
20	m	17+ years	0.846	890.132	115	Grand Index
20	m	17+ years	0.847	890.774	115	Grand Index
20	m	17+ years	0.848	891	115	Grand Index
20	m	17+ years	0.849	891.058	115	Grand Index
20	m	17+ years	0.85	891.7	116	Grand Index
20	m	17+ years	0.853	892	116	Grand Index
20	m	17+ years	0.854	892.268	116	Grand Index
20	m	17+ years	0.855	892.91	116	Grand Index

Age	Gender	Education	Percentile	Raw Score	Norm Score	Assessment
20	m	17+ years	0.856	893.552	116	Grand Index
20	m	17+ years	0.861	894	116	Grand Index
20	m	17+ years	0.862	894.0049423	116	Grand Index
20	m	17+ years	0.863	894.0162491	116	Grand Index
20	m	17+ years	0.864	894.0722927	116	Grand Index
20	m	17+ years	0.865	894.3966843	117	Grand Index
20	m	17+ years	0.866	894.9747868	117	Grand Index
20	m	17+ years	0.867	895.614	117	Grand Index
20	m	17+ years	0.868	896.512	117	Grand Index
20	m	17+ years	0.869	897.796	117	Grand Index
20	m	17+ years	0.87	898.826521	117	Grand Index
20	m	17+ years	0.871	899.7922394	117	Grand Index
20	m	17+ years	0.872	900.7151846	117	Grand Index
20	m	17+ years	0.873	900.9830209	117	Grand Index
20	m	17+ years	0.874	901.0738787	117	Grand Index
20	m	17+ years	0.875	901.5130465	117	Grand Index
20	m	17+ years	0.876	901.8079097	117	Grand Index
20	m	17+ years	0.88	902	118	Grand Index
20	m	17+ years	0.881	902.602	118	Grand Index
20	m	17+ years	0.882	903.0134677	118	Grand Index
20	m	17+ years	0.883	903.0489032	118	Grand Index
20	m	17+ years	0.884	903.465657	118	Grand Index
20	m	17+ years	0.885	903.8610452	118	Grand Index
20	m	17+ years	0.886	903.9685259	118	Grand Index
20	m	17+ years	0.887	904.454	118	Grand Index
20	m	17+ years	0.889	905	118	Grand Index
20	m	17+ years	0.89	905.2775208	118	Grand Index
20	m	17+ years	0.891	905.7362509	118	Grand Index
20	m	17+ years	0.892	905.9093868	119	Grand Index
20	m	17+ years	0.894	906	119	Grand Index
20	m	17+ years	0.895	906.2069138	119	Grand Index
20	m	17+ years	0.896	906.7333387	119	Grand Index
20	m	17+ years	0.897	907.7921884	119	Grand Index
20	m	17+ years	0.9	908	119	Grand Index
20	m	17+ years	0.901	908.2060802	119	Grand Index
20	m	17+ years	0.902	908.5110802	119	Grand Index
20	m	17+ years	0.903	908.8537511	119	Grand Index
20	m	17+ years	0.904	909.368	120	Grand Index
20	m	17+ years	0.905	910.01	120	Grand Index
20	m	17+ years	0.906	910.652	120	Grand Index
20	m	17+ years	0.907	911.0069646	120	Grand Index
20	m	17+ years	0.908	911.0221731	120	Grand Index
20	m	17+ years	0.909	911.5879969	120	Grand Index
20	m	17+ years	0.911	912	120	Grand Index
20	m	17+ years	0.912	912.504	120	Grand Index
20	m	17+ years	0.915	913	121	Grand Index
20	m	17+ years	0.916	913.072	121	Grand Index
20	m	17+ years	0.917	913.714	121	Grand Index
20	m	17+ years	0.918	914.356	121	Grand Index
20	m	17+ years	0.919	914.998	121	Grand Index
20	m	17+ years	0.92	915.1473997	121	Grand Index
20	m	17+ years	0.921	915.447364	121	Grand Index
20	m	17+ years	0.922	915.9415037	121	Grand Index
20	m	17+ years	0.925	916	122	Grand Index
20	m	17+ years	0.926	916.492	122	Grand Index
20	m	17+ years	0.928	917	122	Grand Index

Age	Gender	Education	Percentile	Raw Score	Norm Score	Assessment
20	m	17+ years	0.929	917.418	122	Grand Index
20	m	17+ years	0.931	918	122	Grand Index
20	m	17+ years	0.932	919.032	122	Grand Index
20	m	17+ years	0.933	920.958	122	Grand Index
20	m	17+ years	0.934	921.628	123	Grand Index
20	m	17+ years	0.936	922	123	Grand Index
20	m	17+ years	0.937	922.554	123	Grand Index
20	m	17+ years	0.939	923	123	Grand Index
20	m	17+ years	0.94	923.96	123	Grand Index
20	m	17+ years	0.941	925.244	123	Grand Index
20	m	17+ years	0.942	926.528	124	Grand Index
20	m	17+ years	0.943	927.240045	124	Grand Index
20	m	17+ years	0.944	927.6588641	124	Grand Index
20	m	17+ years	0.945	928.5632856	124	Grand Index
20	m	17+ years	0.946	929.664	124	Grand Index
20	m	17+ years	0.947	930.948	124	Grand Index
20	m	17+ years	0.948	931	124	Grand Index
20	m	17+ years	0.949	931.516	125	Grand Index
20	m	17+ years	0.95	932.8	125	Grand Index
20	m	17+ years	0.951	933.542	125	Grand Index
20	m	17+ years	0.952	934.7065918	125	Grand Index
20	m	17+ years	0.953	937.1719827	125	Grand Index
20	m	17+ years	0.954	938.8977328	125	Grand Index
20	m	17+ years	0.955	940.3387363	125	Grand Index
20	m	17+ years	0.956	941.7325761	126	Grand Index
20	m	17+ years	0.957	942.5818175	126	Grand Index
20	m	17+ years	0.958	943.1297118	126	Grand Index
20	m	17+ years	0.959	944.3752772	126	Grand Index
20	m	17+ years	0.96	946.28	126	Grand Index
20	m	17+ years	0.961	948.848	126	Grand Index
20	m	17+ years	0.962	949.604	127	Grand Index
20	m	17+ years	0.963	950.246	127	Grand Index
20	m	17+ years	0.964	950.888	127	Grand Index
20	m	17+ years	0.965	951.4450288	127	Grand Index
20	m	17+ years	0.966	951.8672526	127	Grand Index
20	m	17+ years	0.967	951.9701799	128	Grand Index
20	m	17+ years	0.968	952.456	128	Grand Index
20	m	17+ years	0.969	953.098	128	Grand Index
20	m	17+ years	0.97	953.74	128	Grand Index
20	m	17+ years	0.971	954.4843886	128	Grand Index
20	m	17+ years	0.972	955.2800662	129	Grand Index
20	m	17+ years	0.973	955.6019548	129	Grand Index
20	m	17+ years	0.974	955.8404366	129	Grand Index
20	m	17+ years	0.975	955.9884709	129	Grand Index
20	m	17+ years	0.976	959.552	130	Grand Index
20	m	17+ years	0.978	962	130	Grand Index
20	m	17+ years	0.979	962.5749875	131	Grand Index
20	m	17+ years	0.98	963.1786426	131	Grand Index
20	m	17+ years	0.981	963.4540134	131	Grand Index
20	m	17+ years	0.982	965.5196511	131	Grand Index
20	m	17+ years	0.983	968.516	132	Grand Index
20	m	17+ years	0.984	972.368	132	Grand Index
20	m	17+ years	0.985	974.1532401	133	Grand Index
20	m	17+ years	0.986	974.4331924	133	Grand Index
20	m	17+ years	0.987	975.4513002	133	Grand Index
20	m	17+ years	0.988	980.2879478	134	Grand Index

Age	Gender	Education	Percentile	Raw Score	Norm Score	Assessment
20	m	17+ years	0.989	989.5881588	134	Grand Index
20	m	17+ years	0.99	996.2772017	135	Grand Index
20	m	17+ years	0.991	1001.254131	135	Grand Index
20	m	17+ years	0.992	1003.520002	136	Grand Index
20	m	17+ years	0.993	1004	137	Grand Index
20	m	17+ years	0.994	1004.267292	138	Grand Index
20	m	17+ years	0.995	1005.426763	139	Grand Index
20	m	17+ years	0.996	1014.26844	140	Grand Index
20	m	17+ years	0.997	1026.549718	141	Grand Index
20	m	17+ years	0.998	1036.568164	143	Grand Index
20	m	17+ years	0.999	1045.186951	146	Grand Index
25	f	0-12 years	0.001	598.0003625	54	Grand Index
25	f	0-12 years	0.002	604.0007251	57	Grand Index
25	f	0-12 years	0.003	610.0010876	59	Grand Index
25	f	0-12 years	0.004	613.3480649	60	Grand Index
25	f	0-12 years	0.005	615.2301524	61	Grand Index
25	f	0-12 years	0.006	617.1122399	62	Grand Index
25	f	0-12 years	0.007	618.8423736	63	Grand Index
25	f	0-12 years	0.008	620.1979235	64	Grand Index
25	f	0-12 years	0.009	621.5534735	65	Grand Index
25	f	0-12 years	0.01	622.9090235	65	Grand Index
25	f	0-12 years	0.011	624.1844029	66	Grand Index
25	f	0-12 years	0.012	625.4540147	66	Grand Index
25	f	0-12 years	0.013	626.7236265	67	Grand Index
25	f	0-12 years	0.014	628.0756462	67	Grand Index
25	f	0-12 years	0.015	629.4880344	67	Grand Index
25	f	0-12 years	0.016	630.9004226	68	Grand Index
25	f	0-12 years	0.017	632.132	68	Grand Index
25	f	0-12 years	0.018	632.728	69	Grand Index
25	f	0-12 years	0.019	633.324	69	Grand Index
25	f	0-12 years	0.02	633.92	69	Grand Index
25	f	0-12 years	0.021	637.096	69	Grand Index
25	f	0-12 years	0.022	640.672	70	Grand Index
25	f	0-12 years	0.023	644.248	70	Grand Index
25	f	0-12 years	0.024	648.888	70	Grand Index
25	f	0-12 years	0.025	654.55	71	Grand Index
25	f	0-12 years	0.026	660.212	71	Grand Index
25	f	0-12 years	0.027	665.175904	71	Grand Index
25	f	0-12 years	0.028	666.3154559	71	Grand Index
25	f	0-12 years	0.029	667.4550078	72	Grand Index
25	f	0-12 years	0.03	668.5945597	72	Grand Index
25	f	0-12 years	0.031	668.8658878	72	Grand Index
25	f	0-12 years	0.032	668.9183359	72	Grand Index
25	f	0-12 years	0.033	668.9707839	72	Grand Index
25	f	0-12 years	0.034	669.528	73	Grand Index
25	f	0-12 years	0.035	670.72	73	Grand Index
25	f	0-12 years	0.036	671.912	73	Grand Index
25	f	0-12 years	0.037	673.104	73	Grand Index
25	f	0-12 years	0.038	674.296	73	Grand Index
25	f	0-12 years	0.039	675.488	74	Grand Index
25	f	0-12 years	0.04	676.68	74	Grand Index
25	f	0-12 years	0.041	679.2757642	74	Grand Index
25	f	0-12 years	0.042	682.3866713	74	Grand Index
25	f	0-12 years	0.043	685.4975784	74	Grand Index
25	f	0-12 years	0.044	687.5020855	74	Grand Index
25	f	0-12 years	0.045	687.6691784	75	Grand Index

Age	Gender	Education	Percentile	Raw Score	Norm Score	Assessment
25	f	0-12 years	0.046	687.8362714	75	Grand Index
25	f	0-12 years	0.047	688.012	75	Grand Index
25	f	0-12 years	0.048	688.608	75	Grand Index
25	f	0-12 years	0.049	689.204	75	Grand Index
25	f	0-12 years	0.05	689.8	75	Grand Index
25	f	0-12 years	0.051	690.198	75	Grand Index
25	f	0-12 years	0.052	690.496	76	Grand Index
25	f	0-12 years	0.053	690.794	76	Grand Index
25	f	0-12 years	0.054	691.46	76	Grand Index
25	f	0-12 years	0.055	692.95	76	Grand Index
25	f	0-12 years	0.056	694.44	76	Grand Index
25	f	0-12 years	0.057	695.93	76	Grand Index
25	f	0-12 years	0.058	697.136	76	Grand Index
25	f	0-12 years	0.059	698.328	77	Grand Index
25	f	0-12 years	0.06	699.52	77	Grand Index
25	f	0-12 years	0.061	700.178	77	Grand Index
25	f	0-12 years	0.062	700.476	77	Grand Index
25	f	0-12 years	0.063	700.774	77	Grand Index
25	f	0-12 years	0.064	701.144	77	Grand Index
25	f	0-12 years	0.065	701.74	77	Grand Index
25	f	0-12 years	0.066	702.336	77	Grand Index
25	f	0-12 years	0.067	702.932	78	Grand Index
25	f	0-12 years	0.068	703.264	78	Grand Index
25	f	0-12 years	0.069	703.562	78	Grand Index
25	f	0-12 years	0.07	703.86	78	Grand Index
25	f	0-12 years	0.071	704.158	78	Grand Index
25	f	0-12 years	0.072	704.456	78	Grand Index
25	f	0-12 years	0.073	704.754	78	Grand Index
25	f	0-12 years	0.077	705	79	Grand Index
25	f	0-12 years	0.078	705.244	79	Grand Index
25	f	0-12 years	0.079	705.542	79	Grand Index
25	f	0-12 years	0.08	705.84	79	Grand Index
25	f	0-12 years	0.083	706	79	Grand Index
25	f	0-12 years	0.084	706.0824715	79	Grand Index
25	f	0-12 years	0.085	706.8504878	79	Grand Index
25	f	0-12 years	0.086	707.6185041	80	Grand Index
25	f	0-12 years	0.087	708.3865203	80	Grand Index
25	f	0-12 years	0.088	710.015935	80	Grand Index
25	f	0-12 years	0.089	711.9299187	80	Grand Index
25	f	0-12 years	0.09	713.8439024	80	Grand Index
25	f	0-12 years	0.091	715.118	80	Grand Index
25	f	0-12 years	0.092	715.416	80	Grand Index
25	f	0-12 years	0.093	715.714	80	Grand Index
25	f	0-12 years	0.097	716	81	Grand Index
25	f	0-12 years	0.098	716.204	81	Grand Index
25	f	0-12 years	0.099	716.502	81	Grand Index
25	f	0-12 years	0.1	716.8	81	Grand Index
25	f	0-12 years	0.101	717.098	81	Grand Index
25	f	0-12 years	0.102	717.396	81	Grand Index
25	f	0-12 years	0.103	717.694	81	Grand Index
25	f	0-12 years	0.104	717.992	81	Grand Index
25	f	0-12 years	0.105	718.58	81	Grand Index
25	f	0-12 years	0.106	719.176	81	Grand Index
25	f	0-12 years	0.107	719.772	81	Grand Index
25	f	0-12 years	0.108	720.6933611	81	Grand Index
25	f	0-12 years	0.109	721.8163046	82	Grand Index

Age	Gender	Education	Percentile	Raw Score	Norm Score	Assessment
25	f	0-12 years	0.11	722.9392481	82	Grand Index
25	f	0-12 years	0.111	723.7863419	82	Grand Index
25	f	0-12 years	0.112	723.8553984	82	Grand Index
25	f	0-12 years	0.113	723.924455	82	Grand Index
25	f	0-12 years	0.114	723.9935115	82	Grand Index
25	f	0-12 years	0.115	724.27	82	Grand Index
25	f	0-12 years	0.116	724.568	82	Grand Index
25	f	0-12 years	0.117	724.866	82	Grand Index
25	f	0-12 years	0.12	725	82	Grand Index
25	f	0-12 years	0.121	725.058	82	Grand Index
25	f	0-12 years	0.122	725.356	83	Grand Index
25	f	0-12 years	0.123	725.654	83	Grand Index
25	f	0-12 years	0.124	725.952	83	Grand Index
25	f	0-12 years	0.125	726.25	83	Grand Index
25	f	0-12 years	0.126	726.548	83	Grand Index
25	f	0-12 years	0.127	726.846	83	Grand Index
25	f	0-12 years	0.128	727.144	83	Grand Index
25	f	0-12 years	0.129	727.442	83	Grand Index
25	f	0-12 years	0.13	727.74	83	Grand Index
25	f	0-12 years	0.134	728	83	Grand Index
25	f	0-12 years	0.135	728.23	83	Grand Index
25	f	0-12 years	0.136	728.528	84	Grand Index
25	f	0-12 years	0.137	728.826	84	Grand Index
25	f	0-12 years	0.138	729.124	84	Grand Index
25	f	0-12 years	0.139	729.422	84	Grand Index
25	f	0-12 years	0.14	729.72	84	Grand Index
25	f	0-12 years	0.141	730.054	84	Grand Index
25	f	0-12 years	0.142	730.948	84	Grand Index
25	f	0-12 years	0.143	731.842	84	Grand Index
25	f	0-12 years	0.144	732.736	84	Grand Index
25	f	0-12 years	0.147	733	84	Grand Index
25	f	0-12 years	0.148	733.312	84	Grand Index
25	f	0-12 years	0.149	734.206	84	Grand Index
25	f	0-12 years	0.15	735.1	84	Grand Index
25	f	0-12 years	0.151	735.994	85	Grand Index
25	f	0-12 years	0.152	736.296	85	Grand Index
25	f	0-12 years	0.153	736.594	85	Grand Index
25	f	0-12 years	0.154	736.892	85	Grand Index
25	f	0-12 years	0.155	737.19	85	Grand Index
25	f	0-12 years	0.156	737.488	85	Grand Index
25	f	0-12 years	0.157	737.786	85	Grand Index
25	f	0-12 years	0.158	738.084	85	Grand Index
25	f	0-12 years	0.159	738.382	85	Grand Index
25	f	0-12 years	0.16	738.68	85	Grand Index
25	f	0-12 years	0.161	738.978	85	Grand Index
25	f	0-12 years	0.162	739.276	85	Grand Index
25	f	0-12 years	0.163	739.574	85	Grand Index
25	f	0-12 years	0.164	739.872	85	Grand Index
25	f	0-12 years	0.165	740.51	85	Grand Index
25	f	0-12 years	0.166	741.404	85	Grand Index
25	f	0-12 years	0.167	742.298	86	Grand Index
25	f	0-12 years	0.168	743.128	86	Grand Index
25	f	0-12 years	0.169	743.724	86	Grand Index
25	f	0-12 years	0.17	744.32	86	Grand Index
25	f	0-12 years	0.171	744.916	86	Grand Index
25	f	0-12 years	0.172	745.512	86	Grand Index

Age	Gender	Education	Percentile	Raw Score	Norm Score	Assessment
25	f	0-12 years	0.173	746.108	86	Grand Index
25	f	0-12 years	0.174	746.704	86	Grand Index
25	f	0-12 years	0.177	747	86	Grand Index
25	f	0-12 years	0.178	747.0323144	86	Grand Index
25	f	0-12 years	0.179	747.2511708	86	Grand Index
25	f	0-12 years	0.18	747.4700271	86	Grand Index
25	f	0-12 years	0.181	747.6888835	86	Grand Index
25	f	0-12 years	0.182	748.0330949	86	Grand Index
25	f	0-12 years	0.183	748.4102385	86	Grand Index
25	f	0-12 years	0.184	748.7873821	86	Grand Index
25	f	0-12 years	0.187	749	87	Grand Index
25	f	0-12 years	0.188	749.024	87	Grand Index
25	f	0-12 years	0.189	749.322	87	Grand Index
25	f	0-12 years	0.19	749.62	87	Grand Index
25	f	0-12 years	0.191	749.918	87	Grand Index
25	f	0-12 years	0.194	750	87	Grand Index
25	f	0-12 years	0.195	750.22	87	Grand Index
25	f	0-12 years	0.196	750.816	87	Grand Index
25	f	0-12 years	0.197	751.412	87	Grand Index
25	f	0-12 years	0.198	752.008	87	Grand Index
25	f	0-12 years	0.199	752.604	87	Grand Index
25	f	0-12 years	0.2	753.2	87	Grand Index
25	f	0-12 years	0.201	753.796	87	Grand Index
25	f	0-12 years	0.202	754.196	87	Grand Index
25	f	0-12 years	0.203	754.494	88	Grand Index
25	f	0-12 years	0.204	754.792	88	Grand Index
25	f	0-12 years	0.205	755.0794896	88	Grand Index
25	f	0-12 years	0.206	755.3426885	88	Grand Index
25	f	0-12 years	0.207	755.6058875	88	Grand Index
25	f	0-12 years	0.208	755.8690864	88	Grand Index
25	f	0-12 years	0.209	755.9551166	88	Grand Index
25	f	0-12 years	0.21	756.0310947	88	Grand Index
25	f	0-12 years	0.211	756.1070728	88	Grand Index
25	f	0-12 years	0.212	756.5705944	88	Grand Index
25	f	0-12 years	0.213	757.3027542	88	Grand Index
25	f	0-12 years	0.214	758.0349141	88	Grand Index
25	f	0-12 years	0.215	758.6234337	88	Grand Index
25	f	0-12 years	0.216	758.7440969	88	Grand Index
25	f	0-12 years	0.217	758.8647601	88	Grand Index
25	f	0-12 years	0.218	758.9854232	88	Grand Index
25	f	0-12 years	0.221	759	88	Grand Index
25	f	0-12 years	0.222	759.156	89	Grand Index
25	f	0-12 years	0.223	759.454	89	Grand Index
25	f	0-12 years	0.224	759.752	89	Grand Index
25	f	0-12 years	0.234	760	89	Grand Index
25	f	0-12 years	0.235	760.0216096	89	Grand Index
25	f	0-12 years	0.236	760.2362645	89	Grand Index
25	f	0-12 years	0.237	760.4509195	89	Grand Index
25	f	0-12 years	0.238	760.6655745	89	Grand Index
25	f	0-12 years	0.239	760.782408	89	Grand Index
25	f	0-12 years	0.24	760.865753	89	Grand Index
25	f	0-12 years	0.241	760.949098	89	Grand Index
25	f	0-12 years	0.248	761	90	Grand Index
25	f	0-12 years	0.249	761.404	90	Grand Index
25	f	0-12 years	0.25	762	90	Grand Index
25	f	0-12 years	0.251	762.596	90	Grand Index

Age	Gender	Education	Percentile	Raw Score	Norm Score	Assessment
25	f	0-12 years	0.252	763.096	90	Grand Index
25	f	0-12 years	0.253	763.394	90	Grand Index
25	f	0-12 years	0.254	763.692	90	Grand Index
25	f	0-12 years	0.255	763.99	90	Grand Index
25	f	0-12 years	0.256	764.288	90	Grand Index
25	f	0-12 years	0.257	764.586	90	Grand Index
25	f	0-12 years	0.258	764.884	90	Grand Index
25	f	0-12 years	0.259	765.182	90	Grand Index
25	f	0-12 years	0.26	765.48	90	Grand Index
25	f	0-12 years	0.261	765.778	90	Grand Index
25	f	0-12 years	0.262	766.076	90	Grand Index
25	f	0-12 years	0.263	766.374	90	Grand Index
25	f	0-12 years	0.264	766.672	91	Grand Index
25	f	0-12 years	0.265	766.97	91	Grand Index
25	f	0-12 years	0.266	767.1355765	91	Grand Index
25	f	0-12 years	0.267	767.2863294	91	Grand Index
25	f	0-12 years	0.268	767.4370824	91	Grand Index
25	f	0-12 years	0.269	767.7479294	91	Grand Index
25	f	0-12 years	0.27	768.1931765	91	Grand Index
25	f	0-12 years	0.271	768.6384235	91	Grand Index
25	f	0-12 years	0.275	769	91	Grand Index
25	f	0-12 years	0.276	769.0050139	91	Grand Index
25	f	0-12 years	0.277	769.0110387	91	Grand Index
25	f	0-12 years	0.278	769.0170635	91	Grand Index
25	f	0-12 years	0.279	769.0665389	91	Grand Index
25	f	0-12 years	0.28	769.1637486	91	Grand Index
25	f	0-12 years	0.281	769.2609583	91	Grand Index
25	f	0-12 years	0.282	769.3699533	91	Grand Index
25	f	0-12 years	0.283	769.5647188	91	Grand Index
25	f	0-12 years	0.284	769.7594843	91	Grand Index
25	f	0-12 years	0.285	769.9542497	91	Grand Index
25	f	0-12 years	0.286	770.456	92	Grand Index
25	f	0-12 years	0.287	771.052	92	Grand Index
25	f	0-12 years	0.288	771.648	92	Grand Index
25	f	0-12 years	0.289	772.122	92	Grand Index
25	f	0-12 years	0.29	772.42	92	Grand Index
25	f	0-12 years	0.291	772.718	92	Grand Index
25	f	0-12 years	0.292	773.016	92	Grand Index
25	f	0-12 years	0.293	773.314	92	Grand Index
25	f	0-12 years	0.294	773.612	92	Grand Index
25	f	0-12 years	0.295	773.91	92	Grand Index
25	f	0-12 years	0.302	774	92	Grand Index
25	f	0-12 years	0.303	774.2615605	92	Grand Index
25	f	0-12 years	0.304	774.5266796	92	Grand Index
25	f	0-12 years	0.305	774.7917987	92	Grand Index
25	f	0-12 years	0.306	774.9012535	92	Grand Index
25	f	0-12 years	0.307	774.9196281	92	Grand Index
25	f	0-12 years	0.308	774.9380027	92	Grand Index
25	f	0-12 years	0.309	774.9553129	93	Grand Index
25	f	0-12 years	0.31	774.9698192	93	Grand Index
25	f	0-12 years	0.311	774.9843255	93	Grand Index
25	f	0-12 years	0.312	774.9988317	93	Grand Index
25	f	0-12 years	0.315	775	93	Grand Index
25	f	0-12 years	0.316	775.168	93	Grand Index
25	f	0-12 years	0.317	775.466	93	Grand Index
25	f	0-12 years	0.318	775.764	93	Grand Index

Age	Gender	Education	Percentile	Raw Score	Norm Score	Assessment
25	f	0-12 years	0.319	776.062	93	Grand Index
25	f	0-12 years	0.32	776.36	93	Grand Index
25	f	0-12 years	0.321	776.658	93	Grand Index
25	f	0-12 years	0.322	776.956	93	Grand Index
25	f	0-12 years	0.323	777.254	93	Grand Index
25	f	0-12 years	0.324	777.552	93	Grand Index
25	f	0-12 years	0.325	777.85	93	Grand Index
25	f	0-12 years	0.326	778.148	93	Grand Index
25	f	0-12 years	0.327	778.446	93	Grand Index
25	f	0-12 years	0.328	778.744	93	Grand Index
25	f	0-12 years	0.329	779.042	93	Grand Index
25	f	0-12 years	0.33	779.34	93	Grand Index
25	f	0-12 years	0.331	779.638	93	Grand Index
25	f	0-12 years	0.332	779.936	93	Grand Index
25	f	0-12 years	0.338	780	94	Grand Index
25	f	0-12 years	0.339	780.044	94	Grand Index
25	f	0-12 years	0.34	780.64	94	Grand Index
25	f	0-12 years	0.341	781.236	94	Grand Index
25	f	0-12 years	0.342	781.832	94	Grand Index
25	f	0-12 years	0.343	782.214	94	Grand Index
25	f	0-12 years	0.344	782.512	94	Grand Index
25	f	0-12 years	0.345	782.81	94	Grand Index
25	f	0-12 years	0.346	783.216	94	Grand Index
25	f	0-12 years	0.347	783.812	94	Grand Index
25	f	0-12 years	0.348	784.408	94	Grand Index
25	f	0-12 years	0.349	785.0006959	94	Grand Index
25	f	0-12 years	0.35	785.1043821	94	Grand Index
25	f	0-12 years	0.351	785.2080682	94	Grand Index
25	f	0-12 years	0.352	785.3117544	94	Grand Index
25	f	0-12 years	0.353	785.6684398	94	Grand Index
25	f	0-12 years	0.354	786.1607536	94	Grand Index
25	f	0-12 years	0.355	786.6530674	94	Grand Index
25	f	0-12 years	0.356	787.088	94	Grand Index
25	f	0-12 years	0.357	787.386	95	Grand Index
25	f	0-12 years	0.358	787.684	95	Grand Index
25	f	0-12 years	0.359	787.982	95	Grand Index
25	f	0-12 years	0.365	788	95	Grand Index
25	f	0-12 years	0.366	788.068	95	Grand Index
25	f	0-12 years	0.367	788.366	95	Grand Index
25	f	0-12 years	0.368	788.664	95	Grand Index
25	f	0-12 years	0.369	788.962	95	Grand Index
25	f	0-12 years	0.372	789	95	Grand Index
25	f	0-12 years	0.373	789.154	95	Grand Index
25	f	0-12 years	0.374	789.452	95	Grand Index
25	f	0-12 years	0.375	789.75	95	Grand Index
25	f	0-12 years	0.379	790	95	Grand Index
25	f	0-12 years	0.38	790.24	95	Grand Index
25	f	0-12 years	0.381	790.538	95	Grand Index
25	f	0-12 years	0.382	790.836	95	Grand Index
25	f	0-12 years	0.383	791.134	96	Grand Index
25	f	0-12 years	0.384	791.432	96	Grand Index
25	f	0-12 years	0.385	791.73	96	Grand Index
25	f	0-12 years	0.392	792	96	Grand Index
25	f	0-12 years	0.393	792.228	96	Grand Index
25	f	0-12 years	0.394	792.824	96	Grand Index
25	f	0-12 years	0.395	793.42	96	Grand Index

Age	Gender	Education	Percentile	Raw Score	Norm Score	Assessment
25	f	0-12 years	0.399	794	96	Grand Index
25	f	0-12 years	0.4	794.1865772	96	Grand Index
25	f	0-12 years	0.401	794.4645772	96	Grand Index
25	f	0-12 years	0.402	794.7425772	96	Grand Index
25	f	0-12 years	0.403	794.9391946	96	Grand Index
25	f	0-12 years	0.404	794.9591946	96	Grand Index
25	f	0-12 years	0.405	794.9791946	96	Grand Index
25	f	0-12 years	0.406	794.9991946	96	Grand Index
25	f	0-12 years	0.409	795	97	Grand Index
25	f	0-12 years	0.41	795.1498776	97	Grand Index
25	f	0-12 years	0.411	795.3980082	97	Grand Index
25	f	0-12 years	0.412	795.6461388	97	Grand Index
25	f	0-12 years	0.413	795.8450367	97	Grand Index
25	f	0-12 years	0.414	795.8949061	97	Grand Index
25	f	0-12 years	0.415	795.9447755	97	Grand Index
25	f	0-12 years	0.416	795.9946449	97	Grand Index
25	f	0-12 years	0.426	796	97	Grand Index
25	f	0-12 years	0.427	796.246	97	Grand Index
25	f	0-12 years	0.428	796.544	97	Grand Index
25	f	0-12 years	0.429	796.842	97	Grand Index
25	f	0-12 years	0.43	797.14	97	Grand Index
25	f	0-12 years	0.431	797.438	97	Grand Index
25	f	0-12 years	0.432	797.736	97	Grand Index
25	f	0-12 years	0.433	798.034	97	Grand Index
25	f	0-12 years	0.434	798.332	98	Grand Index
25	f	0-12 years	0.435	798.63	98	Grand Index
25	f	0-12 years	0.436	798.928	98	Grand Index
25	f	0-12 years	0.446	799	98	Grand Index
25	f	0-12 years	0.447	799.206	98	Grand Index
25	f	0-12 years	0.448	799.504	98	Grand Index
25	f	0-12 years	0.449	799.802	98	Grand Index
25	f	0-12 years	0.45	800.1	98	Grand Index
25	f	0-12 years	0.451	800.398	98	Grand Index
25	f	0-12 years	0.452	800.696	98	Grand Index
25	f	0-12 years	0.453	800.994	98	Grand Index
25	f	0-12 years	0.456	801	98	Grand Index
25	f	0-12 years	0.457	801.186	98	Grand Index
25	f	0-12 years	0.458	801.484	98	Grand Index
25	f	0-12 years	0.459	801.782	98	Grand Index
25	f	0-12 years	0.463	802	99	Grand Index
25	f	0-12 years	0.464	802.816	99	Grand Index
25	f	0-12 years	0.465	803.71	99	Grand Index
25	f	0-12 years	0.466	804.604	99	Grand Index
25	f	0-12 years	0.467	805.166	99	Grand Index
25	f	0-12 years	0.468	805.464	99	Grand Index
25	f	0-12 years	0.469	805.762	99	Grand Index
25	f	0-12 years	0.47	806.12	99	Grand Index
25	f	0-12 years	0.471	806.716	99	Grand Index
25	f	0-12 years	0.472	807.312	99	Grand Index
25	f	0-12 years	0.473	807.908	99	Grand Index
25	f	0-12 years	0.476	808	99	Grand Index
25	f	0-12 years	0.477	808.146	99	Grand Index
25	f	0-12 years	0.478	808.444	99	Grand Index
25	f	0-12 years	0.479	808.742	99	Grand Index
25	f	0-12 years	0.48	809.0363686	99	Grand Index
25	f	0-12 years	0.481	809.3073144	99	Grand Index

Age	Gender	Education	Percentile	Raw Score	Norm Score	Assessment
25	f	0-12 years	0.482	809.5782603	99	Grand Index
25	f	0-12 years	0.483	809.8492061	99	Grand Index
25	f	0-12 years	0.484	809.9302766	99	Grand Index
25	f	0-12 years	0.485	809.9573307	99	Grand Index
25	f	0-12 years	0.486	809.9843849	99	Grand Index
25	f	0-12 years	0.493	810	100	Grand Index
25	f	0-12 years	0.494	810.424	100	Grand Index
25	f	0-12 years	0.495	811.02	100	Grand Index
25	f	0-12 years	0.496	811.616	100	Grand Index
25	f	0-12 years	0.5	812	100	Grand Index
25	f	0-12 years	0.501	812.2335973	100	Grand Index
25	f	0-12 years	0.502	812.4671945	100	Grand Index
25	f	0-12 years	0.503	812.7007918	100	Grand Index
25	f	0-12 years	0.504	812.8253778	100	Grand Index
25	f	0-12 years	0.505	812.8897806	100	Grand Index
25	f	0-12 years	0.506	812.9541833	100	Grand Index
25	f	0-12 years	0.51	813	100	Grand Index
25	f	0-12 years	0.511	813.0457226	100	Grand Index
25	f	0-12 years	0.512	813.0947347	100	Grand Index
25	f	0-12 years	0.513	813.1437467	100	Grand Index
25	f	0-12 years	0.514	813.3081811	101	Grand Index
25	f	0-12 years	0.515	813.5571691	101	Grand Index
25	f	0-12 years	0.516	813.806157	101	Grand Index
25	f	0-12 years	0.517	814.066	101	Grand Index
25	f	0-12 years	0.518	814.364	101	Grand Index
25	f	0-12 years	0.519	814.662	101	Grand Index
25	f	0-12 years	0.52	814.96	101	Grand Index
25	f	0-12 years	0.53	815	101	Grand Index
25	f	0-12 years	0.531	815.0896444	101	Grand Index
25	f	0-12 years	0.532	815.2018882	101	Grand Index
25	f	0-12 years	0.533	815.3141321	101	Grand Index
25	f	0-12 years	0.534	815.5909384	101	Grand Index
25	f	0-12 years	0.535	816.0746946	101	Grand Index
25	f	0-12 years	0.536	816.5584507	101	Grand Index
25	f	0-12 years	0.537	817.0202939	101	Grand Index
25	f	0-12 years	0.538	817.252893	101	Grand Index
25	f	0-12 years	0.539	817.4854922	101	Grand Index
25	f	0-12 years	0.54	817.7180914	102	Grand Index
25	f	0-12 years	0.541	817.8283777	102	Grand Index
25	f	0-12 years	0.542	817.8937785	102	Grand Index
25	f	0-12 years	0.543	817.9591793	102	Grand Index
25	f	0-12 years	0.544	818.224	102	Grand Index
25	f	0-12 years	0.545	818.82	102	Grand Index
25	f	0-12 years	0.546	819.416	102	Grand Index
25	f	0-12 years	0.56	820	102	Grand Index
25	f	0-12 years	0.561	820.178	102	Grand Index
25	f	0-12 years	0.562	820.476	102	Grand Index
25	f	0-12 years	0.563	820.774	102	Grand Index
25	f	0-12 years	0.567	821	103	Grand Index
25	f	0-12 years	0.568	821.264	103	Grand Index
25	f	0-12 years	0.569	821.562	103	Grand Index
25	f	0-12 years	0.57	821.86	103	Grand Index
25	f	0-12 years	0.571	822.158	103	Grand Index
25	f	0-12 years	0.572	822.456	103	Grand Index
25	f	0-12 years	0.573	822.754	103	Grand Index
25	f	0-12 years	0.574	823.0302677	103	Grand Index

Age	Gender	Education	Percentile	Raw Score	Norm Score	Assessment
25	f	0-12 years	0.575	823.2037251	103	Grand Index
25	f	0-12 years	0.576	823.3771825	103	Grand Index
25	f	0-12 years	0.577	823.5506399	103	Grand Index
25	f	0-12 years	0.578	823.6840463	103	Grand Index
25	f	0-12 years	0.579	823.8085889	103	Grand Index
25	f	0-12 years	0.58	823.9331315	103	Grand Index
25	f	0-12 years	0.581	824.276	103	Grand Index
25	f	0-12 years	0.582	824.872	103	Grand Index
25	f	0-12 years	0.583	825.468	103	Grand Index
25	f	0-12 years	0.587	826	103	Grand Index
25	f	0-12 years	0.588	826.224	103	Grand Index
25	f	0-12 years	0.589	826.522	103	Grand Index
25	f	0-12 years	0.59	826.82	103	Grand Index
25	f	0-12 years	0.591	827.118	103	Grand Index
25	f	0-12 years	0.592	827.416	103	Grand Index
25	f	0-12 years	0.593	827.714	104	Grand Index
25	f	0-12 years	0.594	828.012	104	Grand Index
25	f	0-12 years	0.595	828.31	104	Grand Index
25	f	0-12 years	0.596	828.608	104	Grand Index
25	f	0-12 years	0.597	828.906	104	Grand Index
25	f	0-12 years	0.6	829	104	Grand Index
25	f	0-12 years	0.601	829.0321563	104	Grand Index
25	f	0-12 years	0.602	829.1299375	104	Grand Index
25	f	0-12 years	0.603	829.2277188	104	Grand Index
25	f	0-12 years	0.604	829.3255	104	Grand Index
25	f	0-12 years	0.605	830.1029688	104	Grand Index
25	f	0-12 years	0.606	830.8991875	104	Grand Index
25	f	0-12 years	0.607	831.6954063	104	Grand Index
25	f	0-12 years	0.614	832	104	Grand Index
25	f	0-12 years	0.615	832.0211337	104	Grand Index
25	f	0-12 years	0.616	832.044459	104	Grand Index
25	f	0-12 years	0.617	832.0677843	104	Grand Index
25	f	0-12 years	0.618	832.2294361	105	Grand Index
25	f	0-12 years	0.619	832.5041108	105	Grand Index
25	f	0-12 years	0.62	832.7787855	105	Grand Index
25	f	0-12 years	0.624	833	105	Grand Index
25	f	0-12 years	0.625	833.5	105	Grand Index
25	f	0-12 years	0.626	834.096	105	Grand Index
25	f	0-12 years	0.627	834.692	105	Grand Index
25	f	0-12 years	0.63	835	105	Grand Index
25	f	0-12 years	0.631	835.0622187	105	Grand Index
25	f	0-12 years	0.632	835.5501446	105	Grand Index
25	f	0-12 years	0.633	836.0380705	105	Grand Index
25	f	0-12 years	0.634	836.5259964	105	Grand Index
25	f	0-12 years	0.635	836.7207481	105	Grand Index
25	f	0-12 years	0.636	836.8288222	105	Grand Index
25	f	0-12 years	0.637	836.9368963	105	Grand Index
25	f	0-12 years	0.644	837	106	Grand Index
25	f	0-12 years	0.645	837.63	106	Grand Index
25	f	0-12 years	0.646	838.524	106	Grand Index
25	f	0-12 years	0.647	839.418	106	Grand Index
25	f	0-12 years	0.648	840.208	106	Grand Index
25	f	0-12 years	0.649	840.804	106	Grand Index
25	f	0-12 years	0.65	841.4	106	Grand Index
25	f	0-12 years	0.651	841.996	106	Grand Index
25	f	0-12 years	0.652	842.1505425	106	Grand Index

Age	Gender	Education	Percentile	Raw Score	Norm Score	Assessment
25	f	0-12 years	0.653	842.3021023	106	Grand Index
25	f	0-12 years	0.654	842.453662	106	Grand Index
25	f	0-12 years	0.655	842.6019577	106	Grand Index
25	f	0-12 years	0.656	842.7483979	106	Grand Index
25	f	0-12 years	0.657	842.8948382	106	Grand Index
25	f	0-12 years	0.658	843.084	106	Grand Index
25	f	0-12 years	0.659	843.382	106	Grand Index
25	f	0-12 years	0.66	843.68	106	Grand Index
25	f	0-12 years	0.661	843.978	106	Grand Index
25	f	0-12 years	0.664	844	106	Grand Index
25	f	0-12 years	0.665	844.34	106	Grand Index
25	f	0-12 years	0.666	844.936	106	Grand Index
25	f	0-12 years	0.667	845.532	106	Grand Index
25	f	0-12 years	0.668	846.128	107	Grand Index
25	f	0-12 years	0.669	846.724	107	Grand Index
25	f	0-12 years	0.67	847.32	107	Grand Index
25	f	0-12 years	0.671	847.916	107	Grand Index
25	f	0-12 years	0.674	848	107	Grand Index
25	f	0-12 years	0.675	848.15	107	Grand Index
25	f	0-12 years	0.676	848.448	107	Grand Index
25	f	0-12 years	0.677	848.746	107	Grand Index
25	f	0-12 years	0.678	849.0378333	107	Grand Index
25	f	0-12 years	0.679	849.2940677	107	Grand Index
25	f	0-12 years	0.68	849.5503021	107	Grand Index
25	f	0-12 years	0.681	849.8065366	107	Grand Index
25	f	0-12 years	0.682	849.8929232	107	Grand Index
25	f	0-12 years	0.683	849.9346887	107	Grand Index
25	f	0-12 years	0.684	849.9764543	107	Grand Index
25	f	0-12 years	0.685	850.13	107	Grand Index
25	f	0-12 years	0.686	850.428	107	Grand Index
25	f	0-12 years	0.687	850.726	107	Grand Index
25	f	0-12 years	0.688	851.0181395	107	Grand Index
25	f	0-12 years	0.689	851.2433721	107	Grand Index
25	f	0-12 years	0.69	851.4686047	107	Grand Index
25	f	0-12 years	0.691	851.6938372	107	Grand Index
25	f	0-12 years	0.692	851.8085581	108	Grand Index
25	f	0-12 years	0.693	851.8813256	108	Grand Index
25	f	0-12 years	0.694	851.954093	108	Grand Index
25	f	0-12 years	0.701	852	108	Grand Index
25	f	0-12 years	0.702	852.196	108	Grand Index
25	f	0-12 years	0.703	852.494	108	Grand Index
25	f	0-12 years	0.704	852.792	108	Grand Index
25	f	0-12 years	0.705	853.09	108	Grand Index
25	f	0-12 years	0.706	853.388	108	Grand Index
25	f	0-12 years	0.707	853.686	108	Grand Index
25	f	0-12 years	0.708	853.984	108	Grand Index
25	f	0-12 years	0.709	854.282	108	Grand Index
25	f	0-12 years	0.71	854.58	108	Grand Index
25	f	0-12 years	0.711	854.878	108	Grand Index
25	f	0-12 years	0.718	855	109	Grand Index
25	f	0-12 years	0.719	855.262	109	Grand Index
25	f	0-12 years	0.72	855.56	109	Grand Index
25	f	0-12 years	0.721	855.858	109	Grand Index
25	f	0-12 years	0.722	856.2338005	109	Grand Index
25	f	0-12 years	0.723	856.6804194	109	Grand Index
25	f	0-12 years	0.724	857.1270384	109	Grand Index

Age	Gender	Education	Percentile	Raw Score	Norm Score	Assessment
25	f	0-12 years	0.725	857.5237852	109	Grand Index
25	f	0-12 years	0.726	857.6731662	109	Grand Index
25	f	0-12 years	0.727	857.8225473	109	Grand Index
25	f	0-12 years	0.728	857.9719284	109	Grand Index
25	f	0-12 years	0.731	858	109	Grand Index
25	f	0-12 years	0.732	858.0723251	109	Grand Index
25	f	0-12 years	0.733	858.2308021	109	Grand Index
25	f	0-12 years	0.734	858.3892792	109	Grand Index
25	f	0-12 years	0.735	858.5458481	109	Grand Index
25	f	0-12 years	0.736	858.685371	109	Grand Index
25	f	0-12 years	0.737	858.824894	110	Grand Index
25	f	0-12 years	0.738	858.964417	110	Grand Index
25	f	0-12 years	0.739	859.0703558	110	Grand Index
25	f	0-12 years	0.74	859.1647975	110	Grand Index
25	f	0-12 years	0.741	859.2592391	110	Grand Index
25	f	0-12 years	0.742	859.5121557	110	Grand Index
25	f	0-12 years	0.743	860.0137141	110	Grand Index
25	f	0-12 years	0.744	860.5152724	110	Grand Index
25	f	0-12 years	0.745	861.0143752	110	Grand Index
25	f	0-12 years	0.746	861.4427574	110	Grand Index
25	f	0-12 years	0.747	861.8711395	110	Grand Index
25	f	0-12 years	0.748	862.2995217	110	Grand Index
25	f	0-12 years	0.749	862.7531442	110	Grand Index
25	f	0-12 years	0.75	863.218762	110	Grand Index
25	f	0-12 years	0.751	863.6843798	110	Grand Index
25	f	0-12 years	0.752	864.0452624	110	Grand Index
25	f	0-12 years	0.753	864.1857645	110	Grand Index
25	f	0-12 years	0.754	864.3262666	110	Grand Index
25	f	0-12 years	0.755	864.4667687	110	Grand Index
25	f	0-12 years	0.756	864.6236963	110	Grand Index
25	f	0-12 years	0.757	864.7811942	110	Grand Index
25	f	0-12 years	0.758	864.9386921	110	Grand Index
25	f	0-12 years	0.759	865.728	111	Grand Index
25	f	0-12 years	0.76	866.92	111	Grand Index
25	f	0-12 years	0.761	868.112	111	Grand Index
25	f	0-12 years	0.768	869	111	Grand Index
25	f	0-12 years	0.769	869.162	111	Grand Index
25	f	0-12 years	0.77	869.46	111	Grand Index
25	f	0-12 years	0.771	869.758	111	Grand Index
25	f	0-12 years	0.775	870	111	Grand Index
25	f	0-12 years	0.776	870.496	111	Grand Index
25	f	0-12 years	0.777	871.092	111	Grand Index
25	f	0-12 years	0.778	871.688	111	Grand Index
25	f	0-12 years	0.781	872	112	Grand Index
25	f	0-12 years	0.782	872.036	112	Grand Index
25	f	0-12 years	0.783	872.334	112	Grand Index
25	f	0-12 years	0.784	872.632	112	Grand Index
25	f	0-12 years	0.785	872.93	112	Grand Index
25	f	0-12 years	0.786	873.228	112	Grand Index
25	f	0-12 years	0.787	873.526	112	Grand Index
25	f	0-12 years	0.788	873.824	112	Grand Index
25	f	0-12 years	0.791	874	112	Grand Index
25	f	0-12 years	0.792	874.032	112	Grand Index
25	f	0-12 years	0.793	874.628	112	Grand Index
25	f	0-12 years	0.794	875.224	112	Grand Index
25	f	0-12 years	0.795	875.82	112	Grand Index

Age	Gender	Education	Percentile	Raw Score	Norm Score	Assessment
25	f	0-12 years	0.796	876.208	112	Grand Index
25	f	0-12 years	0.797	876.506	112	Grand Index
25	f	0-12 years	0.798	876.804	113	Grand Index
25	f	0-12 years	0.799	877.102	113	Grand Index
25	f	0-12 years	0.8	877.4	113	Grand Index
25	f	0-12 years	0.801	877.698	113	Grand Index
25	f	0-12 years	0.802	877.996	113	Grand Index
25	f	0-12 years	0.803	878.588	113	Grand Index
25	f	0-12 years	0.804	879.184	113	Grand Index
25	f	0-12 years	0.805	879.78	113	Grand Index
25	f	0-12 years	0.812	880	113	Grand Index
25	f	0-12 years	0.813	880.274	113	Grand Index
25	f	0-12 years	0.814	880.572	113	Grand Index
25	f	0-12 years	0.815	880.87	113	Grand Index
25	f	0-12 years	0.816	881.168	114	Grand Index
25	f	0-12 years	0.817	881.466	114	Grand Index
25	f	0-12 years	0.818	881.764	114	Grand Index
25	f	0-12 years	0.819	882.062	114	Grand Index
25	f	0-12 years	0.82	882.36	114	Grand Index
25	f	0-12 years	0.821	882.658	114	Grand Index
25	f	0-12 years	0.822	882.956	114	Grand Index
25	f	0-12 years	0.823	883.254	114	Grand Index
25	f	0-12 years	0.824	883.552	114	Grand Index
25	f	0-12 years	0.825	883.85	114	Grand Index
25	f	0-12 years	0.826	884.296	114	Grand Index
25	f	0-12 years	0.827	884.892	114	Grand Index
25	f	0-12 years	0.828	885.488	114	Grand Index
25	f	0-12 years	0.832	886	114	Grand Index
25	f	0-12 years	0.833	886.234	114	Grand Index
25	f	0-12 years	0.834	886.532	115	Grand Index
25	f	0-12 years	0.835	886.83	115	Grand Index
25	f	0-12 years	0.838	887	115	Grand Index
25	f	0-12 years	0.839	887.0490413	115	Grand Index
25	f	0-12 years	0.84	887.7133278	115	Grand Index
25	f	0-12 years	0.841	888.3776142	115	Grand Index
25	f	0-12 years	0.842	889.0419007	115	Grand Index
25	f	0-12 years	0.843	889.3941113	115	Grand Index
25	f	0-12 years	0.844	889.6238248	115	Grand Index
25	f	0-12 years	0.845	889.8535384	115	Grand Index
25	f	0-12 years	0.846	890.324	115	Grand Index
25	f	0-12 years	0.847	891.218	115	Grand Index
25	f	0-12 years	0.848	892.112	115	Grand Index
25	f	0-12 years	0.849	893.002	115	Grand Index
25	f	0-12 years	0.85	893.3	116	Grand Index
25	f	0-12 years	0.851	893.598	116	Grand Index
25	f	0-12 years	0.852	893.896	116	Grand Index
25	f	0-12 years	0.859	894	116	Grand Index
25	f	0-12 years	0.86	894.28	116	Grand Index
25	f	0-12 years	0.861	894.578	116	Grand Index
25	f	0-12 years	0.862	894.876	116	Grand Index
25	f	0-12 years	0.863	895.174	116	Grand Index
25	f	0-12 years	0.864	895.472	116	Grand Index
25	f	0-12 years	0.865	895.77	117	Grand Index
25	f	0-12 years	0.869	896	117	Grand Index
25	f	0-12 years	0.87	896.52	117	Grand Index
25	f	0-12 years	0.871	897.116	117	Grand Index

Age	Gender	Education	Percentile	Raw Score	Norm Score	Assessment
25	f	0-12 years	0.872	897.712	117	Grand Index
25	f	0-12 years	0.873	898.154	117	Grand Index
25	f	0-12 years	0.874	898.452	117	Grand Index
25	f	0-12 years	0.875	898.75	117	Grand Index
25	f	0-12 years	0.876	899.0963812	117	Grand Index
25	f	0-12 years	0.877	899.6947477	117	Grand Index
25	f	0-12 years	0.878	900.2931142	117	Grand Index
25	f	0-12 years	0.879	900.8914808	118	Grand Index
25	f	0-12 years	0.88	901.2383657	118	Grand Index
25	f	0-12 years	0.881	901.5244759	118	Grand Index
25	f	0-12 years	0.882	901.8105861	118	Grand Index
25	f	0-12 years	0.883	901.972325	118	Grand Index
25	f	0-12 years	0.884	901.9818483	118	Grand Index
25	f	0-12 years	0.885	901.9913715	118	Grand Index
25	f	0-12 years	0.889	902	118	Grand Index
25	f	0-12 years	0.89	902.44	118	Grand Index
25	f	0-12 years	0.891	903.036	118	Grand Index
25	f	0-12 years	0.892	903.632	119	Grand Index
25	f	0-12 years	0.895	904	119	Grand Index
25	f	0-12 years	0.896	904.0474631	119	Grand Index
25	f	0-12 years	0.897	905.8154631	119	Grand Index
25	f	0-12 years	0.898	907.5834631	119	Grand Index
25	f	0-12 years	0.899	909.3514631	119	Grand Index
25	f	0-12 years	0.9	909.9463087	119	Grand Index
25	f	0-12 years	0.901	909.9663087	119	Grand Index
25	f	0-12 years	0.902	909.9863087	119	Grand Index
25	f	0-12 years	0.903	910.094	119	Grand Index
25	f	0-12 years	0.904	910.392	120	Grand Index
25	f	0-12 years	0.905	910.69	120	Grand Index
25	f	0-12 years	0.906	910.988	120	Grand Index
25	f	0-12 years	0.909	911	120	Grand Index
25	f	0-12 years	0.91	911.36	120	Grand Index
25	f	0-12 years	0.911	911.956	120	Grand Index
25	f	0-12 years	0.912	912.552	120	Grand Index
25	f	0-12 years	0.913	913.148	120	Grand Index
25	f	0-12 years	0.914	913.744	120	Grand Index
25	f	0-12 years	0.915	914.34	121	Grand Index
25	f	0-12 years	0.916	914.936	121	Grand Index
25	f	0-12 years	0.917	915.798	121	Grand Index
25	f	0-12 years	0.918	916.692	121	Grand Index
25	f	0-12 years	0.919	917.586	121	Grand Index
25	f	0-12 years	0.92	919.12	121	Grand Index
25	f	0-12 years	0.921	921.206	121	Grand Index
25	f	0-12 years	0.922	923.292	121	Grand Index
25	f	0-12 years	0.923	925.007359	121	Grand Index
25	f	0-12 years	0.924	925.04797	121	Grand Index
25	f	0-12 years	0.925	925.088581	122	Grand Index
25	f	0-12 years	0.926	925.129192	122	Grand Index
25	f	0-12 years	0.927	925.7041944	122	Grand Index
25	f	0-12 years	0.928	926.3921576	122	Grand Index
25	f	0-12 years	0.929	927.0801207	122	Grand Index
25	f	0-12 years	0.93	927.6625966	122	Grand Index
25	f	0-12 years	0.931	928.1260224	122	Grand Index
25	f	0-12 years	0.932	928.5894483	122	Grand Index
25	f	0-12 years	0.933	929.034	122	Grand Index
25	f	0-12 years	0.934	929.332	123	Grand Index

Age	Gender	Education	Percentile	Raw Score	Norm Score	Assessment
25	f	0-12 years	0.935	929.63	123	Grand Index
25	f	0-12 years	0.936	929.928	123	Grand Index
25	f	0-12 years	0.937	930.0987678	123	Grand Index
25	f	0-12 years	0.938	930.2290015	123	Grand Index
25	f	0-12 years	0.939	930.3592352	123	Grand Index
25	f	0-12 years	0.94	930.5045827	123	Grand Index
25	f	0-12 years	0.941	930.672349	123	Grand Index
25	f	0-12 years	0.942	930.8401153	124	Grand Index
25	f	0-12 years	0.943	931.07	124	Grand Index
25	f	0-12 years	0.944	932.56	124	Grand Index
25	f	0-12 years	0.945	934.05	124	Grand Index
25	f	0-12 years	0.946	935.54	124	Grand Index
25	f	0-12 years	0.947	937.1593454	124	Grand Index
25	f	0-12 years	0.948	938.8364566	124	Grand Index
25	f	0-12 years	0.949	940.5135678	125	Grand Index
25	f	0-12 years	0.95	941.9651011	125	Grand Index
25	f	0-12 years	0.951	942.9699898	125	Grand Index
25	f	0-12 years	0.952	943.9748786	125	Grand Index
25	f	0-12 years	0.953	944.9797673	125	Grand Index
25	f	0-12 years	0.956	945	126	Grand Index
25	f	0-12 years	0.957	947.232	126	Grand Index
25	f	0-12 years	0.958	950.808	126	Grand Index
25	f	0-12 years	0.959	954.384	126	Grand Index
25	f	0-12 years	0.96	957.08	126	Grand Index
25	f	0-12 years	0.961	957.378	126	Grand Index
25	f	0-12 years	0.962	957.676	127	Grand Index
25	f	0-12 years	0.963	957.974	127	Grand Index
25	f	0-12 years	0.964	958.544	127	Grand Index
25	f	0-12 years	0.965	959.14	127	Grand Index
25	f	0-12 years	0.966	959.736	127	Grand Index
25	f	0-12 years	0.967	960.2073448	128	Grand Index
25	f	0-12 years	0.968	960.5795661	128	Grand Index
25	f	0-12 years	0.969	960.9517874	128	Grand Index
25	f	0-12 years	0.97	961.7054968	128	Grand Index
25	f	0-12 years	0.971	963.9724417	128	Grand Index
25	f	0-12 years	0.972	966.2393867	129	Grand Index
25	f	0-12 years	0.973	968.5063317	129	Grand Index
25	f	0-12 years	0.974	969.6484846	129	Grand Index
25	f	0-12 years	0.975	970.5853183	129	Grand Index
25	f	0-12 years	0.976	971.522152	130	Grand Index
25	f	0-12 years	0.977	972.438	130	Grand Index
25	f	0-12 years	0.978	973.332	130	Grand Index
25	f	0-12 years	0.979	974.226	131	Grand Index
25	f	0-12 years	0.98	975.44	131	Grand Index
25	f	0-12 years	0.981	978.718	131	Grand Index
25	f	0-12 years	0.982	981.996	131	Grand Index
25	f	0-12 years	0.983	985.274	132	Grand Index
25	f	0-12 years	0.984	987.5175451	132	Grand Index
25	f	0-12 years	0.985	989.4668055	133	Grand Index
25	f	0-12 years	0.986	991.416066	133	Grand Index
25	f	0-12 years	0.987	992.6260316	133	Grand Index
25	f	0-12 years	0.988	992.8268008	134	Grand Index
25	f	0-12 years	0.989	993.0275701	134	Grand Index
25	f	0-12 years	0.99	993.3305676	135	Grand Index
25	f	0-12 years	0.991	995.0545378	135	Grand Index
25	f	0-12 years	0.992	996.7785081	136	Grand Index

Age	Gender	Education	Percentile	Raw Score	Norm Score	Assessment
25	f	0-12 years	0.993	998.5024784	137	Grand Index
25	f	0-12 years	0.994	1000.585107	138	Grand Index
25	f	0-12 years	0.995	1002.813229	139	Grand Index
25	f	0-12 years	0.996	1005.041351	140	Grand Index
25	f	0-12 years	0.997	1010.310661	141	Grand Index
25	f	0-12 years	0.998	1021.088539	143	Grand Index
25	f	0-12 years	0.999	1031.866417	146	Grand Index
25	m	0-12 years	0.001	552.1874186	54	Grand Index
25	m	0-12 years	0.002	560.7164788	57	Grand Index
25	m	0-12 years	0.003	569.1943949	59	Grand Index
25	m	0-12 years	0.004	580.3888982	60	Grand Index
25	m	0-12 years	0.005	584.6424754	61	Grand Index
25	m	0-12 years	0.006	594.192	62	Grand Index
25	m	0-12 years	0.007	606.488	63	Grand Index
25	m	0-12 years	0.008	619.0008357	64	Grand Index
25	m	0-12 years	0.009	619.0401119	65	Grand Index
25	m	0-12 years	0.01	622.1450699	65	Grand Index
25	m	0-12 years	0.011	625.816	66	Grand Index
25	m	0-12 years	0.012	628.12	66	Grand Index
25	m	0-12 years	0.013	631.88	67	Grand Index
25	m	0-12 years	0.014	635.112	67	Grand Index
25	m	0-12 years	0.015	637.891444	67	Grand Index
25	m	0-12 years	0.016	640.2094005	68	Grand Index
25	m	0-12 years	0.017	640.8126929	68	Grand Index
25	m	0-12 years	0.018	640.9934942	69	Grand Index
25	m	0-12 years	0.019	641.288	69	Grand Index
25	m	0-12 years	0.02	642.1908615	69	Grand Index
25	m	0-12 years	0.021	645.7790586	69	Grand Index
25	m	0-12 years	0.022	646.8900344	70	Grand Index
25	m	0-12 years	0.023	649.3605106	70	Grand Index
25	m	0-12 years	0.024	655.048	70	Grand Index
25	m	0-12 years	0.025	655.8	71	Grand Index
25	m	0-12 years	0.026	659.3052037	71	Grand Index
25	m	0-12 years	0.027	661.9914308	71	Grand Index
25	m	0-12 years	0.029	662	72	Grand Index
25	m	0-12 years	0.03	662.5576839	72	Grand Index
25	m	0-12 years	0.031	664.5571545	72	Grand Index
25	m	0-12 years	0.032	668.0693949	72	Grand Index
25	m	0-12 years	0.033	668.8847847	72	Grand Index
25	m	0-12 years	0.034	669.9575161	73	Grand Index
25	m	0-12 years	0.035	670.7427261	73	Grand Index
25	m	0-12 years	0.036	671.072	73	Grand Index
25	m	0-12 years	0.037	671.824	73	Grand Index
25	m	0-12 years	0.038	673.728	73	Grand Index
25	m	0-12 years	0.039	675.328	74	Grand Index
25	m	0-12 years	0.041	676	74	Grand Index
25	m	0-12 years	0.042	676.584	74	Grand Index
25	m	0-12 years	0.043	677	74	Grand Index
25	m	0-12 years	0.044	677.088	74	Grand Index
25	m	0-12 years	0.045	677.84	75	Grand Index
25	m	0-12 years	0.046	678.592	75	Grand Index
25	m	0-12 years	0.047	679.0199747	75	Grand Index
25	m	0-12 years	0.048	679.1484916	75	Grand Index
25	m	0-12 years	0.049	679.856826	75	Grand Index
25	m	0-12 years	0.05	680.6	75	Grand Index
25	m	0-12 years	0.051	681.2873669	75	Grand Index

Age	Gender	Education	Percentile	Raw Score	Norm Score	Assessment
25	m	0-12 years	0.052	681.9491157	76	Grand Index
25	m	0-12 years	0.053	682.9088728	76	Grand Index
25	m	0-12 years	0.054	684.1777827	76	Grand Index
25	m	0-12 years	0.055	685.8672345	76	Grand Index
25	m	0-12 years	0.056	687.780717	76	Grand Index
25	m	0-12 years	0.057	688.813263	76	Grand Index
25	m	0-12 years	0.058	689	76	Grand Index
25	m	0-12 years	0.059	689.368	77	Grand Index
25	m	0-12 years	0.06	690.24	77	Grand Index
25	m	0-12 years	0.061	691.744	77	Grand Index
25	m	0-12 years	0.062	692.9631056	77	Grand Index
25	m	0-12 years	0.063	694.4671056	77	Grand Index
25	m	0-12 years	0.066	696	77	Grand Index
25	m	0-12 years	0.067	696.384	78	Grand Index
25	m	0-12 years	0.068	697.136	78	Grand Index
25	m	0-12 years	0.069	697.888	78	Grand Index
25	m	0-12 years	0.07	699.28	78	Grand Index
25	m	0-12 years	0.071	700.392	78	Grand Index
25	m	0-12 years	0.074	701	78	Grand Index
25	m	0-12 years	0.075	701.4	78	Grand Index
25	m	0-12 years	0.076	702.456	79	Grand Index
25	m	0-12 years	0.077	704.712	79	Grand Index
25	m	0-12 years	0.078	705	79	Grand Index
25	m	0-12 years	0.079	705.0698597	79	Grand Index
25	m	0-12 years	0.08	705.4638288	79	Grand Index
25	m	0-12 years	0.081	706.8390678	79	Grand Index
25	m	0-12 years	0.083	707	79	Grand Index
25	m	0-12 years	0.084	707.168	79	Grand Index
25	m	0-12 years	0.085	707.92	79	Grand Index
25	m	0-12 years	0.087	708	80	Grand Index
25	m	0-12 years	0.088	708.176	80	Grand Index
25	m	0-12 years	0.089	708.928	80	Grand Index
25	m	0-12 years	0.091	709	80	Grand Index
25	m	0-12 years	0.092	709.184	80	Grand Index
25	m	0-12 years	0.093	709.936	80	Grand Index
25	m	0-12 years	0.094	710.688	80	Grand Index
25	m	0-12 years	0.095	711.44	80	Grand Index
25	m	0-12 years	0.096	712.1593125	80	Grand Index
25	m	0-12 years	0.097	712.7832865	81	Grand Index
25	m	0-12 years	0.098	712.9482448	81	Grand Index
25	m	0-12 years	0.099	713	81	Grand Index
25	m	0-12 years	0.1	713.2	81	Grand Index
25	m	0-12 years	0.101	713.952	81	Grand Index
25	m	0-12 years	0.102	714	81	Grand Index
25	m	0-12 years	0.103	714.912	81	Grand Index
25	m	0-12 years	0.104	716.1911838	81	Grand Index
25	m	0-12 years	0.105	716.8823865	81	Grand Index
25	m	0-12 years	0.106	716.976716	81	Grand Index
25	m	0-12 years	0.107	717	81	Grand Index
25	m	0-12 years	0.108	717.1845637	81	Grand Index
25	m	0-12 years	0.109	717.8271188	82	Grand Index
25	m	0-12 years	0.11	717.9592492	82	Grand Index
25	m	0-12 years	0.111	718.472	82	Grand Index
25	m	0-12 years	0.113	719	82	Grand Index
25	m	0-12 years	0.114	719.5906796	82	Grand Index
25	m	0-12 years	0.115	719.8855983	82	Grand Index

Age	Gender	Education	Percentile	Raw Score	Norm Score	Assessment
25	m	0-12 years	0.116	719.9738949	82	Grand Index
25	m	0-12 years	0.117	719.9994561	82	Grand Index
25	m	0-12 years	0.119	720	82	Grand Index
25	m	0-12 years	0.12	720.24	82	Grand Index
25	m	0-12 years	0.121	720.992	82	Grand Index
25	m	0-12 years	0.125	721	83	Grand Index
25	m	0-12 years	0.126	723.256	83	Grand Index
25	m	0-12 years	0.127	724	83	Grand Index
25	m	0-12 years	0.128	724.256	83	Grand Index
25	m	0-12 years	0.132	725	83	Grand Index
25	m	0-12 years	0.133	725.048	83	Grand Index
25	m	0-12 years	0.134	727.304	83	Grand Index
25	m	0-12 years	0.135	728.006688	83	Grand Index
25	m	0-12 years	0.136	728.0307306	84	Grand Index
25	m	0-12 years	0.137	728.091035	84	Grand Index
25	m	0-12 years	0.138	728.4820355	84	Grand Index
25	m	0-12 years	0.139	729.3148724	84	Grand Index
25	m	0-12 years	0.14	729.9677891	84	Grand Index
25	m	0-12 years	0.143	730	84	Grand Index
25	m	0-12 years	0.144	730.288	84	Grand Index
25	m	0-12 years	0.145	731.04	84	Grand Index
25	m	0-12 years	0.146	731.792	84	Grand Index
25	m	0-12 years	0.148	732	84	Grand Index
25	m	0-12 years	0.149	732.048	84	Grand Index
25	m	0-12 years	0.15	732.8	84	Grand Index
25	m	0-12 years	0.151	733.552	85	Grand Index
25	m	0-12 years	0.152	734	85	Grand Index
25	m	0-12 years	0.153	734.056	85	Grand Index
25	m	0-12 years	0.154	734.808	85	Grand Index
25	m	0-12 years	0.156	735	85	Grand Index
25	m	0-12 years	0.157	735.192	85	Grand Index
25	m	0-12 years	0.158	737.448	85	Grand Index
25	m	0-12 years	0.159	738.568	85	Grand Index
25	m	0-12 years	0.16	739.32	85	Grand Index
25	m	0-12 years	0.163	740	85	Grand Index
25	m	0-12 years	0.164	740.328	85	Grand Index
25	m	0-12 years	0.166	741	85	Grand Index
25	m	0-12 years	0.167	741.584	86	Grand Index
25	m	0-12 years	0.168	742.3313147	86	Grand Index
25	m	0-12 years	0.169	742.9872829	86	Grand Index
25	m	0-12 years	0.17	742.9977689	86	Grand Index
25	m	0-12 years	0.174	743	86	Grand Index
25	m	0-12 years	0.175	743.6	86	Grand Index
25	m	0-12 years	0.176	744.352	86	Grand Index
25	m	0-12 years	0.179	745	86	Grand Index
25	m	0-12 years	0.18	745.36	86	Grand Index
25	m	0-12 years	0.182	746	86	Grand Index
25	m	0-12 years	0.183	746.616	86	Grand Index
25	m	0-12 years	0.19	747	87	Grand Index
25	m	0-12 years	0.191	747.632	87	Grand Index
25	m	0-12 years	0.192	748	87	Grand Index
25	m	0-12 years	0.193	748.272	87	Grand Index
25	m	0-12 years	0.194	749.776	87	Grand Index
25	m	0-12 years	0.195	750.5617536	87	Grand Index
25	m	0-12 years	0.196	750.9256659	87	Grand Index
25	m	0-12 years	0.197	751.144	87	Grand Index

Age	Gender	Education	Percentile	Raw Score	Norm Score	Assessment
25	m	0-12 years	0.198	751.896	87	Grand Index
25	m	0-12 years	0.202	752	87	Grand Index
25	m	0-12 years	0.203	752.656	88	Grand Index
25	m	0-12 years	0.206	753	88	Grand Index
25	m	0-12 years	0.207	753.0370919	88	Grand Index
25	m	0-12 years	0.208	753.448623	88	Grand Index
25	m	0-12 years	0.212	754	88	Grand Index
25	m	0-12 years	0.213	754.0016874	88	Grand Index
25	m	0-12 years	0.214	754.008897	88	Grand Index
25	m	0-12 years	0.215	754.6830679	88	Grand Index
25	m	0-12 years	0.218	755	88	Grand Index
25	m	0-12 years	0.219	756.3683001	88	Grand Index
25	m	0-12 years	0.22	756.9937326	88	Grand Index
25	m	0-12 years	0.222	757	89	Grand Index
25	m	0-12 years	0.223	757.696	89	Grand Index
25	m	0-12 years	0.228	758	89	Grand Index
25	m	0-12 years	0.229	758.208	89	Grand Index
25	m	0-12 years	0.23	758.96	89	Grand Index
25	m	0-12 years	0.234	759	89	Grand Index
25	m	0-12 years	0.235	759.72	89	Grand Index
25	m	0-12 years	0.236	760	89	Grand Index
25	m	0-12 years	0.237	760.224	89	Grand Index
25	m	0-12 years	0.238	760.976	89	Grand Index
25	m	0-12 years	0.239	761.0014927	89	Grand Index
25	m	0-12 years	0.24	761.4810662	89	Grand Index
25	m	0-12 years	0.244	762	90	Grand Index
25	m	0-12 years	0.245	762.24	90	Grand Index
25	m	0-12 years	0.246	762.992	90	Grand Index
25	m	0-12 years	0.248	763	90	Grand Index
25	m	0-12 years	0.249	763.496	90	Grand Index
25	m	0-12 years	0.253	765	90	Grand Index
25	m	0-12 years	0.254	765.008	90	Grand Index
25	m	0-12 years	0.255	765.76	90	Grand Index
25	m	0-12 years	0.256	766.512	90	Grand Index
25	m	0-12 years	0.261	767	90	Grand Index
25	m	0-12 years	0.262	767.024	90	Grand Index
25	m	0-12 years	0.263	767.776	90	Grand Index
25	m	0-12 years	0.265	768	91	Grand Index
25	m	0-12 years	0.266	768.064	91	Grand Index
25	m	0-12 years	0.267	769.568	91	Grand Index
25	m	0-12 years	0.272	770	91	Grand Index
25	m	0-12 years	0.273	770.296	91	Grand Index
25	m	0-12 years	0.274	771.0183169	91	Grand Index
25	m	0-12 years	0.275	771.3052811	91	Grand Index
25	m	0-12 years	0.276	771.7229574	91	Grand Index
25	m	0-12 years	0.277	772	91	Grand Index
25	m	0-12 years	0.278	772.0042803	91	Grand Index
25	m	0-12 years	0.279	772.061758	91	Grand Index
25	m	0-12 years	0.28	772.5936306	91	Grand Index
25	m	0-12 years	0.283	773	91	Grand Index
25	m	0-12 years	0.284	773.2987428	91	Grand Index
25	m	0-12 years	0.285	773.6776498	91	Grand Index
25	m	0-12 years	0.287	774	92	Grand Index
25	m	0-12 years	0.288	774.41437	92	Grand Index
25	m	0-12 years	0.289	774.7230944	92	Grand Index
25	m	0-12 years	0.29	774.7522248	92	Grand Index

Age	Gender	Education	Percentile	Raw Score	Norm Score	Assessment
25	m	0-12 years	0.291	774.9547541	92	Grand Index
25	m	0-12 years	0.292	776.168	92	Grand Index
25	m	0-12 years	0.295	777	92	Grand Index
25	m	0-12 years	0.296	777.5345167	92	Grand Index
25	m	0-12 years	0.297	777.9363023	92	Grand Index
25	m	0-12 years	0.303	778	92	Grand Index
25	m	0-12 years	0.304	778.608	92	Grand Index
25	m	0-12 years	0.305	779	92	Grand Index
25	m	0-12 years	0.306	779.0215385	92	Grand Index
25	m	0-12 years	0.307	779.1661538	92	Grand Index
25	m	0-12 years	0.308	779.6898462	92	Grand Index
25	m	0-12 years	0.315	780	93	Grand Index
25	m	0-12 years	0.316	780.223937	93	Grand Index
25	m	0-12 years	0.317	780.6022677	93	Grand Index
25	m	0-12 years	0.319	781	93	Grand Index
25	m	0-12 years	0.32	781.126533	93	Grand Index
25	m	0-12 years	0.321	781.3431083	93	Grand Index
25	m	0-12 years	0.322	781.6039923	93	Grand Index
25	m	0-12 years	0.323	781.7886752	93	Grand Index
25	m	0-12 years	0.324	781.9346042	93	Grand Index
25	m	0-12 years	0.331	782	93	Grand Index
25	m	0-12 years	0.332	782.2329594	93	Grand Index
25	m	0-12 years	0.333	782.4066949	94	Grand Index
25	m	0-12 years	0.334	782.5716058	94	Grand Index
25	m	0-12 years	0.335	782.9588083	94	Grand Index
25	m	0-12 years	0.337	783	94	Grand Index
25	m	0-12 years	0.338	783.176	94	Grand Index
25	m	0-12 years	0.339	783.928	94	Grand Index
25	m	0-12 years	0.347	784	94	Grand Index
25	m	0-12 years	0.348	784.696	94	Grand Index
25	m	0-12 years	0.353	785	94	Grand Index
25	m	0-12 years	0.354	785.0391529	94	Grand Index
25	m	0-12 years	0.355	785.1807059	94	Grand Index
25	m	0-12 years	0.356	785.7662118	94	Grand Index
25	m	0-12 years	0.359	786	95	Grand Index
25	m	0-12 years	0.36	786.2527229	95	Grand Index
25	m	0-12 years	0.361	786.6573301	95	Grand Index
25	m	0-12 years	0.363	787	95	Grand Index
25	m	0-12 years	0.364	787.728	95	Grand Index
25	m	0-12 years	0.365	788.48	95	Grand Index
25	m	0-12 years	0.368	789	95	Grand Index
25	m	0-12 years	0.369	789.488	95	Grand Index
25	m	0-12 years	0.371	790	95	Grand Index
25	m	0-12 years	0.372	790.1784755	95	Grand Index
25	m	0-12 years	0.373	790.6169028	95	Grand Index
25	m	0-12 years	0.377	791	95	Grand Index
25	m	0-12 years	0.378	791.0066017	95	Grand Index
25	m	0-12 years	0.379	791.0335817	95	Grand Index
25	m	0-12 years	0.38	791.7661891	95	Grand Index
25	m	0-12 years	0.381	792.0046461	95	Grand Index
25	m	0-12 years	0.382	792.0460388	95	Grand Index
25	m	0-12 years	0.383	792.1627055	96	Grand Index
25	m	0-12 years	0.384	792.8025891	96	Grand Index
25	m	0-12 years	0.385	793.52	96	Grand Index
25	m	0-12 years	0.393	794	96	Grand Index
25	m	0-12 years	0.394	794.288	96	Grand Index

Age	Gender	Education	Percentile	Raw Score	Norm Score	Assessment
25	m	0-12 years	0.398	795	96	Grand Index
25	m	0-12 years	0.399	795.048	96	Grand Index
25	m	0-12 years	0.4	795.8	96	Grand Index
25	m	0-12 years	0.401	796	96	Grand Index
25	m	0-12 years	0.402	796.304	96	Grand Index
25	m	0-12 years	0.404	797	96	Grand Index
25	m	0-12 years	0.405	797.56	96	Grand Index
25	m	0-12 years	0.406	798.312	96	Grand Index
25	m	0-12 years	0.409	799	97	Grand Index
25	m	0-12 years	0.41	799.32	97	Grand Index
25	m	0-12 years	0.412	800	97	Grand Index
25	m	0-12 years	0.413	800.576	97	Grand Index
25	m	0-12 years	0.424	801	97	Grand Index
25	m	0-12 years	0.425	801.1062673	97	Grand Index
25	m	0-12 years	0.426	801.4667687	97	Grand Index
25	m	0-12 years	0.432	802	97	Grand Index
25	m	0-12 years	0.433	802.616	97	Grand Index
25	m	0-12 years	0.44	803	98	Grand Index
25	m	0-12 years	0.441	803.632	98	Grand Index
25	m	0-12 years	0.442	804	98	Grand Index
25	m	0-12 years	0.443	804.0238779	98	Grand Index
25	m	0-12 years	0.444	804.1559084	98	Grand Index
25	m	0-12 years	0.445	804.7032061	98	Grand Index
25	m	0-12 years	0.448	805	98	Grand Index
25	m	0-12 years	0.449	805.2104011	98	Grand Index
25	m	0-12 years	0.45	805.5948158	98	Grand Index
25	m	0-12 years	0.454	806	98	Grand Index
25	m	0-12 years	0.455	806.1598591	98	Grand Index
25	m	0-12 years	0.456	806.9111968	98	Grand Index
25	m	0-12 years	0.457	806.9997041	98	Grand Index
25	m	0-12 years	0.461	807	99	Grand Index
25	m	0-12 years	0.462	807.424	99	Grand Index
25	m	0-12 years	0.464	808	99	Grand Index
25	m	0-12 years	0.465	808.68	99	Grand Index
25	m	0-12 years	0.469	809	99	Grand Index
25	m	0-12 years	0.47	809.44	99	Grand Index
25	m	0-12 years	0.471	810.029884	99	Grand Index
25	m	0-12 years	0.472	810.1469298	99	Grand Index
25	m	0-12 years	0.473	810.5816194	99	Grand Index
25	m	0-12 years	0.474	810.8717576	99	Grand Index
25	m	0-12 years	0.48	811	99	Grand Index
25	m	0-12 years	0.481	811.712	99	Grand Index
25	m	0-12 years	0.482	812.0405779	99	Grand Index
25	m	0-12 years	0.483	812.2845627	99	Grand Index
25	m	0-12 years	0.484	812.9707985	99	Grand Index
25	m	0-12 years	0.486	813	99	Grand Index
25	m	0-12 years	0.487	813.0346632	100	Grand Index
25	m	0-12 years	0.488	813.1510325	100	Grand Index
25	m	0-12 years	0.489	813.770091	100	Grand Index
25	m	0-12 years	0.496	814	100	Grand Index
25	m	0-12 years	0.497	814.744	100	Grand Index
25	m	0-12 years	0.502	815	100	Grand Index
25	m	0-12 years	0.503	815.0072898	100	Grand Index
25	m	0-12 years	0.504	815.0362479	100	Grand Index
25	m	0-12 years	0.505	815.7668342	100	Grand Index
25	m	0-12 years	0.506	816	100	Grand Index

Age	Gender	Education	Percentile	Raw Score	Norm Score	Assessment
25	m	0-12 years	0.507	816.0309913	100	Grand Index
25	m	0-12 years	0.508	816.131513	100	Grand Index
25	m	0-12 years	0.509	816.7952348	100	Grand Index
25	m	0-12 years	0.513	817	100	Grand Index
25	m	0-12 years	0.514	817.052	101	Grand Index
25	m	0-12 years	0.515	817.3509091	101	Grand Index
25	m	0-12 years	0.518	818	101	Grand Index
25	m	0-12 years	0.519	818.0471273	101	Grand Index
25	m	0-12 years	0.52	818.1970909	101	Grand Index
25	m	0-12 years	0.521	818.8260364	101	Grand Index
25	m	0-12 years	0.522	819	101	Grand Index
25	m	0-12 years	0.523	819.296	101	Grand Index
25	m	0-12 years	0.53	820	101	Grand Index
25	m	0-12 years	0.531	820.2440816	101	Grand Index
25	m	0-12 years	0.532	820.7947768	101	Grand Index
25	m	0-12 years	0.533	820.9412268	101	Grand Index
25	m	0-12 years	0.534	820.9900901	101	Grand Index
25	m	0-12 years	0.538	821	101	Grand Index
25	m	0-12 years	0.539	821.328	101	Grand Index
25	m	0-12 years	0.543	822	102	Grand Index
25	m	0-12 years	0.544	822.0203687	102	Grand Index
25	m	0-12 years	0.545	822.1944289	102	Grand Index
25	m	0-12 years	0.546	822.6864369	102	Grand Index
25	m	0-12 years	0.55	823	102	Grand Index
25	m	0-12 years	0.551	823.352	102	Grand Index
25	m	0-12 years	0.558	824	102	Grand Index
25	m	0-12 years	0.559	824.184608	102	Grand Index
25	m	0-12 years	0.56	824.561454	102	Grand Index
25	m	0-12 years	0.561	824.9362115	102	Grand Index
25	m	0-12 years	0.562	825	102	Grand Index
25	m	0-12 years	0.563	825.1500428	102	Grand Index
25	m	0-12 years	0.564	825.4759715	102	Grand Index
25	m	0-12 years	0.565	825.927886	102	Grand Index
25	m	0-12 years	0.569	826	103	Grand Index
25	m	0-12 years	0.57	826.6223969	103	Grand Index
25	m	0-12 years	0.571	826.983277	103	Grand Index
25	m	0-12 years	0.573	827	103	Grand Index
25	m	0-12 years	0.574	827.443052	103	Grand Index
25	m	0-12 years	0.575	827.8102333	103	Grand Index
25	m	0-12 years	0.579	828	103	Grand Index
25	m	0-12 years	0.58	828.16	103	Grand Index
25	m	0-12 years	0.581	828.912	103	Grand Index
25	m	0-12 years	0.587	829	103	Grand Index
25	m	0-12 years	0.588	829.176	103	Grand Index
25	m	0-12 years	0.589	829.928	103	Grand Index
25	m	0-12 years	0.594	830	104	Grand Index
25	m	0-12 years	0.595	830.44	104	Grand Index
25	m	0-12 years	0.598	831	104	Grand Index
25	m	0-12 years	0.599	831.448	104	Grand Index
25	m	0-12 years	0.603	832	104	Grand Index
25	m	0-12 years	0.604	832.0047012	104	Grand Index
25	m	0-12 years	0.605	832.0216976	104	Grand Index
25	m	0-12 years	0.606	832.5774527	104	Grand Index
25	m	0-12 years	0.607	832.8938113	104	Grand Index
25	m	0-12 years	0.609	833	104	Grand Index
25	m	0-12 years	0.61	833.72	104	Grand Index

Age	Gender	Education	Percentile	Raw Score	Norm Score	Assessment
25	m	0-12 years	0.611	834.472	104	Grand Index
25	m	0-12 years	0.619	835	105	Grand Index
25	m	0-12 years	0.62	835.24	105	Grand Index
25	m	0-12 years	0.621	835.992	105	Grand Index
25	m	0-12 years	0.625	836	105	Grand Index
25	m	0-12 years	0.626	836.752	105	Grand Index
25	m	0-12 years	0.628	837	105	Grand Index
25	m	0-12 years	0.629	837.008	105	Grand Index
25	m	0-12 years	0.63	837.76	105	Grand Index
25	m	0-12 years	0.635	838	105	Grand Index
25	m	0-12 years	0.636	838.0042965	105	Grand Index
25	m	0-12 years	0.637	838.0224882	105	Grand Index
25	m	0-12 years	0.638	838.2321811	105	Grand Index
25	m	0-12 years	0.639	838.6670714	105	Grand Index
25	m	0-12 years	0.647	839	106	Grand Index
25	m	0-12 years	0.648	839.2678919	106	Grand Index
25	m	0-12 years	0.649	839.9095982	106	Grand Index
25	m	0-12 years	0.65	839.981008	106	Grand Index
25	m	0-12 years	0.651	841.104	106	Grand Index
25	m	0-12 years	0.654	842	106	Grand Index
25	m	0-12 years	0.655	842.56	106	Grand Index
25	m	0-12 years	0.659	843	106	Grand Index
25	m	0-12 years	0.66	843.32	106	Grand Index
25	m	0-12 years	0.661	844.072	106	Grand Index
25	m	0-12 years	0.662	844.824	106	Grand Index
25	m	0-12 years	0.667	845	106	Grand Index
25	m	0-12 years	0.668	845.0258462	107	Grand Index
25	m	0-12 years	0.669	845.1581538	107	Grand Index
25	m	0-12 years	0.67	845.8523077	107	Grand Index
25	m	0-12 years	0.671	846	107	Grand Index
25	m	0-12 years	0.672	846.344	107	Grand Index
25	m	0-12 years	0.676	847	107	Grand Index
25	m	0-12 years	0.677	847.104	107	Grand Index
25	m	0-12 years	0.678	847.856	107	Grand Index
25	m	0-12 years	0.686	848	107	Grand Index
25	m	0-12 years	0.687	848.624	107	Grand Index
25	m	0-12 years	0.692	849	108	Grand Index
25	m	0-12 years	0.693	849.0008588	108	Grand Index
25	m	0-12 years	0.694	849.0056072	108	Grand Index
25	m	0-12 years	0.695	849.3671749	108	Grand Index
25	m	0-12 years	0.696	849.7386566	108	Grand Index
25	m	0-12 years	0.702	850	108	Grand Index
25	m	0-12 years	0.703	850.656	108	Grand Index
25	m	0-12 years	0.704	851	108	Grand Index
25	m	0-12 years	0.705	851.16	108	Grand Index
25	m	0-12 years	0.706	851.912	108	Grand Index
25	m	0-12 years	0.707	852.664	108	Grand Index
25	m	0-12 years	0.71	853	108	Grand Index
25	m	0-12 years	0.711	853.4712095	108	Grand Index
25	m	0-12 years	0.712	853.8278939	108	Grand Index
25	m	0-12 years	0.718	854	109	Grand Index
25	m	0-12 years	0.719	854.688	109	Grand Index
25	m	0-12 years	0.72	855	109	Grand Index
25	m	0-12 years	0.721	855.0587184	109	Grand Index
25	m	0-12 years	0.722	855.288699	109	Grand Index
25	m	0-12 years	0.723	855.7889709	109	Grand Index

Age	Gender	Education	Percentile	Raw Score	Norm Score	Assessment
25	m	0-12 years	0.726	856	109	Grand Index
25	m	0-12 years	0.727	856.0042482	109	Grand Index
25	m	0-12 years	0.728	856.4592827	109	Grand Index
25	m	0-12 years	0.732	857	109	Grand Index
25	m	0-12 years	0.733	857.216	109	Grand Index
25	m	0-12 years	0.734	857.968	109	Grand Index
25	m	0-12 years	0.736	858	109	Grand Index
25	m	0-12 years	0.737	858.224	110	Grand Index
25	m	0-12 years	0.738	858.976	110	Grand Index
25	m	0-12 years	0.742	859	110	Grand Index
25	m	0-12 years	0.743	859.6715431	110	Grand Index
25	m	0-12 years	0.744	859.9551604	110	Grand Index
25	m	0-12 years	0.745	860.24	110	Grand Index
25	m	0-12 years	0.746	860.992	110	Grand Index
25	m	0-12 years	0.751	861	110	Grand Index
25	m	0-12 years	0.752	861.0040755	110	Grand Index
25	m	0-12 years	0.753	861.1893782	110	Grand Index
25	m	0-12 years	0.754	861.718528	110	Grand Index
25	m	0-12 years	0.755	861.9319019	110	Grand Index
25	m	0-12 years	0.756	862	110	Grand Index
25	m	0-12 years	0.757	862.0481313	110	Grand Index
25	m	0-12 years	0.758	862.1835951	110	Grand Index
25	m	0-12 years	0.759	862.2437398	111	Grand Index
25	m	0-12 years	0.76	862.6459016	111	Grand Index
25	m	0-12 years	0.764	863	111	Grand Index
25	m	0-12 years	0.765	863.28	111	Grand Index
25	m	0-12 years	0.767	864	111	Grand Index
25	m	0-12 years	0.768	864.5019021	111	Grand Index
25	m	0-12 years	0.769	864.9547057	111	Grand Index
25	m	0-12 years	0.773	865	111	Grand Index
25	m	0-12 years	0.774	865.0113455	111	Grand Index
25	m	0-12 years	0.775	865.1890909	111	Grand Index
25	m	0-12 years	0.776	865.6578909	111	Grand Index
25	m	0-12 years	0.777	866	111	Grand Index
25	m	0-12 years	0.778	866.056	111	Grand Index
25	m	0-12 years	0.779	866.808	112	Grand Index
25	m	0-12 years	0.78	867	112	Grand Index
25	m	0-12 years	0.781	867.3113074	112	Grand Index
25	m	0-12 years	0.782	867.9979223	112	Grand Index
25	m	0-12 years	0.783	867.9995916	112	Grand Index
25	m	0-12 years	0.784	868.568	112	Grand Index
25	m	0-12 years	0.788	869	112	Grand Index
25	m	0-12 years	0.789	869.328	112	Grand Index
25	m	0-12 years	0.792	870	112	Grand Index
25	m	0-12 years	0.793	870.336	112	Grand Index
25	m	0-12 years	0.794	871.176	112	Grand Index
25	m	0-12 years	0.795	872.68	112	Grand Index
25	m	0-12 years	0.796	873	112	Grand Index
25	m	0-12 years	0.797	873.344	112	Grand Index
25	m	0-12 years	0.801	874	113	Grand Index
25	m	0-12 years	0.802	874.104	113	Grand Index
25	m	0-12 years	0.803	874.856	113	Grand Index
25	m	0-12 years	0.804	875	113	Grand Index
25	m	0-12 years	0.805	875.2168981	113	Grand Index
25	m	0-12 years	0.806	875.6470154	113	Grand Index
25	m	0-12 years	0.807	875.9459393	113	Grand Index

Age	Gender	Education	Percentile	Raw Score	Norm Score	Assessment
25	m	0-12 years	0.809	876	113	Grand Index
25	m	0-12 years	0.81	876.12	113	Grand Index
25	m	0-12 years	0.811	876.872	113	Grand Index
25	m	0-12 years	0.812	877.422895	113	Grand Index
25	m	0-12 years	0.813	877.7438706	113	Grand Index
25	m	0-12 years	0.814	878.0492062	113	Grand Index
25	m	0-12 years	0.815	879.1980488	113	Grand Index
25	m	0-12 years	0.816	879.7523529	114	Grand Index
25	m	0-12 years	0.817	879.9805132	114	Grand Index
25	m	0-12 years	0.821	880	114	Grand Index
25	m	0-12 years	0.822	880.144	114	Grand Index
25	m	0-12 years	0.823	880.896	114	Grand Index
25	m	0-12 years	0.825	881	114	Grand Index
25	m	0-12 years	0.826	881.152	114	Grand Index
25	m	0-12 years	0.827	881.904	114	Grand Index
25	m	0-12 years	0.829	882	114	Grand Index
25	m	0-12 years	0.83	882.32	114	Grand Index
25	m	0-12 years	0.831	883.824	114	Grand Index
25	m	0-12 years	0.832	884.664	114	Grand Index
25	m	0-12 years	0.833	885.416	114	Grand Index
25	m	0-12 years	0.834	886.168	115	Grand Index
25	m	0-12 years	0.835	886.92	115	Grand Index
25	m	0-12 years	0.837	887	115	Grand Index
25	m	0-12 years	0.838	887.1697882	115	Grand Index
25	m	0-12 years	0.839	887.8952471	115	Grand Index
25	m	0-12 years	0.84	887.9887059	115	Grand Index
25	m	0-12 years	0.844	888	115	Grand Index
25	m	0-12 years	0.845	888.44	115	Grand Index
25	m	0-12 years	0.847	889	115	Grand Index
25	m	0-12 years	0.848	889.696	115	Grand Index
25	m	0-12 years	0.849	890.448	115	Grand Index
25	m	0-12 years	0.85	891.2	116	Grand Index
25	m	0-12 years	0.851	891.952	116	Grand Index
25	m	0-12 years	0.852	892.704	116	Grand Index
25	m	0-12 years	0.855	893	116	Grand Index
25	m	0-12 years	0.856	894.424	116	Grand Index
25	m	0-12 years	0.86	895	116	Grand Index
25	m	0-12 years	0.861	895.472	116	Grand Index
25	m	0-12 years	0.862	896.01991	116	Grand Index
25	m	0-12 years	0.863	896.0867508	116	Grand Index
25	m	0-12 years	0.864	896.7153764	116	Grand Index
25	m	0-12 years	0.865	896.9737143	117	Grand Index
25	m	0-12 years	0.868	897	117	Grand Index
25	m	0-12 years	0.869	897.488	117	Grand Index
25	m	0-12 years	0.87	898.0022006	117	Grand Index
25	m	0-12 years	0.871	898.0090956	117	Grand Index
25	m	0-12 years	0.872	898.7463473	117	Grand Index
25	m	0-12 years	0.873	899.496	117	Grand Index
25	m	0-12 years	0.874	900.248	117	Grand Index
25	m	0-12 years	0.875	901	117	Grand Index
25	m	0-12 years	0.876	902.504	117	Grand Index
25	m	0-12 years	0.877	903	117	Grand Index
25	m	0-12 years	0.878	903.256	117	Grand Index
25	m	0-12 years	0.879	904.008	118	Grand Index
25	m	0-12 years	0.88	904.76	118	Grand Index
25	m	0-12 years	0.881	906.024	118	Grand Index

Age	Gender	Education	Percentile	Raw Score	Norm Score	Assessment
25	m	0-12 years	0.884	907	118	Grand Index
25	m	0-12 years	0.885	907.5088082	118	Grand Index
25	m	0-12 years	0.886	907.9843314	118	Grand Index
25	m	0-12 years	0.887	908.024	118	Grand Index
25	m	0-12 years	0.888	908.776	118	Grand Index
25	m	0-12 years	0.889	909.0257371	118	Grand Index
25	m	0-12 years	0.89	909.315096	118	Grand Index
25	m	0-12 years	0.892	910	119	Grand Index
25	m	0-12 years	0.893	910.536	119	Grand Index
25	m	0-12 years	0.894	911	119	Grand Index
25	m	0-12 years	0.895	911.04	119	Grand Index
25	m	0-12 years	0.896	911.792	119	Grand Index
25	m	0-12 years	0.897	912	119	Grand Index
25	m	0-12 years	0.898	912.296	119	Grand Index
25	m	0-12 years	0.899	913.048	119	Grand Index
25	m	0-12 years	0.9	913.8	119	Grand Index
25	m	0-12 years	0.901	914.552	119	Grand Index
25	m	0-12 years	0.902	915.304	119	Grand Index
25	m	0-12 years	0.903	916.056	119	Grand Index
25	m	0-12 years	0.904	916.808	120	Grand Index
25	m	0-12 years	0.906	917	120	Grand Index
25	m	0-12 years	0.907	917.0597736	120	Grand Index
25	m	0-12 years	0.908	917.7621132	120	Grand Index
25	m	0-12 years	0.909	917.9714717	120	Grand Index
25	m	0-12 years	0.91	918	120	Grand Index
25	m	0-12 years	0.911	918.072	120	Grand Index
25	m	0-12 years	0.912	918.824	120	Grand Index
25	m	0-12 years	0.913	919.576	120	Grand Index
25	m	0-12 years	0.914	920	120	Grand Index
25	m	0-12 years	0.915	920.0289362	121	Grand Index
25	m	0-12 years	0.916	920.3009362	121	Grand Index
25	m	0-12 years	0.917	921.3184681	121	Grand Index
25	m	0-12 years	0.918	922	121	Grand Index
25	m	0-12 years	0.919	922.088	121	Grand Index
25	m	0-12 years	0.92	922.84	121	Grand Index
25	m	0-12 years	0.921	923.592	121	Grand Index
25	m	0-12 years	0.922	924.7955665	121	Grand Index
25	m	0-12 years	0.923	926.326512	121	Grand Index
25	m	0-12 years	0.924	926.4347587	121	Grand Index
25	m	0-12 years	0.925	926.7826553	122	Grand Index
25	m	0-12 years	0.926	927.524549	122	Grand Index
25	m	0-12 years	0.927	928.5432157	122	Grand Index
25	m	0-12 years	0.928	928.9265882	122	Grand Index
25	m	0-12 years	0.929	929.0736637	122	Grand Index
25	m	0-12 years	0.93	929.4375407	122	Grand Index
25	m	0-12 years	0.934	930	123	Grand Index
25	m	0-12 years	0.935	930.12	123	Grand Index
25	m	0-12 years	0.936	930.872	123	Grand Index
25	m	0-12 years	0.937	931	123	Grand Index
25	m	0-12 years	0.938	931.1870457	123	Grand Index
25	m	0-12 years	0.939	931.5617868	123	Grand Index
25	m	0-12 years	0.94	931.9396954	123	Grand Index
25	m	0-12 years	0.941	932.632	123	Grand Index
25	m	0-12 years	0.942	934.1318239	124	Grand Index
25	m	0-12 years	0.943	935.9546037	124	Grand Index
25	m	0-12 years	0.944	935.9941153	124	Grand Index

Age	Gender	Education	Percentile	Raw Score	Norm Score	Assessment
25	m	0-12 years	0.945	936.4823919	124	Grand Index
25	m	0-12 years	0.946	937.6342723	124	Grand Index
25	m	0-12 years	0.947	939.1215337	124	Grand Index
25	m	0-12 years	0.948	939.7562097	124	Grand Index
25	m	0-12 years	0.949	940.0224709	125	Grand Index
25	m	0-12 years	0.95	941.2716561	125	Grand Index
25	m	0-12 years	0.953	943	125	Grand Index
25	m	0-12 years	0.954	943.408	125	Grand Index
25	m	0-12 years	0.956	944	126	Grand Index
25	m	0-12 years	0.957	944.664	126	Grand Index
25	m	0-12 years	0.958	945.1313942	126	Grand Index
25	m	0-12 years	0.959	945.5987885	126	Grand Index
25	m	0-12 years	0.96	946.8652681	126	Grand Index
25	m	0-12 years	0.961	948.4378702	126	Grand Index
25	m	0-12 years	0.962	949.5044601	127	Grand Index
25	m	0-12 years	0.964	950	127	Grand Index
25	m	0-12 years	0.965	954.08	127	Grand Index
25	m	0-12 years	0.966	956.432	127	Grand Index
25	m	0-12 years	0.968	957	128	Grand Index
25	m	0-12 years	0.969	959.064	128	Grand Index
25	m	0-12 years	0.97	960.2509588	128	Grand Index
25	m	0-12 years	0.971	960.693729	128	Grand Index
25	m	0-12 years	0.972	961.1769209	129	Grand Index
25	m	0-12 years	0.973	961.7607226	129	Grand Index
25	m	0-12 years	0.974	962	129	Grand Index
25	m	0-12 years	0.975	962.8	129	Grand Index
25	m	0-12 years	0.976	965.808	130	Grand Index
25	m	0-12 years	0.977	966	130	Grand Index
25	m	0-12 years	0.978	966.456	130	Grand Index
25	m	0-12 years	0.979	967.208	131	Grand Index
25	m	0-12 years	0.98	967.96	131	Grand Index
25	m	0-12 years	0.981	971.56	131	Grand Index
25	m	0-12 years	0.982	973	131	Grand Index
25	m	0-12 years	0.983	973.432	132	Grand Index
25	m	0-12 years	0.984	974.936	132	Grand Index
25	m	0-12 years	0.985	975	133	Grand Index
25	m	0-12 years	0.986	978.5205204	133	Grand Index
25	m	0-12 years	0.987	983.4759742	133	Grand Index
25	m	0-12 years	0.988	986.8910095	134	Grand Index
25	m	0-12 years	0.989	987.728	134	Grand Index
25	m	0-12 years	0.99	988.3144511	135	Grand Index
25	m	0-12 years	0.991	990.7824895	135	Grand Index
25	m	0-12 years	0.992	997.6781446	136	Grand Index
25	m	0-12 years	0.993	1002.794636	137	Grand Index
25	m	0-12 years	0.994	1004.784437	138	Grand Index
25	m	0-12 years	0.995	1006.203274	139	Grand Index
25	m	0-12 years	0.996	1009.967926	140	Grand Index
25	m	0-12 years	0.997	1012.234042	141	Grand Index
25	m	0-12 years	0.998	1013.992	143	Grand Index
25	m	0-12 years	0.999	1017.701986	146	Grand Index
25	f	13-16 years	0.001	574.4762235	54	Grand Index
25	f	13-16 years	0.002	594.8926528	57	Grand Index
25	f	13-16 years	0.003	599.552	59	Grand Index
25	f	13-16 years	0.004	610.6778898	60	Grand Index
25	f	13-16 years	0.005	612.6833793	61	Grand Index
25	f	13-16 years	0.006	616.913653	62	Grand Index

Age	Gender	Education	Percentile	Raw Score	Norm Score	Assessment
25	f	13-16 years	0.007	623.2110541	63	Grand Index
25	f	13-16 years	0.008	624.527635	64	Grand Index
25	f	13-16 years	0.009	627.212603	65	Grand Index
25	f	13-16 years	0.01	628.9920121	65	Grand Index
25	f	13-16 years	0.011	634.0730966	66	Grand Index
25	f	13-16 years	0.012	637.5931801	66	Grand Index
25	f	13-16 years	0.013	641	67	Grand Index
25	f	13-16 years	0.014	643.576	67	Grand Index
25	f	13-16 years	0.015	647	67	Grand Index
25	f	13-16 years	0.016	650.0580216	68	Grand Index
25	f	13-16 years	0.017	653.3302082	68	Grand Index
25	f	13-16 years	0.018	656.312	69	Grand Index
25	f	13-16 years	0.019	657.496	69	Grand Index
25	f	13-16 years	0.02	659.3228567	69	Grand Index
25	f	13-16 years	0.021	661.0218123	69	Grand Index
25	f	13-16 years	0.022	663	70	Grand Index
25	f	13-16 years	0.023	664	70	Grand Index
25	f	13-16 years	0.024	665.416	70	Grand Index
25	f	13-16 years	0.025	667	71	Grand Index
25	f	13-16 years	0.026	667.784	71	Grand Index
25	f	13-16 years	0.027	668.3183062	71	Grand Index
25	f	13-16 years	0.028	669	71	Grand Index
25	f	13-16 years	0.029	669.6605728	72	Grand Index
25	f	13-16 years	0.03	670.6909885	72	Grand Index
25	f	13-16 years	0.031	672.8379353	72	Grand Index
25	f	13-16 years	0.032	673.888	72	Grand Index
25	f	13-16 years	0.033	676	72	Grand Index
25	f	13-16 years	0.034	677	73	Grand Index
25	f	13-16 years	0.035	678	73	Grand Index
25	f	13-16 years	0.036	679.624	73	Grand Index
25	f	13-16 years	0.037	681	73	Grand Index
25	f	13-16 years	0.038	681.6866441	73	Grand Index
25	f	13-16 years	0.039	682	74	Grand Index
25	f	13-16 years	0.04	683	74	Grand Index
25	f	13-16 years	0.041	684.544	74	Grand Index
25	f	13-16 years	0.042	685.8289187	74	Grand Index
25	f	13-16 years	0.043	686.5420673	74	Grand Index
25	f	13-16 years	0.044	687.0300837	74	Grand Index
25	f	13-16 years	0.045	688	75	Grand Index
25	f	13-16 years	0.046	688.0639212	75	Grand Index
25	f	13-16 years	0.047	689	75	Grand Index
25	f	13-16 years	0.048	689.1114617	75	Grand Index
25	f	13-16 years	0.049	689.6386939	75	Grand Index
25	f	13-16 years	0.05	691.0282749	75	Grand Index
25	f	13-16 years	0.051	692	75	Grand Index
25	f	13-16 years	0.052	692.6184267	76	Grand Index
25	f	13-16 years	0.053	693.0201058	76	Grand Index
25	f	13-16 years	0.054	694.936	76	Grand Index
25	f	13-16 years	0.055	696	76	Grand Index
25	f	13-16 years	0.056	696.2298924	76	Grand Index
25	f	13-16 years	0.057	696.8357964	76	Grand Index
25	f	13-16 years	0.058	697	76	Grand Index
25	f	13-16 years	0.059	697.856	77	Grand Index
25	f	13-16 years	0.06	698.8749003	77	Grand Index
25	f	13-16 years	0.061	699.5023872	77	Grand Index
25	f	13-16 years	0.062	700	77	Grand Index

Age	Gender	Education	Percentile	Raw Score	Norm Score	Assessment
25	f	13-16 years	0.063	700.6928318	77	Grand Index
25	f	13-16 years	0.064	702	77	Grand Index
25	f	13-16 years	0.065	702.1156429	77	Grand Index
25	f	13-16 years	0.066	703.0065387	77	Grand Index
25	f	13-16 years	0.067	704	78	Grand Index
25	f	13-16 years	0.068	704.512	78	Grand Index
25	f	13-16 years	0.069	705	78	Grand Index
25	f	13-16 years	0.07	705.88	78	Grand Index
25	f	13-16 years	0.072	706	78	Grand Index
25	f	13-16 years	0.073	706.2366285	78	Grand Index
25	f	13-16 years	0.074	707	78	Grand Index
25	f	13-16 years	0.075	707.9630218	78	Grand Index
25	f	13-16 years	0.076	708.984	79	Grand Index
25	f	13-16 years	0.077	709	79	Grand Index
25	f	13-16 years	0.08	710	79	Grand Index
25	f	13-16 years	0.081	711	79	Grand Index
25	f	13-16 years	0.084	712	79	Grand Index
25	f	13-16 years	0.085	712.6443009	79	Grand Index
25	f	13-16 years	0.086	713	80	Grand Index
25	f	13-16 years	0.087	713.0336625	80	Grand Index
25	f	13-16 years	0.088	714	80	Grand Index
25	f	13-16 years	0.089	714.9433615	80	Grand Index
25	f	13-16 years	0.091	715	80	Grand Index
25	f	13-16 years	0.092	715.8833285	80	Grand Index
25	f	13-16 years	0.094	716	80	Grand Index
25	f	13-16 years	0.096	717	80	Grand Index
25	f	13-16 years	0.097	718	81	Grand Index
25	f	13-16 years	0.098	718.9985878	81	Grand Index
25	f	13-16 years	0.1	719	81	Grand Index
25	f	13-16 years	0.101	719.6921988	81	Grand Index
25	f	13-16 years	0.103	720	81	Grand Index
25	f	13-16 years	0.105	721	81	Grand Index
25	f	13-16 years	0.106	721.504	81	Grand Index
25	f	13-16 years	0.108	722	81	Grand Index
25	f	13-16 years	0.111	723	82	Grand Index
25	f	13-16 years	0.112	723.6000365	82	Grand Index
25	f	13-16 years	0.113	724	82	Grand Index
25	f	13-16 years	0.114	724.9848538	82	Grand Index
25	f	13-16 years	0.115	725	82	Grand Index
25	f	13-16 years	0.116	725.2273785	82	Grand Index
25	f	13-16 years	0.119	726	82	Grand Index
25	f	13-16 years	0.12	727	82	Grand Index
25	f	13-16 years	0.121	727.1555349	82	Grand Index
25	f	13-16 years	0.122	728	83	Grand Index
25	f	13-16 years	0.123	728.5055579	83	Grand Index
25	f	13-16 years	0.126	729	83	Grand Index
25	f	13-16 years	0.127	729.17321	83	Grand Index
25	f	13-16 years	0.13	730	83	Grand Index
25	f	13-16 years	0.131	730.7793769	83	Grand Index
25	f	13-16 years	0.133	731	83	Grand Index
25	f	13-16 years	0.134	731.656	83	Grand Index
25	f	13-16 years	0.135	732.7544834	83	Grand Index
25	f	13-16 years	0.137	733	84	Grand Index
25	f	13-16 years	0.138	733.0048958	84	Grand Index
25	f	13-16 years	0.139	733.9263641	84	Grand Index
25	f	13-16 years	0.142	734	84	Grand Index

Age	Gender	Education	Percentile	Raw Score	Norm Score	Assessment
25	f	13-16 years	0.144	735	84	Grand Index
25	f	13-16 years	0.145	735.1633892	84	Grand Index
25	f	13-16 years	0.146	735.9250507	84	Grand Index
25	f	13-16 years	0.147	736.0341191	84	Grand Index
25	f	13-16 years	0.148	736.9979418	84	Grand Index
25	f	13-16 years	0.15	737	84	Grand Index
25	f	13-16 years	0.151	737.7329637	85	Grand Index
25	f	13-16 years	0.154	738	85	Grand Index
25	f	13-16 years	0.155	738.285856	85	Grand Index
25	f	13-16 years	0.158	739	85	Grand Index
25	f	13-16 years	0.159	739.5121077	85	Grand Index
25	f	13-16 years	0.161	740	85	Grand Index
25	f	13-16 years	0.165	741	85	Grand Index
25	f	13-16 years	0.166	741.3933272	85	Grand Index
25	f	13-16 years	0.168	742	86	Grand Index
25	f	13-16 years	0.169	742.096	86	Grand Index
25	f	13-16 years	0.173	743	86	Grand Index
25	f	13-16 years	0.174	743.1224641	86	Grand Index
25	f	13-16 years	0.177	744	86	Grand Index
25	f	13-16 years	0.178	744.0104937	86	Grand Index
25	f	13-16 years	0.182	745	86	Grand Index
25	f	13-16 years	0.183	745.7649682	86	Grand Index
25	f	13-16 years	0.186	746	87	Grand Index
25	f	13-16 years	0.188	747	87	Grand Index
25	f	13-16 years	0.192	748	87	Grand Index
25	f	13-16 years	0.193	748.6142402	87	Grand Index
25	f	13-16 years	0.196	749	87	Grand Index
25	f	13-16 years	0.197	749.9868221	87	Grand Index
25	f	13-16 years	0.2	750	87	Grand Index
25	f	13-16 years	0.201	750.4958395	87	Grand Index
25	f	13-16 years	0.204	751	88	Grand Index
25	f	13-16 years	0.207	752	88	Grand Index
25	f	13-16 years	0.208	752.1844589	88	Grand Index
25	f	13-16 years	0.212	753	88	Grand Index
25	f	13-16 years	0.213	753.702972	88	Grand Index
25	f	13-16 years	0.217	754	88	Grand Index
25	f	13-16 years	0.222	755	89	Grand Index
25	f	13-16 years	0.223	755.9856845	89	Grand Index
25	f	13-16 years	0.226	756	89	Grand Index
25	f	13-16 years	0.23	757	89	Grand Index
25	f	13-16 years	0.231	757.7155286	89	Grand Index
25	f	13-16 years	0.237	758	89	Grand Index
25	f	13-16 years	0.238	758.3603577	89	Grand Index
25	f	13-16 years	0.241	759	89	Grand Index
25	f	13-16 years	0.242	759.0082821	90	Grand Index
25	f	13-16 years	0.246	760	90	Grand Index
25	f	13-16 years	0.247	760.3556398	90	Grand Index
25	f	13-16 years	0.25	761	90	Grand Index
25	f	13-16 years	0.251	761.6807994	90	Grand Index
25	f	13-16 years	0.254	762	90	Grand Index
25	f	13-16 years	0.255	762.6076641	90	Grand Index
25	f	13-16 years	0.258	763	90	Grand Index
25	f	13-16 years	0.263	764	90	Grand Index
25	f	13-16 years	0.266	765	91	Grand Index
25	f	13-16 years	0.267	765.8206351	91	Grand Index
25	f	13-16 years	0.271	766	91	Grand Index

Age	Gender	Education	Percentile	Raw Score	Norm Score	Assessment
25	f	13-16 years	0.273	767	91	Grand Index
25	f	13-16 years	0.274	767.4818544	91	Grand Index
25	f	13-16 years	0.278	768	91	Grand Index
25	f	13-16 years	0.279	768.4207267	91	Grand Index
25	f	13-16 years	0.281	769	91	Grand Index
25	f	13-16 years	0.282	769.9997955	91	Grand Index
25	f	13-16 years	0.285	770	91	Grand Index
25	f	13-16 years	0.286	770.0263881	92	Grand Index
25	f	13-16 years	0.29	771	92	Grand Index
25	f	13-16 years	0.293	772	92	Grand Index
25	f	13-16 years	0.294	772.3481861	92	Grand Index
25	f	13-16 years	0.298	773	92	Grand Index
25	f	13-16 years	0.299	773.1776776	92	Grand Index
25	f	13-16 years	0.303	774	92	Grand Index
25	f	13-16 years	0.307	775	92	Grand Index
25	f	13-16 years	0.308	775.7863684	92	Grand Index
25	f	13-16 years	0.312	776	93	Grand Index
25	f	13-16 years	0.313	776.4356666	93	Grand Index
25	f	13-16 years	0.319	777	93	Grand Index
25	f	13-16 years	0.32	777.9719917	93	Grand Index
25	f	13-16 years	0.323	778	93	Grand Index
25	f	13-16 years	0.324	778.0801791	93	Grand Index
25	f	13-16 years	0.327	779	93	Grand Index
25	f	13-16 years	0.328	779.9220301	93	Grand Index
25	f	13-16 years	0.331	780	93	Grand Index
25	f	13-16 years	0.334	781	94	Grand Index
25	f	13-16 years	0.335	781.3967175	94	Grand Index
25	f	13-16 years	0.34	782	94	Grand Index
25	f	13-16 years	0.341	782.7054792	94	Grand Index
25	f	13-16 years	0.345	783	94	Grand Index
25	f	13-16 years	0.346	783.0057171	94	Grand Index
25	f	13-16 years	0.347	783.9193078	94	Grand Index
25	f	13-16 years	0.35	784	94	Grand Index
25	f	13-16 years	0.351	784.2611848	94	Grand Index
25	f	13-16 years	0.352	784.7906464	94	Grand Index
25	f	13-16 years	0.358	785	95	Grand Index
25	f	13-16 years	0.361	786	95	Grand Index
25	f	13-16 years	0.362	786.8099704	95	Grand Index
25	f	13-16 years	0.365	787	95	Grand Index
25	f	13-16 years	0.366	787.0392981	95	Grand Index
25	f	13-16 years	0.369	788	95	Grand Index
25	f	13-16 years	0.37	788.0179231	95	Grand Index
25	f	13-16 years	0.376	789	95	Grand Index
25	f	13-16 years	0.384	790	96	Grand Index
25	f	13-16 years	0.385	790.5256532	96	Grand Index
25	f	13-16 years	0.389	791	96	Grand Index
25	f	13-16 years	0.391	792	96	Grand Index
25	f	13-16 years	0.394	793	96	Grand Index
25	f	13-16 years	0.395	793.0103343	96	Grand Index
25	f	13-16 years	0.396	793.8695275	96	Grand Index
25	f	13-16 years	0.402	794	96	Grand Index
25	f	13-16 years	0.406	795	96	Grand Index
25	f	13-16 years	0.407	795.8863855	96	Grand Index
25	f	13-16 years	0.411	796	97	Grand Index
25	f	13-16 years	0.417	797	97	Grand Index
25	f	13-16 years	0.424	798	97	Grand Index

Age	Gender	Education	Percentile	Raw Score	Norm Score	Assessment
25	f	13-16 years	0.428	799	97	Grand Index
25	f	13-16 years	0.429	799.0523947	97	Grand Index
25	f	13-16 years	0.432	800	97	Grand Index
25	f	13-16 years	0.433	800.1695041	97	Grand Index
25	f	13-16 years	0.438	801	98	Grand Index
25	f	13-16 years	0.446	802	98	Grand Index
25	f	13-16 years	0.453	803	98	Grand Index
25	f	13-16 years	0.454	803.926503	98	Grand Index
25	f	13-16 years	0.457	804	98	Grand Index
25	f	13-16 years	0.458	804.7559946	98	Grand Index
25	f	13-16 years	0.463	805	99	Grand Index
25	f	13-16 years	0.464	805.6112845	99	Grand Index
25	f	13-16 years	0.468	806	99	Grand Index
25	f	13-16 years	0.469	806.6781376	99	Grand Index
25	f	13-16 years	0.474	807	99	Grand Index
25	f	13-16 years	0.475	807.7679445	99	Grand Index
25	f	13-16 years	0.48	808	99	Grand Index
25	f	13-16 years	0.481	808.0251829	99	Grand Index
25	f	13-16 years	0.482	808.9804908	99	Grand Index
25	f	13-16 years	0.485	809	99	Grand Index
25	f	13-16 years	0.488	810	100	Grand Index
25	f	13-16 years	0.489	810.379608	100	Grand Index
25	f	13-16 years	0.494	811	100	Grand Index
25	f	13-16 years	0.495	811.9989449	100	Grand Index
25	f	13-16 years	0.499	812	100	Grand Index
25	f	13-16 years	0.5	812.8314856	100	Grand Index
25	f	13-16 years	0.505	813	100	Grand Index
25	f	13-16 years	0.506	813.0310302	100	Grand Index
25	f	13-16 years	0.51	814	100	Grand Index
25	f	13-16 years	0.511	814.8367245	100	Grand Index
25	f	13-16 years	0.515	815	101	Grand Index
25	f	13-16 years	0.516	815.944	101	Grand Index
25	f	13-16 years	0.52	816	101	Grand Index
25	f	13-16 years	0.521	816.7407729	101	Grand Index
25	f	13-16 years	0.527	817	101	Grand Index
25	f	13-16 years	0.528	817.0334781	101	Grand Index
25	f	13-16 years	0.532	818	101	Grand Index
25	f	13-16 years	0.533	818.2051426	101	Grand Index
25	f	13-16 years	0.538	819	101	Grand Index
25	f	13-16 years	0.539	819.8657713	101	Grand Index
25	f	13-16 years	0.542	820	102	Grand Index
25	f	13-16 years	0.543	820.3413786	102	Grand Index
25	f	13-16 years	0.547	821	102	Grand Index
25	f	13-16 years	0.548	821.99622	102	Grand Index
25	f	13-16 years	0.553	822	102	Grand Index
25	f	13-16 years	0.556	823	102	Grand Index
25	f	13-16 years	0.557	823.488	102	Grand Index
25	f	13-16 years	0.563	824	102	Grand Index
25	f	13-16 years	0.564	824.9964724	102	Grand Index
25	f	13-16 years	0.567	825	103	Grand Index
25	f	13-16 years	0.568	825.8360139	103	Grand Index
25	f	13-16 years	0.573	826	103	Grand Index
25	f	13-16 years	0.574	826.8736015	103	Grand Index
25	f	13-16 years	0.577	827	103	Grand Index
25	f	13-16 years	0.578	827.0391111	103	Grand Index
25	f	13-16 years	0.583	828	103	Grand Index

Age	Gender	Education	Percentile	Raw Score	Norm Score	Assessment
25	f	13-16 years	0.584	828.456	103	Grand Index
25	f	13-16 years	0.587	829	103	Grand Index
25	f	13-16 years	0.588	829.02268	103	Grand Index
25	f	13-16 years	0.589	829.9901732	103	Grand Index
25	f	13-16 years	0.593	830	104	Grand Index
25	f	13-16 years	0.594	830.2287297	104	Grand Index
25	f	13-16 years	0.595	830.9268777	104	Grand Index
25	f	13-16 years	0.599	831	104	Grand Index
25	f	13-16 years	0.6	831.9890216	104	Grand Index
25	f	13-16 years	0.604	832	104	Grand Index
25	f	13-16 years	0.611	833	104	Grand Index
25	f	13-16 years	0.612	833.7959362	104	Grand Index
25	f	13-16 years	0.614	834	104	Grand Index
25	f	13-16 years	0.615	834.3683444	104	Grand Index
25	f	13-16 years	0.619	835	105	Grand Index
25	f	13-16 years	0.62	835.046027	105	Grand Index
25	f	13-16 years	0.625	836	105	Grand Index
25	f	13-16 years	0.632	837	105	Grand Index
25	f	13-16 years	0.633	837.813624	105	Grand Index
25	f	13-16 years	0.637	838	105	Grand Index
25	f	13-16 years	0.638	838.8875563	105	Grand Index
25	f	13-16 years	0.642	839	105	Grand Index
25	f	13-16 years	0.643	839.3211109	105	Grand Index
25	f	13-16 years	0.646	840	106	Grand Index
25	f	13-16 years	0.65	841	106	Grand Index
25	f	13-16 years	0.651	841.3144358	106	Grand Index
25	f	13-16 years	0.655	842	106	Grand Index
25	f	13-16 years	0.656	842.7485764	106	Grand Index
25	f	13-16 years	0.661	843	106	Grand Index
25	f	13-16 years	0.662	843.7923082	106	Grand Index
25	f	13-16 years	0.668	844	107	Grand Index
25	f	13-16 years	0.669	844.8661714	107	Grand Index
25	f	13-16 years	0.673	845	107	Grand Index
25	f	13-16 years	0.674	845.5580265	107	Grand Index
25	f	13-16 years	0.678	846	107	Grand Index
25	f	13-16 years	0.679	846.6106848	107	Grand Index
25	f	13-16 years	0.682	847	107	Grand Index
25	f	13-16 years	0.683	847.672	107	Grand Index
25	f	13-16 years	0.687	848	107	Grand Index
25	f	13-16 years	0.688	848.1977337	107	Grand Index
25	f	13-16 years	0.692	849	108	Grand Index
25	f	13-16 years	0.699	850	108	Grand Index
25	f	13-16 years	0.701	851	108	Grand Index
25	f	13-16 years	0.702	851.0352114	108	Grand Index
25	f	13-16 years	0.703	851.6643324	108	Grand Index
25	f	13-16 years	0.707	852	108	Grand Index
25	f	13-16 years	0.708	852.0809608	108	Grand Index
25	f	13-16 years	0.709	852.966389	108	Grand Index
25	f	13-16 years	0.713	853	108	Grand Index
25	f	13-16 years	0.714	853.0436514	108	Grand Index
25	f	13-16 years	0.715	853.5873463	109	Grand Index
25	f	13-16 years	0.719	854	109	Grand Index
25	f	13-16 years	0.72	854.9895863	109	Grand Index
25	f	13-16 years	0.721	855	109	Grand Index
25	f	13-16 years	0.727	856	109	Grand Index
25	f	13-16 years	0.728	856.9999054	109	Grand Index

Age	Gender	Education	Percentile	Raw Score	Norm Score	Assessment
25	f	13-16 years	0.733	857	109	Grand Index
25	f	13-16 years	0.735	858	109	Grand Index
25	f	13-16 years	0.738	859	110	Grand Index
25	f	13-16 years	0.739	859.9787191	110	Grand Index
25	f	13-16 years	0.741	860	110	Grand Index
25	f	13-16 years	0.742	860.528	110	Grand Index
25	f	13-16 years	0.746	861	110	Grand Index
25	f	13-16 years	0.747	861.4171415	110	Grand Index
25	f	13-16 years	0.751	862	110	Grand Index
25	f	13-16 years	0.752	862.006149	110	Grand Index
25	f	13-16 years	0.757	863	110	Grand Index
25	f	13-16 years	0.758	863.7063069	110	Grand Index
25	f	13-16 years	0.76	864	111	Grand Index
25	f	13-16 years	0.761	864.024	111	Grand Index
25	f	13-16 years	0.764	865	111	Grand Index
25	f	13-16 years	0.765	865.4390554	111	Grand Index
25	f	13-16 years	0.766	866	111	Grand Index
25	f	13-16 years	0.767	866.009776	111	Grand Index
25	f	13-16 years	0.768	866.3695733	111	Grand Index
25	f	13-16 years	0.773	867	111	Grand Index
25	f	13-16 years	0.774	867.2606844	111	Grand Index
25	f	13-16 years	0.777	868	111	Grand Index
25	f	13-16 years	0.78	869	112	Grand Index
25	f	13-16 years	0.784	870	112	Grand Index
25	f	13-16 years	0.785	870.3944047	112	Grand Index
25	f	13-16 years	0.788	871	112	Grand Index
25	f	13-16 years	0.791	872	112	Grand Index
25	f	13-16 years	0.792	872.9769335	112	Grand Index
25	f	13-16 years	0.794	873	112	Grand Index
25	f	13-16 years	0.796	874	112	Grand Index
25	f	13-16 years	0.797	874.9809953	112	Grand Index
25	f	13-16 years	0.801	875	113	Grand Index
25	f	13-16 years	0.804	876	113	Grand Index
25	f	13-16 years	0.805	876.19708	113	Grand Index
25	f	13-16 years	0.806	876.767624	113	Grand Index
25	f	13-16 years	0.809	877	113	Grand Index
25	f	13-16 years	0.81	877.2037296	113	Grand Index
25	f	13-16 years	0.812	878	113	Grand Index
25	f	13-16 years	0.813	878.4492343	113	Grand Index
25	f	13-16 years	0.814	879	113	Grand Index
25	f	13-16 years	0.815	879.3177189	113	Grand Index
25	f	13-16 years	0.818	880	114	Grand Index
25	f	13-16 years	0.819	880.4492264	114	Grand Index
25	f	13-16 years	0.822	881	114	Grand Index
25	f	13-16 years	0.824	882	114	Grand Index
25	f	13-16 years	0.825	882.9260644	114	Grand Index
25	f	13-16 years	0.826	883	114	Grand Index
25	f	13-16 years	0.828	884	114	Grand Index
25	f	13-16 years	0.829	884.2971175	114	Grand Index
25	f	13-16 years	0.831	885	114	Grand Index
25	f	13-16 years	0.832	885.0003738	114	Grand Index
25	f	13-16 years	0.834	886	115	Grand Index
25	f	13-16 years	0.835	886.0486149	115	Grand Index
25	f	13-16 years	0.836	886.9794173	115	Grand Index
25	f	13-16 years	0.837	887	115	Grand Index
25	f	13-16 years	0.838	887.192	115	Grand Index

Age	Gender	Education	Percentile	Raw Score	Norm Score	Assessment
25	f	13-16 years	0.839	888	115	Grand Index
25	f	13-16 years	0.84	888.2807107	115	Grand Index
25	f	13-16 years	0.841	889.182088	115	Grand Index
25	f	13-16 years	0.845	890	115	Grand Index
25	f	13-16 years	0.846	890.7355049	115	Grand Index
25	f	13-16 years	0.85	891	116	Grand Index
25	f	13-16 years	0.851	891.9099173	116	Grand Index
25	f	13-16 years	0.854	892	116	Grand Index
25	f	13-16 years	0.855	892.7933997	116	Grand Index
25	f	13-16 years	0.857	893	116	Grand Index
25	f	13-16 years	0.858	893.9982948	116	Grand Index
25	f	13-16 years	0.86	894	116	Grand Index
25	f	13-16 years	0.861	894.85204	116	Grand Index
25	f	13-16 years	0.864	895	116	Grand Index
25	f	13-16 years	0.865	895.373625	117	Grand Index
25	f	13-16 years	0.867	896	117	Grand Index
25	f	13-16 years	0.868	896.9077769	117	Grand Index
25	f	13-16 years	0.87	897	117	Grand Index
25	f	13-16 years	0.872	898	117	Grand Index
25	f	13-16 years	0.875	899	117	Grand Index
25	f	13-16 years	0.878	900	117	Grand Index
25	f	13-16 years	0.88	901	118	Grand Index
25	f	13-16 years	0.882	902	118	Grand Index
25	f	13-16 years	0.884	903	118	Grand Index
25	f	13-16 years	0.885	903.657063	118	Grand Index
25	f	13-16 years	0.886	904	118	Grand Index
25	f	13-16 years	0.889	905	118	Grand Index
25	f	13-16 years	0.891	906	118	Grand Index
25	f	13-16 years	0.892	906.087811	119	Grand Index
25	f	13-16 years	0.893	906.5633066	119	Grand Index
25	f	13-16 years	0.894	907	119	Grand Index
25	f	13-16 years	0.895	907.6691012	119	Grand Index
25	f	13-16 years	0.896	908	119	Grand Index
25	f	13-16 years	0.897	908.0129123	119	Grand Index
25	f	13-16 years	0.898	908.9232808	119	Grand Index
25	f	13-16 years	0.899	909	119	Grand Index
25	f	13-16 years	0.9	909.0561458	119	Grand Index
25	f	13-16 years	0.901	909.9954906	119	Grand Index
25	f	13-16 years	0.902	910.4186714	119	Grand Index
25	f	13-16 years	0.904	911	120	Grand Index
25	f	13-16 years	0.906	912	120	Grand Index
25	f	13-16 years	0.907	912.8300445	120	Grand Index
25	f	13-16 years	0.908	913	120	Grand Index
25	f	13-16 years	0.909	913.742911	120	Grand Index
25	f	13-16 years	0.911	914	120	Grand Index
25	f	13-16 years	0.912	914.3272813	120	Grand Index
25	f	13-16 years	0.913	915.992	120	Grand Index
25	f	13-16 years	0.914	916	120	Grand Index
25	f	13-16 years	0.915	917	121	Grand Index
25	f	13-16 years	0.916	917.6201231	121	Grand Index
25	f	13-16 years	0.917	918.728	121	Grand Index
25	f	13-16 years	0.918	919.6315278	121	Grand Index
25	f	13-16 years	0.92	920	121	Grand Index
25	f	13-16 years	0.921	921.9947961	121	Grand Index
25	f	13-16 years	0.922	922.145978	121	Grand Index
25	f	13-16 years	0.923	923	121	Grand Index

Age	Gender	Education	Percentile	Raw Score	Norm Score	Assessment
25	f	13-16 years	0.924	923.0154354	121	Grand Index
25	f	13-16 years	0.926	924	122	Grand Index
25	f	13-16 years	0.927	924.568	122	Grand Index
25	f	13-16 years	0.928	925	122	Grand Index
25	f	13-16 years	0.929	925.9467038	122	Grand Index
25	f	13-16 years	0.93	926	122	Grand Index
25	f	13-16 years	0.931	926.6697165	122	Grand Index
25	f	13-16 years	0.932	927	122	Grand Index
25	f	13-16 years	0.934	928	123	Grand Index
25	f	13-16 years	0.935	928.8949088	123	Grand Index
25	f	13-16 years	0.937	929	123	Grand Index
25	f	13-16 years	0.939	930	123	Grand Index
25	f	13-16 years	0.94	930.6159173	123	Grand Index
25	f	13-16 years	0.941	931	123	Grand Index
25	f	13-16 years	0.943	932	124	Grand Index
25	f	13-16 years	0.944	932.696	124	Grand Index
25	f	13-16 years	0.945	933.2913514	124	Grand Index
25	f	13-16 years	0.946	933.7781395	124	Grand Index
25	f	13-16 years	0.947	934.444475	124	Grand Index
25	f	13-16 years	0.948	935.2889808	124	Grand Index
25	f	13-16 years	0.949	936.4082561	125	Grand Index
25	f	13-16 years	0.951	937	125	Grand Index
25	f	13-16 years	0.952	939	125	Grand Index
25	f	13-16 years	0.953	939.2591002	125	Grand Index
25	f	13-16 years	0.954	939.4282985	125	Grand Index
25	f	13-16 years	0.956	940	126	Grand Index
25	f	13-16 years	0.957	941.7945158	126	Grand Index
25	f	13-16 years	0.958	943	126	Grand Index
25	f	13-16 years	0.959	944	126	Grand Index
25	f	13-16 years	0.96	945.7857135	126	Grand Index
25	f	13-16 years	0.961	946.5318428	126	Grand Index
25	f	13-16 years	0.962	947.008	127	Grand Index
25	f	13-16 years	0.963	949	127	Grand Index
25	f	13-16 years	0.964	949.8718964	127	Grand Index
25	f	13-16 years	0.965	950	127	Grand Index
25	f	13-16 years	0.966	950.7746411	127	Grand Index
25	f	13-16 years	0.967	951	128	Grand Index
25	f	13-16 years	0.968	952	128	Grand Index
25	f	13-16 years	0.969	953	128	Grand Index
25	f	13-16 years	0.97	954.3339629	128	Grand Index
25	f	13-16 years	0.971	954.8605594	128	Grand Index
25	f	13-16 years	0.972	955.0784813	129	Grand Index
25	f	13-16 years	0.973	956.0290116	129	Grand Index
25	f	13-16 years	0.974	957.2840547	129	Grand Index
25	f	13-16 years	0.975	958.1021277	129	Grand Index
25	f	13-16 years	0.976	959.584	130	Grand Index
25	f	13-16 years	0.977	960.0194924	130	Grand Index
25	f	13-16 years	0.978	961	130	Grand Index
25	f	13-16 years	0.979	963.0413669	131	Grand Index
25	f	13-16 years	0.98	965	131	Grand Index
25	f	13-16 years	0.981	965.4708835	131	Grand Index
25	f	13-16 years	0.982	967.2116006	131	Grand Index
25	f	13-16 years	0.983	968.3482601	132	Grand Index
25	f	13-16 years	0.984	970.1868697	132	Grand Index
25	f	13-16 years	0.985	973.1494221	133	Grand Index
25	f	13-16 years	0.986	975	133	Grand Index

Age	Gender	Education	Percentile	Raw Score	Norm Score	Assessment
25	f	13-16 years	0.987	976.9665243	133	Grand Index
25	f	13-16 years	0.988	980	134	Grand Index
25	f	13-16 years	0.989	981.4497515	134	Grand Index
25	f	13-16 years	0.99	984.9547798	135	Grand Index
25	f	13-16 years	0.991	988.736765	135	Grand Index
25	f	13-16 years	0.992	990.4736471	136	Grand Index
25	f	13-16 years	0.993	994.8493426	137	Grand Index
25	f	13-16 years	0.994	1003.792	138	Grand Index
25	f	13-16 years	0.995	1007.24	139	Grand Index
25	f	13-16 years	0.996	1012.264	140	Grand Index
25	f	13-16 years	0.997	1015.823045	141	Grand Index
25	f	13-16 years	0.998	1021.632	143	Grand Index
25	f	13-16 years	0.999	1029.5933	146	Grand Index
25	m	13-16 years	0.001	594.516	54	Grand Index
25	m	13-16 years	0.002	615.0946327	57	Grand Index
25	m	13-16 years	0.003	620.774	59	Grand Index
25	m	13-16 years	0.004	626.032	60	Grand Index
25	m	13-16 years	0.005	630	61	Grand Index
25	m	13-16 years	0.006	630.7117	62	Grand Index
25	m	13-16 years	0.007	634.7383835	63	Grand Index
25	m	13-16 years	0.008	638.3820873	64	Grand Index
25	m	13-16 years	0.009	639.6948928	65	Grand Index
25	m	13-16 years	0.01	642	65	Grand Index
25	m	13-16 years	0.011	643	66	Grand Index
25	m	13-16 years	0.012	645.5176372	66	Grand Index
25	m	13-16 years	0.013	648.0287607	67	Grand Index
25	m	13-16 years	0.014	649	67	Grand Index
25	m	13-16 years	0.015	649.9410335	67	Grand Index
25	m	13-16 years	0.016	654	68	Grand Index
25	m	13-16 years	0.017	655.909614	68	Grand Index
25	m	13-16 years	0.018	659	69	Grand Index
25	m	13-16 years	0.019	660	69	Grand Index
25	m	13-16 years	0.02	660.4645261	69	Grand Index
25	m	13-16 years	0.021	661	69	Grand Index
25	m	13-16 years	0.022	662.6276853	70	Grand Index
25	m	13-16 years	0.023	663.0064416	70	Grand Index
25	m	13-16 years	0.024	665.8187455	70	Grand Index
25	m	13-16 years	0.025	667	71	Grand Index
25	m	13-16 years	0.026	667.8762409	71	Grand Index
25	m	13-16 years	0.027	669	71	Grand Index
25	m	13-16 years	0.028	670	71	Grand Index
25	m	13-16 years	0.029	670.3680127	72	Grand Index
25	m	13-16 years	0.03	671	72	Grand Index
25	m	13-16 years	0.031	672	72	Grand Index
25	m	13-16 years	0.032	672.1849635	72	Grand Index
25	m	13-16 years	0.033	672.9967816	72	Grand Index
25	m	13-16 years	0.034	673.4430716	73	Grand Index
25	m	13-16 years	0.035	674	73	Grand Index
25	m	13-16 years	0.036	675.1586417	73	Grand Index
25	m	13-16 years	0.037	677.0177922	73	Grand Index
25	m	13-16 years	0.038	677.9269338	73	Grand Index
25	m	13-16 years	0.039	679	74	Grand Index
25	m	13-16 years	0.04	680	74	Grand Index
25	m	13-16 years	0.041	680.9920265	74	Grand Index
25	m	13-16 years	0.042	681	74	Grand Index
25	m	13-16 years	0.043	682	74	Grand Index

Age	Gender	Education	Percentile	Raw Score	Norm Score	Assessment
25	m	13-16 years	0.044	683	74	Grand Index
25	m	13-16 years	0.045	683.6956565	75	Grand Index
25	m	13-16 years	0.046	684	75	Grand Index
25	m	13-16 years	0.047	684.0049158	75	Grand Index
25	m	13-16 years	0.048	685	75	Grand Index
25	m	13-16 years	0.05	686	75	Grand Index
25	m	13-16 years	0.051	687	75	Grand Index
25	m	13-16 years	0.052	687.1227604	76	Grand Index
25	m	13-16 years	0.053	688.0164404	76	Grand Index
25	m	13-16 years	0.054	689	76	Grand Index
25	m	13-16 years	0.055	689.0784151	76	Grand Index
25	m	13-16 years	0.056	690	76	Grand Index
25	m	13-16 years	0.057	691	76	Grand Index
25	m	13-16 years	0.058	691.8443128	76	Grand Index
25	m	13-16 years	0.059	692.8805198	77	Grand Index
25	m	13-16 years	0.06	693.0586367	77	Grand Index
25	m	13-16 years	0.061	695	77	Grand Index
25	m	13-16 years	0.062	695.0987003	77	Grand Index
25	m	13-16 years	0.063	696	77	Grand Index
25	m	13-16 years	0.064	696.8086059	77	Grand Index
25	m	13-16 years	0.065	697.7036037	77	Grand Index
25	m	13-16 years	0.066	698	77	Grand Index
25	m	13-16 years	0.067	699	78	Grand Index
25	m	13-16 years	0.068	699.87231	78	Grand Index
25	m	13-16 years	0.069	700	78	Grand Index
25	m	13-16 years	0.071	701	78	Grand Index
25	m	13-16 years	0.072	701.1554308	78	Grand Index
25	m	13-16 years	0.073	702	78	Grand Index
25	m	13-16 years	0.074	703	78	Grand Index
25	m	13-16 years	0.076	704	79	Grand Index
25	m	13-16 years	0.077	704.846323	79	Grand Index
25	m	13-16 years	0.078	705	79	Grand Index
25	m	13-16 years	0.079	705.2346602	79	Grand Index
25	m	13-16 years	0.08	706	79	Grand Index
25	m	13-16 years	0.081	706.3644998	79	Grand Index
25	m	13-16 years	0.082	707.0701999	79	Grand Index
25	m	13-16 years	0.083	708	79	Grand Index
25	m	13-16 years	0.084	708.5708981	79	Grand Index
25	m	13-16 years	0.086	709	80	Grand Index
25	m	13-16 years	0.087	709.2258679	80	Grand Index
25	m	13-16 years	0.089	710	80	Grand Index
25	m	13-16 years	0.091	711	80	Grand Index
25	m	13-16 years	0.094	712	80	Grand Index
25	m	13-16 years	0.095	713	80	Grand Index
25	m	13-16 years	0.096	713.4115481	80	Grand Index
25	m	13-16 years	0.099	714	81	Grand Index
25	m	13-16 years	0.1	714.0828173	81	Grand Index
25	m	13-16 years	0.102	715	81	Grand Index
25	m	13-16 years	0.103	715.5910663	81	Grand Index
25	m	13-16 years	0.104	716	81	Grand Index
25	m	13-16 years	0.105	716.8246275	81	Grand Index
25	m	13-16 years	0.107	717	81	Grand Index
25	m	13-16 years	0.108	717.6795342	81	Grand Index
25	m	13-16 years	0.11	718	82	Grand Index
25	m	13-16 years	0.111	718.3299355	82	Grand Index
25	m	13-16 years	0.112	719	82	Grand Index

Age	Gender	Education	Percentile	Raw Score	Norm Score	Assessment
25	m	13-16 years	0.114	720	82	Grand Index
25	m	13-16 years	0.116	721	82	Grand Index
25	m	13-16 years	0.117	721.0271771	82	Grand Index
25	m	13-16 years	0.118	721.8632283	82	Grand Index
25	m	13-16 years	0.119	722	82	Grand Index
25	m	13-16 years	0.12	722.0864195	82	Grand Index
25	m	13-16 years	0.123	723	83	Grand Index
25	m	13-16 years	0.125	724	83	Grand Index
25	m	13-16 years	0.126	724.6240643	83	Grand Index
25	m	13-16 years	0.129	725	83	Grand Index
25	m	13-16 years	0.13	725.6911714	83	Grand Index
25	m	13-16 years	0.133	726	83	Grand Index
25	m	13-16 years	0.134	726.0023662	83	Grand Index
25	m	13-16 years	0.136	727	84	Grand Index
25	m	13-16 years	0.137	727.9799438	84	Grand Index
25	m	13-16 years	0.139	728	84	Grand Index
25	m	13-16 years	0.14	729	84	Grand Index
25	m	13-16 years	0.141	729.2447382	84	Grand Index
25	m	13-16 years	0.143	730	84	Grand Index
25	m	13-16 years	0.144	730.2646678	84	Grand Index
25	m	13-16 years	0.145	730.9945964	84	Grand Index
25	m	13-16 years	0.147	731	84	Grand Index
25	m	13-16 years	0.148	732	84	Grand Index
25	m	13-16 years	0.149	732.4499483	84	Grand Index
25	m	13-16 years	0.152	733	85	Grand Index
25	m	13-16 years	0.153	733.7915945	85	Grand Index
25	m	13-16 years	0.155	734	85	Grand Index
25	m	13-16 years	0.156	734.9701404	85	Grand Index
25	m	13-16 years	0.159	735	85	Grand Index
25	m	13-16 years	0.16	735.9948833	85	Grand Index
25	m	13-16 years	0.163	736	85	Grand Index
25	m	13-16 years	0.164	736.8527067	85	Grand Index
25	m	13-16 years	0.166	737	85	Grand Index
25	m	13-16 years	0.169	738	86	Grand Index
25	m	13-16 years	0.17	738.5394567	86	Grand Index
25	m	13-16 years	0.172	739	86	Grand Index
25	m	13-16 years	0.173	739.634	86	Grand Index
25	m	13-16 years	0.176	740	86	Grand Index
25	m	13-16 years	0.178	741	86	Grand Index
25	m	13-16 years	0.179	741.1464083	86	Grand Index
25	m	13-16 years	0.181	742	86	Grand Index
25	m	13-16 years	0.182	742.9871909	86	Grand Index
25	m	13-16 years	0.185	743	87	Grand Index
25	m	13-16 years	0.188	744	87	Grand Index
25	m	13-16 years	0.189	744.9325789	87	Grand Index
25	m	13-16 years	0.192	745	87	Grand Index
25	m	13-16 years	0.193	745.7686191	87	Grand Index
25	m	13-16 years	0.195	746	87	Grand Index
25	m	13-16 years	0.196	746.7072228	87	Grand Index
25	m	13-16 years	0.199	747	87	Grand Index
25	m	13-16 years	0.201	748	87	Grand Index
25	m	13-16 years	0.202	748.0522664	87	Grand Index
25	m	13-16 years	0.204	749	88	Grand Index
25	m	13-16 years	0.209	750	88	Grand Index
25	m	13-16 years	0.212	751	88	Grand Index
25	m	13-16 years	0.213	751.9867784	88	Grand Index

Age	Gender	Education	Percentile	Raw Score	Norm Score	Assessment
25	m	13-16 years	0.216	752	88	Grand Index
25	m	13-16 years	0.217	752.7519444	88	Grand Index
25	m	13-16 years	0.221	753	88	Grand Index
25	m	13-16 years	0.222	753.137532	89	Grand Index
25	m	13-16 years	0.226	754	89	Grand Index
25	m	13-16 years	0.227	754.7684278	89	Grand Index
25	m	13-16 years	0.229	755	89	Grand Index
25	m	13-16 years	0.23	755.0795135	89	Grand Index
25	m	13-16 years	0.233	756	89	Grand Index
25	m	13-16 years	0.236	757	89	Grand Index
25	m	13-16 years	0.237	757.916686	89	Grand Index
25	m	13-16 years	0.24	758	89	Grand Index
25	m	13-16 years	0.241	758.0345769	89	Grand Index
25	m	13-16 years	0.242	758.5533347	90	Grand Index
25	m	13-16 years	0.246	759	90	Grand Index
25	m	13-16 years	0.25	760	90	Grand Index
25	m	13-16 years	0.251	760.1638983	90	Grand Index
25	m	13-16 years	0.255	761	90	Grand Index
25	m	13-16 years	0.256	761.2702084	90	Grand Index
25	m	13-16 years	0.259	762	90	Grand Index
25	m	13-16 years	0.26	762.700721	90	Grand Index
25	m	13-16 years	0.265	763	91	Grand Index
25	m	13-16 years	0.268	764	91	Grand Index
25	m	13-16 years	0.269	764.2116217	91	Grand Index
25	m	13-16 years	0.271	765	91	Grand Index
25	m	13-16 years	0.272	765.0490854	91	Grand Index
25	m	13-16 years	0.276	766	91	Grand Index
25	m	13-16 years	0.277	766.1735979	91	Grand Index
25	m	13-16 years	0.281	767	91	Grand Index
25	m	13-16 years	0.282	767.2034566	91	Grand Index
25	m	13-16 years	0.286	768	92	Grand Index
25	m	13-16 years	0.291	769	92	Grand Index
25	m	13-16 years	0.292	769.4751931	92	Grand Index
25	m	13-16 years	0.295	770	92	Grand Index
25	m	13-16 years	0.296	770.0937472	92	Grand Index
25	m	13-16 years	0.297	770.9719474	92	Grand Index
25	m	13-16 years	0.3	771	92	Grand Index
25	m	13-16 years	0.301	771.7537431	92	Grand Index
25	m	13-16 years	0.305	772	92	Grand Index
25	m	13-16 years	0.307	773	92	Grand Index
25	m	13-16 years	0.308	773.6348903	92	Grand Index
25	m	13-16 years	0.312	774	93	Grand Index
25	m	13-16 years	0.313	774.1731983	93	Grand Index
25	m	13-16 years	0.316	775	93	Grand Index
25	m	13-16 years	0.317	775.1677954	93	Grand Index
25	m	13-16 years	0.321	776	93	Grand Index
25	m	13-16 years	0.322	776.0079676	93	Grand Index
25	m	13-16 years	0.327	777	93	Grand Index
25	m	13-16 years	0.328	777.017493	93	Grand Index
25	m	13-16 years	0.332	778	93	Grand Index
25	m	13-16 years	0.333	778.9684978	94	Grand Index
25	m	13-16 years	0.336	779	94	Grand Index
25	m	13-16 years	0.337	779.9561854	94	Grand Index
25	m	13-16 years	0.341	780	94	Grand Index
25	m	13-16 years	0.346	781	94	Grand Index
25	m	13-16 years	0.351	782	94	Grand Index

Age	Gender	Education	Percentile	Raw Score	Norm Score	Assessment
25	m	13-16 years	0.352	782.5844212	94	Grand Index
25	m	13-16 years	0.355	783	94	Grand Index
25	m	13-16 years	0.359	784	95	Grand Index
25	m	13-16 years	0.36	784.9805399	95	Grand Index
25	m	13-16 years	0.364	785	95	Grand Index
25	m	13-16 years	0.365	785.4090527	95	Grand Index
25	m	13-16 years	0.371	786	95	Grand Index
25	m	13-16 years	0.372	786.997509	95	Grand Index
25	m	13-16 years	0.375	787	95	Grand Index
25	m	13-16 years	0.379	788	95	Grand Index
25	m	13-16 years	0.383	789	96	Grand Index
25	m	13-16 years	0.384	789.9657965	96	Grand Index
25	m	13-16 years	0.387	790	96	Grand Index
25	m	13-16 years	0.388	790.104	96	Grand Index
25	m	13-16 years	0.391	791	96	Grand Index
25	m	13-16 years	0.392	791.0558595	96	Grand Index
25	m	13-16 years	0.397	792	96	Grand Index
25	m	13-16 years	0.398	792.4260841	96	Grand Index
25	m	13-16 years	0.402	793	96	Grand Index
25	m	13-16 years	0.406	794	96	Grand Index
25	m	13-16 years	0.41	795	97	Grand Index
25	m	13-16 years	0.411	795.5769459	97	Grand Index
25	m	13-16 years	0.416	796	97	Grand Index
25	m	13-16 years	0.417	796.2557999	97	Grand Index
25	m	13-16 years	0.425	797	97	Grand Index
25	m	13-16 years	0.426	797.1084592	97	Grand Index
25	m	13-16 years	0.431	798	97	Grand Index
25	m	13-16 years	0.437	799	98	Grand Index
25	m	13-16 years	0.441	800	98	Grand Index
25	m	13-16 years	0.442	800.0162824	98	Grand Index
25	m	13-16 years	0.445	801	98	Grand Index
25	m	13-16 years	0.449	802	98	Grand Index
25	m	13-16 years	0.45	802.4055248	98	Grand Index
25	m	13-16 years	0.453	803	98	Grand Index
25	m	13-16 years	0.454	803.0324413	98	Grand Index
25	m	13-16 years	0.46	804	98	Grand Index
25	m	13-16 years	0.464	805	99	Grand Index
25	m	13-16 years	0.465	805.9950602	99	Grand Index
25	m	13-16 years	0.469	806	99	Grand Index
25	m	13-16 years	0.47	806.4753958	99	Grand Index
25	m	13-16 years	0.474	807	99	Grand Index
25	m	13-16 years	0.475	807.4255628	99	Grand Index
25	m	13-16 years	0.48	808	99	Grand Index
25	m	13-16 years	0.481	808.0882549	99	Grand Index
25	m	13-16 years	0.485	809	99	Grand Index
25	m	13-16 years	0.486	809.5217227	99	Grand Index
25	m	13-16 years	0.489	810	100	Grand Index
25	m	13-16 years	0.49	810.5283214	100	Grand Index
25	m	13-16 years	0.496	811	100	Grand Index
25	m	13-16 years	0.497	811.0516898	100	Grand Index
25	m	13-16 years	0.501	812	100	Grand Index
25	m	13-16 years	0.502	812.516	100	Grand Index
25	m	13-16 years	0.506	813	100	Grand Index
25	m	13-16 years	0.511	814	100	Grand Index
25	m	13-16 years	0.515	815	101	Grand Index
25	m	13-16 years	0.516	815.128	101	Grand Index

Age	Gender	Education	Percentile	Raw Score	Norm Score	Assessment
25	m	13-16 years	0.519	816	101	Grand Index
25	m	13-16 years	0.52	816.3942885	101	Grand Index
25	m	13-16 years	0.525	817	101	Grand Index
25	m	13-16 years	0.526	817.9765047	101	Grand Index
25	m	13-16 years	0.531	818	101	Grand Index
25	m	13-16 years	0.532	818.3847292	101	Grand Index
25	m	13-16 years	0.538	819	101	Grand Index
25	m	13-16 years	0.539	819.3118941	101	Grand Index
25	m	13-16 years	0.546	820	102	Grand Index
25	m	13-16 years	0.547	820.126	102	Grand Index
25	m	13-16 years	0.551	821	102	Grand Index
25	m	13-16 years	0.556	822	102	Grand Index
25	m	13-16 years	0.557	822.7847331	102	Grand Index
25	m	13-16 years	0.562	823	102	Grand Index
25	m	13-16 years	0.563	823.0177822	102	Grand Index
25	m	13-16 years	0.567	824	103	Grand Index
25	m	13-16 years	0.568	824.5167318	103	Grand Index
25	m	13-16 years	0.573	825	103	Grand Index
25	m	13-16 years	0.578	826	103	Grand Index
25	m	13-16 years	0.579	826.2329382	103	Grand Index
25	m	13-16 years	0.586	827	103	Grand Index
25	m	13-16 years	0.592	828	103	Grand Index
25	m	13-16 years	0.597	829	104	Grand Index
25	m	13-16 years	0.598	829.2578584	104	Grand Index
25	m	13-16 years	0.602	830	104	Grand Index
25	m	13-16 years	0.608	831	104	Grand Index
25	m	13-16 years	0.613	832	104	Grand Index
25	m	13-16 years	0.614	832.9704454	104	Grand Index
25	m	13-16 years	0.616	833	104	Grand Index
25	m	13-16 years	0.617	833.7080537	104	Grand Index
25	m	13-16 years	0.621	834	105	Grand Index
25	m	13-16 years	0.622	834.9579398	105	Grand Index
25	m	13-16 years	0.627	835	105	Grand Index
25	m	13-16 years	0.628	835.9974535	105	Grand Index
25	m	13-16 years	0.632	836	105	Grand Index
25	m	13-16 years	0.633	836.107978	105	Grand Index
25	m	13-16 years	0.637	837	105	Grand Index
25	m	13-16 years	0.638	837.9735481	105	Grand Index
25	m	13-16 years	0.642	838	105	Grand Index
25	m	13-16 years	0.643	838.0906524	105	Grand Index
25	m	13-16 years	0.647	839	106	Grand Index
25	m	13-16 years	0.648	839.2936961	106	Grand Index
25	m	13-16 years	0.653	840	106	Grand Index
25	m	13-16 years	0.657	841	106	Grand Index
25	m	13-16 years	0.658	841.9722218	106	Grand Index
25	m	13-16 years	0.661	842	106	Grand Index
25	m	13-16 years	0.665	843	106	Grand Index
25	m	13-16 years	0.668	844	107	Grand Index
25	m	13-16 years	0.669	844.9807022	107	Grand Index
25	m	13-16 years	0.672	845	107	Grand Index
25	m	13-16 years	0.673	845.8809745	107	Grand Index
25	m	13-16 years	0.676	846	107	Grand Index
25	m	13-16 years	0.677	846.8476285	107	Grand Index
25	m	13-16 years	0.68	847	107	Grand Index
25	m	13-16 years	0.681	847.4122744	107	Grand Index
25	m	13-16 years	0.682	847.8978688	107	Grand Index

Age	Gender	Education	Percentile	Raw Score	Norm Score	Assessment
25	m	13-16 years	0.686	848	107	Grand Index
25	m	13-16 years	0.687	848.246	107	Grand Index
25	m	13-16 years	0.69	849	107	Grand Index
25	m	13-16 years	0.691	849.0173551	107	Grand Index
25	m	13-16 years	0.695	850	108	Grand Index
25	m	13-16 years	0.696	850.1484579	108	Grand Index
25	m	13-16 years	0.701	851	108	Grand Index
25	m	13-16 years	0.704	852	108	Grand Index
25	m	13-16 years	0.705	852.89	108	Grand Index
25	m	13-16 years	0.708	853	108	Grand Index
25	m	13-16 years	0.711	854	108	Grand Index
25	m	13-16 years	0.715	855	109	Grand Index
25	m	13-16 years	0.716	855.209484	109	Grand Index
25	m	13-16 years	0.718	856	109	Grand Index
25	m	13-16 years	0.719	856.3648957	109	Grand Index
25	m	13-16 years	0.72	856.9900319	109	Grand Index
25	m	13-16 years	0.723	857	109	Grand Index
25	m	13-16 years	0.724	857.7730464	109	Grand Index
25	m	13-16 years	0.727	858	109	Grand Index
25	m	13-16 years	0.728	858.7739598	109	Grand Index
25	m	13-16 years	0.732	859	109	Grand Index
25	m	13-16 years	0.733	859.3444355	109	Grand Index
25	m	13-16 years	0.737	860	110	Grand Index
25	m	13-16 years	0.738	860.480472	110	Grand Index
25	m	13-16 years	0.74	861	110	Grand Index
25	m	13-16 years	0.741	861.0426079	110	Grand Index
25	m	13-16 years	0.744	862	110	Grand Index
25	m	13-16 years	0.745	862.0063849	110	Grand Index
25	m	13-16 years	0.746	862.7342955	110	Grand Index
25	m	13-16 years	0.749	863	110	Grand Index
25	m	13-16 years	0.75	863.8328644	110	Grand Index
25	m	13-16 years	0.755	864	110	Grand Index
25	m	13-16 years	0.759	865	111	Grand Index
25	m	13-16 years	0.761	866	111	Grand Index
25	m	13-16 years	0.762	866.6359449	111	Grand Index
25	m	13-16 years	0.765	867	111	Grand Index
25	m	13-16 years	0.766	867.8858854	111	Grand Index
25	m	13-16 years	0.768	868	111	Grand Index
25	m	13-16 years	0.769	868.0770607	111	Grand Index
25	m	13-16 years	0.772	869	111	Grand Index
25	m	13-16 years	0.773	869.1671806	111	Grand Index
25	m	13-16 years	0.776	870	111	Grand Index
25	m	13-16 years	0.779	871	112	Grand Index
25	m	13-16 years	0.78	871.6654571	112	Grand Index
25	m	13-16 years	0.782	872	112	Grand Index
25	m	13-16 years	0.783	872.2848743	112	Grand Index
25	m	13-16 years	0.786	873	112	Grand Index
25	m	13-16 years	0.787	873.4247826	112	Grand Index
25	m	13-16 years	0.789	874	112	Grand Index
25	m	13-16 years	0.79	874.0737466	112	Grand Index
25	m	13-16 years	0.794	875	112	Grand Index
25	m	13-16 years	0.795	875.6531723	112	Grand Index
25	m	13-16 years	0.798	876	113	Grand Index
25	m	13-16 years	0.799	876.9589885	113	Grand Index
25	m	13-16 years	0.801	877	113	Grand Index
25	m	13-16 years	0.804	878	113	Grand Index

Age	Gender	Education	Percentile	Raw Score	Norm Score	Assessment
25	m	13-16 years	0.809	879	113	Grand Index
25	m	13-16 years	0.81	879.508254	113	Grand Index
25	m	13-16 years	0.812	880	113	Grand Index
25	m	13-16 years	0.813	880.0797165	113	Grand Index
25	m	13-16 years	0.816	881	114	Grand Index
25	m	13-16 years	0.817	881.069038	114	Grand Index
25	m	13-16 years	0.821	882	114	Grand Index
25	m	13-16 years	0.824	883	114	Grand Index
25	m	13-16 years	0.825	883.3929435	114	Grand Index
25	m	13-16 years	0.829	884	114	Grand Index
25	m	13-16 years	0.834	885	115	Grand Index
25	m	13-16 years	0.836	886	115	Grand Index
25	m	13-16 years	0.837	886.0127697	115	Grand Index
25	m	13-16 years	0.839	887	115	Grand Index
25	m	13-16 years	0.842	888	115	Grand Index
25	m	13-16 years	0.843	888.8671649	115	Grand Index
25	m	13-16 years	0.845	889	115	Grand Index
25	m	13-16 years	0.847	890	115	Grand Index
25	m	13-16 years	0.848	890.8237264	115	Grand Index
25	m	13-16 years	0.849	891	115	Grand Index
25	m	13-16 years	0.85	891.1960272	116	Grand Index
25	m	13-16 years	0.852	892	116	Grand Index
25	m	13-16 years	0.853	892.7792238	116	Grand Index
25	m	13-16 years	0.855	893	116	Grand Index
25	m	13-16 years	0.856	893.9982797	116	Grand Index
25	m	13-16 years	0.857	894	116	Grand Index
25	m	13-16 years	0.858	894.7291416	116	Grand Index
25	m	13-16 years	0.86	895	116	Grand Index
25	m	13-16 years	0.862	896	116	Grand Index
25	m	13-16 years	0.863	896.8993015	116	Grand Index
25	m	13-16 years	0.866	897	117	Grand Index
25	m	13-16 years	0.868	898	117	Grand Index
25	m	13-16 years	0.869	898.0065044	117	Grand Index
25	m	13-16 years	0.871	899	117	Grand Index
25	m	13-16 years	0.872	899.1261959	117	Grand Index
25	m	13-16 years	0.875	900	117	Grand Index
25	m	13-16 years	0.876	900.3010812	117	Grand Index
25	m	13-16 years	0.879	901	118	Grand Index
25	m	13-16 years	0.88	901.9439606	118	Grand Index
25	m	13-16 years	0.882	902	118	Grand Index
25	m	13-16 years	0.883	902.9054845	118	Grand Index
25	m	13-16 years	0.885	904	118	Grand Index
25	m	13-16 years	0.886	904.9965272	118	Grand Index
25	m	13-16 years	0.888	905	118	Grand Index
25	m	13-16 years	0.89	906	118	Grand Index
25	m	13-16 years	0.892	907	119	Grand Index
25	m	13-16 years	0.895	908	119	Grand Index
25	m	13-16 years	0.896	909	119	Grand Index
25	m	13-16 years	0.897	909.0140253	119	Grand Index
25	m	13-16 years	0.898	910.0073366	119	Grand Index
25	m	13-16 years	0.899	911	119	Grand Index
25	m	13-16 years	0.9	911.2821823	119	Grand Index
25	m	13-16 years	0.901	912	119	Grand Index
25	m	13-16 years	0.902	913	119	Grand Index
25	m	13-16 years	0.903	913.9524562	119	Grand Index
25	m	13-16 years	0.905	914	120	Grand Index

Age	Gender	Education	Percentile	Raw Score	Norm Score	Assessment
25	m	13-16 years	0.906	914.9950838	120	Grand Index
25	m	13-16 years	0.907	915.0016257	120	Grand Index
25	m	13-16 years	0.908	916	120	Grand Index
25	m	13-16 years	0.909	916.0026358	120	Grand Index
25	m	13-16 years	0.91	917	120	Grand Index
25	m	13-16 years	0.911	917.480026	120	Grand Index
25	m	13-16 years	0.912	918	120	Grand Index
25	m	13-16 years	0.913	918.163916	120	Grand Index
25	m	13-16 years	0.915	919	121	Grand Index
25	m	13-16 years	0.916	919.7396719	121	Grand Index
25	m	13-16 years	0.917	920	121	Grand Index
25	m	13-16 years	0.918	920.8115426	121	Grand Index
25	m	13-16 years	0.919	921	121	Grand Index
25	m	13-16 years	0.92	921.8156945	121	Grand Index
25	m	13-16 years	0.921	922	121	Grand Index
25	m	13-16 years	0.922	922.8821012	121	Grand Index
25	m	13-16 years	0.923	923.7126835	121	Grand Index
25	m	13-16 years	0.924	924	121	Grand Index
25	m	13-16 years	0.925	924.4588927	122	Grand Index
25	m	13-16 years	0.927	925	122	Grand Index
25	m	13-16 years	0.929	926	122	Grand Index
25	m	13-16 years	0.93	926.496133	122	Grand Index
25	m	13-16 years	0.931	927	122	Grand Index
25	m	13-16 years	0.932	927.5936765	122	Grand Index
25	m	13-16 years	0.934	928	123	Grand Index
25	m	13-16 years	0.935	929	123	Grand Index
25	m	13-16 years	0.936	930.1960795	123	Grand Index
25	m	13-16 years	0.937	931	123	Grand Index
25	m	13-16 years	0.939	932	123	Grand Index
25	m	13-16 years	0.94	933	123	Grand Index
25	m	13-16 years	0.941	933.7637847	123	Grand Index
25	m	13-16 years	0.942	934	124	Grand Index
25	m	13-16 years	0.943	934.1197997	124	Grand Index
25	m	13-16 years	0.944	935	124	Grand Index
25	m	13-16 years	0.945	936	124	Grand Index
25	m	13-16 years	0.946	937.0520822	124	Grand Index
25	m	13-16 years	0.947	938	124	Grand Index
25	m	13-16 years	0.948	938.100628	124	Grand Index
25	m	13-16 years	0.949	939	125	Grand Index
25	m	13-16 years	0.95	940	125	Grand Index
25	m	13-16 years	0.951	941	125	Grand Index
25	m	13-16 years	0.952	942	125	Grand Index
25	m	13-16 years	0.953	943	125	Grand Index
25	m	13-16 years	0.954	944	125	Grand Index
25	m	13-16 years	0.955	944.073859	125	Grand Index
25	m	13-16 years	0.956	945	126	Grand Index
25	m	13-16 years	0.957	945.906	126	Grand Index
25	m	13-16 years	0.958	946	126	Grand Index
25	m	13-16 years	0.959	946.535162	126	Grand Index
25	m	13-16 years	0.96	947	126	Grand Index
25	m	13-16 years	0.961	948	126	Grand Index
25	m	13-16 years	0.962	949.0053557	127	Grand Index
25	m	13-16 years	0.963	950.1856048	127	Grand Index
25	m	13-16 years	0.964	951.0361862	127	Grand Index
25	m	13-16 years	0.965	952.9907635	127	Grand Index
25	m	13-16 years	0.966	955	127	Grand Index

Age	Gender	Education	Percentile	Raw Score	Norm Score	Assessment
25	m	13-16 years	0.967	956	128	Grand Index
25	m	13-16 years	0.968	956.0735619	128	Grand Index
25	m	13-16 years	0.969	957.0019731	128	Grand Index
25	m	13-16 years	0.97	958	128	Grand Index
25	m	13-16 years	0.971	958.8812718	128	Grand Index
25	m	13-16 years	0.972	961	129	Grand Index
25	m	13-16 years	0.973	963	129	Grand Index
25	m	13-16 years	0.974	964	129	Grand Index
25	m	13-16 years	0.975	964.4332398	129	Grand Index
25	m	13-16 years	0.976	965.2065176	130	Grand Index
25	m	13-16 years	0.977	968.8929101	130	Grand Index
25	m	13-16 years	0.978	969.6732174	130	Grand Index
25	m	13-16 years	0.979	970.5352537	131	Grand Index
25	m	13-16 years	0.98	973.2818688	131	Grand Index
25	m	13-16 years	0.981	975.0372319	131	Grand Index
25	m	13-16 years	0.982	976.4382246	131	Grand Index
25	m	13-16 years	0.983	980.0779115	132	Grand Index
25	m	13-16 years	0.984	981	132	Grand Index
25	m	13-16 years	0.985	982.9737665	133	Grand Index
25	m	13-16 years	0.986	984.5133613	133	Grand Index
25	m	13-16 years	0.987	985.9496956	133	Grand Index
25	m	13-16 years	0.988	988.8156952	134	Grand Index
25	m	13-16 years	0.989	989.5225313	134	Grand Index
25	m	13-16 years	0.99	992	135	Grand Index
25	m	13-16 years	0.991	996.2149106	135	Grand Index
25	m	13-16 years	0.992	1000.936	136	Grand Index
25	m	13-16 years	0.993	1003.991919	137	Grand Index
25	m	13-16 years	0.994	1006.920371	138	Grand Index
25	m	13-16 years	0.995	1013.329277	139	Grand Index
25	m	13-16 years	0.996	1022.778456	140	Grand Index
25	m	13-16 years	0.997	1030.946393	141	Grand Index
25	m	13-16 years	0.998	1042.820945	143	Grand Index
25	m	13-16 years	0.999	1048.533255	146	Grand Index
25	f	17+ years	0.001	586.2638811	54	Grand Index
25	f	17+ years	0.002	595.7157365	57	Grand Index
25	f	17+ years	0.003	612.9540846	59	Grand Index
25	f	17+ years	0.004	616.8576372	60	Grand Index
25	f	17+ years	0.005	629	61	Grand Index
25	f	17+ years	0.006	631.0957225	62	Grand Index
25	f	17+ years	0.007	633.8747575	63	Grand Index
25	f	17+ years	0.008	634	64	Grand Index
25	f	17+ years	0.009	637.5871597	65	Grand Index
25	f	17+ years	0.01	638.0891337	65	Grand Index
25	f	17+ years	0.011	638.9704565	66	Grand Index
25	f	17+ years	0.012	640.4678044	66	Grand Index
25	f	17+ years	0.013	644.9663033	67	Grand Index
25	f	17+ years	0.014	645.872	67	Grand Index
25	f	17+ years	0.015	649.0892636	67	Grand Index
25	f	17+ years	0.016	651.4521358	68	Grand Index
25	f	17+ years	0.017	653.1228601	68	Grand Index
25	f	17+ years	0.018	654.528	69	Grand Index
25	f	17+ years	0.019	656.2189927	69	Grand Index
25	f	17+ years	0.02	659.9182117	69	Grand Index
25	f	17+ years	0.021	661	69	Grand Index
25	f	17+ years	0.022	662.1740014	70	Grand Index
25	f	17+ years	0.023	663.504	70	Grand Index

Age	Gender	Education	Percentile	Raw Score	Norm Score	Assessment
25	f	17+ years	0.024	664.352	70	Grand Index
25	f	17+ years	0.025	667.2	71	Grand Index
25	f	17+ years	0.026	668.2131213	71	Grand Index
25	f	17+ years	0.027	670.896	71	Grand Index
25	f	17+ years	0.028	671	71	Grand Index
25	f	17+ years	0.029	672	72	Grand Index
25	f	17+ years	0.03	673.5586617	72	Grand Index
25	f	17+ years	0.031	674	72	Grand Index
25	f	17+ years	0.032	675.136	72	Grand Index
25	f	17+ years	0.033	676	72	Grand Index
25	f	17+ years	0.034	677.2186252	73	Grand Index
25	f	17+ years	0.035	678	73	Grand Index
25	f	17+ years	0.036	679	73	Grand Index
25	f	17+ years	0.037	679.3510076	73	Grand Index
25	f	17+ years	0.038	680	73	Grand Index
25	f	17+ years	0.039	680.072	74	Grand Index
25	f	17+ years	0.04	681	74	Grand Index
25	f	17+ years	0.041	681.7235262	74	Grand Index
25	f	17+ years	0.042	682	74	Grand Index
25	f	17+ years	0.043	683	74	Grand Index
25	f	17+ years	0.044	683.1940943	74	Grand Index
25	f	17+ years	0.045	684	75	Grand Index
25	f	17+ years	0.046	684.1368051	75	Grand Index
25	f	17+ years	0.047	686.856	75	Grand Index
25	f	17+ years	0.048	687.704	75	Grand Index
25	f	17+ years	0.049	688.2705464	75	Grand Index
25	f	17+ years	0.05	689.1972711	75	Grand Index
25	f	17+ years	0.051	689.8207764	75	Grand Index
25	f	17+ years	0.052	691	76	Grand Index
25	f	17+ years	0.053	691.944	76	Grand Index
25	f	17+ years	0.054	692	76	Grand Index
25	f	17+ years	0.055	692.6429441	76	Grand Index
25	f	17+ years	0.056	693.9574426	76	Grand Index
25	f	17+ years	0.058	695	76	Grand Index
25	f	17+ years	0.059	696.0271227	77	Grand Index
25	f	17+ years	0.06	696.98171	77	Grand Index
25	f	17+ years	0.061	697	77	Grand Index
25	f	17+ years	0.062	697.576	77	Grand Index
25	f	17+ years	0.063	698	77	Grand Index
25	f	17+ years	0.064	698.0017422	77	Grand Index
25	f	17+ years	0.065	699.12	77	Grand Index
25	f	17+ years	0.067	700	78	Grand Index
25	f	17+ years	0.069	701	78	Grand Index
25	f	17+ years	0.07	701.0574802	78	Grand Index
25	f	17+ years	0.071	702.8253278	78	Grand Index
25	f	17+ years	0.072	703.0139866	78	Grand Index
25	f	17+ years	0.073	703.2499778	78	Grand Index
25	f	17+ years	0.074	704	78	Grand Index
25	f	17+ years	0.075	704.6	78	Grand Index
25	f	17+ years	0.076	705	79	Grand Index
25	f	17+ years	0.077	705.0184511	79	Grand Index
25	f	17+ years	0.08	706	79	Grand Index
25	f	17+ years	0.081	706.6683895	79	Grand Index
25	f	17+ years	0.082	707.4908103	79	Grand Index
25	f	17+ years	0.083	708.0040954	79	Grand Index
25	f	17+ years	0.085	709	79	Grand Index

Age	Gender	Education	Percentile	Raw Score	Norm Score	Assessment
25	f	17+ years	0.086	709.928	80	Grand Index
25	f	17+ years	0.087	710.9535641	80	Grand Index
25	f	17+ years	0.088	711.2134833	80	Grand Index
25	f	17+ years	0.089	712	80	Grand Index
25	f	17+ years	0.093	713	80	Grand Index
25	f	17+ years	0.094	714	80	Grand Index
25	f	17+ years	0.095	714.907119	80	Grand Index
25	f	17+ years	0.096	715	80	Grand Index
25	f	17+ years	0.097	715.2473971	81	Grand Index
25	f	17+ years	0.099	716	81	Grand Index
25	f	17+ years	0.101	717	81	Grand Index
25	f	17+ years	0.102	717.6296592	81	Grand Index
25	f	17+ years	0.103	718	81	Grand Index
25	f	17+ years	0.104	718.9885072	81	Grand Index
25	f	17+ years	0.106	719	81	Grand Index
25	f	17+ years	0.107	719.736	81	Grand Index
25	f	17+ years	0.108	720	81	Grand Index
25	f	17+ years	0.11	721	82	Grand Index
25	f	17+ years	0.112	722	82	Grand Index
25	f	17+ years	0.113	722.2104153	82	Grand Index
25	f	17+ years	0.114	722.7842156	82	Grand Index
25	f	17+ years	0.115	723	82	Grand Index
25	f	17+ years	0.116	723.368	82	Grand Index
25	f	17+ years	0.119	724	82	Grand Index
25	f	17+ years	0.12	724.9042408	82	Grand Index
25	f	17+ years	0.122	725	83	Grand Index
25	f	17+ years	0.123	725.8290716	83	Grand Index
25	f	17+ years	0.126	726	83	Grand Index
25	f	17+ years	0.127	727	83	Grand Index
25	f	17+ years	0.128	727.544	83	Grand Index
25	f	17+ years	0.132	728	83	Grand Index
25	f	17+ years	0.133	728.302853	83	Grand Index
25	f	17+ years	0.134	729	83	Grand Index
25	f	17+ years	0.135	729.0763839	83	Grand Index
25	f	17+ years	0.137	730	84	Grand Index
25	f	17+ years	0.138	731.024	84	Grand Index
25	f	17+ years	0.14	732	84	Grand Index
25	f	17+ years	0.141	732.0126289	84	Grand Index
25	f	17+ years	0.143	733	84	Grand Index
25	f	17+ years	0.144	733.0010579	84	Grand Index
25	f	17+ years	0.145	733.9511273	84	Grand Index
25	f	17+ years	0.148	734	84	Grand Index
25	f	17+ years	0.149	734.352	84	Grand Index
25	f	17+ years	0.15	735	84	Grand Index
25	f	17+ years	0.151	735.0011073	85	Grand Index
25	f	17+ years	0.152	735.0996069	85	Grand Index
25	f	17+ years	0.154	736	85	Grand Index
25	f	17+ years	0.155	736.5416584	85	Grand Index
25	f	17+ years	0.156	737	85	Grand Index
25	f	17+ years	0.157	737.0279445	85	Grand Index
25	f	17+ years	0.158	737.5411074	85	Grand Index
25	f	17+ years	0.16	738	85	Grand Index
25	f	17+ years	0.162	739	85	Grand Index
25	f	17+ years	0.163	739.224	85	Grand Index
25	f	17+ years	0.164	740.072	85	Grand Index
25	f	17+ years	0.166	741	85	Grand Index

Age	Gender	Education	Percentile	Raw Score	Norm Score	Assessment
25	f	17+ years	0.167	741.616	86	Grand Index
25	f	17+ years	0.171	742	86	Grand Index
25	f	17+ years	0.172	742.856	86	Grand Index
25	f	17+ years	0.173	743	86	Grand Index
25	f	17+ years	0.175	744	86	Grand Index
25	f	17+ years	0.176	744.248	86	Grand Index
25	f	17+ years	0.178	745	86	Grand Index
25	f	17+ years	0.181	746	86	Grand Index
25	f	17+ years	0.182	746.3192101	86	Grand Index
25	f	17+ years	0.184	747	86	Grand Index
25	f	17+ years	0.185	747.88	87	Grand Index
25	f	17+ years	0.186	748	87	Grand Index
25	f	17+ years	0.187	748.576	87	Grand Index
25	f	17+ years	0.188	749	87	Grand Index
25	f	17+ years	0.191	750	87	Grand Index
25	f	17+ years	0.192	750.8272353	87	Grand Index
25	f	17+ years	0.194	751	87	Grand Index
25	f	17+ years	0.195	751.4854902	87	Grand Index
25	f	17+ years	0.198	752	87	Grand Index
25	f	17+ years	0.199	752.1167269	87	Grand Index
25	f	17+ years	0.201	753	87	Grand Index
25	f	17+ years	0.202	753.6937993	87	Grand Index
25	f	17+ years	0.205	754	88	Grand Index
25	f	17+ years	0.206	754.2822173	88	Grand Index
25	f	17+ years	0.208	755	88	Grand Index
25	f	17+ years	0.209	755.0013878	88	Grand Index
25	f	17+ years	0.212	756	88	Grand Index
25	f	17+ years	0.213	756.1915666	88	Grand Index
25	f	17+ years	0.214	756.6471484	88	Grand Index
25	f	17+ years	0.22	757	88	Grand Index
25	f	17+ years	0.221	757.0738765	88	Grand Index
25	f	17+ years	0.222	757.6748079	89	Grand Index
25	f	17+ years	0.224	758	89	Grand Index
25	f	17+ years	0.225	758.8	89	Grand Index
25	f	17+ years	0.227	759	89	Grand Index
25	f	17+ years	0.228	759.4443231	89	Grand Index
25	f	17+ years	0.231	760	89	Grand Index
25	f	17+ years	0.232	760.736	89	Grand Index
25	f	17+ years	0.234	761	89	Grand Index
25	f	17+ years	0.235	761.6562088	89	Grand Index
25	f	17+ years	0.24	762	89	Grand Index
25	f	17+ years	0.241	762.0220872	89	Grand Index
25	f	17+ years	0.242	762.9930889	90	Grand Index
25	f	17+ years	0.246	763	90	Grand Index
25	f	17+ years	0.247	763.911311	90	Grand Index
25	f	17+ years	0.251	764	90	Grand Index
25	f	17+ years	0.252	764.6565027	90	Grand Index
25	f	17+ years	0.256	765	90	Grand Index
25	f	17+ years	0.257	765.3109381	90	Grand Index
25	f	17+ years	0.259	766	90	Grand Index
25	f	17+ years	0.26	766.9692191	90	Grand Index
25	f	17+ years	0.264	767	91	Grand Index
25	f	17+ years	0.265	767.0461923	91	Grand Index
25	f	17+ years	0.268	768	91	Grand Index
25	f	17+ years	0.269	768.6247432	91	Grand Index
25	f	17+ years	0.272	769	91	Grand Index

Age	Gender	Education	Percentile	Raw Score	Norm Score	Assessment
25	f	17+ years	0.273	769.504	91	Grand Index
25	f	17+ years	0.278	770	91	Grand Index
25	f	17+ years	0.279	770.6579615	91	Grand Index
25	f	17+ years	0.284	771	91	Grand Index
25	f	17+ years	0.286	772	92	Grand Index
25	f	17+ years	0.292	773	92	Grand Index
25	f	17+ years	0.293	773.464	92	Grand Index
25	f	17+ years	0.297	774	92	Grand Index
25	f	17+ years	0.298	774.7708212	92	Grand Index
25	f	17+ years	0.3	775	92	Grand Index
25	f	17+ years	0.301	775.9753573	92	Grand Index
25	f	17+ years	0.306	776	92	Grand Index
25	f	17+ years	0.307	776.336	92	Grand Index
25	f	17+ years	0.308	777	92	Grand Index
25	f	17+ years	0.309	777.0121617	93	Grand Index
25	f	17+ years	0.31	777.9256064	93	Grand Index
25	f	17+ years	0.312	778	93	Grand Index
25	f	17+ years	0.313	778.0159702	93	Grand Index
25	f	17+ years	0.317	779	93	Grand Index
25	f	17+ years	0.318	779.5138852	93	Grand Index
25	f	17+ years	0.321	780	93	Grand Index
25	f	17+ years	0.322	780.7792441	93	Grand Index
25	f	17+ years	0.323	780.9963305	93	Grand Index
25	f	17+ years	0.326	781	93	Grand Index
25	f	17+ years	0.327	781.3202086	93	Grand Index
25	f	17+ years	0.332	782	93	Grand Index
25	f	17+ years	0.333	782.2205762	94	Grand Index
25	f	17+ years	0.339	783	94	Grand Index
25	f	17+ years	0.344	784	94	Grand Index
25	f	17+ years	0.345	784.1224373	94	Grand Index
25	f	17+ years	0.35	785	94	Grand Index
25	f	17+ years	0.351	785.0742443	94	Grand Index
25	f	17+ years	0.352	785.9133248	94	Grand Index
25	f	17+ years	0.355	786	94	Grand Index
25	f	17+ years	0.356	786.2474869	94	Grand Index
25	f	17+ years	0.357	786.8861437	95	Grand Index
25	f	17+ years	0.359	787	95	Grand Index
25	f	17+ years	0.365	788	95	Grand Index
25	f	17+ years	0.366	788.2736822	95	Grand Index
25	f	17+ years	0.369	789	95	Grand Index
25	f	17+ years	0.37	789.867644	95	Grand Index
25	f	17+ years	0.372	790	95	Grand Index
25	f	17+ years	0.377	791	95	Grand Index
25	f	17+ years	0.378	791.0476407	95	Grand Index
25	f	17+ years	0.379	791.4623075	95	Grand Index
25	f	17+ years	0.387	792	96	Grand Index
25	f	17+ years	0.388	792.81483	96	Grand Index
25	f	17+ years	0.39	793	96	Grand Index
25	f	17+ years	0.391	793.9092754	96	Grand Index
25	f	17+ years	0.392	794	96	Grand Index
25	f	17+ years	0.393	794.264	96	Grand Index
25	f	17+ years	0.398	795	96	Grand Index
25	f	17+ years	0.399	795.4985506	96	Grand Index
25	f	17+ years	0.403	796	96	Grand Index
25	f	17+ years	0.404	796.0205022	96	Grand Index
25	f	17+ years	0.408	797	97	Grand Index

Age	Gender	Education	Percentile	Raw Score	Norm Score	Assessment
25	f	17+ years	0.409	797.720369	97	Grand Index
25	f	17+ years	0.414	798	97	Grand Index
25	f	17+ years	0.415	798.0075513	97	Grand Index
25	f	17+ years	0.416	798.912676	97	Grand Index
25	f	17+ years	0.42	799	97	Grand Index
25	f	17+ years	0.421	799.0055854	97	Grand Index
25	f	17+ years	0.422	799.9565377	97	Grand Index
25	f	17+ years	0.429	800	97	Grand Index
25	f	17+ years	0.43	800.9319079	97	Grand Index
25	f	17+ years	0.434	801	98	Grand Index
25	f	17+ years	0.435	801.1381818	98	Grand Index
25	f	17+ years	0.439	802	98	Grand Index
25	f	17+ years	0.44	802.7379653	98	Grand Index
25	f	17+ years	0.445	803	98	Grand Index
25	f	17+ years	0.446	803.0602727	98	Grand Index
25	f	17+ years	0.447	803.887638	98	Grand Index
25	f	17+ years	0.448	803.9956837	98	Grand Index
25	f	17+ years	0.454	804	98	Grand Index
25	f	17+ years	0.455	804.1925854	98	Grand Index
25	f	17+ years	0.456	804.9982762	98	Grand Index
25	f	17+ years	0.458	805	98	Grand Index
25	f	17+ years	0.459	805.2233789	98	Grand Index
25	f	17+ years	0.465	806	99	Grand Index
25	f	17+ years	0.468	807	99	Grand Index
25	f	17+ years	0.469	807.7415024	99	Grand Index
25	f	17+ years	0.472	808	99	Grand Index
25	f	17+ years	0.473	808.104	99	Grand Index
25	f	17+ years	0.475	809	99	Grand Index
25	f	17+ years	0.476	809.3958608	99	Grand Index
25	f	17+ years	0.482	810	99	Grand Index
25	f	17+ years	0.483	810.3280899	99	Grand Index
25	f	17+ years	0.484	810.6248584	99	Grand Index
25	f	17+ years	0.488	811	100	Grand Index
25	f	17+ years	0.489	811.2860462	100	Grand Index
25	f	17+ years	0.49	811.983386	100	Grand Index
25	f	17+ years	0.493	812	100	Grand Index
25	f	17+ years	0.494	812.912	100	Grand Index
25	f	17+ years	0.497	813	100	Grand Index
25	f	17+ years	0.498	813.304	100	Grand Index
25	f	17+ years	0.504	814	100	Grand Index
25	f	17+ years	0.505	814.0568016	100	Grand Index
25	f	17+ years	0.506	814.8401169	100	Grand Index
25	f	17+ years	0.511	815	100	Grand Index
25	f	17+ years	0.512	815.1913484	100	Grand Index
25	f	17+ years	0.52	816	101	Grand Index
25	f	17+ years	0.521	816.1619599	101	Grand Index
25	f	17+ years	0.524	817	101	Grand Index
25	f	17+ years	0.525	817.2	101	Grand Index
25	f	17+ years	0.53	818	101	Grand Index
25	f	17+ years	0.531	818.2137194	101	Grand Index
25	f	17+ years	0.537	819	101	Grand Index
25	f	17+ years	0.538	819.0356516	101	Grand Index
25	f	17+ years	0.539	819.941607	101	Grand Index
25	f	17+ years	0.544	820	102	Grand Index
25	f	17+ years	0.545	820.2287841	102	Grand Index
25	f	17+ years	0.55	821	102	Grand Index

Age	Gender	Education	Percentile	Raw Score	Norm Score	Assessment
25	f	17+ years	0.551	821.4140053	102	Grand Index
25	f	17+ years	0.556	822	102	Grand Index
25	f	17+ years	0.559	823	102	Grand Index
25	f	17+ years	0.56	823.0174719	102	Grand Index
25	f	17+ years	0.561	823.7477127	102	Grand Index
25	f	17+ years	0.566	824	102	Grand Index
25	f	17+ years	0.567	824.816	103	Grand Index
25	f	17+ years	0.57	825	103	Grand Index
25	f	17+ years	0.571	825.5435868	103	Grand Index
25	f	17+ years	0.579	826	103	Grand Index
25	f	17+ years	0.58	826.8630871	103	Grand Index
25	f	17+ years	0.584	827	103	Grand Index
25	f	17+ years	0.585	827.0440614	103	Grand Index
25	f	17+ years	0.586	827.9603584	103	Grand Index
25	f	17+ years	0.589	828	103	Grand Index
25	f	17+ years	0.59	828.1343232	103	Grand Index
25	f	17+ years	0.591	828.9260912	103	Grand Index
25	f	17+ years	0.597	829	104	Grand Index
25	f	17+ years	0.602	830	104	Grand Index
25	f	17+ years	0.603	830.3499993	104	Grand Index
25	f	17+ years	0.608	831	104	Grand Index
25	f	17+ years	0.609	831.5129151	104	Grand Index
25	f	17+ years	0.615	832	104	Grand Index
25	f	17+ years	0.616	832.2936749	104	Grand Index
25	f	17+ years	0.619	833	105	Grand Index
25	f	17+ years	0.62	833.76	105	Grand Index
25	f	17+ years	0.623	834	105	Grand Index
25	f	17+ years	0.624	834.9246687	105	Grand Index
25	f	17+ years	0.629	835	105	Grand Index
25	f	17+ years	0.634	836	105	Grand Index
25	f	17+ years	0.635	836.48	105	Grand Index
25	f	17+ years	0.637	837	105	Grand Index
25	f	17+ years	0.638	837.2712557	105	Grand Index
25	f	17+ years	0.639	837.9306081	105	Grand Index
25	f	17+ years	0.642	838	105	Grand Index
25	f	17+ years	0.643	838.1202277	105	Grand Index
25	f	17+ years	0.645	839	106	Grand Index
25	f	17+ years	0.646	839.0707737	106	Grand Index
25	f	17+ years	0.65	840	106	Grand Index
25	f	17+ years	0.654	841	106	Grand Index
25	f	17+ years	0.655	841.9793072	106	Grand Index
25	f	17+ years	0.659	842	106	Grand Index
25	f	17+ years	0.66	842.9548317	106	Grand Index
25	f	17+ years	0.664	843	106	Grand Index
25	f	17+ years	0.665	843.3370567	106	Grand Index
25	f	17+ years	0.669	844	107	Grand Index
25	f	17+ years	0.67	844.9910432	107	Grand Index
25	f	17+ years	0.674	845	107	Grand Index
25	f	17+ years	0.675	846	107	Grand Index
25	f	17+ years	0.676	846.248	107	Grand Index
25	f	17+ years	0.678	847	107	Grand Index
25	f	17+ years	0.679	847.7753318	107	Grand Index
25	f	17+ years	0.683	848	107	Grand Index
25	f	17+ years	0.688	849	107	Grand Index
25	f	17+ years	0.689	849.7508188	107	Grand Index
25	f	17+ years	0.694	850	108	Grand Index

Age	Gender	Education	Percentile	Raw Score	Norm Score	Assessment
25	f	17+ years	0.695	850.3920339	108	Grand Index
25	f	17+ years	0.699	851	108	Grand Index
25	f	17+ years	0.7	851.515515	108	Grand Index
25	f	17+ years	0.706	852	108	Grand Index
25	f	17+ years	0.707	852.237459	108	Grand Index
25	f	17+ years	0.708	852.9993872	108	Grand Index
25	f	17+ years	0.71	853	108	Grand Index
25	f	17+ years	0.711	853.928	108	Grand Index
25	f	17+ years	0.712	854	108	Grand Index
25	f	17+ years	0.713	854.5597217	108	Grand Index
25	f	17+ years	0.717	855	109	Grand Index
25	f	17+ years	0.718	855.864	109	Grand Index
25	f	17+ years	0.721	856	109	Grand Index
25	f	17+ years	0.727	857	109	Grand Index
25	f	17+ years	0.733	858	109	Grand Index
25	f	17+ years	0.734	858.0044966	109	Grand Index
25	f	17+ years	0.738	859	110	Grand Index
25	f	17+ years	0.739	859.094814	110	Grand Index
25	f	17+ years	0.74	859.3369947	110	Grand Index
25	f	17+ years	0.741	859.9050617	110	Grand Index
25	f	17+ years	0.746	860	110	Grand Index
25	f	17+ years	0.747	860.9689143	110	Grand Index
25	f	17+ years	0.75	861	110	Grand Index
25	f	17+ years	0.751	861.919582	110	Grand Index
25	f	17+ years	0.753	862	110	Grand Index
25	f	17+ years	0.754	862.0023049	110	Grand Index
25	f	17+ years	0.757	863	110	Grand Index
25	f	17+ years	0.758	863.784	110	Grand Index
25	f	17+ years	0.761	864	111	Grand Index
25	f	17+ years	0.762	864.7578386	111	Grand Index
25	f	17+ years	0.765	865	111	Grand Index
25	f	17+ years	0.766	865.0287339	111	Grand Index
25	f	17+ years	0.77	866	111	Grand Index
25	f	17+ years	0.771	866.6392883	111	Grand Index
25	f	17+ years	0.772	866.9658671	111	Grand Index
25	f	17+ years	0.773	867	111	Grand Index
25	f	17+ years	0.774	867.352	111	Grand Index
25	f	17+ years	0.777	868	111	Grand Index
25	f	17+ years	0.778	868.744	111	Grand Index
25	f	17+ years	0.78	869	112	Grand Index
25	f	17+ years	0.781	869.066308	112	Grand Index
25	f	17+ years	0.782	869.6349826	112	Grand Index
25	f	17+ years	0.783	870	112	Grand Index
25	f	17+ years	0.784	870.7704789	112	Grand Index
25	f	17+ years	0.786	871	112	Grand Index
25	f	17+ years	0.787	871.0065202	112	Grand Index
25	f	17+ years	0.788	872	112	Grand Index
25	f	17+ years	0.789	872.7971039	112	Grand Index
25	f	17+ years	0.792	873	112	Grand Index
25	f	17+ years	0.793	873.2617935	112	Grand Index
25	f	17+ years	0.798	874	113	Grand Index
25	f	17+ years	0.799	875	113	Grand Index
25	f	17+ years	0.8	875.8757318	113	Grand Index
25	f	17+ years	0.801	876	113	Grand Index
25	f	17+ years	0.802	876.1263216	113	Grand Index
25	f	17+ years	0.806	877	113	Grand Index

Age	Gender	Education	Percentile	Raw Score	Norm Score	Assessment
25	f	17+ years	0.807	877.2971561	113	Grand Index
25	f	17+ years	0.81	878	113	Grand Index
25	f	17+ years	0.812	879	113	Grand Index
25	f	17+ years	0.813	879.0844957	113	Grand Index
25	f	17+ years	0.816	880	114	Grand Index
25	f	17+ years	0.817	880.816	114	Grand Index
25	f	17+ years	0.818	881	114	Grand Index
25	f	17+ years	0.823	882	114	Grand Index
25	f	17+ years	0.825	883	114	Grand Index
25	f	17+ years	0.826	883.4921684	114	Grand Index
25	f	17+ years	0.829	884	114	Grand Index
25	f	17+ years	0.83	884.3698474	114	Grand Index
25	f	17+ years	0.831	884.999076	114	Grand Index
25	f	17+ years	0.835	885	115	Grand Index
25	f	17+ years	0.836	885.534303	115	Grand Index
25	f	17+ years	0.838	886	115	Grand Index
25	f	17+ years	0.839	886.472	115	Grand Index
25	f	17+ years	0.84	887	115	Grand Index
25	f	17+ years	0.841	887.3104802	115	Grand Index
25	f	17+ years	0.844	888	115	Grand Index
25	f	17+ years	0.845	888.6661933	115	Grand Index
25	f	17+ years	0.847	889	115	Grand Index
25	f	17+ years	0.848	889.355215	115	Grand Index
25	f	17+ years	0.85	890	116	Grand Index
25	f	17+ years	0.851	890.5081421	116	Grand Index
25	f	17+ years	0.852	890.9036279	116	Grand Index
25	f	17+ years	0.853	891.344	116	Grand Index
25	f	17+ years	0.855	892	116	Grand Index
25	f	17+ years	0.856	892.142042	116	Grand Index
25	f	17+ years	0.857	892.9660252	116	Grand Index
25	f	17+ years	0.858	893	116	Grand Index
25	f	17+ years	0.859	893.7245168	116	Grand Index
25	f	17+ years	0.862	894	116	Grand Index
25	f	17+ years	0.863	894.824	116	Grand Index
25	f	17+ years	0.865	895	117	Grand Index
25	f	17+ years	0.866	895.1041509	117	Grand Index
25	f	17+ years	0.869	896	117	Grand Index
25	f	17+ years	0.87	896.0344802	117	Grand Index
25	f	17+ years	0.873	897	117	Grand Index
25	f	17+ years	0.874	897.152	117	Grand Index
25	f	17+ years	0.876	898	117	Grand Index
25	f	17+ years	0.877	898.696	117	Grand Index
25	f	17+ years	0.878	899.544	117	Grand Index
25	f	17+ years	0.879	900	118	Grand Index
25	f	17+ years	0.88	901	118	Grand Index
25	f	17+ years	0.881	901.0654939	118	Grand Index
25	f	17+ years	0.882	901.9836319	118	Grand Index
25	f	17+ years	0.884	902	118	Grand Index
25	f	17+ years	0.885	903	118	Grand Index
25	f	17+ years	0.886	903.328	118	Grand Index
25	f	17+ years	0.889	904	118	Grand Index
25	f	17+ years	0.891	905	118	Grand Index
25	f	17+ years	0.892	905.1797673	119	Grand Index
25	f	17+ years	0.893	906	119	Grand Index
25	f	17+ years	0.894	906.0014015	119	Grand Index
25	f	17+ years	0.895	906.9605005	119	Grand Index

Age	Gender	Education	Percentile	Raw Score	Norm Score	Assessment
25	f	17+ years	0.896	907	119	Grand Index
25	f	17+ years	0.897	907.6327273	119	Grand Index
25	f	17+ years	0.898	908	119	Grand Index
25	f	17+ years	0.899	908.1981287	119	Grand Index
25	f	17+ years	0.9	909	119	Grand Index
25	f	17+ years	0.901	909.0013585	119	Grand Index
25	f	17+ years	0.902	910.7064496	119	Grand Index
25	f	17+ years	0.903	911	119	Grand Index
25	f	17+ years	0.904	911.5720862	120	Grand Index
25	f	17+ years	0.905	912	120	Grand Index
25	f	17+ years	0.908	913	120	Grand Index
25	f	17+ years	0.909	913.5653424	120	Grand Index
25	f	17+ years	0.91	914	120	Grand Index
25	f	17+ years	0.911	914.0210421	120	Grand Index
25	f	17+ years	0.912	915	120	Grand Index
25	f	17+ years	0.913	915.0327625	120	Grand Index
25	f	17+ years	0.914	916.072	120	Grand Index
25	f	17+ years	0.917	917	121	Grand Index
25	f	17+ years	0.918	917.2790975	121	Grand Index
25	f	17+ years	0.92	918	121	Grand Index
25	f	17+ years	0.921	918.3116538	121	Grand Index
25	f	17+ years	0.922	918.9134168	121	Grand Index
25	f	17+ years	0.923	919	121	Grand Index
25	f	17+ years	0.924	919.0304289	121	Grand Index
25	f	17+ years	0.925	920	122	Grand Index
25	f	17+ years	0.926	920.8087018	122	Grand Index
25	f	17+ years	0.927	921	122	Grand Index
25	f	17+ years	0.928	921.944	122	Grand Index
25	f	17+ years	0.929	922.4099853	122	Grand Index
25	f	17+ years	0.93	923	122	Grand Index
25	f	17+ years	0.931	923.976	122	Grand Index
25	f	17+ years	0.932	925.8933372	122	Grand Index
25	f	17+ years	0.933	927	122	Grand Index
25	f	17+ years	0.934	927.032	123	Grand Index
25	f	17+ years	0.936	928	123	Grand Index
25	f	17+ years	0.937	928.3428757	123	Grand Index
25	f	17+ years	0.938	929	123	Grand Index
25	f	17+ years	0.939	929.2503268	123	Grand Index
25	f	17+ years	0.94	930	123	Grand Index
25	f	17+ years	0.941	930.968	123	Grand Index
25	f	17+ years	0.942	932	124	Grand Index
25	f	17+ years	0.943	932.664	124	Grand Index
25	f	17+ years	0.944	933.521593	124	Grand Index
25	f	17+ years	0.945	934	124	Grand Index
25	f	17+ years	0.946	934.9836605	124	Grand Index
25	f	17+ years	0.947	935	124	Grand Index
25	f	17+ years	0.948	935.904	124	Grand Index
25	f	17+ years	0.949	936.6855805	125	Grand Index
25	f	17+ years	0.95	937	125	Grand Index
25	f	17+ years	0.951	937.0188812	125	Grand Index
25	f	17+ years	0.952	938.296	125	Grand Index
25	f	17+ years	0.953	939.941207	125	Grand Index
25	f	17+ years	0.954	941	125	Grand Index
25	f	17+ years	0.955	941.84	125	Grand Index
25	f	17+ years	0.956	942	126	Grand Index
25	f	17+ years	0.957	942.183697	126	Grand Index

Age	Gender	Education	Percentile	Raw Score	Norm Score	Assessment
25	f	17+ years	0.958	942.9314287	126	Grand Index
25	f	17+ years	0.959	943.0859416	126	Grand Index
25	f	17+ years	0.961	944	126	Grand Index
25	f	17+ years	0.962	944.776	127	Grand Index
25	f	17+ years	0.963	946.624	127	Grand Index
25	f	17+ years	0.964	947.0716833	127	Grand Index
25	f	17+ years	0.965	948.369131	127	Grand Index
25	f	17+ years	0.966	949.168	127	Grand Index
25	f	17+ years	0.967	950.079948	128	Grand Index
25	f	17+ years	0.968	951	128	Grand Index
25	f	17+ years	0.969	951.712	128	Grand Index
25	f	17+ years	0.97	952.56	128	Grand Index
25	f	17+ years	0.971	953	128	Grand Index
25	f	17+ years	0.972	953.256	129	Grand Index
25	f	17+ years	0.973	954.208	129	Grand Index
25	f	17+ years	0.974	956.1476117	129	Grand Index
25	f	17+ years	0.975	957.9385933	129	Grand Index
25	f	17+ years	0.976	958.191937	130	Grand Index
25	f	17+ years	0.977	960.5102642	130	Grand Index
25	f	17+ years	0.978	961.1207547	130	Grand Index
25	f	17+ years	0.979	965.96	131	Grand Index
25	f	17+ years	0.98	970.0025145	131	Grand Index
25	f	17+ years	0.981	971.7830406	131	Grand Index
25	f	17+ years	0.982	972.7389945	131	Grand Index
25	f	17+ years	0.983	974.5625954	132	Grand Index
25	f	17+ years	0.984	975.7698901	132	Grand Index
25	f	17+ years	0.985	982.0005186	133	Grand Index
25	f	17+ years	0.986	983	133	Grand Index
25	f	17+ years	0.987	986.904	133	Grand Index
25	f	17+ years	0.988	988	134	Grand Index
25	f	17+ years	0.989	991.3367439	134	Grand Index
25	f	17+ years	0.99	992.8871409	135	Grand Index
25	f	17+ years	0.991	994.2266057	135	Grand Index
25	f	17+ years	0.992	997.9764721	136	Grand Index
25	f	17+ years	0.993	1004.547177	137	Grand Index
25	f	17+ years	0.994	1007.790534	138	Grand Index
25	f	17+ years	0.995	1015.933259	139	Grand Index
25	f	17+ years	0.996	1018.983929	140	Grand Index
25	f	17+ years	0.997	1044.870713	141	Grand Index
25	f	17+ years	0.998	1049.498717	143	Grand Index
25	f	17+ years	0.999	1061.122617	146	Grand Index
25	m	17+ years	0.001	604.6132971	54	Grand Index
25	m	17+ years	0.002	612.056	57	Grand Index
25	m	17+ years	0.003	623.1999071	59	Grand Index
25	m	17+ years	0.004	626.4978291	60	Grand Index
25	m	17+ years	0.005	628.8564341	61	Grand Index
25	m	17+ years	0.006	632.6687575	62	Grand Index
25	m	17+ years	0.007	637.281061	63	Grand Index
25	m	17+ years	0.008	639.5519275	64	Grand Index
25	m	17+ years	0.009	641.6216291	65	Grand Index
25	m	17+ years	0.01	642.9476793	65	Grand Index
25	m	17+ years	0.011	643.8146778	66	Grand Index
25	m	17+ years	0.012	645.792	66	Grand Index
25	m	17+ years	0.013	646.9698456	67	Grand Index
25	m	17+ years	0.014	649.848	67	Grand Index
25	m	17+ years	0.015	651.98	67	Grand Index

Age	Gender	Education	Percentile	Raw Score	Norm Score	Assessment
25	m	17+ years	0.016	655	68	Grand Index
25	m	17+ years	0.017	659.0064032	68	Grand Index
25	m	17+ years	0.018	660.188	69	Grand Index
25	m	17+ years	0.019	661.254	69	Grand Index
25	m	17+ years	0.02	664	69	Grand Index
25	m	17+ years	0.021	665	69	Grand Index
25	m	17+ years	0.022	667.452	70	Grand Index
25	m	17+ years	0.024	668	70	Grand Index
25	m	17+ years	0.025	669.5957328	71	Grand Index
25	m	17+ years	0.026	670	71	Grand Index
25	m	17+ years	0.027	671.782	71	Grand Index
25	m	17+ years	0.028	672.1481531	71	Grand Index
25	m	17+ years	0.03	673	72	Grand Index
25	m	17+ years	0.031	675	72	Grand Index
25	m	17+ years	0.032	675.3065257	72	Grand Index
25	m	17+ years	0.033	676.2430673	72	Grand Index
25	m	17+ years	0.034	677.9485412	73	Grand Index
25	m	17+ years	0.035	678.0221484	73	Grand Index
25	m	17+ years	0.036	678.9188086	73	Grand Index
25	m	17+ years	0.037	679.0622054	73	Grand Index
25	m	17+ years	0.038	680.5073174	73	Grand Index
25	m	17+ years	0.039	681	74	Grand Index
25	m	17+ years	0.04	681.6554526	74	Grand Index
25	m	17+ years	0.041	682	74	Grand Index
25	m	17+ years	0.042	682.1216799	74	Grand Index
25	m	17+ years	0.043	683.150774	74	Grand Index
25	m	17+ years	0.044	684.0706986	74	Grand Index
25	m	17+ years	0.045	686	75	Grand Index
25	m	17+ years	0.046	686.8944839	75	Grand Index
25	m	17+ years	0.047	687	75	Grand Index
25	m	17+ years	0.048	687.168	75	Grand Index
25	m	17+ years	0.049	688.1897501	75	Grand Index
25	m	17+ years	0.05	689	75	Grand Index
25	m	17+ years	0.051	689.1375671	75	Grand Index
25	m	17+ years	0.053	690	76	Grand Index
25	m	17+ years	0.054	690.7807612	76	Grand Index
25	m	17+ years	0.055	691.5939888	76	Grand Index
25	m	17+ years	0.056	691.9608185	76	Grand Index
25	m	17+ years	0.057	692.234868	76	Grand Index
25	m	17+ years	0.058	693	76	Grand Index
25	m	17+ years	0.059	693.7511034	77	Grand Index
25	m	17+ years	0.06	693.9995385	77	Grand Index
25	m	17+ years	0.061	694.9782165	77	Grand Index
25	m	17+ years	0.062	695	77	Grand Index
25	m	17+ years	0.063	695.158	77	Grand Index
25	m	17+ years	0.065	696	77	Grand Index
25	m	17+ years	0.066	696.3616541	77	Grand Index
25	m	17+ years	0.067	697	78	Grand Index
25	m	17+ years	0.068	697.625361	78	Grand Index
25	m	17+ years	0.069	698.554	78	Grand Index
25	m	17+ years	0.07	699.62	78	Grand Index
25	m	17+ years	0.071	700.2299179	78	Grand Index
25	m	17+ years	0.072	701.076999	78	Grand Index
25	m	17+ years	0.073	702	78	Grand Index
25	m	17+ years	0.075	703	78	Grand Index
25	m	17+ years	0.078	704	79	Grand Index

Age	Gender	Education	Percentile	Raw Score	Norm Score	Assessment
25	m	17+ years	0.079	704.214	79	Grand Index
25	m	17+ years	0.082	705	79	Grand Index
25	m	17+ years	0.083	705.8587155	79	Grand Index
25	m	17+ years	0.085	707	79	Grand Index
25	m	17+ years	0.086	707.5193806	80	Grand Index
25	m	17+ years	0.088	708	80	Grand Index
25	m	17+ years	0.089	708.8834384	80	Grand Index
25	m	17+ years	0.09	709	80	Grand Index
25	m	17+ years	0.091	709.8062055	80	Grand Index
25	m	17+ years	0.092	710	80	Grand Index
25	m	17+ years	0.093	710.2924417	80	Grand Index
25	m	17+ years	0.096	711	80	Grand Index
25	m	17+ years	0.097	711.0997265	81	Grand Index
25	m	17+ years	0.098	712	81	Grand Index
25	m	17+ years	0.099	712.3236306	81	Grand Index
25	m	17+ years	0.1	712.7387426	81	Grand Index
25	m	17+ years	0.101	713	81	Grand Index
25	m	17+ years	0.102	713.732	81	Grand Index
25	m	17+ years	0.105	715	81	Grand Index
25	m	17+ years	0.106	715.9977059	81	Grand Index
25	m	17+ years	0.107	716.1476686	81	Grand Index
25	m	17+ years	0.108	716.4408603	81	Grand Index
25	m	17+ years	0.111	717	82	Grand Index
25	m	17+ years	0.112	717.7461534	82	Grand Index
25	m	17+ years	0.113	718.458	82	Grand Index
25	m	17+ years	0.115	719	82	Grand Index
25	m	17+ years	0.116	719.656	82	Grand Index
25	m	17+ years	0.118	720	82	Grand Index
25	m	17+ years	0.119	720.1010021	82	Grand Index
25	m	17+ years	0.12	720.9846685	82	Grand Index
25	m	17+ years	0.123	721	83	Grand Index
25	m	17+ years	0.124	721.8490008	83	Grand Index
25	m	17+ years	0.125	722.25	83	Grand Index
25	m	17+ years	0.126	723	83	Grand Index
25	m	17+ years	0.13	724	83	Grand Index
25	m	17+ years	0.131	724.7877917	83	Grand Index
25	m	17+ years	0.132	725	83	Grand Index
25	m	17+ years	0.133	725.0959467	83	Grand Index
25	m	17+ years	0.135	726	83	Grand Index
25	m	17+ years	0.136	726.9996419	84	Grand Index
25	m	17+ years	0.137	727.0165186	84	Grand Index
25	m	17+ years	0.138	727.7292709	84	Grand Index
25	m	17+ years	0.139	728	84	Grand Index
25	m	17+ years	0.14	728.1795178	84	Grand Index
25	m	17+ years	0.141	729	84	Grand Index
25	m	17+ years	0.144	730	84	Grand Index
25	m	17+ years	0.145	730.57	84	Grand Index
25	m	17+ years	0.146	731.636	84	Grand Index
25	m	17+ years	0.148	732	84	Grand Index
25	m	17+ years	0.149	732.0809031	84	Grand Index
25	m	17+ years	0.15	733	84	Grand Index
25	m	17+ years	0.151	733.990207	85	Grand Index
25	m	17+ years	0.153	735	85	Grand Index
25	m	17+ years	0.154	735.1122488	85	Grand Index
25	m	17+ years	0.155	735.9857468	85	Grand Index
25	m	17+ years	0.157	736	85	Grand Index

Age	Gender	Education	Percentile	Raw Score	Norm Score	Assessment
25	m	17+ years	0.158	736.3855731	85	Grand Index
25	m	17+ years	0.159	737	85	Grand Index
25	m	17+ years	0.16	737.56	85	Grand Index
25	m	17+ years	0.161	738	85	Grand Index
25	m	17+ years	0.162	738.0376688	85	Grand Index
25	m	17+ years	0.163	738.8050964	85	Grand Index
25	m	17+ years	0.164	739.7080142	85	Grand Index
25	m	17+ years	0.168	740	86	Grand Index
25	m	17+ years	0.169	740.154	86	Grand Index
25	m	17+ years	0.171	741	86	Grand Index
25	m	17+ years	0.172	741.1457132	86	Grand Index
25	m	17+ years	0.173	741.8200383	86	Grand Index
25	m	17+ years	0.174	742	86	Grand Index
25	m	17+ years	0.175	742.55	86	Grand Index
25	m	17+ years	0.178	743	86	Grand Index
25	m	17+ years	0.179	743.022	86	Grand Index
25	m	17+ years	0.18	743.6517093	86	Grand Index
25	m	17+ years	0.182	744	86	Grand Index
25	m	17+ years	0.183	744.2352623	86	Grand Index
25	m	17+ years	0.186	745	87	Grand Index
25	m	17+ years	0.187	745.1779779	87	Grand Index
25	m	17+ years	0.188	745.4942888	87	Grand Index
25	m	17+ years	0.192	746	87	Grand Index
25	m	17+ years	0.193	746.0205568	87	Grand Index
25	m	17+ years	0.194	746.1163938	87	Grand Index
25	m	17+ years	0.195	746.9946595	87	Grand Index
25	m	17+ years	0.199	747	87	Grand Index
25	m	17+ years	0.2	747.3767565	87	Grand Index
25	m	17+ years	0.201	748.266	87	Grand Index
25	m	17+ years	0.202	749	87	Grand Index
25	m	17+ years	0.203	749.1063555	88	Grand Index
25	m	17+ years	0.204	750	88	Grand Index
25	m	17+ years	0.205	750.53	88	Grand Index
25	m	17+ years	0.207	751	88	Grand Index
25	m	17+ years	0.208	751.728	88	Grand Index
25	m	17+ years	0.213	752	88	Grand Index
25	m	17+ years	0.214	752.0678788	88	Grand Index
25	m	17+ years	0.216	753	88	Grand Index
25	m	17+ years	0.218	754	88	Grand Index
25	m	17+ years	0.219	754.9856536	88	Grand Index
25	m	17+ years	0.223	755	89	Grand Index
25	m	17+ years	0.224	755.0020592	89	Grand Index
25	m	17+ years	0.228	756	89	Grand Index
25	m	17+ years	0.229	756.114	89	Grand Index
25	m	17+ years	0.232	757	89	Grand Index
25	m	17+ years	0.233	757.2992962	89	Grand Index
25	m	17+ years	0.235	758	89	Grand Index
25	m	17+ years	0.238	759	89	Grand Index
25	m	17+ years	0.239	759.8847176	89	Grand Index
25	m	17+ years	0.241	760	89	Grand Index
25	m	17+ years	0.242	760.0969539	90	Grand Index
25	m	17+ years	0.246	761	90	Grand Index
25	m	17+ years	0.247	761.5621187	90	Grand Index
25	m	17+ years	0.254	762	90	Grand Index
25	m	17+ years	0.255	762.8684922	90	Grand Index
25	m	17+ years	0.258	763	90	Grand Index

Age	Gender	Education	Percentile	Raw Score	Norm Score	Assessment
25	m	17+ years	0.259	763.1759775	90	Grand Index
25	m	17+ years	0.261	764	90	Grand Index
25	m	17+ years	0.262	764.1899654	90	Grand Index
25	m	17+ years	0.263	764.9767064	90	Grand Index
25	m	17+ years	0.265	765	91	Grand Index
25	m	17+ years	0.266	765.0032384	91	Grand Index
25	m	17+ years	0.267	765.9321538	91	Grand Index
25	m	17+ years	0.272	766	91	Grand Index
25	m	17+ years	0.274	767	91	Grand Index
25	m	17+ years	0.275	767.0132793	91	Grand Index
25	m	17+ years	0.276	767.8701389	91	Grand Index
25	m	17+ years	0.28	768	91	Grand Index
25	m	17+ years	0.285	769	91	Grand Index
25	m	17+ years	0.286	769.9196659	92	Grand Index
25	m	17+ years	0.289	770	92	Grand Index
25	m	17+ years	0.294	771	92	Grand Index
25	m	17+ years	0.295	771.4900799	92	Grand Index
25	m	17+ years	0.298	772	92	Grand Index
25	m	17+ years	0.303	773	92	Grand Index
25	m	17+ years	0.309	774	93	Grand Index
25	m	17+ years	0.31	774.0049721	93	Grand Index
25	m	17+ years	0.314	775	93	Grand Index
25	m	17+ years	0.317	776	93	Grand Index
25	m	17+ years	0.318	776.7895373	93	Grand Index
25	m	17+ years	0.321	777	93	Grand Index
25	m	17+ years	0.322	777.0569607	93	Grand Index
25	m	17+ years	0.323	777.7211628	93	Grand Index
25	m	17+ years	0.328	778	93	Grand Index
25	m	17+ years	0.333	779	94	Grand Index
25	m	17+ years	0.339	780	94	Grand Index
25	m	17+ years	0.345	781	94	Grand Index
25	m	17+ years	0.349	782	94	Grand Index
25	m	17+ years	0.35	782.237028	94	Grand Index
25	m	17+ years	0.355	783	94	Grand Index
25	m	17+ years	0.356	783.3184651	94	Grand Index
25	m	17+ years	0.358	784	95	Grand Index
25	m	17+ years	0.359	784.1991751	95	Grand Index
25	m	17+ years	0.36	785	95	Grand Index
25	m	17+ years	0.361	785.051258	95	Grand Index
25	m	17+ years	0.362	785.9945426	95	Grand Index
25	m	17+ years	0.367	786	95	Grand Index
25	m	17+ years	0.368	786.326006	95	Grand Index
25	m	17+ years	0.37	787	95	Grand Index
25	m	17+ years	0.371	787.403517	95	Grand Index
25	m	17+ years	0.372	787.8545745	95	Grand Index
25	m	17+ years	0.377	788	95	Grand Index
25	m	17+ years	0.378	788.7979059	95	Grand Index
25	m	17+ years	0.383	789	96	Grand Index
25	m	17+ years	0.384	789.344	96	Grand Index
25	m	17+ years	0.387	790	96	Grand Index
25	m	17+ years	0.388	790.608	96	Grand Index
25	m	17+ years	0.393	791	96	Grand Index
25	m	17+ years	0.398	792	96	Grand Index
25	m	17+ years	0.399	792.5235623	96	Grand Index
25	m	17+ years	0.406	793	96	Grand Index
25	m	17+ years	0.407	793.4933623	96	Grand Index

Age	Gender	Education	Percentile	Raw Score	Norm Score	Assessment
25	m	17+ years	0.409	794	97	Grand Index
25	m	17+ years	0.41	794.0040588	97	Grand Index
25	m	17+ years	0.411	794.9106765	97	Grand Index
25	m	17+ years	0.415	795	97	Grand Index
25	m	17+ years	0.416	795.1522913	97	Grand Index
25	m	17+ years	0.417	795.9963089	97	Grand Index
25	m	17+ years	0.422	796	97	Grand Index
25	m	17+ years	0.423	796.6720222	97	Grand Index
25	m	17+ years	0.43	797	97	Grand Index
25	m	17+ years	0.431	797.1248594	97	Grand Index
25	m	17+ years	0.432	797.9513536	97	Grand Index
25	m	17+ years	0.434	798	98	Grand Index
25	m	17+ years	0.435	798.589434	98	Grand Index
25	m	17+ years	0.438	799	98	Grand Index
25	m	17+ years	0.439	799.9684048	98	Grand Index
25	m	17+ years	0.443	800	98	Grand Index
25	m	17+ years	0.444	800.304	98	Grand Index
25	m	17+ years	0.446	801	98	Grand Index
25	m	17+ years	0.447	801.3205672	98	Grand Index
25	m	17+ years	0.45	802	98	Grand Index
25	m	17+ years	0.451	802.766	98	Grand Index
25	m	17+ years	0.454	803	98	Grand Index
25	m	17+ years	0.455	803.03	98	Grand Index
25	m	17+ years	0.457	804	98	Grand Index
25	m	17+ years	0.458	804.228	98	Grand Index
25	m	17+ years	0.463	805	99	Grand Index
25	m	17+ years	0.464	805.6282933	99	Grand Index
25	m	17+ years	0.472	806	99	Grand Index
25	m	17+ years	0.474	807	99	Grand Index
25	m	17+ years	0.48	808	99	Grand Index
25	m	17+ years	0.481	808.5193525	99	Grand Index
25	m	17+ years	0.485	809	99	Grand Index
25	m	17+ years	0.486	809.0696283	99	Grand Index
25	m	17+ years	0.487	809.995607	100	Grand Index
25	m	17+ years	0.493	810	100	Grand Index
25	m	17+ years	0.494	810.9616056	100	Grand Index
25	m	17+ years	0.499	811	100	Grand Index
25	m	17+ years	0.503	812	100	Grand Index
25	m	17+ years	0.504	812.264	100	Grand Index
25	m	17+ years	0.51	813	100	Grand Index
25	m	17+ years	0.515	814	101	Grand Index
25	m	17+ years	0.516	814.225618	101	Grand Index
25	m	17+ years	0.517	814.963481	101	Grand Index
25	m	17+ years	0.521	815	101	Grand Index
25	m	17+ years	0.522	815.3549536	101	Grand Index
25	m	17+ years	0.527	816	101	Grand Index
25	m	17+ years	0.528	816.969676	101	Grand Index
25	m	17+ years	0.534	817	101	Grand Index
25	m	17+ years	0.535	817.5333291	101	Grand Index
25	m	17+ years	0.541	818	102	Grand Index
25	m	17+ years	0.542	818.9348239	102	Grand Index
25	m	17+ years	0.547	819	102	Grand Index
25	m	17+ years	0.548	819.2484022	102	Grand Index
25	m	17+ years	0.549	819.540272	102	Grand Index
25	m	17+ years	0.554	820	102	Grand Index
25	m	17+ years	0.555	820.0772616	102	Grand Index

Age	Gender	Education	Percentile	Raw Score	Norm Score	Assessment
25	m	17+ years	0.561	821	102	Grand Index
25	m	17+ years	0.564	822	102	Grand Index
25	m	17+ years	0.565	822.9239572	102	Grand Index
25	m	17+ years	0.568	823	103	Grand Index
25	m	17+ years	0.572	824	103	Grand Index
25	m	17+ years	0.573	824.0824159	103	Grand Index
25	m	17+ years	0.574	824.9113844	103	Grand Index
25	m	17+ years	0.577	825	103	Grand Index
25	m	17+ years	0.582	826	103	Grand Index
25	m	17+ years	0.583	826.7926193	103	Grand Index
25	m	17+ years	0.586	827	103	Grand Index
25	m	17+ years	0.587	827.7506483	103	Grand Index
25	m	17+ years	0.59	828	103	Grand Index
25	m	17+ years	0.591	828.92761	103	Grand Index
25	m	17+ years	0.595	829	104	Grand Index
25	m	17+ years	0.596	829.0440617	104	Grand Index
25	m	17+ years	0.597	829.7858485	104	Grand Index
25	m	17+ years	0.603	830	104	Grand Index
25	m	17+ years	0.604	830.9218848	104	Grand Index
25	m	17+ years	0.609	831	104	Grand Index
25	m	17+ years	0.61	831.1601559	104	Grand Index
25	m	17+ years	0.614	832	104	Grand Index
25	m	17+ years	0.615	832.5073304	104	Grand Index
25	m	17+ years	0.62	833	105	Grand Index
25	m	17+ years	0.621	833.986	105	Grand Index
25	m	17+ years	0.625	834	105	Grand Index
25	m	17+ years	0.629	835	105	Grand Index
25	m	17+ years	0.632	836	105	Grand Index
25	m	17+ years	0.633	836.2582159	105	Grand Index
25	m	17+ years	0.634	836.9691233	105	Grand Index
25	m	17+ years	0.635	837.2588849	105	Grand Index
25	m	17+ years	0.636	837.7748785	105	Grand Index
25	m	17+ years	0.64	838	105	Grand Index
25	m	17+ years	0.641	838.1490008	105	Grand Index
25	m	17+ years	0.644	839	106	Grand Index
25	m	17+ years	0.645	839.0143818	106	Grand Index
25	m	17+ years	0.649	840	106	Grand Index
25	m	17+ years	0.656	841	106	Grand Index
25	m	17+ years	0.657	841.9692	106	Grand Index
25	m	17+ years	0.66	842	106	Grand Index
25	m	17+ years	0.661	842.203475	106	Grand Index
25	m	17+ years	0.666	843	106	Grand Index
25	m	17+ years	0.671	844	107	Grand Index
25	m	17+ years	0.675	845	107	Grand Index
25	m	17+ years	0.676	845.616	107	Grand Index
25	m	17+ years	0.68	846	107	Grand Index
25	m	17+ years	0.681	846.946	107	Grand Index
25	m	17+ years	0.685	847	107	Grand Index
25	m	17+ years	0.686	847.1301536	107	Grand Index
25	m	17+ years	0.69	848	107	Grand Index
25	m	17+ years	0.691	848.606	107	Grand Index
25	m	17+ years	0.695	849	108	Grand Index
25	m	17+ years	0.697	850	108	Grand Index
25	m	17+ years	0.698	850.3550061	108	Grand Index
25	m	17+ years	0.701	851	108	Grand Index
25	m	17+ years	0.702	851.0177926	108	Grand Index

Age	Gender	Education	Percentile	Raw Score	Norm Score	Assessment
25	m	17+ years	0.706	852	108	Grand Index
25	m	17+ years	0.707	852.662	108	Grand Index
25	m	17+ years	0.709	853	108	Grand Index
25	m	17+ years	0.712	854	108	Grand Index
25	m	17+ years	0.713	854.368106	108	Grand Index
25	m	17+ years	0.714	855.124	108	Grand Index
25	m	17+ years	0.716	856	109	Grand Index
25	m	17+ years	0.717	856.0444021	109	Grand Index
25	m	17+ years	0.718	856.5537744	109	Grand Index
25	m	17+ years	0.724	857	109	Grand Index
25	m	17+ years	0.725	857.7610895	109	Grand Index
25	m	17+ years	0.728	858	109	Grand Index
25	m	17+ years	0.729	858.6758982	109	Grand Index
25	m	17+ years	0.733	859	109	Grand Index
25	m	17+ years	0.734	859.444	109	Grand Index
25	m	17+ years	0.736	860	109	Grand Index
25	m	17+ years	0.737	860.9326784	110	Grand Index
25	m	17+ years	0.742	861	110	Grand Index
25	m	17+ years	0.746	862	110	Grand Index
25	m	17+ years	0.747	862.0707554	110	Grand Index
25	m	17+ years	0.749	863	110	Grand Index
25	m	17+ years	0.75	863.6991433	110	Grand Index
25	m	17+ years	0.754	864	110	Grand Index
25	m	17+ years	0.757	865	110	Grand Index
25	m	17+ years	0.758	865.9916509	110	Grand Index
25	m	17+ years	0.761	866	111	Grand Index
25	m	17+ years	0.762	866.2366642	111	Grand Index
25	m	17+ years	0.765	867	111	Grand Index
25	m	17+ years	0.766	867.537315	111	Grand Index
25	m	17+ years	0.767	868	111	Grand Index
25	m	17+ years	0.768	868.3345584	111	Grand Index
25	m	17+ years	0.769	868.9126231	111	Grand Index
25	m	17+ years	0.772	869	111	Grand Index
25	m	17+ years	0.773	869.018	111	Grand Index
25	m	17+ years	0.776	870	111	Grand Index
25	m	17+ years	0.777	870.3463724	111	Grand Index
25	m	17+ years	0.782	871	112	Grand Index
25	m	17+ years	0.783	871.0148564	112	Grand Index
25	m	17+ years	0.787	872	112	Grand Index
25	m	17+ years	0.788	872.008	112	Grand Index
25	m	17+ years	0.791	873	112	Grand Index
25	m	17+ years	0.792	873.4637718	112	Grand Index
25	m	17+ years	0.793	874	112	Grand Index
25	m	17+ years	0.794	874.404	112	Grand Index
25	m	17+ years	0.797	875	112	Grand Index
25	m	17+ years	0.798	875.0404601	113	Grand Index
25	m	17+ years	0.799	875.7464736	113	Grand Index
25	m	17+ years	0.802	876	113	Grand Index
25	m	17+ years	0.803	876.9980154	113	Grand Index
25	m	17+ years	0.804	877	113	Grand Index
25	m	17+ years	0.805	877.1774122	113	Grand Index
25	m	17+ years	0.806	877.9808584	113	Grand Index
25	m	17+ years	0.81	878	113	Grand Index
25	m	17+ years	0.811	878.5464222	113	Grand Index
25	m	17+ years	0.812	879	113	Grand Index
25	m	17+ years	0.813	879.658	113	Grand Index

Age	Gender	Education	Percentile	Raw Score	Norm Score	Assessment
25	m	17+ years	0.817	880	114	Grand Index
25	m	17+ years	0.819	881	114	Grand Index
25	m	17+ years	0.82	881.12	114	Grand Index
25	m	17+ years	0.821	882	114	Grand Index
25	m	17+ years	0.822	882.0689364	114	Grand Index
25	m	17+ years	0.824	883	114	Grand Index
25	m	17+ years	0.825	883.5143954	114	Grand Index
25	m	17+ years	0.827	884	114	Grand Index
25	m	17+ years	0.828	884.132085	114	Grand Index
25	m	17+ years	0.829	885	114	Grand Index
25	m	17+ years	0.83	885.7527773	114	Grand Index
25	m	17+ years	0.832	886	114	Grand Index
25	m	17+ years	0.833	886.5384406	114	Grand Index
25	m	17+ years	0.834	886.8723173	115	Grand Index
25	m	17+ years	0.837	887	115	Grand Index
25	m	17+ years	0.838	888	115	Grand Index
25	m	17+ years	0.839	888.0815176	115	Grand Index
25	m	17+ years	0.842	889	115	Grand Index
25	m	17+ years	0.843	889.5833969	115	Grand Index
25	m	17+ years	0.846	890	115	Grand Index
25	m	17+ years	0.847	890.9036073	115	Grand Index
25	m	17+ years	0.848	891	115	Grand Index
25	m	17+ years	0.849	891.0002867	115	Grand Index
25	m	17+ years	0.85	891.242528	116	Grand Index
25	m	17+ years	0.851	892.166	116	Grand Index
25	m	17+ years	0.853	893	116	Grand Index
25	m	17+ years	0.856	894	116	Grand Index
25	m	17+ years	0.857	894.9185084	116	Grand Index
25	m	17+ years	0.859	895	116	Grand Index
25	m	17+ years	0.86	895.4321134	116	Grand Index
25	m	17+ years	0.863	896	116	Grand Index
25	m	17+ years	0.864	896.0320468	116	Grand Index
25	m	17+ years	0.865	897	117	Grand Index
25	m	17+ years	0.866	897.0936635	117	Grand Index
25	m	17+ years	0.867	898	117	Grand Index
25	m	17+ years	0.871	899	117	Grand Index
25	m	17+ years	0.872	899.552	117	Grand Index
25	m	17+ years	0.874	900	117	Grand Index
25	m	17+ years	0.876	901	117	Grand Index
25	m	17+ years	0.878	902	117	Grand Index
25	m	17+ years	0.879	902.0055911	118	Grand Index
25	m	17+ years	0.88	902.6908573	118	Grand Index
25	m	17+ years	0.882	903	118	Grand Index
25	m	17+ years	0.883	903.0315183	118	Grand Index
25	m	17+ years	0.884	903.8379179	118	Grand Index
25	m	17+ years	0.887	905	118	Grand Index
25	m	17+ years	0.888	905.6057531	118	Grand Index
25	m	17+ years	0.89	906	118	Grand Index
25	m	17+ years	0.891	907	118	Grand Index
25	m	17+ years	0.893	908	119	Grand Index
25	m	17+ years	0.894	908.5777327	119	Grand Index
25	m	17+ years	0.896	909	119	Grand Index
25	m	17+ years	0.897	909.8391892	119	Grand Index
25	m	17+ years	0.898	910.0142121	119	Grand Index
25	m	17+ years	0.899	910.5892824	119	Grand Index
25	m	17+ years	0.901	911	119	Grand Index

Age	Gender	Education	Percentile	Raw Score	Norm Score	Assessment
25	m	17+ years	0.904	912	120	Grand Index
25	m	17+ years	0.905	913.1575915	120	Grand Index
25	m	17+ years	0.909	914	120	Grand Index
25	m	17+ years	0.911	915	120	Grand Index
25	m	17+ years	0.912	915.192	120	Grand Index
25	m	17+ years	0.913	916	120	Grand Index
25	m	17+ years	0.914	916.324	120	Grand Index
25	m	17+ years	0.916	917	121	Grand Index
25	m	17+ years	0.917	917.0468	121	Grand Index
25	m	17+ years	0.919	918	121	Grand Index
25	m	17+ years	0.92	918.8592107	121	Grand Index
25	m	17+ years	0.921	919	121	Grand Index
25	m	17+ years	0.922	920	121	Grand Index
25	m	17+ years	0.923	920.918	121	Grand Index
25	m	17+ years	0.924	921.984	121	Grand Index
25	m	17+ years	0.925	922.020422	122	Grand Index
25	m	17+ years	0.927	923	122	Grand Index
25	m	17+ years	0.928	923.248	122	Grand Index
25	m	17+ years	0.929	924	122	Grand Index
25	m	17+ years	0.93	924.4029658	122	Grand Index
25	m	17+ years	0.931	925.0814358	122	Grand Index
25	m	17+ years	0.932	926	122	Grand Index
25	m	17+ years	0.933	926.578	122	Grand Index
25	m	17+ years	0.934	928.0170338	123	Grand Index
25	m	17+ years	0.935	928.936817	123	Grand Index
25	m	17+ years	0.936	929.2165869	123	Grand Index
25	m	17+ years	0.937	930.9921571	123	Grand Index
25	m	17+ years	0.938	931.2312787	123	Grand Index
25	m	17+ years	0.939	931.9999331	123	Grand Index
25	m	17+ years	0.94	934.1133238	123	Grand Index
25	m	17+ years	0.941	935	123	Grand Index
25	m	17+ years	0.942	936	124	Grand Index
25	m	17+ years	0.943	936.0156703	124	Grand Index
25	m	17+ years	0.944	937	124	Grand Index
25	m	17+ years	0.945	937.171928	124	Grand Index
25	m	17+ years	0.946	938	124	Grand Index
25	m	17+ years	0.947	938.502	124	Grand Index
25	m	17+ years	0.948	939	124	Grand Index
25	m	17+ years	0.949	940.634	125	Grand Index
25	m	17+ years	0.95	941	125	Grand Index
25	m	17+ years	0.951	942	125	Grand Index
25	m	17+ years	0.952	942.832	125	Grand Index
25	m	17+ years	0.953	943	125	Grand Index
25	m	17+ years	0.954	943.9480626	125	Grand Index
25	m	17+ years	0.956	944	126	Grand Index
25	m	17+ years	0.957	945.0078676	126	Grand Index
25	m	17+ years	0.958	946	126	Grand Index
25	m	17+ years	0.959	947.2323581	126	Grand Index
25	m	17+ years	0.96	948.9110183	126	Grand Index
25	m	17+ years	0.961	949.2518373	126	Grand Index
25	m	17+ years	0.962	950.492	127	Grand Index
25	m	17+ years	0.963	951.5734912	127	Grand Index
25	m	17+ years	0.964	953.960582	127	Grand Index
25	m	17+ years	0.965	956.1856896	127	Grand Index
25	m	17+ years	0.966	956.9769717	127	Grand Index
25	m	17+ years	0.967	958.8174532	128	Grand Index

Age	Gender	Education	Percentile	Raw Score	Norm Score	Assessment
25	m	17+ years	0.968	959.6285282	128	Grand Index
25	m	17+ years	0.969	961.2361815	128	Grand Index
25	m	17+ years	0.97	963.75612	128	Grand Index
25	m	17+ years	0.971	965.4265997	128	Grand Index
25	m	17+ years	0.972	966.0110096	129	Grand Index
25	m	17+ years	0.973	967.0383062	129	Grand Index
25	m	17+ years	0.974	969	129	Grand Index
25	m	17+ years	0.975	970.0013143	129	Grand Index
25	m	17+ years	0.976	970.576561	130	Grand Index
25	m	17+ years	0.977	971.7793709	130	Grand Index
25	m	17+ years	0.978	974.5467208	130	Grand Index
25	m	17+ years	0.979	975.614	131	Grand Index
25	m	17+ years	0.98	977.7303123	131	Grand Index
25	m	17+ years	0.981	979.8089043	131	Grand Index
25	m	17+ years	0.982	981	131	Grand Index
25	m	17+ years	0.983	981.9714985	132	Grand Index
25	m	17+ years	0.984	984.8488697	132	Grand Index
25	m	17+ years	0.985	986	133	Grand Index
25	m	17+ years	0.986	986.1115973	133	Grand Index
25	m	17+ years	0.987	990.1145092	133	Grand Index
25	m	17+ years	0.988	991.524887	134	Grand Index
25	m	17+ years	0.989	994.274	134	Grand Index
25	m	17+ years	0.99	995.0180303	135	Grand Index
25	m	17+ years	0.991	999.4536129	135	Grand Index
25	m	17+ years	0.992	1003.337365	136	Grand Index
25	m	17+ years	0.993	1005.66491	137	Grand Index
25	m	17+ years	0.994	1008.604	138	Grand Index
25	m	17+ years	0.995	1015.593543	139	Grand Index
25	m	17+ years	0.996	1025.996169	140	Grand Index
25	m	17+ years	0.997	1030.139454	141	Grand Index
25	m	17+ years	0.998	1037.076	143	Grand Index
25	m	17+ years	0.999	1040.864863	146	Grand Index
30	f	0-12 years	0.001	628.008	54	Grand Index
30	f	0-12 years	0.002	630.016	57	Grand Index
30	f	0-12 years	0.003	632.024	59	Grand Index
30	f	0-12 years	0.004	634.0706515	60	Grand Index
30	f	0-12 years	0.005	638.5040308	61	Grand Index
30	f	0-12 years	0.006	642.9374101	62	Grand Index
30	f	0-12 years	0.007	647.3707893	63	Grand Index
30	f	0-12 years	0.008	651.7134353	64	Grand Index
30	f	0-12 years	0.009	653.300056	65	Grand Index
30	f	0-12 years	0.01	654.8866767	65	Grand Index
30	f	0-12 years	0.011	656.4732974	66	Grand Index
30	f	0-12 years	0.012	657.996255	66	Grand Index
30	f	0-12 years	0.013	658.251255	67	Grand Index
30	f	0-12 years	0.014	658.506255	67	Grand Index
30	f	0-12 years	0.015	658.761255	67	Grand Index
30	f	0-12 years	0.016	659.08	68	Grand Index
30	f	0-12 years	0.017	660.335	68	Grand Index
30	f	0-12 years	0.018	661.59	69	Grand Index
30	f	0-12 years	0.019	662.845	69	Grand Index
30	f	0-12 years	0.02	664.02	69	Grand Index
30	f	0-12 years	0.021	664.271	69	Grand Index
30	f	0-12 years	0.022	664.522	70	Grand Index
30	f	0-12 years	0.023	664.773	70	Grand Index
30	f	0-12 years	0.027	665	71	Grand Index

Age	Gender	Education	Percentile	Raw Score	Norm Score	Assessment
30	f	0-12 years	0.028	665.1097226	71	Grand Index
30	f	0-12 years	0.029	666.0933076	72	Grand Index
30	f	0-12 years	0.03	667.0768925	72	Grand Index
30	f	0-12 years	0.031	668.0604774	72	Grand Index
30	f	0-12 years	0.032	668.9212678	72	Grand Index
30	f	0-12 years	0.033	668.9416829	72	Grand Index
30	f	0-12 years	0.034	668.9620979	73	Grand Index
30	f	0-12 years	0.035	668.982513	73	Grand Index
30	f	0-12 years	0.036	669.108	73	Grand Index
30	f	0-12 years	0.037	669.861	73	Grand Index
30	f	0-12 years	0.038	670.614	73	Grand Index
30	f	0-12 years	0.039	671.367	74	Grand Index
30	f	0-12 years	0.04	672.04	74	Grand Index
30	f	0-12 years	0.041	672.291	74	Grand Index
30	f	0-12 years	0.042	672.542	74	Grand Index
30	f	0-12 years	0.043	672.793	74	Grand Index
30	f	0-12 years	0.044	673.176	74	Grand Index
30	f	0-12 years	0.045	674.18	75	Grand Index
30	f	0-12 years	0.046	675.184	75	Grand Index
30	f	0-12 years	0.047	676.188	75	Grand Index
30	f	0-12 years	0.048	677.24	75	Grand Index
30	f	0-12 years	0.049	678.495	75	Grand Index
30	f	0-12 years	0.05	679.75	75	Grand Index
30	f	0-12 years	0.051	681.005	75	Grand Index
30	f	0-12 years	0.052	682.0504112	76	Grand Index
30	f	0-12 years	0.053	682.293742	76	Grand Index
30	f	0-12 years	0.054	682.5370729	76	Grand Index
30	f	0-12 years	0.055	682.7804038	76	Grand Index
30	f	0-12 years	0.056	683.1391567	76	Grand Index
30	f	0-12 years	0.057	683.8998259	76	Grand Index
30	f	0-12 years	0.058	684.660495	76	Grand Index
30	f	0-12 years	0.059	685.4211641	77	Grand Index
30	f	0-12 years	0.06	686.66	77	Grand Index
30	f	0-12 years	0.061	689.421	77	Grand Index
30	f	0-12 years	0.062	692.182	77	Grand Index
30	f	0-12 years	0.063	694.943	77	Grand Index
30	f	0-12 years	0.067	697	78	Grand Index
30	f	0-12 years	0.068	697.204	78	Grand Index
30	f	0-12 years	0.069	697.957	78	Grand Index
30	f	0-12 years	0.07	698.71	78	Grand Index
30	f	0-12 years	0.071	699.463	78	Grand Index
30	f	0-12 years	0.072	700.144	78	Grand Index
30	f	0-12 years	0.073	700.646	78	Grand Index
30	f	0-12 years	0.074	701.148	78	Grand Index
30	f	0-12 years	0.075	701.65	78	Grand Index
30	f	0-12 years	0.076	702.076	79	Grand Index
30	f	0-12 years	0.077	702.327	79	Grand Index
30	f	0-12 years	0.078	702.578	79	Grand Index
30	f	0-12 years	0.079	702.829	79	Grand Index
30	f	0-12 years	0.08	703.08	79	Grand Index
30	f	0-12 years	0.081	703.331	79	Grand Index
30	f	0-12 years	0.082	703.582	79	Grand Index
30	f	0-12 years	0.083	703.833	79	Grand Index
30	f	0-12 years	0.084	704.6692346	79	Grand Index
30	f	0-12 years	0.085	706.6689712	79	Grand Index
30	f	0-12 years	0.086	708.6687078	80	Grand Index

Age	Gender	Education	Percentile	Raw Score	Norm Score	Assessment
30	f	0-12 years	0.087	710.6684444	80	Grand Index
30	f	0-12 years	0.088	712.0579753	80	Grand Index
30	f	0-12 years	0.089	712.3172387	80	Grand Index
30	f	0-12 years	0.09	712.5765021	80	Grand Index
30	f	0-12 years	0.091	712.8357654	80	Grand Index
30	f	0-12 years	0.095	713	80	Grand Index
30	f	0-12 years	0.096	713.096	80	Grand Index
30	f	0-12 years	0.097	713.347	81	Grand Index
30	f	0-12 years	0.098	713.598	81	Grand Index
30	f	0-12 years	0.099	713.849	81	Grand Index
30	f	0-12 years	0.1	714.3	81	Grand Index
30	f	0-12 years	0.101	715.053	81	Grand Index
30	f	0-12 years	0.102	715.806	81	Grand Index
30	f	0-12 years	0.103	716.559	81	Grand Index
30	f	0-12 years	0.107	717	81	Grand Index
30	f	0-12 years	0.108	717.216	81	Grand Index
30	f	0-12 years	0.109	717.718	82	Grand Index
30	f	0-12 years	0.11	718.22	82	Grand Index
30	f	0-12 years	0.111	718.722	82	Grand Index
30	f	0-12 years	0.112	719.224	82	Grand Index
30	f	0-12 years	0.113	719.726	82	Grand Index
30	f	0-12 years	0.114	720.228	82	Grand Index
30	f	0-12 years	0.115	720.73	82	Grand Index
30	f	0-12 years	0.116	721.2315135	82	Grand Index
30	f	0-12 years	0.117	721.7324609	82	Grand Index
30	f	0-12 years	0.118	722.2334083	82	Grand Index
30	f	0-12 years	0.119	722.7343557	82	Grand Index
30	f	0-12 years	0.12	723.1163095	82	Grand Index
30	f	0-12 years	0.121	723.3683622	82	Grand Index
30	f	0-12 years	0.122	723.6204148	83	Grand Index
30	f	0-12 years	0.123	723.8724674	83	Grand Index
30	f	0-12 years	0.124	724.372	83	Grand Index
30	f	0-12 years	0.125	725.125	83	Grand Index
30	f	0-12 years	0.126	725.878	83	Grand Index
30	f	0-12 years	0.127	726.631	83	Grand Index
30	f	0-12 years	0.128	727.256	83	Grand Index
30	f	0-12 years	0.129	727.758	83	Grand Index
30	f	0-12 years	0.13	728.26	83	Grand Index
30	f	0-12 years	0.131	728.762	83	Grand Index
30	f	0-12 years	0.132	729.132	83	Grand Index
30	f	0-12 years	0.133	729.383	83	Grand Index
30	f	0-12 years	0.134	729.634	83	Grand Index
30	f	0-12 years	0.135	729.885	83	Grand Index
30	f	0-12 years	0.136	730.272	84	Grand Index
30	f	0-12 years	0.137	730.774	84	Grand Index
30	f	0-12 years	0.138	731.276	84	Grand Index
30	f	0-12 years	0.139	731.778	84	Grand Index
30	f	0-12 years	0.143	732	84	Grand Index
30	f	0-12 years	0.144	732.144	84	Grand Index
30	f	0-12 years	0.145	732.395	84	Grand Index
30	f	0-12 years	0.146	732.646	84	Grand Index
30	f	0-12 years	0.147	732.897	84	Grand Index
30	f	0-12 years	0.148	733.148	84	Grand Index
30	f	0-12 years	0.149	733.399	84	Grand Index
30	f	0-12 years	0.15	733.65	84	Grand Index
30	f	0-12 years	0.151	733.901	85	Grand Index

Age	Gender	Education	Percentile	Raw Score	Norm Score	Assessment
30	f	0-12 years	0.152	734.76	85	Grand Index
30	f	0-12 years	0.153	736.015	85	Grand Index
30	f	0-12 years	0.154	737.27	85	Grand Index
30	f	0-12 years	0.155	738.525	85	Grand Index
30	f	0-12 years	0.159	739	85	Grand Index
30	f	0-12 years	0.16	739.1598477	85	Grand Index
30	f	0-12 years	0.161	739.4106088	85	Grand Index
30	f	0-12 years	0.162	739.6613699	85	Grand Index
30	f	0-12 years	0.163	739.9121309	85	Grand Index
30	f	0-12 years	0.164	740.1632042	85	Grand Index
30	f	0-12 years	0.165	740.4144432	85	Grand Index
30	f	0-12 years	0.166	740.6656821	85	Grand Index
30	f	0-12 years	0.167	740.916921	86	Grand Index
30	f	0-12 years	0.168	741.168	86	Grand Index
30	f	0-12 years	0.169	741.419	86	Grand Index
30	f	0-12 years	0.17	741.67	86	Grand Index
30	f	0-12 years	0.171	741.921	86	Grand Index
30	f	0-12 years	0.175	742	86	Grand Index
30	f	0-12 years	0.176	742.352	86	Grand Index
30	f	0-12 years	0.177	742.854	86	Grand Index
30	f	0-12 years	0.178	743.356	86	Grand Index
30	f	0-12 years	0.179	743.858	86	Grand Index
30	f	0-12 years	0.18	744.427031	86	Grand Index
30	f	0-12 years	0.181	745.022502	86	Grand Index
30	f	0-12 years	0.182	745.617973	86	Grand Index
30	f	0-12 years	0.183	746.213444	86	Grand Index
30	f	0-12 years	0.184	747.4078739	86	Grand Index
30	f	0-12 years	0.185	748.8204029	87	Grand Index
30	f	0-12 years	0.186	750.2329319	87	Grand Index
30	f	0-12 years	0.187	751.6454609	87	Grand Index
30	f	0-12 years	0.188	752.188	87	Grand Index
30	f	0-12 years	0.189	752.439	87	Grand Index
30	f	0-12 years	0.19	752.69	87	Grand Index
30	f	0-12 years	0.191	752.941	87	Grand Index
30	f	0-12 years	0.195	753	87	Grand Index
30	f	0-12 years	0.196	753.196	87	Grand Index
30	f	0-12 years	0.197	753.447	87	Grand Index
30	f	0-12 years	0.198	753.698	87	Grand Index
30	f	0-12 years	0.199	753.949	87	Grand Index
30	f	0-12 years	0.2	754.3715295	87	Grand Index
30	f	0-12 years	0.201	754.837799	87	Grand Index
30	f	0-12 years	0.202	755.3040686	87	Grand Index
30	f	0-12 years	0.203	755.7703381	88	Grand Index
30	f	0-12 years	0.204	755.8866874	88	Grand Index
30	f	0-12 years	0.205	755.9224179	88	Grand Index
30	f	0-12 years	0.206	755.9581484	88	Grand Index
30	f	0-12 years	0.207	755.9938788	88	Grand Index
30	f	0-12 years	0.208	756.208	88	Grand Index
30	f	0-12 years	0.209	756.459	88	Grand Index
30	f	0-12 years	0.21	756.71	88	Grand Index
30	f	0-12 years	0.211	756.961	88	Grand Index
30	f	0-12 years	0.219	757	88	Grand Index
30	f	0-12 years	0.22	757.22	88	Grand Index
30	f	0-12 years	0.221	757.471	88	Grand Index
30	f	0-12 years	0.222	757.722	89	Grand Index
30	f	0-12 years	0.223	757.973	89	Grand Index

Age	Gender	Education	Percentile	Raw Score	Norm Score	Assessment
30	f	0-12 years	0.224	758.224	89	Grand Index
30	f	0-12 years	0.225	758.475	89	Grand Index
30	f	0-12 years	0.226	758.726	89	Grand Index
30	f	0-12 years	0.227	758.977	89	Grand Index
30	f	0-12 years	0.228	759.684	89	Grand Index
30	f	0-12 years	0.229	760.437	89	Grand Index
30	f	0-12 years	0.23	761.19	89	Grand Index
30	f	0-12 years	0.231	761.943	89	Grand Index
30	f	0-12 years	0.235	762	89	Grand Index
30	f	0-12 years	0.236	762.472	89	Grand Index
30	f	0-12 years	0.237	762.974	89	Grand Index
30	f	0-12 years	0.238	763.476	89	Grand Index
30	f	0-12 years	0.239	763.978	89	Grand Index
30	f	0-12 years	0.24	764.48	89	Grand Index
30	f	0-12 years	0.241	764.982	89	Grand Index
30	f	0-12 years	0.242	765.484	90	Grand Index
30	f	0-12 years	0.243	765.986	90	Grand Index
30	f	0-12 years	0.244	766.0777234	90	Grand Index
30	f	0-12 years	0.245	766.1576765	90	Grand Index
30	f	0-12 years	0.246	766.2376297	90	Grand Index
30	f	0-12 years	0.247	766.3175829	90	Grand Index
30	f	0-12 years	0.248	766.9835409	90	Grand Index
30	f	0-12 years	0.249	767.6565878	90	Grand Index
30	f	0-12 years	0.25	768.3296346	90	Grand Index
30	f	0-12 years	0.254	769	90	Grand Index
30	f	0-12 years	0.255	769.005	90	Grand Index
30	f	0-12 years	0.256	769.256	90	Grand Index
30	f	0-12 years	0.257	769.507	90	Grand Index
30	f	0-12 years	0.258	769.758	90	Grand Index
30	f	0-12 years	0.262	770	90	Grand Index
30	f	0-12 years	0.263	770.013	90	Grand Index
30	f	0-12 years	0.264	770.264	91	Grand Index
30	f	0-12 years	0.265	770.515	91	Grand Index
30	f	0-12 years	0.266	770.766	91	Grand Index
30	f	0-12 years	0.27	771	91	Grand Index
30	f	0-12 years	0.271	771.021	91	Grand Index
30	f	0-12 years	0.272	771.272	91	Grand Index
30	f	0-12 years	0.273	771.523	91	Grand Index
30	f	0-12 years	0.274	771.774	91	Grand Index
30	f	0-12 years	0.275	772.0001079	91	Grand Index
30	f	0-12 years	0.276	772.0011914	91	Grand Index
30	f	0-12 years	0.277	772.002275	91	Grand Index
30	f	0-12 years	0.278	772.0033585	91	Grand Index
30	f	0-12 years	0.279	772.0331916	91	Grand Index
30	f	0-12 years	0.28	772.2831081	91	Grand Index
30	f	0-12 years	0.281	772.5330246	91	Grand Index
30	f	0-12 years	0.282	772.7829411	91	Grand Index
30	f	0-12 years	0.294	773	92	Grand Index
30	f	0-12 years	0.295	773.09	92	Grand Index
30	f	0-12 years	0.296	773.592	92	Grand Index
30	f	0-12 years	0.297	774.094	92	Grand Index
30	f	0-12 years	0.298	774.596	92	Grand Index
30	f	0-12 years	0.299	775.049	92	Grand Index
30	f	0-12 years	0.3	775.3	92	Grand Index
30	f	0-12 years	0.301	775.551	92	Grand Index
30	f	0-12 years	0.302	775.802	92	Grand Index

Age	Gender	Education	Percentile	Raw Score	Norm Score	Assessment
30	f	0-12 years	0.303	776.106	92	Grand Index
30	f	0-12 years	0.304	776.608	92	Grand Index
30	f	0-12 years	0.305	777.11	92	Grand Index
30	f	0-12 years	0.306	777.612	92	Grand Index
30	f	0-12 years	0.31	778	93	Grand Index
30	f	0-12 years	0.311	778.061	93	Grand Index
30	f	0-12 years	0.312	778.312	93	Grand Index
30	f	0-12 years	0.313	778.563	93	Grand Index
30	f	0-12 years	0.314	778.814	93	Grand Index
30	f	0-12 years	0.315	779.0398288	93	Grand Index
30	f	0-12 years	0.316	779.1936295	93	Grand Index
30	f	0-12 years	0.317	779.3474301	93	Grand Index
30	f	0-12 years	0.318	779.5012307	93	Grand Index
30	f	0-12 years	0.319	779.6394716	93	Grand Index
30	f	0-12 years	0.32	779.736671	93	Grand Index
30	f	0-12 years	0.321	779.8338704	93	Grand Index
30	f	0-12 years	0.322	779.9310698	93	Grand Index
30	f	0-12 years	0.323	780.146	93	Grand Index
30	f	0-12 years	0.324	780.648	93	Grand Index
30	f	0-12 years	0.325	781.15	93	Grand Index
30	f	0-12 years	0.326	781.652	93	Grand Index
30	f	0-12 years	0.33	782	93	Grand Index
30	f	0-12 years	0.331	782.243	93	Grand Index
30	f	0-12 years	0.332	782.996	93	Grand Index
30	f	0-12 years	0.333	783.749	94	Grand Index
30	f	0-12 years	0.334	784.502	94	Grand Index
30	f	0-12 years	0.335	785.085	94	Grand Index
30	f	0-12 years	0.336	785.336	94	Grand Index
30	f	0-12 years	0.337	785.587	94	Grand Index
30	f	0-12 years	0.338	785.838	94	Grand Index
30	f	0-12 years	0.342	786	94	Grand Index
30	f	0-12 years	0.343	786.186	94	Grand Index
30	f	0-12 years	0.344	786.688	94	Grand Index
30	f	0-12 years	0.345	787.19	94	Grand Index
30	f	0-12 years	0.346	787.692	94	Grand Index
30	f	0-12 years	0.354	788	94	Grand Index
30	f	0-12 years	0.355	788.105	94	Grand Index
30	f	0-12 years	0.356	788.356	94	Grand Index
30	f	0-12 years	0.357	788.607	95	Grand Index
30	f	0-12 years	0.358	788.858	95	Grand Index
30	f	0-12 years	0.359	789.109	95	Grand Index
30	f	0-12 years	0.36	789.36	95	Grand Index
30	f	0-12 years	0.361	789.611	95	Grand Index
30	f	0-12 years	0.362	789.862	95	Grand Index
30	f	0-12 years	0.363	790.113	95	Grand Index
30	f	0-12 years	0.364	790.364	95	Grand Index
30	f	0-12 years	0.365	790.615	95	Grand Index
30	f	0-12 years	0.366	790.866	95	Grand Index
30	f	0-12 years	0.37	791	95	Grand Index
30	f	0-12 years	0.371	791.4198143	95	Grand Index
30	f	0-12 years	0.372	792.2906686	95	Grand Index
30	f	0-12 years	0.373	793.161523	95	Grand Index
30	f	0-12 years	0.374	794.0323774	95	Grand Index
30	f	0-12 years	0.375	794.535847	95	Grand Index
30	f	0-12 years	0.376	794.6689926	95	Grand Index
30	f	0-12 years	0.377	794.8021382	95	Grand Index

Age	Gender	Education	Percentile	Raw Score	Norm Score	Assessment
30	f	0-12 years	0.378	794.9352838	95	Grand Index
30	f	0-12 years	0.386	795	96	Grand Index
30	f	0-12 years	0.387	795.137	96	Grand Index
30	f	0-12 years	0.388	795.388	96	Grand Index
30	f	0-12 years	0.389	795.639	96	Grand Index
30	f	0-12 years	0.39	795.89	96	Grand Index
30	f	0-12 years	0.391	796.141	96	Grand Index
30	f	0-12 years	0.392	796.392	96	Grand Index
30	f	0-12 years	0.393	796.643	96	Grand Index
30	f	0-12 years	0.394	796.894	96	Grand Index
30	f	0-12 years	0.398	797	96	Grand Index
30	f	0-12 years	0.399	797.149	96	Grand Index
30	f	0-12 years	0.4	797.4	96	Grand Index
30	f	0-12 years	0.401	797.651	96	Grand Index
30	f	0-12 years	0.402	797.902	96	Grand Index
30	f	0-12 years	0.403	798.1524201	96	Grand Index
30	f	0-12 years	0.404	798.4024688	96	Grand Index
30	f	0-12 years	0.405	798.6525175	96	Grand Index
30	f	0-12 years	0.406	798.9025662	96	Grand Index
30	f	0-12 years	0.407	798.9968049	96	Grand Index
30	f	0-12 years	0.408	798.9977562	97	Grand Index
30	f	0-12 years	0.409	798.9987076	97	Grand Index
30	f	0-12 years	0.41	798.9996589	97	Grand Index
30	f	0-12 years	0.411	799.161	97	Grand Index
30	f	0-12 years	0.412	799.412	97	Grand Index
30	f	0-12 years	0.413	799.663	97	Grand Index
30	f	0-12 years	0.414	799.914	97	Grand Index
30	f	0-12 years	0.422	800	97	Grand Index
30	f	0-12 years	0.423	800.346	97	Grand Index
30	f	0-12 years	0.424	800.848	97	Grand Index
30	f	0-12 years	0.425	801.35	97	Grand Index
30	f	0-12 years	0.426	801.852	97	Grand Index
30	f	0-12 years	0.43	802	97	Grand Index
30	f	0-12 years	0.431	802.181	97	Grand Index
30	f	0-12 years	0.432	802.432	97	Grand Index
30	f	0-12 years	0.433	802.683	97	Grand Index
30	f	0-12 years	0.434	802.934	98	Grand Index
30	f	0-12 years	0.435	803.1415203	98	Grand Index
30	f	0-12 years	0.436	803.3335289	98	Grand Index
30	f	0-12 years	0.437	803.5255375	98	Grand Index
30	f	0-12 years	0.438	803.7175461	98	Grand Index
30	f	0-12 years	0.439	803.7982397	98	Grand Index
30	f	0-12 years	0.44	803.8424173	98	Grand Index
30	f	0-12 years	0.441	803.8865949	98	Grand Index
30	f	0-12 years	0.442	803.9307725	98	Grand Index
30	f	0-12 years	0.443	803.9523715	98	Grand Index
30	f	0-12 years	0.444	803.9671853	98	Grand Index
30	f	0-12 years	0.445	803.9819992	98	Grand Index
30	f	0-12 years	0.446	803.996813	98	Grand Index
30	f	0-12 years	0.447	804.197	98	Grand Index
30	f	0-12 years	0.448	804.448	98	Grand Index
30	f	0-12 years	0.449	804.699	98	Grand Index
30	f	0-12 years	0.45	804.95	98	Grand Index
30	f	0-12 years	0.458	805	98	Grand Index
30	f	0-12 years	0.459	805.209	98	Grand Index
30	f	0-12 years	0.46	805.46	98	Grand Index

Age	Gender	Education	Percentile	Raw Score	Norm Score	Assessment
30	f	0-12 years	0.461	805.711	99	Grand Index
30	f	0-12 years	0.462	805.962	99	Grand Index
30	f	0-12 years	0.466	806	99	Grand Index
30	f	0-12 years	0.467	806.217	99	Grand Index
30	f	0-12 years	0.468	806.468	99	Grand Index
30	f	0-12 years	0.469	806.719	99	Grand Index
30	f	0-12 years	0.47	806.97	99	Grand Index
30	f	0-12 years	0.471	807.221	99	Grand Index
30	f	0-12 years	0.472	807.472	99	Grand Index
30	f	0-12 years	0.473	807.723	99	Grand Index
30	f	0-12 years	0.474	807.974	99	Grand Index
30	f	0-12 years	0.475	808.225	99	Grand Index
30	f	0-12 years	0.476	808.476	99	Grand Index
30	f	0-12 years	0.477	808.727	99	Grand Index
30	f	0-12 years	0.478	808.978	99	Grand Index
30	f	0-12 years	0.482	809	99	Grand Index
30	f	0-12 years	0.483	809.233	99	Grand Index
30	f	0-12 years	0.484	809.484	99	Grand Index
30	f	0-12 years	0.485	809.735	99	Grand Index
30	f	0-12 years	0.486	809.986	99	Grand Index
30	f	0-12 years	0.494	810	100	Grand Index
30	f	0-12 years	0.495	810.4896858	100	Grand Index
30	f	0-12 years	0.496	810.9913639	100	Grand Index
30	f	0-12 years	0.497	811.493042	100	Grand Index
30	f	0-12 years	0.498	811.99472	100	Grand Index
30	f	0-12 years	0.499	812.2480368	100	Grand Index
30	f	0-12 years	0.5	812.4993587	100	Grand Index
30	f	0-12 years	0.501	812.7506807	100	Grand Index
30	f	0-12 years	0.505	813	100	Grand Index
30	f	0-12 years	0.506	813.012	100	Grand Index
30	f	0-12 years	0.507	813.514	100	Grand Index
30	f	0-12 years	0.508	814.016	100	Grand Index
30	f	0-12 years	0.509	814.518	100	Grand Index
30	f	0-12 years	0.513	815	100	Grand Index
30	f	0-12 years	0.514	815.014	101	Grand Index
30	f	0-12 years	0.515	815.265	101	Grand Index
30	f	0-12 years	0.516	815.516	101	Grand Index
30	f	0-12 years	0.517	815.767	101	Grand Index
30	f	0-12 years	0.518	816.018	101	Grand Index
30	f	0-12 years	0.519	816.269	101	Grand Index
30	f	0-12 years	0.52	816.52	101	Grand Index
30	f	0-12 years	0.521	816.771	101	Grand Index
30	f	0-12 years	0.525	817	101	Grand Index
30	f	0-12 years	0.526	817.026	101	Grand Index
30	f	0-12 years	0.527	817.277	101	Grand Index
30	f	0-12 years	0.528	817.528	101	Grand Index
30	f	0-12 years	0.529	817.779	101	Grand Index
30	f	0-12 years	0.53	818.03	101	Grand Index
30	f	0-12 years	0.531	818.281	101	Grand Index
30	f	0-12 years	0.532	818.532	101	Grand Index
30	f	0-12 years	0.533	818.783	101	Grand Index
30	f	0-12 years	0.541	819	102	Grand Index
30	f	0-12 years	0.542	819.084	102	Grand Index
30	f	0-12 years	0.543	819.586	102	Grand Index
30	f	0-12 years	0.544	820.088	102	Grand Index
30	f	0-12 years	0.545	820.59	102	Grand Index

Age	Gender	Education	Percentile	Raw Score	Norm Score	Assessment
30	f	0-12 years	0.546	821.0139257	102	Grand Index
30	f	0-12 years	0.547	821.0899117	102	Grand Index
30	f	0-12 years	0.548	821.1658976	102	Grand Index
30	f	0-12 years	0.549	821.2418835	102	Grand Index
30	f	0-12 years	0.55	821.3375962	102	Grand Index
30	f	0-12 years	0.551	821.5126103	102	Grand Index
30	f	0-12 years	0.552	821.6876243	102	Grand Index
30	f	0-12 years	0.553	821.8626384	102	Grand Index
30	f	0-12 years	0.554	822.054	102	Grand Index
30	f	0-12 years	0.555	822.305	102	Grand Index
30	f	0-12 years	0.556	822.556	102	Grand Index
30	f	0-12 years	0.557	822.807	102	Grand Index
30	f	0-12 years	0.565	823	102	Grand Index
30	f	0-12 years	0.566	823.0153732	102	Grand Index
30	f	0-12 years	0.567	823.0738377	103	Grand Index
30	f	0-12 years	0.568	823.1323023	103	Grand Index
30	f	0-12 years	0.569	823.1907669	103	Grand Index
30	f	0-12 years	0.57	823.2866217	103	Grand Index
30	f	0-12 years	0.571	823.4791572	103	Grand Index
30	f	0-12 years	0.572	823.6716926	103	Grand Index
30	f	0-12 years	0.573	823.864228	103	Grand Index
30	f	0-12 years	0.574	824.148	103	Grand Index
30	f	0-12 years	0.575	824.65	103	Grand Index
30	f	0-12 years	0.576	825.152	103	Grand Index
30	f	0-12 years	0.577	825.654	103	Grand Index
30	f	0-12 years	0.578	826.1745981	103	Grand Index
30	f	0-12 years	0.579	826.7364457	103	Grand Index
30	f	0-12 years	0.58	827.2982933	103	Grand Index
30	f	0-12 years	0.581	827.8601409	103	Grand Index
30	f	0-12 years	0.582	828.3008849	103	Grand Index
30	f	0-12 years	0.583	828.4920373	103	Grand Index
30	f	0-12 years	0.584	828.6831897	103	Grand Index
30	f	0-12 years	0.585	828.8743421	103	Grand Index
30	f	0-12 years	0.586	829.086	103	Grand Index
30	f	0-12 years	0.587	829.337	103	Grand Index
30	f	0-12 years	0.588	829.588	103	Grand Index
30	f	0-12 years	0.589	829.839	103	Grand Index
30	f	0-12 years	0.593	830	104	Grand Index
30	f	0-12 years	0.594	830.188	104	Grand Index
30	f	0-12 years	0.595	830.69	104	Grand Index
30	f	0-12 years	0.596	831.192	104	Grand Index
30	f	0-12 years	0.597	831.694	104	Grand Index
30	f	0-12 years	0.601	832	104	Grand Index
30	f	0-12 years	0.602	832.102	104	Grand Index
30	f	0-12 years	0.603	832.353	104	Grand Index
30	f	0-12 years	0.604	832.604	104	Grand Index
30	f	0-12 years	0.605	832.855	104	Grand Index
30	f	0-12 years	0.606	833.0174383	104	Grand Index
30	f	0-12 years	0.607	833.0587309	104	Grand Index
30	f	0-12 years	0.608	833.1000235	104	Grand Index
30	f	0-12 years	0.609	833.1413161	104	Grand Index
30	f	0-12 years	0.61	833.256416	104	Grand Index
30	f	0-12 years	0.611	833.4661234	104	Grand Index
30	f	0-12 years	0.612	833.6758308	104	Grand Index
30	f	0-12 years	0.613	833.8855382	104	Grand Index
30	f	0-12 years	0.629	834	105	Grand Index

Age	Gender	Education	Percentile	Raw Score	Norm Score	Assessment
30	f	0-12 years	0.63	834.13	105	Grand Index
30	f	0-12 years	0.631	834.381	105	Grand Index
30	f	0-12 years	0.632	834.632	105	Grand Index
30	f	0-12 years	0.633	834.883	105	Grand Index
30	f	0-12 years	0.637	835	105	Grand Index
30	f	0-12 years	0.638	835.276	105	Grand Index
30	f	0-12 years	0.639	835.778	105	Grand Index
30	f	0-12 years	0.64	836.28	105	Grand Index
30	f	0-12 years	0.641	836.782	105	Grand Index
30	f	0-12 years	0.649	837	106	Grand Index
30	f	0-12 years	0.65	837.0034112	106	Grand Index
30	f	0-12 years	0.651	837.0091193	106	Grand Index
30	f	0-12 years	0.652	837.0148274	106	Grand Index
30	f	0-12 years	0.653	837.0205355	106	Grand Index
30	f	0-12 years	0.654	837.1732393	106	Grand Index
30	f	0-12 years	0.655	837.4185312	106	Grand Index
30	f	0-12 years	0.656	837.6638231	106	Grand Index
30	f	0-12 years	0.657	837.909115	106	Grand Index
30	f	0-12 years	0.658	838.158	106	Grand Index
30	f	0-12 years	0.659	838.409	106	Grand Index
30	f	0-12 years	0.66	838.66	106	Grand Index
30	f	0-12 years	0.661	838.911	106	Grand Index
30	f	0-12 years	0.662	839.162	106	Grand Index
30	f	0-12 years	0.663	839.413	106	Grand Index
30	f	0-12 years	0.664	839.664	106	Grand Index
30	f	0-12 years	0.665	839.915	106	Grand Index
30	f	0-12 years	0.666	840.332	106	Grand Index
30	f	0-12 years	0.667	840.834	106	Grand Index
30	f	0-12 years	0.668	841.336	107	Grand Index
30	f	0-12 years	0.669	841.838	107	Grand Index
30	f	0-12 years	0.67	842.17	107	Grand Index
30	f	0-12 years	0.671	842.421	107	Grand Index
30	f	0-12 years	0.672	842.672	107	Grand Index
30	f	0-12 years	0.673	842.923	107	Grand Index
30	f	0-12 years	0.674	843.174	107	Grand Index
30	f	0-12 years	0.675	843.425	107	Grand Index
30	f	0-12 years	0.676	843.676	107	Grand Index
30	f	0-12 years	0.677	843.927	107	Grand Index
30	f	0-12 years	0.678	844.178	107	Grand Index
30	f	0-12 years	0.679	844.429	107	Grand Index
30	f	0-12 years	0.68	844.68	107	Grand Index
30	f	0-12 years	0.681	844.931	107	Grand Index
30	f	0-12 years	0.682	845.0977089	107	Grand Index
30	f	0-12 years	0.683	845.2324613	107	Grand Index
30	f	0-12 years	0.684	845.3672136	107	Grand Index
30	f	0-12 years	0.685	845.501966	107	Grand Index
30	f	0-12 years	0.686	845.8964271	107	Grand Index
30	f	0-12 years	0.687	846.3816466	107	Grand Index
30	f	0-12 years	0.688	846.8668661	107	Grand Index
30	f	0-12 years	0.689	847.3520856	107	Grand Index
30	f	0-12 years	0.69	847.5707061	107	Grand Index
30	f	0-12 years	0.691	847.7037342	107	Grand Index
30	f	0-12 years	0.692	847.8367623	108	Grand Index
30	f	0-12 years	0.693	847.9697904	108	Grand Index
30	f	0-12 years	0.697	848	108	Grand Index
30	f	0-12 years	0.698	848.198	108	Grand Index

Age	Gender	Education	Percentile	Raw Score	Norm Score	Assessment
30	f	0-12 years	0.699	848.449	108	Grand Index
30	f	0-12 years	0.7	848.7	108	Grand Index
30	f	0-12 years	0.701	848.951	108	Grand Index
30	f	0-12 years	0.702	849.404	108	Grand Index
30	f	0-12 years	0.703	849.906	108	Grand Index
30	f	0-12 years	0.704	850.408	108	Grand Index
30	f	0-12 years	0.705	850.91	108	Grand Index
30	f	0-12 years	0.706	851.618	108	Grand Index
30	f	0-12 years	0.707	852.371	108	Grand Index
30	f	0-12 years	0.708	853.124	108	Grand Index
30	f	0-12 years	0.709	853.877	108	Grand Index
30	f	0-12 years	0.713	854	108	Grand Index
30	f	0-12 years	0.714	854.642	108	Grand Index
30	f	0-12 years	0.715	855.395	109	Grand Index
30	f	0-12 years	0.716	856.148	109	Grand Index
30	f	0-12 years	0.717	856.901	109	Grand Index
30	f	0-12 years	0.725	857	109	Grand Index
30	f	0-12 years	0.726	857.226	109	Grand Index
30	f	0-12 years	0.727	857.477	109	Grand Index
30	f	0-12 years	0.728	857.728	109	Grand Index
30	f	0-12 years	0.729	857.979	109	Grand Index
30	f	0-12 years	0.73	858.23	109	Grand Index
30	f	0-12 years	0.731	858.481	109	Grand Index
30	f	0-12 years	0.732	858.732	109	Grand Index
30	f	0-12 years	0.733	858.983	109	Grand Index
30	f	0-12 years	0.737	859	110	Grand Index
30	f	0-12 years	0.738	859.238	110	Grand Index
30	f	0-12 years	0.739	859.489	110	Grand Index
30	f	0-12 years	0.74	859.74	110	Grand Index
30	f	0-12 years	0.741	859.991	110	Grand Index
30	f	0-12 years	0.749	860	110	Grand Index
30	f	0-12 years	0.75	860.25	110	Grand Index
30	f	0-12 years	0.751	860.501	110	Grand Index
30	f	0-12 years	0.752	860.752	110	Grand Index
30	f	0-12 years	0.753	861.0019716	110	Grand Index
30	f	0-12 years	0.754	861.166932	110	Grand Index
30	f	0-12 years	0.755	861.3318923	110	Grand Index
30	f	0-12 years	0.756	861.4968527	110	Grand Index
30	f	0-12 years	0.757	861.659612	110	Grand Index
30	f	0-12 years	0.758	861.7456517	110	Grand Index
30	f	0-12 years	0.759	861.8316914	111	Grand Index
30	f	0-12 years	0.76	861.917731	111	Grand Index
30	f	0-12 years	0.761	862.033	111	Grand Index
30	f	0-12 years	0.762	862.786	111	Grand Index
30	f	0-12 years	0.763	863.539	111	Grand Index
30	f	0-12 years	0.764	864.292	111	Grand Index
30	f	0-12 years	0.765	865.03	111	Grand Index
30	f	0-12 years	0.766	865.532	111	Grand Index
30	f	0-12 years	0.767	866.034	111	Grand Index
30	f	0-12 years	0.768	866.536	111	Grand Index
30	f	0-12 years	0.772	867	111	Grand Index
30	f	0-12 years	0.773	867.023	111	Grand Index
30	f	0-12 years	0.774	867.274	111	Grand Index
30	f	0-12 years	0.775	867.525	111	Grand Index
30	f	0-12 years	0.776	867.776	111	Grand Index
30	f	0-12 years	0.777	868.027	111	Grand Index

Age	Gender	Education	Percentile	Raw Score	Norm Score	Assessment
30	f	0-12 years	0.778	868.278	111	Grand Index
30	f	0-12 years	0.779	868.529	112	Grand Index
30	f	0-12 years	0.78	868.78	112	Grand Index
30	f	0-12 years	0.788	869	112	Grand Index
30	f	0-12 years	0.789	869.078	112	Grand Index
30	f	0-12 years	0.79	869.58	112	Grand Index
30	f	0-12 years	0.791	870.082	112	Grand Index
30	f	0-12 years	0.792	870.584	112	Grand Index
30	f	0-12 years	0.796	871	112	Grand Index
30	f	0-12 years	0.797	871.047	112	Grand Index
30	f	0-12 years	0.798	871.298	113	Grand Index
30	f	0-12 years	0.799	871.549	113	Grand Index
30	f	0-12 years	0.8	871.8	113	Grand Index
30	f	0-12 years	0.801	872.051	113	Grand Index
30	f	0-12 years	0.802	872.302	113	Grand Index
30	f	0-12 years	0.803	872.553	113	Grand Index
30	f	0-12 years	0.804	872.804	113	Grand Index
30	f	0-12 years	0.808	873	113	Grand Index
30	f	0-12 years	0.809	873.059	113	Grand Index
30	f	0-12 years	0.81	873.31	113	Grand Index
30	f	0-12 years	0.811	873.561	113	Grand Index
30	f	0-12 years	0.812	873.812	113	Grand Index
30	f	0-12 years	0.816	874	114	Grand Index
30	f	0-12 years	0.817	874.067	114	Grand Index
30	f	0-12 years	0.818	874.318	114	Grand Index
30	f	0-12 years	0.819	874.569	114	Grand Index
30	f	0-12 years	0.82	874.82	114	Grand Index
30	f	0-12 years	0.821	875.142	114	Grand Index
30	f	0-12 years	0.822	875.644	114	Grand Index
30	f	0-12 years	0.823	876.146	114	Grand Index
30	f	0-12 years	0.824	876.648	114	Grand Index
30	f	0-12 years	0.825	877.15	114	Grand Index
30	f	0-12 years	0.826	877.652	114	Grand Index
30	f	0-12 years	0.827	878.154	114	Grand Index
30	f	0-12 years	0.828	878.656	114	Grand Index
30	f	0-12 years	0.832	879	114	Grand Index
30	f	0-12 years	0.833	879.083	114	Grand Index
30	f	0-12 years	0.834	879.334	115	Grand Index
30	f	0-12 years	0.835	879.585	115	Grand Index
30	f	0-12 years	0.836	879.836	115	Grand Index
30	f	0-12 years	0.837	880.174	115	Grand Index
30	f	0-12 years	0.838	880.676	115	Grand Index
30	f	0-12 years	0.839	881.178	115	Grand Index
30	f	0-12 years	0.84	881.68	115	Grand Index
30	f	0-12 years	0.841	882.1193258	115	Grand Index
30	f	0-12 years	0.842	882.4484553	115	Grand Index
30	f	0-12 years	0.843	882.7775848	115	Grand Index
30	f	0-12 years	0.844	883.1067142	115	Grand Index
30	f	0-12 years	0.845	883.4732158	115	Grand Index
30	f	0-12 years	0.846	883.9010863	115	Grand Index
30	f	0-12 years	0.847	884.3289569	115	Grand Index
30	f	0-12 years	0.848	884.7568274	115	Grand Index
30	f	0-12 years	0.849	885.1430825	115	Grand Index
30	f	0-12 years	0.85	885.4654431	116	Grand Index
30	f	0-12 years	0.851	885.7878037	116	Grand Index
30	f	0-12 years	0.852	886.1101643	116	Grand Index

Age	Gender	Education	Percentile	Raw Score	Norm Score	Assessment
30	f	0-12 years	0.853	886.4753166	116	Grand Index
30	f	0-12 years	0.854	886.901956	116	Grand Index
30	f	0-12 years	0.855	887.3285954	116	Grand Index
30	f	0-12 years	0.856	887.7552348	116	Grand Index
30	f	0-12 years	0.857	888.214	116	Grand Index
30	f	0-12 years	0.858	888.716	116	Grand Index
30	f	0-12 years	0.859	889.218	116	Grand Index
30	f	0-12 years	0.86	889.72	116	Grand Index
30	f	0-12 years	0.864	890	116	Grand Index
30	f	0-12 years	0.865	890.2353956	117	Grand Index
30	f	0-12 years	0.866	890.7491722	117	Grand Index
30	f	0-12 years	0.867	891.2629487	117	Grand Index
30	f	0-12 years	0.868	891.7767252	117	Grand Index
30	f	0-12 years	0.869	892.1603352	117	Grand Index
30	f	0-12 years	0.87	892.3995586	117	Grand Index
30	f	0-12 years	0.871	892.6387821	117	Grand Index
30	f	0-12 years	0.872	892.8780056	117	Grand Index
30	f	0-12 years	0.873	893.123	117	Grand Index
30	f	0-12 years	0.874	893.374	117	Grand Index
30	f	0-12 years	0.875	893.625	117	Grand Index
30	f	0-12 years	0.876	893.876	117	Grand Index
30	f	0-12 years	0.877	895.016	117	Grand Index
30	f	0-12 years	0.878	897.024	117	Grand Index
30	f	0-12 years	0.879	899.032	118	Grand Index
30	f	0-12 years	0.88	901.04	118	Grand Index
30	f	0-12 years	0.881	902.131	118	Grand Index
30	f	0-12 years	0.882	902.382	118	Grand Index
30	f	0-12 years	0.883	902.633	118	Grand Index
30	f	0-12 years	0.884	902.884	118	Grand Index
30	f	0-12 years	0.885	903.675	118	Grand Index
30	f	0-12 years	0.886	904.93	118	Grand Index
30	f	0-12 years	0.887	906.185	118	Grand Index
30	f	0-12 years	0.888	907.44	118	Grand Index
30	f	0-12 years	0.889	908.278	118	Grand Index
30	f	0-12 years	0.89	908.78	118	Grand Index
30	f	0-12 years	0.891	909.282	118	Grand Index
30	f	0-12 years	0.892	909.784	119	Grand Index
30	f	0-12 years	0.893	910.143	119	Grand Index
30	f	0-12 years	0.894	910.394	119	Grand Index
30	f	0-12 years	0.895	910.645	119	Grand Index
30	f	0-12 years	0.896	910.896	119	Grand Index
30	f	0-12 years	0.897	911.7373426	119	Grand Index
30	f	0-12 years	0.898	912.9963426	119	Grand Index
30	f	0-12 years	0.899	914.2553426	119	Grand Index
30	f	0-12 years	0.9	915.5143426	119	Grand Index
30	f	0-12 years	0.901	916.3155299	119	Grand Index
30	f	0-12 years	0.902	916.8135299	119	Grand Index
30	f	0-12 years	0.903	917.3115299	119	Grand Index
30	f	0-12 years	0.904	917.8095299	120	Grand Index
30	f	0-12 years	0.908	918	120	Grand Index
30	f	0-12 years	0.909	918.477	120	Grand Index
30	f	0-12 years	0.91	919.23	120	Grand Index
30	f	0-12 years	0.911	919.983	120	Grand Index
30	f	0-12 years	0.912	920.736	120	Grand Index
30	f	0-12 years	0.913	921.163	120	Grand Index
30	f	0-12 years	0.914	921.414	120	Grand Index

Age	Gender	Education	Percentile	Raw Score	Norm Score	Assessment
30	f	0-12 years	0.915	921.665	121	Grand Index
30	f	0-12 years	0.916	921.916	121	Grand Index
30	f	0-12 years	0.92	922	121	Grand Index
30	f	0-12 years	0.921	922.342	121	Grand Index
30	f	0-12 years	0.922	922.844	121	Grand Index
30	f	0-12 years	0.923	923.346	121	Grand Index
30	f	0-12 years	0.924	923.848	121	Grand Index
30	f	0-12 years	0.925	924.175	122	Grand Index
30	f	0-12 years	0.926	924.426	122	Grand Index
30	f	0-12 years	0.927	924.677	122	Grand Index
30	f	0-12 years	0.928	924.928	122	Grand Index
30	f	0-12 years	0.929	925.3803941	122	Grand Index
30	f	0-12 years	0.93	925.9137959	122	Grand Index
30	f	0-12 years	0.931	926.4471977	122	Grand Index
30	f	0-12 years	0.932	926.9805994	122	Grand Index
30	f	0-12 years	0.933	927.8342122	122	Grand Index
30	f	0-12 years	0.934	928.8068104	123	Grand Index
30	f	0-12 years	0.935	929.7794086	123	Grand Index
30	f	0-12 years	0.936	930.7520068	123	Grand Index
30	f	0-12 years	0.937	933.7499006	123	Grand Index
30	f	0-12 years	0.938	937.4409437	123	Grand Index
30	f	0-12 years	0.939	941.1319868	123	Grand Index
30	f	0-12 years	0.94	944.8230299	123	Grand Index
30	f	0-12 years	0.941	945.7616289	123	Grand Index
30	f	0-12 years	0.942	945.8355858	124	Grand Index
30	f	0-12 years	0.943	945.9095427	124	Grand Index
30	f	0-12 years	0.944	945.9834997	124	Grand Index
30	f	0-12 years	0.945	946.39	124	Grand Index
30	f	0-12 years	0.946	946.892	124	Grand Index
30	f	0-12 years	0.947	947.394	124	Grand Index
30	f	0-12 years	0.948	947.896	124	Grand Index
30	f	0-12 years	0.949	948.2012093	125	Grand Index
30	f	0-12 years	0.95	948.454996	125	Grand Index
30	f	0-12 years	0.951	948.7087826	125	Grand Index
30	f	0-12 years	0.952	948.9625693	125	Grand Index
30	f	0-12 years	0.953	949.8208484	125	Grand Index
30	f	0-12 years	0.954	950.8220618	125	Grand Index
30	f	0-12 years	0.955	951.8232751	125	Grand Index
30	f	0-12 years	0.956	952.8244885	126	Grand Index
30	f	0-12 years	0.957	953.414	126	Grand Index
30	f	0-12 years	0.958	953.916	126	Grand Index
30	f	0-12 years	0.959	954.418	126	Grand Index
30	f	0-12 years	0.96	954.92	126	Grand Index
30	f	0-12 years	0.961	956.266	126	Grand Index
30	f	0-12 years	0.962	957.772	127	Grand Index
30	f	0-12 years	0.963	959.278	127	Grand Index
30	f	0-12 years	0.964	960.784	127	Grand Index
30	f	0-12 years	0.965	961.2329543	127	Grand Index
30	f	0-12 years	0.966	961.5049149	127	Grand Index
30	f	0-12 years	0.967	961.7768755	128	Grand Index
30	f	0-12 years	0.968	962.0488362	128	Grand Index
30	f	0-12 years	0.969	965.4229938	128	Grand Index
30	f	0-12 years	0.97	969.2504404	128	Grand Index
30	f	0-12 years	0.971	973.0778871	128	Grand Index
30	f	0-12 years	0.972	976.9053337	129	Grand Index
30	f	0-12 years	0.973	977.481197	129	Grand Index

Age	Gender	Education	Percentile	Raw Score	Norm Score	Assessment
30	f	0-12 years	0.974	977.6487897	129	Grand Index
30	f	0-12 years	0.975	977.8163825	129	Grand Index
30	f	0-12 years	0.976	977.9839752	130	Grand Index
30	f	0-12 years	0.977	979.362	130	Grand Index
30	f	0-12 years	0.978	980.868	130	Grand Index
30	f	0-12 years	0.979	982.374	131	Grand Index
30	f	0-12 years	0.98	983.88	131	Grand Index
30	f	0-12 years	0.981	987.696	131	Grand Index
30	f	0-12 years	0.982	991.712	131	Grand Index
30	f	0-12 years	0.983	995.728	132	Grand Index
30	f	0-12 years	0.984	999.744	132	Grand Index
30	f	0-12 years	0.985	1001.175	133	Grand Index
30	f	0-12 years	0.986	1002.43	133	Grand Index
30	f	0-12 years	0.987	1003.685	133	Grand Index
30	f	0-12 years	0.988	1004.94	134	Grand Index
30	f	0-12 years	0.989	1005.956	134	Grand Index
30	f	0-12 years	0.99	1006.96	135	Grand Index
30	f	0-12 years	0.991	1007.964	135	Grand Index
30	f	0-12 years	0.992	1008.968	136	Grand Index
30	f	0-12 years	0.993	1010.708624	137	Grand Index
30	f	0-12 years	0.994	1012.473498	138	Grand Index
30	f	0-12 years	0.995	1014.238373	139	Grand Index
30	f	0-12 years	0.996	1016.003247	140	Grand Index
30	f	0-12 years	0.997	1029.856165	141	Grand Index
30	f	0-12 years	0.998	1043.90484	143	Grand Index
30	f	0-12 years	0.999	1057.953516	146	Grand Index
30	m	0-12 years	0.001	566.4896569	54	Grand Index
30	m	0-12 years	0.002	579.4015422	57	Grand Index
30	m	0-12 years	0.003	606.2986079	59	Grand Index
30	m	0-12 years	0.004	621.736	60	Grand Index
30	m	0-12 years	0.005	626.92	61	Grand Index
30	m	0-12 years	0.006	628.8397395	62	Grand Index
30	m	0-12 years	0.007	630.134605	63	Grand Index
30	m	0-12 years	0.008	635.4098813	64	Grand Index
30	m	0-12 years	0.009	639.6476904	65	Grand Index
30	m	0-12 years	0.01	641.6752431	65	Grand Index
30	m	0-12 years	0.011	642.6813176	66	Grand Index
30	m	0-12 years	0.012	642.957765	66	Grand Index
30	m	0-12 years	0.013	645.44	67	Grand Index
30	m	0-12 years	0.014	648.256	67	Grand Index
30	m	0-12 years	0.015	650.56	67	Grand Index
30	m	0-12 years	0.016	652.8572532	68	Grand Index
30	m	0-12 years	0.017	655.1432619	68	Grand Index
30	m	0-12 years	0.018	656.5022817	69	Grand Index
30	m	0-12 years	0.019	657.3373513	69	Grand Index
30	m	0-12 years	0.02	658.7608986	69	Grand Index
30	m	0-12 years	0.021	660.288	69	Grand Index
30	m	0-12 years	0.022	662.016	70	Grand Index
30	m	0-12 years	0.023	663.248	70	Grand Index
30	m	0-12 years	0.024	663.824	70	Grand Index
30	m	0-12 years	0.025	664.8	71	Grand Index
30	m	0-12 years	0.026	665.952	71	Grand Index
30	m	0-12 years	0.027	668.0397061	71	Grand Index
30	m	0-12 years	0.028	669.7341444	71	Grand Index
30	m	0-12 years	0.029	669.9097554	72	Grand Index
30	m	0-12 years	0.031	670	72	Grand Index

Age	Gender	Education	Percentile	Raw Score	Norm Score	Assessment
30	m	0-12 years	0.032	670.083697	72	Grand Index
30	m	0-12 years	0.033	670.200193	72	Grand Index
30	m	0-12 years	0.034	670.6645971	73	Grand Index
30	m	0-12 years	0.035	671.64	73	Grand Index
30	m	0-12 years	0.036	673.944	73	Grand Index
30	m	0-12 years	0.037	676.56	73	Grand Index
30	m	0-12 years	0.038	679.44	73	Grand Index
30	m	0-12 years	0.039	680.928	74	Grand Index
30	m	0-12 years	0.04	682.002657	74	Grand Index
30	m	0-12 years	0.041	682.0409179	74	Grand Index
30	m	0-12 years	0.042	682.1350981	74	Grand Index
30	m	0-12 years	0.043	682.341117	74	Grand Index
30	m	0-12 years	0.044	683.9982075	74	Grand Index
30	m	0-12 years	0.045	686.6339277	75	Grand Index
30	m	0-12 years	0.046	687.9910922	75	Grand Index
30	m	0-12 years	0.047	688.9983016	75	Grand Index
30	m	0-12 years	0.048	688.9993558	75	Grand Index
30	m	0-12 years	0.049	689.448	75	Grand Index
30	m	0-12 years	0.05	690.6	75	Grand Index
30	m	0-12 years	0.051	691.376	75	Grand Index
30	m	0-12 years	0.052	691.952	76	Grand Index
30	m	0-12 years	0.053	693.056	76	Grand Index
30	m	0-12 years	0.054	694.208	76	Grand Index
30	m	0-12 years	0.055	695.36	76	Grand Index
30	m	0-12 years	0.056	696.1394872	76	Grand Index
30	m	0-12 years	0.057	696.4533333	76	Grand Index
30	m	0-12 years	0.058	696.7305641	76	Grand Index
30	m	0-12 years	0.059	696.9927179	77	Grand Index
30	m	0-12 years	0.06	698.4411853	77	Grand Index
30	m	0-12 years	0.061	699.6315431	77	Grand Index
30	m	0-12 years	0.062	699.877181	77	Grand Index
30	m	0-12 years	0.064	700	77	Grand Index
30	m	0-12 years	0.065	700.44	77	Grand Index
30	m	0-12 years	0.066	701.032	77	Grand Index
30	m	0-12 years	0.067	702.184	78	Grand Index
30	m	0-12 years	0.069	703	78	Grand Index
30	m	0-12 years	0.07	703.32	78	Grand Index
30	m	0-12 years	0.071	703.896	78	Grand Index
30	m	0-12 years	0.072	704.846919	78	Grand Index
30	m	0-12 years	0.073	705.8041926	78	Grand Index
30	m	0-12 years	0.074	705.9226643	78	Grand Index
30	m	0-12 years	0.075	706.2	78	Grand Index
30	m	0-12 years	0.076	706.776	79	Grand Index
30	m	0-12 years	0.079	707	79	Grand Index
30	m	0-12 years	0.08	707.08	79	Grand Index
30	m	0-12 years	0.081	707.656	79	Grand Index
30	m	0-12 years	0.085	708	79	Grand Index
30	m	0-12 years	0.086	708.536	80	Grand Index
30	m	0-12 years	0.087	709.0101818	80	Grand Index
30	m	0-12 years	0.088	709.0625455	80	Grand Index
30	m	0-12 years	0.089	709.3309091	80	Grand Index
30	m	0-12 years	0.09	709.8545455	80	Grand Index
30	m	0-12 years	0.091	710.416	80	Grand Index
30	m	0-12 years	0.092	710.992	80	Grand Index
30	m	0-12 years	0.093	711.568	80	Grand Index
30	m	0-12 years	0.095	712	80	Grand Index

Age	Gender	Education	Percentile	Raw Score	Norm Score	Assessment
30	m	0-12 years	0.096	712.296	80	Grand Index
30	m	0-12 years	0.097	712.872	81	Grand Index
30	m	0-12 years	0.098	713.448	81	Grand Index
30	m	0-12 years	0.099	714.001634	81	Grand Index
30	m	0-12 years	0.1	714.0408492	81	Grand Index
30	m	0-12 years	0.101	714.2320996	81	Grand Index
30	m	0-12 years	0.102	714.7688843	81	Grand Index
30	m	0-12 years	0.104	715	81	Grand Index
30	m	0-12 years	0.105	715.48	81	Grand Index
30	m	0-12 years	0.106	716.112	81	Grand Index
30	m	0-12 years	0.107	717.264	81	Grand Index
30	m	0-12 years	0.108	718.4281527	81	Grand Index
30	m	0-12 years	0.109	719.6138064	82	Grand Index
30	m	0-12 years	0.11	720.397393	82	Grand Index
30	m	0-12 years	0.111	720.9397393	82	Grand Index
30	m	0-12 years	0.112	721.512	82	Grand Index
30	m	0-12 years	0.113	722.057505	82	Grand Index
30	m	0-12 years	0.114	722.433901	82	Grand Index
30	m	0-12 years	0.115	722.7366337	82	Grand Index
30	m	0-12 years	0.116	722.9362376	82	Grand Index
30	m	0-12 years	0.117	723.784	82	Grand Index
30	m	0-12 years	0.118	724.936	82	Grand Index
30	m	0-12 years	0.119	725	82	Grand Index
30	m	0-12 years	0.12	725.12	82	Grand Index
30	m	0-12 years	0.121	725.696	82	Grand Index
30	m	0-12 years	0.125	726	83	Grand Index
30	m	0-12 years	0.126	726.576	83	Grand Index
30	m	0-12 years	0.128	727	83	Grand Index
30	m	0-12 years	0.129	727.304	83	Grand Index
30	m	0-12 years	0.13	727.88	83	Grand Index
30	m	0-12 years	0.131	728	83	Grand Index
30	m	0-12 years	0.132	728.032	83	Grand Index
30	m	0-12 years	0.133	728.608	83	Grand Index
30	m	0-12 years	0.134	729.0023985	83	Grand Index
30	m	0-12 years	0.135	729.009907	83	Grand Index
30	m	0-12 years	0.136	729.3446556	84	Grand Index
30	m	0-12 years	0.137	729.9131471	84	Grand Index
30	m	0-12 years	0.138	730	84	Grand Index
30	m	0-12 years	0.139	730.128	84	Grand Index
30	m	0-12 years	0.14	731.28	84	Grand Index
30	m	0-12 years	0.142	732	84	Grand Index
30	m	0-12 years	0.143	732.2626093	84	Grand Index
30	m	0-12 years	0.144	732.6736498	84	Grand Index
30	m	0-12 years	0.145	732.8625338	84	Grand Index
30	m	0-12 years	0.147	733	84	Grand Index
30	m	0-12 years	0.148	733.2774248	84	Grand Index
30	m	0-12 years	0.149	733.9217663	84	Grand Index
30	m	0-12 years	0.15	734.8711891	84	Grand Index
30	m	0-12 years	0.151	735.9548476	85	Grand Index
30	m	0-12 years	0.152	736	85	Grand Index
30	m	0-12 years	0.153	736.128	85	Grand Index
30	m	0-12 years	0.154	736.704	85	Grand Index
30	m	0-12 years	0.155	737.28	85	Grand Index
30	m	0-12 years	0.156	737.856	85	Grand Index
30	m	0-12 years	0.163	738	85	Grand Index
30	m	0-12 years	0.164	738.928	85	Grand Index

Age	Gender	Education	Percentile	Raw Score	Norm Score	Assessment
30	m	0-12 years	0.17	740	86	Grand Index
30	m	0-12 years	0.171	740.496	86	Grand Index
30	m	0-12 years	0.172	741.072	86	Grand Index
30	m	0-12 years	0.173	741.648	86	Grand Index
30	m	0-12 years	0.175	742	86	Grand Index
30	m	0-12 years	0.176	742.314167	86	Grand Index
30	m	0-12 years	0.177	742.7954441	86	Grand Index
30	m	0-12 years	0.178	742.9223798	86	Grand Index
30	m	0-12 years	0.182	743	86	Grand Index
30	m	0-12 years	0.183	743.3165238	86	Grand Index
30	m	0-12 years	0.184	743.763381	86	Grand Index
30	m	0-12 years	0.185	743.8871351	87	Grand Index
30	m	0-12 years	0.186	744.1140697	87	Grand Index
30	m	0-12 years	0.187	744.7046899	87	Grand Index
30	m	0-12 years	0.196	745	87	Grand Index
30	m	0-12 years	0.197	745.472	87	Grand Index
30	m	0-12 years	0.199	746	87	Grand Index
30	m	0-12 years	0.2	746.2	87	Grand Index
30	m	0-12 years	0.201	746.776	87	Grand Index
30	m	0-12 years	0.203	747	88	Grand Index
30	m	0-12 years	0.204	747.504	88	Grand Index
30	m	0-12 years	0.215	748	88	Grand Index
30	m	0-12 years	0.216	748.416	88	Grand Index
30	m	0-12 years	0.217	748.992	88	Grand Index
30	m	0-12 years	0.218	749	88	Grand Index
30	m	0-12 years	0.219	749.144	88	Grand Index
30	m	0-12 years	0.22	749.72	88	Grand Index
30	m	0-12 years	0.221	750.2732308	88	Grand Index
30	m	0-12 years	0.222	750.8049231	89	Grand Index
30	m	0-12 years	0.223	750.9575385	89	Grand Index
30	m	0-12 years	0.225	751	89	Grand Index
30	m	0-12 years	0.226	751.176	89	Grand Index
30	m	0-12 years	0.227	751.752	89	Grand Index
30	m	0-12 years	0.23	752	89	Grand Index
30	m	0-12 years	0.231	752.056	89	Grand Index
30	m	0-12 years	0.232	752.632	89	Grand Index
30	m	0-12 years	0.233	753.208	89	Grand Index
30	m	0-12 years	0.234	753.784	89	Grand Index
30	m	0-12 years	0.236	754	89	Grand Index
30	m	0-12 years	0.237	754.512	89	Grand Index
30	m	0-12 years	0.239	755	89	Grand Index
30	m	0-12 years	0.24	755.24	89	Grand Index
30	m	0-12 years	0.241	755.816	89	Grand Index
30	m	0-12 years	0.242	756.392	90	Grand Index
30	m	0-12 years	0.243	756.968	90	Grand Index
30	m	0-12 years	0.244	758.088	90	Grand Index
30	m	0-12 years	0.248	759	90	Grand Index
30	m	0-12 years	0.249	760.272	90	Grand Index
30	m	0-12 years	0.251	762	90	Grand Index
30	m	0-12 years	0.252	762.152	90	Grand Index
30	m	0-12 years	0.253	762.728	90	Grand Index
30	m	0-12 years	0.255	763	90	Grand Index
30	m	0-12 years	0.256	763.456	90	Grand Index
30	m	0-12 years	0.257	764.032	90	Grand Index
30	m	0-12 years	0.258	764.608	90	Grand Index
30	m	0-12 years	0.26	765	90	Grand Index

Age	Gender	Education	Percentile	Raw Score	Norm Score	Assessment
30	m	0-12 years	0.261	765.336	90	Grand Index
30	m	0-12 years	0.262	765.912	90	Grand Index
30	m	0-12 years	0.27	766	91	Grand Index
30	m	0-12 years	0.271	766.096	91	Grand Index
30	m	0-12 years	0.272	766.672	91	Grand Index
30	m	0-12 years	0.279	767	91	Grand Index
30	m	0-12 years	0.28	767.28	91	Grand Index
30	m	0-12 years	0.281	767.856	91	Grand Index
30	m	0-12 years	0.282	768	91	Grand Index
30	m	0-12 years	0.283	768.008	91	Grand Index
30	m	0-12 years	0.284	768.584	91	Grand Index
30	m	0-12 years	0.286	769	92	Grand Index
30	m	0-12 years	0.287	769.312	92	Grand Index
30	m	0-12 years	0.288	769.888	92	Grand Index
30	m	0-12 years	0.289	770	92	Grand Index
30	m	0-12 years	0.29	770.0353979	92	Grand Index
30	m	0-12 years	0.291	770.5451275	92	Grand Index
30	m	0-12 years	0.292	770.9070374	92	Grand Index
30	m	0-12 years	0.293	770.9733078	92	Grand Index
30	m	0-12 years	0.294	771.344	92	Grand Index
30	m	0-12 years	0.295	771.92	92	Grand Index
30	m	0-12 years	0.296	772.496	92	Grand Index
30	m	0-12 years	0.297	773.072	92	Grand Index
30	m	0-12 years	0.298	773.648	92	Grand Index
30	m	0-12 years	0.303	774	92	Grand Index
30	m	0-12 years	0.304	774.0327496	92	Grand Index
30	m	0-12 years	0.305	774.214132	92	Grand Index
30	m	0-12 years	0.306	774.3518208	92	Grand Index
30	m	0-12 years	0.307	774.4348927	92	Grand Index
30	m	0-12 years	0.308	774.4679333	92	Grand Index
30	m	0-12 years	0.309	774.4803727	93	Grand Index
30	m	0-12 years	0.31	774.771516	93	Grand Index
30	m	0-12 years	0.321	775	93	Grand Index
30	m	0-12 years	0.322	775.944	93	Grand Index
30	m	0-12 years	0.324	777	93	Grand Index
30	m	0-12 years	0.325	777.2	93	Grand Index
30	m	0-12 years	0.326	777.776	93	Grand Index
30	m	0-12 years	0.331	778	93	Grand Index
30	m	0-12 years	0.332	778.232	93	Grand Index
30	m	0-12 years	0.333	778.808	94	Grand Index
30	m	0-12 years	0.334	779.384	94	Grand Index
30	m	0-12 years	0.335	779.96	94	Grand Index
30	m	0-12 years	0.336	780.3603616	94	Grand Index
30	m	0-12 years	0.337	780.7090169	94	Grand Index
30	m	0-12 years	0.338	780.8977627	94	Grand Index
30	m	0-12 years	0.34	781	94	Grand Index
30	m	0-12 years	0.341	781.393037	94	Grand Index
30	m	0-12 years	0.342	781.937242	94	Grand Index
30	m	0-12 years	0.343	781.9761538	94	Grand Index
30	m	0-12 years	0.347	782	94	Grand Index
30	m	0-12 years	0.348	782.0246929	94	Grand Index
30	m	0-12 years	0.349	782.0777953	94	Grand Index
30	m	0-12 years	0.35	782.6220472	94	Grand Index
30	m	0-12 years	0.354	783	94	Grand Index
30	m	0-12 years	0.355	783.96	94	Grand Index
30	m	0-12 years	0.356	785.0022945	94	Grand Index

Age	Gender	Education	Percentile	Raw Score	Norm Score	Assessment
30	m	0-12 years	0.357	785.025895	95	Grand Index
30	m	0-12 years	0.358	785.2404507	95	Grand Index
30	m	0-12 years	0.359	785.7928502	95	Grand Index
30	m	0-12 years	0.361	786	95	Grand Index
30	m	0-12 years	0.362	786.512	95	Grand Index
30	m	0-12 years	0.364	787	95	Grand Index
30	m	0-12 years	0.365	787.24	95	Grand Index
30	m	0-12 years	0.366	787.816	95	Grand Index
30	m	0-12 years	0.367	788.392	95	Grand Index
30	m	0-12 years	0.368	788.968	95	Grand Index
30	m	0-12 years	0.371	789	95	Grand Index
30	m	0-12 years	0.372	789.272	95	Grand Index
30	m	0-12 years	0.373	789.848	95	Grand Index
30	m	0-12 years	0.381	790	95	Grand Index
30	m	0-12 years	0.382	790.032	95	Grand Index
30	m	0-12 years	0.383	790.608	96	Grand Index
30	m	0-12 years	0.385	791	96	Grand Index
30	m	0-12 years	0.386	791.336	96	Grand Index
30	m	0-12 years	0.387	791.912	96	Grand Index
30	m	0-12 years	0.388	792	96	Grand Index
30	m	0-12 years	0.389	792.064	96	Grand Index
30	m	0-12 years	0.39	792.64	96	Grand Index
30	m	0-12 years	0.392	793	96	Grand Index
30	m	0-12 years	0.393	793.368	96	Grand Index
30	m	0-12 years	0.394	793.944	96	Grand Index
30	m	0-12 years	0.404	794	96	Grand Index
30	m	0-12 years	0.405	794.0065025	96	Grand Index
30	m	0-12 years	0.406	794.0198791	96	Grand Index
30	m	0-12 years	0.407	794.4451908	96	Grand Index
30	m	0-12 years	0.413	795	97	Grand Index
30	m	0-12 years	0.414	795.464	97	Grand Index
30	m	0-12 years	0.421	796	97	Grand Index
30	m	0-12 years	0.422	796.072	97	Grand Index
30	m	0-12 years	0.423	796.648	97	Grand Index
30	m	0-12 years	0.425	797	97	Grand Index
30	m	0-12 years	0.426	797.376	97	Grand Index
30	m	0-12 years	0.427	797.952	97	Grand Index
30	m	0-12 years	0.43	798	97	Grand Index
30	m	0-12 years	0.431	798.2508936	97	Grand Index
30	m	0-12 years	0.432	798.8154043	97	Grand Index
30	m	0-12 years	0.433	798.9881915	97	Grand Index
30	m	0-12 years	0.434	798.9996809	98	Grand Index
30	m	0-12 years	0.439	799	98	Grand Index
30	m	0-12 years	0.44	799.44	98	Grand Index
30	m	0-12 years	0.442	800	98	Grand Index
30	m	0-12 years	0.443	800.1568284	98	Grand Index
30	m	0-12 years	0.444	800.6945256	98	Grand Index
30	m	0-12 years	0.445	800.9547815	98	Grand Index
30	m	0-12 years	0.446	800.9930842	98	Grand Index
30	m	0-12 years	0.447	801	98	Grand Index
30	m	0-12 years	0.448	801.0001124	98	Grand Index
30	m	0-12 years	0.449	801.0014614	98	Grand Index
30	m	0-12 years	0.45	801.0032842	98	Grand Index
30	m	0-12 years	0.451	801.0059979	98	Grand Index
30	m	0-12 years	0.452	801.3565705	98	Grand Index
30	m	0-12 years	0.453	801.9285078	98	Grand Index

Age	Gender	Education	Percentile	Raw Score	Norm Score	Assessment
30	m	0-12 years	0.458	802	98	Grand Index
30	m	0-12 years	0.459	802.384	98	Grand Index
30	m	0-12 years	0.46	802.96	98	Grand Index
30	m	0-12 years	0.465	803	99	Grand Index
30	m	0-12 years	0.466	803.416	99	Grand Index
30	m	0-12 years	0.467	803.992	99	Grand Index
30	m	0-12 years	0.468	804	99	Grand Index
30	m	0-12 years	0.469	804.144	99	Grand Index
30	m	0-12 years	0.47	804.72	99	Grand Index
30	m	0-12 years	0.473	805	99	Grand Index
30	m	0-12 years	0.474	805.024	99	Grand Index
30	m	0-12 years	0.475	805.6	99	Grand Index
30	m	0-12 years	0.476	806.176	99	Grand Index
30	m	0-12 years	0.477	806.752	99	Grand Index
30	m	0-12 years	0.482	807	99	Grand Index
30	m	0-12 years	0.483	807.1083333	99	Grand Index
30	m	0-12 years	0.484	807.4083333	99	Grand Index
30	m	0-12 years	0.485	807.6020657	99	Grand Index
30	m	0-12 years	0.486	807.7320376	99	Grand Index
30	m	0-12 years	0.487	807.8762817	100	Grand Index
30	m	0-12 years	0.494	808	100	Grand Index
30	m	0-12 years	0.495	808.12	100	Grand Index
30	m	0-12 years	0.496	808.696	100	Grand Index
30	m	0-12 years	0.497	809.272	100	Grand Index
30	m	0-12 years	0.498	809.848	100	Grand Index
30	m	0-12 years	0.501	810	100	Grand Index
30	m	0-12 years	0.502	810.152	100	Grand Index
30	m	0-12 years	0.503	810.728	100	Grand Index
30	m	0-12 years	0.506	811	100	Grand Index
30	m	0-12 years	0.507	811.032	100	Grand Index
30	m	0-12 years	0.508	811.608	100	Grand Index
30	m	0-12 years	0.51	812	100	Grand Index
30	m	0-12 years	0.511	812.336	100	Grand Index
30	m	0-12 years	0.512	812.912	100	Grand Index
30	m	0-12 years	0.513	813	100	Grand Index
30	m	0-12 years	0.514	813.064	101	Grand Index
30	m	0-12 years	0.515	813.64	101	Grand Index
30	m	0-12 years	0.522	814	101	Grand Index
30	m	0-12 years	0.523	814.248	101	Grand Index
30	m	0-12 years	0.524	814.824	101	Grand Index
30	m	0-12 years	0.529	815	101	Grand Index
30	m	0-12 years	0.53	815.28	101	Grand Index
30	m	0-12 years	0.531	815.856	101	Grand Index
30	m	0-12 years	0.534	816	101	Grand Index
30	m	0-12 years	0.535	816.16	101	Grand Index
30	m	0-12 years	0.536	816.736	101	Grand Index
30	m	0-12 years	0.538	817	101	Grand Index
30	m	0-12 years	0.539	817.464	101	Grand Index
30	m	0-12 years	0.543	818	102	Grand Index
30	m	0-12 years	0.544	818.344	102	Grand Index
30	m	0-12 years	0.545	818.92	102	Grand Index
30	m	0-12 years	0.546	819	102	Grand Index
30	m	0-12 years	0.547	819.021896	102	Grand Index
30	m	0-12 years	0.548	819.1970644	102	Grand Index
30	m	0-12 years	0.549	819.4599907	102	Grand Index
30	m	0-12 years	0.55	819.8608223	102	Grand Index

Age	Gender	Education	Percentile	Raw Score	Norm Score	Assessment
30	m	0-12 years	0.553	820	102	Grand Index
30	m	0-12 years	0.554	820.104	102	Grand Index
30	m	0-12 years	0.555	820.68	102	Grand Index
30	m	0-12 years	0.56	821	102	Grand Index
30	m	0-12 years	0.561	821.1302737	102	Grand Index
30	m	0-12 years	0.562	821.682021	102	Grand Index
30	m	0-12 years	0.563	821.970021	102	Grand Index
30	m	0-12 years	0.564	821.9942737	102	Grand Index
30	m	0-12 years	0.565	823.32	102	Grand Index
30	m	0-12 years	0.569	825	103	Grand Index
30	m	0-12 years	0.57	825.0050324	103	Grand Index
30	m	0-12 years	0.571	825.0140907	103	Grand Index
30	m	0-12 years	0.572	825.2819266	103	Grand Index
30	m	0-12 years	0.573	825.59368	103	Grand Index
30	m	0-12 years	0.574	825.761318	103	Grand Index
30	m	0-12 years	0.575	825.8965986	103	Grand Index
30	m	0-12 years	0.576	825.9710476	103	Grand Index
30	m	0-12 years	0.581	826	103	Grand Index
30	m	0-12 years	0.582	826.232	103	Grand Index
30	m	0-12 years	0.583	826.808	103	Grand Index
30	m	0-12 years	0.585	827	103	Grand Index
30	m	0-12 years	0.586	827.3543051	103	Grand Index
30	m	0-12 years	0.587	827.8109831	103	Grand Index
30	m	0-12 years	0.588	828.5822373	103	Grand Index
30	m	0-12 years	0.589	829.264	103	Grand Index
30	m	0-12 years	0.59	829.84	103	Grand Index
30	m	0-12 years	0.593	830	104	Grand Index
30	m	0-12 years	0.594	830.144	104	Grand Index
30	m	0-12 years	0.595	830.72	104	Grand Index
30	m	0-12 years	0.597	831	104	Grand Index
30	m	0-12 years	0.598	831.448	104	Grand Index
30	m	0-12 years	0.605	832	104	Grand Index
30	m	0-12 years	0.606	832.056	104	Grand Index
30	m	0-12 years	0.607	832.632	104	Grand Index
30	m	0-12 years	0.608	833.208	104	Grand Index
30	m	0-12 years	0.609	833.784	104	Grand Index
30	m	0-12 years	0.612	834	104	Grand Index
30	m	0-12 years	0.613	834.088	104	Grand Index
30	m	0-12 years	0.614	834.664	104	Grand Index
30	m	0-12 years	0.615	835.24	104	Grand Index
30	m	0-12 years	0.616	835.816	104	Grand Index
30	m	0-12 years	0.619	836	105	Grand Index
30	m	0-12 years	0.62	836.12	105	Grand Index
30	m	0-12 years	0.621	836.696	105	Grand Index
30	m	0-12 years	0.625	837	105	Grand Index
30	m	0-12 years	0.626	837.5031325	105	Grand Index
30	m	0-12 years	0.627	837.8927229	105	Grand Index
30	m	0-12 years	0.628	837.9655904	105	Grand Index
30	m	0-12 years	0.631	838	105	Grand Index
30	m	0-12 years	0.632	838.064	105	Grand Index
30	m	0-12 years	0.633	839.216	105	Grand Index
30	m	0-12 years	0.634	840.184	105	Grand Index
30	m	0-12 years	0.635	840.76	105	Grand Index
30	m	0-12 years	0.637	841	105	Grand Index
30	m	0-12 years	0.638	841.488	105	Grand Index
30	m	0-12 years	0.639	842.064	105	Grand Index

Age	Gender	Education	Percentile	Raw Score	Norm Score	Assessment
30	m	0-12 years	0.64	842.64	105	Grand Index
30	m	0-12 years	0.642	843	105	Grand Index
30	m	0-12 years	0.643	843.3475211	105	Grand Index
30	m	0-12 years	0.644	843.8914671	106	Grand Index
30	m	0-12 years	0.645	843.9732884	106	Grand Index
30	m	0-12 years	0.651	844	106	Grand Index
30	m	0-12 years	0.652	844.552	106	Grand Index
30	m	0-12 years	0.653	845.128	106	Grand Index
30	m	0-12 years	0.654	845.704	106	Grand Index
30	m	0-12 years	0.663	846	106	Grand Index
30	m	0-12 years	0.664	846.3787755	106	Grand Index
30	m	0-12 years	0.665	846.8236735	106	Grand Index
30	m	0-12 years	0.666	846.9294694	106	Grand Index
30	m	0-12 years	0.667	847.192	106	Grand Index
30	m	0-12 years	0.668	847.768	107	Grand Index
30	m	0-12 years	0.67	848	107	Grand Index
30	m	0-12 years	0.671	848.496	107	Grand Index
30	m	0-12 years	0.672	849.072	107	Grand Index
30	m	0-12 years	0.673	849.648	107	Grand Index
30	m	0-12 years	0.685	850	107	Grand Index
30	m	0-12 years	0.686	850.136	107	Grand Index
30	m	0-12 years	0.687	850.712	107	Grand Index
30	m	0-12 years	0.69	851	107	Grand Index
30	m	0-12 years	0.691	851.0092952	107	Grand Index
30	m	0-12 years	0.692	851.3439238	108	Grand Index
30	m	0-12 years	0.693	851.6513524	108	Grand Index
30	m	0-12 years	0.694	851.8927238	108	Grand Index
30	m	0-12 years	0.696	852	108	Grand Index
30	m	0-12 years	0.697	852.1291625	108	Grand Index
30	m	0-12 years	0.698	852.3083786	108	Grand Index
30	m	0-12 years	0.699	852.7251303	108	Grand Index
30	m	0-12 years	0.7	852.9977413	108	Grand Index
30	m	0-12 years	0.701	852.9993676	108	Grand Index
30	m	0-12 years	0.702	853.352	108	Grand Index
30	m	0-12 years	0.703	853.928	108	Grand Index
30	m	0-12 years	0.704	854	108	Grand Index
30	m	0-12 years	0.705	854.08	108	Grand Index
30	m	0-12 years	0.706	854.656	108	Grand Index
30	m	0-12 years	0.708	855	108	Grand Index
30	m	0-12 years	0.709	855.384	108	Grand Index
30	m	0-12 years	0.71	855.96	108	Grand Index
30	m	0-12 years	0.717	856	109	Grand Index
30	m	0-12 years	0.718	856.568	109	Grand Index
30	m	0-12 years	0.722	857	109	Grand Index
30	m	0-12 years	0.723	857.448	109	Grand Index
30	m	0-12 years	0.73	858	109	Grand Index
30	m	0-12 years	0.731	858.056	109	Grand Index
30	m	0-12 years	0.732	858.632	109	Grand Index
30	m	0-12 years	0.733	859.208	109	Grand Index
30	m	0-12 years	0.734	859.784	109	Grand Index
30	m	0-12 years	0.735	860.36	109	Grand Index
30	m	0-12 years	0.736	860.936	109	Grand Index
30	m	0-12 years	0.737	861.453515	110	Grand Index
30	m	0-12 years	0.738	861.8958236	110	Grand Index
30	m	0-12 years	0.739	861.9616192	110	Grand Index
30	m	0-12 years	0.743	862	110	Grand Index

Age	Gender	Education	Percentile	Raw Score	Norm Score	Assessment
30	m	0-12 years	0.744	862.544	110	Grand Index
30	m	0-12 years	0.746	863	110	Grand Index
30	m	0-12 years	0.747	863.272	110	Grand Index
30	m	0-12 years	0.748	863.848	110	Grand Index
30	m	0-12 years	0.753	864	110	Grand Index
30	m	0-12 years	0.754	864.304	110	Grand Index
30	m	0-12 years	0.755	864.88	110	Grand Index
30	m	0-12 years	0.76	865	111	Grand Index
30	m	0-12 years	0.761	865.0804493	111	Grand Index
30	m	0-12 years	0.762	865.2183623	111	Grand Index
30	m	0-12 years	0.763	865.6105894	111	Grand Index
30	m	0-12 years	0.769	866	111	Grand Index
30	m	0-12 years	0.77	866.52	111	Grand Index
30	m	0-12 years	0.772	867	111	Grand Index
30	m	0-12 years	0.773	867.248	111	Grand Index
30	m	0-12 years	0.774	867.824	111	Grand Index
30	m	0-12 years	0.776	868	111	Grand Index
30	m	0-12 years	0.777	868.4321751	111	Grand Index
30	m	0-12 years	0.778	868.8107114	111	Grand Index
30	m	0-12 years	0.779	868.9357461	112	Grand Index
30	m	0-12 years	0.78	869.28	112	Grand Index
30	m	0-12 years	0.781	869.856	112	Grand Index
30	m	0-12 years	0.782	870	112	Grand Index
30	m	0-12 years	0.783	870.008	112	Grand Index
30	m	0-12 years	0.784	870.584	112	Grand Index
30	m	0-12 years	0.789	871	112	Grand Index
30	m	0-12 years	0.79	871.04	112	Grand Index
30	m	0-12 years	0.791	871.616	112	Grand Index
30	m	0-12 years	0.792	872.192	112	Grand Index
30	m	0-12 years	0.793	872.768	112	Grand Index
30	m	0-12 years	0.794	873.688	112	Grand Index
30	m	0-12 years	0.795	874.84	112	Grand Index
30	m	0-12 years	0.796	875.1049646	112	Grand Index
30	m	0-12 years	0.797	875.3403854	112	Grand Index
30	m	0-12 years	0.798	876.370491	113	Grand Index
30	m	0-12 years	0.8	877	113	Grand Index
30	m	0-12 years	0.801	877.366481	113	Grand Index
30	m	0-12 years	0.802	877.9278987	113	Grand Index
30	m	0-12 years	0.803	877.9880506	113	Grand Index
30	m	0-12 years	0.805	878	113	Grand Index
30	m	0-12 years	0.806	878.077913	113	Grand Index
30	m	0-12 years	0.807	878.2532174	113	Grand Index
30	m	0-12 years	0.808	878.5881739	113	Grand Index
30	m	0-12 years	0.809	878.9888696	113	Grand Index
30	m	0-12 years	0.81	879.56	113	Grand Index
30	m	0-12 years	0.814	880	113	Grand Index
30	m	0-12 years	0.815	880.44	113	Grand Index
30	m	0-12 years	0.817	881	114	Grand Index
30	m	0-12 years	0.818	881.168	114	Grand Index
30	m	0-12 years	0.819	881.744	114	Grand Index
30	m	0-12 years	0.822	882	114	Grand Index
30	m	0-12 years	0.823	882.096	114	Grand Index
30	m	0-12 years	0.824	883.248	114	Grand Index
30	m	0-12 years	0.826	884	114	Grand Index
30	m	0-12 years	0.827	884.352	114	Grand Index
30	m	0-12 years	0.828	884.928	114	Grand Index

Age	Gender	Education	Percentile	Raw Score	Norm Score	Assessment
30	m	0-12 years	0.829	885.504	114	Grand Index
30	m	0-12 years	0.83	886.08	114	Grand Index
30	m	0-12 years	0.831	886.656	114	Grand Index
30	m	0-12 years	0.833	887	114	Grand Index
30	m	0-12 years	0.834	887.3657469	115	Grand Index
30	m	0-12 years	0.835	887.9143672	115	Grand Index
30	m	0-12 years	0.836	887.9779441	115	Grand Index
30	m	0-12 years	0.838	888	115	Grand Index
30	m	0-12 years	0.839	888.264	115	Grand Index
30	m	0-12 years	0.84	888.84	115	Grand Index
30	m	0-12 years	0.842	889	115	Grand Index
30	m	0-12 years	0.843	889.568	115	Grand Index
30	m	0-12 years	0.845	890	115	Grand Index
30	m	0-12 years	0.846	890.592	115	Grand Index
30	m	0-12 years	0.847	891.744	115	Grand Index
30	m	0-12 years	0.848	893.344	115	Grand Index
30	m	0-12 years	0.849	895.024	115	Grand Index
30	m	0-12 years	0.85	895.6	116	Grand Index
30	m	0-12 years	0.851	896.0308454	116	Grand Index
30	m	0-12 years	0.852	896.1317938	116	Grand Index
30	m	0-12 years	0.853	896.4457732	116	Grand Index
30	m	0-12 years	0.854	896.9208247	116	Grand Index
30	m	0-12 years	0.855	897.48	116	Grand Index
30	m	0-12 years	0.859	898	116	Grand Index
30	m	0-12 years	0.86	898.36	116	Grand Index
30	m	0-12 years	0.861	898.936	116	Grand Index
30	m	0-12 years	0.862	899.512	116	Grand Index
30	m	0-12 years	0.863	900.088	116	Grand Index
30	m	0-12 years	0.864	900.664	116	Grand Index
30	m	0-12 years	0.866	901	117	Grand Index
30	m	0-12 years	0.867	901.3335111	117	Grand Index
30	m	0-12 years	0.868	901.8235683	117	Grand Index
30	m	0-12 years	0.869	901.9319619	117	Grand Index
30	m	0-12 years	0.87	902.12	117	Grand Index
30	m	0-12 years	0.871	902.696	117	Grand Index
30	m	0-12 years	0.878	903	117	Grand Index
30	m	0-12 years	0.879	903.304	118	Grand Index
30	m	0-12 years	0.88	903.88	118	Grand Index
30	m	0-12 years	0.881	905.368	118	Grand Index
30	m	0-12 years	0.885	907	118	Grand Index
30	m	0-12 years	0.886	907.336	118	Grand Index
30	m	0-12 years	0.887	907.912	118	Grand Index
30	m	0-12 years	0.888	908	118	Grand Index
30	m	0-12 years	0.889	908.064	118	Grand Index
30	m	0-12 years	0.89	908.64	118	Grand Index
30	m	0-12 years	0.891	909.387	118	Grand Index
30	m	0-12 years	0.892	910.419	119	Grand Index
30	m	0-12 years	0.893	910.8683333	119	Grand Index
30	m	0-12 years	0.894	910.9883333	119	Grand Index
30	m	0-12 years	0.895	911	119	Grand Index
30	m	0-12 years	0.896	911.192	119	Grand Index
30	m	0-12 years	0.897	912.344	119	Grand Index
30	m	0-12 years	0.899	913	119	Grand Index
30	m	0-12 years	0.9	913.2446352	119	Grand Index
30	m	0-12 years	0.901	913.5969099	119	Grand Index
30	m	0-12 years	0.902	913.8259914	119	Grand Index

Age	Gender	Education	Percentile	Raw Score	Norm Score	Assessment
30	m	0-12 years	0.903	914.128	119	Grand Index
30	m	0-12 years	0.904	914.704	120	Grand Index
30	m	0-12 years	0.906	915	120	Grand Index
30	m	0-12 years	0.907	915.432	120	Grand Index
30	m	0-12 years	0.911	916	120	Grand Index
30	m	0-12 years	0.912	916.624	120	Grand Index
30	m	0-12 years	0.913	917.776	120	Grand Index
30	m	0-12 years	0.914	918.464	120	Grand Index
30	m	0-12 years	0.915	919.0987668	121	Grand Index
30	m	0-12 years	0.916	920.521008	121	Grand Index
30	m	0-12 years	0.917	921.5710885	121	Grand Index
30	m	0-12 years	0.918	921.8768472	121	Grand Index
30	m	0-12 years	0.919	922.866919	121	Grand Index
30	m	0-12 years	0.92	924.3185044	121	Grand Index
30	m	0-12 years	0.921	924.7581372	121	Grand Index
30	m	0-12 years	0.925	925	122	Grand Index
30	m	0-12 years	0.926	925.376	122	Grand Index
30	m	0-12 years	0.927	925.952	122	Grand Index
30	m	0-12 years	0.928	926.306316	122	Grand Index
30	m	0-12 years	0.929	926.6949748	122	Grand Index
30	m	0-12 years	0.93	927.3309612	122	Grand Index
30	m	0-12 years	0.931	927.7651095	122	Grand Index
30	m	0-12 years	0.932	927.9469602	122	Grand Index
30	m	0-12 years	0.933	928.3263473	122	Grand Index
30	m	0-12 years	0.934	928.787073	123	Grand Index
30	m	0-12 years	0.935	928.9119432	123	Grand Index
30	m	0-12 years	0.936	929.136	123	Grand Index
30	m	0-12 years	0.937	929.712	123	Grand Index
30	m	0-12 years	0.938	930.107808	123	Grand Index
30	m	0-12 years	0.939	930.3234241	123	Grand Index
30	m	0-12 years	0.94	930.6496267	123	Grand Index
30	m	0-12 years	0.941	931.0216356	123	Grand Index
30	m	0-12 years	0.942	931.800517	124	Grand Index
30	m	0-12 years	0.943	932.4610509	124	Grand Index
30	m	0-12 years	0.944	932.8341695	124	Grand Index
30	m	0-12 years	0.945	933.32	124	Grand Index
30	m	0-12 years	0.946	933.896	124	Grand Index
30	m	0-12 years	0.947	934.472	124	Grand Index
30	m	0-12 years	0.949	935	125	Grand Index
30	m	0-12 years	0.95	935.2	125	Grand Index
30	m	0-12 years	0.951	935.776	125	Grand Index
30	m	0-12 years	0.952	936.352	125	Grand Index
30	m	0-12 years	0.953	936.928	125	Grand Index
30	m	0-12 years	0.954	938.512	125	Grand Index
30	m	0-12 years	0.955	940.8	125	Grand Index
30	m	0-12 years	0.956	946.56	126	Grand Index
30	m	0-12 years	0.958	950	126	Grand Index
30	m	0-12 years	0.959	951.152	126	Grand Index
30	m	0-12 years	0.96	952.88	126	Grand Index
30	m	0-12 years	0.961	953.9063273	126	Grand Index
30	m	0-12 years	0.962	954.7255273	127	Grand Index
30	m	0-12 years	0.963	954.9035636	127	Grand Index
30	m	0-12 years	0.965	955	127	Grand Index
30	m	0-12 years	0.966	956.248	127	Grand Index
30	m	0-12 years	0.967	957.976	128	Grand Index
30	m	0-12 years	0.968	959.136	128	Grand Index

Age	Gender	Education	Percentile	Raw Score	Norm Score	Assessment
30	m	0-12 years	0.969	960.0543275	128	Grand Index
30	m	0-12 years	0.97	960.2716377	128	Grand Index
30	m	0-12 years	0.971	961.4496013	128	Grand Index
30	m	0-12 years	0.972	963.5362911	129	Grand Index
30	m	0-12 years	0.973	964	129	Grand Index
30	m	0-12 years	0.974	964.12	129	Grand Index
30	m	0-12 years	0.975	967	129	Grand Index
30	m	0-12 years	0.976	969.88	130	Grand Index
30	m	0-12 years	0.977	972.76	130	Grand Index
30	m	0-12 years	0.978	975.9673233	130	Grand Index
30	m	0-12 years	0.979	979.4221349	131	Grand Index
30	m	0-12 years	0.98	979.9989271	131	Grand Index
30	m	0-12 years	0.981	980.2096404	131	Grand Index
30	m	0-12 years	0.982	982.3659421	131	Grand Index
30	m	0-12 years	0.983	984.1434398	132	Grand Index
30	m	0-12 years	0.984	985.2507459	132	Grand Index
30	m	0-12 years	0.985	985.9319621	133	Grand Index
30	m	0-12 years	0.986	986.3575244	133	Grand Index
30	m	0-12 years	0.987	986.7456032	133	Grand Index
30	m	0-12 years	0.988	987.1522254	134	Grand Index
30	m	0-12 years	0.989	987.687662	134	Grand Index
30	m	0-12 years	0.99	988.24	135	Grand Index
30	m	0-12 years	0.991	988.816	135	Grand Index
30	m	0-12 years	0.992	991.744	136	Grand Index
30	m	0-12 years	0.993	995.776	137	Grand Index
30	m	0-12 years	0.994	997.088	138	Grand Index
30	m	0-12 years	0.995	999.9024619	139	Grand Index
30	m	0-12 years	0.996	1009.034279	140	Grand Index
30	m	0-12 years	0.997	1014.981602	141	Grand Index
30	m	0-12 years	0.998	1017.369785	143	Grand Index
30	m	0-12 years	0.999	1018.553274	146	Grand Index
30	f	13-16 years	0.001	568.5636364	54	Grand Index
30	f	13-16 years	0.002	584.7994472	57	Grand Index
30	f	13-16 years	0.003	594.3110931	59	Grand Index
30	f	13-16 years	0.004	603.7810759	60	Grand Index
30	f	13-16 years	0.005	621	61	Grand Index
30	f	13-16 years	0.006	626.6	62	Grand Index
30	f	13-16 years	0.007	630.8	63	Grand Index
30	f	13-16 years	0.008	633.4967136	64	Grand Index
30	f	13-16 years	0.009	634.4790009	65	Grand Index
30	f	13-16 years	0.01	638	65	Grand Index
30	f	13-16 years	0.011	641.4	66	Grand Index
30	f	13-16 years	0.012	646.4484392	66	Grand Index
30	f	13-16 years	0.013	649.8485398	67	Grand Index
30	f	13-16 years	0.014	651.0645437	67	Grand Index
30	f	13-16 years	0.015	652	67	Grand Index
30	f	13-16 years	0.016	653	68	Grand Index
30	f	13-16 years	0.017	653.4801005	68	Grand Index
30	f	13-16 years	0.018	655.1728425	69	Grand Index
30	f	13-16 years	0.019	658.2212372	69	Grand Index
30	f	13-16 years	0.02	660	69	Grand Index
30	f	13-16 years	0.021	662	69	Grand Index
30	f	13-16 years	0.022	662.9979895	70	Grand Index
30	f	13-16 years	0.023	663	70	Grand Index
30	f	13-16 years	0.024	664.8876688	70	Grand Index
30	f	13-16 years	0.025	666	71	Grand Index

Age	Gender	Education	Percentile	Raw Score	Norm Score	Assessment
30	f	13-16 years	0.026	667	71	Grand Index
30	f	13-16 years	0.027	668.0032464	71	Grand Index
30	f	13-16 years	0.028	669	71	Grand Index
30	f	13-16 years	0.029	670.6432432	72	Grand Index
30	f	13-16 years	0.03	671	72	Grand Index
30	f	13-16 years	0.031	671.0491228	72	Grand Index
30	f	13-16 years	0.032	672	72	Grand Index
30	f	13-16 years	0.033	673.2	72	Grand Index
30	f	13-16 years	0.035	674	73	Grand Index
30	f	13-16 years	0.036	674.3904125	73	Grand Index
30	f	13-16 years	0.037	675	73	Grand Index
30	f	13-16 years	0.038	675.6117072	73	Grand Index
30	f	13-16 years	0.039	676	74	Grand Index
30	f	13-16 years	0.04	677	74	Grand Index
30	f	13-16 years	0.041	677.88	74	Grand Index
30	f	13-16 years	0.042	680.8	74	Grand Index
30	f	13-16 years	0.043	681.3424033	74	Grand Index
30	f	13-16 years	0.044	682	74	Grand Index
30	f	13-16 years	0.045	683	75	Grand Index
30	f	13-16 years	0.046	683.4	75	Grand Index
30	f	13-16 years	0.047	684.8	75	Grand Index
30	f	13-16 years	0.048	686	75	Grand Index
30	f	13-16 years	0.049	686.5963748	75	Grand Index
30	f	13-16 years	0.05	687	75	Grand Index
30	f	13-16 years	0.051	689	75	Grand Index
30	f	13-16 years	0.052	690.6	76	Grand Index
30	f	13-16 years	0.053	692.04	76	Grand Index
30	f	13-16 years	0.054	693	76	Grand Index
30	f	13-16 years	0.056	695	76	Grand Index
30	f	13-16 years	0.057	695.9748111	76	Grand Index
30	f	13-16 years	0.058	696	76	Grand Index
30	f	13-16 years	0.059	697	77	Grand Index
30	f	13-16 years	0.06	697.125	77	Grand Index
30	f	13-16 years	0.063	698	77	Grand Index
30	f	13-16 years	0.064	698.3577688	77	Grand Index
30	f	13-16 years	0.065	698.95	77	Grand Index
30	f	13-16 years	0.066	699.3294339	77	Grand Index
30	f	13-16 years	0.067	700	78	Grand Index
30	f	13-16 years	0.069	701	78	Grand Index
30	f	13-16 years	0.07	701.1640241	78	Grand Index
30	f	13-16 years	0.071	701.8955102	78	Grand Index
30	f	13-16 years	0.072	702	78	Grand Index
30	f	13-16 years	0.074	703	78	Grand Index
30	f	13-16 years	0.075	703.9265442	78	Grand Index
30	f	13-16 years	0.076	705	79	Grand Index
30	f	13-16 years	0.077	705.8	79	Grand Index
30	f	13-16 years	0.078	706	79	Grand Index
30	f	13-16 years	0.079	706.6	79	Grand Index
30	f	13-16 years	0.08	707.3566085	79	Grand Index
30	f	13-16 years	0.081	707.7031017	79	Grand Index
30	f	13-16 years	0.084	708	79	Grand Index
30	f	13-16 years	0.086	709	80	Grand Index
30	f	13-16 years	0.087	709.7347584	80	Grand Index
30	f	13-16 years	0.088	710.1085271	80	Grand Index
30	f	13-16 years	0.089	710.9692308	80	Grand Index
30	f	13-16 years	0.09	711.6741573	80	Grand Index

Age	Gender	Education	Percentile	Raw Score	Norm Score	Assessment
30	f	13-16 years	0.091	712.3892125	80	Grand Index
30	f	13-16 years	0.092	712.9989385	80	Grand Index
30	f	13-16 years	0.094	713	80	Grand Index
30	f	13-16 years	0.096	714	80	Grand Index
30	f	13-16 years	0.097	714.839454	81	Grand Index
30	f	13-16 years	0.098	716	81	Grand Index
30	f	13-16 years	0.099	716.4963044	81	Grand Index
30	f	13-16 years	0.1	717	81	Grand Index
30	f	13-16 years	0.101	717.3591925	81	Grand Index
30	f	13-16 years	0.103	718	81	Grand Index
30	f	13-16 years	0.104	718.8068966	81	Grand Index
30	f	13-16 years	0.105	719	81	Grand Index
30	f	13-16 years	0.106	720	81	Grand Index
30	f	13-16 years	0.107	720.8672632	81	Grand Index
30	f	13-16 years	0.108	721	81	Grand Index
30	f	13-16 years	0.109	721.0921502	82	Grand Index
30	f	13-16 years	0.11	722	82	Grand Index
30	f	13-16 years	0.112	723	82	Grand Index
30	f	13-16 years	0.113	723.2	82	Grand Index
30	f	13-16 years	0.115	724	82	Grand Index
30	f	13-16 years	0.117	725	82	Grand Index
30	f	13-16 years	0.118	725.4696629	82	Grand Index
30	f	13-16 years	0.12	726	82	Grand Index
30	f	13-16 years	0.121	726.3879646	82	Grand Index
30	f	13-16 years	0.122	726.9592357	83	Grand Index
30	f	13-16 years	0.124	727	83	Grand Index
30	f	13-16 years	0.125	727.9748111	83	Grand Index
30	f	13-16 years	0.128	728	83	Grand Index
30	f	13-16 years	0.129	728.5348837	83	Grand Index
30	f	13-16 years	0.132	729	83	Grand Index
30	f	13-16 years	0.136	730	84	Grand Index
30	f	13-16 years	0.139	731	84	Grand Index
30	f	13-16 years	0.141	732	84	Grand Index
30	f	13-16 years	0.142	732.2373626	84	Grand Index
30	f	13-16 years	0.145	733	84	Grand Index
30	f	13-16 years	0.146	733.4	84	Grand Index
30	f	13-16 years	0.148	734	84	Grand Index
30	f	13-16 years	0.149	734.63	84	Grand Index
30	f	13-16 years	0.152	735	85	Grand Index
30	f	13-16 years	0.153	736	85	Grand Index
30	f	13-16 years	0.154	736.5002079	85	Grand Index
30	f	13-16 years	0.155	737	85	Grand Index
30	f	13-16 years	0.157	738	85	Grand Index
30	f	13-16 years	0.158	738.1157895	85	Grand Index
30	f	13-16 years	0.16	739	85	Grand Index
30	f	13-16 years	0.161	739.4	85	Grand Index
30	f	13-16 years	0.164	740	85	Grand Index
30	f	13-16 years	0.168	741	86	Grand Index
30	f	13-16 years	0.171	742	86	Grand Index
30	f	13-16 years	0.172	742.9436658	86	Grand Index
30	f	13-16 years	0.174	743	86	Grand Index
30	f	13-16 years	0.176	744	86	Grand Index
30	f	13-16 years	0.177	745	86	Grand Index
30	f	13-16 years	0.178	745.0074074	86	Grand Index
30	f	13-16 years	0.179	745.6447169	86	Grand Index
30	f	13-16 years	0.184	746	86	Grand Index

Age	Gender	Education	Percentile	Raw Score	Norm Score	Assessment
30	f	13-16 years	0.187	747	87	Grand Index
30	f	13-16 years	0.188	747.9702629	87	Grand Index
30	f	13-16 years	0.189	748	87	Grand Index
30	f	13-16 years	0.19	748.6405694	87	Grand Index
30	f	13-16 years	0.194	749	87	Grand Index
30	f	13-16 years	0.197	750	87	Grand Index
30	f	13-16 years	0.198	750.0946095	87	Grand Index
30	f	13-16 years	0.199	751	87	Grand Index
30	f	13-16 years	0.2	751.0870833	87	Grand Index
30	f	13-16 years	0.202	752	87	Grand Index
30	f	13-16 years	0.203	752.8069565	88	Grand Index
30	f	13-16 years	0.207	753	88	Grand Index
30	f	13-16 years	0.208	753.2	88	Grand Index
30	f	13-16 years	0.211	754	88	Grand Index
30	f	13-16 years	0.212	754.2591549	88	Grand Index
30	f	13-16 years	0.215	755	88	Grand Index
30	f	13-16 years	0.216	755.4	88	Grand Index
30	f	13-16 years	0.222	756	89	Grand Index
30	f	13-16 years	0.225	757	89	Grand Index
30	f	13-16 years	0.226	757.1616303	89	Grand Index
30	f	13-16 years	0.227	757.7165774	89	Grand Index
30	f	13-16 years	0.23	758	89	Grand Index
30	f	13-16 years	0.231	758.2487765	89	Grand Index
30	f	13-16 years	0.235	759	89	Grand Index
30	f	13-16 years	0.238	760	89	Grand Index
30	f	13-16 years	0.239	760.3163717	89	Grand Index
30	f	13-16 years	0.246	761	90	Grand Index
30	f	13-16 years	0.247	761.9416327	90	Grand Index
30	f	13-16 years	0.252	762	90	Grand Index
30	f	13-16 years	0.253	763	90	Grand Index
30	f	13-16 years	0.254	763.008802	90	Grand Index
30	f	13-16 years	0.259	764	90	Grand Index
30	f	13-16 years	0.262	765	90	Grand Index
30	f	13-16 years	0.263	765.0184615	90	Grand Index
30	f	13-16 years	0.264	765.9209877	91	Grand Index
30	f	13-16 years	0.268	766	91	Grand Index
30	f	13-16 years	0.269	766.48	91	Grand Index
30	f	13-16 years	0.273	767	91	Grand Index
30	f	13-16 years	0.274	767.4500233	91	Grand Index
30	f	13-16 years	0.277	768	91	Grand Index
30	f	13-16 years	0.278	768.7454348	91	Grand Index
30	f	13-16 years	0.279	768.9895172	91	Grand Index
30	f	13-16 years	0.284	769	91	Grand Index
30	f	13-16 years	0.285	769.16875	91	Grand Index
30	f	13-16 years	0.288	770	92	Grand Index
30	f	13-16 years	0.289	770.0827586	92	Grand Index
30	f	13-16 years	0.294	771	92	Grand Index
30	f	13-16 years	0.295	771.7157895	92	Grand Index
30	f	13-16 years	0.302	772	92	Grand Index
30	f	13-16 years	0.303	772.0118227	92	Grand Index
30	f	13-16 years	0.308	773	92	Grand Index
30	f	13-16 years	0.309	773.7647059	93	Grand Index
30	f	13-16 years	0.311	774	93	Grand Index
30	f	13-16 years	0.312	774.0413793	93	Grand Index
30	f	13-16 years	0.316	775	93	Grand Index
30	f	13-16 years	0.317	775.6460011	93	Grand Index

Age	Gender	Education	Percentile	Raw Score	Norm Score	Assessment
30	f	13-16 years	0.319	776	93	Grand Index
30	f	13-16 years	0.322	777	93	Grand Index
30	f	13-16 years	0.323	777.2	93	Grand Index
30	f	13-16 years	0.326	778	93	Grand Index
30	f	13-16 years	0.327	778.8	93	Grand Index
30	f	13-16 years	0.332	779	93	Grand Index
30	f	13-16 years	0.335	780	94	Grand Index
30	f	13-16 years	0.336	780.0333333	94	Grand Index
30	f	13-16 years	0.337	781	94	Grand Index
30	f	13-16 years	0.338	781.1625	94	Grand Index
30	f	13-16 years	0.341	782	94	Grand Index
30	f	13-16 years	0.342	782.8	94	Grand Index
30	f	13-16 years	0.346	783	94	Grand Index
30	f	13-16 years	0.347	783.7093311	94	Grand Index
30	f	13-16 years	0.353	784	94	Grand Index
30	f	13-16 years	0.354	784.3818292	94	Grand Index
30	f	13-16 years	0.358	785	95	Grand Index
30	f	13-16 years	0.359	785.9235294	95	Grand Index
30	f	13-16 years	0.364	786	95	Grand Index
30	f	13-16 years	0.365	786.1176471	95	Grand Index
30	f	13-16 years	0.367	787	95	Grand Index
30	f	13-16 years	0.368	787.4221188	95	Grand Index
30	f	13-16 years	0.374	788	95	Grand Index
30	f	13-16 years	0.38	789	95	Grand Index
30	f	13-16 years	0.381	790	95	Grand Index
30	f	13-16 years	0.382	790.9724138	95	Grand Index
30	f	13-16 years	0.385	791	96	Grand Index
30	f	13-16 years	0.39	792	96	Grand Index
30	f	13-16 years	0.395	793	96	Grand Index
30	f	13-16 years	0.396	793.5882353	96	Grand Index
30	f	13-16 years	0.401	794	96	Grand Index
30	f	13-16 years	0.402	794.976	96	Grand Index
30	f	13-16 years	0.407	795	96	Grand Index
30	f	13-16 years	0.411	796	97	Grand Index
30	f	13-16 years	0.416	797	97	Grand Index
30	f	13-16 years	0.417	797.0957606	97	Grand Index
30	f	13-16 years	0.419	798	97	Grand Index
30	f	13-16 years	0.428	799	97	Grand Index
30	f	13-16 years	0.429	799.6245304	97	Grand Index
30	f	13-16 years	0.435	800	98	Grand Index
30	f	13-16 years	0.436	800.2013986	98	Grand Index
30	f	13-16 years	0.437	800.8631579	98	Grand Index
30	f	13-16 years	0.442	801	98	Grand Index
30	f	13-16 years	0.443	801.4983784	98	Grand Index
30	f	13-16 years	0.446	802	98	Grand Index
30	f	13-16 years	0.447	802.8	98	Grand Index
30	f	13-16 years	0.449	803	98	Grand Index
30	f	13-16 years	0.456	804	98	Grand Index
30	f	13-16 years	0.457	804.8	98	Grand Index
30	f	13-16 years	0.461	805	99	Grand Index
30	f	13-16 years	0.462	805.6916667	99	Grand Index
30	f	13-16 years	0.464	806	99	Grand Index
30	f	13-16 years	0.471	807	99	Grand Index
30	f	13-16 years	0.472	807.8	99	Grand Index
30	f	13-16 years	0.474	808	99	Grand Index
30	f	13-16 years	0.477	809	99	Grand Index

Age	Gender	Education	Percentile	Raw Score	Norm Score	Assessment
30	f	13-16 years	0.478	809.1524256	99	Grand Index
30	f	13-16 years	0.479	809.9080139	99	Grand Index
30	f	13-16 years	0.483	810	99	Grand Index
30	f	13-16 years	0.488	811	100	Grand Index
30	f	13-16 years	0.489	811.6	100	Grand Index
30	f	13-16 years	0.494	812	100	Grand Index
30	f	13-16 years	0.495	812.2252747	100	Grand Index
30	f	13-16 years	0.496	812.8615385	100	Grand Index
30	f	13-16 years	0.5	813	100	Grand Index
30	f	13-16 years	0.501	813.3505652	100	Grand Index
30	f	13-16 years	0.502	813.9966245	100	Grand Index
30	f	13-16 years	0.503	814	100	Grand Index
30	f	13-16 years	0.504	814.5312923	100	Grand Index
30	f	13-16 years	0.51	815	100	Grand Index
30	f	13-16 years	0.517	816	101	Grand Index
30	f	13-16 years	0.518	816.787289	101	Grand Index
30	f	13-16 years	0.522	817	101	Grand Index
30	f	13-16 years	0.523	817.0070796	101	Grand Index
30	f	13-16 years	0.529	818	101	Grand Index
30	f	13-16 years	0.532	819	101	Grand Index
30	f	13-16 years	0.533	819.3057426	101	Grand Index
30	f	13-16 years	0.537	820	101	Grand Index
30	f	13-16 years	0.545	821	102	Grand Index
30	f	13-16 years	0.546	821.4	102	Grand Index
30	f	13-16 years	0.551	822	102	Grand Index
30	f	13-16 years	0.552	822.6750299	102	Grand Index
30	f	13-16 years	0.555	823	102	Grand Index
30	f	13-16 years	0.556	823.2470524	102	Grand Index
30	f	13-16 years	0.557	823.9070838	102	Grand Index
30	f	13-16 years	0.561	824	102	Grand Index
30	f	13-16 years	0.562	824.2727825	102	Grand Index
30	f	13-16 years	0.567	825	103	Grand Index
30	f	13-16 years	0.568	825.0973004	103	Grand Index
30	f	13-16 years	0.573	826	103	Grand Index
30	f	13-16 years	0.574	826.8633452	103	Grand Index
30	f	13-16 years	0.579	827	103	Grand Index
30	f	13-16 years	0.58	827.9926471	103	Grand Index
30	f	13-16 years	0.585	828	103	Grand Index
30	f	13-16 years	0.586	828.3564806	103	Grand Index
30	f	13-16 years	0.591	829	103	Grand Index
30	f	13-16 years	0.592	829.8088608	103	Grand Index
30	f	13-16 years	0.598	830	104	Grand Index
30	f	13-16 years	0.599	830.5690729	104	Grand Index
30	f	13-16 years	0.602	831	104	Grand Index
30	f	13-16 years	0.603	831.2	104	Grand Index
30	f	13-16 years	0.607	832	104	Grand Index
30	f	13-16 years	0.608	832.0134328	104	Grand Index
30	f	13-16 years	0.609	832.8528	104	Grand Index
30	f	13-16 years	0.614	833	104	Grand Index
30	f	13-16 years	0.617	834	104	Grand Index
30	f	13-16 years	0.618	834.2	105	Grand Index
30	f	13-16 years	0.624	835	105	Grand Index
30	f	13-16 years	0.625	835.4615385	105	Grand Index
30	f	13-16 years	0.628	836	105	Grand Index
30	f	13-16 years	0.629	836.564	105	Grand Index
30	f	13-16 years	0.635	837	105	Grand Index

Age	Gender	Education	Percentile	Raw Score	Norm Score	Assessment
30	f	13-16 years	0.638	838	105	Grand Index
30	f	13-16 years	0.639	838.0827586	105	Grand Index
30	f	13-16 years	0.642	839	105	Grand Index
30	f	13-16 years	0.645	840	106	Grand Index
30	f	13-16 years	0.651	841	106	Grand Index
30	f	13-16 years	0.652	841.7438681	106	Grand Index
30	f	13-16 years	0.658	842	106	Grand Index
30	f	13-16 years	0.659	842.1344828	106	Grand Index
30	f	13-16 years	0.662	843	106	Grand Index
30	f	13-16 years	0.667	844	106	Grand Index
30	f	13-16 years	0.668	844.1925926	107	Grand Index
30	f	13-16 years	0.673	845	107	Grand Index
30	f	13-16 years	0.674	845.6223602	107	Grand Index
30	f	13-16 years	0.677	846	107	Grand Index
30	f	13-16 years	0.678	846.8530612	107	Grand Index
30	f	13-16 years	0.682	847	107	Grand Index
30	f	13-16 years	0.683	847.4666667	107	Grand Index
30	f	13-16 years	0.685	848	107	Grand Index
30	f	13-16 years	0.686	848.112	107	Grand Index
30	f	13-16 years	0.692	849	108	Grand Index
30	f	13-16 years	0.694	850	108	Grand Index
30	f	13-16 years	0.695	850.8736842	108	Grand Index
30	f	13-16 years	0.7	851	108	Grand Index
30	f	13-16 years	0.701	851.0421053	108	Grand Index
30	f	13-16 years	0.703	852	108	Grand Index
30	f	13-16 years	0.704	852.1972028	108	Grand Index
30	f	13-16 years	0.709	853	108	Grand Index
30	f	13-16 years	0.714	854	108	Grand Index
30	f	13-16 years	0.715	854.0590164	109	Grand Index
30	f	13-16 years	0.717	855	109	Grand Index
30	f	13-16 years	0.718	855.222739	109	Grand Index
30	f	13-16 years	0.723	856	109	Grand Index
30	f	13-16 years	0.724	856.9536232	109	Grand Index
30	f	13-16 years	0.726	857	109	Grand Index
30	f	13-16 years	0.727	858	109	Grand Index
30	f	13-16 years	0.728	858.0156522	109	Grand Index
30	f	13-16 years	0.729	858.253087	109	Grand Index
30	f	13-16 years	0.735	859	109	Grand Index
30	f	13-16 years	0.737	860	110	Grand Index
30	f	13-16 years	0.738	860.0055556	110	Grand Index
30	f	13-16 years	0.739	860.7594937	110	Grand Index
30	f	13-16 years	0.742	861	110	Grand Index
30	f	13-16 years	0.743	861.6345987	110	Grand Index
30	f	13-16 years	0.744	861.9417476	110	Grand Index
30	f	13-16 years	0.746	862	110	Grand Index
30	f	13-16 years	0.747	862.8	110	Grand Index
30	f	13-16 years	0.751	863	110	Grand Index
30	f	13-16 years	0.752	863.6774194	110	Grand Index
30	f	13-16 years	0.754	864	110	Grand Index
30	f	13-16 years	0.755	864.7647059	110	Grand Index
30	f	13-16 years	0.758	865	110	Grand Index
30	f	13-16 years	0.759	865.454251	111	Grand Index
30	f	13-16 years	0.762	866	111	Grand Index
30	f	13-16 years	0.763	866.7957447	111	Grand Index
30	f	13-16 years	0.766	867	111	Grand Index
30	f	13-16 years	0.771	868	111	Grand Index

Age	Gender	Education	Percentile	Raw Score	Norm Score	Assessment
30	f	13-16 years	0.773	869	111	Grand Index
30	f	13-16 years	0.774	869.1764919	111	Grand Index
30	f	13-16 years	0.777	870	111	Grand Index
30	f	13-16 years	0.778	870.0103448	111	Grand Index
30	f	13-16 years	0.779	870.9655172	112	Grand Index
30	f	13-16 years	0.78	871	112	Grand Index
30	f	13-16 years	0.781	871.4	112	Grand Index
30	f	13-16 years	0.785	872	112	Grand Index
30	f	13-16 years	0.786	872.5787122	112	Grand Index
30	f	13-16 years	0.788	873	112	Grand Index
30	f	13-16 years	0.789	873.6	112	Grand Index
30	f	13-16 years	0.792	874	112	Grand Index
30	f	13-16 years	0.795	875	112	Grand Index
30	f	13-16 years	0.796	876	112	Grand Index
30	f	13-16 years	0.797	876.7369831	112	Grand Index
30	f	13-16 years	0.802	877	113	Grand Index
30	f	13-16 years	0.803	877.1333333	113	Grand Index
30	f	13-16 years	0.804	877.9157895	113	Grand Index
30	f	13-16 years	0.805	878	113	Grand Index
30	f	13-16 years	0.806	878.3800766	113	Grand Index
30	f	13-16 years	0.808	879	113	Grand Index
30	f	13-16 years	0.809	879.048	113	Grand Index
30	f	13-16 years	0.811	880	113	Grand Index
30	f	13-16 years	0.812	880.4217054	113	Grand Index
30	f	13-16 years	0.813	881	113	Grand Index
30	f	13-16 years	0.814	881.3012236	113	Grand Index
30	f	13-16 years	0.815	882	113	Grand Index
30	f	13-16 years	0.816	882.4	114	Grand Index
30	f	13-16 years	0.819	883	114	Grand Index
30	f	13-16 years	0.82	883	114	Grand Index
30	f	13-16 years	0.821	883.7029703	114	Grand Index
30	f	13-16 years	0.824	884	114	Grand Index
30	f	13-16 years	0.827	885	114	Grand Index
30	f	13-16 years	0.828	885.84	114	Grand Index
30	f	13-16 years	0.83	886	114	Grand Index
30	f	13-16 years	0.831	886.3361144	114	Grand Index
30	f	13-16 years	0.834	887	115	Grand Index
30	f	13-16 years	0.836	888	115	Grand Index
30	f	13-16 years	0.837	888.2909091	115	Grand Index
30	f	13-16 years	0.838	889	115	Grand Index
30	f	13-16 years	0.842	890	115	Grand Index
30	f	13-16 years	0.845	891	115	Grand Index
30	f	13-16 years	0.849	892	115	Grand Index
30	f	13-16 years	0.853	893	116	Grand Index
30	f	13-16 years	0.854	893.0679245	116	Grand Index
30	f	13-16 years	0.857	894	116	Grand Index
30	f	13-16 years	0.858	894.4721649	116	Grand Index
30	f	13-16 years	0.86	895	116	Grand Index
30	f	13-16 years	0.861	895.1417943	116	Grand Index
30	f	13-16 years	0.862	895.7976124	116	Grand Index
30	f	13-16 years	0.865	896	117	Grand Index
30	f	13-16 years	0.866	896.1747573	117	Grand Index
30	f	13-16 years	0.867	896.8947368	117	Grand Index
30	f	13-16 years	0.868	897.004878	117	Grand Index
30	f	13-16 years	0.869	897.6527473	117	Grand Index
30	f	13-16 years	0.871	898	117	Grand Index

Age	Gender	Education	Percentile	Raw Score	Norm Score	Assessment
30	f	13-16 years	0.872	898.269183	117	Grand Index
30	f	13-16 years	0.874	899	117	Grand Index
30	f	13-16 years	0.875	899.5463918	117	Grand Index
30	f	13-16 years	0.877	900	117	Grand Index
30	f	13-16 years	0.879	901	118	Grand Index
30	f	13-16 years	0.883	902	118	Grand Index
30	f	13-16 years	0.884	902.6	118	Grand Index
30	f	13-16 years	0.885	903	118	Grand Index
30	f	13-16 years	0.887	904	118	Grand Index
30	f	13-16 years	0.888	905.2	118	Grand Index
30	f	13-16 years	0.89	906	118	Grand Index
30	f	13-16 years	0.891	906.95	118	Grand Index
30	f	13-16 years	0.894	907	119	Grand Index
30	f	13-16 years	0.896	908	119	Grand Index
30	f	13-16 years	0.897	908.1623188	119	Grand Index
30	f	13-16 years	0.898	909	119	Grand Index
30	f	13-16 years	0.899	909.3961165	119	Grand Index
30	f	13-16 years	0.9	910	119	Grand Index
30	f	13-16 years	0.902	911	119	Grand Index
30	f	13-16 years	0.904	912	120	Grand Index
30	f	13-16 years	0.905	913	120	Grand Index
30	f	13-16 years	0.906	913.1103448	120	Grand Index
30	f	13-16 years	0.909	914	120	Grand Index
30	f	13-16 years	0.911	915	120	Grand Index
30	f	13-16 years	0.914	916	120	Grand Index
30	f	13-16 years	0.916	917	121	Grand Index
30	f	13-16 years	0.917	917.9649123	121	Grand Index
30	f	13-16 years	0.918	918	121	Grand Index
30	f	13-16 years	0.919	918.6097561	121	Grand Index
30	f	13-16 years	0.92	919	121	Grand Index
30	f	13-16 years	0.921	919.607874	121	Grand Index
30	f	13-16 years	0.922	920	121	Grand Index
30	f	13-16 years	0.923	921.2342693	121	Grand Index
30	f	13-16 years	0.925	923	122	Grand Index
30	f	13-16 years	0.926	924	122	Grand Index
30	f	13-16 years	0.927	925	122	Grand Index
30	f	13-16 years	0.928	925.1565217	122	Grand Index
30	f	13-16 years	0.929	925.9140071	122	Grand Index
30	f	13-16 years	0.93	927	122	Grand Index
30	f	13-16 years	0.931	927.8091803	122	Grand Index
30	f	13-16 years	0.932	928.2	122	Grand Index
30	f	13-16 years	0.933	929	122	Grand Index
30	f	13-16 years	0.934	929.3057325	123	Grand Index
30	f	13-16 years	0.936	931	123	Grand Index
30	f	13-16 years	0.937	931.8	123	Grand Index
30	f	13-16 years	0.938	932	123	Grand Index
30	f	13-16 years	0.939	932.9711475	123	Grand Index
30	f	13-16 years	0.94	933	123	Grand Index
30	f	13-16 years	0.941	934	123	Grand Index
30	f	13-16 years	0.942	934.1333333	124	Grand Index
30	f	13-16 years	0.944	936	124	Grand Index
30	f	13-16 years	0.945	936.7814208	124	Grand Index
30	f	13-16 years	0.946	937	124	Grand Index
30	f	13-16 years	0.947	937.7951807	124	Grand Index
30	f	13-16 years	0.948	938.2	124	Grand Index
30	f	13-16 years	0.95	939	125	Grand Index

Age	Gender	Education	Percentile	Raw Score	Norm Score	Assessment
30	f	13-16 years	0.951	940	125	Grand Index
30	f	13-16 years	0.952	940.8792818	125	Grand Index
30	f	13-16 years	0.953	941.0518519	125	Grand Index
30	f	13-16 years	0.954	941.3221496	125	Grand Index
30	f	13-16 years	0.955	942	125	Grand Index
30	f	13-16 years	0.956	943.3309601	126	Grand Index
30	f	13-16 years	0.957	944.8333333	126	Grand Index
30	f	13-16 years	0.958	946	126	Grand Index
30	f	13-16 years	0.959	946.7475541	126	Grand Index
30	f	13-16 years	0.96	948.4736842	126	Grand Index
30	f	13-16 years	0.961	949.6629213	126	Grand Index
30	f	13-16 years	0.962	950	127	Grand Index
30	f	13-16 years	0.963	950.2	127	Grand Index
30	f	13-16 years	0.964	951	127	Grand Index
30	f	13-16 years	0.966	952	127	Grand Index
30	f	13-16 years	0.967	952.8947368	128	Grand Index
30	f	13-16 years	0.968	954.1586207	128	Grand Index
30	f	13-16 years	0.969	955.6	128	Grand Index
30	f	13-16 years	0.97	956.4808743	128	Grand Index
30	f	13-16 years	0.971	958.1142857	128	Grand Index
30	f	13-16 years	0.972	959	129	Grand Index
30	f	13-16 years	0.973	960	129	Grand Index
30	f	13-16 years	0.974	960.6087591	129	Grand Index
30	f	13-16 years	0.975	962	129	Grand Index
30	f	13-16 years	0.976	964	130	Grand Index
30	f	13-16 years	0.977	964.5449393	130	Grand Index
30	f	13-16 years	0.978	965.6612491	130	Grand Index
30	f	13-16 years	0.979	967.1777215	131	Grand Index
30	f	13-16 years	0.98	968	131	Grand Index
30	f	13-16 years	0.981	969	131	Grand Index
30	f	13-16 years	0.982	970.8	131	Grand Index
30	f	13-16 years	0.983	971.0463527	132	Grand Index
30	f	13-16 years	0.984	972.6	132	Grand Index
30	f	13-16 years	0.985	974.0295638	133	Grand Index
30	f	13-16 years	0.986	977.1	133	Grand Index
30	f	13-16 years	0.987	982	133	Grand Index
30	f	13-16 years	0.988	982.7206897	134	Grand Index
30	f	13-16 years	0.989	985.1209122	134	Grand Index
30	f	13-16 years	0.99	988	135	Grand Index
30	f	13-16 years	0.991	989	135	Grand Index
30	f	13-16 years	0.992	990.1520238	136	Grand Index
30	f	13-16 years	0.993	993.0873093	137	Grand Index
30	f	13-16 years	0.994	1008.416339	138	Grand Index
30	f	13-16 years	0.995	1013.75	139	Grand Index
30	f	13-16 years	0.996	1020.906762	140	Grand Index
30	f	13-16 years	0.997	1025.363043	141	Grand Index
30	f	13-16 years	0.998	1035.322741	143	Grand Index
30	f	13-16 years	0.999	1046.002812	146	Grand Index
30	m	13-16 years	0.001	579.5960681	54	Grand Index
30	m	13-16 years	0.002	595	57	Grand Index
30	m	13-16 years	0.003	597.1476985	59	Grand Index
30	m	13-16 years	0.004	605.3937175	60	Grand Index
30	m	13-16 years	0.005	612.1510307	61	Grand Index
30	m	13-16 years	0.006	623.4089319	62	Grand Index
30	m	13-16 years	0.007	626.0916916	63	Grand Index
30	m	13-16 years	0.008	631.1116311	64	Grand Index

Age	Gender	Education	Percentile	Raw Score	Norm Score	Assessment
30	m	13-16 years	0.009	633.476	65	Grand Index
30	m	13-16 years	0.01	635	65	Grand Index
30	m	13-16 years	0.011	638.608	66	Grand Index
30	m	13-16 years	0.012	641	66	Grand Index
30	m	13-16 years	0.013	642.062096	67	Grand Index
30	m	13-16 years	0.014	643.8556227	67	Grand Index
30	m	13-16 years	0.015	645	67	Grand Index
30	m	13-16 years	0.016	646.4606125	68	Grand Index
30	m	13-16 years	0.017	648.7255504	68	Grand Index
30	m	13-16 years	0.018	650.7303461	69	Grand Index
30	m	13-16 years	0.019	652	69	Grand Index
30	m	13-16 years	0.02	653.3460589	69	Grand Index
30	m	13-16 years	0.021	658	69	Grand Index
30	m	13-16 years	0.022	659	70	Grand Index
30	m	13-16 years	0.023	660.566732	70	Grand Index
30	m	13-16 years	0.024	661.6810404	70	Grand Index
30	m	13-16 years	0.025	662.2096507	71	Grand Index
30	m	13-16 years	0.027	666	71	Grand Index
30	m	13-16 years	0.028	666.4947383	71	Grand Index
30	m	13-16 years	0.029	667	72	Grand Index
30	m	13-16 years	0.03	667.9742091	72	Grand Index
30	m	13-16 years	0.031	668.1393391	72	Grand Index
30	m	13-16 years	0.032	669	72	Grand Index
30	m	13-16 years	0.033	669.4937787	72	Grand Index
30	m	13-16 years	0.034	670	73	Grand Index
30	m	13-16 years	0.035	670.1160963	73	Grand Index
30	m	13-16 years	0.036	671	73	Grand Index
30	m	13-16 years	0.037	671.5953403	73	Grand Index
30	m	13-16 years	0.038	673	73	Grand Index
30	m	13-16 years	0.039	673.8091119	74	Grand Index
30	m	13-16 years	0.04	674	74	Grand Index
30	m	13-16 years	0.041	674.9984982	74	Grand Index
30	m	13-16 years	0.042	676.1535878	74	Grand Index
30	m	13-16 years	0.043	678	74	Grand Index
30	m	13-16 years	0.044	678.7529776	74	Grand Index
30	m	13-16 years	0.045	679.38	75	Grand Index
30	m	13-16 years	0.046	680	75	Grand Index
30	m	13-16 years	0.048	681	75	Grand Index
30	m	13-16 years	0.049	682	75	Grand Index
30	m	13-16 years	0.05	682.5511263	75	Grand Index
30	m	13-16 years	0.051	683	75	Grand Index
30	m	13-16 years	0.052	683.7366835	76	Grand Index
30	m	13-16 years	0.053	684.7883681	76	Grand Index
30	m	13-16 years	0.054	685	76	Grand Index
30	m	13-16 years	0.055	686.02	76	Grand Index
30	m	13-16 years	0.056	687.5860323	76	Grand Index
30	m	13-16 years	0.058	688	76	Grand Index
30	m	13-16 years	0.059	688.7768609	77	Grand Index
30	m	13-16 years	0.06	689.1154984	77	Grand Index
30	m	13-16 years	0.061	690.9119775	77	Grand Index
30	m	13-16 years	0.062	691	77	Grand Index
30	m	13-16 years	0.063	691.1960586	77	Grand Index
30	m	13-16 years	0.064	692	77	Grand Index
30	m	13-16 years	0.065	692.9433832	77	Grand Index
30	m	13-16 years	0.066	693	77	Grand Index
30	m	13-16 years	0.067	693.988	78	Grand Index

Age	Gender	Education	Percentile	Raw Score	Norm Score	Assessment
30	m	13-16 years	0.068	694.152	78	Grand Index
30	m	13-16 years	0.069	695	78	Grand Index
30	m	13-16 years	0.07	695.7990863	78	Grand Index
30	m	13-16 years	0.071	696.8370314	78	Grand Index
30	m	13-16 years	0.072	697	78	Grand Index
30	m	13-16 years	0.073	698	78	Grand Index
30	m	13-16 years	0.075	699	78	Grand Index
30	m	13-16 years	0.076	699.3000728	79	Grand Index
30	m	13-16 years	0.077	700	79	Grand Index
30	m	13-16 years	0.079	702	79	Grand Index
30	m	13-16 years	0.08	703	79	Grand Index
30	m	13-16 years	0.081	703.216648	79	Grand Index
30	m	13-16 years	0.083	704	79	Grand Index
30	m	13-16 years	0.084	704.0862647	79	Grand Index
30	m	13-16 years	0.086	705	80	Grand Index
30	m	13-16 years	0.088	706	80	Grand Index
30	m	13-16 years	0.089	706.596	80	Grand Index
30	m	13-16 years	0.09	707	80	Grand Index
30	m	13-16 years	0.091	707.0035593	80	Grand Index
30	m	13-16 years	0.092	708	80	Grand Index
30	m	13-16 years	0.094	709	80	Grand Index
30	m	13-16 years	0.096	710	80	Grand Index
30	m	13-16 years	0.097	710.9404383	81	Grand Index
30	m	13-16 years	0.099	711	81	Grand Index
30	m	13-16 years	0.1	711.4	81	Grand Index
30	m	13-16 years	0.101	712.4757426	81	Grand Index
30	m	13-16 years	0.102	713	81	Grand Index
30	m	13-16 years	0.103	714	81	Grand Index
30	m	13-16 years	0.104	714.0008029	81	Grand Index
30	m	13-16 years	0.105	714.6548702	81	Grand Index
30	m	13-16 years	0.107	715	81	Grand Index
30	m	13-16 years	0.108	715.9117217	81	Grand Index
30	m	13-16 years	0.109	716	82	Grand Index
30	m	13-16 years	0.11	716.0175531	82	Grand Index
30	m	13-16 years	0.112	717	82	Grand Index
30	m	13-16 years	0.114	718	82	Grand Index
30	m	13-16 years	0.115	718.9401907	82	Grand Index
30	m	13-16 years	0.116	719	82	Grand Index
30	m	13-16 years	0.117	719.0365408	82	Grand Index
30	m	13-16 years	0.119	720	82	Grand Index
30	m	13-16 years	0.12	720.728384	82	Grand Index
30	m	13-16 years	0.122	721	83	Grand Index
30	m	13-16 years	0.124	722	83	Grand Index
30	m	13-16 years	0.126	723	83	Grand Index
30	m	13-16 years	0.127	723.0668157	83	Grand Index
30	m	13-16 years	0.129	724	83	Grand Index
30	m	13-16 years	0.13	724.0208986	83	Grand Index
30	m	13-16 years	0.131	724.5524894	83	Grand Index
30	m	13-16 years	0.133	725	83	Grand Index
30	m	13-16 years	0.134	725.9802254	83	Grand Index
30	m	13-16 years	0.136	726	84	Grand Index
30	m	13-16 years	0.139	727	84	Grand Index
30	m	13-16 years	0.14	727.9933194	84	Grand Index
30	m	13-16 years	0.142	728	84	Grand Index
30	m	13-16 years	0.143	728.8564576	84	Grand Index
30	m	13-16 years	0.144	729	84	Grand Index

Age	Gender	Education	Percentile	Raw Score	Norm Score	Assessment
30	m	13-16 years	0.145	729.78	84	Grand Index
30	m	13-16 years	0.147	730	84	Grand Index
30	m	13-16 years	0.148	730.9965804	84	Grand Index
30	m	13-16 years	0.15	731	84	Grand Index
30	m	13-16 years	0.151	731.3752072	85	Grand Index
30	m	13-16 years	0.154	732	85	Grand Index
30	m	13-16 years	0.155	732.2104759	85	Grand Index
30	m	13-16 years	0.159	733	85	Grand Index
30	m	13-16 years	0.16	733.5654598	85	Grand Index
30	m	13-16 years	0.162	734	85	Grand Index
30	m	13-16 years	0.164	735	85	Grand Index
30	m	13-16 years	0.168	736	86	Grand Index
30	m	13-16 years	0.169	736.2937397	86	Grand Index
30	m	13-16 years	0.17	737	86	Grand Index
30	m	13-16 years	0.171	737.0001951	86	Grand Index
30	m	13-16 years	0.174	738	86	Grand Index
30	m	13-16 years	0.177	739	86	Grand Index
30	m	13-16 years	0.178	739.8182986	86	Grand Index
30	m	13-16 years	0.181	740	86	Grand Index
30	m	13-16 years	0.183	741	86	Grand Index
30	m	13-16 years	0.184	741.0143681	86	Grand Index
30	m	13-16 years	0.187	742	87	Grand Index
30	m	13-16 years	0.19	743	87	Grand Index
30	m	13-16 years	0.191	743.6519782	87	Grand Index
30	m	13-16 years	0.193	744	87	Grand Index
30	m	13-16 years	0.194	744.6142859	87	Grand Index
30	m	13-16 years	0.195	745	87	Grand Index
30	m	13-16 years	0.196	745.144	87	Grand Index
30	m	13-16 years	0.199	746	87	Grand Index
30	m	13-16 years	0.201	747	87	Grand Index
30	m	13-16 years	0.204	748	88	Grand Index
30	m	13-16 years	0.205	748.5179955	88	Grand Index
30	m	13-16 years	0.208	749	88	Grand Index
30	m	13-16 years	0.211	750	88	Grand Index
30	m	13-16 years	0.212	750.768	88	Grand Index
30	m	13-16 years	0.214	751	88	Grand Index
30	m	13-16 years	0.215	751.26	88	Grand Index
30	m	13-16 years	0.218	752	88	Grand Index
30	m	13-16 years	0.222	753	89	Grand Index
30	m	13-16 years	0.223	753.9947874	89	Grand Index
30	m	13-16 years	0.226	754	89	Grand Index
30	m	13-16 years	0.227	754.9396671	89	Grand Index
30	m	13-16 years	0.231	755	89	Grand Index
30	m	13-16 years	0.233	756	89	Grand Index
30	m	13-16 years	0.234	756.1092158	89	Grand Index
30	m	13-16 years	0.235	756.8770723	89	Grand Index
30	m	13-16 years	0.237	757	89	Grand Index
30	m	13-16 years	0.24	758	89	Grand Index
30	m	13-16 years	0.241	758.1373205	89	Grand Index
30	m	13-16 years	0.242	758.9297439	90	Grand Index
30	m	13-16 years	0.245	759	90	Grand Index
30	m	13-16 years	0.246	760	90	Grand Index
30	m	13-16 years	0.247	760.0347459	90	Grand Index
30	m	13-16 years	0.248	760.4003905	90	Grand Index
30	m	13-16 years	0.253	761	90	Grand Index
30	m	13-16 years	0.254	761.9137572	90	Grand Index

Age	Gender	Education	Percentile	Raw Score	Norm Score	Assessment
30	m	13-16 years	0.256	762	90	Grand Index
30	m	13-16 years	0.257	762.7402439	90	Grand Index
30	m	13-16 years	0.26	763	90	Grand Index
30	m	13-16 years	0.261	763.804	90	Grand Index
30	m	13-16 years	0.264	764	91	Grand Index
30	m	13-16 years	0.265	764.4710607	91	Grand Index
30	m	13-16 years	0.268	765	91	Grand Index
30	m	13-16 years	0.269	765.2915431	91	Grand Index
30	m	13-16 years	0.272	766	91	Grand Index
30	m	13-16 years	0.273	766.9886414	91	Grand Index
30	m	13-16 years	0.276	767	91	Grand Index
30	m	13-16 years	0.277	767.9670043	91	Grand Index
30	m	13-16 years	0.283	768	91	Grand Index
30	m	13-16 years	0.285	769	91	Grand Index
30	m	13-16 years	0.286	769.4412494	92	Grand Index
30	m	13-16 years	0.289	770	92	Grand Index
30	m	13-16 years	0.29	770.5520067	92	Grand Index
30	m	13-16 years	0.293	771	92	Grand Index
30	m	13-16 years	0.294	771.9256196	92	Grand Index
30	m	13-16 years	0.296	772	92	Grand Index
30	m	13-16 years	0.297	772.8604698	92	Grand Index
30	m	13-16 years	0.301	773	92	Grand Index
30	m	13-16 years	0.304	774	92	Grand Index
30	m	13-16 years	0.305	774.0093971	92	Grand Index
30	m	13-16 years	0.307	775	92	Grand Index
30	m	13-16 years	0.308	775.3067555	92	Grand Index
30	m	13-16 years	0.309	775.9723501	93	Grand Index
30	m	13-16 years	0.312	776	93	Grand Index
30	m	13-16 years	0.315	777	93	Grand Index
30	m	13-16 years	0.316	777.4072869	93	Grand Index
30	m	13-16 years	0.32	778	93	Grand Index
30	m	13-16 years	0.321	778.4271738	93	Grand Index
30	m	13-16 years	0.324	779	93	Grand Index
30	m	13-16 years	0.325	779.2098421	93	Grand Index
30	m	13-16 years	0.33	780	93	Grand Index
30	m	13-16 years	0.331	780.2504511	93	Grand Index
30	m	13-16 years	0.335	781	94	Grand Index
30	m	13-16 years	0.336	781.3639134	94	Grand Index
30	m	13-16 years	0.341	782	94	Grand Index
30	m	13-16 years	0.347	783	94	Grand Index
30	m	13-16 years	0.348	783.369534	94	Grand Index
30	m	13-16 years	0.352	784	94	Grand Index
30	m	13-16 years	0.353	784.7847012	94	Grand Index
30	m	13-16 years	0.358	785	95	Grand Index
30	m	13-16 years	0.36	786	95	Grand Index
30	m	13-16 years	0.361	786.1528387	95	Grand Index
30	m	13-16 years	0.365	787	95	Grand Index
30	m	13-16 years	0.366	787.2491603	95	Grand Index
30	m	13-16 years	0.367	787.9258831	95	Grand Index
30	m	13-16 years	0.372	788	95	Grand Index
30	m	13-16 years	0.377	789	95	Grand Index
30	m	13-16 years	0.378	789.4881312	95	Grand Index
30	m	13-16 years	0.382	790	95	Grand Index
30	m	13-16 years	0.385	791	96	Grand Index
30	m	13-16 years	0.386	791.200423	96	Grand Index
30	m	13-16 years	0.391	792	96	Grand Index

Age	Gender	Education	Percentile	Raw Score	Norm Score	Assessment
30	m	13-16 years	0.392	792.2814394	96	Grand Index
30	m	13-16 years	0.396	793	96	Grand Index
30	m	13-16 years	0.397	793.108	96	Grand Index
30	m	13-16 years	0.403	794	96	Grand Index
30	m	13-16 years	0.404	794.2362962	96	Grand Index
30	m	13-16 years	0.409	795	97	Grand Index
30	m	13-16 years	0.41	795.7538952	97	Grand Index
30	m	13-16 years	0.414	796	97	Grand Index
30	m	13-16 years	0.415	796.06	97	Grand Index
30	m	13-16 years	0.419	797	97	Grand Index
30	m	13-16 years	0.42	797.8425555	97	Grand Index
30	m	13-16 years	0.423	798	97	Grand Index
30	m	13-16 years	0.424	798.2317582	97	Grand Index
30	m	13-16 years	0.425	798.7775962	97	Grand Index
30	m	13-16 years	0.428	799	97	Grand Index
30	m	13-16 years	0.429	799.356	97	Grand Index
30	m	13-16 years	0.431	800	97	Grand Index
30	m	13-16 years	0.435	801	98	Grand Index
30	m	13-16 years	0.441	802	98	Grand Index
30	m	13-16 years	0.442	802.189151	98	Grand Index
30	m	13-16 years	0.445	803	98	Grand Index
30	m	13-16 years	0.446	803.4243891	98	Grand Index
30	m	13-16 years	0.45	804	98	Grand Index
30	m	13-16 years	0.451	804.0802745	98	Grand Index
30	m	13-16 years	0.456	805	98	Grand Index
30	m	13-16 years	0.457	805.9243655	98	Grand Index
30	m	13-16 years	0.461	806	99	Grand Index
30	m	13-16 years	0.466	807	99	Grand Index
30	m	13-16 years	0.467	807.7904017	99	Grand Index
30	m	13-16 years	0.473	808	99	Grand Index
30	m	13-16 years	0.474	808.0537734	99	Grand Index
30	m	13-16 years	0.478	809	99	Grand Index
30	m	13-16 years	0.479	809.2363392	99	Grand Index
30	m	13-16 years	0.483	810	99	Grand Index
30	m	13-16 years	0.484	810.9441255	99	Grand Index
30	m	13-16 years	0.487	811	100	Grand Index
30	m	13-16 years	0.488	811.208847	100	Grand Index
30	m	13-16 years	0.494	812	100	Grand Index
30	m	13-16 years	0.495	812.1867637	100	Grand Index
30	m	13-16 years	0.499	813	100	Grand Index
30	m	13-16 years	0.5	813.4142994	100	Grand Index
30	m	13-16 years	0.507	814	100	Grand Index
30	m	13-16 years	0.508	814.6174966	100	Grand Index
30	m	13-16 years	0.512	815	100	Grand Index
30	m	13-16 years	0.513	815.6182692	100	Grand Index
30	m	13-16 years	0.519	816	101	Grand Index
30	m	13-16 years	0.52	816.3195505	101	Grand Index
30	m	13-16 years	0.526	817	101	Grand Index
30	m	13-16 years	0.53	818	101	Grand Index
30	m	13-16 years	0.535	819	101	Grand Index
30	m	13-16 years	0.536	819.7843218	101	Grand Index
30	m	13-16 years	0.539	820	101	Grand Index
30	m	13-16 years	0.54	820.1309464	102	Grand Index
30	m	13-16 years	0.541	820.9951505	102	Grand Index
30	m	13-16 years	0.545	821	102	Grand Index
30	m	13-16 years	0.546	821.374129	102	Grand Index

Age	Gender	Education	Percentile	Raw Score	Norm Score	Assessment
30	m	13-16 years	0.55	822	102	Grand Index
30	m	13-16 years	0.551	822.1148127	102	Grand Index
30	m	13-16 years	0.556	823	102	Grand Index
30	m	13-16 years	0.557	823.6967683	102	Grand Index
30	m	13-16 years	0.56	824	102	Grand Index
30	m	13-16 years	0.561	824.6991975	102	Grand Index
30	m	13-16 years	0.565	825	102	Grand Index
30	m	13-16 years	0.566	825.9551941	102	Grand Index
30	m	13-16 years	0.57	826	103	Grand Index
30	m	13-16 years	0.571	826.9891546	103	Grand Index
30	m	13-16 years	0.574	827	103	Grand Index
30	m	13-16 years	0.577	828	103	Grand Index
30	m	13-16 years	0.578	828.9306667	103	Grand Index
30	m	13-16 years	0.582	829	103	Grand Index
30	m	13-16 years	0.583	829.6724889	103	Grand Index
30	m	13-16 years	0.588	830	103	Grand Index
30	m	13-16 years	0.589	830.3810791	103	Grand Index
30	m	13-16 years	0.59	830.8699684	103	Grand Index
30	m	13-16 years	0.592	831	103	Grand Index
30	m	13-16 years	0.593	831.4337039	104	Grand Index
30	m	13-16 years	0.594	831.9434683	104	Grand Index
30	m	13-16 years	0.597	832	104	Grand Index
30	m	13-16 years	0.598	832.2502954	104	Grand Index
30	m	13-16 years	0.603	833	104	Grand Index
30	m	13-16 years	0.604	833.0445522	104	Grand Index
30	m	13-16 years	0.606	834	104	Grand Index
30	m	13-16 years	0.607	834.9321351	104	Grand Index
30	m	13-16 years	0.609	835	104	Grand Index
30	m	13-16 years	0.61	835.04	104	Grand Index
30	m	13-16 years	0.612	836	104	Grand Index
30	m	13-16 years	0.613	836.3683894	104	Grand Index
30	m	13-16 years	0.617	837	104	Grand Index
30	m	13-16 years	0.618	837.352	105	Grand Index
30	m	13-16 years	0.62	838	105	Grand Index
30	m	13-16 years	0.626	839	105	Grand Index
30	m	13-16 years	0.629	840	105	Grand Index
30	m	13-16 years	0.63	840.205697	105	Grand Index
30	m	13-16 years	0.633	841	105	Grand Index
30	m	13-16 years	0.634	841.9762313	105	Grand Index
30	m	13-16 years	0.637	842	105	Grand Index
30	m	13-16 years	0.638	842.6053129	105	Grand Index
30	m	13-16 years	0.642	843	105	Grand Index
30	m	13-16 years	0.643	843.3403303	105	Grand Index
30	m	13-16 years	0.644	843.8301491	106	Grand Index
30	m	13-16 years	0.648	844	106	Grand Index
30	m	13-16 years	0.649	844.0895286	106	Grand Index
30	m	13-16 years	0.652	845	106	Grand Index
30	m	13-16 years	0.657	846	106	Grand Index
30	m	13-16 years	0.66	847	106	Grand Index
30	m	13-16 years	0.661	847.9293032	106	Grand Index
30	m	13-16 years	0.662	848	106	Grand Index
30	m	13-16 years	0.663	848.0571928	106	Grand Index
30	m	13-16 years	0.667	849	106	Grand Index
30	m	13-16 years	0.668	849.1147344	107	Grand Index
30	m	13-16 years	0.669	849.9394555	107	Grand Index
30	m	13-16 years	0.674	850	107	Grand Index

Age	Gender	Education	Percentile	Raw Score	Norm Score	Assessment
30	m	13-16 years	0.675	850.9537175	107	Grand Index
30	m	13-16 years	0.678	851	107	Grand Index
30	m	13-16 years	0.679	851.2629816	107	Grand Index
30	m	13-16 years	0.682	852	107	Grand Index
30	m	13-16 years	0.683	852.0032103	107	Grand Index
30	m	13-16 years	0.687	853	107	Grand Index
30	m	13-16 years	0.688	853.832	107	Grand Index
30	m	13-16 years	0.689	854	107	Grand Index
30	m	13-16 years	0.69	854.0970651	107	Grand Index
30	m	13-16 years	0.691	854.9349932	107	Grand Index
30	m	13-16 years	0.695	855	108	Grand Index
30	m	13-16 years	0.7	856	108	Grand Index
30	m	13-16 years	0.706	857	108	Grand Index
30	m	13-16 years	0.707	857.9916244	108	Grand Index
30	m	13-16 years	0.711	858	108	Grand Index
30	m	13-16 years	0.714	859	108	Grand Index
30	m	13-16 years	0.715	859.6490676	109	Grand Index
30	m	13-16 years	0.721	860	109	Grand Index
30	m	13-16 years	0.726	861	109	Grand Index
30	m	13-16 years	0.727	861.0281912	109	Grand Index
30	m	13-16 years	0.73	862	109	Grand Index
30	m	13-16 years	0.731	862.890122	109	Grand Index
30	m	13-16 years	0.736	863	109	Grand Index
30	m	13-16 years	0.74	864	110	Grand Index
30	m	13-16 years	0.741	864.9527148	110	Grand Index
30	m	13-16 years	0.743	865	110	Grand Index
30	m	13-16 years	0.744	865.0028051	110	Grand Index
30	m	13-16 years	0.745	865.7194414	110	Grand Index
30	m	13-16 years	0.75	866	110	Grand Index
30	m	13-16 years	0.751	866.5599161	110	Grand Index
30	m	13-16 years	0.756	867	110	Grand Index
30	m	13-16 years	0.757	867.2460858	110	Grand Index
30	m	13-16 years	0.759	868	111	Grand Index
30	m	13-16 years	0.76	868.0665233	111	Grand Index
30	m	13-16 years	0.763	869	111	Grand Index
30	m	13-16 years	0.764	869.2365041	111	Grand Index
30	m	13-16 years	0.768	870	111	Grand Index
30	m	13-16 years	0.769	870.7191127	111	Grand Index
30	m	13-16 years	0.773	871	111	Grand Index
30	m	13-16 years	0.774	871.7964642	111	Grand Index
30	m	13-16 years	0.778	872	111	Grand Index
30	m	13-16 years	0.779	872.756	112	Grand Index
30	m	13-16 years	0.783	873	112	Grand Index
30	m	13-16 years	0.784	873.576	112	Grand Index
30	m	13-16 years	0.786	874	112	Grand Index
30	m	13-16 years	0.787	874.7500383	112	Grand Index
30	m	13-16 years	0.789	875	112	Grand Index
30	m	13-16 years	0.79	875.1585495	112	Grand Index
30	m	13-16 years	0.791	875.925977	112	Grand Index
30	m	13-16 years	0.794	876	112	Grand Index
30	m	13-16 years	0.797	877	112	Grand Index
30	m	13-16 years	0.8	878	113	Grand Index
30	m	13-16 years	0.801	878.4176214	113	Grand Index
30	m	13-16 years	0.803	879	113	Grand Index
30	m	13-16 years	0.804	879.0395077	113	Grand Index
30	m	13-16 years	0.805	879.8407315	113	Grand Index

Age	Gender	Education	Percentile	Raw Score	Norm Score	Assessment
30	m	13-16 years	0.807	880	113	Grand Index
30	m	13-16 years	0.808	880.189594	113	Grand Index
30	m	13-16 years	0.812	881	113	Grand Index
30	m	13-16 years	0.813	881.6226502	113	Grand Index
30	m	13-16 years	0.815	882	113	Grand Index
30	m	13-16 years	0.816	882.2924881	114	Grand Index
30	m	13-16 years	0.818	883	114	Grand Index
30	m	13-16 years	0.819	883.316	114	Grand Index
30	m	13-16 years	0.822	884	114	Grand Index
30	m	13-16 years	0.826	885	114	Grand Index
30	m	13-16 years	0.827	885.9713416	114	Grand Index
30	m	13-16 years	0.83	886	114	Grand Index
30	m	13-16 years	0.831	886.1322603	114	Grand Index
30	m	13-16 years	0.833	887	114	Grand Index
30	m	13-16 years	0.834	887.776	115	Grand Index
30	m	13-16 years	0.835	888	115	Grand Index
30	m	13-16 years	0.836	888.0077925	115	Grand Index
30	m	13-16 years	0.84	889	115	Grand Index
30	m	13-16 years	0.843	890	115	Grand Index
30	m	13-16 years	0.845	891	115	Grand Index
30	m	13-16 years	0.848	892	115	Grand Index
30	m	13-16 years	0.849	892.2234683	115	Grand Index
30	m	13-16 years	0.852	893	116	Grand Index
30	m	13-16 years	0.853	893.3032822	116	Grand Index
30	m	13-16 years	0.856	894	116	Grand Index
30	m	13-16 years	0.857	894.736684	116	Grand Index
30	m	13-16 years	0.859	895	116	Grand Index
30	m	13-16 years	0.86	895.9030075	116	Grand Index
30	m	13-16 years	0.862	896	116	Grand Index
30	m	13-16 years	0.863	896.2356272	116	Grand Index
30	m	13-16 years	0.864	896.9280492	116	Grand Index
30	m	13-16 years	0.865	897	117	Grand Index
30	m	13-16 years	0.866	897.1332033	117	Grand Index
30	m	13-16 years	0.868	898	117	Grand Index
30	m	13-16 years	0.869	898.6555228	117	Grand Index
30	m	13-16 years	0.87	899	117	Grand Index
30	m	13-16 years	0.871	900	117	Grand Index
30	m	13-16 years	0.872	900.0011731	117	Grand Index
30	m	13-16 years	0.874	901	117	Grand Index
30	m	13-16 years	0.875	901.2359743	117	Grand Index
30	m	13-16 years	0.878	902	117	Grand Index
30	m	13-16 years	0.879	902.5675958	118	Grand Index
30	m	13-16 years	0.882	903	118	Grand Index
30	m	13-16 years	0.883	903.9752492	118	Grand Index
30	m	13-16 years	0.884	904	118	Grand Index
30	m	13-16 years	0.885	904.0194709	118	Grand Index
30	m	13-16 years	0.888	905	118	Grand Index
30	m	13-16 years	0.889	905.8860315	118	Grand Index
30	m	13-16 years	0.891	906	118	Grand Index
30	m	13-16 years	0.892	907	119	Grand Index
30	m	13-16 years	0.893	907.0251429	119	Grand Index
30	m	13-16 years	0.895	908	119	Grand Index
30	m	13-16 years	0.896	908.5864242	119	Grand Index
30	m	13-16 years	0.897	909.0378962	119	Grand Index
30	m	13-16 years	0.899	910	119	Grand Index
30	m	13-16 years	0.9	911	119	Grand Index

Age	Gender	Education	Percentile	Raw Score	Norm Score	Assessment
30	m	13-16 years	0.901	912	119	Grand Index
30	m	13-16 years	0.902	912.928	119	Grand Index
30	m	13-16 years	0.904	914	120	Grand Index
30	m	13-16 years	0.907	915	120	Grand Index
30	m	13-16 years	0.909	916	120	Grand Index
30	m	13-16 years	0.91	916.5743299	120	Grand Index
30	m	13-16 years	0.911	917	120	Grand Index
30	m	13-16 years	0.912	918	120	Grand Index
30	m	13-16 years	0.913	918.081924	120	Grand Index
30	m	13-16 years	0.915	919	121	Grand Index
30	m	13-16 years	0.916	920	121	Grand Index
30	m	13-16 years	0.917	920.0863458	121	Grand Index
30	m	13-16 years	0.918	921.5319728	121	Grand Index
30	m	13-16 years	0.92	922	121	Grand Index
30	m	13-16 years	0.921	922.223568	121	Grand Index
30	m	13-16 years	0.922	922.8293389	121	Grand Index
30	m	13-16 years	0.925	924	122	Grand Index
30	m	13-16 years	0.926	925.8975218	122	Grand Index
30	m	13-16 years	0.927	927	122	Grand Index
30	m	13-16 years	0.928	927.8680547	122	Grand Index
30	m	13-16 years	0.93	928	122	Grand Index
30	m	13-16 years	0.931	929	122	Grand Index
30	m	13-16 years	0.933	930	122	Grand Index
30	m	13-16 years	0.934	930.6878788	123	Grand Index
30	m	13-16 years	0.935	931	123	Grand Index
30	m	13-16 years	0.936	931.2145018	123	Grand Index
30	m	13-16 years	0.937	932	123	Grand Index
30	m	13-16 years	0.938	932.9862249	123	Grand Index
30	m	13-16 years	0.939	933	123	Grand Index
30	m	13-16 years	0.94	933.0074616	123	Grand Index
30	m	13-16 years	0.941	934	123	Grand Index
30	m	13-16 years	0.942	934.5463496	124	Grand Index
30	m	13-16 years	0.943	935	124	Grand Index
30	m	13-16 years	0.944	935.2762249	124	Grand Index
30	m	13-16 years	0.945	936	124	Grand Index
30	m	13-16 years	0.946	936.2390253	124	Grand Index
30	m	13-16 years	0.947	937.7101628	124	Grand Index
30	m	13-16 years	0.948	938	124	Grand Index
30	m	13-16 years	0.949	939	125	Grand Index
30	m	13-16 years	0.95	939.0988231	125	Grand Index
30	m	13-16 years	0.951	940	125	Grand Index
30	m	13-16 years	0.952	941.0703544	125	Grand Index
30	m	13-16 years	0.954	942	125	Grand Index
30	m	13-16 years	0.955	942.9791786	125	Grand Index
30	m	13-16 years	0.956	944.6921039	126	Grand Index
30	m	13-16 years	0.957	945.0142588	126	Grand Index
30	m	13-16 years	0.958	946	126	Grand Index
30	m	13-16 years	0.959	946.276	126	Grand Index
30	m	13-16 years	0.96	947.2974981	126	Grand Index
30	m	13-16 years	0.961	948	126	Grand Index
30	m	13-16 years	0.962	948.4036635	127	Grand Index
30	m	13-16 years	0.963	949	127	Grand Index
30	m	13-16 years	0.964	950	127	Grand Index
30	m	13-16 years	0.965	951.26	127	Grand Index
30	m	13-16 years	0.966	952.4440048	127	Grand Index
30	m	13-16 years	0.967	953.6930388	128	Grand Index

Age	Gender	Education	Percentile	Raw Score	Norm Score	Assessment
30	m	13-16 years	0.968	954.9617284	128	Grand Index
30	m	13-16 years	0.969	955.8875789	128	Grand Index
30	m	13-16 years	0.97	958.08	128	Grand Index
30	m	13-16 years	0.971	959.488	128	Grand Index
30	m	13-16 years	0.972	962	129	Grand Index
30	m	13-16 years	0.973	962.825574	129	Grand Index
30	m	13-16 years	0.974	965.1927608	129	Grand Index
30	m	13-16 years	0.975	967.7091155	129	Grand Index
30	m	13-16 years	0.976	968.0608167	130	Grand Index
30	m	13-16 years	0.977	970	130	Grand Index
30	m	13-16 years	0.978	971.8917975	130	Grand Index
30	m	13-16 years	0.979	975.429309	131	Grand Index
30	m	13-16 years	0.98	976	131	Grand Index
30	m	13-16 years	0.981	978	131	Grand Index
30	m	13-16 years	0.982	979.0204287	131	Grand Index
30	m	13-16 years	0.983	981	132	Grand Index
30	m	13-16 years	0.984	984.1381178	132	Grand Index
30	m	13-16 years	0.985	986.8916975	133	Grand Index
30	m	13-16 years	0.986	988.0030699	133	Grand Index
30	m	13-16 years	0.987	990.2693708	133	Grand Index
30	m	13-16 years	0.988	991.52123	134	Grand Index
30	m	13-16 years	0.989	992.196	134	Grand Index
30	m	13-16 years	0.99	998.9798456	135	Grand Index
30	m	13-16 years	0.991	1002.2254	135	Grand Index
30	m	13-16 years	0.992	1007.434417	136	Grand Index
30	m	13-16 years	0.993	1011.516212	137	Grand Index
30	m	13-16 years	0.994	1014.955961	138	Grand Index
30	m	13-16 years	0.995	1016.36	139	Grand Index
30	m	13-16 years	0.996	1022.039904	140	Grand Index
30	m	13-16 years	0.997	1026.452826	141	Grand Index
30	m	13-16 years	0.998	1033.619072	143	Grand Index
30	m	13-16 years	0.999	1038.538514	146	Grand Index
30	f	17+ years	0.001	602.3084873	54	Grand Index
30	f	17+ years	0.002	612.0913237	57	Grand Index
30	f	17+ years	0.003	615.406	59	Grand Index
30	f	17+ years	0.004	619.6284689	60	Grand Index
30	f	17+ years	0.005	623.3166845	61	Grand Index
30	f	17+ years	0.006	629.2885687	62	Grand Index
30	f	17+ years	0.007	630	63	Grand Index
30	f	17+ years	0.008	632.8862263	64	Grand Index
30	f	17+ years	0.009	636.109	65	Grand Index
30	f	17+ years	0.01	640.6432622	65	Grand Index
30	f	17+ years	0.011	643.5183428	66	Grand Index
30	f	17+ years	0.012	645.812	66	Grand Index
30	f	17+ years	0.013	651.1911909	67	Grand Index
30	f	17+ years	0.014	652.0411401	67	Grand Index
30	f	17+ years	0.015	653.6433319	67	Grand Index
30	f	17+ years	0.016	655	68	Grand Index
30	f	17+ years	0.017	656	68	Grand Index
30	f	17+ years	0.018	656.5304332	69	Grand Index
30	f	17+ years	0.019	659.119	69	Grand Index
30	f	17+ years	0.02	661	69	Grand Index
30	f	17+ years	0.021	661.4047902	69	Grand Index
30	f	17+ years	0.022	663.644	70	Grand Index
30	f	17+ years	0.023	668.1244913	70	Grand Index
30	f	17+ years	0.024	669	70	Grand Index

Age	Gender	Education	Percentile	Raw Score	Norm Score	Assessment
30	f	17+ years	0.025	669.7746202	71	Grand Index
30	f	17+ years	0.026	671.426	71	Grand Index
30	f	17+ years	0.027	672.327	71	Grand Index
30	f	17+ years	0.028	674.228	71	Grand Index
30	f	17+ years	0.029	675.5057658	72	Grand Index
30	f	17+ years	0.03	676.9700712	72	Grand Index
30	f	17+ years	0.031	678	72	Grand Index
30	f	17+ years	0.032	679.3948061	72	Grand Index
30	f	17+ years	0.033	679.9972239	72	Grand Index
30	f	17+ years	0.034	680.9759299	73	Grand Index
30	f	17+ years	0.035	681	73	Grand Index
30	f	17+ years	0.036	681.9997422	73	Grand Index
30	f	17+ years	0.037	682.3260058	73	Grand Index
30	f	17+ years	0.038	683	73	Grand Index
30	f	17+ years	0.039	684	74	Grand Index
30	f	17+ years	0.04	684.04	74	Grand Index
30	f	17+ years	0.041	685.941	74	Grand Index
30	f	17+ years	0.042	686.99048	74	Grand Index
30	f	17+ years	0.043	687	74	Grand Index
30	f	17+ years	0.044	687.9729808	74	Grand Index
30	f	17+ years	0.045	688.545	75	Grand Index
30	f	17+ years	0.046	689.239368	75	Grand Index
30	f	17+ years	0.047	690	75	Grand Index
30	f	17+ years	0.048	690.7308233	75	Grand Index
30	f	17+ years	0.049	691	75	Grand Index
30	f	17+ years	0.05	691.6065574	75	Grand Index
30	f	17+ years	0.051	692	75	Grand Index
30	f	17+ years	0.052	692.109832	76	Grand Index
30	f	17+ years	0.053	693	76	Grand Index
30	f	17+ years	0.054	693.6550035	76	Grand Index
30	f	17+ years	0.055	694	76	Grand Index
30	f	17+ years	0.056	694.0862211	76	Grand Index
30	f	17+ years	0.057	695	76	Grand Index
30	f	17+ years	0.058	695.258	76	Grand Index
30	f	17+ years	0.059	696.1158828	77	Grand Index
30	f	17+ years	0.061	697	77	Grand Index
30	f	17+ years	0.062	697.7585555	77	Grand Index
30	f	17+ years	0.063	697.9851575	77	Grand Index
30	f	17+ years	0.064	698.664	77	Grand Index
30	f	17+ years	0.065	699	77	Grand Index
30	f	17+ years	0.066	699.466	77	Grand Index
30	f	17+ years	0.068	700	78	Grand Index
30	f	17+ years	0.069	700.9831517	78	Grand Index
30	f	17+ years	0.071	701	78	Grand Index
30	f	17+ years	0.072	702	78	Grand Index
30	f	17+ years	0.073	702.773	78	Grand Index
30	f	17+ years	0.074	703	78	Grand Index
30	f	17+ years	0.075	704	78	Grand Index
30	f	17+ years	0.076	704.7586826	79	Grand Index
30	f	17+ years	0.077	705	79	Grand Index
30	f	17+ years	0.078	705.2689754	79	Grand Index
30	f	17+ years	0.082	706	79	Grand Index
30	f	17+ years	0.083	706.783	79	Grand Index
30	f	17+ years	0.085	707	79	Grand Index
30	f	17+ years	0.086	707.3896057	80	Grand Index
30	f	17+ years	0.087	707.9317773	80	Grand Index

Age	Gender	Education	Percentile	Raw Score	Norm Score	Assessment
30	f	17+ years	0.088	708	80	Grand Index
30	f	17+ years	0.089	708.8586606	80	Grand Index
30	f	17+ years	0.09	709.1904911	80	Grand Index
30	f	17+ years	0.091	709.999177	80	Grand Index
30	f	17+ years	0.092	710.0643143	80	Grand Index
30	f	17+ years	0.093	710.9271642	80	Grand Index
30	f	17+ years	0.094	711.3194837	80	Grand Index
30	f	17+ years	0.095	712	80	Grand Index
30	f	17+ years	0.096	712.6304685	80	Grand Index
30	f	17+ years	0.099	713	81	Grand Index
30	f	17+ years	0.1	713.0347529	81	Grand Index
30	f	17+ years	0.102	714	81	Grand Index
30	f	17+ years	0.103	714.3798203	81	Grand Index
30	f	17+ years	0.104	714.9126612	81	Grand Index
30	f	17+ years	0.105	716	81	Grand Index
30	f	17+ years	0.106	716.3200752	81	Grand Index
30	f	17+ years	0.107	716.9637676	81	Grand Index
30	f	17+ years	0.108	717	81	Grand Index
30	f	17+ years	0.109	717.0169348	82	Grand Index
30	f	17+ years	0.11	718	82	Grand Index
30	f	17+ years	0.111	718.011	82	Grand Index
30	f	17+ years	0.113	719	82	Grand Index
30	f	17+ years	0.114	719.0199623	82	Grand Index
30	f	17+ years	0.115	720	82	Grand Index
30	f	17+ years	0.116	720.516	82	Grand Index
30	f	17+ years	0.117	721	82	Grand Index
30	f	17+ years	0.118	721.6177469	82	Grand Index
30	f	17+ years	0.119	722	82	Grand Index
30	f	17+ years	0.12	722.0009776	82	Grand Index
30	f	17+ years	0.121	722.2821966	82	Grand Index
30	f	17+ years	0.124	723	83	Grand Index
30	f	17+ years	0.125	724	83	Grand Index
30	f	17+ years	0.126	724.4377645	83	Grand Index
30	f	17+ years	0.127	725	83	Grand Index
30	f	17+ years	0.128	725.3085345	83	Grand Index
30	f	17+ years	0.131	726	83	Grand Index
30	f	17+ years	0.132	726.932	83	Grand Index
30	f	17+ years	0.134	727	83	Grand Index
30	f	17+ years	0.135	727.635	83	Grand Index
30	f	17+ years	0.137	728	84	Grand Index
30	f	17+ years	0.138	728.140834	84	Grand Index
30	f	17+ years	0.14	729	84	Grand Index
30	f	17+ years	0.143	730	84	Grand Index
30	f	17+ years	0.144	730.7246913	84	Grand Index
30	f	17+ years	0.148	731	84	Grand Index
30	f	17+ years	0.149	731.3069958	84	Grand Index
30	f	17+ years	0.152	732	85	Grand Index
30	f	17+ years	0.153	732.1420356	85	Grand Index
30	f	17+ years	0.156	733	85	Grand Index
30	f	17+ years	0.157	733.0378188	85	Grand Index
30	f	17+ years	0.158	733.5682265	85	Grand Index
30	f	17+ years	0.159	734	85	Grand Index
30	f	17+ years	0.16	734.16	85	Grand Index
30	f	17+ years	0.163	735	85	Grand Index
30	f	17+ years	0.164	735.8841428	85	Grand Index
30	f	17+ years	0.167	736	86	Grand Index

Age	Gender	Education	Percentile	Raw Score	Norm Score	Assessment
30	f	17+ years	0.17	737	86	Grand Index
30	f	17+ years	0.171	737.4083445	86	Grand Index
30	f	17+ years	0.174	738	86	Grand Index
30	f	17+ years	0.176	739	86	Grand Index
30	f	17+ years	0.177	739.5336392	86	Grand Index
30	f	17+ years	0.179	740	86	Grand Index
30	f	17+ years	0.18	740.1983789	86	Grand Index
30	f	17+ years	0.184	741	86	Grand Index
30	f	17+ years	0.185	741.685	87	Grand Index
30	f	17+ years	0.187	742	87	Grand Index
30	f	17+ years	0.188	743	87	Grand Index
30	f	17+ years	0.189	743.0837554	87	Grand Index
30	f	17+ years	0.194	744	87	Grand Index
30	f	17+ years	0.195	744.695	87	Grand Index
30	f	17+ years	0.198	745	87	Grand Index
30	f	17+ years	0.199	745.1772379	87	Grand Index
30	f	17+ years	0.203	746	88	Grand Index
30	f	17+ years	0.204	746.9233828	88	Grand Index
30	f	17+ years	0.209	747	88	Grand Index
30	f	17+ years	0.21	747.108146	88	Grand Index
30	f	17+ years	0.211	747.9470253	88	Grand Index
30	f	17+ years	0.213	748	88	Grand Index
30	f	17+ years	0.214	748.8158457	88	Grand Index
30	f	17+ years	0.216	749	88	Grand Index
30	f	17+ years	0.217	749.330752	88	Grand Index
30	f	17+ years	0.221	750	88	Grand Index
30	f	17+ years	0.222	750.0016553	89	Grand Index
30	f	17+ years	0.223	750.8347515	89	Grand Index
30	f	17+ years	0.226	751	89	Grand Index
30	f	17+ years	0.227	751.3164321	89	Grand Index
30	f	17+ years	0.231	752	89	Grand Index
30	f	17+ years	0.232	752.3533801	89	Grand Index
30	f	17+ years	0.233	752.9829133	89	Grand Index
30	f	17+ years	0.235	753	89	Grand Index
30	f	17+ years	0.236	753.4476453	89	Grand Index
30	f	17+ years	0.237	754	89	Grand Index
30	f	17+ years	0.238	754.8142353	89	Grand Index
30	f	17+ years	0.24	755	89	Grand Index
30	f	17+ years	0.241	755.1218825	89	Grand Index
30	f	17+ years	0.246	756	90	Grand Index
30	f	17+ years	0.249	757	90	Grand Index
30	f	17+ years	0.25	757.25	90	Grand Index
30	f	17+ years	0.252	758	90	Grand Index
30	f	17+ years	0.253	758.0757959	90	Grand Index
30	f	17+ years	0.254	758.9948279	90	Grand Index
30	f	17+ years	0.257	759	90	Grand Index
30	f	17+ years	0.258	759.4292806	90	Grand Index
30	f	17+ years	0.261	760	90	Grand Index
30	f	17+ years	0.266	761	91	Grand Index
30	f	17+ years	0.267	761.9960926	91	Grand Index
30	f	17+ years	0.269	762	91	Grand Index
30	f	17+ years	0.27	762.2651577	91	Grand Index
30	f	17+ years	0.271	763	91	Grand Index
30	f	17+ years	0.272	763.0088153	91	Grand Index
30	f	17+ years	0.273	763.536631	91	Grand Index
30	f	17+ years	0.275	764	91	Grand Index

Age	Gender	Education	Percentile	Raw Score	Norm Score	Assessment
30	f	17+ years	0.276	764.0178672	91	Grand Index
30	f	17+ years	0.277	764.9567745	91	Grand Index
30	f	17+ years	0.278	765	91	Grand Index
30	f	17+ years	0.28	766	91	Grand Index
30	f	17+ years	0.281	766.0025911	91	Grand Index
30	f	17+ years	0.285	767	91	Grand Index
30	f	17+ years	0.286	767.2894542	92	Grand Index
30	f	17+ years	0.287	767.8176008	92	Grand Index
30	f	17+ years	0.288	768	92	Grand Index
30	f	17+ years	0.289	768.389	92	Grand Index
30	f	17+ years	0.293	769	92	Grand Index
30	f	17+ years	0.294	769.894	92	Grand Index
30	f	17+ years	0.296	770	92	Grand Index
30	f	17+ years	0.297	770.0379518	92	Grand Index
30	f	17+ years	0.299	771	92	Grand Index
30	f	17+ years	0.3	771.7562148	92	Grand Index
30	f	17+ years	0.304	772	92	Grand Index
30	f	17+ years	0.305	772.805	92	Grand Index
30	f	17+ years	0.308	773	92	Grand Index
30	f	17+ years	0.309	773.3478528	93	Grand Index
30	f	17+ years	0.313	774	93	Grand Index
30	f	17+ years	0.314	774.0427639	93	Grand Index
30	f	17+ years	0.315	774.9299695	93	Grand Index
30	f	17+ years	0.319	775	93	Grand Index
30	f	17+ years	0.32	775.0287554	93	Grand Index
30	f	17+ years	0.324	776	93	Grand Index
30	f	17+ years	0.325	776.9529608	93	Grand Index
30	f	17+ years	0.33	777	93	Grand Index
30	f	17+ years	0.331	777.5228344	93	Grand Index
30	f	17+ years	0.335	778	94	Grand Index
30	f	17+ years	0.34	779	94	Grand Index
30	f	17+ years	0.341	779.4835075	94	Grand Index
30	f	17+ years	0.344	780	94	Grand Index
30	f	17+ years	0.345	780.1883732	94	Grand Index
30	f	17+ years	0.346	780.869595	94	Grand Index
30	f	17+ years	0.347	781	94	Grand Index
30	f	17+ years	0.348	781.9285678	94	Grand Index
30	f	17+ years	0.351	782	94	Grand Index
30	f	17+ years	0.352	782.4377886	94	Grand Index
30	f	17+ years	0.357	783	95	Grand Index
30	f	17+ years	0.358	783.558	95	Grand Index
30	f	17+ years	0.361	784	95	Grand Index
30	f	17+ years	0.362	784.2115757	95	Grand Index
30	f	17+ years	0.365	785	95	Grand Index
30	f	17+ years	0.366	785.0133513	95	Grand Index
30	f	17+ years	0.368	786	95	Grand Index
30	f	17+ years	0.369	786.1689119	95	Grand Index
30	f	17+ years	0.37	786.6483816	95	Grand Index
30	f	17+ years	0.372	787	95	Grand Index
30	f	17+ years	0.38	788	95	Grand Index
30	f	17+ years	0.381	788.4860842	95	Grand Index
30	f	17+ years	0.385	789	96	Grand Index
30	f	17+ years	0.386	789.334696	96	Grand Index
30	f	17+ years	0.387	789.9990238	96	Grand Index
30	f	17+ years	0.392	790	96	Grand Index
30	f	17+ years	0.393	790.5604696	96	Grand Index

Age	Gender	Education	Percentile	Raw Score	Norm Score	Assessment
30	f	17+ years	0.396	791	96	Grand Index
30	f	17+ years	0.397	791.1391301	96	Grand Index
30	f	17+ years	0.4	792	96	Grand Index
30	f	17+ years	0.401	792.1695967	96	Grand Index
30	f	17+ years	0.408	793	97	Grand Index
30	f	17+ years	0.409	793.8991341	97	Grand Index
30	f	17+ years	0.412	794	97	Grand Index
30	f	17+ years	0.413	794.113	97	Grand Index
30	f	17+ years	0.418	795	97	Grand Index
30	f	17+ years	0.419	795.5048091	97	Grand Index
30	f	17+ years	0.424	796	97	Grand Index
30	f	17+ years	0.425	796.6198172	97	Grand Index
30	f	17+ years	0.43	797	97	Grand Index
30	f	17+ years	0.432	798	97	Grand Index
30	f	17+ years	0.433	798.133	97	Grand Index
30	f	17+ years	0.435	799	98	Grand Index
30	f	17+ years	0.436	799.8405111	98	Grand Index
30	f	17+ years	0.442	800	98	Grand Index
30	f	17+ years	0.443	800.8461696	98	Grand Index
30	f	17+ years	0.448	801	98	Grand Index
30	f	17+ years	0.449	801.549	98	Grand Index
30	f	17+ years	0.453	802	98	Grand Index
30	f	17+ years	0.454	802.5431172	98	Grand Index
30	f	17+ years	0.459	803	98	Grand Index
30	f	17+ years	0.46	803.46	98	Grand Index
30	f	17+ years	0.461	804	99	Grand Index
30	f	17+ years	0.462	804.0746553	99	Grand Index
30	f	17+ years	0.463	804.6278816	99	Grand Index
30	f	17+ years	0.464	804.9859733	99	Grand Index
30	f	17+ years	0.467	805	99	Grand Index
30	f	17+ years	0.468	805.4785252	99	Grand Index
30	f	17+ years	0.473	806	99	Grand Index
30	f	17+ years	0.475	807	99	Grand Index
30	f	17+ years	0.476	807.3982327	99	Grand Index
30	f	17+ years	0.477	807.9015171	99	Grand Index
30	f	17+ years	0.481	808	99	Grand Index
30	f	17+ years	0.482	808.0038723	99	Grand Index
30	f	17+ years	0.486	809	99	Grand Index
30	f	17+ years	0.492	810	100	Grand Index
30	f	17+ years	0.493	810.2356075	100	Grand Index
30	f	17+ years	0.499	811	100	Grand Index
30	f	17+ years	0.5	811.747467	100	Grand Index
30	f	17+ years	0.503	812	100	Grand Index
30	f	17+ years	0.504	812.0459278	100	Grand Index
30	f	17+ years	0.505	812.3050194	100	Grand Index
30	f	17+ years	0.512	813	100	Grand Index
30	f	17+ years	0.513	813.3370226	100	Grand Index
30	f	17+ years	0.519	814	101	Grand Index
30	f	17+ years	0.52	814.472406	101	Grand Index
30	f	17+ years	0.523	815	101	Grand Index
30	f	17+ years	0.524	815.124	101	Grand Index
30	f	17+ years	0.528	816	101	Grand Index
30	f	17+ years	0.529	816.7088354	101	Grand Index
30	f	17+ years	0.533	817	101	Grand Index
30	f	17+ years	0.537	818	101	Grand Index
30	f	17+ years	0.538	818.738	101	Grand Index

Age	Gender	Education	Percentile	Raw Score	Norm Score	Assessment
30	f	17+ years	0.543	819	102	Grand Index
30	f	17+ years	0.544	819.0091901	102	Grand Index
30	f	17+ years	0.545	819.6443076	102	Grand Index
30	f	17+ years	0.546	819.9937049	102	Grand Index
30	f	17+ years	0.548	820	102	Grand Index
30	f	17+ years	0.549	820.2192256	102	Grand Index
30	f	17+ years	0.55	820.7316122	102	Grand Index
30	f	17+ years	0.556	821	102	Grand Index
30	f	17+ years	0.557	821.857	102	Grand Index
30	f	17+ years	0.559	822	102	Grand Index
30	f	17+ years	0.56	822.3449738	102	Grand Index
30	f	17+ years	0.561	822.932956	102	Grand Index
30	f	17+ years	0.566	823	102	Grand Index
30	f	17+ years	0.567	823.9986901	103	Grand Index
30	f	17+ years	0.571	824	103	Grand Index
30	f	17+ years	0.572	824.0772912	103	Grand Index
30	f	17+ years	0.573	824.7818945	103	Grand Index
30	f	17+ years	0.577	825	103	Grand Index
30	f	17+ years	0.583	826	103	Grand Index
30	f	17+ years	0.584	826.2398898	103	Grand Index
30	f	17+ years	0.587	827	103	Grand Index
30	f	17+ years	0.588	827.8665611	103	Grand Index
30	f	17+ years	0.591	828	103	Grand Index
30	f	17+ years	0.594	829	104	Grand Index
30	f	17+ years	0.595	829.0450443	104	Grand Index
30	f	17+ years	0.596	829.9978966	104	Grand Index
30	f	17+ years	0.6	830	104	Grand Index
30	f	17+ years	0.601	830.501	104	Grand Index
30	f	17+ years	0.604	831	104	Grand Index
30	f	17+ years	0.605	831.105	104	Grand Index
30	f	17+ years	0.606	832	104	Grand Index
30	f	17+ years	0.607	832.4336051	104	Grand Index
30	f	17+ years	0.612	833	104	Grand Index
30	f	17+ years	0.613	833.3259294	104	Grand Index
30	f	17+ years	0.618	834	105	Grand Index
30	f	17+ years	0.619	834.9955232	105	Grand Index
30	f	17+ years	0.621	835	105	Grand Index
30	f	17+ years	0.622	835.1206169	105	Grand Index
30	f	17+ years	0.623	835.3970711	105	Grand Index
30	f	17+ years	0.631	836	105	Grand Index
30	f	17+ years	0.632	836.0672397	105	Grand Index
30	f	17+ years	0.633	836.8761709	105	Grand Index
30	f	17+ years	0.637	837	105	Grand Index
30	f	17+ years	0.641	838	105	Grand Index
30	f	17+ years	0.642	838.8664534	105	Grand Index
30	f	17+ years	0.645	839	106	Grand Index
30	f	17+ years	0.646	839.9523856	106	Grand Index
30	f	17+ years	0.649	840	106	Grand Index
30	f	17+ years	0.653	841	106	Grand Index
30	f	17+ years	0.654	841.254	106	Grand Index
30	f	17+ years	0.659	842	106	Grand Index
30	f	17+ years	0.66	842.0906246	106	Grand Index
30	f	17+ years	0.661	842.7513623	106	Grand Index
30	f	17+ years	0.664	843	106	Grand Index
30	f	17+ years	0.667	844	106	Grand Index
30	f	17+ years	0.668	844.7581662	107	Grand Index

Age	Gender	Education	Percentile	Raw Score	Norm Score	Assessment
30	f	17+ years	0.671	845	107	Grand Index
30	f	17+ years	0.672	845.472	107	Grand Index
30	f	17+ years	0.673	846	107	Grand Index
30	f	17+ years	0.674	846.0479417	107	Grand Index
30	f	17+ years	0.675	846.8001619	107	Grand Index
30	f	17+ years	0.682	847	107	Grand Index
30	f	17+ years	0.687	848	107	Grand Index
30	f	17+ years	0.688	848.888	107	Grand Index
30	f	17+ years	0.693	849	108	Grand Index
30	f	17+ years	0.694	849.294	108	Grand Index
30	f	17+ years	0.698	850	108	Grand Index
30	f	17+ years	0.7	851	108	Grand Index
30	f	17+ years	0.701	851.4363334	108	Grand Index
30	f	17+ years	0.704	852	108	Grand Index
30	f	17+ years	0.705	852.6746287	108	Grand Index
30	f	17+ years	0.707	853	108	Grand Index
30	f	17+ years	0.708	853.467985	108	Grand Index
30	f	17+ years	0.712	854	108	Grand Index
30	f	17+ years	0.713	854.213038	108	Grand Index
30	f	17+ years	0.715	855	109	Grand Index
30	f	17+ years	0.716	855.116	109	Grand Index
30	f	17+ years	0.717	856.0105413	109	Grand Index
30	f	17+ years	0.718	856.9688463	109	Grand Index
30	f	17+ years	0.72	857	109	Grand Index
30	f	17+ years	0.721	857.6855017	109	Grand Index
30	f	17+ years	0.723	858	109	Grand Index
30	f	17+ years	0.724	858.0314054	109	Grand Index
30	f	17+ years	0.728	859	109	Grand Index
30	f	17+ years	0.729	859.1014989	109	Grand Index
30	f	17+ years	0.73	859.8951618	109	Grand Index
30	f	17+ years	0.732	860	109	Grand Index
30	f	17+ years	0.733	860.4621479	109	Grand Index
30	f	17+ years	0.737	861	110	Grand Index
30	f	17+ years	0.738	861.0043849	110	Grand Index
30	f	17+ years	0.741	862	110	Grand Index
30	f	17+ years	0.744	863	110	Grand Index
30	f	17+ years	0.745	863.8020126	110	Grand Index
30	f	17+ years	0.748	864	110	Grand Index
30	f	17+ years	0.749	864.849	110	Grand Index
30	f	17+ years	0.75	865	110	Grand Index
30	f	17+ years	0.751	865.9541206	110	Grand Index
30	f	17+ years	0.752	866	110	Grand Index
30	f	17+ years	0.753	866.467201	110	Grand Index
30	f	17+ years	0.755	867	110	Grand Index
30	f	17+ years	0.756	867.6557511	110	Grand Index
30	f	17+ years	0.76	868	111	Grand Index
30	f	17+ years	0.761	868.5761573	111	Grand Index
30	f	17+ years	0.763	869	111	Grand Index
30	f	17+ years	0.764	869.5600849	111	Grand Index
30	f	17+ years	0.769	870	111	Grand Index
30	f	17+ years	0.773	871	111	Grand Index
30	f	17+ years	0.774	872	111	Grand Index
30	f	17+ years	0.775	872.275	111	Grand Index
30	f	17+ years	0.778	873	111	Grand Index
30	f	17+ years	0.781	874	112	Grand Index
30	f	17+ years	0.782	874.6372987	112	Grand Index

Age	Gender	Education	Percentile	Raw Score	Norm Score	Assessment
30	f	17+ years	0.784	875	112	Grand Index
30	f	17+ years	0.788	876	112	Grand Index
30	f	17+ years	0.791	877	112	Grand Index
30	f	17+ years	0.792	877.9641223	112	Grand Index
30	f	17+ years	0.794	878	112	Grand Index
30	f	17+ years	0.795	878.0033344	112	Grand Index
30	f	17+ years	0.797	879	112	Grand Index
30	f	17+ years	0.798	879.998	113	Grand Index
30	f	17+ years	0.8	880	113	Grand Index
30	f	17+ years	0.801	880.7091135	113	Grand Index
30	f	17+ years	0.804	881	113	Grand Index
30	f	17+ years	0.805	881.7251152	113	Grand Index
30	f	17+ years	0.806	881.9796879	113	Grand Index
30	f	17+ years	0.81	882	113	Grand Index
30	f	17+ years	0.811	882.5695118	113	Grand Index
30	f	17+ years	0.812	883	113	Grand Index
30	f	17+ years	0.815	884	113	Grand Index
30	f	17+ years	0.816	884.601589	114	Grand Index
30	f	17+ years	0.817	885	114	Grand Index
30	f	17+ years	0.818	885.0131737	114	Grand Index
30	f	17+ years	0.819	885.5995517	114	Grand Index
30	f	17+ years	0.822	886	114	Grand Index
30	f	17+ years	0.823	886.523	114	Grand Index
30	f	17+ years	0.824	887	114	Grand Index
30	f	17+ years	0.825	887.325	114	Grand Index
30	f	17+ years	0.828	888	114	Grand Index
30	f	17+ years	0.829	888.929	114	Grand Index
30	f	17+ years	0.831	889	114	Grand Index
30	f	17+ years	0.832	889.632	114	Grand Index
30	f	17+ years	0.835	890	115	Grand Index
30	f	17+ years	0.836	890.0942459	115	Grand Index
30	f	17+ years	0.837	890.9685916	115	Grand Index
30	f	17+ years	0.839	891	115	Grand Index
30	f	17+ years	0.84	891.9479119	115	Grand Index
30	f	17+ years	0.843	892	115	Grand Index
30	f	17+ years	0.845	893	115	Grand Index
30	f	17+ years	0.846	893.1849947	115	Grand Index
30	f	17+ years	0.847	893.9484419	115	Grand Index
30	f	17+ years	0.849	894	115	Grand Index
30	f	17+ years	0.852	895	116	Grand Index
30	f	17+ years	0.853	895.543596	116	Grand Index
30	f	17+ years	0.856	896	116	Grand Index
30	f	17+ years	0.857	897.0067679	116	Grand Index
30	f	17+ years	0.858	897.3650753	116	Grand Index
30	f	17+ years	0.862	898	116	Grand Index
30	f	17+ years	0.863	898.0086562	116	Grand Index
30	f	17+ years	0.864	899	116	Grand Index
30	f	17+ years	0.865	899.2523161	117	Grand Index
30	f	17+ years	0.867	900	117	Grand Index
30	f	17+ years	0.868	900.1407934	117	Grand Index
30	f	17+ years	0.87	901	117	Grand Index
30	f	17+ years	0.871	902.4771444	117	Grand Index
30	f	17+ years	0.874	903	117	Grand Index
30	f	17+ years	0.875	903.9520196	117	Grand Index
30	f	17+ years	0.876	904.0915963	117	Grand Index
30	f	17+ years	0.877	905	117	Grand Index

Age	Gender	Education	Percentile	Raw Score	Norm Score	Assessment
30	f	17+ years	0.878	905.049968	117	Grand Index
30	f	17+ years	0.879	905.9924529	118	Grand Index
30	f	17+ years	0.88	906	118	Grand Index
30	f	17+ years	0.881	906.781	118	Grand Index
30	f	17+ years	0.885	907	118	Grand Index
30	f	17+ years	0.886	907.286	118	Grand Index
30	f	17+ years	0.889	908	118	Grand Index
30	f	17+ years	0.89	908.9537915	118	Grand Index
30	f	17+ years	0.891	909	118	Grand Index
30	f	17+ years	0.893	910	119	Grand Index
30	f	17+ years	0.894	910.494	119	Grand Index
30	f	17+ years	0.896	911	119	Grand Index
30	f	17+ years	0.897	911.4133092	119	Grand Index
30	f	17+ years	0.899	912	119	Grand Index
30	f	17+ years	0.9	912.5360023	119	Grand Index
30	f	17+ years	0.901	912.7398708	119	Grand Index
30	f	17+ years	0.902	913	119	Grand Index
30	f	17+ years	0.903	914	119	Grand Index
30	f	17+ years	0.904	914.203857	120	Grand Index
30	f	17+ years	0.905	914.8485123	120	Grand Index
30	f	17+ years	0.906	915	120	Grand Index
30	f	17+ years	0.907	916	120	Grand Index
30	f	17+ years	0.908	916.108	120	Grand Index
30	f	17+ years	0.909	917.0020208	120	Grand Index
30	f	17+ years	0.91	917.2929887	120	Grand Index
30	f	17+ years	0.912	918	120	Grand Index
30	f	17+ years	0.913	918.613	120	Grand Index
30	f	17+ years	0.915	919	121	Grand Index
30	f	17+ years	0.916	919.9584523	121	Grand Index
30	f	17+ years	0.917	920	121	Grand Index
30	f	17+ years	0.918	920.1263858	121	Grand Index
30	f	17+ years	0.92	921	121	Grand Index
30	f	17+ years	0.921	922	121	Grand Index
30	f	17+ years	0.922	922.722	121	Grand Index
30	f	17+ years	0.923	923	121	Grand Index
30	f	17+ years	0.924	924	121	Grand Index
30	f	17+ years	0.925	924.2906559	122	Grand Index
30	f	17+ years	0.926	924.9693045	122	Grand Index
30	f	17+ years	0.927	925.0043386	122	Grand Index
30	f	17+ years	0.928	925.1637743	122	Grand Index
30	f	17+ years	0.929	926	122	Grand Index
30	f	17+ years	0.93	926.93	122	Grand Index
30	f	17+ years	0.931	927.831	122	Grand Index
30	f	17+ years	0.932	928.9983	122	Grand Index
30	f	17+ years	0.933	929	122	Grand Index
30	f	17+ years	0.934	930.2737803	123	Grand Index
30	f	17+ years	0.936	931	123	Grand Index
30	f	17+ years	0.937	932	123	Grand Index
30	f	17+ years	0.938	932.9217607	123	Grand Index
30	f	17+ years	0.939	933	123	Grand Index
30	f	17+ years	0.94	933.94	123	Grand Index
30	f	17+ years	0.941	934	123	Grand Index
30	f	17+ years	0.942	935.755542	124	Grand Index
30	f	17+ years	0.943	936	124	Grand Index
30	f	17+ years	0.944	936.5588322	124	Grand Index
30	f	17+ years	0.945	937	124	Grand Index

Age	Gender	Education	Percentile	Raw Score	Norm Score	Assessment
30	f	17+ years	0.946	938.1916736	124	Grand Index
30	f	17+ years	0.947	940	124	Grand Index
30	f	17+ years	0.948	941.0701135	124	Grand Index
30	f	17+ years	0.949	942.2189314	125	Grand Index
30	f	17+ years	0.95	943.316713	125	Grand Index
30	f	17+ years	0.951	945.4853344	125	Grand Index
30	f	17+ years	0.952	946.0270119	125	Grand Index
30	f	17+ years	0.953	946.8574483	125	Grand Index
30	f	17+ years	0.954	947.4675585	125	Grand Index
30	f	17+ years	0.955	949.070845	125	Grand Index
30	f	17+ years	0.956	950	126	Grand Index
30	f	17+ years	0.957	950.257	126	Grand Index
30	f	17+ years	0.958	951	126	Grand Index
30	f	17+ years	0.959	953.059	126	Grand Index
30	f	17+ years	0.96	954.96	126	Grand Index
30	f	17+ years	0.961	955	126	Grand Index
30	f	17+ years	0.962	955.5770141	127	Grand Index
30	f	17+ years	0.963	956	127	Grand Index
30	f	17+ years	0.964	957.4907049	127	Grand Index
30	f	17+ years	0.965	960.0551137	127	Grand Index
30	f	17+ years	0.966	963.366	127	Grand Index
30	f	17+ years	0.967	965.0323008	128	Grand Index
30	f	17+ years	0.968	965.3425679	128	Grand Index
30	f	17+ years	0.969	966.4011791	128	Grand Index
30	f	17+ years	0.97	967.97	128	Grand Index
30	f	17+ years	0.971	968	128	Grand Index
30	f	17+ years	0.972	968.8599637	129	Grand Index
30	f	17+ years	0.973	969.673	129	Grand Index
30	f	17+ years	0.974	971	129	Grand Index
30	f	17+ years	0.975	973	129	Grand Index
30	f	17+ years	0.976	973.81423	130	Grand Index
30	f	17+ years	0.977	974.859353	130	Grand Index
30	f	17+ years	0.978	977.9452103	130	Grand Index
30	f	17+ years	0.979	978.6592529	131	Grand Index
30	f	17+ years	0.98	978.9972722	131	Grand Index
30	f	17+ years	0.981	979.9378287	131	Grand Index
30	f	17+ years	0.982	982.0946038	131	Grand Index
30	f	17+ years	0.983	983.7000099	132	Grand Index
30	f	17+ years	0.984	985.584	132	Grand Index
30	f	17+ years	0.985	986.8145743	133	Grand Index
30	f	17+ years	0.986	987.346516	133	Grand Index
30	f	17+ years	0.987	992.193251	133	Grand Index
30	f	17+ years	0.988	999.1227507	134	Grand Index
30	f	17+ years	0.989	1000.075977	134	Grand Index
30	f	17+ years	0.99	1000.998537	135	Grand Index
30	f	17+ years	0.991	1002.070201	135	Grand Index
30	f	17+ years	0.992	1005.789652	136	Grand Index
30	f	17+ years	0.993	1008.545982	137	Grand Index
30	f	17+ years	0.994	1010.594	138	Grand Index
30	f	17+ years	0.995	1015.161558	139	Grand Index
30	f	17+ years	0.996	1017.341242	140	Grand Index
30	f	17+ years	0.997	1023.705936	141	Grand Index
30	f	17+ years	0.998	1044.379234	143	Grand Index
30	f	17+ years	0.999	1046.755287	146	Grand Index
30	m	17+ years	0.001	598.1308208	54	Grand Index
30	m	17+ years	0.002	601.948	57	Grand Index

Age	Gender	Education	Percentile	Raw Score	Norm Score	Assessment
30	m	17+ years	0.003	609.5279679	59	Grand Index
30	m	17+ years	0.004	616.316	60	Grand Index
30	m	17+ years	0.005	619	61	Grand Index
30	m	17+ years	0.006	620.7870513	62	Grand Index
30	m	17+ years	0.007	627.3768682	63	Grand Index
30	m	17+ years	0.008	631.9242715	64	Grand Index
30	m	17+ years	0.009	633.8887962	65	Grand Index
30	m	17+ years	0.01	636.2172508	65	Grand Index
30	m	17+ years	0.011	641	66	Grand Index
30	m	17+ years	0.012	641.2006679	66	Grand Index
30	m	17+ years	0.013	643.027	67	Grand Index
30	m	17+ years	0.014	646.2030677	67	Grand Index
30	m	17+ years	0.015	649	67	Grand Index
30	m	17+ years	0.016	649.0282311	68	Grand Index
30	m	17+ years	0.017	650.1167559	68	Grand Index
30	m	17+ years	0.018	651.0368763	69	Grand Index
30	m	17+ years	0.019	652	69	Grand Index
30	m	17+ years	0.02	653.58	69	Grand Index
30	m	17+ years	0.021	655.4845984	69	Grand Index
30	m	17+ years	0.022	657.738	70	Grand Index
30	m	17+ years	0.023	660.4252753	70	Grand Index
30	m	17+ years	0.024	661.9733292	70	Grand Index
30	m	17+ years	0.025	663.1163278	71	Grand Index
30	m	17+ years	0.026	664.0106678	71	Grand Index
30	m	17+ years	0.027	665	71	Grand Index
30	m	17+ years	0.028	666.9563966	71	Grand Index
30	m	17+ years	0.029	668	72	Grand Index
30	m	17+ years	0.03	668.37	72	Grand Index
30	m	17+ years	0.031	669	72	Grand Index
30	m	17+ years	0.032	669.2745588	72	Grand Index
30	m	17+ years	0.033	669.903876	72	Grand Index
30	m	17+ years	0.034	672.0550599	73	Grand Index
30	m	17+ years	0.035	673	73	Grand Index
30	m	17+ years	0.036	673.7666132	73	Grand Index
30	m	17+ years	0.037	674	73	Grand Index
30	m	17+ years	0.038	677	73	Grand Index
30	m	17+ years	0.039	677.081	74	Grand Index
30	m	17+ years	0.04	678.16	74	Grand Index
30	m	17+ years	0.041	679	74	Grand Index
30	m	17+ years	0.042	679.510831	74	Grand Index
30	m	17+ years	0.043	681	74	Grand Index
30	m	17+ years	0.044	681.9249898	74	Grand Index
30	m	17+ years	0.045	683	75	Grand Index
30	m	17+ years	0.046	683.634	75	Grand Index
30	m	17+ years	0.047	684	75	Grand Index
30	m	17+ years	0.048	685	75	Grand Index
30	m	17+ years	0.051	687	75	Grand Index
30	m	17+ years	0.053	688	76	Grand Index
30	m	17+ years	0.054	689.266	76	Grand Index
30	m	17+ years	0.055	690	76	Grand Index
30	m	17+ years	0.056	690.3941828	76	Grand Index
30	m	17+ years	0.057	691.5259777	76	Grand Index
30	m	17+ years	0.058	692.9737031	76	Grand Index
30	m	17+ years	0.059	693	77	Grand Index
30	m	17+ years	0.06	693.6669309	77	Grand Index
30	m	17+ years	0.061	694	77	Grand Index

Age	Gender	Education	Percentile	Raw Score	Norm Score	Assessment
30	m	17+ years	0.062	694.0928752	77	Grand Index
30	m	17+ years	0.063	694.3428008	77	Grand Index
30	m	17+ years	0.064	695	77	Grand Index
30	m	17+ years	0.065	695.135	77	Grand Index
30	m	17+ years	0.066	696	77	Grand Index
30	m	17+ years	0.067	696.4295932	78	Grand Index
30	m	17+ years	0.068	697.3127561	78	Grand Index
30	m	17+ years	0.069	698.451	78	Grand Index
30	m	17+ years	0.07	700.3952836	78	Grand Index
30	m	17+ years	0.071	701.3739322	78	Grand Index
30	m	17+ years	0.072	702.5063618	78	Grand Index
30	m	17+ years	0.073	703	78	Grand Index
30	m	17+ years	0.074	703.3460436	78	Grand Index
30	m	17+ years	0.075	704.925	78	Grand Index
30	m	17+ years	0.076	705.0111399	79	Grand Index
30	m	17+ years	0.078	706	79	Grand Index
30	m	17+ years	0.08	707	79	Grand Index
30	m	17+ years	0.081	707.399	79	Grand Index
30	m	17+ years	0.083	709	79	Grand Index
30	m	17+ years	0.084	709.7885198	79	Grand Index
30	m	17+ years	0.085	710.1288369	79	Grand Index
30	m	17+ years	0.087	711	80	Grand Index
30	m	17+ years	0.088	711.612697	80	Grand Index
30	m	17+ years	0.089	712.2885411	80	Grand Index
30	m	17+ years	0.091	713	80	Grand Index
30	m	17+ years	0.092	713.2677545	80	Grand Index
30	m	17+ years	0.095	714	80	Grand Index
30	m	17+ years	0.096	714.9110339	80	Grand Index
30	m	17+ years	0.097	715	81	Grand Index
30	m	17+ years	0.1	716	81	Grand Index
30	m	17+ years	0.101	716.9058544	81	Grand Index
30	m	17+ years	0.104	717	81	Grand Index
30	m	17+ years	0.105	717.0051733	81	Grand Index
30	m	17+ years	0.106	717.4999138	81	Grand Index
30	m	17+ years	0.107	717.8809373	81	Grand Index
30	m	17+ years	0.108	718	81	Grand Index
30	m	17+ years	0.109	718.8025787	82	Grand Index
30	m	17+ years	0.111	719	82	Grand Index
30	m	17+ years	0.112	719.8103782	82	Grand Index
30	m	17+ years	0.114	720	82	Grand Index
30	m	17+ years	0.115	720.0195607	82	Grand Index
30	m	17+ years	0.116	720.6244746	82	Grand Index
30	m	17+ years	0.117	721	82	Grand Index
30	m	17+ years	0.118	721.461544	82	Grand Index
30	m	17+ years	0.121	722	82	Grand Index
30	m	17+ years	0.122	722.0468026	83	Grand Index
30	m	17+ years	0.123	722.781785	83	Grand Index
30	m	17+ years	0.125	723	83	Grand Index
30	m	17+ years	0.126	723.8113339	83	Grand Index
30	m	17+ years	0.129	724	83	Grand Index
30	m	17+ years	0.13	724.0769411	83	Grand Index
30	m	17+ years	0.131	724.6721596	83	Grand Index
30	m	17+ years	0.133	725	83	Grand Index
30	m	17+ years	0.134	725.160661	83	Grand Index
30	m	17+ years	0.137	726	84	Grand Index
30	m	17+ years	0.138	726.9047437	84	Grand Index

Age	Gender	Education	Percentile	Raw Score	Norm Score	Assessment
30	m	17+ years	0.139	727.0219058	84	Grand Index
30	m	17+ years	0.14	728.9171879	84	Grand Index
30	m	17+ years	0.142	729	84	Grand Index
30	m	17+ years	0.143	729.1650012	84	Grand Index
30	m	17+ years	0.144	730	84	Grand Index
30	m	17+ years	0.146	731	84	Grand Index
30	m	17+ years	0.147	731.613	84	Grand Index
30	m	17+ years	0.15	732	84	Grand Index
30	m	17+ years	0.151	732.7250224	85	Grand Index
30	m	17+ years	0.152	733.008	85	Grand Index
30	m	17+ years	0.155	734	85	Grand Index
30	m	17+ years	0.156	734.5512657	85	Grand Index
30	m	17+ years	0.157	734.9130546	85	Grand Index
30	m	17+ years	0.159	735	85	Grand Index
30	m	17+ years	0.16	735.5887473	85	Grand Index
30	m	17+ years	0.163	736	85	Grand Index
30	m	17+ years	0.164	736.0553681	85	Grand Index
30	m	17+ years	0.167	737	86	Grand Index
30	m	17+ years	0.168	737.272	86	Grand Index
30	m	17+ years	0.173	738	86	Grand Index
30	m	17+ years	0.174	738.746	86	Grand Index
30	m	17+ years	0.176	739	86	Grand Index
30	m	17+ years	0.177	739.5987593	86	Grand Index
30	m	17+ years	0.179	740	86	Grand Index
30	m	17+ years	0.18	740.22	86	Grand Index
30	m	17+ years	0.182	741	86	Grand Index
30	m	17+ years	0.183	741.1386659	86	Grand Index
30	m	17+ years	0.188	742	87	Grand Index
30	m	17+ years	0.189	742.9345371	87	Grand Index
30	m	17+ years	0.192	743	87	Grand Index
30	m	17+ years	0.193	743.0066098	87	Grand Index
30	m	17+ years	0.194	743.9442556	87	Grand Index
30	m	17+ years	0.195	744	87	Grand Index
30	m	17+ years	0.196	744.0711749	87	Grand Index
30	m	17+ years	0.197	744.9638571	87	Grand Index
30	m	17+ years	0.2	745	87	Grand Index
30	m	17+ years	0.204	746	88	Grand Index
30	m	17+ years	0.205	746.0677785	88	Grand Index
30	m	17+ years	0.206	746.9459526	88	Grand Index
30	m	17+ years	0.207	747	88	Grand Index
30	m	17+ years	0.208	747.4325118	88	Grand Index
30	m	17+ years	0.211	748	88	Grand Index
30	m	17+ years	0.212	748.3154591	88	Grand Index
30	m	17+ years	0.213	749	88	Grand Index
30	m	17+ years	0.214	749.906	88	Grand Index
30	m	17+ years	0.217	750	88	Grand Index
30	m	17+ years	0.218	750.4422686	88	Grand Index
30	m	17+ years	0.221	751	88	Grand Index
30	m	17+ years	0.222	751.5088926	89	Grand Index
30	m	17+ years	0.225	752	89	Grand Index
30	m	17+ years	0.226	752.7713541	89	Grand Index
30	m	17+ years	0.229	753	89	Grand Index
30	m	17+ years	0.23	753.17	89	Grand Index
30	m	17+ years	0.231	754	89	Grand Index
30	m	17+ years	0.233	755	89	Grand Index
30	m	17+ years	0.234	755.1216364	89	Grand Index

Age	Gender	Education	Percentile	Raw Score	Norm Score	Assessment
30	m	17+ years	0.235	755.9920004	89	Grand Index
30	m	17+ years	0.238	756	89	Grand Index
30	m	17+ years	0.239	756.6785304	89	Grand Index
30	m	17+ years	0.24	756.9960823	89	Grand Index
30	m	17+ years	0.242	757	90	Grand Index
30	m	17+ years	0.245	758	90	Grand Index
30	m	17+ years	0.25	759	90	Grand Index
30	m	17+ years	0.251	759.6788726	90	Grand Index
30	m	17+ years	0.253	760	90	Grand Index
30	m	17+ years	0.26	761	90	Grand Index
30	m	17+ years	0.261	761.5784781	90	Grand Index
30	m	17+ years	0.265	762	91	Grand Index
30	m	17+ years	0.266	762.2574498	91	Grand Index
30	m	17+ years	0.272	763	91	Grand Index
30	m	17+ years	0.273	763.7512062	91	Grand Index
30	m	17+ years	0.277	764	91	Grand Index
30	m	17+ years	0.278	764.7014608	91	Grand Index
30	m	17+ years	0.28	765	91	Grand Index
30	m	17+ years	0.281	765.199	91	Grand Index
30	m	17+ years	0.284	766	91	Grand Index
30	m	17+ years	0.287	767	92	Grand Index
30	m	17+ years	0.289	768	92	Grand Index
30	m	17+ years	0.29	768.5211715	92	Grand Index
30	m	17+ years	0.293	769	92	Grand Index
30	m	17+ years	0.294	769.3018858	92	Grand Index
30	m	17+ years	0.297	770	92	Grand Index
30	m	17+ years	0.298	770.2816293	92	Grand Index
30	m	17+ years	0.303	771	92	Grand Index
30	m	17+ years	0.304	771.064338	92	Grand Index
30	m	17+ years	0.307	772	92	Grand Index
30	m	17+ years	0.308	772.2828393	92	Grand Index
30	m	17+ years	0.313	773	93	Grand Index
30	m	17+ years	0.314	773.1698081	93	Grand Index
30	m	17+ years	0.315	773.9172523	93	Grand Index
30	m	17+ years	0.317	774	93	Grand Index
30	m	17+ years	0.318	774.0009339	93	Grand Index
30	m	17+ years	0.319	774.9507573	93	Grand Index
30	m	17+ years	0.321	775	93	Grand Index
30	m	17+ years	0.322	775.1994161	93	Grand Index
30	m	17+ years	0.323	775.9524079	93	Grand Index
30	m	17+ years	0.329	776	93	Grand Index
30	m	17+ years	0.33	776.1317519	93	Grand Index
30	m	17+ years	0.334	777	94	Grand Index
30	m	17+ years	0.335	777.0405831	94	Grand Index
30	m	17+ years	0.336	777.6166209	94	Grand Index
30	m	17+ years	0.339	778	94	Grand Index
30	m	17+ years	0.34	778.86	94	Grand Index
30	m	17+ years	0.345	779	94	Grand Index
30	m	17+ years	0.346	779.334	94	Grand Index
30	m	17+ years	0.349	780	94	Grand Index
30	m	17+ years	0.35	780.1247326	94	Grand Index
30	m	17+ years	0.351	780.8173611	94	Grand Index
30	m	17+ years	0.355	781	94	Grand Index
30	m	17+ years	0.356	781.124	94	Grand Index
30	m	17+ years	0.359	782	95	Grand Index
30	m	17+ years	0.36	782.0476877	95	Grand Index

Age	Gender	Education	Percentile	Raw Score	Norm Score	Assessment
30	m	17+ years	0.364	783	95	Grand Index
30	m	17+ years	0.367	784	95	Grand Index
30	m	17+ years	0.373	785	95	Grand Index
30	m	17+ years	0.374	785.4854431	95	Grand Index
30	m	17+ years	0.377	786	95	Grand Index
30	m	17+ years	0.378	786.0966876	95	Grand Index
30	m	17+ years	0.379	786.9802328	95	Grand Index
30	m	17+ years	0.381	787	95	Grand Index
30	m	17+ years	0.382	787.0679501	95	Grand Index
30	m	17+ years	0.39	788	96	Grand Index
30	m	17+ years	0.391	788.9488679	96	Grand Index
30	m	17+ years	0.395	789	96	Grand Index
30	m	17+ years	0.396	789.4659934	96	Grand Index
30	m	17+ years	0.399	790	96	Grand Index
30	m	17+ years	0.4	790.6	96	Grand Index
30	m	17+ years	0.403	791	96	Grand Index
30	m	17+ years	0.404	791.980092	96	Grand Index
30	m	17+ years	0.406	792	96	Grand Index
30	m	17+ years	0.407	792.0117578	96	Grand Index
30	m	17+ years	0.408	792.3921579	97	Grand Index
30	m	17+ years	0.412	793	97	Grand Index
30	m	17+ years	0.413	793.627	97	Grand Index
30	m	17+ years	0.416	794	97	Grand Index
30	m	17+ years	0.417	794.968164	97	Grand Index
30	m	17+ years	0.422	795	97	Grand Index
30	m	17+ years	0.423	795.2210005	97	Grand Index
30	m	17+ years	0.424	795.8737614	97	Grand Index
30	m	17+ years	0.429	796	97	Grand Index
30	m	17+ years	0.432	797	97	Grand Index
30	m	17+ years	0.433	797.207	97	Grand Index
30	m	17+ years	0.436	798	98	Grand Index
30	m	17+ years	0.437	798.4082059	98	Grand Index
30	m	17+ years	0.443	799	98	Grand Index
30	m	17+ years	0.444	799.7308902	98	Grand Index
30	m	17+ years	0.45	800	98	Grand Index
30	m	17+ years	0.451	800.1266709	98	Grand Index
30	m	17+ years	0.452	800.7769524	98	Grand Index
30	m	17+ years	0.454	801	98	Grand Index
30	m	17+ years	0.455	801.4403841	98	Grand Index
30	m	17+ years	0.458	802	98	Grand Index
30	m	17+ years	0.459	802.261	98	Grand Index
30	m	17+ years	0.463	803	99	Grand Index
30	m	17+ years	0.467	804	99	Grand Index
30	m	17+ years	0.468	804.994392	99	Grand Index
30	m	17+ years	0.47	805	99	Grand Index
30	m	17+ years	0.471	805.5736618	99	Grand Index
30	m	17+ years	0.473	806	99	Grand Index
30	m	17+ years	0.474	806.1214215	99	Grand Index
30	m	17+ years	0.475	806.4667292	99	Grand Index
30	m	17+ years	0.476	806.9935785	99	Grand Index
30	m	17+ years	0.478	807	99	Grand Index
30	m	17+ years	0.479	807.841	99	Grand Index
30	m	17+ years	0.482	808	99	Grand Index
30	m	17+ years	0.483	808.157	99	Grand Index
30	m	17+ years	0.485	809	99	Grand Index
30	m	17+ years	0.489	810	100	Grand Index

Age	Gender	Education	Percentile	Raw Score	Norm Score	Assessment
30	m	17+ years	0.49	810.2726033	100	Grand Index
30	m	17+ years	0.493	811	100	Grand Index
30	m	17+ years	0.494	811.026	100	Grand Index
30	m	17+ years	0.499	812	100	Grand Index
30	m	17+ years	0.5	812.5	100	Grand Index
30	m	17+ years	0.503	813	100	Grand Index
30	m	17+ years	0.504	813.816	100	Grand Index
30	m	17+ years	0.506	814	100	Grand Index
30	m	17+ years	0.507	814.5164159	100	Grand Index
30	m	17+ years	0.514	815	101	Grand Index
30	m	17+ years	0.515	815.3559547	101	Grand Index
30	m	17+ years	0.516	815.8373188	101	Grand Index
30	m	17+ years	0.52	816	101	Grand Index
30	m	17+ years	0.521	816.159	101	Grand Index
30	m	17+ years	0.526	817	101	Grand Index
30	m	17+ years	0.527	817.691159	101	Grand Index
30	m	17+ years	0.531	818	101	Grand Index
30	m	17+ years	0.532	818.027758	101	Grand Index
30	m	17+ years	0.536	819	101	Grand Index
30	m	17+ years	0.537	819.8857435	101	Grand Index
30	m	17+ years	0.54	820	102	Grand Index
30	m	17+ years	0.541	820.739	102	Grand Index
30	m	17+ years	0.546	821	102	Grand Index
30	m	17+ years	0.547	821.0348967	102	Grand Index
30	m	17+ years	0.552	822	102	Grand Index
30	m	17+ years	0.553	822.1785239	102	Grand Index
30	m	17+ years	0.554	822.9365105	102	Grand Index
30	m	17+ years	0.556	823	102	Grand Index
30	m	17+ years	0.557	823.3696051	102	Grand Index
30	m	17+ years	0.56	824	102	Grand Index
30	m	17+ years	0.561	824.319	102	Grand Index
30	m	17+ years	0.565	825	102	Grand Index
30	m	17+ years	0.566	825.714	102	Grand Index
30	m	17+ years	0.572	826	103	Grand Index
30	m	17+ years	0.573	826.267	103	Grand Index
30	m	17+ years	0.578	827	103	Grand Index
30	m	17+ years	0.579	827.4226069	103	Grand Index
30	m	17+ years	0.584	828	103	Grand Index
30	m	17+ years	0.585	828.3440345	103	Grand Index
30	m	17+ years	0.587	829	103	Grand Index
30	m	17+ years	0.588	829.6803948	103	Grand Index
30	m	17+ years	0.591	830	103	Grand Index
30	m	17+ years	0.595	831	104	Grand Index
30	m	17+ years	0.596	831.059193	104	Grand Index
30	m	17+ years	0.601	832	104	Grand Index
30	m	17+ years	0.602	832.6886264	104	Grand Index
30	m	17+ years	0.603	832.9973725	104	Grand Index
30	m	17+ years	0.605	833	104	Grand Index
30	m	17+ years	0.606	833.874	104	Grand Index
30	m	17+ years	0.61	834	104	Grand Index
30	m	17+ years	0.611	834.2426534	104	Grand Index
30	m	17+ years	0.612	834.9907806	104	Grand Index
30	m	17+ years	0.613	835	104	Grand Index
30	m	17+ years	0.614	835.0228324	104	Grand Index
30	m	17+ years	0.615	835.8602105	104	Grand Index
30	m	17+ years	0.617	836	104	Grand Index

Age	Gender	Education	Percentile	Raw Score	Norm Score	Assessment
30	m	17+ years	0.618	836.2379171	105	Grand Index
30	m	17+ years	0.625	837	105	Grand Index
30	m	17+ years	0.626	837.7010588	105	Grand Index
30	m	17+ years	0.63	838	105	Grand Index
30	m	17+ years	0.631	838.6527941	105	Grand Index
30	m	17+ years	0.635	839	105	Grand Index
30	m	17+ years	0.636	839.1072439	105	Grand Index
30	m	17+ years	0.642	840	105	Grand Index
30	m	17+ years	0.643	840.5966955	105	Grand Index
30	m	17+ years	0.645	841	106	Grand Index
30	m	17+ years	0.651	842	106	Grand Index
30	m	17+ years	0.652	842.9000461	106	Grand Index
30	m	17+ years	0.657	843	106	Grand Index
30	m	17+ years	0.66	844	106	Grand Index
30	m	17+ years	0.661	844.1294619	106	Grand Index
30	m	17+ years	0.667	845	106	Grand Index
30	m	17+ years	0.668	845.7753769	107	Grand Index
30	m	17+ years	0.67	846	107	Grand Index
30	m	17+ years	0.674	847	107	Grand Index
30	m	17+ years	0.675	847.6623355	107	Grand Index
30	m	17+ years	0.678	848	107	Grand Index
30	m	17+ years	0.679	848.5304493	107	Grand Index
30	m	17+ years	0.681	849	107	Grand Index
30	m	17+ years	0.684	850	107	Grand Index
30	m	17+ years	0.685	850.078989	107	Grand Index
30	m	17+ years	0.686	850.9161556	107	Grand Index
30	m	17+ years	0.688	851	107	Grand Index
30	m	17+ years	0.689	851.8486512	107	Grand Index
30	m	17+ years	0.69	852	107	Grand Index
30	m	17+ years	0.691	852.311034	107	Grand Index
30	m	17+ years	0.692	852.7944737	108	Grand Index
30	m	17+ years	0.693	852.993939	108	Grand Index
30	m	17+ years	0.695	853	108	Grand Index
30	m	17+ years	0.696	853.9846025	108	Grand Index
30	m	17+ years	0.7	854	108	Grand Index
30	m	17+ years	0.701	854.5046513	108	Grand Index
30	m	17+ years	0.702	854.9624586	108	Grand Index
30	m	17+ years	0.705	855	108	Grand Index
30	m	17+ years	0.706	855.774	108	Grand Index
30	m	17+ years	0.711	856	108	Grand Index
30	m	17+ years	0.712	856.3083121	108	Grand Index
30	m	17+ years	0.714	857	108	Grand Index
30	m	17+ years	0.715	857.0826021	109	Grand Index
30	m	17+ years	0.718	858	109	Grand Index
30	m	17+ years	0.719	858.9040562	109	Grand Index
30	m	17+ years	0.723	859	109	Grand Index
30	m	17+ years	0.727	860	109	Grand Index
30	m	17+ years	0.728	860.7825478	109	Grand Index
30	m	17+ years	0.73	861	109	Grand Index
30	m	17+ years	0.731	861.6060078	109	Grand Index
30	m	17+ years	0.735	862	109	Grand Index
30	m	17+ years	0.736	862.008867	109	Grand Index
30	m	17+ years	0.74	863	110	Grand Index
30	m	17+ years	0.741	863.5766496	110	Grand Index
30	m	17+ years	0.745	864	110	Grand Index
30	m	17+ years	0.746	864.95346	110	Grand Index

Age	Gender	Education	Percentile	Raw Score	Norm Score	Assessment
30	m	17+ years	0.749	865	110	Grand Index
30	m	17+ years	0.75	865.1940493	110	Grand Index
30	m	17+ years	0.751	865.8753649	110	Grand Index
30	m	17+ years	0.752	866	110	Grand Index
30	m	17+ years	0.753	866.0096512	110	Grand Index
30	m	17+ years	0.756	867	110	Grand Index
30	m	17+ years	0.76	868	111	Grand Index
30	m	17+ years	0.761	868.102899	111	Grand Index
30	m	17+ years	0.765	869	111	Grand Index
30	m	17+ years	0.766	869.9817083	111	Grand Index
30	m	17+ years	0.767	870	111	Grand Index
30	m	17+ years	0.768	870.3644229	111	Grand Index
30	m	17+ years	0.772	871	111	Grand Index
30	m	17+ years	0.773	871.1975822	111	Grand Index
30	m	17+ years	0.774	871.8850606	111	Grand Index
30	m	17+ years	0.776	872	111	Grand Index
30	m	17+ years	0.777	872.1107896	111	Grand Index
30	m	17+ years	0.781	873	112	Grand Index
30	m	17+ years	0.782	873.9780734	112	Grand Index
30	m	17+ years	0.784	874	112	Grand Index
30	m	17+ years	0.785	874.9417344	112	Grand Index
30	m	17+ years	0.788	875	112	Grand Index
30	m	17+ years	0.789	875.5368966	112	Grand Index
30	m	17+ years	0.792	876	112	Grand Index
30	m	17+ years	0.793	876.5205675	112	Grand Index
30	m	17+ years	0.794	876.9583034	112	Grand Index
30	m	17+ years	0.797	877	112	Grand Index
30	m	17+ years	0.802	878	113	Grand Index
30	m	17+ years	0.803	878.8800713	113	Grand Index
30	m	17+ years	0.807	879	113	Grand Index
30	m	17+ years	0.808	879.832	113	Grand Index
30	m	17+ years	0.813	880	113	Grand Index
30	m	17+ years	0.815	881	113	Grand Index
30	m	17+ years	0.816	881.464	114	Grand Index
30	m	17+ years	0.821	882	114	Grand Index
30	m	17+ years	0.822	882.9826316	114	Grand Index
30	m	17+ years	0.825	883	114	Grand Index
30	m	17+ years	0.828	884	114	Grand Index
30	m	17+ years	0.829	885	114	Grand Index
30	m	17+ years	0.83	885.0330529	114	Grand Index
30	m	17+ years	0.831	885.7229487	114	Grand Index
30	m	17+ years	0.833	886	114	Grand Index
30	m	17+ years	0.834	886.8997559	115	Grand Index
30	m	17+ years	0.836	887	115	Grand Index
30	m	17+ years	0.837	887.123	115	Grand Index
30	m	17+ years	0.838	888	115	Grand Index
30	m	17+ years	0.841	889	115	Grand Index
30	m	17+ years	0.842	889.518	115	Grand Index
30	m	17+ years	0.847	890	115	Grand Index
30	m	17+ years	0.848	890.5361657	115	Grand Index
30	m	17+ years	0.849	891	115	Grand Index
30	m	17+ years	0.85	891.7240544	116	Grand Index
30	m	17+ years	0.852	892	116	Grand Index
30	m	17+ years	0.853	893.387	116	Grand Index
30	m	17+ years	0.855	894	116	Grand Index
30	m	17+ years	0.856	894.2720612	116	Grand Index

Age	Gender	Education	Percentile	Raw Score	Norm Score	Assessment
30	m	17+ years	0.858	895	116	Grand Index
30	m	17+ years	0.859	895.3985723	116	Grand Index
30	m	17+ years	0.861	896	116	Grand Index
30	m	17+ years	0.862	896.098	116	Grand Index
30	m	17+ years	0.863	897	116	Grand Index
30	m	17+ years	0.864	897.2227982	116	Grand Index
30	m	17+ years	0.865	898	117	Grand Index
30	m	17+ years	0.866	898.414	117	Grand Index
30	m	17+ years	0.87	900	117	Grand Index
30	m	17+ years	0.873	901	117	Grand Index
30	m	17+ years	0.876	902	117	Grand Index
30	m	17+ years	0.877	902.0829038	117	Grand Index
30	m	17+ years	0.879	903	118	Grand Index
30	m	17+ years	0.88	903.5461351	118	Grand Index
30	m	17+ years	0.881	904	118	Grand Index
30	m	17+ years	0.882	904.9971909	118	Grand Index
30	m	17+ years	0.883	905	118	Grand Index
30	m	17+ years	0.884	905.908082	118	Grand Index
30	m	17+ years	0.885	906	118	Grand Index
30	m	17+ years	0.887	907	118	Grand Index
30	m	17+ years	0.888	907.2259706	118	Grand Index
30	m	17+ years	0.889	908.2301583	118	Grand Index
30	m	17+ years	0.891	909	118	Grand Index
30	m	17+ years	0.892	909.4481641	119	Grand Index
30	m	17+ years	0.893	910.9856388	119	Grand Index
30	m	17+ years	0.894	911	119	Grand Index
30	m	17+ years	0.895	912.705	119	Grand Index
30	m	17+ years	0.896	913	119	Grand Index
30	m	17+ years	0.897	913.863	119	Grand Index
30	m	17+ years	0.898	914.0516926	119	Grand Index
30	m	17+ years	0.899	915	119	Grand Index
30	m	17+ years	0.9	915.1	119	Grand Index
30	m	17+ years	0.901	916	119	Grand Index
30	m	17+ years	0.902	916.0291186	119	Grand Index
30	m	17+ years	0.903	916.2410464	119	Grand Index
30	m	17+ years	0.904	917	120	Grand Index
30	m	17+ years	0.905	917.4728121	120	Grand Index
30	m	17+ years	0.907	918	120	Grand Index
30	m	17+ years	0.908	918.732	120	Grand Index
30	m	17+ years	0.912	919	120	Grand Index
30	m	17+ years	0.913	919.8666816	120	Grand Index
30	m	17+ years	0.914	920	120	Grand Index
30	m	17+ years	0.915	920.285	121	Grand Index
30	m	17+ years	0.916	921	121	Grand Index
30	m	17+ years	0.917	921.2001991	121	Grand Index
30	m	17+ years	0.918	922	121	Grand Index
30	m	17+ years	0.919	922.2275464	121	Grand Index
30	m	17+ years	0.92	923.0746288	121	Grand Index
30	m	17+ years	0.921	923.9902595	121	Grand Index
30	m	17+ years	0.922	924.838	121	Grand Index
30	m	17+ years	0.923	925.7774969	121	Grand Index
30	m	17+ years	0.924	926	121	Grand Index
30	m	17+ years	0.925	926.1997629	122	Grand Index
30	m	17+ years	0.926	927	122	Grand Index
30	m	17+ years	0.927	927.233	122	Grand Index
30	m	17+ years	0.928	929.7138248	122	Grand Index

Age	Gender	Education	Percentile	Raw Score	Norm Score	Assessment
30	m	17+ years	0.929	931.9985377	122	Grand Index
30	m	17+ years	0.93	932.0146894	122	Grand Index
30	m	17+ years	0.931	932.1550655	122	Grand Index
30	m	17+ years	0.932	932.9759412	122	Grand Index
30	m	17+ years	0.933	933.121092	122	Grand Index
30	m	17+ years	0.934	934.786	123	Grand Index
30	m	17+ years	0.935	935.865	123	Grand Index
30	m	17+ years	0.938	936	123	Grand Index
30	m	17+ years	0.939	936.1323099	123	Grand Index
30	m	17+ years	0.94	937	123	Grand Index
30	m	17+ years	0.941	937.7116007	123	Grand Index
30	m	17+ years	0.942	938.418	124	Grand Index
30	m	17+ years	0.943	939	124	Grand Index
30	m	17+ years	0.944	939.576	124	Grand Index
30	m	17+ years	0.945	940	124	Grand Index
30	m	17+ years	0.946	941	124	Grand Index
30	m	17+ years	0.947	941.813	124	Grand Index
30	m	17+ years	0.948	943	124	Grand Index
30	m	17+ years	0.949	943.1720592	125	Grand Index
30	m	17+ years	0.95	944.05	125	Grand Index
30	m	17+ years	0.951	945.02426	125	Grand Index
30	m	17+ years	0.952	945.7998193	125	Grand Index
30	m	17+ years	0.953	946	125	Grand Index
30	m	17+ years	0.954	946.366	125	Grand Index
30	m	17+ years	0.956	947	126	Grand Index
30	m	17+ years	0.957	948.603	126	Grand Index
30	m	17+ years	0.958	949.5237918	126	Grand Index
30	m	17+ years	0.959	949.8228368	126	Grand Index
30	m	17+ years	0.961	950	126	Grand Index
30	m	17+ years	0.962	951.9980762	127	Grand Index
30	m	17+ years	0.963	953.1453807	127	Grand Index
30	m	17+ years	0.964	955	127	Grand Index
30	m	17+ years	0.965	956.0936217	127	Grand Index
30	m	17+ years	0.966	957	127	Grand Index
30	m	17+ years	0.967	958.0647877	128	Grand Index
30	m	17+ years	0.968	959.3766255	128	Grand Index
30	m	17+ years	0.969	960.2859718	128	Grand Index
30	m	17+ years	0.97	963.9516198	128	Grand Index
30	m	17+ years	0.971	964.3216418	128	Grand Index
30	m	17+ years	0.972	964.969498	129	Grand Index
30	m	17+ years	0.973	965	129	Grand Index
30	m	17+ years	0.974	965.1418309	129	Grand Index
30	m	17+ years	0.975	966	129	Grand Index
30	m	17+ years	0.976	967.0245789	130	Grand Index
30	m	17+ years	0.977	967.5326973	130	Grand Index
30	m	17+ years	0.979	968	131	Grand Index
30	m	17+ years	0.98	969.4638241	131	Grand Index
30	m	17+ years	0.981	972.5875323	131	Grand Index
30	m	17+ years	0.982	975.7424639	131	Grand Index
30	m	17+ years	0.983	981.285	132	Grand Index
30	m	17+ years	0.984	984.736	132	Grand Index
30	m	17+ years	0.985	988.517327	133	Grand Index
30	m	17+ years	0.986	990.445509	133	Grand Index
30	m	17+ years	0.987	993.9836736	133	Grand Index
30	m	17+ years	0.988	995.052	134	Grand Index
30	m	17+ years	0.989	1000.481404	134	Grand Index

Age	Gender	Education	Percentile	Raw Score	Norm Score	Assessment
30	m	17+ years	0.99	1002.382492	135	Grand Index
30	m	17+ years	0.991	1004	135	Grand Index
30	m	17+ years	0.992	1006.808668	136	Grand Index
30	m	17+ years	0.993	1013.154735	137	Grand Index
30	m	17+ years	0.994	1014.713249	138	Grand Index
30	m	17+ years	0.995	1019.209866	139	Grand Index
30	m	17+ years	0.996	1022.855902	140	Grand Index
30	m	17+ years	0.997	1036.802072	141	Grand Index
30	m	17+ years	0.998	1055.052808	143	Grand Index
30	m	17+ years	0.999	1063.605	146	Grand Index
35	f	0-12 years	0.001	589.3655865	54	Grand Index
35	f	0-12 years	0.002	599.7311729	57	Grand Index
35	f	0-12 years	0.003	610.0967594	59	Grand Index
35	f	0-12 years	0.004	620.4623458	60	Grand Index
35	f	0-12 years	0.005	630.1510722	61	Grand Index
35	f	0-12 years	0.006	631.3564858	62	Grand Index
35	f	0-12 years	0.007	632.5618993	63	Grand Index
35	f	0-12 years	0.008	633.7673129	64	Grand Index
35	f	0-12 years	0.009	634.9727264	65	Grand Index
35	f	0-12 years	0.01	636.39	65	Grand Index
35	f	0-12 years	0.011	639.029	66	Grand Index
35	f	0-12 years	0.012	641.668	66	Grand Index
35	f	0-12 years	0.013	644.307	67	Grand Index
35	f	0-12 years	0.014	646.946	67	Grand Index
35	f	0-12 years	0.015	649.4101456	67	Grand Index
35	f	0-12 years	0.016	651.2603579	68	Grand Index
35	f	0-12 years	0.017	653.1105702	68	Grand Index
35	f	0-12 years	0.018	654.9607825	69	Grand Index
35	f	0-12 years	0.019	656.8109948	69	Grand Index
35	f	0-12 years	0.02	658.2874856	69	Grand Index
35	f	0-12 years	0.021	658.8732732	69	Grand Index
35	f	0-12 years	0.022	659.4590609	70	Grand Index
35	f	0-12 years	0.023	660.0448486	70	Grand Index
35	f	0-12 years	0.024	660.6306363	70	Grand Index
35	f	0-12 years	0.025	661.0636513	71	Grand Index
35	f	0-12 years	0.026	661.2359342	71	Grand Index
35	f	0-12 years	0.027	661.4082171	71	Grand Index
35	f	0-12 years	0.028	661.5804999	71	Grand Index
35	f	0-12 years	0.029	661.7527828	72	Grand Index
35	f	0-12 years	0.03	662.0600631	72	Grand Index
35	f	0-12 years	0.031	662.5368401	72	Grand Index
35	f	0-12 years	0.032	663.0136171	72	Grand Index
35	f	0-12 years	0.033	663.4903941	72	Grand Index
35	f	0-12 years	0.034	663.9671711	73	Grand Index
35	f	0-12 years	0.035	664.4916187	73	Grand Index
35	f	0-12 years	0.036	665.0605588	73	Grand Index
35	f	0-12 years	0.037	665.6294989	73	Grand Index
35	f	0-12 years	0.038	666.1984391	73	Grand Index
35	f	0-12 years	0.039	666.7673792	74	Grand Index
35	f	0-12 years	0.044	667	74	Grand Index
35	f	0-12 years	0.045	667.135	75	Grand Index
35	f	0-12 years	0.046	667.338	75	Grand Index
35	f	0-12 years	0.047	667.541	75	Grand Index
35	f	0-12 years	0.048	667.744	75	Grand Index
35	f	0-12 years	0.049	667.947	75	Grand Index
35	f	0-12 years	0.05	668.5154728	75	Grand Index

Age	Gender	Education	Percentile	Raw Score	Norm Score	Assessment
35	f	0-12 years	0.051	669.2130793	75	Grand Index
35	f	0-12 years	0.052	669.9106858	76	Grand Index
35	f	0-12 years	0.053	670.6082923	76	Grand Index
35	f	0-12 years	0.054	671.3058988	76	Grand Index
35	f	0-12 years	0.055	671.8594652	76	Grand Index
35	f	0-12 years	0.056	672.3798587	76	Grand Index
35	f	0-12 years	0.057	672.9002522	76	Grand Index
35	f	0-12 years	0.058	673.4206457	76	Grand Index
35	f	0-12 years	0.059	673.9410392	77	Grand Index
35	f	0-12 years	0.06	674.9	77	Grand Index
35	f	0-12 years	0.061	675.915	77	Grand Index
35	f	0-12 years	0.062	676.93	77	Grand Index
35	f	0-12 years	0.063	677.945	77	Grand Index
35	f	0-12 years	0.064	678.96	77	Grand Index
35	f	0-12 years	0.065	679.195	77	Grand Index
35	f	0-12 years	0.066	679.398	77	Grand Index
35	f	0-12 years	0.067	679.601	78	Grand Index
35	f	0-12 years	0.068	679.804	78	Grand Index
35	f	0-12 years	0.069	680.105	78	Grand Index
35	f	0-12 years	0.07	683.15	78	Grand Index
35	f	0-12 years	0.071	686.195	78	Grand Index
35	f	0-12 years	0.072	689.24	78	Grand Index
35	f	0-12 years	0.073	692.285	78	Grand Index
35	f	0-12 years	0.074	695.022	78	Grand Index
35	f	0-12 years	0.075	695.225	78	Grand Index
35	f	0-12 years	0.076	695.428	79	Grand Index
35	f	0-12 years	0.077	695.631	79	Grand Index
35	f	0-12 years	0.078	695.834	79	Grand Index
35	f	0-12 years	0.079	696.444	79	Grand Index
35	f	0-12 years	0.08	698.88	79	Grand Index
35	f	0-12 years	0.081	701.316	79	Grand Index
35	f	0-12 years	0.082	703.752	79	Grand Index
35	f	0-12 years	0.083	706.188	79	Grand Index
35	f	0-12 years	0.084	708.26	79	Grand Index
35	f	0-12 years	0.085	709.275	79	Grand Index
35	f	0-12 years	0.086	710.29	80	Grand Index
35	f	0-12 years	0.087	711.305	80	Grand Index
35	f	0-12 years	0.088	712.32	80	Grand Index
35	f	0-12 years	0.089	713.067	80	Grand Index
35	f	0-12 years	0.09	713.27	80	Grand Index
35	f	0-12 years	0.091	713.473	80	Grand Index
35	f	0-12 years	0.092	713.676	80	Grand Index
35	f	0-12 years	0.093	713.879	80	Grand Index
35	f	0-12 years	0.098	714	81	Grand Index
35	f	0-12 years	0.099	714.485	81	Grand Index
35	f	0-12 years	0.1	715.5	81	Grand Index
35	f	0-12 years	0.101	716.515	81	Grand Index
35	f	0-12 years	0.102	717.53	81	Grand Index
35	f	0-12 years	0.103	718.545	81	Grand Index
35	f	0-12 years	0.104	719.2915537	81	Grand Index
35	f	0-12 years	0.105	719.8199947	81	Grand Index
35	f	0-12 years	0.106	720.3484358	81	Grand Index
35	f	0-12 years	0.107	720.8768769	81	Grand Index
35	f	0-12 years	0.108	721.4053179	81	Grand Index
35	f	0-12 years	0.109	721.6535569	82	Grand Index
35	f	0-12 years	0.11	721.7341158	82	Grand Index

Age	Gender	Education	Percentile	Raw Score	Norm Score	Assessment
35	f	0-12 years	0.111	721.8146748	82	Grand Index
35	f	0-12 years	0.112	721.8952337	82	Grand Index
35	f	0-12 years	0.113	721.9757926	82	Grand Index
35	f	0-12 years	0.118	722	82	Grand Index
35	f	0-12 years	0.119	722.4698694	82	Grand Index
35	f	0-12 years	0.12	723.0774076	82	Grand Index
35	f	0-12 years	0.121	723.6849458	82	Grand Index
35	f	0-12 years	0.122	724.2924839	83	Grand Index
35	f	0-12 years	0.123	724.9000221	83	Grand Index
35	f	0-12 years	0.124	725.1660374	83	Grand Index
35	f	0-12 years	0.125	725.3704993	83	Grand Index
35	f	0-12 years	0.126	725.5749611	83	Grand Index
35	f	0-12 years	0.127	725.7794229	83	Grand Index
35	f	0-12 years	0.128	725.9838848	83	Grand Index
35	f	0-12 years	0.129	726.935	83	Grand Index
35	f	0-12 years	0.13	727.95	83	Grand Index
35	f	0-12 years	0.131	728.965	83	Grand Index
35	f	0-12 years	0.132	729.98	83	Grand Index
35	f	0-12 years	0.133	730.995	83	Grand Index
35	f	0-12 years	0.134	731.202	83	Grand Index
35	f	0-12 years	0.135	731.405	83	Grand Index
35	f	0-12 years	0.136	731.608	84	Grand Index
35	f	0-12 years	0.137	731.811	84	Grand Index
35	f	0-12 years	0.138	732.028	84	Grand Index
35	f	0-12 years	0.139	732.434	84	Grand Index
35	f	0-12 years	0.14	732.84	84	Grand Index
35	f	0-12 years	0.141	733.246	84	Grand Index
35	f	0-12 years	0.142	733.652	84	Grand Index
35	f	0-12 years	0.143	734.058	84	Grand Index
35	f	0-12 years	0.144	734.464	84	Grand Index
35	f	0-12 years	0.145	734.87	84	Grand Index
35	f	0-12 years	0.146	735.276	84	Grand Index
35	f	0-12 years	0.147	735.682	84	Grand Index
35	f	0-12 years	0.148	736.088	84	Grand Index
35	f	0-12 years	0.149	736.494	84	Grand Index
35	f	0-12 years	0.15	736.9	84	Grand Index
35	f	0-12 years	0.151	737.306	85	Grand Index
35	f	0-12 years	0.152	737.712	85	Grand Index
35	f	0-12 years	0.153	738.059	85	Grand Index
35	f	0-12 years	0.154	738.262	85	Grand Index
35	f	0-12 years	0.155	738.465	85	Grand Index
35	f	0-12 years	0.156	738.668	85	Grand Index
35	f	0-12 years	0.157	738.871	85	Grand Index
35	f	0-12 years	0.158	739.37	85	Grand Index
35	f	0-12 years	0.159	740.385	85	Grand Index
35	f	0-12 years	0.16	741.4	85	Grand Index
35	f	0-12 years	0.161	742.415	85	Grand Index
35	f	0-12 years	0.162	743.43	85	Grand Index
35	f	0-12 years	0.163	744.089	85	Grand Index
35	f	0-12 years	0.164	744.292	85	Grand Index
35	f	0-12 years	0.165	744.495	85	Grand Index
35	f	0-12 years	0.166	744.698	85	Grand Index
35	f	0-12 years	0.167	744.901	86	Grand Index
35	f	0-12 years	0.168	745.104	86	Grand Index
35	f	0-12 years	0.169	745.307	86	Grand Index
35	f	0-12 years	0.17	745.51	86	Grand Index

Age	Gender	Education	Percentile	Raw Score	Norm Score	Assessment
35	f	0-12 years	0.171	745.713	86	Grand Index
35	f	0-12 years	0.172	745.916	86	Grand Index
35	f	0-12 years	0.177	746	86	Grand Index
35	f	0-12 years	0.178	746.0582609	86	Grand Index
35	f	0-12 years	0.179	746.1465217	86	Grand Index
35	f	0-12 years	0.18	746.2347826	86	Grand Index
35	f	0-12 years	0.181	746.3230435	86	Grand Index
35	f	0-12 years	0.182	746.4113043	86	Grand Index
35	f	0-12 years	0.183	746.5689113	86	Grand Index
35	f	0-12 years	0.184	746.7516505	86	Grand Index
35	f	0-12 years	0.185	746.9343896	87	Grand Index
35	f	0-12 years	0.186	747.1171287	87	Grand Index
35	f	0-12 years	0.187	747.2998679	87	Grand Index
35	f	0-12 years	0.188	747.6080394	87	Grand Index
35	f	0-12 years	0.189	747.9460394	87	Grand Index
35	f	0-12 years	0.19	748.2840394	87	Grand Index
35	f	0-12 years	0.191	748.6220394	87	Grand Index
35	f	0-12 years	0.192	748.9600394	87	Grand Index
35	f	0-12 years	0.193	749.179	87	Grand Index
35	f	0-12 years	0.194	749.382	87	Grand Index
35	f	0-12 years	0.195	749.585	87	Grand Index
35	f	0-12 years	0.196	749.788	87	Grand Index
35	f	0-12 years	0.197	749.991	87	Grand Index
35	f	0-12 years	0.198	750.388	87	Grand Index
35	f	0-12 years	0.199	750.794	87	Grand Index
35	f	0-12 years	0.2	751.2	87	Grand Index
35	f	0-12 years	0.201	751.606	87	Grand Index
35	f	0-12 years	0.202	752.018	87	Grand Index
35	f	0-12 years	0.203	752.627	88	Grand Index
35	f	0-12 years	0.204	753.236	88	Grand Index
35	f	0-12 years	0.205	753.845	88	Grand Index
35	f	0-12 years	0.206	754.454	88	Grand Index
35	f	0-12 years	0.207	755.021	88	Grand Index
35	f	0-12 years	0.208	755.224	88	Grand Index
35	f	0-12 years	0.209	755.427	88	Grand Index
35	f	0-12 years	0.21	755.63	88	Grand Index
35	f	0-12 years	0.211	755.833	88	Grand Index
35	f	0-12 years	0.212	756.072	88	Grand Index
35	f	0-12 years	0.213	756.478	88	Grand Index
35	f	0-12 years	0.214	756.884	88	Grand Index
35	f	0-12 years	0.215	757.29	88	Grand Index
35	f	0-12 years	0.216	757.696	88	Grand Index
35	f	0-12 years	0.221	758	88	Grand Index
35	f	0-12 years	0.222	758.066	89	Grand Index
35	f	0-12 years	0.223	758.269	89	Grand Index
35	f	0-12 years	0.224	758.472	89	Grand Index
35	f	0-12 years	0.225	758.675	89	Grand Index
35	f	0-12 years	0.226	758.878	89	Grand Index
35	f	0-12 years	0.227	759.162	89	Grand Index
35	f	0-12 years	0.228	759.568	89	Grand Index
35	f	0-12 years	0.229	759.974	89	Grand Index
35	f	0-12 years	0.23	760.38	89	Grand Index
35	f	0-12 years	0.231	760.786	89	Grand Index
35	f	0-12 years	0.232	761.096	89	Grand Index
35	f	0-12 years	0.233	761.299	89	Grand Index
35	f	0-12 years	0.234	761.502	89	Grand Index

Age	Gender	Education	Percentile	Raw Score	Norm Score	Assessment
35	f	0-12 years	0.235	761.705	89	Grand Index
35	f	0-12 years	0.236	761.908	89	Grand Index
35	f	0-12 years	0.237	762.111	89	Grand Index
35	f	0-12 years	0.238	762.314	89	Grand Index
35	f	0-12 years	0.239	762.517	89	Grand Index
35	f	0-12 years	0.24	762.72	89	Grand Index
35	f	0-12 years	0.241	762.923	89	Grand Index
35	f	0-12 years	0.242	763.378	90	Grand Index
35	f	0-12 years	0.243	763.987	90	Grand Index
35	f	0-12 years	0.244	764.596	90	Grand Index
35	f	0-12 years	0.245	765.205	90	Grand Index
35	f	0-12 years	0.246	765.814	90	Grand Index
35	f	0-12 years	0.247	766.141	90	Grand Index
35	f	0-12 years	0.248	766.344	90	Grand Index
35	f	0-12 years	0.249	766.547	90	Grand Index
35	f	0-12 years	0.25	766.75	90	Grand Index
35	f	0-12 years	0.251	766.953	90	Grand Index
35	f	0-12 years	0.252	767.4103645	90	Grand Index
35	f	0-12 years	0.253	767.9443645	90	Grand Index
35	f	0-12 years	0.254	768.4783645	90	Grand Index
35	f	0-12 years	0.255	769.0123645	90	Grand Index
35	f	0-12 years	0.256	769.5463645	90	Grand Index
35	f	0-12 years	0.257	769.6937192	90	Grand Index
35	f	0-12 years	0.258	769.7687192	90	Grand Index
35	f	0-12 years	0.259	769.8437192	90	Grand Index
35	f	0-12 years	0.26	769.9187192	90	Grand Index
35	f	0-12 years	0.261	769.9937192	90	Grand Index
35	f	0-12 years	0.262	770.186	90	Grand Index
35	f	0-12 years	0.263	770.389	90	Grand Index
35	f	0-12 years	0.264	770.592	91	Grand Index
35	f	0-12 years	0.265	770.795	91	Grand Index
35	f	0-12 years	0.266	770.998	91	Grand Index
35	f	0-12 years	0.27	771	91	Grand Index
35	f	0-12 years	0.271	771.0164878	91	Grand Index
35	f	0-12 years	0.272	771.2739512	91	Grand Index
35	f	0-12 years	0.273	771.5314146	91	Grand Index
35	f	0-12 years	0.274	771.788878	91	Grand Index
35	f	0-12 years	0.275	772.0463415	91	Grand Index
35	f	0-12 years	0.276	772.2887805	91	Grand Index
35	f	0-12 years	0.277	772.4373171	91	Grand Index
35	f	0-12 years	0.278	772.5858537	91	Grand Index
35	f	0-12 years	0.279	772.7343902	91	Grand Index
35	f	0-12 years	0.28	772.8829268	91	Grand Index
35	f	0-12 years	0.285	773	91	Grand Index
35	f	0-12 years	0.286	773.116	92	Grand Index
35	f	0-12 years	0.287	773.522	92	Grand Index
35	f	0-12 years	0.288	773.928	92	Grand Index
35	f	0-12 years	0.289	774.334	92	Grand Index
35	f	0-12 years	0.29	774.74	92	Grand Index
35	f	0-12 years	0.295	775	92	Grand Index
35	f	0-12 years	0.296	775.088	92	Grand Index
35	f	0-12 years	0.297	775.291	92	Grand Index
35	f	0-12 years	0.298	775.494	92	Grand Index
35	f	0-12 years	0.299	775.697	92	Grand Index
35	f	0-12 years	0.3	775.9	92	Grand Index
35	f	0-12 years	0.301	776.309	92	Grand Index

Age	Gender	Education	Percentile	Raw Score	Norm Score	Assessment
35	f	0-12 years	0.302	776.918	92	Grand Index
35	f	0-12 years	0.303	777.527	92	Grand Index
35	f	0-12 years	0.304	778.136	92	Grand Index
35	f	0-12 years	0.305	778.745	92	Grand Index
35	f	0-12 years	0.306	779.118	92	Grand Index
35	f	0-12 years	0.307	779.321	92	Grand Index
35	f	0-12 years	0.308	779.524	92	Grand Index
35	f	0-12 years	0.309	779.727	93	Grand Index
35	f	0-12 years	0.31	779.93	93	Grand Index
35	f	0-12 years	0.311	780.266	93	Grand Index
35	f	0-12 years	0.312	780.672	93	Grand Index
35	f	0-12 years	0.313	781.078	93	Grand Index
35	f	0-12 years	0.314	781.484	93	Grand Index
35	f	0-12 years	0.315	781.89	93	Grand Index
35	f	0-12 years	0.32	782	93	Grand Index
35	f	0-12 years	0.321	782.815	93	Grand Index
35	f	0-12 years	0.322	783.83	93	Grand Index
35	f	0-12 years	0.323	784.845	93	Grand Index
35	f	0-12 years	0.324	785.86	93	Grand Index
35	f	0-12 years	0.325	786.875	93	Grand Index
35	f	0-12 years	0.326	787.178	93	Grand Index
35	f	0-12 years	0.327	787.381	93	Grand Index
35	f	0-12 years	0.328	787.584	93	Grand Index
35	f	0-12 years	0.329	787.787	93	Grand Index
35	f	0-12 years	0.33	787.99	93	Grand Index
35	f	0-12 years	0.331	788.193	93	Grand Index
35	f	0-12 years	0.332	788.396	93	Grand Index
35	f	0-12 years	0.333	788.599	94	Grand Index
35	f	0-12 years	0.334	788.802	94	Grand Index
35	f	0-12 years	0.335	789.008916	94	Grand Index
35	f	0-12 years	0.336	789.3709036	94	Grand Index
35	f	0-12 years	0.337	789.7328912	94	Grand Index
35	f	0-12 years	0.338	790.0948788	94	Grand Index
35	f	0-12 years	0.339	790.4568664	94	Grand Index
35	f	0-12 years	0.34	790.7875264	94	Grand Index
35	f	0-12 years	0.341	790.8315388	94	Grand Index
35	f	0-12 years	0.342	790.8755512	94	Grand Index
35	f	0-12 years	0.343	790.9195636	94	Grand Index
35	f	0-12 years	0.344	790.963576	94	Grand Index
35	f	0-12 years	0.349	791	94	Grand Index
35	f	0-12 years	0.35	791.05	94	Grand Index
35	f	0-12 years	0.351	791.253	94	Grand Index
35	f	0-12 years	0.352	791.456	94	Grand Index
35	f	0-12 years	0.353	791.659	94	Grand Index
35	f	0-12 years	0.354	791.862	94	Grand Index
35	f	0-12 years	0.355	792.065	94	Grand Index
35	f	0-12 years	0.356	792.268	94	Grand Index
35	f	0-12 years	0.357	792.471	95	Grand Index
35	f	0-12 years	0.358	792.674	95	Grand Index
35	f	0-12 years	0.359	792.877	95	Grand Index
35	f	0-12 years	0.36	793.08	95	Grand Index
35	f	0-12 years	0.361	793.283	95	Grand Index
35	f	0-12 years	0.362	793.486	95	Grand Index
35	f	0-12 years	0.363	793.689	95	Grand Index
35	f	0-12 years	0.364	793.892	95	Grand Index
35	f	0-12 years	0.365	794.19	95	Grand Index

Age	Gender	Education	Percentile	Raw Score	Norm Score	Assessment
35	f	0-12 years	0.366	794.596	95	Grand Index
35	f	0-12 years	0.367	795.002	95	Grand Index
35	f	0-12 years	0.368	795.408	95	Grand Index
35	f	0-12 years	0.369	795.814	95	Grand Index
35	f	0-12 years	0.37	796.11	95	Grand Index
35	f	0-12 years	0.371	796.313	95	Grand Index
35	f	0-12 years	0.372	796.516	95	Grand Index
35	f	0-12 years	0.373	796.719	95	Grand Index
35	f	0-12 years	0.374	796.922	95	Grand Index
35	f	0-12 years	0.379	797	95	Grand Index
35	f	0-12 years	0.38	797.0830966	95	Grand Index
35	f	0-12 years	0.381	797.2035867	95	Grand Index
35	f	0-12 years	0.382	797.3240768	95	Grand Index
35	f	0-12 years	0.383	797.4445668	96	Grand Index
35	f	0-12 years	0.384	797.5650569	96	Grand Index
35	f	0-12 years	0.385	797.6565474	96	Grand Index
35	f	0-12 years	0.386	797.7390573	96	Grand Index
35	f	0-12 years	0.387	797.8215672	96	Grand Index
35	f	0-12 years	0.388	797.9040771	96	Grand Index
35	f	0-12 years	0.389	797.9865871	96	Grand Index
35	f	0-12 years	0.394	798	96	Grand Index
35	f	0-12 years	0.395	798.1838034	96	Grand Index
35	f	0-12 years	0.396	798.3854904	96	Grand Index
35	f	0-12 years	0.397	798.5871774	96	Grand Index
35	f	0-12 years	0.398	798.7888644	96	Grand Index
35	f	0-12 years	0.399	798.9905514	96	Grand Index
35	f	0-12 years	0.4	798.9948256	96	Grand Index
35	f	0-12 years	0.401	798.9961386	96	Grand Index
35	f	0-12 years	0.402	798.9974516	96	Grand Index
35	f	0-12 years	0.403	798.9987646	96	Grand Index
35	f	0-12 years	0.404	799.012	96	Grand Index
35	f	0-12 years	0.405	799.215	96	Grand Index
35	f	0-12 years	0.406	799.418	96	Grand Index
35	f	0-12 years	0.407	799.621	96	Grand Index
35	f	0-12 years	0.408	799.824	97	Grand Index
35	f	0-12 years	0.409	800.0059621	97	Grand Index
35	f	0-12 years	0.41	800.0507884	97	Grand Index
35	f	0-12 years	0.411	800.0956146	97	Grand Index
35	f	0-12 years	0.412	800.1404409	97	Grand Index
35	f	0-12 years	0.413	800.1852672	97	Grand Index
35	f	0-12 years	0.414	800.2535446	97	Grand Index
35	f	0-12 years	0.415	800.4117184	97	Grand Index
35	f	0-12 years	0.416	800.5698921	97	Grand Index
35	f	0-12 years	0.417	800.7280658	97	Grand Index
35	f	0-12 years	0.418	800.8862396	97	Grand Index
35	f	0-12 years	0.423	801	97	Grand Index
35	f	0-12 years	0.424	801.072	97	Grand Index
35	f	0-12 years	0.425	801.275	97	Grand Index
35	f	0-12 years	0.426	801.478	97	Grand Index
35	f	0-12 years	0.427	801.681	97	Grand Index
35	f	0-12 years	0.428	801.884	97	Grand Index
35	f	0-12 years	0.429	802.087	97	Grand Index
35	f	0-12 years	0.43	802.29	97	Grand Index
35	f	0-12 years	0.431	802.493	97	Grand Index
35	f	0-12 years	0.432	802.696	97	Grand Index
35	f	0-12 years	0.433	802.899	97	Grand Index

Age	Gender	Education	Percentile	Raw Score	Norm Score	Assessment
35	f	0-12 years	0.438	803	98	Grand Index
35	f	0-12 years	0.439	803.0194742	98	Grand Index
35	f	0-12 years	0.44	803.0532629	98	Grand Index
35	f	0-12 years	0.441	803.0870515	98	Grand Index
35	f	0-12 years	0.442	803.1208402	98	Grand Index
35	f	0-12 years	0.443	803.1546288	98	Grand Index
35	f	0-12 years	0.444	803.4084756	98	Grand Index
35	f	0-12 years	0.445	803.780687	98	Grand Index
35	f	0-12 years	0.446	804.1528983	98	Grand Index
35	f	0-12 years	0.447	804.5251097	98	Grand Index
35	f	0-12 years	0.448	804.897321	98	Grand Index
35	f	0-12 years	0.449	805.294	98	Grand Index
35	f	0-12 years	0.45	805.7	98	Grand Index
35	f	0-12 years	0.451	806.106	98	Grand Index
35	f	0-12 years	0.452	806.512	98	Grand Index
35	f	0-12 years	0.453	806.918	98	Grand Index
35	f	0-12 years	0.454	807.162	98	Grand Index
35	f	0-12 years	0.455	807.365	98	Grand Index
35	f	0-12 years	0.456	807.568	98	Grand Index
35	f	0-12 years	0.457	807.771	98	Grand Index
35	f	0-12 years	0.458	807.974	98	Grand Index
35	f	0-12 years	0.463	808	99	Grand Index
35	f	0-12 years	0.464	808.1536	99	Grand Index
35	f	0-12 years	0.465	808.316	99	Grand Index
35	f	0-12 years	0.466	808.4784	99	Grand Index
35	f	0-12 years	0.467	808.6408	99	Grand Index
35	f	0-12 years	0.468	808.8008	99	Grand Index
35	f	0-12 years	0.469	808.8414	99	Grand Index
35	f	0-12 years	0.47	808.882	99	Grand Index
35	f	0-12 years	0.471	808.9226	99	Grand Index
35	f	0-12 years	0.472	808.9632	99	Grand Index
35	f	0-12 years	0.477	809	99	Grand Index
35	f	0-12 years	0.478	809.034	99	Grand Index
35	f	0-12 years	0.479	809.237	99	Grand Index
35	f	0-12 years	0.48	809.44	99	Grand Index
35	f	0-12 years	0.481	809.643	99	Grand Index
35	f	0-12 years	0.482	809.846	99	Grand Index
35	f	0-12 years	0.483	810.098	99	Grand Index
35	f	0-12 years	0.484	810.504	99	Grand Index
35	f	0-12 years	0.485	810.91	99	Grand Index
35	f	0-12 years	0.486	811.316	99	Grand Index
35	f	0-12 years	0.487	811.722	100	Grand Index
35	f	0-12 years	0.488	812.064	100	Grand Index
35	f	0-12 years	0.489	812.267	100	Grand Index
35	f	0-12 years	0.49	812.47	100	Grand Index
35	f	0-12 years	0.491	812.673	100	Grand Index
35	f	0-12 years	0.492	812.876	100	Grand Index
35	f	0-12 years	0.497	813	100	Grand Index
35	f	0-12 years	0.498	813.0792637	100	Grand Index
35	f	0-12 years	0.499	813.2504394	100	Grand Index
35	f	0-12 years	0.5	813.4216152	100	Grand Index
35	f	0-12 years	0.501	813.592791	100	Grand Index
35	f	0-12 years	0.502	813.7639667	100	Grand Index
35	f	0-12 years	0.503	814.1873183	100	Grand Index
35	f	0-12 years	0.504	814.8281425	100	Grand Index
35	f	0-12 years	0.505	815.4689667	100	Grand Index

Age	Gender	Education	Percentile	Raw Score	Norm Score	Assessment
35	f	0-12 years	0.506	816.109791	100	Grand Index
35	f	0-12 years	0.507	816.7506152	100	Grand Index
35	f	0-12 years	0.508	817.248	100	Grand Index
35	f	0-12 years	0.509	817.654	100	Grand Index
35	f	0-12 years	0.51	818.06	100	Grand Index
35	f	0-12 years	0.511	818.466	100	Grand Index
35	f	0-12 years	0.512	818.872	100	Grand Index
35	f	0-12 years	0.527	819	101	Grand Index
35	f	0-12 years	0.528	819.184	101	Grand Index
35	f	0-12 years	0.529	819.387	101	Grand Index
35	f	0-12 years	0.53	819.59	101	Grand Index
35	f	0-12 years	0.531	819.793	101	Grand Index
35	f	0-12 years	0.532	819.996	101	Grand Index
35	f	0-12 years	0.533	820.199	101	Grand Index
35	f	0-12 years	0.534	820.402	101	Grand Index
35	f	0-12 years	0.535	820.605	101	Grand Index
35	f	0-12 years	0.536	820.808	101	Grand Index
35	f	0-12 years	0.541	821	102	Grand Index
35	f	0-12 years	0.542	821.026	102	Grand Index
35	f	0-12 years	0.543	821.229	102	Grand Index
35	f	0-12 years	0.544	821.432	102	Grand Index
35	f	0-12 years	0.545	821.635	102	Grand Index
35	f	0-12 years	0.546	821.838	102	Grand Index
35	f	0-12 years	0.547	822.041	102	Grand Index
35	f	0-12 years	0.548	822.244	102	Grand Index
35	f	0-12 years	0.549	822.447	102	Grand Index
35	f	0-12 years	0.55	822.65	102	Grand Index
35	f	0-12 years	0.551	822.853	102	Grand Index
35	f	0-12 years	0.552	823.056	102	Grand Index
35	f	0-12 years	0.553	823.259	102	Grand Index
35	f	0-12 years	0.554	823.462	102	Grand Index
35	f	0-12 years	0.555	823.665	102	Grand Index
35	f	0-12 years	0.556	823.868	102	Grand Index
35	f	0-12 years	0.561	824	102	Grand Index
35	f	0-12 years	0.562	824.0078597	102	Grand Index
35	f	0-12 years	0.563	824.0264123	102	Grand Index
35	f	0-12 years	0.564	824.0449649	102	Grand Index
35	f	0-12 years	0.565	824.0635175	102	Grand Index
35	f	0-12 years	0.566	824.0820701	102	Grand Index
35	f	0-12 years	0.567	824.1831615	103	Grand Index
35	f	0-12 years	0.568	824.3676089	103	Grand Index
35	f	0-12 years	0.569	824.5520563	103	Grand Index
35	f	0-12 years	0.57	824.7365037	103	Grand Index
35	f	0-12 years	0.571	824.9209511	103	Grand Index
35	f	0-12 years	0.576	825	103	Grand Index
35	f	0-12 years	0.577	825.262	103	Grand Index
35	f	0-12 years	0.578	825.668	103	Grand Index
35	f	0-12 years	0.579	826.074	103	Grand Index
35	f	0-12 years	0.58	826.48	103	Grand Index
35	f	0-12 years	0.581	826.886	103	Grand Index
35	f	0-12 years	0.582	827.146	103	Grand Index
35	f	0-12 years	0.583	827.349	103	Grand Index
35	f	0-12 years	0.584	827.552	103	Grand Index
35	f	0-12 years	0.585	827.755	103	Grand Index
35	f	0-12 years	0.586	827.958	103	Grand Index
35	f	0-12 years	0.587	828.3585937	103	Grand Index

Age	Gender	Education	Percentile	Raw Score	Norm Score	Assessment
35	f	0-12 years	0.588	828.8107335	103	Grand Index
35	f	0-12 years	0.589	829.2628734	103	Grand Index
35	f	0-12 years	0.59	829.7150133	103	Grand Index
35	f	0-12 years	0.591	830.1671531	103	Grand Index
35	f	0-12 years	0.592	830.2618281	103	Grand Index
35	f	0-12 years	0.593	830.3016647	104	Grand Index
35	f	0-12 years	0.594	830.3415013	104	Grand Index
35	f	0-12 years	0.595	830.3813379	104	Grand Index
35	f	0-12 years	0.596	830.4211745	104	Grand Index
35	f	0-12 years	0.597	830.5336353	104	Grand Index
35	f	0-12 years	0.598	830.6506588	104	Grand Index
35	f	0-12 years	0.599	830.7676824	104	Grand Index
35	f	0-12 years	0.6	830.8847059	104	Grand Index
35	f	0-12 years	0.601	831.006	104	Grand Index
35	f	0-12 years	0.602	831.412	104	Grand Index
35	f	0-12 years	0.603	831.818	104	Grand Index
35	f	0-12 years	0.604	832.224	104	Grand Index
35	f	0-12 years	0.605	832.63	104	Grand Index
35	f	0-12 years	0.61	833	104	Grand Index
35	f	0-12 years	0.611	833.0131809	104	Grand Index
35	f	0-12 years	0.612	833.0942631	104	Grand Index
35	f	0-12 years	0.613	833.1753453	104	Grand Index
35	f	0-12 years	0.614	833.2564276	104	Grand Index
35	f	0-12 years	0.615	833.3375098	104	Grand Index
35	f	0-12 years	0.616	833.4282477	104	Grand Index
35	f	0-12 years	0.617	833.5501655	104	Grand Index
35	f	0-12 years	0.618	833.6720832	105	Grand Index
35	f	0-12 years	0.619	833.794001	105	Grand Index
35	f	0-12 years	0.62	833.9159188	105	Grand Index
35	f	0-12 years	0.621	834.0003066	105	Grand Index
35	f	0-12 years	0.622	834.0012946	105	Grand Index
35	f	0-12 years	0.623	834.0022827	105	Grand Index
35	f	0-12 years	0.624	834.0032707	105	Grand Index
35	f	0-12 years	0.625	834.0042587	105	Grand Index
35	f	0-12 years	0.626	834.0824874	105	Grand Index
35	f	0-12 years	0.627	834.2844994	105	Grand Index
35	f	0-12 years	0.628	834.4865114	105	Grand Index
35	f	0-12 years	0.629	834.6885234	105	Grand Index
35	f	0-12 years	0.63	834.8905354	105	Grand Index
35	f	0-12 years	0.631	835.279	105	Grand Index
35	f	0-12 years	0.632	835.888	105	Grand Index
35	f	0-12 years	0.633	836.497	105	Grand Index
35	f	0-12 years	0.634	837.106	105	Grand Index
35	f	0-12 years	0.635	837.715	105	Grand Index
35	f	0-12 years	0.636	838.108	105	Grand Index
35	f	0-12 years	0.637	838.311	105	Grand Index
35	f	0-12 years	0.638	838.514	105	Grand Index
35	f	0-12 years	0.639	838.717	105	Grand Index
35	f	0-12 years	0.64	838.92	105	Grand Index
35	f	0-12 years	0.641	839.369	105	Grand Index
35	f	0-12 years	0.642	839.978	105	Grand Index
35	f	0-12 years	0.643	840.587	105	Grand Index
35	f	0-12 years	0.644	841.196	106	Grand Index
35	f	0-12 years	0.645	841.805	106	Grand Index
35	f	0-12 years	0.665	842	106	Grand Index
35	f	0-12 years	0.666	842.198	106	Grand Index

Age	Gender	Education	Percentile	Raw Score	Norm Score	Assessment
35	f	0-12 years	0.667	842.401	106	Grand Index
35	f	0-12 years	0.668	842.604	107	Grand Index
35	f	0-12 years	0.669	842.807	107	Grand Index
35	f	0-12 years	0.67	843.01	107	Grand Index
35	f	0-12 years	0.671	843.213	107	Grand Index
35	f	0-12 years	0.672	843.416	107	Grand Index
35	f	0-12 years	0.673	843.619	107	Grand Index
35	f	0-12 years	0.674	843.822	107	Grand Index
35	f	0-12 years	0.675	844.0471767	107	Grand Index
35	f	0-12 years	0.676	844.4302514	107	Grand Index
35	f	0-12 years	0.677	844.8133262	107	Grand Index
35	f	0-12 years	0.678	845.1964009	107	Grand Index
35	f	0-12 years	0.679	845.5794756	107	Grand Index
35	f	0-12 years	0.68	845.891585	107	Grand Index
35	f	0-12 years	0.681	845.9145102	107	Grand Index
35	f	0-12 years	0.682	845.9374355	107	Grand Index
35	f	0-12 years	0.683	845.9603607	107	Grand Index
35	f	0-12 years	0.684	845.983286	107	Grand Index
35	f	0-12 years	0.685	846.055	107	Grand Index
35	f	0-12 years	0.686	846.258	107	Grand Index
35	f	0-12 years	0.687	846.461	107	Grand Index
35	f	0-12 years	0.688	846.664	107	Grand Index
35	f	0-12 years	0.689	846.867	107	Grand Index
35	f	0-12 years	0.69	847.21	107	Grand Index
35	f	0-12 years	0.691	847.819	107	Grand Index
35	f	0-12 years	0.692	848.428	108	Grand Index
35	f	0-12 years	0.693	849.037	108	Grand Index
35	f	0-12 years	0.694	849.646	108	Grand Index
35	f	0-12 years	0.695	850.085	108	Grand Index
35	f	0-12 years	0.696	850.288	108	Grand Index
35	f	0-12 years	0.697	850.491	108	Grand Index
35	f	0-12 years	0.698	850.694	108	Grand Index
35	f	0-12 years	0.699	850.897	108	Grand Index
35	f	0-12 years	0.7	851.2	108	Grand Index
35	f	0-12 years	0.701	851.606	108	Grand Index
35	f	0-12 years	0.702	852.012	108	Grand Index
35	f	0-12 years	0.703	852.418	108	Grand Index
35	f	0-12 years	0.704	852.824	108	Grand Index
35	f	0-12 years	0.705	853.345	108	Grand Index
35	f	0-12 years	0.706	853.954	108	Grand Index
35	f	0-12 years	0.707	854.563	108	Grand Index
35	f	0-12 years	0.708	855.172	108	Grand Index
35	f	0-12 years	0.709	855.781	108	Grand Index
35	f	0-12 years	0.71	856.39	108	Grand Index
35	f	0-12 years	0.711	856.999	108	Grand Index
35	f	0-12 years	0.712	857.608	108	Grand Index
35	f	0-12 years	0.713	858.217	108	Grand Index
35	f	0-12 years	0.714	858.826	108	Grand Index
35	f	0-12 years	0.715	859.145	109	Grand Index
35	f	0-12 years	0.716	859.348	109	Grand Index
35	f	0-12 years	0.717	859.551	109	Grand Index
35	f	0-12 years	0.718	859.754	109	Grand Index
35	f	0-12 years	0.719	859.957	109	Grand Index
35	f	0-12 years	0.72	860.16	109	Grand Index
35	f	0-12 years	0.721	860.363	109	Grand Index
35	f	0-12 years	0.722	860.566	109	Grand Index

Age	Gender	Education	Percentile	Raw Score	Norm Score	Assessment
35	f	0-12 years	0.723	860.769	109	Grand Index
35	f	0-12 years	0.724	860.972	109	Grand Index
35	f	0-12 years	0.725	861.175	109	Grand Index
35	f	0-12 years	0.726	861.378	109	Grand Index
35	f	0-12 years	0.727	861.581	109	Grand Index
35	f	0-12 years	0.728	861.784	109	Grand Index
35	f	0-12 years	0.729	861.987	109	Grand Index
35	f	0-12 years	0.743	862	110	Grand Index
35	f	0-12 years	0.744	862.032	110	Grand Index
35	f	0-12 years	0.745	862.235	110	Grand Index
35	f	0-12 years	0.746	862.438	110	Grand Index
35	f	0-12 years	0.747	862.641	110	Grand Index
35	f	0-12 years	0.748	862.844	110	Grand Index
35	f	0-12 years	0.749	863.059838	110	Grand Index
35	f	0-12 years	0.75	863.3182873	110	Grand Index
35	f	0-12 years	0.751	863.5767366	110	Grand Index
35	f	0-12 years	0.752	863.8351859	110	Grand Index
35	f	0-12 years	0.753	864.0936352	110	Grand Index
35	f	0-12 years	0.754	864.296155	110	Grand Index
35	f	0-12 years	0.755	864.3714802	110	Grand Index
35	f	0-12 years	0.756	864.4468055	110	Grand Index
35	f	0-12 years	0.757	864.5221308	110	Grand Index
35	f	0-12 years	0.758	864.5974561	110	Grand Index
35	f	0-12 years	0.759	864.6484001	111	Grand Index
35	f	0-12 years	0.76	864.6594474	111	Grand Index
35	f	0-12 years	0.761	864.6704948	111	Grand Index
35	f	0-12 years	0.762	864.6815421	111	Grand Index
35	f	0-12 years	0.763	864.6925895	111	Grand Index
35	f	0-12 years	0.764	864.7363417	111	Grand Index
35	f	0-12 years	0.765	864.8195531	111	Grand Index
35	f	0-12 years	0.766	864.9027644	111	Grand Index
35	f	0-12 years	0.767	864.9859758	111	Grand Index
35	f	0-12 years	0.768	865.0691872	111	Grand Index
35	f	0-12 years	0.769	865.3109247	111	Grand Index
35	f	0-12 years	0.77	865.6948915	111	Grand Index
35	f	0-12 years	0.771	866.0788582	111	Grand Index
35	f	0-12 years	0.772	866.4628249	111	Grand Index
35	f	0-12 years	0.773	866.8467916	111	Grand Index
35	f	0-12 years	0.774	867.2399177	111	Grand Index
35	f	0-12 years	0.775	867.6391251	111	Grand Index
35	f	0-12 years	0.776	868.0383325	111	Grand Index
35	f	0-12 years	0.777	868.4375398	111	Grand Index
35	f	0-12 years	0.778	868.8367472	111	Grand Index
35	f	0-12 years	0.779	869.0958851	112	Grand Index
35	f	0-12 years	0.78	869.2875442	112	Grand Index
35	f	0-12 years	0.781	869.4792034	112	Grand Index
35	f	0-12 years	0.782	869.6708625	112	Grand Index
35	f	0-12 years	0.783	869.8625216	112	Grand Index
35	f	0-12 years	0.784	869.9242502	112	Grand Index
35	f	0-12 years	0.785	869.9423837	112	Grand Index
35	f	0-12 years	0.786	869.9605172	112	Grand Index
35	f	0-12 years	0.787	869.9786507	112	Grand Index
35	f	0-12 years	0.788	869.9967842	112	Grand Index
35	f	0-12 years	0.789	870.167	112	Grand Index
35	f	0-12 years	0.79	870.37	112	Grand Index
35	f	0-12 years	0.791	870.573	112	Grand Index

Age	Gender	Education	Percentile	Raw Score	Norm Score	Assessment
35	f	0-12 years	0.792	870.776	112	Grand Index
35	f	0-12 years	0.793	870.979	112	Grand Index
35	f	0-12 years	0.794	871.182	112	Grand Index
35	f	0-12 years	0.795	871.385	112	Grand Index
35	f	0-12 years	0.796	871.588	112	Grand Index
35	f	0-12 years	0.797	871.791	112	Grand Index
35	f	0-12 years	0.798	871.994	113	Grand Index
35	f	0-12 years	0.799	872.197	113	Grand Index
35	f	0-12 years	0.8	872.4	113	Grand Index
35	f	0-12 years	0.801	872.603	113	Grand Index
35	f	0-12 years	0.802	872.806	113	Grand Index
35	f	0-12 years	0.803	873.009	113	Grand Index
35	f	0-12 years	0.804	873.212	113	Grand Index
35	f	0-12 years	0.805	873.415	113	Grand Index
35	f	0-12 years	0.806	873.618	113	Grand Index
35	f	0-12 years	0.807	873.821	113	Grand Index
35	f	0-12 years	0.808	874.024	113	Grand Index
35	f	0-12 years	0.809	874.227	113	Grand Index
35	f	0-12 years	0.81	874.43	113	Grand Index
35	f	0-12 years	0.811	874.633	113	Grand Index
35	f	0-12 years	0.812	874.836	113	Grand Index
35	f	0-12 years	0.813	875.195	113	Grand Index
35	f	0-12 years	0.814	876.21	113	Grand Index
35	f	0-12 years	0.815	877.225	113	Grand Index
35	f	0-12 years	0.816	878.24	114	Grand Index
35	f	0-12 years	0.817	879.255	114	Grand Index
35	f	0-12 years	0.818	880.108	114	Grand Index
35	f	0-12 years	0.819	880.514	114	Grand Index
35	f	0-12 years	0.82	880.92	114	Grand Index
35	f	0-12 years	0.821	881.326	114	Grand Index
35	f	0-12 years	0.822	881.732	114	Grand Index
35	f	0-12 years	0.823	882.069	114	Grand Index
35	f	0-12 years	0.824	882.272	114	Grand Index
35	f	0-12 years	0.825	882.475	114	Grand Index
35	f	0-12 years	0.826	882.678	114	Grand Index
35	f	0-12 years	0.827	882.881	114	Grand Index
35	f	0-12 years	0.832	883	114	Grand Index
35	f	0-12 years	0.833	883.0952884	114	Grand Index
35	f	0-12 years	0.834	883.2906779	115	Grand Index
35	f	0-12 years	0.835	883.4860673	115	Grand Index
35	f	0-12 years	0.836	883.6814567	115	Grand Index
35	f	0-12 years	0.837	883.8768462	115	Grand Index
35	f	0-12 years	0.838	883.9667834	115	Grand Index
35	f	0-12 years	0.839	883.974394	115	Grand Index
35	f	0-12 years	0.84	883.9820046	115	Grand Index
35	f	0-12 years	0.841	883.9896151	115	Grand Index
35	f	0-12 years	0.842	883.9972257	115	Grand Index
35	f	0-12 years	0.843	884.258	115	Grand Index
35	f	0-12 years	0.844	884.664	115	Grand Index
35	f	0-12 years	0.845	885.07	115	Grand Index
35	f	0-12 years	0.846	885.476	115	Grand Index
35	f	0-12 years	0.847	885.882	115	Grand Index
35	f	0-12 years	0.848	886.0106404	115	Grand Index
35	f	0-12 years	0.849	886.0256404	115	Grand Index
35	f	0-12 years	0.85	886.0406404	116	Grand Index
35	f	0-12 years	0.851	886.0556404	116	Grand Index

Age	Gender	Education	Percentile	Raw Score	Norm Score	Assessment
35	f	0-12 years	0.852	886.0706404	116	Grand Index
35	f	0-12 years	0.853	886.2211429	116	Grand Index
35	f	0-12 years	0.854	886.4091429	116	Grand Index
35	f	0-12 years	0.855	886.5971429	116	Grand Index
35	f	0-12 years	0.856	886.7851429	116	Grand Index
35	f	0-12 years	0.857	886.9731429	116	Grand Index
35	f	0-12 years	0.858	887.4460804	116	Grand Index
35	f	0-12 years	0.859	887.9665075	116	Grand Index
35	f	0-12 years	0.86	888.4869346	116	Grand Index
35	f	0-12 years	0.861	889.0073617	116	Grand Index
35	f	0-12 years	0.862	889.5277888	116	Grand Index
35	f	0-12 years	0.863	889.6461447	116	Grand Index
35	f	0-12 years	0.864	889.7347176	116	Grand Index
35	f	0-12 years	0.865	889.8232905	117	Grand Index
35	f	0-12 years	0.866	889.9118634	117	Grand Index
35	f	0-12 years	0.867	890.003	117	Grand Index
35	f	0-12 years	0.868	890.612	117	Grand Index
35	f	0-12 years	0.869	891.221	117	Grand Index
35	f	0-12 years	0.87	891.83	117	Grand Index
35	f	0-12 years	0.871	892.439	117	Grand Index
35	f	0-12 years	0.876	893	117	Grand Index
35	f	0-12 years	0.877	893.279	117	Grand Index
35	f	0-12 years	0.878	895.106	117	Grand Index
35	f	0-12 years	0.879	896.933	118	Grand Index
35	f	0-12 years	0.88	898.76	118	Grand Index
35	f	0-12 years	0.881	900.587	118	Grand Index
35	f	0-12 years	0.882	902.046	118	Grand Index
35	f	0-12 years	0.883	902.249	118	Grand Index
35	f	0-12 years	0.884	902.452	118	Grand Index
35	f	0-12 years	0.885	902.655	118	Grand Index
35	f	0-12 years	0.886	902.858	118	Grand Index
35	f	0-12 years	0.887	903.061	118	Grand Index
35	f	0-12 years	0.888	903.264	118	Grand Index
35	f	0-12 years	0.889	903.467	118	Grand Index
35	f	0-12 years	0.89	903.67	118	Grand Index
35	f	0-12 years	0.891	903.873	118	Grand Index
35	f	0-12 years	0.892	904.152	119	Grand Index
35	f	0-12 years	0.893	904.558	119	Grand Index
35	f	0-12 years	0.894	904.964	119	Grand Index
35	f	0-12 years	0.895	905.37	119	Grand Index
35	f	0-12 years	0.896	905.776	119	Grand Index
35	f	0-12 years	0.897	906.091	119	Grand Index
35	f	0-12 years	0.898	906.294	119	Grand Index
35	f	0-12 years	0.899	906.497	119	Grand Index
35	f	0-12 years	0.9	906.7	119	Grand Index
35	f	0-12 years	0.901	906.903	119	Grand Index
35	f	0-12 years	0.902	907.212	119	Grand Index
35	f	0-12 years	0.903	907.618	119	Grand Index
35	f	0-12 years	0.904	908.024	120	Grand Index
35	f	0-12 years	0.905	908.43	120	Grand Index
35	f	0-12 years	0.906	908.836	120	Grand Index
35	f	0-12 years	0.907	909.363	120	Grand Index
35	f	0-12 years	0.908	909.972	120	Grand Index
35	f	0-12 years	0.909	910.581	120	Grand Index
35	f	0-12 years	0.91	911.19	120	Grand Index
35	f	0-12 years	0.911	911.799	120	Grand Index

Age	Gender	Education	Percentile	Raw Score	Norm Score	Assessment
35	f	0-12 years	0.912	912.544	120	Grand Index
35	f	0-12 years	0.913	913.356	120	Grand Index
35	f	0-12 years	0.914	914.168	120	Grand Index
35	f	0-12 years	0.915	914.98	121	Grand Index
35	f	0-12 years	0.916	915.792	121	Grand Index
35	f	0-12 years	0.917	916.151	121	Grand Index
35	f	0-12 years	0.918	916.354	121	Grand Index
35	f	0-12 years	0.919	916.557	121	Grand Index
35	f	0-12 years	0.92	916.76	121	Grand Index
35	f	0-12 years	0.921	916.963	121	Grand Index
35	f	0-12 years	0.922	918.162	121	Grand Index
35	f	0-12 years	0.923	919.583	121	Grand Index
35	f	0-12 years	0.924	921.004	121	Grand Index
35	f	0-12 years	0.925	922.425	122	Grand Index
35	f	0-12 years	0.926	923.846	122	Grand Index
35	f	0-12 years	0.927	924.181	122	Grand Index
35	f	0-12 years	0.928	924.384	122	Grand Index
35	f	0-12 years	0.929	924.587	122	Grand Index
35	f	0-12 years	0.93	924.79	122	Grand Index
35	f	0-12 years	0.931	924.993	122	Grand Index
35	f	0-12 years	0.932	925.588	122	Grand Index
35	f	0-12 years	0.933	926.197	122	Grand Index
35	f	0-12 years	0.934	926.806	123	Grand Index
35	f	0-12 years	0.935	927.415	123	Grand Index
35	f	0-12 years	0.936	928.0081257	123	Grand Index
35	f	0-12 years	0.937	928.2143141	123	Grand Index
35	f	0-12 years	0.938	928.4205026	123	Grand Index
35	f	0-12 years	0.939	928.6266911	123	Grand Index
35	f	0-12 years	0.94	928.8328796	123	Grand Index
35	f	0-12 years	0.941	929.0613455	123	Grand Index
35	f	0-12 years	0.942	929.4641571	124	Grand Index
35	f	0-12 years	0.943	929.8669686	124	Grand Index
35	f	0-12 years	0.944	930.2697801	124	Grand Index
35	f	0-12 years	0.945	930.6725916	124	Grand Index
35	f	0-12 years	0.946	931.0776736	124	Grand Index
35	f	0-12 years	0.947	931.4926143	124	Grand Index
35	f	0-12 years	0.948	931.907555	124	Grand Index
35	f	0-12 years	0.949	932.3224956	125	Grand Index
35	f	0-12 years	0.95	932.7374363	125	Grand Index
35	f	0-12 years	0.951	933.5187084	125	Grand Index
35	f	0-12 years	0.952	935.3367678	125	Grand Index
35	f	0-12 years	0.953	937.1548271	125	Grand Index
35	f	0-12 years	0.954	938.9728864	125	Grand Index
35	f	0-12 years	0.955	940.7909458	125	Grand Index
35	f	0-12 years	0.956	942.3450246	126	Grand Index
35	f	0-12 years	0.957	943.3750246	126	Grand Index
35	f	0-12 years	0.958	944.4050246	126	Grand Index
35	f	0-12 years	0.959	945.4350246	126	Grand Index
35	f	0-12 years	0.96	946.4650246	126	Grand Index
35	f	0-12 years	0.961	947.1507586	126	Grand Index
35	f	0-12 years	0.962	947.3387586	127	Grand Index
35	f	0-12 years	0.963	947.5267586	127	Grand Index
35	f	0-12 years	0.964	947.7147586	127	Grand Index
35	f	0-12 years	0.965	947.9027586	127	Grand Index
35	f	0-12 years	0.966	948.196	127	Grand Index
35	f	0-12 years	0.967	948.602	128	Grand Index

Age	Gender	Education	Percentile	Raw Score	Norm Score	Assessment
35	f	0-12 years	0.968	949.008	128	Grand Index
35	f	0-12 years	0.969	949.414	128	Grand Index
35	f	0-12 years	0.97	949.82	128	Grand Index
35	f	0-12 years	0.971	950.1159275	128	Grand Index
35	f	0-12 years	0.972	950.3241865	129	Grand Index
35	f	0-12 years	0.973	950.5324456	129	Grand Index
35	f	0-12 years	0.974	950.7407047	129	Grand Index
35	f	0-12 years	0.975	950.9489637	129	Grand Index
35	f	0-12 years	0.976	951.5345907	130	Grand Index
35	f	0-12 years	0.977	952.3413316	130	Grand Index
35	f	0-12 years	0.978	953.1480725	130	Grand Index
35	f	0-12 years	0.979	953.9548135	131	Grand Index
35	f	0-12 years	0.98	954.7615544	131	Grand Index
35	f	0-12 years	0.981	956.573	131	Grand Index
35	f	0-12 years	0.982	958.806	131	Grand Index
35	f	0-12 years	0.983	961.039	132	Grand Index
35	f	0-12 years	0.984	963.272	132	Grand Index
35	f	0-12 years	0.985	965.505	133	Grand Index
35	f	0-12 years	0.986	966.5112407	133	Grand Index
35	f	0-12 years	0.987	967.168088	133	Grand Index
35	f	0-12 years	0.988	967.8249353	134	Grand Index
35	f	0-12 years	0.989	968.4817825	134	Grand Index
35	f	0-12 years	0.99	969.1386298	135	Grand Index
35	f	0-12 years	0.991	978.8829246	135	Grand Index
35	f	0-12 years	0.992	990.2030773	136	Grand Index
35	f	0-12 years	0.993	1001.52323	137	Grand Index
35	f	0-12 years	0.994	1012.843383	138	Grand Index
35	f	0-12 years	0.995	1024.163536	139	Grand Index
35	f	0-12 years	0.996	1038.724	140	Grand Index
35	f	0-12 years	0.997	1053.543	141	Grand Index
35	f	0-12 years	0.998	1068.362	143	Grand Index
35	f	0-12 years	0.999	1083.181	146	Grand Index
35	m	0-12 years	0.001	587.7516854	54	Grand Index
35	m	0-12 years	0.002	596.3128328	57	Grand Index
35	m	0-12 years	0.003	600.4730332	59	Grand Index
35	m	0-12 years	0.004	601.429197	60	Grand Index
35	m	0-12 years	0.005	602.934608	61	Grand Index
35	m	0-12 years	0.006	607.3806047	62	Grand Index
35	m	0-12 years	0.007	611.8266014	63	Grand Index
35	m	0-12 years	0.008	620.1907334	64	Grand Index
35	m	0-12 years	0.009	629.9597231	65	Grand Index
35	m	0-12 years	0.01	637.6161119	65	Grand Index
35	m	0-12 years	0.011	640.6735312	66	Grand Index
35	m	0-12 years	0.012	643.7309504	66	Grand Index
35	m	0-12 years	0.013	644.4047652	67	Grand Index
35	m	0-12 years	0.014	644.7943563	67	Grand Index
35	m	0-12 years	0.015	645.39	67	Grand Index
35	m	0-12 years	0.016	646.216	68	Grand Index
35	m	0-12 years	0.017	647.378	68	Grand Index
35	m	0-12 years	0.018	654.812	69	Grand Index
35	m	0-12 years	0.019	662.246	69	Grand Index
35	m	0-12 years	0.02	665.78	69	Grand Index
35	m	0-12 years	0.021	667.019	69	Grand Index
35	m	0-12 years	0.022	668.199323	70	Grand Index
35	m	0-12 years	0.023	669.1565371	70	Grand Index
35	m	0-12 years	0.024	670.1137511	70	Grand Index

Age	Gender	Education	Percentile	Raw Score	Norm Score	Assessment
35	m	0-12 years	0.025	671.3809404	71	Grand Index
35	m	0-12 years	0.026	672.7320615	71	Grand Index
35	m	0-12 years	0.027	673.6512216	71	Grand Index
35	m	0-12 years	0.028	673.8208865	71	Grand Index
35	m	0-12 years	0.029	673.9905514	72	Grand Index
35	m	0-12 years	0.03	674.39	72	Grand Index
35	m	0-12 years	0.031	674.803	72	Grand Index
35	m	0-12 years	0.032	675.159674	72	Grand Index
35	m	0-12 years	0.033	675.4649767	72	Grand Index
35	m	0-12 years	0.034	675.7523993	73	Grand Index
35	m	0-12 years	0.035	675.8818814	73	Grand Index
35	m	0-12 years	0.036	676.0113635	73	Grand Index
35	m	0-12 years	0.037	676.3189255	73	Grand Index
35	m	0-12 years	0.038	676.7101408	73	Grand Index
35	m	0-12 years	0.039	677.4033562	74	Grand Index
35	m	0-12 years	0.04	678.9602357	74	Grand Index
35	m	0-12 years	0.041	680.5171153	74	Grand Index
35	m	0-12 years	0.042	680.906496	74	Grand Index
35	m	0-12 years	0.043	681.0698005	74	Grand Index
35	m	0-12 years	0.044	681.3086981	74	Grand Index
35	m	0-12 years	0.045	681.6535142	75	Grand Index
35	m	0-12 years	0.046	681.9983302	75	Grand Index
35	m	0-12 years	0.047	684.466	75	Grand Index
35	m	0-12 years	0.048	686.944	75	Grand Index
35	m	0-12 years	0.049	688.2184529	75	Grand Index
35	m	0-12 years	0.05	688.5991325	75	Grand Index
35	m	0-12 years	0.051	688.9226729	75	Grand Index
35	m	0-12 years	0.052	688.9287734	76	Grand Index
35	m	0-12 years	0.053	688.9348739	76	Grand Index
35	m	0-12 years	0.054	689.8616864	76	Grand Index
35	m	0-12 years	0.055	691.1269064	76	Grand Index
35	m	0-12 years	0.056	692.256	76	Grand Index
35	m	0-12 years	0.057	693.082	76	Grand Index
35	m	0-12 years	0.058	693.908	76	Grand Index
35	m	0-12 years	0.059	694.367	77	Grand Index
35	m	0-12 years	0.06	694.78	77	Grand Index
35	m	0-12 years	0.061	695.193	77	Grand Index
35	m	0-12 years	0.062	695.606	77	Grand Index
35	m	0-12 years	0.063	696.0004076	77	Grand Index
35	m	0-12 years	0.064	696.0092668	77	Grand Index
35	m	0-12 years	0.065	696.018126	77	Grand Index
35	m	0-12 years	0.066	696.2739166	77	Grand Index
35	m	0-12 years	0.067	696.6780573	78	Grand Index
35	m	0-12 years	0.068	697.252	78	Grand Index
35	m	0-12 years	0.069	698.491	78	Grand Index
35	m	0-12 years	0.07	699.73	78	Grand Index
35	m	0-12 years	0.071	700.323	78	Grand Index
35	m	0-12 years	0.072	700.736	78	Grand Index
35	m	0-12 years	0.073	701.4848138	78	Grand Index
35	m	0-12 years	0.074	702.8286265	78	Grand Index
35	m	0-12 years	0.075	704.1724392	78	Grand Index
35	m	0-12 years	0.076	704.5433157	79	Grand Index
35	m	0-12 years	0.077	704.851503	79	Grand Index
35	m	0-12 years	0.078	705.428	79	Grand Index
35	m	0-12 years	0.079	706.254	79	Grand Index
35	m	0-12 years	0.08	707.0077138	79	Grand Index

Age	Gender	Education	Percentile	Raw Score	Norm Score	Assessment
35	m	0-12 years	0.081	707.0873588	79	Grand Index
35	m	0-12 years	0.082	707.1670038	79	Grand Index
35	m	0-12 years	0.083	707.4140022	79	Grand Index
35	m	0-12 years	0.084	707.7413782	79	Grand Index
35	m	0-12 years	0.085	707.9870431	79	Grand Index
35	m	0-12 years	0.086	707.9930221	80	Grand Index
35	m	0-12 years	0.087	707.9990011	80	Grand Index
35	m	0-12 years	0.088	708.344	80	Grand Index
35	m	0-12 years	0.089	708.757	80	Grand Index
35	m	0-12 years	0.09	709.34	80	Grand Index
35	m	0-12 years	0.091	710.166	80	Grand Index
35	m	0-12 years	0.092	710.992	80	Grand Index
35	m	0-12 years	0.093	714.272	80	Grand Index
35	m	0-12 years	0.094	717.576	80	Grand Index
35	m	0-12 years	0.095	719.7670218	80	Grand Index
35	m	0-12 years	0.096	721.1150218	80	Grand Index
35	m	0-12 years	0.097	722.3088232	81	Grand Index
35	m	0-12 years	0.098	722.6128232	81	Grand Index
35	m	0-12 years	0.099	722.9168232	81	Grand Index
35	m	0-12 years	0.1	723.3	81	Grand Index
35	m	0-12 years	0.101	723.713	81	Grand Index
35	m	0-12 years	0.102	724.126	81	Grand Index
35	m	0-12 years	0.103	724.539	81	Grand Index
35	m	0-12 years	0.104	724.952	81	Grand Index
35	m	0-12 years	0.111	725	82	Grand Index
35	m	0-12 years	0.112	725.256	82	Grand Index
35	m	0-12 years	0.113	725.669	82	Grand Index
35	m	0-12 years	0.118	726	82	Grand Index
35	m	0-12 years	0.119	726.441	82	Grand Index
35	m	0-12 years	0.12	727.68	82	Grand Index
35	m	0-12 years	0.121	728.919	82	Grand Index
35	m	0-12 years	0.122	729.386	83	Grand Index
35	m	0-12 years	0.123	729.799	83	Grand Index
35	m	0-12 years	0.124	730.212	83	Grand Index
35	m	0-12 years	0.125	730.625	83	Grand Index
35	m	0-12 years	0.128	731	83	Grand Index
35	m	0-12 years	0.129	731.831	83	Grand Index
35	m	0-12 years	0.13	733.07	83	Grand Index
35	m	0-12 years	0.135	734	83	Grand Index
35	m	0-12 years	0.136	734.168	84	Grand Index
35	m	0-12 years	0.137	734.581	84	Grand Index
35	m	0-12 years	0.138	734.994	84	Grand Index
35	m	0-12 years	0.145	735	84	Grand Index
35	m	0-12 years	0.146	735.596	84	Grand Index
35	m	0-12 years	0.147	736.422	84	Grand Index
35	m	0-12 years	0.15	737	84	Grand Index
35	m	0-12 years	0.151	737.363	85	Grand Index
35	m	0-12 years	0.152	737.776	85	Grand Index
35	m	0-12 years	0.159	738	85	Grand Index
35	m	0-12 years	0.16	738.08	85	Grand Index
35	m	0-12 years	0.161	738.493	85	Grand Index
35	m	0-12 years	0.162	738.906	85	Grand Index
35	m	0-12 years	0.163	739.319	85	Grand Index
35	m	0-12 years	0.164	739.732	85	Grand Index
35	m	0-12 years	0.165	740.145	85	Grand Index
35	m	0-12 years	0.166	740.558	85	Grand Index

Age	Gender	Education	Percentile	Raw Score	Norm Score	Assessment
35	m	0-12 years	0.167	740.971	86	Grand Index
35	m	0-12 years	0.168	741.384	86	Grand Index
35	m	0-12 years	0.169	741.797	86	Grand Index
35	m	0-12 years	0.174	742	86	Grand Index
35	m	0-12 years	0.175	742.2562664	86	Grand Index
35	m	0-12 years	0.176	742.641132	86	Grand Index
35	m	0-12 years	0.177	742.9387582	86	Grand Index
35	m	0-12 years	0.178	742.9668927	86	Grand Index
35	m	0-12 years	0.179	742.9950271	86	Grand Index
35	m	0-12 years	0.181	743	86	Grand Index
35	m	0-12 years	0.182	743.166	86	Grand Index
35	m	0-12 years	0.183	743.579	86	Grand Index
35	m	0-12 years	0.184	743.992	86	Grand Index
35	m	0-12 years	0.185	744.405	87	Grand Index
35	m	0-12 years	0.186	744.818	87	Grand Index
35	m	0-12 years	0.187	745.462	87	Grand Index
35	m	0-12 years	0.188	746.288	87	Grand Index
35	m	0-12 years	0.189	747.1210809	87	Grand Index
35	m	0-12 years	0.19	747.998386	87	Grand Index
35	m	0-12 years	0.191	748.8756911	87	Grand Index
35	m	0-12 years	0.192	749.3834547	87	Grand Index
35	m	0-12 years	0.193	749.7451496	87	Grand Index
35	m	0-12 years	0.198	750	87	Grand Index
35	m	0-12 years	0.199	750.187	87	Grand Index
35	m	0-12 years	0.2	750.6	87	Grand Index
35	m	0-12 years	0.203	751	88	Grand Index
35	m	0-12 years	0.204	751.504	88	Grand Index
35	m	0-12 years	0.205	752.33	88	Grand Index
35	m	0-12 years	0.206	753.0093773	88	Grand Index
35	m	0-12 years	0.207	753.0590288	88	Grand Index
35	m	0-12 years	0.208	753.1086803	88	Grand Index
35	m	0-12 years	0.209	753.3584909	88	Grand Index
35	m	0-12 years	0.21	753.6689174	88	Grand Index
35	m	0-12 years	0.211	753.8860187	88	Grand Index
35	m	0-12 years	0.212	753.9269115	88	Grand Index
35	m	0-12 years	0.213	753.9678043	88	Grand Index
35	m	0-12 years	0.214	753.982	88	Grand Index
35	m	0-12 years	0.215	753.9940291	88	Grand Index
35	m	0-12 years	0.217	754	88	Grand Index
35	m	0-12 years	0.218	754.0286489	88	Grand Index
35	m	0-12 years	0.219	754.3766489	88	Grand Index
35	m	0-12 years	0.22	754.7246489	88	Grand Index
35	m	0-12 years	0.221	754.8855811	88	Grand Index
35	m	0-12 years	0.222	754.9505811	89	Grand Index
35	m	0-12 years	0.225	755	89	Grand Index
35	m	0-12 years	0.226	755.676	89	Grand Index
35	m	0-12 years	0.227	756.502	89	Grand Index
35	m	0-12 years	0.228	757.0061654	89	Grand Index
35	m	0-12 years	0.229	757.0216917	89	Grand Index
35	m	0-12 years	0.23	757.037218	89	Grand Index
35	m	0-12 years	0.231	757.4254436	89	Grand Index
35	m	0-12 years	0.232	757.8229173	89	Grand Index
35	m	0-12 years	0.239	758	89	Grand Index
35	m	0-12 years	0.24	758.0033933	89	Grand Index
35	m	0-12 years	0.241	758.0150718	89	Grand Index
35	m	0-12 years	0.242	758.0267504	90	Grand Index

Age	Gender	Education	Percentile	Raw Score	Norm Score	Assessment
35	m	0-12 years	0.243	758.298423	90	Grand Index
35	m	0-12 years	0.244	758.6092034	90	Grand Index
35	m	0-12 years	0.245	758.8213294	90	Grand Index
35	m	0-12 years	0.246	758.9118704	90	Grand Index
35	m	0-12 years	0.247	759.011	90	Grand Index
35	m	0-12 years	0.248	759.424	90	Grand Index
35	m	0-12 years	0.249	759.837	90	Grand Index
35	m	0-12 years	0.254	760	90	Grand Index
35	m	0-12 years	0.255	760.315	90	Grand Index
35	m	0-12 years	0.256	760.728	90	Grand Index
35	m	0-12 years	0.266	761	91	Grand Index
35	m	0-12 years	0.267	761.271	91	Grand Index
35	m	0-12 years	0.268	761.684	91	Grand Index
35	m	0-12 years	0.271	762	91	Grand Index
35	m	0-12 years	0.272	762.336	91	Grand Index
35	m	0-12 years	0.273	762.749	91	Grand Index
35	m	0-12 years	0.28	763	91	Grand Index
35	m	0-12 years	0.281	763.053	91	Grand Index
35	m	0-12 years	0.282	763.466	91	Grand Index
35	m	0-12 years	0.283	763.879	91	Grand Index
35	m	0-12 years	0.285	764	91	Grand Index
35	m	0-12 years	0.286	764.1048177	92	Grand Index
35	m	0-12 years	0.287	764.4716799	92	Grand Index
35	m	0-12 years	0.288	764.838542	92	Grand Index
35	m	0-12 years	0.289	764.9281679	92	Grand Index
35	m	0-12 years	0.29	764.9743058	92	Grand Index
35	m	0-12 years	0.292	765	92	Grand Index
35	m	0-12 years	0.293	765.009	92	Grand Index
35	m	0-12 years	0.294	765.422	92	Grand Index
35	m	0-12 years	0.295	765.835	92	Grand Index
35	m	0-12 years	0.297	766	92	Grand Index
35	m	0-12 years	0.298	766.074	92	Grand Index
35	m	0-12 years	0.299	766.487	92	Grand Index
35	m	0-12 years	0.3	766.9	92	Grand Index
35	m	0-12 years	0.301	767.313	92	Grand Index
35	m	0-12 years	0.302	767.726	92	Grand Index
35	m	0-12 years	0.305	768	92	Grand Index
35	m	0-12 years	0.306	768.378	92	Grand Index
35	m	0-12 years	0.307	768.791	92	Grand Index
35	m	0-12 years	0.308	769.0051544	92	Grand Index
35	m	0-12 years	0.309	769.0155894	93	Grand Index
35	m	0-12 years	0.31	769.0545084	93	Grand Index
35	m	0-12 years	0.311	769.4570734	93	Grand Index
35	m	0-12 years	0.312	769.8596384	93	Grand Index
35	m	0-12 years	0.314	770	93	Grand Index
35	m	0-12 years	0.315	770.19	93	Grand Index
35	m	0-12 years	0.316	771.016	93	Grand Index
35	m	0-12 years	0.317	771.842	93	Grand Index
35	m	0-12 years	0.322	772	93	Grand Index
35	m	0-12 years	0.323	772.399	93	Grand Index
35	m	0-12 years	0.324	772.812	93	Grand Index
35	m	0-12 years	0.325	773.225	93	Grand Index
35	m	0-12 years	0.326	773.638	93	Grand Index
35	m	0-12 years	0.329	774	93	Grand Index
35	m	0-12 years	0.33	774.0911222	93	Grand Index
35	m	0-12 years	0.331	774.2208928	93	Grand Index

Age	Gender	Education	Percentile	Raw Score	Norm Score	Assessment
35	m	0-12 years	0.332	774.3937656	93	Grand Index
35	m	0-12 years	0.333	774.676995	94	Grand Index
35	m	0-12 years	0.334	774.9602244	94	Grand Index
35	m	0-12 years	0.338	775	94	Grand Index
35	m	0-12 years	0.339	775.007	94	Grand Index
35	m	0-12 years	0.34	775.42	94	Grand Index
35	m	0-12 years	0.341	775.833	94	Grand Index
35	m	0-12 years	0.342	776.1701378	94	Grand Index
35	m	0-12 years	0.343	776.4557758	94	Grand Index
35	m	0-12 years	0.344	776.7138208	94	Grand Index
35	m	0-12 years	0.345	776.8411829	94	Grand Index
35	m	0-12 years	0.346	776.968545	94	Grand Index
35	m	0-12 years	0.348	777	94	Grand Index
35	m	0-12 years	0.349	777.0079972	94	Grand Index
35	m	0-12 years	0.35	777.0321054	94	Grand Index
35	m	0-12 years	0.351	777.0562137	94	Grand Index
35	m	0-12 years	0.352	777.4124251	94	Grand Index
35	m	0-12 years	0.353	777.8013168	94	Grand Index
35	m	0-12 years	0.355	778	94	Grand Index
35	m	0-12 years	0.356	778.084	94	Grand Index
35	m	0-12 years	0.357	779.323	95	Grand Index
35	m	0-12 years	0.358	780.562	95	Grand Index
35	m	0-12 years	0.36	781	95	Grand Index
35	m	0-12 years	0.361	781.0797005	95	Grand Index
35	m	0-12 years	0.362	781.4336391	95	Grand Index
35	m	0-12 years	0.363	781.7875778	95	Grand Index
35	m	0-12 years	0.364	781.9044722	95	Grand Index
35	m	0-12 years	0.365	781.9635335	95	Grand Index
35	m	0-12 years	0.37	782	95	Grand Index
35	m	0-12 years	0.371	782.4405064	95	Grand Index
35	m	0-12 years	0.372	783.2563321	95	Grand Index
35	m	0-12 years	0.373	784.0255721	95	Grand Index
35	m	0-12 years	0.374	784.4487464	95	Grand Index
35	m	0-12 years	0.375	784.8719206	95	Grand Index
35	m	0-12 years	0.376	785.288	95	Grand Index
35	m	0-12 years	0.377	785.701	95	Grand Index
35	m	0-12 years	0.382	786	95	Grand Index
35	m	0-12 years	0.383	786.1829204	96	Grand Index
35	m	0-12 years	0.384	786.6049657	96	Grand Index
35	m	0-12 years	0.385	787.0267919	96	Grand Index
35	m	0-12 years	0.386	787.4307466	96	Grand Index
35	m	0-12 years	0.387	787.8347013	96	Grand Index
35	m	0-12 years	0.388	788.244	96	Grand Index
35	m	0-12 years	0.389	788.657	96	Grand Index
35	m	0-12 years	0.39	789.07	96	Grand Index
35	m	0-12 years	0.391	789.483	96	Grand Index
35	m	0-12 years	0.392	789.896	96	Grand Index
35	m	0-12 years	0.394	790	96	Grand Index
35	m	0-12 years	0.395	790.0014499	96	Grand Index
35	m	0-12 years	0.396	790.0058853	96	Grand Index
35	m	0-12 years	0.397	790.0103208	96	Grand Index
35	m	0-12 years	0.398	790.0186168	96	Grand Index
35	m	0-12 years	0.399	790.0273152	96	Grand Index
35	m	0-12 years	0.4	790.2254411	96	Grand Index
35	m	0-12 years	0.401	790.6253071	96	Grand Index
35	m	0-12 years	0.402	791.026	96	Grand Index

Age	Gender	Education	Percentile	Raw Score	Norm Score	Assessment
35	m	0-12 years	0.403	791.439	96	Grand Index
35	m	0-12 years	0.404	791.852	96	Grand Index
35	m	0-12 years	0.405	792.1639273	96	Grand Index
35	m	0-12 years	0.406	792.4194065	96	Grand Index
35	m	0-12 years	0.407	792.6533017	96	Grand Index
35	m	0-12 years	0.408	792.8108225	97	Grand Index
35	m	0-12 years	0.409	792.9683433	97	Grand Index
35	m	0-12 years	0.41	793.33	97	Grand Index
35	m	0-12 years	0.411	793.743	97	Grand Index
35	m	0-12 years	0.416	794	97	Grand Index
35	m	0-12 years	0.417	794.221	97	Grand Index
35	m	0-12 years	0.418	794.634	97	Grand Index
35	m	0-12 years	0.421	795	97	Grand Index
35	m	0-12 years	0.422	795.286	97	Grand Index
35	m	0-12 years	0.423	795.699	97	Grand Index
35	m	0-12 years	0.424	796.1536143	97	Grand Index
35	m	0-12 years	0.425	796.7200669	97	Grand Index
35	m	0-12 years	0.426	797.2865194	97	Grand Index
35	m	0-12 years	0.427	797.5921398	97	Grand Index
35	m	0-12 years	0.428	797.8516872	97	Grand Index
35	m	0-12 years	0.43	798	97	Grand Index
35	m	0-12 years	0.431	798.0000561	97	Grand Index
35	m	0-12 years	0.432	798.0077777	97	Grand Index
35	m	0-12 years	0.433	798.0154993	97	Grand Index
35	m	0-12 years	0.434	798.2561718	98	Grand Index
35	m	0-12 years	0.435	798.6614502	98	Grand Index
35	m	0-12 years	0.438	799	98	Grand Index
35	m	0-12 years	0.439	799.3055862	98	Grand Index
35	m	0-12 years	0.44	799.7166843	98	Grand Index
35	m	0-12 years	0.441	799.9960074	98	Grand Index
35	m	0-12 years	0.442	799.9979093	98	Grand Index
35	m	0-12 years	0.443	799.9998112	98	Grand Index
35	m	0-12 years	0.45	800	98	Grand Index
35	m	0-12 years	0.451	800.263	98	Grand Index
35	m	0-12 years	0.452	800.676	98	Grand Index
35	m	0-12 years	0.464	801	99	Grand Index
35	m	0-12 years	0.465	801.0055851	99	Grand Index
35	m	0-12 years	0.466	801.056844	99	Grand Index
35	m	0-12 years	0.467	801.1081028	99	Grand Index
35	m	0-12 years	0.468	801.3728652	99	Grand Index
35	m	0-12 years	0.469	801.7346064	99	Grand Index
35	m	0-12 years	0.472	802	99	Grand Index
35	m	0-12 years	0.473	802.349	99	Grand Index
35	m	0-12 years	0.474	802.762	99	Grand Index
35	m	0-12 years	0.476	803	99	Grand Index
35	m	0-12 years	0.477	803.0000158	99	Grand Index
35	m	0-12 years	0.478	803.006551	99	Grand Index
35	m	0-12 years	0.479	803.0130862	99	Grand Index
35	m	0-12 years	0.48	803.1970285	99	Grand Index
35	m	0-12 years	0.481	803.5088518	99	Grand Index
35	m	0-12 years	0.482	803.785968	99	Grand Index
35	m	0-12 years	0.483	803.8806096	99	Grand Index
35	m	0-12 years	0.484	803.9752511	99	Grand Index
35	m	0-12 years	0.489	804	100	Grand Index
35	m	0-12 years	0.49	804.37	100	Grand Index
35	m	0-12 years	0.491	804.783	100	Grand Index

Age	Gender	Education	Percentile	Raw Score	Norm Score	Assessment
35	m	0-12 years	0.492	805.196	100	Grand Index
35	m	0-12 years	0.493	805.609	100	Grand Index
35	m	0-12 years	0.496	806	100	Grand Index
35	m	0-12 years	0.497	806.2597458	100	Grand Index
35	m	0-12 years	0.498	806.6707612	100	Grand Index
35	m	0-12 years	0.499	806.9956127	100	Grand Index
35	m	0-12 years	0.5	806.9975973	100	Grand Index
35	m	0-12 years	0.501	806.9995819	100	Grand Index
35	m	0-12 years	0.506	807	100	Grand Index
35	m	0-12 years	0.507	807.391	100	Grand Index
35	m	0-12 years	0.508	807.804	100	Grand Index
35	m	0-12 years	0.515	808	101	Grand Index
35	m	0-12 years	0.516	808.0772826	101	Grand Index
35	m	0-12 years	0.517	808.3728169	101	Grand Index
35	m	0-12 years	0.518	808.6683511	101	Grand Index
35	m	0-12 years	0.519	808.8142733	101	Grand Index
35	m	0-12 years	0.52	808.9317391	101	Grand Index
35	m	0-12 years	0.525	809	101	Grand Index
35	m	0-12 years	0.526	809.238	101	Grand Index
35	m	0-12 years	0.527	809.651	101	Grand Index
35	m	0-12 years	0.528	810.064	101	Grand Index
35	m	0-12 years	0.529	810.477	101	Grand Index
35	m	0-12 years	0.53	810.89	101	Grand Index
35	m	0-12 years	0.532	811	101	Grand Index
35	m	0-12 years	0.533	811.129	101	Grand Index
35	m	0-12 years	0.534	811.542	101	Grand Index
35	m	0-12 years	0.535	811.955	101	Grand Index
35	m	0-12 years	0.537	812	101	Grand Index
35	m	0-12 years	0.538	812.194	101	Grand Index
35	m	0-12 years	0.539	812.607	101	Grand Index
35	m	0-12 years	0.54	813.02	102	Grand Index
35	m	0-12 years	0.541	813.433	102	Grand Index
35	m	0-12 years	0.542	813.846	102	Grand Index
35	m	0-12 years	0.543	814.518	102	Grand Index
35	m	0-12 years	0.544	815.344	102	Grand Index
35	m	0-12 years	0.547	816	102	Grand Index
35	m	0-12 years	0.548	816.648	102	Grand Index
35	m	0-12 years	0.549	817.474	102	Grand Index
35	m	0-12 years	0.556	818	102	Grand Index
35	m	0-12 years	0.557	818.041	102	Grand Index
35	m	0-12 years	0.558	818.454	102	Grand Index
35	m	0-12 years	0.559	818.867	102	Grand Index
35	m	0-12 years	0.561	819	102	Grand Index
35	m	0-12 years	0.562	819.106	102	Grand Index
35	m	0-12 years	0.563	819.519	102	Grand Index
35	m	0-12 years	0.564	819.932	102	Grand Index
35	m	0-12 years	0.565	820.345	102	Grand Index
35	m	0-12 years	0.566	820.758	102	Grand Index
35	m	0-12 years	0.569	821	103	Grand Index
35	m	0-12 years	0.57	821.41	103	Grand Index
35	m	0-12 years	0.571	821.823	103	Grand Index
35	m	0-12 years	0.572	822.0313157	103	Grand Index
35	m	0-12 years	0.573	822.0861182	103	Grand Index
35	m	0-12 years	0.574	822.1864666	103	Grand Index
35	m	0-12 years	0.575	822.5446642	103	Grand Index
35	m	0-12 years	0.576	822.9028617	103	Grand Index

Age	Gender	Education	Percentile	Raw Score	Norm Score	Assessment
35	m	0-12 years	0.578	823	103	Grand Index
35	m	0-12 years	0.579	823.127	103	Grand Index
35	m	0-12 years	0.58	823.54	103	Grand Index
35	m	0-12 years	0.581	823.953	103	Grand Index
35	m	0-12 years	0.583	824	103	Grand Index
35	m	0-12 years	0.584	824.1939261	103	Grand Index
35	m	0-12 years	0.585	824.6110694	103	Grand Index
35	m	0-12 years	0.586	825.0249578	103	Grand Index
35	m	0-12 years	0.587	825.3674222	103	Grand Index
35	m	0-12 years	0.588	825.7098865	103	Grand Index
35	m	0-12 years	0.589	825.8805579	103	Grand Index
35	m	0-12 years	0.59	825.9469504	103	Grand Index
35	m	0-12 years	0.593	826	104	Grand Index
35	m	0-12 years	0.594	826.325748	104	Grand Index
35	m	0-12 years	0.595	826.7435552	104	Grand Index
35	m	0-12 years	0.596	827.1579171	104	Grand Index
35	m	0-12 years	0.597	827.5661099	104	Grand Index
35	m	0-12 years	0.598	827.9743026	104	Grand Index
35	m	0-12 years	0.6	828	104	Grand Index
35	m	0-12 years	0.601	828.0759165	104	Grand Index
35	m	0-12 years	0.602	828.2231161	104	Grand Index
35	m	0-12 years	0.603	828.4205153	104	Grand Index
35	m	0-12 years	0.604	829.0993157	104	Grand Index
35	m	0-12 years	0.605	829.7781161	104	Grand Index
35	m	0-12 years	0.606	830.278	104	Grand Index
35	m	0-12 years	0.607	830.691	104	Grand Index
35	m	0-12 years	0.608	831.104	104	Grand Index
35	m	0-12 years	0.609	831.517	104	Grand Index
35	m	0-12 years	0.61	831.93	104	Grand Index
35	m	0-12 years	0.617	832	104	Grand Index
35	m	0-12 years	0.618	832.234	105	Grand Index
35	m	0-12 years	0.619	832.647	105	Grand Index
35	m	0-12 years	0.62	833.06	105	Grand Index
35	m	0-12 years	0.621	833.473	105	Grand Index
35	m	0-12 years	0.622	833.886	105	Grand Index
35	m	0-12 years	0.627	834	105	Grand Index
35	m	0-12 years	0.628	835.092	105	Grand Index
35	m	0-12 years	0.629	836.331	105	Grand Index
35	m	0-12 years	0.634	837	105	Grand Index
35	m	0-12 years	0.635	837.255	105	Grand Index
35	m	0-12 years	0.636	837.668	105	Grand Index
35	m	0-12 years	0.641	838	105	Grand Index
35	m	0-12 years	0.642	838.146	105	Grand Index
35	m	0-12 years	0.643	838.559	105	Grand Index
35	m	0-12 years	0.644	838.972	106	Grand Index
35	m	0-12 years	0.645	839.385	106	Grand Index
35	m	0-12 years	0.646	839.798	106	Grand Index
35	m	0-12 years	0.647	840.211	106	Grand Index
35	m	0-12 years	0.648	840.624	106	Grand Index
35	m	0-12 years	0.651	841	106	Grand Index
35	m	0-12 years	0.652	841.1440478	106	Grand Index
35	m	0-12 years	0.653	841.3595976	106	Grand Index
35	m	0-12 years	0.654	841.5706773	106	Grand Index
35	m	0-12 years	0.655	841.7681275	106	Grand Index
35	m	0-12 years	0.656	841.9655777	106	Grand Index
35	m	0-12 years	0.657	842.341	106	Grand Index

Age	Gender	Education	Percentile	Raw Score	Norm Score	Assessment
35	m	0-12 years	0.658	842.754	106	Grand Index
35	m	0-12 years	0.661	843	106	Grand Index
35	m	0-12 years	0.662	843.406	106	Grand Index
35	m	0-12 years	0.663	843.819	106	Grand Index
35	m	0-12 years	0.668	844	107	Grand Index
35	m	0-12 years	0.669	844.0198191	107	Grand Index
35	m	0-12 years	0.67	844.0473791	107	Grand Index
35	m	0-12 years	0.671	844.3045232	107	Grand Index
35	m	0-12 years	0.672	845.1029632	107	Grand Index
35	m	0-12 years	0.673	845.9014033	107	Grand Index
35	m	0-12 years	0.674	846.362	107	Grand Index
35	m	0-12 years	0.675	846.775	107	Grand Index
35	m	0-12 years	0.677	847	107	Grand Index
35	m	0-12 years	0.678	847.014	107	Grand Index
35	m	0-12 years	0.679	847.427	107	Grand Index
35	m	0-12 years	0.68	847.84	107	Grand Index
35	m	0-12 years	0.685	848	107	Grand Index
35	m	0-12 years	0.686	848.318	107	Grand Index
35	m	0-12 years	0.687	848.731	107	Grand Index
35	m	0-12 years	0.69	849	107	Grand Index
35	m	0-12 years	0.691	850.149	107	Grand Index
35	m	0-12 years	0.692	851.388	108	Grand Index
35	m	0-12 years	0.694	852	108	Grand Index
35	m	0-12 years	0.695	852.0303071	108	Grand Index
35	m	0-12 years	0.696	852.3879304	108	Grand Index
35	m	0-12 years	0.697	852.7455537	108	Grand Index
35	m	0-12 years	0.698	852.9026551	108	Grand Index
35	m	0-12 years	0.699	852.9580317	108	Grand Index
35	m	0-12 years	0.7	853.1	108	Grand Index
35	m	0-12 years	0.701	853.513	108	Grand Index
35	m	0-12 years	0.702	853.926	108	Grand Index
35	m	0-12 years	0.703	854.339	108	Grand Index
35	m	0-12 years	0.704	854.752	108	Grand Index
35	m	0-12 years	0.705	855.165	108	Grand Index
35	m	0-12 years	0.706	855.578	108	Grand Index
35	m	0-12 years	0.707	855.991	108	Grand Index
35	m	0-12 years	0.708	856.404	108	Grand Index
35	m	0-12 years	0.709	856.817	108	Grand Index
35	m	0-12 years	0.71	857.23	108	Grand Index
35	m	0-12 years	0.711	857.643	108	Grand Index
35	m	0-12 years	0.712	858.056	108	Grand Index
35	m	0-12 years	0.713	858.469	108	Grand Index
35	m	0-12 years	0.714	858.882	108	Grand Index
35	m	0-12 years	0.715	859.295	109	Grand Index
35	m	0-12 years	0.716	859.708	109	Grand Index
35	m	0-12 years	0.717	860.121	109	Grand Index
35	m	0-12 years	0.718	860.534	109	Grand Index
35	m	0-12 years	0.719	860.947	109	Grand Index
35	m	0-12 years	0.721	861	109	Grand Index
35	m	0-12 years	0.722	861.186	109	Grand Index
35	m	0-12 years	0.723	861.599	109	Grand Index
35	m	0-12 years	0.726	862	109	Grand Index
35	m	0-12 years	0.727	862.251	109	Grand Index
35	m	0-12 years	0.728	862.664	109	Grand Index
35	m	0-12 years	0.731	863	109	Grand Index
35	m	0-12 years	0.732	863.1069293	109	Grand Index

Age	Gender	Education	Percentile	Raw Score	Norm Score	Assessment
35	m	0-12 years	0.733	863.2466818	109	Grand Index
35	m	0-12 years	0.734	863.4323333	109	Grand Index
35	m	0-12 years	0.735	863.7055808	109	Grand Index
35	m	0-12 years	0.736	863.9788283	109	Grand Index
35	m	0-12 years	0.738	864	110	Grand Index
35	m	0-12 years	0.739	864.0649412	110	Grand Index
35	m	0-12 years	0.74	864.1945098	110	Grand Index
35	m	0-12 years	0.741	864.4023725	110	Grand Index
35	m	0-12 years	0.742	865.5118039	110	Grand Index
35	m	0-12 years	0.743	866.6212353	110	Grand Index
35	m	0-12 years	0.744	867.272	110	Grand Index
35	m	0-12 years	0.745	867.685	110	Grand Index
35	m	0-12 years	0.748	868	110	Grand Index
35	m	0-12 years	0.749	868.337	110	Grand Index
35	m	0-12 years	0.75	868.75	110	Grand Index
35	m	0-12 years	0.751	869.163	110	Grand Index
35	m	0-12 years	0.752	869.576	110	Grand Index
35	m	0-12 years	0.753	869.989	110	Grand Index
35	m	0-12 years	0.754	870.9828846	110	Grand Index
35	m	0-12 years	0.755	871.9926641	110	Grand Index
35	m	0-12 years	0.756	873.0275296	110	Grand Index
35	m	0-12 years	0.757	874.0827502	110	Grand Index
35	m	0-12 years	0.758	875.054	110	Grand Index
35	m	0-12 years	0.759	875.467	111	Grand Index
35	m	0-12 years	0.76	875.88	111	Grand Index
35	m	0-12 years	0.762	876	111	Grand Index
35	m	0-12 years	0.763	876.119	111	Grand Index
35	m	0-12 years	0.764	876.532	111	Grand Index
35	m	0-12 years	0.765	876.945	111	Grand Index
35	m	0-12 years	0.766	877.358	111	Grand Index
35	m	0-12 years	0.767	877.771	111	Grand Index
35	m	0-12 years	0.769	878	111	Grand Index
35	m	0-12 years	0.77	878.0001319	111	Grand Index
35	m	0-12 years	0.771	878.0055776	111	Grand Index
35	m	0-12 years	0.772	878.0110233	111	Grand Index
35	m	0-12 years	0.773	878.2589025	111	Grand Index
35	m	0-12 years	0.774	878.6664568	111	Grand Index
35	m	0-12 years	0.777	879	111	Grand Index
35	m	0-12 years	0.778	879.314	111	Grand Index
35	m	0-12 years	0.779	879.727	112	Grand Index
35	m	0-12 years	0.782	880	112	Grand Index
35	m	0-12 years	0.783	880.2433971	112	Grand Index
35	m	0-12 years	0.784	880.5086292	112	Grand Index
35	m	0-12 years	0.785	880.7155559	112	Grand Index
35	m	0-12 years	0.786	880.8633237	112	Grand Index
35	m	0-12 years	0.791	881	112	Grand Index
35	m	0-12 years	0.792	881.096	112	Grand Index
35	m	0-12 years	0.793	881.509	112	Grand Index
35	m	0-12 years	0.794	881.922	112	Grand Index
35	m	0-12 years	0.799	882	113	Grand Index
35	m	0-12 years	0.8	882.1115426	113	Grand Index
35	m	0-12 years	0.801	882.2267104	113	Grand Index
35	m	0-12 years	0.802	882.382936	113	Grand Index
35	m	0-12 years	0.803	882.5731342	113	Grand Index
35	m	0-12 years	0.804	882.7991073	113	Grand Index
35	m	0-12 years	0.805	883.2734409	113	Grand Index

Age	Gender	Education	Percentile	Raw Score	Norm Score	Assessment
35	m	0-12 years	0.806	883.7477745	113	Grand Index
35	m	0-12 years	0.807	883.9205157	113	Grand Index
35	m	0-12 years	0.808	883.9668161	113	Grand Index
35	m	0-12 years	0.811	884	113	Grand Index
35	m	0-12 years	0.812	884.356	113	Grand Index
35	m	0-12 years	0.813	884.769	113	Grand Index
35	m	0-12 years	0.814	885.182	113	Grand Index
35	m	0-12 years	0.815	885.595	113	Grand Index
35	m	0-12 years	0.818	886	114	Grand Index
35	m	0-12 years	0.819	886.0437722	114	Grand Index
35	m	0-12 years	0.82	886.116962	114	Grand Index
35	m	0-12 years	0.821	886.3080579	114	Grand Index
35	m	0-12 years	0.822	887.0483053	114	Grand Index
35	m	0-12 years	0.823	887.7885527	114	Grand Index
35	m	0-12 years	0.824	887.9790722	114	Grand Index
35	m	0-12 years	0.825	887.991635	114	Grand Index
35	m	0-12 years	0.826	888.0763805	114	Grand Index
35	m	0-12 years	0.827	888.3049685	114	Grand Index
35	m	0-12 years	0.828	888.5335565	114	Grand Index
35	m	0-12 years	0.829	888.7218192	114	Grand Index
35	m	0-12 years	0.83	888.9062312	114	Grand Index
35	m	0-12 years	0.831	889.203	114	Grand Index
35	m	0-12 years	0.832	889.616	114	Grand Index
35	m	0-12 years	0.833	890.029	114	Grand Index
35	m	0-12 years	0.834	890.442	115	Grand Index
35	m	0-12 years	0.835	890.855	115	Grand Index
35	m	0-12 years	0.845	891	115	Grand Index
35	m	0-12 years	0.846	891.398	115	Grand Index
35	m	0-12 years	0.847	891.811	115	Grand Index
35	m	0-12 years	0.848	892.0004234	115	Grand Index
35	m	0-12 years	0.849	892.0012042	115	Grand Index
35	m	0-12 years	0.85	892.0517958	116	Grand Index
35	m	0-12 years	0.851	892.4640151	116	Grand Index
35	m	0-12 years	0.852	892.8762344	116	Grand Index
35	m	0-12 years	0.853	894.0781493	116	Grand Index
35	m	0-12 years	0.854	895.6188955	116	Grand Index
35	m	0-12 years	0.855	896.761599	116	Grand Index
35	m	0-12 years	0.856	896.8728528	116	Grand Index
35	m	0-12 years	0.857	896.9841066	116	Grand Index
35	m	0-12 years	0.861	897	116	Grand Index
35	m	0-12 years	0.862	897.006	116	Grand Index
35	m	0-12 years	0.863	897.419	116	Grand Index
35	m	0-12 years	0.864	897.832	116	Grand Index
35	m	0-12 years	0.865	898.0385593	117	Grand Index
35	m	0-12 years	0.866	898.1035593	117	Grand Index
35	m	0-12 years	0.867	898.2442127	117	Grand Index
35	m	0-12 years	0.868	898.7492811	117	Grand Index
35	m	0-12 years	0.869	899.2543495	117	Grand Index
35	m	0-12 years	0.87	899.5724145	117	Grand Index
35	m	0-12 years	0.871	899.8283461	117	Grand Index
35	m	0-12 years	0.872	900.136	117	Grand Index
35	m	0-12 years	0.873	900.549	117	Grand Index
35	m	0-12 years	0.874	900.962	117	Grand Index
35	m	0-12 years	0.875	901.0415247	117	Grand Index
35	m	0-12 years	0.876	901.0872572	117	Grand Index
35	m	0-12 years	0.877	901.4904753	117	Grand Index

Age	Gender	Education	Percentile	Raw Score	Norm Score	Assessment
35	m	0-12 years	0.878	902.2707428	117	Grand Index
35	m	0-12 years	0.879	903.027	118	Grand Index
35	m	0-12 years	0.88	903.44	118	Grand Index
35	m	0-12 years	0.881	903.853	118	Grand Index
35	m	0-12 years	0.883	904	118	Grand Index
35	m	0-12 years	0.884	904.0786291	118	Grand Index
35	m	0-12 years	0.885	904.4316052	118	Grand Index
35	m	0-12 years	0.886	904.7845813	118	Grand Index
35	m	0-12 years	0.887	904.9548644	118	Grand Index
35	m	0-12 years	0.888	905.0798883	118	Grand Index
35	m	0-12 years	0.889	905.2211231	118	Grand Index
35	m	0-12 years	0.89	905.3887908	118	Grand Index
35	m	0-12 years	0.891	905.5564585	118	Grand Index
35	m	0-12 years	0.892	905.5863498	119	Grand Index
35	m	0-12 years	0.893	905.6103264	119	Grand Index
35	m	0-12 years	0.894	905.7054608	119	Grand Index
35	m	0-12 years	0.895	905.8618164	119	Grand Index
35	m	0-12 years	0.9	906	119	Grand Index
35	m	0-12 years	0.901	906.0943856	119	Grand Index
35	m	0-12 years	0.902	906.4393524	119	Grand Index
35	m	0-12 years	0.903	906.7843191	119	Grand Index
35	m	0-12 years	0.904	907.2452554	120	Grand Index
35	m	0-12 years	0.905	907.7262886	120	Grand Index
35	m	0-12 years	0.906	908.356	120	Grand Index
35	m	0-12 years	0.907	909.182	120	Grand Index
35	m	0-12 years	0.908	910.004	120	Grand Index
35	m	0-12 years	0.909	910.417	120	Grand Index
35	m	0-12 years	0.91	910.83	120	Grand Index
35	m	0-12 years	0.911	912.215	120	Grand Index
35	m	0-12 years	0.912	914.28	120	Grand Index
35	m	0-12 years	0.915	916	121	Grand Index
35	m	0-12 years	0.916	916.616	121	Grand Index
35	m	0-12 years	0.917	917.442	121	Grand Index
35	m	0-12 years	0.918	918.1995649	121	Grand Index
35	m	0-12 years	0.919	918.8146419	121	Grand Index
35	m	0-12 years	0.92	919.4297189	121	Grand Index
35	m	0-12 years	0.921	920.7987851	121	Grand Index
35	m	0-12 years	0.922	922.2487082	121	Grand Index
35	m	0-12 years	0.923	923.995	121	Grand Index
35	m	0-12 years	0.924	926.06	121	Grand Index
35	m	0-12 years	0.925	928.0098889	122	Grand Index
35	m	0-12 years	0.926	928.1732531	122	Grand Index
35	m	0-12 years	0.927	928.3366173	122	Grand Index
35	m	0-12 years	0.928	928.5551285	122	Grand Index
35	m	0-12 years	0.929	928.8047643	122	Grand Index
35	m	0-12 years	0.932	929	122	Grand Index
35	m	0-12 years	0.933	929.329	122	Grand Index
35	m	0-12 years	0.934	929.742	123	Grand Index
35	m	0-12 years	0.935	930.465	123	Grand Index
35	m	0-12 years	0.936	931.704	123	Grand Index
35	m	0-12 years	0.937	932.943	123	Grand Index
35	m	0-12 years	0.938	933.3357685	123	Grand Index
35	m	0-12 years	0.939	933.6877288	123	Grand Index
35	m	0-12 years	0.94	933.8847193	123	Grand Index
35	m	0-12 years	0.941	933.945759	123	Grand Index
35	m	0-12 years	0.942	934.184	124	Grand Index

Age	Gender	Education	Percentile	Raw Score	Norm Score	Assessment
35	m	0-12 years	0.943	935.836	124	Grand Index
35	m	0-12 years	0.944	937.488	124	Grand Index
35	m	0-12 years	0.946	938	124	Grand Index
35	m	0-12 years	0.947	938.222	124	Grand Index
35	m	0-12 years	0.948	939.048	124	Grand Index
35	m	0-12 years	0.949	939.874	125	Grand Index
35	m	0-12 years	0.95	940.7	125	Grand Index
35	m	0-12 years	0.951	941.526	125	Grand Index
35	m	0-12 years	0.952	942.0456497	125	Grand Index
35	m	0-12 years	0.953	942.1527709	125	Grand Index
35	m	0-12 years	0.954	942.2664825	125	Grand Index
35	m	0-12 years	0.955	943.7345431	125	Grand Index
35	m	0-12 years	0.956	945.2026036	126	Grand Index
35	m	0-12 years	0.957	946.3408254	126	Grand Index
35	m	0-12 years	0.958	947.2436437	126	Grand Index
35	m	0-12 years	0.959	948.1128807	126	Grand Index
35	m	0-12 years	0.96	948.8086979	126	Grand Index
35	m	0-12 years	0.961	949.504515	126	Grand Index
35	m	0-12 years	0.962	950.0872423	127	Grand Index
35	m	0-12 years	0.963	950.6304252	127	Grand Index
35	m	0-12 years	0.964	951.528	127	Grand Index
35	m	0-12 years	0.965	953.18	127	Grand Index
35	m	0-12 years	0.966	954.832	127	Grand Index
35	m	0-12 years	0.968	955	128	Grand Index
35	m	0-12 years	0.969	955.4425979	128	Grand Index
35	m	0-12 years	0.97	956.3704808	128	Grand Index
35	m	0-12 years	0.971	957.2851386	128	Grand Index
35	m	0-12 years	0.972	957.9755437	129	Grand Index
35	m	0-12 years	0.973	958.6659488	129	Grand Index
35	m	0-12 years	0.974	959.2017592	129	Grand Index
35	m	0-12 years	0.975	959.6484712	129	Grand Index
35	m	0-12 years	0.976	960.176	130	Grand Index
35	m	0-12 years	0.977	961.002	130	Grand Index
35	m	0-12 years	0.978	961.828	130	Grand Index
35	m	0-12 years	0.979	963.2755648	131	Grand Index
35	m	0-12 years	0.98	964.8865992	131	Grand Index
35	m	0-12 years	0.981	967.5989859	131	Grand Index
35	m	0-12 years	0.982	972.1829514	131	Grand Index
35	m	0-12 years	0.983	976.766917	132	Grand Index
35	m	0-12 years	0.984	980.8936269	132	Grand Index
35	m	0-12 years	0.985	984.995841	133	Grand Index
35	m	0-12 years	0.986	989.5633884	133	Grand Index
35	m	0-12 years	0.987	994.5471743	133	Grand Index
35	m	0-12 years	0.988	999.1790224	134	Grand Index
35	m	0-12 years	0.989	1000.859391	134	Grand Index
35	m	0-12 years	0.99	1002.53976	135	Grand Index
35	m	0-12 years	0.991	1003.898251	135	Grand Index
35	m	0-12 years	0.992	1005.108882	136	Grand Index
35	m	0-12 years	0.993	1007.635	137	Grand Index
35	m	0-12 years	0.994	1013.83	138	Grand Index
35	m	0-12 years	0.995	1020.025	139	Grand Index
35	m	0-12 years	0.996	1022.876971	140	Grand Index
35	m	0-12 years	0.997	1025.104525	141	Grand Index
35	m	0-12 years	0.998	1031.512799	143	Grand Index
35	m	0-12 years	0.999	1043.663555	146	Grand Index
35	f	13-16 years	0.001	567.8562861	54	Grand Index

Age	Gender	Education	Percentile	Raw Score	Norm Score	Assessment
35	f	13-16 years	0.002	569.9789322	57	Grand Index
35	f	13-16 years	0.003	575.0557879	59	Grand Index
35	f	13-16 years	0.004	600.8852307	60	Grand Index
35	f	13-16 years	0.005	616.2387475	61	Grand Index
35	f	13-16 years	0.006	622.1920435	62	Grand Index
35	f	13-16 years	0.007	626.0721881	63	Grand Index
35	f	13-16 years	0.008	627.9638616	64	Grand Index
35	f	13-16 years	0.009	632.576	65	Grand Index
35	f	13-16 years	0.01	636.6658328	65	Grand Index
35	f	13-16 years	0.011	643.2528211	66	Grand Index
35	f	13-16 years	0.012	645.1747695	66	Grand Index
35	f	13-16 years	0.013	646.4342417	67	Grand Index
35	f	13-16 years	0.014	652.835731	67	Grand Index
35	f	13-16 years	0.015	656	67	Grand Index
35	f	13-16 years	0.016	658	68	Grand Index
35	f	13-16 years	0.017	659.4905092	68	Grand Index
35	f	13-16 years	0.018	662.9439025	69	Grand Index
35	f	13-16 years	0.019	663.9993288	69	Grand Index
35	f	13-16 years	0.02	664.0104805	69	Grand Index
35	f	13-16 years	0.021	665	69	Grand Index
35	f	13-16 years	0.022	667.3171184	70	Grand Index
35	f	13-16 years	0.023	668.4231973	70	Grand Index
35	f	13-16 years	0.024	669.7523685	70	Grand Index
35	f	13-16 years	0.025	670.8861962	71	Grand Index
35	f	13-16 years	0.026	672	71	Grand Index
35	f	13-16 years	0.027	673.198248	71	Grand Index
35	f	13-16 years	0.028	673.8472207	71	Grand Index
35	f	13-16 years	0.029	674.9493543	72	Grand Index
35	f	13-16 years	0.03	675.96	72	Grand Index
35	f	13-16 years	0.031	677.6334545	72	Grand Index
35	f	13-16 years	0.032	678.4209355	72	Grand Index
35	f	13-16 years	0.033	679	72	Grand Index
35	f	13-16 years	0.034	679.6987407	73	Grand Index
35	f	13-16 years	0.035	680.56	73	Grand Index
35	f	13-16 years	0.036	682.3453802	73	Grand Index
35	f	13-16 years	0.037	683.5167206	73	Grand Index
35	f	13-16 years	0.038	684	73	Grand Index
35	f	13-16 years	0.039	684.624	74	Grand Index
35	f	13-16 years	0.04	685.9932012	74	Grand Index
35	f	13-16 years	0.041	686	74	Grand Index
35	f	13-16 years	0.042	687.6799011	74	Grand Index
35	f	13-16 years	0.043	689	74	Grand Index
35	f	13-16 years	0.044	689.455601	74	Grand Index
35	f	13-16 years	0.045	690.3770462	75	Grand Index
35	f	13-16 years	0.046	690.8916681	75	Grand Index
35	f	13-16 years	0.047	691.9773785	75	Grand Index
35	f	13-16 years	0.049	692	75	Grand Index
35	f	13-16 years	0.05	692.870966	75	Grand Index
35	f	13-16 years	0.051	693.816	75	Grand Index
35	f	13-16 years	0.052	694.026034	76	Grand Index
35	f	13-16 years	0.054	695	76	Grand Index
35	f	13-16 years	0.055	695.88	76	Grand Index
35	f	13-16 years	0.057	697	76	Grand Index
35	f	13-16 years	0.06	698	77	Grand Index
35	f	13-16 years	0.061	698.9978978	77	Grand Index
35	f	13-16 years	0.062	699	77	Grand Index

Age	Gender	Education	Percentile	Raw Score	Norm Score	Assessment
35	f	13-16 years	0.063	700	77	Grand Index
35	f	13-16 years	0.064	700.1610818	77	Grand Index
35	f	13-16 years	0.065	701	77	Grand Index
35	f	13-16 years	0.066	701.0016569	77	Grand Index
35	f	13-16 years	0.067	701.855913	78	Grand Index
35	f	13-16 years	0.068	702	78	Grand Index
35	f	13-16 years	0.069	702.0605255	78	Grand Index
35	f	13-16 years	0.07	702.647132	78	Grand Index
35	f	13-16 years	0.071	703	78	Grand Index
35	f	13-16 years	0.072	703.152	78	Grand Index
35	f	13-16 years	0.073	704.047744	78	Grand Index
35	f	13-16 years	0.075	705	78	Grand Index
35	f	13-16 years	0.076	705.0398463	79	Grand Index
35	f	13-16 years	0.079	706	79	Grand Index
35	f	13-16 years	0.081	707	79	Grand Index
35	f	13-16 years	0.082	707.312	79	Grand Index
35	f	13-16 years	0.084	708	79	Grand Index
35	f	13-16 years	0.085	708.964902	79	Grand Index
35	f	13-16 years	0.086	709	80	Grand Index
35	f	13-16 years	0.087	710	80	Grand Index
35	f	13-16 years	0.088	710.408	80	Grand Index
35	f	13-16 years	0.09	711	80	Grand Index
35	f	13-16 years	0.091	711.0270288	80	Grand Index
35	f	13-16 years	0.092	712.0217419	80	Grand Index
35	f	13-16 years	0.093	713.4841725	80	Grand Index
35	f	13-16 years	0.095	714	80	Grand Index
35	f	13-16 years	0.096	715.42948	80	Grand Index
35	f	13-16 years	0.098	716	81	Grand Index
35	f	13-16 years	0.099	716.584	81	Grand Index
35	f	13-16 years	0.1	717.6	81	Grand Index
35	f	13-16 years	0.101	718	81	Grand Index
35	f	13-16 years	0.102	718.4831257	81	Grand Index
35	f	13-16 years	0.104	719	81	Grand Index
35	f	13-16 years	0.107	720	81	Grand Index
35	f	13-16 years	0.108	720.728	81	Grand Index
35	f	13-16 years	0.11	721	82	Grand Index
35	f	13-16 years	0.111	721.776	82	Grand Index
35	f	13-16 years	0.113	722	82	Grand Index
35	f	13-16 years	0.114	722.7689337	82	Grand Index
35	f	13-16 years	0.116	723	82	Grand Index
35	f	13-16 years	0.117	724	82	Grand Index
35	f	13-16 years	0.118	724.9757691	82	Grand Index
35	f	13-16 years	0.119	725	82	Grand Index
35	f	13-16 years	0.121	726	82	Grand Index
35	f	13-16 years	0.122	726.3467305	83	Grand Index
35	f	13-16 years	0.125	727	83	Grand Index
35	f	13-16 years	0.126	727.000327	83	Grand Index
35	f	13-16 years	0.128	728	83	Grand Index
35	f	13-16 years	0.133	729	83	Grand Index
35	f	13-16 years	0.135	730	83	Grand Index
35	f	13-16 years	0.136	730.176	84	Grand Index
35	f	13-16 years	0.139	731	84	Grand Index
35	f	13-16 years	0.14	731.371392	84	Grand Index
35	f	13-16 years	0.143	732	84	Grand Index
35	f	13-16 years	0.144	732.304	84	Grand Index
35	f	13-16 years	0.145	733	84	Grand Index

Age	Gender	Education	Percentile	Raw Score	Norm Score	Assessment
35	f	13-16 years	0.146	733.336	84	Grand Index
35	f	13-16 years	0.148	734	84	Grand Index
35	f	13-16 years	0.149	734.384	84	Grand Index
35	f	13-16 years	0.151	735	85	Grand Index
35	f	13-16 years	0.152	735.7989443	85	Grand Index
35	f	13-16 years	0.154	736	85	Grand Index
35	f	13-16 years	0.155	736.48	85	Grand Index
35	f	13-16 years	0.158	737	85	Grand Index
35	f	13-16 years	0.159	737.449312	85	Grand Index
35	f	13-16 years	0.164	738	85	Grand Index
35	f	13-16 years	0.165	738.6903379	85	Grand Index
35	f	13-16 years	0.166	739	85	Grand Index
35	f	13-16 years	0.167	739.6496668	86	Grand Index
35	f	13-16 years	0.169	740	86	Grand Index
35	f	13-16 years	0.17	740.365558	86	Grand Index
35	f	13-16 years	0.174	741	86	Grand Index
35	f	13-16 years	0.178	742	86	Grand Index
35	f	13-16 years	0.179	742.9632895	86	Grand Index
35	f	13-16 years	0.181	743	86	Grand Index
35	f	13-16 years	0.182	743.912	86	Grand Index
35	f	13-16 years	0.183	744	86	Grand Index
35	f	13-16 years	0.184	744.944	86	Grand Index
35	f	13-16 years	0.187	745	87	Grand Index
35	f	13-16 years	0.188	745.0038352	87	Grand Index
35	f	13-16 years	0.189	746	87	Grand Index
35	f	13-16 years	0.19	746.0221712	87	Grand Index
35	f	13-16 years	0.194	747	87	Grand Index
35	f	13-16 years	0.195	747.0276112	87	Grand Index
35	f	13-16 years	0.2	748	87	Grand Index
35	f	13-16 years	0.201	748.0776173	87	Grand Index
35	f	13-16 years	0.202	748.6549799	87	Grand Index
35	f	13-16 years	0.205	749	88	Grand Index
35	f	13-16 years	0.206	749.2462897	88	Grand Index
35	f	13-16 years	0.207	749.8873358	88	Grand Index
35	f	13-16 years	0.21	750	88	Grand Index
35	f	13-16 years	0.211	750.7808014	88	Grand Index
35	f	13-16 years	0.214	751	88	Grand Index
35	f	13-16 years	0.215	751.0089168	88	Grand Index
35	f	13-16 years	0.216	751.754076	88	Grand Index
35	f	13-16 years	0.217	752	88	Grand Index
35	f	13-16 years	0.218	752.9819786	88	Grand Index
35	f	13-16 years	0.223	753	89	Grand Index
35	f	13-16 years	0.224	753.584	89	Grand Index
35	f	13-16 years	0.227	754	89	Grand Index
35	f	13-16 years	0.228	754.0066608	89	Grand Index
35	f	13-16 years	0.231	755	89	Grand Index
35	f	13-16 years	0.233	756	89	Grand Index
35	f	13-16 years	0.234	756.9396965	89	Grand Index
35	f	13-16 years	0.239	757	89	Grand Index
35	f	13-16 years	0.24	757.951895	89	Grand Index
35	f	13-16 years	0.243	758	90	Grand Index
35	f	13-16 years	0.244	758.0746422	90	Grand Index
35	f	13-16 years	0.245	758.9547739	90	Grand Index
35	f	13-16 years	0.246	759	90	Grand Index
35	f	13-16 years	0.249	760	90	Grand Index
35	f	13-16 years	0.25	760.974381	90	Grand Index

Age	Gender	Education	Percentile	Raw Score	Norm Score	Assessment
35	f	13-16 years	0.254	761	90	Grand Index
35	f	13-16 years	0.255	761.7785045	90	Grand Index
35	f	13-16 years	0.257	762	90	Grand Index
35	f	13-16 years	0.258	762.0131885	90	Grand Index
35	f	13-16 years	0.259	762.4242997	90	Grand Index
35	f	13-16 years	0.261	763	90	Grand Index
35	f	13-16 years	0.263	764	90	Grand Index
35	f	13-16 years	0.264	764.0274354	91	Grand Index
35	f	13-16 years	0.266	765	91	Grand Index
35	f	13-16 years	0.267	765.1808595	91	Grand Index
35	f	13-16 years	0.27	766	91	Grand Index
35	f	13-16 years	0.271	766.909727	91	Grand Index
35	f	13-16 years	0.274	767	91	Grand Index
35	f	13-16 years	0.278	768	91	Grand Index
35	f	13-16 years	0.279	768.9473419	91	Grand Index
35	f	13-16 years	0.284	769	91	Grand Index
35	f	13-16 years	0.285	770	91	Grand Index
35	f	13-16 years	0.286	770.9923357	92	Grand Index
35	f	13-16 years	0.29	771	92	Grand Index
35	f	13-16 years	0.295	772	92	Grand Index
35	f	13-16 years	0.296	772.3118835	92	Grand Index
35	f	13-16 years	0.297	772.8804701	92	Grand Index
35	f	13-16 years	0.299	773	92	Grand Index
35	f	13-16 years	0.303	774	92	Grand Index
35	f	13-16 years	0.304	774.1520357	92	Grand Index
35	f	13-16 years	0.308	775	92	Grand Index
35	f	13-16 years	0.309	775.944	93	Grand Index
35	f	13-16 years	0.314	776	93	Grand Index
35	f	13-16 years	0.317	777	93	Grand Index
35	f	13-16 years	0.318	777.0457679	93	Grand Index
35	f	13-16 years	0.319	777.9037569	93	Grand Index
35	f	13-16 years	0.322	778	93	Grand Index
35	f	13-16 years	0.323	778.4814512	93	Grand Index
35	f	13-16 years	0.328	779	93	Grand Index
35	f	13-16 years	0.329	779.3256035	93	Grand Index
35	f	13-16 years	0.332	780	93	Grand Index
35	f	13-16 years	0.333	780.3389665	94	Grand Index
35	f	13-16 years	0.339	781	94	Grand Index
35	f	13-16 years	0.34	781.4702738	94	Grand Index
35	f	13-16 years	0.344	782	94	Grand Index
35	f	13-16 years	0.345	782.5033186	94	Grand Index
35	f	13-16 years	0.351	783	94	Grand Index
35	f	13-16 years	0.352	783.3226679	94	Grand Index
35	f	13-16 years	0.357	784	95	Grand Index
35	f	13-16 years	0.358	784.6689602	95	Grand Index
35	f	13-16 years	0.363	785	95	Grand Index
35	f	13-16 years	0.364	785.9985681	95	Grand Index
35	f	13-16 years	0.368	786	95	Grand Index
35	f	13-16 years	0.369	786.904	95	Grand Index
35	f	13-16 years	0.373	787	95	Grand Index
35	f	13-16 years	0.374	787.2989926	95	Grand Index
35	f	13-16 years	0.381	788	95	Grand Index
35	f	13-16 years	0.382	788.0248471	95	Grand Index
35	f	13-16 years	0.383	788.5249103	96	Grand Index
35	f	13-16 years	0.384	789	96	Grand Index
35	f	13-16 years	0.385	790	96	Grand Index

Age	Gender	Education	Percentile	Raw Score	Norm Score	Assessment
35	f	13-16 years	0.391	791	96	Grand Index
35	f	13-16 years	0.392	791.3901896	96	Grand Index
35	f	13-16 years	0.395	792	96	Grand Index
35	f	13-16 years	0.396	792.2132642	96	Grand Index
35	f	13-16 years	0.4	793	96	Grand Index
35	f	13-16 years	0.401	793.0886904	96	Grand Index
35	f	13-16 years	0.409	794	97	Grand Index
35	f	13-16 years	0.41	794.56	97	Grand Index
35	f	13-16 years	0.415	795	97	Grand Index
35	f	13-16 years	0.416	795.6937191	97	Grand Index
35	f	13-16 years	0.422	796	97	Grand Index
35	f	13-16 years	0.423	796.7189825	97	Grand Index
35	f	13-16 years	0.427	797	97	Grand Index
35	f	13-16 years	0.428	797.9551261	97	Grand Index
35	f	13-16 years	0.43	798	97	Grand Index
35	f	13-16 years	0.431	798.5600962	97	Grand Index
35	f	13-16 years	0.434	799	98	Grand Index
35	f	13-16 years	0.435	799.4232513	98	Grand Index
35	f	13-16 years	0.439	800	98	Grand Index
35	f	13-16 years	0.44	800.1362533	98	Grand Index
35	f	13-16 years	0.444	801	98	Grand Index
35	f	13-16 years	0.449	802	98	Grand Index
35	f	13-16 years	0.45	802.3101559	98	Grand Index
35	f	13-16 years	0.456	803	98	Grand Index
35	f	13-16 years	0.457	803.0276225	98	Grand Index
35	f	13-16 years	0.458	803.5182832	98	Grand Index
35	f	13-16 years	0.46	804	98	Grand Index
35	f	13-16 years	0.461	804.376	99	Grand Index
35	f	13-16 years	0.464	805	99	Grand Index
35	f	13-16 years	0.465	805.1531563	99	Grand Index
35	f	13-16 years	0.471	806	99	Grand Index
35	f	13-16 years	0.475	807	99	Grand Index
35	f	13-16 years	0.476	807.1545076	99	Grand Index
35	f	13-16 years	0.477	807.9035025	99	Grand Index
35	f	13-16 years	0.481	808	99	Grand Index
35	f	13-16 years	0.482	808.1974454	99	Grand Index
35	f	13-16 years	0.483	808.9733275	99	Grand Index
35	f	13-16 years	0.484	809	99	Grand Index
35	f	13-16 years	0.485	809.0627523	99	Grand Index
35	f	13-16 years	0.486	809.9517957	99	Grand Index
35	f	13-16 years	0.487	810	100	Grand Index
35	f	13-16 years	0.488	810.0141312	100	Grand Index
35	f	13-16 years	0.489	810.8709147	100	Grand Index
35	f	13-16 years	0.491	811	100	Grand Index
35	f	13-16 years	0.492	811.0472587	100	Grand Index
35	f	13-16 years	0.493	811.5906213	100	Grand Index
35	f	13-16 years	0.5	812	100	Grand Index
35	f	13-16 years	0.501	812.5364995	100	Grand Index
35	f	13-16 years	0.505	813	100	Grand Index
35	f	13-16 years	0.511	814	100	Grand Index
35	f	13-16 years	0.512	814.3853955	100	Grand Index
35	f	13-16 years	0.517	815	101	Grand Index
35	f	13-16 years	0.518	815.0499537	101	Grand Index
35	f	13-16 years	0.519	815.6130979	101	Grand Index
35	f	13-16 years	0.524	816	101	Grand Index
35	f	13-16 years	0.525	816.9431712	101	Grand Index

Age	Gender	Education	Percentile	Raw Score	Norm Score	Assessment
35	f	13-16 years	0.532	817	101	Grand Index
35	f	13-16 years	0.533	817.5541325	101	Grand Index
35	f	13-16 years	0.537	818	101	Grand Index
35	f	13-16 years	0.538	818.0069288	101	Grand Index
35	f	13-16 years	0.541	819	102	Grand Index
35	f	13-16 years	0.542	819.8096796	102	Grand Index
35	f	13-16 years	0.547	820	102	Grand Index
35	f	13-16 years	0.553	821	102	Grand Index
35	f	13-16 years	0.554	821.8849231	102	Grand Index
35	f	13-16 years	0.557	822	102	Grand Index
35	f	13-16 years	0.558	822.9289482	102	Grand Index
35	f	13-16 years	0.566	823	102	Grand Index
35	f	13-16 years	0.567	823.0814615	103	Grand Index
35	f	13-16 years	0.571	824	103	Grand Index
35	f	13-16 years	0.572	824.152	103	Grand Index
35	f	13-16 years	0.577	825	103	Grand Index
35	f	13-16 years	0.578	825.1528079	103	Grand Index
35	f	13-16 years	0.585	826	103	Grand Index
35	f	13-16 years	0.586	826.0966794	103	Grand Index
35	f	13-16 years	0.588	827	103	Grand Index
35	f	13-16 years	0.589	827.2483509	103	Grand Index
35	f	13-16 years	0.59	827.7170382	103	Grand Index
35	f	13-16 years	0.593	828	104	Grand Index
35	f	13-16 years	0.594	829	104	Grand Index
35	f	13-16 years	0.595	829.52	104	Grand Index
35	f	13-16 years	0.598	830	104	Grand Index
35	f	13-16 years	0.599	830.584	104	Grand Index
35	f	13-16 years	0.602	831	104	Grand Index
35	f	13-16 years	0.603	831.0916621	104	Grand Index
35	f	13-16 years	0.604	831.6777736	104	Grand Index
35	f	13-16 years	0.607	832	104	Grand Index
35	f	13-16 years	0.608	832.728	104	Grand Index
35	f	13-16 years	0.614	833	104	Grand Index
35	f	13-16 years	0.616	834	104	Grand Index
35	f	13-16 years	0.617	834.872	104	Grand Index
35	f	13-16 years	0.618	835	105	Grand Index
35	f	13-16 years	0.619	835.9727677	105	Grand Index
35	f	13-16 years	0.626	836	105	Grand Index
35	f	13-16 years	0.627	836.232368	105	Grand Index
35	f	13-16 years	0.631	837	105	Grand Index
35	f	13-16 years	0.632	837.9390328	105	Grand Index
35	f	13-16 years	0.634	838	105	Grand Index
35	f	13-16 years	0.635	838.074364	105	Grand Index
35	f	13-16 years	0.638	839	105	Grand Index
35	f	13-16 years	0.639	839.0343226	105	Grand Index
35	f	13-16 years	0.644	840	106	Grand Index
35	f	13-16 years	0.645	840.3451364	106	Grand Index
35	f	13-16 years	0.646	840.976539	106	Grand Index
35	f	13-16 years	0.649	841	106	Grand Index
35	f	13-16 years	0.65	841.4	106	Grand Index
35	f	13-16 years	0.652	842	106	Grand Index
35	f	13-16 years	0.653	842.448	106	Grand Index
35	f	13-16 years	0.656	843	106	Grand Index
35	f	13-16 years	0.657	843.8981962	106	Grand Index
35	f	13-16 years	0.661	844	106	Grand Index
35	f	13-16 years	0.662	844.8949517	106	Grand Index

Age	Gender	Education	Percentile	Raw Score	Norm Score	Assessment
35	f	13-16 years	0.663	845	106	Grand Index
35	f	13-16 years	0.664	845.351704	106	Grand Index
35	f	13-16 years	0.665	845.8615056	106	Grand Index
35	f	13-16 years	0.666	846.0212009	106	Grand Index
35	f	13-16 years	0.667	846.7962674	106	Grand Index
35	f	13-16 years	0.671	847	107	Grand Index
35	f	13-16 years	0.672	847.1837455	107	Grand Index
35	f	13-16 years	0.673	847.9904613	107	Grand Index
35	f	13-16 years	0.675	848	107	Grand Index
35	f	13-16 years	0.676	848.077776	107	Grand Index
35	f	13-16 years	0.679	849	107	Grand Index
35	f	13-16 years	0.68	849.0347766	107	Grand Index
35	f	13-16 years	0.683	850	107	Grand Index
35	f	13-16 years	0.684	850.6702164	107	Grand Index
35	f	13-16 years	0.688	851	107	Grand Index
35	f	13-16 years	0.689	851.024	107	Grand Index
35	f	13-16 years	0.692	852	108	Grand Index
35	f	13-16 years	0.693	852.1445175	108	Grand Index
35	f	13-16 years	0.699	853	108	Grand Index
35	f	13-16 years	0.7	853.5494554	108	Grand Index
35	f	13-16 years	0.702	854	108	Grand Index
35	f	13-16 years	0.705	855	108	Grand Index
35	f	13-16 years	0.706	855.119725	108	Grand Index
35	f	13-16 years	0.707	855.904014	108	Grand Index
35	f	13-16 years	0.711	856	108	Grand Index
35	f	13-16 years	0.712	856.392	108	Grand Index
35	f	13-16 years	0.716	857	109	Grand Index
35	f	13-16 years	0.717	857.046596	109	Grand Index
35	f	13-16 years	0.718	857.8677758	109	Grand Index
35	f	13-16 years	0.72	858	109	Grand Index
35	f	13-16 years	0.721	858.536	109	Grand Index
35	f	13-16 years	0.723	859	109	Grand Index
35	f	13-16 years	0.724	859.4923761	109	Grand Index
35	f	13-16 years	0.728	860	109	Grand Index
35	f	13-16 years	0.729	860.8135459	109	Grand Index
35	f	13-16 years	0.731	861	109	Grand Index
35	f	13-16 years	0.732	861.712	109	Grand Index
35	f	13-16 years	0.734	862	109	Grand Index
35	f	13-16 years	0.735	862.071811	109	Grand Index
35	f	13-16 years	0.737	863	110	Grand Index
35	f	13-16 years	0.738	863.808	110	Grand Index
35	f	13-16 years	0.744	864	110	Grand Index
35	f	13-16 years	0.745	864.8197841	110	Grand Index
35	f	13-16 years	0.748	865	110	Grand Index
35	f	13-16 years	0.749	865.984	110	Grand Index
35	f	13-16 years	0.751	866	110	Grand Index
35	f	13-16 years	0.756	867	110	Grand Index
35	f	13-16 years	0.757	867.1673428	110	Grand Index
35	f	13-16 years	0.758	868	110	Grand Index
35	f	13-16 years	0.759	868.0005762	111	Grand Index
35	f	13-16 years	0.76	868.4138867	111	Grand Index
35	f	13-16 years	0.764	869	111	Grand Index
35	f	13-16 years	0.765	869.2328641	111	Grand Index
35	f	13-16 years	0.766	869.9973455	111	Grand Index
35	f	13-16 years	0.769	870	111	Grand Index
35	f	13-16 years	0.77	870.32	111	Grand Index

Age	Gender	Education	Percentile	Raw Score	Norm Score	Assessment
35	f	13-16 years	0.775	871	111	Grand Index
35	f	13-16 years	0.779	872	112	Grand Index
35	f	13-16 years	0.78	872.6591641	112	Grand Index
35	f	13-16 years	0.784	873	112	Grand Index
35	f	13-16 years	0.785	873.2647365	112	Grand Index
35	f	13-16 years	0.786	873.7314667	112	Grand Index
35	f	13-16 years	0.787	874	112	Grand Index
35	f	13-16 years	0.788	874.608	112	Grand Index
35	f	13-16 years	0.79	875	112	Grand Index
35	f	13-16 years	0.791	875.2149573	112	Grand Index
35	f	13-16 years	0.795	876	112	Grand Index
35	f	13-16 years	0.796	876.2910522	112	Grand Index
35	f	13-16 years	0.8	877	113	Grand Index
35	f	13-16 years	0.801	877.816	113	Grand Index
35	f	13-16 years	0.803	878	113	Grand Index
35	f	13-16 years	0.804	878.864	113	Grand Index
35	f	13-16 years	0.809	879	113	Grand Index
35	f	13-16 years	0.81	879.5247444	113	Grand Index
35	f	13-16 years	0.811	879.9311983	113	Grand Index
35	f	13-16 years	0.812	880	113	Grand Index
35	f	13-16 years	0.815	881	113	Grand Index
35	f	13-16 years	0.816	881.0016979	114	Grand Index
35	f	13-16 years	0.817	881.2297054	114	Grand Index
35	f	13-16 years	0.819	882	114	Grand Index
35	f	13-16 years	0.82	882.0142326	114	Grand Index
35	f	13-16 years	0.821	882.9883275	114	Grand Index
35	f	13-16 years	0.826	883	114	Grand Index
35	f	13-16 years	0.828	884	114	Grand Index
35	f	13-16 years	0.829	884.264	114	Grand Index
35	f	13-16 years	0.83	885.28	114	Grand Index
35	f	13-16 years	0.831	886.0096	114	Grand Index
35	f	13-16 years	0.834	887	115	Grand Index
35	f	13-16 years	0.835	887.959235	115	Grand Index
35	f	13-16 years	0.836	888	115	Grand Index
35	f	13-16 years	0.837	888.0310897	115	Grand Index
35	f	13-16 years	0.841	889	115	Grand Index
35	f	13-16 years	0.842	889.5392586	115	Grand Index
35	f	13-16 years	0.846	890	115	Grand Index
35	f	13-16 years	0.848	891	115	Grand Index
35	f	13-16 years	0.849	891.3388235	115	Grand Index
35	f	13-16 years	0.851	892	116	Grand Index
35	f	13-16 years	0.852	892.3019435	116	Grand Index
35	f	13-16 years	0.853	892.9940435	116	Grand Index
35	f	13-16 years	0.854	893	116	Grand Index
35	f	13-16 years	0.855	893.5570899	116	Grand Index
35	f	13-16 years	0.856	893.997367	116	Grand Index
35	f	13-16 years	0.859	894	116	Grand Index
35	f	13-16 years	0.86	894.7724827	116	Grand Index
35	f	13-16 years	0.862	895	116	Grand Index
35	f	13-16 years	0.865	896	117	Grand Index
35	f	13-16 years	0.866	896.6329359	117	Grand Index
35	f	13-16 years	0.868	897	117	Grand Index
35	f	13-16 years	0.869	897.7672357	117	Grand Index
35	f	13-16 years	0.872	898	117	Grand Index
35	f	13-16 years	0.873	898.968	117	Grand Index
35	f	13-16 years	0.874	899	117	Grand Index

Age	Gender	Education	Percentile	Raw Score	Norm Score	Assessment
35	f	13-16 years	0.875	899.2063492	117	Grand Index
35	f	13-16 years	0.876	900.0147122	117	Grand Index
35	f	13-16 years	0.877	900.9935752	117	Grand Index
35	f	13-16 years	0.878	901	117	Grand Index
35	f	13-16 years	0.879	901.0954071	118	Grand Index
35	f	13-16 years	0.882	902	118	Grand Index
35	f	13-16 years	0.883	902.082852	118	Grand Index
35	f	13-16 years	0.884	903	118	Grand Index
35	f	13-16 years	0.885	903.16	118	Grand Index
35	f	13-16 years	0.887	904	118	Grand Index
35	f	13-16 years	0.888	904.0587038	118	Grand Index
35	f	13-16 years	0.889	905	118	Grand Index
35	f	13-16 years	0.89	905.0342193	118	Grand Index
35	f	13-16 years	0.891	905.6910299	118	Grand Index
35	f	13-16 years	0.895	906	119	Grand Index
35	f	13-16 years	0.896	906.8103907	119	Grand Index
35	f	13-16 years	0.898	907	119	Grand Index
35	f	13-16 years	0.899	908	119	Grand Index
35	f	13-16 years	0.9	908.0709746	119	Grand Index
35	f	13-16 years	0.901	909	119	Grand Index
35	f	13-16 years	0.902	909.1473865	119	Grand Index
35	f	13-16 years	0.903	910	119	Grand Index
35	f	13-16 years	0.904	910.464	120	Grand Index
35	f	13-16 years	0.907	911	120	Grand Index
35	f	13-16 years	0.908	911.5534312	120	Grand Index
35	f	13-16 years	0.909	912	120	Grand Index
35	f	13-16 years	0.91	912.4191318	120	Grand Index
35	f	13-16 years	0.911	913	120	Grand Index
35	f	13-16 years	0.912	914	120	Grand Index
35	f	13-16 years	0.913	914.9662089	120	Grand Index
35	f	13-16 years	0.914	915	120	Grand Index
35	f	13-16 years	0.915	915.64	121	Grand Index
35	f	13-16 years	0.916	916	121	Grand Index
35	f	13-16 years	0.917	917	121	Grand Index
35	f	13-16 years	0.918	917.3933496	121	Grand Index
35	f	13-16 years	0.92	918	121	Grand Index
35	f	13-16 years	0.921	918.3424717	121	Grand Index
35	f	13-16 years	0.925	919	122	Grand Index
35	f	13-16 years	0.926	919.8194891	122	Grand Index
35	f	13-16 years	0.927	920.8666686	122	Grand Index
35	f	13-16 years	0.928	921.8952539	122	Grand Index
35	f	13-16 years	0.929	922	122	Grand Index
35	f	13-16 years	0.93	923	122	Grand Index
35	f	13-16 years	0.931	923.896	122	Grand Index
35	f	13-16 years	0.933	925	122	Grand Index
35	f	13-16 years	0.934	926.0620158	123	Grand Index
35	f	13-16 years	0.935	926.964209	123	Grand Index
35	f	13-16 years	0.936	927.976	123	Grand Index
35	f	13-16 years	0.937	928	123	Grand Index
35	f	13-16 years	0.938	928.4914958	123	Grand Index
35	f	13-16 years	0.939	929	123	Grand Index
35	f	13-16 years	0.94	929.0063432	123	Grand Index
35	f	13-16 years	0.941	929.5990872	123	Grand Index
35	f	13-16 years	0.942	930	124	Grand Index
35	f	13-16 years	0.943	931.096315	124	Grand Index
35	f	13-16 years	0.944	932.9707069	124	Grand Index

Age	Gender	Education	Percentile	Raw Score	Norm Score	Assessment
35	f	13-16 years	0.945	933.332093	124	Grand Index
35	f	13-16 years	0.946	934.1482475	124	Grand Index
35	f	13-16 years	0.948	936	124	Grand Index
35	f	13-16 years	0.949	936.4838974	125	Grand Index
35	f	13-16 years	0.95	937.1342563	125	Grand Index
35	f	13-16 years	0.951	939	125	Grand Index
35	f	13-16 years	0.952	939.232	125	Grand Index
35	f	13-16 years	0.953	940	125	Grand Index
35	f	13-16 years	0.954	941.264	125	Grand Index
35	f	13-16 years	0.956	942	126	Grand Index
35	f	13-16 years	0.957	942.4145978	126	Grand Index
35	f	13-16 years	0.958	944.2394454	126	Grand Index
35	f	13-16 years	0.959	945.2069873	126	Grand Index
35	f	13-16 years	0.96	946.36	126	Grand Index
35	f	13-16 years	0.961	948	126	Grand Index
35	f	13-16 years	0.962	949	127	Grand Index
35	f	13-16 years	0.963	949.0714745	127	Grand Index
35	f	13-16 years	0.964	949.7189677	127	Grand Index
35	f	13-16 years	0.965	950.2530312	127	Grand Index
35	f	13-16 years	0.966	950.9168408	127	Grand Index
35	f	13-16 years	0.967	951.472	128	Grand Index
35	f	13-16 years	0.968	953	128	Grand Index
35	f	13-16 years	0.969	953.784942	128	Grand Index
35	f	13-16 years	0.97	956.0013897	128	Grand Index
35	f	13-16 years	0.971	958	128	Grand Index
35	f	13-16 years	0.972	958.5760661	129	Grand Index
35	f	13-16 years	0.973	959.484646	129	Grand Index
35	f	13-16 years	0.974	960.996981	129	Grand Index
35	f	13-16 years	0.975	961	129	Grand Index
35	f	13-16 years	0.976	962.5564189	130	Grand Index
35	f	13-16 years	0.977	963.9041302	130	Grand Index
35	f	13-16 years	0.978	966.296	130	Grand Index
35	f	13-16 years	0.979	967	131	Grand Index
35	f	13-16 years	0.98	968.266806	131	Grand Index
35	f	13-16 years	0.981	970.3888887	131	Grand Index
35	f	13-16 years	0.982	973.9773248	131	Grand Index
35	f	13-16 years	0.983	977.3018981	132	Grand Index
35	f	13-16 years	0.984	979.6655028	132	Grand Index
35	f	13-16 years	0.985	982.28	133	Grand Index
35	f	13-16 years	0.986	984.2170808	133	Grand Index
35	f	13-16 years	0.987	985.7208092	133	Grand Index
35	f	13-16 years	0.988	986	134	Grand Index
35	f	13-16 years	0.989	990.4798582	134	Grand Index
35	f	13-16 years	0.99	995.36	135	Grand Index
35	f	13-16 years	0.991	1002.181144	135	Grand Index
35	f	13-16 years	0.992	1004.886204	136	Grand Index
35	f	13-16 years	0.993	1009.847273	137	Grand Index
35	f	13-16 years	0.994	1014.59697	138	Grand Index
35	f	13-16 years	0.995	1019.709691	139	Grand Index
35	f	13-16 years	0.996	1026.753719	140	Grand Index
35	f	13-16 years	0.997	1033.597232	141	Grand Index
35	f	13-16 years	0.998	1041.818495	143	Grand Index
35	f	13-16 years	0.999	1047.989494	146	Grand Index
35	m	13-16 years	0.001	568.4418087	54	Grand Index
35	m	13-16 years	0.002	590.512	57	Grand Index
35	m	13-16 years	0.003	609	59	Grand Index

Age	Gender	Education	Percentile	Raw Score	Norm Score	Assessment
35	m	13-16 years	0.004	614.554684	60	Grand Index
35	m	13-16 years	0.005	618.5885073	61	Grand Index
35	m	13-16 years	0.006	626.134	62	Grand Index
35	m	13-16 years	0.007	630.959026	63	Grand Index
35	m	13-16 years	0.008	637.4053503	64	Grand Index
35	m	13-16 years	0.009	639	65	Grand Index
35	m	13-16 years	0.01	639.3922708	65	Grand Index
35	m	13-16 years	0.011	644	66	Grand Index
35	m	13-16 years	0.012	645	66	Grand Index
35	m	13-16 years	0.013	645.1519699	67	Grand Index
35	m	13-16 years	0.014	645.9954445	67	Grand Index
35	m	13-16 years	0.015	647.6774574	67	Grand Index
35	m	13-16 years	0.016	651	68	Grand Index
35	m	13-16 years	0.017	651.2138432	68	Grand Index
35	m	13-16 years	0.018	652.402	69	Grand Index
35	m	13-16 years	0.019	655.1874598	69	Grand Index
35	m	13-16 years	0.02	657.1712448	69	Grand Index
35	m	13-16 years	0.021	658.4871	69	Grand Index
35	m	13-16 years	0.022	660.0002288	70	Grand Index
35	m	13-16 years	0.023	661.2078824	70	Grand Index
35	m	13-16 years	0.024	662	70	Grand Index
35	m	13-16 years	0.025	663.2810272	71	Grand Index
35	m	13-16 years	0.026	663.9863216	71	Grand Index
35	m	13-16 years	0.027	665.0007226	71	Grand Index
35	m	13-16 years	0.028	665.4990289	71	Grand Index
35	m	13-16 years	0.029	666	72	Grand Index
35	m	13-16 years	0.03	666.9140894	72	Grand Index
35	m	13-16 years	0.032	668	72	Grand Index
35	m	13-16 years	0.033	668.237	72	Grand Index
35	m	13-16 years	0.035	670	73	Grand Index
35	m	13-16 years	0.036	670.804	73	Grand Index
35	m	13-16 years	0.037	671	73	Grand Index
35	m	13-16 years	0.038	672	73	Grand Index
35	m	13-16 years	0.039	673	74	Grand Index
35	m	13-16 years	0.04	673.5079481	74	Grand Index
35	m	13-16 years	0.041	674	74	Grand Index
35	m	13-16 years	0.042	674.9870108	74	Grand Index
35	m	13-16 years	0.043	675.8722097	74	Grand Index
35	m	13-16 years	0.044	676.431501	74	Grand Index
35	m	13-16 years	0.045	677.4024395	75	Grand Index
35	m	13-16 years	0.046	677.9949507	75	Grand Index
35	m	13-16 years	0.047	679.2581435	75	Grand Index
35	m	13-16 years	0.048	679.9557499	75	Grand Index
35	m	13-16 years	0.049	680.261	75	Grand Index
35	m	13-16 years	0.05	681	75	Grand Index
35	m	13-16 years	0.051	682.4942241	75	Grand Index
35	m	13-16 years	0.052	683	76	Grand Index
35	m	13-16 years	0.053	683.8770929	76	Grand Index
35	m	13-16 years	0.054	684.206	76	Grand Index
35	m	13-16 years	0.055	686.79	76	Grand Index
35	m	13-16 years	0.056	688	76	Grand Index
35	m	13-16 years	0.057	688.076575	76	Grand Index
35	m	13-16 years	0.058	688.6717393	76	Grand Index
35	m	13-16 years	0.059	689	77	Grand Index
35	m	13-16 years	0.06	689.34	77	Grand Index
35	m	13-16 years	0.061	690	77	Grand Index

Age	Gender	Education	Percentile	Raw Score	Norm Score	Assessment
35	m	13-16 years	0.062	690.7253576	77	Grand Index
35	m	13-16 years	0.063	691	77	Grand Index
35	m	13-16 years	0.064	692	77	Grand Index
35	m	13-16 years	0.066	693	77	Grand Index
35	m	13-16 years	0.067	693.6879728	78	Grand Index
35	m	13-16 years	0.069	694	78	Grand Index
35	m	13-16 years	0.07	694.0837736	78	Grand Index
35	m	13-16 years	0.072	695	78	Grand Index
35	m	13-16 years	0.073	695.9957377	78	Grand Index
35	m	13-16 years	0.074	696	78	Grand Index
35	m	13-16 years	0.075	696.0999188	78	Grand Index
35	m	13-16 years	0.076	697	79	Grand Index
35	m	13-16 years	0.077	697.48724	79	Grand Index
35	m	13-16 years	0.078	698.742	79	Grand Index
35	m	13-16 years	0.079	699.931	79	Grand Index
35	m	13-16 years	0.08	700	79	Grand Index
35	m	13-16 years	0.081	700.4442354	79	Grand Index
35	m	13-16 years	0.082	701.498	79	Grand Index
35	m	13-16 years	0.083	702.687	79	Grand Index
35	m	13-16 years	0.084	704.0413493	79	Grand Index
35	m	13-16 years	0.085	705	79	Grand Index
35	m	13-16 years	0.086	706	80	Grand Index
35	m	13-16 years	0.087	706.7143772	80	Grand Index
35	m	13-16 years	0.088	707	80	Grand Index
35	m	13-16 years	0.089	707.9706334	80	Grand Index
35	m	13-16 years	0.09	708.0006261	80	Grand Index
35	m	13-16 years	0.091	708.7734789	80	Grand Index
35	m	13-16 years	0.092	709	80	Grand Index
35	m	13-16 years	0.093	709.5593478	80	Grand Index
35	m	13-16 years	0.095	710	80	Grand Index
35	m	13-16 years	0.096	711	80	Grand Index
35	m	13-16 years	0.097	711.3028317	81	Grand Index
35	m	13-16 years	0.098	712	81	Grand Index
35	m	13-16 years	0.099	712.0490601	81	Grand Index
35	m	13-16 years	0.1	712.9111161	81	Grand Index
35	m	13-16 years	0.101	714	81	Grand Index
35	m	13-16 years	0.104	715	81	Grand Index
35	m	13-16 years	0.105	715.865574	81	Grand Index
35	m	13-16 years	0.106	716	81	Grand Index
35	m	13-16 years	0.107	716.223	81	Grand Index
35	m	13-16 years	0.109	717	82	Grand Index
35	m	13-16 years	0.11	717.484068	82	Grand Index
35	m	13-16 years	0.111	717.9958091	82	Grand Index
35	m	13-16 years	0.112	718.0416971	82	Grand Index
35	m	13-16 years	0.113	718.6530549	82	Grand Index
35	m	13-16 years	0.114	719	82	Grand Index
35	m	13-16 years	0.115	719.0042735	82	Grand Index
35	m	13-16 years	0.116	720	82	Grand Index
35	m	13-16 years	0.117	721	82	Grand Index
35	m	13-16 years	0.118	721.2027506	82	Grand Index
35	m	13-16 years	0.119	721.9225977	82	Grand Index
35	m	13-16 years	0.12	722	82	Grand Index
35	m	13-16 years	0.121	722.0401976	82	Grand Index
35	m	13-16 years	0.122	723.0464746	83	Grand Index
35	m	13-16 years	0.124	724	83	Grand Index
35	m	13-16 years	0.125	724.6560734	83	Grand Index

Age	Gender	Education	Percentile	Raw Score	Norm Score	Assessment
35	m	13-16 years	0.126	725	83	Grand Index
35	m	13-16 years	0.127	725.0028585	83	Grand Index
35	m	13-16 years	0.129	726	83	Grand Index
35	m	13-16 years	0.131	727	83	Grand Index
35	m	13-16 years	0.132	727.9571131	83	Grand Index
35	m	13-16 years	0.133	729	83	Grand Index
35	m	13-16 years	0.134	729.5093069	83	Grand Index
35	m	13-16 years	0.136	730	84	Grand Index
35	m	13-16 years	0.138	731	84	Grand Index
35	m	13-16 years	0.139	732	84	Grand Index
35	m	13-16 years	0.14	732.2340177	84	Grand Index
35	m	13-16 years	0.141	732.8479058	84	Grand Index
35	m	13-16 years	0.143	733	84	Grand Index
35	m	13-16 years	0.144	733.2471578	84	Grand Index
35	m	13-16 years	0.146	734	84	Grand Index
35	m	13-16 years	0.147	734.0018213	84	Grand Index
35	m	13-16 years	0.148	735	84	Grand Index
35	m	13-16 years	0.149	735.2350526	84	Grand Index
35	m	13-16 years	0.151	736	85	Grand Index
35	m	13-16 years	0.152	736.728	85	Grand Index
35	m	13-16 years	0.153	737	85	Grand Index
35	m	13-16 years	0.158	738	85	Grand Index
35	m	13-16 years	0.159	738.1239665	85	Grand Index
35	m	13-16 years	0.162	739	85	Grand Index
35	m	13-16 years	0.163	739.7040041	85	Grand Index
35	m	13-16 years	0.164	739.9999143	85	Grand Index
35	m	13-16 years	0.168	740	86	Grand Index
35	m	13-16 years	0.169	740.1747432	86	Grand Index
35	m	13-16 years	0.17	740.833443	86	Grand Index
35	m	13-16 years	0.172	741	86	Grand Index
35	m	13-16 years	0.173	741.6934255	86	Grand Index
35	m	13-16 years	0.177	742	86	Grand Index
35	m	13-16 years	0.179	743	86	Grand Index
35	m	13-16 years	0.18	743.02	86	Grand Index
35	m	13-16 years	0.183	744	86	Grand Index
35	m	13-16 years	0.184	744.0794745	86	Grand Index
35	m	13-16 years	0.186	745	87	Grand Index
35	m	13-16 years	0.187	745.3596845	87	Grand Index
35	m	13-16 years	0.19	746	87	Grand Index
35	m	13-16 years	0.191	746.1461779	87	Grand Index
35	m	13-16 years	0.192	746.8929564	87	Grand Index
35	m	13-16 years	0.195	747	87	Grand Index
35	m	13-16 years	0.196	747.1875805	87	Grand Index
35	m	13-16 years	0.197	748	87	Grand Index
35	m	13-16 years	0.198	748.422	87	Grand Index
35	m	13-16 years	0.2	749	87	Grand Index
35	m	13-16 years	0.201	749.3667644	87	Grand Index
35	m	13-16 years	0.202	749.5305757	87	Grand Index
35	m	13-16 years	0.203	749.9964473	88	Grand Index
35	m	13-16 years	0.206	750	88	Grand Index
35	m	13-16 years	0.207	751	88	Grand Index
35	m	13-16 years	0.208	751.2712642	88	Grand Index
35	m	13-16 years	0.211	752	88	Grand Index
35	m	13-16 years	0.212	752.068	88	Grand Index
35	m	13-16 years	0.215	753	88	Grand Index
35	m	13-16 years	0.216	753.824	88	Grand Index

Age	Gender	Education	Percentile	Raw Score	Norm Score	Assessment
35	m	13-16 years	0.218	754	88	Grand Index
35	m	13-16 years	0.219	754.3624366	88	Grand Index
35	m	13-16 years	0.22	754.8827346	88	Grand Index
35	m	13-16 years	0.222	755	89	Grand Index
35	m	13-16 years	0.226	756	89	Grand Index
35	m	13-16 years	0.227	756.9267741	89	Grand Index
35	m	13-16 years	0.23	757	89	Grand Index
35	m	13-16 years	0.231	757.659	89	Grand Index
35	m	13-16 years	0.234	758	89	Grand Index
35	m	13-16 years	0.235	758.8972494	89	Grand Index
35	m	13-16 years	0.238	759	89	Grand Index
35	m	13-16 years	0.239	759.171	89	Grand Index
35	m	13-16 years	0.24	760	89	Grand Index
35	m	13-16 years	0.241	760.4569861	89	Grand Index
35	m	13-16 years	0.242	761	90	Grand Index
35	m	13-16 years	0.243	761.927	90	Grand Index
35	m	13-16 years	0.246	762	90	Grand Index
35	m	13-16 years	0.248	763	90	Grand Index
35	m	13-16 years	0.249	763.0112281	90	Grand Index
35	m	13-16 years	0.254	764	90	Grand Index
35	m	13-16 years	0.255	764.195	90	Grand Index
35	m	13-16 years	0.257	765	90	Grand Index
35	m	13-16 years	0.258	765.8989393	90	Grand Index
35	m	13-16 years	0.263	766	90	Grand Index
35	m	13-16 years	0.264	767	91	Grand Index
35	m	13-16 years	0.265	767.085	91	Grand Index
35	m	13-16 years	0.269	768	91	Grand Index
35	m	13-16 years	0.27	768.8961715	91	Grand Index
35	m	13-16 years	0.272	769	91	Grand Index
35	m	13-16 years	0.273	769.4622467	91	Grand Index
35	m	13-16 years	0.274	769.9802637	91	Grand Index
35	m	13-16 years	0.277	770	91	Grand Index
35	m	13-16 years	0.278	770.474191	91	Grand Index
35	m	13-16 years	0.279	770.9965232	91	Grand Index
35	m	13-16 years	0.282	771	91	Grand Index
35	m	13-16 years	0.285	772	91	Grand Index
35	m	13-16 years	0.286	772.0173412	92	Grand Index
35	m	13-16 years	0.288	773	92	Grand Index
35	m	13-16 years	0.289	773.621	92	Grand Index
35	m	13-16 years	0.292	774	92	Grand Index
35	m	13-16 years	0.296	775	92	Grand Index
35	m	13-16 years	0.297	775.0904225	92	Grand Index
35	m	13-16 years	0.298	775.9826317	92	Grand Index
35	m	13-16 years	0.301	776	92	Grand Index
35	m	13-16 years	0.309	777	93	Grand Index
35	m	13-16 years	0.31	777.59	93	Grand Index
35	m	13-16 years	0.314	778	93	Grand Index
35	m	13-16 years	0.318	779	93	Grand Index
35	m	13-16 years	0.319	779.2631352	93	Grand Index
35	m	13-16 years	0.324	780	93	Grand Index
35	m	13-16 years	0.325	780.0529573	93	Grand Index
35	m	13-16 years	0.33	781	93	Grand Index
35	m	13-16 years	0.331	781.3140881	93	Grand Index
35	m	13-16 years	0.332	781.7033595	93	Grand Index
35	m	13-16 years	0.333	781.994138	94	Grand Index
35	m	13-16 years	0.338	782	94	Grand Index

Age	Gender	Education	Percentile	Raw Score	Norm Score	Assessment
35	m	13-16 years	0.339	782.0073249	94	Grand Index
35	m	13-16 years	0.34	782.7226615	94	Grand Index
35	m	13-16 years	0.345	783	94	Grand Index
35	m	13-16 years	0.346	783.394	94	Grand Index
35	m	13-16 years	0.35	784	94	Grand Index
35	m	13-16 years	0.351	784.0120236	94	Grand Index
35	m	13-16 years	0.352	784.8043518	94	Grand Index
35	m	13-16 years	0.355	785	94	Grand Index
35	m	13-16 years	0.356	785.9955301	94	Grand Index
35	m	13-16 years	0.36	786	95	Grand Index
35	m	13-16 years	0.361	786.229	95	Grand Index
35	m	13-16 years	0.364	787	95	Grand Index
35	m	13-16 years	0.365	787.9850415	95	Grand Index
35	m	13-16 years	0.368	788	95	Grand Index
35	m	13-16 years	0.369	788.0373184	95	Grand Index
35	m	13-16 years	0.374	789	95	Grand Index
35	m	13-16 years	0.375	789.2156175	95	Grand Index
35	m	13-16 years	0.38	790	95	Grand Index
35	m	13-16 years	0.381	790.0010201	95	Grand Index
35	m	13-16 years	0.386	791	96	Grand Index
35	m	13-16 years	0.387	791.8259207	96	Grand Index
35	m	13-16 years	0.391	792	96	Grand Index
35	m	13-16 years	0.392	792.4929022	96	Grand Index
35	m	13-16 years	0.394	793	96	Grand Index
35	m	13-16 years	0.395	793.655	96	Grand Index
35	m	13-16 years	0.397	794	96	Grand Index
35	m	13-16 years	0.398	794.222	96	Grand Index
35	m	13-16 years	0.401	795	96	Grand Index
35	m	13-16 years	0.404	796	96	Grand Index
35	m	13-16 years	0.405	796.1141414	96	Grand Index
35	m	13-16 years	0.411	797	97	Grand Index
35	m	13-16 years	0.412	797.8700348	97	Grand Index
35	m	13-16 years	0.415	798	97	Grand Index
35	m	13-16 years	0.416	798.1228898	97	Grand Index
35	m	13-16 years	0.417	798.9921689	97	Grand Index
35	m	13-16 years	0.424	799	97	Grand Index
35	m	13-16 years	0.425	799.0160284	97	Grand Index
35	m	13-16 years	0.433	800	97	Grand Index
35	m	13-16 years	0.434	800.8495041	98	Grand Index
35	m	13-16 years	0.435	801	98	Grand Index
35	m	13-16 years	0.436	801.2468577	98	Grand Index
35	m	13-16 years	0.437	801.9597286	98	Grand Index
35	m	13-16 years	0.443	802	98	Grand Index
35	m	13-16 years	0.444	802.0270867	98	Grand Index
35	m	13-16 years	0.448	803	98	Grand Index
35	m	13-16 years	0.449	803.9741379	98	Grand Index
35	m	13-16 years	0.452	804	98	Grand Index
35	m	13-16 years	0.453	804.5252543	98	Grand Index
35	m	13-16 years	0.458	805	98	Grand Index
35	m	13-16 years	0.459	805.5835095	98	Grand Index
35	m	13-16 years	0.462	806	99	Grand Index
35	m	13-16 years	0.463	806.6168223	99	Grand Index
35	m	13-16 years	0.467	807	99	Grand Index
35	m	13-16 years	0.468	807.452	99	Grand Index
35	m	13-16 years	0.471	808	99	Grand Index
35	m	13-16 years	0.472	808.0366834	99	Grand Index

Age	Gender	Education	Percentile	Raw Score	Norm Score	Assessment
35	m	13-16 years	0.478	809	99	Grand Index
35	m	13-16 years	0.479	809.8468118	99	Grand Index
35	m	13-16 years	0.486	810	99	Grand Index
35	m	13-16 years	0.49	811	100	Grand Index
35	m	13-16 years	0.491	811.6141284	100	Grand Index
35	m	13-16 years	0.492	811.9995787	100	Grand Index
35	m	13-16 years	0.495	812	100	Grand Index
35	m	13-16 years	0.496	812.0463124	100	Grand Index
35	m	13-16 years	0.497	812.5406672	100	Grand Index
35	m	13-16 years	0.501	813	100	Grand Index
35	m	13-16 years	0.502	813.7992703	100	Grand Index
35	m	13-16 years	0.509	814	100	Grand Index
35	m	13-16 years	0.511	815	100	Grand Index
35	m	13-16 years	0.512	815.6399778	100	Grand Index
35	m	13-16 years	0.513	815.9983503	100	Grand Index
35	m	13-16 years	0.516	816	101	Grand Index
35	m	13-16 years	0.525	817	101	Grand Index
35	m	13-16 years	0.526	817.9441034	101	Grand Index
35	m	13-16 years	0.529	818	101	Grand Index
35	m	13-16 years	0.53	818.3684878	101	Grand Index
35	m	13-16 years	0.531	819	101	Grand Index
35	m	13-16 years	0.532	819.9538766	101	Grand Index
35	m	13-16 years	0.533	820	101	Grand Index
35	m	13-16 years	0.534	820.6509107	101	Grand Index
35	m	13-16 years	0.539	821	101	Grand Index
35	m	13-16 years	0.54	821.1821866	102	Grand Index
35	m	13-16 years	0.544	822	102	Grand Index
35	m	13-16 years	0.545	822.0141075	102	Grand Index
35	m	13-16 years	0.546	822.9542678	102	Grand Index
35	m	13-16 years	0.547	822.9961915	102	Grand Index
35	m	13-16 years	0.549	823	102	Grand Index
35	m	13-16 years	0.555	824	102	Grand Index
35	m	13-16 years	0.558	825	102	Grand Index
35	m	13-16 years	0.559	825.9470511	102	Grand Index
35	m	13-16 years	0.563	826	102	Grand Index
35	m	13-16 years	0.564	826.596	102	Grand Index
35	m	13-16 years	0.568	827	103	Grand Index
35	m	13-16 years	0.569	827.1437778	103	Grand Index
35	m	13-16 years	0.575	828	103	Grand Index
35	m	13-16 years	0.58	829	103	Grand Index
35	m	13-16 years	0.581	829.809	103	Grand Index
35	m	13-16 years	0.583	830	103	Grand Index
35	m	13-16 years	0.584	830.376	103	Grand Index
35	m	13-16 years	0.587	831	103	Grand Index
35	m	13-16 years	0.588	831.9120817	103	Grand Index
35	m	13-16 years	0.589	831.9960452	103	Grand Index
35	m	13-16 years	0.591	832	103	Grand Index
35	m	13-16 years	0.592	832.8848786	103	Grand Index
35	m	13-16 years	0.597	833	104	Grand Index
35	m	13-16 years	0.598	833.793955	104	Grand Index
35	m	13-16 years	0.602	834	104	Grand Index
35	m	13-16 years	0.603	834.9609617	104	Grand Index
35	m	13-16 years	0.606	835	104	Grand Index
35	m	13-16 years	0.607	835.9147622	104	Grand Index
35	m	13-16 years	0.613	836	104	Grand Index
35	m	13-16 years	0.616	837	104	Grand Index

Age	Gender	Education	Percentile	Raw Score	Norm Score	Assessment
35	m	13-16 years	0.617	837.3042266	104	Grand Index
35	m	13-16 years	0.618	837.9285019	105	Grand Index
35	m	13-16 years	0.624	838	105	Grand Index
35	m	13-16 years	0.625	838.125	105	Grand Index
35	m	13-16 years	0.631	839	105	Grand Index
35	m	13-16 years	0.632	839.3714374	105	Grand Index
35	m	13-16 years	0.635	840	105	Grand Index
35	m	13-16 years	0.636	840.4398796	105	Grand Index
35	m	13-16 years	0.64	841	105	Grand Index
35	m	13-16 years	0.641	841.0936466	105	Grand Index
35	m	13-16 years	0.642	841.794908	105	Grand Index
35	m	13-16 years	0.647	842	106	Grand Index
35	m	13-16 years	0.648	842.0894133	106	Grand Index
35	m	13-16 years	0.649	842.7246571	106	Grand Index
35	m	13-16 years	0.653	843	106	Grand Index
35	m	13-16 years	0.654	843.5554324	106	Grand Index
35	m	13-16 years	0.655	843.9995236	106	Grand Index
35	m	13-16 years	0.659	844	106	Grand Index
35	m	13-16 years	0.66	844.1815766	106	Grand Index
35	m	13-16 years	0.663	845	106	Grand Index
35	m	13-16 years	0.669	846	107	Grand Index
35	m	13-16 years	0.67	846.403819	107	Grand Index
35	m	13-16 years	0.673	847	107	Grand Index
35	m	13-16 years	0.674	847.1609381	107	Grand Index
35	m	13-16 years	0.677	848	107	Grand Index
35	m	13-16 years	0.678	848.142	107	Grand Index
35	m	13-16 years	0.685	849	107	Grand Index
35	m	13-16 years	0.686	849.654	107	Grand Index
35	m	13-16 years	0.689	850	107	Grand Index
35	m	13-16 years	0.69	850.922188	107	Grand Index
35	m	13-16 years	0.693	851	108	Grand Index
35	m	13-16 years	0.694	851.0601163	108	Grand Index
35	m	13-16 years	0.698	852	108	Grand Index
35	m	13-16 years	0.704	853	108	Grand Index
35	m	13-16 years	0.705	853.135232	108	Grand Index
35	m	13-16 years	0.708	854	108	Grand Index
35	m	13-16 years	0.709	854.000927	108	Grand Index
35	m	13-16 years	0.712	855	108	Grand Index
35	m	13-16 years	0.713	855.8365628	108	Grand Index
35	m	13-16 years	0.714	856	108	Grand Index
35	m	13-16 years	0.72	857	109	Grand Index
35	m	13-16 years	0.722	858	109	Grand Index
35	m	13-16 years	0.723	858.6263484	109	Grand Index
35	m	13-16 years	0.728	859	109	Grand Index
35	m	13-16 years	0.729	859.4146174	109	Grand Index
35	m	13-16 years	0.73	859.992672	109	Grand Index
35	m	13-16 years	0.732	860	109	Grand Index
35	m	13-16 years	0.733	860.6183971	109	Grand Index
35	m	13-16 years	0.739	861	110	Grand Index
35	m	13-16 years	0.742	862	110	Grand Index
35	m	13-16 years	0.743	862.427	110	Grand Index
35	m	13-16 years	0.746	863	110	Grand Index
35	m	13-16 years	0.751	864	110	Grand Index
35	m	13-16 years	0.752	864.428276	110	Grand Index
35	m	13-16 years	0.756	865	110	Grand Index
35	m	13-16 years	0.757	865.4239385	110	Grand Index

Age	Gender	Education	Percentile	Raw Score	Norm Score	Assessment
35	m	13-16 years	0.759	866	111	Grand Index
35	m	13-16 years	0.76	866.3073597	111	Grand Index
35	m	13-16 years	0.763	867	111	Grand Index
35	m	13-16 years	0.764	867.1892589	111	Grand Index
35	m	13-16 years	0.765	867.9027423	111	Grand Index
35	m	13-16 years	0.768	868	111	Grand Index
35	m	13-16 years	0.769	868.458266	111	Grand Index
35	m	13-16 years	0.77	869	111	Grand Index
35	m	13-16 years	0.771	869.6998912	111	Grand Index
35	m	13-16 years	0.773	870	111	Grand Index
35	m	13-16 years	0.778	871	111	Grand Index
35	m	13-16 years	0.781	872	112	Grand Index
35	m	13-16 years	0.782	872.798	112	Grand Index
35	m	13-16 years	0.785	873	112	Grand Index
35	m	13-16 years	0.786	873.554	112	Grand Index
35	m	13-16 years	0.789	874	112	Grand Index
35	m	13-16 years	0.79	874.2976061	112	Grand Index
35	m	13-16 years	0.795	875	112	Grand Index
35	m	13-16 years	0.796	875.0194624	112	Grand Index
35	m	13-16 years	0.797	875.7218292	112	Grand Index
35	m	13-16 years	0.799	876	113	Grand Index
35	m	13-16 years	0.8	876.3485453	113	Grand Index
35	m	13-16 years	0.804	877	113	Grand Index
35	m	13-16 years	0.805	877.0582993	113	Grand Index
35	m	13-16 years	0.806	877.9967041	113	Grand Index
35	m	13-16 years	0.807	878	113	Grand Index
35	m	13-16 years	0.808	878.712	113	Grand Index
35	m	13-16 years	0.811	879	113	Grand Index
35	m	13-16 years	0.812	879.468	113	Grand Index
35	m	13-16 years	0.813	880	113	Grand Index
35	m	13-16 years	0.817	881	114	Grand Index
35	m	13-16 years	0.818	881.9166057	114	Grand Index
35	m	13-16 years	0.821	882	114	Grand Index
35	m	13-16 years	0.822	882.358	114	Grand Index
35	m	13-16 years	0.825	883	114	Grand Index
35	m	13-16 years	0.826	883.2011476	114	Grand Index
35	m	13-16 years	0.827	884	114	Grand Index
35	m	13-16 years	0.828	884.8625685	114	Grand Index
35	m	13-16 years	0.83	885	114	Grand Index
35	m	13-16 years	0.832	886	114	Grand Index
35	m	13-16 years	0.833	886.1583149	114	Grand Index
35	m	13-16 years	0.834	886.778538	115	Grand Index
35	m	13-16 years	0.836	887	115	Grand Index
35	m	13-16 years	0.837	887.0221109	115	Grand Index
35	m	13-16 years	0.838	887.6800027	115	Grand Index
35	m	13-16 years	0.84	888	115	Grand Index
35	m	13-16 years	0.841	888.0590732	115	Grand Index
35	m	13-16 years	0.843	889	115	Grand Index
35	m	13-16 years	0.844	889.516	115	Grand Index
35	m	13-16 years	0.846	890	115	Grand Index
35	m	13-16 years	0.847	890.083	115	Grand Index
35	m	13-16 years	0.85	891	116	Grand Index
35	m	13-16 years	0.851	892	116	Grand Index
35	m	13-16 years	0.852	892.028	116	Grand Index
35	m	13-16 years	0.854	893	116	Grand Index
35	m	13-16 years	0.857	894	116	Grand Index

Age	Gender	Education	Percentile	Raw Score	Norm Score	Assessment
35	m	13-16 years	0.858	894.5732957	116	Grand Index
35	m	13-16 years	0.859	895.9568062	116	Grand Index
35	m	13-16 years	0.862	896	116	Grand Index
35	m	13-16 years	0.863	896.5153296	116	Grand Index
35	m	13-16 years	0.866	897	117	Grand Index
35	m	13-16 years	0.867	897.8804435	117	Grand Index
35	m	13-16 years	0.868	898	117	Grand Index
35	m	13-16 years	0.869	898.2145475	117	Grand Index
35	m	13-16 years	0.87	899	117	Grand Index
35	m	13-16 years	0.871	899.0377477	117	Grand Index
35	m	13-16 years	0.873	900	117	Grand Index
35	m	13-16 years	0.874	901.1705339	117	Grand Index
35	m	13-16 years	0.877	902	117	Grand Index
35	m	13-16 years	0.878	902.1661279	117	Grand Index
35	m	13-16 years	0.879	903	118	Grand Index
35	m	13-16 years	0.88	903.2987054	118	Grand Index
35	m	13-16 years	0.883	904	118	Grand Index
35	m	13-16 years	0.884	904.0088391	118	Grand Index
35	m	13-16 years	0.886	905	118	Grand Index
35	m	13-16 years	0.887	905.9990197	118	Grand Index
35	m	13-16 years	0.889	906	118	Grand Index
35	m	13-16 years	0.891	907	118	Grand Index
35	m	13-16 years	0.892	907.4522249	119	Grand Index
35	m	13-16 years	0.894	908	119	Grand Index
35	m	13-16 years	0.895	908.155	119	Grand Index
35	m	13-16 years	0.896	909.344	119	Grand Index
35	m	13-16 years	0.898	910	119	Grand Index
35	m	13-16 years	0.899	910.8093565	119	Grand Index
35	m	13-16 years	0.9	911	119	Grand Index
35	m	13-16 years	0.901	912	119	Grand Index
35	m	13-16 years	0.902	912.478	119	Grand Index
35	m	13-16 years	0.903	913	119	Grand Index
35	m	13-16 years	0.904	913.3941259	120	Grand Index
35	m	13-16 years	0.907	914	120	Grand Index
35	m	13-16 years	0.908	914.1420222	120	Grand Index
35	m	13-16 years	0.91	915	120	Grand Index
35	m	13-16 years	0.911	915.0210979	120	Grand Index
35	m	13-16 years	0.913	916	120	Grand Index
35	m	13-16 years	0.915	917	121	Grand Index
35	m	13-16 years	0.917	918	121	Grand Index
35	m	13-16 years	0.919	919	121	Grand Index
35	m	13-16 years	0.92	919.0547964	121	Grand Index
35	m	13-16 years	0.921	919.9753624	121	Grand Index
35	m	13-16 years	0.923	920	121	Grand Index
35	m	13-16 years	0.924	920.0165623	121	Grand Index
35	m	13-16 years	0.925	921	122	Grand Index
35	m	13-16 years	0.926	921.0050547	122	Grand Index
35	m	13-16 years	0.927	922	122	Grand Index
35	m	13-16 years	0.928	922.392	122	Grand Index
35	m	13-16 years	0.929	924	122	Grand Index
35	m	13-16 years	0.93	924.5314489	122	Grand Index
35	m	13-16 years	0.931	924.9896659	122	Grand Index
35	m	13-16 years	0.932	925.0995352	122	Grand Index
35	m	13-16 years	0.933	925.9827391	122	Grand Index
35	m	13-16 years	0.934	926.2243395	123	Grand Index
35	m	13-16 years	0.935	926.9672044	123	Grand Index

Age	Gender	Education	Percentile	Raw Score	Norm Score	Assessment
35	m	13-16 years	0.936	927.4670833	123	Grand Index
35	m	13-16 years	0.937	928	123	Grand Index
35	m	13-16 years	0.939	929	123	Grand Index
35	m	13-16 years	0.94	929.9207907	123	Grand Index
35	m	13-16 years	0.941	930	123	Grand Index
35	m	13-16 years	0.942	930.0156759	124	Grand Index
35	m	13-16 years	0.943	930.9053663	124	Grand Index
35	m	13-16 years	0.944	932.3916158	124	Grand Index
35	m	13-16 years	0.945	934.3728847	124	Grand Index
35	m	13-16 years	0.946	934.9952362	124	Grand Index
35	m	13-16 years	0.947	936	124	Grand Index
35	m	13-16 years	0.948	937.8272755	124	Grand Index
35	m	13-16 years	0.949	938	125	Grand Index
35	m	13-16 years	0.95	939	125	Grand Index
35	m	13-16 years	0.951	939.8553834	125	Grand Index
35	m	13-16 years	0.952	940	125	Grand Index
35	m	13-16 years	0.953	940.1116819	125	Grand Index
35	m	13-16 years	0.954	940.6719776	125	Grand Index
35	m	13-16 years	0.955	941	125	Grand Index
35	m	13-16 years	0.956	941.684	126	Grand Index
35	m	13-16 years	0.957	942.588194	126	Grand Index
35	m	13-16 years	0.958	943.062	126	Grand Index
35	m	13-16 years	0.959	944.502	126	Grand Index
35	m	13-16 years	0.96	946.0432508	126	Grand Index
35	m	13-16 years	0.961	946.7508958	126	Grand Index
35	m	13-16 years	0.962	947	127	Grand Index
35	m	13-16 years	0.963	947.3157477	127	Grand Index
35	m	13-16 years	0.964	948	127	Grand Index
35	m	13-16 years	0.965	948.0205439	127	Grand Index
35	m	13-16 years	0.966	949.2514625	127	Grand Index
35	m	13-16 years	0.967	950.763	128	Grand Index
35	m	13-16 years	0.968	951.2547173	128	Grand Index
35	m	13-16 years	0.969	952.9312511	128	Grand Index
35	m	13-16 years	0.97	953.5332782	128	Grand Index
35	m	13-16 years	0.971	955.3048691	128	Grand Index
35	m	13-16 years	0.972	955.9926877	129	Grand Index
35	m	13-16 years	0.973	956.950307	129	Grand Index
35	m	13-16 years	0.974	958.1492694	129	Grand Index
35	m	13-16 years	0.975	959.3226756	129	Grand Index
35	m	13-16 years	0.976	960.464	130	Grand Index
35	m	13-16 years	0.977	964.8056951	130	Grand Index
35	m	13-16 years	0.978	965.1507382	130	Grand Index
35	m	13-16 years	0.979	968.0135593	131	Grand Index
35	m	13-16 years	0.98	969.5050849	131	Grand Index
35	m	13-16 years	0.981	970.7793269	131	Grand Index
35	m	13-16 years	0.982	971.8448554	131	Grand Index
35	m	13-16 years	0.983	973	132	Grand Index
35	m	13-16 years	0.984	976.9305982	132	Grand Index
35	m	13-16 years	0.985	977.165	133	Grand Index
35	m	13-16 years	0.986	981.0858875	133	Grand Index
35	m	13-16 years	0.987	983.828612	133	Grand Index
35	m	13-16 years	0.988	985.8586533	134	Grand Index
35	m	13-16 years	0.989	988.0795393	134	Grand Index
35	m	13-16 years	0.99	989.6615299	135	Grand Index
35	m	13-16 years	0.991	991.1516882	135	Grand Index
35	m	13-16 years	0.992	996.5956003	136	Grand Index

Age	Gender	Education	Percentile	Raw Score	Norm Score	Assessment
35	m	13-16 years	0.993	1001.157772	137	Grand Index
35	m	13-16 years	0.994	1004.598	138	Grand Index
35	m	13-16 years	0.995	1011.276014	139	Grand Index
35	m	13-16 years	0.996	1027.845334	140	Grand Index
35	m	13-16 years	0.997	1034.408911	141	Grand Index
35	m	13-16 years	0.998	1050.756888	143	Grand Index
35	m	13-16 years	0.999	1071.730698	146	Grand Index
35	f	17+ years	0.001	574.634048	54	Grand Index
35	f	17+ years	0.002	602.896	57	Grand Index
35	f	17+ years	0.003	605.9618918	59	Grand Index
35	f	17+ years	0.004	616.856	60	Grand Index
35	f	17+ years	0.005	618.3452792	61	Grand Index
35	f	17+ years	0.006	621.9646917	62	Grand Index
35	f	17+ years	0.007	623	63	Grand Index
35	f	17+ years	0.008	624.8114612	64	Grand Index
35	f	17+ years	0.009	628.928	65	Grand Index
35	f	17+ years	0.01	630.1626026	65	Grand Index
35	f	17+ years	0.011	632.456	66	Grand Index
35	f	17+ years	0.012	633.3386556	66	Grand Index
35	f	17+ years	0.013	634.448	67	Grand Index
35	f	17+ years	0.014	635.4467632	67	Grand Index
35	f	17+ years	0.015	638.2836325	67	Grand Index
35	f	17+ years	0.016	641	68	Grand Index
35	f	17+ years	0.017	643.3390523	68	Grand Index
35	f	17+ years	0.018	647.8168741	69	Grand Index
35	f	17+ years	0.019	650.7030405	69	Grand Index
35	f	17+ years	0.02	653.8312979	69	Grand Index
35	f	17+ years	0.021	655.248	69	Grand Index
35	f	17+ years	0.022	658.824	70	Grand Index
35	f	17+ years	0.023	660.408	70	Grand Index
35	f	17+ years	0.024	664.616	70	Grand Index
35	f	17+ years	0.025	666	71	Grand Index
35	f	17+ years	0.026	667.792	71	Grand Index
35	f	17+ years	0.027	670.392	71	Grand Index
35	f	17+ years	0.028	671	71	Grand Index
35	f	17+ years	0.029	672	72	Grand Index
35	f	17+ years	0.03	672.7476531	72	Grand Index
35	f	17+ years	0.031	674.7560466	72	Grand Index
35	f	17+ years	0.032	676.8329515	72	Grand Index
35	f	17+ years	0.033	677	72	Grand Index
35	f	17+ years	0.034	677.864	73	Grand Index
35	f	17+ years	0.035	678	73	Grand Index
35	f	17+ years	0.036	678.856	73	Grand Index
35	f	17+ years	0.037	679.352	73	Grand Index
35	f	17+ years	0.038	680.2385738	73	Grand Index
35	f	17+ years	0.039	681	74	Grand Index
35	f	17+ years	0.04	683.4589318	74	Grand Index
35	f	17+ years	0.041	684.336	74	Grand Index
35	f	17+ years	0.042	685	74	Grand Index
35	f	17+ years	0.043	685.6974522	74	Grand Index
35	f	17+ years	0.044	685.9740131	74	Grand Index
35	f	17+ years	0.045	686.32	75	Grand Index
35	f	17+ years	0.048	687	75	Grand Index
35	f	17+ years	0.049	687.9077918	75	Grand Index
35	f	17+ years	0.05	689.8	75	Grand Index
35	f	17+ years	0.051	690	75	Grand Index

Age	Gender	Education	Percentile	Raw Score	Norm Score	Assessment
35	f	17+ years	0.052	690.4512578	76	Grand Index
35	f	17+ years	0.053	690.9584578	76	Grand Index
35	f	17+ years	0.055	691	76	Grand Index
35	f	17+ years	0.056	691.776	76	Grand Index
35	f	17+ years	0.057	692.2895329	76	Grand Index
35	f	17+ years	0.058	693	76	Grand Index
35	f	17+ years	0.059	693.264	77	Grand Index
35	f	17+ years	0.06	694	77	Grand Index
35	f	17+ years	0.061	694.6498643	77	Grand Index
35	f	17+ years	0.062	695.990649	77	Grand Index
35	f	17+ years	0.064	696	77	Grand Index
35	f	17+ years	0.066	697	77	Grand Index
35	f	17+ years	0.067	697.1176428	78	Grand Index
35	f	17+ years	0.068	698.140958	78	Grand Index
35	f	17+ years	0.07	699	78	Grand Index
35	f	17+ years	0.071	699.9388673	78	Grand Index
35	f	17+ years	0.072	700.712	78	Grand Index
35	f	17+ years	0.075	701	78	Grand Index
35	f	17+ years	0.076	701.3006829	79	Grand Index
35	f	17+ years	0.077	701.5846331	79	Grand Index
35	f	17+ years	0.078	702.0359338	79	Grand Index
35	f	17+ years	0.08	703	79	Grand Index
35	f	17+ years	0.081	703.0443063	79	Grand Index
35	f	17+ years	0.082	703.7162247	79	Grand Index
35	f	17+ years	0.083	704	79	Grand Index
35	f	17+ years	0.084	704.664	79	Grand Index
35	f	17+ years	0.085	706.0811625	79	Grand Index
35	f	17+ years	0.086	706.8304994	80	Grand Index
35	f	17+ years	0.087	707.0359877	80	Grand Index
35	f	17+ years	0.088	707.73134	80	Grand Index
35	f	17+ years	0.089	708	80	Grand Index
35	f	17+ years	0.09	708.64	80	Grand Index
35	f	17+ years	0.091	709	80	Grand Index
35	f	17+ years	0.092	709.4855024	80	Grand Index
35	f	17+ years	0.093	710	80	Grand Index
35	f	17+ years	0.094	710.6277178	80	Grand Index
35	f	17+ years	0.095	711.1703033	80	Grand Index
35	f	17+ years	0.096	712.616	80	Grand Index
35	f	17+ years	0.097	713	81	Grand Index
35	f	17+ years	0.098	713.001392	81	Grand Index
35	f	17+ years	0.099	714	81	Grand Index
35	f	17+ years	0.1	714.3269942	81	Grand Index
35	f	17+ years	0.101	715	81	Grand Index
35	f	17+ years	0.102	715.3470274	81	Grand Index
35	f	17+ years	0.103	715.8269183	81	Grand Index
35	f	17+ years	0.104	716	81	Grand Index
35	f	17+ years	0.105	717	81	Grand Index
35	f	17+ years	0.106	717.1776138	81	Grand Index
35	f	17+ years	0.108	718	81	Grand Index
35	f	17+ years	0.109	718.2288129	82	Grand Index
35	f	17+ years	0.11	719	82	Grand Index
35	f	17+ years	0.113	720	82	Grand Index
35	f	17+ years	0.114	720.0439379	82	Grand Index
35	f	17+ years	0.115	720.6957861	82	Grand Index
35	f	17+ years	0.116	720.9897849	82	Grand Index
35	f	17+ years	0.117	721	82	Grand Index

Age	Gender	Education	Percentile	Raw Score	Norm Score	Assessment
35	f	17+ years	0.118	721.528	82	Grand Index
35	f	17+ years	0.121	722	82	Grand Index
35	f	17+ years	0.122	722.0898449	83	Grand Index
35	f	17+ years	0.123	723	83	Grand Index
35	f	17+ years	0.124	723.504	83	Grand Index
35	f	17+ years	0.125	724	83	Grand Index
35	f	17+ years	0.126	724.0259988	83	Grand Index
35	f	17+ years	0.127	724.9924193	83	Grand Index
35	f	17+ years	0.129	725	83	Grand Index
35	f	17+ years	0.13	725.4826929	83	Grand Index
35	f	17+ years	0.131	725.999524	83	Grand Index
35	f	17+ years	0.132	726	83	Grand Index
35	f	17+ years	0.133	726.968	83	Grand Index
35	f	17+ years	0.135	727	83	Grand Index
35	f	17+ years	0.136	727.456	84	Grand Index
35	f	17+ years	0.139	728	84	Grand Index
35	f	17+ years	0.14	728.3109434	84	Grand Index
35	f	17+ years	0.141	728.9812281	84	Grand Index
35	f	17+ years	0.143	729	84	Grand Index
35	f	17+ years	0.144	729.6849396	84	Grand Index
35	f	17+ years	0.149	730	84	Grand Index
35	f	17+ years	0.15	730.4331451	84	Grand Index
35	f	17+ years	0.151	730.9459347	85	Grand Index
35	f	17+ years	0.152	731	85	Grand Index
35	f	17+ years	0.153	731.085547	85	Grand Index
35	f	17+ years	0.155	732	85	Grand Index
35	f	17+ years	0.156	732.376	85	Grand Index
35	f	17+ years	0.159	733	85	Grand Index
35	f	17+ years	0.16	733.7890958	85	Grand Index
35	f	17+ years	0.163	734	85	Grand Index
35	f	17+ years	0.164	734.5077041	85	Grand Index
35	f	17+ years	0.166	735	85	Grand Index
35	f	17+ years	0.167	735.2851559	86	Grand Index
35	f	17+ years	0.168	736	86	Grand Index
35	f	17+ years	0.169	736.5583684	86	Grand Index
35	f	17+ years	0.17	736.8879769	86	Grand Index
35	f	17+ years	0.171	737	86	Grand Index
35	f	17+ years	0.175	738	86	Grand Index
35	f	17+ years	0.176	738.100546	86	Grand Index
35	f	17+ years	0.177	738.862654	86	Grand Index
35	f	17+ years	0.182	740	86	Grand Index
35	f	17+ years	0.183	740.768	86	Grand Index
35	f	17+ years	0.187	741	87	Grand Index
35	f	17+ years	0.188	741.248	87	Grand Index
35	f	17+ years	0.189	742.5544093	87	Grand Index
35	f	17+ years	0.191	743	87	Grand Index
35	f	17+ years	0.192	743.8856981	87	Grand Index
35	f	17+ years	0.195	744	87	Grand Index
35	f	17+ years	0.196	744.2121059	87	Grand Index
35	f	17+ years	0.197	744.9792927	87	Grand Index
35	f	17+ years	0.199	745	87	Grand Index
35	f	17+ years	0.2	745.4506752	87	Grand Index
35	f	17+ years	0.201	745.8390791	87	Grand Index
35	f	17+ years	0.203	746	88	Grand Index
35	f	17+ years	0.204	746.0062367	88	Grand Index
35	f	17+ years	0.205	746.6682342	88	Grand Index

Age	Gender	Education	Percentile	Raw Score	Norm Score	Assessment
35	f	17+ years	0.206	747.524107	88	Grand Index
35	f	17+ years	0.209	748	88	Grand Index
35	f	17+ years	0.21	748.1517802	88	Grand Index
35	f	17+ years	0.211	748.9823275	88	Grand Index
35	f	17+ years	0.213	749	88	Grand Index
35	f	17+ years	0.214	749.956253	88	Grand Index
35	f	17+ years	0.215	750	88	Grand Index
35	f	17+ years	0.216	750.0060013	88	Grand Index
35	f	17+ years	0.217	750.6482388	88	Grand Index
35	f	17+ years	0.219	751	88	Grand Index
35	f	17+ years	0.22	751.6790646	88	Grand Index
35	f	17+ years	0.221	751.9543285	88	Grand Index
35	f	17+ years	0.225	752	89	Grand Index
35	f	17+ years	0.226	752.0005226	89	Grand Index
35	f	17+ years	0.227	752.2410984	89	Grand Index
35	f	17+ years	0.228	752.9322344	89	Grand Index
35	f	17+ years	0.233	753	89	Grand Index
35	f	17+ years	0.234	753.064	89	Grand Index
35	f	17+ years	0.237	754	89	Grand Index
35	f	17+ years	0.238	754.2272727	89	Grand Index
35	f	17+ years	0.241	755	89	Grand Index
35	f	17+ years	0.245	756	90	Grand Index
35	f	17+ years	0.247	757	90	Grand Index
35	f	17+ years	0.248	757.6649825	90	Grand Index
35	f	17+ years	0.249	758	90	Grand Index
35	f	17+ years	0.252	759	90	Grand Index
35	f	17+ years	0.253	759.488	90	Grand Index
35	f	17+ years	0.256	760	90	Grand Index
35	f	17+ years	0.257	760.472	90	Grand Index
35	f	17+ years	0.259	761	90	Grand Index
35	f	17+ years	0.26	761.4287135	90	Grand Index
35	f	17+ years	0.262	762	90	Grand Index
35	f	17+ years	0.266	763	91	Grand Index
35	f	17+ years	0.27	764	91	Grand Index
35	f	17+ years	0.274	765	91	Grand Index
35	f	17+ years	0.275	765.4815948	91	Grand Index
35	f	17+ years	0.278	766	91	Grand Index
35	f	17+ years	0.279	766.384	91	Grand Index
35	f	17+ years	0.284	767	91	Grand Index
35	f	17+ years	0.286	768	92	Grand Index
35	f	17+ years	0.287	768.352	92	Grand Index
35	f	17+ years	0.292	769	92	Grand Index
35	f	17+ years	0.293	769.3141242	92	Grand Index
35	f	17+ years	0.294	769.9925545	92	Grand Index
35	f	17+ years	0.296	770	92	Grand Index
35	f	17+ years	0.297	770.1668119	92	Grand Index
35	f	17+ years	0.298	770.9106535	92	Grand Index
35	f	17+ years	0.304	771	92	Grand Index
35	f	17+ years	0.305	771.4024842	92	Grand Index
35	f	17+ years	0.31	772	93	Grand Index
35	f	17+ years	0.311	772.2003066	93	Grand Index
35	f	17+ years	0.312	772.8487969	93	Grand Index
35	f	17+ years	0.314	773	93	Grand Index
35	f	17+ years	0.315	773.9590917	93	Grand Index
35	f	17+ years	0.318	774	93	Grand Index
35	f	17+ years	0.319	774.224	93	Grand Index

Age	Gender	Education	Percentile	Raw Score	Norm Score	Assessment
35	f	17+ years	0.322	775	93	Grand Index
35	f	17+ years	0.328	776	93	Grand Index
35	f	17+ years	0.33	777	93	Grand Index
35	f	17+ years	0.331	777.0019474	93	Grand Index
35	f	17+ years	0.332	777.6385051	93	Grand Index
35	f	17+ years	0.333	777.9936184	94	Grand Index
35	f	17+ years	0.334	778	94	Grand Index
35	f	17+ years	0.336	779	94	Grand Index
35	f	17+ years	0.337	779.9757337	94	Grand Index
35	f	17+ years	0.342	780	94	Grand Index
35	f	17+ years	0.343	780.091499	94	Grand Index
35	f	17+ years	0.344	780.8927784	94	Grand Index
35	f	17+ years	0.345	781.12	94	Grand Index
35	f	17+ years	0.348	782	94	Grand Index
35	f	17+ years	0.349	783.1204258	94	Grand Index
35	f	17+ years	0.35	783.7077432	94	Grand Index
35	f	17+ years	0.356	784	94	Grand Index
35	f	17+ years	0.357	784.7855618	95	Grand Index
35	f	17+ years	0.359	785	95	Grand Index
35	f	17+ years	0.36	785.56	95	Grand Index
35	f	17+ years	0.362	786	95	Grand Index
35	f	17+ years	0.363	786.3326583	95	Grand Index
35	f	17+ years	0.364	786.9333942	95	Grand Index
35	f	17+ years	0.366	787	95	Grand Index
35	f	17+ years	0.367	787.032	95	Grand Index
35	f	17+ years	0.371	788	95	Grand Index
35	f	17+ years	0.372	788.512	95	Grand Index
35	f	17+ years	0.374	789	95	Grand Index
35	f	17+ years	0.375	789.046777	95	Grand Index
35	f	17+ years	0.376	789.74755	95	Grand Index
35	f	17+ years	0.38	790	95	Grand Index
35	f	17+ years	0.381	790.976	95	Grand Index
35	f	17+ years	0.382	791	95	Grand Index
35	f	17+ years	0.383	791.968	96	Grand Index
35	f	17+ years	0.384	792.0051761	96	Grand Index
35	f	17+ years	0.385	792.2756817	96	Grand Index
35	f	17+ years	0.39	793	96	Grand Index
35	f	17+ years	0.391	793.936	96	Grand Index
35	f	17+ years	0.396	794	96	Grand Index
35	f	17+ years	0.397	794.0220926	96	Grand Index
35	f	17+ years	0.4	795	96	Grand Index
35	f	17+ years	0.401	795.896	96	Grand Index
35	f	17+ years	0.403	796	96	Grand Index
35	f	17+ years	0.404	796.1933168	96	Grand Index
35	f	17+ years	0.405	796.9404115	96	Grand Index
35	f	17+ years	0.411	797	97	Grand Index
35	f	17+ years	0.412	797.5265888	97	Grand Index
35	f	17+ years	0.414	798	97	Grand Index
35	f	17+ years	0.415	798.84	97	Grand Index
35	f	17+ years	0.419	799	97	Grand Index
35	f	17+ years	0.42	799.7381431	97	Grand Index
35	f	17+ years	0.425	800	97	Grand Index
35	f	17+ years	0.426	800.9389176	97	Grand Index
35	f	17+ years	0.429	801	97	Grand Index
35	f	17+ years	0.43	801.4115028	97	Grand Index
35	f	17+ years	0.433	802	97	Grand Index

Age	Gender	Education	Percentile	Raw Score	Norm Score	Assessment
35	f	17+ years	0.434	802.264	98	Grand Index
35	f	17+ years	0.44	803	98	Grand Index
35	f	17+ years	0.441	803.0489257	98	Grand Index
35	f	17+ years	0.442	803.8539165	98	Grand Index
35	f	17+ years	0.445	804	98	Grand Index
35	f	17+ years	0.446	804.216	98	Grand Index
35	f	17+ years	0.451	805	98	Grand Index
35	f	17+ years	0.452	805.0520159	98	Grand Index
35	f	17+ years	0.453	805.61945	98	Grand Index
35	f	17+ years	0.454	805.9583831	98	Grand Index
35	f	17+ years	0.458	806	98	Grand Index
35	f	17+ years	0.459	806.664	98	Grand Index
35	f	17+ years	0.461	807	99	Grand Index
35	f	17+ years	0.462	807.0036912	99	Grand Index
35	f	17+ years	0.463	807.071166	99	Grand Index
35	f	17+ years	0.464	807.4023068	99	Grand Index
35	f	17+ years	0.469	808	99	Grand Index
35	f	17+ years	0.47	808.12	99	Grand Index
35	f	17+ years	0.472	809	99	Grand Index
35	f	17+ years	0.473	809.608	99	Grand Index
35	f	17+ years	0.474	810	99	Grand Index
35	f	17+ years	0.475	810.6	99	Grand Index
35	f	17+ years	0.478	811	99	Grand Index
35	f	17+ years	0.479	811.3236369	99	Grand Index
35	f	17+ years	0.483	812	99	Grand Index
35	f	17+ years	0.484	812.064	99	Grand Index
35	f	17+ years	0.489	813	100	Grand Index
35	f	17+ years	0.495	814	100	Grand Index
35	f	17+ years	0.502	815	100	Grand Index
35	f	17+ years	0.503	815.488	100	Grand Index
35	f	17+ years	0.506	816	100	Grand Index
35	f	17+ years	0.507	816.604	100	Grand Index
35	f	17+ years	0.509	817	100	Grand Index
35	f	17+ years	0.51	817.2005063	100	Grand Index
35	f	17+ years	0.514	818	101	Grand Index
35	f	17+ years	0.515	818.8491973	101	Grand Index
35	f	17+ years	0.521	819	101	Grand Index
35	f	17+ years	0.522	819.0299425	101	Grand Index
35	f	17+ years	0.523	819.6173848	101	Grand Index
35	f	17+ years	0.526	820	101	Grand Index
35	f	17+ years	0.527	820.392	101	Grand Index
35	f	17+ years	0.528	821	101	Grand Index
35	f	17+ years	0.529	821.384	101	Grand Index
35	f	17+ years	0.534	822	101	Grand Index
35	f	17+ years	0.535	822.4808392	101	Grand Index
35	f	17+ years	0.54	823	102	Grand Index
35	f	17+ years	0.541	823.4691328	102	Grand Index
35	f	17+ years	0.547	824	102	Grand Index
35	f	17+ years	0.548	824.4131433	102	Grand Index
35	f	17+ years	0.549	824.8425443	102	Grand Index
35	f	17+ years	0.554	825	102	Grand Index
35	f	17+ years	0.555	825.6789809	102	Grand Index
35	f	17+ years	0.558	826	102	Grand Index
35	f	17+ years	0.559	826.9627342	102	Grand Index
35	f	17+ years	0.562	827	102	Grand Index
35	f	17+ years	0.563	827.248	102	Grand Index

Age	Gender	Education	Percentile	Raw Score	Norm Score	Assessment
35	f	17+ years	0.564	828.744	102	Grand Index
35	f	17+ years	0.565	829.0801171	102	Grand Index
35	f	17+ years	0.566	829.8241288	102	Grand Index
35	f	17+ years	0.568	830	103	Grand Index
35	f	17+ years	0.569	830.4580867	103	Grand Index
35	f	17+ years	0.574	831	103	Grand Index
35	f	17+ years	0.575	831.0351464	103	Grand Index
35	f	17+ years	0.576	831.4487448	103	Grand Index
35	f	17+ years	0.581	832	103	Grand Index
35	f	17+ years	0.582	832.672	103	Grand Index
35	f	17+ years	0.585	833	103	Grand Index
35	f	17+ years	0.586	833.656	103	Grand Index
35	f	17+ years	0.589	834	103	Grand Index
35	f	17+ years	0.59	834.64	103	Grand Index
35	f	17+ years	0.592	835	103	Grand Index
35	f	17+ years	0.593	835.558747	104	Grand Index
35	f	17+ years	0.596	836	104	Grand Index
35	f	17+ years	0.597	836.112	104	Grand Index
35	f	17+ years	0.6	837	104	Grand Index
35	f	17+ years	0.604	838	104	Grand Index
35	f	17+ years	0.605	838.0176071	104	Grand Index
35	f	17+ years	0.606	838.6693179	104	Grand Index
35	f	17+ years	0.609	839	104	Grand Index
35	f	17+ years	0.61	839.0257398	104	Grand Index
35	f	17+ years	0.611	839.8822495	104	Grand Index
35	f	17+ years	0.614	840	104	Grand Index
35	f	17+ years	0.62	841	105	Grand Index
35	f	17+ years	0.621	841.5143538	105	Grand Index
35	f	17+ years	0.622	841.9794186	105	Grand Index
35	f	17+ years	0.624	842	105	Grand Index
35	f	17+ years	0.625	842.0150973	105	Grand Index
35	f	17+ years	0.626	842.6283243	105	Grand Index
35	f	17+ years	0.63	843	105	Grand Index
35	f	17+ years	0.631	843.1026597	105	Grand Index
35	f	17+ years	0.638	844	105	Grand Index
35	f	17+ years	0.639	844.0018119	105	Grand Index
35	f	17+ years	0.644	845	106	Grand Index
35	f	17+ years	0.645	845.92	106	Grand Index
35	f	17+ years	0.649	846	106	Grand Index
35	f	17+ years	0.653	847	106	Grand Index
35	f	17+ years	0.654	847.384	106	Grand Index
35	f	17+ years	0.663	848	106	Grand Index
35	f	17+ years	0.669	849	107	Grand Index
35	f	17+ years	0.67	849.3357225	107	Grand Index
35	f	17+ years	0.674	850	107	Grand Index
35	f	17+ years	0.675	850.05632	107	Grand Index
35	f	17+ years	0.676	850.5084182	107	Grand Index
35	f	17+ years	0.677	850.9983005	107	Grand Index
35	f	17+ years	0.679	851	107	Grand Index
35	f	17+ years	0.68	851.2175	107	Grand Index
35	f	17+ years	0.681	851.95	107	Grand Index
35	f	17+ years	0.686	852	107	Grand Index
35	f	17+ years	0.687	852.752	107	Grand Index
35	f	17+ years	0.688	853	107	Grand Index
35	f	17+ years	0.689	853.744	107	Grand Index
35	f	17+ years	0.695	854	108	Grand Index

Age	Gender	Education	Percentile	Raw Score	Norm Score	Assessment
35	f	17+ years	0.698	855	108	Grand Index
35	f	17+ years	0.699	855.4672216	108	Grand Index
35	f	17+ years	0.708	856	108	Grand Index
35	f	17+ years	0.709	856.664	108	Grand Index
35	f	17+ years	0.712	857	108	Grand Index
35	f	17+ years	0.713	857.4357809	108	Grand Index
35	f	17+ years	0.716	858	109	Grand Index
35	f	17+ years	0.717	858.632	109	Grand Index
35	f	17+ years	0.719	859	109	Grand Index
35	f	17+ years	0.72	859.0286728	109	Grand Index
35	f	17+ years	0.721	859.7077531	109	Grand Index
35	f	17+ years	0.727	860	109	Grand Index
35	f	17+ years	0.731	861	109	Grand Index
35	f	17+ years	0.733	862	109	Grand Index
35	f	17+ years	0.736	863	109	Grand Index
35	f	17+ years	0.737	863.032	110	Grand Index
35	f	17+ years	0.738	863.9423214	110	Grand Index
35	f	17+ years	0.741	864	110	Grand Index
35	f	17+ years	0.742	864.032	110	Grand Index
35	f	17+ years	0.744	865	110	Grand Index
35	f	17+ years	0.745	865.0708104	110	Grand Index
35	f	17+ years	0.746	865.7291977	110	Grand Index
35	f	17+ years	0.749	866	110	Grand Index
35	f	17+ years	0.75	866.8461538	110	Grand Index
35	f	17+ years	0.752	867	110	Grand Index
35	f	17+ years	0.753	867.1559802	110	Grand Index
35	f	17+ years	0.754	867.7084543	110	Grand Index
35	f	17+ years	0.756	868	110	Grand Index
35	f	17+ years	0.761	869	111	Grand Index
35	f	17+ years	0.762	869.3154884	111	Grand Index
35	f	17+ years	0.764	870	111	Grand Index
35	f	17+ years	0.765	870.44	111	Grand Index
35	f	17+ years	0.767	871	111	Grand Index
35	f	17+ years	0.768	871.928	111	Grand Index
35	f	17+ years	0.773	872	111	Grand Index
35	f	17+ years	0.774	872.904	111	Grand Index
35	f	17+ years	0.778	873	111	Grand Index
35	f	17+ years	0.782	874	112	Grand Index
35	f	17+ years	0.787	875	112	Grand Index
35	f	17+ years	0.788	875.848	112	Grand Index
35	f	17+ years	0.79	876	112	Grand Index
35	f	17+ years	0.791	876.086635	112	Grand Index
35	f	17+ years	0.792	876.8629448	112	Grand Index
35	f	17+ years	0.796	877	112	Grand Index
35	f	17+ years	0.797	877.3423683	112	Grand Index
35	f	17+ years	0.798	878	113	Grand Index
35	f	17+ years	0.799	878.2982929	113	Grand Index
35	f	17+ years	0.8	878.9962453	113	Grand Index
35	f	17+ years	0.802	879	113	Grand Index
35	f	17+ years	0.803	879.0013056	113	Grand Index
35	f	17+ years	0.804	879.7849792	113	Grand Index
35	f	17+ years	0.807	880	113	Grand Index
35	f	17+ years	0.808	880.768	113	Grand Index
35	f	17+ years	0.812	881	113	Grand Index
35	f	17+ years	0.813	881.248	113	Grand Index
35	f	17+ years	0.815	882	113	Grand Index

Age	Gender	Education	Percentile	Raw Score	Norm Score	Assessment
35	f	17+ years	0.816	882.736	114	Grand Index
35	f	17+ years	0.819	883	114	Grand Index
35	f	17+ years	0.82	883.4395807	114	Grand Index
35	f	17+ years	0.821	883.6890229	114	Grand Index
35	f	17+ years	0.822	883.9690389	114	Grand Index
35	f	17+ years	0.823	884.208	114	Grand Index
35	f	17+ years	0.824	885	114	Grand Index
35	f	17+ years	0.825	886	114	Grand Index
35	f	17+ years	0.826	886.696	114	Grand Index
35	f	17+ years	0.827	887.192	114	Grand Index
35	f	17+ years	0.828	888	114	Grand Index
35	f	17+ years	0.83	889	114	Grand Index
35	f	17+ years	0.831	889.3958584	114	Grand Index
35	f	17+ years	0.832	889.5622499	114	Grand Index
35	f	17+ years	0.833	889.8442685	114	Grand Index
35	f	17+ years	0.834	889.9726721	115	Grand Index
35	f	17+ years	0.836	890	115	Grand Index
35	f	17+ years	0.84	891	115	Grand Index
35	f	17+ years	0.843	892	115	Grand Index
35	f	17+ years	0.844	892.624	115	Grand Index
35	f	17+ years	0.847	893	115	Grand Index
35	f	17+ years	0.848	893.0671753	115	Grand Index
35	f	17+ years	0.85	894	116	Grand Index
35	f	17+ years	0.851	895	116	Grand Index
35	f	17+ years	0.852	895.592	116	Grand Index
35	f	17+ years	0.853	896	116	Grand Index
35	f	17+ years	0.854	896.4546329	116	Grand Index
35	f	17+ years	0.855	896.9413639	116	Grand Index
35	f	17+ years	0.858	897	116	Grand Index
35	f	17+ years	0.859	897.0833863	116	Grand Index
35	f	17+ years	0.86	897.9970003	116	Grand Index
35	f	17+ years	0.864	898	116	Grand Index
35	f	17+ years	0.865	898.7810282	117	Grand Index
35	f	17+ years	0.867	899	117	Grand Index
35	f	17+ years	0.868	899.0057916	117	Grand Index
35	f	17+ years	0.869	899.7862391	117	Grand Index
35	f	17+ years	0.87	900	117	Grand Index
35	f	17+ years	0.871	900.0565139	117	Grand Index
35	f	17+ years	0.872	900.5450612	117	Grand Index
35	f	17+ years	0.873	901	117	Grand Index
35	f	17+ years	0.874	901.504	117	Grand Index
35	f	17+ years	0.876	902	117	Grand Index
35	f	17+ years	0.877	902.6942548	117	Grand Index
35	f	17+ years	0.878	902.9611533	117	Grand Index
35	f	17+ years	0.879	903	118	Grand Index
35	f	17+ years	0.88	903.2401231	118	Grand Index
35	f	17+ years	0.881	903.9880062	118	Grand Index
35	f	17+ years	0.885	904	118	Grand Index
35	f	17+ years	0.888	905	118	Grand Index
35	f	17+ years	0.889	905.944	118	Grand Index
35	f	17+ years	0.89	906	118	Grand Index
35	f	17+ years	0.891	906.936	118	Grand Index
35	f	17+ years	0.892	907	119	Grand Index
35	f	17+ years	0.893	907.774294	119	Grand Index
35	f	17+ years	0.894	908.0177247	119	Grand Index
35	f	17+ years	0.895	908.9233443	119	Grand Index

Age	Gender	Education	Percentile	Raw Score	Norm Score	Assessment
35	f	17+ years	0.896	909	119	Grand Index
35	f	17+ years	0.897	909.3743015	119	Grand Index
35	f	17+ years	0.898	910	119	Grand Index
35	f	17+ years	0.899	910.2297856	119	Grand Index
35	f	17+ years	0.901	911	119	Grand Index
35	f	17+ years	0.902	911.392	119	Grand Index
35	f	17+ years	0.903	912	119	Grand Index
35	f	17+ years	0.907	913	120	Grand Index
35	f	17+ years	0.91	914	120	Grand Index
35	f	17+ years	0.911	914.3889225	120	Grand Index
35	f	17+ years	0.912	914.9740223	120	Grand Index
35	f	17+ years	0.913	915	120	Grand Index
35	f	17+ years	0.914	915.344	120	Grand Index
35	f	17+ years	0.915	916	121	Grand Index
35	f	17+ years	0.916	916.0145158	121	Grand Index
35	f	17+ years	0.917	916.7438379	121	Grand Index
35	f	17+ years	0.918	916.9871514	121	Grand Index
35	f	17+ years	0.919	917	121	Grand Index
35	f	17+ years	0.921	918	121	Grand Index
35	f	17+ years	0.922	918.1096477	121	Grand Index
35	f	17+ years	0.923	918.849411	121	Grand Index
35	f	17+ years	0.925	920	122	Grand Index
35	f	17+ years	0.926	920.8634604	122	Grand Index
35	f	17+ years	0.928	921	122	Grand Index
35	f	17+ years	0.929	921.1606632	122	Grand Index
35	f	17+ years	0.93	922.28	122	Grand Index
35	f	17+ years	0.932	923	122	Grand Index
35	f	17+ years	0.933	923.768	122	Grand Index
35	f	17+ years	0.934	924	123	Grand Index
35	f	17+ years	0.935	924.76	123	Grand Index
35	f	17+ years	0.936	926	123	Grand Index
35	f	17+ years	0.937	926.752	123	Grand Index
35	f	17+ years	0.938	927.2453476	123	Grand Index
35	f	17+ years	0.939	927.997262	123	Grand Index
35	f	17+ years	0.94	928.1732054	123	Grand Index
35	f	17+ years	0.941	928.9265259	123	Grand Index
35	f	17+ years	0.942	929.232	124	Grand Index
35	f	17+ years	0.943	930	124	Grand Index
35	f	17+ years	0.944	932.224	124	Grand Index
35	f	17+ years	0.945	933	124	Grand Index
35	f	17+ years	0.946	933.9594483	124	Grand Index
35	f	17+ years	0.947	934	124	Grand Index
35	f	17+ years	0.948	934.9595144	124	Grand Index
35	f	17+ years	0.949	935.5022383	125	Grand Index
35	f	17+ years	0.95	936	125	Grand Index
35	f	17+ years	0.951	937.1537227	125	Grand Index
35	f	17+ years	0.952	938.192	125	Grand Index
35	f	17+ years	0.953	939.1830885	125	Grand Index
35	f	17+ years	0.954	939.5248409	125	Grand Index
35	f	17+ years	0.955	940	125	Grand Index
35	f	17+ years	0.956	940.176	126	Grand Index
35	f	17+ years	0.957	941	126	Grand Index
35	f	17+ years	0.959	944	126	Grand Index
35	f	17+ years	0.96	944.2589431	126	Grand Index
35	f	17+ years	0.961	946.312	126	Grand Index
35	f	17+ years	0.962	947.5277768	127	Grand Index

Age	Gender	Education	Percentile	Raw Score	Norm Score	Assessment
35	f	17+ years	0.963	949.6467473	127	Grand Index
35	f	17+ years	0.964	950.144	127	Grand Index
35	f	17+ years	0.965	951.64	127	Grand Index
35	f	17+ years	0.966	952.7159887	127	Grand Index
35	f	17+ years	0.968	953	128	Grand Index
35	f	17+ years	0.969	953.1216027	128	Grand Index
35	f	17+ years	0.97	954.5241	128	Grand Index
35	f	17+ years	0.971	955.5976872	128	Grand Index
35	f	17+ years	0.972	956.224	129	Grand Index
35	f	17+ years	0.973	959.0968604	129	Grand Index
35	f	17+ years	0.974	961	129	Grand Index
35	f	17+ years	0.975	961.5002375	129	Grand Index
35	f	17+ years	0.977	962	130	Grand Index
35	f	17+ years	0.978	962.9544555	130	Grand Index
35	f	17+ years	0.979	962.9813226	131	Grand Index
35	f	17+ years	0.98	963.08	131	Grand Index
35	f	17+ years	0.981	965.728	131	Grand Index
35	f	17+ years	0.982	967.144	131	Grand Index
35	f	17+ years	0.983	969	132	Grand Index
35	f	17+ years	0.984	974.4975089	132	Grand Index
35	f	17+ years	0.985	976.9337067	133	Grand Index
35	f	17+ years	0.986	977.9710815	133	Grand Index
35	f	17+ years	0.987	978.1198292	133	Grand Index
35	f	17+ years	0.988	979.6382775	134	Grand Index
35	f	17+ years	0.989	980.7114345	134	Grand Index
35	f	17+ years	0.99	981	135	Grand Index
35	f	17+ years	0.991	982.4293333	135	Grand Index
35	f	17+ years	0.992	984.1181098	136	Grand Index
35	f	17+ years	0.993	988.5208568	137	Grand Index
35	f	17+ years	0.994	990.192	138	Grand Index
35	f	17+ years	0.995	998.1576831	139	Grand Index
35	f	17+ years	0.996	999.0477895	140	Grand Index
35	f	17+ years	0.997	1005.508656	141	Grand Index
35	f	17+ years	0.998	1025.024	143	Grand Index
35	f	17+ years	0.999	1036.56	146	Grand Index
35	m	17+ years	0.001	585.0555878	54	Grand Index
35	m	17+ years	0.002	594.3001685	57	Grand Index
35	m	17+ years	0.003	608.8	59	Grand Index
35	m	17+ years	0.004	615.2457093	60	Grand Index
35	m	17+ years	0.005	621.2911795	61	Grand Index
35	m	17+ years	0.006	625.9019612	62	Grand Index
35	m	17+ years	0.007	628.9234801	63	Grand Index
35	m	17+ years	0.008	633.4621068	64	Grand Index
35	m	17+ years	0.009	635.94	65	Grand Index
35	m	17+ years	0.01	640.4	65	Grand Index
35	m	17+ years	0.011	645.8026074	66	Grand Index
35	m	17+ years	0.012	646	66	Grand Index
35	m	17+ years	0.013	648.6159512	67	Grand Index
35	m	17+ years	0.014	651.24	67	Grand Index
35	m	17+ years	0.015	652.8900707	67	Grand Index
35	m	17+ years	0.016	654.8569139	68	Grand Index
35	m	17+ years	0.017	657.2560983	68	Grand Index
35	m	17+ years	0.018	657.9942682	69	Grand Index
35	m	17+ years	0.019	659	69	Grand Index
35	m	17+ years	0.02	660	69	Grand Index
35	m	17+ years	0.021	660.5121351	69	Grand Index

Age	Gender	Education	Percentile	Raw Score	Norm Score	Assessment
35	m	17+ years	0.022	661.52	70	Grand Index
35	m	17+ years	0.023	662.18	70	Grand Index
35	m	17+ years	0.024	664.3696923	70	Grand Index
35	m	17+ years	0.025	668	71	Grand Index
35	m	17+ years	0.026	669	71	Grand Index
35	m	17+ years	0.027	669.5507463	71	Grand Index
35	m	17+ years	0.028	670.48	71	Grand Index
35	m	17+ years	0.029	671.2752484	72	Grand Index
35	m	17+ years	0.03	673.0728582	72	Grand Index
35	m	17+ years	0.031	674.4529049	72	Grand Index
35	m	17+ years	0.032	676.0585733	72	Grand Index
35	m	17+ years	0.033	676.8454406	72	Grand Index
35	m	17+ years	0.035	677	73	Grand Index
35	m	17+ years	0.036	677.7262753	73	Grand Index
35	m	17+ years	0.037	678.3609247	73	Grand Index
35	m	17+ years	0.038	679.0667927	73	Grand Index
35	m	17+ years	0.039	679.9570762	74	Grand Index
35	m	17+ years	0.04	680.0759571	74	Grand Index
35	m	17+ years	0.042	681	74	Grand Index
35	m	17+ years	0.043	682.0392886	74	Grand Index
35	m	17+ years	0.044	682.9725236	74	Grand Index
35	m	17+ years	0.045	684.4	75	Grand Index
35	m	17+ years	0.046	685	75	Grand Index
35	m	17+ years	0.047	686.0008068	75	Grand Index
35	m	17+ years	0.048	686.6929084	75	Grand Index
35	m	17+ years	0.05	688	75	Grand Index
35	m	17+ years	0.051	690	75	Grand Index
35	m	17+ years	0.052	690.5225684	76	Grand Index
35	m	17+ years	0.053	691	76	Grand Index
35	m	17+ years	0.054	691.5772363	76	Grand Index
35	m	17+ years	0.056	692	76	Grand Index
35	m	17+ years	0.057	693	76	Grand Index
35	m	17+ years	0.058	693.56	76	Grand Index
35	m	17+ years	0.059	695	77	Grand Index
35	m	17+ years	0.06	695.1315131	77	Grand Index
35	m	17+ years	0.061	696.6107006	77	Grand Index
35	m	17+ years	0.062	697.1295054	77	Grand Index
35	m	17+ years	0.063	697.9424	77	Grand Index
35	m	17+ years	0.065	699	77	Grand Index
35	m	17+ years	0.066	699.56	77	Grand Index
35	m	17+ years	0.068	700	78	Grand Index
35	m	17+ years	0.069	701	78	Grand Index
35	m	17+ years	0.07	701.0050117	78	Grand Index
35	m	17+ years	0.071	701.0608157	78	Grand Index
35	m	17+ years	0.072	702	78	Grand Index
35	m	17+ years	0.073	702.0617259	78	Grand Index
35	m	17+ years	0.074	702.8948675	78	Grand Index
35	m	17+ years	0.075	703.3999471	78	Grand Index
35	m	17+ years	0.077	704	79	Grand Index
35	m	17+ years	0.078	704.4788282	79	Grand Index
35	m	17+ years	0.08	705	79	Grand Index
35	m	17+ years	0.081	706	79	Grand Index
35	m	17+ years	0.082	706.12	79	Grand Index
35	m	17+ years	0.083	707	79	Grand Index
35	m	17+ years	0.084	707.1509275	79	Grand Index
35	m	17+ years	0.085	707.9070423	79	Grand Index

Age	Gender	Education	Percentile	Raw Score	Norm Score	Assessment
35	m	17+ years	0.086	708	80	Grand Index
35	m	17+ years	0.087	708.1317647	80	Grand Index
35	m	17+ years	0.088	708.7402142	80	Grand Index
35	m	17+ years	0.092	709	80	Grand Index
35	m	17+ years	0.093	709.0555605	80	Grand Index
35	m	17+ years	0.094	709.2282912	80	Grand Index
35	m	17+ years	0.095	710.4728477	80	Grand Index
35	m	17+ years	0.097	711	81	Grand Index
35	m	17+ years	0.098	711.68	81	Grand Index
35	m	17+ years	0.1	712	81	Grand Index
35	m	17+ years	0.101	712.66	81	Grand Index
35	m	17+ years	0.103	713	81	Grand Index
35	m	17+ years	0.104	713.6865955	81	Grand Index
35	m	17+ years	0.105	714.0470439	81	Grand Index
35	m	17+ years	0.106	714.9662725	81	Grand Index
35	m	17+ years	0.109	715	82	Grand Index
35	m	17+ years	0.11	715.6	82	Grand Index
35	m	17+ years	0.111	716.26	82	Grand Index
35	m	17+ years	0.112	717	82	Grand Index
35	m	17+ years	0.113	718	82	Grand Index
35	m	17+ years	0.114	718.1485149	82	Grand Index
35	m	17+ years	0.115	718.9618812	82	Grand Index
35	m	17+ years	0.117	719	82	Grand Index
35	m	17+ years	0.118	719.88	82	Grand Index
35	m	17+ years	0.119	720.54	82	Grand Index
35	m	17+ years	0.122	722	83	Grand Index
35	m	17+ years	0.123	722.0063043	83	Grand Index
35	m	17+ years	0.124	722.8456038	83	Grand Index
35	m	17+ years	0.127	723	83	Grand Index
35	m	17+ years	0.128	723.9384557	83	Grand Index
35	m	17+ years	0.129	724.14	83	Grand Index
35	m	17+ years	0.131	725	83	Grand Index
35	m	17+ years	0.132	725.12	83	Grand Index
35	m	17+ years	0.134	726	83	Grand Index
35	m	17+ years	0.135	726.816281	83	Grand Index
35	m	17+ years	0.137	727	84	Grand Index
35	m	17+ years	0.139	728	84	Grand Index
35	m	17+ years	0.14	728.4114573	84	Grand Index
35	m	17+ years	0.141	729	84	Grand Index
35	m	17+ years	0.142	729.5804125	84	Grand Index
35	m	17+ years	0.144	730	84	Grand Index
35	m	17+ years	0.145	730.7	84	Grand Index
35	m	17+ years	0.146	731.0113966	84	Grand Index
35	m	17+ years	0.147	731.0478904	84	Grand Index
35	m	17+ years	0.148	731.8416524	84	Grand Index
35	m	17+ years	0.15	732	84	Grand Index
35	m	17+ years	0.151	732.6767793	85	Grand Index
35	m	17+ years	0.153	733	85	Grand Index
35	m	17+ years	0.156	735	85	Grand Index
35	m	17+ years	0.162	736	85	Grand Index
35	m	17+ years	0.163	736.9987815	85	Grand Index
35	m	17+ years	0.165	737	85	Grand Index
35	m	17+ years	0.168	738	86	Grand Index
35	m	17+ years	0.169	739	86	Grand Index
35	m	17+ years	0.17	739.4729996	86	Grand Index
35	m	17+ years	0.175	740	86	Grand Index

Age	Gender	Education	Percentile	Raw Score	Norm Score	Assessment
35	m	17+ years	0.176	740.9108682	86	Grand Index
35	m	17+ years	0.18	741	86	Grand Index
35	m	17+ years	0.181	741.8671592	86	Grand Index
35	m	17+ years	0.183	742	86	Grand Index
35	m	17+ years	0.184	742.1061333	86	Grand Index
35	m	17+ years	0.185	742.8352288	87	Grand Index
35	m	17+ years	0.189	743	87	Grand Index
35	m	17+ years	0.191	744	87	Grand Index
35	m	17+ years	0.192	744.1640651	87	Grand Index
35	m	17+ years	0.195	745	87	Grand Index
35	m	17+ years	0.196	745.0762872	87	Grand Index
35	m	17+ years	0.197	745.8992417	87	Grand Index
35	m	17+ years	0.2	746	87	Grand Index
35	m	17+ years	0.201	746.66	87	Grand Index
35	m	17+ years	0.203	747	88	Grand Index
35	m	17+ years	0.204	747.64	88	Grand Index
35	m	17+ years	0.206	748	88	Grand Index
35	m	17+ years	0.207	748.4864022	88	Grand Index
35	m	17+ years	0.213	749	88	Grand Index
35	m	17+ years	0.216	750	88	Grand Index
35	m	17+ years	0.217	750.7574919	88	Grand Index
35	m	17+ years	0.219	751	88	Grand Index
35	m	17+ years	0.22	751.9701493	88	Grand Index
35	m	17+ years	0.223	752	89	Grand Index
35	m	17+ years	0.224	752.1798489	89	Grand Index
35	m	17+ years	0.225	752.9836601	89	Grand Index
35	m	17+ years	0.228	753	89	Grand Index
35	m	17+ years	0.229	753.14	89	Grand Index
35	m	17+ years	0.231	754	89	Grand Index
35	m	17+ years	0.232	755.12	89	Grand Index
35	m	17+ years	0.236	756	89	Grand Index
35	m	17+ years	0.237	756.8559236	89	Grand Index
35	m	17+ years	0.242	757	90	Grand Index
35	m	17+ years	0.243	757.4535612	90	Grand Index
35	m	17+ years	0.247	758	90	Grand Index
35	m	17+ years	0.248	758.6326582	90	Grand Index
35	m	17+ years	0.253	759	90	Grand Index
35	m	17+ years	0.254	759.64	90	Grand Index
35	m	17+ years	0.256	760	90	Grand Index
35	m	17+ years	0.257	760.935122	90	Grand Index
35	m	17+ years	0.258	761.28	90	Grand Index
35	m	17+ years	0.26	762	90	Grand Index
35	m	17+ years	0.261	762.1893827	90	Grand Index
35	m	17+ years	0.262	762.9782716	90	Grand Index
35	m	17+ years	0.269	763	91	Grand Index
35	m	17+ years	0.27	763.5891892	91	Grand Index
35	m	17+ years	0.273	764	91	Grand Index
35	m	17+ years	0.274	764.2290909	91	Grand Index
35	m	17+ years	0.278	765	91	Grand Index
35	m	17+ years	0.279	765.0759477	91	Grand Index
35	m	17+ years	0.28	765.776174	91	Grand Index
35	m	17+ years	0.284	766	91	Grand Index
35	m	17+ years	0.285	766.1	91	Grand Index
35	m	17+ years	0.289	767	92	Grand Index
35	m	17+ years	0.29	767.4	92	Grand Index
35	m	17+ years	0.292	768	92	Grand Index

Age	Gender	Education	Percentile	Raw Score	Norm Score	Assessment
35	m	17+ years	0.293	768.5328866	92	Grand Index
35	m	17+ years	0.296	769	92	Grand Index
35	m	17+ years	0.3	770	92	Grand Index
35	m	17+ years	0.301	770.66	92	Grand Index
35	m	17+ years	0.303	771	92	Grand Index
35	m	17+ years	0.304	771.7515493	92	Grand Index
35	m	17+ years	0.307	772	92	Grand Index
35	m	17+ years	0.308	772.6539929	92	Grand Index
35	m	17+ years	0.31	773	93	Grand Index
35	m	17+ years	0.311	773.26	93	Grand Index
35	m	17+ years	0.313	774	93	Grand Index
35	m	17+ years	0.314	774.3146195	93	Grand Index
35	m	17+ years	0.315	774.9435028	93	Grand Index
35	m	17+ years	0.319	775	93	Grand Index
35	m	17+ years	0.32	775.0224784	93	Grand Index
35	m	17+ years	0.321	775.4285349	93	Grand Index
35	m	17+ years	0.322	775.9480394	93	Grand Index
35	m	17+ years	0.325	776	93	Grand Index
35	m	17+ years	0.326	776.2185688	93	Grand Index
35	m	17+ years	0.329	777	93	Grand Index
35	m	17+ years	0.33	777.6372881	93	Grand Index
35	m	17+ years	0.331	778	93	Grand Index
35	m	17+ years	0.332	778.0520482	93	Grand Index
35	m	17+ years	0.333	778.8754217	94	Grand Index
35	m	17+ years	0.34	779	94	Grand Index
35	m	17+ years	0.341	779.3571927	94	Grand Index
35	m	17+ years	0.346	780	94	Grand Index
35	m	17+ years	0.347	780.0041459	94	Grand Index
35	m	17+ years	0.348	780.2181681	94	Grand Index
35	m	17+ years	0.349	780.7673405	94	Grand Index
35	m	17+ years	0.356	781	94	Grand Index
35	m	17+ years	0.359	782	95	Grand Index
35	m	17+ years	0.36	782.6	95	Grand Index
35	m	17+ years	0.362	783	95	Grand Index
35	m	17+ years	0.366	784	95	Grand Index
35	m	17+ years	0.371	785	95	Grand Index
35	m	17+ years	0.372	785.1822448	95	Grand Index
35	m	17+ years	0.373	785.532656	95	Grand Index
35	m	17+ years	0.374	785.9895214	95	Grand Index
35	m	17+ years	0.377	786	95	Grand Index
35	m	17+ years	0.378	786.4064173	95	Grand Index
35	m	17+ years	0.381	787	95	Grand Index
35	m	17+ years	0.382	787.12	95	Grand Index
35	m	17+ years	0.387	788	96	Grand Index
35	m	17+ years	0.391	789	96	Grand Index
35	m	17+ years	0.392	789.72	96	Grand Index
35	m	17+ years	0.396	790	96	Grand Index
35	m	17+ years	0.397	790.0599591	96	Grand Index
35	m	17+ years	0.401	791	96	Grand Index
35	m	17+ years	0.402	791.32	96	Grand Index
35	m	17+ years	0.406	792	96	Grand Index
35	m	17+ years	0.407	792.2683227	96	Grand Index
35	m	17+ years	0.41	793	97	Grand Index
35	m	17+ years	0.411	793.039661	97	Grand Index
35	m	17+ years	0.412	793.9322034	97	Grand Index
35	m	17+ years	0.416	794	97	Grand Index

Age	Gender	Education	Percentile	Raw Score	Norm Score	Assessment
35	m	17+ years	0.417	794.1933973	97	Grand Index
35	m	17+ years	0.418	794.9105958	97	Grand Index
35	m	17+ years	0.421	795	97	Grand Index
35	m	17+ years	0.422	795.7229268	97	Grand Index
35	m	17+ years	0.426	796	97	Grand Index
35	m	17+ years	0.427	796.8172437	97	Grand Index
35	m	17+ years	0.433	797	97	Grand Index
35	m	17+ years	0.434	797.44	98	Grand Index
35	m	17+ years	0.437	798	98	Grand Index
35	m	17+ years	0.438	798.0038362	98	Grand Index
35	m	17+ years	0.439	798.4195122	98	Grand Index
35	m	17+ years	0.442	799	98	Grand Index
35	m	17+ years	0.443	799.0600696	98	Grand Index
35	m	17+ years	0.444	800	98	Grand Index
35	m	17+ years	0.445	800.1898095	98	Grand Index
35	m	17+ years	0.451	801	98	Grand Index
35	m	17+ years	0.455	802	98	Grand Index
35	m	17+ years	0.456	802.96	98	Grand Index
35	m	17+ years	0.461	803	99	Grand Index
35	m	17+ years	0.462	803.0195004	99	Grand Index
35	m	17+ years	0.463	803.4818479	99	Grand Index
35	m	17+ years	0.469	804	99	Grand Index
35	m	17+ years	0.47	804.7065393	99	Grand Index
35	m	17+ years	0.471	804.9897775	99	Grand Index
35	m	17+ years	0.476	805	99	Grand Index
35	m	17+ years	0.477	805.82	99	Grand Index
35	m	17+ years	0.479	806	99	Grand Index
35	m	17+ years	0.48	806.0136364	99	Grand Index
35	m	17+ years	0.483	807	99	Grand Index
35	m	17+ years	0.484	807.9711566	99	Grand Index
35	m	17+ years	0.487	808	100	Grand Index
35	m	17+ years	0.488	808.000923	100	Grand Index
35	m	17+ years	0.489	808.7429996	100	Grand Index
35	m	17+ years	0.492	809	100	Grand Index
35	m	17+ years	0.493	809.6411461	100	Grand Index
35	m	17+ years	0.499	810	100	Grand Index
35	m	17+ years	0.504	811	100	Grand Index
35	m	17+ years	0.508	812	100	Grand Index
35	m	17+ years	0.509	812.94	100	Grand Index
35	m	17+ years	0.514	813	101	Grand Index
35	m	17+ years	0.515	813.21	101	Grand Index
35	m	17+ years	0.519	814	101	Grand Index
35	m	17+ years	0.52	814.1194805	101	Grand Index
35	m	17+ years	0.521	814.9436364	101	Grand Index
35	m	17+ years	0.528	815	101	Grand Index
35	m	17+ years	0.529	816	101	Grand Index
35	m	17+ years	0.53	816.27492	101	Grand Index
35	m	17+ years	0.532	817	101	Grand Index
35	m	17+ years	0.533	817.366506	101	Grand Index
35	m	17+ years	0.536	818	101	Grand Index
35	m	17+ years	0.537	818.8304871	101	Grand Index
35	m	17+ years	0.54	819	102	Grand Index
35	m	17+ years	0.541	819.337183	102	Grand Index
35	m	17+ years	0.542	819.909607	102	Grand Index
35	m	17+ years	0.545	820	102	Grand Index
35	m	17+ years	0.546	820.36	102	Grand Index

Age	Gender	Education	Percentile	Raw Score	Norm Score	Assessment
35	m	17+ years	0.55	821	102	Grand Index
35	m	17+ years	0.551	821.2502331	102	Grand Index
35	m	17+ years	0.552	821.6224256	102	Grand Index
35	m	17+ years	0.553	821.9924521	102	Grand Index
35	m	17+ years	0.556	822	102	Grand Index
35	m	17+ years	0.557	822.4863092	102	Grand Index
35	m	17+ years	0.561	823	102	Grand Index
35	m	17+ years	0.562	823.2700089	102	Grand Index
35	m	17+ years	0.563	823.8710236	102	Grand Index
35	m	17+ years	0.565	824	102	Grand Index
35	m	17+ years	0.566	824.5479385	102	Grand Index
35	m	17+ years	0.572	825	103	Grand Index
35	m	17+ years	0.573	825.0050784	103	Grand Index
35	m	17+ years	0.574	825.7197326	103	Grand Index
35	m	17+ years	0.577	826	103	Grand Index
35	m	17+ years	0.578	826.48	103	Grand Index
35	m	17+ years	0.581	827	103	Grand Index
35	m	17+ years	0.582	827.0804825	103	Grand Index
35	m	17+ years	0.583	827.9275513	103	Grand Index
35	m	17+ years	0.588	828	103	Grand Index
35	m	17+ years	0.589	828.1966434	103	Grand Index
35	m	17+ years	0.59	828.6571429	103	Grand Index
35	m	17+ years	0.595	829	104	Grand Index
35	m	17+ years	0.596	829.3912101	104	Grand Index
35	m	17+ years	0.597	829.9874128	104	Grand Index
35	m	17+ years	0.598	830	104	Grand Index
35	m	17+ years	0.599	830.34	104	Grand Index
35	m	17+ years	0.6	831	104	Grand Index
35	m	17+ years	0.601	831.66	104	Grand Index
35	m	17+ years	0.603	832	104	Grand Index
35	m	17+ years	0.604	832.64	104	Grand Index
35	m	17+ years	0.609	833	104	Grand Index
35	m	17+ years	0.61	833.6058701	104	Grand Index
35	m	17+ years	0.613	834	104	Grand Index
35	m	17+ years	0.614	834.4407713	104	Grand Index
35	m	17+ years	0.615	834.9964187	104	Grand Index
35	m	17+ years	0.617	835	104	Grand Index
35	m	17+ years	0.618	835.4935211	105	Grand Index
35	m	17+ years	0.623	836	105	Grand Index
35	m	17+ years	0.624	836.84	105	Grand Index
35	m	17+ years	0.625	837	105	Grand Index
35	m	17+ years	0.626	837.0332947	105	Grand Index
35	m	17+ years	0.627	837.6760386	105	Grand Index
35	m	17+ years	0.631	838	105	Grand Index
35	m	17+ years	0.632	838.1161446	105	Grand Index
35	m	17+ years	0.633	838.9929317	105	Grand Index
35	m	17+ years	0.636	839	105	Grand Index
35	m	17+ years	0.637	839.42	105	Grand Index
35	m	17+ years	0.643	840	105	Grand Index
35	m	17+ years	0.646	841	106	Grand Index
35	m	17+ years	0.651	842	106	Grand Index
35	m	17+ years	0.652	842.32	106	Grand Index
35	m	17+ years	0.656	843	106	Grand Index
35	m	17+ years	0.657	843.3581941	106	Grand Index
35	m	17+ years	0.658	843.5301348	106	Grand Index
35	m	17+ years	0.659	843.978657	106	Grand Index

Age	Gender	Education	Percentile	Raw Score	Norm Score	Assessment
35	m	17+ years	0.662	844	106	Grand Index
35	m	17+ years	0.663	844.6011623	106	Grand Index
35	m	17+ years	0.667	845	106	Grand Index
35	m	17+ years	0.668	845.88	107	Grand Index
35	m	17+ years	0.669	846	107	Grand Index
35	m	17+ years	0.67	846.2	107	Grand Index
35	m	17+ years	0.672	847	107	Grand Index
35	m	17+ years	0.673	847.2269856	107	Grand Index
35	m	17+ years	0.675	848	107	Grand Index
35	m	17+ years	0.676	848.0102326	107	Grand Index
35	m	17+ years	0.677	848.8315116	107	Grand Index
35	m	17+ years	0.683	849	107	Grand Index
35	m	17+ years	0.684	849.1885714	107	Grand Index
35	m	17+ years	0.691	850	107	Grand Index
35	m	17+ years	0.692	850.0334051	108	Grand Index
35	m	17+ years	0.694	851	108	Grand Index
35	m	17+ years	0.695	851.6983901	108	Grand Index
35	m	17+ years	0.7	852	108	Grand Index
35	m	17+ years	0.705	853	108	Grand Index
35	m	17+ years	0.706	853.0270846	108	Grand Index
35	m	17+ years	0.707	853.9854645	108	Grand Index
35	m	17+ years	0.712	855	108	Grand Index
35	m	17+ years	0.713	855.2361499	108	Grand Index
35	m	17+ years	0.714	855.5530476	108	Grand Index
35	m	17+ years	0.716	856	109	Grand Index
35	m	17+ years	0.717	856.0905882	109	Grand Index
35	m	17+ years	0.718	856.9294118	109	Grand Index
35	m	17+ years	0.72	857	109	Grand Index
35	m	17+ years	0.721	857.86	109	Grand Index
35	m	17+ years	0.722	858	109	Grand Index
35	m	17+ years	0.723	858.2493305	109	Grand Index
35	m	17+ years	0.724	858.9593651	109	Grand Index
35	m	17+ years	0.726	859	109	Grand Index
35	m	17+ years	0.727	859.3869663	109	Grand Index
35	m	17+ years	0.728	859.9720805	109	Grand Index
35	m	17+ years	0.73	860	109	Grand Index
35	m	17+ years	0.731	861	109	Grand Index
35	m	17+ years	0.732	861.0734694	109	Grand Index
35	m	17+ years	0.733	861.9146939	109	Grand Index
35	m	17+ years	0.734	862.44	109	Grand Index
35	m	17+ years	0.74	863	110	Grand Index
35	m	17+ years	0.741	863.4736	110	Grand Index
35	m	17+ years	0.746	864	110	Grand Index
35	m	17+ years	0.747	864.0121399	110	Grand Index
35	m	17+ years	0.748	864.8742389	110	Grand Index
35	m	17+ years	0.749	865	110	Grand Index
35	m	17+ years	0.752	866	110	Grand Index
35	m	17+ years	0.753	866.98	110	Grand Index
35	m	17+ years	0.754	867	110	Grand Index
35	m	17+ years	0.755	867.1080569	110	Grand Index
35	m	17+ years	0.756	867.6437592	110	Grand Index
35	m	17+ years	0.757	868	110	Grand Index
35	m	17+ years	0.758	868.28	110	Grand Index
35	m	17+ years	0.759	869	111	Grand Index
35	m	17+ years	0.76	869.6	111	Grand Index
35	m	17+ years	0.765	870	111	Grand Index

Age	Gender	Education	Percentile	Raw Score	Norm Score	Assessment
35	m	17+ years	0.766	870.1774486	111	Grand Index
35	m	17+ years	0.768	871	111	Grand Index
35	m	17+ years	0.769	871.54	111	Grand Index
35	m	17+ years	0.772	872	111	Grand Index
35	m	17+ years	0.773	872.18	111	Grand Index
35	m	17+ years	0.776	873	111	Grand Index
35	m	17+ years	0.777	873.82	111	Grand Index
35	m	17+ years	0.778	874	111	Grand Index
35	m	17+ years	0.779	874.6106468	112	Grand Index
35	m	17+ years	0.783	875	112	Grand Index
35	m	17+ years	0.784	875.4650513	112	Grand Index
35	m	17+ years	0.787	876	112	Grand Index
35	m	17+ years	0.792	877	112	Grand Index
35	m	17+ years	0.794	878	112	Grand Index
35	m	17+ years	0.795	878.7	112	Grand Index
35	m	17+ years	0.798	879	113	Grand Index
35	m	17+ years	0.801	880	113	Grand Index
35	m	17+ years	0.802	880.5854773	113	Grand Index
35	m	17+ years	0.803	880.9945455	113	Grand Index
35	m	17+ years	0.806	881	113	Grand Index
35	m	17+ years	0.809	882	113	Grand Index
35	m	17+ years	0.813	883	113	Grand Index
35	m	17+ years	0.814	883.7295294	113	Grand Index
35	m	17+ years	0.818	884	114	Grand Index
35	m	17+ years	0.819	884.639397	114	Grand Index
35	m	17+ years	0.821	885	114	Grand Index
35	m	17+ years	0.822	885.1039692	114	Grand Index
35	m	17+ years	0.823	885.4591225	114	Grand Index
35	m	17+ years	0.826	886	114	Grand Index
35	m	17+ years	0.827	886.0400967	114	Grand Index
35	m	17+ years	0.832	887	114	Grand Index
35	m	17+ years	0.833	887.78	114	Grand Index
35	m	17+ years	0.834	888	115	Grand Index
35	m	17+ years	0.835	889	115	Grand Index
35	m	17+ years	0.836	889.4243073	115	Grand Index
35	m	17+ years	0.837	889.6910322	115	Grand Index
35	m	17+ years	0.838	890	115	Grand Index
35	m	17+ years	0.839	890.106988	115	Grand Index
35	m	17+ years	0.84	891	115	Grand Index
35	m	17+ years	0.841	891.0954435	115	Grand Index
35	m	17+ years	0.842	891.9454908	115	Grand Index
35	m	17+ years	0.844	892	115	Grand Index
35	m	17+ years	0.845	892.7	115	Grand Index
35	m	17+ years	0.846	893	115	Grand Index
35	m	17+ years	0.847	893.91391	115	Grand Index
35	m	17+ years	0.848	894.68	115	Grand Index
35	m	17+ years	0.853	895	116	Grand Index
35	m	17+ years	0.854	895.64	116	Grand Index
35	m	17+ years	0.855	896	116	Grand Index
35	m	17+ years	0.856	896.96	116	Grand Index
35	m	17+ years	0.859	897	116	Grand Index
35	m	17+ years	0.86	897.3477315	116	Grand Index
35	m	17+ years	0.863	899	116	Grand Index
35	m	17+ years	0.864	899.24	116	Grand Index
35	m	17+ years	0.866	900	117	Grand Index
35	m	17+ years	0.867	900.4618451	117	Grand Index

Age	Gender	Education	Percentile	Raw Score	Norm Score	Assessment
35	m	17+ years	0.868	901.8842211	117	Grand Index
35	m	17+ years	0.869	902	117	Grand Index
35	m	17+ years	0.87	902.2	117	Grand Index
35	m	17+ years	0.871	903	117	Grand Index
35	m	17+ years	0.872	903.2773996	117	Grand Index
35	m	17+ years	0.875	904	117	Grand Index
35	m	17+ years	0.878	905	117	Grand Index
35	m	17+ years	0.879	905.8957576	118	Grand Index
35	m	17+ years	0.881	906	118	Grand Index
35	m	17+ years	0.882	906.399795	118	Grand Index
35	m	17+ years	0.883	907.9907042	118	Grand Index
35	m	17+ years	0.886	908	118	Grand Index
35	m	17+ years	0.887	908.3690909	118	Grand Index
35	m	17+ years	0.888	909.0762463	118	Grand Index
35	m	17+ years	0.889	909.9878006	118	Grand Index
35	m	17+ years	0.89	910	118	Grand Index
35	m	17+ years	0.891	910.0077647	118	Grand Index
35	m	17+ years	0.892	910.7562353	119	Grand Index
35	m	17+ years	0.893	911.38	119	Grand Index
35	m	17+ years	0.895	912	119	Grand Index
35	m	17+ years	0.896	913	119	Grand Index
35	m	17+ years	0.897	913.02	119	Grand Index
35	m	17+ years	0.9	914	119	Grand Index
35	m	17+ years	0.901	914.0947013	119	Grand Index
35	m	17+ years	0.902	915	119	Grand Index
35	m	17+ years	0.903	916.96	119	Grand Index
35	m	17+ years	0.904	917	120	Grand Index
35	m	17+ years	0.905	917.3	120	Grand Index
35	m	17+ years	0.906	918	120	Grand Index
35	m	17+ years	0.907	918.62	120	Grand Index
35	m	17+ years	0.908	919.4333614	120	Grand Index
35	m	17+ years	0.909	919.9932979	120	Grand Index
35	m	17+ years	0.911	920	120	Grand Index
35	m	17+ years	0.912	920.2031248	120	Grand Index
35	m	17+ years	0.915	921	121	Grand Index
35	m	17+ years	0.916	921.6330742	121	Grand Index
35	m	17+ years	0.917	922.22	121	Grand Index
35	m	17+ years	0.918	923	121	Grand Index
35	m	17+ years	0.919	924.1892647	121	Grand Index
35	m	17+ years	0.92	925.2	121	Grand Index
35	m	17+ years	0.921	926	121	Grand Index
35	m	17+ years	0.922	926.7185561	121	Grand Index
35	m	17+ years	0.923	927.18	121	Grand Index
35	m	17+ years	0.924	928	121	Grand Index
35	m	17+ years	0.925	929	122	Grand Index
35	m	17+ years	0.926	929.015971	122	Grand Index
35	m	17+ years	0.927	929.2459304	122	Grand Index
35	m	17+ years	0.928	930.5136587	122	Grand Index
35	m	17+ years	0.929	931.1084041	122	Grand Index
35	m	17+ years	0.93	931.954863	122	Grand Index
35	m	17+ years	0.931	933	122	Grand Index
35	m	17+ years	0.932	933.0105793	122	Grand Index
35	m	17+ years	0.933	933.7993955	122	Grand Index
35	m	17+ years	0.934	934.2533333	123	Grand Index
35	m	17+ years	0.935	935	123	Grand Index
35	m	17+ years	0.936	935.76	123	Grand Index

Age	Gender	Education	Percentile	Raw Score	Norm Score	Assessment
35	m	17+ years	0.937	936.42	123	Grand Index
35	m	17+ years	0.938	937.5460526	123	Grand Index
35	m	17+ years	0.939	938	123	Grand Index
35	m	17+ years	0.94	939	123	Grand Index
35	m	17+ years	0.941	939.0539623	123	Grand Index
35	m	17+ years	0.942	939.9718239	124	Grand Index
35	m	17+ years	0.943	940.38	124	Grand Index
35	m	17+ years	0.944	942	124	Grand Index
35	m	17+ years	0.945	942.7	124	Grand Index
35	m	17+ years	0.946	943	124	Grand Index
35	m	17+ years	0.947	944.02	124	Grand Index
35	m	17+ years	0.948	945	124	Grand Index
35	m	17+ years	0.949	946.0891262	125	Grand Index
35	m	17+ years	0.95	947	125	Grand Index
35	m	17+ years	0.951	949.1512846	125	Grand Index
35	m	17+ years	0.952	950.32	125	Grand Index
35	m	17+ years	0.953	951.98	125	Grand Index
35	m	17+ years	0.954	952.64	125	Grand Index
35	m	17+ years	0.956	954	126	Grand Index
35	m	17+ years	0.957	955.2923399	126	Grand Index
35	m	17+ years	0.958	956.0371126	126	Grand Index
35	m	17+ years	0.959	956.9479527	126	Grand Index
35	m	17+ years	0.96	957	126	Grand Index
35	m	17+ years	0.961	957.9308544	126	Grand Index
35	m	17+ years	0.962	958	127	Grand Index
35	m	17+ years	0.963	959.164415	127	Grand Index
35	m	17+ years	0.964	959.9133557	127	Grand Index
35	m	17+ years	0.965	960.9436597	127	Grand Index
35	m	17+ years	0.966	961.1806452	127	Grand Index
35	m	17+ years	0.968	962	128	Grand Index
35	m	17+ years	0.969	962.7977171	128	Grand Index
35	m	17+ years	0.97	964	128	Grand Index
35	m	17+ years	0.971	964.86	128	Grand Index
35	m	17+ years	0.972	966	129	Grand Index
35	m	17+ years	0.973	966.082478	129	Grand Index
35	m	17+ years	0.974	966.9133138	129	Grand Index
35	m	17+ years	0.975	967.5	129	Grand Index
35	m	17+ years	0.976	969.16	130	Grand Index
35	m	17+ years	0.977	970.82	130	Grand Index
35	m	17+ years	0.978	971.8349085	130	Grand Index
35	m	17+ years	0.979	973.7464095	131	Grand Index
35	m	17+ years	0.98	975.6852207	131	Grand Index
35	m	17+ years	0.981	977.46	131	Grand Index
35	m	17+ years	0.982	978.3284268	131	Grand Index
35	m	17+ years	0.983	979.9972027	132	Grand Index
35	m	17+ years	0.984	980.5347439	132	Grand Index
35	m	17+ years	0.985	982.8991736	133	Grand Index
35	m	17+ years	0.986	986.28	133	Grand Index
35	m	17+ years	0.987	989.0222923	133	Grand Index
35	m	17+ years	0.988	992.0669797	134	Grand Index
35	m	17+ years	0.989	995.9176841	134	Grand Index
35	m	17+ years	0.99	997.7946309	135	Grand Index
35	m	17+ years	0.991	998.064131	135	Grand Index
35	m	17+ years	0.992	1001.899278	136	Grand Index
35	m	17+ years	0.993	1006.122019	137	Grand Index
35	m	17+ years	0.994	1009.972843	138	Grand Index

Age	Gender	Education	Percentile	Raw Score	Norm Score	Assessment
35	m	17+ years	0.995	1016.18427	139	Grand Index
35	m	17+ years	0.996	1023.310923	140	Grand Index
35	m	17+ years	0.997	1025.495428	141	Grand Index
35	m	17+ years	0.998	1030.319184	143	Grand Index
35	m	17+ years	0.999	1056.576125	146	Grand Index
40	f	0-12 years	0.001	604.7092583	54	Grand Index
40	f	0-12 years	0.002	610.433561	57	Grand Index
40	f	0-12 years	0.003	616.1578637	59	Grand Index
40	f	0-12 years	0.004	621.9781391	60	Grand Index
40	f	0-12 years	0.005	627.8930543	61	Grand Index
40	f	0-12 years	0.006	633.8079695	62	Grand Index
40	f	0-12 years	0.007	639.6841586	63	Grand Index
40	f	0-12 years	0.008	640.0612434	64	Grand Index
40	f	0-12 years	0.009	640.4383282	65	Grand Index
40	f	0-12 years	0.01	640.815413	65	Grand Index
40	f	0-12 years	0.011	642.2158969	66	Grand Index
40	f	0-12 years	0.012	644.5977223	66	Grand Index
40	f	0-12 years	0.013	646.9795477	67	Grand Index
40	f	0-12 years	0.014	649.3728806	67	Grand Index
40	f	0-12 years	0.015	652.5774895	67	Grand Index
40	f	0-12 years	0.016	655.7820984	68	Grand Index
40	f	0-12 years	0.017	658.9867072	68	Grand Index
40	f	0-12 years	0.018	660.7501049	69	Grand Index
40	f	0-12 years	0.019	661.1696706	69	Grand Index
40	f	0-12 years	0.02	661.5892364	69	Grand Index
40	f	0-12 years	0.021	662.059325	69	Grand Index
40	f	0-12 years	0.022	664.88715	70	Grand Index
40	f	0-12 years	0.023	667.714975	70	Grand Index
40	f	0-12 years	0.024	670.5428	70	Grand Index
40	f	0-12 years	0.025	671.904375	71	Grand Index
40	f	0-12 years	0.026	671.93655	71	Grand Index
40	f	0-12 years	0.027	671.968725	71	Grand Index
40	f	0-12 years	0.028	672.0402413	71	Grand Index
40	f	0-12 years	0.029	673.4788693	72	Grand Index
40	f	0-12 years	0.03	674.9174972	72	Grand Index
40	f	0-12 years	0.031	676.3561251	72	Grand Index
40	f	0-12 years	0.032	677.1775821	72	Grand Index
40	f	0-12 years	0.033	677.4549542	72	Grand Index
40	f	0-12 years	0.034	677.7323263	73	Grand Index
40	f	0-12 years	0.035	678.03	73	Grand Index
40	f	0-12 years	0.036	678.888	73	Grand Index
40	f	0-12 years	0.037	679.746	73	Grand Index
40	f	0-12 years	0.038	680.604	73	Grand Index
40	f	0-12 years	0.041	681	74	Grand Index
40	f	0-12 years	0.042	681.0675925	74	Grand Index
40	f	0-12 years	0.043	682.6785465	74	Grand Index
40	f	0-12 years	0.044	684.2895005	74	Grand Index
40	f	0-12 years	0.045	685.9004545	75	Grand Index
40	f	0-12 years	0.046	687.120707	75	Grand Index
40	f	0-12 years	0.047	688.015375	75	Grand Index
40	f	0-12 years	0.048	688.910043	75	Grand Index
40	f	0-12 years	0.049	689.7630062	75	Grand Index
40	f	0-12 years	0.05	689.8057062	75	Grand Index
40	f	0-12 years	0.051	689.8484062	75	Grand Index
40	f	0-12 years	0.052	689.8911062	76	Grand Index
40	f	0-12 years	0.053	690.5564025	76	Grand Index

Age	Gender	Education	Percentile	Raw Score	Norm Score	Assessment
40	f	0-12 years	0.054	691.7260805	76	Grand Index
40	f	0-12 years	0.055	692.8957585	76	Grand Index
40	f	0-12 years	0.056	694.01091	76	Grand Index
40	f	0-12 years	0.057	694.2059269	76	Grand Index
40	f	0-12 years	0.058	694.4009437	76	Grand Index
40	f	0-12 years	0.059	694.5959605	77	Grand Index
40	f	0-12 years	0.06	694.7327767	77	Grand Index
40	f	0-12 years	0.061	694.8237599	77	Grand Index
40	f	0-12 years	0.062	694.914743	77	Grand Index
40	f	0-12 years	0.063	695.0539585	77	Grand Index
40	f	0-12 years	0.064	695.9112994	77	Grand Index
40	f	0-12 years	0.065	696.7686402	77	Grand Index
40	f	0-12 years	0.066	697.625981	77	Grand Index
40	f	0-12 years	0.067	698.1600686	78	Grand Index
40	f	0-12 years	0.068	698.4467278	78	Grand Index
40	f	0-12 years	0.069	698.7333869	78	Grand Index
40	f	0-12 years	0.07	699.0673712	78	Grand Index
40	f	0-12 years	0.071	700.03078	78	Grand Index
40	f	0-12 years	0.072	700.9941888	78	Grand Index
40	f	0-12 years	0.073	701.9575975	78	Grand Index
40	f	0-12 years	0.074	702.9641179	78	Grand Index
40	f	0-12 years	0.075	704.0027092	78	Grand Index
40	f	0-12 years	0.076	705.0413004	79	Grand Index
40	f	0-12 years	0.077	706.0226714	79	Grand Index
40	f	0-12 years	0.078	706.3173993	79	Grand Index
40	f	0-12 years	0.079	706.6121272	79	Grand Index
40	f	0-12 years	0.08	706.906855	79	Grand Index
40	f	0-12 years	0.081	707.1699926	79	Grand Index
40	f	0-12 years	0.082	707.4102937	79	Grand Index
40	f	0-12 years	0.083	707.6505949	79	Grand Index
40	f	0-12 years	0.084	707.8736322	79	Grand Index
40	f	0-12 years	0.085	707.9082063	79	Grand Index
40	f	0-12 years	0.086	707.9427803	80	Grand Index
40	f	0-12 years	0.087	707.9773544	80	Grand Index
40	f	0-12 years	0.088	708.6650272	80	Grand Index
40	f	0-12 years	0.089	709.8114241	80	Grand Index
40	f	0-12 years	0.09	710.957821	80	Grand Index
40	f	0-12 years	0.097	712	81	Grand Index
40	f	0-12 years	0.098	712.028	81	Grand Index
40	f	0-12 years	0.099	712.314	81	Grand Index
40	f	0-12 years	0.1	712.6	81	Grand Index
40	f	0-12 years	0.101	712.886	81	Grand Index
40	f	0-12 years	0.102	713.172	81	Grand Index
40	f	0-12 years	0.103	713.458	81	Grand Index
40	f	0-12 years	0.104	713.744	81	Grand Index
40	f	0-12 years	0.105	714.06	81	Grand Index
40	f	0-12 years	0.106	714.632	81	Grand Index
40	f	0-12 years	0.107	715.204	81	Grand Index
40	f	0-12 years	0.108	715.776	81	Grand Index
40	f	0-12 years	0.109	716.174	82	Grand Index
40	f	0-12 years	0.11	716.46	82	Grand Index
40	f	0-12 years	0.111	716.746	82	Grand Index
40	f	0-12 years	0.112	717.064	82	Grand Index
40	f	0-12 years	0.113	717.636	82	Grand Index
40	f	0-12 years	0.114	718.208	82	Grand Index
40	f	0-12 years	0.115	718.78	82	Grand Index

Age	Gender	Education	Percentile	Raw Score	Norm Score	Assessment
40	f	0-12 years	0.118	719	82	Grand Index
40	f	0-12 years	0.119	719.034	82	Grand Index
40	f	0-12 years	0.12	719.32	82	Grand Index
40	f	0-12 years	0.121	719.606	82	Grand Index
40	f	0-12 years	0.122	719.892	83	Grand Index
40	f	0-12 years	0.123	721.0613287	83	Grand Index
40	f	0-12 years	0.124	722.7666096	83	Grand Index
40	f	0-12 years	0.125	724.4718905	83	Grand Index
40	f	0-12 years	0.126	725.9998699	83	Grand Index
40	f	0-12 years	0.127	726.296589	83	Grand Index
40	f	0-12 years	0.128	726.5933081	83	Grand Index
40	f	0-12 years	0.129	726.8900272	83	Grand Index
40	f	0-12 years	0.13	727.18	83	Grand Index
40	f	0-12 years	0.131	727.466	83	Grand Index
40	f	0-12 years	0.132	727.752	83	Grand Index
40	f	0-12 years	0.136	728	84	Grand Index
40	f	0-12 years	0.137	728.182	84	Grand Index
40	f	0-12 years	0.138	728.468	84	Grand Index
40	f	0-12 years	0.139	728.754	84	Grand Index
40	f	0-12 years	0.14	729.04	84	Grand Index
40	f	0-12 years	0.141	729.326	84	Grand Index
40	f	0-12 years	0.142	729.612	84	Grand Index
40	f	0-12 years	0.143	729.898	84	Grand Index
40	f	0-12 years	0.144	730.184	84	Grand Index
40	f	0-12 years	0.145	730.47	84	Grand Index
40	f	0-12 years	0.146	730.756	84	Grand Index
40	f	0-12 years	0.147	731.042	84	Grand Index
40	f	0-12 years	0.148	731.328	84	Grand Index
40	f	0-12 years	0.149	731.614	84	Grand Index
40	f	0-12 years	0.15	731.9	84	Grand Index
40	f	0-12 years	0.153	732	85	Grand Index
40	f	0-12 years	0.154	732.044	85	Grand Index
40	f	0-12 years	0.155	732.33	85	Grand Index
40	f	0-12 years	0.156	732.616	85	Grand Index
40	f	0-12 years	0.157	732.902	85	Grand Index
40	f	0-12 years	0.158	733.188	85	Grand Index
40	f	0-12 years	0.159	733.474	85	Grand Index
40	f	0-12 years	0.16	733.76	85	Grand Index
40	f	0-12 years	0.164	734	85	Grand Index
40	f	0-12 years	0.165	734.19	85	Grand Index
40	f	0-12 years	0.166	734.476	85	Grand Index
40	f	0-12 years	0.167	734.762	86	Grand Index
40	f	0-12 years	0.168	735.144	86	Grand Index
40	f	0-12 years	0.169	736.002	86	Grand Index
40	f	0-12 years	0.17	736.86	86	Grand Index
40	f	0-12 years	0.171	737.718	86	Grand Index
40	f	0-12 years	0.172	738.384	86	Grand Index
40	f	0-12 years	0.173	738.956	86	Grand Index
40	f	0-12 years	0.174	739.528	86	Grand Index
40	f	0-12 years	0.175	740.25	86	Grand Index
40	f	0-12 years	0.176	741.68	86	Grand Index
40	f	0-12 years	0.177	743.11	86	Grand Index
40	f	0-12 years	0.178	744.54	86	Grand Index
40	f	0-12 years	0.179	745.5414813	86	Grand Index
40	f	0-12 years	0.18	746.3397474	86	Grand Index
40	f	0-12 years	0.181	747.1380136	86	Grand Index

Age	Gender	Education	Percentile	Raw Score	Norm Score	Assessment
40	f	0-12 years	0.182	747.8540012	86	Grand Index
40	f	0-12 years	0.183	748.199735	86	Grand Index
40	f	0-12 years	0.184	748.5454688	86	Grand Index
40	f	0-12 years	0.185	748.8912026	87	Grand Index
40	f	0-12 years	0.186	749.196	87	Grand Index
40	f	0-12 years	0.187	749.482	87	Grand Index
40	f	0-12 years	0.188	749.768	87	Grand Index
40	f	0-12 years	0.189	750.054	87	Grand Index
40	f	0-12 years	0.19	750.34	87	Grand Index
40	f	0-12 years	0.191	750.626	87	Grand Index
40	f	0-12 years	0.192	750.912	87	Grand Index
40	f	0-12 years	0.193	751.015368	87	Grand Index
40	f	0-12 years	0.194	751.0375663	87	Grand Index
40	f	0-12 years	0.195	751.0597646	87	Grand Index
40	f	0-12 years	0.196	751.1292698	87	Grand Index
40	f	0-12 years	0.197	751.3930716	87	Grand Index
40	f	0-12 years	0.198	751.6568733	87	Grand Index
40	f	0-12 years	0.199	751.920675	87	Grand Index
40	f	0-12 years	0.2	752.2	87	Grand Index
40	f	0-12 years	0.201	752.486	87	Grand Index
40	f	0-12 years	0.202	752.772	87	Grand Index
40	f	0-12 years	0.203	753.116	88	Grand Index
40	f	0-12 years	0.204	753.688	88	Grand Index
40	f	0-12 years	0.205	754.26	88	Grand Index
40	f	0-12 years	0.206	754.832	88	Grand Index
40	f	0-12 years	0.207	755.0398028	88	Grand Index
40	f	0-12 years	0.208	755.0961572	88	Grand Index
40	f	0-12 years	0.209	755.1525116	88	Grand Index
40	f	0-12 years	0.21	755.2452208	88	Grand Index
40	f	0-12 years	0.211	755.4748664	88	Grand Index
40	f	0-12 years	0.212	755.704512	88	Grand Index
40	f	0-12 years	0.213	755.9341576	88	Grand Index
40	f	0-12 years	0.214	756.1279233	88	Grand Index
40	f	0-12 years	0.215	756.3072667	88	Grand Index
40	f	0-12 years	0.216	756.48661	88	Grand Index
40	f	0-12 years	0.217	756.6501962	88	Grand Index
40	f	0-12 years	0.218	756.7568528	88	Grand Index
40	f	0-12 years	0.219	756.8635094	88	Grand Index
40	f	0-12 years	0.22	756.970166	88	Grand Index
40	f	0-12 years	0.221	757.206	88	Grand Index
40	f	0-12 years	0.222	757.492	89	Grand Index
40	f	0-12 years	0.223	757.778	89	Grand Index
40	f	0-12 years	0.227	758	89	Grand Index
40	f	0-12 years	0.228	758.1437117	89	Grand Index
40	f	0-12 years	0.229	758.3413154	89	Grand Index
40	f	0-12 years	0.23	758.538919	89	Grand Index
40	f	0-12 years	0.231	758.777321	89	Grand Index
40	f	0-12 years	0.232	759.1517173	89	Grand Index
40	f	0-12 years	0.233	759.5261137	89	Grand Index
40	f	0-12 years	0.234	759.9005101	89	Grand Index
40	f	0-12 years	0.237	760	89	Grand Index
40	f	0-12 years	0.238	760.068	89	Grand Index
40	f	0-12 years	0.239	760.354	89	Grand Index
40	f	0-12 years	0.24	760.64	89	Grand Index
40	f	0-12 years	0.241	760.926	89	Grand Index
40	f	0-12 years	0.242	761.212	90	Grand Index

Age	Gender	Education	Percentile	Raw Score	Norm Score	Assessment
40	f	0-12 years	0.243	761.498	90	Grand Index
40	f	0-12 years	0.244	761.784	90	Grand Index
40	f	0-12 years	0.245	762.1338264	90	Grand Index
40	f	0-12 years	0.246	762.6806029	90	Grand Index
40	f	0-12 years	0.247	763.2273793	90	Grand Index
40	f	0-12 years	0.248	763.7741558	90	Grand Index
40	f	0-12 years	0.249	764.1446793	90	Grand Index
40	f	0-12 years	0.25	764.4559029	90	Grand Index
40	f	0-12 years	0.251	764.7671264	90	Grand Index
40	f	0-12 years	0.252	765.216	90	Grand Index
40	f	0-12 years	0.253	766.074	90	Grand Index
40	f	0-12 years	0.254	766.932	90	Grand Index
40	f	0-12 years	0.255	767.79	90	Grand Index
40	f	0-12 years	0.258	768	90	Grand Index
40	f	0-12 years	0.259	768.035951	90	Grand Index
40	f	0-12 years	0.26	768.1748969	90	Grand Index
40	f	0-12 years	0.261	768.3138428	90	Grand Index
40	f	0-12 years	0.262	768.4527887	90	Grand Index
40	f	0-12 years	0.263	768.5979149	90	Grand Index
40	f	0-12 years	0.264	768.7449691	91	Grand Index
40	f	0-12 years	0.265	768.8920232	91	Grand Index
40	f	0-12 years	0.272	769	91	Grand Index
40	f	0-12 years	0.273	769.078	91	Grand Index
40	f	0-12 years	0.274	769.364	91	Grand Index
40	f	0-12 years	0.275	769.65	91	Grand Index
40	f	0-12 years	0.276	769.936	91	Grand Index
40	f	0-12 years	0.277	770.222	91	Grand Index
40	f	0-12 years	0.278	770.508	91	Grand Index
40	f	0-12 years	0.279	770.794	91	Grand Index
40	f	0-12 years	0.28	771.16	91	Grand Index
40	f	0-12 years	0.281	771.732	91	Grand Index
40	f	0-12 years	0.282	772.304	91	Grand Index
40	f	0-12 years	0.283	772.876	91	Grand Index
40	f	0-12 years	0.29	773	92	Grand Index
40	f	0-12 years	0.291	773.1876525	92	Grand Index
40	f	0-12 years	0.292	773.4251242	92	Grand Index
40	f	0-12 years	0.293	773.6625959	92	Grand Index
40	f	0-12 years	0.294	773.9285737	92	Grand Index
40	f	0-12 years	0.295	774.263102	92	Grand Index
40	f	0-12 years	0.296	774.5976303	92	Grand Index
40	f	0-12 years	0.297	774.9321586	92	Grand Index
40	f	0-12 years	0.307	775	92	Grand Index
40	f	0-12 years	0.308	775.1211697	92	Grand Index
40	f	0-12 years	0.309	775.5149713	93	Grand Index
40	f	0-12 years	0.31	775.9087728	93	Grand Index
40	f	0-12 years	0.311	776.3025743	93	Grand Index
40	f	0-12 years	0.312	776.6429229	93	Grand Index
40	f	0-12 years	0.313	776.9708298	93	Grand Index
40	f	0-12 years	0.314	777.2987367	93	Grand Index
40	f	0-12 years	0.315	777.5919969	93	Grand Index
40	f	0-12 years	0.316	777.8098044	93	Grand Index
40	f	0-12 years	0.317	778.0276118	93	Grand Index
40	f	0-12 years	0.318	778.2454192	93	Grand Index
40	f	0-12 years	0.319	778.9203257	93	Grand Index
40	f	0-12 years	0.32	779.6968099	93	Grand Index
40	f	0-12 years	0.321	780.473294	93	Grand Index

Age	Gender	Education	Percentile	Raw Score	Norm Score	Assessment
40	f	0-12 years	0.325	781	93	Grand Index
40	f	0-12 years	0.326	781.236	93	Grand Index
40	f	0-12 years	0.327	781.522	93	Grand Index
40	f	0-12 years	0.328	781.808	93	Grand Index
40	f	0-12 years	0.335	782	94	Grand Index
40	f	0-12 years	0.336	782.096	94	Grand Index
40	f	0-12 years	0.337	782.382	94	Grand Index
40	f	0-12 years	0.338	782.668	94	Grand Index
40	f	0-12 years	0.339	782.954	94	Grand Index
40	f	0-12 years	0.346	783	94	Grand Index
40	f	0-12 years	0.347	783.484	94	Grand Index
40	f	0-12 years	0.348	784.056	94	Grand Index
40	f	0-12 years	0.349	784.628	94	Grand Index
40	f	0-12 years	0.353	785	94	Grand Index
40	f	0-12 years	0.354	785.0921574	94	Grand Index
40	f	0-12 years	0.355	785.2001779	94	Grand Index
40	f	0-12 years	0.356	785.3081984	94	Grand Index
40	f	0-12 years	0.357	785.4411693	95	Grand Index
40	f	0-12 years	0.358	785.6191488	95	Grand Index
40	f	0-12 years	0.359	785.7971283	95	Grand Index
40	f	0-12 years	0.36	785.9751078	95	Grand Index
40	f	0-12 years	0.363	786	95	Grand Index
40	f	0-12 years	0.364	786.104	95	Grand Index
40	f	0-12 years	0.365	786.39	95	Grand Index
40	f	0-12 years	0.366	786.676	95	Grand Index
40	f	0-12 years	0.367	786.962	95	Grand Index
40	f	0-12 years	0.368	787.248	95	Grand Index
40	f	0-12 years	0.369	787.534	95	Grand Index
40	f	0-12 years	0.37	787.82	95	Grand Index
40	f	0-12 years	0.374	788	95	Grand Index
40	f	0-12 years	0.375	788.25	95	Grand Index
40	f	0-12 years	0.376	788.536	95	Grand Index
40	f	0-12 years	0.377	788.822	95	Grand Index
40	f	0-12 years	0.378	789.108	95	Grand Index
40	f	0-12 years	0.379	789.394	95	Grand Index
40	f	0-12 years	0.38	789.68	95	Grand Index
40	f	0-12 years	0.381	789.966	95	Grand Index
40	f	0-12 years	0.382	790.252	95	Grand Index
40	f	0-12 years	0.383	790.538	96	Grand Index
40	f	0-12 years	0.384	790.824	96	Grand Index
40	f	0-12 years	0.388	791	96	Grand Index
40	f	0-12 years	0.389	791.254	96	Grand Index
40	f	0-12 years	0.39	791.54	96	Grand Index
40	f	0-12 years	0.391	791.826	96	Grand Index
40	f	0-12 years	0.392	792.2204116	96	Grand Index
40	f	0-12 years	0.393	792.7832482	96	Grand Index
40	f	0-12 years	0.394	793.3460849	96	Grand Index
40	f	0-12 years	0.395	793.9089215	96	Grand Index
40	f	0-12 years	0.396	794.2321625	96	Grand Index
40	f	0-12 years	0.397	794.5273258	96	Grand Index
40	f	0-12 years	0.398	794.8224892	96	Grand Index
40	f	0-12 years	0.399	795.114	96	Grand Index
40	f	0-12 years	0.4	795.4	96	Grand Index
40	f	0-12 years	0.401	795.686	96	Grand Index
40	f	0-12 years	0.402	795.972	96	Grand Index
40	f	0-12 years	0.405	796	96	Grand Index

Age	Gender	Education	Percentile	Raw Score	Norm Score	Assessment
40	f	0-12 years	0.406	796.116	96	Grand Index
40	f	0-12 years	0.407	796.402	96	Grand Index
40	f	0-12 years	0.408	796.688	97	Grand Index
40	f	0-12 years	0.409	796.974	97	Grand Index
40	f	0-12 years	0.41	797.0649339	97	Grand Index
40	f	0-12 years	0.411	797.1363612	97	Grand Index
40	f	0-12 years	0.412	797.2077885	97	Grand Index
40	f	0-12 years	0.413	797.4562758	97	Grand Index
40	f	0-12 years	0.414	797.9568485	97	Grand Index
40	f	0-12 years	0.415	798.4574212	97	Grand Index
40	f	0-12 years	0.416	798.9579939	97	Grand Index
40	f	0-12 years	0.417	799.262	97	Grand Index
40	f	0-12 years	0.418	799.548	97	Grand Index
40	f	0-12 years	0.419	799.834	97	Grand Index
40	f	0-12 years	0.423	800	97	Grand Index
40	f	0-12 years	0.424	800.0776956	97	Grand Index
40	f	0-12 years	0.425	800.1618658	97	Grand Index
40	f	0-12 years	0.426	800.246036	97	Grand Index
40	f	0-12 years	0.427	800.3803967	97	Grand Index
40	f	0-12 years	0.428	800.5822265	97	Grand Index
40	f	0-12 years	0.429	800.7840562	97	Grand Index
40	f	0-12 years	0.43	800.985886	97	Grand Index
40	f	0-12 years	0.433	801	97	Grand Index
40	f	0-12 years	0.434	801.124	98	Grand Index
40	f	0-12 years	0.435	801.41	98	Grand Index
40	f	0-12 years	0.436	801.696	98	Grand Index
40	f	0-12 years	0.437	801.982	98	Grand Index
40	f	0-12 years	0.438	802.268	98	Grand Index
40	f	0-12 years	0.439	802.554	98	Grand Index
40	f	0-12 years	0.44	802.84	98	Grand Index
40	f	0-12 years	0.441	803.126	98	Grand Index
40	f	0-12 years	0.442	803.412	98	Grand Index
40	f	0-12 years	0.443	803.698	98	Grand Index
40	f	0-12 years	0.444	803.984	98	Grand Index
40	f	0-12 years	0.445	804.27	98	Grand Index
40	f	0-12 years	0.446	804.556	98	Grand Index
40	f	0-12 years	0.447	804.842	98	Grand Index
40	f	0-12 years	0.451	805	98	Grand Index
40	f	0-12 years	0.452	805.272	98	Grand Index
40	f	0-12 years	0.453	805.558	98	Grand Index
40	f	0-12 years	0.454	805.844	98	Grand Index
40	f	0-12 years	0.458	806	98	Grand Index
40	f	0-12 years	0.459	806.274	98	Grand Index
40	f	0-12 years	0.46	806.56	98	Grand Index
40	f	0-12 years	0.461	806.846	99	Grand Index
40	f	0-12 years	0.462	807.264	99	Grand Index
40	f	0-12 years	0.463	807.836	99	Grand Index
40	f	0-12 years	0.464	808.408	99	Grand Index
40	f	0-12 years	0.465	808.98	99	Grand Index
40	f	0-12 years	0.466	809.23018	99	Grand Index
40	f	0-12 years	0.467	809.4686999	99	Grand Index
40	f	0-12 years	0.468	809.7072198	99	Grand Index
40	f	0-12 years	0.469	809.8562315	99	Grand Index
40	f	0-12 years	0.47	809.9037117	99	Grand Index
40	f	0-12 years	0.471	809.9511918	99	Grand Index
40	f	0-12 years	0.472	809.9986719	99	Grand Index

Age	Gender	Education	Percentile	Raw Score	Norm Score	Assessment
40	f	0-12 years	0.475	810	99	Grand Index
40	f	0-12 years	0.476	810.136	99	Grand Index
40	f	0-12 years	0.477	810.422	99	Grand Index
40	f	0-12 years	0.478	810.708	99	Grand Index
40	f	0-12 years	0.479	810.994	99	Grand Index
40	f	0-12 years	0.48	811.28	99	Grand Index
40	f	0-12 years	0.481	811.566	99	Grand Index
40	f	0-12 years	0.482	811.852	99	Grand Index
40	f	0-12 years	0.486	812	99	Grand Index
40	f	0-12 years	0.487	812.0847804	100	Grand Index
40	f	0-12 years	0.488	812.1707633	100	Grand Index
40	f	0-12 years	0.489	812.2567463	100	Grand Index
40	f	0-12 years	0.49	812.3985501	100	Grand Index
40	f	0-12 years	0.491	812.5985672	100	Grand Index
40	f	0-12 years	0.492	812.7985842	100	Grand Index
40	f	0-12 years	0.493	812.9986013	100	Grand Index
40	f	0-12 years	0.494	813.0618485	100	Grand Index
40	f	0-12 years	0.495	813.1241326	100	Grand Index
40	f	0-12 years	0.496	813.1864167	100	Grand Index
40	f	0-12 years	0.497	813.5047138	100	Grand Index
40	f	0-12 years	0.498	814.0826297	100	Grand Index
40	f	0-12 years	0.499	814.6605456	100	Grand Index
40	f	0-12 years	0.5	815.2384615	100	Grand Index
40	f	0-12 years	0.501	816.0282615	100	Grand Index
40	f	0-12 years	0.502	816.8180615	100	Grand Index
40	f	0-12 years	0.503	817.6078615	100	Grand Index
40	f	0-12 years	0.504	818.0812133	100	Grand Index
40	f	0-12 years	0.505	818.2425118	100	Grand Index
40	f	0-12 years	0.506	818.4038104	100	Grand Index
40	f	0-12 years	0.507	818.5668531	100	Grand Index
40	f	0-12 years	0.508	818.9775545	100	Grand Index
40	f	0-12 years	0.509	819.3882559	100	Grand Index
40	f	0-12 years	0.51	819.7989573	100	Grand Index
40	f	0-12 years	0.511	820.1316982	100	Grand Index
40	f	0-12 years	0.512	820.3896823	100	Grand Index
40	f	0-12 years	0.513	820.6476664	100	Grand Index
40	f	0-12 years	0.514	820.9024342	101	Grand Index
40	f	0-12 years	0.515	820.9304501	101	Grand Index
40	f	0-12 years	0.516	820.958466	101	Grand Index
40	f	0-12 years	0.517	820.9864818	101	Grand Index
40	f	0-12 years	0.52	821	101	Grand Index
40	f	0-12 years	0.521	821.012	101	Grand Index
40	f	0-12 years	0.522	821.584	101	Grand Index
40	f	0-12 years	0.523	822.156	101	Grand Index
40	f	0-12 years	0.524	822.728	101	Grand Index
40	f	0-12 years	0.527	823	101	Grand Index
40	f	0-12 years	0.528	823.016	101	Grand Index
40	f	0-12 years	0.529	823.588	101	Grand Index
40	f	0-12 years	0.53	824.16	101	Grand Index
40	f	0-12 years	0.531	824.732	101	Grand Index
40	f	0-12 years	0.538	825	101	Grand Index
40	f	0-12 years	0.539	825.154	101	Grand Index
40	f	0-12 years	0.54	825.44	102	Grand Index
40	f	0-12 years	0.541	825.726	102	Grand Index
40	f	0-12 years	0.542	826.012	102	Grand Index
40	f	0-12 years	0.543	826.298	102	Grand Index

Age	Gender	Education	Percentile	Raw Score	Norm Score	Assessment
40	f	0-12 years	0.544	826.584	102	Grand Index
40	f	0-12 years	0.545	826.87	102	Grand Index
40	f	0-12 years	0.546	827.1182007	102	Grand Index
40	f	0-12 years	0.547	827.334902	102	Grand Index
40	f	0-12 years	0.548	827.5516033	102	Grand Index
40	f	0-12 years	0.549	827.789089	102	Grand Index
40	f	0-12 years	0.55	828.4303878	102	Grand Index
40	f	0-12 years	0.551	829.0716865	102	Grand Index
40	f	0-12 years	0.552	829.7129852	102	Grand Index
40	f	0-12 years	0.559	830	102	Grand Index
40	f	0-12 years	0.56	830.32	102	Grand Index
40	f	0-12 years	0.561	830.892	102	Grand Index
40	f	0-12 years	0.562	831.464	102	Grand Index
40	f	0-12 years	0.563	832.018	102	Grand Index
40	f	0-12 years	0.564	832.304	102	Grand Index
40	f	0-12 years	0.565	832.59	102	Grand Index
40	f	0-12 years	0.566	832.876	102	Grand Index
40	f	0-12 years	0.567	833.162	103	Grand Index
40	f	0-12 years	0.568	833.448	103	Grand Index
40	f	0-12 years	0.569	833.734	103	Grand Index
40	f	0-12 years	0.573	834	103	Grand Index
40	f	0-12 years	0.574	834.164	103	Grand Index
40	f	0-12 years	0.575	834.45	103	Grand Index
40	f	0-12 years	0.576	834.736	103	Grand Index
40	f	0-12 years	0.583	835	103	Grand Index
40	f	0-12 years	0.584	835.0159252	103	Grand Index
40	f	0-12 years	0.585	835.2057009	103	Grand Index
40	f	0-12 years	0.586	835.3954766	103	Grand Index
40	f	0-12 years	0.587	835.5852523	103	Grand Index
40	f	0-12 years	0.588	835.7200748	103	Grand Index
40	f	0-12 years	0.589	835.8162991	103	Grand Index
40	f	0-12 years	0.59	835.9125234	103	Grand Index
40	f	0-12 years	0.591	836.026	103	Grand Index
40	f	0-12 years	0.592	836.312	103	Grand Index
40	f	0-12 years	0.593	836.598	104	Grand Index
40	f	0-12 years	0.594	836.884	104	Grand Index
40	f	0-12 years	0.595	837.17	104	Grand Index
40	f	0-12 years	0.596	837.456	104	Grand Index
40	f	0-12 years	0.597	837.742	104	Grand Index
40	f	0-12 years	0.598	838.0275988	104	Grand Index
40	f	0-12 years	0.599	838.3095012	104	Grand Index
40	f	0-12 years	0.6	838.5914036	104	Grand Index
40	f	0-12 years	0.601	838.873306	104	Grand Index
40	f	0-12 years	0.602	838.988137	104	Grand Index
40	f	0-12 years	0.603	838.9922346	104	Grand Index
40	f	0-12 years	0.604	838.9963322	104	Grand Index
40	f	0-12 years	0.611	839	104	Grand Index
40	f	0-12 years	0.612	839.064	104	Grand Index
40	f	0-12 years	0.613	839.636	104	Grand Index
40	f	0-12 years	0.614	840.208	104	Grand Index
40	f	0-12 years	0.615	840.78	104	Grand Index
40	f	0-12 years	0.618	841	105	Grand Index
40	f	0-12 years	0.619	841.034	105	Grand Index
40	f	0-12 years	0.62	841.32	105	Grand Index
40	f	0-12 years	0.621	841.606	105	Grand Index
40	f	0-12 years	0.622	841.892	105	Grand Index

Age	Gender	Education	Percentile	Raw Score	Norm Score	Assessment
40	f	0-12 years	0.623	842.178	105	Grand Index
40	f	0-12 years	0.624	842.464	105	Grand Index
40	f	0-12 years	0.625	842.75	105	Grand Index
40	f	0-12 years	0.626	843.036	105	Grand Index
40	f	0-12 years	0.627	843.322	105	Grand Index
40	f	0-12 years	0.628	843.608	105	Grand Index
40	f	0-12 years	0.629	843.894	105	Grand Index
40	f	0-12 years	0.636	844	105	Grand Index
40	f	0-12 years	0.637	844.182	105	Grand Index
40	f	0-12 years	0.638	844.468	105	Grand Index
40	f	0-12 years	0.639	844.754	105	Grand Index
40	f	0-12 years	0.64	845.04	105	Grand Index
40	f	0-12 years	0.641	845.326	105	Grand Index
40	f	0-12 years	0.642	845.612	105	Grand Index
40	f	0-12 years	0.643	845.898	105	Grand Index
40	f	0-12 years	0.65	846	106	Grand Index
40	f	0-12 years	0.651	846.372	106	Grand Index
40	f	0-12 years	0.652	846.944	106	Grand Index
40	f	0-12 years	0.653	847.516	106	Grand Index
40	f	0-12 years	0.654	848.0418418	106	Grand Index
40	f	0-12 years	0.655	848.3138136	106	Grand Index
40	f	0-12 years	0.656	848.5857854	106	Grand Index
40	f	0-12 years	0.657	848.8577572	106	Grand Index
40	f	0-12 years	0.658	848.9601717	106	Grand Index
40	f	0-12 years	0.659	848.9741999	106	Grand Index
40	f	0-12 years	0.66	848.9882281	106	Grand Index
40	f	0-12 years	0.671	849	107	Grand Index
40	f	0-12 years	0.672	849.384	107	Grand Index
40	f	0-12 years	0.673	849.956	107	Grand Index
40	f	0-12 years	0.674	850.528	107	Grand Index
40	f	0-12 years	0.681	851	107	Grand Index
40	f	0-12 years	0.682	851.052	107	Grand Index
40	f	0-12 years	0.683	851.338	107	Grand Index
40	f	0-12 years	0.684	851.624	107	Grand Index
40	f	0-12 years	0.685	851.91	107	Grand Index
40	f	0-12 years	0.692	852	108	Grand Index
40	f	0-12 years	0.693	852.198	108	Grand Index
40	f	0-12 years	0.694	852.484	108	Grand Index
40	f	0-12 years	0.695	852.77	108	Grand Index
40	f	0-12 years	0.696	853.056	108	Grand Index
40	f	0-12 years	0.697	853.342	108	Grand Index
40	f	0-12 years	0.698	853.628	108	Grand Index
40	f	0-12 years	0.699	853.914	108	Grand Index
40	f	0-12 years	0.7	854.2	108	Grand Index
40	f	0-12 years	0.701	854.486	108	Grand Index
40	f	0-12 years	0.702	854.772	108	Grand Index
40	f	0-12 years	0.703	855.058	108	Grand Index
40	f	0-12 years	0.704	855.344	108	Grand Index
40	f	0-12 years	0.705	855.63	108	Grand Index
40	f	0-12 years	0.706	855.916	108	Grand Index
40	f	0-12 years	0.707	856.404	108	Grand Index
40	f	0-12 years	0.708	856.976	108	Grand Index
40	f	0-12 years	0.709	857.548	108	Grand Index
40	f	0-12 years	0.716	858	109	Grand Index
40	f	0-12 years	0.717	858.0599455	109	Grand Index
40	f	0-12 years	0.718	858.3364682	109	Grand Index

Age	Gender	Education	Percentile	Raw Score	Norm Score	Assessment
40	f	0-12 years	0.719	858.6129909	109	Grand Index
40	f	0-12 years	0.72	858.8895136	109	Grand Index
40	f	0-12 years	0.721	858.9736889	109	Grand Index
40	f	0-12 years	0.722	858.9831662	109	Grand Index
40	f	0-12 years	0.723	858.9926435	109	Grand Index
40	f	0-12 years	0.724	859.064	109	Grand Index
40	f	0-12 years	0.725	859.35	109	Grand Index
40	f	0-12 years	0.726	859.636	109	Grand Index
40	f	0-12 years	0.727	859.922	109	Grand Index
40	f	0-12 years	0.728	860.0767735	109	Grand Index
40	f	0-12 years	0.729	860.182337	109	Grand Index
40	f	0-12 years	0.73	860.2879006	109	Grand Index
40	f	0-12 years	0.731	860.4107425	109	Grand Index
40	f	0-12 years	0.732	860.591179	109	Grand Index
40	f	0-12 years	0.733	860.7716154	109	Grand Index
40	f	0-12 years	0.734	860.9520519	109	Grand Index
40	f	0-12 years	0.741	861	110	Grand Index
40	f	0-12 years	0.742	861.424	110	Grand Index
40	f	0-12 years	0.743	861.996	110	Grand Index
40	f	0-12 years	0.744	862.568	110	Grand Index
40	f	0-12 years	0.745	863.07	110	Grand Index
40	f	0-12 years	0.746	863.356	110	Grand Index
40	f	0-12 years	0.747	863.642	110	Grand Index
40	f	0-12 years	0.748	863.928	110	Grand Index
40	f	0-12 years	0.749	864.214	110	Grand Index
40	f	0-12 years	0.75	864.5	110	Grand Index
40	f	0-12 years	0.751	864.786	110	Grand Index
40	f	0-12 years	0.755	865	110	Grand Index
40	f	0-12 years	0.756	865.0015105	110	Grand Index
40	f	0-12 years	0.757	865.0035105	110	Grand Index
40	f	0-12 years	0.758	865.0055105	110	Grand Index
40	f	0-12 years	0.759	865.0804755	111	Grand Index
40	f	0-12 years	0.76	865.3644755	111	Grand Index
40	f	0-12 years	0.761	865.6484755	111	Grand Index
40	f	0-12 years	0.762	865.9324755	111	Grand Index
40	f	0-12 years	0.763	866.218	111	Grand Index
40	f	0-12 years	0.764	866.504	111	Grand Index
40	f	0-12 years	0.765	866.79	111	Grand Index
40	f	0-12 years	0.769	867	111	Grand Index
40	f	0-12 years	0.77	867.44	111	Grand Index
40	f	0-12 years	0.771	868.012	111	Grand Index
40	f	0-12 years	0.772	868.584	111	Grand Index
40	f	0-12 years	0.776	869	111	Grand Index
40	f	0-12 years	0.777	869.666	111	Grand Index
40	f	0-12 years	0.778	870.524	111	Grand Index
40	f	0-12 years	0.779	871.382	112	Grand Index
40	f	0-12 years	0.78	872.16	112	Grand Index
40	f	0-12 years	0.781	872.732	112	Grand Index
40	f	0-12 years	0.782	873.304	112	Grand Index
40	f	0-12 years	0.783	873.876	112	Grand Index
40	f	0-12 years	0.786	874	112	Grand Index
40	f	0-12 years	0.787	874.0176585	112	Grand Index
40	f	0-12 years	0.788	874.0792481	112	Grand Index
40	f	0-12 years	0.789	874.1408376	112	Grand Index
40	f	0-12 years	0.79	874.2024272	112	Grand Index
40	f	0-12 years	0.791	874.3926794	112	Grand Index

Age	Gender	Education	Percentile	Raw Score	Norm Score	Assessment
40	f	0-12 years	0.792	874.6170898	112	Grand Index
40	f	0-12 years	0.793	874.8415003	112	Grand Index
40	f	0-12 years	0.794	875.084	112	Grand Index
40	f	0-12 years	0.795	875.37	112	Grand Index
40	f	0-12 years	0.796	875.656	112	Grand Index
40	f	0-12 years	0.797	875.942	112	Grand Index
40	f	0-12 years	0.8	876	113	Grand Index
40	f	0-12 years	0.801	876.086	113	Grand Index
40	f	0-12 years	0.802	876.372	113	Grand Index
40	f	0-12 years	0.803	876.658	113	Grand Index
40	f	0-12 years	0.804	876.944	113	Grand Index
40	f	0-12 years	0.807	877	113	Grand Index
40	f	0-12 years	0.808	877.088	113	Grand Index
40	f	0-12 years	0.809	877.374	113	Grand Index
40	f	0-12 years	0.81	877.66	113	Grand Index
40	f	0-12 years	0.811	877.946	113	Grand Index
40	f	0-12 years	0.814	878	113	Grand Index
40	f	0-12 years	0.815	878.09	113	Grand Index
40	f	0-12 years	0.816	878.376	114	Grand Index
40	f	0-12 years	0.817	878.662	114	Grand Index
40	f	0-12 years	0.818	878.948	114	Grand Index
40	f	0-12 years	0.825	879	114	Grand Index
40	f	0-12 years	0.826	879.236	114	Grand Index
40	f	0-12 years	0.827	879.522	114	Grand Index
40	f	0-12 years	0.828	879.808	114	Grand Index
40	f	0-12 years	0.832	880	114	Grand Index
40	f	0-12 years	0.833	880.0640521	114	Grand Index
40	f	0-12 years	0.834	880.1410222	115	Grand Index
40	f	0-12 years	0.835	880.2179924	115	Grand Index
40	f	0-12 years	0.836	880.4352903	115	Grand Index
40	f	0-12 years	0.837	880.9303201	115	Grand Index
40	f	0-12 years	0.838	881.42535	115	Grand Index
40	f	0-12 years	0.839	881.9203798	115	Grand Index
40	f	0-12 years	0.846	882	115	Grand Index
40	f	0-12 years	0.847	882.6092308	115	Grand Index
40	f	0-12 years	0.848	883.3292308	115	Grand Index
40	f	0-12 years	0.849	884.0492308	115	Grand Index
40	f	0-12 years	0.85	884.7657343	116	Grand Index
40	f	0-12 years	0.851	885.4757343	116	Grand Index
40	f	0-12 years	0.852	886.1857343	116	Grand Index
40	f	0-12 years	0.853	886.8957343	116	Grand Index
40	f	0-12 years	0.854	887.0107869	116	Grand Index
40	f	0-12 years	0.855	887.0234306	116	Grand Index
40	f	0-12 years	0.856	887.0360743	116	Grand Index
40	f	0-12 years	0.857	887.1416994	116	Grand Index
40	f	0-12 years	0.858	887.4150557	116	Grand Index
40	f	0-12 years	0.859	887.688412	116	Grand Index
40	f	0-12 years	0.86	887.9617683	116	Grand Index
40	f	0-12 years	0.863	888	116	Grand Index
40	f	0-12 years	0.864	888.104	116	Grand Index
40	f	0-12 years	0.865	888.39	117	Grand Index
40	f	0-12 years	0.866	888.676	117	Grand Index
40	f	0-12 years	0.867	888.962	117	Grand Index
40	f	0-12 years	0.868	890.736	117	Grand Index
40	f	0-12 years	0.869	892.738	117	Grand Index
40	f	0-12 years	0.87	894.74	117	Grand Index

Age	Gender	Education	Percentile	Raw Score	Norm Score	Assessment
40	f	0-12 years	0.871	896.106	117	Grand Index
40	f	0-12 years	0.872	896.392	117	Grand Index
40	f	0-12 years	0.873	896.678	117	Grand Index
40	f	0-12 years	0.874	896.964	117	Grand Index
40	f	0-12 years	0.875	897.250216	117	Grand Index
40	f	0-12 years	0.876	897.5364631	117	Grand Index
40	f	0-12 years	0.877	897.8227102	117	Grand Index
40	f	0-12 years	0.878	898.1087707	117	Grand Index
40	f	0-12 years	0.879	898.3945236	118	Grand Index
40	f	0-12 years	0.88	898.6802765	118	Grand Index
40	f	0-12 years	0.881	898.9660294	118	Grand Index
40	f	0-12 years	0.882	899.252	118	Grand Index
40	f	0-12 years	0.883	899.538	118	Grand Index
40	f	0-12 years	0.884	899.824	118	Grand Index
40	f	0-12 years	0.885	900.22	118	Grand Index
40	f	0-12 years	0.886	900.792	118	Grand Index
40	f	0-12 years	0.887	901.364	118	Grand Index
40	f	0-12 years	0.888	901.936	118	Grand Index
40	f	0-12 years	0.891	902	118	Grand Index
40	f	0-12 years	0.892	902.448	119	Grand Index
40	f	0-12 years	0.893	903.592	119	Grand Index
40	f	0-12 years	0.894	904.736	119	Grand Index
40	f	0-12 years	0.895	905.88	119	Grand Index
40	f	0-12 years	0.898	906	119	Grand Index
40	f	0-12 years	0.899	906.0976631	119	Grand Index
40	f	0-12 years	0.9	906.3426776	119	Grand Index
40	f	0-12 years	0.901	906.587692	119	Grand Index
40	f	0-12 years	0.902	906.8327065	119	Grand Index
40	f	0-12 years	0.903	906.8936669	119	Grand Index
40	f	0-12 years	0.904	906.9346524	120	Grand Index
40	f	0-12 years	0.905	906.975638	120	Grand Index
40	f	0-12 years	0.906	907.116	120	Grand Index
40	f	0-12 years	0.907	907.402	120	Grand Index
40	f	0-12 years	0.908	907.688	120	Grand Index
40	f	0-12 years	0.909	907.974	120	Grand Index
40	f	0-12 years	0.91	908.1176612	120	Grand Index
40	f	0-12 years	0.911	908.2470885	120	Grand Index
40	f	0-12 years	0.912	908.3765158	120	Grand Index
40	f	0-12 years	0.913	908.7531429	120	Grand Index
40	f	0-12 years	0.914	909.4817156	120	Grand Index
40	f	0-12 years	0.915	910.2102883	121	Grand Index
40	f	0-12 years	0.916	910.938861	121	Grand Index
40	f	0-12 years	0.917	911.524	121	Grand Index
40	f	0-12 years	0.918	912.096	121	Grand Index
40	f	0-12 years	0.919	912.668	121	Grand Index
40	f	0-12 years	0.92	913.24	121	Grand Index
40	f	0-12 years	0.921	913.812	121	Grand Index
40	f	0-12 years	0.922	914.384	121	Grand Index
40	f	0-12 years	0.923	914.956	121	Grand Index
40	f	0-12 years	0.924	915.4497675	121	Grand Index
40	f	0-12 years	0.925	915.9370157	122	Grand Index
40	f	0-12 years	0.926	916.4242638	122	Grand Index
40	f	0-12 years	0.927	916.7084166	122	Grand Index
40	f	0-12 years	0.928	916.7195558	122	Grand Index
40	f	0-12 years	0.929	916.730695	122	Grand Index
40	f	0-12 years	0.93	916.7418343	122	Grand Index

Age	Gender	Education	Percentile	Raw Score	Norm Score	Assessment
40	f	0-12 years	0.931	918.1410781	122	Grand Index
40	f	0-12 years	0.932	919.6446907	122	Grand Index
40	f	0-12 years	0.933	921.1483033	122	Grand Index
40	f	0-12 years	0.934	922.248	123	Grand Index
40	f	0-12 years	0.935	922.82	123	Grand Index
40	f	0-12 years	0.936	923.392	123	Grand Index
40	f	0-12 years	0.937	923.964	123	Grand Index
40	f	0-12 years	0.938	924.587132	123	Grand Index
40	f	0-12 years	0.939	925.2136982	123	Grand Index
40	f	0-12 years	0.94	925.8402644	123	Grand Index
40	f	0-12 years	0.941	926.2927513	123	Grand Index
40	f	0-12 years	0.942	926.5241851	124	Grand Index
40	f	0-12 years	0.943	926.7556189	124	Grand Index
40	f	0-12 years	0.944	926.9870527	124	Grand Index
40	f	0-12 years	0.945	927.27	124	Grand Index
40	f	0-12 years	0.946	927.556	124	Grand Index
40	f	0-12 years	0.947	927.842	124	Grand Index
40	f	0-12 years	0.948	928.4731329	124	Grand Index
40	f	0-12 years	0.949	929.5302893	125	Grand Index
40	f	0-12 years	0.95	930.5874457	125	Grand Index
40	f	0-12 years	0.951	931.6446021	125	Grand Index
40	f	0-12 years	0.952	932.0509435	125	Grand Index
40	f	0-12 years	0.953	932.4237871	125	Grand Index
40	f	0-12 years	0.954	932.7966308	125	Grand Index
40	f	0-12 years	0.955	934.0739644	125	Grand Index
40	f	0-12 years	0.956	936.4366861	126	Grand Index
40	f	0-12 years	0.957	938.7994077	126	Grand Index
40	f	0-12 years	0.958	941.1621294	126	Grand Index
40	f	0-12 years	0.959	941.4636781	126	Grand Index
40	f	0-12 years	0.96	941.6749564	126	Grand Index
40	f	0-12 years	0.961	941.8862347	126	Grand Index
40	f	0-12 years	0.962	942.3960059	127	Grand Index
40	f	0-12 years	0.963	943.2540186	127	Grand Index
40	f	0-12 years	0.964	944.1120314	127	Grand Index
40	f	0-12 years	0.965	944.9700441	127	Grand Index
40	f	0-12 years	0.966	945.0691961	127	Grand Index
40	f	0-12 years	0.967	945.1408531	128	Grand Index
40	f	0-12 years	0.968	945.2125101	128	Grand Index
40	f	0-12 years	0.969	946.021014	128	Grand Index
40	f	0-12 years	0.97	947.6653443	128	Grand Index
40	f	0-12 years	0.971	949.3096745	128	Grand Index
40	f	0-12 years	0.972	950.9540047	129	Grand Index
40	f	0-12 years	0.973	952.39	129	Grand Index
40	f	0-12 years	0.974	953.82	129	Grand Index
40	f	0-12 years	0.975	955.25	129	Grand Index
40	f	0-12 years	0.976	956.004697	130	Grand Index
40	f	0-12 years	0.977	956.0145746	130	Grand Index
40	f	0-12 years	0.978	956.0244522	130	Grand Index
40	f	0-12 years	0.979	956.0343298	131	Grand Index
40	f	0-12 years	0.98	956.3048667	131	Grand Index
40	f	0-12 years	0.981	956.5809891	131	Grand Index
40	f	0-12 years	0.982	956.8571115	131	Grand Index
40	f	0-12 years	0.983	958.242	132	Grand Index
40	f	0-12 years	0.984	960.816	132	Grand Index
40	f	0-12 years	0.985	963.39	133	Grand Index
40	f	0-12 years	0.986	965.964	133	Grand Index

Age	Gender	Education	Percentile	Raw Score	Norm Score	Assessment
40	f	0-12 years	0.987	967.2660808	133	Grand Index
40	f	0-12 years	0.988	968.5501203	134	Grand Index
40	f	0-12 years	0.989	969.8341597	134	Grand Index
40	f	0-12 years	0.99	972.8010976	135	Grand Index
40	f	0-12 years	0.991	977.5230582	135	Grand Index
40	f	0-12 years	0.992	982.2450187	136	Grand Index
40	f	0-12 years	0.993	986.9669793	137	Grand Index
40	f	0-12 years	0.994	996.656	138	Grand Index
40	f	0-12 years	0.995	1006.38	139	Grand Index
40	f	0-12 years	0.996	1016.104	140	Grand Index
40	f	0-12 years	0.997	1023.722046	141	Grand Index
40	f	0-12 years	0.998	1029.204476	143	Grand Index
40	f	0-12 years	0.999	1034.686906	146	Grand Index
40	m	0-12 years	0.001	609.8418649	54	Grand Index
40	m	0-12 years	0.002	615.1017151	57	Grand Index
40	m	0-12 years	0.003	621.6088423	59	Grand Index
40	m	0-12 years	0.004	629.6467182	60	Grand Index
40	m	0-12 years	0.005	637.6845941	61	Grand Index
40	m	0-12 years	0.006	642.0326667	62	Grand Index
40	m	0-12 years	0.007	645.9614434	63	Grand Index
40	m	0-12 years	0.008	648.6844625	64	Grand Index
40	m	0-12 years	0.009	649.1378203	65	Grand Index
40	m	0-12 years	0.01	649.5911781	65	Grand Index
40	m	0-12 years	0.011	650.75773	66	Grand Index
40	m	0-12 years	0.012	652.1071522	66	Grand Index
40	m	0-12 years	0.013	653.9731659	67	Grand Index
40	m	0-12 years	0.014	657.4320035	67	Grand Index
40	m	0-12 years	0.015	660.890841	67	Grand Index
40	m	0-12 years	0.016	662.5073589	68	Grand Index
40	m	0-12 years	0.017	663.3110887	68	Grand Index
40	m	0-12 years	0.018	664.168	69	Grand Index
40	m	0-12 years	0.019	665.344	69	Grand Index
40	m	0-12 years	0.02	666.52	69	Grand Index
40	m	0-12 years	0.021	668.6302735	69	Grand Index
40	m	0-12 years	0.022	671.3848735	70	Grand Index
40	m	0-12 years	0.023	674.0270535	70	Grand Index
40	m	0-12 years	0.024	674.0273637	70	Grand Index
40	m	0-12 years	0.025	674.0276739	71	Grand Index
40	m	0-12 years	0.026	674.5984884	71	Grand Index
40	m	0-12 years	0.027	675.7635782	71	Grand Index
40	m	0-12 years	0.028	676.928668	71	Grand Index
40	m	0-12 years	0.029	677.4029631	72	Grand Index
40	m	0-12 years	0.03	677.8322064	72	Grand Index
40	m	0-12 years	0.031	678.6885671	72	Grand Index
40	m	0-12 years	0.032	680.2193238	72	Grand Index
40	m	0-12 years	0.033	681.7500805	72	Grand Index
40	m	0-12 years	0.034	682.0168177	73	Grand Index
40	m	0-12 years	0.035	682.036917	73	Grand Index
40	m	0-12 years	0.036	682.269531	73	Grand Index
40	m	0-12 years	0.037	683.0334317	73	Grand Index
40	m	0-12 years	0.038	683.7973325	73	Grand Index
40	m	0-12 years	0.039	684.576	74	Grand Index
40	m	0-12 years	0.04	685.36	74	Grand Index
40	m	0-12 years	0.041	686.0857143	74	Grand Index
40	m	0-12 years	0.042	686.552381	74	Grand Index
40	m	0-12 years	0.043	687.0190476	74	Grand Index

Age	Gender	Education	Percentile	Raw Score	Norm Score	Assessment
40	m	0-12 years	0.044	687.3912381	74	Grand Index
40	m	0-12 years	0.045	687.7085714	75	Grand Index
40	m	0-12 years	0.048	688	75	Grand Index
40	m	0-12 years	0.049	688.832	75	Grand Index
40	m	0-12 years	0.05	690.4	75	Grand Index
40	m	0-12 years	0.051	691.968	75	Grand Index
40	m	0-12 years	0.053	692	76	Grand Index
40	m	0-12 years	0.054	692.504	76	Grand Index
40	m	0-12 years	0.055	693.68	76	Grand Index
40	m	0-12 years	0.056	694.856	76	Grand Index
40	m	0-12 years	0.057	695.4837766	76	Grand Index
40	m	0-12 years	0.058	696.0350569	76	Grand Index
40	m	0-12 years	0.059	696.4585262	77	Grand Index
40	m	0-12 years	0.06	696.6183851	77	Grand Index
40	m	0-12 years	0.061	696.778244	77	Grand Index
40	m	0-12 years	0.062	697.1746349	77	Grand Index
40	m	0-12 years	0.063	697.6394957	77	Grand Index
40	m	0-12 years	0.064	698.264	77	Grand Index
40	m	0-12 years	0.065	699.44	77	Grand Index
40	m	0-12 years	0.066	700.616	77	Grand Index
40	m	0-12 years	0.067	701.792	78	Grand Index
40	m	0-12 years	0.068	702.968	78	Grand Index
40	m	0-12 years	0.071	704	78	Grand Index
40	m	0-12 years	0.072	704.448	78	Grand Index
40	m	0-12 years	0.073	705.232	78	Grand Index
40	m	0-12 years	0.076	706	79	Grand Index
40	m	0-12 years	0.077	706.0187755	79	Grand Index
40	m	0-12 years	0.078	706.0587755	79	Grand Index
40	m	0-12 years	0.079	706.0987755	79	Grand Index
40	m	0-12 years	0.08	706.7853061	79	Grand Index
40	m	0-12 years	0.081	707.5293061	79	Grand Index
40	m	0-12 years	0.086	708	80	Grand Index
40	m	0-12 years	0.087	708.0195489	80	Grand Index
40	m	0-12 years	0.088	708.0932331	80	Grand Index
40	m	0-12 years	0.089	708.1669173	80	Grand Index
40	m	0-12 years	0.09	708.5387396	80	Grand Index
40	m	0-12 years	0.091	709.0298171	80	Grand Index
40	m	0-12 years	0.092	709.4765127	80	Grand Index
40	m	0-12 years	0.093	709.695751	80	Grand Index
40	m	0-12 years	0.094	709.9149892	80	Grand Index
40	m	0-12 years	0.095	710.1965222	80	Grand Index
40	m	0-12 years	0.096	710.5175084	80	Grand Index
40	m	0-12 years	0.097	710.870712	81	Grand Index
40	m	0-12 years	0.098	711.7179153	81	Grand Index
40	m	0-12 years	0.099	712.5651187	81	Grand Index
40	m	0-12 years	0.1	713.3840604	81	Grand Index
40	m	0-12 years	0.101	714.1758708	81	Grand Index
40	m	0-12 years	0.102	714.9676812	81	Grand Index
40	m	0-12 years	0.103	715.376	81	Grand Index
40	m	0-12 years	0.104	715.768	81	Grand Index
40	m	0-12 years	0.109	716	82	Grand Index
40	m	0-12 years	0.11	716.4799944	82	Grand Index
40	m	0-12 years	0.111	718.047976	82	Grand Index
40	m	0-12 years	0.112	719.6159576	82	Grand Index
40	m	0-12 years	0.113	719.999967	82	Grand Index
40	m	0-12 years	0.114	719.9999854	82	Grand Index

Age	Gender	Education	Percentile	Raw Score	Norm Score	Assessment
40	m	0-12 years	0.115	720.16	82	Grand Index
40	m	0-12 years	0.116	720.944	82	Grand Index
40	m	0-12 years	0.117	721.728	82	Grand Index
40	m	0-12 years	0.118	722.512	82	Grand Index
40	m	0-12 years	0.119	723.296	82	Grand Index
40	m	0-12 years	0.12	724.04	82	Grand Index
40	m	0-12 years	0.121	724.432	82	Grand Index
40	m	0-12 years	0.122	724.824	83	Grand Index
40	m	0-12 years	0.125	725	83	Grand Index
40	m	0-12 years	0.126	725.784	83	Grand Index
40	m	0-12 years	0.127	726.568	83	Grand Index
40	m	0-12 years	0.132	727	83	Grand Index
40	m	0-12 years	0.133	727.2272232	83	Grand Index
40	m	0-12 years	0.134	727.8821607	83	Grand Index
40	m	0-12 years	0.135	728.5370983	83	Grand Index
40	m	0-12 years	0.136	729.0854822	84	Grand Index
40	m	0-12 years	0.137	729.6065447	84	Grand Index
40	m	0-12 years	0.14	730	84	Grand Index
40	m	0-12 years	0.141	730.272	84	Grand Index
40	m	0-12 years	0.142	730.664	84	Grand Index
40	m	0-12 years	0.143	731.0007266	84	Grand Index
40	m	0-12 years	0.144	731.0058129	84	Grand Index
40	m	0-12 years	0.145	731.0108992	84	Grand Index
40	m	0-12 years	0.146	731.241965	84	Grand Index
40	m	0-12 years	0.147	731.6288787	84	Grand Index
40	m	0-12 years	0.15	732	84	Grand Index
40	m	0-12 years	0.151	732.192	85	Grand Index
40	m	0-12 years	0.152	732.584	85	Grand Index
40	m	0-12 years	0.153	732.976	85	Grand Index
40	m	0-12 years	0.155	733	85	Grand Index
40	m	0-12 years	0.156	733.1385635	85	Grand Index
40	m	0-12 years	0.157	733.4959116	85	Grand Index
40	m	0-12 years	0.158	733.8532597	85	Grand Index
40	m	0-12 years	0.159	733.9406609	85	Grand Index
40	m	0-12 years	0.16	733.9753896	85	Grand Index
40	m	0-12 years	0.161	734.159182	85	Grand Index
40	m	0-12 years	0.162	734.7156335	85	Grand Index
40	m	0-12 years	0.163	735.272085	85	Grand Index
40	m	0-12 years	0.164	735.5868371	85	Grand Index
40	m	0-12 years	0.165	735.8143088	85	Grand Index
40	m	0-12 years	0.166	736.0225153	85	Grand Index
40	m	0-12 years	0.167	736.1450986	86	Grand Index
40	m	0-12 years	0.168	736.2676818	86	Grand Index
40	m	0-12 years	0.169	736.7311598	86	Grand Index
40	m	0-12 years	0.17	737.3925765	86	Grand Index
40	m	0-12 years	0.171	738.0174161	86	Grand Index
40	m	0-12 years	0.172	738.2307635	86	Grand Index
40	m	0-12 years	0.173	738.4441108	86	Grand Index
40	m	0-12 years	0.174	738.8440165	86	Grand Index
40	m	0-12 years	0.175	739.4089546	86	Grand Index
40	m	0-12 years	0.176	739.9738928	86	Grand Index
40	m	0-12 years	0.177	739.99102	86	Grand Index
40	m	0-12 years	0.178	739.9967346	86	Grand Index
40	m	0-12 years	0.179	740.168	86	Grand Index
40	m	0-12 years	0.18	740.56	86	Grand Index
40	m	0-12 years	0.181	740.952	86	Grand Index

Age	Gender	Education	Percentile	Raw Score	Norm Score	Assessment
40	m	0-12 years	0.188	741	87	Grand Index
40	m	0-12 years	0.189	741.088	87	Grand Index
40	m	0-12 years	0.19	741.48	87	Grand Index
40	m	0-12 years	0.191	741.872	87	Grand Index
40	m	0-12 years	0.193	742	87	Grand Index
40	m	0-12 years	0.194	742.048	87	Grand Index
40	m	0-12 years	0.195	742.44	87	Grand Index
40	m	0-12 years	0.196	742.832	87	Grand Index
40	m	0-12 years	0.197	743.224	87	Grand Index
40	m	0-12 years	0.198	743.616	87	Grand Index
40	m	0-12 years	0.199	744.024	87	Grand Index
40	m	0-12 years	0.2	745.2	87	Grand Index
40	m	0-12 years	0.201	746.376	87	Grand Index
40	m	0-12 years	0.206	747	88	Grand Index
40	m	0-12 years	0.207	747.288	88	Grand Index
40	m	0-12 years	0.208	748.072	88	Grand Index
40	m	0-12 years	0.209	748.856	88	Grand Index
40	m	0-12 years	0.21	749.32	88	Grand Index
40	m	0-12 years	0.211	749.712	88	Grand Index
40	m	0-12 years	0.216	750	88	Grand Index
40	m	0-12 years	0.217	750.064	88	Grand Index
40	m	0-12 years	0.218	750.456	88	Grand Index
40	m	0-12 years	0.219	750.848	88	Grand Index
40	m	0-12 years	0.22	751.48	88	Grand Index
40	m	0-12 years	0.221	752.264	88	Grand Index
40	m	0-12 years	0.222	753.0009905	89	Grand Index
40	m	0-12 years	0.223	753.0171681	89	Grand Index
40	m	0-12 years	0.224	753.0333458	89	Grand Index
40	m	0-12 years	0.225	753.2330157	89	Grand Index
40	m	0-12 years	0.226	753.608838	89	Grand Index
40	m	0-12 years	0.227	753.9846603	89	Grand Index
40	m	0-12 years	0.229	754	89	Grand Index
40	m	0-12 years	0.23	754.1933474	89	Grand Index
40	m	0-12 years	0.231	754.6670484	89	Grand Index
40	m	0-12 years	0.232	755.1407495	89	Grand Index
40	m	0-12 years	0.233	755.3407559	89	Grand Index
40	m	0-12 years	0.234	755.4951466	89	Grand Index
40	m	0-12 years	0.235	755.6500018	89	Grand Index
40	m	0-12 years	0.236	755.8059101	89	Grand Index
40	m	0-12 years	0.237	755.9618184	89	Grand Index
40	m	0-12 years	0.239	756	89	Grand Index
40	m	0-12 years	0.24	756.0584795	89	Grand Index
40	m	0-12 years	0.241	756.3450292	89	Grand Index
40	m	0-12 years	0.242	756.6315789	90	Grand Index
40	m	0-12 years	0.243	756.7998596	90	Grand Index
40	m	0-12 years	0.244	756.9053099	90	Grand Index
40	m	0-12 years	0.245	757.04	90	Grand Index
40	m	0-12 years	0.246	757.432	90	Grand Index
40	m	0-12 years	0.247	757.824	90	Grand Index
40	m	0-12 years	0.248	758.216	90	Grand Index
40	m	0-12 years	0.249	758.608	90	Grand Index
40	m	0-12 years	0.25	759	90	Grand Index
40	m	0-12 years	0.251	759.0825963	90	Grand Index
40	m	0-12 years	0.252	759.1651927	90	Grand Index
40	m	0-12 years	0.253	759.5256209	90	Grand Index
40	m	0-12 years	0.254	760.2270245	90	Grand Index

Age	Gender	Education	Percentile	Raw Score	Norm Score	Assessment
40	m	0-12 years	0.255	760.9284282	90	Grand Index
40	m	0-12 years	0.262	761	90	Grand Index
40	m	0-12 years	0.263	761.096	90	Grand Index
40	m	0-12 years	0.264	761.488	91	Grand Index
40	m	0-12 years	0.265	761.88	91	Grand Index
40	m	0-12 years	0.275	762	91	Grand Index
40	m	0-12 years	0.276	762.192	91	Grand Index
40	m	0-12 years	0.277	762.584	91	Grand Index
40	m	0-12 years	0.278	762.976	91	Grand Index
40	m	0-12 years	0.28	763	91	Grand Index
40	m	0-12 years	0.281	763.152	91	Grand Index
40	m	0-12 years	0.282	763.544	91	Grand Index
40	m	0-12 years	0.283	763.936	91	Grand Index
40	m	0-12 years	0.284	764.264	91	Grand Index
40	m	0-12 years	0.285	764.5795122	91	Grand Index
40	m	0-12 years	0.286	764.8267317	92	Grand Index
40	m	0-12 years	0.287	764.9032195	92	Grand Index
40	m	0-12 years	0.288	764.9797073	92	Grand Index
40	m	0-12 years	0.293	765	92	Grand Index
40	m	0-12 years	0.294	765.248	92	Grand Index
40	m	0-12 years	0.295	765.64	92	Grand Index
40	m	0-12 years	0.296	766.032	92	Grand Index
40	m	0-12 years	0.297	766.424	92	Grand Index
40	m	0-12 years	0.298	766.816	92	Grand Index
40	m	0-12 years	0.299	767.208	92	Grand Index
40	m	0-12 years	0.3	767.6	92	Grand Index
40	m	0-12 years	0.301	767.992	92	Grand Index
40	m	0-12 years	0.302	768.768	92	Grand Index
40	m	0-12 years	0.303	769.552	92	Grand Index
40	m	0-12 years	0.306	770	92	Grand Index
40	m	0-12 years	0.307	770.688	92	Grand Index
40	m	0-12 years	0.308	771.472	92	Grand Index
40	m	0-12 years	0.313	772	93	Grand Index
40	m	0-12 years	0.314	772.0214132	93	Grand Index
40	m	0-12 years	0.315	772.1167992	93	Grand Index
40	m	0-12 years	0.316	772.2121852	93	Grand Index
40	m	0-12 years	0.317	772.9710921	93	Grand Index
40	m	0-12 years	0.318	774.0517061	93	Grand Index
40	m	0-12 years	0.323	775	93	Grand Index
40	m	0-12 years	0.324	775.008	93	Grand Index
40	m	0-12 years	0.325	775.4	93	Grand Index
40	m	0-12 years	0.326	775.792	93	Grand Index
40	m	0-12 years	0.327	776.184	93	Grand Index
40	m	0-12 years	0.328	776.576	93	Grand Index
40	m	0-12 years	0.329	776.968	93	Grand Index
40	m	0-12 years	0.33	777.36	93	Grand Index
40	m	0-12 years	0.331	777.752	93	Grand Index
40	m	0-12 years	0.332	778.144	93	Grand Index
40	m	0-12 years	0.333	778.536	94	Grand Index
40	m	0-12 years	0.334	778.928	94	Grand Index
40	m	0-12 years	0.336	779	94	Grand Index
40	m	0-12 years	0.337	779.104	94	Grand Index
40	m	0-12 years	0.338	779.496	94	Grand Index
40	m	0-12 years	0.339	779.888	94	Grand Index
40	m	0-12 years	0.34	780.28	94	Grand Index
40	m	0-12 years	0.341	780.672	94	Grand Index

Age	Gender	Education	Percentile	Raw Score	Norm Score	Assessment
40	m	0-12 years	0.344	781	94	Grand Index
40	m	0-12 years	0.345	781.1836399	94	Grand Index
40	m	0-12 years	0.346	781.4835851	94	Grand Index
40	m	0-12 years	0.347	781.7928713	94	Grand Index
40	m	0-12 years	0.348	782.2453859	94	Grand Index
40	m	0-12 years	0.349	782.6979004	94	Grand Index
40	m	0-12 years	0.35	782.9356322	94	Grand Index
40	m	0-12 years	0.351	782.9671724	94	Grand Index
40	m	0-12 years	0.352	782.9987126	94	Grand Index
40	m	0-12 years	0.357	783	95	Grand Index
40	m	0-12 years	0.358	783.336	95	Grand Index
40	m	0-12 years	0.359	783.728	95	Grand Index
40	m	0-12 years	0.362	784	95	Grand Index
40	m	0-12 years	0.363	784.296	95	Grand Index
40	m	0-12 years	0.364	784.688	95	Grand Index
40	m	0-12 years	0.367	785	95	Grand Index
40	m	0-12 years	0.368	785.256	95	Grand Index
40	m	0-12 years	0.369	785.648	95	Grand Index
40	m	0-12 years	0.372	786	95	Grand Index
40	m	0-12 years	0.373	786.025743	95	Grand Index
40	m	0-12 years	0.374	786.0724618	95	Grand Index
40	m	0-12 years	0.375	786.1191806	95	Grand Index
40	m	0-12 years	0.376	786.4644618	95	Grand Index
40	m	0-12 years	0.377	786.809743	95	Grand Index
40	m	0-12 years	0.382	787	95	Grand Index
40	m	0-12 years	0.383	787.136	96	Grand Index
40	m	0-12 years	0.384	787.528	96	Grand Index
40	m	0-12 years	0.385	787.92	96	Grand Index
40	m	0-12 years	0.387	788	96	Grand Index
40	m	0-12 years	0.388	788.096	96	Grand Index
40	m	0-12 years	0.389	788.488	96	Grand Index
40	m	0-12 years	0.39	788.88	96	Grand Index
40	m	0-12 years	0.391	789.272	96	Grand Index
40	m	0-12 years	0.392	789.664	96	Grand Index
40	m	0-12 years	0.393	790.0489572	96	Grand Index
40	m	0-12 years	0.394	790.3916576	96	Grand Index
40	m	0-12 years	0.395	790.7343581	96	Grand Index
40	m	0-12 years	0.396	790.9034131	96	Grand Index
40	m	0-12 years	0.397	790.9527127	96	Grand Index
40	m	0-12 years	0.403	791	96	Grand Index
40	m	0-12 years	0.404	791.368	96	Grand Index
40	m	0-12 years	0.405	791.76	96	Grand Index
40	m	0-12 years	0.408	792	97	Grand Index
40	m	0-12 years	0.409	792.328	97	Grand Index
40	m	0-12 years	0.41	792.72	97	Grand Index
40	m	0-12 years	0.411	793.112	97	Grand Index
40	m	0-12 years	0.412	793.504	97	Grand Index
40	m	0-12 years	0.413	793.896	97	Grand Index
40	m	0-12 years	0.414	794.288	97	Grand Index
40	m	0-12 years	0.415	794.68	97	Grand Index
40	m	0-12 years	0.416	795.072	97	Grand Index
40	m	0-12 years	0.417	795.464	97	Grand Index
40	m	0-12 years	0.418	795.856	97	Grand Index
40	m	0-12 years	0.423	796	97	Grand Index
40	m	0-12 years	0.424	796.064228	97	Grand Index
40	m	0-12 years	0.425	796.1852732	97	Grand Index

Age	Gender	Education	Percentile	Raw Score	Norm Score	Assessment
40	m	0-12 years	0.426	796.3063183	97	Grand Index
40	m	0-12 years	0.427	796.5742138	97	Grand Index
40	m	0-12 years	0.428	796.8451686	97	Grand Index
40	m	0-12 years	0.431	797	97	Grand Index
40	m	0-12 years	0.432	797.688	97	Grand Index
40	m	0-12 years	0.433	798.472	97	Grand Index
40	m	0-12 years	0.434	799.128	98	Grand Index
40	m	0-12 years	0.435	799.52	98	Grand Index
40	m	0-12 years	0.436	799.912	98	Grand Index
40	m	0-12 years	0.437	800.304	98	Grand Index
40	m	0-12 years	0.438	800.696	98	Grand Index
40	m	0-12 years	0.439	801.176	98	Grand Index
40	m	0-12 years	0.44	801.96	98	Grand Index
40	m	0-12 years	0.441	802.744	98	Grand Index
40	m	0-12 years	0.446	803	98	Grand Index
40	m	0-12 years	0.447	803.224	98	Grand Index
40	m	0-12 years	0.448	803.616	98	Grand Index
40	m	0-12 years	0.451	804	98	Grand Index
40	m	0-12 years	0.452	804.184	98	Grand Index
40	m	0-12 years	0.453	804.576	98	Grand Index
40	m	0-12 years	0.454	804.968	98	Grand Index
40	m	0-12 years	0.461	805	99	Grand Index
40	m	0-12 years	0.462	805.104	99	Grand Index
40	m	0-12 years	0.463	805.496	99	Grand Index
40	m	0-12 years	0.464	805.888	99	Grand Index
40	m	0-12 years	0.469	806	99	Grand Index
40	m	0-12 years	0.47	806.24	99	Grand Index
40	m	0-12 years	0.471	806.632	99	Grand Index
40	m	0-12 years	0.472	807.024	99	Grand Index
40	m	0-12 years	0.473	807.416	99	Grand Index
40	m	0-12 years	0.474	807.808	99	Grand Index
40	m	0-12 years	0.479	808	99	Grand Index
40	m	0-12 years	0.48	808.16	99	Grand Index
40	m	0-12 years	0.481	808.552	99	Grand Index
40	m	0-12 years	0.482	808.944	99	Grand Index
40	m	0-12 years	0.483	809.336	99	Grand Index
40	m	0-12 years	0.484	809.728	99	Grand Index
40	m	0-12 years	0.489	810	100	Grand Index
40	m	0-12 years	0.49	810.08	100	Grand Index
40	m	0-12 years	0.491	810.472	100	Grand Index
40	m	0-12 years	0.492	810.864	100	Grand Index
40	m	0-12 years	0.493	811.172041	100	Grand Index
40	m	0-12 years	0.494	811.4354788	100	Grand Index
40	m	0-12 years	0.495	811.6776081	100	Grand Index
40	m	0-12 years	0.496	811.7322231	100	Grand Index
40	m	0-12 years	0.497	811.786838	100	Grand Index
40	m	0-12 years	0.498	811.8626697	100	Grand Index
40	m	0-12 years	0.499	811.955789	100	Grand Index
40	m	0-12 years	0.5	812.0489083	100	Grand Index
40	m	0-12 years	0.501	812.4217362	100	Grand Index
40	m	0-12 years	0.502	812.7945642	100	Grand Index
40	m	0-12 years	0.503	813.176	100	Grand Index
40	m	0-12 years	0.504	813.568	100	Grand Index
40	m	0-12 years	0.505	813.96	100	Grand Index
40	m	0-12 years	0.506	814.352	100	Grand Index
40	m	0-12 years	0.507	814.744	100	Grand Index

Age	Gender	Education	Percentile	Raw Score	Norm Score	Assessment
40	m	0-12 years	0.508	815.136	100	Grand Index
40	m	0-12 years	0.509	815.528	100	Grand Index
40	m	0-12 years	0.51	815.92	100	Grand Index
40	m	0-12 years	0.511	816.312	100	Grand Index
40	m	0-12 years	0.512	816.704	100	Grand Index
40	m	0-12 years	0.517	817	101	Grand Index
40	m	0-12 years	0.518	817.0085666	101	Grand Index
40	m	0-12 years	0.519	817.0685326	101	Grand Index
40	m	0-12 years	0.52	817.1284986	101	Grand Index
40	m	0-12 years	0.521	817.3494844	101	Grand Index
40	m	0-12 years	0.522	817.6815184	101	Grand Index
40	m	0-12 years	0.528	818	101	Grand Index
40	m	0-12 years	0.529	818.368	101	Grand Index
40	m	0-12 years	0.53	818.76	101	Grand Index
40	m	0-12 years	0.535	819	101	Grand Index
40	m	0-12 years	0.536	819.0076211	101	Grand Index
40	m	0-12 years	0.537	819.034295	101	Grand Index
40	m	0-12 years	0.538	819.060969	101	Grand Index
40	m	0-12 years	0.539	819.6244485	101	Grand Index
40	m	0-12 years	0.54	820.3817746	102	Grand Index
40	m	0-12 years	0.541	821.0243967	102	Grand Index
40	m	0-12 years	0.542	821.1572231	102	Grand Index
40	m	0-12 years	0.543	821.2900496	102	Grand Index
40	m	0-12 years	0.544	821.5028099	102	Grand Index
40	m	0-12 years	0.545	821.7619835	102	Grand Index
40	m	0-12 years	0.546	822.064	102	Grand Index
40	m	0-12 years	0.547	822.848	102	Grand Index
40	m	0-12 years	0.548	823.632	102	Grand Index
40	m	0-12 years	0.549	824.208	102	Grand Index
40	m	0-12 years	0.55	824.6	102	Grand Index
40	m	0-12 years	0.551	824.992	102	Grand Index
40	m	0-12 years	0.552	825.4102047	102	Grand Index
40	m	0-12 years	0.553	825.8289554	102	Grand Index
40	m	0-12 years	0.554	826.2247769	102	Grand Index
40	m	0-12 years	0.555	826.5900262	102	Grand Index
40	m	0-12 years	0.556	826.9552756	102	Grand Index
40	m	0-12 years	0.557	827.344	102	Grand Index
40	m	0-12 years	0.558	827.736	102	Grand Index
40	m	0-12 years	0.563	828	102	Grand Index
40	m	0-12 years	0.564	828.088	102	Grand Index
40	m	0-12 years	0.565	828.48	102	Grand Index
40	m	0-12 years	0.566	828.872	102	Grand Index
40	m	0-12 years	0.579	829	103	Grand Index
40	m	0-12 years	0.58	829.36	103	Grand Index
40	m	0-12 years	0.581	829.752	103	Grand Index
40	m	0-12 years	0.589	830	103	Grand Index
40	m	0-12 years	0.59	830.1512834	103	Grand Index
40	m	0-12 years	0.591	830.3630802	103	Grand Index
40	m	0-12 years	0.592	830.5697188	103	Grand Index
40	m	0-12 years	0.593	830.749922	104	Grand Index
40	m	0-12 years	0.594	830.9301253	104	Grand Index
40	m	0-12 years	0.596	831	104	Grand Index
40	m	0-12 years	0.597	831.024	104	Grand Index
40	m	0-12 years	0.598	831.416	104	Grand Index
40	m	0-12 years	0.599	831.808	104	Grand Index
40	m	0-12 years	0.6	832.2	104	Grand Index

Age	Gender	Education	Percentile	Raw Score	Norm Score	Assessment
40	m	0-12 years	0.601	832.592	104	Grand Index
40	m	0-12 years	0.602	832.984	104	Grand Index
40	m	0-12 years	0.604	833	104	Grand Index
40	m	0-12 years	0.605	833.16	104	Grand Index
40	m	0-12 years	0.606	833.552	104	Grand Index
40	m	0-12 years	0.607	833.944	104	Grand Index
40	m	0-12 years	0.608	834.336	104	Grand Index
40	m	0-12 years	0.609	834.728	104	Grand Index
40	m	0-12 years	0.61	835.12	104	Grand Index
40	m	0-12 years	0.611	835.512	104	Grand Index
40	m	0-12 years	0.612	835.904	104	Grand Index
40	m	0-12 years	0.614	836	104	Grand Index
40	m	0-12 years	0.615	836.041624	104	Grand Index
40	m	0-12 years	0.616	836.2455815	104	Grand Index
40	m	0-12 years	0.617	836.449539	104	Grand Index
40	m	0-12 years	0.618	836.6431031	105	Grand Index
40	m	0-12 years	0.619	836.8311455	105	Grand Index
40	m	0-12 years	0.62	837.04	105	Grand Index
40	m	0-12 years	0.621	837.432	105	Grand Index
40	m	0-12 years	0.622	837.824	105	Grand Index
40	m	0-12 years	0.623	838.216	105	Grand Index
40	m	0-12 years	0.624	838.608	105	Grand Index
40	m	0-12 years	0.625	839	105	Grand Index
40	m	0-12 years	0.626	839.392	105	Grand Index
40	m	0-12 years	0.627	839.784	105	Grand Index
40	m	0-12 years	0.628	840.302979	105	Grand Index
40	m	0-12 years	0.629	840.9777959	105	Grand Index
40	m	0-12 years	0.63	841.6526128	105	Grand Index
40	m	0-12 years	0.631	841.8195136	105	Grand Index
40	m	0-12 years	0.632	841.9286967	105	Grand Index
40	m	0-12 years	0.635	842	105	Grand Index
40	m	0-12 years	0.636	842.312	105	Grand Index
40	m	0-12 years	0.637	842.704	105	Grand Index
40	m	0-12 years	0.64	843	105	Grand Index
40	m	0-12 years	0.641	843.0694468	105	Grand Index
40	m	0-12 years	0.642	843.1695319	105	Grand Index
40	m	0-12 years	0.643	843.2970213	105	Grand Index
40	m	0-12 years	0.644	843.5889362	106	Grand Index
40	m	0-12 years	0.645	843.8808511	106	Grand Index
40	m	0-12 years	0.647	844	106	Grand Index
40	m	0-12 years	0.648	844.0095238	106	Grand Index
40	m	0-12 years	0.649	844.2428571	106	Grand Index
40	m	0-12 years	0.65	844.4761905	106	Grand Index
40	m	0-12 years	0.651	844.6729524	106	Grand Index
40	m	0-12 years	0.652	844.831619	106	Grand Index
40	m	0-12 years	0.653	844.9902857	106	Grand Index
40	m	0-12 years	0.655	845	106	Grand Index
40	m	0-12 years	0.656	845.152	106	Grand Index
40	m	0-12 years	0.657	845.544	106	Grand Index
40	m	0-12 years	0.658	845.936	106	Grand Index
40	m	0-12 years	0.663	846	106	Grand Index
40	m	0-12 years	0.664	846.288	106	Grand Index
40	m	0-12 years	0.665	846.68	106	Grand Index
40	m	0-12 years	0.668	847	107	Grand Index
40	m	0-12 years	0.669	847.248	107	Grand Index
40	m	0-12 years	0.67	847.64	107	Grand Index

Age	Gender	Education	Percentile	Raw Score	Norm Score	Assessment
40	m	0-12 years	0.681	848	107	Grand Index
40	m	0-12 years	0.682	848.0019232	107	Grand Index
40	m	0-12 years	0.683	848.0041148	107	Grand Index
40	m	0-12 years	0.684	848.1328751	107	Grand Index
40	m	0-12 years	0.685	848.5226836	107	Grand Index
40	m	0-12 years	0.686	848.912492	107	Grand Index
40	m	0-12 years	0.687	849.304	107	Grand Index
40	m	0-12 years	0.688	849.696	107	Grand Index
40	m	0-12 years	0.693	850	108	Grand Index
40	m	0-12 years	0.694	850.048	108	Grand Index
40	m	0-12 years	0.695	850.44	108	Grand Index
40	m	0-12 years	0.696	850.832	108	Grand Index
40	m	0-12 years	0.698	851	108	Grand Index
40	m	0-12 years	0.699	851.0077628	108	Grand Index
40	m	0-12 years	0.7	851.3881402	108	Grand Index
40	m	0-12 years	0.701	851.7685175	108	Grand Index
40	m	0-12 years	0.702	851.9758059	108	Grand Index
40	m	0-12 years	0.703	851.9874286	108	Grand Index
40	m	0-12 years	0.704	851.9990512	108	Grand Index
40	m	0-12 years	0.709	852	108	Grand Index
40	m	0-12 years	0.71	852.32	108	Grand Index
40	m	0-12 years	0.711	852.712	108	Grand Index
40	m	0-12 years	0.712	853.104	108	Grand Index
40	m	0-12 years	0.713	853.496	108	Grand Index
40	m	0-12 years	0.714	853.888	108	Grand Index
40	m	0-12 years	0.716	854	109	Grand Index
40	m	0-12 years	0.717	854.064	109	Grand Index
40	m	0-12 years	0.718	854.456	109	Grand Index
40	m	0-12 years	0.719	854.848	109	Grand Index
40	m	0-12 years	0.721	855	109	Grand Index
40	m	0-12 years	0.722	855.024	109	Grand Index
40	m	0-12 years	0.723	855.416	109	Grand Index
40	m	0-12 years	0.724	855.808	109	Grand Index
40	m	0-12 years	0.725	856.4	109	Grand Index
40	m	0-12 years	0.726	857.184	109	Grand Index
40	m	0-12 years	0.727	857.968	109	Grand Index
40	m	0-12 years	0.732	858	109	Grand Index
40	m	0-12 years	0.733	858.336	109	Grand Index
40	m	0-12 years	0.734	858.728	109	Grand Index
40	m	0-12 years	0.735	859.117703	109	Grand Index
40	m	0-12 years	0.736	859.5021994	109	Grand Index
40	m	0-12 years	0.737	859.8866959	110	Grand Index
40	m	0-12 years	0.738	859.9865242	110	Grand Index
40	m	0-12 years	0.739	859.9940278	110	Grand Index
40	m	0-12 years	0.74	860.0647732	110	Grand Index
40	m	0-12 years	0.741	860.3821621	110	Grand Index
40	m	0-12 years	0.742	860.6995509	110	Grand Index
40	m	0-12 years	0.743	860.8583911	110	Grand Index
40	m	0-12 years	0.744	860.9330022	110	Grand Index
40	m	0-12 years	0.747	861	110	Grand Index
40	m	0-12 years	0.748	861.216	110	Grand Index
40	m	0-12 years	0.749	861.608	110	Grand Index
40	m	0-12 years	0.75	862	110	Grand Index
40	m	0-12 years	0.751	862.784	110	Grand Index
40	m	0-12 years	0.752	863.568	110	Grand Index
40	m	0-12 years	0.753	864.1201769	110	Grand Index

Age	Gender	Education	Percentile	Raw Score	Norm Score	Assessment
40	m	0-12 years	0.754	864.3878437	110	Grand Index
40	m	0-12 years	0.755	864.6555105	110	Grand Index
40	m	0-12 years	0.756	865.1464696	110	Grand Index
40	m	0-12 years	0.757	865.6628028	110	Grand Index
40	m	0-12 years	0.76	866	111	Grand Index
40	m	0-12 years	0.761	866.0988673	111	Grand Index
40	m	0-12 years	0.762	866.2230852	111	Grand Index
40	m	0-12 years	0.763	866.3824616	111	Grand Index
40	m	0-12 years	0.764	866.6502438	111	Grand Index
40	m	0-12 years	0.765	866.9180259	111	Grand Index
40	m	0-12 years	0.77	867	111	Grand Index
40	m	0-12 years	0.771	867.215337	111	Grand Index
40	m	0-12 years	0.772	867.5791823	111	Grand Index
40	m	0-12 years	0.773	867.929326	111	Grand Index
40	m	0-12 years	0.774	867.9574807	111	Grand Index
40	m	0-12 years	0.775	867.9856354	111	Grand Index
40	m	0-12 years	0.776	868.0020926	111	Grand Index
40	m	0-12 years	0.777	868.0063651	111	Grand Index
40	m	0-12 years	0.778	868.0106376	111	Grand Index
40	m	0-12 years	0.779	868.3748883	112	Grand Index
40	m	0-12 years	0.78	868.7626158	112	Grand Index
40	m	0-12 years	0.781	869.152	112	Grand Index
40	m	0-12 years	0.782	869.544	112	Grand Index
40	m	0-12 years	0.783	869.936	112	Grand Index
40	m	0-12 years	0.784	870.328	112	Grand Index
40	m	0-12 years	0.785	870.72	112	Grand Index
40	m	0-12 years	0.786	871.112	112	Grand Index
40	m	0-12 years	0.787	871.504	112	Grand Index
40	m	0-12 years	0.788	871.896	112	Grand Index
40	m	0-12 years	0.79	872	112	Grand Index
40	m	0-12 years	0.791	872.072	112	Grand Index
40	m	0-12 years	0.792	872.464	112	Grand Index
40	m	0-12 years	0.793	872.856	112	Grand Index
40	m	0-12 years	0.794	873.0450909	112	Grand Index
40	m	0-12 years	0.795	873.1163636	112	Grand Index
40	m	0-12 years	0.796	873.208	112	Grand Index
40	m	0-12 years	0.797	873.5287273	112	Grand Index
40	m	0-12 years	0.798	873.8494545	113	Grand Index
40	m	0-12 years	0.808	874	113	Grand Index
40	m	0-12 years	0.809	874.256	113	Grand Index
40	m	0-12 years	0.81	875.04	113	Grand Index
40	m	0-12 years	0.811	875.824	113	Grand Index
40	m	0-12 years	0.812	876.4635133	113	Grand Index
40	m	0-12 years	0.813	877.0612015	113	Grand Index
40	m	0-12 years	0.814	877.5665399	113	Grand Index
40	m	0-12 years	0.815	877.7528517	113	Grand Index
40	m	0-12 years	0.816	877.9391635	114	Grand Index
40	m	0-12 years	0.817	878.264	114	Grand Index
40	m	0-12 years	0.818	878.656	114	Grand Index
40	m	0-12 years	0.826	879	114	Grand Index
40	m	0-12 years	0.827	879.552	114	Grand Index
40	m	0-12 years	0.828	880.728	114	Grand Index
40	m	0-12 years	0.829	881.904	114	Grand Index
40	m	0-12 years	0.83	883.44	114	Grand Index
40	m	0-12 years	0.831	885.008	114	Grand Index
40	m	0-12 years	0.832	886.144	114	Grand Index

Age	Gender	Education	Percentile	Raw Score	Norm Score	Assessment
40	m	0-12 years	0.833	886.536	114	Grand Index
40	m	0-12 years	0.834	886.928	115	Grand Index
40	m	0-12 years	0.836	887	115	Grand Index
40	m	0-12 years	0.837	887.104	115	Grand Index
40	m	0-12 years	0.838	887.496	115	Grand Index
40	m	0-12 years	0.839	887.888	115	Grand Index
40	m	0-12 years	0.84	888.8342857	115	Grand Index
40	m	0-12 years	0.841	890.0022857	115	Grand Index
40	m	0-12 years	0.842	891.044898	115	Grand Index
40	m	0-12 years	0.843	891.444898	115	Grand Index
40	m	0-12 years	0.844	891.844898	115	Grand Index
40	m	0-12 years	0.845	892.1626378	115	Grand Index
40	m	0-12 years	0.846	892.4282796	115	Grand Index
40	m	0-12 years	0.847	892.7333938	115	Grand Index
40	m	0-12 years	0.848	893.643752	115	Grand Index
40	m	0-12 years	0.849	894.5541103	115	Grand Index
40	m	0-12 years	0.85	895.2	116	Grand Index
40	m	0-12 years	0.851	895.592	116	Grand Index
40	m	0-12 years	0.852	895.984	116	Grand Index
40	m	0-12 years	0.854	896	116	Grand Index
40	m	0-12 years	0.855	896.0220784	116	Grand Index
40	m	0-12 years	0.856	896.0761704	116	Grand Index
40	m	0-12 years	0.857	896.1302624	116	Grand Index
40	m	0-12 years	0.858	896.4276252	116	Grand Index
40	m	0-12 years	0.859	896.7655332	116	Grand Index
40	m	0-12 years	0.86	897.12	116	Grand Index
40	m	0-12 years	0.861	897.512	116	Grand Index
40	m	0-12 years	0.862	897.904	116	Grand Index
40	m	0-12 years	0.863	898.592	116	Grand Index
40	m	0-12 years	0.864	899.376	116	Grand Index
40	m	0-12 years	0.865	900.08	117	Grand Index
40	m	0-12 years	0.866	900.472	117	Grand Index
40	m	0-12 years	0.867	900.864	117	Grand Index
40	m	0-12 years	0.872	901	117	Grand Index
40	m	0-12 years	0.873	901.216	117	Grand Index
40	m	0-12 years	0.874	901.608	117	Grand Index
40	m	0-12 years	0.875	902	117	Grand Index
40	m	0-12 years	0.876	902.392	117	Grand Index
40	m	0-12 years	0.877	902.784	117	Grand Index
40	m	0-12 years	0.878	903.176	117	Grand Index
40	m	0-12 years	0.879	903.568	118	Grand Index
40	m	0-12 years	0.88	903.96	118	Grand Index
40	m	0-12 years	0.882	904	118	Grand Index
40	m	0-12 years	0.883	904.136	118	Grand Index
40	m	0-12 years	0.884	904.528	118	Grand Index
40	m	0-12 years	0.885	904.92	118	Grand Index
40	m	0-12 years	0.887	905	118	Grand Index
40	m	0-12 years	0.888	905.096	118	Grand Index
40	m	0-12 years	0.889	905.488	118	Grand Index
40	m	0-12 years	0.89	905.88	118	Grand Index
40	m	0-12 years	0.891	906.0390837	118	Grand Index
40	m	0-12 years	0.892	906.0954103	119	Grand Index
40	m	0-12 years	0.893	906.1916436	119	Grand Index
40	m	0-12 years	0.894	906.527317	119	Grand Index
40	m	0-12 years	0.895	906.8629904	119	Grand Index
40	m	0-12 years	0.897	907	119	Grand Index

Age	Gender	Education	Percentile	Raw Score	Norm Score	Assessment
40	m	0-12 years	0.898	907.0022326	119	Grand Index
40	m	0-12 years	0.899	907.0569302	119	Grand Index
40	m	0-12 years	0.9	907.1116279	119	Grand Index
40	m	0-12 years	0.901	907.4967442	119	Grand Index
40	m	0-12 years	0.902	908.2260465	119	Grand Index
40	m	0-12 years	0.903	908.9553488	119	Grand Index
40	m	0-12 years	0.904	911.1297021	120	Grand Index
40	m	0-12 years	0.905	913.3982979	120	Grand Index
40	m	0-12 years	0.906	914.8195745	120	Grand Index
40	m	0-12 years	0.907	914.9029787	120	Grand Index
40	m	0-12 years	0.908	914.986383	120	Grand Index
40	m	0-12 years	0.909	915.328	120	Grand Index
40	m	0-12 years	0.91	915.72	120	Grand Index
40	m	0-12 years	0.911	916.112	120	Grand Index
40	m	0-12 years	0.912	916.504	120	Grand Index
40	m	0-12 years	0.913	916.896	120	Grand Index
40	m	0-12 years	0.914	917.288	120	Grand Index
40	m	0-12 years	0.915	917.68	121	Grand Index
40	m	0-12 years	0.918	918	121	Grand Index
40	m	0-12 years	0.919	918.248	121	Grand Index
40	m	0-12 years	0.92	918.64	121	Grand Index
40	m	0-12 years	0.921	919.064	121	Grand Index
40	m	0-12 years	0.922	919.848	121	Grand Index
40	m	0-12 years	0.923	920.632	121	Grand Index
40	m	0-12 years	0.924	921.208	121	Grand Index
40	m	0-12 years	0.925	921.6	122	Grand Index
40	m	0-12 years	0.926	921.992	122	Grand Index
40	m	0-12 years	0.927	922.8832626	122	Grand Index
40	m	0-12 years	0.928	923.7849266	122	Grand Index
40	m	0-12 years	0.929	924.5857357	122	Grand Index
40	m	0-12 years	0.93	925.2520718	122	Grand Index
40	m	0-12 years	0.931	925.9184078	122	Grand Index
40	m	0-12 years	0.932	928.2421429	122	Grand Index
40	m	0-12 years	0.933	930.7971429	122	Grand Index
40	m	0-12 years	0.934	932.5795714	123	Grand Index
40	m	0-12 years	0.935	932.7685714	123	Grand Index
40	m	0-12 years	0.936	932.9575714	123	Grand Index
40	m	0-12 years	0.937	933.608	123	Grand Index
40	m	0-12 years	0.938	934.392	123	Grand Index
40	m	0-12 years	0.939	935.088	123	Grand Index
40	m	0-12 years	0.94	935.48	123	Grand Index
40	m	0-12 years	0.941	935.872	123	Grand Index
40	m	0-12 years	0.942	936.264	124	Grand Index
40	m	0-12 years	0.943	936.656	124	Grand Index
40	m	0-12 years	0.944	937.0754237	124	Grand Index
40	m	0-12 years	0.945	937.6913842	124	Grand Index
40	m	0-12 years	0.946	938.3073448	124	Grand Index
40	m	0-12 years	0.947	938.6673504	124	Grand Index
40	m	0-12 years	0.948	938.8353899	124	Grand Index
40	m	0-12 years	0.949	939.016	125	Grand Index
40	m	0-12 years	0.95	939.8	125	Grand Index
40	m	0-12 years	0.951	940.584	125	Grand Index
40	m	0-12 years	0.952	941.0452643	125	Grand Index
40	m	0-12 years	0.953	941.1416969	125	Grand Index
40	m	0-12 years	0.954	941.2381295	125	Grand Index
40	m	0-12 years	0.955	942.6206648	125	Grand Index

Age	Gender	Education	Percentile	Raw Score	Norm Score	Assessment
40	m	0-12 years	0.956	944.1175205	126	Grand Index
40	m	0-12 years	0.957	946.2844895	126	Grand Index
40	m	0-12 years	0.958	949.6055427	126	Grand Index
40	m	0-12 years	0.959	952.926596	126	Grand Index
40	m	0-12 years	0.96	955.1999801	126	Grand Index
40	m	0-12 years	0.961	957.2376386	126	Grand Index
40	m	0-12 years	0.962	959.0742857	127	Grand Index
40	m	0-12 years	0.963	960.3542857	127	Grand Index
40	m	0-12 years	0.964	961.6342857	127	Grand Index
40	m	0-12 years	0.965	962.28	127	Grand Index
40	m	0-12 years	0.966	962.672	127	Grand Index
40	m	0-12 years	0.969	963	128	Grand Index
40	m	0-12 years	0.97	964.042363	128	Grand Index
40	m	0-12 years	0.971	965.7448891	128	Grand Index
40	m	0-12 years	0.972	967.5274325	129	Grand Index
40	m	0-12 years	0.973	970.5369063	129	Grand Index
40	m	0-12 years	0.974	973.5463802	129	Grand Index
40	m	0-12 years	0.975	975.8163265	129	Grand Index
40	m	0-12 years	0.976	977.3763265	130	Grand Index
40	m	0-12 years	0.977	978.9363265	130	Grand Index
40	m	0-12 years	0.978	979.6099083	130	Grand Index
40	m	0-12 years	0.979	980.2457701	131	Grand Index
40	m	0-12 years	0.98	982.8333149	131	Grand Index
40	m	0-12 years	0.981	988.2507998	131	Grand Index
40	m	0-12 years	0.982	993.6682846	131	Grand Index
40	m	0-12 years	0.983	999.024352	132	Grand Index
40	m	0-12 years	0.984	1004.370183	132	Grand Index
40	m	0-12 years	0.985	1008.394884	133	Grand Index
40	m	0-12 years	0.986	1009.425022	133	Grand Index
40	m	0-12 years	0.987	1010.45516	133	Grand Index
40	m	0-12 years	0.988	1011.090037	134	Grand Index
40	m	0-12 years	0.989	1011.596721	134	Grand Index
40	m	0-12 years	0.99	1013.12	135	Grand Index
40	m	0-12 years	0.991	1018.608	135	Grand Index
40	m	0-12 years	0.992	1024.096	136	Grand Index
40	m	0-12 years	0.993	1027.792	137	Grand Index
40	m	0-12 years	0.994	1030.536	138	Grand Index
40	m	0-12 years	0.995	1033.238183	139	Grand Index
40	m	0-12 years	0.996	1035.572377	140	Grand Index
40	m	0-12 years	0.997	1037.906571	141	Grand Index
40	m	0-12 years	0.998	1040.253505	143	Grand Index
40	m	0-12 years	0.999	1042.610819	146	Grand Index
40	f	13-16 years	0.001	587.348	54	Grand Index
40	f	13-16 years	0.002	599	57	Grand Index
40	f	13-16 years	0.003	602.9188238	59	Grand Index
40	f	13-16 years	0.004	607.348	60	Grand Index
40	f	13-16 years	0.005	611.1208022	61	Grand Index
40	f	13-16 years	0.006	617.6689471	62	Grand Index
40	f	13-16 years	0.007	622.0453195	63	Grand Index
40	f	13-16 years	0.008	626	64	Grand Index
40	f	13-16 years	0.009	627.9480593	65	Grand Index
40	f	13-16 years	0.01	633.936745	65	Grand Index
40	f	13-16 years	0.011	638.0082684	66	Grand Index
40	f	13-16 years	0.012	640.117722	66	Grand Index
40	f	13-16 years	0.013	644.3279525	67	Grand Index
40	f	13-16 years	0.014	646	67	Grand Index

Age	Gender	Education	Percentile	Raw Score	Norm Score	Assessment
40	f	13-16 years	0.015	648.0774569	67	Grand Index
40	f	13-16 years	0.016	649.6242479	68	Grand Index
40	f	13-16 years	0.017	651	68	Grand Index
40	f	13-16 years	0.018	652.2275164	69	Grand Index
40	f	13-16 years	0.02	654	69	Grand Index
40	f	13-16 years	0.021	658.4815162	69	Grand Index
40	f	13-16 years	0.022	659.7420988	70	Grand Index
40	f	13-16 years	0.023	661	70	Grand Index
40	f	13-16 years	0.024	662.7030162	70	Grand Index
40	f	13-16 years	0.025	664.4063385	71	Grand Index
40	f	13-16 years	0.026	666	71	Grand Index
40	f	13-16 years	0.027	667.7749472	71	Grand Index
40	f	13-16 years	0.028	669.4780272	71	Grand Index
40	f	13-16 years	0.029	671	72	Grand Index
40	f	13-16 years	0.03	671.3452434	72	Grand Index
40	f	13-16 years	0.031	672.9822363	72	Grand Index
40	f	13-16 years	0.032	674	72	Grand Index
40	f	13-16 years	0.033	674.5312653	72	Grand Index
40	f	13-16 years	0.034	675	73	Grand Index
40	f	13-16 years	0.035	676	73	Grand Index
40	f	13-16 years	0.036	677.6359461	73	Grand Index
40	f	13-16 years	0.037	678	73	Grand Index
40	f	13-16 years	0.038	679.58781	73	Grand Index
40	f	13-16 years	0.039	680.9204085	74	Grand Index
40	f	13-16 years	0.04	681	74	Grand Index
40	f	13-16 years	0.042	682	74	Grand Index
40	f	13-16 years	0.043	682.9994828	74	Grand Index
40	f	13-16 years	0.044	683.552	74	Grand Index
40	f	13-16 years	0.045	685.2864643	75	Grand Index
40	f	13-16 years	0.046	686	75	Grand Index
40	f	13-16 years	0.047	686.4847182	75	Grand Index
40	f	13-16 years	0.048	687	75	Grand Index
40	f	13-16 years	0.049	688	75	Grand Index
40	f	13-16 years	0.051	689	75	Grand Index
40	f	13-16 years	0.052	689.2475641	76	Grand Index
40	f	13-16 years	0.053	690.787	76	Grand Index
40	f	13-16 years	0.054	691	76	Grand Index
40	f	13-16 years	0.055	692	76	Grand Index
40	f	13-16 years	0.056	692.8527858	76	Grand Index
40	f	13-16 years	0.057	693.0102856	76	Grand Index
40	f	13-16 years	0.058	694	76	Grand Index
40	f	13-16 years	0.059	694.4811393	77	Grand Index
40	f	13-16 years	0.06	695	77	Grand Index
40	f	13-16 years	0.061	696	77	Grand Index
40	f	13-16 years	0.062	696.3912233	77	Grand Index
40	f	13-16 years	0.063	697	77	Grand Index
40	f	13-16 years	0.064	697.9981447	77	Grand Index
40	f	13-16 years	0.065	699.9777872	77	Grand Index
40	f	13-16 years	0.066	700	77	Grand Index
40	f	13-16 years	0.067	700.693	78	Grand Index
40	f	13-16 years	0.068	701	78	Grand Index
40	f	13-16 years	0.069	701.988644	78	Grand Index
40	f	13-16 years	0.07	702	78	Grand Index
40	f	13-16 years	0.071	702.8327326	78	Grand Index
40	f	13-16 years	0.072	703.088	78	Grand Index
40	f	13-16 years	0.074	704	78	Grand Index

Age	Gender	Education	Percentile	Raw Score	Norm Score	Assessment
40	f	13-16 years	0.075	704.9352567	78	Grand Index
40	f	13-16 years	0.076	705.2407207	79	Grand Index
40	f	13-16 years	0.077	706	79	Grand Index
40	f	13-16 years	0.078	706.5991588	79	Grand Index
40	f	13-16 years	0.08	707	79	Grand Index
40	f	13-16 years	0.081	708	79	Grand Index
40	f	13-16 years	0.082	708.3193394	79	Grand Index
40	f	13-16 years	0.084	709	79	Grand Index
40	f	13-16 years	0.085	709.7245254	79	Grand Index
40	f	13-16 years	0.087	710	80	Grand Index
40	f	13-16 years	0.089	711	80	Grand Index
40	f	13-16 years	0.09	711.11	80	Grand Index
40	f	13-16 years	0.092	712	80	Grand Index
40	f	13-16 years	0.093	712.8673593	80	Grand Index
40	f	13-16 years	0.094	713	80	Grand Index
40	f	13-16 years	0.096	714	80	Grand Index
40	f	13-16 years	0.097	714.0097848	81	Grand Index
40	f	13-16 years	0.098	714.5373013	81	Grand Index
40	f	13-16 years	0.099	715	81	Grand Index
40	f	13-16 years	0.101	716	81	Grand Index
40	f	13-16 years	0.102	716.2523518	81	Grand Index
40	f	13-16 years	0.103	716.9679084	81	Grand Index
40	f	13-16 years	0.104	717.016	81	Grand Index
40	f	13-16 years	0.105	718.2946741	81	Grand Index
40	f	13-16 years	0.106	719	81	Grand Index
40	f	13-16 years	0.107	719.9632558	81	Grand Index
40	f	13-16 years	0.108	720	81	Grand Index
40	f	13-16 years	0.109	720.4160436	82	Grand Index
40	f	13-16 years	0.112	721	82	Grand Index
40	f	13-16 years	0.113	721.2480039	82	Grand Index
40	f	13-16 years	0.114	722	82	Grand Index
40	f	13-16 years	0.115	722.8463666	82	Grand Index
40	f	13-16 years	0.117	723	82	Grand Index
40	f	13-16 years	0.118	723.110485	82	Grand Index
40	f	13-16 years	0.119	724	82	Grand Index
40	f	13-16 years	0.12	724.5665089	82	Grand Index
40	f	13-16 years	0.121	725	82	Grand Index
40	f	13-16 years	0.122	725.0001753	83	Grand Index
40	f	13-16 years	0.123	725.5972769	83	Grand Index
40	f	13-16 years	0.124	725.9309087	83	Grand Index
40	f	13-16 years	0.125	726	83	Grand Index
40	f	13-16 years	0.126	726.1198779	83	Grand Index
40	f	13-16 years	0.127	727	83	Grand Index
40	f	13-16 years	0.128	727.712	83	Grand Index
40	f	13-16 years	0.131	729	83	Grand Index
40	f	13-16 years	0.134	730	83	Grand Index
40	f	13-16 years	0.135	730.649325	83	Grand Index
40	f	13-16 years	0.137	731	84	Grand Index
40	f	13-16 years	0.138	731.5755337	84	Grand Index
40	f	13-16 years	0.14	732	84	Grand Index
40	f	13-16 years	0.141	732.4815283	84	Grand Index
40	f	13-16 years	0.143	733	84	Grand Index
40	f	13-16 years	0.144	733.176	84	Grand Index
40	f	13-16 years	0.146	734	84	Grand Index
40	f	13-16 years	0.147	735.3445296	84	Grand Index
40	f	13-16 years	0.149	736	84	Grand Index

Age	Gender	Education	Percentile	Raw Score	Norm Score	Assessment
40	f	13-16 years	0.15	736.85	84	Grand Index
40	f	13-16 years	0.151	737.8577466	85	Grand Index
40	f	13-16 years	0.153	738	85	Grand Index
40	f	13-16 years	0.154	738.9819792	85	Grand Index
40	f	13-16 years	0.155	739	85	Grand Index
40	f	13-16 years	0.158	740	85	Grand Index
40	f	13-16 years	0.159	740.9695748	85	Grand Index
40	f	13-16 years	0.162	741	85	Grand Index
40	f	13-16 years	0.163	741.8657374	85	Grand Index
40	f	13-16 years	0.167	742	86	Grand Index
40	f	13-16 years	0.168	742.9777803	86	Grand Index
40	f	13-16 years	0.173	743	86	Grand Index
40	f	13-16 years	0.174	743.546	86	Grand Index
40	f	13-16 years	0.176	744	86	Grand Index
40	f	13-16 years	0.177	744.3361698	86	Grand Index
40	f	13-16 years	0.178	744.9061975	86	Grand Index
40	f	13-16 years	0.179	745	86	Grand Index
40	f	13-16 years	0.18	745.8295924	86	Grand Index
40	f	13-16 years	0.181	746	86	Grand Index
40	f	13-16 years	0.182	746.1304211	86	Grand Index
40	f	13-16 years	0.183	746.7876489	86	Grand Index
40	f	13-16 years	0.185	747	87	Grand Index
40	f	13-16 years	0.188	748	87	Grand Index
40	f	13-16 years	0.189	748.731	87	Grand Index
40	f	13-16 years	0.191	749	87	Grand Index
40	f	13-16 years	0.192	749.0206705	87	Grand Index
40	f	13-16 years	0.193	749.9061382	87	Grand Index
40	f	13-16 years	0.196	750	87	Grand Index
40	f	13-16 years	0.2	751	87	Grand Index
40	f	13-16 years	0.201	751.1845019	87	Grand Index
40	f	13-16 years	0.205	752	88	Grand Index
40	f	13-16 years	0.206	752.3752382	88	Grand Index
40	f	13-16 years	0.207	752.9909206	88	Grand Index
40	f	13-16 years	0.21	753	88	Grand Index
40	f	13-16 years	0.211	753.6680053	88	Grand Index
40	f	13-16 years	0.214	754	88	Grand Index
40	f	13-16 years	0.215	754.0260719	88	Grand Index
40	f	13-16 years	0.222	755	89	Grand Index
40	f	13-16 years	0.223	755.0561639	89	Grand Index
40	f	13-16 years	0.229	756	89	Grand Index
40	f	13-16 years	0.23	756.0040775	89	Grand Index
40	f	13-16 years	0.231	756.8424077	89	Grand Index
40	f	13-16 years	0.234	757	89	Grand Index
40	f	13-16 years	0.235	757.0858293	89	Grand Index
40	f	13-16 years	0.238	758	89	Grand Index
40	f	13-16 years	0.239	758.681	89	Grand Index
40	f	13-16 years	0.241	759	89	Grand Index
40	f	13-16 years	0.242	759.3698282	90	Grand Index
40	f	13-16 years	0.243	760	90	Grand Index
40	f	13-16 years	0.247	761	90	Grand Index
40	f	13-16 years	0.248	761.192	90	Grand Index
40	f	13-16 years	0.253	762	90	Grand Index
40	f	13-16 years	0.254	762.045202	90	Grand Index
40	f	13-16 years	0.255	762.4490259	90	Grand Index
40	f	13-16 years	0.259	763	90	Grand Index
40	f	13-16 years	0.26	763.0995792	90	Grand Index

Age	Gender	Education	Percentile	Raw Score	Norm Score	Assessment
40	f	13-16 years	0.262	764	90	Grand Index
40	f	13-16 years	0.263	764.8808567	90	Grand Index
40	f	13-16 years	0.266	765	91	Grand Index
40	f	13-16 years	0.267	765.4812276	91	Grand Index
40	f	13-16 years	0.269	766	91	Grand Index
40	f	13-16 years	0.27	766.9555204	91	Grand Index
40	f	13-16 years	0.274	767	91	Grand Index
40	f	13-16 years	0.276	768	91	Grand Index
40	f	13-16 years	0.277	768.1484764	91	Grand Index
40	f	13-16 years	0.278	768.6690615	91	Grand Index
40	f	13-16 years	0.281	769	91	Grand Index
40	f	13-16 years	0.284	770	91	Grand Index
40	f	13-16 years	0.285	770.0394013	91	Grand Index
40	f	13-16 years	0.286	770.929901	92	Grand Index
40	f	13-16 years	0.287	771	92	Grand Index
40	f	13-16 years	0.288	771.2819562	92	Grand Index
40	f	13-16 years	0.293	772	92	Grand Index
40	f	13-16 years	0.294	772.8935192	92	Grand Index
40	f	13-16 years	0.297	773	92	Grand Index
40	f	13-16 years	0.298	773.142	92	Grand Index
40	f	13-16 years	0.301	774	92	Grand Index
40	f	13-16 years	0.302	774.1033646	92	Grand Index
40	f	13-16 years	0.306	775	92	Grand Index
40	f	13-16 years	0.307	775.0403928	92	Grand Index
40	f	13-16 years	0.311	776	93	Grand Index
40	f	13-16 years	0.312	776.021972	93	Grand Index
40	f	13-16 years	0.313	777	93	Grand Index
40	f	13-16 years	0.314	777.212665	93	Grand Index
40	f	13-16 years	0.315	777.9472929	93	Grand Index
40	f	13-16 years	0.317	778	93	Grand Index
40	f	13-16 years	0.319	779	93	Grand Index
40	f	13-16 years	0.32	779.1426833	93	Grand Index
40	f	13-16 years	0.322	780	93	Grand Index
40	f	13-16 years	0.323	780.0435908	93	Grand Index
40	f	13-16 years	0.33	781	93	Grand Index
40	f	13-16 years	0.331	781.2794705	93	Grand Index
40	f	13-16 years	0.337	782	94	Grand Index
40	f	13-16 years	0.338	782.7772109	94	Grand Index
40	f	13-16 years	0.343	783	94	Grand Index
40	f	13-16 years	0.344	783.2711232	94	Grand Index
40	f	13-16 years	0.347	784	94	Grand Index
40	f	13-16 years	0.348	784.0793599	94	Grand Index
40	f	13-16 years	0.349	784.9235396	94	Grand Index
40	f	13-16 years	0.354	785	94	Grand Index
40	f	13-16 years	0.355	785.8817569	94	Grand Index
40	f	13-16 years	0.362	786	95	Grand Index
40	f	13-16 years	0.363	786.0071629	95	Grand Index
40	f	13-16 years	0.364	786.59349	95	Grand Index
40	f	13-16 years	0.368	787	95	Grand Index
40	f	13-16 years	0.369	787.7201482	95	Grand Index
40	f	13-16 years	0.371	788	95	Grand Index
40	f	13-16 years	0.372	788.788	95	Grand Index
40	f	13-16 years	0.376	789	95	Grand Index
40	f	13-16 years	0.377	789.7110839	95	Grand Index
40	f	13-16 years	0.381	790	95	Grand Index
40	f	13-16 years	0.382	790.429618	95	Grand Index

Age	Gender	Education	Percentile	Raw Score	Norm Score	Assessment
40	f	13-16 years	0.383	790.9678613	96	Grand Index
40	f	13-16 years	0.389	791	96	Grand Index
40	f	13-16 years	0.39	791.6149437	96	Grand Index
40	f	13-16 years	0.392	792	96	Grand Index
40	f	13-16 years	0.397	793	96	Grand Index
40	f	13-16 years	0.401	794	96	Grand Index
40	f	13-16 years	0.404	795	96	Grand Index
40	f	13-16 years	0.405	795.770561	96	Grand Index
40	f	13-16 years	0.41	796	97	Grand Index
40	f	13-16 years	0.411	796.0155371	97	Grand Index
40	f	13-16 years	0.412	796.9904472	97	Grand Index
40	f	13-16 years	0.415	797	97	Grand Index
40	f	13-16 years	0.416	797.0490968	97	Grand Index
40	f	13-16 years	0.422	798	97	Grand Index
40	f	13-16 years	0.423	798.8895972	97	Grand Index
40	f	13-16 years	0.429	799	97	Grand Index
40	f	13-16 years	0.435	800	98	Grand Index
40	f	13-16 years	0.436	800.5357379	98	Grand Index
40	f	13-16 years	0.441	801	98	Grand Index
40	f	13-16 years	0.442	801.1316468	98	Grand Index
40	f	13-16 years	0.448	802	98	Grand Index
40	f	13-16 years	0.453	803	98	Grand Index
40	f	13-16 years	0.458	804	98	Grand Index
40	f	13-16 years	0.459	804.983797	98	Grand Index
40	f	13-16 years	0.463	805	99	Grand Index
40	f	13-16 years	0.464	805.4107996	99	Grand Index
40	f	13-16 years	0.469	806	99	Grand Index
40	f	13-16 years	0.47	806.0538495	99	Grand Index
40	f	13-16 years	0.473	807	99	Grand Index
40	f	13-16 years	0.479	808	99	Grand Index
40	f	13-16 years	0.48	808.0595095	99	Grand Index
40	f	13-16 years	0.482	809	99	Grand Index
40	f	13-16 years	0.483	809.0297808	99	Grand Index
40	f	13-16 years	0.484	809.9488937	99	Grand Index
40	f	13-16 years	0.489	810	100	Grand Index
40	f	13-16 years	0.49	810.4856284	100	Grand Index
40	f	13-16 years	0.498	811	100	Grand Index
40	f	13-16 years	0.504	812	100	Grand Index
40	f	13-16 years	0.509	813	100	Grand Index
40	f	13-16 years	0.51	813.0408863	100	Grand Index
40	f	13-16 years	0.511	813.3107192	100	Grand Index
40	f	13-16 years	0.512	813.9334063	100	Grand Index
40	f	13-16 years	0.517	814	101	Grand Index
40	f	13-16 years	0.518	814.7827273	101	Grand Index
40	f	13-16 years	0.523	815	101	Grand Index
40	f	13-16 years	0.524	815.0919451	101	Grand Index
40	f	13-16 years	0.525	815.9113197	101	Grand Index
40	f	13-16 years	0.528	816	101	Grand Index
40	f	13-16 years	0.532	817	101	Grand Index
40	f	13-16 years	0.534	818	101	Grand Index
40	f	13-16 years	0.535	818.7912297	101	Grand Index
40	f	13-16 years	0.537	819	101	Grand Index
40	f	13-16 years	0.538	819.0795165	101	Grand Index
40	f	13-16 years	0.543	820	102	Grand Index
40	f	13-16 years	0.544	820.776	102	Grand Index
40	f	13-16 years	0.549	821	102	Grand Index

Age	Gender	Education	Percentile	Raw Score	Norm Score	Assessment
40	f	13-16 years	0.556	822	102	Grand Index
40	f	13-16 years	0.557	822.3964158	102	Grand Index
40	f	13-16 years	0.561	823	102	Grand Index
40	f	13-16 years	0.565	824	102	Grand Index
40	f	13-16 years	0.568	825	103	Grand Index
40	f	13-16 years	0.571	826	103	Grand Index
40	f	13-16 years	0.572	826.5388476	103	Grand Index
40	f	13-16 years	0.577	827	103	Grand Index
40	f	13-16 years	0.578	827.1681603	103	Grand Index
40	f	13-16 years	0.58	828	103	Grand Index
40	f	13-16 years	0.581	828.099	103	Grand Index
40	f	13-16 years	0.585	829	103	Grand Index
40	f	13-16 years	0.586	829.6416658	103	Grand Index
40	f	13-16 years	0.59	830	103	Grand Index
40	f	13-16 years	0.591	830.889	103	Grand Index
40	f	13-16 years	0.593	831	104	Grand Index
40	f	13-16 years	0.594	831.897508	104	Grand Index
40	f	13-16 years	0.599	832	104	Grand Index
40	f	13-16 years	0.6	832.1187462	104	Grand Index
40	f	13-16 years	0.605	833	104	Grand Index
40	f	13-16 years	0.606	833.0359696	104	Grand Index
40	f	13-16 years	0.607	833.857384	104	Grand Index
40	f	13-16 years	0.612	834	104	Grand Index
40	f	13-16 years	0.613	834.0573091	104	Grand Index
40	f	13-16 years	0.614	834.7467194	104	Grand Index
40	f	13-16 years	0.616	835	104	Grand Index
40	f	13-16 years	0.617	835.0068147	104	Grand Index
40	f	13-16 years	0.621	836	105	Grand Index
40	f	13-16 years	0.622	836.723352	105	Grand Index
40	f	13-16 years	0.624	837	105	Grand Index
40	f	13-16 years	0.625	837.0120216	105	Grand Index
40	f	13-16 years	0.63	838	105	Grand Index
40	f	13-16 years	0.635	839	105	Grand Index
40	f	13-16 years	0.636	839.444	105	Grand Index
40	f	13-16 years	0.64	840	105	Grand Index
40	f	13-16 years	0.641	840.825904	105	Grand Index
40	f	13-16 years	0.645	841	106	Grand Index
40	f	13-16 years	0.646	841.0253557	106	Grand Index
40	f	13-16 years	0.649	842	106	Grand Index
40	f	13-16 years	0.65	842.1706761	106	Grand Index
40	f	13-16 years	0.651	842.846035	106	Grand Index
40	f	13-16 years	0.654	843	106	Grand Index
40	f	13-16 years	0.655	843.855217	106	Grand Index
40	f	13-16 years	0.657	844	106	Grand Index
40	f	13-16 years	0.662	845	106	Grand Index
40	f	13-16 years	0.663	845.977857	106	Grand Index
40	f	13-16 years	0.666	846	106	Grand Index
40	f	13-16 years	0.667	846.884578	106	Grand Index
40	f	13-16 years	0.67	847	107	Grand Index
40	f	13-16 years	0.671	847.209	107	Grand Index
40	f	13-16 years	0.677	848	107	Grand Index
40	f	13-16 years	0.681	849	107	Grand Index
40	f	13-16 years	0.682	849.0812808	107	Grand Index
40	f	13-16 years	0.683	849.8775245	107	Grand Index
40	f	13-16 years	0.688	850	107	Grand Index
40	f	13-16 years	0.689	850.2404191	107	Grand Index

Age	Gender	Education	Percentile	Raw Score	Norm Score	Assessment
40	f	13-16 years	0.691	851	107	Grand Index
40	f	13-16 years	0.696	852	108	Grand Index
40	f	13-16 years	0.697	852.1434126	108	Grand Index
40	f	13-16 years	0.701	853	108	Grand Index
40	f	13-16 years	0.702	853.9064559	108	Grand Index
40	f	13-16 years	0.705	854	108	Grand Index
40	f	13-16 years	0.706	854.6466176	108	Grand Index
40	f	13-16 years	0.71	855	108	Grand Index
40	f	13-16 years	0.711	855.5236897	108	Grand Index
40	f	13-16 years	0.715	856	109	Grand Index
40	f	13-16 years	0.716	856.0827854	109	Grand Index
40	f	13-16 years	0.717	856.3603873	109	Grand Index
40	f	13-16 years	0.723	857	109	Grand Index
40	f	13-16 years	0.724	857.996	109	Grand Index
40	f	13-16 years	0.73	858	109	Grand Index
40	f	13-16 years	0.733	859	109	Grand Index
40	f	13-16 years	0.734	859.3080754	109	Grand Index
40	f	13-16 years	0.737	860	110	Grand Index
40	f	13-16 years	0.738	860.9376152	110	Grand Index
40	f	13-16 years	0.741	861	110	Grand Index
40	f	13-16 years	0.747	862	110	Grand Index
40	f	13-16 years	0.748	862.692	110	Grand Index
40	f	13-16 years	0.753	863	110	Grand Index
40	f	13-16 years	0.754	863.8145454	110	Grand Index
40	f	13-16 years	0.757	864	110	Grand Index
40	f	13-16 years	0.758	864.2086392	110	Grand Index
40	f	13-16 years	0.762	865	111	Grand Index
40	f	13-16 years	0.763	865.877	111	Grand Index
40	f	13-16 years	0.765	866	111	Grand Index
40	f	13-16 years	0.766	866.5952355	111	Grand Index
40	f	13-16 years	0.769	867	111	Grand Index
40	f	13-16 years	0.77	867.966849	111	Grand Index
40	f	13-16 years	0.774	868	111	Grand Index
40	f	13-16 years	0.775	868.8706125	111	Grand Index
40	f	13-16 years	0.777	869	111	Grand Index
40	f	13-16 years	0.778	869.2301892	111	Grand Index
40	f	13-16 years	0.783	870	112	Grand Index
40	f	13-16 years	0.784	870.5812303	112	Grand Index
40	f	13-16 years	0.787	871	112	Grand Index
40	f	13-16 years	0.788	871.3240383	112	Grand Index
40	f	13-16 years	0.79	872	112	Grand Index
40	f	13-16 years	0.791	872.6648784	112	Grand Index
40	f	13-16 years	0.794	873	112	Grand Index
40	f	13-16 years	0.795	873.4179442	112	Grand Index
40	f	13-16 years	0.799	874	113	Grand Index
40	f	13-16 years	0.8	874.2973807	113	Grand Index
40	f	13-16 years	0.801	874.9912031	113	Grand Index
40	f	13-16 years	0.803	875	113	Grand Index
40	f	13-16 years	0.804	875.2789912	113	Grand Index
40	f	13-16 years	0.806	876	113	Grand Index
40	f	13-16 years	0.807	877	113	Grand Index
40	f	13-16 years	0.808	877.1910495	113	Grand Index
40	f	13-16 years	0.809	877.9572537	113	Grand Index
40	f	13-16 years	0.811	878	113	Grand Index
40	f	13-16 years	0.812	878.548	113	Grand Index
40	f	13-16 years	0.815	879	113	Grand Index

Age	Gender	Education	Percentile	Raw Score	Norm Score	Assessment
40	f	13-16 years	0.817	880	114	Grand Index
40	f	13-16 years	0.82	881	114	Grand Index
40	f	13-16 years	0.823	882	114	Grand Index
40	f	13-16 years	0.824	882.9693148	114	Grand Index
40	f	13-16 years	0.825	883	114	Grand Index
40	f	13-16 years	0.829	884	114	Grand Index
40	f	13-16 years	0.83	884.7742094	114	Grand Index
40	f	13-16 years	0.831	885	114	Grand Index
40	f	13-16 years	0.832	885.0719109	114	Grand Index
40	f	13-16 years	0.833	885.9567859	114	Grand Index
40	f	13-16 years	0.835	886	115	Grand Index
40	f	13-16 years	0.838	887	115	Grand Index
40	f	13-16 years	0.839	887.1086574	115	Grand Index
40	f	13-16 years	0.842	888	115	Grand Index
40	f	13-16 years	0.845	889	115	Grand Index
40	f	13-16 years	0.846	889.1687106	115	Grand Index
40	f	13-16 years	0.848	890	115	Grand Index
40	f	13-16 years	0.849	890.6133744	115	Grand Index
40	f	13-16 years	0.85	891	116	Grand Index
40	f	13-16 years	0.851	891.0819224	116	Grand Index
40	f	13-16 years	0.852	891.8432395	116	Grand Index
40	f	13-16 years	0.853	891.9998235	116	Grand Index
40	f	13-16 years	0.854	892	116	Grand Index
40	f	13-16 years	0.855	892.545	116	Grand Index
40	f	13-16 years	0.857	893	116	Grand Index
40	f	13-16 years	0.858	893.382	116	Grand Index
40	f	13-16 years	0.861	894	116	Grand Index
40	f	13-16 years	0.862	894.0304902	116	Grand Index
40	f	13-16 years	0.864	895	116	Grand Index
40	f	13-16 years	0.865	895.335	117	Grand Index
40	f	13-16 years	0.866	896	117	Grand Index
40	f	13-16 years	0.867	896.8311371	117	Grand Index
40	f	13-16 years	0.868	897.172	117	Grand Index
40	f	13-16 years	0.869	898	117	Grand Index
40	f	13-16 years	0.87	898.0372219	117	Grand Index
40	f	13-16 years	0.871	899.0088593	117	Grand Index
40	f	13-16 years	0.874	900	117	Grand Index
40	f	13-16 years	0.875	901.125	117	Grand Index
40	f	13-16 years	0.876	902	117	Grand Index
40	f	13-16 years	0.877	902.0647024	117	Grand Index
40	f	13-16 years	0.878	902.998794	117	Grand Index
40	f	13-16 years	0.881	903	118	Grand Index
40	f	13-16 years	0.882	903.0660606	118	Grand Index
40	f	13-16 years	0.884	904	118	Grand Index
40	f	13-16 years	0.885	904.3954499	118	Grand Index
40	f	13-16 years	0.888	905	118	Grand Index
40	f	13-16 years	0.889	905.0278455	118	Grand Index
40	f	13-16 years	0.891	906	118	Grand Index
40	f	13-16 years	0.892	906.5394315	119	Grand Index
40	f	13-16 years	0.893	907	119	Grand Index
40	f	13-16 years	0.894	907.5017616	119	Grand Index
40	f	13-16 years	0.895	908	119	Grand Index
40	f	13-16 years	0.896	909.177621	119	Grand Index
40	f	13-16 years	0.897	910.0672762	119	Grand Index
40	f	13-16 years	0.898	910.9746549	119	Grand Index
40	f	13-16 years	0.899	911.006723	119	Grand Index

Age	Gender	Education	Percentile	Raw Score	Norm Score	Assessment
40	f	13-16 years	0.901	912	119	Grand Index
40	f	13-16 years	0.902	912.658	119	Grand Index
40	f	13-16 years	0.903	913	119	Grand Index
40	f	13-16 years	0.904	913.8544152	120	Grand Index
40	f	13-16 years	0.906	914	120	Grand Index
40	f	13-16 years	0.908	915	120	Grand Index
40	f	13-16 years	0.909	915.611	120	Grand Index
40	f	13-16 years	0.91	916	120	Grand Index
40	f	13-16 years	0.912	917	120	Grand Index
40	f	13-16 years	0.913	917.011843	120	Grand Index
40	f	13-16 years	0.914	918	120	Grand Index
40	f	13-16 years	0.915	918.711326	121	Grand Index
40	f	13-16 years	0.916	919	121	Grand Index
40	f	13-16 years	0.917	919.8034327	121	Grand Index
40	f	13-16 years	0.919	920	121	Grand Index
40	f	13-16 years	0.92	920.4992648	121	Grand Index
40	f	13-16 years	0.921	921	121	Grand Index
40	f	13-16 years	0.922	921.9495015	121	Grand Index
40	f	13-16 years	0.923	922	121	Grand Index
40	f	13-16 years	0.924	922.796	121	Grand Index
40	f	13-16 years	0.925	923.075	122	Grand Index
40	f	13-16 years	0.926	924.354	122	Grand Index
40	f	13-16 years	0.927	925	122	Grand Index
40	f	13-16 years	0.928	925.9637278	122	Grand Index
40	f	13-16 years	0.929	927	122	Grand Index
40	f	13-16 years	0.93	927.0424114	122	Grand Index
40	f	13-16 years	0.931	928.0030198	122	Grand Index
40	f	13-16 years	0.932	929	122	Grand Index
40	f	13-16 years	0.933	929.2984027	122	Grand Index
40	f	13-16 years	0.934	930	123	Grand Index
40	f	13-16 years	0.935	930.9576588	123	Grand Index
40	f	13-16 years	0.936	931	123	Grand Index
40	f	13-16 years	0.937	931.4404448	123	Grand Index
40	f	13-16 years	0.938	932	123	Grand Index
40	f	13-16 years	0.939	933	123	Grand Index
40	f	13-16 years	0.94	934	123	Grand Index
40	f	13-16 years	0.941	934.0002679	123	Grand Index
40	f	13-16 years	0.942	934.8706688	124	Grand Index
40	f	13-16 years	0.943	935.0750704	124	Grand Index
40	f	13-16 years	0.944	936.376	124	Grand Index
40	f	13-16 years	0.945	937	124	Grand Index
40	f	13-16 years	0.946	938.9122902	124	Grand Index
40	f	13-16 years	0.947	939	124	Grand Index
40	f	13-16 years	0.949	940	125	Grand Index
40	f	13-16 years	0.95	940.8795328	125	Grand Index
40	f	13-16 years	0.951	941	125	Grand Index
40	f	13-16 years	0.952	941.3227176	125	Grand Index
40	f	13-16 years	0.953	942	125	Grand Index
40	f	13-16 years	0.954	942.166	125	Grand Index
40	f	13-16 years	0.955	944	125	Grand Index
40	f	13-16 years	0.956	945.2778462	126	Grand Index
40	f	13-16 years	0.957	946.9162604	126	Grand Index
40	f	13-16 years	0.959	947	126	Grand Index
40	f	13-16 years	0.961	948	126	Grand Index
40	f	13-16 years	0.962	948.0109881	127	Grand Index
40	f	13-16 years	0.963	948.9974574	127	Grand Index

Age	Gender	Education	Percentile	Raw Score	Norm Score	Assessment
40	f	13-16 years	0.964	949.9569474	127	Grand Index
40	f	13-16 years	0.965	951	127	Grand Index
40	f	13-16 years	0.966	951.8640743	127	Grand Index
40	f	13-16 years	0.967	952	128	Grand Index
40	f	13-16 years	0.969	954	128	Grand Index
40	f	13-16 years	0.97	955.6374042	128	Grand Index
40	f	13-16 years	0.971	958	128	Grand Index
40	f	13-16 years	0.972	959	129	Grand Index
40	f	13-16 years	0.973	959.8138354	129	Grand Index
40	f	13-16 years	0.974	960.9176883	129	Grand Index
40	f	13-16 years	0.975	961.0019488	129	Grand Index
40	f	13-16 years	0.976	961.4120415	130	Grand Index
40	f	13-16 years	0.977	962.3136214	130	Grand Index
40	f	13-16 years	0.978	965.9078564	130	Grand Index
40	f	13-16 years	0.979	967.2393568	131	Grand Index
40	f	13-16 years	0.98	969.84	131	Grand Index
40	f	13-16 years	0.981	971.4370206	131	Grand Index
40	f	13-16 years	0.982	974.1228741	131	Grand Index
40	f	13-16 years	0.983	977	132	Grand Index
40	f	13-16 years	0.984	979	132	Grand Index
40	f	13-16 years	0.985	981.815	133	Grand Index
40	f	13-16 years	0.986	983.3200895	133	Grand Index
40	f	13-16 years	0.987	985.7777629	133	Grand Index
40	f	13-16 years	0.988	987	134	Grand Index
40	f	13-16 years	0.989	988	134	Grand Index
40	f	13-16 years	0.99	991.7762851	135	Grand Index
40	f	13-16 years	0.991	997.5452994	135	Grand Index
40	f	13-16 years	0.992	999	136	Grand Index
40	f	13-16 years	0.993	1002.596622	137	Grand Index
40	f	13-16 years	0.994	1004.652	138	Grand Index
40	f	13-16 years	0.995	1008.42	139	Grand Index
40	f	13-16 years	0.996	1015.095919	140	Grand Index
40	f	13-16 years	0.997	1017.087101	141	Grand Index
40	f	13-16 years	0.998	1021.097595	143	Grand Index
40	f	13-16 years	0.999	1025.282007	146	Grand Index
40	m	13-16 years	0.001	512.887643	54	Grand Index
40	m	13-16 years	0.002	580.0022094	57	Grand Index
40	m	13-16 years	0.003	605.0587592	59	Grand Index
40	m	13-16 years	0.004	607.0310099	60	Grand Index
40	m	13-16 years	0.005	610.27	61	Grand Index
40	m	13-16 years	0.006	615.9374923	62	Grand Index
40	m	13-16 years	0.007	626.0757863	63	Grand Index
40	m	13-16 years	0.008	628.144	64	Grand Index
40	m	13-16 years	0.009	633.1018949	65	Grand Index
40	m	13-16 years	0.01	639	65	Grand Index
40	m	13-16 years	0.011	640.134268	66	Grand Index
40	m	13-16 years	0.012	641.5446783	66	Grand Index
40	m	13-16 years	0.013	644.958636	67	Grand Index
40	m	13-16 years	0.014	646.0722264	67	Grand Index
40	m	13-16 years	0.015	648.684551	67	Grand Index
40	m	13-16 years	0.016	652.1028826	68	Grand Index
40	m	13-16 years	0.017	653.4296069	68	Grand Index
40	m	13-16 years	0.018	657.324	69	Grand Index
40	m	13-16 years	0.019	660.1375795	69	Grand Index
40	m	13-16 years	0.02	662.1421124	69	Grand Index
40	m	13-16 years	0.021	663.756	69	Grand Index

Age	Gender	Education	Percentile	Raw Score	Norm Score	Assessment
40	m	13-16 years	0.022	665.396	70	Grand Index
40	m	13-16 years	0.023	667.0087568	70	Grand Index
40	m	13-16 years	0.024	667.7003822	70	Grand Index
40	m	13-16 years	0.025	668	71	Grand Index
40	m	13-16 years	0.026	669.2944265	71	Grand Index
40	m	13-16 years	0.028	672	71	Grand Index
40	m	13-16 years	0.029	672.5877751	72	Grand Index
40	m	13-16 years	0.03	674.4092246	72	Grand Index
40	m	13-16 years	0.031	675	72	Grand Index
40	m	13-16 years	0.032	675.576	72	Grand Index
40	m	13-16 years	0.033	676	72	Grand Index
40	m	13-16 years	0.034	676.5315128	73	Grand Index
40	m	13-16 years	0.035	676.9968876	73	Grand Index
40	m	13-16 years	0.036	677.3657434	73	Grand Index
40	m	13-16 years	0.037	678	73	Grand Index
40	m	13-16 years	0.038	678.0432523	73	Grand Index
40	m	13-16 years	0.039	678.9951912	74	Grand Index
40	m	13-16 years	0.04	679.9109581	74	Grand Index
40	m	13-16 years	0.041	680.4511018	74	Grand Index
40	m	13-16 years	0.042	680.9927723	74	Grand Index
40	m	13-16 years	0.043	681.6709366	74	Grand Index
40	m	13-16 years	0.044	681.9561544	74	Grand Index
40	m	13-16 years	0.045	682	75	Grand Index
40	m	13-16 years	0.046	682.8650304	75	Grand Index
40	m	13-16 years	0.047	683	75	Grand Index
40	m	13-16 years	0.048	684.864	75	Grand Index
40	m	13-16 years	0.049	685	75	Grand Index
40	m	13-16 years	0.05	685.0118696	75	Grand Index
40	m	13-16 years	0.051	685.9609629	75	Grand Index
40	m	13-16 years	0.052	686.6617152	76	Grand Index
40	m	13-16 years	0.053	687.954	76	Grand Index
40	m	13-16 years	0.054	688.972	76	Grand Index
40	m	13-16 years	0.055	689	76	Grand Index
40	m	13-16 years	0.056	690.008	76	Grand Index
40	m	13-16 years	0.057	691	76	Grand Index
40	m	13-16 years	0.058	691.504463	76	Grand Index
40	m	13-16 years	0.06	692	77	Grand Index
40	m	13-16 years	0.061	692.8097814	77	Grand Index
40	m	13-16 years	0.062	693.1145828	77	Grand Index
40	m	13-16 years	0.063	694	77	Grand Index
40	m	13-16 years	0.064	694.8053516	77	Grand Index
40	m	13-16 years	0.065	695.3570666	77	Grand Index
40	m	13-16 years	0.067	696	78	Grand Index
40	m	13-16 years	0.068	696.7090254	78	Grand Index
40	m	13-16 years	0.069	697.5833389	78	Grand Index
40	m	13-16 years	0.07	698.7551665	78	Grand Index
40	m	13-16 years	0.071	701	78	Grand Index
40	m	13-16 years	0.072	701.7687012	78	Grand Index
40	m	13-16 years	0.073	702.8233142	78	Grand Index
40	m	13-16 years	0.074	703.332	78	Grand Index
40	m	13-16 years	0.075	704	78	Grand Index
40	m	13-16 years	0.076	704.1531249	79	Grand Index
40	m	13-16 years	0.077	704.8374468	79	Grand Index
40	m	13-16 years	0.078	705.4001855	79	Grand Index
40	m	13-16 years	0.081	706	79	Grand Index
40	m	13-16 years	0.082	706.6391777	79	Grand Index

Age	Gender	Education	Percentile	Raw Score	Norm Score	Assessment
40	m	13-16 years	0.084	707	79	Grand Index
40	m	13-16 years	0.085	707.8405512	79	Grand Index
40	m	13-16 years	0.086	708.4753188	80	Grand Index
40	m	13-16 years	0.087	709	80	Grand Index
40	m	13-16 years	0.088	710.20002	80	Grand Index
40	m	13-16 years	0.089	711	80	Grand Index
40	m	13-16 years	0.09	711.6201185	80	Grand Index
40	m	13-16 years	0.091	712	80	Grand Index
40	m	13-16 years	0.092	712.4195227	80	Grand Index
40	m	13-16 years	0.093	712.9153474	80	Grand Index
40	m	13-16 years	0.094	713.5570454	80	Grand Index
40	m	13-16 years	0.095	714	80	Grand Index
40	m	13-16 years	0.096	714.7065733	80	Grand Index
40	m	13-16 years	0.098	715	81	Grand Index
40	m	13-16 years	0.1	716	81	Grand Index
40	m	13-16 years	0.101	717	81	Grand Index
40	m	13-16 years	0.102	717.5015341	81	Grand Index
40	m	13-16 years	0.103	718	81	Grand Index
40	m	13-16 years	0.104	718.7119063	81	Grand Index
40	m	13-16 years	0.105	718.9997736	81	Grand Index
40	m	13-16 years	0.106	719	81	Grand Index
40	m	13-16 years	0.107	719.926	81	Grand Index
40	m	13-16 years	0.109	720	82	Grand Index
40	m	13-16 years	0.11	720.98	82	Grand Index
40	m	13-16 years	0.111	721	82	Grand Index
40	m	13-16 years	0.112	721.0049916	82	Grand Index
40	m	13-16 years	0.113	722	82	Grand Index
40	m	13-16 years	0.114	722.0580282	82	Grand Index
40	m	13-16 years	0.115	723.4116289	82	Grand Index
40	m	13-16 years	0.116	724	82	Grand Index
40	m	13-16 years	0.117	724.106	82	Grand Index
40	m	13-16 years	0.12	725	82	Grand Index
40	m	13-16 years	0.123	726	83	Grand Index
40	m	13-16 years	0.126	727	83	Grand Index
40	m	13-16 years	0.127	727.9577741	83	Grand Index
40	m	13-16 years	0.128	728.115296	83	Grand Index
40	m	13-16 years	0.129	728.6940571	83	Grand Index
40	m	13-16 years	0.132	729	83	Grand Index
40	m	13-16 years	0.136	730	84	Grand Index
40	m	13-16 years	0.137	730.8677856	84	Grand Index
40	m	13-16 years	0.139	731	84	Grand Index
40	m	13-16 years	0.14	731.4227204	84	Grand Index
40	m	13-16 years	0.142	732	84	Grand Index
40	m	13-16 years	0.143	732.7706178	84	Grand Index
40	m	13-16 years	0.145	733	84	Grand Index
40	m	13-16 years	0.146	733.1124299	84	Grand Index
40	m	13-16 years	0.147	734	84	Grand Index
40	m	13-16 years	0.148	734.0076606	84	Grand Index
40	m	13-16 years	0.149	734.7398913	84	Grand Index
40	m	13-16 years	0.151	735	85	Grand Index
40	m	13-16 years	0.154	736	85	Grand Index
40	m	13-16 years	0.155	736.79	85	Grand Index
40	m	13-16 years	0.157	737	85	Grand Index
40	m	13-16 years	0.158	737.8425116	85	Grand Index
40	m	13-16 years	0.161	738	85	Grand Index
40	m	13-16 years	0.162	738.9979187	85	Grand Index

Age	Gender	Education	Percentile	Raw Score	Norm Score	Assessment
40	m	13-16 years	0.163	739	85	Grand Index
40	m	13-16 years	0.164	739.2929343	85	Grand Index
40	m	13-16 years	0.165	739.9995452	85	Grand Index
40	m	13-16 years	0.167	740	86	Grand Index
40	m	13-16 years	0.168	740.0210295	86	Grand Index
40	m	13-16 years	0.172	741	86	Grand Index
40	m	13-16 years	0.173	742	86	Grand Index
40	m	13-16 years	0.174	742.132	86	Grand Index
40	m	13-16 years	0.176	743	86	Grand Index
40	m	13-16 years	0.177	744	86	Grand Index
40	m	13-16 years	0.178	744.204	86	Grand Index
40	m	13-16 years	0.18	745	86	Grand Index
40	m	13-16 years	0.181	745.9160787	86	Grand Index
40	m	13-16 years	0.185	746	87	Grand Index
40	m	13-16 years	0.186	746.348	87	Grand Index
40	m	13-16 years	0.189	747	87	Grand Index
40	m	13-16 years	0.19	748	87	Grand Index
40	m	13-16 years	0.194	749	87	Grand Index
40	m	13-16 years	0.195	749.51	87	Grand Index
40	m	13-16 years	0.196	750	87	Grand Index
40	m	13-16 years	0.197	750.7298358	87	Grand Index
40	m	13-16 years	0.201	751	87	Grand Index
40	m	13-16 years	0.202	751.619575	87	Grand Index
40	m	13-16 years	0.203	751.9948768	88	Grand Index
40	m	13-16 years	0.207	752	88	Grand Index
40	m	13-16 years	0.208	752.2599581	88	Grand Index
40	m	13-16 years	0.21	753	88	Grand Index
40	m	13-16 years	0.211	753.2397559	88	Grand Index
40	m	13-16 years	0.215	754	88	Grand Index
40	m	13-16 years	0.216	754.9129435	88	Grand Index
40	m	13-16 years	0.218	755	88	Grand Index
40	m	13-16 years	0.219	755.5409441	88	Grand Index
40	m	13-16 years	0.223	756	89	Grand Index
40	m	13-16 years	0.224	756.0025597	89	Grand Index
40	m	13-16 years	0.225	756.205534	89	Grand Index
40	m	13-16 years	0.229	757	89	Grand Index
40	m	13-16 years	0.23	757.14	89	Grand Index
40	m	13-16 years	0.234	758	89	Grand Index
40	m	13-16 years	0.235	758.9273124	89	Grand Index
40	m	13-16 years	0.238	759	89	Grand Index
40	m	13-16 years	0.239	759.668441	89	Grand Index
40	m	13-16 years	0.24	759.8864683	89	Grand Index
40	m	13-16 years	0.242	760	90	Grand Index
40	m	13-16 years	0.247	761	90	Grand Index
40	m	13-16 years	0.248	761.0020397	90	Grand Index
40	m	13-16 years	0.249	761.5143078	90	Grand Index
40	m	13-16 years	0.253	762	90	Grand Index
40	m	13-16 years	0.254	762.4651799	90	Grand Index
40	m	13-16 years	0.258	763	90	Grand Index
40	m	13-16 years	0.259	763.9515273	90	Grand Index
40	m	13-16 years	0.263	764	90	Grand Index
40	m	13-16 years	0.264	764.9387478	91	Grand Index
40	m	13-16 years	0.265	765	91	Grand Index
40	m	13-16 years	0.266	765.788	91	Grand Index
40	m	13-16 years	0.269	766	91	Grand Index
40	m	13-16 years	0.27	766.5363189	91	Grand Index

Age	Gender	Education	Percentile	Raw Score	Norm Score	Assessment
40	m	13-16 years	0.273	767	91	Grand Index
40	m	13-16 years	0.278	768	91	Grand Index
40	m	13-16 years	0.279	768.022	91	Grand Index
40	m	13-16 years	0.28	769	91	Grand Index
40	m	13-16 years	0.286	770	92	Grand Index
40	m	13-16 years	0.287	770.7905069	92	Grand Index
40	m	13-16 years	0.29	771	92	Grand Index
40	m	13-16 years	0.291	771.0451138	92	Grand Index
40	m	13-16 years	0.294	772	92	Grand Index
40	m	13-16 years	0.295	772.3535008	92	Grand Index
40	m	13-16 years	0.3	773	92	Grand Index
40	m	13-16 years	0.301	773.5595661	92	Grand Index
40	m	13-16 years	0.306	774	92	Grand Index
40	m	13-16 years	0.307	774.4562498	92	Grand Index
40	m	13-16 years	0.311	775	93	Grand Index
40	m	13-16 years	0.312	775.616	93	Grand Index
40	m	13-16 years	0.318	776	93	Grand Index
40	m	13-16 years	0.319	776.9739802	93	Grand Index
40	m	13-16 years	0.323	777	93	Grand Index
40	m	13-16 years	0.324	777.832	93	Grand Index
40	m	13-16 years	0.326	778	93	Grand Index
40	m	13-16 years	0.327	778.886	93	Grand Index
40	m	13-16 years	0.33	779	93	Grand Index
40	m	13-16 years	0.331	779.9890951	93	Grand Index
40	m	13-16 years	0.335	780	94	Grand Index
40	m	13-16 years	0.336	780.1740829	94	Grand Index
40	m	13-16 years	0.339	781	94	Grand Index
40	m	13-16 years	0.345	782	94	Grand Index
40	m	13-16 years	0.346	782.0050584	94	Grand Index
40	m	13-16 years	0.351	783	94	Grand Index
40	m	13-16 years	0.352	783.7341786	94	Grand Index
40	m	13-16 years	0.356	784	94	Grand Index
40	m	13-16 years	0.357	784.6778995	95	Grand Index
40	m	13-16 years	0.361	785	95	Grand Index
40	m	13-16 years	0.362	785.1144585	95	Grand Index
40	m	13-16 years	0.363	785.8445899	95	Grand Index
40	m	13-16 years	0.366	786	95	Grand Index
40	m	13-16 years	0.371	787	95	Grand Index
40	m	13-16 years	0.374	788	95	Grand Index
40	m	13-16 years	0.375	788.4074092	95	Grand Index
40	m	13-16 years	0.376	788.9521753	95	Grand Index
40	m	13-16 years	0.382	789	95	Grand Index
40	m	13-16 years	0.383	789.894	96	Grand Index
40	m	13-16 years	0.384	790	96	Grand Index
40	m	13-16 years	0.389	791	96	Grand Index
40	m	13-16 years	0.39	791.02	96	Grand Index
40	m	13-16 years	0.395	792	96	Grand Index
40	m	13-16 years	0.396	792.0972489	96	Grand Index
40	m	13-16 years	0.397	792.8076651	96	Grand Index
40	m	13-16 years	0.403	793	96	Grand Index
40	m	13-16 years	0.404	793.221556	96	Grand Index
40	m	13-16 years	0.409	794	97	Grand Index
40	m	13-16 years	0.41	794.38	97	Grand Index
40	m	13-16 years	0.413	795	97	Grand Index
40	m	13-16 years	0.414	796	97	Grand Index
40	m	13-16 years	0.415	796.47	97	Grand Index

Age	Gender	Education	Percentile	Raw Score	Norm Score	Assessment
40	m	13-16 years	0.416	797	97	Grand Index
40	m	13-16 years	0.417	797.506	97	Grand Index
40	m	13-16 years	0.421	798	97	Grand Index
40	m	13-16 years	0.425	799	97	Grand Index
40	m	13-16 years	0.433	800	97	Grand Index
40	m	13-16 years	0.438	801	98	Grand Index
40	m	13-16 years	0.443	802	98	Grand Index
40	m	13-16 years	0.444	802.6137731	98	Grand Index
40	m	13-16 years	0.448	803	98	Grand Index
40	m	13-16 years	0.449	803.1105032	98	Grand Index
40	m	13-16 years	0.45	803.7920183	98	Grand Index
40	m	13-16 years	0.454	804	98	Grand Index
40	m	13-16 years	0.455	804.19	98	Grand Index
40	m	13-16 years	0.461	805	99	Grand Index
40	m	13-16 years	0.462	805.316	99	Grand Index
40	m	13-16 years	0.467	806	99	Grand Index
40	m	13-16 years	0.471	807	99	Grand Index
40	m	13-16 years	0.472	807.6521909	99	Grand Index
40	m	13-16 years	0.474	808	99	Grand Index
40	m	13-16 years	0.475	808.55	99	Grand Index
40	m	13-16 years	0.48	809	99	Grand Index
40	m	13-16 years	0.481	809.3156695	99	Grand Index
40	m	13-16 years	0.484	810	99	Grand Index
40	m	13-16 years	0.485	810.0123716	99	Grand Index
40	m	13-16 years	0.486	810.1489888	99	Grand Index
40	m	13-16 years	0.49	811	100	Grand Index
40	m	13-16 years	0.491	811.8497534	100	Grand Index
40	m	13-16 years	0.496	812	100	Grand Index
40	m	13-16 years	0.497	812.946	100	Grand Index
40	m	13-16 years	0.502	813	100	Grand Index
40	m	13-16 years	0.506	814	100	Grand Index
40	m	13-16 years	0.507	814.0367649	100	Grand Index
40	m	13-16 years	0.511	815	100	Grand Index
40	m	13-16 years	0.512	815.0131417	100	Grand Index
40	m	13-16 years	0.518	816	101	Grand Index
40	m	13-16 years	0.519	816.7803021	101	Grand Index
40	m	13-16 years	0.524	817	101	Grand Index
40	m	13-16 years	0.525	817.4231432	101	Grand Index
40	m	13-16 years	0.528	818	101	Grand Index
40	m	13-16 years	0.529	818.5143742	101	Grand Index
40	m	13-16 years	0.536	819	101	Grand Index
40	m	13-16 years	0.537	819.1555578	101	Grand Index
40	m	13-16 years	0.538	819.9877145	101	Grand Index
40	m	13-16 years	0.541	820	102	Grand Index
40	m	13-16 years	0.542	820.0670995	102	Grand Index
40	m	13-16 years	0.543	820.9878062	102	Grand Index
40	m	13-16 years	0.55	821	102	Grand Index
40	m	13-16 years	0.551	821.5922351	102	Grand Index
40	m	13-16 years	0.554	822	102	Grand Index
40	m	13-16 years	0.555	822.99	102	Grand Index
40	m	13-16 years	0.559	823	102	Grand Index
40	m	13-16 years	0.563	824	102	Grand Index
40	m	13-16 years	0.564	824.0504802	102	Grand Index
40	m	13-16 years	0.567	825	103	Grand Index
40	m	13-16 years	0.568	825.0963335	103	Grand Index
40	m	13-16 years	0.574	826	103	Grand Index

Age	Gender	Education	Percentile	Raw Score	Norm Score	Assessment
40	m	13-16 years	0.575	826.2055846	103	Grand Index
40	m	13-16 years	0.576	826.9559052	103	Grand Index
40	m	13-16 years	0.583	827	103	Grand Index
40	m	13-16 years	0.584	827.5982512	103	Grand Index
40	m	13-16 years	0.587	828	103	Grand Index
40	m	13-16 years	0.588	828.1070215	103	Grand Index
40	m	13-16 years	0.592	829	103	Grand Index
40	m	13-16 years	0.593	829.0108756	104	Grand Index
40	m	13-16 years	0.594	829.6873378	104	Grand Index
40	m	13-16 years	0.598	830	104	Grand Index
40	m	13-16 years	0.599	830.864876	104	Grand Index
40	m	13-16 years	0.603	831	104	Grand Index
40	m	13-16 years	0.604	831.2461799	104	Grand Index
40	m	13-16 years	0.605	831.7719499	104	Grand Index
40	m	13-16 years	0.607	832	104	Grand Index
40	m	13-16 years	0.608	832.4375759	104	Grand Index
40	m	13-16 years	0.61	833	104	Grand Index
40	m	13-16 years	0.611	833.9668408	104	Grand Index
40	m	13-16 years	0.617	834	104	Grand Index
40	m	13-16 years	0.618	834.124	105	Grand Index
40	m	13-16 years	0.621	835	105	Grand Index
40	m	13-16 years	0.622	835.1430946	105	Grand Index
40	m	13-16 years	0.627	836	105	Grand Index
40	m	13-16 years	0.628	836.6967814	105	Grand Index
40	m	13-16 years	0.629	837	105	Grand Index
40	m	13-16 years	0.63	837.1331959	105	Grand Index
40	m	13-16 years	0.634	838	105	Grand Index
40	m	13-16 years	0.635	838.4209027	105	Grand Index
40	m	13-16 years	0.641	839	105	Grand Index
40	m	13-16 years	0.642	839.556	105	Grand Index
40	m	13-16 years	0.648	840	106	Grand Index
40	m	13-16 years	0.649	840.1533164	106	Grand Index
40	m	13-16 years	0.65	840.8933917	106	Grand Index
40	m	13-16 years	0.656	841	106	Grand Index
40	m	13-16 years	0.657	841.6676193	106	Grand Index
40	m	13-16 years	0.661	842	106	Grand Index
40	m	13-16 years	0.662	842.0848422	106	Grand Index
40	m	13-16 years	0.663	842.8771453	106	Grand Index
40	m	13-16 years	0.666	843	106	Grand Index
40	m	13-16 years	0.668	844	107	Grand Index
40	m	13-16 years	0.669	844.115208	107	Grand Index
40	m	13-16 years	0.672	845	107	Grand Index
40	m	13-16 years	0.673	845.2449219	107	Grand Index
40	m	13-16 years	0.676	846	107	Grand Index
40	m	13-16 years	0.677	846.355435	107	Grand Index
40	m	13-16 years	0.679	847	107	Grand Index
40	m	13-16 years	0.68	847.24	107	Grand Index
40	m	13-16 years	0.682	848	107	Grand Index
40	m	13-16 years	0.683	848.0261792	107	Grand Index
40	m	13-16 years	0.688	849	107	Grand Index
40	m	13-16 years	0.689	849.9471755	107	Grand Index
40	m	13-16 years	0.69	850	107	Grand Index
40	m	13-16 years	0.691	850.0047615	107	Grand Index
40	m	13-16 years	0.694	851	108	Grand Index
40	m	13-16 years	0.695	851.51	108	Grand Index
40	m	13-16 years	0.699	852	108	Grand Index

Age	Gender	Education	Percentile	Raw Score	Norm Score	Assessment
40	m	13-16 years	0.7	852.6608526	108	Grand Index
40	m	13-16 years	0.703	853	108	Grand Index
40	m	13-16 years	0.704	853.543825	108	Grand Index
40	m	13-16 years	0.708	854	108	Grand Index
40	m	13-16 years	0.709	855.4660167	108	Grand Index
40	m	13-16 years	0.71	855.9196471	108	Grand Index
40	m	13-16 years	0.713	856	108	Grand Index
40	m	13-16 years	0.714	856.8436438	108	Grand Index
40	m	13-16 years	0.72	857	109	Grand Index
40	m	13-16 years	0.721	857.978	109	Grand Index
40	m	13-16 years	0.724	858	109	Grand Index
40	m	13-16 years	0.725	858.0008565	109	Grand Index
40	m	13-16 years	0.726	858.1864259	109	Grand Index
40	m	13-16 years	0.728	859	109	Grand Index
40	m	13-16 years	0.729	859.1200601	109	Grand Index
40	m	13-16 years	0.732	860	109	Grand Index
40	m	13-16 years	0.733	860.0018266	109	Grand Index
40	m	13-16 years	0.737	861	110	Grand Index
40	m	13-16 years	0.738	861.1038641	110	Grand Index
40	m	13-16 years	0.739	861.8972379	110	Grand Index
40	m	13-16 years	0.744	862	110	Grand Index
40	m	13-16 years	0.745	862.41	110	Grand Index
40	m	13-16 years	0.746	863.2221819	110	Grand Index
40	m	13-16 years	0.747	864	110	Grand Index
40	m	13-16 years	0.748	864.728305	110	Grand Index
40	m	13-16 years	0.751	865	110	Grand Index
40	m	13-16 years	0.752	865.2357352	110	Grand Index
40	m	13-16 years	0.753	865.8267579	110	Grand Index
40	m	13-16 years	0.757	866	110	Grand Index
40	m	13-16 years	0.76	867	111	Grand Index
40	m	13-16 years	0.761	867.6957178	111	Grand Index
40	m	13-16 years	0.764	868	111	Grand Index
40	m	13-16 years	0.765	868.136098	111	Grand Index
40	m	13-16 years	0.768	869	111	Grand Index
40	m	13-16 years	0.769	869.2394789	111	Grand Index
40	m	13-16 years	0.772	870	111	Grand Index
40	m	13-16 years	0.773	870.914	111	Grand Index
40	m	13-16 years	0.776	871	111	Grand Index
40	m	13-16 years	0.778	872	111	Grand Index
40	m	13-16 years	0.779	872.0026798	112	Grand Index
40	m	13-16 years	0.78	872.5111212	112	Grand Index
40	m	13-16 years	0.781	873	112	Grand Index
40	m	13-16 years	0.782	873.0020897	112	Grand Index
40	m	13-16 years	0.785	874	112	Grand Index
40	m	13-16 years	0.786	874.5324581	112	Grand Index
40	m	13-16 years	0.789	875	112	Grand Index
40	m	13-16 years	0.79	875.0077762	112	Grand Index
40	m	13-16 years	0.791	875.3984676	112	Grand Index
40	m	13-16 years	0.793	876	112	Grand Index
40	m	13-16 years	0.794	876.730665	112	Grand Index
40	m	13-16 years	0.796	877	112	Grand Index
40	m	13-16 years	0.799	878	113	Grand Index
40	m	13-16 years	0.801	879	113	Grand Index
40	m	13-16 years	0.802	879.3745397	113	Grand Index
40	m	13-16 years	0.803	879.9945736	113	Grand Index
40	m	13-16 years	0.805	880	113	Grand Index

Age	Gender	Education	Percentile	Raw Score	Norm Score	Assessment
40	m	13-16 years	0.808	881	113	Grand Index
40	m	13-16 years	0.809	881.562	113	Grand Index
40	m	13-16 years	0.81	882	113	Grand Index
40	m	13-16 years	0.817	884	114	Grand Index
40	m	13-16 years	0.818	884.0287088	114	Grand Index
40	m	13-16 years	0.821	885	114	Grand Index
40	m	13-16 years	0.822	885.796	114	Grand Index
40	m	13-16 years	0.824	886	114	Grand Index
40	m	13-16 years	0.825	886.7366789	114	Grand Index
40	m	13-16 years	0.828	887	114	Grand Index
40	m	13-16 years	0.829	887.9266898	114	Grand Index
40	m	13-16 years	0.83	888.7982114	114	Grand Index
40	m	13-16 years	0.832	889	114	Grand Index
40	m	13-16 years	0.833	889.1381568	114	Grand Index
40	m	13-16 years	0.835	890	115	Grand Index
40	m	13-16 years	0.836	890.2103034	115	Grand Index
40	m	13-16 years	0.837	891	115	Grand Index
40	m	13-16 years	0.838	891.6314931	115	Grand Index
40	m	13-16 years	0.841	892	115	Grand Index
40	m	13-16 years	0.843	893	115	Grand Index
40	m	13-16 years	0.844	893.192	115	Grand Index
40	m	13-16 years	0.847	894	115	Grand Index
40	m	13-16 years	0.848	894.1313409	115	Grand Index
40	m	13-16 years	0.851	895	116	Grand Index
40	m	13-16 years	0.852	895.2590985	116	Grand Index
40	m	13-16 years	0.853	895.9305083	116	Grand Index
40	m	13-16 years	0.855	896	116	Grand Index
40	m	13-16 years	0.858	897	116	Grand Index
40	m	13-16 years	0.862	898	116	Grand Index
40	m	13-16 years	0.863	898.534	116	Grand Index
40	m	13-16 years	0.865	899	117	Grand Index
40	m	13-16 years	0.866	899.0482281	117	Grand Index
40	m	13-16 years	0.867	899.751328	117	Grand Index
40	m	13-16 years	0.868	900	117	Grand Index
40	m	13-16 years	0.869	900.9955377	117	Grand Index
40	m	13-16 years	0.87	901	117	Grand Index
40	m	13-16 years	0.871	901.2966125	117	Grand Index
40	m	13-16 years	0.872	901.7683831	117	Grand Index
40	m	13-16 years	0.875	902	117	Grand Index
40	m	13-16 years	0.876	902.4108566	117	Grand Index
40	m	13-16 years	0.877	902.9959156	117	Grand Index
40	m	13-16 years	0.878	903	117	Grand Index
40	m	13-16 years	0.88	904	118	Grand Index
40	m	13-16 years	0.881	904.6604736	118	Grand Index
40	m	13-16 years	0.882	905	118	Grand Index
40	m	13-16 years	0.883	905.894	118	Grand Index
40	m	13-16 years	0.885	906	118	Grand Index
40	m	13-16 years	0.886	906.2241967	118	Grand Index
40	m	13-16 years	0.887	906.984217	118	Grand Index
40	m	13-16 years	0.888	907.2507361	118	Grand Index
40	m	13-16 years	0.889	908	118	Grand Index
40	m	13-16 years	0.89	908.0070557	118	Grand Index
40	m	13-16 years	0.891	909.005095	118	Grand Index
40	m	13-16 years	0.892	910	119	Grand Index
40	m	13-16 years	0.893	910.4827043	119	Grand Index
40	m	13-16 years	0.897	911	119	Grand Index

Age	Gender	Education	Percentile	Raw Score	Norm Score	Assessment
40	m	13-16 years	0.898	911.164	119	Grand Index
40	m	13-16 years	0.899	912	119	Grand Index
40	m	13-16 years	0.9	912.0048883	119	Grand Index
40	m	13-16 years	0.901	912.4736268	119	Grand Index
40	m	13-16 years	0.902	913	119	Grand Index
40	m	13-16 years	0.903	913.9907014	119	Grand Index
40	m	13-16 years	0.904	914	120	Grand Index
40	m	13-16 years	0.906	915	120	Grand Index
40	m	13-16 years	0.908	916	120	Grand Index
40	m	13-16 years	0.909	916.3523127	120	Grand Index
40	m	13-16 years	0.91	917	120	Grand Index
40	m	13-16 years	0.911	917.398	120	Grand Index
40	m	13-16 years	0.912	918	120	Grand Index
40	m	13-16 years	0.913	919	120	Grand Index
40	m	13-16 years	0.914	919.5791224	120	Grand Index
40	m	13-16 years	0.915	919.9755439	121	Grand Index
40	m	13-16 years	0.917	920	121	Grand Index
40	m	13-16 years	0.918	920.0980313	121	Grand Index
40	m	13-16 years	0.919	920.6631503	121	Grand Index
40	m	13-16 years	0.92	921	121	Grand Index
40	m	13-16 years	0.921	921.5616622	121	Grand Index
40	m	13-16 years	0.925	922	122	Grand Index
40	m	13-16 years	0.926	922.2294659	122	Grand Index
40	m	13-16 years	0.928	923	122	Grand Index
40	m	13-16 years	0.929	923.0073957	122	Grand Index
40	m	13-16 years	0.93	923.7468165	122	Grand Index
40	m	13-16 years	0.931	924.758	122	Grand Index
40	m	13-16 years	0.932	925	122	Grand Index
40	m	13-16 years	0.933	925.5803073	122	Grand Index
40	m	13-16 years	0.934	926.8222442	123	Grand Index
40	m	13-16 years	0.935	927.2199551	123	Grand Index
40	m	13-16 years	0.936	927.8941843	123	Grand Index
40	m	13-16 years	0.937	928.866	123	Grand Index
40	m	13-16 years	0.938	929.6448126	123	Grand Index
40	m	13-16 years	0.939	930	123	Grand Index
40	m	13-16 years	0.94	931.84	123	Grand Index
40	m	13-16 years	0.941	932.9801897	123	Grand Index
40	m	13-16 years	0.943	933	124	Grand Index
40	m	13-16 years	0.944	933.992	124	Grand Index
40	m	13-16 years	0.945	936.009555	124	Grand Index
40	m	13-16 years	0.947	937	124	Grand Index
40	m	13-16 years	0.948	938	124	Grand Index
40	m	13-16 years	0.949	938.082	125	Grand Index
40	m	13-16 years	0.95	939	125	Grand Index
40	m	13-16 years	0.951	939.0009922	125	Grand Index
40	m	13-16 years	0.952	939.547374	125	Grand Index
40	m	13-16 years	0.953	940.3392586	125	Grand Index
40	m	13-16 years	0.954	941	125	Grand Index
40	m	13-16 years	0.955	941.4143743	125	Grand Index
40	m	13-16 years	0.957	942	126	Grand Index
40	m	13-16 years	0.958	943	126	Grand Index
40	m	13-16 years	0.959	943.2312273	126	Grand Index
40	m	13-16 years	0.96	944	126	Grand Index
40	m	13-16 years	0.961	945.2097418	126	Grand Index
40	m	13-16 years	0.962	946.0787182	127	Grand Index
40	m	13-16 years	0.963	947	127	Grand Index

Age	Gender	Education	Percentile	Raw Score	Norm Score	Assessment
40	m	13-16 years	0.964	947.1849375	127	Grand Index
40	m	13-16 years	0.965	948	127	Grand Index
40	m	13-16 years	0.966	949.3455612	127	Grand Index
40	m	13-16 years	0.967	949.8669324	128	Grand Index
40	m	13-16 years	0.968	950.424	128	Grand Index
40	m	13-16 years	0.969	951.4363452	128	Grand Index
40	m	13-16 years	0.97	953.38	128	Grand Index
40	m	13-16 years	0.971	955.4964597	128	Grand Index
40	m	13-16 years	0.972	956.8809644	129	Grand Index
40	m	13-16 years	0.973	959.028	129	Grand Index
40	m	13-16 years	0.974	960.7215048	129	Grand Index
40	m	13-16 years	0.975	962	129	Grand Index
40	m	13-16 years	0.976	963.6282272	130	Grand Index
40	m	13-16 years	0.977	964	130	Grand Index
40	m	13-16 years	0.978	965.604	130	Grand Index
40	m	13-16 years	0.979	966.3393941	131	Grand Index
40	m	13-16 years	0.98	968	131	Grand Index
40	m	13-16 years	0.981	970.5846141	131	Grand Index
40	m	13-16 years	0.982	972.676	131	Grand Index
40	m	13-16 years	0.983	973.2989204	132	Grand Index
40	m	13-16 years	0.984	976.424	132	Grand Index
40	m	13-16 years	0.985	977.9894801	133	Grand Index
40	m	13-16 years	0.986	980.6292933	133	Grand Index
40	m	13-16 years	0.987	982.9613819	133	Grand Index
40	m	13-16 years	0.988	983.8139703	134	Grand Index
40	m	13-16 years	0.989	984.9364196	134	Grand Index
40	m	13-16 years	0.99	987	135	Grand Index
40	m	13-16 years	0.991	990.8015764	135	Grand Index
40	m	13-16 years	0.992	994.7312435	136	Grand Index
40	m	13-16 years	0.993	1002.244	137	Grand Index
40	m	13-16 years	0.994	1003.077742	138	Grand Index
40	m	13-16 years	0.995	1005.757448	139	Grand Index
40	m	13-16 years	0.996	1007.012388	140	Grand Index
40	m	13-16 years	0.997	1025.462738	141	Grand Index
40	m	13-16 years	0.998	1046.568	143	Grand Index
40	m	13-16 years	0.999	1049.027657	146	Grand Index
40	f	17+ years	0.001	586.9371038	54	Grand Index
40	f	17+ years	0.002	600	57	Grand Index
40	f	17+ years	0.003	603.500811	59	Grand Index
40	f	17+ years	0.004	607.2101992	60	Grand Index
40	f	17+ years	0.005	609.0183939	61	Grand Index
40	f	17+ years	0.006	613.0391794	62	Grand Index
40	f	17+ years	0.007	619.4471966	63	Grand Index
40	f	17+ years	0.008	621.0780634	64	Grand Index
40	f	17+ years	0.009	639.5484103	65	Grand Index
40	f	17+ years	0.01	641.12	65	Grand Index
40	f	17+ years	0.011	644.332	66	Grand Index
40	f	17+ years	0.012	646.2827379	66	Grand Index
40	f	17+ years	0.013	649.6388163	67	Grand Index
40	f	17+ years	0.014	651.2575556	67	Grand Index
40	f	17+ years	0.015	653	67	Grand Index
40	f	17+ years	0.016	654.392	68	Grand Index
40	f	17+ years	0.017	655.1292018	68	Grand Index
40	f	17+ years	0.018	659.5793595	69	Grand Index
40	f	17+ years	0.019	661.514	69	Grand Index
40	f	17+ years	0.02	663.1094304	69	Grand Index

Age	Gender	Education	Percentile	Raw Score	Norm Score	Assessment
40	f	17+ years	0.021	665.452	69	Grand Index
40	f	17+ years	0.022	666.332	70	Grand Index
40	f	17+ years	0.024	667	70	Grand Index
40	f	17+ years	0.025	667.8064545	71	Grand Index
40	f	17+ years	0.026	668.0317465	71	Grand Index
40	f	17+ years	0.027	672.0252058	71	Grand Index
40	f	17+ years	0.028	673.9946259	71	Grand Index
40	f	17+ years	0.029	675.9878078	72	Grand Index
40	f	17+ years	0.031	677	72	Grand Index
40	f	17+ years	0.032	677.8489017	72	Grand Index
40	f	17+ years	0.033	678.9978469	72	Grand Index
40	f	17+ years	0.034	680.0381312	73	Grand Index
40	f	17+ years	0.036	682	73	Grand Index
40	f	17+ years	0.037	682.6847889	73	Grand Index
40	f	17+ years	0.038	683.0220964	73	Grand Index
40	f	17+ years	0.039	683.8636822	74	Grand Index
40	f	17+ years	0.041	684	74	Grand Index
40	f	17+ years	0.042	685.4349041	74	Grand Index
40	f	17+ years	0.043	687.6436422	74	Grand Index
40	f	17+ years	0.044	688	74	Grand Index
40	f	17+ years	0.045	688.9625671	75	Grand Index
40	f	17+ years	0.046	689	75	Grand Index
40	f	17+ years	0.047	689.482	75	Grand Index
40	f	17+ years	0.048	690	75	Grand Index
40	f	17+ years	0.049	690.694	75	Grand Index
40	f	17+ years	0.05	691	75	Grand Index
40	f	17+ years	0.051	691.0365758	75	Grand Index
40	f	17+ years	0.054	692	76	Grand Index
40	f	17+ years	0.055	692.33	76	Grand Index
40	f	17+ years	0.056	693.936	76	Grand Index
40	f	17+ years	0.057	697	76	Grand Index
40	f	17+ years	0.058	698	76	Grand Index
40	f	17+ years	0.059	698.754	77	Grand Index
40	f	17+ years	0.06	699.3566499	77	Grand Index
40	f	17+ years	0.061	699.9996836	77	Grand Index
40	f	17+ years	0.062	700	77	Grand Index
40	f	17+ years	0.063	700.178	77	Grand Index
40	f	17+ years	0.064	701.0043947	77	Grand Index
40	f	17+ years	0.065	701.6186906	77	Grand Index
40	f	17+ years	0.066	702.996	77	Grand Index
40	f	17+ years	0.067	703.0488953	78	Grand Index
40	f	17+ years	0.069	704	78	Grand Index
40	f	17+ years	0.071	705	78	Grand Index
40	f	17+ years	0.072	705.4822021	78	Grand Index
40	f	17+ years	0.073	705.9832999	78	Grand Index
40	f	17+ years	0.074	706.844	78	Grand Index
40	f	17+ years	0.075	707	78	Grand Index
40	f	17+ years	0.076	707.056	79	Grand Index
40	f	17+ years	0.077	708	79	Grand Index
40	f	17+ years	0.078	709	79	Grand Index
40	f	17+ years	0.079	709.874	79	Grand Index
40	f	17+ years	0.081	710	79	Grand Index
40	f	17+ years	0.082	710.692	79	Grand Index
40	f	17+ years	0.083	711.298	79	Grand Index
40	f	17+ years	0.084	712.904	79	Grand Index
40	f	17+ years	0.087	713	80	Grand Index

Age	Gender	Education	Percentile	Raw Score	Norm Score	Assessment
40	f	17+ years	0.088	713.328	80	Grand Index
40	f	17+ years	0.089	714	80	Grand Index
40	f	17+ years	0.09	714.54	80	Grand Index
40	f	17+ years	0.094	715	80	Grand Index
40	f	17+ years	0.095	715.3175503	80	Grand Index
40	f	17+ years	0.096	715.9871714	80	Grand Index
40	f	17+ years	0.097	716	81	Grand Index
40	f	17+ years	0.098	716.1667257	81	Grand Index
40	f	17+ years	0.099	716.9965782	81	Grand Index
40	f	17+ years	0.1	717	81	Grand Index
40	f	17+ years	0.101	717.206	81	Grand Index
40	f	17+ years	0.102	718	81	Grand Index
40	f	17+ years	0.104	719	81	Grand Index
40	f	17+ years	0.105	719.63	81	Grand Index
40	f	17+ years	0.106	720.236	81	Grand Index
40	f	17+ years	0.107	721	81	Grand Index
40	f	17+ years	0.108	721.448	81	Grand Index
40	f	17+ years	0.109	722	82	Grand Index
40	f	17+ years	0.11	722.66	82	Grand Index
40	f	17+ years	0.112	723	82	Grand Index
40	f	17+ years	0.113	723.8305049	82	Grand Index
40	f	17+ years	0.114	724.9939338	82	Grand Index
40	f	17+ years	0.117	725	82	Grand Index
40	f	17+ years	0.118	725.3250948	82	Grand Index
40	f	17+ years	0.119	725.7051327	82	Grand Index
40	f	17+ years	0.12	725.9754857	82	Grand Index
40	f	17+ years	0.121	726.326	82	Grand Index
40	f	17+ years	0.122	727	83	Grand Index
40	f	17+ years	0.123	727.0332376	83	Grand Index
40	f	17+ years	0.124	728	83	Grand Index
40	f	17+ years	0.125	728.0560199	83	Grand Index
40	f	17+ years	0.128	729	83	Grand Index
40	f	17+ years	0.129	730.0009109	83	Grand Index
40	f	17+ years	0.13	730.7811518	83	Grand Index
40	f	17+ years	0.133	731	83	Grand Index
40	f	17+ years	0.134	731.9760846	83	Grand Index
40	f	17+ years	0.135	732	83	Grand Index
40	f	17+ years	0.136	732.9908112	84	Grand Index
40	f	17+ years	0.137	733.022	84	Grand Index
40	f	17+ years	0.14	734	84	Grand Index
40	f	17+ years	0.141	734.446	84	Grand Index
40	f	17+ years	0.145	735	84	Grand Index
40	f	17+ years	0.146	735.0230875	84	Grand Index
40	f	17+ years	0.147	735.9503877	84	Grand Index
40	f	17+ years	0.15	736	84	Grand Index
40	f	17+ years	0.151	737	85	Grand Index
40	f	17+ years	0.152	737.112	85	Grand Index
40	f	17+ years	0.154	738	85	Grand Index
40	f	17+ years	0.155	738.93	85	Grand Index
40	f	17+ years	0.158	739	85	Grand Index
40	f	17+ years	0.159	739.354	85	Grand Index
40	f	17+ years	0.16	740.8968313	85	Grand Index
40	f	17+ years	0.164	741	85	Grand Index
40	f	17+ years	0.165	741.1878941	85	Grand Index
40	f	17+ years	0.17	742	86	Grand Index
40	f	17+ years	0.171	742.626	86	Grand Index

Age	Gender	Education	Percentile	Raw Score	Norm Score	Assessment
40	f	17+ years	0.176	743	86	Grand Index
40	f	17+ years	0.177	743.3113114	86	Grand Index
40	f	17+ years	0.178	743.9657976	86	Grand Index
40	f	17+ years	0.18	744	86	Grand Index
40	f	17+ years	0.181	744.3129045	86	Grand Index
40	f	17+ years	0.182	744.817354	86	Grand Index
40	f	17+ years	0.185	745	87	Grand Index
40	f	17+ years	0.186	745.3335391	87	Grand Index
40	f	17+ years	0.187	745.6461177	87	Grand Index
40	f	17+ years	0.188	746	87	Grand Index
40	f	17+ years	0.189	746.3247778	87	Grand Index
40	f	17+ years	0.194	747	87	Grand Index
40	f	17+ years	0.198	748	87	Grand Index
40	f	17+ years	0.199	748.594	87	Grand Index
40	f	17+ years	0.201	749	87	Grand Index
40	f	17+ years	0.202	749.2096853	87	Grand Index
40	f	17+ years	0.204	750	88	Grand Index
40	f	17+ years	0.205	750.9841556	88	Grand Index
40	f	17+ years	0.209	751	88	Grand Index
40	f	17+ years	0.21	751.9683218	88	Grand Index
40	f	17+ years	0.211	752	88	Grand Index
40	f	17+ years	0.212	752.9778475	88	Grand Index
40	f	17+ years	0.213	753	88	Grand Index
40	f	17+ years	0.214	753.0076076	88	Grand Index
40	f	17+ years	0.219	754	88	Grand Index
40	f	17+ years	0.22	754.6321259	88	Grand Index
40	f	17+ years	0.222	755	89	Grand Index
40	f	17+ years	0.223	755.059449	89	Grand Index
40	f	17+ years	0.224	755.5550736	89	Grand Index
40	f	17+ years	0.23	756	89	Grand Index
40	f	17+ years	0.231	756.986	89	Grand Index
40	f	17+ years	0.232	757	89	Grand Index
40	f	17+ years	0.233	757.9485923	89	Grand Index
40	f	17+ years	0.237	758	89	Grand Index
40	f	17+ years	0.238	758.2408845	89	Grand Index
40	f	17+ years	0.239	758.919231	89	Grand Index
40	f	17+ years	0.241	759	89	Grand Index
40	f	17+ years	0.242	759.652	90	Grand Index
40	f	17+ years	0.244	760	90	Grand Index
40	f	17+ years	0.245	760.347366	90	Grand Index
40	f	17+ years	0.248	761	90	Grand Index
40	f	17+ years	0.249	761.894	90	Grand Index
40	f	17+ years	0.25	762.2604291	90	Grand Index
40	f	17+ years	0.254	763	90	Grand Index
40	f	17+ years	0.255	763.0275223	90	Grand Index
40	f	17+ years	0.259	764	90	Grand Index
40	f	17+ years	0.26	764.56	90	Grand Index
40	f	17+ years	0.261	765.1768557	90	Grand Index
40	f	17+ years	0.264	766	91	Grand Index
40	f	17+ years	0.265	766.0567704	91	Grand Index
40	f	17+ years	0.268	767	91	Grand Index
40	f	17+ years	0.269	767.0007958	91	Grand Index
40	f	17+ years	0.27	767.6309019	91	Grand Index
40	f	17+ years	0.272	768	91	Grand Index
40	f	17+ years	0.273	768.0007633	91	Grand Index
40	f	17+ years	0.274	768.2779288	91	Grand Index

Age	Gender	Education	Percentile	Raw Score	Norm Score	Assessment
40	f	17+ years	0.277	769	91	Grand Index
40	f	17+ years	0.278	769.0502363	91	Grand Index
40	f	17+ years	0.279	769.2675578	91	Grand Index
40	f	17+ years	0.28	769.9734385	91	Grand Index
40	f	17+ years	0.283	770	91	Grand Index
40	f	17+ years	0.284	770.104	91	Grand Index
40	f	17+ years	0.291	771	92	Grand Index
40	f	17+ years	0.292	771.952	92	Grand Index
40	f	17+ years	0.297	772	92	Grand Index
40	f	17+ years	0.298	772.588	92	Grand Index
40	f	17+ years	0.3	773	92	Grand Index
40	f	17+ years	0.301	773.0160746	92	Grand Index
40	f	17+ years	0.302	773.1408888	92	Grand Index
40	f	17+ years	0.308	774	92	Grand Index
40	f	17+ years	0.309	774.6586646	93	Grand Index
40	f	17+ years	0.315	775	93	Grand Index
40	f	17+ years	0.316	775.496	93	Grand Index
40	f	17+ years	0.319	776	93	Grand Index
40	f	17+ years	0.32	776.7159213	93	Grand Index
40	f	17+ years	0.325	777	93	Grand Index
40	f	17+ years	0.326	777.556	93	Grand Index
40	f	17+ years	0.329	778	93	Grand Index
40	f	17+ years	0.33	778.0188542	93	Grand Index
40	f	17+ years	0.331	778.5950306	93	Grand Index
40	f	17+ years	0.333	779	94	Grand Index
40	f	17+ years	0.336	780	94	Grand Index
40	f	17+ years	0.337	780.2175589	94	Grand Index
40	f	17+ years	0.338	780.9965591	94	Grand Index
40	f	17+ years	0.339	781	94	Grand Index
40	f	17+ years	0.34	781.2856588	94	Grand Index
40	f	17+ years	0.341	781.8791245	94	Grand Index
40	f	17+ years	0.342	782	94	Grand Index
40	f	17+ years	0.343	782.858	94	Grand Index
40	f	17+ years	0.346	783	94	Grand Index
40	f	17+ years	0.347	783.9762527	94	Grand Index
40	f	17+ years	0.351	784	94	Grand Index
40	f	17+ years	0.352	784.3811441	94	Grand Index
40	f	17+ years	0.353	784.9829685	94	Grand Index
40	f	17+ years	0.357	785	95	Grand Index
40	f	17+ years	0.358	785.0380704	95	Grand Index
40	f	17+ years	0.359	785.8619454	95	Grand Index
40	f	17+ years	0.361	786	95	Grand Index
40	f	17+ years	0.362	786.0000107	95	Grand Index
40	f	17+ years	0.363	786.9780006	95	Grand Index
40	f	17+ years	0.364	787	95	Grand Index
40	f	17+ years	0.365	787.19	95	Grand Index
40	f	17+ years	0.368	788	95	Grand Index
40	f	17+ years	0.369	788.0447275	95	Grand Index
40	f	17+ years	0.37	788.2369848	95	Grand Index
40	f	17+ years	0.371	788.8775228	95	Grand Index
40	f	17+ years	0.374	789	95	Grand Index
40	f	17+ years	0.375	789.0174302	95	Grand Index
40	f	17+ years	0.376	789.1043195	95	Grand Index
40	f	17+ years	0.377	789.7190257	95	Grand Index
40	f	17+ years	0.378	790.068	95	Grand Index
40	f	17+ years	0.384	791	96	Grand Index

Age	Gender	Education	Percentile	Raw Score	Norm Score	Assessment
40	f	17+ years	0.385	791.0751035	96	Grand Index
40	f	17+ years	0.386	791.9363506	96	Grand Index
40	f	17+ years	0.39	792	96	Grand Index
40	f	17+ years	0.391	792.0492922	96	Grand Index
40	f	17+ years	0.395	793	96	Grand Index
40	f	17+ years	0.396	793.8566179	96	Grand Index
40	f	17+ years	0.398	794	96	Grand Index
40	f	17+ years	0.399	794.794	96	Grand Index
40	f	17+ years	0.402	795	96	Grand Index
40	f	17+ years	0.403	795.9465271	96	Grand Index
40	f	17+ years	0.408	796	97	Grand Index
40	f	17+ years	0.409	796.0998579	97	Grand Index
40	f	17+ years	0.41	796.9673001	97	Grand Index
40	f	17+ years	0.412	797	97	Grand Index
40	f	17+ years	0.417	798	97	Grand Index
40	f	17+ years	0.418	798.1465564	97	Grand Index
40	f	17+ years	0.419	798.9549216	97	Grand Index
40	f	17+ years	0.42	799	97	Grand Index
40	f	17+ years	0.421	799.3734591	97	Grand Index
40	f	17+ years	0.422	799.5230212	97	Grand Index
40	f	17+ years	0.425	800	97	Grand Index
40	f	17+ years	0.426	800.4475978	97	Grand Index
40	f	17+ years	0.427	800.9677922	97	Grand Index
40	f	17+ years	0.429	801	97	Grand Index
40	f	17+ years	0.43	801.4658753	97	Grand Index
40	f	17+ years	0.434	802	98	Grand Index
40	f	17+ years	0.435	802.038	98	Grand Index
40	f	17+ years	0.436	802.8236508	98	Grand Index
40	f	17+ years	0.441	803	98	Grand Index
40	f	17+ years	0.442	803.0162648	98	Grand Index
40	f	17+ years	0.445	804	98	Grand Index
40	f	17+ years	0.446	804.276	98	Grand Index
40	f	17+ years	0.453	805	98	Grand Index
40	f	17+ years	0.457	806	98	Grand Index
40	f	17+ years	0.458	806.548	98	Grand Index
40	f	17+ years	0.462	807	99	Grand Index
40	f	17+ years	0.463	807.6036233	99	Grand Index
40	f	17+ years	0.466	808	99	Grand Index
40	f	17+ years	0.468	809	99	Grand Index
40	f	17+ years	0.469	809.0173071	99	Grand Index
40	f	17+ years	0.47	809.8345573	99	Grand Index
40	f	17+ years	0.471	810	99	Grand Index
40	f	17+ years	0.472	810.0626011	99	Grand Index
40	f	17+ years	0.473	810.2603021	99	Grand Index
40	f	17+ years	0.474	810.7490386	99	Grand Index
40	f	17+ years	0.481	811	99	Grand Index
40	f	17+ years	0.482	811.7972231	99	Grand Index
40	f	17+ years	0.483	811.9760074	99	Grand Index
40	f	17+ years	0.489	812	100	Grand Index
40	f	17+ years	0.49	812.0552941	100	Grand Index
40	f	17+ years	0.491	812.8823644	100	Grand Index
40	f	17+ years	0.498	813	100	Grand Index
40	f	17+ years	0.499	813.394	100	Grand Index
40	f	17+ years	0.502	814	100	Grand Index
40	f	17+ years	0.503	814.0821646	100	Grand Index
40	f	17+ years	0.504	814.512117	100	Grand Index

Age	Gender	Education	Percentile	Raw Score	Norm Score	Assessment
40	f	17+ years	0.506	815	100	Grand Index
40	f	17+ years	0.507	815.0005518	100	Grand Index
40	f	17+ years	0.508	815.8157558	100	Grand Index
40	f	17+ years	0.514	816	101	Grand Index
40	f	17+ years	0.515	816.0608113	101	Grand Index
40	f	17+ years	0.516	816.8398224	101	Grand Index
40	f	17+ years	0.523	817	101	Grand Index
40	f	17+ years	0.524	817.5908661	101	Grand Index
40	f	17+ years	0.526	818	101	Grand Index
40	f	17+ years	0.527	818.2706991	101	Grand Index
40	f	17+ years	0.528	818.9919292	101	Grand Index
40	f	17+ years	0.529	819	101	Grand Index
40	f	17+ years	0.53	819.18	101	Grand Index
40	f	17+ years	0.534	820	101	Grand Index
40	f	17+ years	0.535	820.0703063	101	Grand Index
40	f	17+ years	0.536	820.8515279	101	Grand Index
40	f	17+ years	0.539	821	101	Grand Index
40	f	17+ years	0.54	821.24	102	Grand Index
40	f	17+ years	0.541	822	102	Grand Index
40	f	17+ years	0.542	822.0068678	102	Grand Index
40	f	17+ years	0.548	823	102	Grand Index
40	f	17+ years	0.549	823.0432328	102	Grand Index
40	f	17+ years	0.55	823.2725454	102	Grand Index
40	f	17+ years	0.551	823.9763323	102	Grand Index
40	f	17+ years	0.554	824	102	Grand Index
40	f	17+ years	0.555	824.245213	102	Grand Index
40	f	17+ years	0.556	824.9835565	102	Grand Index
40	f	17+ years	0.562	825	102	Grand Index
40	f	17+ years	0.563	825.2457221	102	Grand Index
40	f	17+ years	0.568	826	103	Grand Index
40	f	17+ years	0.569	826.6825702	103	Grand Index
40	f	17+ years	0.573	827	103	Grand Index
40	f	17+ years	0.574	827.844	103	Grand Index
40	f	17+ years	0.577	828	103	Grand Index
40	f	17+ years	0.578	828.0004558	103	Grand Index
40	f	17+ years	0.579	828.3509233	103	Grand Index
40	f	17+ years	0.58	828.9894228	103	Grand Index
40	f	17+ years	0.583	829	103	Grand Index
40	f	17+ years	0.584	829.8987941	103	Grand Index
40	f	17+ years	0.59	830	103	Grand Index
40	f	17+ years	0.591	830.965253	103	Grand Index
40	f	17+ years	0.595	831	104	Grand Index
40	f	17+ years	0.596	831.1241824	104	Grand Index
40	f	17+ years	0.597	831.858241	104	Grand Index
40	f	17+ years	0.603	832	104	Grand Index
40	f	17+ years	0.604	832.0157855	104	Grand Index
40	f	17+ years	0.605	832.7409388	104	Grand Index
40	f	17+ years	0.61	833	104	Grand Index
40	f	17+ years	0.611	833.2586103	104	Grand Index
40	f	17+ years	0.612	833.9156689	104	Grand Index
40	f	17+ years	0.618	834	105	Grand Index
40	f	17+ years	0.619	834.0418974	105	Grand Index
40	f	17+ years	0.62	834.7734539	105	Grand Index
40	f	17+ years	0.623	835	105	Grand Index
40	f	17+ years	0.627	836	105	Grand Index
40	f	17+ years	0.628	836.6046256	105	Grand Index

Age	Gender	Education	Percentile	Raw Score	Norm Score	Assessment
40	f	17+ years	0.63	837	105	Grand Index
40	f	17+ years	0.631	837.1805572	105	Grand Index
40	f	17+ years	0.632	837.8710529	105	Grand Index
40	f	17+ years	0.633	838	105	Grand Index
40	f	17+ years	0.64	839	105	Grand Index
40	f	17+ years	0.641	839.4342041	105	Grand Index
40	f	17+ years	0.646	840	106	Grand Index
40	f	17+ years	0.647	840.9965754	106	Grand Index
40	f	17+ years	0.653	841	106	Grand Index
40	f	17+ years	0.654	841.2778429	106	Grand Index
40	f	17+ years	0.655	841.9582418	106	Grand Index
40	f	17+ years	0.66	842	106	Grand Index
40	f	17+ years	0.661	842.9860334	106	Grand Index
40	f	17+ years	0.664	843	106	Grand Index
40	f	17+ years	0.665	843.0324687	106	Grand Index
40	f	17+ years	0.669	844	107	Grand Index
40	f	17+ years	0.673	845	107	Grand Index
40	f	17+ years	0.674	845.1027241	107	Grand Index
40	f	17+ years	0.679	846	107	Grand Index
40	f	17+ years	0.68	846.0488162	107	Grand Index
40	f	17+ years	0.681	846.8776035	107	Grand Index
40	f	17+ years	0.685	847	107	Grand Index
40	f	17+ years	0.686	847.0039745	107	Grand Index
40	f	17+ years	0.687	847.612373	107	Grand Index
40	f	17+ years	0.688	847.9918388	107	Grand Index
40	f	17+ years	0.69	848	107	Grand Index
40	f	17+ years	0.691	848.2611185	107	Grand Index
40	f	17+ years	0.693	849	108	Grand Index
40	f	17+ years	0.694	849.2017399	108	Grand Index
40	f	17+ years	0.695	849.9118709	108	Grand Index
40	f	17+ years	0.699	850	108	Grand Index
40	f	17+ years	0.7	850.1796701	108	Grand Index
40	f	17+ years	0.701	850.98028	108	Grand Index
40	f	17+ years	0.702	851.2735726	108	Grand Index
40	f	17+ years	0.703	851.9819239	108	Grand Index
40	f	17+ years	0.707	852	108	Grand Index
40	f	17+ years	0.708	852.048	108	Grand Index
40	f	17+ years	0.711	853	108	Grand Index
40	f	17+ years	0.715	854	109	Grand Index
40	f	17+ years	0.716	854.7748262	109	Grand Index
40	f	17+ years	0.72	855	109	Grand Index
40	f	17+ years	0.721	855.926	109	Grand Index
40	f	17+ years	0.722	856	109	Grand Index
40	f	17+ years	0.723	856.1102653	109	Grand Index
40	f	17+ years	0.724	856.8832997	109	Grand Index
40	f	17+ years	0.725	856.9857143	109	Grand Index
40	f	17+ years	0.727	857	109	Grand Index
40	f	17+ years	0.731	858	109	Grand Index
40	f	17+ years	0.732	858.592	109	Grand Index
40	f	17+ years	0.737	859	110	Grand Index
40	f	17+ years	0.738	859.2256944	110	Grand Index
40	f	17+ years	0.739	859.9983214	110	Grand Index
40	f	17+ years	0.744	860	110	Grand Index
40	f	17+ years	0.745	860.0837861	110	Grand Index
40	f	17+ years	0.749	861	110	Grand Index
40	f	17+ years	0.75	861.4872237	110	Grand Index

Age	Gender	Education	Percentile	Raw Score	Norm Score	Assessment
40	f	17+ years	0.752	862	110	Grand Index
40	f	17+ years	0.753	862.318	110	Grand Index
40	f	17+ years	0.756	863	110	Grand Index
40	f	17+ years	0.757	863.742	110	Grand Index
40	f	17+ years	0.759	864	111	Grand Index
40	f	17+ years	0.76	865	111	Grand Index
40	f	17+ years	0.761	865.166	111	Grand Index
40	f	17+ years	0.765	866	111	Grand Index
40	f	17+ years	0.766	866.196	111	Grand Index
40	f	17+ years	0.77	867	111	Grand Index
40	f	17+ years	0.771	867.5396017	111	Grand Index
40	f	17+ years	0.772	867.9619471	111	Grand Index
40	f	17+ years	0.773	868	111	Grand Index
40	f	17+ years	0.774	868.0128686	111	Grand Index
40	f	17+ years	0.775	868.7513928	111	Grand Index
40	f	17+ years	0.777	869	111	Grand Index
40	f	17+ years	0.778	869.468	111	Grand Index
40	f	17+ years	0.784	870	112	Grand Index
40	f	17+ years	0.785	870.6781491	112	Grand Index
40	f	17+ years	0.79	871	112	Grand Index
40	f	17+ years	0.791	871.346	112	Grand Index
40	f	17+ years	0.792	872	112	Grand Index
40	f	17+ years	0.793	872.558	112	Grand Index
40	f	17+ years	0.797	873	112	Grand Index
40	f	17+ years	0.799	874	113	Grand Index
40	f	17+ years	0.8	874.1224411	113	Grand Index
40	f	17+ years	0.803	875	113	Grand Index
40	f	17+ years	0.806	876	113	Grand Index
40	f	17+ years	0.807	876.0066777	113	Grand Index
40	f	17+ years	0.808	876.1627004	113	Grand Index
40	f	17+ years	0.809	876.3180274	113	Grand Index
40	f	17+ years	0.81	876.9383373	113	Grand Index
40	f	17+ years	0.812	877	113	Grand Index
40	f	17+ years	0.813	877.678	113	Grand Index
40	f	17+ years	0.815	878	113	Grand Index
40	f	17+ years	0.816	878.494601	114	Grand Index
40	f	17+ years	0.817	879	114	Grand Index
40	f	17+ years	0.818	879.708	114	Grand Index
40	f	17+ years	0.819	880.129549	114	Grand Index
40	f	17+ years	0.82	880.9530061	114	Grand Index
40	f	17+ years	0.821	881	114	Grand Index
40	f	17+ years	0.822	881.3784563	114	Grand Index
40	f	17+ years	0.825	882	114	Grand Index
40	f	17+ years	0.826	882.8893796	114	Grand Index
40	f	17+ years	0.827	883	114	Grand Index
40	f	17+ years	0.828	883.768	114	Grand Index
40	f	17+ years	0.829	884	114	Grand Index
40	f	17+ years	0.83	884.2912197	114	Grand Index
40	f	17+ years	0.831	884.7984834	114	Grand Index
40	f	17+ years	0.835	885	115	Grand Index
40	f	17+ years	0.836	885.4621463	115	Grand Index
40	f	17+ years	0.839	886	115	Grand Index
40	f	17+ years	0.84	886.04	115	Grand Index
40	f	17+ years	0.843	887	115	Grand Index
40	f	17+ years	0.844	887.176107	115	Grand Index
40	f	17+ years	0.845	887.5652303	115	Grand Index

Age	Gender	Education	Percentile	Raw Score	Norm Score	Assessment
40	f	17+ years	0.848	888	115	Grand Index
40	f	17+ years	0.849	888.2644261	115	Grand Index
40	f	17+ years	0.853	889	116	Grand Index
40	f	17+ years	0.854	889.524	116	Grand Index
40	f	17+ years	0.855	890	116	Grand Index
40	f	17+ years	0.856	890.736	116	Grand Index
40	f	17+ years	0.857	891.342	116	Grand Index
40	f	17+ years	0.858	892	116	Grand Index
40	f	17+ years	0.859	892.5958787	116	Grand Index
40	f	17+ years	0.861	893	116	Grand Index
40	f	17+ years	0.862	893.4185323	116	Grand Index
40	f	17+ years	0.863	894	116	Grand Index
40	f	17+ years	0.864	894.242131	116	Grand Index
40	f	17+ years	0.866	895	117	Grand Index
40	f	17+ years	0.867	895.402	117	Grand Index
40	f	17+ years	0.868	897	117	Grand Index
40	f	17+ years	0.869	897.0220886	117	Grand Index
40	f	17+ years	0.871	898	117	Grand Index
40	f	17+ years	0.873	899	117	Grand Index
40	f	17+ years	0.874	899.644	117	Grand Index
40	f	17+ years	0.876	901	117	Grand Index
40	f	17+ years	0.877	902	117	Grand Index
40	f	17+ years	0.878	902.4128853	117	Grand Index
40	f	17+ years	0.881	903	118	Grand Index
40	f	17+ years	0.882	903.0037016	118	Grand Index
40	f	17+ years	0.885	904	118	Grand Index
40	f	17+ years	0.886	904.916	118	Grand Index
40	f	17+ years	0.888	905	118	Grand Index
40	f	17+ years	0.889	905.734	118	Grand Index
40	f	17+ years	0.891	906	118	Grand Index
40	f	17+ years	0.892	907	119	Grand Index
40	f	17+ years	0.894	908	119	Grand Index
40	f	17+ years	0.895	908.37	119	Grand Index
40	f	17+ years	0.897	909	119	Grand Index
40	f	17+ years	0.898	909.0447512	119	Grand Index
40	f	17+ years	0.899	909.4356536	119	Grand Index
40	f	17+ years	0.901	910	119	Grand Index
40	f	17+ years	0.902	910.612	119	Grand Index
40	f	17+ years	0.903	912	119	Grand Index
40	f	17+ years	0.904	912.824	120	Grand Index
40	f	17+ years	0.908	913	120	Grand Index
40	f	17+ years	0.909	913.854	120	Grand Index
40	f	17+ years	0.91	914	120	Grand Index
40	f	17+ years	0.911	914.925545	120	Grand Index
40	f	17+ years	0.916	915	121	Grand Index
40	f	17+ years	0.917	915.6281458	121	Grand Index
40	f	17+ years	0.918	916	121	Grand Index
40	f	17+ years	0.919	916.4975378	121	Grand Index
40	f	17+ years	0.922	917	121	Grand Index
40	f	17+ years	0.923	917.5557024	121	Grand Index
40	f	17+ years	0.924	918.944	121	Grand Index
40	f	17+ years	0.925	919	122	Grand Index
40	f	17+ years	0.926	919.8963609	122	Grand Index
40	f	17+ years	0.927	919.981178	122	Grand Index
40	f	17+ years	0.929	920	122	Grand Index
40	f	17+ years	0.93	920.58	122	Grand Index

Age	Gender	Education	Percentile	Raw Score	Norm Score	Assessment
40	f	17+ years	0.931	921	122	Grand Index
40	f	17+ years	0.932	921.4941825	122	Grand Index
40	f	17+ years	0.933	923.1828936	122	Grand Index
40	f	17+ years	0.934	923.7739244	123	Grand Index
40	f	17+ years	0.935	924	123	Grand Index
40	f	17+ years	0.936	924.216	123	Grand Index
40	f	17+ years	0.937	925	123	Grand Index
40	f	17+ years	0.938	926.2847151	123	Grand Index
40	f	17+ years	0.939	928	123	Grand Index
40	f	17+ years	0.94	928.3676124	123	Grand Index
40	f	17+ years	0.941	929.0110664	123	Grand Index
40	f	17+ years	0.942	929.3469262	124	Grand Index
40	f	17+ years	0.943	930	124	Grand Index
40	f	17+ years	0.944	930.064	124	Grand Index
40	f	17+ years	0.945	931	124	Grand Index
40	f	17+ years	0.946	931.276	124	Grand Index
40	f	17+ years	0.947	932	124	Grand Index
40	f	17+ years	0.948	932.488	124	Grand Index
40	f	17+ years	0.949	933.094	125	Grand Index
40	f	17+ years	0.951	934	125	Grand Index
40	f	17+ years	0.952	934.1910066	125	Grand Index
40	f	17+ years	0.953	935.5158558	125	Grand Index
40	f	17+ years	0.954	936.078864	125	Grand Index
40	f	17+ years	0.955	936.90172	125	Grand Index
40	f	17+ years	0.956	938	126	Grand Index
40	f	17+ years	0.957	939.6102594	126	Grand Index
40	f	17+ years	0.958	941.1547214	126	Grand Index
40	f	17+ years	0.959	942.0289374	126	Grand Index
40	f	17+ years	0.96	942.9340915	126	Grand Index
40	f	17+ years	0.961	943	126	Grand Index
40	f	17+ years	0.962	943.0943219	127	Grand Index
40	f	17+ years	0.963	945.578	127	Grand Index
40	f	17+ years	0.964	946	127	Grand Index
40	f	17+ years	0.965	946.79	127	Grand Index
40	f	17+ years	0.966	947.1826486	127	Grand Index
40	f	17+ years	0.967	948.3081193	128	Grand Index
40	f	17+ years	0.968	948.9959569	128	Grand Index
40	f	17+ years	0.97	949	128	Grand Index
40	f	17+ years	0.971	951.278	128	Grand Index
40	f	17+ years	0.972	954.1669381	129	Grand Index
40	f	17+ years	0.973	956.7959966	129	Grand Index
40	f	17+ years	0.974	958.183557	129	Grand Index
40	f	17+ years	0.975	960.3416358	129	Grand Index
40	f	17+ years	0.976	962	130	Grand Index
40	f	17+ years	0.977	962.5299692	130	Grand Index
40	f	17+ years	0.978	963	130	Grand Index
40	f	17+ years	0.979	963.274	131	Grand Index
40	f	17+ years	0.98	964.6441549	131	Grand Index
40	f	17+ years	0.981	965.2106202	131	Grand Index
40	f	17+ years	0.982	966.46	131	Grand Index
40	f	17+ years	0.983	971.4458107	132	Grand Index
40	f	17+ years	0.984	973.52	132	Grand Index
40	f	17+ years	0.985	977.8282696	133	Grand Index
40	f	17+ years	0.986	981.22938	133	Grand Index
40	f	17+ years	0.987	985.7149223	133	Grand Index
40	f	17+ years	0.988	988.8436228	134	Grand Index

Age	Gender	Education	Percentile	Raw Score	Norm Score	Assessment
40	f	17+ years	0.989	995.668	134	Grand Index
40	f	17+ years	0.99	997.94	135	Grand Index
40	f	17+ years	0.991	998	135	Grand Index
40	f	17+ years	0.992	999.6813135	136	Grand Index
40	f	17+ years	0.993	1007.393886	137	Grand Index
40	f	17+ years	0.994	1011.558276	138	Grand Index
40	f	17+ years	0.995	1021.73	139	Grand Index
40	f	17+ years	0.996	1032.215044	140	Grand Index
40	f	17+ years	0.997	1038.695957	141	Grand Index
40	f	17+ years	0.998	1039.675486	143	Grand Index
40	f	17+ years	0.999	1044.622007	146	Grand Index
40	m	17+ years	0.001	590.6717737	54	Grand Index
40	m	17+ years	0.002	606.182639	57	Grand Index
40	m	17+ years	0.003	615.866	59	Grand Index
40	m	17+ years	0.004	622.992	60	Grand Index
40	m	17+ years	0.005	628.74	61	Grand Index
40	m	17+ years	0.006	636.976	62	Grand Index
40	m	17+ years	0.007	640.8376704	63	Grand Index
40	m	17+ years	0.008	642	64	Grand Index
40	m	17+ years	0.009	642.933	65	Grand Index
40	m	17+ years	0.01	645.4695365	65	Grand Index
40	m	17+ years	0.011	646	66	Grand Index
40	m	17+ years	0.012	649.244	66	Grand Index
40	m	17+ years	0.013	651.1696553	67	Grand Index
40	m	17+ years	0.014	652.8976753	67	Grand Index
40	m	17+ years	0.015	655.0827127	67	Grand Index
40	m	17+ years	0.016	655.9949608	68	Grand Index
40	m	17+ years	0.017	656.0752317	68	Grand Index
40	m	17+ years	0.018	657.755499	69	Grand Index
40	m	17+ years	0.019	658.5324933	69	Grand Index
40	m	17+ years	0.02	659.3337563	69	Grand Index
40	m	17+ years	0.021	660	69	Grand Index
40	m	17+ years	0.022	661.228	70	Grand Index
40	m	17+ years	0.023	662.9531912	70	Grand Index
40	m	17+ years	0.025	664	71	Grand Index
40	m	17+ years	0.026	664.6314558	71	Grand Index
40	m	17+ years	0.027	665	71	Grand Index
40	m	17+ years	0.028	666.236	71	Grand Index
40	m	17+ years	0.029	668.346	72	Grand Index
40	m	17+ years	0.03	672.11	72	Grand Index
40	m	17+ years	0.031	673.1168738	72	Grand Index
40	m	17+ years	0.032	673.9874186	72	Grand Index
40	m	17+ years	0.033	674.421	72	Grand Index
40	m	17+ years	0.034	677.5480478	73	Grand Index
40	m	17+ years	0.035	678	73	Grand Index
40	m	17+ years	0.036	678.2297516	73	Grand Index
40	m	17+ years	0.037	679.2664554	73	Grand Index
40	m	17+ years	0.038	680.8332037	73	Grand Index
40	m	17+ years	0.039	681.043	74	Grand Index
40	m	17+ years	0.04	682	74	Grand Index
40	m	17+ years	0.041	682.2236045	74	Grand Index
40	m	17+ years	0.042	683	74	Grand Index
40	m	17+ years	0.043	683.791	74	Grand Index
40	m	17+ years	0.045	685	75	Grand Index
40	m	17+ years	0.046	686.0391818	75	Grand Index
40	m	17+ years	0.047	686.7160865	75	Grand Index

Age	Gender	Education	Percentile	Raw Score	Norm Score	Assessment
40	m	17+ years	0.048	687	75	Grand Index
40	m	17+ years	0.049	688.413	75	Grand Index
40	m	17+ years	0.05	689.85	75	Grand Index
40	m	17+ years	0.051	690	75	Grand Index
40	m	17+ years	0.052	690.724	76	Grand Index
40	m	17+ years	0.053	691.2999755	76	Grand Index
40	m	17+ years	0.054	692.8823394	76	Grand Index
40	m	17+ years	0.055	693	76	Grand Index
40	m	17+ years	0.056	693.4072091	76	Grand Index
40	m	17+ years	0.057	693.9875085	76	Grand Index
40	m	17+ years	0.058	694.0068419	76	Grand Index
40	m	17+ years	0.059	694.787291	77	Grand Index
40	m	17+ years	0.061	695	77	Grand Index
40	m	17+ years	0.062	695.094	77	Grand Index
40	m	17+ years	0.066	696	77	Grand Index
40	m	17+ years	0.067	696.0125109	78	Grand Index
40	m	17+ years	0.068	696.7287351	78	Grand Index
40	m	17+ years	0.069	697.1778834	78	Grand Index
40	m	17+ years	0.07	698	78	Grand Index
40	m	17+ years	0.071	698.054	78	Grand Index
40	m	17+ years	0.072	700	78	Grand Index
40	m	17+ years	0.073	701.802	78	Grand Index
40	m	17+ years	0.074	702.338	78	Grand Index
40	m	17+ years	0.075	703	78	Grand Index
40	m	17+ years	0.076	703.212	79	Grand Index
40	m	17+ years	0.079	704	79	Grand Index
40	m	17+ years	0.08	704.96	79	Grand Index
40	m	17+ years	0.081	705	79	Grand Index
40	m	17+ years	0.082	705.8313221	79	Grand Index
40	m	17+ years	0.083	706.271	79	Grand Index
40	m	17+ years	0.084	707	79	Grand Index
40	m	17+ years	0.085	707.6761268	79	Grand Index
40	m	17+ years	0.086	707.8719099	80	Grand Index
40	m	17+ years	0.087	708.1309644	80	Grand Index
40	m	17+ years	0.088	709.456	80	Grand Index
40	m	17+ years	0.089	711.786	80	Grand Index
40	m	17+ years	0.091	712	80	Grand Index
40	m	17+ years	0.092	712.0080505	80	Grand Index
40	m	17+ years	0.093	712.6551673	80	Grand Index
40	m	17+ years	0.094	713	80	Grand Index
40	m	17+ years	0.095	713.515	80	Grand Index
40	m	17+ years	0.096	714	80	Grand Index
40	m	17+ years	0.097	714.1899311	81	Grand Index
40	m	17+ years	0.098	714.8468701	81	Grand Index
40	m	17+ years	0.1	715	81	Grand Index
40	m	17+ years	0.101	715.137	81	Grand Index
40	m	17+ years	0.103	716	81	Grand Index
40	m	17+ years	0.104	716.8942665	81	Grand Index
40	m	17+ years	0.105	719.7609426	81	Grand Index
40	m	17+ years	0.107	720	81	Grand Index
40	m	17+ years	0.108	721.1722542	81	Grand Index
40	m	17+ years	0.109	721.8892015	82	Grand Index
40	m	17+ years	0.111	722	82	Grand Index
40	m	17+ years	0.112	722.3171136	82	Grand Index
40	m	17+ years	0.113	723.0584619	82	Grand Index
40	m	17+ years	0.115	724	82	Grand Index

Age	Gender	Education	Percentile	Raw Score	Norm Score	Assessment
40	m	17+ years	0.116	724.6677385	82	Grand Index
40	m	17+ years	0.118	725	82	Grand Index
40	m	17+ years	0.121	726	82	Grand Index
40	m	17+ years	0.122	726.8091787	83	Grand Index
40	m	17+ years	0.123	726.9659826	83	Grand Index
40	m	17+ years	0.124	727.188	83	Grand Index
40	m	17+ years	0.128	728	83	Grand Index
40	m	17+ years	0.129	728.9293337	83	Grand Index
40	m	17+ years	0.13	729	83	Grand Index
40	m	17+ years	0.131	729.026573	83	Grand Index
40	m	17+ years	0.132	729.7179963	83	Grand Index
40	m	17+ years	0.135	730	83	Grand Index
40	m	17+ years	0.136	730.432	84	Grand Index
40	m	17+ years	0.139	731	84	Grand Index
40	m	17+ years	0.14	731.7203424	84	Grand Index
40	m	17+ years	0.141	732	84	Grand Index
40	m	17+ years	0.142	732.054	84	Grand Index
40	m	17+ years	0.144	733	84	Grand Index
40	m	17+ years	0.145	733.365	84	Grand Index
40	m	17+ years	0.15	734	84	Grand Index
40	m	17+ years	0.151	734.987	85	Grand Index
40	m	17+ years	0.152	735.312947	85	Grand Index
40	m	17+ years	0.153	735.7870538	85	Grand Index
40	m	17+ years	0.154	736.298	85	Grand Index
40	m	17+ years	0.159	737	85	Grand Index
40	m	17+ years	0.16	737.92	85	Grand Index
40	m	17+ years	0.161	738	85	Grand Index
40	m	17+ years	0.162	738.794	85	Grand Index
40	m	17+ years	0.164	739	85	Grand Index
40	m	17+ years	0.167	740	86	Grand Index
40	m	17+ years	0.168	740.0181025	86	Grand Index
40	m	17+ years	0.169	740.8593968	86	Grand Index
40	m	17+ years	0.171	741	86	Grand Index
40	m	17+ years	0.172	742	86	Grand Index
40	m	17+ years	0.173	742.601	86	Grand Index
40	m	17+ years	0.178	743	86	Grand Index
40	m	17+ years	0.179	743.2807819	86	Grand Index
40	m	17+ years	0.183	744	86	Grand Index
40	m	17+ years	0.184	744.6903178	86	Grand Index
40	m	17+ years	0.185	745	87	Grand Index
40	m	17+ years	0.186	745.1285088	87	Grand Index
40	m	17+ years	0.187	745.8470531	87	Grand Index
40	m	17+ years	0.191	746	87	Grand Index
40	m	17+ years	0.192	746.0544132	87	Grand Index
40	m	17+ years	0.193	746.4156778	87	Grand Index
40	m	17+ years	0.194	747	87	Grand Index
40	m	17+ years	0.195	747.1649198	87	Grand Index
40	m	17+ years	0.196	747.9189399	87	Grand Index
40	m	17+ years	0.199	748	87	Grand Index
40	m	17+ years	0.2	748.0492106	87	Grand Index
40	m	17+ years	0.201	748.8570533	87	Grand Index
40	m	17+ years	0.203	749	88	Grand Index
40	m	17+ years	0.204	749.148	88	Grand Index
40	m	17+ years	0.207	750	88	Grand Index
40	m	17+ years	0.208	750.896	88	Grand Index
40	m	17+ years	0.21	751	88	Grand Index

Age	Gender	Education	Percentile	Raw Score	Norm Score	Assessment
40	m	17+ years	0.211	751.207	88	Grand Index
40	m	17+ years	0.212	752	88	Grand Index
40	m	17+ years	0.213	752.0729962	88	Grand Index
40	m	17+ years	0.214	752.9523724	88	Grand Index
40	m	17+ years	0.216	753	88	Grand Index
40	m	17+ years	0.217	753.4034271	88	Grand Index
40	m	17+ years	0.221	754	88	Grand Index
40	m	17+ years	0.222	754.008895	89	Grand Index
40	m	17+ years	0.223	754.7998121	89	Grand Index
40	m	17+ years	0.227	755	89	Grand Index
40	m	17+ years	0.228	755.0801723	89	Grand Index
40	m	17+ years	0.229	755.8778826	89	Grand Index
40	m	17+ years	0.23	755.9585953	89	Grand Index
40	m	17+ years	0.235	756	89	Grand Index
40	m	17+ years	0.236	756.4907883	89	Grand Index
40	m	17+ years	0.241	757	89	Grand Index
40	m	17+ years	0.242	757.754	90	Grand Index
40	m	17+ years	0.245	758	90	Grand Index
40	m	17+ years	0.246	758.3801531	90	Grand Index
40	m	17+ years	0.247	758.9851939	90	Grand Index
40	m	17+ years	0.25	759	90	Grand Index
40	m	17+ years	0.251	759.2141584	90	Grand Index
40	m	17+ years	0.252	759.88173	90	Grand Index
40	m	17+ years	0.254	760	90	Grand Index
40	m	17+ years	0.255	760.435	90	Grand Index
40	m	17+ years	0.257	761	90	Grand Index
40	m	17+ years	0.258	761.746	90	Grand Index
40	m	17+ years	0.26	762	90	Grand Index
40	m	17+ years	0.263	763	90	Grand Index
40	m	17+ years	0.264	763.499189	91	Grand Index
40	m	17+ years	0.267	764	91	Grand Index
40	m	17+ years	0.268	764.0017101	91	Grand Index
40	m	17+ years	0.269	764.5595896	91	Grand Index
40	m	17+ years	0.27	765	91	Grand Index
40	m	17+ years	0.271	765.427	91	Grand Index
40	m	17+ years	0.274	766	91	Grand Index
40	m	17+ years	0.275	766.175	91	Grand Index
40	m	17+ years	0.278	767	91	Grand Index
40	m	17+ years	0.279	767.763712	91	Grand Index
40	m	17+ years	0.281	768	91	Grand Index
40	m	17+ years	0.282	768.0016109	91	Grand Index
40	m	17+ years	0.283	768.6732649	91	Grand Index
40	m	17+ years	0.288	769	92	Grand Index
40	m	17+ years	0.289	770	92	Grand Index
40	m	17+ years	0.29	770.6733621	92	Grand Index
40	m	17+ years	0.292	771	92	Grand Index
40	m	17+ years	0.293	771.0014536	92	Grand Index
40	m	17+ years	0.294	771.1769813	92	Grand Index
40	m	17+ years	0.295	771.9431807	92	Grand Index
40	m	17+ years	0.297	772	92	Grand Index
40	m	17+ years	0.298	772.226	92	Grand Index
40	m	17+ years	0.301	773	92	Grand Index
40	m	17+ years	0.302	773.7250858	92	Grand Index
40	m	17+ years	0.308	774	92	Grand Index
40	m	17+ years	0.309	774.007261	93	Grand Index
40	m	17+ years	0.31	774.3793033	93	Grand Index

Age	Gender	Education	Percentile	Raw Score	Norm Score	Assessment
40	m	17+ years	0.311	774.9589786	93	Grand Index
40	m	17+ years	0.313	775	93	Grand Index
40	m	17+ years	0.314	775.1017562	93	Grand Index
40	m	17+ years	0.315	775.8160362	93	Grand Index
40	m	17+ years	0.318	776	93	Grand Index
40	m	17+ years	0.319	776.403	93	Grand Index
40	m	17+ years	0.322	777	93	Grand Index
40	m	17+ years	0.323	777.151	93	Grand Index
40	m	17+ years	0.326	778	93	Grand Index
40	m	17+ years	0.327	778.8965272	93	Grand Index
40	m	17+ years	0.331	779	93	Grand Index
40	m	17+ years	0.332	779.5024936	93	Grand Index
40	m	17+ years	0.333	779.9377395	94	Grand Index
40	m	17+ years	0.34	780	94	Grand Index
40	m	17+ years	0.346	781	94	Grand Index
40	m	17+ years	0.347	781.0001667	94	Grand Index
40	m	17+ years	0.351	782	94	Grand Index
40	m	17+ years	0.352	782.824	94	Grand Index
40	m	17+ years	0.357	783	95	Grand Index
40	m	17+ years	0.358	783.0120813	95	Grand Index
40	m	17+ years	0.359	783.8680632	95	Grand Index
40	m	17+ years	0.364	784	95	Grand Index
40	m	17+ years	0.365	784.505	95	Grand Index
40	m	17+ years	0.37	785	95	Grand Index
40	m	17+ years	0.371	785.7826294	95	Grand Index
40	m	17+ years	0.372	785.999604	95	Grand Index
40	m	17+ years	0.377	786	95	Grand Index
40	m	17+ years	0.378	786.0451874	95	Grand Index
40	m	17+ years	0.379	786.3924659	95	Grand Index
40	m	17+ years	0.383	787	96	Grand Index
40	m	17+ years	0.384	787.0510482	96	Grand Index
40	m	17+ years	0.387	788	96	Grand Index
40	m	17+ years	0.388	788.556	96	Grand Index
40	m	17+ years	0.395	789	96	Grand Index
40	m	17+ years	0.396	789.2349702	96	Grand Index
40	m	17+ years	0.398	790	96	Grand Index
40	m	17+ years	0.399	790.0356843	96	Grand Index
40	m	17+ years	0.4	790.8196608	96	Grand Index
40	m	17+ years	0.402	791	96	Grand Index
40	m	17+ years	0.403	791.3265638	96	Grand Index
40	m	17+ years	0.407	792	96	Grand Index
40	m	17+ years	0.41	793	97	Grand Index
40	m	17+ years	0.411	793.0148935	97	Grand Index
40	m	17+ years	0.412	793.0454454	97	Grand Index
40	m	17+ years	0.413	793.4704744	97	Grand Index
40	m	17+ years	0.414	793.9812667	97	Grand Index
40	m	17+ years	0.418	794	97	Grand Index
40	m	17+ years	0.42	795	97	Grand Index
40	m	17+ years	0.421	795.5297789	97	Grand Index
40	m	17+ years	0.422	795.9512386	97	Grand Index
40	m	17+ years	0.427	796	97	Grand Index
40	m	17+ years	0.431	797	97	Grand Index
40	m	17+ years	0.432	797.7204192	97	Grand Index
40	m	17+ years	0.435	798	98	Grand Index
40	m	17+ years	0.436	798.3576414	98	Grand Index
40	m	17+ years	0.437	798.98984	98	Grand Index

Age	Gender	Education	Percentile	Raw Score	Norm Score	Assessment
40	m	17+ years	0.44	799	98	Grand Index
40	m	17+ years	0.441	799.7041655	98	Grand Index
40	m	17+ years	0.445	800	98	Grand Index
40	m	17+ years	0.446	800.902	98	Grand Index
40	m	17+ years	0.448	801	98	Grand Index
40	m	17+ years	0.449	801.093758	98	Grand Index
40	m	17+ years	0.45	801.8040625	98	Grand Index
40	m	17+ years	0.454	802	98	Grand Index
40	m	17+ years	0.455	802.835	98	Grand Index
40	m	17+ years	0.458	804	98	Grand Index
40	m	17+ years	0.459	804.583	98	Grand Index
40	m	17+ years	0.464	805	99	Grand Index
40	m	17+ years	0.466	806	99	Grand Index
40	m	17+ years	0.467	806.0054583	99	Grand Index
40	m	17+ years	0.468	806.5494408	99	Grand Index
40	m	17+ years	0.469	807	99	Grand Index
40	m	17+ years	0.47	807.1906298	99	Grand Index
40	m	17+ years	0.471	807.9115614	99	Grand Index
40	m	17+ years	0.477	808	99	Grand Index
40	m	17+ years	0.478	808.0085068	99	Grand Index
40	m	17+ years	0.479	808.913793	99	Grand Index
40	m	17+ years	0.483	809	99	Grand Index
40	m	17+ years	0.484	809.4457909	99	Grand Index
40	m	17+ years	0.485	809.9932648	99	Grand Index
40	m	17+ years	0.489	810	100	Grand Index
40	m	17+ years	0.49	810.8610595	100	Grand Index
40	m	17+ years	0.495	811	100	Grand Index
40	m	17+ years	0.496	811.3443396	100	Grand Index
40	m	17+ years	0.501	812	100	Grand Index
40	m	17+ years	0.507	813	100	Grand Index
40	m	17+ years	0.508	813.996	100	Grand Index
40	m	17+ years	0.509	814	100	Grand Index
40	m	17+ years	0.51	814.8422743	100	Grand Index
40	m	17+ years	0.515	815	101	Grand Index
40	m	17+ years	0.516	815.492	101	Grand Index
40	m	17+ years	0.518	816	101	Grand Index
40	m	17+ years	0.519	816.1697372	101	Grand Index
40	m	17+ years	0.52	816.8222127	101	Grand Index
40	m	17+ years	0.526	817	101	Grand Index
40	m	17+ years	0.528	818	101	Grand Index
40	m	17+ years	0.529	818.1299329	101	Grand Index
40	m	17+ years	0.53	818.7286823	101	Grand Index
40	m	17+ years	0.536	819	101	Grand Index
40	m	17+ years	0.537	819.099151	101	Grand Index
40	m	17+ years	0.54	820	102	Grand Index
40	m	17+ years	0.544	821	102	Grand Index
40	m	17+ years	0.545	821.3043201	102	Grand Index
40	m	17+ years	0.546	822	102	Grand Index
40	m	17+ years	0.552	823	102	Grand Index
40	m	17+ years	0.553	823.658484	102	Grand Index
40	m	17+ years	0.555	824	102	Grand Index
40	m	17+ years	0.556	824.2030576	102	Grand Index
40	m	17+ years	0.56	825	102	Grand Index
40	m	17+ years	0.565	826	102	Grand Index
40	m	17+ years	0.566	826.4028792	102	Grand Index
40	m	17+ years	0.569	827	103	Grand Index

Age	Gender	Education	Percentile	Raw Score	Norm Score	Assessment
40	m	17+ years	0.57	827.0182596	103	Grand Index
40	m	17+ years	0.571	827.6229645	103	Grand Index
40	m	17+ years	0.577	828	103	Grand Index
40	m	17+ years	0.578	828.586	103	Grand Index
40	m	17+ years	0.582	829	103	Grand Index
40	m	17+ years	0.583	829.771	103	Grand Index
40	m	17+ years	0.585	830	103	Grand Index
40	m	17+ years	0.586	830.0809639	103	Grand Index
40	m	17+ years	0.587	830.9203104	103	Grand Index
40	m	17+ years	0.592	831	103	Grand Index
40	m	17+ years	0.595	832	104	Grand Index
40	m	17+ years	0.596	832.452	104	Grand Index
40	m	17+ years	0.599	833	104	Grand Index
40	m	17+ years	0.604	834	104	Grand Index
40	m	17+ years	0.609	835	104	Grand Index
40	m	17+ years	0.61	835.57	104	Grand Index
40	m	17+ years	0.614	836	104	Grand Index
40	m	17+ years	0.615	836.755	104	Grand Index
40	m	17+ years	0.62	837	105	Grand Index
40	m	17+ years	0.621	837.5076574	105	Grand Index
40	m	17+ years	0.625	838	105	Grand Index
40	m	17+ years	0.626	838.562	105	Grand Index
40	m	17+ years	0.628	839	105	Grand Index
40	m	17+ years	0.629	839.8429794	105	Grand Index
40	m	17+ years	0.633	840	105	Grand Index
40	m	17+ years	0.634	840.0783449	105	Grand Index
40	m	17+ years	0.638	841	105	Grand Index
40	m	17+ years	0.639	841.017029	105	Grand Index
40	m	17+ years	0.64	841.452014	105	Grand Index
40	m	17+ years	0.641	841.9947174	105	Grand Index
40	m	17+ years	0.643	842	105	Grand Index
40	m	17+ years	0.644	842.428	106	Grand Index
40	m	17+ years	0.649	843	106	Grand Index
40	m	17+ years	0.65	843.05	106	Grand Index
40	m	17+ years	0.654	844	106	Grand Index
40	m	17+ years	0.658	845	106	Grand Index
40	m	17+ years	0.659	845.983	106	Grand Index
40	m	17+ years	0.662	846	106	Grand Index
40	m	17+ years	0.663	846.4395095	106	Grand Index
40	m	17+ years	0.668	847	107	Grand Index
40	m	17+ years	0.669	847.4586149	107	Grand Index
40	m	17+ years	0.671	848	107	Grand Index
40	m	17+ years	0.672	848.664	107	Grand Index
40	m	17+ years	0.68	849	107	Grand Index
40	m	17+ years	0.681	849.4727537	107	Grand Index
40	m	17+ years	0.684	850	107	Grand Index
40	m	17+ years	0.685	850.4396607	107	Grand Index
40	m	17+ years	0.688	851	107	Grand Index
40	m	17+ years	0.689	852.093	107	Grand Index
40	m	17+ years	0.694	853	108	Grand Index
40	m	17+ years	0.695	853.715	108	Grand Index
40	m	17+ years	0.7	854	108	Grand Index
40	m	17+ years	0.701	854.2119295	108	Grand Index
40	m	17+ years	0.702	854.9151748	108	Grand Index
40	m	17+ years	0.707	855	108	Grand Index
40	m	17+ years	0.708	855.4117863	108	Grand Index

Age	Gender	Education	Percentile	Raw Score	Norm Score	Assessment
40	m	17+ years	0.713	856	108	Grand Index
40	m	17+ years	0.714	856.05053	108	Grand Index
40	m	17+ years	0.718	857	109	Grand Index
40	m	17+ years	0.719	857.1623398	109	Grand Index
40	m	17+ years	0.72	857.9049432	109	Grand Index
40	m	17+ years	0.724	858	109	Grand Index
40	m	17+ years	0.725	858.7966287	109	Grand Index
40	m	17+ years	0.732	859	109	Grand Index
40	m	17+ years	0.733	859.1566845	109	Grand Index
40	m	17+ years	0.734	859.8761235	109	Grand Index
40	m	17+ years	0.738	860	110	Grand Index
40	m	17+ years	0.739	860.943	110	Grand Index
40	m	17+ years	0.741	861	110	Grand Index
40	m	17+ years	0.742	861.254	110	Grand Index
40	m	17+ years	0.743	862	110	Grand Index
40	m	17+ years	0.744	862.128	110	Grand Index
40	m	17+ years	0.748	863	110	Grand Index
40	m	17+ years	0.752	864	110	Grand Index
40	m	17+ years	0.753	864.016771	110	Grand Index
40	m	17+ years	0.754	864.3121312	110	Grand Index
40	m	17+ years	0.755	864.8757005	110	Grand Index
40	m	17+ years	0.758	865	110	Grand Index
40	m	17+ years	0.759	865.683	111	Grand Index
40	m	17+ years	0.763	866	111	Grand Index
40	m	17+ years	0.764	866.8514544	111	Grand Index
40	m	17+ years	0.768	867	111	Grand Index
40	m	17+ years	0.769	867.0004814	111	Grand Index
40	m	17+ years	0.77	867.494632	111	Grand Index
40	m	17+ years	0.772	868	111	Grand Index
40	m	17+ years	0.773	868.7488223	111	Grand Index
40	m	17+ years	0.778	869	111	Grand Index
40	m	17+ years	0.779	869.423	112	Grand Index
40	m	17+ years	0.781	870	112	Grand Index
40	m	17+ years	0.782	870.734	112	Grand Index
40	m	17+ years	0.783	871	112	Grand Index
40	m	17+ years	0.784	871.4327617	112	Grand Index
40	m	17+ years	0.785	871.9818229	112	Grand Index
40	m	17+ years	0.789	872	112	Grand Index
40	m	17+ years	0.79	872.1869492	112	Grand Index
40	m	17+ years	0.791	872.9376699	112	Grand Index
40	m	17+ years	0.792	873	112	Grand Index
40	m	17+ years	0.793	873.541	112	Grand Index
40	m	17+ years	0.796	874	112	Grand Index
40	m	17+ years	0.797	874.4825281	112	Grand Index
40	m	17+ years	0.803	875	113	Grand Index
40	m	17+ years	0.804	875.0982972	113	Grand Index
40	m	17+ years	0.805	875.8457296	113	Grand Index
40	m	17+ years	0.806	876	113	Grand Index
40	m	17+ years	0.807	876.659	113	Grand Index
40	m	17+ years	0.813	877	113	Grand Index
40	m	17+ years	0.814	877.1624635	113	Grand Index
40	m	17+ years	0.815	877.8912968	113	Grand Index
40	m	17+ years	0.817	878	114	Grand Index
40	m	17+ years	0.818	878.0121574	114	Grand Index
40	m	17+ years	0.819	878.368255	114	Grand Index
40	m	17+ years	0.821	879	114	Grand Index

Age	Gender	Education	Percentile	Raw Score	Norm Score	Assessment
40	m	17+ years	0.822	879.2040886	114	Grand Index
40	m	17+ years	0.823	879.9838361	114	Grand Index
40	m	17+ years	0.826	880	114	Grand Index
40	m	17+ years	0.827	880.3585549	114	Grand Index
40	m	17+ years	0.828	880.983376	114	Grand Index
40	m	17+ years	0.831	881	114	Grand Index
40	m	17+ years	0.832	881.584	114	Grand Index
40	m	17+ years	0.835	882	115	Grand Index
40	m	17+ years	0.836	883	115	Grand Index
40	m	17+ years	0.837	883.180646	115	Grand Index
40	m	17+ years	0.84	884	115	Grand Index
40	m	17+ years	0.841	884.517	115	Grand Index
40	m	17+ years	0.842	885.9379634	115	Grand Index
40	m	17+ years	0.844	886	115	Grand Index
40	m	17+ years	0.846	887	115	Grand Index
40	m	17+ years	0.849	888	115	Grand Index
40	m	17+ years	0.85	888.45	116	Grand Index
40	m	17+ years	0.851	889.1341117	116	Grand Index
40	m	17+ years	0.852	889.5232947	116	Grand Index
40	m	17+ years	0.853	890.761	116	Grand Index
40	m	17+ years	0.854	891	116	Grand Index
40	m	17+ years	0.855	891.3140512	116	Grand Index
40	m	17+ years	0.858	892	116	Grand Index
40	m	17+ years	0.859	892.383	116	Grand Index
40	m	17+ years	0.86	893	116	Grand Index
40	m	17+ years	0.862	894	116	Grand Index
40	m	17+ years	0.864	895	116	Grand Index
40	m	17+ years	0.865	895.1850669	117	Grand Index
40	m	17+ years	0.866	896.442	117	Grand Index
40	m	17+ years	0.867	897.4603676	117	Grand Index
40	m	17+ years	0.869	898	117	Grand Index
40	m	17+ years	0.872	899	117	Grand Index
40	m	17+ years	0.873	899.501	117	Grand Index
40	m	17+ years	0.875	900	117	Grand Index
40	m	17+ years	0.876	900.812	117	Grand Index
40	m	17+ years	0.877	901	117	Grand Index
40	m	17+ years	0.878	902.372	117	Grand Index
40	m	17+ years	0.881	903	118	Grand Index
40	m	17+ years	0.882	903.6301172	118	Grand Index
40	m	17+ years	0.883	903.9690272	118	Grand Index
40	m	17+ years	0.884	905	118	Grand Index
40	m	17+ years	0.885	905.745	118	Grand Index
40	m	17+ years	0.886	906.182	118	Grand Index
40	m	17+ years	0.887	907.2959544	118	Grand Index
40	m	17+ years	0.89	908	118	Grand Index
40	m	17+ years	0.891	909.367	118	Grand Index
40	m	17+ years	0.894	910	119	Grand Index
40	m	17+ years	0.896	911	119	Grand Index
40	m	17+ years	0.897	911.989	119	Grand Index
40	m	17+ years	0.899	912	119	Grand Index
40	m	17+ years	0.901	913	119	Grand Index
40	m	17+ years	0.903	914	119	Grand Index
40	m	17+ years	0.904	915	120	Grand Index
40	m	17+ years	0.905	915.485	120	Grand Index
40	m	17+ years	0.906	916	120	Grand Index
40	m	17+ years	0.907	916.359	120	Grand Index

Age	Gender	Education	Percentile	Raw Score	Norm Score	Assessment
40	m	17+ years	0.909	917	120	Grand Index
40	m	17+ years	0.91	917.637124	120	Grand Index
40	m	17+ years	0.911	918	120	Grand Index
40	m	17+ years	0.912	918.544	120	Grand Index
40	m	17+ years	0.913	919.871199	120	Grand Index
40	m	17+ years	0.915	920	121	Grand Index
40	m	17+ years	0.916	921.292	121	Grand Index
40	m	17+ years	0.917	922	121	Grand Index
40	m	17+ years	0.919	923	121	Grand Index
40	m	17+ years	0.92	923.0245055	121	Grand Index
40	m	17+ years	0.921	923.7974093	121	Grand Index
40	m	17+ years	0.922	924.914	121	Grand Index
40	m	17+ years	0.923	925.351	121	Grand Index
40	m	17+ years	0.924	926	121	Grand Index
40	m	17+ years	0.925	926.225	122	Grand Index
40	m	17+ years	0.927	927	122	Grand Index
40	m	17+ years	0.928	927.536	122	Grand Index
40	m	17+ years	0.929	928	122	Grand Index
40	m	17+ years	0.93	928.41	122	Grand Index
40	m	17+ years	0.931	929	122	Grand Index
40	m	17+ years	0.932	929.3427386	122	Grand Index
40	m	17+ years	0.933	930.4262663	122	Grand Index
40	m	17+ years	0.934	932.03119	123	Grand Index
40	m	17+ years	0.935	933.2000102	123	Grand Index
40	m	17+ years	0.937	934	123	Grand Index
40	m	17+ years	0.938	934.4397794	123	Grand Index
40	m	17+ years	0.939	934.8044969	123	Grand Index
40	m	17+ years	0.94	935.78	123	Grand Index
40	m	17+ years	0.941	936.9618049	123	Grand Index
40	m	17+ years	0.942	937.654	124	Grand Index
40	m	17+ years	0.943	939	124	Grand Index
40	m	17+ years	0.944	939.2468625	124	Grand Index
40	m	17+ years	0.945	940.1146085	124	Grand Index
40	m	17+ years	0.946	940.587978	124	Grand Index
40	m	17+ years	0.947	941	124	Grand Index
40	m	17+ years	0.948	941.2059022	124	Grand Index
40	m	17+ years	0.949	942.1985754	125	Grand Index
40	m	17+ years	0.95	942.8334917	125	Grand Index
40	m	17+ years	0.951	943	125	Grand Index
40	m	17+ years	0.952	943.0589355	125	Grand Index
40	m	17+ years	0.953	943.4982426	125	Grand Index
40	m	17+ years	0.954	943.9510939	125	Grand Index
40	m	17+ years	0.956	947	126	Grand Index
40	m	17+ years	0.957	947.7491674	126	Grand Index
40	m	17+ years	0.958	948.052898	126	Grand Index
40	m	17+ years	0.959	949	126	Grand Index
40	m	17+ years	0.96	950.04	126	Grand Index
40	m	17+ years	0.961	951	126	Grand Index
40	m	17+ years	0.962	953.3509694	127	Grand Index
40	m	17+ years	0.963	953.9815427	127	Grand Index
40	m	17+ years	0.964	954.2221498	127	Grand Index
40	m	17+ years	0.965	954.9495306	127	Grand Index
40	m	17+ years	0.966	955.142	127	Grand Index
40	m	17+ years	0.967	956	128	Grand Index
40	m	17+ years	0.968	958.048	128	Grand Index
40	m	17+ years	0.969	961.906	128	Grand Index

Age	Gender	Education	Percentile	Raw Score	Norm Score	Assessment
40	m	17+ years	0.97	963	128	Grand Index
40	m	17+ years	0.971	963.4047802	128	Grand Index
40	m	17+ years	0.972	964.3318235	129	Grand Index
40	m	17+ years	0.973	964.7687514	129	Grand Index
40	m	17+ years	0.974	965.6442661	129	Grand Index
40	m	17+ years	0.975	967.0532294	129	Grand Index
40	m	17+ years	0.976	968.3703459	130	Grand Index
40	m	17+ years	0.977	969.0648996	130	Grand Index
40	m	17+ years	0.978	971.197369	130	Grand Index
40	m	17+ years	0.979	972	131	Grand Index
40	m	17+ years	0.98	973.26	131	Grand Index
40	m	17+ years	0.981	975.0075565	131	Grand Index
40	m	17+ years	0.982	976.134	131	Grand Index
40	m	17+ years	0.983	977.1338617	132	Grand Index
40	m	17+ years	0.984	978.4123317	132	Grand Index
40	m	17+ years	0.985	979.9794045	133	Grand Index
40	m	17+ years	0.986	981.6594573	133	Grand Index
40	m	17+ years	0.987	984.8570323	133	Grand Index
40	m	17+ years	0.988	991.4560424	134	Grand Index
40	m	17+ years	0.989	999.7276584	134	Grand Index
40	m	17+ years	0.99	1004.804993	135	Grand Index
40	m	17+ years	0.991	1006.826659	135	Grand Index
40	m	17+ years	0.992	1007.781952	136	Grand Index
40	m	17+ years	0.993	1008.51907	137	Grand Index
40	m	17+ years	0.994	1009.416217	138	Grand Index
40	m	17+ years	0.995	1012.463704	139	Grand Index
40	m	17+ years	0.996	1013.719446	140	Grand Index
40	m	17+ years	0.997	1017.332888	141	Grand Index
40	m	17+ years	0.998	1018.671192	143	Grand Index
40	m	17+ years	0.999	1026.698179	146	Grand Index
45	f	0-12 years	0.001	572.4132733	54	Grand Index
45	f	0-12 years	0.002	594.8200261	57	Grand Index
45	f	0-12 years	0.003	610.314	59	Grand Index
45	f	0-12 years	0.004	625.752	60	Grand Index
45	f	0-12 years	0.005	628.0738115	61	Grand Index
45	f	0-12 years	0.006	630.1814812	62	Grand Index
45	f	0-12 years	0.007	635.465386	63	Grand Index
45	f	0-12 years	0.008	640.8277163	64	Grand Index
45	f	0-12 years	0.009	642.4539679	65	Grand Index
45	f	0-12 years	0.01	643.9562002	65	Grand Index
45	f	0-12 years	0.011	645.9588502	66	Grand Index
45	f	0-12 years	0.012	647.9824381	66	Grand Index
45	f	0-12 years	0.013	648.5623031	67	Grand Index
45	f	0-12 years	0.014	649.0690682	67	Grand Index
45	f	0-12 years	0.015	652.3870085	67	Grand Index
45	f	0-12 years	0.016	655.8724232	68	Grand Index
45	f	0-12 years	0.017	656.9834699	68	Grand Index
45	f	0-12 years	0.018	657.9314699	69	Grand Index
45	f	0-12 years	0.022	658	70	Grand Index
45	f	0-12 years	0.023	658.7377061	70	Grand Index
45	f	0-12 years	0.024	659.546908	70	Grand Index
45	f	0-12 years	0.025	661.1833277	71	Grand Index
45	f	0-12 years	0.026	662.907984	71	Grand Index
45	f	0-12 years	0.027	663.49479	71	Grand Index
45	f	0-12 years	0.028	663.9489318	71	Grand Index
45	f	0-12 years	0.029	665.326	72	Grand Index

Age	Gender	Education	Percentile	Raw Score	Norm Score	Assessment
45	f	0-12 years	0.03	666.82	72	Grand Index
45	f	0-12 years	0.031	667.438	72	Grand Index
45	f	0-12 years	0.032	667.936	72	Grand Index
45	f	0-12 years	0.033	668.1456933	72	Grand Index
45	f	0-12 years	0.034	668.3128713	73	Grand Index
45	f	0-12 years	0.035	669.4813483	73	Grand Index
45	f	0-12 years	0.036	670.8081703	73	Grand Index
45	f	0-12 years	0.037	671.852	73	Grand Index
45	f	0-12 years	0.038	672.848	73	Grand Index
45	f	0-12 years	0.039	673.422	74	Grand Index
45	f	0-12 years	0.04	673.92	74	Grand Index
45	f	0-12 years	0.041	674.836	74	Grand Index
45	f	0-12 years	0.042	675.832	74	Grand Index
45	f	0-12 years	0.043	678.07	74	Grand Index
45	f	0-12 years	0.044	680.56	74	Grand Index
45	f	0-12 years	0.045	681.82	75	Grand Index
45	f	0-12 years	0.046	682.816	75	Grand Index
45	f	0-12 years	0.047	683.812	75	Grand Index
45	f	0-12 years	0.048	684.808	75	Grand Index
45	f	0-12 years	0.049	687.01	75	Grand Index
45	f	0-12 years	0.05	689.5	75	Grand Index
45	f	0-12 years	0.052	690	76	Grand Index
45	f	0-12 years	0.053	690.394	76	Grand Index
45	f	0-12 years	0.054	690.892	76	Grand Index
45	f	0-12 years	0.055	691.39	76	Grand Index
45	f	0-12 years	0.056	691.888	76	Grand Index
45	f	0-12 years	0.057	692.386	76	Grand Index
45	f	0-12 years	0.058	692.884	76	Grand Index
45	f	0-12 years	0.059	693.382	77	Grand Index
45	f	0-12 years	0.06	693.88	77	Grand Index
45	f	0-12 years	0.061	695.3903169	77	Grand Index
45	f	0-12 years	0.062	697.2220043	77	Grand Index
45	f	0-12 years	0.063	697.7984825	77	Grand Index
45	f	0-12 years	0.064	697.9587951	77	Grand Index
45	f	0-12 years	0.065	699.11	77	Grand Index
45	f	0-12 years	0.066	700.604	77	Grand Index
45	f	0-12 years	0.068	701	78	Grand Index
45	f	0-12 years	0.069	701.0651309	78	Grand Index
45	f	0-12 years	0.07	701.1547309	78	Grand Index
45	f	0-12 years	0.071	701.4735084	78	Grand Index
45	f	0-12 years	0.072	701.8819084	78	Grand Index
45	f	0-12 years	0.073	702.0211327	78	Grand Index
45	f	0-12 years	0.074	702.0508617	78	Grand Index
45	f	0-12 years	0.075	703.0888029	78	Grand Index
45	f	0-12 years	0.076	704.5530739	79	Grand Index
45	f	0-12 years	0.077	705.346	79	Grand Index
45	f	0-12 years	0.078	705.844	79	Grand Index
45	f	0-12 years	0.08	706	79	Grand Index
45	f	0-12 years	0.081	707.352	79	Grand Index
45	f	0-12 years	0.082	709.344	79	Grand Index
45	f	0-12 years	0.086	710	80	Grand Index
45	f	0-12 years	0.087	710.652	80	Grand Index
45	f	0-12 years	0.088	711.648	80	Grand Index
45	f	0-12 years	0.09	712	80	Grand Index
45	f	0-12 years	0.091	712.318	80	Grand Index
45	f	0-12 years	0.092	712.816	80	Grand Index

Age	Gender	Education	Percentile	Raw Score	Norm Score	Assessment
45	f	0-12 years	0.093	713.4687409	80	Grand Index
45	f	0-12 years	0.094	714.2121579	80	Grand Index
45	f	0-12 years	0.095	714.6500357	80	Grand Index
45	f	0-12 years	0.096	714.9026186	80	Grand Index
45	f	0-12 years	0.098	715	81	Grand Index
45	f	0-12 years	0.099	715.302	81	Grand Index
45	f	0-12 years	0.1	715.8	81	Grand Index
45	f	0-12 years	0.101	716.6046478	81	Grand Index
45	f	0-12 years	0.102	717.6150995	81	Grand Index
45	f	0-12 years	0.103	718.3144877	81	Grand Index
45	f	0-12 years	0.104	718.798036	81	Grand Index
45	f	0-12 years	0.105	719.29	81	Grand Index
45	f	0-12 years	0.106	719.788	81	Grand Index
45	f	0-12 years	0.107	720.572	81	Grand Index
45	f	0-12 years	0.108	721.568	81	Grand Index
45	f	0-12 years	0.109	722.846	82	Grand Index
45	f	0-12 years	0.11	724.34	82	Grand Index
45	f	0-12 years	0.111	725.278	82	Grand Index
45	f	0-12 years	0.112	725.776	82	Grand Index
45	f	0-12 years	0.113	726.274	82	Grand Index
45	f	0-12 years	0.114	726.772	82	Grand Index
45	f	0-12 years	0.115	727.27	82	Grand Index
45	f	0-12 years	0.116	727.768	82	Grand Index
45	f	0-12 years	0.117	728.532	82	Grand Index
45	f	0-12 years	0.118	729.528	82	Grand Index
45	f	0-12 years	0.119	730.262	82	Grand Index
45	f	0-12 years	0.12	730.76	82	Grand Index
45	f	0-12 years	0.121	731.258	82	Grand Index
45	f	0-12 years	0.122	731.756	83	Grand Index
45	f	0-12 years	0.123	732.508	83	Grand Index
45	f	0-12 years	0.124	733.504	83	Grand Index
45	f	0-12 years	0.125	734.25	83	Grand Index
45	f	0-12 years	0.126	734.748	83	Grand Index
45	f	0-12 years	0.13	735	83	Grand Index
45	f	0-12 years	0.131	735.238	83	Grand Index
45	f	0-12 years	0.132	735.736	83	Grand Index
45	f	0-12 years	0.133	736.234	83	Grand Index
45	f	0-12 years	0.134	736.732	83	Grand Index
45	f	0-12 years	0.135	737.23	83	Grand Index
45	f	0-12 years	0.136	737.728	84	Grand Index
45	f	0-12 years	0.142	738	84	Grand Index
45	f	0-12 years	0.143	738.0685222	84	Grand Index
45	f	0-12 years	0.144	738.2279803	84	Grand Index
45	f	0-12 years	0.145	738.4629557	84	Grand Index
45	f	0-12 years	0.146	738.8014975	84	Grand Index
45	f	0-12 years	0.148	739	84	Grand Index
45	f	0-12 years	0.149	739.202	84	Grand Index
45	f	0-12 years	0.15	739.7	84	Grand Index
45	f	0-12 years	0.158	740	85	Grand Index
45	f	0-12 years	0.159	740.0547496	85	Grand Index
45	f	0-12 years	0.16	740.2045589	85	Grand Index
45	f	0-12 years	0.161	740.4376792	85	Grand Index
45	f	0-12 years	0.162	740.8205721	85	Grand Index
45	f	0-12 years	0.163	741.0767741	85	Grand Index
45	f	0-12 years	0.164	741.0970688	85	Grand Index
45	f	0-12 years	0.165	741.4316616	85	Grand Index

Age	Gender	Education	Percentile	Raw Score	Norm Score	Assessment
45	f	0-12 years	0.166	742.3726646	85	Grand Index
45	f	0-12 years	0.167	743.1627358	86	Grand Index
45	f	0-12 years	0.168	743.6509434	86	Grand Index
45	f	0-12 years	0.169	743.9835219	86	Grand Index
45	f	0-12 years	0.17	743.9933144	86	Grand Index
45	f	0-12 years	0.171	744.158	86	Grand Index
45	f	0-12 years	0.172	744.656	86	Grand Index
45	f	0-12 years	0.174	745	86	Grand Index
45	f	0-12 years	0.175	745.0029396	86	Grand Index
45	f	0-12 years	0.176	745.0126992	86	Grand Index
45	f	0-12 years	0.177	745.1627363	86	Grand Index
45	f	0-12 years	0.178	745.6509767	86	Grand Index
45	f	0-12 years	0.18	746	86	Grand Index
45	f	0-12 years	0.181	746.0061141	86	Grand Index
45	f	0-12 years	0.182	746.0281779	86	Grand Index
45	f	0-12 years	0.183	746.172368	86	Grand Index
45	f	0-12 years	0.184	746.6483042	86	Grand Index
45	f	0-12 years	0.186	747	87	Grand Index
45	f	0-12 years	0.187	747.252	87	Grand Index
45	f	0-12 years	0.188	748.248	87	Grand Index
45	f	0-12 years	0.192	749	87	Grand Index
45	f	0-12 years	0.193	749.114	87	Grand Index
45	f	0-12 years	0.194	749.612	87	Grand Index
45	f	0-12 years	0.195	750.0016324	87	Grand Index
45	f	0-12 years	0.196	750.0090226	87	Grand Index
45	f	0-12 years	0.197	750.1520718	87	Grand Index
45	f	0-12 years	0.198	750.7968029	87	Grand Index
45	f	0-12 years	0.199	751.4819136	87	Grand Index
45	f	0-12 years	0.2	752.3237923	87	Grand Index
45	f	0-12 years	0.202	753	87	Grand Index
45	f	0-12 years	0.203	753.0796801	88	Grand Index
45	f	0-12 years	0.204	753.5018154	88	Grand Index
45	f	0-12 years	0.205	753.8613716	88	Grand Index
45	f	0-12 years	0.206	753.9372364	88	Grand Index
45	f	0-12 years	0.208	754	88	Grand Index
45	f	0-12 years	0.209	754.082	88	Grand Index
45	f	0-12 years	0.21	754.58	88	Grand Index
45	f	0-12 years	0.211	755.0550329	88	Grand Index
45	f	0-12 years	0.212	755.4063966	88	Grand Index
45	f	0-12 years	0.213	755.727339	88	Grand Index
45	f	0-12 years	0.214	755.8739753	88	Grand Index
45	f	0-12 years	0.216	756	88	Grand Index
45	f	0-12 years	0.217	756.066	88	Grand Index
45	f	0-12 years	0.218	756.564	88	Grand Index
45	f	0-12 years	0.219	757.062	88	Grand Index
45	f	0-12 years	0.22	757.56	88	Grand Index
45	f	0-12 years	0.226	758	89	Grand Index
45	f	0-12 years	0.227	758.046	89	Grand Index
45	f	0-12 years	0.228	758.544	89	Grand Index
45	f	0-12 years	0.234	759	89	Grand Index
45	f	0-12 years	0.235	759.06	89	Grand Index
45	f	0-12 years	0.236	760.056	89	Grand Index
45	f	0-12 years	0.238	761	89	Grand Index
45	f	0-12 years	0.239	761.022	89	Grand Index
45	f	0-12 years	0.24	761.52	89	Grand Index
45	f	0-12 years	0.241	762.0004016	89	Grand Index

Age	Gender	Education	Percentile	Raw Score	Norm Score	Assessment
45	f	0-12 years	0.242	762.011513	90	Grand Index
45	f	0-12 years	0.243	762.0359996	90	Grand Index
45	f	0-12 years	0.244	762.5228882	90	Grand Index
45	f	0-12 years	0.245	763.01	90	Grand Index
45	f	0-12 years	0.246	763.508	90	Grand Index
45	f	0-12 years	0.247	764.006	90	Grand Index
45	f	0-12 years	0.248	764.504	90	Grand Index
45	f	0-12 years	0.249	765.0006974	90	Grand Index
45	f	0-12 years	0.25	765.1743375	90	Grand Index
45	f	0-12 years	0.251	765.3479777	90	Grand Index
45	f	0-12 years	0.252	765.6717322	90	Grand Index
45	f	0-12 years	0.253	765.9960921	90	Grand Index
45	f	0-12 years	0.257	766	90	Grand Index
45	f	0-12 years	0.258	766.484	90	Grand Index
45	f	0-12 years	0.259	766.982	90	Grand Index
45	f	0-12 years	0.265	767	91	Grand Index
45	f	0-12 years	0.266	767.468	91	Grand Index
45	f	0-12 years	0.267	767.966	91	Grand Index
45	f	0-12 years	0.275	768	91	Grand Index
45	f	0-12 years	0.276	768.448	91	Grand Index
45	f	0-12 years	0.277	768.946	91	Grand Index
45	f	0-12 years	0.281	769	91	Grand Index
45	f	0-12 years	0.282	769.436	91	Grand Index
45	f	0-12 years	0.283	769.934	91	Grand Index
45	f	0-12 years	0.284	770.1054337	91	Grand Index
45	f	0-12 years	0.285	770.2269753	91	Grand Index
45	f	0-12 years	0.286	770.567602	92	Grand Index
45	f	0-12 years	0.287	770.9440604	92	Grand Index
45	f	0-12 years	0.291	771	92	Grand Index
45	f	0-12 years	0.292	771.416	92	Grand Index
45	f	0-12 years	0.293	771.914	92	Grand Index
45	f	0-12 years	0.295	772	92	Grand Index
45	f	0-12 years	0.296	772.408	92	Grand Index
45	f	0-12 years	0.297	772.906	92	Grand Index
45	f	0-12 years	0.298	773.404	92	Grand Index
45	f	0-12 years	0.299	773.902	92	Grand Index
45	f	0-12 years	0.301	774	92	Grand Index
45	f	0-12 years	0.302	774.396	92	Grand Index
45	f	0-12 years	0.303	774.894	92	Grand Index
45	f	0-12 years	0.305	775	92	Grand Index
45	f	0-12 years	0.306	775.776	92	Grand Index
45	f	0-12 years	0.307	776.772	92	Grand Index
45	f	0-12 years	0.311	777	93	Grand Index
45	f	0-12 years	0.312	777.376	93	Grand Index
45	f	0-12 years	0.313	777.874	93	Grand Index
45	f	0-12 years	0.315	778	93	Grand Index
45	f	0-12 years	0.316	778.5526073	93	Grand Index
45	f	0-12 years	0.317	779.300429	93	Grand Index
45	f	0-12 years	0.318	779.6830495	93	Grand Index
45	f	0-12 years	0.319	779.9312277	93	Grand Index
45	f	0-12 years	0.325	780	93	Grand Index
45	f	0-12 years	0.326	780.1526165	93	Grand Index
45	f	0-12 years	0.327	780.371016	93	Grand Index
45	f	0-12 years	0.328	780.6316909	93	Grand Index
45	f	0-12 years	0.329	780.9112914	93	Grand Index
45	f	0-12 years	0.333	781	94	Grand Index

Age	Gender	Education	Percentile	Raw Score	Norm Score	Assessment
45	f	0-12 years	0.334	781.0043168	94	Grand Index
45	f	0-12 years	0.335	781.0107921	94	Grand Index
45	f	0-12 years	0.336	781.3367377	94	Grand Index
45	f	0-12 years	0.337	781.8282624	94	Grand Index
45	f	0-12 years	0.338	782.324	94	Grand Index
45	f	0-12 years	0.339	782.822	94	Grand Index
45	f	0-12 years	0.34	783.32	94	Grand Index
45	f	0-12 years	0.341	783.818	94	Grand Index
45	f	0-12 years	0.345	784	94	Grand Index
45	f	0-12 years	0.346	784.0685597	94	Grand Index
45	f	0-12 years	0.347	784.1794127	94	Grand Index
45	f	0-12 years	0.348	784.4589271	94	Grand Index
45	f	0-12 years	0.349	784.8460741	94	Grand Index
45	f	0-12 years	0.353	785	94	Grand Index
45	f	0-12 years	0.354	785.292	94	Grand Index
45	f	0-12 years	0.355	785.79	94	Grand Index
45	f	0-12 years	0.356	786.0982637	94	Grand Index
45	f	0-12 years	0.357	786.268178	95	Grand Index
45	f	0-12 years	0.358	786.5282944	95	Grand Index
45	f	0-12 years	0.359	786.8563802	95	Grand Index
45	f	0-12 years	0.363	787	95	Grand Index
45	f	0-12 years	0.364	787.054507	95	Grand Index
45	f	0-12 years	0.365	787.154303	95	Grand Index
45	f	0-12 years	0.366	787.6335759	95	Grand Index
45	f	0-12 years	0.367	788.4385193	95	Grand Index
45	f	0-12 years	0.368	788.8651248	95	Grand Index
45	f	0-12 years	0.369	788.9563855	95	Grand Index
45	f	0-12 years	0.373	789	95	Grand Index
45	f	0-12 years	0.374	789.2473468	95	Grand Index
45	f	0-12 years	0.375	789.7361512	95	Grand Index
45	f	0-12 years	0.376	789.9861143	95	Grand Index
45	f	0-12 years	0.377	789.9953099	95	Grand Index
45	f	0-12 years	0.379	790	95	Grand Index
45	f	0-12 years	0.38	790.24	95	Grand Index
45	f	0-12 years	0.381	790.738	95	Grand Index
45	f	0-12 years	0.389	791	96	Grand Index
45	f	0-12 years	0.39	791.0053012	96	Grand Index
45	f	0-12 years	0.391	791.0173012	96	Grand Index
45	f	0-12 years	0.392	791.4508916	96	Grand Index
45	f	0-12 years	0.393	792.4348916	96	Grand Index
45	f	0-12 years	0.394	793.212	96	Grand Index
45	f	0-12 years	0.395	793.71	96	Grand Index
45	f	0-12 years	0.397	794	96	Grand Index
45	f	0-12 years	0.398	794.0390129	96	Grand Index
45	f	0-12 years	0.399	794.1342503	96	Grand Index
45	f	0-12 years	0.4	794.3529918	96	Grand Index
45	f	0-12 years	0.401	794.7557544	96	Grand Index
45	f	0-12 years	0.403	795	96	Grand Index
45	f	0-12 years	0.404	795.0215788	96	Grand Index
45	f	0-12 years	0.405	795.0775488	96	Grand Index
45	f	0-12 years	0.406	795.2792604	96	Grand Index
45	f	0-12 years	0.407	795.7212903	96	Grand Index
45	f	0-12 years	0.411	796	97	Grand Index
45	f	0-12 years	0.412	796.176	97	Grand Index
45	f	0-12 years	0.413	796.674	97	Grand Index
45	f	0-12 years	0.419	797	97	Grand Index

Age	Gender	Education	Percentile	Raw Score	Norm Score	Assessment
45	f	0-12 years	0.42	797.16	97	Grand Index
45	f	0-12 years	0.421	797.658	97	Grand Index
45	f	0-12 years	0.423	798	97	Grand Index
45	f	0-12 years	0.424	798.1030393	97	Grand Index
45	f	0-12 years	0.425	798.4406287	97	Grand Index
45	f	0-12 years	0.426	798.7255625	97	Grand Index
45	f	0-12 years	0.427	798.8859732	97	Grand Index
45	f	0-12 years	0.428	799.144	97	Grand Index
45	f	0-12 years	0.429	799.642	97	Grand Index
45	f	0-12 years	0.431	800	97	Grand Index
45	f	0-12 years	0.432	800.136	97	Grand Index
45	f	0-12 years	0.433	800.634	97	Grand Index
45	f	0-12 years	0.435	801	98	Grand Index
45	f	0-12 years	0.436	801.0484242	98	Grand Index
45	f	0-12 years	0.437	801.2368248	98	Grand Index
45	f	0-12 years	0.438	801.4554034	98	Grand Index
45	f	0-12 years	0.439	801.7650028	98	Grand Index
45	f	0-12 years	0.447	802	98	Grand Index
45	f	0-12 years	0.448	802.1020627	98	Grand Index
45	f	0-12 years	0.449	802.5907858	98	Grand Index
45	f	0-12 years	0.45	802.9832345	98	Grand Index
45	f	0-12 years	0.451	802.9925114	98	Grand Index
45	f	0-12 years	0.452	803.096	98	Grand Index
45	f	0-12 years	0.453	803.594	98	Grand Index
45	f	0-12 years	0.457	804	98	Grand Index
45	f	0-12 years	0.458	804.084	98	Grand Index
45	f	0-12 years	0.459	804.582	98	Grand Index
45	f	0-12 years	0.461	805	99	Grand Index
45	f	0-12 years	0.462	805.076	99	Grand Index
45	f	0-12 years	0.463	805.574	99	Grand Index
45	f	0-12 years	0.467	806	99	Grand Index
45	f	0-12 years	0.468	806.064	99	Grand Index
45	f	0-12 years	0.469	806.562	99	Grand Index
45	f	0-12 years	0.471	807	99	Grand Index
45	f	0-12 years	0.472	807.056	99	Grand Index
45	f	0-12 years	0.473	807.554	99	Grand Index
45	f	0-12 years	0.475	808	99	Grand Index
45	f	0-12 years	0.476	808.048	99	Grand Index
45	f	0-12 years	0.477	808.546	99	Grand Index
45	f	0-12 years	0.481	809	99	Grand Index
45	f	0-12 years	0.482	809.036	99	Grand Index
45	f	0-12 years	0.483	809.534	99	Grand Index
45	f	0-12 years	0.485	810	99	Grand Index
45	f	0-12 years	0.486	810.028	99	Grand Index
45	f	0-12 years	0.487	810.526	100	Grand Index
45	f	0-12 years	0.493	811	100	Grand Index
45	f	0-12 years	0.494	811.012	100	Grand Index
45	f	0-12 years	0.495	811.51	100	Grand Index
45	f	0-12 years	0.496	812.0001049	100	Grand Index
45	f	0-12 years	0.497	812.0066366	100	Grand Index
45	f	0-12 years	0.498	812.0170633	100	Grand Index
45	f	0-12 years	0.499	812.5085316	100	Grand Index
45	f	0-12 years	0.502	813	100	Grand Index
45	f	0-12 years	0.503	813.494	100	Grand Index
45	f	0-12 years	0.504	813.992	100	Grand Index
45	f	0-12 years	0.506	814	100	Grand Index

Age	Gender	Education	Percentile	Raw Score	Norm Score	Assessment
45	f	0-12 years	0.507	814.486	100	Grand Index
45	f	0-12 years	0.508	814.984	100	Grand Index
45	f	0-12 years	0.512	815	100	Grand Index
45	f	0-12 years	0.513	815.474	100	Grand Index
45	f	0-12 years	0.514	815.972	101	Grand Index
45	f	0-12 years	0.515	816.47	101	Grand Index
45	f	0-12 years	0.516	816.968	101	Grand Index
45	f	0-12 years	0.518	817	101	Grand Index
45	f	0-12 years	0.519	817.462	101	Grand Index
45	f	0-12 years	0.52	817.96	101	Grand Index
45	f	0-12 years	0.532	818	101	Grand Index
45	f	0-12 years	0.533	818.2357809	101	Grand Index
45	f	0-12 years	0.534	818.5063314	101	Grand Index
45	f	0-12 years	0.535	818.725592	101	Grand Index
45	f	0-12 years	0.536	818.9367417	101	Grand Index
45	f	0-12 years	0.537	818.9812126	101	Grand Index
45	f	0-12 years	0.538	818.9975125	101	Grand Index
45	f	0-12 years	0.54	819	102	Grand Index
45	f	0-12 years	0.541	819.418	102	Grand Index
45	f	0-12 years	0.542	819.916	102	Grand Index
45	f	0-12 years	0.548	820	102	Grand Index
45	f	0-12 years	0.549	820.402	102	Grand Index
45	f	0-12 years	0.55	820.9	102	Grand Index
45	f	0-12 years	0.556	821	102	Grand Index
45	f	0-12 years	0.557	821.386	102	Grand Index
45	f	0-12 years	0.558	821.884	102	Grand Index
45	f	0-12 years	0.566	822	102	Grand Index
45	f	0-12 years	0.567	822.366	103	Grand Index
45	f	0-12 years	0.568	822.864	103	Grand Index
45	f	0-12 years	0.576	823	103	Grand Index
45	f	0-12 years	0.577	823.346	103	Grand Index
45	f	0-12 years	0.578	823.844	103	Grand Index
45	f	0-12 years	0.579	824.342	103	Grand Index
45	f	0-12 years	0.58	824.84	103	Grand Index
45	f	0-12 years	0.584	825	103	Grand Index
45	f	0-12 years	0.585	825.33	103	Grand Index
45	f	0-12 years	0.586	825.828	103	Grand Index
45	f	0-12 years	0.587	826.326	103	Grand Index
45	f	0-12 years	0.588	826.824	103	Grand Index
45	f	0-12 years	0.59	827	103	Grand Index
45	f	0-12 years	0.591	827.318	103	Grand Index
45	f	0-12 years	0.592	827.816	103	Grand Index
45	f	0-12 years	0.596	828	104	Grand Index
45	f	0-12 years	0.597	828.306	104	Grand Index
45	f	0-12 years	0.598	828.804	104	Grand Index
45	f	0-12 years	0.6	829	104	Grand Index
45	f	0-12 years	0.601	829.2991313	104	Grand Index
45	f	0-12 years	0.602	829.7990218	104	Grand Index
45	f	0-12 years	0.603	830.2966802	104	Grand Index
45	f	0-12 years	0.604	830.7927896	104	Grand Index
45	f	0-12 years	0.606	831	104	Grand Index
45	f	0-12 years	0.607	831.286	104	Grand Index
45	f	0-12 years	0.608	831.784	104	Grand Index
45	f	0-12 years	0.618	832	105	Grand Index
45	f	0-12 years	0.619	832.262	105	Grand Index
45	f	0-12 years	0.62	832.76	105	Grand Index

Age	Gender	Education	Percentile	Raw Score	Norm Score	Assessment
45	f	0-12 years	0.626	833	105	Grand Index
45	f	0-12 years	0.627	833.246	105	Grand Index
45	f	0-12 years	0.628	833.744	105	Grand Index
45	f	0-12 years	0.63	834	105	Grand Index
45	f	0-12 years	0.631	834.238	105	Grand Index
45	f	0-12 years	0.632	834.736	105	Grand Index
45	f	0-12 years	0.634	835	105	Grand Index
45	f	0-12 years	0.635	835.23	105	Grand Index
45	f	0-12 years	0.636	835.728	105	Grand Index
45	f	0-12 years	0.638	836	105	Grand Index
45	f	0-12 years	0.639	836.222	105	Grand Index
45	f	0-12 years	0.64	836.72	105	Grand Index
45	f	0-12 years	0.642	837	105	Grand Index
45	f	0-12 years	0.643	837.214	105	Grand Index
45	f	0-12 years	0.644	837.712	106	Grand Index
45	f	0-12 years	0.646	838	106	Grand Index
45	f	0-12 years	0.647	838.1345093	106	Grand Index
45	f	0-12 years	0.648	838.4596823	106	Grand Index
45	f	0-12 years	0.649	838.7093765	106	Grand Index
45	f	0-12 years	0.65	838.8484683	106	Grand Index
45	f	0-12 years	0.651	839.1436714	106	Grand Index
45	f	0-12 years	0.652	839.6754066	106	Grand Index
45	f	0-12 years	0.658	840	106	Grand Index
45	f	0-12 years	0.659	840.0895894	106	Grand Index
45	f	0-12 years	0.66	840.3347298	106	Grand Index
45	f	0-12 years	0.661	840.5826292	106	Grand Index
45	f	0-12 years	0.662	840.8354889	106	Grand Index
45	f	0-12 years	0.663	841.174	106	Grand Index
45	f	0-12 years	0.664	841.672	106	Grand Index
45	f	0-12 years	0.668	842	107	Grand Index
45	f	0-12 years	0.669	842.162	107	Grand Index
45	f	0-12 years	0.67	842.66	107	Grand Index
45	f	0-12 years	0.672	843	107	Grand Index
45	f	0-12 years	0.673	843.154	107	Grand Index
45	f	0-12 years	0.674	843.652	107	Grand Index
45	f	0-12 years	0.675	844.2169312	107	Grand Index
45	f	0-12 years	0.676	844.9371429	107	Grand Index
45	f	0-12 years	0.677	845.5389094	107	Grand Index
45	f	0-12 years	0.678	845.8551098	107	Grand Index
45	f	0-12 years	0.679	846.1463014	107	Grand Index
45	f	0-12 years	0.68	846.3747951	107	Grand Index
45	f	0-12 years	0.681	846.6034553	107	Grand Index
45	f	0-12 years	0.682	846.8325496	107	Grand Index
45	f	0-12 years	0.683	847.129101	107	Grand Index
45	f	0-12 years	0.684	847.6088941	107	Grand Index
45	f	0-12 years	0.685	847.9681929	107	Grand Index
45	f	0-12 years	0.686	847.9863997	107	Grand Index
45	f	0-12 years	0.688	848	107	Grand Index
45	f	0-12 years	0.689	848.366	107	Grand Index
45	f	0-12 years	0.69	849.86	107	Grand Index
45	f	0-12 years	0.692	851	108	Grand Index
45	f	0-12 years	0.693	851.114	108	Grand Index
45	f	0-12 years	0.694	851.612	108	Grand Index
45	f	0-12 years	0.696	852	108	Grand Index
45	f	0-12 years	0.697	852.0015268	108	Grand Index
45	f	0-12 years	0.698	852.0086999	108	Grand Index

Age	Gender	Education	Percentile	Raw Score	Norm Score	Assessment
45	f	0-12 years	0.699	852.1149346	108	Grand Index
45	f	0-12 years	0.7	852.6057615	108	Grand Index
45	f	0-12 years	0.701	853.098	108	Grand Index
45	f	0-12 years	0.702	853.596	108	Grand Index
45	f	0-12 years	0.71	854	108	Grand Index
45	f	0-12 years	0.711	854.078	108	Grand Index
45	f	0-12 years	0.712	854.576	108	Grand Index
45	f	0-12 years	0.718	855	109	Grand Index
45	f	0-12 years	0.719	855.0011354	109	Grand Index
45	f	0-12 years	0.72	855.0102553	109	Grand Index
45	f	0-12 years	0.721	855.0752508	109	Grand Index
45	f	0-12 years	0.722	855.564131	109	Grand Index
45	f	0-12 years	0.723	856.054	109	Grand Index
45	f	0-12 years	0.724	856.552	109	Grand Index
45	f	0-12 years	0.726	857	109	Grand Index
45	f	0-12 years	0.727	857.046	109	Grand Index
45	f	0-12 years	0.728	857.544	109	Grand Index
45	f	0-12 years	0.729	858.042	109	Grand Index
45	f	0-12 years	0.73	858.54	109	Grand Index
45	f	0-12 years	0.734	859	109	Grand Index
45	f	0-12 years	0.735	859.0111298	109	Grand Index
45	f	0-12 years	0.736	859.1958848	109	Grand Index
45	f	0-12 years	0.737	859.387348	110	Grand Index
45	f	0-12 years	0.738	859.7005931	110	Grand Index
45	f	0-12 years	0.739	860.022	110	Grand Index
45	f	0-12 years	0.74	860.52	110	Grand Index
45	f	0-12 years	0.744	861	110	Grand Index
45	f	0-12 years	0.745	861.01	110	Grand Index
45	f	0-12 years	0.746	861.508	110	Grand Index
45	f	0-12 years	0.747	862.006	110	Grand Index
45	f	0-12 years	0.748	862.504	110	Grand Index
45	f	0-12 years	0.753	863	110	Grand Index
45	f	0-12 years	0.754	863.492	110	Grand Index
45	f	0-12 years	0.755	863.99	110	Grand Index
45	f	0-12 years	0.757	864	110	Grand Index
45	f	0-12 years	0.758	864.484	110	Grand Index
45	f	0-12 years	0.759	864.982	111	Grand Index
45	f	0-12 years	0.761	865	111	Grand Index
45	f	0-12 years	0.762	865.476	111	Grand Index
45	f	0-12 years	0.763	865.974	111	Grand Index
45	f	0-12 years	0.765	866	111	Grand Index
45	f	0-12 years	0.766	866.468	111	Grand Index
45	f	0-12 years	0.767	866.966	111	Grand Index
45	f	0-12 years	0.771	867	111	Grand Index
45	f	0-12 years	0.772	867.456	111	Grand Index
45	f	0-12 years	0.773	867.954	111	Grand Index
45	f	0-12 years	0.774	868.904	111	Grand Index
45	f	0-12 years	0.775	869.9	111	Grand Index
45	f	0-12 years	0.777	870	111	Grand Index
45	f	0-12 years	0.778	870.2800775	111	Grand Index
45	f	0-12 years	0.779	870.5942185	112	Grand Index
45	f	0-12 years	0.78	870.7932509	112	Grand Index
45	f	0-12 years	0.781	870.9771099	112	Grand Index
45	f	0-12 years	0.783	871	112	Grand Index
45	f	0-12 years	0.784	871.432	112	Grand Index
45	f	0-12 years	0.785	871.93	112	Grand Index

Age	Gender	Education	Percentile	Raw Score	Norm Score	Assessment
45	f	0-12 years	0.787	872	112	Grand Index
45	f	0-12 years	0.788	872.424	112	Grand Index
45	f	0-12 years	0.789	872.922	112	Grand Index
45	f	0-12 years	0.791	873	112	Grand Index
45	f	0-12 years	0.792	873.416	112	Grand Index
45	f	0-12 years	0.793	873.914	112	Grand Index
45	f	0-12 years	0.795	874	112	Grand Index
45	f	0-12 years	0.796	874.816	112	Grand Index
45	f	0-12 years	0.797	875.812	112	Grand Index
45	f	0-12 years	0.798	876.404	113	Grand Index
45	f	0-12 years	0.799	876.902	113	Grand Index
45	f	0-12 years	0.8	877.3349964	113	Grand Index
45	f	0-12 years	0.801	877.752067	113	Grand Index
45	f	0-12 years	0.802	878.2978446	113	Grand Index
45	f	0-12 years	0.803	878.8767741	113	Grand Index
45	f	0-12 years	0.804	879.392	113	Grand Index
45	f	0-12 years	0.805	879.89	113	Grand Index
45	f	0-12 years	0.807	880	113	Grand Index
45	f	0-12 years	0.808	880.384	113	Grand Index
45	f	0-12 years	0.809	880.882	113	Grand Index
45	f	0-12 years	0.81	881.379722	113	Grand Index
45	f	0-12 years	0.811	881.8773576	113	Grand Index
45	f	0-12 years	0.812	881.9995434	113	Grand Index
45	f	0-12 years	0.813	881.9999078	113	Grand Index
45	f	0-12 years	0.814	882.372	113	Grand Index
45	f	0-12 years	0.815	882.87	113	Grand Index
45	f	0-12 years	0.816	883.368	114	Grand Index
45	f	0-12 years	0.817	883.866	114	Grand Index
45	f	0-12 years	0.818	884.4508426	114	Grand Index
45	f	0-12 years	0.819	885.0676548	114	Grand Index
45	f	0-12 years	0.82	885.5126904	114	Grand Index
45	f	0-12 years	0.821	885.8918782	114	Grand Index
45	f	0-12 years	0.822	886.356	114	Grand Index
45	f	0-12 years	0.823	886.854	114	Grand Index
45	f	0-12 years	0.825	887	114	Grand Index
45	f	0-12 years	0.826	887.6086325	114	Grand Index
45	f	0-12 years	0.827	888.4796065	114	Grand Index
45	f	0-12 years	0.828	888.8041792	114	Grand Index
45	f	0-12 years	0.829	888.8841419	114	Grand Index
45	f	0-12 years	0.83	888.9402776	114	Grand Index
45	f	0-12 years	0.831	888.9853409	114	Grand Index
45	f	0-12 years	0.832	889.2879331	114	Grand Index
45	f	0-12 years	0.833	889.7146911	114	Grand Index
45	f	0-12 years	0.834	889.9044385	115	Grand Index
45	f	0-12 years	0.835	889.9756804	115	Grand Index
45	f	0-12 years	0.837	890	115	Grand Index
45	f	0-12 years	0.838	890.324	115	Grand Index
45	f	0-12 years	0.839	890.822	115	Grand Index
45	f	0-12 years	0.84	891.090397	115	Grand Index
45	f	0-12 years	0.841	891.2310774	115	Grand Index
45	f	0-12 years	0.842	891.3179401	115	Grand Index
45	f	0-12 years	0.843	891.3738066	115	Grand Index
45	f	0-12 years	0.844	891.5168938	115	Grand Index
45	f	0-12 years	0.845	891.711978	115	Grand Index
45	f	0-12 years	0.846	891.8521942	115	Grand Index
45	f	0-12 years	0.847	891.9585631	115	Grand Index

Age	Gender	Education	Percentile	Raw Score	Norm Score	Assessment
45	f	0-12 years	0.848	892.304	115	Grand Index
45	f	0-12 years	0.849	892.802	115	Grand Index
45	f	0-12 years	0.851	893	116	Grand Index
45	f	0-12 years	0.852	893.592	116	Grand Index
45	f	0-12 years	0.853	894.588	116	Grand Index
45	f	0-12 years	0.854	895.292	116	Grand Index
45	f	0-12 years	0.855	895.79	116	Grand Index
45	f	0-12 years	0.856	896.288	116	Grand Index
45	f	0-12 years	0.857	896.786	116	Grand Index
45	f	0-12 years	0.859	897	116	Grand Index
45	f	0-12 years	0.86	897.275502	116	Grand Index
45	f	0-12 years	0.861	897.765502	116	Grand Index
45	f	0-12 years	0.862	897.9883695	116	Grand Index
45	f	0-12 years	0.863	897.9963695	116	Grand Index
45	f	0-12 years	0.867	898	117	Grand Index
45	f	0-12 years	0.868	898.264	117	Grand Index
45	f	0-12 years	0.869	898.762	117	Grand Index
45	f	0-12 years	0.871	899	117	Grand Index
45	f	0-12 years	0.872	899.256	117	Grand Index
45	f	0-12 years	0.873	899.754	117	Grand Index
45	f	0-12 years	0.875	900	117	Grand Index
45	f	0-12 years	0.876	900.248	117	Grand Index
45	f	0-12 years	0.877	900.746	117	Grand Index
45	f	0-12 years	0.879	901	118	Grand Index
45	f	0-12 years	0.88	901.4748563	118	Grand Index
45	f	0-12 years	0.881	902.4601831	118	Grand Index
45	f	0-12 years	0.882	902.9836259	118	Grand Index
45	f	0-12 years	0.883	902.9942991	118	Grand Index
45	f	0-12 years	0.885	903	118	Grand Index
45	f	0-12 years	0.886	903.6658028	118	Grand Index
45	f	0-12 years	0.887	905.1200563	118	Grand Index
45	f	0-12 years	0.888	905.9364219	118	Grand Index
45	f	0-12 years	0.889	905.9725137	118	Grand Index
45	f	0-12 years	0.89	905.9942758	118	Grand Index
45	f	0-12 years	0.891	905.9979305	118	Grand Index
45	f	0-12 years	0.895	906	119	Grand Index
45	f	0-12 years	0.896	906.208	119	Grand Index
45	f	0-12 years	0.897	906.706	119	Grand Index
45	f	0-12 years	0.898	907.204	119	Grand Index
45	f	0-12 years	0.899	907.702	119	Grand Index
45	f	0-12 years	0.9	908.2	119	Grand Index
45	f	0-12 years	0.901	908.698	119	Grand Index
45	f	0-12 years	0.903	909	119	Grand Index
45	f	0-12 years	0.904	909.192	120	Grand Index
45	f	0-12 years	0.905	909.69	120	Grand Index
45	f	0-12 years	0.906	910.188	120	Grand Index
45	f	0-12 years	0.907	910.686	120	Grand Index
45	f	0-12 years	0.908	911.289995	120	Grand Index
45	f	0-12 years	0.909	912.0748728	120	Grand Index
45	f	0-12 years	0.91	912.8323691	120	Grand Index
45	f	0-12 years	0.911	913.5414913	120	Grand Index
45	f	0-12 years	0.912	914.352	120	Grand Index
45	f	0-12 years	0.913	915.348	120	Grand Index
45	f	0-12 years	0.915	916	121	Grand Index
45	f	0-12 years	0.916	916.043691	121	Grand Index
45	f	0-12 years	0.917	916.1732035	121	Grand Index

Age	Gender	Education	Percentile	Raw Score	Norm Score	Assessment
45	f	0-12 years	0.918	916.5454146	121	Grand Index
45	f	0-12 years	0.919	917.4119021	121	Grand Index
45	f	0-12 years	0.92	918.16	121	Grand Index
45	f	0-12 years	0.921	918.658	121	Grand Index
45	f	0-12 years	0.922	919.156	121	Grand Index
45	f	0-12 years	0.923	919.654	121	Grand Index
45	f	0-12 years	0.924	920.76	121	Grand Index
45	f	0-12 years	0.925	923.25	122	Grand Index
45	f	0-12 years	0.926	925.1355552	122	Grand Index
45	f	0-12 years	0.927	925.5916802	122	Grand Index
45	f	0-12 years	0.928	926.1192872	122	Grand Index
45	f	0-12 years	0.929	926.8226209	122	Grand Index
45	f	0-12 years	0.93	927.4222781	122	Grand Index
45	f	0-12 years	0.931	927.7568194	122	Grand Index
45	f	0-12 years	0.932	928.136	122	Grand Index
45	f	0-12 years	0.933	928.634	122	Grand Index
45	f	0-12 years	0.935	929	123	Grand Index
45	f	0-12 years	0.936	929.256	123	Grand Index
45	f	0-12 years	0.937	930.252	123	Grand Index
45	f	0-12 years	0.938	931.124	123	Grand Index
45	f	0-12 years	0.939	931.622	123	Grand Index
45	f	0-12 years	0.94	932.1180723	123	Grand Index
45	f	0-12 years	0.941	932.6080723	123	Grand Index
45	f	0-12 years	0.942	932.9857992	124	Grand Index
45	f	0-12 years	0.943	932.9937992	124	Grand Index
45	f	0-12 years	0.945	933	124	Grand Index
45	f	0-12 years	0.946	933.108	124	Grand Index
45	f	0-12 years	0.947	933.606	124	Grand Index
45	f	0-12 years	0.948	934.2627977	124	Grand Index
45	f	0-12 years	0.949	935.5211943	125	Grand Index
45	f	0-12 years	0.95	936.5724454	125	Grand Index
45	f	0-12 years	0.951	936.7992572	125	Grand Index
45	f	0-12 years	0.952	937.1103999	125	Grand Index
45	f	0-12 years	0.953	937.7746787	125	Grand Index
45	f	0-12 years	0.954	938.5620051	125	Grand Index
45	f	0-12 years	0.955	939.892346	125	Grand Index
45	f	0-12 years	0.956	940.9886971	126	Grand Index
45	f	0-12 years	0.957	940.9948691	126	Grand Index
45	f	0-12 years	0.958	941.3433876	126	Grand Index
45	f	0-12 years	0.959	943.3791857	126	Grand Index
45	f	0-12 years	0.96	945.1800291	126	Grand Index
45	f	0-12 years	0.961	945.7532346	126	Grand Index
45	f	0-12 years	0.962	946.4488018	127	Grand Index
45	f	0-12 years	0.963	947.8237982	127	Grand Index
45	f	0-12 years	0.964	949.288	127	Grand Index
45	f	0-12 years	0.965	951.28	127	Grand Index
45	f	0-12 years	0.966	953.0572159	127	Grand Index
45	f	0-12 years	0.967	953.4762379	128	Grand Index
45	f	0-12 years	0.968	953.9795595	128	Grand Index
45	f	0-12 years	0.969	955.0545374	128	Grand Index
45	f	0-12 years	0.97	956.06	128	Grand Index
45	f	0-12 years	0.971	956.558	128	Grand Index
45	f	0-12 years	0.972	957.168	129	Grand Index
45	f	0-12 years	0.973	958.662	129	Grand Index
45	f	0-12 years	0.974	960.2562034	129	Grand Index
45	f	0-12 years	0.975	962.7098435	129	Grand Index

Age	Gender	Education	Percentile	Raw Score	Norm Score	Assessment
45	f	0-12 years	0.976	964.9304928	130	Grand Index
45	f	0-12 years	0.977	964.9668527	130	Grand Index
45	f	0-12 years	0.978	965.0281203	130	Grand Index
45	f	0-12 years	0.979	965.346391	131	Grand Index
45	f	0-12 years	0.98	965.9297469	131	Grand Index
45	f	0-12 years	0.981	969.5483282	131	Grand Index
45	f	0-12 years	0.982	973.3047337	131	Grand Index
45	f	0-12 years	0.983	978.8298817	132	Grand Index
45	f	0-12 years	0.984	984.0242562	132	Grand Index
45	f	0-12 years	0.985	984.4017438	133	Grand Index
45	f	0-12 years	0.986	984.79433	133	Grand Index
45	f	0-12 years	0.987	985.4403586	133	Grand Index
45	f	0-12 years	0.988	986.1259272	134	Grand Index
45	f	0-12 years	0.989	987.5924111	134	Grand Index
45	f	0-12 years	0.99	989.0999704	135	Grand Index
45	f	0-12 years	0.991	991.5892323	135	Grand Index
45	f	0-12 years	0.992	994.2225417	136	Grand Index
45	f	0-12 years	0.993	1001.19528	137	Grand Index
45	f	0-12 years	0.994	1008.191903	138	Grand Index
45	f	0-12 years	0.995	1016.155887	139	Grand Index
45	f	0-12 years	0.996	1024.134763	140	Grand Index
45	f	0-12 years	0.997	1033.025769	141	Grand Index
45	f	0-12 years	0.998	1041.85398	143	Grand Index
45	f	0-12 years	0.999	1042.92699	146	Grand Index
45	m	0-12 years	0.001	592.008	54	Grand Index
45	m	0-12 years	0.002	609.016	57	Grand Index
45	m	0-12 years	0.003	616.6871068	59	Grand Index
45	m	0-12 years	0.004	617.3951068	60	Grand Index
45	m	0-12 years	0.005	619.02	61	Grand Index
45	m	0-12 years	0.006	626.024	62	Grand Index
45	m	0-12 years	0.007	633.028	63	Grand Index
45	m	0-12 years	0.008	636.2005218	64	Grand Index
45	m	0-12 years	0.009	637.8715184	65	Grand Index
45	m	0-12 years	0.01	640.2316432	65	Grand Index
45	m	0-12 years	0.011	644.2686466	66	Grand Index
45	m	0-12 years	0.012	648.30565	66	Grand Index
45	m	0-12 years	0.013	649.2622136	67	Grand Index
45	m	0-12 years	0.014	649.7342136	67	Grand Index
45	m	0-12 years	0.015	650.36	67	Grand Index
45	m	0-12 years	0.016	651.184	68	Grand Index
45	m	0-12 years	0.017	652.016	68	Grand Index
45	m	0-12 years	0.018	653.664	69	Grand Index
45	m	0-12 years	0.019	655.312	69	Grand Index
45	m	0-12 years	0.02	657.7068916	69	Grand Index
45	m	0-12 years	0.021	660.6370556	69	Grand Index
45	m	0-12 years	0.022	663.3608596	70	Grand Index
45	m	0-12 years	0.023	664.9625814	70	Grand Index
45	m	0-12 years	0.024	666.5643033	70	Grand Index
45	m	0-12 years	0.025	667.0213328	71	Grand Index
45	m	0-12 years	0.026	667.0510104	71	Grand Index
45	m	0-12 years	0.027	667.8473373	71	Grand Index
45	m	0-12 years	0.028	670.424269	71	Grand Index
45	m	0-12 years	0.029	673.0012008	72	Grand Index
45	m	0-12 years	0.03	673.9289246	72	Grand Index
45	m	0-12 years	0.031	674.6184294	72	Grand Index
45	m	0-12 years	0.032	675.1365887	72	Grand Index

Age	Gender	Education	Percentile	Raw Score	Norm Score	Assessment
45	m	0-12 years	0.033	675.4424287	72	Grand Index
45	m	0-12 years	0.034	675.7443913	73	Grand Index
45	m	0-12 years	0.035	675.8505514	73	Grand Index
45	m	0-12 years	0.036	675.9567114	73	Grand Index
45	m	0-12 years	0.037	676.732	73	Grand Index
45	m	0-12 years	0.038	677.968	73	Grand Index
45	m	0-12 years	0.039	679.136	74	Grand Index
45	m	0-12 years	0.04	679.96	74	Grand Index
45	m	0-12 years	0.041	680.784	74	Grand Index
45	m	0-12 years	0.043	681	74	Grand Index
45	m	0-12 years	0.044	681.3653592	74	Grand Index
45	m	0-12 years	0.045	682.5413592	75	Grand Index
45	m	0-12 years	0.046	683.7173592	75	Grand Index
45	m	0-12 years	0.047	683.9073786	75	Grand Index
45	m	0-12 years	0.048	683.9673786	75	Grand Index
45	m	0-12 years	0.05	684	75	Grand Index
45	m	0-12 years	0.051	684.036	75	Grand Index
45	m	0-12 years	0.052	685.272	76	Grand Index
45	m	0-12 years	0.053	686.508	76	Grand Index
45	m	0-12 years	0.055	687	76	Grand Index
45	m	0-12 years	0.056	687.0708084	76	Grand Index
45	m	0-12 years	0.057	687.47599	76	Grand Index
45	m	0-12 years	0.058	687.8811715	76	Grand Index
45	m	0-12 years	0.059	687.9885476	77	Grand Index
45	m	0-12 years	0.06	687.9953661	77	Grand Index
45	m	0-12 years	0.063	688	77	Grand Index
45	m	0-12 years	0.064	690.8360262	77	Grand Index
45	m	0-12 years	0.065	694.0111424	77	Grand Index
45	m	0-12 years	0.066	695.762927	77	Grand Index
45	m	0-12 years	0.067	695.8838108	78	Grand Index
45	m	0-12 years	0.07	696	78	Grand Index
45	m	0-12 years	0.071	696.756	78	Grand Index
45	m	0-12 years	0.072	697.992	78	Grand Index
45	m	0-12 years	0.073	699.304	78	Grand Index
45	m	0-12 years	0.074	700.952	78	Grand Index
45	m	0-12 years	0.075	702.6	78	Grand Index
45	m	0-12 years	0.076	703.312	79	Grand Index
45	m	0-12 years	0.077	703.724	79	Grand Index
45	m	0-12 years	0.078	704.136	79	Grand Index
45	m	0-12 years	0.079	704.548	79	Grand Index
45	m	0-12 years	0.08	704.96	79	Grand Index
45	m	0-12 years	0.081	705.2312408	79	Grand Index
45	m	0-12 years	0.082	705.4873461	79	Grand Index
45	m	0-12 years	0.083	705.6957784	79	Grand Index
45	m	0-12 years	0.084	705.8516731	79	Grand Index
45	m	0-12 years	0.085	706.04	79	Grand Index
45	m	0-12 years	0.086	706.864	80	Grand Index
45	m	0-12 years	0.087	707.688	80	Grand Index
45	m	0-12 years	0.088	709.2671835	80	Grand Index
45	m	0-12 years	0.089	711.3065568	80	Grand Index
45	m	0-12 years	0.09	713.0339405	80	Grand Index
45	m	0-12 years	0.091	713.4665672	80	Grand Index
45	m	0-12 years	0.092	713.8991938	80	Grand Index
45	m	0-12 years	0.093	714.0201139	80	Grand Index
45	m	0-12 years	0.094	714.0463384	80	Grand Index
45	m	0-12 years	0.095	714.1029832	80	Grand Index

Age	Gender	Education	Percentile	Raw Score	Norm Score	Assessment
45	m	0-12 years	0.096	714.2187305	80	Grand Index
45	m	0-12 years	0.097	714.3344777	81	Grand Index
45	m	0-12 years	0.098	714.5910251	81	Grand Index
45	m	0-12 years	0.099	714.8610534	81	Grand Index
45	m	0-12 years	0.104	715	81	Grand Index
45	m	0-12 years	0.105	715.78	81	Grand Index
45	m	0-12 years	0.106	717.016	81	Grand Index
45	m	0-12 years	0.107	718.084	81	Grand Index
45	m	0-12 years	0.108	718.496	81	Grand Index
45	m	0-12 years	0.109	718.908	82	Grand Index
45	m	0-12 years	0.114	719	82	Grand Index
45	m	0-12 years	0.115	719.38	82	Grand Index
45	m	0-12 years	0.116	719.792	82	Grand Index
45	m	0-12 years	0.118	720	82	Grand Index
45	m	0-12 years	0.119	720.0215759	82	Grand Index
45	m	0-12 years	0.12	720.3390502	82	Grand Index
45	m	0-12 years	0.121	720.6565244	82	Grand Index
45	m	0-12 years	0.122	720.8311385	83	Grand Index
45	m	0-12 years	0.123	720.9256642	83	Grand Index
45	m	0-12 years	0.124	721.088	83	Grand Index
45	m	0-12 years	0.125	721.5	83	Grand Index
45	m	0-12 years	0.126	721.912	83	Grand Index
45	m	0-12 years	0.127	722.972	83	Grand Index
45	m	0-12 years	0.128	724.208	83	Grand Index
45	m	0-12 years	0.135	725	83	Grand Index
45	m	0-12 years	0.136	725.0173598	84	Grand Index
45	m	0-12 years	0.137	725.2408666	84	Grand Index
45	m	0-12 years	0.138	725.4643735	84	Grand Index
45	m	0-12 years	0.139	725.9331044	84	Grand Index
45	m	0-12 years	0.14	726.5335976	84	Grand Index
45	m	0-12 years	0.143	727	84	Grand Index
45	m	0-12 years	0.144	727.328	84	Grand Index
45	m	0-12 years	0.145	727.74	84	Grand Index
45	m	0-12 years	0.148	728	84	Grand Index
45	m	0-12 years	0.149	728.388	84	Grand Index
45	m	0-12 years	0.15	728.8	84	Grand Index
45	m	0-12 years	0.151	729.1506733	85	Grand Index
45	m	0-12 years	0.152	729.4434913	85	Grand Index
45	m	0-12 years	0.153	729.7211372	85	Grand Index
45	m	0-12 years	0.154	729.8403192	85	Grand Index
45	m	0-12 years	0.155	729.9595012	85	Grand Index
45	m	0-12 years	0.157	730	85	Grand Index
45	m	0-12 years	0.158	730.1143055	85	Grand Index
45	m	0-12 years	0.159	730.6048666	85	Grand Index
45	m	0-12 years	0.16	731.0954277	85	Grand Index
45	m	0-12 years	0.161	731.4593758	85	Grand Index
45	m	0-12 years	0.162	731.7928147	85	Grand Index
45	m	0-12 years	0.163	732.156	85	Grand Index
45	m	0-12 years	0.164	732.568	85	Grand Index
45	m	0-12 years	0.165	732.98	85	Grand Index
45	m	0-12 years	0.166	733.0162441	85	Grand Index
45	m	0-12 years	0.167	733.033317	86	Grand Index
45	m	0-12 years	0.168	733.2484882	86	Grand Index
45	m	0-12 years	0.169	733.6434153	86	Grand Index
45	m	0-12 years	0.17	734.0055979	86	Grand Index
45	m	0-12 years	0.171	734.0632567	86	Grand Index

Age	Gender	Education	Percentile	Raw Score	Norm Score	Assessment
45	m	0-12 years	0.172	734.1209155	86	Grand Index
45	m	0-12 years	0.173	734.3773227	86	Grand Index
45	m	0-12 years	0.174	734.7316639	86	Grand Index
45	m	0-12 years	0.175	735.2	86	Grand Index
45	m	0-12 years	0.176	736.024	86	Grand Index
45	m	0-12 years	0.177	736.848	86	Grand Index
45	m	0-12 years	0.178	737.336	86	Grand Index
45	m	0-12 years	0.179	737.748	86	Grand Index
45	m	0-12 years	0.18	738.1435215	86	Grand Index
45	m	0-12 years	0.181	738.5130894	86	Grand Index
45	m	0-12 years	0.182	738.8826572	86	Grand Index
45	m	0-12 years	0.183	738.9377937	86	Grand Index
45	m	0-12 years	0.184	738.9802258	86	Grand Index
45	m	0-12 years	0.189	739	87	Grand Index
45	m	0-12 years	0.19	739.4623256	87	Grand Index
45	m	0-12 years	0.191	740.1426047	87	Grand Index
45	m	0-12 years	0.192	740.6767649	87	Grand Index
45	m	0-12 years	0.193	740.7781887	87	Grand Index
45	m	0-12 years	0.194	740.8796124	87	Grand Index
45	m	0-12 years	0.195	740.9322424	87	Grand Index
45	m	0-12 years	0.196	740.9745396	87	Grand Index
45	m	0-12 years	0.201	741	87	Grand Index
45	m	0-12 years	0.202	741.224	87	Grand Index
45	m	0-12 years	0.203	741.636	88	Grand Index
45	m	0-12 years	0.206	742	88	Grand Index
45	m	0-12 years	0.207	742.284	88	Grand Index
45	m	0-12 years	0.208	742.696	88	Grand Index
45	m	0-12 years	0.211	743	88	Grand Index
45	m	0-12 years	0.212	743.1988724	88	Grand Index
45	m	0-12 years	0.213	743.4370568	88	Grand Index
45	m	0-12 years	0.214	743.6489937	88	Grand Index
45	m	0-12 years	0.215	743.8228093	88	Grand Index
45	m	0-12 years	0.216	743.9966249	88	Grand Index
45	m	0-12 years	0.217	744.4985157	88	Grand Index
45	m	0-12 years	0.218	745.006903	88	Grand Index
45	m	0-12 years	0.219	745.4086092	88	Grand Index
45	m	0-12 years	0.22	745.7242219	88	Grand Index
45	m	0-12 years	0.221	746.052	88	Grand Index
45	m	0-12 years	0.222	746.464	89	Grand Index
45	m	0-12 years	0.223	746.876	89	Grand Index
45	m	0-12 years	0.228	747	89	Grand Index
45	m	0-12 years	0.229	748.044	89	Grand Index
45	m	0-12 years	0.23	749.28	89	Grand Index
45	m	0-12 years	0.231	750.344	89	Grand Index
45	m	0-12 years	0.232	751.168	89	Grand Index
45	m	0-12 years	0.233	751.992	89	Grand Index
45	m	0-12 years	0.237	752	89	Grand Index
45	m	0-12 years	0.238	752.056	89	Grand Index
45	m	0-12 years	0.239	752.468	89	Grand Index
45	m	0-12 years	0.24	752.88	89	Grand Index
45	m	0-12 years	0.242	753	90	Grand Index
45	m	0-12 years	0.243	753.116	90	Grand Index
45	m	0-12 years	0.244	753.528	90	Grand Index
45	m	0-12 years	0.245	753.94	90	Grand Index
45	m	0-12 years	0.246	754.704	90	Grand Index
45	m	0-12 years	0.247	755.528	90	Grand Index

Age	Gender	Education	Percentile	Raw Score	Norm Score	Assessment
45	m	0-12 years	0.248	756.176	90	Grand Index
45	m	0-12 years	0.249	756.588	90	Grand Index
45	m	0-12 years	0.25	757	90	Grand Index
45	m	0-12 years	0.251	757.412	90	Grand Index
45	m	0-12 years	0.252	757.824	90	Grand Index
45	m	0-12 years	0.253	758.236	90	Grand Index
45	m	0-12 years	0.254	758.648	90	Grand Index
45	m	0-12 years	0.255	759.0593492	90	Grand Index
45	m	0-12 years	0.256	759.4668804	90	Grand Index
45	m	0-12 years	0.257	759.8744116	90	Grand Index
45	m	0-12 years	0.258	759.9915951	90	Grand Index
45	m	0-12 years	0.259	759.9949936	90	Grand Index
45	m	0-12 years	0.26	759.997714	90	Grand Index
45	m	0-12 years	0.261	759.9987843	90	Grand Index
45	m	0-12 years	0.262	759.9998545	90	Grand Index
45	m	0-12 years	0.263	760.712	90	Grand Index
45	m	0-12 years	0.264	761.536	91	Grand Index
45	m	0-12 years	0.266	762	91	Grand Index
45	m	0-12 years	0.267	762.000231	91	Grand Index
45	m	0-12 years	0.268	762.0240289	91	Grand Index
45	m	0-12 years	0.269	762.0478267	91	Grand Index
45	m	0-12 years	0.27	762.2838989	91	Grand Index
45	m	0-12 years	0.271	762.6721011	91	Grand Index
45	m	0-12 years	0.274	763	91	Grand Index
45	m	0-12 years	0.275	763.3	91	Grand Index
45	m	0-12 years	0.276	763.712	91	Grand Index
45	m	0-12 years	0.281	764	91	Grand Index
45	m	0-12 years	0.282	764.184	91	Grand Index
45	m	0-12 years	0.283	764.596	91	Grand Index
45	m	0-12 years	0.291	765	92	Grand Index
45	m	0-12 years	0.292	765.608	92	Grand Index
45	m	0-12 years	0.293	766.432	92	Grand Index
45	m	0-12 years	0.296	767	92	Grand Index
45	m	0-12 years	0.297	767.364	92	Grand Index
45	m	0-12 years	0.298	767.776	92	Grand Index
45	m	0-12 years	0.299	768.188	92	Grand Index
45	m	0-12 years	0.3	768.6	92	Grand Index
45	m	0-12 years	0.305	769	92	Grand Index
45	m	0-12 years	0.306	769.072	92	Grand Index
45	m	0-12 years	0.307	769.484	92	Grand Index
45	m	0-12 years	0.308	769.896	92	Grand Index
45	m	0-12 years	0.309	770.616	93	Grand Index
45	m	0-12 years	0.31	771.44	93	Grand Index
45	m	0-12 years	0.315	772	93	Grand Index
45	m	0-12 years	0.316	772.1889331	93	Grand Index
45	m	0-12 years	0.317	772.5943522	93	Grand Index
45	m	0-12 years	0.318	772.9842823	93	Grand Index
45	m	0-12 years	0.319	772.9908633	93	Grand Index
45	m	0-12 years	0.32	772.9974443	93	Grand Index
45	m	0-12 years	0.322	773	93	Grand Index
45	m	0-12 years	0.323	773.076	93	Grand Index
45	m	0-12 years	0.324	773.488	93	Grand Index
45	m	0-12 years	0.325	773.9	93	Grand Index
45	m	0-12 years	0.327	774	93	Grand Index
45	m	0-12 years	0.328	774.136	93	Grand Index
45	m	0-12 years	0.329	774.548	93	Grand Index

Age	Gender	Education	Percentile	Raw Score	Norm Score	Assessment
45	m	0-12 years	0.33	774.96	93	Grand Index
45	m	0-12 years	0.334	775	94	Grand Index
45	m	0-12 years	0.335	775.0014244	94	Grand Index
45	m	0-12 years	0.336	775.0307674	94	Grand Index
45	m	0-12 years	0.337	775.0601105	94	Grand Index
45	m	0-12 years	0.338	775.2006088	94	Grand Index
45	m	0-12 years	0.339	775.4088424	94	Grand Index
45	m	0-12 years	0.34	775.6905109	94	Grand Index
45	m	0-12 years	0.341	776.2769343	94	Grand Index
45	m	0-12 years	0.342	776.8633577	94	Grand Index
45	m	0-12 years	0.343	777.316	94	Grand Index
45	m	0-12 years	0.344	777.728	94	Grand Index
45	m	0-12 years	0.345	778.14	94	Grand Index
45	m	0-12 years	0.346	778.552	94	Grand Index
45	m	0-12 years	0.347	778.964	94	Grand Index
45	m	0-12 years	0.348	779.376	94	Grand Index
45	m	0-12 years	0.349	779.788	94	Grand Index
45	m	0-12 years	0.351	780	94	Grand Index
45	m	0-12 years	0.352	780.0069837	94	Grand Index
45	m	0-12 years	0.353	780.1268704	94	Grand Index
45	m	0-12 years	0.354	780.2467571	94	Grand Index
45	m	0-12 years	0.355	780.4129766	94	Grand Index
45	m	0-12 years	0.356	780.606283	94	Grand Index
45	m	0-12 years	0.357	780.7803225	95	Grand Index
45	m	0-12 years	0.358	780.8791294	95	Grand Index
45	m	0-12 years	0.359	780.9779363	95	Grand Index
45	m	0-12 years	0.361	781	95	Grand Index
45	m	0-12 years	0.362	781.144	95	Grand Index
45	m	0-12 years	0.363	781.556	95	Grand Index
45	m	0-12 years	0.364	781.968	95	Grand Index
45	m	0-12 years	0.365	782.38	95	Grand Index
45	m	0-12 years	0.366	782.792	95	Grand Index
45	m	0-12 years	0.367	783.1889801	95	Grand Index
45	m	0-12 years	0.368	783.5706459	95	Grand Index
45	m	0-12 years	0.369	783.9564347	95	Grand Index
45	m	0-12 years	0.37	784.398769	95	Grand Index
45	m	0-12 years	0.371	784.8411032	95	Grand Index
45	m	0-12 years	0.372	785.1455755	95	Grand Index
45	m	0-12 years	0.373	785.3727615	95	Grand Index
45	m	0-12 years	0.374	785.5908972	95	Grand Index
45	m	0-12 years	0.375	785.7757112	95	Grand Index
45	m	0-12 years	0.376	785.9605252	95	Grand Index
45	m	0-12 years	0.377	786.648	95	Grand Index
45	m	0-12 years	0.378	787.472	95	Grand Index
45	m	0-12 years	0.381	788	95	Grand Index
45	m	0-12 years	0.382	788.768	95	Grand Index
45	m	0-12 years	0.383	789.592	96	Grand Index
45	m	0-12 years	0.385	790	96	Grand Index
45	m	0-12 years	0.386	790.032	96	Grand Index
45	m	0-12 years	0.387	790.444	96	Grand Index
45	m	0-12 years	0.388	790.856	96	Grand Index
45	m	0-12 years	0.389	791.268	96	Grand Index
45	m	0-12 years	0.39	791.68	96	Grand Index
45	m	0-12 years	0.391	792.092	96	Grand Index
45	m	0-12 years	0.392	792.504	96	Grand Index
45	m	0-12 years	0.393	792.916	96	Grand Index

Age	Gender	Education	Percentile	Raw Score	Norm Score	Assessment
45	m	0-12 years	0.395	793	96	Grand Index
45	m	0-12 years	0.396	793.152	96	Grand Index
45	m	0-12 years	0.397	793.564	96	Grand Index
45	m	0-12 years	0.398	793.976	96	Grand Index
45	m	0-12 years	0.402	794	96	Grand Index
45	m	0-12 years	0.403	794.036	96	Grand Index
45	m	0-12 years	0.404	794.448	96	Grand Index
45	m	0-12 years	0.405	794.86	96	Grand Index
45	m	0-12 years	0.407	795	96	Grand Index
45	m	0-12 years	0.408	795.192	97	Grand Index
45	m	0-12 years	0.409	796.016	97	Grand Index
45	m	0-12 years	0.41	796.84	97	Grand Index
45	m	0-12 years	0.411	797.332	97	Grand Index
45	m	0-12 years	0.412	797.744	97	Grand Index
45	m	0-12 years	0.422	798	97	Grand Index
45	m	0-12 years	0.423	798.1805283	97	Grand Index
45	m	0-12 years	0.424	798.4500126	97	Grand Index
45	m	0-12 years	0.425	798.6886792	97	Grand Index
45	m	0-12 years	0.426	798.831195	97	Grand Index
45	m	0-12 years	0.427	798.9737107	97	Grand Index
45	m	0-12 years	0.428	799.336	97	Grand Index
45	m	0-12 years	0.429	799.748	97	Grand Index
45	m	0-12 years	0.43	800.0207388	97	Grand Index
45	m	0-12 years	0.431	800.0741413	97	Grand Index
45	m	0-12 years	0.432	800.1275437	97	Grand Index
45	m	0-12 years	0.433	800.458675	97	Grand Index
45	m	0-12 years	0.434	800.8010276	98	Grand Index
45	m	0-12 years	0.435	801.1892451	98	Grand Index
45	m	0-12 years	0.436	801.61749	98	Grand Index
45	m	0-12 years	0.439	802	98	Grand Index
45	m	0-12 years	0.44	802.84	98	Grand Index
45	m	0-12 years	0.441	804.076	98	Grand Index
45	m	0-12 years	0.442	805.0081373	98	Grand Index
45	m	0-12 years	0.443	805.0403734	98	Grand Index
45	m	0-12 years	0.444	805.0726095	98	Grand Index
45	m	0-12 years	0.445	805.3916404	98	Grand Index
45	m	0-12 years	0.446	805.7714043	98	Grand Index
45	m	0-12 years	0.453	806	98	Grand Index
45	m	0-12 years	0.454	806.048	98	Grand Index
45	m	0-12 years	0.455	806.46	98	Grand Index
45	m	0-12 years	0.456	806.872	98	Grand Index
45	m	0-12 years	0.457	807.284	98	Grand Index
45	m	0-12 years	0.458	807.696	98	Grand Index
45	m	0-12 years	0.466	808	99	Grand Index
45	m	0-12 years	0.467	808.404	99	Grand Index
45	m	0-12 years	0.468	808.816	99	Grand Index
45	m	0-12 years	0.473	809	99	Grand Index
45	m	0-12 years	0.474	809.288	99	Grand Index
45	m	0-12 years	0.475	809.7	99	Grand Index
45	m	0-12 years	0.48	810	99	Grand Index
45	m	0-12 years	0.481	810.172	99	Grand Index
45	m	0-12 years	0.482	810.584	99	Grand Index
45	m	0-12 years	0.483	810.996	99	Grand Index
45	m	0-12 years	0.484	811.408	99	Grand Index
45	m	0-12 years	0.485	811.82	99	Grand Index
45	m	0-12 years	0.486	812.232	99	Grand Index

Age	Gender	Education	Percentile	Raw Score	Norm Score	Assessment
45	m	0-12 years	0.487	812.644	100	Grand Index
45	m	0-12 years	0.492	813	100	Grand Index
45	m	0-12 years	0.493	813.116	100	Grand Index
45	m	0-12 years	0.494	813.528	100	Grand Index
45	m	0-12 years	0.495	813.94	100	Grand Index
45	m	0-12 years	0.497	814	100	Grand Index
45	m	0-12 years	0.498	814.176	100	Grand Index
45	m	0-12 years	0.499	814.588	100	Grand Index
45	m	0-12 years	0.504	815	100	Grand Index
45	m	0-12 years	0.505	815.06	100	Grand Index
45	m	0-12 years	0.506	815.472	100	Grand Index
45	m	0-12 years	0.507	815.884	100	Grand Index
45	m	0-12 years	0.508	816.296	100	Grand Index
45	m	0-12 years	0.509	816.708	100	Grand Index
45	m	0-12 years	0.51	817.12	100	Grand Index
45	m	0-12 years	0.511	817.532	100	Grand Index
45	m	0-12 years	0.512	817.944	100	Grand Index
45	m	0-12 years	0.513	818.356	100	Grand Index
45	m	0-12 years	0.514	818.768	101	Grand Index
45	m	0-12 years	0.524	819	101	Grand Index
45	m	0-12 years	0.525	819.3	101	Grand Index
45	m	0-12 years	0.526	819.712	101	Grand Index
45	m	0-12 years	0.531	820	101	Grand Index
45	m	0-12 years	0.532	820.184	101	Grand Index
45	m	0-12 years	0.533	820.596	101	Grand Index
45	m	0-12 years	0.541	821	102	Grand Index
45	m	0-12 years	0.542	821.304	102	Grand Index
45	m	0-12 years	0.543	821.716	102	Grand Index
45	m	0-12 years	0.546	822	102	Grand Index
45	m	0-12 years	0.547	822.364	102	Grand Index
45	m	0-12 years	0.548	822.776	102	Grand Index
45	m	0-12 years	0.555	823	102	Grand Index
45	m	0-12 years	0.556	823.072	102	Grand Index
45	m	0-12 years	0.557	823.484	102	Grand Index
45	m	0-12 years	0.558	823.896	102	Grand Index
45	m	0-12 years	0.565	824	102	Grand Index
45	m	0-12 years	0.566	824.576	102	Grand Index
45	m	0-12 years	0.567	825.812	103	Grand Index
45	m	0-12 years	0.572	827	103	Grand Index
45	m	0-12 years	0.573	827.076	103	Grand Index
45	m	0-12 years	0.574	827.488	103	Grand Index
45	m	0-12 years	0.575	827.9	103	Grand Index
45	m	0-12 years	0.582	828	103	Grand Index
45	m	0-12 years	0.583	828.392	103	Grand Index
45	m	0-12 years	0.584	829.216	103	Grand Index
45	m	0-12 years	0.589	830	103	Grand Index
45	m	0-12 years	0.59	830.08	103	Grand Index
45	m	0-12 years	0.591	830.492	103	Grand Index
45	m	0-12 years	0.592	830.904	103	Grand Index
45	m	0-12 years	0.593	831.0180347	104	Grand Index
45	m	0-12 years	0.594	831.0415484	104	Grand Index
45	m	0-12 years	0.595	831.1890819	104	Grand Index
45	m	0-12 years	0.596	831.5775682	104	Grand Index
45	m	0-12 years	0.597	831.9660546	104	Grand Index
45	m	0-12 years	0.598	832.4537592	104	Grand Index
45	m	0-12 years	0.599	832.9509634	104	Grand Index

Age	Gender	Education	Percentile	Raw Score	Norm Score	Assessment
45	m	0-12 years	0.6	833.365445	104	Grand Index
45	m	0-12 years	0.601	833.6922408	104	Grand Index
45	m	0-12 years	0.606	834	104	Grand Index
45	m	0-12 years	0.607	834.0159158	104	Grand Index
45	m	0-12 years	0.608	834.0939789	104	Grand Index
45	m	0-12 years	0.609	834.1720421	104	Grand Index
45	m	0-12 years	0.61	834.4488421	104	Grand Index
45	m	0-12 years	0.611	834.7827789	104	Grand Index
45	m	0-12 years	0.612	835.144	104	Grand Index
45	m	0-12 years	0.613	835.556	104	Grand Index
45	m	0-12 years	0.614	835.968	104	Grand Index
45	m	0-12 years	0.616	836	104	Grand Index
45	m	0-12 years	0.617	836.204	104	Grand Index
45	m	0-12 years	0.618	836.616	105	Grand Index
45	m	0-12 years	0.619	837.056	105	Grand Index
45	m	0-12 years	0.62	837.88	105	Grand Index
45	m	0-12 years	0.621	838.704	105	Grand Index
45	m	0-12 years	0.626	839	105	Grand Index
45	m	0-12 years	0.627	839.3047576	105	Grand Index
45	m	0-12 years	0.628	839.6922888	105	Grand Index
45	m	0-12 years	0.629	839.9493995	105	Grand Index
45	m	0-12 years	0.63	839.9738683	105	Grand Index
45	m	0-12 years	0.631	839.9983371	105	Grand Index
45	m	0-12 years	0.632	840.3124068	105	Grand Index
45	m	0-12 years	0.633	840.6475932	105	Grand Index
45	m	0-12 years	0.634	841.060339	105	Grand Index
45	m	0-12 years	0.635	841.5491525	105	Grand Index
45	m	0-12 years	0.64	842	105	Grand Index
45	m	0-12 years	0.641	842.092	105	Grand Index
45	m	0-12 years	0.642	842.504	105	Grand Index
45	m	0-12 years	0.643	842.916	105	Grand Index
45	m	0-12 years	0.645	843	106	Grand Index
45	m	0-12 years	0.646	843.0668021	106	Grand Index
45	m	0-12 years	0.647	843.2478708	106	Grand Index
45	m	0-12 years	0.648	843.4289396	106	Grand Index
45	m	0-12 years	0.649	843.6569662	106	Grand Index
45	m	0-12 years	0.65	843.8878975	106	Grand Index
45	m	0-12 years	0.652	844	106	Grand Index
45	m	0-12 years	0.653	844.036	106	Grand Index
45	m	0-12 years	0.654	844.448	106	Grand Index
45	m	0-12 years	0.655	844.86	106	Grand Index
45	m	0-12 years	0.657	845	106	Grand Index
45	m	0-12 years	0.658	845.096	106	Grand Index
45	m	0-12 years	0.659	845.508	106	Grand Index
45	m	0-12 years	0.66	845.92	106	Grand Index
45	m	0-12 years	0.665	846	106	Grand Index
45	m	0-12 years	0.666	846.392	106	Grand Index
45	m	0-12 years	0.667	846.804	106	Grand Index
45	m	0-12 years	0.672	847	107	Grand Index
45	m	0-12 years	0.673	847.276	107	Grand Index
45	m	0-12 years	0.674	847.688	107	Grand Index
45	m	0-12 years	0.679	848	107	Grand Index
45	m	0-12 years	0.68	848.16	107	Grand Index
45	m	0-12 years	0.681	848.572	107	Grand Index
45	m	0-12 years	0.682	848.984	107	Grand Index
45	m	0-12 years	0.683	849.792	107	Grand Index

Age	Gender	Education	Percentile	Raw Score	Norm Score	Assessment
45	m	0-12 years	0.684	850.616	107	Grand Index
45	m	0-12 years	0.686	851	107	Grand Index
45	m	0-12 years	0.687	851.0557348	107	Grand Index
45	m	0-12 years	0.688	851.577615	107	Grand Index
45	m	0-12 years	0.689	852.0994953	107	Grand Index
45	m	0-12 years	0.69	852.4720238	107	Grand Index
45	m	0-12 years	0.691	852.7741435	107	Grand Index
45	m	0-12 years	0.699	853	108	Grand Index
45	m	0-12 years	0.7	853.3870926	108	Grand Index
45	m	0-12 years	0.701	853.7857979	108	Grand Index
45	m	0-12 years	0.702	853.9749596	108	Grand Index
45	m	0-12 years	0.703	853.9882542	108	Grand Index
45	m	0-12 years	0.706	854	108	Grand Index
45	m	0-12 years	0.707	854.284	108	Grand Index
45	m	0-12 years	0.708	854.696	108	Grand Index
45	m	0-12 years	0.718	855	109	Grand Index
45	m	0-12 years	0.719	855.228	109	Grand Index
45	m	0-12 years	0.72	855.64	109	Grand Index
45	m	0-12 years	0.721	856.052	109	Grand Index
45	m	0-12 years	0.722	856.464	109	Grand Index
45	m	0-12 years	0.723	856.876	109	Grand Index
45	m	0-12 years	0.724	857.288	109	Grand Index
45	m	0-12 years	0.725	857.7	109	Grand Index
45	m	0-12 years	0.73	858	109	Grand Index
45	m	0-12 years	0.731	858.172	109	Grand Index
45	m	0-12 years	0.732	858.584	109	Grand Index
45	m	0-12 years	0.733	858.996	109	Grand Index
45	m	0-12 years	0.737	859	110	Grand Index
45	m	0-12 years	0.738	859.056	110	Grand Index
45	m	0-12 years	0.739	859.468	110	Grand Index
45	m	0-12 years	0.74	859.88	110	Grand Index
45	m	0-12 years	0.745	860	110	Grand Index
45	m	0-12 years	0.746	860.352	110	Grand Index
45	m	0-12 years	0.747	860.764	110	Grand Index
45	m	0-12 years	0.748	861.176	110	Grand Index
45	m	0-12 years	0.749	861.588	110	Grand Index
45	m	0-12 years	0.752	862	110	Grand Index
45	m	0-12 years	0.753	862.236	110	Grand Index
45	m	0-12 years	0.754	862.648	110	Grand Index
45	m	0-12 years	0.755	863.0131757	110	Grand Index
45	m	0-12 years	0.756	863.1036486	110	Grand Index
45	m	0-12 years	0.757	863.1941216	110	Grand Index
45	m	0-12 years	0.758	864.0425946	110	Grand Index
45	m	0-12 years	0.759	865.1881216	111	Grand Index
45	m	0-12 years	0.76	866.12	111	Grand Index
45	m	0-12 years	0.761	866.532	111	Grand Index
45	m	0-12 years	0.762	866.944	111	Grand Index
45	m	0-12 years	0.766	867	111	Grand Index
45	m	0-12 years	0.767	867.008	111	Grand Index
45	m	0-12 years	0.768	867.832	111	Grand Index
45	m	0-12 years	0.769	868.656	111	Grand Index
45	m	0-12 years	0.771	869	111	Grand Index
45	m	0-12 years	0.772	869.064	111	Grand Index
45	m	0-12 years	0.773	869.476	111	Grand Index
45	m	0-12 years	0.774	869.888	111	Grand Index
45	m	0-12 years	0.775	870.3	111	Grand Index

Age	Gender	Education	Percentile	Raw Score	Norm Score	Assessment
45	m	0-12 years	0.776	870.712	111	Grand Index
45	m	0-12 years	0.786	871	112	Grand Index
45	m	0-12 years	0.787	871.244	112	Grand Index
45	m	0-12 years	0.788	871.656	112	Grand Index
45	m	0-12 years	0.793	872	112	Grand Index
45	m	0-12 years	0.794	872.128	112	Grand Index
45	m	0-12 years	0.795	872.54	112	Grand Index
45	m	0-12 years	0.796	872.952	112	Grand Index
45	m	0-12 years	0.798	873	113	Grand Index
45	m	0-12 years	0.799	873.070706	113	Grand Index
45	m	0-12 years	0.8	873.2256574	113	Grand Index
45	m	0-12 years	0.801	873.3835825	113	Grand Index
45	m	0-12 years	0.802	873.6406311	113	Grand Index
45	m	0-12 years	0.803	873.8976797	113	Grand Index
45	m	0-12 years	0.804	874.248	113	Grand Index
45	m	0-12 years	0.805	874.66	113	Grand Index
45	m	0-12 years	0.806	875.072	113	Grand Index
45	m	0-12 years	0.807	875.484	113	Grand Index
45	m	0-12 years	0.808	875.896	113	Grand Index
45	m	0-12 years	0.809	876.308	113	Grand Index
45	m	0-12 years	0.81	876.72	113	Grand Index
45	m	0-12 years	0.813	877	113	Grand Index
45	m	0-12 years	0.814	877.0403224	113	Grand Index
45	m	0-12 years	0.815	877.085466	113	Grand Index
45	m	0-12 years	0.816	877.472534	114	Grand Index
45	m	0-12 years	0.817	878.2513904	114	Grand Index
45	m	0-12 years	0.818	879.048	114	Grand Index
45	m	0-12 years	0.819	880.284	114	Grand Index
45	m	0-12 years	0.82	881.52	114	Grand Index
45	m	0-12 years	0.821	882.252	114	Grand Index
45	m	0-12 years	0.822	882.664	114	Grand Index
45	m	0-12 years	0.825	883	114	Grand Index
45	m	0-12 years	0.826	883.312	114	Grand Index
45	m	0-12 years	0.827	883.724	114	Grand Index
45	m	0-12 years	0.83	884	114	Grand Index
45	m	0-12 years	0.831	884.372	114	Grand Index
45	m	0-12 years	0.832	884.784	114	Grand Index
45	m	0-12 years	0.834	885	115	Grand Index
45	m	0-12 years	0.835	885.02	115	Grand Index
45	m	0-12 years	0.836	885.432	115	Grand Index
45	m	0-12 years	0.837	885.844	115	Grand Index
45	m	0-12 years	0.838	886.6713557	115	Grand Index
45	m	0-12 years	0.839	887.7518188	115	Grand Index
45	m	0-12 years	0.84	888.6526846	115	Grand Index
45	m	0-12 years	0.841	888.8082215	115	Grand Index
45	m	0-12 years	0.842	888.9637584	115	Grand Index
45	m	0-12 years	0.843	889.316	115	Grand Index
45	m	0-12 years	0.844	889.728	115	Grand Index
45	m	0-12 years	0.847	890	115	Grand Index
45	m	0-12 years	0.848	890.376	115	Grand Index
45	m	0-12 years	0.849	890.788	115	Grand Index
45	m	0-12 years	0.854	891	116	Grand Index
45	m	0-12 years	0.855	891.26	116	Grand Index
45	m	0-12 years	0.856	891.672	116	Grand Index
45	m	0-12 years	0.857	892.084	116	Grand Index
45	m	0-12 years	0.858	892.496	116	Grand Index

Age	Gender	Education	Percentile	Raw Score	Norm Score	Assessment
45	m	0-12 years	0.859	892.908	116	Grand Index
45	m	0-12 years	0.861	893	116	Grand Index
45	m	0-12 years	0.862	893.432	116	Grand Index
45	m	0-12 years	0.863	894.668	116	Grand Index
45	m	0-12 years	0.864	895.904	116	Grand Index
45	m	0-12 years	0.865	897.14	117	Grand Index
45	m	0-12 years	0.866	898.376	117	Grand Index
45	m	0-12 years	0.867	899.408	117	Grand Index
45	m	0-12 years	0.868	900.232	117	Grand Index
45	m	0-12 years	0.869	901.112	117	Grand Index
45	m	0-12 years	0.87	902.76	117	Grand Index
45	m	0-12 years	0.871	904.408	117	Grand Index
45	m	0-12 years	0.873	905	117	Grand Index
45	m	0-12 years	0.874	905.176	117	Grand Index
45	m	0-12 years	0.875	906	117	Grand Index
45	m	0-12 years	0.876	906.824	117	Grand Index
45	m	0-12 years	0.877	907.324	117	Grand Index
45	m	0-12 years	0.878	907.736	117	Grand Index
45	m	0-12 years	0.881	908	118	Grand Index
45	m	0-12 years	0.882	909.536	118	Grand Index
45	m	0-12 years	0.883	911.184	118	Grand Index
45	m	0-12 years	0.884	912.624	118	Grand Index
45	m	0-12 years	0.885	913.86	118	Grand Index
45	m	0-12 years	0.886	915.0092569	118	Grand Index
45	m	0-12 years	0.887	915.1284389	118	Grand Index
45	m	0-12 years	0.888	915.2476209	118	Grand Index
45	m	0-12 years	0.889	915.4797506	118	Grand Index
45	m	0-12 years	0.89	915.7725686	118	Grand Index
45	m	0-12 years	0.891	916.552	118	Grand Index
45	m	0-12 years	0.892	919.024	119	Grand Index
45	m	0-12 years	0.893	921.496	119	Grand Index
45	m	0-12 years	0.895	922	119	Grand Index
45	m	0-12 years	0.896	922.304	119	Grand Index
45	m	0-12 years	0.897	923.128	119	Grand Index
45	m	0-12 years	0.898	923.952	119	Grand Index
45	m	0-12 years	0.899	924.388	119	Grand Index
45	m	0-12 years	0.9	924.8	119	Grand Index
45	m	0-12 years	0.901	925.424	119	Grand Index
45	m	0-12 years	0.902	926.248	119	Grand Index
45	m	0-12 years	0.903	927.0049655	119	Grand Index
45	m	0-12 years	0.904	927.0617931	120	Grand Index
45	m	0-12 years	0.905	927.1186207	120	Grand Index
45	m	0-12 years	0.906	927.3724138	120	Grand Index
45	m	0-12 years	0.907	927.7275862	120	Grand Index
45	m	0-12 years	0.91	928	120	Grand Index
45	m	0-12 years	0.911	928.664	120	Grand Index
45	m	0-12 years	0.912	929.488	120	Grand Index
45	m	0-12 years	0.915	930	121	Grand Index
45	m	0-12 years	0.916	930.392	121	Grand Index
45	m	0-12 years	0.917	930.804	121	Grand Index
45	m	0-12 years	0.918	931.648	121	Grand Index
45	m	0-12 years	0.919	932.884	121	Grand Index
45	m	0-12 years	0.92	934.1120721	121	Grand Index
45	m	0-12 years	0.921	935.2664151	121	Grand Index
45	m	0-12 years	0.922	936.4207581	121	Grand Index
45	m	0-12 years	0.923	936.8565056	121	Grand Index

Age	Gender	Education	Percentile	Raw Score	Norm Score	Assessment
45	m	0-12 years	0.924	936.9381626	121	Grand Index
45	m	0-12 years	0.925	937.1029662	122	Grand Index
45	m	0-12 years	0.926	937.5271867	122	Grand Index
45	m	0-12 years	0.927	937.9514072	122	Grand Index
45	m	0-12 years	0.928	938.3556952	122	Grand Index
45	m	0-12 years	0.929	938.7554747	122	Grand Index
45	m	0-12 years	0.93	939.0030544	122	Grand Index
45	m	0-12 years	0.931	939.0109194	122	Grand Index
45	m	0-12 years	0.932	939.0187844	122	Grand Index
45	m	0-12 years	0.933	940.1995303	122	Grand Index
45	m	0-12 years	0.934	941.4276652	123	Grand Index
45	m	0-12 years	0.935	942.44	123	Grand Index
45	m	0-12 years	0.936	943.264	123	Grand Index
45	m	0-12 years	0.939	944	123	Grand Index
45	m	0-12 years	0.94	944.1374517	123	Grand Index
45	m	0-12 years	0.941	944.3397022	123	Grand Index
45	m	0-12 years	0.942	944.5438456	124	Grand Index
45	m	0-12 years	0.943	944.7535951	124	Grand Index
45	m	0-12 years	0.944	944.9633447	124	Grand Index
45	m	0-12 years	0.945	946.4487317	124	Grand Index
45	m	0-12 years	0.946	948.2042537	124	Grand Index
45	m	0-12 years	0.947	949.5461756	124	Grand Index
45	m	0-12 years	0.948	950.2626537	124	Grand Index
45	m	0-12 years	0.949	950.9791317	125	Grand Index
45	m	0-12 years	0.95	953.8	125	Grand Index
45	m	0-12 years	0.951	956.684	125	Grand Index
45	m	0-12 years	0.952	958.7530766	125	Grand Index
45	m	0-12 years	0.953	960.1381995	125	Grand Index
45	m	0-12 years	0.954	961.3925754	125	Grand Index
45	m	0-12 years	0.955	961.6554524	125	Grand Index
45	m	0-12 years	0.956	961.9183295	126	Grand Index
45	m	0-12 years	0.958	962	126	Grand Index
45	m	0-12 years	0.959	962.0878041	126	Grand Index
45	m	0-12 years	0.96	962.4227603	126	Grand Index
45	m	0-12 years	0.961	962.7577165	126	Grand Index
45	m	0-12 years	0.962	962.8773284	127	Grand Index
45	m	0-12 years	0.963	962.9543721	127	Grand Index
45	m	0-12 years	0.964	963.1569717	127	Grand Index
45	m	0-12 years	0.965	963.5419261	127	Grand Index
45	m	0-12 years	0.966	963.9268805	127	Grand Index
45	m	0-12 years	0.967	966.0362439	128	Grand Index
45	m	0-12 years	0.968	968.179754	128	Grand Index
45	m	0-12 years	0.969	969.8189924	128	Grand Index
45	m	0-12 years	0.97	971.0512747	128	Grand Index
45	m	0-12 years	0.971	972.1670444	128	Grand Index
45	m	0-12 years	0.972	972.4761884	129	Grand Index
45	m	0-12 years	0.973	972.7853323	129	Grand Index
45	m	0-12 years	0.974	973.4693014	129	Grand Index
45	m	0-12 years	0.975	974.3146536	129	Grand Index
45	m	0-12 years	0.976	975.0008067	130	Grand Index
45	m	0-12 years	0.977	975.2605334	130	Grand Index
45	m	0-12 years	0.978	975.5202602	130	Grand Index
45	m	0-12 years	0.979	975.6970464	131	Grand Index
45	m	0-12 years	0.98	975.8585793	131	Grand Index
45	m	0-12 years	0.981	975.9608158	131	Grand Index
45	m	0-12 years	0.982	975.9803132	131	Grand Index

Age	Gender	Education	Percentile	Raw Score	Norm Score	Assessment
45	m	0-12 years	0.983	975.9998107	132	Grand Index
45	m	0-12 years	0.984	979.7696986	132	Grand Index
45	m	0-12 years	0.985	983.576355	133	Grand Index
45	m	0-12 years	0.986	985.8799032	133	Grand Index
45	m	0-12 years	0.987	987.0172468	133	Grand Index
45	m	0-12 years	0.988	988.448	134	Grand Index
45	m	0-12 years	0.989	991.744	134	Grand Index
45	m	0-12 years	0.99	995.04	135	Grand Index
45	m	0-12 years	0.991	996.2120662	135	Grand Index
45	m	0-12 years	0.992	996.5112828	136	Grand Index
45	m	0-12 years	0.993	997.5700085	137	Grand Index
45	m	0-12 years	0.994	1000.566792	138	Grand Index
45	m	0-12 years	0.995	1003.563575	139	Grand Index
45	m	0-12 years	0.996	1008.628787	140	Grand Index
45	m	0-12 years	0.997	1014.046571	141	Grand Index
45	m	0-12 years	0.998	1020.665114	143	Grand Index
45	m	0-12 years	0.999	1028.893765	146	Grand Index
45	f	13-16 years	0.001	577.8356732	54	Grand Index
45	f	13-16 years	0.002	595.8398111	57	Grand Index
45	f	13-16 years	0.003	602.2860648	59	Grand Index
45	f	13-16 years	0.004	608	60	Grand Index
45	f	13-16 years	0.005	613	61	Grand Index
45	f	13-16 years	0.006	616	62	Grand Index
45	f	13-16 years	0.007	619.885	63	Grand Index
45	f	13-16 years	0.008	624.8386432	64	Grand Index
45	f	13-16 years	0.009	627.2827024	65	Grand Index
45	f	13-16 years	0.01	631.9113028	65	Grand Index
45	f	13-16 years	0.011	637.035	66	Grand Index
45	f	13-16 years	0.012	642.3416892	66	Grand Index
45	f	13-16 years	0.013	646	67	Grand Index
45	f	13-16 years	0.014	648.7112183	67	Grand Index
45	f	13-16 years	0.015	652.3258598	67	Grand Index
45	f	13-16 years	0.016	656.1803702	68	Grand Index
45	f	13-16 years	0.017	658.145	68	Grand Index
45	f	13-16 years	0.018	660.9450759	69	Grand Index
45	f	13-16 years	0.019	661	69	Grand Index
45	f	13-16 years	0.02	662.7	69	Grand Index
45	f	13-16 years	0.021	663	69	Grand Index
45	f	13-16 years	0.022	663.9549911	70	Grand Index
45	f	13-16 years	0.023	664	70	Grand Index
45	f	13-16 years	0.024	665.9987987	70	Grand Index
45	f	13-16 years	0.025	667	71	Grand Index
45	f	13-16 years	0.026	669.7780091	71	Grand Index
45	f	13-16 years	0.027	671.995	71	Grand Index
45	f	13-16 years	0.028	672	71	Grand Index
45	f	13-16 years	0.029	673.7622225	72	Grand Index
45	f	13-16 years	0.03	674.7819077	72	Grand Index
45	f	13-16 years	0.031	676	72	Grand Index
45	f	13-16 years	0.032	677	72	Grand Index
45	f	13-16 years	0.033	678.0333098	72	Grand Index
45	f	13-16 years	0.034	678.9799362	73	Grand Index
45	f	13-16 years	0.035	679.475	73	Grand Index
45	f	13-16 years	0.036	680.0043255	73	Grand Index
45	f	13-16 years	0.037	681	73	Grand Index
45	f	13-16 years	0.038	681.5566972	73	Grand Index
45	f	13-16 years	0.039	682.215	74	Grand Index

Age	Gender	Education	Percentile	Raw Score	Norm Score	Assessment
45	f	13-16 years	0.04	683	74	Grand Index
45	f	13-16 years	0.042	684	74	Grand Index
45	f	13-16 years	0.043	685	74	Grand Index
45	f	13-16 years	0.044	686	74	Grand Index
45	f	13-16 years	0.045	687	75	Grand Index
45	f	13-16 years	0.046	687.9938694	75	Grand Index
45	f	13-16 years	0.047	688	75	Grand Index
45	f	13-16 years	0.048	688.8867205	75	Grand Index
45	f	13-16 years	0.05	690	75	Grand Index
45	f	13-16 years	0.051	691	75	Grand Index
45	f	13-16 years	0.052	692	76	Grand Index
45	f	13-16 years	0.053	692.3324027	76	Grand Index
45	f	13-16 years	0.054	693	76	Grand Index
45	f	13-16 years	0.055	693.5094595	76	Grand Index
45	f	13-16 years	0.056	694.0352911	76	Grand Index
45	f	13-16 years	0.057	694.9503944	76	Grand Index
45	f	13-16 years	0.058	696.0069055	76	Grand Index
45	f	13-16 years	0.06	697	77	Grand Index
45	f	13-16 years	0.061	698	77	Grand Index
45	f	13-16 years	0.062	698.3312303	77	Grand Index
45	f	13-16 years	0.063	699.0228951	77	Grand Index
45	f	13-16 years	0.064	700	77	Grand Index
45	f	13-16 years	0.065	700.0230855	77	Grand Index
45	f	13-16 years	0.066	701	77	Grand Index
45	f	13-16 years	0.067	701.9989632	78	Grand Index
45	f	13-16 years	0.069	702	78	Grand Index
45	f	13-16 years	0.07	702.9932881	78	Grand Index
45	f	13-16 years	0.071	703	78	Grand Index
45	f	13-16 years	0.072	703.139997	78	Grand Index
45	f	13-16 years	0.073	704	78	Grand Index
45	f	13-16 years	0.074	705.3311843	78	Grand Index
45	f	13-16 years	0.075	706	78	Grand Index
45	f	13-16 years	0.077	707	79	Grand Index
45	f	13-16 years	0.078	707.6379205	79	Grand Index
45	f	13-16 years	0.079	708	79	Grand Index
45	f	13-16 years	0.08	708.8	79	Grand Index
45	f	13-16 years	0.081	709	79	Grand Index
45	f	13-16 years	0.082	709.1419256	79	Grand Index
45	f	13-16 years	0.083	710	79	Grand Index
45	f	13-16 years	0.084	710.9920747	79	Grand Index
45	f	13-16 years	0.085	711.1466763	79	Grand Index
45	f	13-16 years	0.088	712	80	Grand Index
45	f	13-16 years	0.089	712.39061	80	Grand Index
45	f	13-16 years	0.092	713	80	Grand Index
45	f	13-16 years	0.093	714	80	Grand Index
45	f	13-16 years	0.094	714.0380553	80	Grand Index
45	f	13-16 years	0.095	714.9496045	80	Grand Index
45	f	13-16 years	0.097	715	81	Grand Index
45	f	13-16 years	0.098	715.0327642	81	Grand Index
45	f	13-16 years	0.099	715.6587713	81	Grand Index
45	f	13-16 years	0.1	716.5	81	Grand Index
45	f	13-16 years	0.101	717.172735	81	Grand Index
45	f	13-16 years	0.102	717.6796741	81	Grand Index
45	f	13-16 years	0.105	718	81	Grand Index
45	f	13-16 years	0.106	718.0619171	81	Grand Index
45	f	13-16 years	0.109	719	82	Grand Index

Age	Gender	Education	Percentile	Raw Score	Norm Score	Assessment
45	f	13-16 years	0.11	719.6646604	82	Grand Index
45	f	13-16 years	0.111	720.0431854	82	Grand Index
45	f	13-16 years	0.112	720.9317673	82	Grand Index
45	f	13-16 years	0.113	721	82	Grand Index
45	f	13-16 years	0.114	721.4905343	82	Grand Index
45	f	13-16 years	0.115	722	82	Grand Index
45	f	13-16 years	0.116	722.4774484	82	Grand Index
45	f	13-16 years	0.117	723	82	Grand Index
45	f	13-16 years	0.118	723.7954434	82	Grand Index
45	f	13-16 years	0.119	724	82	Grand Index
45	f	13-16 years	0.12	724.9986325	82	Grand Index
45	f	13-16 years	0.121	725	82	Grand Index
45	f	13-16 years	0.122	725.5101058	83	Grand Index
45	f	13-16 years	0.123	725.9978711	83	Grand Index
45	f	13-16 years	0.125	726	83	Grand Index
45	f	13-16 years	0.126	726.2291084	83	Grand Index
45	f	13-16 years	0.128	727	83	Grand Index
45	f	13-16 years	0.129	727.5006989	83	Grand Index
45	f	13-16 years	0.132	728	83	Grand Index
45	f	13-16 years	0.134	729	83	Grand Index
45	f	13-16 years	0.137	730	84	Grand Index
45	f	13-16 years	0.14	731	84	Grand Index
45	f	13-16 years	0.142	732	84	Grand Index
45	f	13-16 years	0.145	733	84	Grand Index
45	f	13-16 years	0.146	733.0037052	84	Grand Index
45	f	13-16 years	0.147	734	84	Grand Index
45	f	13-16 years	0.148	734.8790418	84	Grand Index
45	f	13-16 years	0.15	735	84	Grand Index
45	f	13-16 years	0.151	735.6367032	85	Grand Index
45	f	13-16 years	0.155	736	85	Grand Index
45	f	13-16 years	0.156	736.293849	85	Grand Index
45	f	13-16 years	0.159	737	85	Grand Index
45	f	13-16 years	0.16	737.18901	85	Grand Index
45	f	13-16 years	0.161	737.9991229	85	Grand Index
45	f	13-16 years	0.163	738	85	Grand Index
45	f	13-16 years	0.164	738.0946365	85	Grand Index
45	f	13-16 years	0.165	738.9982668	85	Grand Index
45	f	13-16 years	0.167	739	86	Grand Index
45	f	13-16 years	0.169	740	86	Grand Index
45	f	13-16 years	0.17	740.45	86	Grand Index
45	f	13-16 years	0.172	741	86	Grand Index
45	f	13-16 years	0.175	742	86	Grand Index
45	f	13-16 years	0.176	742.466902	86	Grand Index
45	f	13-16 years	0.179	743	86	Grand Index
45	f	13-16 years	0.18	743.6698999	86	Grand Index
45	f	13-16 years	0.183	744	86	Grand Index
45	f	13-16 years	0.186	745	87	Grand Index
45	f	13-16 years	0.187	745.092986	87	Grand Index
45	f	13-16 years	0.188	745.4708336	87	Grand Index
45	f	13-16 years	0.191	746	87	Grand Index
45	f	13-16 years	0.194	747	87	Grand Index
45	f	13-16 years	0.195	747.0047139	87	Grand Index
45	f	13-16 years	0.198	748	87	Grand Index
45	f	13-16 years	0.199	748.7024816	87	Grand Index
45	f	13-16 years	0.202	749	87	Grand Index
45	f	13-16 years	0.205	750	88	Grand Index

Age	Gender	Education	Percentile	Raw Score	Norm Score	Assessment
45	f	13-16 years	0.206	750.5322874	88	Grand Index
45	f	13-16 years	0.21	751	88	Grand Index
45	f	13-16 years	0.212	752	88	Grand Index
45	f	13-16 years	0.213	752.2571359	88	Grand Index
45	f	13-16 years	0.216	753	88	Grand Index
45	f	13-16 years	0.217	753.0482587	88	Grand Index
45	f	13-16 years	0.221	754	88	Grand Index
45	f	13-16 years	0.222	754.2896447	89	Grand Index
45	f	13-16 years	0.223	754.8724303	89	Grand Index
45	f	13-16 years	0.226	755	89	Grand Index
45	f	13-16 years	0.227	755.999734	89	Grand Index
45	f	13-16 years	0.23	756	89	Grand Index
45	f	13-16 years	0.233	757	89	Grand Index
45	f	13-16 years	0.237	758	89	Grand Index
45	f	13-16 years	0.24	759	89	Grand Index
45	f	13-16 years	0.241	759.9381342	89	Grand Index
45	f	13-16 years	0.244	760	90	Grand Index
45	f	13-16 years	0.245	760.8446359	90	Grand Index
45	f	13-16 years	0.248	761	90	Grand Index
45	f	13-16 years	0.249	761.2838152	90	Grand Index
45	f	13-16 years	0.25	762	90	Grand Index
45	f	13-16 years	0.251	762.4992419	90	Grand Index
45	f	13-16 years	0.254	763	90	Grand Index
45	f	13-16 years	0.255	763.7681769	90	Grand Index
45	f	13-16 years	0.257	764	90	Grand Index
45	f	13-16 years	0.258	764.8371394	90	Grand Index
45	f	13-16 years	0.261	765	90	Grand Index
45	f	13-16 years	0.262	765.505603	90	Grand Index
45	f	13-16 years	0.265	766	91	Grand Index
45	f	13-16 years	0.266	766.0248606	91	Grand Index
45	f	13-16 years	0.267	766.2015884	91	Grand Index
45	f	13-16 years	0.27	767	91	Grand Index
45	f	13-16 years	0.271	767.135	91	Grand Index
45	f	13-16 years	0.274	768	91	Grand Index
45	f	13-16 years	0.275	768.881122	91	Grand Index
45	f	13-16 years	0.278	769	91	Grand Index
45	f	13-16 years	0.279	769.1219933	91	Grand Index
45	f	13-16 years	0.282	770	91	Grand Index
45	f	13-16 years	0.283	770.769101	91	Grand Index
45	f	13-16 years	0.287	771	92	Grand Index
45	f	13-16 years	0.288	771.0056423	92	Grand Index
45	f	13-16 years	0.289	771.2959905	92	Grand Index
45	f	13-16 years	0.293	772	92	Grand Index
45	f	13-16 years	0.294	772.1366987	92	Grand Index
45	f	13-16 years	0.298	773	92	Grand Index
45	f	13-16 years	0.299	773.9403692	92	Grand Index
45	f	13-16 years	0.3	774	92	Grand Index
45	f	13-16 years	0.301	774.422642	92	Grand Index
45	f	13-16 years	0.304	775	92	Grand Index
45	f	13-16 years	0.305	775.1126832	92	Grand Index
45	f	13-16 years	0.309	776	93	Grand Index
45	f	13-16 years	0.31	776.0327698	93	Grand Index
45	f	13-16 years	0.311	776.7696528	93	Grand Index
45	f	13-16 years	0.314	777	93	Grand Index
45	f	13-16 years	0.315	777.0404843	93	Grand Index
45	f	13-16 years	0.318	778	93	Grand Index

Age	Gender	Education	Percentile	Raw Score	Norm Score	Assessment
45	f	13-16 years	0.322	779	93	Grand Index
45	f	13-16 years	0.323	779.4308	93	Grand Index
45	f	13-16 years	0.328	780	93	Grand Index
45	f	13-16 years	0.329	780.0482544	93	Grand Index
45	f	13-16 years	0.334	781	94	Grand Index
45	f	13-16 years	0.342	782	94	Grand Index
45	f	13-16 years	0.345	783	94	Grand Index
45	f	13-16 years	0.35	784	94	Grand Index
45	f	13-16 years	0.351	784.8964895	94	Grand Index
45	f	13-16 years	0.352	785	94	Grand Index
45	f	13-16 years	0.353	785.1289801	94	Grand Index
45	f	13-16 years	0.357	786	95	Grand Index
45	f	13-16 years	0.358	786.2509317	95	Grand Index
45	f	13-16 years	0.362	787	95	Grand Index
45	f	13-16 years	0.363	787.260443	95	Grand Index
45	f	13-16 years	0.367	788	95	Grand Index
45	f	13-16 years	0.368	788.189737	95	Grand Index
45	f	13-16 years	0.372	789	95	Grand Index
45	f	13-16 years	0.373	789.1311782	95	Grand Index
45	f	13-16 years	0.378	790	95	Grand Index
45	f	13-16 years	0.379	790.1087604	95	Grand Index
45	f	13-16 years	0.383	791	96	Grand Index
45	f	13-16 years	0.387	792	96	Grand Index
45	f	13-16 years	0.388	792.1342299	96	Grand Index
45	f	13-16 years	0.394	793	96	Grand Index
45	f	13-16 years	0.395	793.9984079	96	Grand Index
45	f	13-16 years	0.398	794	96	Grand Index
45	f	13-16 years	0.399	794.8819106	96	Grand Index
45	f	13-16 years	0.403	795	96	Grand Index
45	f	13-16 years	0.404	795.8290927	96	Grand Index
45	f	13-16 years	0.408	796	97	Grand Index
45	f	13-16 years	0.409	796.0648562	97	Grand Index
45	f	13-16 years	0.413	797	97	Grand Index
45	f	13-16 years	0.414	797.9686402	97	Grand Index
45	f	13-16 years	0.419	798	97	Grand Index
45	f	13-16 years	0.42	798.2273239	97	Grand Index
45	f	13-16 years	0.424	799	97	Grand Index
45	f	13-16 years	0.428	800	97	Grand Index
45	f	13-16 years	0.429	800.9382255	97	Grand Index
45	f	13-16 years	0.432	801	97	Grand Index
45	f	13-16 years	0.433	801.4705717	97	Grand Index
45	f	13-16 years	0.438	802	98	Grand Index
45	f	13-16 years	0.443	803	98	Grand Index
45	f	13-16 years	0.447	804	98	Grand Index
45	f	13-16 years	0.448	804.2520323	98	Grand Index
45	f	13-16 years	0.453	805	98	Grand Index
45	f	13-16 years	0.454	805.0370874	98	Grand Index
45	f	13-16 years	0.46	806	98	Grand Index
45	f	13-16 years	0.465	807	99	Grand Index
45	f	13-16 years	0.466	807.1230171	99	Grand Index
45	f	13-16 years	0.47	808	99	Grand Index
45	f	13-16 years	0.474	809	99	Grand Index
45	f	13-16 years	0.475	809.0790608	99	Grand Index
45	f	13-16 years	0.478	810	99	Grand Index
45	f	13-16 years	0.479	810.6274852	99	Grand Index
45	f	13-16 years	0.482	811	99	Grand Index

Age	Gender	Education	Percentile	Raw Score	Norm Score	Assessment
45	f	13-16 years	0.483	811.355	99	Grand Index
45	f	13-16 years	0.487	812	100	Grand Index
45	f	13-16 years	0.488	812.5890282	100	Grand Index
45	f	13-16 years	0.494	813	100	Grand Index
45	f	13-16 years	0.499	814	100	Grand Index
45	f	13-16 years	0.5	814.899588	100	Grand Index
45	f	13-16 years	0.505	815	100	Grand Index
45	f	13-16 years	0.506	815.0004311	100	Grand Index
45	f	13-16 years	0.509	816	100	Grand Index
45	f	13-16 years	0.51	816.2587026	100	Grand Index
45	f	13-16 years	0.513	817	100	Grand Index
45	f	13-16 years	0.516	818	101	Grand Index
45	f	13-16 years	0.517	818.2208262	101	Grand Index
45	f	13-16 years	0.518	818.9932736	101	Grand Index
45	f	13-16 years	0.522	819	101	Grand Index
45	f	13-16 years	0.523	819.755	101	Grand Index
45	f	13-16 years	0.526	820	101	Grand Index
45	f	13-16 years	0.527	820.0889253	101	Grand Index
45	f	13-16 years	0.528	820.6902046	101	Grand Index
45	f	13-16 years	0.531	821	101	Grand Index
45	f	13-16 years	0.536	822	101	Grand Index
45	f	13-16 years	0.537	822.0144847	101	Grand Index
45	f	13-16 years	0.538	822.5739207	101	Grand Index
45	f	13-16 years	0.539	822.9696485	101	Grand Index
45	f	13-16 years	0.544	823	102	Grand Index
45	f	13-16 years	0.545	823.3444534	102	Grand Index
45	f	13-16 years	0.551	824	102	Grand Index
45	f	13-16 years	0.554	825	102	Grand Index
45	f	13-16 years	0.555	825.9568124	102	Grand Index
45	f	13-16 years	0.559	826	102	Grand Index
45	f	13-16 years	0.56	826.0448092	102	Grand Index
45	f	13-16 years	0.561	826.5653645	102	Grand Index
45	f	13-16 years	0.566	827	102	Grand Index
45	f	13-16 years	0.567	827.858426	103	Grand Index
45	f	13-16 years	0.573	828	103	Grand Index
45	f	13-16 years	0.574	828.5014777	103	Grand Index
45	f	13-16 years	0.577	829	103	Grand Index
45	f	13-16 years	0.578	829.9822727	103	Grand Index
45	f	13-16 years	0.58	830	103	Grand Index
45	f	13-16 years	0.581	830.7359522	103	Grand Index
45	f	13-16 years	0.585	831	103	Grand Index
45	f	13-16 years	0.586	831.7588813	103	Grand Index
45	f	13-16 years	0.591	832	103	Grand Index
45	f	13-16 years	0.592	832.52	103	Grand Index
45	f	13-16 years	0.596	833	104	Grand Index
45	f	13-16 years	0.597	833.1294351	104	Grand Index
45	f	13-16 years	0.598	833.9578935	104	Grand Index
45	f	13-16 years	0.603	834	104	Grand Index
45	f	13-16 years	0.608	835	104	Grand Index
45	f	13-16 years	0.613	836	104	Grand Index
45	f	13-16 years	0.618	837	105	Grand Index
45	f	13-16 years	0.619	837.8072621	105	Grand Index
45	f	13-16 years	0.624	838	105	Grand Index
45	f	13-16 years	0.625	838.0007295	105	Grand Index
45	f	13-16 years	0.626	838.9877252	105	Grand Index
45	f	13-16 years	0.631	839	105	Grand Index

Age	Gender	Education	Percentile	Raw Score	Norm Score	Assessment
45	f	13-16 years	0.632	839.9861432	105	Grand Index
45	f	13-16 years	0.636	840	105	Grand Index
45	f	13-16 years	0.637	840.1569594	105	Grand Index
45	f	13-16 years	0.642	841	105	Grand Index
45	f	13-16 years	0.643	841.0127449	105	Grand Index
45	f	13-16 years	0.644	841.9940979	106	Grand Index
45	f	13-16 years	0.649	842	106	Grand Index
45	f	13-16 years	0.65	842.1632822	106	Grand Index
45	f	13-16 years	0.652	843	106	Grand Index
45	f	13-16 years	0.653	843.3641433	106	Grand Index
45	f	13-16 years	0.654	843.8029584	106	Grand Index
45	f	13-16 years	0.656	844	106	Grand Index
45	f	13-16 years	0.657	844.4762815	106	Grand Index
45	f	13-16 years	0.66	845	106	Grand Index
45	f	13-16 years	0.661	845.0874327	106	Grand Index
45	f	13-16 years	0.665	846	106	Grand Index
45	f	13-16 years	0.666	846.6830911	106	Grand Index
45	f	13-16 years	0.67	847	107	Grand Index
45	f	13-16 years	0.671	847.4765773	107	Grand Index
45	f	13-16 years	0.676	848	107	Grand Index
45	f	13-16 years	0.677	848.9455598	107	Grand Index
45	f	13-16 years	0.68	849	107	Grand Index
45	f	13-16 years	0.681	849.5609934	107	Grand Index
45	f	13-16 years	0.686	850	107	Grand Index
45	f	13-16 years	0.687	850.0920415	107	Grand Index
45	f	13-16 years	0.691	851	107	Grand Index
45	f	13-16 years	0.695	852	108	Grand Index
45	f	13-16 years	0.696	852.76	108	Grand Index
45	f	13-16 years	0.699	853	108	Grand Index
45	f	13-16 years	0.7	853.0031638	108	Grand Index
45	f	13-16 years	0.701	853.9739775	108	Grand Index
45	f	13-16 years	0.704	854	108	Grand Index
45	f	13-16 years	0.705	854.0108974	108	Grand Index
45	f	13-16 years	0.706	854.9858799	108	Grand Index
45	f	13-16 years	0.709	855	108	Grand Index
45	f	13-16 years	0.71	855.7461097	108	Grand Index
45	f	13-16 years	0.713	856	108	Grand Index
45	f	13-16 years	0.714	856.7089656	108	Grand Index
45	f	13-16 years	0.719	857	109	Grand Index
45	f	13-16 years	0.72	857.9407022	109	Grand Index
45	f	13-16 years	0.722	858	109	Grand Index
45	f	13-16 years	0.723	858.53987	109	Grand Index
45	f	13-16 years	0.726	859	109	Grand Index
45	f	13-16 years	0.727	859.5522653	109	Grand Index
45	f	13-16 years	0.732	860	109	Grand Index
45	f	13-16 years	0.736	861	109	Grand Index
45	f	13-16 years	0.737	861.1133209	110	Grand Index
45	f	13-16 years	0.741	862	110	Grand Index
45	f	13-16 years	0.742	862.27	110	Grand Index
45	f	13-16 years	0.745	863	110	Grand Index
45	f	13-16 years	0.746	863.3893893	110	Grand Index
45	f	13-16 years	0.75	864	110	Grand Index
45	f	13-16 years	0.751	864.3159603	110	Grand Index
45	f	13-16 years	0.757	865	110	Grand Index
45	f	13-16 years	0.758	865.1367568	110	Grand Index
45	f	13-16 years	0.76	866	111	Grand Index

Age	Gender	Education	Percentile	Raw Score	Norm Score	Assessment
45	f	13-16 years	0.761	866.9980294	111	Grand Index
45	f	13-16 years	0.764	867	111	Grand Index
45	f	13-16 years	0.765	867.0744701	111	Grand Index
45	f	13-16 years	0.77	868	111	Grand Index
45	f	13-16 years	0.771	868.9885019	111	Grand Index
45	f	13-16 years	0.775	869	111	Grand Index
45	f	13-16 years	0.78	870	112	Grand Index
45	f	13-16 years	0.783	871	112	Grand Index
45	f	13-16 years	0.784	871.0352137	112	Grand Index
45	f	13-16 years	0.788	872	112	Grand Index
45	f	13-16 years	0.79	873	112	Grand Index
45	f	13-16 years	0.791	873.4258026	112	Grand Index
45	f	13-16 years	0.794	874	112	Grand Index
45	f	13-16 years	0.796	875	112	Grand Index
45	f	13-16 years	0.797	875.445	112	Grand Index
45	f	13-16 years	0.801	876	113	Grand Index
45	f	13-16 years	0.805	877	113	Grand Index
45	f	13-16 years	0.806	877.9554655	113	Grand Index
45	f	13-16 years	0.809	878	113	Grand Index
45	f	13-16 years	0.811	879	113	Grand Index
45	f	13-16 years	0.812	879.107889	113	Grand Index
45	f	13-16 years	0.813	879.9195084	113	Grand Index
45	f	13-16 years	0.817	880	114	Grand Index
45	f	13-16 years	0.818	880.0356145	114	Grand Index
45	f	13-16 years	0.819	880.7694248	114	Grand Index
45	f	13-16 years	0.822	881	114	Grand Index
45	f	13-16 years	0.825	882	114	Grand Index
45	f	13-16 years	0.827	883	114	Grand Index
45	f	13-16 years	0.828	883.1172562	114	Grand Index
45	f	13-16 years	0.83	884	114	Grand Index
45	f	13-16 years	0.835	885	115	Grand Index
45	f	13-16 years	0.836	885.7706543	115	Grand Index
45	f	13-16 years	0.839	886	115	Grand Index
45	f	13-16 years	0.841	887	115	Grand Index
45	f	13-16 years	0.842	887.2600662	115	Grand Index
45	f	13-16 years	0.843	887.9543922	115	Grand Index
45	f	13-16 years	0.845	888	115	Grand Index
45	f	13-16 years	0.849	889	115	Grand Index
45	f	13-16 years	0.85	889.1539446	116	Grand Index
45	f	13-16 years	0.853	890	116	Grand Index
45	f	13-16 years	0.854	890.4229328	116	Grand Index
45	f	13-16 years	0.856	891	116	Grand Index
45	f	13-16 years	0.858	892	116	Grand Index
45	f	13-16 years	0.859	892.566174	116	Grand Index
45	f	13-16 years	0.86	893	116	Grand Index
45	f	13-16 years	0.861	893.0215695	116	Grand Index
45	f	13-16 years	0.862	894	116	Grand Index
45	f	13-16 years	0.863	894.1575458	116	Grand Index
45	f	13-16 years	0.865	895	117	Grand Index
45	f	13-16 years	0.866	895.0902302	117	Grand Index
45	f	13-16 years	0.867	895.8100828	117	Grand Index
45	f	13-16 years	0.868	896	117	Grand Index
45	f	13-16 years	0.869	896.5104435	117	Grand Index
45	f	13-16 years	0.872	897	117	Grand Index
45	f	13-16 years	0.875	898	117	Grand Index
45	f	13-16 years	0.876	898.0649844	117	Grand Index

Age	Gender	Education	Percentile	Raw Score	Norm Score	Assessment
45	f	13-16 years	0.877	899	117	Grand Index
45	f	13-16 years	0.878	899.8244637	117	Grand Index
45	f	13-16 years	0.879	900	118	Grand Index
45	f	13-16 years	0.88	900.9844402	118	Grand Index
45	f	13-16 years	0.881	901.1933446	118	Grand Index
45	f	13-16 years	0.883	902	118	Grand Index
45	f	13-16 years	0.884	902.6322301	118	Grand Index
45	f	13-16 years	0.886	903	118	Grand Index
45	f	13-16 years	0.888	904	118	Grand Index
45	f	13-16 years	0.89	905	118	Grand Index
45	f	13-16 years	0.891	905.3926735	118	Grand Index
45	f	13-16 years	0.892	906.9213663	119	Grand Index
45	f	13-16 years	0.893	907	119	Grand Index
45	f	13-16 years	0.894	907.6863753	119	Grand Index
45	f	13-16 years	0.897	908	119	Grand Index
45	f	13-16 years	0.898	909	119	Grand Index
45	f	13-16 years	0.899	910.1600229	119	Grand Index
45	f	13-16 years	0.9	910.9951076	119	Grand Index
45	f	13-16 years	0.901	911	119	Grand Index
45	f	13-16 years	0.902	911.4390832	119	Grand Index
45	f	13-16 years	0.904	912	120	Grand Index
45	f	13-16 years	0.905	912.0082517	120	Grand Index
45	f	13-16 years	0.908	913	120	Grand Index
45	f	13-16 years	0.909	913.165	120	Grand Index
45	f	13-16 years	0.91	914	120	Grand Index
45	f	13-16 years	0.913	915	120	Grand Index
45	f	13-16 years	0.914	916.0312269	120	Grand Index
45	f	13-16 years	0.916	917	121	Grand Index
45	f	13-16 years	0.917	917.3845176	121	Grand Index
45	f	13-16 years	0.918	918	121	Grand Index
45	f	13-16 years	0.919	919	121	Grand Index
45	f	13-16 years	0.92	919.0137996	121	Grand Index
45	f	13-16 years	0.923	920	121	Grand Index
45	f	13-16 years	0.925	921	122	Grand Index
45	f	13-16 years	0.927	922	122	Grand Index
45	f	13-16 years	0.928	922.9889959	122	Grand Index
45	f	13-16 years	0.929	923	122	Grand Index
45	f	13-16 years	0.93	924	122	Grand Index
45	f	13-16 years	0.931	924.1365581	122	Grand Index
45	f	13-16 years	0.932	925	122	Grand Index
45	f	13-16 years	0.934	926	123	Grand Index
45	f	13-16 years	0.935	926.6446871	123	Grand Index
45	f	13-16 years	0.936	927	123	Grand Index
45	f	13-16 years	0.937	927.0689035	123	Grand Index
45	f	13-16 years	0.939	928	123	Grand Index
45	f	13-16 years	0.94	928.4794791	123	Grand Index
45	f	13-16 years	0.941	929	123	Grand Index
45	f	13-16 years	0.942	929.8400606	124	Grand Index
45	f	13-16 years	0.943	930	124	Grand Index
45	f	13-16 years	0.944	930.475126	124	Grand Index
45	f	13-16 years	0.945	930.983125	124	Grand Index
45	f	13-16 years	0.946	931	124	Grand Index
45	f	13-16 years	0.947	932	124	Grand Index
45	f	13-16 years	0.948	933	124	Grand Index
45	f	13-16 years	0.949	934	125	Grand Index
45	f	13-16 years	0.95	935	125	Grand Index

Age	Gender	Education	Percentile	Raw Score	Norm Score	Assessment
45	f	13-16 years	0.951	935.494709	125	Grand Index
45	f	13-16 years	0.952	936	125	Grand Index
45	f	13-16 years	0.953	936.9298957	125	Grand Index
45	f	13-16 years	0.954	937.1527283	125	Grand Index
45	f	13-16 years	0.955	938	125	Grand Index
45	f	13-16 years	0.956	938.86	126	Grand Index
45	f	13-16 years	0.957	940.0180588	126	Grand Index
45	f	13-16 years	0.958	941.1900172	126	Grand Index
45	f	13-16 years	0.959	942	126	Grand Index
45	f	13-16 years	0.96	943	126	Grand Index
45	f	13-16 years	0.961	944.4018251	126	Grand Index
45	f	13-16 years	0.962	945	127	Grand Index
45	f	13-16 years	0.963	946	127	Grand Index
45	f	13-16 years	0.964	946.8738959	127	Grand Index
45	f	13-16 years	0.965	948	127	Grand Index
45	f	13-16 years	0.966	948.3521684	127	Grand Index
45	f	13-16 years	0.968	949	128	Grand Index
45	f	13-16 years	0.969	949.0020963	128	Grand Index
45	f	13-16 years	0.97	950.45	128	Grand Index
45	f	13-16 years	0.971	953.635	128	Grand Index
45	f	13-16 years	0.972	954.0255721	129	Grand Index
45	f	13-16 years	0.973	955.0037651	129	Grand Index
45	f	13-16 years	0.974	956.19	129	Grand Index
45	f	13-16 years	0.975	957.1392426	129	Grand Index
45	f	13-16 years	0.976	960.0038035	130	Grand Index
45	f	13-16 years	0.977	961.5460258	130	Grand Index
45	f	13-16 years	0.978	963	130	Grand Index
45	f	13-16 years	0.979	964.23	131	Grand Index
45	f	13-16 years	0.98	966.1088384	131	Grand Index
45	f	13-16 years	0.981	967	131	Grand Index
45	f	13-16 years	0.982	967.9269851	131	Grand Index
45	f	13-16 years	0.983	970.4637641	132	Grand Index
45	f	13-16 years	0.984	972.0044443	132	Grand Index
45	f	13-16 years	0.985	974.225	133	Grand Index
45	f	13-16 years	0.986	975.7340738	133	Grand Index
45	f	13-16 years	0.987	978.2102983	133	Grand Index
45	f	13-16 years	0.988	983.3439516	134	Grand Index
45	f	13-16 years	0.989	986.989402	134	Grand Index
45	f	13-16 years	0.99	987.3585018	135	Grand Index
45	f	13-16 years	0.991	988.8682244	135	Grand Index
45	f	13-16 years	0.992	990.997435	136	Grand Index
45	f	13-16 years	0.993	996.115	137	Grand Index
45	f	13-16 years	0.994	1000.780259	138	Grand Index
45	f	13-16 years	0.995	1006.91869	139	Grand Index
45	f	13-16 years	0.996	1010.78	140	Grand Index
45	f	13-16 years	0.997	1022.995532	141	Grand Index
45	f	13-16 years	0.998	1031.989148	143	Grand Index
45	f	13-16 years	0.999	1046.461498	146	Grand Index
45	m	13-16 years	0.001	582.0133728	54	Grand Index
45	m	13-16 years	0.002	600	57	Grand Index
45	m	13-16 years	0.003	613.67	59	Grand Index
45	m	13-16 years	0.004	618.7639485	60	Grand Index
45	m	13-16 years	0.005	620.0365822	61	Grand Index
45	m	13-16 years	0.006	624.4586177	62	Grand Index
45	m	13-16 years	0.007	626.8828776	63	Grand Index
45	m	13-16 years	0.008	632.0466005	64	Grand Index

Age	Gender	Education	Percentile	Raw Score	Norm Score	Assessment
45	m	13-16 years	0.009	635.334	65	Grand Index
45	m	13-16 years	0.01	636.5823326	65	Grand Index
45	m	13-16 years	0.011	640.8685418	66	Grand Index
45	m	13-16 years	0.012	644.56	66	Grand Index
45	m	13-16 years	0.013	649.0866152	67	Grand Index
45	m	13-16 years	0.014	651.9740565	67	Grand Index
45	m	13-16 years	0.015	652.8944955	67	Grand Index
45	m	13-16 years	0.016	655.9668609	68	Grand Index
45	m	13-16 years	0.017	658.0038189	68	Grand Index
45	m	13-16 years	0.018	659	69	Grand Index
45	m	13-16 years	0.019	659.594	69	Grand Index
45	m	13-16 years	0.02	662	69	Grand Index
45	m	13-16 years	0.021	662.5788164	69	Grand Index
45	m	13-16 years	0.022	663.372	70	Grand Index
45	m	13-16 years	0.023	668.0292721	70	Grand Index
45	m	13-16 years	0.024	669.027918	70	Grand Index
45	m	13-16 years	0.025	669.2642811	71	Grand Index
45	m	13-16 years	0.026	671.0638336	71	Grand Index
45	m	13-16 years	0.027	674.004	71	Grand Index
45	m	13-16 years	0.028	676	71	Grand Index
45	m	13-16 years	0.029	677.3710448	72	Grand Index
45	m	13-16 years	0.03	677.9528198	72	Grand Index
45	m	13-16 years	0.032	678	72	Grand Index
45	m	13-16 years	0.033	678.235202	72	Grand Index
45	m	13-16 years	0.034	679.484	73	Grand Index
45	m	13-16 years	0.035	680	73	Grand Index
45	m	13-16 years	0.036	680.336	73	Grand Index
45	m	13-16 years	0.037	681.182905	73	Grand Index
45	m	13-16 years	0.038	681.8936471	73	Grand Index
45	m	13-16 years	0.039	682	74	Grand Index
45	m	13-16 years	0.04	682.001957	74	Grand Index
45	m	13-16 years	0.041	682.1718227	74	Grand Index
45	m	13-16 years	0.042	683	74	Grand Index
45	m	13-16 years	0.043	683.8553819	74	Grand Index
45	m	13-16 years	0.044	684.1617923	74	Grand Index
45	m	13-16 years	0.045	685	75	Grand Index
45	m	13-16 years	0.046	685.9511406	75	Grand Index
45	m	13-16 years	0.047	687.1251447	75	Grand Index
45	m	13-16 years	0.048	687.9119317	75	Grand Index
45	m	13-16 years	0.049	688	75	Grand Index
45	m	13-16 years	0.05	689	75	Grand Index
45	m	13-16 years	0.051	690	75	Grand Index
45	m	13-16 years	0.052	691.003643	76	Grand Index
45	m	13-16 years	0.053	691.988765	76	Grand Index
45	m	13-16 years	0.054	693.5438655	76	Grand Index
45	m	13-16 years	0.055	694	76	Grand Index
45	m	13-16 years	0.056	694.856	76	Grand Index
45	m	13-16 years	0.057	695.8629923	76	Grand Index
45	m	13-16 years	0.059	696	77	Grand Index
45	m	13-16 years	0.06	696.56	77	Grand Index
45	m	13-16 years	0.061	697	77	Grand Index
45	m	13-16 years	0.062	697.1348986	77	Grand Index
45	m	13-16 years	0.063	697.9694129	77	Grand Index
45	m	13-16 years	0.064	699.6213312	77	Grand Index
45	m	13-16 years	0.065	700	77	Grand Index
45	m	13-16 years	0.066	701	77	Grand Index

Age	Gender	Education	Percentile	Raw Score	Norm Score	Assessment
45	m	13-16 years	0.067	701.084	78	Grand Index
45	m	13-16 years	0.068	703	78	Grand Index
45	m	13-16 years	0.069	703.894	78	Grand Index
45	m	13-16 years	0.071	704	78	Grand Index
45	m	13-16 years	0.072	704.4408992	78	Grand Index
45	m	13-16 years	0.074	705	78	Grand Index
45	m	13-16 years	0.075	706	78	Grand Index
45	m	13-16 years	0.076	706.5438782	79	Grand Index
45	m	13-16 years	0.077	707	79	Grand Index
45	m	13-16 years	0.078	707.550782	79	Grand Index
45	m	13-16 years	0.079	708	79	Grand Index
45	m	13-16 years	0.08	708.08	79	Grand Index
45	m	13-16 years	0.081	709.0039633	79	Grand Index
45	m	13-16 years	0.082	709.7042343	79	Grand Index
45	m	13-16 years	0.083	710	79	Grand Index
45	m	13-16 years	0.084	711	79	Grand Index
45	m	13-16 years	0.085	711.7829693	79	Grand Index
45	m	13-16 years	0.086	712	80	Grand Index
45	m	13-16 years	0.087	712.645565	80	Grand Index
45	m	13-16 years	0.089	713	80	Grand Index
45	m	13-16 years	0.09	714	80	Grand Index
45	m	13-16 years	0.091	714.364581	80	Grand Index
45	m	13-16 years	0.092	715	80	Grand Index
45	m	13-16 years	0.093	715.5307968	80	Grand Index
45	m	13-16 years	0.094	715.8952368	80	Grand Index
45	m	13-16 years	0.096	716	80	Grand Index
45	m	13-16 years	0.097	717.4790884	81	Grand Index
45	m	13-16 years	0.099	718	81	Grand Index
45	m	13-16 years	0.103	719	81	Grand Index
45	m	13-16 years	0.104	719.304	81	Grand Index
45	m	13-16 years	0.106	720	81	Grand Index
45	m	13-16 years	0.107	720.2291471	81	Grand Index
45	m	13-16 years	0.109	721	82	Grand Index
45	m	13-16 years	0.11	721.5124225	82	Grand Index
45	m	13-16 years	0.111	722	82	Grand Index
45	m	13-16 years	0.113	723	82	Grand Index
45	m	13-16 years	0.114	724	82	Grand Index
45	m	13-16 years	0.115	724.4482751	82	Grand Index
45	m	13-16 years	0.119	725	82	Grand Index
45	m	13-16 years	0.12	725.12	82	Grand Index
45	m	13-16 years	0.123	726	83	Grand Index
45	m	13-16 years	0.124	726.7415048	83	Grand Index
45	m	13-16 years	0.125	726.9419704	83	Grand Index
45	m	13-16 years	0.126	727	83	Grand Index
45	m	13-16 years	0.127	727.0137133	83	Grand Index
45	m	13-16 years	0.128	727.556825	83	Grand Index
45	m	13-16 years	0.129	728	83	Grand Index
45	m	13-16 years	0.13	729	83	Grand Index
45	m	13-16 years	0.131	729.3885797	83	Grand Index
45	m	13-16 years	0.132	729.971813	83	Grand Index
45	m	13-16 years	0.133	730	83	Grand Index
45	m	13-16 years	0.134	730.0199712	83	Grand Index
45	m	13-16 years	0.135	730.8875242	83	Grand Index
45	m	13-16 years	0.137	731	84	Grand Index
45	m	13-16 years	0.138	731.8170321	84	Grand Index
45	m	13-16 years	0.141	732	84	Grand Index

Age	Gender	Education	Percentile	Raw Score	Norm Score	Assessment
45	m	13-16 years	0.142	733	84	Grand Index
45	m	13-16 years	0.145	734	84	Grand Index
45	m	13-16 years	0.146	734.8864769	84	Grand Index
45	m	13-16 years	0.148	735	84	Grand Index
45	m	13-16 years	0.149	735.0248012	84	Grand Index
45	m	13-16 years	0.15	735.9141713	84	Grand Index
45	m	13-16 years	0.152	736	85	Grand Index
45	m	13-16 years	0.156	737	85	Grand Index
45	m	13-16 years	0.157	737.5075552	85	Grand Index
45	m	13-16 years	0.159	738	85	Grand Index
45	m	13-16 years	0.16	738.16	85	Grand Index
45	m	13-16 years	0.163	739	85	Grand Index
45	m	13-16 years	0.165	740	85	Grand Index
45	m	13-16 years	0.166	740.0958685	85	Grand Index
45	m	13-16 years	0.167	740.9611422	86	Grand Index
45	m	13-16 years	0.171	741	86	Grand Index
45	m	13-16 years	0.172	741.0068412	86	Grand Index
45	m	13-16 years	0.173	741.6953176	86	Grand Index
45	m	13-16 years	0.174	742.2081481	86	Grand Index
45	m	13-16 years	0.177	743	86	Grand Index
45	m	13-16 years	0.178	743.828	86	Grand Index
45	m	13-16 years	0.18	744	86	Grand Index
45	m	13-16 years	0.181	744.054994	86	Grand Index
45	m	13-16 years	0.184	745	86	Grand Index
45	m	13-16 years	0.19	747	87	Grand Index
45	m	13-16 years	0.194	748	87	Grand Index
45	m	13-16 years	0.197	749	87	Grand Index
45	m	13-16 years	0.198	749.348	87	Grand Index
45	m	13-16 years	0.2	750	87	Grand Index
45	m	13-16 years	0.206	751	88	Grand Index
45	m	13-16 years	0.207	751.8554132	88	Grand Index
45	m	13-16 years	0.211	752	88	Grand Index
45	m	13-16 years	0.212	752.060224	88	Grand Index
45	m	13-16 years	0.213	752.6879259	88	Grand Index
45	m	13-16 years	0.214	752.9523331	88	Grand Index
45	m	13-16 years	0.217	753	88	Grand Index
45	m	13-16 years	0.218	753.868	88	Grand Index
45	m	13-16 years	0.222	754	89	Grand Index
45	m	13-16 years	0.223	754.4922676	89	Grand Index
45	m	13-16 years	0.225	755	89	Grand Index
45	m	13-16 years	0.226	755.3193844	89	Grand Index
45	m	13-16 years	0.23	756	89	Grand Index
45	m	13-16 years	0.232	757	89	Grand Index
45	m	13-16 years	0.233	757.1120139	89	Grand Index
45	m	13-16 years	0.237	758	89	Grand Index
45	m	13-16 years	0.238	758.388	89	Grand Index
45	m	13-16 years	0.24	759	89	Grand Index
45	m	13-16 years	0.241	759.039797	89	Grand Index
45	m	13-16 years	0.246	760	90	Grand Index
45	m	13-16 years	0.248	761	90	Grand Index
45	m	13-16 years	0.249	761.0862521	90	Grand Index
45	m	13-16 years	0.25	761.5213141	90	Grand Index
45	m	13-16 years	0.252	762	90	Grand Index
45	m	13-16 years	0.253	762.2218985	90	Grand Index
45	m	13-16 years	0.258	763	90	Grand Index
45	m	13-16 years	0.26	764	90	Grand Index

Age	Gender	Education	Percentile	Raw Score	Norm Score	Assessment
45	m	13-16 years	0.261	764.686	90	Grand Index
45	m	13-16 years	0.263	765	90	Grand Index
45	m	13-16 years	0.264	765.2558356	91	Grand Index
45	m	13-16 years	0.267	766	91	Grand Index
45	m	13-16 years	0.268	766.168	91	Grand Index
45	m	13-16 years	0.269	767	91	Grand Index
45	m	13-16 years	0.27	767.0913012	91	Grand Index
45	m	13-16 years	0.271	767.30535	91	Grand Index
45	m	13-16 years	0.272	767.9538207	91	Grand Index
45	m	13-16 years	0.276	768	91	Grand Index
45	m	13-16 years	0.277	768.0950911	91	Grand Index
45	m	13-16 years	0.278	768.8624272	91	Grand Index
45	m	13-16 years	0.281	769	91	Grand Index
45	m	13-16 years	0.282	769.1245534	91	Grand Index
45	m	13-16 years	0.286	770	92	Grand Index
45	m	13-16 years	0.287	770.8400716	92	Grand Index
45	m	13-16 years	0.289	771	92	Grand Index
45	m	13-16 years	0.29	771.0377215	92	Grand Index
45	m	13-16 years	0.291	771.5370224	92	Grand Index
45	m	13-16 years	0.296	772	92	Grand Index
45	m	13-16 years	0.297	772.0076697	92	Grand Index
45	m	13-16 years	0.298	772.9661284	92	Grand Index
45	m	13-16 years	0.301	773	92	Grand Index
45	m	13-16 years	0.302	773.0737623	92	Grand Index
45	m	13-16 years	0.305	774	92	Grand Index
45	m	13-16 years	0.306	774.2486388	92	Grand Index
45	m	13-16 years	0.313	775	93	Grand Index
45	m	13-16 years	0.314	775.9051038	93	Grand Index
45	m	13-16 years	0.316	776	93	Grand Index
45	m	13-16 years	0.317	776.5262566	93	Grand Index
45	m	13-16 years	0.32	777	93	Grand Index
45	m	13-16 years	0.323	778	93	Grand Index
45	m	13-16 years	0.324	778.9965498	93	Grand Index
45	m	13-16 years	0.331	779	93	Grand Index
45	m	13-16 years	0.332	779.9961947	93	Grand Index
45	m	13-16 years	0.336	780	94	Grand Index
45	m	13-16 years	0.337	780.1040628	94	Grand Index
45	m	13-16 years	0.338	780.9925494	94	Grand Index
45	m	13-16 years	0.343	781	94	Grand Index
45	m	13-16 years	0.348	782	94	Grand Index
45	m	13-16 years	0.349	782.174	94	Grand Index
45	m	13-16 years	0.354	783	94	Grand Index
45	m	13-16 years	0.355	783.0706253	94	Grand Index
45	m	13-16 years	0.356	783.8531625	94	Grand Index
45	m	13-16 years	0.358	784	95	Grand Index
45	m	13-16 years	0.359	784.5450862	95	Grand Index
45	m	13-16 years	0.365	785	95	Grand Index
45	m	13-16 years	0.366	785.9756014	95	Grand Index
45	m	13-16 years	0.368	786	95	Grand Index
45	m	13-16 years	0.369	786.21347	95	Grand Index
45	m	13-16 years	0.371	787	95	Grand Index
45	m	13-16 years	0.376	788	95	Grand Index
45	m	13-16 years	0.377	788.0530981	95	Grand Index
45	m	13-16 years	0.378	788.8389045	95	Grand Index
45	m	13-16 years	0.382	789	95	Grand Index
45	m	13-16 years	0.383	789.4487864	96	Grand Index

Age	Gender	Education	Percentile	Raw Score	Norm Score	Assessment
45	m	13-16 years	0.386	790	96	Grand Index
45	m	13-16 years	0.387	790.1755475	96	Grand Index
45	m	13-16 years	0.394	791	96	Grand Index
45	m	13-16 years	0.395	791.77	96	Grand Index
45	m	13-16 years	0.4	792	96	Grand Index
45	m	13-16 years	0.401	792.326	96	Grand Index
45	m	13-16 years	0.403	793	96	Grand Index
45	m	13-16 years	0.404	793.6132277	96	Grand Index
45	m	13-16 years	0.409	794	97	Grand Index
45	m	13-16 years	0.41	794.6329229	97	Grand Index
45	m	13-16 years	0.412	795	97	Grand Index
45	m	13-16 years	0.413	795.2559775	97	Grand Index
45	m	13-16 years	0.417	796	97	Grand Index
45	m	13-16 years	0.418	796.7181112	97	Grand Index
45	m	13-16 years	0.419	796.9996306	97	Grand Index
45	m	13-16 years	0.424	797	97	Grand Index
45	m	13-16 years	0.428	798	97	Grand Index
45	m	13-16 years	0.429	798.7212773	97	Grand Index
45	m	13-16 years	0.433	799	97	Grand Index
45	m	13-16 years	0.437	800	98	Grand Index
45	m	13-16 years	0.438	800.0350343	98	Grand Index
45	m	13-16 years	0.441	801	98	Grand Index
45	m	13-16 years	0.442	801.4621549	98	Grand Index
45	m	13-16 years	0.447	802	98	Grand Index
45	m	13-16 years	0.448	802.600239	98	Grand Index
45	m	13-16 years	0.449	802.9964679	98	Grand Index
45	m	13-16 years	0.451	803	98	Grand Index
45	m	13-16 years	0.452	803.5563504	98	Grand Index
45	m	13-16 years	0.456	804	98	Grand Index
45	m	13-16 years	0.457	804.2297295	98	Grand Index
45	m	13-16 years	0.46	805	98	Grand Index
45	m	13-16 years	0.461	805.0790035	99	Grand Index
45	m	13-16 years	0.466	806	99	Grand Index
45	m	13-16 years	0.467	806.0949224	99	Grand Index
45	m	13-16 years	0.469	807	99	Grand Index
45	m	13-16 years	0.47	807.2587611	99	Grand Index
45	m	13-16 years	0.478	808	99	Grand Index
45	m	13-16 years	0.479	808.3938088	99	Grand Index
45	m	13-16 years	0.483	809	99	Grand Index
45	m	13-16 years	0.484	809.0734143	99	Grand Index
45	m	13-16 years	0.485	809.8478391	99	Grand Index
45	m	13-16 years	0.489	810	100	Grand Index
45	m	13-16 years	0.49	810.0745663	100	Grand Index
45	m	13-16 years	0.498	811	100	Grand Index
45	m	13-16 years	0.499	811.0592246	100	Grand Index
45	m	13-16 years	0.503	812	100	Grand Index
45	m	13-16 years	0.504	812.5310055	100	Grand Index
45	m	13-16 years	0.505	812.9392768	100	Grand Index
45	m	13-16 years	0.509	813	100	Grand Index
45	m	13-16 years	0.51	813.0188481	100	Grand Index
45	m	13-16 years	0.511	813.9037555	100	Grand Index
45	m	13-16 years	0.513	814	100	Grand Index
45	m	13-16 years	0.514	814.964	101	Grand Index
45	m	13-16 years	0.518	815	101	Grand Index
45	m	13-16 years	0.519	815.594	101	Grand Index
45	m	13-16 years	0.523	816	101	Grand Index

Age	Gender	Education	Percentile	Raw Score	Norm Score	Assessment
45	m	13-16 years	0.524	816.0023963	101	Grand Index
45	m	13-16 years	0.525	816.8099138	101	Grand Index
45	m	13-16 years	0.529	817	101	Grand Index
45	m	13-16 years	0.53	817.3302932	101	Grand Index
45	m	13-16 years	0.535	818	101	Grand Index
45	m	13-16 years	0.536	818.967533	101	Grand Index
45	m	13-16 years	0.542	819	102	Grand Index
45	m	13-16 years	0.543	819.6700429	102	Grand Index
45	m	13-16 years	0.544	819.9748508	102	Grand Index
45	m	13-16 years	0.549	820	102	Grand Index
45	m	13-16 years	0.553	821	102	Grand Index
45	m	13-16 years	0.558	822	102	Grand Index
45	m	13-16 years	0.559	822.6833202	102	Grand Index
45	m	13-16 years	0.562	823	102	Grand Index
45	m	13-16 years	0.563	823.338	102	Grand Index
45	m	13-16 years	0.565	824	102	Grand Index
45	m	13-16 years	0.566	824.5915437	102	Grand Index
45	m	13-16 years	0.57	825	103	Grand Index
45	m	13-16 years	0.571	825.746	103	Grand Index
45	m	13-16 years	0.576	826	103	Grand Index
45	m	13-16 years	0.582	827	103	Grand Index
45	m	13-16 years	0.583	827.858	103	Grand Index
45	m	13-16 years	0.586	828	103	Grand Index
45	m	13-16 years	0.591	829	103	Grand Index
45	m	13-16 years	0.592	829.1571131	103	Grand Index
45	m	13-16 years	0.593	829.980912	104	Grand Index
45	m	13-16 years	0.595	830	104	Grand Index
45	m	13-16 years	0.596	830.1904171	104	Grand Index
45	m	13-16 years	0.6	831	104	Grand Index
45	m	13-16 years	0.601	831.5164497	104	Grand Index
45	m	13-16 years	0.606	832	104	Grand Index
45	m	13-16 years	0.607	832.082	104	Grand Index
45	m	13-16 years	0.609	833	104	Grand Index
45	m	13-16 years	0.61	833.2694107	104	Grand Index
45	m	13-16 years	0.611	833.9431765	104	Grand Index
45	m	13-16 years	0.616	834	104	Grand Index
45	m	13-16 years	0.617	834.9981651	104	Grand Index
45	m	13-16 years	0.62	835	105	Grand Index
45	m	13-16 years	0.621	835.046	105	Grand Index
45	m	13-16 years	0.625	836	105	Grand Index
45	m	13-16 years	0.626	836.0197218	105	Grand Index
45	m	13-16 years	0.629	837	105	Grand Index
45	m	13-16 years	0.63	837.6989807	105	Grand Index
45	m	13-16 years	0.634	838	105	Grand Index
45	m	13-16 years	0.639	839	105	Grand Index
45	m	13-16 years	0.644	840	106	Grand Index
45	m	13-16 years	0.645	840.27	106	Grand Index
45	m	13-16 years	0.648	841	106	Grand Index
45	m	13-16 years	0.649	841.0791902	106	Grand Index
45	m	13-16 years	0.65	841.9915716	106	Grand Index
45	m	13-16 years	0.654	842	106	Grand Index
45	m	13-16 years	0.655	842.53	106	Grand Index
45	m	13-16 years	0.658	843	106	Grand Index
45	m	13-16 years	0.659	843.0436727	106	Grand Index
45	m	13-16 years	0.66	843.493297	106	Grand Index
45	m	13-16 years	0.661	844	106	Grand Index

Age	Gender	Education	Percentile	Raw Score	Norm Score	Assessment
45	m	13-16 years	0.662	844.5045179	106	Grand Index
45	m	13-16 years	0.666	845	106	Grand Index
45	m	13-16 years	0.667	845.9305272	106	Grand Index
45	m	13-16 years	0.671	846	107	Grand Index
45	m	13-16 years	0.672	846.272	107	Grand Index
45	m	13-16 years	0.675	847	107	Grand Index
45	m	13-16 years	0.676	847.2870588	107	Grand Index
45	m	13-16 years	0.677	847.9347147	107	Grand Index
45	m	13-16 years	0.68	848	107	Grand Index
45	m	13-16 years	0.681	848.5517756	107	Grand Index
45	m	13-16 years	0.684	849	107	Grand Index
45	m	13-16 years	0.685	849.31	107	Grand Index
45	m	13-16 years	0.689	850	107	Grand Index
45	m	13-16 years	0.69	850.94	107	Grand Index
45	m	13-16 years	0.695	851	108	Grand Index
45	m	13-16 years	0.696	851.0863564	108	Grand Index
45	m	13-16 years	0.699	852	108	Grand Index
45	m	13-16 years	0.7	852.3978044	108	Grand Index
45	m	13-16 years	0.701	852.9880875	108	Grand Index
45	m	13-16 years	0.708	853	108	Grand Index
45	m	13-16 years	0.709	853.6639279	108	Grand Index
45	m	13-16 years	0.713	854	108	Grand Index
45	m	13-16 years	0.714	854.3909099	108	Grand Index
45	m	13-16 years	0.719	855	109	Grand Index
45	m	13-16 years	0.72	855.7258175	109	Grand Index
45	m	13-16 years	0.724	856	109	Grand Index
45	m	13-16 years	0.728	857	109	Grand Index
45	m	13-16 years	0.729	857.8747522	109	Grand Index
45	m	13-16 years	0.73	857.9988603	109	Grand Index
45	m	13-16 years	0.732	858	109	Grand Index
45	m	13-16 years	0.733	858.758	109	Grand Index
45	m	13-16 years	0.736	859	109	Grand Index
45	m	13-16 years	0.737	859.574766	110	Grand Index
45	m	13-16 years	0.74	860	110	Grand Index
45	m	13-16 years	0.741	860.196948	110	Grand Index
45	m	13-16 years	0.745	861	110	Grand Index
45	m	13-16 years	0.746	861.110254	110	Grand Index
45	m	13-16 years	0.749	862	110	Grand Index
45	m	13-16 years	0.75	862.572189	110	Grand Index
45	m	13-16 years	0.754	863	110	Grand Index
45	m	13-16 years	0.755	864	110	Grand Index
45	m	13-16 years	0.756	864.0140889	110	Grand Index
45	m	13-16 years	0.757	864.7674763	110	Grand Index
45	m	13-16 years	0.759	865	111	Grand Index
45	m	13-16 years	0.76	865.8497952	111	Grand Index
45	m	13-16 years	0.764	866	111	Grand Index
45	m	13-16 years	0.766	867	111	Grand Index
45	m	13-16 years	0.767	867.0009121	111	Grand Index
45	m	13-16 years	0.768	867.2675782	111	Grand Index
45	m	13-16 years	0.773	868	111	Grand Index
45	m	13-16 years	0.774	868.6732749	111	Grand Index
45	m	13-16 years	0.775	869	111	Grand Index
45	m	13-16 years	0.776	869.2728685	111	Grand Index
45	m	13-16 years	0.777	869.9460343	111	Grand Index
45	m	13-16 years	0.78	870	112	Grand Index
45	m	13-16 years	0.781	870.2026928	112	Grand Index

Age	Gender	Education	Percentile	Raw Score	Norm Score	Assessment
45	m	13-16 years	0.784	871	112	Grand Index
45	m	13-16 years	0.785	871.91	112	Grand Index
45	m	13-16 years	0.787	872	112	Grand Index
45	m	13-16 years	0.788	872.1587212	112	Grand Index
45	m	13-16 years	0.789	872.7091924	112	Grand Index
45	m	13-16 years	0.79	873.1549644	112	Grand Index
45	m	13-16 years	0.791	874	112	Grand Index
45	m	13-16 years	0.792	874.1016597	112	Grand Index
45	m	13-16 years	0.795	875	112	Grand Index
45	m	13-16 years	0.796	875.4606058	112	Grand Index
45	m	13-16 years	0.8	876	113	Grand Index
45	m	13-16 years	0.801	876.3008324	113	Grand Index
45	m	13-16 years	0.802	876.9444243	113	Grand Index
45	m	13-16 years	0.803	877.5139031	113	Grand Index
45	m	13-16 years	0.804	877.8022493	113	Grand Index
45	m	13-16 years	0.808	878	113	Grand Index
45	m	13-16 years	0.809	878.134	113	Grand Index
45	m	13-16 years	0.812	879	113	Grand Index
45	m	13-16 years	0.813	879.838	113	Grand Index
45	m	13-16 years	0.814	880	113	Grand Index
45	m	13-16 years	0.815	880.7018609	113	Grand Index
45	m	13-16 years	0.817	881	114	Grand Index
45	m	13-16 years	0.818	881.0541739	114	Grand Index
45	m	13-16 years	0.819	882	114	Grand Index
45	m	13-16 years	0.82	882.1048523	114	Grand Index
45	m	13-16 years	0.823	883	114	Grand Index
45	m	13-16 years	0.824	883.0025015	114	Grand Index
45	m	13-16 years	0.825	883.1364252	114	Grand Index
45	m	13-16 years	0.826	884	114	Grand Index
45	m	13-16 years	0.827	884.5948588	114	Grand Index
45	m	13-16 years	0.828	884.9655035	114	Grand Index
45	m	13-16 years	0.831	885	114	Grand Index
45	m	13-16 years	0.832	886	114	Grand Index
45	m	13-16 years	0.833	886.358	114	Grand Index
45	m	13-16 years	0.835	887	115	Grand Index
45	m	13-16 years	0.836	887.9930984	115	Grand Index
45	m	13-16 years	0.838	888	115	Grand Index
45	m	13-16 years	0.839	888.914	115	Grand Index
45	m	13-16 years	0.841	889	115	Grand Index
45	m	13-16 years	0.842	889.5508239	115	Grand Index
45	m	13-16 years	0.844	890	115	Grand Index
45	m	13-16 years	0.846	891	115	Grand Index
45	m	13-16 years	0.847	891.3183023	115	Grand Index
45	m	13-16 years	0.849	892	115	Grand Index
45	m	13-16 years	0.85	892.5452486	116	Grand Index
45	m	13-16 years	0.852	893	116	Grand Index
45	m	13-16 years	0.853	893.940125	116	Grand Index
45	m	13-16 years	0.855	894	116	Grand Index
45	m	13-16 years	0.856	894.9883484	116	Grand Index
45	m	13-16 years	0.859	895	116	Grand Index
45	m	13-16 years	0.86	895.6712275	116	Grand Index
45	m	13-16 years	0.861	896	116	Grand Index
45	m	13-16 years	0.862	897	116	Grand Index
45	m	13-16 years	0.866	898	117	Grand Index
45	m	13-16 years	0.87	899	117	Grand Index
45	m	13-16 years	0.871	900	117	Grand Index

Age	Gender	Education	Percentile	Raw Score	Norm Score	Assessment
45	m	13-16 years	0.873	901	117	Grand Index
45	m	13-16 years	0.874	902	117	Grand Index
45	m	13-16 years	0.877	903	117	Grand Index
45	m	13-16 years	0.878	903.1500988	117	Grand Index
45	m	13-16 years	0.879	903.9615397	118	Grand Index
45	m	13-16 years	0.88	904	118	Grand Index
45	m	13-16 years	0.881	904.1529483	118	Grand Index
45	m	13-16 years	0.883	905	118	Grand Index
45	m	13-16 years	0.886	906	118	Grand Index
45	m	13-16 years	0.887	906.2445879	118	Grand Index
45	m	13-16 years	0.888	907	118	Grand Index
45	m	13-16 years	0.889	907.853911	118	Grand Index
45	m	13-16 years	0.891	908	118	Grand Index
45	m	13-16 years	0.892	908.2509539	119	Grand Index
45	m	13-16 years	0.894	909	119	Grand Index
45	m	13-16 years	0.895	909.77	119	Grand Index
45	m	13-16 years	0.896	910.696	119	Grand Index
45	m	13-16 years	0.897	911.5209969	119	Grand Index
45	m	13-16 years	0.898	911.9781611	119	Grand Index
45	m	13-16 years	0.9	912	119	Grand Index
45	m	13-16 years	0.901	912.5511342	119	Grand Index
45	m	13-16 years	0.902	913.0434394	119	Grand Index
45	m	13-16 years	0.903	914	119	Grand Index
45	m	13-16 years	0.904	915.2090746	120	Grand Index
45	m	13-16 years	0.905	916	120	Grand Index
45	m	13-16 years	0.906	916.956	120	Grand Index
45	m	13-16 years	0.909	917	120	Grand Index
45	m	13-16 years	0.91	917.66	120	Grand Index
45	m	13-16 years	0.911	918.9818542	120	Grand Index
45	m	13-16 years	0.912	919.512	120	Grand Index
45	m	13-16 years	0.915	920	121	Grand Index
45	m	13-16 years	0.916	920.1955101	121	Grand Index
45	m	13-16 years	0.917	920.6794657	121	Grand Index
45	m	13-16 years	0.919	921	121	Grand Index
45	m	13-16 years	0.92	921.92	121	Grand Index
45	m	13-16 years	0.921	922.6923077	121	Grand Index
45	m	13-16 years	0.922	923.0559046	121	Grand Index
45	m	13-16 years	0.923	923.8082876	121	Grand Index
45	m	13-16 years	0.924	924.624	121	Grand Index
45	m	13-16 years	0.925	925.4173633	122	Grand Index
45	m	13-16 years	0.926	926	122	Grand Index
45	m	13-16 years	0.927	926.3962063	122	Grand Index
45	m	13-16 years	0.928	927	122	Grand Index
45	m	13-16 years	0.929	927.254	122	Grand Index
45	m	13-16 years	0.93	928.18	122	Grand Index
45	m	13-16 years	0.932	929	122	Grand Index
45	m	13-16 years	0.933	929.636879	122	Grand Index
45	m	13-16 years	0.934	930	123	Grand Index
45	m	13-16 years	0.935	931.8765934	123	Grand Index
45	m	13-16 years	0.936	932	123	Grand Index
45	m	13-16 years	0.937	932.19505	123	Grand Index
45	m	13-16 years	0.938	933	123	Grand Index
45	m	13-16 years	0.94	934	123	Grand Index
45	m	13-16 years	0.941	934.366	123	Grand Index
45	m	13-16 years	0.943	935	124	Grand Index
45	m	13-16 years	0.945	936	124	Grand Index

Age	Gender	Education	Percentile	Raw Score	Norm Score	Assessment
45	m	13-16 years	0.946	936.996	124	Grand Index
45	m	13-16 years	0.947	937.922	124	Grand Index
45	m	13-16 years	0.948	938.848	124	Grand Index
45	m	13-16 years	0.949	939.9264755	125	Grand Index
45	m	13-16 years	0.95	940	125	Grand Index
45	m	13-16 years	0.951	940.6681532	125	Grand Index
45	m	13-16 years	0.952	941.8587273	125	Grand Index
45	m	13-16 years	0.953	942	125	Grand Index
45	m	13-16 years	0.954	942.6500377	125	Grand Index
45	m	13-16 years	0.955	943	125	Grand Index
45	m	13-16 years	0.956	943.256	126	Grand Index
45	m	13-16 years	0.957	944.0023452	126	Grand Index
45	m	13-16 years	0.958	945.108	126	Grand Index
45	m	13-16 years	0.959	946.1455421	126	Grand Index
45	m	13-16 years	0.96	947.4715035	126	Grand Index
45	m	13-16 years	0.961	949.7722101	126	Grand Index
45	m	13-16 years	0.962	950.8323068	127	Grand Index
45	m	13-16 years	0.963	951	127	Grand Index
45	m	13-16 years	0.964	952	127	Grand Index
45	m	13-16 years	0.965	952.59	127	Grand Index
45	m	13-16 years	0.966	953.6821675	127	Grand Index
45	m	13-16 years	0.967	954.378568	128	Grand Index
45	m	13-16 years	0.968	954.8007045	128	Grand Index
45	m	13-16 years	0.969	956	128	Grand Index
45	m	13-16 years	0.97	957	128	Grand Index
45	m	13-16 years	0.971	958	128	Grand Index
45	m	13-16 years	0.972	960.072	129	Grand Index
45	m	13-16 years	0.973	961	129	Grand Index
45	m	13-16 years	0.974	961.8532811	129	Grand Index
45	m	13-16 years	0.975	963.4273338	129	Grand Index
45	m	13-16 years	0.976	964.6636276	130	Grand Index
45	m	13-16 years	0.977	965.9428534	130	Grand Index
45	m	13-16 years	0.978	967.256	130	Grand Index
45	m	13-16 years	0.979	970.594161	131	Grand Index
45	m	13-16 years	0.98	971.5870756	131	Grand Index
45	m	13-16 years	0.981	973.406	131	Grand Index
45	m	13-16 years	0.982	974.1963834	131	Grand Index
45	m	13-16 years	0.983	976.5120596	132	Grand Index
45	m	13-16 years	0.984	978.6498744	132	Grand Index
45	m	13-16 years	0.985	979.39832	133	Grand Index
45	m	13-16 years	0.986	983.254527	133	Grand Index
45	m	13-16 years	0.987	990.8493521	133	Grand Index
45	m	13-16 years	0.988	992.2640261	134	Grand Index
45	m	13-16 years	0.989	993	134	Grand Index
45	m	13-16 years	0.991	995	135	Grand Index
45	m	13-16 years	0.992	996.0410703	136	Grand Index
45	m	13-16 years	0.993	998.0903877	137	Grand Index
45	m	13-16 years	0.994	1000.530579	138	Grand Index
45	m	13-16 years	0.995	1002.821113	139	Grand Index
45	m	13-16 years	0.996	1016.855874	140	Grand Index
45	m	13-16 years	0.997	1029.491235	141	Grand Index
45	m	13-16 years	0.998	1043.87998	143	Grand Index
45	m	13-16 years	0.999	1056.401092	146	Grand Index
45	f	17+ years	0.001	605.7808302	54	Grand Index
45	f	17+ years	0.002	614.576	57	Grand Index
45	f	17+ years	0.003	618.092	59	Grand Index

Age	Gender	Education	Percentile	Raw Score	Norm Score	Assessment
45	f	17+ years	0.004	628.304	60	Grand Index
45	f	17+ years	0.005	632.3895045	61	Grand Index
45	f	17+ years	0.006	633.7096078	62	Grand Index
45	f	17+ years	0.007	637.5661319	63	Grand Index
45	f	17+ years	0.008	640.3570446	64	Grand Index
45	f	17+ years	0.009	641.0692487	65	Grand Index
45	f	17+ years	0.01	641.9703244	65	Grand Index
45	f	17+ years	0.011	642.8635565	66	Grand Index
45	f	17+ years	0.012	644.1195941	66	Grand Index
45	f	17+ years	0.013	647	67	Grand Index
45	f	17+ years	0.015	649	67	Grand Index
45	f	17+ years	0.016	652.608	68	Grand Index
45	f	17+ years	0.017	656.2106359	68	Grand Index
45	f	17+ years	0.018	658.790803	69	Grand Index
45	f	17+ years	0.019	659.7084365	69	Grand Index
45	f	17+ years	0.02	662.8751799	69	Grand Index
45	f	17+ years	0.021	664	69	Grand Index
45	f	17+ years	0.022	664.8900948	70	Grand Index
45	f	17+ years	0.023	665.124	70	Grand Index
45	f	17+ years	0.024	666.513119	70	Grand Index
45	f	17+ years	0.025	667	71	Grand Index
45	f	17+ years	0.026	667.488	71	Grand Index
45	f	17+ years	0.027	668.828	71	Grand Index
45	f	17+ years	0.028	671.064	71	Grand Index
45	f	17+ years	0.029	672.852	72	Grand Index
45	f	17+ years	0.03	673	72	Grand Index
45	f	17+ years	0.031	675.1402323	72	Grand Index
45	f	17+ years	0.032	675.5317225	72	Grand Index
45	f	17+ years	0.033	677	72	Grand Index
45	f	17+ years	0.034	678.2238182	73	Grand Index
45	f	17+ years	0.035	679.7565217	73	Grand Index
45	f	17+ years	0.036	681.368	73	Grand Index
45	f	17+ years	0.037	682.0026351	73	Grand Index
45	f	17+ years	0.038	682.6708155	73	Grand Index
45	f	17+ years	0.039	684.1992526	74	Grand Index
45	f	17+ years	0.04	685.4231872	74	Grand Index
45	f	17+ years	0.041	686	74	Grand Index
45	f	17+ years	0.042	687.096	74	Grand Index
45	f	17+ years	0.043	688	74	Grand Index
45	f	17+ years	0.044	689	74	Grand Index
45	f	17+ years	0.045	689.46	75	Grand Index
45	f	17+ years	0.046	690.248	75	Grand Index
45	f	17+ years	0.047	691	75	Grand Index
45	f	17+ years	0.048	691.824	75	Grand Index
45	f	17+ years	0.049	693.612	75	Grand Index
45	f	17+ years	0.05	694	75	Grand Index
45	f	17+ years	0.051	694.188	75	Grand Index
45	f	17+ years	0.052	695	76	Grand Index
45	f	17+ years	0.053	695.8055979	76	Grand Index
45	f	17+ years	0.054	696	76	Grand Index
45	f	17+ years	0.055	696.0591068	76	Grand Index
45	f	17+ years	0.056	696.799461	76	Grand Index
45	f	17+ years	0.057	697	76	Grand Index
45	f	17+ years	0.058	697.7032853	76	Grand Index
45	f	17+ years	0.06	698	77	Grand Index
45	f	17+ years	0.061	699	77	Grand Index

Age	Gender	Education	Percentile	Raw Score	Norm Score	Assessment
45	f	17+ years	0.062	699.0104763	77	Grand Index
45	f	17+ years	0.063	700.0869223	77	Grand Index
45	f	17+ years	0.066	702	77	Grand Index
45	f	17+ years	0.067	702.796	78	Grand Index
45	f	17+ years	0.068	703.0149931	78	Grand Index
45	f	17+ years	0.069	704	78	Grand Index
45	f	17+ years	0.073	705	78	Grand Index
45	f	17+ years	0.074	706.295094	78	Grand Index
45	f	17+ years	0.076	708	79	Grand Index
45	f	17+ years	0.077	708.676	79	Grand Index
45	f	17+ years	0.078	709.1499211	79	Grand Index
45	f	17+ years	0.079	709.9903468	79	Grand Index
45	f	17+ years	0.081	710	79	Grand Index
45	f	17+ years	0.082	710.616	79	Grand Index
45	f	17+ years	0.083	711	79	Grand Index
45	f	17+ years	0.084	711.2263157	79	Grand Index
45	f	17+ years	0.086	712	80	Grand Index
45	f	17+ years	0.087	712.4925107	80	Grand Index
45	f	17+ years	0.088	712.9852479	80	Grand Index
45	f	17+ years	0.089	713.1177947	80	Grand Index
45	f	17+ years	0.09	713.9913907	80	Grand Index
45	f	17+ years	0.091	714	80	Grand Index
45	f	17+ years	0.092	714.5640734	80	Grand Index
45	f	17+ years	0.093	715.8053753	80	Grand Index
45	f	17+ years	0.094	716.0034032	80	Grand Index
45	f	17+ years	0.095	716.5467822	80	Grand Index
45	f	17+ years	0.096	717.3753193	80	Grand Index
45	f	17+ years	0.097	717.847611	81	Grand Index
45	f	17+ years	0.098	717.9495977	81	Grand Index
45	f	17+ years	0.099	718	81	Grand Index
45	f	17+ years	0.1	718.8	81	Grand Index
45	f	17+ years	0.101	719.588	81	Grand Index
45	f	17+ years	0.102	720	81	Grand Index
45	f	17+ years	0.103	720.164	81	Grand Index
45	f	17+ years	0.107	721	81	Grand Index
45	f	17+ years	0.109	722	82	Grand Index
45	f	17+ years	0.11	722.0203033	82	Grand Index
45	f	17+ years	0.111	722.9765025	82	Grand Index
45	f	17+ years	0.112	723	82	Grand Index
45	f	17+ years	0.115	724	82	Grand Index
45	f	17+ years	0.116	724.9577544	82	Grand Index
45	f	17+ years	0.118	725	82	Grand Index
45	f	17+ years	0.123	726	83	Grand Index
45	f	17+ years	0.126	727	83	Grand Index
45	f	17+ years	0.127	727.076	83	Grand Index
45	f	17+ years	0.128	728	83	Grand Index
45	f	17+ years	0.129	728.7694597	83	Grand Index
45	f	17+ years	0.131	729	83	Grand Index
45	f	17+ years	0.132	729.0042019	83	Grand Index
45	f	17+ years	0.133	729.8554737	83	Grand Index
45	f	17+ years	0.135	730	83	Grand Index
45	f	17+ years	0.136	730.4512738	84	Grand Index
45	f	17+ years	0.14	731	84	Grand Index
45	f	17+ years	0.141	731.1164595	84	Grand Index
45	f	17+ years	0.142	732.896	84	Grand Index
45	f	17+ years	0.143	733	84	Grand Index

Age	Gender	Education	Percentile	Raw Score	Norm Score	Assessment
45	f	17+ years	0.144	733.2717308	84	Grand Index
45	f	17+ years	0.145	733.689014	84	Grand Index
45	f	17+ years	0.148	734	84	Grand Index
45	f	17+ years	0.149	734.412	84	Grand Index
45	f	17+ years	0.151	735	85	Grand Index
45	f	17+ years	0.152	735.776	85	Grand Index
45	f	17+ years	0.153	736	85	Grand Index
45	f	17+ years	0.154	736.2831489	85	Grand Index
45	f	17+ years	0.157	737	85	Grand Index
45	f	17+ years	0.158	737.504	85	Grand Index
45	f	17+ years	0.16	738	85	Grand Index
45	f	17+ years	0.161	738.868	85	Grand Index
45	f	17+ years	0.163	739	85	Grand Index
45	f	17+ years	0.164	739.7274573	85	Grand Index
45	f	17+ years	0.165	740.02	85	Grand Index
45	f	17+ years	0.167	741	86	Grand Index
45	f	17+ years	0.168	741.0046996	86	Grand Index
45	f	17+ years	0.172	742	86	Grand Index
45	f	17+ years	0.173	742.6877243	86	Grand Index
45	f	17+ years	0.177	743	86	Grand Index
45	f	17+ years	0.178	743.2502913	86	Grand Index
45	f	17+ years	0.182	744	86	Grand Index
45	f	17+ years	0.183	744.204	86	Grand Index
45	f	17+ years	0.187	745	87	Grand Index
45	f	17+ years	0.188	746.0934928	87	Grand Index
45	f	17+ years	0.189	746.9761494	87	Grand Index
45	f	17+ years	0.191	747	87	Grand Index
45	f	17+ years	0.192	747.296	87	Grand Index
45	f	17+ years	0.194	748	87	Grand Index
45	f	17+ years	0.195	748.0631727	87	Grand Index
45	f	17+ years	0.196	748.2384521	87	Grand Index
45	f	17+ years	0.199	749	87	Grand Index
45	f	17+ years	0.2	749.5607203	87	Grand Index
45	f	17+ years	0.202	750	87	Grand Index
45	f	17+ years	0.203	750.0440703	88	Grand Index
45	f	17+ years	0.204	750.7916697	88	Grand Index
45	f	17+ years	0.205	751	88	Grand Index
45	f	17+ years	0.206	751.328	88	Grand Index
45	f	17+ years	0.209	752	88	Grand Index
45	f	17+ years	0.212	753	88	Grand Index
45	f	17+ years	0.213	753.7601534	88	Grand Index
45	f	17+ years	0.218	754	88	Grand Index
45	f	17+ years	0.219	754.1000936	88	Grand Index
45	f	17+ years	0.225	755	89	Grand Index
45	f	17+ years	0.228	756	89	Grand Index
45	f	17+ years	0.229	756.0553018	89	Grand Index
45	f	17+ years	0.231	757	89	Grand Index
45	f	17+ years	0.232	757.816	89	Grand Index
45	f	17+ years	0.233	758	89	Grand Index
45	f	17+ years	0.234	758.1802082	89	Grand Index
45	f	17+ years	0.235	758.6923929	89	Grand Index
45	f	17+ years	0.238	759	89	Grand Index
45	f	17+ years	0.239	759.332	89	Grand Index
45	f	17+ years	0.242	760	90	Grand Index
45	f	17+ years	0.243	760.5913665	90	Grand Index
45	f	17+ years	0.244	761	90	Grand Index

Age	Gender	Education	Percentile	Raw Score	Norm Score	Assessment
45	f	17+ years	0.245	761.06	90	Grand Index
45	f	17+ years	0.249	762	90	Grand Index
45	f	17+ years	0.253	763	90	Grand Index
45	f	17+ years	0.254	763.6288093	90	Grand Index
45	f	17+ years	0.258	764	90	Grand Index
45	f	17+ years	0.259	764.2381576	90	Grand Index
45	f	17+ years	0.26	764.6305115	90	Grand Index
45	f	17+ years	0.261	764.9189952	90	Grand Index
45	f	17+ years	0.265	765	91	Grand Index
45	f	17+ years	0.268	766	91	Grand Index
45	f	17+ years	0.269	766.3833638	91	Grand Index
45	f	17+ years	0.271	767	91	Grand Index
45	f	17+ years	0.272	767.0212951	91	Grand Index
45	f	17+ years	0.276	768	91	Grand Index
45	f	17+ years	0.277	768.8910202	91	Grand Index
45	f	17+ years	0.281	769	91	Grand Index
45	f	17+ years	0.282	769.2351031	91	Grand Index
45	f	17+ years	0.285	770	91	Grand Index
45	f	17+ years	0.289	771	92	Grand Index
45	f	17+ years	0.29	771.339974	92	Grand Index
45	f	17+ years	0.293	772	92	Grand Index
45	f	17+ years	0.294	772.2843969	92	Grand Index
45	f	17+ years	0.295	773	92	Grand Index
45	f	17+ years	0.297	774	92	Grand Index
45	f	17+ years	0.298	774.7524959	92	Grand Index
45	f	17+ years	0.299	774.9801773	92	Grand Index
45	f	17+ years	0.304	775	92	Grand Index
45	f	17+ years	0.306	776	92	Grand Index
45	f	17+ years	0.307	776.3380359	92	Grand Index
45	f	17+ years	0.314	777	93	Grand Index
45	f	17+ years	0.315	777.1223514	93	Grand Index
45	f	17+ years	0.318	778	93	Grand Index
45	f	17+ years	0.319	778.372	93	Grand Index
45	f	17+ years	0.323	779	93	Grand Index
45	f	17+ years	0.324	779.2212364	93	Grand Index
45	f	17+ years	0.325	779.7923489	93	Grand Index
45	f	17+ years	0.329	780	93	Grand Index
45	f	17+ years	0.33	780.04	93	Grand Index
45	f	17+ years	0.335	781	94	Grand Index
45	f	17+ years	0.336	781.768	94	Grand Index
45	f	17+ years	0.34	782	94	Grand Index
45	f	17+ years	0.341	782.7957956	94	Grand Index
45	f	17+ years	0.343	783	94	Grand Index
45	f	17+ years	0.344	783.0552112	94	Grand Index
45	f	17+ years	0.345	783.967355	94	Grand Index
45	f	17+ years	0.348	784	94	Grand Index
45	f	17+ years	0.349	784.0050981	94	Grand Index
45	f	17+ years	0.35	784.8849685	94	Grand Index
45	f	17+ years	0.355	785	94	Grand Index
45	f	17+ years	0.356	785.528	94	Grand Index
45	f	17+ years	0.357	786	95	Grand Index
45	f	17+ years	0.358	786.104	95	Grand Index
45	f	17+ years	0.364	787	95	Grand Index
45	f	17+ years	0.365	787.62	95	Grand Index
45	f	17+ years	0.371	788	95	Grand Index
45	f	17+ years	0.376	789	95	Grand Index

Age	Gender	Education	Percentile	Raw Score	Norm Score	Assessment
45	f	17+ years	0.377	789.9916757	95	Grand Index
45	f	17+ years	0.382	790	95	Grand Index
45	f	17+ years	0.383	790.804	96	Grand Index
45	f	17+ years	0.386	791	96	Grand Index
45	f	17+ years	0.387	791.4038262	96	Grand Index
45	f	17+ years	0.392	792	96	Grand Index
45	f	17+ years	0.393	792.4327121	96	Grand Index
45	f	17+ years	0.401	793	96	Grand Index
45	f	17+ years	0.402	793.8451464	96	Grand Index
45	f	17+ years	0.404	794	96	Grand Index
45	f	17+ years	0.405	794.1194748	96	Grand Index
45	f	17+ years	0.406	794.9502618	96	Grand Index
45	f	17+ years	0.411	795	97	Grand Index
45	f	17+ years	0.412	795.3781416	97	Grand Index
45	f	17+ years	0.414	796	97	Grand Index
45	f	17+ years	0.415	796.9881502	97	Grand Index
45	f	17+ years	0.417	797	97	Grand Index
45	f	17+ years	0.418	797.384	97	Grand Index
45	f	17+ years	0.421	798	97	Grand Index
45	f	17+ years	0.422	798.536	97	Grand Index
45	f	17+ years	0.426	799	97	Grand Index
45	f	17+ years	0.427	799.9304024	97	Grand Index
45	f	17+ years	0.434	800	98	Grand Index
45	f	17+ years	0.435	800.8118229	98	Grand Index
45	f	17+ years	0.438	801	98	Grand Index
45	f	17+ years	0.439	801.3575399	98	Grand Index
45	f	17+ years	0.44	801.8755871	98	Grand Index
45	f	17+ years	0.443	802	98	Grand Index
45	f	17+ years	0.444	802.872	98	Grand Index
45	f	17+ years	0.447	803	98	Grand Index
45	f	17+ years	0.448	803.0024156	98	Grand Index
45	f	17+ years	0.449	803.6866448	98	Grand Index
45	f	17+ years	0.451	804	98	Grand Index
45	f	17+ years	0.458	805	98	Grand Index
45	f	17+ years	0.459	805.7018435	98	Grand Index
45	f	17+ years	0.464	806	99	Grand Index
45	f	17+ years	0.465	806.0830641	99	Grand Index
45	f	17+ years	0.466	806.4047161	99	Grand Index
45	f	17+ years	0.469	807	99	Grand Index
45	f	17+ years	0.47	807.0186609	99	Grand Index
45	f	17+ years	0.477	808	99	Grand Index
45	f	17+ years	0.478	808.0201154	99	Grand Index
45	f	17+ years	0.479	808.9107064	99	Grand Index
45	f	17+ years	0.482	809	99	Grand Index
45	f	17+ years	0.483	809.4849105	99	Grand Index
45	f	17+ years	0.488	810	100	Grand Index
45	f	17+ years	0.489	810.0706686	100	Grand Index
45	f	17+ years	0.493	811	100	Grand Index
45	f	17+ years	0.494	811.1553716	100	Grand Index
45	f	17+ years	0.498	812	100	Grand Index
45	f	17+ years	0.499	812.9560062	100	Grand Index
45	f	17+ years	0.502	813	100	Grand Index
45	f	17+ years	0.503	813.5048808	100	Grand Index
45	f	17+ years	0.508	814	100	Grand Index
45	f	17+ years	0.509	814.4076456	100	Grand Index
45	f	17+ years	0.51	814.8125874	100	Grand Index

Age	Gender	Education	Percentile	Raw Score	Norm Score	Assessment
45	f	17+ years	0.511	814.9389046	100	Grand Index
45	f	17+ years	0.515	815	101	Grand Index
45	f	17+ years	0.516	815.2526724	101	Grand Index
45	f	17+ years	0.52	816	101	Grand Index
45	f	17+ years	0.521	816.548	101	Grand Index
45	f	17+ years	0.526	817	101	Grand Index
45	f	17+ years	0.527	817.2242333	101	Grand Index
45	f	17+ years	0.53	818	101	Grand Index
45	f	17+ years	0.531	818.083704	101	Grand Index
45	f	17+ years	0.532	818.7505649	101	Grand Index
45	f	17+ years	0.536	819	101	Grand Index
45	f	17+ years	0.537	819.138814	101	Grand Index
45	f	17+ years	0.538	819.9525562	101	Grand Index
45	f	17+ years	0.544	820	102	Grand Index
45	f	17+ years	0.545	820.0762194	102	Grand Index
45	f	17+ years	0.546	821	102	Grand Index
45	f	17+ years	0.547	821.8563647	102	Grand Index
45	f	17+ years	0.55	822	102	Grand Index
45	f	17+ years	0.551	822.188	102	Grand Index
45	f	17+ years	0.554	823	102	Grand Index
45	f	17+ years	0.555	823.320594	102	Grand Index
45	f	17+ years	0.559	824	102	Grand Index
45	f	17+ years	0.56	824.28	102	Grand Index
45	f	17+ years	0.564	825	102	Grand Index
45	f	17+ years	0.565	825.0006733	102	Grand Index
45	f	17+ years	0.566	825.8545646	102	Grand Index
45	f	17+ years	0.571	826	103	Grand Index
45	f	17+ years	0.572	826.1585856	103	Grand Index
45	f	17+ years	0.573	826.916468	103	Grand Index
45	f	17+ years	0.575	827	103	Grand Index
45	f	17+ years	0.576	827.498454	103	Grand Index
45	f	17+ years	0.577	827.9999009	103	Grand Index
45	f	17+ years	0.58	828	103	Grand Index
45	f	17+ years	0.581	828.5344364	103	Grand Index
45	f	17+ years	0.582	828.9659991	103	Grand Index
45	f	17+ years	0.588	829	103	Grand Index
45	f	17+ years	0.589	829.132	103	Grand Index
45	f	17+ years	0.594	830	104	Grand Index
45	f	17+ years	0.595	830.4009226	104	Grand Index
45	f	17+ years	0.596	830.8998539	104	Grand Index
45	f	17+ years	0.598	831	104	Grand Index
45	f	17+ years	0.599	831.0098815	104	Grand Index
45	f	17+ years	0.6	831.9646915	104	Grand Index
45	f	17+ years	0.604	832	104	Grand Index
45	f	17+ years	0.608	833	104	Grand Index
45	f	17+ years	0.609	833.2551916	104	Grand Index
45	f	17+ years	0.61	834	104	Grand Index
45	f	17+ years	0.614	835	104	Grand Index
45	f	17+ years	0.615	835.2484625	104	Grand Index
45	f	17+ years	0.616	835.9626482	104	Grand Index
45	f	17+ years	0.62	836	105	Grand Index
45	f	17+ years	0.621	836.0058446	105	Grand Index
45	f	17+ years	0.622	836.6255391	105	Grand Index
45	f	17+ years	0.624	837	105	Grand Index
45	f	17+ years	0.628	838	105	Grand Index
45	f	17+ years	0.633	839	105	Grand Index

Age	Gender	Education	Percentile	Raw Score	Norm Score	Assessment
45	f	17+ years	0.634	839.0080444	105	Grand Index
45	f	17+ years	0.635	839.3906362	105	Grand Index
45	f	17+ years	0.64	840	105	Grand Index
45	f	17+ years	0.641	840.108	105	Grand Index
45	f	17+ years	0.644	841	106	Grand Index
45	f	17+ years	0.645	841.0438483	106	Grand Index
45	f	17+ years	0.648	842	106	Grand Index
45	f	17+ years	0.649	842.0491857	106	Grand Index
45	f	17+ years	0.654	843	106	Grand Index
45	f	17+ years	0.655	843.4069545	106	Grand Index
45	f	17+ years	0.657	844	106	Grand Index
45	f	17+ years	0.658	844.5005131	106	Grand Index
45	f	17+ years	0.662	845	106	Grand Index
45	f	17+ years	0.663	846	106	Grand Index
45	f	17+ years	0.664	846.232	106	Grand Index
45	f	17+ years	0.665	847	106	Grand Index
45	f	17+ years	0.666	847.4817888	106	Grand Index
45	f	17+ years	0.667	847.9059541	106	Grand Index
45	f	17+ years	0.671	848	107	Grand Index
45	f	17+ years	0.672	848.536	107	Grand Index
45	f	17+ years	0.675	849	107	Grand Index
45	f	17+ years	0.676	849.5277516	107	Grand Index
45	f	17+ years	0.68	850	107	Grand Index
45	f	17+ years	0.681	850.4493711	107	Grand Index
45	f	17+ years	0.682	850.8925607	107	Grand Index
45	f	17+ years	0.686	851	107	Grand Index
45	f	17+ years	0.687	851.356	107	Grand Index
45	f	17+ years	0.689	852	107	Grand Index
45	f	17+ years	0.69	852.0677785	107	Grand Index
45	f	17+ years	0.691	852.1374694	107	Grand Index
45	f	17+ years	0.697	853	108	Grand Index
45	f	17+ years	0.698	853.0043601	108	Grand Index
45	f	17+ years	0.699	853.8461538	108	Grand Index
45	f	17+ years	0.704	854	108	Grand Index
45	f	17+ years	0.705	854.8922286	108	Grand Index
45	f	17+ years	0.709	855	108	Grand Index
45	f	17+ years	0.71	855.018773	108	Grand Index
45	f	17+ years	0.714	856	108	Grand Index
45	f	17+ years	0.715	856.7728537	109	Grand Index
45	f	17+ years	0.719	857	109	Grand Index
45	f	17+ years	0.724	858	109	Grand Index
45	f	17+ years	0.725	858.0074942	109	Grand Index
45	f	17+ years	0.726	858.3343706	109	Grand Index
45	f	17+ years	0.73	859	109	Grand Index
45	f	17+ years	0.731	859.028	109	Grand Index
45	f	17+ years	0.736	860	109	Grand Index
45	f	17+ years	0.737	860.756	110	Grand Index
45	f	17+ years	0.74	861	110	Grand Index
45	f	17+ years	0.741	861.7967089	110	Grand Index
45	f	17+ years	0.743	862	110	Grand Index
45	f	17+ years	0.744	862.2703394	110	Grand Index
45	f	17+ years	0.75	863	110	Grand Index
45	f	17+ years	0.752	864	110	Grand Index
45	f	17+ years	0.753	864.364	110	Grand Index
45	f	17+ years	0.757	865	110	Grand Index
45	f	17+ years	0.758	865.1704761	110	Grand Index

Age	Gender	Education	Percentile	Raw Score	Norm Score	Assessment
45	f	17+ years	0.763	866	111	Grand Index
45	f	17+ years	0.764	866.0426724	111	Grand Index
45	f	17+ years	0.767	867	111	Grand Index
45	f	17+ years	0.768	867.0269497	111	Grand Index
45	f	17+ years	0.769	867.3946649	111	Grand Index
45	f	17+ years	0.772	868	111	Grand Index
45	f	17+ years	0.773	868.124	111	Grand Index
45	f	17+ years	0.774	869	111	Grand Index
45	f	17+ years	0.775	869.7	111	Grand Index
45	f	17+ years	0.779	870	112	Grand Index
45	f	17+ years	0.78	870.8943432	112	Grand Index
45	f	17+ years	0.784	871	112	Grand Index
45	f	17+ years	0.785	871.4163978	112	Grand Index
45	f	17+ years	0.788	872	112	Grand Index
45	f	17+ years	0.79	873	112	Grand Index
45	f	17+ years	0.791	873.5160481	112	Grand Index
45	f	17+ years	0.792	874.0112719	112	Grand Index
45	f	17+ years	0.793	874.8276107	112	Grand Index
45	f	17+ years	0.797	875	112	Grand Index
45	f	17+ years	0.798	875.824	113	Grand Index
45	f	17+ years	0.801	876	113	Grand Index
45	f	17+ years	0.802	876.976	113	Grand Index
45	f	17+ years	0.805	877	113	Grand Index
45	f	17+ years	0.806	877.0591681	113	Grand Index
45	f	17+ years	0.807	877.9548291	113	Grand Index
45	f	17+ years	0.811	878	113	Grand Index
45	f	17+ years	0.812	878.856	113	Grand Index
45	f	17+ years	0.814	879	113	Grand Index
45	f	17+ years	0.815	879.1578245	113	Grand Index
45	f	17+ years	0.818	880	114	Grand Index
45	f	17+ years	0.819	880.372	114	Grand Index
45	f	17+ years	0.822	881	114	Grand Index
45	f	17+ years	0.823	881.524	114	Grand Index
45	f	17+ years	0.827	882	114	Grand Index
45	f	17+ years	0.829	883	114	Grand Index
45	f	17+ years	0.83	883.0024306	114	Grand Index
45	f	17+ years	0.831	883.8384518	114	Grand Index
45	f	17+ years	0.832	885.6009959	114	Grand Index
45	f	17+ years	0.833	886.404	114	Grand Index
45	f	17+ years	0.837	887	115	Grand Index
45	f	17+ years	0.838	887.365738	115	Grand Index
45	f	17+ years	0.84	888	115	Grand Index
45	f	17+ years	0.841	888.708	115	Grand Index
45	f	17+ years	0.844	889	115	Grand Index
45	f	17+ years	0.845	889.86	115	Grand Index
45	f	17+ years	0.846	890.7609552	115	Grand Index
45	f	17+ years	0.847	891.4202615	115	Grand Index
45	f	17+ years	0.848	892	115	Grand Index
45	f	17+ years	0.849	892.0408414	115	Grand Index
45	f	17+ years	0.85	892.6402533	116	Grand Index
45	f	17+ years	0.851	892.9447474	116	Grand Index
45	f	17+ years	0.852	893	116	Grand Index
45	f	17+ years	0.853	893.0596712	116	Grand Index
45	f	17+ years	0.854	893.9694647	116	Grand Index
45	f	17+ years	0.857	894	116	Grand Index
45	f	17+ years	0.858	894.0457785	116	Grand Index

Age	Gender	Education	Percentile	Raw Score	Norm Score	Assessment
45	f	17+ years	0.859	894.7826293	116	Grand Index
45	f	17+ years	0.862	895	116	Grand Index
45	f	17+ years	0.864	896	116	Grand Index
45	f	17+ years	0.865	896.62	117	Grand Index
45	f	17+ years	0.866	897	117	Grand Index
45	f	17+ years	0.867	897.0230158	117	Grand Index
45	f	17+ years	0.868	897.1889202	117	Grand Index
45	f	17+ years	0.87	898	117	Grand Index
45	f	17+ years	0.871	899	117	Grand Index
45	f	17+ years	0.872	899.136	117	Grand Index
45	f	17+ years	0.874	900	117	Grand Index
45	f	17+ years	0.875	900.5843979	117	Grand Index
45	f	17+ years	0.879	901	118	Grand Index
45	f	17+ years	0.88	901.44	118	Grand Index
45	f	17+ years	0.881	902	118	Grand Index
45	f	17+ years	0.882	902.0113025	118	Grand Index
45	f	17+ years	0.883	902.7757576	118	Grand Index
45	f	17+ years	0.885	903	118	Grand Index
45	f	17+ years	0.886	903.168	118	Grand Index
45	f	17+ years	0.887	904	118	Grand Index
45	f	17+ years	0.888	904.9848962	118	Grand Index
45	f	17+ years	0.889	905	118	Grand Index
45	f	17+ years	0.89	906.02548	118	Grand Index
45	f	17+ years	0.891	906.6891278	118	Grand Index
45	f	17+ years	0.892	907.896	119	Grand Index
45	f	17+ years	0.893	908.6638692	119	Grand Index
45	f	17+ years	0.895	909	119	Grand Index
45	f	17+ years	0.896	909.4763904	119	Grand Index
45	f	17+ years	0.897	909.9981221	119	Grand Index
45	f	17+ years	0.898	910	119	Grand Index
45	f	17+ years	0.899	910.83536	119	Grand Index
45	f	17+ years	0.9	911	119	Grand Index
45	f	17+ years	0.901	911.988	119	Grand Index
45	f	17+ years	0.902	912.776	119	Grand Index
45	f	17+ years	0.904	913	120	Grand Index
45	f	17+ years	0.905	913.14	120	Grand Index
45	f	17+ years	0.906	914	120	Grand Index
45	f	17+ years	0.907	914.941183	120	Grand Index
45	f	17+ years	0.908	915.504	120	Grand Index
45	f	17+ years	0.909	917.0175931	120	Grand Index
45	f	17+ years	0.91	917.239818	120	Grand Index
45	f	17+ years	0.911	917.9520402	120	Grand Index
45	f	17+ years	0.912	919	120	Grand Index
45	f	17+ years	0.913	920	120	Grand Index
45	f	17+ years	0.914	920.0322444	120	Grand Index
45	f	17+ years	0.917	921	121	Grand Index
45	f	17+ years	0.918	921.0300073	121	Grand Index
45	f	17+ years	0.92	922	121	Grand Index
45	f	17+ years	0.921	922.0003821	121	Grand Index
45	f	17+ years	0.922	922.6168971	121	Grand Index
45	f	17+ years	0.923	923.324	121	Grand Index
45	f	17+ years	0.924	924	121	Grand Index
45	f	17+ years	0.925	924.9	122	Grand Index
45	f	17+ years	0.926	925	122	Grand Index
45	f	17+ years	0.927	925.9775788	122	Grand Index
45	f	17+ years	0.928	927.264	122	Grand Index

Age	Gender	Education	Percentile	Raw Score	Norm Score	Assessment
45	f	17+ years	0.929	928.052	122	Grand Index
45	f	17+ years	0.93	929	122	Grand Index
45	f	17+ years	0.931	929.0550259	122	Grand Index
45	f	17+ years	0.932	929.9680324	122	Grand Index
45	f	17+ years	0.933	930.204	122	Grand Index
45	f	17+ years	0.934	931	123	Grand Index
45	f	17+ years	0.935	931.1259297	123	Grand Index
45	f	17+ years	0.936	931.9998476	123	Grand Index
45	f	17+ years	0.937	932	123	Grand Index
45	f	17+ years	0.938	932.0725287	123	Grand Index
45	f	17+ years	0.939	932.8243208	123	Grand Index
45	f	17+ years	0.94	933.6852452	123	Grand Index
45	f	17+ years	0.941	934.4667824	123	Grand Index
45	f	17+ years	0.942	935	124	Grand Index
45	f	17+ years	0.943	935.8100497	124	Grand Index
45	f	17+ years	0.944	936.872	124	Grand Index
45	f	17+ years	0.946	937	124	Grand Index
45	f	17+ years	0.947	937.3020087	124	Grand Index
45	f	17+ years	0.948	938.2169016	124	Grand Index
45	f	17+ years	0.949	939	125	Grand Index
45	f	17+ years	0.95	939.9116504	125	Grand Index
45	f	17+ years	0.951	940	125	Grand Index
45	f	17+ years	0.952	941	125	Grand Index
45	f	17+ years	0.953	941.0241072	125	Grand Index
45	f	17+ years	0.954	943.7233028	125	Grand Index
45	f	17+ years	0.956	944	126	Grand Index
45	f	17+ years	0.957	944.203379	126	Grand Index
45	f	17+ years	0.958	945.960887	126	Grand Index
45	f	17+ years	0.959	947.692	126	Grand Index
45	f	17+ years	0.96	948.231619	126	Grand Index
45	f	17+ years	0.961	948.9891998	126	Grand Index
45	f	17+ years	0.962	950.2945622	127	Grand Index
45	f	17+ years	0.963	951.2086246	127	Grand Index
45	f	17+ years	0.964	952.672134	127	Grand Index
45	f	17+ years	0.965	953	127	Grand Index
45	f	17+ years	0.966	953.208	127	Grand Index
45	f	17+ years	0.967	954.3225143	128	Grand Index
45	f	17+ years	0.968	955	128	Grand Index
45	f	17+ years	0.969	955.9131481	128	Grand Index
45	f	17+ years	0.97	956.3509107	128	Grand Index
45	f	17+ years	0.971	957	128	Grand Index
45	f	17+ years	0.972	957.936	129	Grand Index
45	f	17+ years	0.973	960.1869871	129	Grand Index
45	f	17+ years	0.974	962	129	Grand Index
45	f	17+ years	0.975	963.2981711	129	Grand Index
45	f	17+ years	0.976	964.5409396	130	Grand Index
45	f	17+ years	0.977	967.628	130	Grand Index
45	f	17+ years	0.978	971.6929171	130	Grand Index
45	f	17+ years	0.979	973.669883	131	Grand Index
45	f	17+ years	0.98	974.24	131	Grand Index
45	f	17+ years	0.981	976.1024059	131	Grand Index
45	f	17+ years	0.982	978	131	Grand Index
45	f	17+ years	0.983	979.9737375	132	Grand Index
45	f	17+ years	0.984	980.919625	132	Grand Index
45	f	17+ years	0.985	985	133	Grand Index
45	f	17+ years	0.986	985.968	133	Grand Index

Age	Gender	Education	Percentile	Raw Score	Norm Score	Assessment
45	f	17+ years	0.987	987.756	133	Grand Index
45	f	17+ years	0.988	990.5178781	134	Grand Index
45	f	17+ years	0.989	992.4219475	134	Grand Index
45	f	17+ years	0.99	993.2295993	135	Grand Index
45	f	17+ years	0.991	994.6143346	135	Grand Index
45	f	17+ years	0.992	1001.614281	136	Grand Index
45	f	17+ years	0.993	1003.647479	137	Grand Index
45	f	17+ years	0.994	1009.317732	138	Grand Index
45	f	17+ years	0.995	1014.106245	139	Grand Index
45	f	17+ years	0.996	1018.731151	140	Grand Index
45	f	17+ years	0.997	1024.272	141	Grand Index
45	f	17+ years	0.998	1042.620357	143	Grand Index
45	f	17+ years	0.999	1050.640988	146	Grand Index
45	m	17+ years	0.001	582.2403573	54	Grand Index
45	m	17+ years	0.002	594.6044496	57	Grand Index
45	m	17+ years	0.003	599.1339975	59	Grand Index
45	m	17+ years	0.004	603.892	60	Grand Index
45	m	17+ years	0.005	616.0514428	61	Grand Index
45	m	17+ years	0.006	623.6840627	62	Grand Index
45	m	17+ years	0.007	630.0580594	63	Grand Index
45	m	17+ years	0.008	632.8108031	64	Grand Index
45	m	17+ years	0.009	636	65	Grand Index
45	m	17+ years	0.01	642.51	65	Grand Index
45	m	17+ years	0.011	643.1326907	66	Grand Index
45	m	17+ years	0.012	644.7233526	66	Grand Index
45	m	17+ years	0.013	646.7673058	67	Grand Index
45	m	17+ years	0.014	648.2000737	67	Grand Index
45	m	17+ years	0.015	650.2800339	67	Grand Index
45	m	17+ years	0.016	651.615586	68	Grand Index
45	m	17+ years	0.017	654.7591023	68	Grand Index
45	m	17+ years	0.018	655.4386785	69	Grand Index
45	m	17+ years	0.019	656.5095489	69	Grand Index
45	m	17+ years	0.02	657.8527186	69	Grand Index
45	m	17+ years	0.021	659.306	69	Grand Index
45	m	17+ years	0.022	661	70	Grand Index
45	m	17+ years	0.023	661.739	70	Grand Index
45	m	17+ years	0.024	663.0063606	70	Grand Index
45	m	17+ years	0.025	664.7591684	71	Grand Index
45	m	17+ years	0.026	668	71	Grand Index
45	m	17+ years	0.027	668.0072802	71	Grand Index
45	m	17+ years	0.028	668.4565472	71	Grand Index
45	m	17+ years	0.029	668.9002584	72	Grand Index
45	m	17+ years	0.03	669.79	72	Grand Index
45	m	17+ years	0.031	670.902133	72	Grand Index
45	m	17+ years	0.032	672	72	Grand Index
45	m	17+ years	0.033	672.078815	72	Grand Index
45	m	17+ years	0.034	672.9664768	73	Grand Index
45	m	17+ years	0.035	673.255	73	Grand Index
45	m	17+ years	0.036	674	73	Grand Index
45	m	17+ years	0.037	676.523	73	Grand Index
45	m	17+ years	0.038	677.1708425	73	Grand Index
45	m	17+ years	0.039	677.9843551	74	Grand Index
45	m	17+ years	0.04	678	74	Grand Index
45	m	17+ years	0.041	680.013	74	Grand Index
45	m	17+ years	0.042	681	74	Grand Index
45	m	17+ years	0.043	682.4769005	74	Grand Index

Age	Gender	Education	Percentile	Raw Score	Norm Score	Assessment
45	m	17+ years	0.044	683.9422859	74	Grand Index
45	m	17+ years	0.045	685.185	75	Grand Index
45	m	17+ years	0.046	686	75	Grand Index
45	m	17+ years	0.047	686.771	75	Grand Index
45	m	17+ years	0.048	689.192	75	Grand Index
45	m	17+ years	0.049	692	75	Grand Index
45	m	17+ years	0.05	692.3930648	75	Grand Index
45	m	17+ years	0.051	692.7160449	75	Grand Index
45	m	17+ years	0.052	693	76	Grand Index
45	m	17+ years	0.053	693.1673772	76	Grand Index
45	m	17+ years	0.054	693.60188	76	Grand Index
45	m	17+ years	0.055	694.115	76	Grand Index
45	m	17+ years	0.056	696.224	76	Grand Index
45	m	17+ years	0.057	698	76	Grand Index
45	m	17+ years	0.058	698.994	76	Grand Index
45	m	17+ years	0.059	699	77	Grand Index
45	m	17+ years	0.06	699.58	77	Grand Index
45	m	17+ years	0.061	700	77	Grand Index
45	m	17+ years	0.062	701.7832434	77	Grand Index
45	m	17+ years	0.063	702.459	77	Grand Index
45	m	17+ years	0.064	703.752	77	Grand Index
45	m	17+ years	0.065	704	77	Grand Index
45	m	17+ years	0.066	704.3318746	77	Grand Index
45	m	17+ years	0.067	704.9933128	78	Grand Index
45	m	17+ years	0.068	705.7282764	78	Grand Index
45	m	17+ years	0.07	706	78	Grand Index
45	m	17+ years	0.071	706.803	78	Grand Index
45	m	17+ years	0.072	708	78	Grand Index
45	m	17+ years	0.073	708.389	78	Grand Index
45	m	17+ years	0.074	709	78	Grand Index
45	m	17+ years	0.075	709.7183099	78	Grand Index
45	m	17+ years	0.077	710	79	Grand Index
45	m	17+ years	0.078	710.3209992	79	Grand Index
45	m	17+ years	0.079	711	79	Grand Index
45	m	17+ years	0.08	711.1302735	79	Grand Index
45	m	17+ years	0.081	711.8120523	79	Grand Index
45	m	17+ years	0.082	712.000513	79	Grand Index
45	m	17+ years	0.083	712.0689826	79	Grand Index
45	m	17+ years	0.084	712.6638682	79	Grand Index
45	m	17+ years	0.085	712.9975639	79	Grand Index
45	m	17+ years	0.086	713.198	80	Grand Index
45	m	17+ years	0.088	714	80	Grand Index
45	m	17+ years	0.089	715	80	Grand Index
45	m	17+ years	0.09	715.37	80	Grand Index
45	m	17+ years	0.091	716.0328756	80	Grand Index
45	m	17+ years	0.092	716.130857	80	Grand Index
45	m	17+ years	0.093	716.5909732	80	Grand Index
45	m	17+ years	0.094	716.941135	80	Grand Index
45	m	17+ years	0.097	717	81	Grand Index
45	m	17+ years	0.098	717.2473972	81	Grand Index
45	m	17+ years	0.102	718	81	Grand Index
45	m	17+ years	0.103	718.0185264	81	Grand Index
45	m	17+ years	0.104	718.5266476	81	Grand Index
45	m	17+ years	0.105	719	81	Grand Index
45	m	17+ years	0.106	719.4680688	81	Grand Index
45	m	17+ years	0.108	720	81	Grand Index

Age	Gender	Education	Percentile	Raw Score	Norm Score	Assessment
45	m	17+ years	0.109	720.937	82	Grand Index
45	m	17+ years	0.11	721	82	Grand Index
45	m	17+ years	0.111	721.1579562	82	Grand Index
45	m	17+ years	0.112	721.8715716	82	Grand Index
45	m	17+ years	0.113	722.109	82	Grand Index
45	m	17+ years	0.116	723	82	Grand Index
45	m	17+ years	0.117	723.8991374	82	Grand Index
45	m	17+ years	0.122	724	83	Grand Index
45	m	17+ years	0.124	725	83	Grand Index
45	m	17+ years	0.125	725.625	83	Grand Index
45	m	17+ years	0.126	726	83	Grand Index
45	m	17+ years	0.127	726.0520267	83	Grand Index
45	m	17+ years	0.128	726.6262997	83	Grand Index
45	m	17+ years	0.131	727	83	Grand Index
45	m	17+ years	0.132	727.676	83	Grand Index
45	m	17+ years	0.136	728	84	Grand Index
45	m	17+ years	0.137	728.7135364	84	Grand Index
45	m	17+ years	0.139	729	84	Grand Index
45	m	17+ years	0.14	729.02	84	Grand Index
45	m	17+ years	0.142	730	84	Grand Index
45	m	17+ years	0.143	730.4248907	84	Grand Index
45	m	17+ years	0.144	730.7026212	84	Grand Index
45	m	17+ years	0.146	731	84	Grand Index
45	m	17+ years	0.147	731.001883	84	Grand Index
45	m	17+ years	0.148	731.2468996	84	Grand Index
45	m	17+ years	0.149	731.8737612	84	Grand Index
45	m	17+ years	0.151	732	85	Grand Index
45	m	17+ years	0.152	732.0170927	85	Grand Index
45	m	17+ years	0.153	732.8344531	85	Grand Index
45	m	17+ years	0.154	733.122	85	Grand Index
45	m	17+ years	0.158	734	85	Grand Index
45	m	17+ years	0.159	735.174	85	Grand Index
45	m	17+ years	0.163	736	85	Grand Index
45	m	17+ years	0.164	736.0606584	85	Grand Index
45	m	17+ years	0.165	737.0067741	85	Grand Index
45	m	17+ years	0.166	737.0234629	85	Grand Index
45	m	17+ years	0.167	737.9327688	86	Grand Index
45	m	17+ years	0.168	738.1775434	86	Grand Index
45	m	17+ years	0.169	738.8350885	86	Grand Index
45	m	17+ years	0.17	738.9762079	86	Grand Index
45	m	17+ years	0.172	739	86	Grand Index
45	m	17+ years	0.173	739.689	86	Grand Index
45	m	17+ years	0.175	740	86	Grand Index
45	m	17+ years	0.176	740.568	86	Grand Index
45	m	17+ years	0.177	741	86	Grand Index
45	m	17+ years	0.178	742	86	Grand Index
45	m	17+ years	0.179	742.447	86	Grand Index
45	m	17+ years	0.18	743	86	Grand Index
45	m	17+ years	0.183	744	86	Grand Index
45	m	17+ years	0.184	744.5537176	86	Grand Index
45	m	17+ years	0.185	744.758564	87	Grand Index
45	m	17+ years	0.186	745	87	Grand Index
45	m	17+ years	0.187	745.791	87	Grand Index
45	m	17+ years	0.188	747.084	87	Grand Index
45	m	17+ years	0.191	748	87	Grand Index
45	m	17+ years	0.192	748.9767211	87	Grand Index

Age	Gender	Education	Percentile	Raw Score	Norm Score	Assessment
45	m	17+ years	0.194	749	87	Grand Index
45	m	17+ years	0.195	749.5373208	87	Grand Index
45	m	17+ years	0.197	750	87	Grand Index
45	m	17+ years	0.203	751	88	Grand Index
45	m	17+ years	0.204	751.5153183	88	Grand Index
45	m	17+ years	0.205	752	88	Grand Index
45	m	17+ years	0.206	752.358	88	Grand Index
45	m	17+ years	0.208	753	88	Grand Index
45	m	17+ years	0.209	753.2913725	88	Grand Index
45	m	17+ years	0.21	753.8415343	88	Grand Index
45	m	17+ years	0.212	754	88	Grand Index
45	m	17+ years	0.213	754.3242084	88	Grand Index
45	m	17+ years	0.214	754.9382203	88	Grand Index
45	m	17+ years	0.215	755	88	Grand Index
45	m	17+ years	0.216	755.000071	88	Grand Index
45	m	17+ years	0.217	755.0824431	88	Grand Index
45	m	17+ years	0.218	755.8918568	88	Grand Index
45	m	17+ years	0.221	756	88	Grand Index
45	m	17+ years	0.222	756.046	89	Grand Index
45	m	17+ years	0.224	757	89	Grand Index
45	m	17+ years	0.225	757.925	89	Grand Index
45	m	17+ years	0.226	759	89	Grand Index
45	m	17+ years	0.227	759.4304843	89	Grand Index
45	m	17+ years	0.228	759.9691173	89	Grand Index
45	m	17+ years	0.232	760	89	Grand Index
45	m	17+ years	0.234	761	89	Grand Index
45	m	17+ years	0.235	761.855	89	Grand Index
45	m	17+ years	0.24	762	89	Grand Index
45	m	17+ years	0.241	762.613	89	Grand Index
45	m	17+ years	0.242	763	90	Grand Index
45	m	17+ years	0.246	764	90	Grand Index
45	m	17+ years	0.247	764.371	90	Grand Index
45	m	17+ years	0.248	765	90	Grand Index
45	m	17+ years	0.249	765.742001	90	Grand Index
45	m	17+ years	0.25	766	90	Grand Index
45	m	17+ years	0.251	766.1280227	90	Grand Index
45	m	17+ years	0.252	766.2663289	90	Grand Index
45	m	17+ years	0.257	767	90	Grand Index
45	m	17+ years	0.258	767.1450286	90	Grand Index
45	m	17+ years	0.259	767.9145896	90	Grand Index
45	m	17+ years	0.262	768	90	Grand Index
45	m	17+ years	0.263	768.059	90	Grand Index
45	m	17+ years	0.269	769	91	Grand Index
45	m	17+ years	0.271	770	91	Grand Index
45	m	17+ years	0.272	770.696	91	Grand Index
45	m	17+ years	0.274	771	91	Grand Index
45	m	17+ years	0.275	771.3450732	91	Grand Index
45	m	17+ years	0.276	771.9472168	91	Grand Index
45	m	17+ years	0.28	772	91	Grand Index
45	m	17+ years	0.281	772.333	91	Grand Index
45	m	17+ years	0.286	773	92	Grand Index
45	m	17+ years	0.287	773.1032876	92	Grand Index
45	m	17+ years	0.288	773.7441671	92	Grand Index
45	m	17+ years	0.289	774	92	Grand Index
45	m	17+ years	0.29	774.97	92	Grand Index
45	m	17+ years	0.296	775	92	Grand Index

Age	Gender	Education	Percentile	Raw Score	Norm Score	Assessment
45	m	17+ years	0.302	776	92	Grand Index
45	m	17+ years	0.303	776.0269751	92	Grand Index
45	m	17+ years	0.31	777	93	Grand Index
45	m	17+ years	0.312	778	93	Grand Index
45	m	17+ years	0.313	778.0443818	93	Grand Index
45	m	17+ years	0.316	779	93	Grand Index
45	m	17+ years	0.317	779.2326003	93	Grand Index
45	m	17+ years	0.322	780	93	Grand Index
45	m	17+ years	0.323	780.639	93	Grand Index
45	m	17+ years	0.326	781	93	Grand Index
45	m	17+ years	0.327	781.811	93	Grand Index
45	m	17+ years	0.331	782	93	Grand Index
45	m	17+ years	0.332	782.2712145	93	Grand Index
45	m	17+ years	0.333	782.9838479	94	Grand Index
45	m	17+ years	0.334	782.9978951	94	Grand Index
45	m	17+ years	0.341	783	94	Grand Index
45	m	17+ years	0.342	783.3563547	94	Grand Index
45	m	17+ years	0.343	783.9001992	94	Grand Index
45	m	17+ years	0.346	784	94	Grand Index
45	m	17+ years	0.347	784.671	94	Grand Index
45	m	17+ years	0.351	785	94	Grand Index
45	m	17+ years	0.352	785.9987414	94	Grand Index
45	m	17+ years	0.358	786	95	Grand Index
45	m	17+ years	0.359	786.0018349	95	Grand Index
45	m	17+ years	0.36	786.1252104	95	Grand Index
45	m	17+ years	0.361	786.7917035	95	Grand Index
45	m	17+ years	0.368	787	95	Grand Index
45	m	17+ years	0.369	787.016376	95	Grand Index
45	m	17+ years	0.37	787.4925799	95	Grand Index
45	m	17+ years	0.378	788	95	Grand Index
45	m	17+ years	0.379	788.5622223	95	Grand Index
45	m	17+ years	0.383	789	96	Grand Index
45	m	17+ years	0.384	789.4916054	96	Grand Index
45	m	17+ years	0.385	789.9922325	96	Grand Index
45	m	17+ years	0.395	790	96	Grand Index
45	m	17+ years	0.396	790.0232794	96	Grand Index
45	m	17+ years	0.397	790.8855248	96	Grand Index
45	m	17+ years	0.401	791	96	Grand Index
45	m	17+ years	0.402	791.786	96	Grand Index
45	m	17+ years	0.406	792	96	Grand Index
45	m	17+ years	0.409	793	97	Grand Index
45	m	17+ years	0.412	794	97	Grand Index
45	m	17+ years	0.416	795	97	Grand Index
45	m	17+ years	0.417	795.0503464	97	Grand Index
45	m	17+ years	0.418	795.6203106	97	Grand Index
45	m	17+ years	0.419	796	97	Grand Index
45	m	17+ years	0.42	796.06	97	Grand Index
45	m	17+ years	0.427	797	97	Grand Index
45	m	17+ years	0.428	797.0352155	97	Grand Index
45	m	17+ years	0.429	797.7234116	97	Grand Index
45	m	17+ years	0.433	798	97	Grand Index
45	m	17+ years	0.434	798.7146559	98	Grand Index
45	m	17+ years	0.438	799	98	Grand Index
45	m	17+ years	0.439	799.1256969	98	Grand Index
45	m	17+ years	0.44	799.9360379	98	Grand Index
45	m	17+ years	0.443	800	98	Grand Index

Age	Gender	Education	Percentile	Raw Score	Norm Score	Assessment
45	m	17+ years	0.445	801	98	Grand Index
45	m	17+ years	0.446	801.678	98	Grand Index
45	m	17+ years	0.45	802	98	Grand Index
45	m	17+ years	0.451	803	98	Grand Index
45	m	17+ years	0.452	803.436	98	Grand Index
45	m	17+ years	0.457	804	98	Grand Index
45	m	17+ years	0.458	804.0016015	98	Grand Index
45	m	17+ years	0.459	804.491235	98	Grand Index
45	m	17+ years	0.46	805	98	Grand Index
45	m	17+ years	0.461	805.0690664	99	Grand Index
45	m	17+ years	0.462	805.9658371	99	Grand Index
45	m	17+ years	0.467	806	99	Grand Index
45	m	17+ years	0.47	807	99	Grand Index
45	m	17+ years	0.471	807.4192463	99	Grand Index
45	m	17+ years	0.472	807.9814951	99	Grand Index
45	m	17+ years	0.474	808	99	Grand Index
45	m	17+ years	0.475	808.0281759	99	Grand Index
45	m	17+ years	0.476	808.1723597	99	Grand Index
45	m	17+ years	0.477	808.8052788	99	Grand Index
45	m	17+ years	0.478	809	99	Grand Index
45	m	17+ years	0.479	809.347	99	Grand Index
45	m	17+ years	0.482	810	99	Grand Index
45	m	17+ years	0.483	810.519	99	Grand Index
45	m	17+ years	0.486	811	99	Grand Index
45	m	17+ years	0.487	811.691	100	Grand Index
45	m	17+ years	0.491	812	100	Grand Index
45	m	17+ years	0.492	812.0123964	100	Grand Index
45	m	17+ years	0.493	812.1576718	100	Grand Index
45	m	17+ years	0.494	812.8074407	100	Grand Index
45	m	17+ years	0.495	813.1556515	100	Grand Index
45	m	17+ years	0.496	814.328	100	Grand Index
45	m	17+ years	0.502	815	100	Grand Index
45	m	17+ years	0.503	815.3298278	100	Grand Index
45	m	17+ years	0.504	815.9493996	100	Grand Index
45	m	17+ years	0.505	815.999581	100	Grand Index
45	m	17+ years	0.507	816	100	Grand Index
45	m	17+ years	0.508	816.8315646	100	Grand Index
45	m	17+ years	0.515	817	101	Grand Index
45	m	17+ years	0.516	817.3062356	101	Grand Index
45	m	17+ years	0.517	817.6496092	101	Grand Index
45	m	17+ years	0.518	817.9336323	101	Grand Index
45	m	17+ years	0.522	818	101	Grand Index
45	m	17+ years	0.523	818.2395513	101	Grand Index
45	m	17+ years	0.524	818.995813	101	Grand Index
45	m	17+ years	0.532	819	101	Grand Index
45	m	17+ years	0.533	819.0764532	101	Grand Index
45	m	17+ years	0.534	819.7053835	101	Grand Index
45	m	17+ years	0.538	820	101	Grand Index
45	m	17+ years	0.539	820.4693551	101	Grand Index
45	m	17+ years	0.54	820.8569707	102	Grand Index
45	m	17+ years	0.546	821	102	Grand Index
45	m	17+ years	0.55	822	102	Grand Index
45	m	17+ years	0.551	822.443	102	Grand Index
45	m	17+ years	0.552	823	102	Grand Index
45	m	17+ years	0.553	823.2612271	102	Grand Index
45	m	17+ years	0.554	823.7009417	102	Grand Index

Age	Gender	Education	Percentile	Raw Score	Norm Score	Assessment
45	m	17+ years	0.556	824	102	Grand Index
45	m	17+ years	0.557	824.201	102	Grand Index
45	m	17+ years	0.559	825	102	Grand Index
45	m	17+ years	0.56	825.0715416	102	Grand Index
45	m	17+ years	0.561	825.9278368	102	Grand Index
45	m	17+ years	0.562	825.9947435	102	Grand Index
45	m	17+ years	0.569	826	103	Grand Index
45	m	17+ years	0.57	826.0014194	103	Grand Index
45	m	17+ years	0.571	826.401929	103	Grand Index
45	m	17+ years	0.577	827	103	Grand Index
45	m	17+ years	0.578	827.2726046	103	Grand Index
45	m	17+ years	0.579	827.9188345	103	Grand Index
45	m	17+ years	0.588	828	103	Grand Index
45	m	17+ years	0.589	828.577	103	Grand Index
45	m	17+ years	0.593	829	104	Grand Index
45	m	17+ years	0.594	829.042	104	Grand Index
45	m	17+ years	0.602	830	104	Grand Index
45	m	17+ years	0.603	830.679	104	Grand Index
45	m	17+ years	0.608	831	104	Grand Index
45	m	17+ years	0.609	831.437	104	Grand Index
45	m	17+ years	0.613	832	104	Grand Index
45	m	17+ years	0.614	832.0036488	104	Grand Index
45	m	17+ years	0.615	832.7095875	104	Grand Index
45	m	17+ years	0.619	833	105	Grand Index
45	m	17+ years	0.62	833.0189793	105	Grand Index
45	m	17+ years	0.621	833.9543516	105	Grand Index
45	m	17+ years	0.622	834.9548063	105	Grand Index
45	m	17+ years	0.624	835	105	Grand Index
45	m	17+ years	0.626	836	105	Grand Index
45	m	17+ years	0.627	836.1022004	105	Grand Index
45	m	17+ years	0.628	836.9790131	105	Grand Index
45	m	17+ years	0.63	837	105	Grand Index
45	m	17+ years	0.631	837.883	105	Grand Index
45	m	17+ years	0.634	838	105	Grand Index
45	m	17+ years	0.635	838.055	105	Grand Index
45	m	17+ years	0.639	839	105	Grand Index
45	m	17+ years	0.64	839.5230061	105	Grand Index
45	m	17+ years	0.641	840.6521289	105	Grand Index
45	m	17+ years	0.646	841	106	Grand Index
45	m	17+ years	0.647	841.571	106	Grand Index
45	m	17+ years	0.648	842	106	Grand Index
45	m	17+ years	0.649	842.157	106	Grand Index
45	m	17+ years	0.655	843	106	Grand Index
45	m	17+ years	0.661	844	106	Grand Index
45	m	17+ years	0.662	844.966	106	Grand Index
45	m	17+ years	0.666	845	106	Grand Index
45	m	17+ years	0.667	845.2356455	106	Grand Index
45	m	17+ years	0.668	845.8749006	107	Grand Index
45	m	17+ years	0.672	846	107	Grand Index
45	m	17+ years	0.673	846.1851423	107	Grand Index
45	m	17+ years	0.674	846.9894271	107	Grand Index
45	m	17+ years	0.679	847	107	Grand Index
45	m	17+ years	0.68	847.2377681	107	Grand Index
45	m	17+ years	0.681	847.995657	107	Grand Index
45	m	17+ years	0.686	848	107	Grand Index
45	m	17+ years	0.687	848.291	107	Grand Index

Age	Gender	Education	Percentile	Raw Score	Norm Score	Assessment
45	m	17+ years	0.688	849.1516078	107	Grand Index
45	m	17+ years	0.689	849.9089311	107	Grand Index
45	m	17+ years	0.693	850	108	Grand Index
45	m	17+ years	0.694	850.342	108	Grand Index
45	m	17+ years	0.697	851	108	Grand Index
45	m	17+ years	0.698	851.2648536	108	Grand Index
45	m	17+ years	0.699	851.9064489	108	Grand Index
45	m	17+ years	0.704	852	108	Grand Index
45	m	17+ years	0.705	852.2168332	108	Grand Index
45	m	17+ years	0.706	852.9124961	108	Grand Index
45	m	17+ years	0.712	853	108	Grand Index
45	m	17+ years	0.713	853.394676	108	Grand Index
45	m	17+ years	0.714	853.5961769	108	Grand Index
45	m	17+ years	0.716	854	109	Grand Index
45	m	17+ years	0.717	854.0758805	109	Grand Index
45	m	17+ years	0.718	854.9604345	109	Grand Index
45	m	17+ years	0.721	855	109	Grand Index
45	m	17+ years	0.722	855.546	109	Grand Index
45	m	17+ years	0.726	856	109	Grand Index
45	m	17+ years	0.73	857	109	Grand Index
45	m	17+ years	0.731	858	109	Grand Index
45	m	17+ years	0.732	858.476	109	Grand Index
45	m	17+ years	0.735	859	109	Grand Index
45	m	17+ years	0.736	859.4722411	109	Grand Index
45	m	17+ years	0.737	859.9839973	110	Grand Index
45	m	17+ years	0.738	860.2247811	110	Grand Index
45	m	17+ years	0.739	860.9813652	110	Grand Index
45	m	17+ years	0.743	861	110	Grand Index
45	m	17+ years	0.744	861.992	110	Grand Index
45	m	17+ years	0.746	862	110	Grand Index
45	m	17+ years	0.747	862.871	110	Grand Index
45	m	17+ years	0.75	863	110	Grand Index
45	m	17+ years	0.751	863.1485951	110	Grand Index
45	m	17+ years	0.754	864	110	Grand Index
45	m	17+ years	0.755	864.3693031	110	Grand Index
45	m	17+ years	0.757	865	110	Grand Index
45	m	17+ years	0.762	866	111	Grand Index
45	m	17+ years	0.763	866.559	111	Grand Index
45	m	17+ years	0.766	867	111	Grand Index
45	m	17+ years	0.767	867.2198343	111	Grand Index
45	m	17+ years	0.77	868	111	Grand Index
45	m	17+ years	0.771	868.903	111	Grand Index
45	m	17+ years	0.773	869	111	Grand Index
45	m	17+ years	0.774	869.4355928	111	Grand Index
45	m	17+ years	0.775	869.9915884	111	Grand Index
45	m	17+ years	0.778	870	111	Grand Index
45	m	17+ years	0.779	870.2710223	112	Grand Index
45	m	17+ years	0.782	871	112	Grand Index
45	m	17+ years	0.783	871.419	112	Grand Index
45	m	17+ years	0.784	872	112	Grand Index
45	m	17+ years	0.785	872.005	112	Grand Index
45	m	17+ years	0.786	873	112	Grand Index
45	m	17+ years	0.787	873.205997	112	Grand Index
45	m	17+ years	0.788	873.9244326	112	Grand Index
45	m	17+ years	0.791	874	112	Grand Index
45	m	17+ years	0.792	874.8564957	112	Grand Index

Age	Gender	Education	Percentile	Raw Score	Norm Score	Assessment
45	m	17+ years	0.793	875	112	Grand Index
45	m	17+ years	0.794	875.642	112	Grand Index
45	m	17+ years	0.796	876	112	Grand Index
45	m	17+ years	0.797	876.494348	112	Grand Index
45	m	17+ years	0.798	876.9904851	113	Grand Index
45	m	17+ years	0.802	877	113	Grand Index
45	m	17+ years	0.803	877.1218709	113	Grand Index
45	m	17+ years	0.804	877.7589561	113	Grand Index
45	m	17+ years	0.805	878.865	113	Grand Index
45	m	17+ years	0.806	879.0244497	113	Grand Index
45	m	17+ years	0.807	879.5359549	113	Grand Index
45	m	17+ years	0.808	880	113	Grand Index
45	m	17+ years	0.809	880.0296244	113	Grand Index
45	m	17+ years	0.81	880.8103816	113	Grand Index
45	m	17+ years	0.811	880.9359548	113	Grand Index
45	m	17+ years	0.812	881	113	Grand Index
45	m	17+ years	0.813	881.209	113	Grand Index
45	m	17+ years	0.815	882	113	Grand Index
45	m	17+ years	0.816	882.0239308	114	Grand Index
45	m	17+ years	0.817	882.5493315	114	Grand Index
45	m	17+ years	0.822	883	114	Grand Index
45	m	17+ years	0.823	883.139	114	Grand Index
45	m	17+ years	0.826	884	114	Grand Index
45	m	17+ years	0.827	884.3093058	114	Grand Index
45	m	17+ years	0.828	884.9978427	114	Grand Index
45	m	17+ years	0.831	885	114	Grand Index
45	m	17+ years	0.832	885.776	114	Grand Index
45	m	17+ years	0.833	886	114	Grand Index
45	m	17+ years	0.834	886.0819111	115	Grand Index
45	m	17+ years	0.835	886.465201	115	Grand Index
45	m	17+ years	0.836	886.9403945	115	Grand Index
45	m	17+ years	0.837	886.9747263	115	Grand Index
45	m	17+ years	0.84	887	115	Grand Index
45	m	17+ years	0.841	887.413	115	Grand Index
45	m	17+ years	0.842	888	115	Grand Index
45	m	17+ years	0.843	888.368017	115	Grand Index
45	m	17+ years	0.846	890	115	Grand Index
45	m	17+ years	0.847	890.3915538	115	Grand Index
45	m	17+ years	0.848	891	115	Grand Index
45	m	17+ years	0.849	891.1828401	115	Grand Index
45	m	17+ years	0.85	891.922828	116	Grand Index
45	m	17+ years	0.851	892.343	116	Grand Index
45	m	17+ years	0.853	893	116	Grand Index
45	m	17+ years	0.854	893.222	116	Grand Index
45	m	17+ years	0.856	894	116	Grand Index
45	m	17+ years	0.857	894.832151	116	Grand Index
45	m	17+ years	0.858	895.0087658	116	Grand Index
45	m	17+ years	0.859	895.0309627	116	Grand Index
45	m	17+ years	0.86	895.0966075	116	Grand Index
45	m	17+ years	0.861	895.8622333	116	Grand Index
45	m	17+ years	0.863	896	116	Grand Index
45	m	17+ years	0.866	897	117	Grand Index
45	m	17+ years	0.867	897.0023796	117	Grand Index
45	m	17+ years	0.868	897.3230773	117	Grand Index
45	m	17+ years	0.869	898.5545706	117	Grand Index
45	m	17+ years	0.87	899	117	Grand Index

Age	Gender	Education	Percentile	Raw Score	Norm Score	Assessment
45	m	17+ years	0.871	899.203	117	Grand Index
45	m	17+ years	0.873	900	117	Grand Index
45	m	17+ years	0.874	900.6530706	117	Grand Index
45	m	17+ years	0.877	901	117	Grand Index
45	m	17+ years	0.88	903	118	Grand Index
45	m	17+ years	0.881	903.4757496	118	Grand Index
45	m	17+ years	0.882	903.9817734	118	Grand Index
45	m	17+ years	0.883	904	118	Grand Index
45	m	17+ years	0.884	904.0011785	118	Grand Index
45	m	17+ years	0.885	904.3732523	118	Grand Index
45	m	17+ years	0.887	905	118	Grand Index
45	m	17+ years	0.888	905.2065343	118	Grand Index
45	m	17+ years	0.89	906	118	Grand Index
45	m	17+ years	0.891	906.063	118	Grand Index
45	m	17+ years	0.893	907	119	Grand Index
45	m	17+ years	0.894	907.5314954	119	Grand Index
45	m	17+ years	0.895	908	119	Grand Index
45	m	17+ years	0.896	908.528	119	Grand Index
45	m	17+ years	0.897	909.821	119	Grand Index
45	m	17+ years	0.898	910	119	Grand Index
45	m	17+ years	0.899	910.814	119	Grand Index
45	m	17+ years	0.901	912	119	Grand Index
45	m	17+ years	0.903	914	119	Grand Index
45	m	17+ years	0.904	914.872	120	Grand Index
45	m	17+ years	0.906	915	120	Grand Index
45	m	17+ years	0.907	915.7018692	120	Grand Index
45	m	17+ years	0.908	916.043737	120	Grand Index
45	m	17+ years	0.909	916.9960375	120	Grand Index
45	m	17+ years	0.911	917	120	Grand Index
45	m	17+ years	0.912	917.2301776	120	Grand Index
45	m	17+ years	0.913	918.1102649	120	Grand Index
45	m	17+ years	0.914	918.8323563	120	Grand Index
45	m	17+ years	0.915	919.095	121	Grand Index
45	m	17+ years	0.916	920.4595629	121	Grand Index
45	m	17+ years	0.917	921.7398365	121	Grand Index
45	m	17+ years	0.918	922	121	Grand Index
45	m	17+ years	0.919	922.9646196	121	Grand Index
45	m	17+ years	0.92	923.8018603	121	Grand Index
45	m	17+ years	0.921	924.3542622	121	Grand Index
45	m	17+ years	0.922	924.6295168	121	Grand Index
45	m	17+ years	0.923	924.7573746	121	Grand Index
45	m	17+ years	0.924	924.9710723	121	Grand Index
45	m	17+ years	0.926	925	122	Grand Index
45	m	17+ years	0.927	925.0085621	122	Grand Index
45	m	17+ years	0.928	925.0606812	122	Grand Index
45	m	17+ years	0.929	926.9888115	122	Grand Index
45	m	17+ years	0.931	927	122	Grand Index
45	m	17+ years	0.932	927.1977001	122	Grand Index
45	m	17+ years	0.934	928	123	Grand Index
45	m	17+ years	0.935	929.91	123	Grand Index
45	m	17+ years	0.936	930	123	Grand Index
45	m	17+ years	0.937	930.541	123	Grand Index
45	m	17+ years	0.938	931.834	123	Grand Index
45	m	17+ years	0.939	933	123	Grand Index
45	m	17+ years	0.94	933.42	123	Grand Index
45	m	17+ years	0.941	934	123	Grand Index

Age	Gender	Education	Percentile	Raw Score	Norm Score	Assessment
45	m	17+ years	0.942	935	124	Grand Index
45	m	17+ years	0.943	935.0950559	124	Grand Index
45	m	17+ years	0.944	936.9057084	124	Grand Index
45	m	17+ years	0.945	938	124	Grand Index
45	m	17+ years	0.946	938.178	124	Grand Index
45	m	17+ years	0.948	939	124	Grand Index
45	m	17+ years	0.949	939.0151784	125	Grand Index
45	m	17+ years	0.95	939.3748039	125	Grand Index
45	m	17+ years	0.951	939.8487508	125	Grand Index
45	m	17+ years	0.952	940	125	Grand Index
45	m	17+ years	0.953	940.4897633	125	Grand Index
45	m	17+ years	0.954	942.1914558	125	Grand Index
45	m	17+ years	0.955	942.8828531	125	Grand Index
45	m	17+ years	0.956	943.0301629	126	Grand Index
45	m	17+ years	0.957	943.5682922	126	Grand Index
45	m	17+ years	0.958	944.2380954	126	Grand Index
45	m	17+ years	0.959	945.97846	126	Grand Index
45	m	17+ years	0.96	946.28	126	Grand Index
45	m	17+ years	0.961	947	126	Grand Index
45	m	17+ years	0.962	947.866	127	Grand Index
45	m	17+ years	0.963	948.159	127	Grand Index
45	m	17+ years	0.964	949	127	Grand Index
45	m	17+ years	0.965	949.745	127	Grand Index
45	m	17+ years	0.966	952	127	Grand Index
45	m	17+ years	0.967	952.6536117	128	Grand Index
45	m	17+ years	0.968	953.9904713	128	Grand Index
45	m	17+ years	0.969	954.0626064	128	Grand Index
45	m	17+ years	0.97	955	128	Grand Index
45	m	17+ years	0.971	955.7408176	128	Grand Index
45	m	17+ years	0.972	957.4365007	129	Grand Index
45	m	17+ years	0.973	958.089	129	Grand Index
45	m	17+ years	0.974	959.3785824	129	Grand Index
45	m	17+ years	0.975	960.0115658	129	Grand Index
45	m	17+ years	0.976	960.9686861	130	Grand Index
45	m	17+ years	0.977	963	130	Grand Index
45	m	17+ years	0.978	963.4459155	130	Grand Index
45	m	17+ years	0.979	963.97015	131	Grand Index
45	m	17+ years	0.98	965.14	131	Grand Index
45	m	17+ years	0.981	966	131	Grand Index
45	m	17+ years	0.982	966.726	131	Grand Index
45	m	17+ years	0.983	967.0441079	132	Grand Index
45	m	17+ years	0.984	969.56	132	Grand Index
45	m	17+ years	0.985	974.2095269	133	Grand Index
45	m	17+ years	0.986	975.6572413	133	Grand Index
45	m	17+ years	0.987	976.9831983	133	Grand Index
45	m	17+ years	0.988	978.936	134	Grand Index
45	m	17+ years	0.989	981.777	134	Grand Index
45	m	17+ years	0.99	983.14	135	Grand Index
45	m	17+ years	0.991	986.9985348	135	Grand Index
45	m	17+ years	0.992	996.5906579	136	Grand Index
45	m	17+ years	0.993	1002.1005	137	Grand Index
45	m	17+ years	0.994	1007.42	138	Grand Index
45	m	17+ years	0.995	1015.535	139	Grand Index
45	m	17+ years	0.996	1020.349028	140	Grand Index
45	m	17+ years	0.997	1023.561438	141	Grand Index
45	m	17+ years	0.998	1037.656	143	Grand Index

Age	Gender	Education	Percentile	Raw Score	Norm Score	Assessment
45	m	17+ years	0.999	1043.902298	146	Grand Index
50	f	0-12 years	0.001	575.4022526	54	Grand Index
50	f	0-12 years	0.002	591.1257306	57	Grand Index
50	f	0-12 years	0.003	602.788	59	Grand Index
50	f	0-12 years	0.004	612.0948005	60	Grand Index
50	f	0-12 years	0.005	617.5823652	61	Grand Index
50	f	0-12 years	0.006	623.8270561	62	Grand Index
50	f	0-12 years	0.007	637.0922204	63	Grand Index
50	f	0-12 years	0.008	645.8761133	64	Grand Index
50	f	0-12 years	0.009	648.8231133	65	Grand Index
50	f	0-12 years	0.01	649.1318564	65	Grand Index
50	f	0-12 years	0.011	649.7585937	66	Grand Index
50	f	0-12 years	0.012	650.596	66	Grand Index
50	f	0-12 years	0.013	654.1042211	67	Grand Index
50	f	0-12 years	0.014	658.0887228	67	Grand Index
50	f	0-12 years	0.015	660.3394171	67	Grand Index
50	f	0-12 years	0.016	664.2207423	68	Grand Index
50	f	0-12 years	0.017	665.9233852	68	Grand Index
50	f	0-12 years	0.018	666.8846095	69	Grand Index
50	f	0-12 years	0.019	667.777	69	Grand Index
50	f	0-12 years	0.02	668	69	Grand Index
50	f	0-12 years	0.021	669.5410116	69	Grand Index
50	f	0-12 years	0.022	671.9847732	70	Grand Index
50	f	0-12 years	0.023	673.6738414	70	Grand Index
50	f	0-12 years	0.024	674.5723486	70	Grand Index
50	f	0-12 years	0.026	677	71	Grand Index
50	f	0-12 years	0.027	677.841	71	Grand Index
50	f	0-12 years	0.028	678.8928596	71	Grand Index
50	f	0-12 years	0.029	680.9196047	72	Grand Index
50	f	0-12 years	0.03	682.363458	72	Grand Index
50	f	0-12 years	0.031	683.2036115	72	Grand Index
50	f	0-12 years	0.032	684.256	72	Grand Index
50	f	0-12 years	0.033	685.045209	72	Grand Index
50	f	0-12 years	0.034	685.3840891	73	Grand Index
50	f	0-12 years	0.035	687.7458982	73	Grand Index
50	f	0-12 years	0.036	688	73	Grand Index
50	f	0-12 years	0.037	688.223037	73	Grand Index
50	f	0-12 years	0.038	688.6901558	73	Grand Index
50	f	0-12 years	0.039	689.8617112	74	Grand Index
50	f	0-12 years	0.04	691	74	Grand Index
50	f	0-12 years	0.041	691.203	74	Grand Index
50	f	0-12 years	0.043	692	74	Grand Index
50	f	0-12 years	0.044	693.5882295	74	Grand Index
50	f	0-12 years	0.045	693.9639916	75	Grand Index
50	f	0-12 years	0.046	694.618	75	Grand Index
50	f	0-12 years	0.047	696.002	75	Grand Index
50	f	0-12 years	0.048	697	75	Grand Index
50	f	0-12 years	0.049	697.267	75	Grand Index
50	f	0-12 years	0.05	698	75	Grand Index
50	f	0-12 years	0.051	698.033	75	Grand Index
50	f	0-12 years	0.052	698.916	76	Grand Index
50	f	0-12 years	0.053	699	76	Grand Index
50	f	0-12 years	0.054	699.682	76	Grand Index
50	f	0-12 years	0.055	700.565	76	Grand Index
50	f	0-12 years	0.056	701	76	Grand Index
50	f	0-12 years	0.057	701.331	76	Grand Index

Age	Gender	Education	Percentile	Raw Score	Norm Score	Assessment
50	f	0-12 years	0.061	702	77	Grand Index
50	f	0-12 years	0.062	703.492	77	Grand Index
50	f	0-12 years	0.063	705.258	77	Grand Index
50	f	0-12 years	0.064	706	77	Grand Index
50	f	0-12 years	0.065	706.79	77	Grand Index
50	f	0-12 years	0.067	708	78	Grand Index
50	f	0-12 years	0.068	708.088	78	Grand Index
50	f	0-12 years	0.069	709.854	78	Grand Index
50	f	0-12 years	0.07	710.0167961	78	Grand Index
50	f	0-12 years	0.071	710.6993659	78	Grand Index
50	f	0-12 years	0.072	711	78	Grand Index
50	f	0-12 years	0.073	711.0778465	78	Grand Index
50	f	0-12 years	0.074	711.453597	78	Grand Index
50	f	0-12 years	0.075	712.0436287	78	Grand Index
50	f	0-12 years	0.076	712.2809635	79	Grand Index
50	f	0-12 years	0.077	712.9927451	79	Grand Index
50	f	0-12 years	0.078	713	79	Grand Index
50	f	0-12 years	0.079	713.757	79	Grand Index
50	f	0-12 years	0.08	714	79	Grand Index
50	f	0-12 years	0.081	714.3406731	79	Grand Index
50	f	0-12 years	0.082	714.7929212	79	Grand Index
50	f	0-12 years	0.087	715	80	Grand Index
50	f	0-12 years	0.088	715.0776706	80	Grand Index
50	f	0-12 years	0.089	715.6325653	80	Grand Index
50	f	0-12 years	0.09	716	80	Grand Index
50	f	0-12 years	0.091	716.353	80	Grand Index
50	f	0-12 years	0.092	717	80	Grand Index
50	f	0-12 years	0.093	717.119	80	Grand Index
50	f	0-12 years	0.097	718	81	Grand Index
50	f	0-12 years	0.098	718.534	81	Grand Index
50	f	0-12 years	0.1	719	81	Grand Index
50	f	0-12 years	0.101	719.366	81	Grand Index
50	f	0-12 years	0.102	721.066	81	Grand Index
50	f	0-12 years	0.103	721.949	81	Grand Index
50	f	0-12 years	0.104	722	81	Grand Index
50	f	0-12 years	0.105	722.715	81	Grand Index
50	f	0-12 years	0.106	723.0729145	81	Grand Index
50	f	0-12 years	0.107	723.5127684	81	Grand Index
50	f	0-12 years	0.108	723.9583313	81	Grand Index
50	f	0-12 years	0.109	724	82	Grand Index
50	f	0-12 years	0.11	724.2571887	82	Grand Index
50	f	0-12 years	0.111	725.978566	82	Grand Index
50	f	0-12 years	0.112	725.9915548	82	Grand Index
50	f	0-12 years	0.113	726.8352381	82	Grand Index
50	f	0-12 years	0.114	727.6870642	82	Grand Index
50	f	0-12 years	0.115	728.545	82	Grand Index
50	f	0-12 years	0.116	729.428	82	Grand Index
50	f	0-12 years	0.117	730.311	82	Grand Index
50	f	0-12 years	0.12	731	82	Grand Index
50	f	0-12 years	0.121	731.9002494	82	Grand Index
50	f	0-12 years	0.122	732.7446078	83	Grand Index
50	f	0-12 years	0.123	733.609	83	Grand Index
50	f	0-12 years	0.124	734.3662615	83	Grand Index
50	f	0-12 years	0.125	734.8402712	83	Grand Index
50	f	0-12 years	0.126	735.2561199	83	Grand Index
50	f	0-12 years	0.127	735.9937402	83	Grand Index

Age	Gender	Education	Percentile	Raw Score	Norm Score	Assessment
50	f	0-12 years	0.129	736	83	Grand Index
50	f	0-12 years	0.13	736.79	83	Grand Index
50	f	0-12 years	0.137	737	84	Grand Index
50	f	0-12 years	0.138	737.854	84	Grand Index
50	f	0-12 years	0.139	738	84	Grand Index
50	f	0-12 years	0.14	738.62	84	Grand Index
50	f	0-12 years	0.141	739.3881491	84	Grand Index
50	f	0-12 years	0.142	739.8598043	84	Grand Index
50	f	0-12 years	0.143	740	84	Grand Index
50	f	0-12 years	0.144	740.152	84	Grand Index
50	f	0-12 years	0.147	741	84	Grand Index
50	f	0-12 years	0.148	741.5740914	84	Grand Index
50	f	0-12 years	0.149	741.8425225	84	Grand Index
50	f	0-12 years	0.15	741.8889921	84	Grand Index
50	f	0-12 years	0.151	741.9618438	85	Grand Index
50	f	0-12 years	0.152	742.216	85	Grand Index
50	f	0-12 years	0.155	743	85	Grand Index
50	f	0-12 years	0.156	743.748	85	Grand Index
50	f	0-12 years	0.16	744	85	Grand Index
50	f	0-12 years	0.161	744.1441224	85	Grand Index
50	f	0-12 years	0.162	744.8895138	85	Grand Index
50	f	0-12 years	0.163	744.9917772	85	Grand Index
50	f	0-12 years	0.164	745.812	85	Grand Index
50	f	0-12 years	0.165	746.695	85	Grand Index
50	f	0-12 years	0.166	748.156	85	Grand Index
50	f	0-12 years	0.168	749	86	Grand Index
50	f	0-12 years	0.169	749.0176901	86	Grand Index
50	f	0-12 years	0.17	749.1793575	86	Grand Index
50	f	0-12 years	0.171	749.9935455	86	Grand Index
50	f	0-12 years	0.172	750	86	Grand Index
50	f	0-12 years	0.173	750.759	86	Grand Index
50	f	0-12 years	0.174	751	86	Grand Index
50	f	0-12 years	0.175	751.2995718	86	Grand Index
50	f	0-12 years	0.176	751.7458029	86	Grand Index
50	f	0-12 years	0.177	752	86	Grand Index
50	f	0-12 years	0.178	752.174	86	Grand Index
50	f	0-12 years	0.184	753	86	Grand Index
50	f	0-12 years	0.185	753.0726889	87	Grand Index
50	f	0-12 years	0.186	753.3937262	87	Grand Index
50	f	0-12 years	0.187	754.0595165	87	Grand Index
50	f	0-12 years	0.188	754.5029937	87	Grand Index
50	f	0-12 years	0.189	754.9436127	87	Grand Index
50	f	0-12 years	0.198	755	87	Grand Index
50	f	0-12 years	0.199	755.5164349	87	Grand Index
50	f	0-12 years	0.2	755.8881087	87	Grand Index
50	f	0-12 years	0.201	756.0143194	87	Grand Index
50	f	0-12 years	0.202	756.3847961	87	Grand Index
50	f	0-12 years	0.204	757	88	Grand Index
50	f	0-12 years	0.205	757.012814	88	Grand Index
50	f	0-12 years	0.206	757.7671295	88	Grand Index
50	f	0-12 years	0.207	757.9680839	88	Grand Index
50	f	0-12 years	0.21	758	88	Grand Index
50	f	0-12 years	0.211	758.313	88	Grand Index
50	f	0-12 years	0.212	759	88	Grand Index
50	f	0-12 years	0.213	759.079	88	Grand Index
50	f	0-12 years	0.214	759.962	88	Grand Index

Age	Gender	Education	Percentile	Raw Score	Norm Score	Assessment
50	f	0-12 years	0.215	760.7798599	88	Grand Index
50	f	0-12 years	0.216	760.9790318	88	Grand Index
50	f	0-12 years	0.217	761	88	Grand Index
50	f	0-12 years	0.218	761.494	88	Grand Index
50	f	0-12 years	0.22	762	88	Grand Index
50	f	0-12 years	0.221	762.0015375	88	Grand Index
50	f	0-12 years	0.222	762.0307617	89	Grand Index
50	f	0-12 years	0.223	762.7103218	89	Grand Index
50	f	0-12 years	0.224	762.954314	89	Grand Index
50	f	0-12 years	0.225	763.675	89	Grand Index
50	f	0-12 years	0.226	764.558	89	Grand Index
50	f	0-12 years	0.228	765	89	Grand Index
50	f	0-12 years	0.229	765.207	89	Grand Index
50	f	0-12 years	0.232	766	89	Grand Index
50	f	0-12 years	0.233	766.739	89	Grand Index
50	f	0-12 years	0.234	767.622	89	Grand Index
50	f	0-12 years	0.236	768	89	Grand Index
50	f	0-12 years	0.237	768.0651354	89	Grand Index
50	f	0-12 years	0.238	768.3573378	89	Grand Index
50	f	0-12 years	0.243	769	90	Grand Index
50	f	0-12 years	0.244	769.452	90	Grand Index
50	f	0-12 years	0.246	770	90	Grand Index
50	f	0-12 years	0.247	770.101	90	Grand Index
50	f	0-12 years	0.248	770.984	90	Grand Index
50	f	0-12 years	0.25	771	90	Grand Index
50	f	0-12 years	0.251	771.0065776	90	Grand Index
50	f	0-12 years	0.252	771.211185	90	Grand Index
50	f	0-12 years	0.253	771.6037192	90	Grand Index
50	f	0-12 years	0.254	771.9363045	90	Grand Index
50	f	0-12 years	0.258	772	90	Grand Index
50	f	0-12 years	0.259	772.6598925	90	Grand Index
50	f	0-12 years	0.26	772.9776397	90	Grand Index
50	f	0-12 years	0.266	773	91	Grand Index
50	f	0-12 years	0.267	773.4461701	91	Grand Index
50	f	0-12 years	0.268	773.6304303	91	Grand Index
50	f	0-12 years	0.269	773.8367338	91	Grand Index
50	f	0-12 years	0.27	774.41	91	Grand Index
50	f	0-12 years	0.274	775	91	Grand Index
50	f	0-12 years	0.275	775.1815526	91	Grand Index
50	f	0-12 years	0.276	775.7722586	91	Grand Index
50	f	0-12 years	0.279	776	91	Grand Index
50	f	0-12 years	0.28	776.1710589	91	Grand Index
50	f	0-12 years	0.281	776.7480776	91	Grand Index
50	f	0-12 years	0.285	777	91	Grand Index
50	f	0-12 years	0.286	777.1701312	92	Grand Index
50	f	0-12 years	0.287	777.6040966	92	Grand Index
50	f	0-12 years	0.289	778	92	Grand Index
50	f	0-12 years	0.29	778.07	92	Grand Index
50	f	0-12 years	0.291	778.953	92	Grand Index
50	f	0-12 years	0.293	779	92	Grand Index
50	f	0-12 years	0.294	779.0967163	92	Grand Index
50	f	0-12 years	0.295	779.567739	92	Grand Index
50	f	0-12 years	0.301	780	92	Grand Index
50	f	0-12 years	0.302	780.0460458	92	Grand Index
50	f	0-12 years	0.303	780.5801812	92	Grand Index
50	f	0-12 years	0.31	781	93	Grand Index

Age	Gender	Education	Percentile	Raw Score	Norm Score	Assessment
50	f	0-12 years	0.311	781.613	93	Grand Index
50	f	0-12 years	0.315	782	93	Grand Index
50	f	0-12 years	0.316	782.028	93	Grand Index
50	f	0-12 years	0.317	782.911	93	Grand Index
50	f	0-12 years	0.323	783	93	Grand Index
50	f	0-12 years	0.324	783.092	93	Grand Index
50	f	0-12 years	0.325	783.975	93	Grand Index
50	f	0-12 years	0.33	784	93	Grand Index
50	f	0-12 years	0.331	784.1427337	93	Grand Index
50	f	0-12 years	0.332	784.5972721	93	Grand Index
50	f	0-12 years	0.334	785	94	Grand Index
50	f	0-12 years	0.335	785.805	94	Grand Index
50	f	0-12 years	0.336	786	94	Grand Index
50	f	0-12 years	0.337	786.1979057	94	Grand Index
50	f	0-12 years	0.338	786.6432408	94	Grand Index
50	f	0-12 years	0.339	787	94	Grand Index
50	f	0-12 years	0.34	787.22	94	Grand Index
50	f	0-12 years	0.345	788	94	Grand Index
50	f	0-12 years	0.346	788.518	94	Grand Index
50	f	0-12 years	0.348	789	94	Grand Index
50	f	0-12 years	0.349	789.167	94	Grand Index
50	f	0-12 years	0.352	790	94	Grand Index
50	f	0-12 years	0.353	790.699	94	Grand Index
50	f	0-12 years	0.354	791	94	Grand Index
50	f	0-12 years	0.355	791.3402522	94	Grand Index
50	f	0-12 years	0.356	791.8250848	94	Grand Index
50	f	0-12 years	0.357	792	95	Grand Index
50	f	0-12 years	0.358	792.0730204	95	Grand Index
50	f	0-12 years	0.359	792.6386085	95	Grand Index
50	f	0-12 years	0.36	792.9568636	95	Grand Index
50	f	0-12 years	0.361	793	95	Grand Index
50	f	0-12 years	0.362	793.0422796	95	Grand Index
50	f	0-12 years	0.363	793.5598261	95	Grand Index
50	f	0-12 years	0.37	794	95	Grand Index
50	f	0-12 years	0.371	794.593	95	Grand Index
50	f	0-12 years	0.374	795	95	Grand Index
50	f	0-12 years	0.375	795.1182949	95	Grand Index
50	f	0-12 years	0.376	795.9467881	95	Grand Index
50	f	0-12 years	0.377	795.9941531	95	Grand Index
50	f	0-12 years	0.381	796	95	Grand Index
50	f	0-12 years	0.382	796.2642638	95	Grand Index
50	f	0-12 years	0.383	796.8893855	96	Grand Index
50	f	0-12 years	0.389	797	96	Grand Index
50	f	0-12 years	0.39	797.37	96	Grand Index
50	f	0-12 years	0.392	798	96	Grand Index
50	f	0-12 years	0.393	798.019	96	Grand Index
50	f	0-12 years	0.394	798.902	96	Grand Index
50	f	0-12 years	0.396	799	96	Grand Index
50	f	0-12 years	0.397	799.551	96	Grand Index
50	f	0-12 years	0.404	800	96	Grand Index
50	f	0-12 years	0.405	800.615	96	Grand Index
50	f	0-12 years	0.408	801	97	Grand Index
50	f	0-12 years	0.409	801.147	97	Grand Index
50	f	0-12 years	0.417	802	97	Grand Index
50	f	0-12 years	0.418	802.0319082	97	Grand Index
50	f	0-12 years	0.419	802.3316413	97	Grand Index

Age	Gender	Education	Percentile	Raw Score	Norm Score	Assessment
50	f	0-12 years	0.42	802.9075228	97	Grand Index
50	f	0-12 years	0.426	803	97	Grand Index
50	f	0-12 years	0.427	803.0401821	97	Grand Index
50	f	0-12 years	0.428	803.9055673	97	Grand Index
50	f	0-12 years	0.429	803.9961499	97	Grand Index
50	f	0-12 years	0.436	804	98	Grand Index
50	f	0-12 years	0.437	804.4826973	98	Grand Index
50	f	0-12 years	0.438	804.8903301	98	Grand Index
50	f	0-12 years	0.439	805.0026057	98	Grand Index
50	f	0-12 years	0.44	805.5219635	98	Grand Index
50	f	0-12 years	0.441	806.0342299	98	Grand Index
50	f	0-12 years	0.442	806.3466455	98	Grand Index
50	f	0-12 years	0.451	807	98	Grand Index
50	f	0-12 years	0.452	807.0528236	98	Grand Index
50	f	0-12 years	0.453	807.4549206	98	Grand Index
50	f	0-12 years	0.454	807.7311325	98	Grand Index
50	f	0-12 years	0.455	807.9257827	98	Grand Index
50	f	0-12 years	0.456	807.990934	98	Grand Index
50	f	0-12 years	0.457	808	98	Grand Index
50	f	0-12 years	0.458	808.414	98	Grand Index
50	f	0-12 years	0.462	809	99	Grand Index
50	f	0-12 years	0.463	809.8147621	99	Grand Index
50	f	0-12 years	0.464	809.9950537	99	Grand Index
50	f	0-12 years	0.472	810	99	Grand Index
50	f	0-12 years	0.473	810.6083964	99	Grand Index
50	f	0-12 years	0.474	810.9648309	99	Grand Index
50	f	0-12 years	0.479	811	99	Grand Index
50	f	0-12 years	0.48	811.84	99	Grand Index
50	f	0-12 years	0.486	812	99	Grand Index
50	f	0-12 years	0.487	812.021	100	Grand Index
50	f	0-12 years	0.488	812.904	100	Grand Index
50	f	0-12 years	0.491	813	100	Grand Index
50	f	0-12 years	0.492	813.436	100	Grand Index
50	f	0-12 years	0.501	814	100	Grand Index
50	f	0-12 years	0.502	814.266	100	Grand Index
50	f	0-12 years	0.507	815	100	Grand Index
50	f	0-12 years	0.508	815.5364545	100	Grand Index
50	f	0-12 years	0.509	815.9647447	100	Grand Index
50	f	0-12 years	0.51	815.9876386	100	Grand Index
50	f	0-12 years	0.516	816	101	Grand Index
50	f	0-12 years	0.517	816.511	101	Grand Index
50	f	0-12 years	0.522	817	101	Grand Index
50	f	0-12 years	0.523	817.809	101	Grand Index
50	f	0-12 years	0.524	818	101	Grand Index
50	f	0-12 years	0.525	818.1077056	101	Grand Index
50	f	0-12 years	0.526	818.5595243	101	Grand Index
50	f	0-12 years	0.53	819	101	Grand Index
50	f	0-12 years	0.531	819.873	101	Grand Index
50	f	0-12 years	0.533	820	101	Grand Index
50	f	0-12 years	0.534	820.522	101	Grand Index
50	f	0-12 years	0.539	821	101	Grand Index
50	f	0-12 years	0.54	821.82	102	Grand Index
50	f	0-12 years	0.543	822	102	Grand Index
50	f	0-12 years	0.544	822.352	102	Grand Index
50	f	0-12 years	0.548	823	102	Grand Index
50	f	0-12 years	0.549	823.767	102	Grand Index

Age	Gender	Education	Percentile	Raw Score	Norm Score	Assessment
50	f	0-12 years	0.552	824	102	Grand Index
50	f	0-12 years	0.553	824.0209287	102	Grand Index
50	f	0-12 years	0.554	824.2392564	102	Grand Index
50	f	0-12 years	0.558	825	102	Grand Index
50	f	0-12 years	0.559	825.597	102	Grand Index
50	f	0-12 years	0.562	826	102	Grand Index
50	f	0-12 years	0.563	826.129	102	Grand Index
50	f	0-12 years	0.573	827	103	Grand Index
50	f	0-12 years	0.574	827.842	103	Grand Index
50	f	0-12 years	0.578	828	103	Grand Index
50	f	0-12 years	0.579	828.257	103	Grand Index
50	f	0-12 years	0.584	829	103	Grand Index
50	f	0-12 years	0.585	829.555	103	Grand Index
50	f	0-12 years	0.594	830	104	Grand Index
50	f	0-12 years	0.595	830.242685	104	Grand Index
50	f	0-12 years	0.596	830.7294167	104	Grand Index
50	f	0-12 years	0.602	831	104	Grand Index
50	f	0-12 years	0.603	831.1339155	104	Grand Index
50	f	0-12 years	0.604	831.5308244	104	Grand Index
50	f	0-12 years	0.605	831.9990341	104	Grand Index
50	f	0-12 years	0.61	832	104	Grand Index
50	f	0-12 years	0.611	832.513	104	Grand Index
50	f	0-12 years	0.612	833	104	Grand Index
50	f	0-12 years	0.613	833.2649249	104	Grand Index
50	f	0-12 years	0.614	833.9577242	104	Grand Index
50	f	0-12 years	0.619	834	105	Grand Index
50	f	0-12 years	0.62	834.46	105	Grand Index
50	f	0-12 years	0.63	835	105	Grand Index
50	f	0-12 years	0.631	835.0102293	105	Grand Index
50	f	0-12 years	0.632	835.1118175	105	Grand Index
50	f	0-12 years	0.633	835.9426069	105	Grand Index
50	f	0-12 years	0.639	836	105	Grand Index
50	f	0-12 years	0.64	836.1192736	105	Grand Index
50	f	0-12 years	0.641	836.9939645	105	Grand Index
50	f	0-12 years	0.642	836.9993099	105	Grand Index
50	f	0-12 years	0.644	837	106	Grand Index
50	f	0-12 years	0.645	837.535	106	Grand Index
50	f	0-12 years	0.651	838	106	Grand Index
50	f	0-12 years	0.652	838.716	106	Grand Index
50	f	0-12 years	0.659	839	106	Grand Index
50	f	0-12 years	0.66	839.7653476	106	Grand Index
50	f	0-12 years	0.661	839.9936694	106	Grand Index
50	f	0-12 years	0.664	840	106	Grand Index
50	f	0-12 years	0.665	840.1630325	106	Grand Index
50	f	0-12 years	0.666	840.848851	106	Grand Index
50	f	0-12 years	0.667	840.9936065	106	Grand Index
50	f	0-12 years	0.676	841	107	Grand Index
50	f	0-12 years	0.677	841.791	107	Grand Index
50	f	0-12 years	0.681	842	107	Grand Index
50	f	0-12 years	0.682	842.087658	107	Grand Index
50	f	0-12 years	0.683	842.4766525	107	Grand Index
50	f	0-12 years	0.684	842.9839147	107	Grand Index
50	f	0-12 years	0.685	843	107	Grand Index
50	f	0-12 years	0.686	843.738	107	Grand Index
50	f	0-12 years	0.689	844	107	Grand Index
50	f	0-12 years	0.69	844.0406319	107	Grand Index

Age	Gender	Education	Percentile	Raw Score	Norm Score	Assessment
50	f	0-12 years	0.691	844.2804638	107	Grand Index
50	f	0-12 years	0.694	845	108	Grand Index
50	f	0-12 years	0.695	846.37	108	Grand Index
50	f	0-12 years	0.696	847.568	108	Grand Index
50	f	0-12 years	0.697	848	108	Grand Index
50	f	0-12 years	0.698	848.0054888	108	Grand Index
50	f	0-12 years	0.699	848.2042271	108	Grand Index
50	f	0-12 years	0.7	848.8936573	108	Grand Index
50	f	0-12 years	0.701	848.9979913	108	Grand Index
50	f	0-12 years	0.702	849.866	108	Grand Index
50	f	0-12 years	0.703	850.1357611	108	Grand Index
50	f	0-12 years	0.704	850.6987024	108	Grand Index
50	f	0-12 years	0.706	851	108	Grand Index
50	f	0-12 years	0.707	851.0804841	108	Grand Index
50	f	0-12 years	0.708	851.3788262	108	Grand Index
50	f	0-12 years	0.709	851.856927	108	Grand Index
50	f	0-12 years	0.71	851.989491	108	Grand Index
50	f	0-12 years	0.714	852	108	Grand Index
50	f	0-12 years	0.715	852.345	109	Grand Index
50	f	0-12 years	0.72	853	109	Grand Index
50	f	0-12 years	0.721	853.643	109	Grand Index
50	f	0-12 years	0.727	854	109	Grand Index
50	f	0-12 years	0.728	854.824	109	Grand Index
50	f	0-12 years	0.732	855	109	Grand Index
50	f	0-12 years	0.733	855.0009036	109	Grand Index
50	f	0-12 years	0.734	855.1253193	109	Grand Index
50	f	0-12 years	0.736	856	109	Grand Index
50	f	0-12 years	0.737	856.771	110	Grand Index
50	f	0-12 years	0.741	857	110	Grand Index
50	f	0-12 years	0.742	857.0955178	110	Grand Index
50	f	0-12 years	0.743	857.535955	110	Grand Index
50	f	0-12 years	0.744	857.8228478	110	Grand Index
50	f	0-12 years	0.745	857.9733431	110	Grand Index
50	f	0-12 years	0.746	858.718	110	Grand Index
50	f	0-12 years	0.748	859	110	Grand Index
50	f	0-12 years	0.749	859.367	110	Grand Index
50	f	0-12 years	0.75	860	110	Grand Index
50	f	0-12 years	0.751	860.133	110	Grand Index
50	f	0-12 years	0.757	861	110	Grand Index
50	f	0-12 years	0.758	861.314	110	Grand Index
50	f	0-12 years	0.762	862	111	Grand Index
50	f	0-12 years	0.763	862.4509083	111	Grand Index
50	f	0-12 years	0.764	862.8519896	111	Grand Index
50	f	0-12 years	0.767	863	111	Grand Index
50	f	0-12 years	0.768	863.144	111	Grand Index
50	f	0-12 years	0.772	864	111	Grand Index
50	f	0-12 years	0.773	864.0267884	111	Grand Index
50	f	0-12 years	0.774	864.2139657	111	Grand Index
50	f	0-12 years	0.775	864.6109208	111	Grand Index
50	f	0-12 years	0.779	865	112	Grand Index
50	f	0-12 years	0.78	865.9572936	112	Grand Index
50	f	0-12 years	0.781	866.7337023	112	Grand Index
50	f	0-12 years	0.782	867	112	Grand Index
50	f	0-12 years	0.783	867.389	112	Grand Index
50	f	0-12 years	0.787	868	112	Grand Index
50	f	0-12 years	0.788	869.608	112	Grand Index

Age	Gender	Education	Percentile	Raw Score	Norm Score	Assessment
50	f	0-12 years	0.789	870.687	112	Grand Index
50	f	0-12 years	0.795	871	112	Grand Index
50	f	0-12 years	0.796	871.868	112	Grand Index
50	f	0-12 years	0.798	872	113	Grand Index
50	f	0-12 years	0.799	872.0158314	113	Grand Index
50	f	0-12 years	0.8	872.418373	113	Grand Index
50	f	0-12 years	0.801	873.283	113	Grand Index
50	f	0-12 years	0.805	874	113	Grand Index
50	f	0-12 years	0.806	874.1703547	113	Grand Index
50	f	0-12 years	0.807	874.6832616	113	Grand Index
50	f	0-12 years	0.812	875	113	Grand Index
50	f	0-12 years	0.813	875.879	113	Grand Index
50	f	0-12 years	0.814	876	113	Grand Index
50	f	0-12 years	0.815	876.645	113	Grand Index
50	f	0-12 years	0.817	877	114	Grand Index
50	f	0-12 years	0.818	877.294	114	Grand Index
50	f	0-12 years	0.823	878	114	Grand Index
50	f	0-12 years	0.824	878.3120162	114	Grand Index
50	f	0-12 years	0.825	878.6197237	114	Grand Index
50	f	0-12 years	0.826	878.8216188	114	Grand Index
50	f	0-12 years	0.827	879.241	114	Grand Index
50	f	0-12 years	0.832	880	114	Grand Index
50	f	0-12 years	0.833	880.0187885	114	Grand Index
50	f	0-12 years	0.834	880.442148	115	Grand Index
50	f	0-12 years	0.838	881	115	Grand Index
50	f	0-12 years	0.839	881.837	115	Grand Index
50	f	0-12 years	0.843	882	115	Grand Index
50	f	0-12 years	0.844	882.504	115	Grand Index
50	f	0-12 years	0.847	884	115	Grand Index
50	f	0-12 years	0.848	884.784	115	Grand Index
50	f	0-12 years	0.851	885	116	Grand Index
50	f	0-12 years	0.852	885.0037809	116	Grand Index
50	f	0-12 years	0.853	885.2085838	116	Grand Index
50	f	0-12 years	0.859	886	116	Grand Index
50	f	0-12 years	0.86	886.342036	116	Grand Index
50	f	0-12 years	0.861	886.9263697	116	Grand Index
50	f	0-12 years	0.864	887	116	Grand Index
50	f	0-12 years	0.865	887.795	117	Grand Index
50	f	0-12 years	0.867	888	117	Grand Index
50	f	0-12 years	0.868	888.444	117	Grand Index
50	f	0-12 years	0.869	889.327	117	Grand Index
50	f	0-12 years	0.87	890	117	Grand Index
50	f	0-12 years	0.871	890.186	117	Grand Index
50	f	0-12 years	0.872	891.952	117	Grand Index
50	f	0-12 years	0.873	892.859	117	Grand Index
50	f	0-12 years	0.876	893	117	Grand Index
50	f	0-12 years	0.877	893.391	117	Grand Index
50	f	0-12 years	0.878	894	117	Grand Index
50	f	0-12 years	0.879	894.157	118	Grand Index
50	f	0-12 years	0.882	895	118	Grand Index
50	f	0-12 years	0.883	895.689	118	Grand Index
50	f	0-12 years	0.884	896	118	Grand Index
50	f	0-12 years	0.885	896.455	118	Grand Index
50	f	0-12 years	0.886	897	118	Grand Index
50	f	0-12 years	0.887	897.221	118	Grand Index
50	f	0-12 years	0.891	898	118	Grand Index

Age	Gender	Education	Percentile	Raw Score	Norm Score	Assessment
50	f	0-12 years	0.892	898.636	119	Grand Index
50	f	0-12 years	0.893	899	119	Grand Index
50	f	0-12 years	0.894	899.402	119	Grand Index
50	f	0-12 years	0.896	900	119	Grand Index
50	f	0-12 years	0.897	900.0437384	119	Grand Index
50	f	0-12 years	0.898	900.8010127	119	Grand Index
50	f	0-12 years	0.899	900.9739436	119	Grand Index
50	f	0-12 years	0.9	901	119	Grand Index
50	f	0-12 years	0.901	901.583	119	Grand Index
50	f	0-12 years	0.902	902.466	119	Grand Index
50	f	0-12 years	0.904	903	120	Grand Index
50	f	0-12 years	0.905	903.23	120	Grand Index
50	f	0-12 years	0.906	904.996	120	Grand Index
50	f	0-12 years	0.907	905.0284113	120	Grand Index
50	f	0-12 years	0.908	906.5356107	120	Grand Index
50	f	0-12 years	0.909	907	120	Grand Index
50	f	0-12 years	0.91	907.9506709	120	Grand Index
50	f	0-12 years	0.911	908.8789129	120	Grand Index
50	f	0-12 years	0.912	909	120	Grand Index
50	f	0-12 years	0.913	909.179	120	Grand Index
50	f	0-12 years	0.914	910.107482	120	Grand Index
50	f	0-12 years	0.915	911.6382333	121	Grand Index
50	f	0-12 years	0.916	911.9541758	121	Grand Index
50	f	0-12 years	0.917	912	121	Grand Index
50	f	0-12 years	0.918	912.594	121	Grand Index
50	f	0-12 years	0.919	913.1150202	121	Grand Index
50	f	0-12 years	0.92	913.5143248	121	Grand Index
50	f	0-12 years	0.921	914.243	121	Grand Index
50	f	0-12 years	0.922	915.126	121	Grand Index
50	f	0-12 years	0.923	916.009	121	Grand Index
50	f	0-12 years	0.924	916.892	121	Grand Index
50	f	0-12 years	0.925	917.775	122	Grand Index
50	f	0-12 years	0.926	919.0048828	122	Grand Index
50	f	0-12 years	0.927	919.7829745	122	Grand Index
50	f	0-12 years	0.928	920.424	122	Grand Index
50	f	0-12 years	0.929	921.307	122	Grand Index
50	f	0-12 years	0.933	922	122	Grand Index
50	f	0-12 years	0.934	922.0949175	123	Grand Index
50	f	0-12 years	0.935	922.6569285	123	Grand Index
50	f	0-12 years	0.936	923.488	123	Grand Index
50	f	0-12 years	0.937	924.3404498	123	Grand Index
50	f	0-12 years	0.938	925.0877849	123	Grand Index
50	f	0-12 years	0.939	925.5895188	123	Grand Index
50	f	0-12 years	0.94	925.6304417	123	Grand Index
50	f	0-12 years	0.941	926.8644417	123	Grand Index
50	f	0-12 years	0.942	928.572	124	Grand Index
50	f	0-12 years	0.943	929.4984064	124	Grand Index
50	f	0-12 years	0.944	930.4377609	124	Grand Index
50	f	0-12 years	0.945	931	124	Grand Index
50	f	0-12 years	0.946	931.0105526	124	Grand Index
50	f	0-12 years	0.947	931.2275143	124	Grand Index
50	f	0-12 years	0.948	932.084	124	Grand Index
50	f	0-12 years	0.949	932.967	125	Grand Index
50	f	0-12 years	0.95	933.85	125	Grand Index
50	f	0-12 years	0.951	934.0126415	125	Grand Index
50	f	0-12 years	0.952	934.2537591	125	Grand Index

Age	Gender	Education	Percentile	Raw Score	Norm Score	Assessment
50	f	0-12 years	0.953	934.4186352	125	Grand Index
50	f	0-12 years	0.954	935.0765027	125	Grand Index
50	f	0-12 years	0.955	936.500912	125	Grand Index
50	f	0-12 years	0.956	937.6406911	126	Grand Index
50	f	0-12 years	0.957	938.0670233	126	Grand Index
50	f	0-12 years	0.958	939.9761056	126	Grand Index
50	f	0-12 years	0.959	940.5972781	126	Grand Index
50	f	0-12 years	0.96	942.2666032	126	Grand Index
50	f	0-12 years	0.961	943	126	Grand Index
50	f	0-12 years	0.962	943.446	127	Grand Index
50	f	0-12 years	0.963	944	127	Grand Index
50	f	0-12 years	0.964	944.212	127	Grand Index
50	f	0-12 years	0.966	945	127	Grand Index
50	f	0-12 years	0.967	945.861	128	Grand Index
50	f	0-12 years	0.968	946	128	Grand Index
50	f	0-12 years	0.969	946.5263106	128	Grand Index
50	f	0-12 years	0.97	947.3321521	128	Grand Index
50	f	0-12 years	0.971	947.881981	128	Grand Index
50	f	0-12 years	0.972	948.0028954	129	Grand Index
50	f	0-12 years	0.973	948.5715703	129	Grand Index
50	f	0-12 years	0.974	951.6426434	129	Grand Index
50	f	0-12 years	0.975	953.815447	129	Grand Index
50	f	0-12 years	0.976	958.04	130	Grand Index
50	f	0-12 years	0.977	959.3045831	130	Grand Index
50	f	0-12 years	0.978	961.4837748	130	Grand Index
50	f	0-12 years	0.979	963.0996115	131	Grand Index
50	f	0-12 years	0.98	963.483859	131	Grand Index
50	f	0-12 years	0.981	964.223	131	Grand Index
50	f	0-12 years	0.982	965.1029908	131	Grand Index
50	f	0-12 years	0.983	965.960924	132	Grand Index
50	f	0-12 years	0.984	965.9963663	132	Grand Index
50	f	0-12 years	0.985	973.55	133	Grand Index
50	f	0-12 years	0.986	985.4350406	133	Grand Index
50	f	0-12 years	0.987	991.7928872	133	Grand Index
50	f	0-12 years	0.988	992.8754619	134	Grand Index
50	f	0-12 years	0.989	993.5344934	134	Grand Index
50	f	0-12 years	0.99	995.5085	135	Grand Index
50	f	0-12 years	0.991	1000.053	135	Grand Index
50	f	0-12 years	0.992	1000.936	136	Grand Index
50	f	0-12 years	0.993	1004.60278	137	Grand Index
50	f	0-12 years	0.994	1005.820902	138	Grand Index
50	f	0-12 years	0.995	1008.577392	139	Grand Index
50	f	0-12 years	0.996	1010.915923	140	Grand Index
50	f	0-12 years	0.997	1012.374779	141	Grand Index
50	f	0-12 years	0.998	1014.234	143	Grand Index
50	f	0-12 years	0.999	1018.042	146	Grand Index
50	m	0-12 years	0.001	607.9861019	54	Grand Index
50	m	0-12 years	0.002	616.8105859	57	Grand Index
50	m	0-12 years	0.003	617.5212268	59	Grand Index
50	m	0-12 years	0.004	617.9591335	60	Grand Index
50	m	0-12 years	0.005	625.8133122	61	Grand Index
50	m	0-12 years	0.006	634.1834055	62	Grand Index
50	m	0-12 years	0.007	639.0052331	63	Grand Index
50	m	0-12 years	0.008	643.4434644	64	Grand Index
50	m	0-12 years	0.009	646.1518949	65	Grand Index
50	m	0-12 years	0.01	648.6016636	65	Grand Index

Age	Gender	Education	Percentile	Raw Score	Norm Score	Assessment
50	m	0-12 years	0.011	649.824	66	Grand Index
50	m	0-12 years	0.012	650.808	66	Grand Index
50	m	0-12 years	0.013	652.98	67	Grand Index
50	m	0-12 years	0.014	655.44	67	Grand Index
50	m	0-12 years	0.016	656	68	Grand Index
50	m	0-12 years	0.017	658.548	68	Grand Index
50	m	0-12 years	0.018	661.992	69	Grand Index
50	m	0-12 years	0.019	663.696	69	Grand Index
50	m	0-12 years	0.02	664.68	69	Grand Index
50	m	0-12 years	0.021	665.664	69	Grand Index
50	m	0-12 years	0.022	666.648	70	Grand Index
50	m	0-12 years	0.023	667.316	70	Grand Index
50	m	0-12 years	0.024	667.808	70	Grand Index
50	m	0-12 years	0.025	668.2452282	71	Grand Index
50	m	0-12 years	0.026	668.6474023	71	Grand Index
50	m	0-12 years	0.027	669.4372779	71	Grand Index
50	m	0-12 years	0.028	670.5111037	71	Grand Index
50	m	0-12 years	0.029	671.536	72	Grand Index
50	m	0-12 years	0.03	672.52	72	Grand Index
50	m	0-12 years	0.031	673.3068571	72	Grand Index
50	m	0-12 years	0.032	673.9059592	72	Grand Index
50	m	0-12 years	0.033	674.6383129	72	Grand Index
50	m	0-12 years	0.034	675.5152109	73	Grand Index
50	m	0-12 years	0.035	677.3128638	73	Grand Index
50	m	0-12 years	0.036	680.2489045	73	Grand Index
50	m	0-12 years	0.037	682.1781798	73	Grand Index
50	m	0-12 years	0.038	682.686139	73	Grand Index
50	m	0-12 years	0.039	683.564	74	Grand Index
50	m	0-12 years	0.04	685.04	74	Grand Index
50	m	0-12 years	0.041	686.172	74	Grand Index
50	m	0-12 years	0.042	686.664	74	Grand Index
50	m	0-12 years	0.043	687.468	74	Grand Index
50	m	0-12 years	0.044	688.944	74	Grand Index
50	m	0-12 years	0.046	690	75	Grand Index
50	m	0-12 years	0.047	690.1580556	75	Grand Index
50	m	0-12 years	0.048	690.7851795	75	Grand Index
50	m	0-12 years	0.049	691.3529808	75	Grand Index
50	m	0-12 years	0.05	691.7098568	75	Grand Index
50	m	0-12 years	0.052	692	76	Grand Index
50	m	0-12 years	0.053	692.0627482	76	Grand Index
50	m	0-12 years	0.054	692.46896	76	Grand Index
50	m	0-12 years	0.055	692.8992606	76	Grand Index
50	m	0-12 years	0.056	693.5029997	76	Grand Index
50	m	0-12 years	0.057	694.182425	76	Grand Index
50	m	0-12 years	0.058	695.6324741	76	Grand Index
50	m	0-12 years	0.059	697.0346096	77	Grand Index
50	m	0-12 years	0.06	697.6427499	77	Grand Index
50	m	0-12 years	0.061	698.2692248	77	Grand Index
50	m	0-12 years	0.062	699.6290845	77	Grand Index
50	m	0-12 years	0.063	700.9889442	77	Grand Index
50	m	0-12 years	0.064	701.488	77	Grand Index
50	m	0-12 years	0.065	701.98	77	Grand Index
50	m	0-12 years	0.066	702.472	77	Grand Index
50	m	0-12 years	0.067	702.964	78	Grand Index
50	m	0-12 years	0.068	703.0192742	78	Grand Index
50	m	0-12 years	0.069	703.04007	78	Grand Index

Age	Gender	Education	Percentile	Raw Score	Norm Score	Assessment
50	m	0-12 years	0.07	703.4636701	78	Grand Index
50	m	0-12 years	0.071	703.9348742	78	Grand Index
50	m	0-12 years	0.073	704	78	Grand Index
50	m	0-12 years	0.074	704.408	78	Grand Index
50	m	0-12 years	0.075	704.9	78	Grand Index
50	m	0-12 years	0.076	706.1961026	79	Grand Index
50	m	0-12 years	0.077	707.6973333	79	Grand Index
50	m	0-12 years	0.078	708.7668734	79	Grand Index
50	m	0-12 years	0.079	709.7032323	79	Grand Index
50	m	0-12 years	0.08	709.9708484	79	Grand Index
50	m	0-12 years	0.081	709.9932587	79	Grand Index
50	m	0-12 years	0.082	710.344	79	Grand Index
50	m	0-12 years	0.083	710.836	79	Grand Index
50	m	0-12 years	0.084	711.2796899	79	Grand Index
50	m	0-12 years	0.085	711.6992248	79	Grand Index
50	m	0-12 years	0.086	712.1800982	80	Grand Index
50	m	0-12 years	0.087	712.6963592	80	Grand Index
50	m	0-12 years	0.088	712.931025	80	Grand Index
50	m	0-12 years	0.089	712.9792291	80	Grand Index
50	m	0-12 years	0.093	713	80	Grand Index
50	m	0-12 years	0.094	714.1871519	80	Grand Index
50	m	0-12 years	0.095	716.5423082	80	Grand Index
50	m	0-12 years	0.096	717.8363415	80	Grand Index
50	m	0-12 years	0.097	717.9411852	81	Grand Index
50	m	0-12 years	0.099	718	81	Grand Index
50	m	0-12 years	0.1	718.0985245	81	Grand Index
50	m	0-12 years	0.101	718.3408948	81	Grand Index
50	m	0-12 years	0.102	718.58598	81	Grand Index
50	m	0-12 years	0.103	718.8356097	81	Grand Index
50	m	0-12 years	0.105	719	81	Grand Index
50	m	0-12 years	0.106	719.0039113	81	Grand Index
50	m	0-12 years	0.107	719.0165714	81	Grand Index
50	m	0-12 years	0.108	719.1582324	81	Grand Index
50	m	0-12 years	0.109	719.6375723	82	Grand Index
50	m	0-12 years	0.113	720	82	Grand Index
50	m	0-12 years	0.114	720.088	82	Grand Index
50	m	0-12 years	0.115	720.58	82	Grand Index
50	m	0-12 years	0.116	721.072	82	Grand Index
50	m	0-12 years	0.117	721.564	82	Grand Index
50	m	0-12 years	0.118	722.056	82	Grand Index
50	m	0-12 years	0.119	722.548	82	Grand Index
50	m	0-12 years	0.123	723	83	Grand Index
50	m	0-12 years	0.124	723.016	83	Grand Index
50	m	0-12 years	0.125	724	83	Grand Index
50	m	0-12 years	0.126	724.984	83	Grand Index
50	m	0-12 years	0.127	725.0739657	83	Grand Index
50	m	0-12 years	0.128	725.1491539	83	Grand Index
50	m	0-12 years	0.129	725.4248933	83	Grand Index
50	m	0-12 years	0.13	725.7109173	83	Grand Index
50	m	0-12 years	0.131	725.8543259	83	Grand Index
50	m	0-12 years	0.132	725.9851136	83	Grand Index
50	m	0-12 years	0.134	726	83	Grand Index
50	m	0-12 years	0.135	726.42	83	Grand Index
50	m	0-12 years	0.136	726.912	84	Grand Index
50	m	0-12 years	0.137	727.404	84	Grand Index
50	m	0-12 years	0.138	727.896	84	Grand Index

Age	Gender	Education	Percentile	Raw Score	Norm Score	Assessment
50	m	0-12 years	0.139	728.388	84	Grand Index
50	m	0-12 years	0.14	728.88	84	Grand Index
50	m	0-12 years	0.141	729.181007	84	Grand Index
50	m	0-12 years	0.142	729.4204033	84	Grand Index
50	m	0-12 years	0.143	729.6175325	84	Grand Index
50	m	0-12 years	0.144	729.7985147	84	Grand Index
50	m	0-12 years	0.145	729.9039224	84	Grand Index
50	m	0-12 years	0.146	729.9755439	84	Grand Index
50	m	0-12 years	0.147	730.324	84	Grand Index
50	m	0-12 years	0.148	730.816	84	Grand Index
50	m	0-12 years	0.149	731.1102973	84	Grand Index
50	m	0-12 years	0.15	731.2864865	84	Grand Index
50	m	0-12 years	0.151	731.5455406	85	Grand Index
50	m	0-12 years	0.152	731.8613514	85	Grand Index
50	m	0-12 years	0.153	732.276	85	Grand Index
50	m	0-12 years	0.154	732.768	85	Grand Index
50	m	0-12 years	0.158	733	85	Grand Index
50	m	0-12 years	0.159	733.456	85	Grand Index
50	m	0-12 years	0.16	734.44	85	Grand Index
50	m	0-12 years	0.162	735	85	Grand Index
50	m	0-12 years	0.163	735.588	85	Grand Index
50	m	0-12 years	0.164	737.064	85	Grand Index
50	m	0-12 years	0.165	738.36	85	Grand Index
50	m	0-12 years	0.166	739.344	85	Grand Index
50	m	0-12 years	0.167	740.2948813	86	Grand Index
50	m	0-12 years	0.168	741.1795252	86	Grand Index
50	m	0-12 years	0.169	741.835327	86	Grand Index
50	m	0-12 years	0.17	741.9592255	86	Grand Index
50	m	0-12 years	0.171	742.1752984	86	Grand Index
50	m	0-12 years	0.172	742.642756	86	Grand Index
50	m	0-12 years	0.178	743	86	Grand Index
50	m	0-12 years	0.179	743.0677872	86	Grand Index
50	m	0-12 years	0.18	743.5582473	86	Grand Index
50	m	0-12 years	0.181	743.9970329	86	Grand Index
50	m	0-12 years	0.182	743.9985728	86	Grand Index
50	m	0-12 years	0.191	744	87	Grand Index
50	m	0-12 years	0.192	744.928	87	Grand Index
50	m	0-12 years	0.193	745.912	87	Grand Index
50	m	0-12 years	0.197	746	87	Grand Index
50	m	0-12 years	0.198	746.3815255	87	Grand Index
50	m	0-12 years	0.199	746.8327528	87	Grand Index
50	m	0-12 years	0.2	746.9502772	87	Grand Index
50	m	0-12 years	0.201	746.9910499	87	Grand Index
50	m	0-12 years	0.203	747	88	Grand Index
50	m	0-12 years	0.204	747.736	88	Grand Index
50	m	0-12 years	0.205	748.72	88	Grand Index
50	m	0-12 years	0.207	749	88	Grand Index
50	m	0-12 years	0.208	749.2261064	88	Grand Index
50	m	0-12 years	0.209	749.5571908	88	Grand Index
50	m	0-12 years	0.21	749.7775963	88	Grand Index
50	m	0-12 years	0.211	749.9385119	88	Grand Index
50	m	0-12 years	0.212	750.304	88	Grand Index
50	m	0-12 years	0.213	750.796	88	Grand Index
50	m	0-12 years	0.215	751	88	Grand Index
50	m	0-12 years	0.216	751.272	88	Grand Index
50	m	0-12 years	0.217	751.764	88	Grand Index

Age	Gender	Education	Percentile	Raw Score	Norm Score	Assessment
50	m	0-12 years	0.218	752.256	88	Grand Index
50	m	0-12 years	0.219	752.748	88	Grand Index
50	m	0-12 years	0.223	753	89	Grand Index
50	m	0-12 years	0.224	753.208	89	Grand Index
50	m	0-12 years	0.225	753.7	89	Grand Index
50	m	0-12 years	0.226	754.3307349	89	Grand Index
50	m	0-12 years	0.227	755.1782432	89	Grand Index
50	m	0-12 years	0.228	755.9474041	89	Grand Index
50	m	0-12 years	0.229	756.5758958	89	Grand Index
50	m	0-12 years	0.233	757	89	Grand Index
50	m	0-12 years	0.234	757.256	89	Grand Index
50	m	0-12 years	0.235	758.24	89	Grand Index
50	m	0-12 years	0.237	759	89	Grand Index
50	m	0-12 years	0.238	759.096	89	Grand Index
50	m	0-12 years	0.239	759.588	89	Grand Index
50	m	0-12 years	0.243	760	90	Grand Index
50	m	0-12 years	0.244	760.048	90	Grand Index
50	m	0-12 years	0.245	760.54	90	Grand Index
50	m	0-12 years	0.247	761	90	Grand Index
50	m	0-12 years	0.248	761.032	90	Grand Index
50	m	0-12 years	0.249	762.016	90	Grand Index
50	m	0-12 years	0.25	763	90	Grand Index
50	m	0-12 years	0.251	763.984	90	Grand Index
50	m	0-12 years	0.252	764.968	90	Grand Index
50	m	0-12 years	0.256	765	90	Grand Index
50	m	0-12 years	0.257	765.0487533	90	Grand Index
50	m	0-12 years	0.258	765.1027772	90	Grand Index
50	m	0-12 years	0.259	765.4908083	90	Grand Index
50	m	0-12 years	0.26	765.9287844	90	Grand Index
50	m	0-12 years	0.261	766.341947	90	Grand Index
50	m	0-12 years	0.262	766.7502916	90	Grand Index
50	m	0-12 years	0.263	766.8964179	90	Grand Index
50	m	0-12 years	0.264	766.9789762	91	Grand Index
50	m	0-12 years	0.265	766.9986174	91	Grand Index
50	m	0-12 years	0.266	766.9997146	91	Grand Index
50	m	0-12 years	0.272	767	91	Grand Index
50	m	0-12 years	0.273	767.316	91	Grand Index
50	m	0-12 years	0.274	767.808	91	Grand Index
50	m	0-12 years	0.276	768	91	Grand Index
50	m	0-12 years	0.277	768.284	91	Grand Index
50	m	0-12 years	0.278	768.776	91	Grand Index
50	m	0-12 years	0.28	769	91	Grand Index
50	m	0-12 years	0.281	769.252	91	Grand Index
50	m	0-12 years	0.282	769.744	91	Grand Index
50	m	0-12 years	0.284	770	91	Grand Index
50	m	0-12 years	0.285	770.22	91	Grand Index
50	m	0-12 years	0.286	770.712	92	Grand Index
50	m	0-12 years	0.29	771	92	Grand Index
50	m	0-12 years	0.291	771.011187	92	Grand Index
50	m	0-12 years	0.292	771.043187	92	Grand Index
50	m	0-12 years	0.293	771.2108943	92	Grand Index
50	m	0-12 years	0.294	771.6708943	92	Grand Index
50	m	0-12 years	0.296	772	92	Grand Index
50	m	0-12 years	0.297	772.124	92	Grand Index
50	m	0-12 years	0.298	772.616	92	Grand Index
50	m	0-12 years	0.3	773	92	Grand Index

Age	Gender	Education	Percentile	Raw Score	Norm Score	Assessment
50	m	0-12 years	0.301	773.092	92	Grand Index
50	m	0-12 years	0.302	773.584	92	Grand Index
50	m	0-12 years	0.303	774.076	92	Grand Index
50	m	0-12 years	0.304	774.568	92	Grand Index
50	m	0-12 years	0.305	775.06	92	Grand Index
50	m	0-12 years	0.306	775.552	92	Grand Index
50	m	0-12 years	0.308	776	92	Grand Index
50	m	0-12 years	0.309	776.028	93	Grand Index
50	m	0-12 years	0.31	776.52	93	Grand Index
50	m	0-12 years	0.313	777	93	Grand Index
50	m	0-12 years	0.314	777.488	93	Grand Index
50	m	0-12 years	0.315	777.98	93	Grand Index
50	m	0-12 years	0.317	778	93	Grand Index
50	m	0-12 years	0.318	778.456	93	Grand Index
50	m	0-12 years	0.319	778.948	93	Grand Index
50	m	0-12 years	0.323	779	93	Grand Index
50	m	0-12 years	0.324	779.7052518	93	Grand Index
50	m	0-12 years	0.325	780.5557024	93	Grand Index
50	m	0-12 years	0.326	780.8349634	93	Grand Index
50	m	0-12 years	0.327	780.9685128	93	Grand Index
50	m	0-12 years	0.333	781	94	Grand Index
50	m	0-12 years	0.334	781.328	94	Grand Index
50	m	0-12 years	0.335	781.82	94	Grand Index
50	m	0-12 years	0.336	782.2668573	94	Grand Index
50	m	0-12 years	0.337	782.6876707	94	Grand Index
50	m	0-12 years	0.338	782.8981396	94	Grand Index
50	m	0-12 years	0.339	782.9693261	94	Grand Index
50	m	0-12 years	0.343	783	94	Grand Index
50	m	0-12 years	0.344	783.0075606	94	Grand Index
50	m	0-12 years	0.345	783.0225598	94	Grand Index
50	m	0-12 years	0.346	783.2554134	94	Grand Index
50	m	0-12 years	0.347	783.7324142	94	Grand Index
50	m	0-12 years	0.348	784.216	94	Grand Index
50	m	0-12 years	0.349	784.708	94	Grand Index
50	m	0-12 years	0.35	785.2	94	Grand Index
50	m	0-12 years	0.351	785.692	94	Grand Index
50	m	0-12 years	0.355	786	94	Grand Index
50	m	0-12 years	0.356	786.152	94	Grand Index
50	m	0-12 years	0.357	786.644	95	Grand Index
50	m	0-12 years	0.359	787	95	Grand Index
50	m	0-12 years	0.36	787.12	95	Grand Index
50	m	0-12 years	0.361	787.612	95	Grand Index
50	m	0-12 years	0.369	788	95	Grand Index
50	m	0-12 years	0.37	788.04	95	Grand Index
50	m	0-12 years	0.371	788.532	95	Grand Index
50	m	0-12 years	0.384	789	96	Grand Index
50	m	0-12 years	0.385	789.42	96	Grand Index
50	m	0-12 years	0.386	789.912	96	Grand Index
50	m	0-12 years	0.392	790	96	Grand Index
50	m	0-12 years	0.393	790.356	96	Grand Index
50	m	0-12 years	0.394	790.848	96	Grand Index
50	m	0-12 years	0.4	791	96	Grand Index
50	m	0-12 years	0.401	791.292	96	Grand Index
50	m	0-12 years	0.402	791.784	96	Grand Index
50	m	0-12 years	0.408	792	97	Grand Index
50	m	0-12 years	0.409	792.228	97	Grand Index

Age	Gender	Education	Percentile	Raw Score	Norm Score	Assessment
50	m	0-12 years	0.41	792.72	97	Grand Index
50	m	0-12 years	0.416	793	97	Grand Index
50	m	0-12 years	0.417	793.164	97	Grand Index
50	m	0-12 years	0.418	793.656	97	Grand Index
50	m	0-12 years	0.419	794.148	97	Grand Index
50	m	0-12 years	0.42	794.64	97	Grand Index
50	m	0-12 years	0.422	795	97	Grand Index
50	m	0-12 years	0.423	795.116	97	Grand Index
50	m	0-12 years	0.424	795.608	97	Grand Index
50	m	0-12 years	0.426	796	97	Grand Index
50	m	0-12 years	0.427	796.084	97	Grand Index
50	m	0-12 years	0.428	796.576	97	Grand Index
50	m	0-12 years	0.434	797	98	Grand Index
50	m	0-12 years	0.435	797.0013555	98	Grand Index
50	m	0-12 years	0.436	797.0347007	98	Grand Index
50	m	0-12 years	0.437	797.0715036	98	Grand Index
50	m	0-12 years	0.438	797.5301585	98	Grand Index
50	m	0-12 years	0.439	797.9888133	98	Grand Index
50	m	0-12 years	0.441	798	98	Grand Index
50	m	0-12 years	0.442	798.464	98	Grand Index
50	m	0-12 years	0.443	798.956	98	Grand Index
50	m	0-12 years	0.449	799	98	Grand Index
50	m	0-12 years	0.45	799.4	98	Grand Index
50	m	0-12 years	0.451	799.892	98	Grand Index
50	m	0-12 years	0.455	800	98	Grand Index
50	m	0-12 years	0.456	800.352	98	Grand Index
50	m	0-12 years	0.457	800.844	98	Grand Index
50	m	0-12 years	0.463	801	99	Grand Index
50	m	0-12 years	0.464	801.288	99	Grand Index
50	m	0-12 years	0.465	801.78	99	Grand Index
50	m	0-12 years	0.471	802	99	Grand Index
50	m	0-12 years	0.472	802.224	99	Grand Index
50	m	0-12 years	0.473	802.716	99	Grand Index
50	m	0-12 years	0.474	803.208	99	Grand Index
50	m	0-12 years	0.475	803.7	99	Grand Index
50	m	0-12 years	0.483	804	99	Grand Index
50	m	0-12 years	0.484	804.128	99	Grand Index
50	m	0-12 years	0.485	804.62	99	Grand Index
50	m	0-12 years	0.493	805	100	Grand Index
50	m	0-12 years	0.494	805.048	100	Grand Index
50	m	0-12 years	0.495	805.54	100	Grand Index
50	m	0-12 years	0.5	806	100	Grand Index
50	m	0-12 years	0.501	806.492	100	Grand Index
50	m	0-12 years	0.502	806.984	100	Grand Index
50	m	0-12 years	0.503	807.952	100	Grand Index
50	m	0-12 years	0.504	808.936	100	Grand Index
50	m	0-12 years	0.508	809	100	Grand Index
50	m	0-12 years	0.509	809.428	100	Grand Index
50	m	0-12 years	0.51	809.92	100	Grand Index
50	m	0-12 years	0.511	810.412	100	Grand Index
50	m	0-12 years	0.512	810.904	100	Grand Index
50	m	0-12 years	0.514	811	101	Grand Index
50	m	0-12 years	0.515	811.38	101	Grand Index
50	m	0-12 years	0.516	811.872	101	Grand Index
50	m	0-12 years	0.518	812	101	Grand Index
50	m	0-12 years	0.519	812.348	101	Grand Index

Age	Gender	Education	Percentile	Raw Score	Norm Score	Assessment
50	m	0-12 years	0.52	812.84	101	Grand Index
50	m	0-12 years	0.521	813.332	101	Grand Index
50	m	0-12 years	0.522	813.824	101	Grand Index
50	m	0-12 years	0.523	814.316	101	Grand Index
50	m	0-12 years	0.524	814.808	101	Grand Index
50	m	0-12 years	0.525	815.3	101	Grand Index
50	m	0-12 years	0.526	815.792	101	Grand Index
50	m	0-12 years	0.527	816.284	101	Grand Index
50	m	0-12 years	0.528	816.776	101	Grand Index
50	m	0-12 years	0.534	817	101	Grand Index
50	m	0-12 years	0.535	817.22	101	Grand Index
50	m	0-12 years	0.536	817.712	101	Grand Index
50	m	0-12 years	0.54	818	102	Grand Index
50	m	0-12 years	0.541	818.172	102	Grand Index
50	m	0-12 years	0.542	818.664	102	Grand Index
50	m	0-12 years	0.544	819	102	Grand Index
50	m	0-12 years	0.545	819.14	102	Grand Index
50	m	0-12 years	0.546	819.632	102	Grand Index
50	m	0-12 years	0.556	820	102	Grand Index
50	m	0-12 years	0.557	820.044	102	Grand Index
50	m	0-12 years	0.558	820.536	102	Grand Index
50	m	0-12 years	0.559	821.0272987	102	Grand Index
50	m	0-12 years	0.56	821.5069767	102	Grand Index
50	m	0-12 years	0.561	821.9872558	102	Grand Index
50	m	0-12 years	0.562	822.4915778	102	Grand Index
50	m	0-12 years	0.563	822.9958998	102	Grand Index
50	m	0-12 years	0.569	823	103	Grand Index
50	m	0-12 years	0.57	823.44	103	Grand Index
50	m	0-12 years	0.571	823.932	103	Grand Index
50	m	0-12 years	0.575	824	103	Grand Index
50	m	0-12 years	0.576	824.392	103	Grand Index
50	m	0-12 years	0.577	824.884	103	Grand Index
50	m	0-12 years	0.579	825	103	Grand Index
50	m	0-12 years	0.58	825.2366641	103	Grand Index
50	m	0-12 years	0.581	825.5601049	103	Grand Index
50	m	0-12 years	0.582	825.7752545	103	Grand Index
50	m	0-12 years	0.583	825.9438136	103	Grand Index
50	m	0-12 years	0.585	826	103	Grand Index
50	m	0-12 years	0.586	826.624	103	Grand Index
50	m	0-12 years	0.587	827.608	103	Grand Index
50	m	0-12 years	0.589	828	103	Grand Index
50	m	0-12 years	0.59	828.1289493	103	Grand Index
50	m	0-12 years	0.591	828.3555318	103	Grand Index
50	m	0-12 years	0.592	828.8260805	103	Grand Index
50	m	0-12 years	0.593	829.5073275	104	Grand Index
50	m	0-12 years	0.594	829.8835766	104	Grand Index
50	m	0-12 years	0.595	829.9597472	104	Grand Index
50	m	0-12 years	0.601	830	104	Grand Index
50	m	0-12 years	0.602	830.184	104	Grand Index
50	m	0-12 years	0.603	830.676	104	Grand Index
50	m	0-12 years	0.613	831	104	Grand Index
50	m	0-12 years	0.614	831.088	104	Grand Index
50	m	0-12 years	0.615	831.58	104	Grand Index
50	m	0-12 years	0.621	832	105	Grand Index
50	m	0-12 years	0.622	832.048	105	Grand Index
50	m	0-12 years	0.623	833.032	105	Grand Index

Age	Gender	Education	Percentile	Raw Score	Norm Score	Assessment
50	m	0-12 years	0.626	834	105	Grand Index
50	m	0-12 years	0.627	834.484	105	Grand Index
50	m	0-12 years	0.628	834.976	105	Grand Index
50	m	0-12 years	0.63	835	105	Grand Index
50	m	0-12 years	0.631	835.452	105	Grand Index
50	m	0-12 years	0.632	835.944	105	Grand Index
50	m	0-12 years	0.633	836.872	105	Grand Index
50	m	0-12 years	0.634	837.856	105	Grand Index
50	m	0-12 years	0.638	838	105	Grand Index
50	m	0-12 years	0.639	838.776	105	Grand Index
50	m	0-12 years	0.64	839.76	105	Grand Index
50	m	0-12 years	0.641	840.2045746	105	Grand Index
50	m	0-12 years	0.642	840.4751409	105	Grand Index
50	m	0-12 years	0.643	840.710156	105	Grand Index
50	m	0-12 years	0.644	840.9315896	106	Grand Index
50	m	0-12 years	0.646	841	106	Grand Index
50	m	0-12 years	0.647	841.2384549	106	Grand Index
50	m	0-12 years	0.648	841.6005532	106	Grand Index
50	m	0-12 years	0.649	841.8172927	106	Grand Index
50	m	0-12 years	0.65	841.9471944	106	Grand Index
50	m	0-12 years	0.651	842.292	106	Grand Index
50	m	0-12 years	0.652	842.784	106	Grand Index
50	m	0-12 years	0.654	843	106	Grand Index
50	m	0-12 years	0.655	843.1576412	106	Grand Index
50	m	0-12 years	0.656	843.4559468	106	Grand Index
50	m	0-12 years	0.657	843.7127131	106	Grand Index
50	m	0-12 years	0.658	843.9272592	106	Grand Index
50	m	0-12 years	0.659	844.2607185	106	Grand Index
50	m	0-12 years	0.66	844.7318668	106	Grand Index
50	m	0-12 years	0.664	845	106	Grand Index
50	m	0-12 years	0.665	845.2334545	106	Grand Index
50	m	0-12 years	0.666	845.8715636	106	Grand Index
50	m	0-12 years	0.667	846.4122667	106	Grand Index
50	m	0-12 years	0.668	846.7581576	107	Grand Index
50	m	0-12 years	0.67	847	107	Grand Index
50	m	0-12 years	0.671	847.132	107	Grand Index
50	m	0-12 years	0.672	847.624	107	Grand Index
50	m	0-12 years	0.674	848	107	Grand Index
50	m	0-12 years	0.675	848.1	107	Grand Index
50	m	0-12 years	0.676	848.592	107	Grand Index
50	m	0-12 years	0.678	849	107	Grand Index
50	m	0-12 years	0.679	849.1337521	107	Grand Index
50	m	0-12 years	0.68	850.1014876	107	Grand Index
50	m	0-12 years	0.681	850.9686612	107	Grand Index
50	m	0-12 years	0.682	850.9849256	107	Grand Index
50	m	0-12 years	0.684	851	107	Grand Index
50	m	0-12 years	0.685	851.02	107	Grand Index
50	m	0-12 years	0.686	851.512	107	Grand Index
50	m	0-12 years	0.687	852.004	107	Grand Index
50	m	0-12 years	0.688	852.496	107	Grand Index
50	m	0-12 years	0.689	852.988	107	Grand Index
50	m	0-12 years	0.693	853	108	Grand Index
50	m	0-12 years	0.694	853.448	108	Grand Index
50	m	0-12 years	0.695	853.94	108	Grand Index
50	m	0-12 years	0.697	854	108	Grand Index
50	m	0-12 years	0.698	854.416	108	Grand Index

Age	Gender	Education	Percentile	Raw Score	Norm Score	Assessment
50	m	0-12 years	0.699	854.908	108	Grand Index
50	m	0-12 years	0.701	855	108	Grand Index
50	m	0-12 years	0.702	855.384	108	Grand Index
50	m	0-12 years	0.703	855.876	108	Grand Index
50	m	0-12 years	0.707	856	108	Grand Index
50	m	0-12 years	0.708	856.336	108	Grand Index
50	m	0-12 years	0.709	856.828	108	Grand Index
50	m	0-12 years	0.713	857	108	Grand Index
50	m	0-12 years	0.714	857.0687637	108	Grand Index
50	m	0-12 years	0.715	857.1862349	109	Grand Index
50	m	0-12 years	0.716	857.4458192	109	Grand Index
50	m	0-12 years	0.717	857.820348	109	Grand Index
50	m	0-12 years	0.719	858	109	Grand Index
50	m	0-12 years	0.72	858.24	109	Grand Index
50	m	0-12 years	0.721	858.732	109	Grand Index
50	m	0-12 years	0.722	859.224	109	Grand Index
50	m	0-12 years	0.723	859.716	109	Grand Index
50	m	0-12 years	0.731	860	109	Grand Index
50	m	0-12 years	0.732	860.144	109	Grand Index
50	m	0-12 years	0.733	860.636	109	Grand Index
50	m	0-12 years	0.737	861	110	Grand Index
50	m	0-12 years	0.738	861.096	110	Grand Index
50	m	0-12 years	0.739	861.588	110	Grand Index
50	m	0-12 years	0.74	862.16	110	Grand Index
50	m	0-12 years	0.741	863.144	110	Grand Index
50	m	0-12 years	0.743	864	110	Grand Index
50	m	0-12 years	0.744	864.048	110	Grand Index
50	m	0-12 years	0.745	864.54	110	Grand Index
50	m	0-12 years	0.75	865	110	Grand Index
50	m	0-12 years	0.751	865.492	110	Grand Index
50	m	0-12 years	0.752	865.984	110	Grand Index
50	m	0-12 years	0.753	866.476	110	Grand Index
50	m	0-12 years	0.754	866.968	110	Grand Index
50	m	0-12 years	0.755	867.46	110	Grand Index
50	m	0-12 years	0.756	867.952	110	Grand Index
50	m	0-12 years	0.757	868.444	110	Grand Index
50	m	0-12 years	0.758	868.936	110	Grand Index
50	m	0-12 years	0.76	869	111	Grand Index
50	m	0-12 years	0.761	869.824	111	Grand Index
50	m	0-12 years	0.762	870.808	111	Grand Index
50	m	0-12 years	0.768	871	111	Grand Index
50	m	0-12 years	0.769	871.2991579	111	Grand Index
50	m	0-12 years	0.77	871.7221053	111	Grand Index
50	m	0-12 years	0.771	871.9062456	111	Grand Index
50	m	0-12 years	0.772	871.9752982	111	Grand Index
50	m	0-12 years	0.784	872	112	Grand Index
50	m	0-12 years	0.785	872.3638462	112	Grand Index
50	m	0-12 years	0.786	873.1775385	112	Grand Index
50	m	0-12 years	0.787	873.7244615	112	Grand Index
50	m	0-12 years	0.788	873.8947692	112	Grand Index
50	m	0-12 years	0.792	874	112	Grand Index
50	m	0-12 years	0.793	874.156	112	Grand Index
50	m	0-12 years	0.794	874.648	112	Grand Index
50	m	0-12 years	0.796	875	112	Grand Index
50	m	0-12 years	0.797	875.248	112	Grand Index
50	m	0-12 years	0.798	876.232	113	Grand Index

Age	Gender	Education	Percentile	Raw Score	Norm Score	Assessment
50	m	0-12 years	0.802	877	113	Grand Index
50	m	0-12 years	0.803	877.015421	113	Grand Index
50	m	0-12 years	0.804	877.1152514	113	Grand Index
50	m	0-12 years	0.805	877.2507329	113	Grand Index
50	m	0-12 years	0.806	877.6429025	113	Grand Index
50	m	0-12 years	0.807	878.044	113	Grand Index
50	m	0-12 years	0.808	878.536	113	Grand Index
50	m	0-12 years	0.809	879.028	113	Grand Index
50	m	0-12 years	0.81	879.52	113	Grand Index
50	m	0-12 years	0.811	880.012	113	Grand Index
50	m	0-12 years	0.812	880.504	113	Grand Index
50	m	0-12 years	0.813	880.996	113	Grand Index
50	m	0-12 years	0.814	881.0110244	113	Grand Index
50	m	0-12 years	0.815	881.0221391	113	Grand Index
50	m	0-12 years	0.816	881.483928	114	Grand Index
50	m	0-12 years	0.817	881.9648133	114	Grand Index
50	m	0-12 years	0.818	882.1742439	114	Grand Index
50	m	0-12 years	0.819	882.3622439	114	Grand Index
50	m	0-12 years	0.82	882.6539837	114	Grand Index
50	m	0-12 years	0.821	882.9579837	114	Grand Index
50	m	0-12 years	0.822	883.424	114	Grand Index
50	m	0-12 years	0.823	883.916	114	Grand Index
50	m	0-12 years	0.825	884	114	Grand Index
50	m	0-12 years	0.826	884.392	114	Grand Index
50	m	0-12 years	0.827	884.884	114	Grand Index
50	m	0-12 years	0.828	885.376	114	Grand Index
50	m	0-12 years	0.829	885.868	114	Grand Index
50	m	0-12 years	0.831	886	114	Grand Index
50	m	0-12 years	0.832	886.1617724	114	Grand Index
50	m	0-12 years	0.833	886.3931447	114	Grand Index
50	m	0-12 years	0.834	886.848386	115	Grand Index
50	m	0-12 years	0.835	887.415562	115	Grand Index
50	m	0-12 years	0.836	887.740669	115	Grand Index
50	m	0-12 years	0.837	887.9261208	115	Grand Index
50	m	0-12 years	0.838	888.296	115	Grand Index
50	m	0-12 years	0.839	888.788	115	Grand Index
50	m	0-12 years	0.845	889	115	Grand Index
50	m	0-12 years	0.846	889.8250596	115	Grand Index
50	m	0-12 years	0.847	891.574755	115	Grand Index
50	m	0-12 years	0.848	892.6521325	115	Grand Index
50	m	0-12 years	0.849	892.8704371	115	Grand Index
50	m	0-12 years	0.85	893.2	116	Grand Index
50	m	0-12 years	0.851	893.692	116	Grand Index
50	m	0-12 years	0.853	894	116	Grand Index
50	m	0-12 years	0.854	894.168	116	Grand Index
50	m	0-12 years	0.855	894.66	116	Grand Index
50	m	0-12 years	0.856	895.110493	116	Grand Index
50	m	0-12 years	0.857	895.4681416	116	Grand Index
50	m	0-12 years	0.858	895.7640657	116	Grand Index
50	m	0-12 years	0.859	895.8984172	116	Grand Index
50	m	0-12 years	0.865	896	117	Grand Index
50	m	0-12 years	0.866	896.0072669	117	Grand Index
50	m	0-12 years	0.867	896.0569239	117	Grand Index
50	m	0-12 years	0.868	896.2072769	117	Grand Index
50	m	0-12 years	0.869	897.1416199	117	Grand Index
50	m	0-12 years	0.87	898.08	117	Grand Index

Age	Gender	Education	Percentile	Raw Score	Norm Score	Assessment
50	m	0-12 years	0.871	899.064	117	Grand Index
50	m	0-12 years	0.872	900.024	117	Grand Index
50	m	0-12 years	0.873	900.516	117	Grand Index
50	m	0-12 years	0.874	901.0048463	117	Grand Index
50	m	0-12 years	0.875	901.3028915	117	Grand Index
50	m	0-12 years	0.876	901.6009367	117	Grand Index
50	m	0-12 years	0.877	902.280584	117	Grand Index
50	m	0-12 years	0.878	902.9665388	117	Grand Index
50	m	0-12 years	0.88	903	118	Grand Index
50	m	0-12 years	0.881	903.452	118	Grand Index
50	m	0-12 years	0.882	903.944	118	Grand Index
50	m	0-12 years	0.884	904	118	Grand Index
50	m	0-12 years	0.885	904.42	118	Grand Index
50	m	0-12 years	0.886	904.912	118	Grand Index
50	m	0-12 years	0.887	905.404	118	Grand Index
50	m	0-12 years	0.888	905.896	118	Grand Index
50	m	0-12 years	0.889	906.388	118	Grand Index
50	m	0-12 years	0.89	906.88	118	Grand Index
50	m	0-12 years	0.892	907	119	Grand Index
50	m	0-12 years	0.893	907.9593995	119	Grand Index
50	m	0-12 years	0.894	909.2853112	119	Grand Index
50	m	0-12 years	0.895	910.138662	119	Grand Index
50	m	0-12 years	0.896	910.7807503	119	Grand Index
50	m	0-12 years	0.897	912.296	119	Grand Index
50	m	0-12 years	0.898	914.264	119	Grand Index
50	m	0-12 years	0.899	915.8009844	119	Grand Index
50	m	0-12 years	0.9	917.0804789	119	Grand Index
50	m	0-12 years	0.901	917.7172238	119	Grand Index
50	m	0-12 years	0.902	917.9137293	119	Grand Index
50	m	0-12 years	0.904	918	120	Grand Index
50	m	0-12 years	0.905	918.78	120	Grand Index
50	m	0-12 years	0.906	920.256	120	Grand Index
50	m	0-12 years	0.907	921.244	120	Grand Index
50	m	0-12 years	0.908	921.736	120	Grand Index
50	m	0-12 years	0.909	922.456	120	Grand Index
50	m	0-12 years	0.91	923.44	120	Grand Index
50	m	0-12 years	0.911	924.424	120	Grand Index
50	m	0-12 years	0.912	925.408	120	Grand Index
50	m	0-12 years	0.913	926.196	120	Grand Index
50	m	0-12 years	0.914	926.688	120	Grand Index
50	m	0-12 years	0.915	927.3042527	121	Grand Index
50	m	0-12 years	0.916	928.1358767	121	Grand Index
50	m	0-12 years	0.917	928.7410847	121	Grand Index
50	m	0-12 years	0.918	928.8934607	121	Grand Index
50	m	0-12 years	0.919	929.148	121	Grand Index
50	m	0-12 years	0.92	929.64	121	Grand Index
50	m	0-12 years	0.921	930.132	121	Grand Index
50	m	0-12 years	0.922	930.624	121	Grand Index
50	m	0-12 years	0.923	931.116	121	Grand Index
50	m	0-12 years	0.924	931.608	121	Grand Index
50	m	0-12 years	0.926	932	122	Grand Index
50	m	0-12 years	0.927	932.168	122	Grand Index
50	m	0-12 years	0.928	933.152	122	Grand Index
50	m	0-12 years	0.929	934.068	122	Grand Index
50	m	0-12 years	0.93	934.56	122	Grand Index
50	m	0-12 years	0.932	935	122	Grand Index

Age	Gender	Education	Percentile	Raw Score	Norm Score	Assessment
50	m	0-12 years	0.933	935.0165581	122	Grand Index
50	m	0-12 years	0.934	935.2428515	123	Grand Index
50	m	0-12 years	0.935	935.4707471	123	Grand Index
50	m	0-12 years	0.936	935.7364536	123	Grand Index
50	m	0-12 years	0.939	936	123	Grand Index
50	m	0-12 years	0.94	936.4764948	123	Grand Index
50	m	0-12 years	0.941	936.9649019	123	Grand Index
50	m	0-12 years	0.942	938.4002237	124	Grand Index
50	m	0-12 years	0.943	939.892687	124	Grand Index
50	m	0-12 years	0.944	940.4812529	124	Grand Index
50	m	0-12 years	0.945	940.9810432	124	Grand Index
50	m	0-12 years	0.946	941.4558522	124	Grand Index
50	m	0-12 years	0.947	941.9271915	124	Grand Index
50	m	0-12 years	0.948	943.1025291	124	Grand Index
50	m	0-12 years	0.949	944.4064817	125	Grand Index
50	m	0-12 years	0.95	944.7901862	125	Grand Index
50	m	0-12 years	0.951	944.9622335	125	Grand Index
50	m	0-12 years	0.953	945	125	Grand Index
50	m	0-12 years	0.954	945.368	125	Grand Index
50	m	0-12 years	0.955	945.86	125	Grand Index
50	m	0-12 years	0.956	948.464	126	Grand Index
50	m	0-12 years	0.957	951.908	126	Grand Index
50	m	0-12 years	0.958	953.336	126	Grand Index
50	m	0-12 years	0.959	953.828	126	Grand Index
50	m	0-12 years	0.96	954.32	126	Grand Index
50	m	0-12 years	0.961	954.812	126	Grand Index
50	m	0-12 years	0.962	955.608	127	Grand Index
50	m	0-12 years	0.963	956.592	127	Grand Index
50	m	0-12 years	0.964	957.864	127	Grand Index
50	m	0-12 years	0.965	959.34	127	Grand Index
50	m	0-12 years	0.966	961.0569769	127	Grand Index
50	m	0-12 years	0.967	962.9688617	128	Grand Index
50	m	0-12 years	0.968	963.9151428	128	Grand Index
50	m	0-12 years	0.969	963.971258	128	Grand Index
50	m	0-12 years	0.971	964	128	Grand Index
50	m	0-12 years	0.972	965.0789728	129	Grand Index
50	m	0-12 years	0.973	967.4488595	129	Grand Index
50	m	0-12 years	0.974	969.2709396	129	Grand Index
50	m	0-12 years	0.975	970.3450529	129	Grand Index
50	m	0-12 years	0.977	971	130	Grand Index
50	m	0-12 years	0.978	971.3359368	130	Grand Index
50	m	0-12 years	0.979	972.2750328	131	Grand Index
50	m	0-12 years	0.98	973.3450054	131	Grand Index
50	m	0-12 years	0.981	974.6865471	131	Grand Index
50	m	0-12 years	0.982	975.6879387	131	Grand Index
50	m	0-12 years	0.983	975.867301	132	Grand Index
50	m	0-12 years	0.984	976.512	132	Grand Index
50	m	0-12 years	0.985	978.48	133	Grand Index
50	m	0-12 years	0.986	980.112	133	Grand Index
50	m	0-12 years	0.987	980.604	133	Grand Index
50	m	0-12 years	0.988	981.1836996	134	Grand Index
50	m	0-12 years	0.989	982.1251599	134	Grand Index
50	m	0-12 years	0.99	983.4136874	135	Grand Index
50	m	0-12 years	0.991	986.4896106	135	Grand Index
50	m	0-12 years	0.992	989.4608242	136	Grand Index
50	m	0-12 years	0.993	991.7317921	137	Grand Index

Age	Gender	Education	Percentile	Raw Score	Norm Score	Assessment
50	m	0-12 years	0.994	994.8910799	138	Grand Index
50	m	0-12 years	0.995	1006.267327	139	Grand Index
50	m	0-12 years	0.996	1017.257321	140	Grand Index
50	m	0-12 years	0.997	1022.694916	141	Grand Index
50	m	0-12 years	0.998	1028.228388	143	Grand Index
50	m	0-12 years	0.999	1036.614194	146	Grand Index
50	f	13-16 years	0.001	600.4485535	54	Grand Index
50	f	13-16 years	0.002	605.4966899	57	Grand Index
50	f	13-16 years	0.003	621.355	59	Grand Index
50	f	13-16 years	0.004	624.7099688	60	Grand Index
50	f	13-16 years	0.005	626.9884159	61	Grand Index
50	f	13-16 years	0.006	631.4121259	62	Grand Index
50	f	13-16 years	0.007	636	63	Grand Index
50	f	13-16 years	0.008	637	64	Grand Index
50	f	13-16 years	0.009	639.0712675	65	Grand Index
50	f	13-16 years	0.01	641.8336303	65	Grand Index
50	f	13-16 years	0.011	645.1522339	66	Grand Index
50	f	13-16 years	0.012	647.948682	66	Grand Index
50	f	13-16 years	0.013	650.735	67	Grand Index
50	f	13-16 years	0.014	653.009024	67	Grand Index
50	f	13-16 years	0.015	655	67	Grand Index
50	f	13-16 years	0.016	657.095419	68	Grand Index
50	f	13-16 years	0.017	658	68	Grand Index
50	f	13-16 years	0.018	658.9678864	69	Grand Index
50	f	13-16 years	0.019	661	69	Grand Index
50	f	13-16 years	0.02	663.5858823	69	Grand Index
50	f	13-16 years	0.021	665.3597745	69	Grand Index
50	f	13-16 years	0.022	666	70	Grand Index
50	f	13-16 years	0.024	667	70	Grand Index
50	f	13-16 years	0.025	668.896228	71	Grand Index
50	f	13-16 years	0.026	669.189049	71	Grand Index
50	f	13-16 years	0.027	670	71	Grand Index
50	f	13-16 years	0.028	671	71	Grand Index
50	f	13-16 years	0.029	672.3654518	72	Grand Index
50	f	13-16 years	0.03	673	72	Grand Index
50	f	13-16 years	0.031	674	72	Grand Index
50	f	13-16 years	0.032	675	72	Grand Index
50	f	13-16 years	0.033	677.0641839	72	Grand Index
50	f	13-16 years	0.034	678.1824312	73	Grand Index
50	f	13-16 years	0.035	679	73	Grand Index
50	f	13-16 years	0.036	680.7063594	73	Grand Index
50	f	13-16 years	0.037	681	73	Grand Index
50	f	13-16 years	0.038	682	73	Grand Index
50	f	13-16 years	0.039	683	74	Grand Index
50	f	13-16 years	0.04	683.4604989	74	Grand Index
50	f	13-16 years	0.041	685.6996538	74	Grand Index
50	f	13-16 years	0.042	686	74	Grand Index
50	f	13-16 years	0.043	687	74	Grand Index
50	f	13-16 years	0.044	688	74	Grand Index
50	f	13-16 years	0.045	688.7836967	75	Grand Index
50	f	13-16 years	0.046	690	75	Grand Index
50	f	13-16 years	0.047	690.9998482	75	Grand Index
50	f	13-16 years	0.048	692.4473428	75	Grand Index
50	f	13-16 years	0.049	693.0249563	75	Grand Index
50	f	13-16 years	0.05	694	75	Grand Index
50	f	13-16 years	0.051	694.3114004	75	Grand Index

Age	Gender	Education	Percentile	Raw Score	Norm Score	Assessment
50	f	13-16 years	0.053	695	76	Grand Index
50	f	13-16 years	0.054	695.8257542	76	Grand Index
50	f	13-16 years	0.055	696.725	76	Grand Index
50	f	13-16 years	0.056	697.0976711	76	Grand Index
50	f	13-16 years	0.057	698	76	Grand Index
50	f	13-16 years	0.058	699.51	76	Grand Index
50	f	13-16 years	0.059	700.1145858	77	Grand Index
50	f	13-16 years	0.06	701	77	Grand Index
50	f	13-16 years	0.061	701.9231455	77	Grand Index
50	f	13-16 years	0.062	702	77	Grand Index
50	f	13-16 years	0.063	703	77	Grand Index
50	f	13-16 years	0.064	704	77	Grand Index
50	f	13-16 years	0.065	704.056136	77	Grand Index
50	f	13-16 years	0.067	705	78	Grand Index
50	f	13-16 years	0.068	705.695181	78	Grand Index
50	f	13-16 years	0.069	706	78	Grand Index
50	f	13-16 years	0.071	707	78	Grand Index
50	f	13-16 years	0.072	707.6994315	78	Grand Index
50	f	13-16 years	0.074	708	78	Grand Index
50	f	13-16 years	0.075	708.8211064	78	Grand Index
50	f	13-16 years	0.077	709	79	Grand Index
50	f	13-16 years	0.079	710	79	Grand Index
50	f	13-16 years	0.08	711	79	Grand Index
50	f	13-16 years	0.081	711.1862346	79	Grand Index
50	f	13-16 years	0.082	712	79	Grand Index
50	f	13-16 years	0.083	712.7771366	79	Grand Index
50	f	13-16 years	0.084	713	79	Grand Index
50	f	13-16 years	0.085	713.2898939	79	Grand Index
50	f	13-16 years	0.088	714	80	Grand Index
50	f	13-16 years	0.09	715	80	Grand Index
50	f	13-16 years	0.092	716	80	Grand Index
50	f	13-16 years	0.095	717	80	Grand Index
50	f	13-16 years	0.097	718	81	Grand Index
50	f	13-16 years	0.099	719	81	Grand Index
50	f	13-16 years	0.101	720	81	Grand Index
50	f	13-16 years	0.102	720.16315	81	Grand Index
50	f	13-16 years	0.104	721	81	Grand Index
50	f	13-16 years	0.105	721.3475199	81	Grand Index
50	f	13-16 years	0.107	722	81	Grand Index
50	f	13-16 years	0.108	722.1571131	81	Grand Index
50	f	13-16 years	0.111	723	82	Grand Index
50	f	13-16 years	0.112	723.2155145	82	Grand Index
50	f	13-16 years	0.114	724	82	Grand Index
50	f	13-16 years	0.117	725	82	Grand Index
50	f	13-16 years	0.118	726	82	Grand Index
50	f	13-16 years	0.119	726.0659067	82	Grand Index
50	f	13-16 years	0.121	727	82	Grand Index
50	f	13-16 years	0.122	727.1496504	83	Grand Index
50	f	13-16 years	0.124	728	83	Grand Index
50	f	13-16 years	0.125	728.5111499	83	Grand Index
50	f	13-16 years	0.126	729	83	Grand Index
50	f	13-16 years	0.127	729.6472093	83	Grand Index
50	f	13-16 years	0.129	730	83	Grand Index
50	f	13-16 years	0.131	731	83	Grand Index
50	f	13-16 years	0.133	732	83	Grand Index
50	f	13-16 years	0.136	733	84	Grand Index

Age	Gender	Education	Percentile	Raw Score	Norm Score	Assessment
50	f	13-16 years	0.137	733.8329995	84	Grand Index
50	f	13-16 years	0.138	734	84	Grand Index
50	f	13-16 years	0.139	734.5122243	84	Grand Index
50	f	13-16 years	0.144	735	84	Grand Index
50	f	13-16 years	0.146	736	84	Grand Index
50	f	13-16 years	0.147	736.0975204	84	Grand Index
50	f	13-16 years	0.149	737	84	Grand Index
50	f	13-16 years	0.15	737.2676912	84	Grand Index
50	f	13-16 years	0.153	738	85	Grand Index
50	f	13-16 years	0.154	738.5483776	85	Grand Index
50	f	13-16 years	0.156	739	85	Grand Index
50	f	13-16 years	0.157	739.0677197	85	Grand Index
50	f	13-16 years	0.161	740	85	Grand Index
50	f	13-16 years	0.165	741	85	Grand Index
50	f	13-16 years	0.166	741.6365095	85	Grand Index
50	f	13-16 years	0.168	742	86	Grand Index
50	f	13-16 years	0.171	743	86	Grand Index
50	f	13-16 years	0.174	744	86	Grand Index
50	f	13-16 years	0.175	744.017245	86	Grand Index
50	f	13-16 years	0.178	745	86	Grand Index
50	f	13-16 years	0.179	745.5873572	86	Grand Index
50	f	13-16 years	0.183	746	86	Grand Index
50	f	13-16 years	0.187	747	87	Grand Index
50	f	13-16 years	0.191	748	87	Grand Index
50	f	13-16 years	0.195	749	87	Grand Index
50	f	13-16 years	0.196	749.9245677	87	Grand Index
50	f	13-16 years	0.2	750	87	Grand Index
50	f	13-16 years	0.204	751	88	Grand Index
50	f	13-16 years	0.205	751.9796337	88	Grand Index
50	f	13-16 years	0.209	752	88	Grand Index
50	f	13-16 years	0.21	752.7457003	88	Grand Index
50	f	13-16 years	0.213	753	88	Grand Index
50	f	13-16 years	0.214	753.5396124	88	Grand Index
50	f	13-16 years	0.217	754	88	Grand Index
50	f	13-16 years	0.218	754.7174672	88	Grand Index
50	f	13-16 years	0.222	755	89	Grand Index
50	f	13-16 years	0.228	756	89	Grand Index
50	f	13-16 years	0.233	757	89	Grand Index
50	f	13-16 years	0.234	757.7386874	89	Grand Index
50	f	13-16 years	0.235	758	89	Grand Index
50	f	13-16 years	0.236	758.0847781	89	Grand Index
50	f	13-16 years	0.242	759	90	Grand Index
50	f	13-16 years	0.243	759.2231097	90	Grand Index
50	f	13-16 years	0.245	760	90	Grand Index
50	f	13-16 years	0.246	760.3095247	90	Grand Index
50	f	13-16 years	0.249	761	90	Grand Index
50	f	13-16 years	0.25	761.7171341	90	Grand Index
50	f	13-16 years	0.253	762	90	Grand Index
50	f	13-16 years	0.258	763	90	Grand Index
50	f	13-16 years	0.261	764	90	Grand Index
50	f	13-16 years	0.262	764.0427819	90	Grand Index
50	f	13-16 years	0.265	765	91	Grand Index
50	f	13-16 years	0.266	765.7649497	91	Grand Index
50	f	13-16 years	0.269	766	91	Grand Index
50	f	13-16 years	0.27	766.344296	91	Grand Index
50	f	13-16 years	0.273	767	91	Grand Index

Age	Gender	Education	Percentile	Raw Score	Norm Score	Assessment
50	f	13-16 years	0.274	767.0116935	91	Grand Index
50	f	13-16 years	0.277	768	91	Grand Index
50	f	13-16 years	0.278	768.3839001	91	Grand Index
50	f	13-16 years	0.281	769	91	Grand Index
50	f	13-16 years	0.282	769.0620702	91	Grand Index
50	f	13-16 years	0.286	770	92	Grand Index
50	f	13-16 years	0.29	771	92	Grand Index
50	f	13-16 years	0.291	771.478841	92	Grand Index
50	f	13-16 years	0.294	772	92	Grand Index
50	f	13-16 years	0.295	772.0551239	92	Grand Index
50	f	13-16 years	0.3	773	92	Grand Index
50	f	13-16 years	0.301	773.6026103	92	Grand Index
50	f	13-16 years	0.304	774	92	Grand Index
50	f	13-16 years	0.305	774.0730769	92	Grand Index
50	f	13-16 years	0.31	775	93	Grand Index
50	f	13-16 years	0.314	776	93	Grand Index
50	f	13-16 years	0.315	776.6029906	93	Grand Index
50	f	13-16 years	0.32	777	93	Grand Index
50	f	13-16 years	0.324	778	93	Grand Index
50	f	13-16 years	0.329	779	93	Grand Index
50	f	13-16 years	0.33	779.4249801	93	Grand Index
50	f	13-16 years	0.333	780	94	Grand Index
50	f	13-16 years	0.334	780.4120464	94	Grand Index
50	f	13-16 years	0.34	781	94	Grand Index
50	f	13-16 years	0.345	782	94	Grand Index
50	f	13-16 years	0.349	783	94	Grand Index
50	f	13-16 years	0.35	783.000704	94	Grand Index
50	f	13-16 years	0.351	783.8934281	94	Grand Index
50	f	13-16 years	0.355	784	94	Grand Index
50	f	13-16 years	0.356	784.2109109	94	Grand Index
50	f	13-16 years	0.361	785	95	Grand Index
50	f	13-16 years	0.362	785.6257291	95	Grand Index
50	f	13-16 years	0.366	786	95	Grand Index
50	f	13-16 years	0.37	787	95	Grand Index
50	f	13-16 years	0.371	787.193986	95	Grand Index
50	f	13-16 years	0.374	788	95	Grand Index
50	f	13-16 years	0.375	788.2586546	95	Grand Index
50	f	13-16 years	0.379	789	95	Grand Index
50	f	13-16 years	0.385	790	96	Grand Index
50	f	13-16 years	0.386	790.3023817	96	Grand Index
50	f	13-16 years	0.39	791	96	Grand Index
50	f	13-16 years	0.391	791.8246279	96	Grand Index
50	f	13-16 years	0.395	792	96	Grand Index
50	f	13-16 years	0.396	792.0640935	96	Grand Index
50	f	13-16 years	0.4	793	96	Grand Index
50	f	13-16 years	0.401	793.8684641	96	Grand Index
50	f	13-16 years	0.406	794	96	Grand Index
50	f	13-16 years	0.41	795	97	Grand Index
50	f	13-16 years	0.411	795.0248985	97	Grand Index
50	f	13-16 years	0.415	796	97	Grand Index
50	f	13-16 years	0.421	797	97	Grand Index
50	f	13-16 years	0.422	797.0218358	97	Grand Index
50	f	13-16 years	0.423	797.9761861	97	Grand Index
50	f	13-16 years	0.426	798	97	Grand Index
50	f	13-16 years	0.43	799	97	Grand Index
50	f	13-16 years	0.434	800	98	Grand Index

Age	Gender	Education	Percentile	Raw Score	Norm Score	Assessment
50	f	13-16 years	0.435	800.540757	98	Grand Index
50	f	13-16 years	0.439	801	98	Grand Index
50	f	13-16 years	0.44	801.9845367	98	Grand Index
50	f	13-16 years	0.444	802	98	Grand Index
50	f	13-16 years	0.445	802.4415541	98	Grand Index
50	f	13-16 years	0.449	803	98	Grand Index
50	f	13-16 years	0.455	804	98	Grand Index
50	f	13-16 years	0.456	804.2488845	98	Grand Index
50	f	13-16 years	0.461	805	99	Grand Index
50	f	13-16 years	0.462	805.6884725	99	Grand Index
50	f	13-16 years	0.466	806	99	Grand Index
50	f	13-16 years	0.467	806.0330277	99	Grand Index
50	f	13-16 years	0.473	807	99	Grand Index
50	f	13-16 years	0.474	807.401171	99	Grand Index
50	f	13-16 years	0.478	808	99	Grand Index
50	f	13-16 years	0.479	808.9605355	99	Grand Index
50	f	13-16 years	0.482	809	99	Grand Index
50	f	13-16 years	0.487	810	100	Grand Index
50	f	13-16 years	0.488	810.7583674	100	Grand Index
50	f	13-16 years	0.493	811	100	Grand Index
50	f	13-16 years	0.494	811.3504542	100	Grand Index
50	f	13-16 years	0.499	812	100	Grand Index
50	f	13-16 years	0.5	812.9961661	100	Grand Index
50	f	13-16 years	0.505	813	100	Grand Index
50	f	13-16 years	0.509	814	100	Grand Index
50	f	13-16 years	0.51	814.6296183	100	Grand Index
50	f	13-16 years	0.514	815	101	Grand Index
50	f	13-16 years	0.518	816	101	Grand Index
50	f	13-16 years	0.519	816.6992556	101	Grand Index
50	f	13-16 years	0.523	817	101	Grand Index
50	f	13-16 years	0.524	817.0601563	101	Grand Index
50	f	13-16 years	0.525	817.902402	101	Grand Index
50	f	13-16 years	0.527	818	101	Grand Index
50	f	13-16 years	0.528	818.0079264	101	Grand Index
50	f	13-16 years	0.529	818.9291678	101	Grand Index
50	f	13-16 years	0.533	819	101	Grand Index
50	f	13-16 years	0.534	819.1751915	101	Grand Index
50	f	13-16 years	0.54	820	102	Grand Index
50	f	13-16 years	0.541	820.9987898	102	Grand Index
50	f	13-16 years	0.545	821	102	Grand Index
50	f	13-16 years	0.546	821.0768222	102	Grand Index
50	f	13-16 years	0.55	822	102	Grand Index
50	f	13-16 years	0.551	822.7817097	102	Grand Index
50	f	13-16 years	0.556	823	102	Grand Index
50	f	13-16 years	0.56	824	102	Grand Index
50	f	13-16 years	0.561	824.4410315	102	Grand Index
50	f	13-16 years	0.566	825	102	Grand Index
50	f	13-16 years	0.571	826	103	Grand Index
50	f	13-16 years	0.572	826.0086	103	Grand Index
50	f	13-16 years	0.577	827	103	Grand Index
50	f	13-16 years	0.578	827.9834145	103	Grand Index
50	f	13-16 years	0.582	828	103	Grand Index
50	f	13-16 years	0.583	828.885	103	Grand Index
50	f	13-16 years	0.586	829	103	Grand Index
50	f	13-16 years	0.589	830	103	Grand Index
50	f	13-16 years	0.59	830.5101299	103	Grand Index

Age	Gender	Education	Percentile	Raw Score	Norm Score	Assessment
50	f	13-16 years	0.594	831	104	Grand Index
50	f	13-16 years	0.595	831.0003128	104	Grand Index
50	f	13-16 years	0.6	832	104	Grand Index
50	f	13-16 years	0.601	832.7968999	104	Grand Index
50	f	13-16 years	0.604	833	104	Grand Index
50	f	13-16 years	0.605	833.0542177	104	Grand Index
50	f	13-16 years	0.606	833.9965542	104	Grand Index
50	f	13-16 years	0.611	834	104	Grand Index
50	f	13-16 years	0.614	835	104	Grand Index
50	f	13-16 years	0.615	835.6467858	104	Grand Index
50	f	13-16 years	0.62	836	105	Grand Index
50	f	13-16 years	0.621	836.7435454	105	Grand Index
50	f	13-16 years	0.625	837	105	Grand Index
50	f	13-16 years	0.626	837.4631006	105	Grand Index
50	f	13-16 years	0.631	838	105	Grand Index
50	f	13-16 years	0.637	839	105	Grand Index
50	f	13-16 years	0.638	839.6418932	105	Grand Index
50	f	13-16 years	0.641	840	105	Grand Index
50	f	13-16 years	0.642	840.045098	105	Grand Index
50	f	13-16 years	0.646	841	106	Grand Index
50	f	13-16 years	0.649	842	106	Grand Index
50	f	13-16 years	0.65	842.0296137	106	Grand Index
50	f	13-16 years	0.655	843	106	Grand Index
50	f	13-16 years	0.656	843.3046832	106	Grand Index
50	f	13-16 years	0.66	844	106	Grand Index
50	f	13-16 years	0.661	844.9617574	106	Grand Index
50	f	13-16 years	0.666	845	106	Grand Index
50	f	13-16 years	0.667	845.810017	106	Grand Index
50	f	13-16 years	0.672	846	107	Grand Index
50	f	13-16 years	0.675	847	107	Grand Index
50	f	13-16 years	0.68	848	107	Grand Index
50	f	13-16 years	0.685	849	107	Grand Index
50	f	13-16 years	0.688	850	107	Grand Index
50	f	13-16 years	0.693	851	108	Grand Index
50	f	13-16 years	0.694	851.1781508	108	Grand Index
50	f	13-16 years	0.697	852	108	Grand Index
50	f	13-16 years	0.698	852.2591814	108	Grand Index
50	f	13-16 years	0.699	852.9900276	108	Grand Index
50	f	13-16 years	0.701	853	108	Grand Index
50	f	13-16 years	0.702	853.7775433	108	Grand Index
50	f	13-16 years	0.705	854	108	Grand Index
50	f	13-16 years	0.706	854.9765139	108	Grand Index
50	f	13-16 years	0.709	855	108	Grand Index
50	f	13-16 years	0.71	855.3882219	108	Grand Index
50	f	13-16 years	0.715	856	109	Grand Index
50	f	13-16 years	0.716	856.4781987	109	Grand Index
50	f	13-16 years	0.721	857	109	Grand Index
50	f	13-16 years	0.722	857.7552197	109	Grand Index
50	f	13-16 years	0.726	858	109	Grand Index
50	f	13-16 years	0.727	858.3254935	109	Grand Index
50	f	13-16 years	0.732	859	109	Grand Index
50	f	13-16 years	0.733	859.0948279	109	Grand Index
50	f	13-16 years	0.734	859.9743145	109	Grand Index
50	f	13-16 years	0.738	860	110	Grand Index
50	f	13-16 years	0.739	860.7166481	110	Grand Index
50	f	13-16 years	0.743	861	110	Grand Index

Age	Gender	Education	Percentile	Raw Score	Norm Score	Assessment
50	f	13-16 years	0.744	861.1947463	110	Grand Index
50	f	13-16 years	0.748	862	110	Grand Index
50	f	13-16 years	0.749	862.4960441	110	Grand Index
50	f	13-16 years	0.75	863	110	Grand Index
50	f	13-16 years	0.751	863.884567	110	Grand Index
50	f	13-16 years	0.755	864	110	Grand Index
50	f	13-16 years	0.756	864.1958644	110	Grand Index
50	f	13-16 years	0.759	865	111	Grand Index
50	f	13-16 years	0.76	865.2076105	111	Grand Index
50	f	13-16 years	0.762	866	111	Grand Index
50	f	13-16 years	0.764	867	111	Grand Index
50	f	13-16 years	0.765	867.0295684	111	Grand Index
50	f	13-16 years	0.766	867.8530251	111	Grand Index
50	f	13-16 years	0.771	868	111	Grand Index
50	f	13-16 years	0.772	868.9394709	111	Grand Index
50	f	13-16 years	0.774	869	111	Grand Index
50	f	13-16 years	0.775	869.0182173	111	Grand Index
50	f	13-16 years	0.776	869.987855	111	Grand Index
50	f	13-16 years	0.779	870	112	Grand Index
50	f	13-16 years	0.78	870.3954521	112	Grand Index
50	f	13-16 years	0.784	871	112	Grand Index
50	f	13-16 years	0.785	871.9538212	112	Grand Index
50	f	13-16 years	0.788	872	112	Grand Index
50	f	13-16 years	0.789	872.9273653	112	Grand Index
50	f	13-16 years	0.792	873	112	Grand Index
50	f	13-16 years	0.793	873.7591267	112	Grand Index
50	f	13-16 years	0.796	874	112	Grand Index
50	f	13-16 years	0.8	875	113	Grand Index
50	f	13-16 years	0.801	875.1592122	113	Grand Index
50	f	13-16 years	0.805	876	113	Grand Index
50	f	13-16 years	0.807	877	113	Grand Index
50	f	13-16 years	0.808	877.7102618	113	Grand Index
50	f	13-16 years	0.81	878	113	Grand Index
50	f	13-16 years	0.811	878.2325567	113	Grand Index
50	f	13-16 years	0.814	879	113	Grand Index
50	f	13-16 years	0.815	879.7394314	113	Grand Index
50	f	13-16 years	0.817	880	114	Grand Index
50	f	13-16 years	0.818	880.8613588	114	Grand Index
50	f	13-16 years	0.821	881	114	Grand Index
50	f	13-16 years	0.822	881.4702568	114	Grand Index
50	f	13-16 years	0.825	882	114	Grand Index
50	f	13-16 years	0.827	883	114	Grand Index
50	f	13-16 years	0.828	883.4649007	114	Grand Index
50	f	13-16 years	0.831	884	114	Grand Index
50	f	13-16 years	0.832	884.1596518	114	Grand Index
50	f	13-16 years	0.835	885	115	Grand Index
50	f	13-16 years	0.836	885.4309157	115	Grand Index
50	f	13-16 years	0.838	886	115	Grand Index
50	f	13-16 years	0.839	886.2065215	115	Grand Index
50	f	13-16 years	0.842	887	115	Grand Index
50	f	13-16 years	0.845	888	115	Grand Index
50	f	13-16 years	0.848	889	115	Grand Index
50	f	13-16 years	0.849	889.2833544	115	Grand Index
50	f	13-16 years	0.851	890	116	Grand Index
50	f	13-16 years	0.852	890.9708449	116	Grand Index
50	f	13-16 years	0.853	891	116	Grand Index

Age	Gender	Education	Percentile	Raw Score	Norm Score	Assessment
50	f	13-16 years	0.854	891.2300832	116	Grand Index
50	f	13-16 years	0.857	892	116	Grand Index
50	f	13-16 years	0.86	893	116	Grand Index
50	f	13-16 years	0.861	893.2118708	116	Grand Index
50	f	13-16 years	0.864	894	116	Grand Index
50	f	13-16 years	0.866	895	117	Grand Index
50	f	13-16 years	0.867	895.0370012	117	Grand Index
50	f	13-16 years	0.868	896	117	Grand Index
50	f	13-16 years	0.869	896.2880214	117	Grand Index
50	f	13-16 years	0.872	897	117	Grand Index
50	f	13-16 years	0.875	898	117	Grand Index
50	f	13-16 years	0.877	899	117	Grand Index
50	f	13-16 years	0.878	899.2529031	117	Grand Index
50	f	13-16 years	0.879	900	118	Grand Index
50	f	13-16 years	0.88	900.4891618	118	Grand Index
50	f	13-16 years	0.882	901	118	Grand Index
50	f	13-16 years	0.884	902	118	Grand Index
50	f	13-16 years	0.885	902.5710108	118	Grand Index
50	f	13-16 years	0.886	903	118	Grand Index
50	f	13-16 years	0.887	903.765	118	Grand Index
50	f	13-16 years	0.888	904	118	Grand Index
50	f	13-16 years	0.889	905	118	Grand Index
50	f	13-16 years	0.89	905.0131498	118	Grand Index
50	f	13-16 years	0.891	906	118	Grand Index
50	f	13-16 years	0.893	907	119	Grand Index
50	f	13-16 years	0.894	907.7676859	119	Grand Index
50	f	13-16 years	0.896	908	119	Grand Index
50	f	13-16 years	0.898	909	119	Grand Index
50	f	13-16 years	0.9	910	119	Grand Index
50	f	13-16 years	0.901	910.1891479	119	Grand Index
50	f	13-16 years	0.903	911	119	Grand Index
50	f	13-16 years	0.904	911.086008	120	Grand Index
50	f	13-16 years	0.905	912	120	Grand Index
50	f	13-16 years	0.906	912.0078594	120	Grand Index
50	f	13-16 years	0.907	913.196373	120	Grand Index
50	f	13-16 years	0.908	914	120	Grand Index
50	f	13-16 years	0.91	915	120	Grand Index
50	f	13-16 years	0.911	915.0979391	120	Grand Index
50	f	13-16 years	0.913	916	120	Grand Index
50	f	13-16 years	0.914	916.9527273	120	Grand Index
50	f	13-16 years	0.915	917.4008982	121	Grand Index
50	f	13-16 years	0.918	918	121	Grand Index
50	f	13-16 years	0.92	919	121	Grand Index
50	f	13-16 years	0.921	919.3424544	121	Grand Index
50	f	13-16 years	0.923	920	121	Grand Index
50	f	13-16 years	0.924	920.2192523	121	Grand Index
50	f	13-16 years	0.926	921	122	Grand Index
50	f	13-16 years	0.928	922	122	Grand Index
50	f	13-16 years	0.929	922.8911111	122	Grand Index
50	f	13-16 years	0.93	923.580696	122	Grand Index
50	f	13-16 years	0.931	924	122	Grand Index
50	f	13-16 years	0.932	924.072039	122	Grand Index
50	f	13-16 years	0.934	925	123	Grand Index
50	f	13-16 years	0.936	926	123	Grand Index
50	f	13-16 years	0.937	927.6655853	123	Grand Index
50	f	13-16 years	0.939	929	123	Grand Index

Age	Gender	Education	Percentile	Raw Score	Norm Score	Assessment
50	f	13-16 years	0.94	930	123	Grand Index
50	f	13-16 years	0.941	930.4451694	123	Grand Index
50	f	13-16 years	0.942	931	124	Grand Index
50	f	13-16 years	0.943	931.0667807	124	Grand Index
50	f	13-16 years	0.944	932	124	Grand Index
50	f	13-16 years	0.945	932.8803745	124	Grand Index
50	f	13-16 years	0.946	934.2510128	124	Grand Index
50	f	13-16 years	0.947	935	124	Grand Index
50	f	13-16 years	0.948	936	124	Grand Index
50	f	13-16 years	0.949	936.8135755	125	Grand Index
50	f	13-16 years	0.95	938.012963	125	Grand Index
50	f	13-16 years	0.951	939	125	Grand Index
50	f	13-16 years	0.952	940.0631623	125	Grand Index
50	f	13-16 years	0.953	942	125	Grand Index
50	f	13-16 years	0.954	943	125	Grand Index
50	f	13-16 years	0.956	944	126	Grand Index
50	f	13-16 years	0.957	945	126	Grand Index
50	f	13-16 years	0.958	945.9814514	126	Grand Index
50	f	13-16 years	0.959	946	126	Grand Index
50	f	13-16 years	0.96	946.2	126	Grand Index
50	f	13-16 years	0.961	947.9938967	126	Grand Index
50	f	13-16 years	0.962	948.4122089	127	Grand Index
50	f	13-16 years	0.963	949.0270866	127	Grand Index
50	f	13-16 years	0.964	950	127	Grand Index
50	f	13-16 years	0.965	951.0010742	127	Grand Index
50	f	13-16 years	0.966	952	127	Grand Index
50	f	13-16 years	0.967	954.1725725	128	Grand Index
50	f	13-16 years	0.968	955.003966	128	Grand Index
50	f	13-16 years	0.969	956	128	Grand Index
50	f	13-16 years	0.97	957	128	Grand Index
50	f	13-16 years	0.971	957.804797	128	Grand Index
50	f	13-16 years	0.972	960	129	Grand Index
50	f	13-16 years	0.973	961.2386786	129	Grand Index
50	f	13-16 years	0.974	962.6957038	129	Grand Index
50	f	13-16 years	0.975	963.1319204	129	Grand Index
50	f	13-16 years	0.976	964.7442684	130	Grand Index
50	f	13-16 years	0.977	966	130	Grand Index
50	f	13-16 years	0.978	968	130	Grand Index
50	f	13-16 years	0.979	970.4561804	131	Grand Index
50	f	13-16 years	0.98	971	131	Grand Index
50	f	13-16 years	0.981	971.6844143	131	Grand Index
50	f	13-16 years	0.982	973.061945	131	Grand Index
50	f	13-16 years	0.983	975	132	Grand Index
50	f	13-16 years	0.984	976	132	Grand Index
50	f	13-16 years	0.985	977.8465772	133	Grand Index
50	f	13-16 years	0.986	979	133	Grand Index
50	f	13-16 years	0.987	984	133	Grand Index
50	f	13-16 years	0.988	986.0028798	134	Grand Index
50	f	13-16 years	0.989	991.139196	134	Grand Index
50	f	13-16 years	0.99	993.2291871	135	Grand Index
50	f	13-16 years	0.991	995.8485979	135	Grand Index
50	f	13-16 years	0.992	998.24	136	Grand Index
50	f	13-16 years	0.993	1001.240605	137	Grand Index
50	f	13-16 years	0.994	1007.543632	138	Grand Index
50	f	13-16 years	0.995	1010.034627	139	Grand Index
50	f	13-16 years	0.996	1014.797678	140	Grand Index

Age	Gender	Education	Percentile	Raw Score	Norm Score	Assessment
50	f	13-16 years	0.997	1021.804592	141	Grand Index
50	f	13-16 years	0.998	1030.870459	143	Grand Index
50	f	13-16 years	0.999	1055.627718	146	Grand Index
50	m	13-16 years	0.001	603.056	54	Grand Index
50	m	13-16 years	0.002	605.4678985	57	Grand Index
50	m	13-16 years	0.003	618.8682265	59	Grand Index
50	m	13-16 years	0.004	630.8143537	60	Grand Index
50	m	13-16 years	0.005	636.5080562	61	Grand Index
50	m	13-16 years	0.006	639.344	62	Grand Index
50	m	13-16 years	0.007	644	63	Grand Index
50	m	13-16 years	0.008	647.7361578	64	Grand Index
50	m	13-16 years	0.009	648	65	Grand Index
50	m	13-16 years	0.01	651.56	65	Grand Index
50	m	13-16 years	0.011	654.232	66	Grand Index
50	m	13-16 years	0.012	655.5634783	66	Grand Index
50	m	13-16 years	0.013	656.728	67	Grand Index
50	m	13-16 years	0.014	657.9317525	67	Grand Index
50	m	13-16 years	0.015	658.84	67	Grand Index
50	m	13-16 years	0.016	659	68	Grand Index
50	m	13-16 years	0.017	659.3724675	68	Grand Index
50	m	13-16 years	0.018	662.4867975	69	Grand Index
50	m	13-16 years	0.019	665.0149633	69	Grand Index
50	m	13-16 years	0.02	666.1425699	69	Grand Index
50	m	13-16 years	0.021	667.6464913	69	Grand Index
50	m	13-16 years	0.022	669	70	Grand Index
50	m	13-16 years	0.023	669.4251772	70	Grand Index
50	m	13-16 years	0.024	670.9179456	70	Grand Index
50	m	13-16 years	0.025	672	71	Grand Index
50	m	13-16 years	0.026	674.8298385	71	Grand Index
50	m	13-16 years	0.027	675.7204167	71	Grand Index
50	m	13-16 years	0.028	676.7223173	71	Grand Index
50	m	13-16 years	0.029	678	72	Grand Index
50	m	13-16 years	0.03	678.68	72	Grand Index
50	m	13-16 years	0.031	679.736	72	Grand Index
50	m	13-16 years	0.032	681.5966868	72	Grand Index
50	m	13-16 years	0.033	682.0290815	72	Grand Index
50	m	13-16 years	0.034	683.4157901	73	Grand Index
50	m	13-16 years	0.035	685	73	Grand Index
50	m	13-16 years	0.037	686	73	Grand Index
50	m	13-16 years	0.038	687.128	73	Grand Index
50	m	13-16 years	0.039	688.184	74	Grand Index
50	m	13-16 years	0.04	689	74	Grand Index
50	m	13-16 years	0.041	689.7972714	74	Grand Index
50	m	13-16 years	0.043	690	74	Grand Index
50	m	13-16 years	0.044	691.0324663	74	Grand Index
50	m	13-16 years	0.045	692.015051	75	Grand Index
50	m	13-16 years	0.046	692.4219111	75	Grand Index
50	m	13-16 years	0.048	693	75	Grand Index
50	m	13-16 years	0.049	693.0967342	75	Grand Index
50	m	13-16 years	0.051	694	75	Grand Index
50	m	13-16 years	0.052	694.9251513	76	Grand Index
50	m	13-16 years	0.053	695	76	Grand Index
50	m	13-16 years	0.054	695.2482878	76	Grand Index
50	m	13-16 years	0.055	695.74125	76	Grand Index
50	m	13-16 years	0.056	696	76	Grand Index
50	m	13-16 years	0.057	696.0451488	76	Grand Index

Age	Gender	Education	Percentile	Raw Score	Norm Score	Assessment
50	m	13-16 years	0.058	696.8983097	76	Grand Index
50	m	13-16 years	0.059	697.0798824	77	Grand Index
50	m	13-16 years	0.062	698	77	Grand Index
50	m	13-16 years	0.063	698.5179025	77	Grand Index
50	m	13-16 years	0.065	699	77	Grand Index
50	m	13-16 years	0.066	700.696	77	Grand Index
50	m	13-16 years	0.067	701.097881	78	Grand Index
50	m	13-16 years	0.068	702.808	78	Grand Index
50	m	13-16 years	0.069	703	78	Grand Index
50	m	13-16 years	0.07	703.8856215	78	Grand Index
50	m	13-16 years	0.071	704.97825	78	Grand Index
50	m	13-16 years	0.073	705	78	Grand Index
50	m	13-16 years	0.074	705.988358	78	Grand Index
50	m	13-16 years	0.075	706	78	Grand Index
50	m	13-16 years	0.076	706.3557525	79	Grand Index
50	m	13-16 years	0.077	707	79	Grand Index
50	m	13-16 years	0.078	708.011038	79	Grand Index
50	m	13-16 years	0.079	708.7339102	79	Grand Index
50	m	13-16 years	0.08	709.48	79	Grand Index
50	m	13-16 years	0.081	710.5030206	79	Grand Index
50	m	13-16 years	0.084	711	79	Grand Index
50	m	13-16 years	0.085	711.9189733	79	Grand Index
50	m	13-16 years	0.086	712	80	Grand Index
50	m	13-16 years	0.087	712.9076418	80	Grand Index
50	m	13-16 years	0.089	713	80	Grand Index
50	m	13-16 years	0.09	713.0192953	80	Grand Index
50	m	13-16 years	0.092	714	80	Grand Index
50	m	13-16 years	0.093	714.1377829	80	Grand Index
50	m	13-16 years	0.094	715	80	Grand Index
50	m	13-16 years	0.095	715.5501671	80	Grand Index
50	m	13-16 years	0.096	716.376	80	Grand Index
50	m	13-16 years	0.097	717	81	Grand Index
50	m	13-16 years	0.1	718	81	Grand Index
50	m	13-16 years	0.101	718.656	81	Grand Index
50	m	13-16 years	0.103	719	81	Grand Index
50	m	13-16 years	0.104	719.2659128	81	Grand Index
50	m	13-16 years	0.105	719.9965366	81	Grand Index
50	m	13-16 years	0.106	720	81	Grand Index
50	m	13-16 years	0.107	721	81	Grand Index
50	m	13-16 years	0.108	721.048	81	Grand Index
50	m	13-16 years	0.109	722	82	Grand Index
50	m	13-16 years	0.11	722.9380571	82	Grand Index
50	m	13-16 years	0.112	723	82	Grand Index
50	m	13-16 years	0.113	723.328	82	Grand Index
50	m	13-16 years	0.114	724	82	Grand Index
50	m	13-16 years	0.115	724.3584236	82	Grand Index
50	m	13-16 years	0.119	725	82	Grand Index
50	m	13-16 years	0.12	725.8856991	82	Grand Index
50	m	13-16 years	0.122	726	83	Grand Index
50	m	13-16 years	0.123	726.888	83	Grand Index
50	m	13-16 years	0.124	727.944	83	Grand Index
50	m	13-16 years	0.126	728	83	Grand Index
50	m	13-16 years	0.127	728.112	83	Grand Index
50	m	13-16 years	0.129	729	83	Grand Index
50	m	13-16 years	0.13	729.2698083	83	Grand Index
50	m	13-16 years	0.131	730	83	Grand Index

Age	Gender	Education	Percentile	Raw Score	Norm Score	Assessment
50	m	13-16 years	0.134	731	83	Grand Index
50	m	13-16 years	0.135	731.7514257	83	Grand Index
50	m	13-16 years	0.137	732	84	Grand Index
50	m	13-16 years	0.138	732.5211842	84	Grand Index
50	m	13-16 years	0.14	733	84	Grand Index
50	m	13-16 years	0.141	734	84	Grand Index
50	m	13-16 years	0.142	734.0088876	84	Grand Index
50	m	13-16 years	0.143	734.9379236	84	Grand Index
50	m	13-16 years	0.144	735	84	Grand Index
50	m	13-16 years	0.145	735.074262	84	Grand Index
50	m	13-16 years	0.146	735.8547036	84	Grand Index
50	m	13-16 years	0.149	736	84	Grand Index
50	m	13-16 years	0.15	736.5537714	84	Grand Index
50	m	13-16 years	0.153	737	85	Grand Index
50	m	13-16 years	0.157	738	85	Grand Index
50	m	13-16 years	0.158	738.848	85	Grand Index
50	m	13-16 years	0.161	739	85	Grand Index
50	m	13-16 years	0.162	739.1541114	85	Grand Index
50	m	13-16 years	0.165	740	85	Grand Index
50	m	13-16 years	0.166	740.296	85	Grand Index
50	m	13-16 years	0.169	741	86	Grand Index
50	m	13-16 years	0.17	741.1214833	86	Grand Index
50	m	13-16 years	0.173	742	86	Grand Index
50	m	13-16 years	0.174	742.9903172	86	Grand Index
50	m	13-16 years	0.176	743	86	Grand Index
50	m	13-16 years	0.177	743.0835756	86	Grand Index
50	m	13-16 years	0.178	743.4496238	86	Grand Index
50	m	13-16 years	0.181	744	86	Grand Index
50	m	13-16 years	0.182	744.192	86	Grand Index
50	m	13-16 years	0.185	745	87	Grand Index
50	m	13-16 years	0.186	745.4733217	87	Grand Index
50	m	13-16 years	0.191	746	87	Grand Index
50	m	13-16 years	0.192	746.752	87	Grand Index
50	m	13-16 years	0.198	747	87	Grand Index
50	m	13-16 years	0.199	747.4817503	87	Grand Index
50	m	13-16 years	0.201	748	87	Grand Index
50	m	13-16 years	0.202	748.2041441	87	Grand Index
50	m	13-16 years	0.206	749	88	Grand Index
50	m	13-16 years	0.209	750	88	Grand Index
50	m	13-16 years	0.21	750.9962255	88	Grand Index
50	m	13-16 years	0.213	751	88	Grand Index
50	m	13-16 years	0.214	751.984	88	Grand Index
50	m	13-16 years	0.215	752	88	Grand Index
50	m	13-16 years	0.218	753	88	Grand Index
50	m	13-16 years	0.221	754	88	Grand Index
50	m	13-16 years	0.222	754.2178334	89	Grand Index
50	m	13-16 years	0.223	754.7414653	89	Grand Index
50	m	13-16 years	0.225	755	89	Grand Index
50	m	13-16 years	0.226	755.9394001	89	Grand Index
50	m	13-16 years	0.228	756	89	Grand Index
50	m	13-16 years	0.229	756.4055724	89	Grand Index
50	m	13-16 years	0.232	757	89	Grand Index
50	m	13-16 years	0.233	758	89	Grand Index
50	m	13-16 years	0.234	758.018325	89	Grand Index
50	m	13-16 years	0.236	759	89	Grand Index
50	m	13-16 years	0.237	759.0286359	89	Grand Index

Age	Gender	Education	Percentile	Raw Score	Norm Score	Assessment
50	m	13-16 years	0.241	760	89	Grand Index
50	m	13-16 years	0.242	760.8904609	90	Grand Index
50	m	13-16 years	0.247	761	90	Grand Index
50	m	13-16 years	0.248	761.8890228	90	Grand Index
50	m	13-16 years	0.25	762	90	Grand Index
50	m	13-16 years	0.253	763	90	Grand Index
50	m	13-16 years	0.254	763.0482452	90	Grand Index
50	m	13-16 years	0.255	763.8309384	90	Grand Index
50	m	13-16 years	0.258	764	90	Grand Index
50	m	13-16 years	0.259	764.4894679	90	Grand Index
50	m	13-16 years	0.263	765	90	Grand Index
50	m	13-16 years	0.264	765.3315632	91	Grand Index
50	m	13-16 years	0.265	765.9213954	91	Grand Index
50	m	13-16 years	0.268	766	91	Grand Index
50	m	13-16 years	0.271	767	91	Grand Index
50	m	13-16 years	0.272	767.5629951	91	Grand Index
50	m	13-16 years	0.275	768	91	Grand Index
50	m	13-16 years	0.276	768.456	91	Grand Index
50	m	13-16 years	0.28	769	91	Grand Index
50	m	13-16 years	0.281	769.9283698	91	Grand Index
50	m	13-16 years	0.282	770	91	Grand Index
50	m	13-16 years	0.283	770.848	91	Grand Index
50	m	13-16 years	0.286	771	92	Grand Index
50	m	13-16 years	0.287	771.072	92	Grand Index
50	m	13-16 years	0.292	772	92	Grand Index
50	m	13-16 years	0.293	772.238	92	Grand Index
50	m	13-16 years	0.297	773	92	Grand Index
50	m	13-16 years	0.298	773.6238711	92	Grand Index
50	m	13-16 years	0.302	774	92	Grand Index
50	m	13-16 years	0.303	774.989275	92	Grand Index
50	m	13-16 years	0.305	775	92	Grand Index
50	m	13-16 years	0.306	775.0946546	92	Grand Index
50	m	13-16 years	0.312	776	93	Grand Index
50	m	13-16 years	0.317	777	93	Grand Index
50	m	13-16 years	0.318	777.9464565	93	Grand Index
50	m	13-16 years	0.321	778	93	Grand Index
50	m	13-16 years	0.322	778.0297242	93	Grand Index
50	m	13-16 years	0.325	779	93	Grand Index
50	m	13-16 years	0.326	779.2473046	93	Grand Index
50	m	13-16 years	0.33	780	93	Grand Index
50	m	13-16 years	0.331	780.536	93	Grand Index
50	m	13-16 years	0.336	781	94	Grand Index
50	m	13-16 years	0.337	781.9939686	94	Grand Index
50	m	13-16 years	0.34	782	94	Grand Index
50	m	13-16 years	0.341	782.0110118	94	Grand Index
50	m	13-16 years	0.343	783	94	Grand Index
50	m	13-16 years	0.344	783.0786714	94	Grand Index
50	m	13-16 years	0.345	783.7562264	94	Grand Index
50	m	13-16 years	0.349	784	94	Grand Index
50	m	13-16 years	0.35	784.6	94	Grand Index
50	m	13-16 years	0.353	785	94	Grand Index
50	m	13-16 years	0.354	785.1223808	94	Grand Index
50	m	13-16 years	0.355	785.9715535	94	Grand Index
50	m	13-16 years	0.36	786	95	Grand Index
50	m	13-16 years	0.361	786.1767672	95	Grand Index
50	m	13-16 years	0.362	786.9595416	95	Grand Index

Age	Gender	Education	Percentile	Raw Score	Norm Score	Assessment
50	m	13-16 years	0.365	787	95	Grand Index
50	m	13-16 years	0.366	787.2656638	95	Grand Index
50	m	13-16 years	0.371	788	95	Grand Index
50	m	13-16 years	0.372	788.9593392	95	Grand Index
50	m	13-16 years	0.376	789	95	Grand Index
50	m	13-16 years	0.377	789.1362955	95	Grand Index
50	m	13-16 years	0.381	790	95	Grand Index
50	m	13-16 years	0.382	790.2911933	95	Grand Index
50	m	13-16 years	0.389	791	96	Grand Index
50	m	13-16 years	0.392	792	96	Grand Index
50	m	13-16 years	0.396	793	96	Grand Index
50	m	13-16 years	0.397	793.1010479	96	Grand Index
50	m	13-16 years	0.398	793.9874787	96	Grand Index
50	m	13-16 years	0.402	794	96	Grand Index
50	m	13-16 years	0.403	794.0156906	96	Grand Index
50	m	13-16 years	0.404	794.5719086	96	Grand Index
50	m	13-16 years	0.406	795	96	Grand Index
50	m	13-16 years	0.407	795.792	96	Grand Index
50	m	13-16 years	0.412	796	97	Grand Index
50	m	13-16 years	0.413	796.4063635	97	Grand Index
50	m	13-16 years	0.419	797	97	Grand Index
50	m	13-16 years	0.422	798	97	Grand Index
50	m	13-16 years	0.423	798.496934	97	Grand Index
50	m	13-16 years	0.426	799	97	Grand Index
50	m	13-16 years	0.427	799.0189433	97	Grand Index
50	m	13-16 years	0.428	799.2009235	97	Grand Index
50	m	13-16 years	0.433	800	97	Grand Index
50	m	13-16 years	0.434	800.9315091	98	Grand Index
50	m	13-16 years	0.437	801	98	Grand Index
50	m	13-16 years	0.438	801.8021479	98	Grand Index
50	m	13-16 years	0.443	802	98	Grand Index
50	m	13-16 years	0.444	802.0529187	98	Grand Index
50	m	13-16 years	0.445	802.9615561	98	Grand Index
50	m	13-16 years	0.447	803	98	Grand Index
50	m	13-16 years	0.455	804	98	Grand Index
50	m	13-16 years	0.456	804.330351	98	Grand Index
50	m	13-16 years	0.457	804.9730419	98	Grand Index
50	m	13-16 years	0.461	805	99	Grand Index
50	m	13-16 years	0.462	805.812592	99	Grand Index
50	m	13-16 years	0.463	805.9994118	99	Grand Index
50	m	13-16 years	0.47	806	99	Grand Index
50	m	13-16 years	0.471	806.0034338	99	Grand Index
50	m	13-16 years	0.472	806.8986634	99	Grand Index
50	m	13-16 years	0.478	807	99	Grand Index
50	m	13-16 years	0.479	807.07725	99	Grand Index
50	m	13-16 years	0.48	807.3411729	99	Grand Index
50	m	13-16 years	0.483	808	99	Grand Index
50	m	13-16 years	0.484	808.1036206	99	Grand Index
50	m	13-16 years	0.488	809	100	Grand Index
50	m	13-16 years	0.489	809.4121361	100	Grand Index
50	m	13-16 years	0.49	809.9624134	100	Grand Index
50	m	13-16 years	0.492	810	100	Grand Index
50	m	13-16 years	0.493	810.2571138	100	Grand Index
50	m	13-16 years	0.494	810.8616234	100	Grand Index
50	m	13-16 years	0.5	811	100	Grand Index
50	m	13-16 years	0.501	811.9766785	100	Grand Index

Age	Gender	Education	Percentile	Raw Score	Norm Score	Assessment
50	m	13-16 years	0.504	812	100	Grand Index
50	m	13-16 years	0.505	812.28	100	Grand Index
50	m	13-16 years	0.511	813	100	Grand Index
50	m	13-16 years	0.512	813.0711438	100	Grand Index
50	m	13-16 years	0.513	813.9822649	100	Grand Index
50	m	13-16 years	0.517	814	101	Grand Index
50	m	13-16 years	0.52	815	101	Grand Index
50	m	13-16 years	0.521	815.176	101	Grand Index
50	m	13-16 years	0.526	816	101	Grand Index
50	m	13-16 years	0.527	816.0170138	101	Grand Index
50	m	13-16 years	0.531	817	101	Grand Index
50	m	13-16 years	0.532	817.792	101	Grand Index
50	m	13-16 years	0.535	818	101	Grand Index
50	m	13-16 years	0.536	818.016	101	Grand Index
50	m	13-16 years	0.537	819	101	Grand Index
50	m	13-16 years	0.538	819.4355816	101	Grand Index
50	m	13-16 years	0.542	820	102	Grand Index
50	m	13-16 years	0.547	821	102	Grand Index
50	m	13-16 years	0.548	821.2750548	102	Grand Index
50	m	13-16 years	0.549	821.968055	102	Grand Index
50	m	13-16 years	0.551	822	102	Grand Index
50	m	13-16 years	0.552	822.1465146	102	Grand Index
50	m	13-16 years	0.555	823	102	Grand Index
50	m	13-16 years	0.556	823.136	102	Grand Index
50	m	13-16 years	0.561	824	102	Grand Index
50	m	13-16 years	0.562	824.472	102	Grand Index
50	m	13-16 years	0.564	825	102	Grand Index
50	m	13-16 years	0.565	825.8370803	102	Grand Index
50	m	13-16 years	0.57	826	103	Grand Index
50	m	13-16 years	0.571	826.0272854	103	Grand Index
50	m	13-16 years	0.572	826.0447869	103	Grand Index
50	m	13-16 years	0.579	827	103	Grand Index
50	m	13-16 years	0.58	827.3233387	103	Grand Index
50	m	13-16 years	0.586	828	103	Grand Index
50	m	13-16 years	0.587	828.872	103	Grand Index
50	m	13-16 years	0.593	829	104	Grand Index
50	m	13-16 years	0.594	829.264	104	Grand Index
50	m	13-16 years	0.602	830	104	Grand Index
50	m	13-16 years	0.607	831	104	Grand Index
50	m	13-16 years	0.611	832	104	Grand Index
50	m	13-16 years	0.612	832.8597431	104	Grand Index
50	m	13-16 years	0.616	833	104	Grand Index
50	m	13-16 years	0.617	833.0379892	104	Grand Index
50	m	13-16 years	0.618	833.7260331	105	Grand Index
50	m	13-16 years	0.622	834	105	Grand Index
50	m	13-16 years	0.623	834.8561209	105	Grand Index
50	m	13-16 years	0.627	835	105	Grand Index
50	m	13-16 years	0.628	835.168	105	Grand Index
50	m	13-16 years	0.634	836	105	Grand Index
50	m	13-16 years	0.635	836.8425076	105	Grand Index
50	m	13-16 years	0.639	837	105	Grand Index
50	m	13-16 years	0.64	837.1126531	105	Grand Index
50	m	13-16 years	0.641	837.6424221	105	Grand Index
50	m	13-16 years	0.642	837.9863503	105	Grand Index
50	m	13-16 years	0.646	838	106	Grand Index
50	m	13-16 years	0.647	838.2196332	106	Grand Index

Age	Gender	Education	Percentile	Raw Score	Norm Score	Assessment
50	m	13-16 years	0.648	838.9959853	106	Grand Index
50	m	13-16 years	0.653	839	106	Grand Index
50	m	13-16 years	0.658	840	106	Grand Index
50	m	13-16 years	0.659	840.904	106	Grand Index
50	m	13-16 years	0.66	841	106	Grand Index
50	m	13-16 years	0.664	842	106	Grand Index
50	m	13-16 years	0.665	842.2140171	106	Grand Index
50	m	13-16 years	0.666	842.9980811	106	Grand Index
50	m	13-16 years	0.67	843	107	Grand Index
50	m	13-16 years	0.671	843.576	107	Grand Index
50	m	13-16 years	0.672	844	107	Grand Index
50	m	13-16 years	0.673	844.2462356	107	Grand Index
50	m	13-16 years	0.674	844.8575646	107	Grand Index
50	m	13-16 years	0.678	845	107	Grand Index
50	m	13-16 years	0.679	845.4873811	107	Grand Index
50	m	13-16 years	0.681	846	107	Grand Index
50	m	13-16 years	0.682	846.1320382	107	Grand Index
50	m	13-16 years	0.684	847	107	Grand Index
50	m	13-16 years	0.685	847.0018713	107	Grand Index
50	m	13-16 years	0.686	847.3180585	107	Grand Index
50	m	13-16 years	0.687	847.8363384	107	Grand Index
50	m	13-16 years	0.689	848	107	Grand Index
50	m	13-16 years	0.69	848.4596685	107	Grand Index
50	m	13-16 years	0.695	849	108	Grand Index
50	m	13-16 years	0.696	849.5262726	108	Grand Index
50	m	13-16 years	0.702	850	108	Grand Index
50	m	13-16 years	0.703	850.7441905	108	Grand Index
50	m	13-16 years	0.706	851	108	Grand Index
50	m	13-16 years	0.707	851.592	108	Grand Index
50	m	13-16 years	0.711	852	108	Grand Index
50	m	13-16 years	0.714	853	108	Grand Index
50	m	13-16 years	0.715	854	109	Grand Index
50	m	13-16 years	0.716	854.096	109	Grand Index
50	m	13-16 years	0.718	855	109	Grand Index
50	m	13-16 years	0.723	856	109	Grand Index
50	m	13-16 years	0.724	856.0651268	109	Grand Index
50	m	13-16 years	0.725	856.7390511	109	Grand Index
50	m	13-16 years	0.729	857	109	Grand Index
50	m	13-16 years	0.73	857.3374454	109	Grand Index
50	m	13-16 years	0.732	858	109	Grand Index
50	m	13-16 years	0.733	858.0399961	109	Grand Index
50	m	13-16 years	0.734	858.9339745	109	Grand Index
50	m	13-16 years	0.736	859	109	Grand Index
50	m	13-16 years	0.737	859.5326273	110	Grand Index
50	m	13-16 years	0.74	860	110	Grand Index
50	m	13-16 years	0.744	861	110	Grand Index
50	m	13-16 years	0.745	861.5320992	110	Grand Index
50	m	13-16 years	0.746	861.9917754	110	Grand Index
50	m	13-16 years	0.749	862	110	Grand Index
50	m	13-16 years	0.753	863	110	Grand Index
50	m	13-16 years	0.754	863.0688502	110	Grand Index
50	m	13-16 years	0.759	864	111	Grand Index
50	m	13-16 years	0.76	864.56	111	Grand Index
50	m	13-16 years	0.764	865	111	Grand Index
50	m	13-16 years	0.765	865.0889298	111	Grand Index
50	m	13-16 years	0.769	866	111	Grand Index

Age	Gender	Education	Percentile	Raw Score	Norm Score	Assessment
50	m	13-16 years	0.77	866.8670844	111	Grand Index
50	m	13-16 years	0.772	867	111	Grand Index
50	m	13-16 years	0.773	867.288	111	Grand Index
50	m	13-16 years	0.774	868	111	Grand Index
50	m	13-16 years	0.775	868.2446872	111	Grand Index
50	m	13-16 years	0.78	869	112	Grand Index
50	m	13-16 years	0.781	869.9148387	112	Grand Index
50	m	13-16 years	0.783	870	112	Grand Index
50	m	13-16 years	0.784	870.904	112	Grand Index
50	m	13-16 years	0.789	871	112	Grand Index
50	m	13-16 years	0.79	871.3259752	112	Grand Index
50	m	13-16 years	0.792	872	112	Grand Index
50	m	13-16 years	0.793	872.4353306	112	Grand Index
50	m	13-16 years	0.796	873	112	Grand Index
50	m	13-16 years	0.797	873.6470223	112	Grand Index
50	m	13-16 years	0.798	874	113	Grand Index
50	m	13-16 years	0.799	874.744	113	Grand Index
50	m	13-16 years	0.803	875	113	Grand Index
50	m	13-16 years	0.804	875.1163786	113	Grand Index
50	m	13-16 years	0.807	876	113	Grand Index
50	m	13-16 years	0.808	876.2193482	113	Grand Index
50	m	13-16 years	0.81	877	113	Grand Index
50	m	13-16 years	0.811	877.3880597	113	Grand Index
50	m	13-16 years	0.813	878	113	Grand Index
50	m	13-16 years	0.814	878.6011508	113	Grand Index
50	m	13-16 years	0.818	879	114	Grand Index
50	m	13-16 years	0.819	879.8860231	114	Grand Index
50	m	13-16 years	0.821	880	114	Grand Index
50	m	13-16 years	0.822	880.4987962	114	Grand Index
50	m	13-16 years	0.825	881	114	Grand Index
50	m	13-16 years	0.826	881.256	114	Grand Index
50	m	13-16 years	0.829	882	114	Grand Index
50	m	13-16 years	0.832	883	114	Grand Index
50	m	13-16 years	0.833	883.648	114	Grand Index
50	m	13-16 years	0.834	884	115	Grand Index
50	m	13-16 years	0.835	884.8096943	115	Grand Index
50	m	13-16 years	0.836	885	115	Grand Index
50	m	13-16 years	0.838	886	115	Grand Index
50	m	13-16 years	0.839	886.6339926	115	Grand Index
50	m	13-16 years	0.841	887	115	Grand Index
50	m	13-16 years	0.842	887.7525858	115	Grand Index
50	m	13-16 years	0.843	888	115	Grand Index
50	m	13-16 years	0.844	888.2680292	115	Grand Index
50	m	13-16 years	0.846	889	115	Grand Index
50	m	13-16 years	0.849	890	115	Grand Index
50	m	13-16 years	0.85	890.1380452	116	Grand Index
50	m	13-16 years	0.851	890.5683165	116	Grand Index
50	m	13-16 years	0.852	891	116	Grand Index
50	m	13-16 years	0.854	892	116	Grand Index
50	m	13-16 years	0.855	892.1463655	116	Grand Index
50	m	13-16 years	0.857	893	116	Grand Index
50	m	13-16 years	0.858	894.0100315	116	Grand Index
50	m	13-16 years	0.859	895	116	Grand Index
50	m	13-16 years	0.86	895.0677322	116	Grand Index
50	m	13-16 years	0.862	896	116	Grand Index
50	m	13-16 years	0.863	896.5737801	116	Grand Index

Age	Gender	Education	Percentile	Raw Score	Norm Score	Assessment
50	m	13-16 years	0.865	897	117	Grand Index
50	m	13-16 years	0.866	897.7675211	117	Grand Index
50	m	13-16 years	0.867	898	117	Grand Index
50	m	13-16 years	0.868	898.608	117	Grand Index
50	m	13-16 years	0.869	900	117	Grand Index
50	m	13-16 years	0.87	900.6108404	117	Grand Index
50	m	13-16 years	0.871	900.9753626	117	Grand Index
50	m	13-16 years	0.874	901	117	Grand Index
50	m	13-16 years	0.875	901.3497525	117	Grand Index
50	m	13-16 years	0.877	902	117	Grand Index
50	m	13-16 years	0.88	903	118	Grand Index
50	m	13-16 years	0.881	904	118	Grand Index
50	m	13-16 years	0.882	904.392	118	Grand Index
50	m	13-16 years	0.883	905.448	118	Grand Index
50	m	13-16 years	0.885	906	118	Grand Index
50	m	13-16 years	0.886	906.5981398	118	Grand Index
50	m	13-16 years	0.888	907	118	Grand Index
50	m	13-16 years	0.889	907.3385455	118	Grand Index
50	m	13-16 years	0.89	908	118	Grand Index
50	m	13-16 years	0.891	908.8154255	118	Grand Index
50	m	13-16 years	0.892	909	119	Grand Index
50	m	13-16 years	0.893	909.550113	119	Grand Index
50	m	13-16 years	0.894	910.6494858	119	Grand Index
50	m	13-16 years	0.895	910.7996991	119	Grand Index
50	m	13-16 years	0.898	911	119	Grand Index
50	m	13-16 years	0.899	911.9995949	119	Grand Index
50	m	13-16 years	0.903	912	119	Grand Index
50	m	13-16 years	0.904	912.624	120	Grand Index
50	m	13-16 years	0.905	913	120	Grand Index
50	m	13-16 years	0.906	913.4926312	120	Grand Index
50	m	13-16 years	0.907	914	120	Grand Index
50	m	13-16 years	0.908	915	120	Grand Index
50	m	13-16 years	0.909	915.0715155	120	Grand Index
50	m	13-16 years	0.91	915.9771668	120	Grand Index
50	m	13-16 years	0.911	916.016	120	Grand Index
50	m	13-16 years	0.912	917.3322436	120	Grand Index
50	m	13-16 years	0.913	918.0671292	120	Grand Index
50	m	13-16 years	0.914	919	120	Grand Index
50	m	13-16 years	0.915	919.0127853	121	Grand Index
50	m	13-16 years	0.916	920	121	Grand Index
50	m	13-16 years	0.918	921	121	Grand Index
50	m	13-16 years	0.919	921.5370761	121	Grand Index
50	m	13-16 years	0.92	922.0393939	121	Grand Index
50	m	13-16 years	0.921	922.6329505	121	Grand Index
50	m	13-16 years	0.922	923	121	Grand Index
50	m	13-16 years	0.923	923.476351	121	Grand Index
50	m	13-16 years	0.924	924	121	Grand Index
50	m	13-16 years	0.925	924.2189182	122	Grand Index
50	m	13-16 years	0.926	925.2037932	122	Grand Index
50	m	13-16 years	0.927	925.9852162	122	Grand Index
50	m	13-16 years	0.928	926	122	Grand Index
50	m	13-16 years	0.929	926.6873593	122	Grand Index
50	m	13-16 years	0.93	927	122	Grand Index
50	m	13-16 years	0.931	927.136	122	Grand Index
50	m	13-16 years	0.932	928.192	122	Grand Index
50	m	13-16 years	0.933	929.4124524	122	Grand Index

Age	Gender	Education	Percentile	Raw Score	Norm Score	Assessment
50	m	13-16 years	0.935	930	123	Grand Index
50	m	13-16 years	0.936	930.416	123	Grand Index
50	m	13-16 years	0.937	932.472	123	Grand Index
50	m	13-16 years	0.939	933	123	Grand Index
50	m	13-16 years	0.94	934.64	123	Grand Index
50	m	13-16 years	0.941	935.696	123	Grand Index
50	m	13-16 years	0.943	936	124	Grand Index
50	m	13-16 years	0.944	936.884445	124	Grand Index
50	m	13-16 years	0.945	937.7434146	124	Grand Index
50	m	13-16 years	0.946	938	124	Grand Index
50	m	13-16 years	0.947	938.0017552	124	Grand Index
50	m	13-16 years	0.948	938.6878389	124	Grand Index
50	m	13-16 years	0.949	939.1344163	125	Grand Index
50	m	13-16 years	0.95	940	125	Grand Index
50	m	13-16 years	0.951	941.256	125	Grand Index
50	m	13-16 years	0.952	943.624	125	Grand Index
50	m	13-16 years	0.953	945	125	Grand Index
50	m	13-16 years	0.954	945.736397	125	Grand Index
50	m	13-16 years	0.955	946.1793481	125	Grand Index
50	m	13-16 years	0.956	947.4239381	126	Grand Index
50	m	13-16 years	0.957	948.2590949	126	Grand Index
50	m	13-16 years	0.958	948.9536009	126	Grand Index
50	m	13-16 years	0.959	949.8214786	126	Grand Index
50	m	13-16 years	0.96	950	126	Grand Index
50	m	13-16 years	0.961	950.3206199	126	Grand Index
50	m	13-16 years	0.962	952	127	Grand Index
50	m	13-16 years	0.964	953	127	Grand Index
50	m	13-16 years	0.965	953.3169568	127	Grand Index
50	m	13-16 years	0.966	954.10562	127	Grand Index
50	m	13-16 years	0.967	956	128	Grand Index
50	m	13-16 years	0.968	956.188084	128	Grand Index
50	m	13-16 years	0.97	957	128	Grand Index
50	m	13-16 years	0.971	957.6995197	128	Grand Index
50	m	13-16 years	0.972	961.0535094	129	Grand Index
50	m	13-16 years	0.973	964.5541245	129	Grand Index
50	m	13-16 years	0.974	966.9502216	129	Grand Index
50	m	13-16 years	0.975	967	129	Grand Index
50	m	13-16 years	0.976	968.656	130	Grand Index
50	m	13-16 years	0.977	969.9770435	130	Grand Index
50	m	13-16 years	0.978	971.1031731	130	Grand Index
50	m	13-16 years	0.979	971.9322215	131	Grand Index
50	m	13-16 years	0.98	974.7185234	131	Grand Index
50	m	13-16 years	0.981	976.803338	131	Grand Index
50	m	13-16 years	0.982	977	131	Grand Index
50	m	13-16 years	0.983	979.048	132	Grand Index
50	m	13-16 years	0.984	981.0376916	132	Grand Index
50	m	13-16 years	0.985	981.9134426	133	Grand Index
50	m	13-16 years	0.986	982.0231508	133	Grand Index
50	m	13-16 years	0.987	988.5722827	133	Grand Index
50	m	13-16 years	0.988	989	134	Grand Index
50	m	13-16 years	0.989	990.1636317	134	Grand Index
50	m	13-16 years	0.99	990.7216766	135	Grand Index
50	m	13-16 years	0.991	992	135	Grand Index
50	m	13-16 years	0.992	993.9279134	136	Grand Index
50	m	13-16 years	0.993	997.0805071	137	Grand Index
50	m	13-16 years	0.994	998.8983288	138	Grand Index

Age	Gender	Education	Percentile	Raw Score	Norm Score	Assessment
50	m	13-16 years	0.995	1002	139	Grand Index
50	m	13-16 years	0.996	1003.915003	140	Grand Index
50	m	13-16 years	0.997	1007.753109	141	Grand Index
50	m	13-16 years	0.998	1010.337974	143	Grand Index
50	m	13-16 years	0.999	1035.904268	146	Grand Index
50	f	17+ years	0.001	606.2822299	54	Grand Index
50	f	17+ years	0.002	614	57	Grand Index
50	f	17+ years	0.003	617.9934962	59	Grand Index
50	f	17+ years	0.004	627.928	60	Grand Index
50	f	17+ years	0.005	628.4814195	61	Grand Index
50	f	17+ years	0.006	631.488	62	Grand Index
50	f	17+ years	0.007	635.208	63	Grand Index
50	f	17+ years	0.008	637.9839437	64	Grand Index
50	f	17+ years	0.009	641	65	Grand Index
50	f	17+ years	0.01	641.5008053	65	Grand Index
50	f	17+ years	0.011	645.139971	66	Grand Index
50	f	17+ years	0.012	646.2025043	66	Grand Index
50	f	17+ years	0.013	649.224	67	Grand Index
50	f	17+ years	0.014	652.6644486	67	Grand Index
50	f	17+ years	0.015	654	67	Grand Index
50	f	17+ years	0.016	654.1171947	68	Grand Index
50	f	17+ years	0.017	657.229104	68	Grand Index
50	f	17+ years	0.018	659	69	Grand Index
50	f	17+ years	0.019	660	69	Grand Index
50	f	17+ years	0.02	662.3116279	69	Grand Index
50	f	17+ years	0.021	663.007364	69	Grand Index
50	f	17+ years	0.022	665.456	70	Grand Index
50	f	17+ years	0.023	667.2547293	70	Grand Index
50	f	17+ years	0.024	668.2973825	70	Grand Index
50	f	17+ years	0.025	670.2	71	Grand Index
50	f	17+ years	0.026	672.9295256	71	Grand Index
50	f	17+ years	0.027	674	71	Grand Index
50	f	17+ years	0.028	676	71	Grand Index
50	f	17+ years	0.029	677.192	72	Grand Index
50	f	17+ years	0.03	678.6324013	72	Grand Index
50	f	17+ years	0.031	679.3200511	72	Grand Index
50	f	17+ years	0.032	680.9097705	72	Grand Index
50	f	17+ years	0.033	681	72	Grand Index
50	f	17+ years	0.034	682	73	Grand Index
50	f	17+ years	0.035	682.68	73	Grand Index
50	f	17+ years	0.036	684	73	Grand Index
50	f	17+ years	0.037	684.3604519	73	Grand Index
50	f	17+ years	0.038	685	73	Grand Index
50	f	17+ years	0.039	685.672	74	Grand Index
50	f	17+ years	0.04	686.92	74	Grand Index
50	f	17+ years	0.041	687.1645998	74	Grand Index
50	f	17+ years	0.042	688	74	Grand Index
50	f	17+ years	0.043	688.0067269	74	Grand Index
50	f	17+ years	0.044	688.8795013	74	Grand Index
50	f	17+ years	0.045	689.0566549	75	Grand Index
50	f	17+ years	0.046	690	75	Grand Index
50	f	17+ years	0.047	690.6555902	75	Grand Index
50	f	17+ years	0.048	691	75	Grand Index
50	f	17+ years	0.049	694.152	75	Grand Index
50	f	17+ years	0.05	695.4146379	75	Grand Index
50	f	17+ years	0.051	696	75	Grand Index

Age	Gender	Education	Percentile	Raw Score	Norm Score	Assessment
50	f	17+ years	0.052	697.4571615	76	Grand Index
50	f	17+ years	0.053	698	76	Grand Index
50	f	17+ years	0.054	698.392	76	Grand Index
50	f	17+ years	0.056	699	76	Grand Index
50	f	17+ years	0.057	699.1379739	76	Grand Index
50	f	17+ years	0.058	700	76	Grand Index
50	f	17+ years	0.059	700.6321222	77	Grand Index
50	f	17+ years	0.06	701	77	Grand Index
50	f	17+ years	0.061	701.128	77	Grand Index
50	f	17+ years	0.062	702.079212	77	Grand Index
50	f	17+ years	0.063	702.8166605	77	Grand Index
50	f	17+ years	0.064	703	77	Grand Index
50	f	17+ years	0.065	703.1234396	77	Grand Index
50	f	17+ years	0.066	704.0015068	77	Grand Index
50	f	17+ years	0.068	705	78	Grand Index
50	f	17+ years	0.07	706	78	Grand Index
50	f	17+ years	0.071	706.579026	78	Grand Index
50	f	17+ years	0.072	707.9863763	78	Grand Index
50	f	17+ years	0.073	708.0747982	78	Grand Index
50	f	17+ years	0.074	709	78	Grand Index
50	f	17+ years	0.075	710	78	Grand Index
50	f	17+ years	0.076	710.3157017	79	Grand Index
50	f	17+ years	0.077	711	79	Grand Index
50	f	17+ years	0.078	711.1519195	79	Grand Index
50	f	17+ years	0.079	711.8788049	79	Grand Index
50	f	17+ years	0.08	712.9976754	79	Grand Index
50	f	17+ years	0.081	713	79	Grand Index
50	f	17+ years	0.082	714	79	Grand Index
50	f	17+ years	0.083	714.3980023	79	Grand Index
50	f	17+ years	0.086	715	80	Grand Index
50	f	17+ years	0.087	715.3917957	80	Grand Index
50	f	17+ years	0.088	716.3500383	80	Grand Index
50	f	17+ years	0.089	716.9670969	80	Grand Index
50	f	17+ years	0.09	717	80	Grand Index
50	f	17+ years	0.091	717.0996894	80	Grand Index
50	f	17+ years	0.092	718	80	Grand Index
50	f	17+ years	0.093	719	80	Grand Index
50	f	17+ years	0.094	719.2636323	80	Grand Index
50	f	17+ years	0.095	720	80	Grand Index
50	f	17+ years	0.096	720.808	80	Grand Index
50	f	17+ years	0.1	721	81	Grand Index
50	f	17+ years	0.101	721.8680157	81	Grand Index
50	f	17+ years	0.103	722	81	Grand Index
50	f	17+ years	0.104	722.7954708	81	Grand Index
50	f	17+ years	0.105	723.0014267	81	Grand Index
50	f	17+ years	0.106	723.8202053	81	Grand Index
50	f	17+ years	0.107	724	81	Grand Index
50	f	17+ years	0.108	725	81	Grand Index
50	f	17+ years	0.109	725.0037064	82	Grand Index
50	f	17+ years	0.111	726	82	Grand Index
50	f	17+ years	0.113	727	82	Grand Index
50	f	17+ years	0.114	727.2060473	82	Grand Index
50	f	17+ years	0.115	727.9526466	82	Grand Index
50	f	17+ years	0.116	729.768	82	Grand Index
50	f	17+ years	0.117	730.0007099	82	Grand Index
50	f	17+ years	0.118	730.4811016	82	Grand Index

Age	Gender	Education	Percentile	Raw Score	Norm Score	Assessment
50	f	17+ years	0.121	731	82	Grand Index
50	f	17+ years	0.124	732	83	Grand Index
50	f	17+ years	0.125	732.945337	83	Grand Index
50	f	17+ years	0.127	733	83	Grand Index
50	f	17+ years	0.128	733.8998672	83	Grand Index
50	f	17+ years	0.13	734	83	Grand Index
50	f	17+ years	0.131	734.0599002	83	Grand Index
50	f	17+ years	0.132	734.5772771	83	Grand Index
50	f	17+ years	0.133	734.9984608	83	Grand Index
50	f	17+ years	0.134	735	83	Grand Index
50	f	17+ years	0.135	735.2339413	83	Grand Index
50	f	17+ years	0.137	736	84	Grand Index
50	f	17+ years	0.14	737	84	Grand Index
50	f	17+ years	0.141	737.8048229	84	Grand Index
50	f	17+ years	0.143	738	84	Grand Index
50	f	17+ years	0.144	738.1138222	84	Grand Index
50	f	17+ years	0.145	738.9700356	84	Grand Index
50	f	17+ years	0.146	739	84	Grand Index
50	f	17+ years	0.147	739.2808036	84	Grand Index
50	f	17+ years	0.149	740	84	Grand Index
50	f	17+ years	0.15	740.935152	84	Grand Index
50	f	17+ years	0.152	741	85	Grand Index
50	f	17+ years	0.153	741.8943158	85	Grand Index
50	f	17+ years	0.157	742	85	Grand Index
50	f	17+ years	0.158	742.7488731	85	Grand Index
50	f	17+ years	0.16	743	85	Grand Index
50	f	17+ years	0.161	743.6101417	85	Grand Index
50	f	17+ years	0.164	744	85	Grand Index
50	f	17+ years	0.165	745	85	Grand Index
50	f	17+ years	0.166	745.168	85	Grand Index
50	f	17+ years	0.168	746	86	Grand Index
50	f	17+ years	0.169	746.6971485	86	Grand Index
50	f	17+ years	0.175	747	86	Grand Index
50	f	17+ years	0.176	747.3157806	86	Grand Index
50	f	17+ years	0.18	748	86	Grand Index
50	f	17+ years	0.181	748.4867952	86	Grand Index
50	f	17+ years	0.185	749	87	Grand Index
50	f	17+ years	0.186	749.128	87	Grand Index
50	f	17+ years	0.188	750	87	Grand Index
50	f	17+ years	0.189	750.3485001	87	Grand Index
50	f	17+ years	0.191	751	87	Grand Index
50	f	17+ years	0.192	751.4405436	87	Grand Index
50	f	17+ years	0.194	752	87	Grand Index
50	f	17+ years	0.195	752.36	87	Grand Index
50	f	17+ years	0.197	753	87	Grand Index
50	f	17+ years	0.199	754	87	Grand Index
50	f	17+ years	0.203	755	88	Grand Index
50	f	17+ years	0.204	755.592	88	Grand Index
50	f	17+ years	0.208	756	88	Grand Index
50	f	17+ years	0.209	756.9545439	88	Grand Index
50	f	17+ years	0.213	757	88	Grand Index
50	f	17+ years	0.214	757.1264629	88	Grand Index
50	f	17+ years	0.22	758	88	Grand Index
50	f	17+ years	0.224	759	89	Grand Index
50	f	17+ years	0.227	760	89	Grand Index
50	f	17+ years	0.228	760.544	89	Grand Index

Age	Gender	Education	Percentile	Raw Score	Norm Score	Assessment
50	f	17+ years	0.231	761	89	Grand Index
50	f	17+ years	0.232	761.1794614	89	Grand Index
50	f	17+ years	0.233	761.8837416	89	Grand Index
50	f	17+ years	0.236	762	89	Grand Index
50	f	17+ years	0.237	762.9782971	89	Grand Index
50	f	17+ years	0.238	763	89	Grand Index
50	f	17+ years	0.239	763.6616024	89	Grand Index
50	f	17+ years	0.241	764	89	Grand Index
50	f	17+ years	0.242	764.0024308	90	Grand Index
50	f	17+ years	0.243	764.6994232	90	Grand Index
50	f	17+ years	0.245	765	90	Grand Index
50	f	17+ years	0.247	766	90	Grand Index
50	f	17+ years	0.248	766.504	90	Grand Index
50	f	17+ years	0.251	767	90	Grand Index
50	f	17+ years	0.252	767.496	90	Grand Index
50	f	17+ years	0.255	768	90	Grand Index
50	f	17+ years	0.256	768.488	90	Grand Index
50	f	17+ years	0.258	769	90	Grand Index
50	f	17+ years	0.259	769.0001626	90	Grand Index
50	f	17+ years	0.26	769.5155214	90	Grand Index
50	f	17+ years	0.265	770	91	Grand Index
50	f	17+ years	0.266	770.8971854	91	Grand Index
50	f	17+ years	0.271	771	91	Grand Index
50	f	17+ years	0.272	771.9783391	91	Grand Index
50	f	17+ years	0.273	772	91	Grand Index
50	f	17+ years	0.274	772.939391	91	Grand Index
50	f	17+ years	0.28	773	91	Grand Index
50	f	17+ years	0.281	773.0053901	91	Grand Index
50	f	17+ years	0.282	773.9099785	91	Grand Index
50	f	17+ years	0.287	774	92	Grand Index
50	f	17+ years	0.288	774.9972797	92	Grand Index
50	f	17+ years	0.291	775	92	Grand Index
50	f	17+ years	0.292	775.9068986	92	Grand Index
50	f	17+ years	0.296	776	92	Grand Index
50	f	17+ years	0.297	776.5106348	92	Grand Index
50	f	17+ years	0.298	776.9919172	92	Grand Index
50	f	17+ years	0.302	777	92	Grand Index
50	f	17+ years	0.309	778	93	Grand Index
50	f	17+ years	0.31	778.9944655	93	Grand Index
50	f	17+ years	0.314	779	93	Grand Index
50	f	17+ years	0.319	780	93	Grand Index
50	f	17+ years	0.32	780.5115537	93	Grand Index
50	f	17+ years	0.325	781	93	Grand Index
50	f	17+ years	0.326	781.0687389	93	Grand Index
50	f	17+ years	0.33	782	93	Grand Index
50	f	17+ years	0.331	782.0962981	93	Grand Index
50	f	17+ years	0.332	782.8157835	93	Grand Index
50	f	17+ years	0.337	783	94	Grand Index
50	f	17+ years	0.338	783.7672791	94	Grand Index
50	f	17+ years	0.342	784	94	Grand Index
50	f	17+ years	0.345	785	94	Grand Index
50	f	17+ years	0.346	785.0634701	94	Grand Index
50	f	17+ years	0.35	786	94	Grand Index
50	f	17+ years	0.351	786.048	94	Grand Index
50	f	17+ years	0.356	787	94	Grand Index
50	f	17+ years	0.357	787.2943686	95	Grand Index

Age	Gender	Education	Percentile	Raw Score	Norm Score	Assessment
50	f	17+ years	0.358	787.7269706	95	Grand Index
50	f	17+ years	0.362	788	95	Grand Index
50	f	17+ years	0.363	788.0067197	95	Grand Index
50	f	17+ years	0.364	788.5121699	95	Grand Index
50	f	17+ years	0.369	789	95	Grand Index
50	f	17+ years	0.37	789.5564418	95	Grand Index
50	f	17+ years	0.374	790	95	Grand Index
50	f	17+ years	0.375	790.4618273	95	Grand Index
50	f	17+ years	0.376	790.985662	95	Grand Index
50	f	17+ years	0.379	791	95	Grand Index
50	f	17+ years	0.38	791.8718857	95	Grand Index
50	f	17+ years	0.382	792	95	Grand Index
50	f	17+ years	0.383	792.422311	96	Grand Index
50	f	17+ years	0.387	793	96	Grand Index
50	f	17+ years	0.388	793.9267426	96	Grand Index
50	f	17+ years	0.389	794	96	Grand Index
50	f	17+ years	0.39	794.1828382	96	Grand Index
50	f	17+ years	0.391	794.9906014	96	Grand Index
50	f	17+ years	0.393	795	96	Grand Index
50	f	17+ years	0.394	795.0717079	96	Grand Index
50	f	17+ years	0.395	795.9604567	96	Grand Index
50	f	17+ years	0.399	796	96	Grand Index
50	f	17+ years	0.4	796.5429167	96	Grand Index
50	f	17+ years	0.402	797	96	Grand Index
50	f	17+ years	0.403	797.944	96	Grand Index
50	f	17+ years	0.407	798	96	Grand Index
50	f	17+ years	0.408	798.0156266	97	Grand Index
50	f	17+ years	0.409	798.4786401	97	Grand Index
50	f	17+ years	0.41	798.9982611	97	Grand Index
50	f	17+ years	0.414	799	97	Grand Index
50	f	17+ years	0.415	799.5767645	97	Grand Index
50	f	17+ years	0.42	800	97	Grand Index
50	f	17+ years	0.424	801	97	Grand Index
50	f	17+ years	0.425	801.1855385	97	Grand Index
50	f	17+ years	0.426	801.9782064	97	Grand Index
50	f	17+ years	0.431	802	97	Grand Index
50	f	17+ years	0.432	802.1077199	97	Grand Index
50	f	17+ years	0.435	803	98	Grand Index
50	f	17+ years	0.439	804	98	Grand Index
50	f	17+ years	0.44	804.7200063	98	Grand Index
50	f	17+ years	0.441	804.9368929	98	Grand Index
50	f	17+ years	0.445	805	98	Grand Index
50	f	17+ years	0.45	806	98	Grand Index
50	f	17+ years	0.451	806.7150191	98	Grand Index
50	f	17+ years	0.456	807	98	Grand Index
50	f	17+ years	0.457	807.0503848	98	Grand Index
50	f	17+ years	0.458	807.6548356	98	Grand Index
50	f	17+ years	0.459	807.9951748	98	Grand Index
50	f	17+ years	0.467	808	99	Grand Index
50	f	17+ years	0.468	808.0608618	99	Grand Index
50	f	17+ years	0.473	809	99	Grand Index
50	f	17+ years	0.474	809.4490811	99	Grand Index
50	f	17+ years	0.48	810	99	Grand Index
50	f	17+ years	0.481	810.7463113	99	Grand Index
50	f	17+ years	0.488	811	100	Grand Index
50	f	17+ years	0.489	811.0583323	100	Grand Index

Age	Gender	Education	Percentile	Raw Score	Norm Score	Assessment
50	f	17+ years	0.49	811.9789246	100	Grand Index
50	f	17+ years	0.495	812	100	Grand Index
50	f	17+ years	0.496	812.0066687	100	Grand Index
50	f	17+ years	0.499	813	100	Grand Index
50	f	17+ years	0.5	813.6499303	100	Grand Index
50	f	17+ years	0.501	813.9828991	100	Grand Index
50	f	17+ years	0.508	814	100	Grand Index
50	f	17+ years	0.509	814.232	100	Grand Index
50	f	17+ years	0.515	815	101	Grand Index
50	f	17+ years	0.516	815.968	101	Grand Index
50	f	17+ years	0.522	816	101	Grand Index
50	f	17+ years	0.525	817	101	Grand Index
50	f	17+ years	0.526	817.448	101	Grand Index
50	f	17+ years	0.53	818	101	Grand Index
50	f	17+ years	0.531	818.6398353	101	Grand Index
50	f	17+ years	0.532	818.9952981	101	Grand Index
50	f	17+ years	0.536	819	101	Grand Index
50	f	17+ years	0.537	819.0909011	101	Grand Index
50	f	17+ years	0.544	820	102	Grand Index
50	f	17+ years	0.545	820.1558979	102	Grand Index
50	f	17+ years	0.551	821	102	Grand Index
50	f	17+ years	0.552	821.896	102	Grand Index
50	f	17+ years	0.556	822	102	Grand Index
50	f	17+ years	0.557	822.8684649	102	Grand Index
50	f	17+ years	0.561	823	102	Grand Index
50	f	17+ years	0.562	823.4200501	102	Grand Index
50	f	17+ years	0.57	824	103	Grand Index
50	f	17+ years	0.571	824.608	103	Grand Index
50	f	17+ years	0.577	825	103	Grand Index
50	f	17+ years	0.578	825.5264152	103	Grand Index
50	f	17+ years	0.582	826	103	Grand Index
50	f	17+ years	0.585	827	103	Grand Index
50	f	17+ years	0.586	827.328	103	Grand Index
50	f	17+ years	0.591	828	103	Grand Index
50	f	17+ years	0.592	828.816	103	Grand Index
50	f	17+ years	0.596	829	104	Grand Index
50	f	17+ years	0.597	829.056	104	Grand Index
50	f	17+ years	0.6	830	104	Grand Index
50	f	17+ years	0.601	830.0035316	104	Grand Index
50	f	17+ years	0.602	830.9014672	104	Grand Index
50	f	17+ years	0.607	831	104	Grand Index
50	f	17+ years	0.608	831.9148098	104	Grand Index
50	f	17+ years	0.612	832	104	Grand Index
50	f	17+ years	0.613	832.9312346	104	Grand Index
50	f	17+ years	0.616	833	104	Grand Index
50	f	17+ years	0.617	833.0997828	104	Grand Index
50	f	17+ years	0.618	833.9481441	105	Grand Index
50	f	17+ years	0.624	834	105	Grand Index
50	f	17+ years	0.625	834.5099325	105	Grand Index
50	f	17+ years	0.63	835	105	Grand Index
50	f	17+ years	0.631	835.2090579	105	Grand Index
50	f	17+ years	0.636	836	105	Grand Index
50	f	17+ years	0.637	836.976	105	Grand Index
50	f	17+ years	0.64	837	105	Grand Index
50	f	17+ years	0.641	837.4248219	105	Grand Index
50	f	17+ years	0.642	837.7463813	105	Grand Index

Age	Gender	Education	Percentile	Raw Score	Norm Score	Assessment
50	f	17+ years	0.646	838	106	Grand Index
50	f	17+ years	0.649	839	106	Grand Index
50	f	17+ years	0.652	840	106	Grand Index
50	f	17+ years	0.653	840.944	106	Grand Index
50	f	17+ years	0.655	841	106	Grand Index
50	f	17+ years	0.656	841.639017	106	Grand Index
50	f	17+ years	0.658	842	106	Grand Index
50	f	17+ years	0.659	842.0525163	106	Grand Index
50	f	17+ years	0.66	842.796098	106	Grand Index
50	f	17+ years	0.665	843	106	Grand Index
50	f	17+ years	0.666	843.0952069	106	Grand Index
50	f	17+ years	0.671	844	107	Grand Index
50	f	17+ years	0.672	844.5429978	107	Grand Index
50	f	17+ years	0.677	845	107	Grand Index
50	f	17+ years	0.683	846	107	Grand Index
50	f	17+ years	0.684	846.5016801	107	Grand Index
50	f	17+ years	0.687	847	107	Grand Index
50	f	17+ years	0.688	847.0804355	107	Grand Index
50	f	17+ years	0.692	848	108	Grand Index
50	f	17+ years	0.693	848.9274563	108	Grand Index
50	f	17+ years	0.697	849	108	Grand Index
50	f	17+ years	0.702	850	108	Grand Index
50	f	17+ years	0.703	850.1844358	108	Grand Index
50	f	17+ years	0.704	850.905344	108	Grand Index
50	f	17+ years	0.707	851	108	Grand Index
50	f	17+ years	0.708	851.3744194	108	Grand Index
50	f	17+ years	0.709	851.9928387	108	Grand Index
50	f	17+ years	0.71	852	108	Grand Index
50	f	17+ years	0.711	852.328	108	Grand Index
50	f	17+ years	0.714	853	108	Grand Index
50	f	17+ years	0.715	853.0098089	109	Grand Index
50	f	17+ years	0.716	853.2864869	109	Grand Index
50	f	17+ years	0.721	854	109	Grand Index
50	f	17+ years	0.722	854.0069305	109	Grand Index
50	f	17+ years	0.723	854.8987583	109	Grand Index
50	f	17+ years	0.727	855	109	Grand Index
50	f	17+ years	0.728	855.6404805	109	Grand Index
50	f	17+ years	0.73	856	109	Grand Index
50	f	17+ years	0.731	856.288	109	Grand Index
50	f	17+ years	0.735	857	109	Grand Index
50	f	17+ years	0.736	857.0582733	109	Grand Index
50	f	17+ years	0.739	858	110	Grand Index
50	f	17+ years	0.74	858.1561538	110	Grand Index
50	f	17+ years	0.743	859	110	Grand Index
50	f	17+ years	0.745	860	110	Grand Index
50	f	17+ years	0.746	860.6748469	110	Grand Index
50	f	17+ years	0.748	861	110	Grand Index
50	f	17+ years	0.749	861.752	110	Grand Index
50	f	17+ years	0.751	862	110	Grand Index
50	f	17+ years	0.752	862.0216436	110	Grand Index
50	f	17+ years	0.756	863	110	Grand Index
50	f	17+ years	0.757	863.2452066	110	Grand Index
50	f	17+ years	0.758	863.9913053	110	Grand Index
50	f	17+ years	0.761	864	111	Grand Index
50	f	17+ years	0.762	864.0382866	111	Grand Index
50	f	17+ years	0.767	865	111	Grand Index

Age	Gender	Education	Percentile	Raw Score	Norm Score	Assessment
50	f	17+ years	0.768	865.4057206	111	Grand Index
50	f	17+ years	0.769	865.9693321	111	Grand Index
50	f	17+ years	0.772	866	111	Grand Index
50	f	17+ years	0.773	866.1328407	111	Grand Index
50	f	17+ years	0.775	867	111	Grand Index
50	f	17+ years	0.776	867.448	111	Grand Index
50	f	17+ years	0.78	868	112	Grand Index
50	f	17+ years	0.783	869	112	Grand Index
50	f	17+ years	0.784	869.473572	112	Grand Index
50	f	17+ years	0.788	870	112	Grand Index
50	f	17+ years	0.789	870.6582624	112	Grand Index
50	f	17+ years	0.791	871	112	Grand Index
50	f	17+ years	0.792	871.4431402	112	Grand Index
50	f	17+ years	0.798	872	113	Grand Index
50	f	17+ years	0.799	872.9197838	113	Grand Index
50	f	17+ years	0.802	873	113	Grand Index
50	f	17+ years	0.803	873.400727	113	Grand Index
50	f	17+ years	0.806	874	113	Grand Index
50	f	17+ years	0.807	874.2674263	113	Grand Index
50	f	17+ years	0.81	875	113	Grand Index
50	f	17+ years	0.811	875.128	113	Grand Index
50	f	17+ years	0.812	876	113	Grand Index
50	f	17+ years	0.813	876.624	113	Grand Index
50	f	17+ years	0.816	877	114	Grand Index
50	f	17+ years	0.817	877.9819426	114	Grand Index
50	f	17+ years	0.819	878	114	Grand Index
50	f	17+ years	0.821	879	114	Grand Index
50	f	17+ years	0.823	880	114	Grand Index
50	f	17+ years	0.824	880.657442	114	Grand Index
50	f	17+ years	0.826	881	114	Grand Index
50	f	17+ years	0.827	881.0403076	114	Grand Index
50	f	17+ years	0.828	881.8290347	114	Grand Index
50	f	17+ years	0.831	882	114	Grand Index
50	f	17+ years	0.832	882.4350082	114	Grand Index
50	f	17+ years	0.834	883	115	Grand Index
50	f	17+ years	0.835	883.8818349	115	Grand Index
50	f	17+ years	0.837	884	115	Grand Index
50	f	17+ years	0.838	884.6225173	115	Grand Index
50	f	17+ years	0.841	885	115	Grand Index
50	f	17+ years	0.842	885.816	115	Grand Index
50	f	17+ years	0.845	886	115	Grand Index
50	f	17+ years	0.846	886.5106917	115	Grand Index
50	f	17+ years	0.847	887	115	Grand Index
50	f	17+ years	0.848	887.304	115	Grand Index
50	f	17+ years	0.85	888	116	Grand Index
50	f	17+ years	0.851	888.248462	116	Grand Index
50	f	17+ years	0.852	888.7657091	116	Grand Index
50	f	17+ years	0.853	889	116	Grand Index
50	f	17+ years	0.854	889.0656095	116	Grand Index
50	f	17+ years	0.855	889.9288582	116	Grand Index
50	f	17+ years	0.857	890	116	Grand Index
50	f	17+ years	0.858	890.784	116	Grand Index
50	f	17+ years	0.859	891	116	Grand Index
50	f	17+ years	0.86	891.28	116	Grand Index
50	f	17+ years	0.861	892.4514556	116	Grand Index
50	f	17+ years	0.862	892.8715453	116	Grand Index

Age	Gender	Education	Percentile	Raw Score	Norm Score	Assessment
50	f	17+ years	0.863	893	116	Grand Index
50	f	17+ years	0.864	893.272	116	Grand Index
50	f	17+ years	0.865	894.3921227	117	Grand Index
50	f	17+ years	0.866	895	117	Grand Index
50	f	17+ years	0.868	896	117	Grand Index
50	f	17+ years	0.869	896.2438571	117	Grand Index
50	f	17+ years	0.87	896.9877001	117	Grand Index
50	f	17+ years	0.874	897	117	Grand Index
50	f	17+ years	0.877	898	117	Grand Index
50	f	17+ years	0.878	898.3365628	117	Grand Index
50	f	17+ years	0.879	899	118	Grand Index
50	f	17+ years	0.88	899.48	118	Grand Index
50	f	17+ years	0.883	901	118	Grand Index
50	f	17+ years	0.884	902	118	Grand Index
50	f	17+ years	0.885	902.3717675	118	Grand Index
50	f	17+ years	0.887	904	118	Grand Index
50	f	17+ years	0.889	905	118	Grand Index
50	f	17+ years	0.89	905.0182928	118	Grand Index
50	f	17+ years	0.891	906	118	Grand Index
50	f	17+ years	0.892	906.9249722	119	Grand Index
50	f	17+ years	0.895	907	119	Grand Index
50	f	17+ years	0.896	907.1800561	119	Grand Index
50	f	17+ years	0.898	908	119	Grand Index
50	f	17+ years	0.899	908.8206348	119	Grand Index
50	f	17+ years	0.902	909	119	Grand Index
50	f	17+ years	0.903	909.8226961	119	Grand Index
50	f	17+ years	0.905	910	120	Grand Index
50	f	17+ years	0.906	910.688	120	Grand Index
50	f	17+ years	0.907	911.1097561	120	Grand Index
50	f	17+ years	0.909	912	120	Grand Index
50	f	17+ years	0.91	912.68	120	Grand Index
50	f	17+ years	0.911	913	120	Grand Index
50	f	17+ years	0.912	913.7628756	120	Grand Index
50	f	17+ years	0.913	914	120	Grand Index
50	f	17+ years	0.914	914.125853	120	Grand Index
50	f	17+ years	0.915	914.9949864	121	Grand Index
50	f	17+ years	0.916	916	121	Grand Index
50	f	17+ years	0.918	917	121	Grand Index
50	f	17+ years	0.919	917.9996868	121	Grand Index
50	f	17+ years	0.92	918	121	Grand Index
50	f	17+ years	0.922	919	121	Grand Index
50	f	17+ years	0.923	919.0064064	121	Grand Index
50	f	17+ years	0.924	919.8262295	121	Grand Index
50	f	17+ years	0.926	920	122	Grand Index
50	f	17+ years	0.927	920.9263233	122	Grand Index
50	f	17+ years	0.928	921	122	Grand Index
50	f	17+ years	0.929	921.1365357	122	Grand Index
50	f	17+ years	0.93	921.9728916	122	Grand Index
50	f	17+ years	0.931	922	122	Grand Index
50	f	17+ years	0.932	923.136	122	Grand Index
50	f	17+ years	0.933	924	122	Grand Index
50	f	17+ years	0.934	924.0228345	123	Grand Index
50	f	17+ years	0.935	924.9052887	123	Grand Index
50	f	17+ years	0.936	925	123	Grand Index
50	f	17+ years	0.938	926	123	Grand Index
50	f	17+ years	0.939	926.8093732	123	Grand Index

Age	Gender	Education	Percentile	Raw Score	Norm Score	Assessment
50	f	17+ years	0.94	927.9632709	123	Grand Index
50	f	17+ years	0.943	928	124	Grand Index
50	f	17+ years	0.944	928.8423669	124	Grand Index
50	f	17+ years	0.945	929	124	Grand Index
50	f	17+ years	0.946	929.608	124	Grand Index
50	f	17+ years	0.947	930	124	Grand Index
50	f	17+ years	0.948	931.9991568	124	Grand Index
50	f	17+ years	0.949	932.0168598	125	Grand Index
50	f	17+ years	0.95	933.2515983	125	Grand Index
50	f	17+ years	0.951	933.9854244	125	Grand Index
50	f	17+ years	0.952	934.0008412	125	Grand Index
50	f	17+ years	0.953	935.3410545	125	Grand Index
50	f	17+ years	0.954	936	125	Grand Index
50	f	17+ years	0.955	936.4806215	125	Grand Index
50	f	17+ years	0.956	938.0063445	126	Grand Index
50	f	17+ years	0.957	938.8713282	126	Grand Index
50	f	17+ years	0.958	939.613193	126	Grand Index
50	f	17+ years	0.959	940	126	Grand Index
50	f	17+ years	0.96	942	126	Grand Index
50	f	17+ years	0.961	943.2396862	126	Grand Index
50	f	17+ years	0.962	944.3616096	127	Grand Index
50	f	17+ years	0.963	945.0401951	127	Grand Index
50	f	17+ years	0.964	946.0075055	127	Grand Index
50	f	17+ years	0.965	947	127	Grand Index
50	f	17+ years	0.966	948	127	Grand Index
50	f	17+ years	0.967	948.1554137	128	Grand Index
50	f	17+ years	0.968	950.9776433	128	Grand Index
50	f	17+ years	0.969	951.624	128	Grand Index
50	f	17+ years	0.97	953.4785455	128	Grand Index
50	f	17+ years	0.971	955	128	Grand Index
50	f	17+ years	0.972	956.2465577	129	Grand Index
50	f	17+ years	0.973	957.9814783	129	Grand Index
50	f	17+ years	0.974	959.0034871	129	Grand Index
50	f	17+ years	0.975	960	129	Grand Index
50	f	17+ years	0.976	961.0004206	130	Grand Index
50	f	17+ years	0.977	962	130	Grand Index
50	f	17+ years	0.978	963	130	Grand Index
50	f	17+ years	0.979	964	131	Grand Index
50	f	17+ years	0.98	965.04	131	Grand Index
50	f	17+ years	0.981	966.0213134	131	Grand Index
50	f	17+ years	0.982	967.6884707	131	Grand Index
50	f	17+ years	0.983	969.8731861	132	Grand Index
50	f	17+ years	0.984	971	132	Grand Index
50	f	17+ years	0.985	972.9743979	133	Grand Index
50	f	17+ years	0.986	975.0113869	133	Grand Index
50	f	17+ years	0.987	975.8190769	133	Grand Index
50	f	17+ years	0.988	977.8622818	134	Grand Index
50	f	17+ years	0.989	979.9915282	134	Grand Index
50	f	17+ years	0.99	980.9232761	135	Grand Index
50	f	17+ years	0.991	982.8697941	135	Grand Index
50	f	17+ years	0.992	984.0158549	136	Grand Index
50	f	17+ years	0.993	986.528	137	Grand Index
50	f	17+ years	0.994	993.5576338	138	Grand Index
50	f	17+ years	0.995	997.2073873	139	Grand Index
50	f	17+ years	0.996	1002.061385	140	Grand Index
50	f	17+ years	0.997	1015.167077	141	Grand Index

Age	Gender	Education	Percentile	Raw Score	Norm Score	Assessment
50	f	17+ years	0.998	1035.131358	143	Grand Index
50	f	17+ years	0.999	1055.228742	146	Grand Index
50	m	17+ years	0.001	615.582	54	Grand Index
50	m	17+ years	0.002	619.552	57	Grand Index
50	m	17+ years	0.003	628.6531675	59	Grand Index
50	m	17+ years	0.004	633.8697913	60	Grand Index
50	m	17+ years	0.005	633.9998887	61	Grand Index
50	m	17+ years	0.006	634.408918	62	Grand Index
50	m	17+ years	0.007	637.859486	63	Grand Index
50	m	17+ years	0.008	640.6905567	64	Grand Index
50	m	17+ years	0.009	643.984	65	Grand Index
50	m	17+ years	0.01	650.64	65	Grand Index
50	m	17+ years	0.011	653.536	66	Grand Index
50	m	17+ years	0.012	657.3319594	66	Grand Index
50	m	17+ years	0.013	659.0497701	67	Grand Index
50	m	17+ years	0.014	660.716	67	Grand Index
50	m	17+ years	0.015	663.73	67	Grand Index
50	m	17+ years	0.016	666.0292868	68	Grand Index
50	m	17+ years	0.017	667.596	68	Grand Index
50	m	17+ years	0.018	669	69	Grand Index
50	m	17+ years	0.019	669.686	69	Grand Index
50	m	17+ years	0.02	670.88	69	Grand Index
50	m	17+ years	0.021	671.1463953	69	Grand Index
50	m	17+ years	0.022	673.5201266	70	Grand Index
50	m	17+ years	0.023	675.462	70	Grand Index
50	m	17+ years	0.024	676	70	Grand Index
50	m	17+ years	0.025	678.7430407	71	Grand Index
50	m	17+ years	0.026	680	71	Grand Index
50	m	17+ years	0.027	680.0075524	71	Grand Index
50	m	17+ years	0.028	680.0991045	71	Grand Index
50	m	17+ years	0.029	680.6961944	72	Grand Index
50	m	17+ years	0.03	681.82	72	Grand Index
50	m	17+ years	0.031	682.014	72	Grand Index
50	m	17+ years	0.032	683.208	72	Grand Index
50	m	17+ years	0.033	684.4007764	72	Grand Index
50	m	17+ years	0.034	685.4673077	73	Grand Index
50	m	17+ years	0.035	685.9550887	73	Grand Index
50	m	17+ years	0.036	686	73	Grand Index
50	m	17+ years	0.037	687	73	Grand Index
50	m	17+ years	0.038	687.372	73	Grand Index
50	m	17+ years	0.039	688.2533372	74	Grand Index
50	m	17+ years	0.04	689.6274221	74	Grand Index
50	m	17+ years	0.041	690	74	Grand Index
50	m	17+ years	0.042	691	74	Grand Index
50	m	17+ years	0.043	691.0072304	74	Grand Index
50	m	17+ years	0.044	692.0818096	74	Grand Index
50	m	17+ years	0.045	693.0517641	75	Grand Index
50	m	17+ years	0.046	693.9293891	75	Grand Index
50	m	17+ years	0.048	694	75	Grand Index
50	m	17+ years	0.049	694.451167	75	Grand Index
50	m	17+ years	0.05	694.9674903	75	Grand Index
50	m	17+ years	0.051	695	75	Grand Index
50	m	17+ years	0.054	696	76	Grand Index
50	m	17+ years	0.055	696.67	76	Grand Index
50	m	17+ years	0.056	697	76	Grand Index
50	m	17+ years	0.058	698	76	Grand Index

Age	Gender	Education	Percentile	Raw Score	Norm Score	Assessment
50	m	17+ years	0.059	698.446	77	Grand Index
50	m	17+ years	0.06	699.0277098	77	Grand Index
50	m	17+ years	0.061	699.8411872	77	Grand Index
50	m	17+ years	0.063	701	77	Grand Index
50	m	17+ years	0.064	701.416	77	Grand Index
50	m	17+ years	0.065	702	77	Grand Index
50	m	17+ years	0.066	702.7618202	77	Grand Index
50	m	17+ years	0.067	702.9998951	78	Grand Index
50	m	17+ years	0.068	704.1190759	78	Grand Index
50	m	17+ years	0.069	705.386	78	Grand Index
50	m	17+ years	0.07	706.0115726	78	Grand Index
50	m	17+ years	0.071	706.2748809	78	Grand Index
50	m	17+ years	0.072	706.9791782	78	Grand Index
50	m	17+ years	0.073	707.1546936	78	Grand Index
50	m	17+ years	0.074	707.9709549	78	Grand Index
50	m	17+ years	0.075	708	78	Grand Index
50	m	17+ years	0.076	708.744	79	Grand Index
50	m	17+ years	0.077	709.5279279	79	Grand Index
50	m	17+ years	0.08	710	79	Grand Index
50	m	17+ years	0.081	711.428	79	Grand Index
50	m	17+ years	0.082	712	79	Grand Index
50	m	17+ years	0.083	712.0710551	79	Grand Index
50	m	17+ years	0.084	712.7864195	79	Grand Index
50	m	17+ years	0.085	713.49	79	Grand Index
50	m	17+ years	0.087	714	80	Grand Index
50	m	17+ years	0.088	714.0692712	80	Grand Index
50	m	17+ years	0.089	714.9721811	80	Grand Index
50	m	17+ years	0.091	715	80	Grand Index
50	m	17+ years	0.092	715.848	80	Grand Index
50	m	17+ years	0.094	716	80	Grand Index
50	m	17+ years	0.095	716.43	80	Grand Index
50	m	17+ years	0.096	717.5960209	80	Grand Index
50	m	17+ years	0.097	717.9918394	81	Grand Index
50	m	17+ years	0.098	718.0013272	81	Grand Index
50	m	17+ years	0.099	718.1690768	81	Grand Index
50	m	17+ years	0.1	718.6288464	81	Grand Index
50	m	17+ years	0.101	718.9920442	81	Grand Index
50	m	17+ years	0.102	719	81	Grand Index
50	m	17+ years	0.103	719.982	81	Grand Index
50	m	17+ years	0.104	720.176	81	Grand Index
50	m	17+ years	0.105	721.37	81	Grand Index
50	m	17+ years	0.106	722.564	81	Grand Index
50	m	17+ years	0.107	723.9530324	81	Grand Index
50	m	17+ years	0.108	724.2628766	81	Grand Index
50	m	17+ years	0.109	724.7350206	82	Grand Index
50	m	17+ years	0.11	725	82	Grand Index
50	m	17+ years	0.111	725.534	82	Grand Index
50	m	17+ years	0.113	726	82	Grand Index
50	m	17+ years	0.114	726.0199768	82	Grand Index
50	m	17+ years	0.115	726.4288276	82	Grand Index
50	m	17+ years	0.118	727	82	Grand Index
50	m	17+ years	0.119	727.1345617	82	Grand Index
50	m	17+ years	0.12	728.28	82	Grand Index
50	m	17+ years	0.122	729	83	Grand Index
50	m	17+ years	0.123	729.862	83	Grand Index
50	m	17+ years	0.124	730	83	Grand Index

Age	Gender	Education	Percentile	Raw Score	Norm Score	Assessment
50	m	17+ years	0.125	730.25	83	Grand Index
50	m	17+ years	0.128	731	83	Grand Index
50	m	17+ years	0.13	732	83	Grand Index
50	m	17+ years	0.131	732.0368808	83	Grand Index
50	m	17+ years	0.132	732.642921	83	Grand Index
50	m	17+ years	0.134	733	83	Grand Index
50	m	17+ years	0.137	734	84	Grand Index
50	m	17+ years	0.138	734.0902254	84	Grand Index
50	m	17+ years	0.139	734.7848981	84	Grand Index
50	m	17+ years	0.14	735	84	Grand Index
50	m	17+ years	0.141	735.354	84	Grand Index
50	m	17+ years	0.143	736	84	Grand Index
50	m	17+ years	0.144	736.936	84	Grand Index
50	m	17+ years	0.145	737	84	Grand Index
50	m	17+ years	0.146	737.324	84	Grand Index
50	m	17+ years	0.147	738	84	Grand Index
50	m	17+ years	0.148	738.712	84	Grand Index
50	m	17+ years	0.149	739	84	Grand Index
50	m	17+ years	0.15	739.1567469	84	Grand Index
50	m	17+ years	0.151	740	85	Grand Index
50	m	17+ years	0.152	740.488	85	Grand Index
50	m	17+ years	0.156	741	85	Grand Index
50	m	17+ years	0.157	741.4067126	85	Grand Index
50	m	17+ years	0.158	741.8941337	85	Grand Index
50	m	17+ years	0.159	741.9841992	85	Grand Index
50	m	17+ years	0.161	742	85	Grand Index
50	m	17+ years	0.162	742.1951888	85	Grand Index
50	m	17+ years	0.163	742.7943863	85	Grand Index
50	m	17+ years	0.166	743	85	Grand Index
50	m	17+ years	0.167	743.0030574	86	Grand Index
50	m	17+ years	0.168	743.248997	86	Grand Index
50	m	17+ years	0.169	743.8748761	86	Grand Index
50	m	17+ years	0.172	744	86	Grand Index
50	m	17+ years	0.173	744.562	86	Grand Index
50	m	17+ years	0.175	745	86	Grand Index
50	m	17+ years	0.176	746	86	Grand Index
50	m	17+ years	0.177	746.338	86	Grand Index
50	m	17+ years	0.18	747	86	Grand Index
50	m	17+ years	0.181	747.2108306	86	Grand Index
50	m	17+ years	0.184	748	86	Grand Index
50	m	17+ years	0.185	748.3196438	87	Grand Index
50	m	17+ years	0.187	749	87	Grand Index
50	m	17+ years	0.188	749.472	87	Grand Index
50	m	17+ years	0.19	750	87	Grand Index
50	m	17+ years	0.191	750.0455911	87	Grand Index
50	m	17+ years	0.192	750.882899	87	Grand Index
50	m	17+ years	0.195	751	87	Grand Index
50	m	17+ years	0.201	752	87	Grand Index
50	m	17+ years	0.202	753.188	87	Grand Index
50	m	17+ years	0.206	754	88	Grand Index
50	m	17+ years	0.207	754.6145245	88	Grand Index
50	m	17+ years	0.208	754.7769201	88	Grand Index
50	m	17+ years	0.211	755	88	Grand Index
50	m	17+ years	0.212	756	88	Grand Index
50	m	17+ years	0.213	756.0430339	88	Grand Index
50	m	17+ years	0.214	756.2242722	88	Grand Index

Age	Gender	Education	Percentile	Raw Score	Norm Score	Assessment
50	m	17+ years	0.215	756.7996907	88	Grand Index
50	m	17+ years	0.217	757	88	Grand Index
50	m	17+ years	0.218	757.0073367	88	Grand Index
50	m	17+ years	0.219	757.0292116	88	Grand Index
50	m	17+ years	0.22	757.1182937	88	Grand Index
50	m	17+ years	0.221	757.4953015	88	Grand Index
50	m	17+ years	0.222	757.7692313	89	Grand Index
50	m	17+ years	0.225	758	89	Grand Index
50	m	17+ years	0.226	758.844	89	Grand Index
50	m	17+ years	0.228	759	89	Grand Index
50	m	17+ years	0.229	759.426	89	Grand Index
50	m	17+ years	0.233	760	89	Grand Index
50	m	17+ years	0.234	760.396	89	Grand Index
50	m	17+ years	0.237	761	89	Grand Index
50	m	17+ years	0.242	762	90	Grand Index
50	m	17+ years	0.243	762.1316757	90	Grand Index
50	m	17+ years	0.244	762.9517229	90	Grand Index
50	m	17+ years	0.247	763	90	Grand Index
50	m	17+ years	0.248	763.0020923	90	Grand Index
50	m	17+ years	0.249	763.0908741	90	Grand Index
50	m	17+ years	0.25	763.40262	90	Grand Index
50	m	17+ years	0.251	763.8624939	90	Grand Index
50	m	17+ years	0.253	764	90	Grand Index
50	m	17+ years	0.254	764.0751963	90	Grand Index
50	m	17+ years	0.255	764.6143988	90	Grand Index
50	m	17+ years	0.259	765	90	Grand Index
50	m	17+ years	0.26	765.44	90	Grand Index
50	m	17+ years	0.262	766	90	Grand Index
50	m	17+ years	0.263	766.0048665	90	Grand Index
50	m	17+ years	0.264	766.3454567	91	Grand Index
50	m	17+ years	0.265	766.8798987	91	Grand Index
50	m	17+ years	0.268	767	91	Grand Index
50	m	17+ years	0.269	767.1442634	91	Grand Index
50	m	17+ years	0.27	767.8608781	91	Grand Index
50	m	17+ years	0.276	768	91	Grand Index
50	m	17+ years	0.277	768.3816457	91	Grand Index
50	m	17+ years	0.278	768.9671652	91	Grand Index
50	m	17+ years	0.281	769	91	Grand Index
50	m	17+ years	0.282	769.0348418	91	Grand Index
50	m	17+ years	0.283	769.2596305	91	Grand Index
50	m	17+ years	0.29	770	92	Grand Index
50	m	17+ years	0.291	770.0155045	92	Grand Index
50	m	17+ years	0.292	770.6600211	92	Grand Index
50	m	17+ years	0.294	771	92	Grand Index
50	m	17+ years	0.295	771.1075348	92	Grand Index
50	m	17+ years	0.296	771.6933044	92	Grand Index
50	m	17+ years	0.303	772	92	Grand Index
50	m	17+ years	0.304	772.976	92	Grand Index
50	m	17+ years	0.308	773	92	Grand Index
50	m	17+ years	0.309	773.946	93	Grand Index
50	m	17+ years	0.313	774	93	Grand Index
50	m	17+ years	0.314	774.916	93	Grand Index
50	m	17+ years	0.315	775.11	93	Grand Index
50	m	17+ years	0.319	776	93	Grand Index
50	m	17+ years	0.32	776.6835506	93	Grand Index
50	m	17+ years	0.326	777	93	Grand Index

Age	Gender	Education	Percentile	Raw Score	Norm Score	Assessment
50	m	17+ years	0.327	777.4307953	93	Grand Index
50	m	17+ years	0.328	777.9939467	93	Grand Index
50	m	17+ years	0.331	778	93	Grand Index
50	m	17+ years	0.332	778.0802199	93	Grand Index
50	m	17+ years	0.333	778.6802537	94	Grand Index
50	m	17+ years	0.339	779	94	Grand Index
50	m	17+ years	0.34	779.1314959	94	Grand Index
50	m	17+ years	0.345	780	94	Grand Index
50	m	17+ years	0.346	780.0351584	94	Grand Index
50	m	17+ years	0.347	780.1226418	94	Grand Index
50	m	17+ years	0.348	780.5616342	94	Grand Index
50	m	17+ years	0.349	780.9806325	94	Grand Index
50	m	17+ years	0.351	781	94	Grand Index
50	m	17+ years	0.352	781.0118408	94	Grand Index
50	m	17+ years	0.353	781.3404319	94	Grand Index
50	m	17+ years	0.354	781.8905223	94	Grand Index
50	m	17+ years	0.357	782	95	Grand Index
50	m	17+ years	0.358	782.0341951	95	Grand Index
50	m	17+ years	0.359	782.6727812	95	Grand Index
50	m	17+ years	0.36	783	95	Grand Index
50	m	17+ years	0.361	783.034	95	Grand Index
50	m	17+ years	0.366	784	95	Grand Index
50	m	17+ years	0.367	784.198	95	Grand Index
50	m	17+ years	0.371	785	95	Grand Index
50	m	17+ years	0.372	785.0054618	95	Grand Index
50	m	17+ years	0.373	785.3580304	95	Grand Index
50	m	17+ years	0.374	785.969691	95	Grand Index
50	m	17+ years	0.378	786	95	Grand Index
50	m	17+ years	0.379	786.526	95	Grand Index
50	m	17+ years	0.381	787	95	Grand Index
50	m	17+ years	0.382	787.0038735	95	Grand Index
50	m	17+ years	0.383	787.3270342	96	Grand Index
50	m	17+ years	0.388	788	96	Grand Index
50	m	17+ years	0.389	788.3400908	96	Grand Index
50	m	17+ years	0.39	788.7871125	96	Grand Index
50	m	17+ years	0.391	788.8293378	96	Grand Index
50	m	17+ years	0.395	789	96	Grand Index
50	m	17+ years	0.396	789.824	96	Grand Index
50	m	17+ years	0.399	790	96	Grand Index
50	m	17+ years	0.4	790.4995305	96	Grand Index
50	m	17+ years	0.401	790.9655055	96	Grand Index
50	m	17+ years	0.402	791	96	Grand Index
50	m	17+ years	0.403	791.6041329	96	Grand Index
50	m	17+ years	0.407	792	96	Grand Index
50	m	17+ years	0.408	792.109106	97	Grand Index
50	m	17+ years	0.409	792.815443	97	Grand Index
50	m	17+ years	0.415	793	97	Grand Index
50	m	17+ years	0.416	793.6761941	97	Grand Index
50	m	17+ years	0.417	793.9959713	97	Grand Index
50	m	17+ years	0.421	794	97	Grand Index
50	m	17+ years	0.422	794.868	97	Grand Index
50	m	17+ years	0.425	795	97	Grand Index
50	m	17+ years	0.426	795.586574	97	Grand Index
50	m	17+ years	0.427	795.9715553	97	Grand Index
50	m	17+ years	0.431	796	97	Grand Index
50	m	17+ years	0.432	796.7833536	97	Grand Index

Age	Gender	Education	Percentile	Raw Score	Norm Score	Assessment
50	m	17+ years	0.438	797	98	Grand Index
50	m	17+ years	0.443	798	98	Grand Index
50	m	17+ years	0.444	798.9897457	98	Grand Index
50	m	17+ years	0.446	799	98	Grand Index
50	m	17+ years	0.447	799.6030232	98	Grand Index
50	m	17+ years	0.448	799.9859081	98	Grand Index
50	m	17+ years	0.451	800	98	Grand Index
50	m	17+ years	0.452	800.4156117	98	Grand Index
50	m	17+ years	0.453	800.9532822	98	Grand Index
50	m	17+ years	0.456	801	98	Grand Index
50	m	17+ years	0.457	801.658	98	Grand Index
50	m	17+ years	0.459	802	98	Grand Index
50	m	17+ years	0.46	802.24	98	Grand Index
50	m	17+ years	0.463	803	99	Grand Index
50	m	17+ years	0.464	803.0072551	99	Grand Index
50	m	17+ years	0.465	803.5389163	99	Grand Index
50	m	17+ years	0.466	803.8650578	99	Grand Index
50	m	17+ years	0.467	803.9484769	99	Grand Index
50	m	17+ years	0.472	804	99	Grand Index
50	m	17+ years	0.473	805.524	99	Grand Index
50	m	17+ years	0.474	806	99	Grand Index
50	m	17+ years	0.475	806.0032237	99	Grand Index
50	m	17+ years	0.476	806.1287367	99	Grand Index
50	m	17+ years	0.477	806.6919618	99	Grand Index
50	m	17+ years	0.481	807	99	Grand Index
50	m	17+ years	0.482	807.508	99	Grand Index
50	m	17+ years	0.485	808	99	Grand Index
50	m	17+ years	0.486	808.2573878	99	Grand Index
50	m	17+ years	0.487	808.9510861	100	Grand Index
50	m	17+ years	0.488	809	100	Grand Index
50	m	17+ years	0.489	809.866	100	Grand Index
50	m	17+ years	0.494	810	100	Grand Index
50	m	17+ years	0.495	811	100	Grand Index
50	m	17+ years	0.496	811.224	100	Grand Index
50	m	17+ years	0.503	812	100	Grand Index
50	m	17+ years	0.504	812.776	100	Grand Index
50	m	17+ years	0.505	813	100	Grand Index
50	m	17+ years	0.506	813.164	100	Grand Index
50	m	17+ years	0.511	814	100	Grand Index
50	m	17+ years	0.512	814.328	100	Grand Index
50	m	17+ years	0.515	815	101	Grand Index
50	m	17+ years	0.516	815.1004611	101	Grand Index
50	m	17+ years	0.517	815.9761123	101	Grand Index
50	m	17+ years	0.525	816	101	Grand Index
50	m	17+ years	0.526	816.044	101	Grand Index
50	m	17+ years	0.534	817	101	Grand Index
50	m	17+ years	0.535	817.1018762	101	Grand Index
50	m	17+ years	0.536	817.9860633	101	Grand Index
50	m	17+ years	0.543	818	102	Grand Index
50	m	17+ years	0.544	818.536	102	Grand Index
50	m	17+ years	0.55	819	102	Grand Index
50	m	17+ years	0.551	819.894	102	Grand Index
50	m	17+ years	0.556	820	102	Grand Index
50	m	17+ years	0.557	820.058	102	Grand Index
50	m	17+ years	0.558	821	102	Grand Index
50	m	17+ years	0.559	821.446	102	Grand Index

Age	Gender	Education	Percentile	Raw Score	Norm Score	Assessment
50	m	17+ years	0.567	822	103	Grand Index
50	m	17+ years	0.572	823	103	Grand Index
50	m	17+ years	0.577	824	103	Grand Index
50	m	17+ years	0.579	825	103	Grand Index
50	m	17+ years	0.58	825.52	103	Grand Index
50	m	17+ years	0.585	826	103	Grand Index
50	m	17+ years	0.586	826.684	103	Grand Index
50	m	17+ years	0.587	827.878	103	Grand Index
50	m	17+ years	0.594	828	104	Grand Index
50	m	17+ years	0.595	828.4224332	104	Grand Index
50	m	17+ years	0.596	828.9933834	104	Grand Index
50	m	17+ years	0.601	829	104	Grand Index
50	m	17+ years	0.602	829.788	104	Grand Index
50	m	17+ years	0.603	830	104	Grand Index
50	m	17+ years	0.604	830.176	104	Grand Index
50	m	17+ years	0.609	831	104	Grand Index
50	m	17+ years	0.61	831.34	104	Grand Index
50	m	17+ years	0.613	832	104	Grand Index
50	m	17+ years	0.614	832.116	104	Grand Index
50	m	17+ years	0.618	833	105	Grand Index
50	m	17+ years	0.619	833.335927	105	Grand Index
50	m	17+ years	0.624	834	105	Grand Index
50	m	17+ years	0.625	834.25	105	Grand Index
50	m	17+ years	0.626	835	105	Grand Index
50	m	17+ years	0.627	835.638	105	Grand Index
50	m	17+ years	0.629	836	105	Grand Index
50	m	17+ years	0.63	836.22	105	Grand Index
50	m	17+ years	0.634	837	105	Grand Index
50	m	17+ years	0.642	838	105	Grand Index
50	m	17+ years	0.643	838.2245353	105	Grand Index
50	m	17+ years	0.644	838.9553669	106	Grand Index
50	m	17+ years	0.648	839	106	Grand Index
50	m	17+ years	0.649	839.906	106	Grand Index
50	m	17+ years	0.655	840	106	Grand Index
50	m	17+ years	0.656	840.264	106	Grand Index
50	m	17+ years	0.66	841	106	Grand Index
50	m	17+ years	0.661	841.0088652	106	Grand Index
50	m	17+ years	0.662	841.2665808	106	Grand Index
50	m	17+ years	0.663	841.8382993	106	Grand Index
50	m	17+ years	0.666	842	106	Grand Index
50	m	17+ years	0.667	842.398	106	Grand Index
50	m	17+ years	0.671	843	107	Grand Index
50	m	17+ years	0.672	843.368	107	Grand Index
50	m	17+ years	0.675	844	107	Grand Index
50	m	17+ years	0.679	845	107	Grand Index
50	m	17+ years	0.68	845.92	107	Grand Index
50	m	17+ years	0.681	846	107	Grand Index
50	m	17+ years	0.682	846.2862352	107	Grand Index
50	m	17+ years	0.683	846.9648089	107	Grand Index
50	m	17+ years	0.687	847	107	Grand Index
50	m	17+ years	0.688	847.2846822	107	Grand Index
50	m	17+ years	0.689	847.8674489	107	Grand Index
50	m	17+ years	0.694	848	108	Grand Index
50	m	17+ years	0.695	848.83	108	Grand Index
50	m	17+ years	0.699	849	108	Grand Index
50	m	17+ years	0.7	849.1088608	108	Grand Index

Age	Gender	Education	Percentile	Raw Score	Norm Score	Assessment
50	m	17+ years	0.701	849.9948165	108	Grand Index
50	m	17+ years	0.703	850	108	Grand Index
50	m	17+ years	0.704	850.576	108	Grand Index
50	m	17+ years	0.705	851	108	Grand Index
50	m	17+ years	0.706	851.7120408	108	Grand Index
50	m	17+ years	0.711	852	108	Grand Index
50	m	17+ years	0.715	853	109	Grand Index
50	m	17+ years	0.716	853.8361917	109	Grand Index
50	m	17+ years	0.717	854	109	Grand Index
50	m	17+ years	0.718	854.0012961	109	Grand Index
50	m	17+ years	0.719	854.0576608	109	Grand Index
50	m	17+ years	0.72	854.7164637	109	Grand Index
50	m	17+ years	0.722	855	109	Grand Index
50	m	17+ years	0.723	855.0823326	109	Grand Index
50	m	17+ years	0.724	855.6269501	109	Grand Index
50	m	17+ years	0.726	856	109	Grand Index
50	m	17+ years	0.727	856.038	109	Grand Index
50	m	17+ years	0.728	857	109	Grand Index
50	m	17+ years	0.729	857.426	109	Grand Index
50	m	17+ years	0.732	858	109	Grand Index
50	m	17+ years	0.733	858.202	109	Grand Index
50	m	17+ years	0.736	859	109	Grand Index
50	m	17+ years	0.737	859.4684433	110	Grand Index
50	m	17+ years	0.74	860	110	Grand Index
50	m	17+ years	0.741	860.754	110	Grand Index
50	m	17+ years	0.743	861	110	Grand Index
50	m	17+ years	0.744	861.0235364	110	Grand Index
50	m	17+ years	0.745	861.4367259	110	Grand Index
50	m	17+ years	0.746	861.8377489	110	Grand Index
50	m	17+ years	0.747	861.9890667	110	Grand Index
50	m	17+ years	0.748	862.0823442	110	Grand Index
50	m	17+ years	0.749	862.8162401	110	Grand Index
50	m	17+ years	0.751	863	110	Grand Index
50	m	17+ years	0.752	863.5695513	110	Grand Index
50	m	17+ years	0.753	864	110	Grand Index
50	m	17+ years	0.754	864.276	110	Grand Index
50	m	17+ years	0.759	865	111	Grand Index
50	m	17+ years	0.76	865.0800776	111	Grand Index
50	m	17+ years	0.761	865.70061	111	Grand Index
50	m	17+ years	0.764	866	111	Grand Index
50	m	17+ years	0.765	866.1867269	111	Grand Index
50	m	17+ years	0.766	866.6069203	111	Grand Index
50	m	17+ years	0.767	866.9406606	111	Grand Index
50	m	17+ years	0.771	867	111	Grand Index
50	m	17+ years	0.772	867.2488547	111	Grand Index
50	m	17+ years	0.773	867.8615298	111	Grand Index
50	m	17+ years	0.776	868	111	Grand Index
50	m	17+ years	0.777	868.738	111	Grand Index
50	m	17+ years	0.779	869	112	Grand Index
50	m	17+ years	0.78	869.1602696	112	Grand Index
50	m	17+ years	0.781	869.7574094	112	Grand Index
50	m	17+ years	0.783	870	112	Grand Index
50	m	17+ years	0.784	870.192	112	Grand Index
50	m	17+ years	0.786	872	112	Grand Index
50	m	17+ years	0.787	872.2984355	112	Grand Index
50	m	17+ years	0.788	872.9283418	112	Grand Index

Age	Gender	Education	Percentile	Raw Score	Norm Score	Assessment
50	m	17+ years	0.79	873	112	Grand Index
50	m	17+ years	0.791	873.246898	112	Grand Index
50	m	17+ years	0.792	873.8394275	112	Grand Index
50	m	17+ years	0.793	874	112	Grand Index
50	m	17+ years	0.794	874.012	112	Grand Index
50	m	17+ years	0.795	874.4866667	112	Grand Index
50	m	17+ years	0.798	875	113	Grand Index
50	m	17+ years	0.803	876	113	Grand Index
50	m	17+ years	0.804	876.966733	113	Grand Index
50	m	17+ years	0.805	877.7270685	113	Grand Index
50	m	17+ years	0.807	878	113	Grand Index
50	m	17+ years	0.808	878.752	113	Grand Index
50	m	17+ years	0.811	879	113	Grand Index
50	m	17+ years	0.812	879.528	113	Grand Index
50	m	17+ years	0.815	880	113	Grand Index
50	m	17+ years	0.816	880.2806534	114	Grand Index
50	m	17+ years	0.817	880.9614474	114	Grand Index
50	m	17+ years	0.819	881	114	Grand Index
50	m	17+ years	0.821	882	114	Grand Index
50	m	17+ years	0.822	882.468	114	Grand Index
50	m	17+ years	0.828	883	114	Grand Index
50	m	17+ years	0.829	883.826	114	Grand Index
50	m	17+ years	0.83	884	114	Grand Index
50	m	17+ years	0.831	884.214	114	Grand Index
50	m	17+ years	0.832	885	114	Grand Index
50	m	17+ years	0.833	885.4140966	114	Grand Index
50	m	17+ years	0.834	885.9363251	115	Grand Index
50	m	17+ years	0.836	886	115	Grand Index
50	m	17+ years	0.837	886.378	115	Grand Index
50	m	17+ years	0.839	887	115	Grand Index
50	m	17+ years	0.84	887.4628621	115	Grand Index
50	m	17+ years	0.841	888	115	Grand Index
50	m	17+ years	0.842	888.1650112	115	Grand Index
50	m	17+ years	0.843	888.7591699	115	Grand Index
50	m	17+ years	0.844	889	115	Grand Index
50	m	17+ years	0.845	889.5131034	115	Grand Index
50	m	17+ years	0.846	889.8832	115	Grand Index
50	m	17+ years	0.848	890	115	Grand Index
50	m	17+ years	0.849	890.706	115	Grand Index
50	m	17+ years	0.852	891	116	Grand Index
50	m	17+ years	0.853	891.482	116	Grand Index
50	m	17+ years	0.855	892	116	Grand Index
50	m	17+ years	0.856	892.064	116	Grand Index
50	m	17+ years	0.857	893.258	116	Grand Index
50	m	17+ years	0.86	894	116	Grand Index
50	m	17+ years	0.861	894.034	116	Grand Index
50	m	17+ years	0.864	895	116	Grand Index
50	m	17+ years	0.865	896.62	117	Grand Index
50	m	17+ years	0.866	897.0028542	117	Grand Index
50	m	17+ years	0.867	897.7282195	117	Grand Index
50	m	17+ years	0.868	897.8708822	117	Grand Index
50	m	17+ years	0.869	898	117	Grand Index
50	m	17+ years	0.87	898.6255649	117	Grand Index
50	m	17+ years	0.871	898.9948522	117	Grand Index
50	m	17+ years	0.873	899	117	Grand Index
50	m	17+ years	0.874	900.112	117	Grand Index

Age	Gender	Education	Percentile	Raw Score	Norm Score	Assessment
50	m	17+ years	0.875	901	117	Grand Index
50	m	17+ years	0.876	901.667501	117	Grand Index
50	m	17+ years	0.878	902	117	Grand Index
50	m	17+ years	0.879	902.0248684	118	Grand Index
50	m	17+ years	0.88	902.2218013	118	Grand Index
50	m	17+ years	0.881	902.9389117	118	Grand Index
50	m	17+ years	0.883	904	118	Grand Index
50	m	17+ years	0.884	904.496	118	Grand Index
50	m	17+ years	0.885	905	118	Grand Index
50	m	17+ years	0.886	905.0418043	118	Grand Index
50	m	17+ years	0.887	905.7041867	118	Grand Index
50	m	17+ years	0.89	906	118	Grand Index
50	m	17+ years	0.891	906.6536856	118	Grand Index
50	m	17+ years	0.892	907.03857	119	Grand Index
50	m	17+ years	0.893	907.9666704	119	Grand Index
50	m	17+ years	0.894	908.7053817	119	Grand Index
50	m	17+ years	0.896	909	119	Grand Index
50	m	17+ years	0.897	910	119	Grand Index
50	m	17+ years	0.898	910.1064775	119	Grand Index
50	m	17+ years	0.899	911.1103378	119	Grand Index
50	m	17+ years	0.9	912	119	Grand Index
50	m	17+ years	0.901	912.794	119	Grand Index
50	m	17+ years	0.902	913	119	Grand Index
50	m	17+ years	0.903	913.1177562	119	Grand Index
50	m	17+ years	0.904	913.4792457	120	Grand Index
50	m	17+ years	0.905	914.3068752	120	Grand Index
50	m	17+ years	0.906	914.8910571	120	Grand Index
50	m	17+ years	0.907	915	120	Grand Index
50	m	17+ years	0.909	916	120	Grand Index
50	m	17+ years	0.91	916.4747357	120	Grand Index
50	m	17+ years	0.911	916.9678513	120	Grand Index
50	m	17+ years	0.914	917	120	Grand Index
50	m	17+ years	0.915	918.02	121	Grand Index
50	m	17+ years	0.917	919	121	Grand Index
50	m	17+ years	0.918	919.0426228	121	Grand Index
50	m	17+ years	0.919	919.9027899	121	Grand Index
50	m	17+ years	0.922	921	121	Grand Index
50	m	17+ years	0.923	921.8860785	121	Grand Index
50	m	17+ years	0.925	922	122	Grand Index
50	m	17+ years	0.926	922.0711776	122	Grand Index
50	m	17+ years	0.927	923.6939049	122	Grand Index
50	m	17+ years	0.928	926	122	Grand Index
50	m	17+ years	0.929	926.2203216	122	Grand Index
50	m	17+ years	0.93	926.9854271	122	Grand Index
50	m	17+ years	0.932	927	122	Grand Index
50	m	17+ years	0.933	927.002	122	Grand Index
50	m	17+ years	0.935	928	123	Grand Index
50	m	17+ years	0.936	928.242258	123	Grand Index
50	m	17+ years	0.937	928.8700912	123	Grand Index
50	m	17+ years	0.938	929	123	Grand Index
50	m	17+ years	0.939	930.0369433	123	Grand Index
50	m	17+ years	0.94	930.4790797	123	Grand Index
50	m	17+ years	0.941	931.5840549	123	Grand Index
50	m	17+ years	0.942	932.748	124	Grand Index
50	m	17+ years	0.943	933	124	Grand Index
50	m	17+ years	0.944	933.0314755	124	Grand Index

Age	Gender	Education	Percentile	Raw Score	Norm Score	Assessment
50	m	17+ years	0.945	933.7531534	124	Grand Index
50	m	17+ years	0.946	934.9107002	124	Grand Index
50	m	17+ years	0.947	935	124	Grand Index
50	m	17+ years	0.948	935.6819297	124	Grand Index
50	m	17+ years	0.949	936	125	Grand Index
50	m	17+ years	0.95	936.8906642	125	Grand Index
50	m	17+ years	0.951	938.9842536	125	Grand Index
50	m	17+ years	0.952	939.688	125	Grand Index
50	m	17+ years	0.953	940	125	Grand Index
50	m	17+ years	0.954	940.152	125	Grand Index
50	m	17+ years	0.955	942	125	Grand Index
50	m	17+ years	0.956	943.0123276	126	Grand Index
50	m	17+ years	0.957	944.232716	126	Grand Index
50	m	17+ years	0.958	944.8903632	126	Grand Index
50	m	17+ years	0.959	945.0164714	126	Grand Index
50	m	17+ years	0.96	945.5121355	126	Grand Index
50	m	17+ years	0.961	946.434	126	Grand Index
50	m	17+ years	0.962	947	127	Grand Index
50	m	17+ years	0.963	950.288	127	Grand Index
50	m	17+ years	0.964	951.6166195	127	Grand Index
50	m	17+ years	0.965	953	127	Grand Index
50	m	17+ years	0.966	953.3792476	127	Grand Index
50	m	17+ years	0.967	953.9753701	128	Grand Index
50	m	17+ years	0.968	954	128	Grand Index
50	m	17+ years	0.969	958.8259012	128	Grand Index
50	m	17+ years	0.97	961.0073471	128	Grand Index
50	m	17+ years	0.971	962.1475514	128	Grand Index
50	m	17+ years	0.972	964	129	Grand Index
50	m	17+ years	0.973	965.524	129	Grand Index
50	m	17+ years	0.974	966	129	Grand Index
50	m	17+ years	0.975	966.6515716	129	Grand Index
50	m	17+ years	0.976	968.3815001	130	Grand Index
50	m	17+ years	0.977	969.9672404	130	Grand Index
50	m	17+ years	0.978	971.7160052	130	Grand Index
50	m	17+ years	0.979	972.4456558	131	Grand Index
50	m	17+ years	0.98	973.1136908	131	Grand Index
50	m	17+ years	0.981	974.5919325	131	Grand Index
50	m	17+ years	0.982	976	131	Grand Index
50	m	17+ years	0.983	976.702	132	Grand Index
50	m	17+ years	0.984	979.6646008	132	Grand Index
50	m	17+ years	0.985	981	133	Grand Index
50	m	17+ years	0.986	981.1570338	133	Grand Index
50	m	17+ years	0.987	982.8560883	133	Grand Index
50	m	17+ years	0.988	988.2467191	134	Grand Index
50	m	17+ years	0.989	990.8905553	134	Grand Index
50	m	17+ years	0.99	994.0305678	135	Grand Index
50	m	17+ years	0.991	994.6340602	135	Grand Index
50	m	17+ years	0.992	1000.376	136	Grand Index
50	m	17+ years	0.993	1008.926	137	Grand Index
50	m	17+ years	0.994	1018.06733	138	Grand Index
50	m	17+ years	0.995	1020.620151	139	Grand Index
50	m	17+ years	0.996	1028.145368	140	Grand Index
50	m	17+ years	0.997	1030.379516	141	Grand Index
50	m	17+ years	0.998	1034.592251	143	Grand Index
50	m	17+ years	0.999	1049.051207	146	Grand Index
55	f	0-12 years	0.001	590.012	54	Grand Index

Age	Gender	Education	Percentile	Raw Score	Norm Score	Assessment
55	f	0-12 years	0.002	591	57	Grand Index
55	f	0-12 years	0.003	591.072	59	Grand Index
55	f	0-12 years	0.004	593.288	60	Grand Index
55	f	0-12 years	0.005	599.4672096	61	Grand Index
55	f	0-12 years	0.006	606.9364732	62	Grand Index
55	f	0-12 years	0.007	608.8689698	63	Grand Index
55	f	0-12 years	0.008	610.0676188	64	Grand Index
55	f	0-12 years	0.009	621.4086827	65	Grand Index
55	f	0-12 years	0.01	625.24	65	Grand Index
55	f	0-12 years	0.011	628.98	66	Grand Index
55	f	0-12 years	0.012	642.8491501	66	Grand Index
55	f	0-12 years	0.013	648.068963	67	Grand Index
55	f	0-12 years	0.014	650.008	67	Grand Index
55	f	0-12 years	0.015	655.9	67	Grand Index
55	f	0-12 years	0.016	660.192	68	Grand Index
55	f	0-12 years	0.017	661.5025929	68	Grand Index
55	f	0-12 years	0.018	663.5795336	69	Grand Index
55	f	0-12 years	0.019	664.912	69	Grand Index
55	f	0-12 years	0.02	668.24	69	Grand Index
55	f	0-12 years	0.021	669.252	69	Grand Index
55	f	0-12 years	0.022	670	70	Grand Index
55	f	0-12 years	0.023	670.0055974	70	Grand Index
55	f	0-12 years	0.024	670.5904396	70	Grand Index
55	f	0-12 years	0.025	672.5983126	71	Grand Index
55	f	0-12 years	0.026	673.995513	71	Grand Index
55	f	0-12 years	0.027	673.9986626	71	Grand Index
55	f	0-12 years	0.028	674.6686344	71	Grand Index
55	f	0-12 years	0.029	675.9934691	72	Grand Index
55	f	0-12 years	0.03	676.36	72	Grand Index
55	f	0-12 years	0.031	677	72	Grand Index
55	f	0-12 years	0.032	677.384	72	Grand Index
55	f	0-12 years	0.033	678	72	Grand Index
55	f	0-12 years	0.034	678.408	73	Grand Index
55	f	0-12 years	0.035	679	73	Grand Index
55	f	0-12 years	0.036	679.2454729	73	Grand Index
55	f	0-12 years	0.037	679.7599327	73	Grand Index
55	f	0-12 years	0.04	680	74	Grand Index
55	f	0-12 years	0.041	681.476	74	Grand Index
55	f	0-12 years	0.042	683.1704252	74	Grand Index
55	f	0-12 years	0.043	685.2276623	74	Grand Index
55	f	0-12 years	0.044	687.528	74	Grand Index
55	f	0-12 years	0.045	688	75	Grand Index
55	f	0-12 years	0.046	688.552	75	Grand Index
55	f	0-12 years	0.047	689.564	75	Grand Index
55	f	0-12 years	0.048	691.1622811	75	Grand Index
55	f	0-12 years	0.049	692.5953538	75	Grand Index
55	f	0-12 years	0.05	693.3509919	75	Grand Index
55	f	0-12 years	0.051	693.8389748	75	Grand Index
55	f	0-12 years	0.052	694	76	Grand Index
55	f	0-12 years	0.053	694.2983989	76	Grand Index
55	f	0-12 years	0.054	695.0907777	76	Grand Index
55	f	0-12 years	0.055	695.7595921	76	Grand Index
55	f	0-12 years	0.056	696.6491016	76	Grand Index
55	f	0-12 years	0.057	698.368	76	Grand Index
55	f	0-12 years	0.058	699.696	76	Grand Index
55	f	0-12 years	0.059	700.0474489	77	Grand Index

Age	Gender	Education	Percentile	Raw Score	Norm Score	Assessment
55	f	0-12 years	0.06	700.6082441	77	Grand Index
55	f	0-12 years	0.061	700.9433893	77	Grand Index
55	f	0-12 years	0.062	701.7411924	77	Grand Index
55	f	0-12 years	0.063	702.4458596	77	Grand Index
55	f	0-12 years	0.064	702.9048246	77	Grand Index
55	f	0-12 years	0.065	704.2796387	77	Grand Index
55	f	0-12 years	0.066	704.925237	77	Grand Index
55	f	0-12 years	0.067	705.7898343	78	Grand Index
55	f	0-12 years	0.068	705.9967581	78	Grand Index
55	f	0-12 years	0.069	706	78	Grand Index
55	f	0-12 years	0.07	706.84	78	Grand Index
55	f	0-12 years	0.071	707.6923296	78	Grand Index
55	f	0-12 years	0.072	707.9745127	78	Grand Index
55	f	0-12 years	0.073	708.592851	78	Grand Index
55	f	0-12 years	0.074	708.9637983	78	Grand Index
55	f	0-12 years	0.075	709.9	78	Grand Index
55	f	0-12 years	0.078	710	79	Grand Index
55	f	0-12 years	0.079	710.948	79	Grand Index
55	f	0-12 years	0.08	711.96	79	Grand Index
55	f	0-12 years	0.081	712	79	Grand Index
55	f	0-12 years	0.082	712.9737286	79	Grand Index
55	f	0-12 years	0.083	712.9999582	79	Grand Index
55	f	0-12 years	0.084	714.0058222	79	Grand Index
55	f	0-12 years	0.085	716.8282987	79	Grand Index
55	f	0-12 years	0.086	717	80	Grand Index
55	f	0-12 years	0.087	717.0732231	80	Grand Index
55	f	0-12 years	0.088	718.6829692	80	Grand Index
55	f	0-12 years	0.089	719.068	80	Grand Index
55	f	0-12 years	0.091	720	80	Grand Index
55	f	0-12 years	0.092	720.104	80	Grand Index
55	f	0-12 years	0.093	721.116	80	Grand Index
55	f	0-12 years	0.096	722	80	Grand Index
55	f	0-12 years	0.097	722.164	81	Grand Index
55	f	0-12 years	0.098	723	81	Grand Index
55	f	0-12 years	0.099	723.188	81	Grand Index
55	f	0-12 years	0.1	724	81	Grand Index
55	f	0-12 years	0.101	724.189178	81	Grand Index
55	f	0-12 years	0.102	725.1404627	81	Grand Index
55	f	0-12 years	0.103	726.236	81	Grand Index
55	f	0-12 years	0.105	727	81	Grand Index
55	f	0-12 years	0.106	727.272	81	Grand Index
55	f	0-12 years	0.107	728	81	Grand Index
55	f	0-12 years	0.108	728.2938689	81	Grand Index
55	f	0-12 years	0.109	728.9950178	82	Grand Index
55	f	0-12 years	0.112	729	82	Grand Index
55	f	0-12 years	0.113	729.356	82	Grand Index
55	f	0-12 years	0.115	730	82	Grand Index
55	f	0-12 years	0.116	730.392	82	Grand Index
55	f	0-12 years	0.119	731	82	Grand Index
55	f	0-12 years	0.12	731.44	82	Grand Index
55	f	0-12 years	0.122	732	83	Grand Index
55	f	0-12 years	0.123	732.4614582	83	Grand Index
55	f	0-12 years	0.124	732.9843585	83	Grand Index
55	f	0-12 years	0.125	733	83	Grand Index
55	f	0-12 years	0.126	733.512	83	Grand Index
55	f	0-12 years	0.127	734	83	Grand Index

Age	Gender	Education	Percentile	Raw Score	Norm Score	Assessment
55	f	0-12 years	0.128	734.536	83	Grand Index
55	f	0-12 years	0.131	735	83	Grand Index
55	f	0-12 years	0.132	735.584	83	Grand Index
55	f	0-12 years	0.137	736	84	Grand Index
55	f	0-12 years	0.138	736.6069405	84	Grand Index
55	f	0-12 years	0.139	736.9751711	84	Grand Index
55	f	0-12 years	0.14	737.68	84	Grand Index
55	f	0-12 years	0.143	738	84	Grand Index
55	f	0-12 years	0.144	738.010502	84	Grand Index
55	f	0-12 years	0.145	738.7046833	84	Grand Index
55	f	0-12 years	0.146	738.9610065	84	Grand Index
55	f	0-12 years	0.147	738.9918716	84	Grand Index
55	f	0-12 years	0.148	739	84	Grand Index
55	f	0-12 years	0.149	739.788	84	Grand Index
55	f	0-12 years	0.151	740	85	Grand Index
55	f	0-12 years	0.152	740.2604497	85	Grand Index
55	f	0-12 years	0.153	740.6482293	85	Grand Index
55	f	0-12 years	0.154	740.9564349	85	Grand Index
55	f	0-12 years	0.155	741	85	Grand Index
55	f	0-12 years	0.156	741.872	85	Grand Index
55	f	0-12 years	0.157	742	85	Grand Index
55	f	0-12 years	0.158	742.896	85	Grand Index
55	f	0-12 years	0.162	743	85	Grand Index
55	f	0-12 years	0.163	743.956	85	Grand Index
55	f	0-12 years	0.166	744	85	Grand Index
55	f	0-12 years	0.167	744.004	86	Grand Index
55	f	0-12 years	0.168	745	86	Grand Index
55	f	0-12 years	0.169	745.028	86	Grand Index
55	f	0-12 years	0.172	746	86	Grand Index
55	f	0-12 years	0.173	746.0774837	86	Grand Index
55	f	0-12 years	0.174	747.1058048	86	Grand Index
55	f	0-12 years	0.177	748	86	Grand Index
55	f	0-12 years	0.178	748.1156173	86	Grand Index
55	f	0-12 years	0.179	748.8723082	86	Grand Index
55	f	0-12 years	0.18	749	86	Grand Index
55	f	0-12 years	0.181	749.0063492	86	Grand Index
55	f	0-12 years	0.182	749.2141217	86	Grand Index
55	f	0-12 years	0.183	750	86	Grand Index
55	f	0-12 years	0.184	750.208	86	Grand Index
55	f	0-12 years	0.188	751	87	Grand Index
55	f	0-12 years	0.189	751.1078143	87	Grand Index
55	f	0-12 years	0.19	751.4438039	87	Grand Index
55	f	0-12 years	0.191	751.6817881	87	Grand Index
55	f	0-12 years	0.193	752	87	Grand Index
55	f	0-12 years	0.194	752.328	87	Grand Index
55	f	0-12 years	0.195	753	87	Grand Index
55	f	0-12 years	0.196	753.352	87	Grand Index
55	f	0-12 years	0.199	754	87	Grand Index
55	f	0-12 years	0.2	754.4	87	Grand Index
55	f	0-12 years	0.201	755.412	87	Grand Index
55	f	0-12 years	0.206	756	88	Grand Index
55	f	0-12 years	0.207	756.2056613	88	Grand Index
55	f	0-12 years	0.208	756.4908027	88	Grand Index
55	f	0-12 years	0.209	756.782412	88	Grand Index
55	f	0-12 years	0.211	757	88	Grand Index
55	f	0-12 years	0.212	757.544	88	Grand Index

Age	Gender	Education	Percentile	Raw Score	Norm Score	Assessment
55	f	0-12 years	0.213	758.0730161	88	Grand Index
55	f	0-12 years	0.214	758.6247319	88	Grand Index
55	f	0-12 years	0.217	759	88	Grand Index
55	f	0-12 years	0.218	759.616	88	Grand Index
55	f	0-12 years	0.224	760	89	Grand Index
55	f	0-12 years	0.225	760.7	89	Grand Index
55	f	0-12 years	0.229	761	89	Grand Index
55	f	0-12 years	0.23	761.76	89	Grand Index
55	f	0-12 years	0.236	762	89	Grand Index
55	f	0-12 years	0.237	762.844	89	Grand Index
55	f	0-12 years	0.242	763	90	Grand Index
55	f	0-12 years	0.243	763.916	90	Grand Index
55	f	0-12 years	0.245	764	90	Grand Index
55	f	0-12 years	0.246	764.952	90	Grand Index
55	f	0-12 years	0.25	765	90	Grand Index
55	f	0-12 years	0.251	765.0102702	90	Grand Index
55	f	0-12 years	0.252	765.8593098	90	Grand Index
55	f	0-12 years	0.256	766	90	Grand Index
55	f	0-12 years	0.257	766.084	90	Grand Index
55	f	0-12 years	0.26	767	90	Grand Index
55	f	0-12 years	0.261	767.132	90	Grand Index
55	f	0-12 years	0.264	768	91	Grand Index
55	f	0-12 years	0.265	768.18	91	Grand Index
55	f	0-12 years	0.268	769	91	Grand Index
55	f	0-12 years	0.269	769.228	91	Grand Index
55	f	0-12 years	0.27	770	91	Grand Index
55	f	0-12 years	0.271	770.0654354	91	Grand Index
55	f	0-12 years	0.272	770.4551128	91	Grand Index
55	f	0-12 years	0.276	771	91	Grand Index
55	f	0-12 years	0.277	771.0962863	91	Grand Index
55	f	0-12 years	0.278	771.4477969	91	Grand Index
55	f	0-12 years	0.279	771.8340298	91	Grand Index
55	f	0-12 years	0.283	772	91	Grand Index
55	f	0-12 years	0.284	772.408	91	Grand Index
55	f	0-12 years	0.286	773	92	Grand Index
55	f	0-12 years	0.287	773.0043386	92	Grand Index
55	f	0-12 years	0.288	773.4613158	92	Grand Index
55	f	0-12 years	0.293	774	92	Grand Index
55	f	0-12 years	0.294	774.416807	92	Grand Index
55	f	0-12 years	0.295	774.8915761	92	Grand Index
55	f	0-12 years	0.296	774.9904168	92	Grand Index
55	f	0-12 years	0.299	775	92	Grand Index
55	f	0-12 years	0.3	775.0004327	92	Grand Index
55	f	0-12 years	0.301	775.6062053	92	Grand Index
55	f	0-12 years	0.302	775.996268	92	Grand Index
55	f	0-12 years	0.303	776	92	Grand Index
55	f	0-12 years	0.304	776.1812733	92	Grand Index
55	f	0-12 years	0.305	776.6422611	92	Grand Index
55	f	0-12 years	0.306	776.9439163	92	Grand Index
55	f	0-12 years	0.309	777	93	Grand Index
55	f	0-12 years	0.31	777.0039654	93	Grand Index
55	f	0-12 years	0.311	777.733476	93	Grand Index
55	f	0-12 years	0.312	778	93	Grand Index
55	f	0-12 years	0.313	778.756	93	Grand Index
55	f	0-12 years	0.319	779	93	Grand Index
55	f	0-12 years	0.32	779.2627488	93	Grand Index

Age	Gender	Education	Percentile	Raw Score	Norm Score	Assessment
55	f	0-12 years	0.321	779.8982938	93	Grand Index
55	f	0-12 years	0.323	780	93	Grand Index
55	f	0-12 years	0.324	780.888	93	Grand Index
55	f	0-12 years	0.326	781	93	Grand Index
55	f	0-12 years	0.327	781.6890249	93	Grand Index
55	f	0-12 years	0.328	781.9837247	93	Grand Index
55	f	0-12 years	0.33	782	93	Grand Index
55	f	0-12 years	0.331	782.9575044	93	Grand Index
55	f	0-12 years	0.332	782.9997614	93	Grand Index
55	f	0-12 years	0.336	783	94	Grand Index
55	f	0-12 years	0.337	783.044	94	Grand Index
55	f	0-12 years	0.341	784	94	Grand Index
55	f	0-12 years	0.342	784.0326888	94	Grand Index
55	f	0-12 years	0.343	784.3938544	94	Grand Index
55	f	0-12 years	0.344	785	94	Grand Index
55	f	0-12 years	0.345	785.14	94	Grand Index
55	f	0-12 years	0.354	786	94	Grand Index
55	f	0-12 years	0.355	786.0894165	94	Grand Index
55	f	0-12 years	0.356	786.5216863	94	Grand Index
55	f	0-12 years	0.357	786.9981409	95	Grand Index
55	f	0-12 years	0.358	786.9998287	95	Grand Index
55	f	0-12 years	0.366	787	95	Grand Index
55	f	0-12 years	0.367	787.404	95	Grand Index
55	f	0-12 years	0.368	788	95	Grand Index
55	f	0-12 years	0.369	788.428	95	Grand Index
55	f	0-12 years	0.371	789	95	Grand Index
55	f	0-12 years	0.372	789.464	95	Grand Index
55	f	0-12 years	0.373	790	95	Grand Index
55	f	0-12 years	0.374	790.488	95	Grand Index
55	f	0-12 years	0.377	791	95	Grand Index
55	f	0-12 years	0.378	791.0138578	95	Grand Index
55	f	0-12 years	0.379	791.5596861	95	Grand Index
55	f	0-12 years	0.386	792	96	Grand Index
55	f	0-12 years	0.387	792.2201861	96	Grand Index
55	f	0-12 years	0.388	793.320293	96	Grand Index
55	f	0-12 years	0.389	793.9446724	96	Grand Index
55	f	0-12 years	0.393	794	96	Grand Index
55	f	0-12 years	0.394	794.1179763	96	Grand Index
55	f	0-12 years	0.395	794.7821344	96	Grand Index
55	f	0-12 years	0.398	795	96	Grand Index
55	f	0-12 years	0.399	795.757161	96	Grand Index
55	f	0-12 years	0.4	795.9921728	96	Grand Index
55	f	0-12 years	0.405	796	96	Grand Index
55	f	0-12 years	0.406	796.1561729	96	Grand Index
55	f	0-12 years	0.407	796.896071	96	Grand Index
55	f	0-12 years	0.408	796.998976	97	Grand Index
55	f	0-12 years	0.414	797	97	Grand Index
55	f	0-12 years	0.415	797.98	97	Grand Index
55	f	0-12 years	0.419	798	97	Grand Index
55	f	0-12 years	0.42	798.0212905	97	Grand Index
55	f	0-12 years	0.421	798.5565846	97	Grand Index
55	f	0-12 years	0.423	799	97	Grand Index
55	f	0-12 years	0.424	799.088	97	Grand Index
55	f	0-12 years	0.426	800	97	Grand Index
55	f	0-12 years	0.427	800.0875268	97	Grand Index
55	f	0-12 years	0.428	800.7458644	97	Grand Index

Age	Gender	Education	Percentile	Raw Score	Norm Score	Assessment
55	f	0-12 years	0.43	801	97	Grand Index
55	f	0-12 years	0.431	801.172	97	Grand Index
55	f	0-12 years	0.432	802	97	Grand Index
55	f	0-12 years	0.433	802.0141318	97	Grand Index
55	f	0-12 years	0.434	802.0738867	98	Grand Index
55	f	0-12 years	0.435	802.1228572	98	Grand Index
55	f	0-12 years	0.436	802.4290714	98	Grand Index
55	f	0-12 years	0.437	802.9605354	98	Grand Index
55	f	0-12 years	0.441	803	98	Grand Index
55	f	0-12 years	0.442	803.304	98	Grand Index
55	f	0-12 years	0.446	804	98	Grand Index
55	f	0-12 years	0.447	804.2680777	98	Grand Index
55	f	0-12 years	0.448	804.8355617	98	Grand Index
55	f	0-12 years	0.451	805	98	Grand Index
55	f	0-12 years	0.452	805.0280864	98	Grand Index
55	f	0-12 years	0.453	805.072992	98	Grand Index
55	f	0-12 years	0.454	805.4931118	98	Grand Index
55	f	0-12 years	0.457	806	98	Grand Index
55	f	0-12 years	0.458	806.496	98	Grand Index
55	f	0-12 years	0.463	807	99	Grand Index
55	f	0-12 years	0.464	807.2140611	99	Grand Index
55	f	0-12 years	0.465	807.7382846	99	Grand Index
55	f	0-12 years	0.466	808.592	99	Grand Index
55	f	0-12 years	0.474	809	99	Grand Index
55	f	0-12 years	0.475	809.7	99	Grand Index
55	f	0-12 years	0.48	810	99	Grand Index
55	f	0-12 years	0.481	810.0955662	99	Grand Index
55	f	0-12 years	0.482	810.7652608	99	Grand Index
55	f	0-12 years	0.483	810.9881665	99	Grand Index
55	f	0-12 years	0.49	811	100	Grand Index
55	f	0-12 years	0.491	811.4357639	100	Grand Index
55	f	0-12 years	0.492	811.9508984	100	Grand Index
55	f	0-12 years	0.496	812	100	Grand Index
55	f	0-12 years	0.497	812.964	100	Grand Index
55	f	0-12 years	0.502	813	100	Grand Index
55	f	0-12 years	0.503	813.036	100	Grand Index
55	f	0-12 years	0.505	814	100	Grand Index
55	f	0-12 years	0.506	814.072	100	Grand Index
55	f	0-12 years	0.51	815	100	Grand Index
55	f	0-12 years	0.511	815.0195266	100	Grand Index
55	f	0-12 years	0.512	815.152277	100	Grand Index
55	f	0-12 years	0.513	815.3063362	100	Grand Index
55	f	0-12 years	0.517	816	101	Grand Index
55	f	0-12 years	0.518	816.216	101	Grand Index
55	f	0-12 years	0.524	817	101	Grand Index
55	f	0-12 years	0.525	817.3	101	Grand Index
55	f	0-12 years	0.53	818	101	Grand Index
55	f	0-12 years	0.531	818.372	101	Grand Index
55	f	0-12 years	0.532	819	101	Grand Index
55	f	0-12 years	0.533	819.396	101	Grand Index
55	f	0-12 years	0.537	820	101	Grand Index
55	f	0-12 years	0.538	820.084116	101	Grand Index
55	f	0-12 years	0.539	820.5661354	101	Grand Index
55	f	0-12 years	0.541	821	102	Grand Index
55	f	0-12 years	0.542	821.4613169	102	Grand Index
55	f	0-12 years	0.543	821.9590107	102	Grand Index

Age	Gender	Education	Percentile	Raw Score	Norm Score	Assessment
55	f	0-12 years	0.547	822	102	Grand Index
55	f	0-12 years	0.548	822.4291572	102	Grand Index
55	f	0-12 years	0.549	822.8949666	102	Grand Index
55	f	0-12 years	0.551	823	102	Grand Index
55	f	0-12 years	0.552	823.624	102	Grand Index
55	f	0-12 years	0.554	824	102	Grand Index
55	f	0-12 years	0.555	824.465676	102	Grand Index
55	f	0-12 years	0.556	824.9034269	102	Grand Index
55	f	0-12 years	0.56	825	102	Grand Index
55	f	0-12 years	0.561	825.0259023	102	Grand Index
55	f	0-12 years	0.562	825.7530587	102	Grand Index
55	f	0-12 years	0.567	826	103	Grand Index
55	f	0-12 years	0.568	826.3865263	103	Grand Index
55	f	0-12 years	0.569	826.6973086	103	Grand Index
55	f	0-12 years	0.57	826.9108411	103	Grand Index
55	f	0-12 years	0.571	826.9915145	103	Grand Index
55	f	0-12 years	0.577	827	103	Grand Index
55	f	0-12 years	0.578	827.8631132	103	Grand Index
55	f	0-12 years	0.579	827.9878675	103	Grand Index
55	f	0-12 years	0.58	827.9996589	103	Grand Index
55	f	0-12 years	0.583	828	103	Grand Index
55	f	0-12 years	0.588	829	103	Grand Index
55	f	0-12 years	0.589	829.0310437	103	Grand Index
55	f	0-12 years	0.59	829.5000029	103	Grand Index
55	f	0-12 years	0.599	830	104	Grand Index
55	f	0-12 years	0.6	830.0071341	104	Grand Index
55	f	0-12 years	0.601	830.154684	104	Grand Index
55	f	0-12 years	0.602	830.687315	104	Grand Index
55	f	0-12 years	0.606	831	104	Grand Index
55	f	0-12 years	0.607	831.284	104	Grand Index
55	f	0-12 years	0.611	832	104	Grand Index
55	f	0-12 years	0.612	832.344	104	Grand Index
55	f	0-12 years	0.619	833	105	Grand Index
55	f	0-12 years	0.62	833.44	105	Grand Index
55	f	0-12 years	0.621	834.452	105	Grand Index
55	f	0-12 years	0.624	835	105	Grand Index
55	f	0-12 years	0.625	835.4960784	105	Grand Index
55	f	0-12 years	0.626	836.5081725	105	Grand Index
55	f	0-12 years	0.63	837	105	Grand Index
55	f	0-12 years	0.631	837.572	105	Grand Index
55	f	0-12 years	0.633	838	105	Grand Index
55	f	0-12 years	0.634	838.608	105	Grand Index
55	f	0-12 years	0.636	839	105	Grand Index
55	f	0-12 years	0.637	839.644	105	Grand Index
55	f	0-12 years	0.64	840	105	Grand Index
55	f	0-12 years	0.641	840.692	105	Grand Index
55	f	0-12 years	0.642	841	105	Grand Index
55	f	0-12 years	0.643	841.5209808	105	Grand Index
55	f	0-12 years	0.644	841.9259145	106	Grand Index
55	f	0-12 years	0.647	842	106	Grand Index
55	f	0-12 years	0.648	842.776	106	Grand Index
55	f	0-12 years	0.653	843	106	Grand Index
55	f	0-12 years	0.654	843.6163726	106	Grand Index
55	f	0-12 years	0.655	843.9617596	106	Grand Index
55	f	0-12 years	0.658	844	106	Grand Index
55	f	0-12 years	0.659	844.908	106	Grand Index

Age	Gender	Education	Percentile	Raw Score	Norm Score	Assessment
55	f	0-12 years	0.663	845	106	Grand Index
55	f	0-12 years	0.664	845.0996095	106	Grand Index
55	f	0-12 years	0.665	845.982058	106	Grand Index
55	f	0-12 years	0.666	846	106	Grand Index
55	f	0-12 years	0.67	847	107	Grand Index
55	f	0-12 years	0.671	847.052	107	Grand Index
55	f	0-12 years	0.675	848	107	Grand Index
55	f	0-12 years	0.676	848.112	107	Grand Index
55	f	0-12 years	0.677	849.124	107	Grand Index
55	f	0-12 years	0.682	850	107	Grand Index
55	f	0-12 years	0.683	850.0002896	107	Grand Index
55	f	0-12 years	0.684	850.2091701	107	Grand Index
55	f	0-12 years	0.69	851	107	Grand Index
55	f	0-12 years	0.691	851.0376157	107	Grand Index
55	f	0-12 years	0.692	851.3837277	108	Grand Index
55	f	0-12 years	0.693	851.9776538	108	Grand Index
55	f	0-12 years	0.696	852	108	Grand Index
55	f	0-12 years	0.697	852.3399746	108	Grand Index
55	f	0-12 years	0.698	852.9588135	108	Grand Index
55	f	0-12 years	0.704	853	108	Grand Index
55	f	0-12 years	0.705	853.46	108	Grand Index
55	f	0-12 years	0.711	854	108	Grand Index
55	f	0-12 years	0.712	854.2971595	108	Grand Index
55	f	0-12 years	0.713	854.7985346	108	Grand Index
55	f	0-12 years	0.717	855	109	Grand Index
55	f	0-12 years	0.718	855.616	109	Grand Index
55	f	0-12 years	0.719	856	109	Grand Index
55	f	0-12 years	0.72	856.64	109	Grand Index
55	f	0-12 years	0.728	857	109	Grand Index
55	f	0-12 years	0.729	857.748	109	Grand Index
55	f	0-12 years	0.73	858.76	109	Grand Index
55	f	0-12 years	0.731	859.4742043	109	Grand Index
55	f	0-12 years	0.732	859.9166789	109	Grand Index
55	f	0-12 years	0.736	860	109	Grand Index
55	f	0-12 years	0.737	860.844	110	Grand Index
55	f	0-12 years	0.743	861	110	Grand Index
55	f	0-12 years	0.744	861.928	110	Grand Index
55	f	0-12 years	0.749	862	110	Grand Index
55	f	0-12 years	0.753	863	110	Grand Index
55	f	0-12 years	0.754	863.048	110	Grand Index
55	f	0-12 years	0.757	864	110	Grand Index
55	f	0-12 years	0.758	864.0336896	110	Grand Index
55	f	0-12 years	0.759	864.3532255	111	Grand Index
55	f	0-12 years	0.76	864.4474956	111	Grand Index
55	f	0-12 years	0.762	865	111	Grand Index
55	f	0-12 years	0.763	865.156	111	Grand Index
55	f	0-12 years	0.767	866	111	Grand Index
55	f	0-12 years	0.768	866.216	111	Grand Index
55	f	0-12 years	0.772	867	111	Grand Index
55	f	0-12 years	0.773	867.2255602	111	Grand Index
55	f	0-12 years	0.774	867.8338206	111	Grand Index
55	f	0-12 years	0.775	867.9123558	111	Grand Index
55	f	0-12 years	0.777	868	111	Grand Index
55	f	0-12 years	0.778	868.336	111	Grand Index
55	f	0-12 years	0.779	869	112	Grand Index
55	f	0-12 years	0.78	869.36	112	Grand Index

Age	Gender	Education	Percentile	Raw Score	Norm Score	Assessment
55	f	0-12 years	0.782	870	112	Grand Index
55	f	0-12 years	0.783	870.0737016	112	Grand Index
55	f	0-12 years	0.784	870.5181802	112	Grand Index
55	f	0-12 years	0.786	871	112	Grand Index
55	f	0-12 years	0.787	871.444	112	Grand Index
55	f	0-12 years	0.788	872	112	Grand Index
55	f	0-12 years	0.789	872.468	112	Grand Index
55	f	0-12 years	0.79	873	112	Grand Index
55	f	0-12 years	0.791	873.2025882	112	Grand Index
55	f	0-12 years	0.792	873.7082353	112	Grand Index
55	f	0-12 years	0.794	874	112	Grand Index
55	f	0-12 years	0.795	874.54	112	Grand Index
55	f	0-12 years	0.8	875	113	Grand Index
55	f	0-12 years	0.801	875.0043023	113	Grand Index
55	f	0-12 years	0.802	875.0456294	113	Grand Index
55	f	0-12 years	0.803	875.6610753	113	Grand Index
55	f	0-12 years	0.805	876	113	Grand Index
55	f	0-12 years	0.806	876.672	113	Grand Index
55	f	0-12 years	0.807	877	113	Grand Index
55	f	0-12 years	0.808	877.0983459	113	Grand Index
55	f	0-12 years	0.809	877.74926	113	Grand Index
55	f	0-12 years	0.81	878	113	Grand Index
55	f	0-12 years	0.811	878.2241267	113	Grand Index
55	f	0-12 years	0.812	878.8223831	113	Grand Index
55	f	0-12 years	0.815	879	113	Grand Index
55	f	0-12 years	0.816	879.792	114	Grand Index
55	f	0-12 years	0.818	880	114	Grand Index
55	f	0-12 years	0.819	880.7958205	114	Grand Index
55	f	0-12 years	0.82	880.9872701	114	Grand Index
55	f	0-12 years	0.821	880.9988527	114	Grand Index
55	f	0-12 years	0.822	881.864	114	Grand Index
55	f	0-12 years	0.825	882	114	Grand Index
55	f	0-12 years	0.826	882.1630878	114	Grand Index
55	f	0-12 years	0.827	882.9375907	114	Grand Index
55	f	0-12 years	0.828	883	114	Grand Index
55	f	0-12 years	0.829	883.948	114	Grand Index
55	f	0-12 years	0.831	884	114	Grand Index
55	f	0-12 years	0.832	884.984	114	Grand Index
55	f	0-12 years	0.833	885	114	Grand Index
55	f	0-12 years	0.834	885.0004126	115	Grand Index
55	f	0-12 years	0.835	885.0520548	115	Grand Index
55	f	0-12 years	0.836	885.1370502	115	Grand Index
55	f	0-12 years	0.839	887	115	Grand Index
55	f	0-12 years	0.84	887.0205195	115	Grand Index
55	f	0-12 years	0.841	887.3248961	115	Grand Index
55	f	0-12 years	0.845	888	115	Grand Index
55	f	0-12 years	0.846	888.152	115	Grand Index
55	f	0-12 years	0.85	889	116	Grand Index
55	f	0-12 years	0.851	889.0078599	116	Grand Index
55	f	0-12 years	0.852	889.2527703	116	Grand Index
55	f	0-12 years	0.853	890	116	Grand Index
55	f	0-12 years	0.854	890.248	116	Grand Index
55	f	0-12 years	0.855	891	116	Grand Index
55	f	0-12 years	0.856	891.228024	116	Grand Index
55	f	0-12 years	0.857	891.8842395	116	Grand Index
55	f	0-12 years	0.858	892	116	Grand Index

Age	Gender	Education	Percentile	Raw Score	Norm Score	Assessment
55	f	0-12 years	0.859	892.308	116	Grand Index
55	f	0-12 years	0.861	893	116	Grand Index
55	f	0-12 years	0.862	893.344	116	Grand Index
55	f	0-12 years	0.865	894	117	Grand Index
55	f	0-12 years	0.866	894.392	117	Grand Index
55	f	0-12 years	0.869	895	117	Grand Index
55	f	0-12 years	0.87	895.44	117	Grand Index
55	f	0-12 years	0.873	896	117	Grand Index
55	f	0-12 years	0.874	896.2128511	117	Grand Index
55	f	0-12 years	0.875	896.7180851	117	Grand Index
55	f	0-12 years	0.877	897	117	Grand Index
55	f	0-12 years	0.878	897.2963752	117	Grand Index
55	f	0-12 years	0.879	897.7979283	118	Grand Index
55	f	0-12 years	0.88	898	118	Grand Index
55	f	0-12 years	0.881	898.572	118	Grand Index
55	f	0-12 years	0.883	899	118	Grand Index
55	f	0-12 years	0.884	899.5385448	118	Grand Index
55	f	0-12 years	0.885	899.9565905	118	Grand Index
55	f	0-12 years	0.887	900	118	Grand Index
55	f	0-12 years	0.888	900.0539382	118	Grand Index
55	f	0-12 years	0.889	900.2015311	118	Grand Index
55	f	0-12 years	0.89	900.4088965	118	Grand Index
55	f	0-12 years	0.891	900.8394014	118	Grand Index
55	f	0-12 years	0.892	901	119	Grand Index
55	f	0-12 years	0.893	901.1447133	119	Grand Index
55	f	0-12 years	0.894	902.5109749	119	Grand Index
55	f	0-12 years	0.895	903.74	119	Grand Index
55	f	0-12 years	0.897	904	119	Grand Index
55	f	0-12 years	0.898	904.776	119	Grand Index
55	f	0-12 years	0.9	905	119	Grand Index
55	f	0-12 years	0.901	905.2417732	119	Grand Index
55	f	0-12 years	0.902	905.876404	119	Grand Index
55	f	0-12 years	0.903	906.836	119	Grand Index
55	f	0-12 years	0.904	907	120	Grand Index
55	f	0-12 years	0.905	907.0187446	120	Grand Index
55	f	0-12 years	0.906	907.8747899	120	Grand Index
55	f	0-12 years	0.908	908	120	Grand Index
55	f	0-12 years	0.909	909.2550734	120	Grand Index
55	f	0-12 years	0.91	909.9505792	120	Grand Index
55	f	0-12 years	0.912	910	120	Grand Index
55	f	0-12 years	0.913	910.956	120	Grand Index
55	f	0-12 years	0.914	911.968	120	Grand Index
55	f	0-12 years	0.915	912.98	121	Grand Index
55	f	0-12 years	0.916	913	121	Grand Index
55	f	0-12 years	0.917	913.004	121	Grand Index
55	f	0-12 years	0.918	914.0097882	121	Grand Index
55	f	0-12 years	0.919	914.6506353	121	Grand Index
55	f	0-12 years	0.92	916	121	Grand Index
55	f	0-12 years	0.921	916.052	121	Grand Index
55	f	0-12 years	0.922	917	121	Grand Index
55	f	0-12 years	0.923	917.076	121	Grand Index
55	f	0-12 years	0.924	918	121	Grand Index
55	f	0-12 years	0.925	918.1	122	Grand Index
55	f	0-12 years	0.927	919	122	Grand Index
55	f	0-12 years	0.928	919.0725941	122	Grand Index
55	f	0-12 years	0.929	919.6027808	122	Grand Index

Age	Gender	Education	Percentile	Raw Score	Norm Score	Assessment
55	f	0-12 years	0.93	920	122	Grand Index
55	f	0-12 years	0.931	920.172	122	Grand Index
55	f	0-12 years	0.932	921.184	122	Grand Index
55	f	0-12 years	0.933	922.196	122	Grand Index
55	f	0-12 years	0.935	923	123	Grand Index
55	f	0-12 years	0.936	923.232	123	Grand Index
55	f	0-12 years	0.937	924	123	Grand Index
55	f	0-12 years	0.938	924.256	123	Grand Index
55	f	0-12 years	0.94	925	123	Grand Index
55	f	0-12 years	0.941	925.292	123	Grand Index
55	f	0-12 years	0.942	926.304	124	Grand Index
55	f	0-12 years	0.943	927.316	124	Grand Index
55	f	0-12 years	0.944	928.328	124	Grand Index
55	f	0-12 years	0.945	929.34	124	Grand Index
55	f	0-12 years	0.946	930	124	Grand Index
55	f	0-12 years	0.947	930.364	124	Grand Index
55	f	0-12 years	0.948	931.376	124	Grand Index
55	f	0-12 years	0.949	932.388	125	Grand Index
55	f	0-12 years	0.95	934.6	125	Grand Index
55	f	0-12 years	0.951	937	125	Grand Index
55	f	0-12 years	0.952	937.0113725	125	Grand Index
55	f	0-12 years	0.953	937.4511275	125	Grand Index
55	f	0-12 years	0.954	938	125	Grand Index
55	f	0-12 years	0.955	938.46	125	Grand Index
55	f	0-12 years	0.958	939	126	Grand Index
55	f	0-12 years	0.959	939.508	126	Grand Index
55	f	0-12 years	0.96	940.52	126	Grand Index
55	f	0-12 years	0.961	943.128	126	Grand Index
55	f	0-12 years	0.962	946.088	127	Grand Index
55	f	0-12 years	0.963	947	127	Grand Index
55	f	0-12 years	0.964	947.0226749	127	Grand Index
55	f	0-12 years	0.965	947.0418801	127	Grand Index
55	f	0-12 years	0.966	947.1216925	127	Grand Index
55	f	0-12 years	0.967	947.6735853	128	Grand Index
55	f	0-12 years	0.968	948.616	128	Grand Index
55	f	0-12 years	0.969	949.628	128	Grand Index
55	f	0-12 years	0.97	950.097186	128	Grand Index
55	f	0-12 years	0.971	951.4400789	128	Grand Index
55	f	0-12 years	0.972	952.7068936	129	Grand Index
55	f	0-12 years	0.973	953.5669821	129	Grand Index
55	f	0-12 years	0.974	953.9496841	129	Grand Index
55	f	0-12 years	0.975	954.7	129	Grand Index
55	f	0-12 years	0.976	955.3251853	130	Grand Index
55	f	0-12 years	0.977	961.642055	130	Grand Index
55	f	0-12 years	0.978	964.736	130	Grand Index
55	f	0-12 years	0.979	966.496	131	Grand Index
55	f	0-12 years	0.98	967.76	131	Grand Index
55	f	0-12 years	0.981	970.3356375	131	Grand Index
55	f	0-12 years	0.982	971.7894944	131	Grand Index
55	f	0-12 years	0.983	972.5270069	132	Grand Index
55	f	0-12 years	0.984	973.2203268	132	Grand Index
55	f	0-12 years	0.985	976.0641035	133	Grand Index
55	f	0-12 years	0.986	980.1156463	133	Grand Index
55	f	0-12 years	0.987	981.8172405	133	Grand Index
55	f	0-12 years	0.988	985.4722895	134	Grand Index
55	f	0-12 years	0.989	991.2499299	134	Grand Index

Age	Gender	Education	Percentile	Raw Score	Norm Score	Assessment
55	f	0-12 years	0.99	994.6589664	135	Grand Index
55	f	0-12 years	0.991	995.8937416	135	Grand Index
55	f	0-12 years	0.992	997.0038192	136	Grand Index
55	f	0-12 years	0.993	998.8412752	137	Grand Index
55	f	0-12 years	0.994	1007.5379	138	Grand Index
55	f	0-12 years	0.995	1013.677241	139	Grand Index
55	f	0-12 years	0.996	1014.762807	140	Grand Index
55	f	0-12 years	0.997	1014.992797	141	Grand Index
55	f	0-12 years	0.998	1017.84504	143	Grand Index
55	f	0-12 years	0.999	1027.87898	146	Grand Index
55	m	0-12 years	0.001	581.9669797	54	Grand Index
55	m	0-12 years	0.002	601.2740702	57	Grand Index
55	m	0-12 years	0.003	614.052957	59	Grand Index
55	m	0-12 years	0.004	625.2707516	60	Grand Index
55	m	0-12 years	0.005	630.5523072	61	Grand Index
55	m	0-12 years	0.006	632.1025126	62	Grand Index
55	m	0-12 years	0.007	633.9319074	63	Grand Index
55	m	0-12 years	0.008	636.1451876	64	Grand Index
55	m	0-12 years	0.009	638.303807	65	Grand Index
55	m	0-12 years	0.01	640.2774208	65	Grand Index
55	m	0-12 years	0.011	642.2268764	66	Grand Index
55	m	0-12 years	0.012	643.5119825	66	Grand Index
55	m	0-12 years	0.013	644.7970885	67	Grand Index
55	m	0-12 years	0.014	646.8988471	67	Grand Index
55	m	0-12 years	0.015	649.153728	67	Grand Index
55	m	0-12 years	0.016	651.1452196	68	Grand Index
55	m	0-12 years	0.017	652.9943387	68	Grand Index
55	m	0-12 years	0.018	655.8715215	69	Grand Index
55	m	0-12 years	0.019	659.9744726	69	Grand Index
55	m	0-12 years	0.02	663.2379757	69	Grand Index
55	m	0-12 years	0.021	664.1510246	69	Grand Index
55	m	0-12 years	0.022	665.352	70	Grand Index
55	m	0-12 years	0.023	670.368	70	Grand Index
55	m	0-12 years	0.024	675.384	70	Grand Index
55	m	0-12 years	0.025	676.8	71	Grand Index
55	m	0-12 years	0.026	677.712	71	Grand Index
55	m	0-12 years	0.027	678.312	71	Grand Index
55	m	0-12 years	0.028	678.768	71	Grand Index
55	m	0-12 years	0.029	679.672	72	Grand Index
55	m	0-12 years	0.03	681.04	72	Grand Index
55	m	0-12 years	0.031	682.136	72	Grand Index
55	m	0-12 years	0.032	682.592	72	Grand Index
55	m	0-12 years	0.033	683.1510872	72	Grand Index
55	m	0-12 years	0.034	684.5864161	73	Grand Index
55	m	0-12 years	0.035	686.021745	73	Grand Index
55	m	0-12 years	0.036	686.9182282	73	Grand Index
55	m	0-12 years	0.037	687.7628993	73	Grand Index
55	m	0-12 years	0.038	688.984	73	Grand Index
55	m	0-12 years	0.039	690.352	74	Grand Index
55	m	0-12 years	0.04	691.72	74	Grand Index
55	m	0-12 years	0.041	693.088	74	Grand Index
55	m	0-12 years	0.042	694.304	74	Grand Index
55	m	0-12 years	0.043	695.216	74	Grand Index
55	m	0-12 years	0.044	696.448	74	Grand Index
55	m	0-12 years	0.045	699.64	75	Grand Index
55	m	0-12 years	0.046	702.832	75	Grand Index

Age	Gender	Education	Percentile	Raw Score	Norm Score	Assessment
55	m	0-12 years	0.047	703.0182894	75	Grand Index
55	m	0-12 years	0.048	703.0375949	75	Grand Index
55	m	0-12 years	0.049	703.7157728	75	Grand Index
55	m	0-12 years	0.05	704.6084673	75	Grand Index
55	m	0-12 years	0.052	705	76	Grand Index
55	m	0-12 years	0.053	705.168	76	Grand Index
55	m	0-12 years	0.054	705.624	76	Grand Index
55	m	0-12 years	0.055	706.1695793	76	Grand Index
55	m	0-12 years	0.056	707.1361812	76	Grand Index
55	m	0-12 years	0.057	708.1027832	76	Grand Index
55	m	0-12 years	0.058	708.5140971	76	Grand Index
55	m	0-12 years	0.059	708.9154951	77	Grand Index
55	m	0-12 years	0.06	709.7155556	77	Grand Index
55	m	0-12 years	0.061	710.6219259	77	Grand Index
55	m	0-12 years	0.062	710.9910123	77	Grand Index
55	m	0-12 years	0.063	710.996642	77	Grand Index
55	m	0-12 years	0.064	711.184	77	Grand Index
55	m	0-12 years	0.065	711.64	77	Grand Index
55	m	0-12 years	0.066	712.0643992	77	Grand Index
55	m	0-12 years	0.067	712.3702952	78	Grand Index
55	m	0-12 years	0.068	712.6782037	78	Grand Index
55	m	0-12 years	0.069	713.0988057	78	Grand Index
55	m	0-12 years	0.07	713.5194078	78	Grand Index
55	m	0-12 years	0.071	714.4981553	78	Grand Index
55	m	0-12 years	0.072	715.5956572	78	Grand Index
55	m	0-12 years	0.073	716.0411429	78	Grand Index
55	m	0-12 years	0.074	716.1062857	78	Grand Index
55	m	0-12 years	0.075	716.1726898	78	Grand Index
55	m	0-12 years	0.076	716.2407083	79	Grand Index
55	m	0-12 years	0.077	716.3713142	79	Grand Index
55	m	0-12 years	0.078	716.6941529	79	Grand Index
55	m	0-12 years	0.079	717.048	79	Grand Index
55	m	0-12 years	0.08	717.96	79	Grand Index
55	m	0-12 years	0.081	718.872	79	Grand Index
55	m	0-12 years	0.083	719	79	Grand Index
55	m	0-12 years	0.084	719.304	79	Grand Index
55	m	0-12 years	0.085	719.76	79	Grand Index
55	m	0-12 years	0.087	720	80	Grand Index
55	m	0-12 years	0.088	720.1215503	80	Grand Index
55	m	0-12 years	0.089	720.5545735	80	Grand Index
55	m	0-12 years	0.09	720.9516276	80	Grand Index
55	m	0-12 years	0.091	720.9746045	80	Grand Index
55	m	0-12 years	0.092	720.9975814	80	Grand Index
55	m	0-12 years	0.093	721.408	80	Grand Index
55	m	0-12 years	0.094	721.864	80	Grand Index
55	m	0-12 years	0.096	722	80	Grand Index
55	m	0-12 years	0.097	722.232	81	Grand Index
55	m	0-12 years	0.098	722.688	81	Grand Index
55	m	0-12 years	0.1	723	81	Grand Index
55	m	0-12 years	0.101	723.056	81	Grand Index
55	m	0-12 years	0.102	723.512	81	Grand Index
55	m	0-12 years	0.103	723.968	81	Grand Index
55	m	0-12 years	0.104	724.5216051	81	Grand Index
55	m	0-12 years	0.105	725.0825766	81	Grand Index
55	m	0-12 years	0.106	725.416992	81	Grand Index
55	m	0-12 years	0.107	725.6704945	81	Grand Index

Age	Gender	Education	Percentile	Raw Score	Norm Score	Assessment
55	m	0-12 years	0.108	725.8391676	81	Grand Index
55	m	0-12 years	0.109	725.9366936	82	Grand Index
55	m	0-12 years	0.11	726.16	82	Grand Index
55	m	0-12 years	0.111	726.616	82	Grand Index
55	m	0-12 years	0.116	727	82	Grand Index
55	m	0-12 years	0.117	727.352	82	Grand Index
55	m	0-12 years	0.118	727.808	82	Grand Index
55	m	0-12 years	0.119	728.032056	82	Grand Index
55	m	0-12 years	0.12	728.0874256	82	Grand Index
55	m	0-12 years	0.121	728.1589447	82	Grand Index
55	m	0-12 years	0.122	728.2561565	83	Grand Index
55	m	0-12 years	0.123	728.4811625	83	Grand Index
55	m	0-12 years	0.124	729.2405813	83	Grand Index
55	m	0-12 years	0.127	730	83	Grand Index
55	m	0-12 years	0.128	730.368	83	Grand Index
55	m	0-12 years	0.129	730.824	83	Grand Index
55	m	0-12 years	0.13	731.28	83	Grand Index
55	m	0-12 years	0.131	731.736	83	Grand Index
55	m	0-12 years	0.132	732.1670581	83	Grand Index
55	m	0-12 years	0.133	732.5638211	83	Grand Index
55	m	0-12 years	0.134	732.8836045	83	Grand Index
55	m	0-12 years	0.135	732.9428415	83	Grand Index
55	m	0-12 years	0.136	733.032	84	Grand Index
55	m	0-12 years	0.137	733.944	84	Grand Index
55	m	0-12 years	0.138	734.856	84	Grand Index
55	m	0-12 years	0.139	735.384	84	Grand Index
55	m	0-12 years	0.14	735.84	84	Grand Index
55	m	0-12 years	0.141	736.296	84	Grand Index
55	m	0-12 years	0.142	736.752	84	Grand Index
55	m	0-12 years	0.143	737.416	84	Grand Index
55	m	0-12 years	0.144	738.328	84	Grand Index
55	m	0-12 years	0.146	739	84	Grand Index
55	m	0-12 years	0.147	739.032	84	Grand Index
55	m	0-12 years	0.148	739.488	84	Grand Index
55	m	0-12 years	0.149	739.944	84	Grand Index
55	m	0-12 years	0.15	740.4	84	Grand Index
55	m	0-12 years	0.151	740.856	85	Grand Index
55	m	0-12 years	0.152	741.312	85	Grand Index
55	m	0-12 years	0.153	741.768	85	Grand Index
55	m	0-12 years	0.157	742	85	Grand Index
55	m	0-12 years	0.158	742.048	85	Grand Index
55	m	0-12 years	0.159	742.504	85	Grand Index
55	m	0-12 years	0.16	742.96	85	Grand Index
55	m	0-12 years	0.161	743.416	85	Grand Index
55	m	0-12 years	0.162	743.872	85	Grand Index
55	m	0-12 years	0.164	744	85	Grand Index
55	m	0-12 years	0.165	744.24	85	Grand Index
55	m	0-12 years	0.166	744.696	85	Grand Index
55	m	0-12 years	0.173	745	86	Grand Index
55	m	0-12 years	0.174	745.344	86	Grand Index
55	m	0-12 years	0.175	745.8	86	Grand Index
55	m	0-12 years	0.177	746	86	Grand Index
55	m	0-12 years	0.178	746.168	86	Grand Index
55	m	0-12 years	0.179	746.624	86	Grand Index
55	m	0-12 years	0.18	747.0798147	86	Grand Index
55	m	0-12 years	0.181	747.5347584	86	Grand Index

Age	Gender	Education	Percentile	Raw Score	Norm Score	Assessment
55	m	0-12 years	0.182	747.9897021	86	Grand Index
55	m	0-12 years	0.183	747.9987213	86	Grand Index
55	m	0-12 years	0.184	747.9997776	86	Grand Index
55	m	0-12 years	0.186	748	87	Grand Index
55	m	0-12 years	0.187	748.272	87	Grand Index
55	m	0-12 years	0.188	748.728	87	Grand Index
55	m	0-12 years	0.192	749	87	Grand Index
55	m	0-12 years	0.193	749.0078657	87	Grand Index
55	m	0-12 years	0.194	749.4562121	87	Grand Index
55	m	0-12 years	0.195	749.9045585	87	Grand Index
55	m	0-12 years	0.196	750.3655266	87	Grand Index
55	m	0-12 years	0.197	750.8291802	87	Grand Index
55	m	0-12 years	0.203	751	88	Grand Index
55	m	0-12 years	0.204	751.072	88	Grand Index
55	m	0-12 years	0.205	752.44	88	Grand Index
55	m	0-12 years	0.206	753.808	88	Grand Index
55	m	0-12 years	0.208	754	88	Grand Index
55	m	0-12 years	0.209	754.304	88	Grand Index
55	m	0-12 years	0.21	754.76	88	Grand Index
55	m	0-12 years	0.217	755	88	Grand Index
55	m	0-12 years	0.218	755.408	88	Grand Index
55	m	0-12 years	0.219	755.864	88	Grand Index
55	m	0-12 years	0.22	756.1651613	88	Grand Index
55	m	0-12 years	0.221	756.4005161	88	Grand Index
55	m	0-12 years	0.222	756.6283871	89	Grand Index
55	m	0-12 years	0.223	756.8490323	89	Grand Index
55	m	0-12 years	0.224	757.144	89	Grand Index
55	m	0-12 years	0.225	757.6	89	Grand Index
55	m	0-12 years	0.232	758	89	Grand Index
55	m	0-12 years	0.233	758.248	89	Grand Index
55	m	0-12 years	0.234	758.704	89	Grand Index
55	m	0-12 years	0.235	759.2415686	89	Grand Index
55	m	0-12 years	0.236	759.9300392	89	Grand Index
55	m	0-12 years	0.237	760.545098	89	Grand Index
55	m	0-12 years	0.238	760.7686275	89	Grand Index
55	m	0-12 years	0.239	760.9921569	89	Grand Index
55	m	0-12 years	0.241	761	89	Grand Index
55	m	0-12 years	0.242	761.352	90	Grand Index
55	m	0-12 years	0.243	761.808	90	Grand Index
55	m	0-12 years	0.244	762.039384	90	Grand Index
55	m	0-12 years	0.245	762.107411	90	Grand Index
55	m	0-12 years	0.246	762.2989259	90	Grand Index
55	m	0-12 years	0.247	762.6868989	90	Grand Index
55	m	0-12 years	0.254	763	90	Grand Index
55	m	0-12 years	0.255	763.56	90	Grand Index
55	m	0-12 years	0.256	764.472	90	Grand Index
55	m	0-12 years	0.26	765	90	Grand Index
55	m	0-12 years	0.261	765.016	90	Grand Index
55	m	0-12 years	0.262	765.472	90	Grand Index
55	m	0-12 years	0.263	765.928	90	Grand Index
55	m	0-12 years	0.264	766.384	91	Grand Index
55	m	0-12 years	0.265	766.84	91	Grand Index
55	m	0-12 years	0.267	767	91	Grand Index
55	m	0-12 years	0.268	767.208	91	Grand Index
55	m	0-12 years	0.269	767.664	91	Grand Index
55	m	0-12 years	0.27	768.12	91	Grand Index

Age	Gender	Education	Percentile	Raw Score	Norm Score	Assessment
55	m	0-12 years	0.271	768.576	91	Grand Index
55	m	0-12 years	0.276	769	91	Grand Index
55	m	0-12 years	0.277	769.2770883	91	Grand Index
55	m	0-12 years	0.278	769.6820636	91	Grand Index
55	m	0-12 years	0.279	769.9131684	91	Grand Index
55	m	0-12 years	0.28	769.9641932	91	Grand Index
55	m	0-12 years	0.289	770	92	Grand Index
55	m	0-12 years	0.29	770.24	92	Grand Index
55	m	0-12 years	0.291	770.696	92	Grand Index
55	m	0-12 years	0.296	771	92	Grand Index
55	m	0-12 years	0.297	771.0488637	92	Grand Index
55	m	0-12 years	0.298	771.1004421	92	Grand Index
55	m	0-12 years	0.299	771.4182005	92	Grand Index
55	m	0-12 years	0.3	771.8226221	92	Grand Index
55	m	0-12 years	0.302	772	92	Grand Index
55	m	0-12 years	0.303	772.3086031	92	Grand Index
55	m	0-12 years	0.304	773.14624	92	Grand Index
55	m	0-12 years	0.305	773.8499692	92	Grand Index
55	m	0-12 years	0.306	773.9243323	92	Grand Index
55	m	0-12 years	0.307	773.9986954	92	Grand Index
55	m	0-12 years	0.308	774.448	92	Grand Index
55	m	0-12 years	0.309	774.904	93	Grand Index
55	m	0-12 years	0.315	775	93	Grand Index
55	m	0-12 years	0.316	775.096	93	Grand Index
55	m	0-12 years	0.317	775.552	93	Grand Index
55	m	0-12 years	0.326	776	93	Grand Index
55	m	0-12 years	0.327	776.112	93	Grand Index
55	m	0-12 years	0.328	776.568	93	Grand Index
55	m	0-12 years	0.335	777	94	Grand Index
55	m	0-12 years	0.336	777.216	94	Grand Index
55	m	0-12 years	0.337	777.672	94	Grand Index
55	m	0-12 years	0.342	778	94	Grand Index
55	m	0-12 years	0.343	778.408	94	Grand Index
55	m	0-12 years	0.344	778.864	94	Grand Index
55	m	0-12 years	0.345	779.32	94	Grand Index
55	m	0-12 years	0.346	779.776	94	Grand Index
55	m	0-12 years	0.347	780.232	94	Grand Index
55	m	0-12 years	0.348	780.688	94	Grand Index
55	m	0-12 years	0.353	781	94	Grand Index
55	m	0-12 years	0.354	781.424	94	Grand Index
55	m	0-12 years	0.355	781.88	94	Grand Index
55	m	0-12 years	0.357	782	95	Grand Index
55	m	0-12 years	0.358	782.0229682	95	Grand Index
55	m	0-12 years	0.359	782.0651999	95	Grand Index
55	m	0-12 years	0.36	782.2377954	95	Grand Index
55	m	0-12 years	0.361	782.6515636	95	Grand Index
55	m	0-12 years	0.362	783.072	95	Grand Index
55	m	0-12 years	0.363	783.528	95	Grand Index
55	m	0-12 years	0.364	783.984	95	Grand Index
55	m	0-12 years	0.365	784.3185276	95	Grand Index
55	m	0-12 years	0.366	784.648638	95	Grand Index
55	m	0-12 years	0.367	784.8211043	95	Grand Index
55	m	0-12 years	0.368	784.9469939	95	Grand Index
55	m	0-12 years	0.372	785	95	Grand Index
55	m	0-12 years	0.373	785.0658321	95	Grand Index
55	m	0-12 years	0.374	785.4069618	95	Grand Index

Age	Gender	Education	Percentile	Raw Score	Norm Score	Assessment
55	m	0-12 years	0.375	785.7480916	95	Grand Index
55	m	0-12 years	0.376	785.7771956	95	Grand Index
55	m	0-12 years	0.377	785.8062995	95	Grand Index
55	m	0-12 years	0.378	785.881131	95	Grand Index
55	m	0-12 years	0.379	785.9668972	95	Grand Index
55	m	0-12 years	0.381	786	95	Grand Index
55	m	0-12 years	0.382	786.033039	95	Grand Index
55	m	0-12 years	0.383	786.1115065	96	Grand Index
55	m	0-12 years	0.384	786.2581818	96	Grand Index
55	m	0-12 years	0.385	786.6357143	96	Grand Index
55	m	0-12 years	0.392	787	96	Grand Index
55	m	0-12 years	0.393	787.0877229	96	Grand Index
55	m	0-12 years	0.394	787.2800385	96	Grand Index
55	m	0-12 years	0.395	787.4911354	96	Grand Index
55	m	0-12 years	0.396	787.7548198	96	Grand Index
55	m	0-12 years	0.399	788	96	Grand Index
55	m	0-12 years	0.4	788.8	96	Grand Index
55	m	0-12 years	0.401	789.712	96	Grand Index
55	m	0-12 years	0.405	790	96	Grand Index
55	m	0-12 years	0.406	790.136	96	Grand Index
55	m	0-12 years	0.407	790.592	96	Grand Index
55	m	0-12 years	0.412	791	97	Grand Index
55	m	0-12 years	0.413	791.328	97	Grand Index
55	m	0-12 years	0.414	791.784	97	Grand Index
[truncated: 425,506 more chars]
